# Landscape-level effectiveness of fuel treatments in a forest-dominated ecosystem in the Southern United States

**DOI:** 10.1371/journal.pone.0342049

**Published:** 2026-02-13

**Authors:** Junyeong Choi, Tomás Lagos, Brittany Segundo, Lina M. Villa-Zapata, Oleg A. Prokopyev, Lewis Ntaimo, Curt W. Stripling, Jianbang Gan

**Affiliations:** 1 Department of Ecology and Conservation Biology, Texas A&M University, College Station, Texas, United States of America; 2 Discipline of Business Analytics, The University of Sydney, Sydney, Australia; 3 Wm Michael Barnes ’64 Department of Industrial and Systems Engineering, Texas A&M University, College Station, Texas, United States of America; 4 Department of Business Administration, University of Zurich, Zurich, Switzerland; 5 Texas A&M Forest Service, College Station, Texas, United States of America; Dev Bhoomi Uttarakhand University, INDIA

## Abstract

Wildfire has become an increasing threat to natural ecosystems and human livelihood alike in many parts of the world. Vegetation fuel treatments are considered a viable option for mitigating wildfire risk and damage; yet existing studies have yielded mixed or inconclusive results on fuel treatment effectiveness especially at the landscape level. Using fire behavior simulations and statistical analysis of simulation outputs, we assessed landscape-level effectiveness of prescribed burning (PB) and thinning from below (TFB) relative to their site-level effectiveness in terms of area burned (AB) and total cost of treatment and timber loss (TC) in a forest-dominated ecosystem in the southern United States. We found that effectiveness of a treatment varied with measurement metrics and extent, vegetation characteristics and dynamics, and their interactions with the treatment. PB and TFB were less effective at the landscape level than at the site level where fires burned only inside the treatment area. At both site and landscape levels, the effectiveness of PB and TFB in reducing AB and TC largely depended on the quantity of biomass and fire ignition location. TFB outperformed PB in mitigating both AB and TC with a larger timber volume, a longer delay in fire occurrence after treatment, or a higher uncertainty of fire ignition location. TFB was also more effective than PB in reducing TC at the landscape level. By clarifying the conditions under which a fuel treatment can mitigate the area burned and the total cost, this study advanced knowledge of fuel treatment effectiveness especially at the landscape level. Such knowledge can aid in developing and deploying treatment strategies to minimize fire extent and adverse economic consequences in the study region and beyond.

## Introduction

Wildfire has emerged as an increasing threat to both natural ecosystems and human livelihood in many parts of the world, accounting for 27.5% of the world’s total forest cover loss from 2001 to 2022 [1] and engendering detrimental environmental and economic consequences [2,3]. The wildfire risk and damage are expected to rise with a changing climate [4,5] and the accumulated vegetation fuel loads resulting from land-use change, forest and rangeland management practices, and wildfire response policies over the past several decades [6,7]. Fuel treatments, involving prescribed burning and mechanical methods, represent a key approach for mitigating wildfire risk and damage. These treatments manipulate the quantity, composition, and structure of vegetation fuels in both temporal and spatial dimensions [8–10], thus altering fire behavior [11], improving firefighting opportunities, and reducing the adverse impacts of wildfire on ecosystems, cultural resources, and human communities [12,13].

Extensive studies have been undertaken to assess the effectiveness of fuel treatments using both simulation and empirical approaches [14–17]. Due to high costs and liabilities associated with field experiments, simulations have dominated existing studies. Effectiveness is often interpreted as the extent to which one or more desirable objectives or outcome metrics of fuel treatments are achieved, such as burn probability [18,19], fire intensity [20,21], fire extent (area burned) [22,23], fire progression [24,25], and ecological as well as socioeconomic impacts [26,27]. Of these metrics, area burned has been widely used partly because it can be easily measured and interpreted [28–31]. Treatment design including size, shape, spatial placement, prescription (treatment options), and timing is thought to have influences on effectiveness, depending on environmental factors such as fuel, weather, and terrain conditions [15]. Moreover, these factors often interact with one another. For instance, the effectiveness of a fuel treatment may vary with treatment timing and vegetation characteristics that are often heterogeneous and dynamic across the landscape [9,32,33].

Such interactions create challenges for assessing the effectiveness of treatments, particularly at the landscape level. While most existing empirical studies focus on site-level effectiveness, defined here as the impact of a treatment where a fire starts and remains contained within a treated area, wildfires are a landscape-level phenomenon as they often burn across boundaries of vegetation cover and ownership. Consequently, we define landscape-level effectiveness as the ability of a treatment to mitigate fire behavior where a fire interacts with the broader untreated environment, specifically where a fire ignites within a treated area and spreads beyond its boundaries, or where a fire starts in an untreated area and spreads into a treated area. Understanding these specific conditions is essential to clarify under what circumstances treatments can succeed or fail to mitigate negative fire impacts at a scale larger than an individual forest stand or a treated area.

Wildfire is a landscape-level phenomenon as it often burns across the boundaries of vegetation covers, ownerships, and administrative units [34]. Due to budgetary and operational constraints, fuel treatments are typically applied to only a fraction of an area. As a result, a fire can burn across treatment boundaries into non-treated areas even if it starts from a treated area. However, most of the existing studies, especially empirical studies, have focused on the site-level rather than landscape-level effects of fuel treatments due to barriers to conducting landscape-level experiments. These barriers include the multitude of interrelated factors that affect wildfire behavior at the landscape level along with execution complexity, considerable expenses, potential hazards, and liabilities [14,28].

In the United States, existing landscape-level studies have concentrated on the western parts of the country and primarily used fire behavior metrics to measure treatment effectiveness. There is higher potential for damaging or severe wildfires in the western region than in other regions of the United States [35,36], which may undermine the effectiveness of fuel treatments. Budgetary limitations, along with liabilities for using prescribed burning, have been a major barrier to widely adopting fuel treatments in the United States [37,38]. While the effectiveness of fuel treatments in modifying fire behavior at the stand level is well established [32], their effectiveness at the landscape level is more complex and highly dependent on factors like treatment size, placement, age, and the specific weather conditions [15,28,39]. This context dependency can lead to variable outcomes, making it challenging to generalize effectiveness across different ecosystems and fire events. Thus, there is a compelling need to better understand the specific conditions under which treatments succeed or fail to mitigate negative fire impacts at the landscape scale.

In this study we attempted to conduct a simulation study on the effectiveness of fuel treatments at the landscape level in a forest-dominated ecosystem in East Texas, a diverse ecological and wildfire-prone area with landcover and weather patterns resembling characteristics of both eastern and western United States and other parts of the world. Our main objective was to clarify the conditions under which a fuel treatment is effective compared to non-treatment or more effective than another by untangling the potential interactions between fuel treatments and other factors such as vegetation characteristics and weather conditions. We were also interested in understanding the longevity of treatment effectiveness and the possible linkage or differences between the site-level and landscape-level effectiveness of fuel treatments.

To these ends, we simulated wildfire behavior in terms of area burned in various segments of the ecosystem treated with prescribed burning and thinning in different years after treatment and estimate the total cost of treatment and timber loss to wildfire. We generated a large set of simulation outputs representing wildfires burning within and beyond the treated area for all major fuel types in the ecosystem under various weather scenarios. We then used the simulation outputs and regression analysis to generalize the effectiveness of fuel treatments in terms of area burned and total cost at both site and landscape levels. Our findings offer new insights into the effectiveness of fuel treatments that can aid in developing and deploying more effective fuel treatment strategies in the study region and beyond.

## Methods

### Study site and fuel treatment design

**Fig 1:**
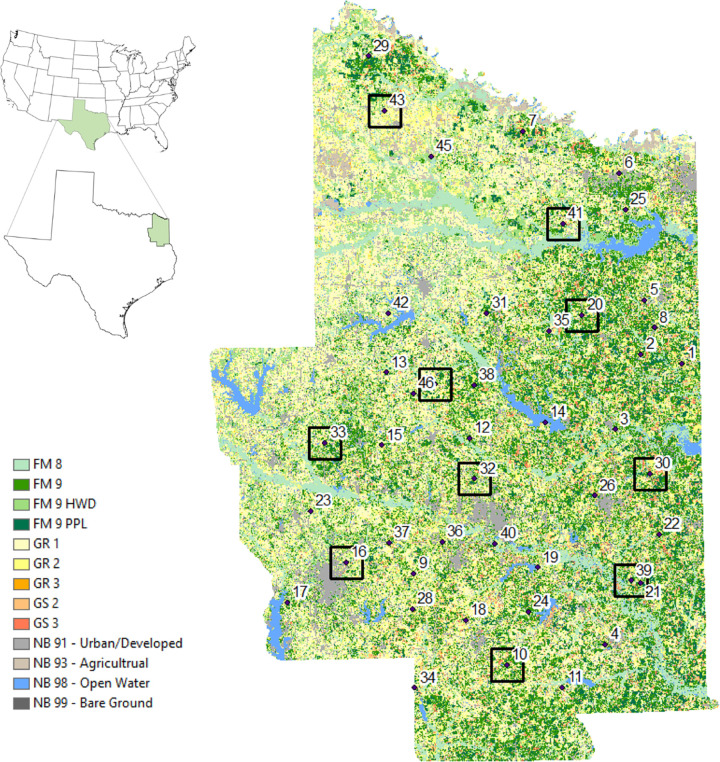
Surface fuel distribution, representative fire locations (RFLs), and the experimental areas of the selected RFLs in the study site. Dots indicate the centers of RFLs; numbers near the dots represent RFL numbers; red circles mark weather stations; small squares denote the RFLs selected for simulation experiments. Vegetation types or fuel models in the legend are described in S1 Table.

The study site was in Texas District 12 (TX12), one of the fire planning units of the Texas A&M Forest Service in East Texas, United States (Fig 1), covering 2,648,585 ha. In this district, on average, 480 wildfires occur annually, with a mean fire size of 8.9 ha; the largest recorded fire burned 404 ha in Smith County in August 2020 [40]. TX12 faces high wildfire risk based on reported indicators such as the wildfire threat index, value response index, and pine plantation response index [41]. Due to its proximity to dense human communities and diverse natural ecosystems, wildfires in this district are more likely to cause adverse socioeconomic and ecological consequences.

The study site was recommended by Texas A&M Forest Service, a state agency responsible for vegetation fuel and wildfire management in Texas, so that the outcomes of this study can help guide fuel and fire management strategies and practices on the site and in similar forest ecosystems in the region. This is a modeling and simulation study that did not require physical access to the study site. Additionally, all data used in this manuscript, including the vegetation cover, forest inventory, past weather, terrain conditions, historical wildfires, and timber stumpage prices, are publicly available. Hence, no permits were required.

This site represents a forest-dominated ecosystem and an urban-wildland interface area that exists widely throughout the southern United States. Located in an ecological transition zone from Eastern Temperate Forests to Great Plains in the United States [42], it consists of diverse vegetation types and mixes ranging from forests to shrubs and to grasses (Fig 1, S1 Table, S2 Table) with a semi-arid climate [43–45]. The Eastern Temperate Forests component is represented by oak-hickory-pine mixed forests, with dominant tree species including various oaks (*Quercus* sp.), hickories (*Carya* sp.), and pines (*Pinus* sp.). The Great Plains component introduces natural grasslands, ranging from tall-grass prairie to, further south, mesquite-acacia savanna. Common fuel treatment objectives in this region include reducing wildfire risk to commercial pine plantations, protecting the expanding wildland-urban interface, and restoring fire-adapted ecosystems. Because the study area encompasses this range of conditions, findings about fuel treatment effectiveness here may provide insights into fuel and fire management in other regions.

For the tractability of simulations, we utilized the concept of a representative fire location (RFL), which represents a set of historical fire occurrences within an area of similar fuel characteristics and wildfire risk [41]. From a total of 46 RFLs, we randomly selected 10 RFLs to optimize the computational resources necessary for simulation while pursuing as much diversity in vegetation and land-use covers as TX12 can offer. Each RFL was assigned an experimental area of 10 km × 10 km, within which a set of simulations was performed. The relatively large experimental area allowed us to examine the effectiveness of fuel treatments at both site and landscape levels. While the RFLs in the west of the study site showed a high proportion of grassland and urban area, the RFLs located in the east were dominated by forestland (S2 Table).

We considered three fuel treatment options: non-treatment (NT), prescribed burning (PB), and thinning from below (TFB). PB and TFB are commonly used fuel treatment methods as well as silvicultural practices in the United States with TFB being more costly than PB [46]. PB, when properly implemented, can reduce surface and ground fuels without hurting bigger trees using controlled fires. TFB is a mechanical method to remove ladder fuels that help surface fires climb to tree crowns, creating more space between crowns to decrease the probability, spreading, and intensity of crown fires while lessening surface fire intensity. Although simulation studies have reported that mixed PB and mechanical treatments could be more effective than PB or thinning alone [47,48], we applied one treatment (NT, PB, or TFB) at a time (in each simulation) to an RFL. This approach enables keeping the experiments simple to clearly identify the effectiveness of each specific treatment relative to the baseline before investigating more complicated combinations.

### Simulation and statistical analysis

**Fig 2:**
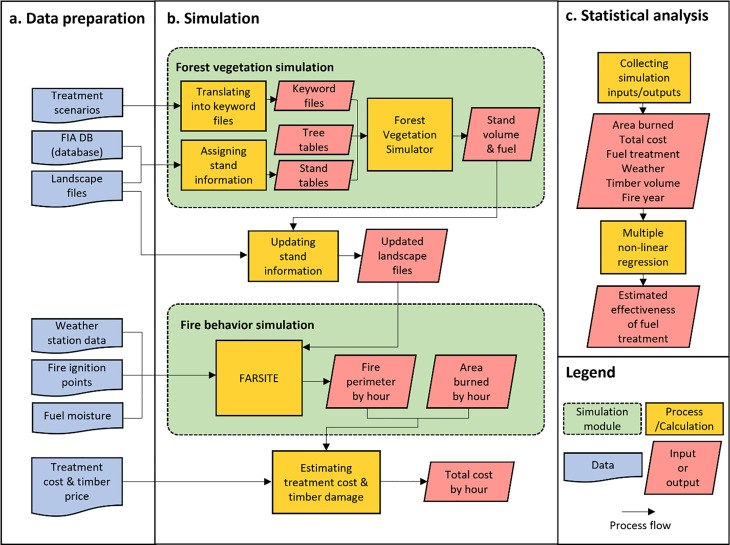
Overall modeling approach including data processing, vegetation dynamics and wildfire simulations, and statistical analysis.

Our overall modeling approach (Fig 2) consisted of three components: i) data preparation, ii) simulations of vegetation dynamics after fuel treatments and simulations of wildfire behaviors under different fuel treatment options and weather scenarios, and iii) regression analysis to assess the effectiveness of fuel treatments.

### Forest vegetation dynamics

We collected forest inventory data for the study site from the Forest Inventory and Analysis (FIA) database (FIADB) [49]. This database contained detailed information on the status and trends for all forests in the United States [50]. Specifically, the inventory information included the conditions and changes of the following: forest area and location; species, size, and health of trees; tree growth, mortality, and removals by harvest; wood production and utilization rates for various products; and forestland ownership [51]. The database was developed and updated periodically based on measurements from the permanent sampling plots established and distributed across forestlands in the United States, with 20% of the plots measured each year [51].

The inventory information was assigned to forest stands in each RFL. The assignment was based on the nearest sampling plot to the forest stand. The forest stands in the RFLs were identified using the fuel models from a map predefined and provided by the LANDFIRE program [52]. The map showed how surface fuel models were spatially distributed in a 30-meter grid spatial resolution. It consisted of the most common fire-carrying fuel types (grass, shrub, timber, or slash), fuel loading and surface area-to-volume ratios by size class and component, fuel bed depth, and fuel moisture of extinction [43]. We selected the pixels of forest-based fuel types and converted them into a forest stand distribution raster dataset. We assumed that one group of continuously connected pixels represented one forest stand and assigned a unique identification number to each pixel group. The distinct pixel groups were related to their nearest sampling plots from the FIADB.

After the forest stand was constructed, we used the Forest Vegetation Simulator (FVS) to simulate its dynamics including biomass growth and changes in stand structure and composition over time. Simulations using FVS require two inputs: a keyword record file and a tree data file. The former contains the user’s directions of the model, while the latter consists of tree-level information such as diameter and species [53]. The FIADB provides tree data files of the sampling plots in the form of FVS tables, and FVS uses those tables to simulate tree growth and yield in the plots.

FVS can also predict the effects of fuel treatments on the dynamics of a forest stand. We ran simulations without fuel treatment (NT) and with PB and TFB. The simulation outputs were collected at 1, 2, and 3 years after fuel treatment, respectively. In the simulations, PB was applied using mean weather conditions of early spring (February–March) before green-up, as recommended by the Texas Parks and Wildlife Department (S3 Table) [54], while TFB was implemented with a target residual basal area of 14 m²/ha, following guidelines from previous studies (S4 Table) [55,56]. Using the Fire and Fuels Extension to the Forest Vegetation Simulator (FVS-FFE) [57], the simulation outputs were converted into five predictors of potential fire activity: Fuel Model (FM), Canopy Cover (CC), Canopy Height (CH), Crown Base Height (CBH), and Crown Bulk Density (CBD). These predictors were combined with topographic predictors (elevation, slope, and aspect) to produce an input file for the fire behavior simulator in the landscape format (.lcp) at a 30-meter spatial grid using ArcFuels 10 [58].

### Wildfire behavior

We used the FlamMap fire mapping and analysis system to simulate fire behavior. The system can analyze and depict potential fire behavior such as rate of spread, fire intensity, and crown fire using fuel and environmental conditions as input [59]. FlamMap consists of several fire behavior models including FARSITE. We selected FARSITE as a primary modeling system because it can utilize sequentially changing weather conditions, which were not available in other models.

FARSITE requires a landscape file, a weather condition file, an initial fuel file, and an ignition location file as input. The landscape file describes characteristics of the simulation landscape including slope, aspect, elevation, fuel model, canopy cover, canopy height, crown base height, and crown bulk density. This file was produced in the previous step using outputs from FVS-FFE combined with topographic variables and formatted into the required landscape format. The weather condition file includes the hourly data of temperature, relative humidity, precipitation, wind speed, wind direction, and cloud cover. The initial fuel moisture file is composed of the percentages of fuel moisture according to fuel characteristics: 1-hour dead fuel, 10-hour dead fuel, 100-hour dead fuel, herbaceous live fuel, and woody live fuel. The ignition location file shows where a fire starts.

With these inputs, FARSITE produces a set of simulated fire behaviors including arrival time, flame length, rate of spread, and perimeters at the same spatial resolution of the input landscape file. In this study, simulations were conducted at a 30-meter grid resolution, with additional settings—such as spot fires, crown fires, and perimeter/distance resolution—detailed in S5 Table. It also generates the fire growth report that summarizes burned areas for a specific simulation time.

The weather data were downloaded from Mesowest [60]. This dataset included hourly records of relative humidity, air temperature, and wind speed from different weather stations. Four weather stations were considered, which were the nearest stations to the selected RFLs, including Gilmer, Linden, Henderson, and Clarksville (Fig 1). We created 30 weather scenarios based on the Hot-Dry-Windy index (*HDW*) calculated from the dataset [61]. *HDW* is defined as:


$$HDW=U\frac{\text{6}\text{1}\text{0}.\text{7}{\times \text{1}\text{0}}^{\text{7}.\text{5}T/(\text{2}\text{7}\text{3}.\text{3}+T)}}{\text{1}\text{0}\text{0}}\frac{RH}{\text{1}\text{0}\text{0}}$$

where *U*, *T*, and *RH* are wind speed (m/s), air temperature (°C), and relative humidity (%), respectively.

Wind speed was measured at 6.1 m above ground, while temperature and relative humidity were measured at 2.0 m [62]. That is, *HDW* is composed of two effects of weather condition: the effect of wind and the effect of atmospheric heat and moisture. Wind affects both the rate of fire spread and the drying of fuels, with a higher wind speed accelerating fire propagation and promoting faster fuel drying. Atmospheric heat and moisture influence fire behaviors by modifying the evaporation rate of fuel moisture. Intuitively, *HDW* combines them into one indicator with a higher *HDW* value indicating conditions prone to more active fires. We focused on weather scenarios of high *HDW* values because mega fires have often been associated with severe or anomalous weather conditions [63–66], and the main objective of fuel treatments is to alleviate mega fires and their catastrophic impacts [67–69]. Conducting analyses on a diverse array of meteorological conditions is helpful for understanding the bigger picture. However, evaluating the effectiveness of fuel treatments under these most destructive scenarios is more urgently needed to ensure our preparedness for mitigating large, high-intensity wildfires that pose the most significant threat to ecosystems and communities. The high *HDW* values represented the 95th percentile of its hourly observations during the two simulation experiment years (21 April 2019–20 April 2021). Each weather scenario represented continuous weather records for 24 hours with the starting weather condition reflecting a high *HDW* value randomly selected from the 95th percentile and a starting time randomly selected between 6 and 17 hours of the day. In other words, after a starting *HDW* value and a starting time were selected, the historical weather records for the next 24 hours constituted a weather scenario.

We applied fuel treatments (NT, PB, and TFB) to each RFL where a circular treatment area with a 1.35 km radius was located at its center. The circular treatment area was chosen over other shapes partly because it is more likely to cover variations in terrain (e.g., aspect and slope), weather (e.g., wind speed and direction), and vegetation/fuel conditions (related to terrain and weather at least) at a given location and partly because it simplifies the selection of fire ignition points inside and outside the treatment area (described below). The radius of the circular area was set to be 1.35 km given the considerations of fire response time (from fire ignition/detection to initial response), fuel treatment cost, and average size of forest tracts owned by landowners, particularly private landowners (major landowner group) in the study region.

To assess both site- and landscape-level effects, we simulated fires igniting at random distances from the treatment boundary in each RFL. Specifically, ignition points were randomly selected within a range of [*-d*, *+ d*] relative to the boundary. We assumed that a fire can start equally likely from any location point of [-*d*, + *d*]. Based on our simulation outcomes of fires starting from the center of the treatment area, we selected *d* = 0.65 km that would allow for approximately 50% of fires to burn across the treatment boundary. We randomly selected fire ignition locations from each quadrant of a RFL to represent the variations in terrain, weather, and vegetation conditions. In cases where a randomly selected ignition location did not develop into a fire (e.g., in a non-burnable area), we relocated that ignition point to the nearest burnable location. The shape and size of the fuel treatment area and the fire ignition locations were so designed that fire simulations would generate statistically adequate and valid data for evaluating and comparing both site- and landscape-level effectiveness of fuel treatments.

We conducted a total of 10,800 wildfire simulations across 10 RFLs. For each RFL, we modeled three fuel treatment scenarios (NT, PB, and TFB) combined with three post-treatment fire occurrence timings (*τ* = 1, 2, and 3 years), four randomly selected ignition locations inside and outside the treatment area, and 30 distinct weather scenarios. This resulted in 1,080 simulations per RFL. Following the simulations, we extracted spatial fire behavior outputs across all grid cells, including fire arrival time, flame length, fire intensity, and rate of spread. From the fire arrival time data, we calculated hourly cumulative area burned for each of the 24 simulation hours. We selected Area Burned (AB) as the primary response variable to represent the overall extent of wildfire. This metric is widely used for assessing landscape-level fire impacts, which allows our results to be readily comparable with the existing body of literature [28–31]. To provide a broader understanding of treatment effectiveness, we supplemented this primary analysis by modeling four other fire behavior metrics that drive fire severity and ecological effects: flame length (FL), fireline intensity (FI), crown fire activity (CFA), and rate of spread (ROS).

### Fire behavior model validation

We validated FARSITE parameters using two historical fires near our study site: the Mayfield Fire and the Clear Lake Fire. The Mayfield Fire took place in a grassland-dominated landscape while the Clear Lake Fire occurred in a forestland-dominated area. Both forest and grass/shrub vegetation covers were chosen for validation because the RFLs selected for this study contained a mix of forest and grass/shrub vegetation types (Fig 1, S2 Table). There were no fuel treatments on these validation sites. More details about these two fires are described in S6 Table.

The observed perimeters were obtained from the Wildland Fire Interagency Geospatial Services Current Interagency Fire Perimeters dataset [70]. The characteristics of the simulation landscape were derived from LANDFIRE [52]. The weather data were downloaded from the nearest Remote Automatic Weather Station (RAWS) on the RAWS USA Climate Archive site [71].

We used the widely-adopted Sorensen metric (*SM*) [72] to compare the observed and simulated fire perimeters and area burned (S1 Fig.) [73–77]. The metric is defined as:


$$SM=\frac{\text{2}A}{\text{2}A+B+C}$$

where *A* is the area burned included in both the observed and simulated perimeters (correct estimate), *B* is the area burned included only in the observed perimeters (underestimate), and *C* is the area burned included only in the simulated perimeters (overestimate). Observed and simulated fire perimeters and area burned are considered to be in substantial agreement if the *SM* value is between 0.6 and 0.8 [72,75]. The calculated *SM* value for the Mayfield Fire and the Clear Lake Fire was 0.77 and 0.68, respectively (S7 Table).

### Economic impact estimation

Besides area burned, we were also interested in the sum of fuel treatment costs and economic damage caused by wildfires, referred as total cost herein. We focused on the damage of timber loss to wildfire, which reflected a major concern for private landowners in the study area where most forestlands were privately owned [78]. The post-TFB basal area closely aligns with silvicultural recommendations, which support long-term timber productivity and quality [79–81]. Similarly, properly designed and applied PB can reduce surface fuel loads without undermining timber productivity. While it may cause some mortality of smaller trees, which is often intended in silviculture for stand density control in overstocked forests where fire risk is also high, PB can improve forest health and stimulate tree growth. We designed and applied the PB treatment in the simulations based on silvicultural guidelines and practices in the region, thus causing little negative impact on long-term timber yield [82].

We estimated timber loss by integrating spatially explicit fire behavior from FARSITE with stand-level vegetation data from FVS at a 30-meter grid resolution. For each burned grid cell, total timber loss was calculated by multiplying the standing timber volume by the probability of tree mortality:


$$Timber\;loss=\;\sum_{i}^{N}{Vol}_{i}{Pm}_{i}$$

where *i* represents an individual grid cell, *N* denotes the total number of burned cells, *Vol*_*i*_ represents the standing timber volume for grid cell *i*, and *Pm*_*i*_ shows the probability of tree mortality for grid cell *i*. While the concept of tree mortality is embedded within the FVS-FFE post-fire logic, we employed these mortality functions explicitly at the pixel level to capture the spatial heterogeneity of fire behavior across the landscape. *Pm* was determined using a logistic function based on bark thickness (*b*) and the percentage of crown volume scorched (*c*) [83]:


$${Pm}_{i}=\frac{\text{1}}{\text{1}+e^{\left(-\text{1}.\text{9}\text{4}\text{1}+\text{6}.\text{3}\text{1}\text{6}\left(\text{1}-e^{-b}\right)-\text{0}.\text{0}\text{0}\text{0}\text{5}\text{3}\text{5}c^{\text{2}}\right)}}$$

Bark thickness is defined as $b=v_{i}d_{i}$, where *v* is a bark thickness parameter and *d* is the tree diameter. The percentage of crown volume scorched is calculated as $c=\text{1}\text{0}\text{0}s_{i}\left(\frac{\text{2}l_{i}-s_{i}}{{l_{i}}^{\text{2}}}\right)$, where *s* is the scorch height and *l* is the total crown length. We derived the scorch height (*s*) for each pixel based on the fireline intensity values simulated by FARSITE. Tree-level attributes—including species, diameter (*d*), and crown length (*l*)—along with the standing timber volume (*Vol*) were provided by FVS based on the nearest forest inventory plot data to that cell. By calculating mortality independently for every pixel, this approach explicitly accounts for the spatial heterogeneity of fire behavior and tree characteristics across the burned landscape. Finally, the total timber loss for a simulated wildfire was determined by summing the estimated losses from all affected grid cells within the landscape.

The damaged timber value was calculated by multiplying the 5-year average stumpage price (US$/m^3^) [84] by the damaged timber volume for each tree species. This calculation assumed a 100% loss of pre-fire stumpage value for mortality-affected timber. It might overestimate the economic damage because it did not account for potential salvage logging revenue. However, salvaged timber is often used only for producing low-value products. Given the rising logging cost and the low price of low-quality timber in the study region, including the value of salvaged timber is unlikely to considerably change the estimated timber loss value. Finally, we aggregated the timber losses across all grid cells within a landscape area to yield the total timber loss value at the landscape level. We calculated fuel treatment costs, based on the regional costs and trends for southern forestry practices [85]. We used a cost of US$77/ha for PB and US$356/ha for TFB, respectively.

### Statistical analysis

We analyzed the effectiveness of fuel treatments using the Gaussian Generalized Linear Model (GLM) approach to estimate the relationship between a response variable and a set of explanatory variables, which were selected based on previous studies on wildfire behavior [86–88] and fuel treatment effectiveness [89–92]. We considered two response variables, area burned (*AB*) and total cost of fuel treatment and timber loss (*TC*) (Table 1). We used the bidirectional stepwise variable selection method to determine the best set of explanatory variables. We also considered interaction terms of explanatory variables based on the interaction hierarchy, effect sparsity, and effect heredity principles [93] and the literature on fuel treatment effectiveness [32,94–96].

**Table 1:** Descriptions of variables included in the regression models.

Variable	Min^a^	Mean (Std Dev)^a^	Max^a^	Frequency^b^	Description
*Response variables*					
*AB*	0.1	150.4 (236.9)	2,336.4		Hourly cumulative area burned, derived from FARSITE simulations (ha)
*TC*	0	505.0 (1,274.0)	18,921.9		Total cost – the sum of fuel treatment cost and timber value loss due to the fire (US$1,000)
*Explanatory variables*					
*h*				10,800 (each hour)	Wildfire simulation hour – hours of burning since fire ignition (1, 2, 3, …, 24 hours)
*PB*				86,400 (*PB* = 1)	Prescribed burning – a binary variable indicating whether prescribed burning is applied (0: no, 1: yes)
*TFB*				86,400 (*TFB* = 1)	Thinning from below – a binary variable indicating whether thinning from below is applied (0: no, 1: yes)
*Bdnt*				64,007 (*Bdnt* = 1)	Fire behavior – a binary variable indicating whether a fire igniting outside a treated area advances into it.
*Bdtn*				75,230 (*Bdtn* = 1)	Fire behavior – a binary variable indicating whether a fire starting inside a treated area spreads beyond it.
*d*	−0.6	0 (0.4)	0.8		Ignition location – the distance between the treatment boundary to an ignition location (km)
*Bm*	2.5	80.8 (73.6)	309.8		Vegetation characteristics – merchantable timber volume around an ignition location prior to fuel treatment (m^3^/ha)
*τ*				86,400 (each year)	Delay in fire occurrence after fuel treatment – years since fuel treatment when a fire occurs (0: 1 year, 1: 2 years, 2: 3 years)
*T*	2.2	24.3 (6.6)	36.6		Weather condition – hourly air temperature derived from historical weather data (°C)
*WS*	0.4	2.9 (1.5)	10.3		Weather condition – hourly wind speed derived from historical weather data (m/s)
*RH*	16.0	67.4 (20.7)	100.0		Weather condition – hourly relative humidity derived from historical weather data (%)

^a^Minimum, mean (standard deviation), and maximum are for continuous variables. The maximum value of *d* is 0.8, greater than its initially set upper bound of 0.65. This is because one randomly selected fire ignition location was in a non-burnable area and had to be relocated to its nearest burnable point that happened to be farther away from the treatment boundary.

^b^Frequency is for discrete variables; *n* = 259,200.

Starting from the base model consisting of one constant (one constant and all possible explanatory variables and their interaction terms), this method attempted to add or delete one variable in each step so that the statistical quality of the model improved. If adding or removing an additional variable did not improve the model’s quality, then the process stopped and the best model was identified. The metrics used to guide the selection of explanatory variables included the Akaike Information Criterion (AIC) for measuring the overall goodness of fit and the variance inflation factor (VIF) for detecting multicollinearity [97,98]. While stepwise selection methods have limitations, such as overfitting or multicollinearity [99,100], we employed this approach as a systematic tool to reduce a large set of potential predictors to a parsimonious and interpretable final model. The statistical analysis was conducted using R [101].

We then obtained the following regression model for both response variables:


$$\begin{array}{cc} \text{log}\left({AB}_{i\tau h}\;\text{or}\;{TC}_{i\tau h}\right)\\ & \;\;\;\;\;\;\;\;\;\;\;\;\;\;\;\;\;\;=\beta_{\text{0}}+{\beta_{\text{1}}h+\beta }_{\text{2}}{PB}_{i}+\beta_{\text{3}}{TFB}_{i}+\beta_{\text{4}}\tau +\beta_{\text{5}}{Bm}_{i}+\beta_{\text{6}}{Bdtn}_{\;i\tau h}+\beta_{\text{7}}{Bdnt}_{\;i\tau h}\\ & \;\;\;\;\;\;\;\;\;\;\;\;\;\;\;\;\;\;+\beta_{\text{8}}{PB}_{i}\times \tau +\beta_{\text{9}}{TFB}_{i}\times \tau +\beta_{\text{1}\text{0}}{PB}_{i}{\times Bm}_{i}+\beta_{\text{1}\text{1}}{TFB}_{i}{\times Bm}_{i}+\beta_{\text{1}\text{2}}{PB}_{i}{\times Bdtn}_{\;i\tau h}\\ & \;\;\;\;\;\;\;\;\;\;\;\;\;\;\;\;\;\;+\;\beta_{\text{1}\text{3}}{TFB}_{i}{\times Bdtn}_{i\tau h}+\beta_{\text{1}\text{4}}{PB}_{i}{\times Bdnt}_{i\tau h}+\beta_{\text{1}\text{5}}{TFB}_{i}{\times Bdnt}_{\;i\tau h}+\beta_{\text{1}\text{6}}{PB}_{i}{\times d}_{i}+\beta_{\text{1}\text{7}}{TFB}_{i}{\times d}_{i}\\ & \;\;\;\;\;\;\;\;\;\;\;\;\;\;\;\;\;\;+\sum {\mathrm{\backslash nolimits}}_{j=\text{1}}^{\text{J}}\beta_{\text{1}\text{7}+j}X_{ji\tau h}+\varepsilon_{i\tau h} \end{array}$$

where *i* represents RFLs, *τ* shows the number of years since fuel treatment when the fire occurs, *h* denotes the number of hours since the fire is ignited, *j* indicates the regression coefficients (*β*’s) for *X*, *ε* is the error term, and all variables are explained in Table 1 with *X* representing *T*, *WS*, and *RH*. The inclusion of the weather variables, *T*, *WS*, and *RH*, in the model was to remove their effects on the FARSITE-simulated outputs because they were also the input variables of FARSITE. As such, the estimated regression coefficients associated with the weather variables were not be interpreted. We selected the semi-log (response) function after testing and comparing it with linear, log-log, and semi-log (explanatory) forms. We also tested for potential spatial autocorrelation, homoscedasticity, and multi-collinearity in our models. The models were fit using our FARSITE simulation output and economic impact estimation data (S1 File). Table 1 summarizes the descriptive statistics of the response and explanatory variables.

To provide a clear framework for evaluating treatment performance, we distinguished between site-level and landscape-level effectiveness based on the ignition location and boundary-crossing behavior of a fire. Site-level effectiveness refers to scenarios where a fire starts and remains entirely within the boundaries of a treated area (*d* < 0 and *Bdtn* = 0). In contrast, landscape-level effectiveness represents the treatment impact where fires burn across treatment boundaries—where a fire either ignites within and spreads beyond a treated area (*d* < 0 and *Bdtn* = 1) or ignites in an untreated area and spreads into a treated area (*d* > 0 and *Bdnt* = 1).

To explore the treatment effectiveness beyond area burned and total cost, we also considered four other fire behavior metrics, including flame length (FL), fireline intensity (FI), crown fire activity (CFA), and rate of spread (ROS). We fit a regression model for each of these metrics using the same set of explanatory variables and interaction terms shown above (S8 Table), respectively. Using the same set of independent variables made it convenient to compare the treatment effectiveness between different fire behavior metrics. The statistical results for these supplementary models are provided in the Supporting Information (S9–S12 Tables).

## Results

### Wildfire simulation results

There are considerable variations in the area burned and total cost, which indicates a wide-ranging dataset for regression analysis. Such variations were illustrated in S2 Fig, which shows different responses of fire perimeters to fuel treatment options in two different years after treatment in three RFLs. For instance, in RFLs 20 and 33, the fires starting inside the treatment area tended to have a smaller area burned than those starting outside the treatment area, but the opposite was true for RFL 43. From 2019 to 2021 (a delay of two more years in fire occurrence after treatment), the fires starting inside the treatment area in RFL 20 burned larger areas, but this delay effect was less obvious for the fires starting outside the treatment area in RFL 20, inside the treatment area in RFL 33, or inside and outside the treatment area in RFL 43.

The descriptive statistics of area burned (AB) and total cost (TC) provide further evidence of these variations (S3 and S4 Figs). The means of AB did not differ significantly across non-treatment (NT), prescribed burning (PB), and thinning from below (TFB) if all RFLs were considered. In contrast, of the three treatments (NT, PB, and TFB), TFB had the lowest mean of AB in the high-biomass RFLs (RFLs 20, 30, and 39; mean *Bm* = 155.2 m^3^/ha), whereas PB had the lowest in the low-biomass RFLs (RFLs 16, 41, and 44; mean *Bm* = 28.8 m^3^/ha). The mean of TC for PB and TFB was similar but lower than that of NT in the high-biomass RFLs, but PB had the lowest mean of TC in all RFLs and the low-biomass RFLs.

### Regression results

Two semi-log regression models were estimated. One showed the statistical relationship between hourly accumulative area burned (*AB*) and a set of explanatory variables including fire duration (*h*), fuel treatment options (*PB* and *TFB*), fire spreading from the treatment area to the non-treatment area (*Bdtn*) or from the non-treatment area to the treatment area (*Bdnt*), timber volume (*Bm*), delay in fire occurrence after treatment (*τ*), distance of the ignition location from the treatment boundary (*d*), and their interaction terms (Table 2). The Moran’s I test did not detect spatial autocorrelation for both AB (p = 0.215) and TC (p = 0.471) models. The values of the variance inflation factor (VIF) did not show evidence of multicollinearity either (Tables 1 and 2). To avoid the heteroscedasticity problem, we utilized the Gaussian Generalized Linear Model (GLM) approach.

**Table 2:** The estimated regression model for area burned.

Variable^a^	Estimated *β*	Std Error	p-value	VIF
*Intercept*	−7.418	0.125	< 0.001	
*Fire duration (h)*	0.160	0.000	< 0.001	1.557
*Relative humidity (RH)*	−0.027	0.000	< 0.001	1.291
*Wind speed (WS)*	0.142	0.002	< 0.001	1.181
*Temperature (T)*	0.015	0.000	< 0.001	1.040
*Prescribed burning (PB)*	−0.063	0.014	< 0.001	6.182
*Thinning from below (TFB)*	0.042	0.014	< 0.001	6.322
*Fire spreading from the treatment area to the non-treatment area (Bdtn)*	2.105	0.012	< 0.001	3.974
*Fire spreading from the non-treatment area to the treatment area (Bdnt)*	2.266	0.013	< 0.001	4.041
*Timber volume (Bm)*	−0.001	0.000	< 0.001	3.029
*Delay in fire occurrence after treatment (τ)*	0.152	0.006	< 0.001	3.007
*PB*×*Bdtn*	0.202	0.016	< 0.001	3.225
*TFB*×*Bdtn*	0.168	0.016	< 0.001	3.371
*PB*×*Bdnt*	−0.242	0.017	< 0.001	2.977
*TFB*×*Bdnt*	−0.163	0.017	< 0.001	2.957
*PB* × *Bm*	0.000	0.000	0.035	3.587
*TFB* × *Bm*	−0.001	0.000	< 0.001	3.583
*PB* × *τ*	−0.033	0.008	< 0.001	4.008
*TFB* × *τ*	−0.071	0.008	< 0.001	4.007
*PB* × *d*	0.819	0.015	< 0.001	1.530
*TFB*× *d*	0.753	0.015	< 0.001	1.587

^a^All variables are described in Table 1 with × denoting the interaction between two variables.

The model was a significant improvement over the intercept-only model (Likelihood-ratio test: χ^2^ (20) = 334,061, p < 0.001). A Nagelkerke’s pseudo-R² of 0.740 and an RMSE of 1.352 (within the data range of −7.013 to 3.151) both indicate a good model fit. VIF is the variance inflation factor.

The other model depicted the relationship between total cost of fuel treatment and timber loss (*TC*) and the explanatory variables (Table 3). For the purpose of interpreting our modeling results, we defined ‘site-level’ effectiveness as scenarios where fires ignited and remained within the treated area (i.e., *d* < 0 and *Bdtn* = 0). In contrast, we defined ‘landscape-level’ effectiveness as scenarios where fire crossed the treatment boundary, either by starting within the treatment and spreading out (*Bdtn* = 1) or by starting outside and spreading into (*Bdnt* = 1). This framework allowed us to statistically distinguish the performance of treatments when their influence was localized versus when they interacted with the surrounding untreated landscape. Only the explanatory variables that were statistically significant at 5% or better were included in the final regression models. The models showed good model fits with Nagelkerke’s pseudo-R² of 0.740 and 0.630 for *AB* and *TC*, respectively.

**Table 3:** The estimated regression model for total cost of fuel treatment and timber loss.

Variable^a^	Estimated *β*	Std Error	p-value	VIF
*Intercept*	−2.211	0.176	< 0.001	
*Fire duration (h)*	0.119	0.001	< 0.001	1.553
*Relative humidity (RH)*	−0.016	0.000	< 0.001	1.291
*Wind speed (WS)*	0.052	0.003	< 0.001	1.177
*Temperature (T)*	0.001	0.001	0.021	1.039
*Prescribed burning (PB)*	4.404	0.017	< 0.001	4.314
*Thinning from below (TFB)*	5.910	0.019	< 0.001	5.817
*Fire spreading from the treatment area to the non-treatment area (Bdtn)*	4.518	0.016	< 0.001	3.972
*Fire spreading from the non-treatment area to the treatment area (Bdnt)*	4.732	0.018	< 0.001	4.036
*Timber volume (Bm)*	0.017	0.000	< 0.001	3.029
*Delay in fire occurrence after treatment (τ)*	0.143	0.006	< 0.001	1.504
*PB*×*Bdtn*	−3.785	0.023	< 0.001	3.223
*TFB*×*Bdtn*	−4.494	0.022	< 0.001	2.973
*PB*×*Bdnt*	−3.754	0.024	< 0.001	2.972
*TFB*×*Bdnt*	−4.413	0.023	< 0.001	2.804
*PB* × *Bm*	−0.006	0.000	< 0.001	3.587
*TFB* × *Bm*	−0.010	0.000	< 0.001	3.583
*TFB* × *τ*	−0.053	0.010	0.002	3.005
*PB* × *d*	0.311	0.022	< 0.001	1.530

^a^All variables are described in Table 1 with × denoting the interaction between two variables.

The model was a significant improvement over the intercept-only model (Likelihood-ratio test: χ^2^ (18) = 247,531, p < 0.001). A Nagelkerke’s pseudo-R² of 0.630 and an RMSE of 1.894 (within the data range of −6.908 to 9.848) both indicate a good model fit. VIF is the variance inflation factor.

Obviously, AB expanded as the fire continued to burn. For one additional hour of burning, *ceteris paribus*, area burned on average increased by 16.0% of the total area burned at the beginning of the hour (Table 2). TC showed a similar pattern of correlations with fire duration (*h*), with a 11.9% increase for each additional hour of burning (Table 3). Both AB and TC increased with longer delays in fire occurrence after treatment (*τ*). However, timber volume (*Bm*) had opposite impacts on AB and TC—a higher *Bm* value tended to lower AB but heighten TC (Tables 2 and 3). It is important to note that while the regression coefficient for *Bm* is small on a per-unit basis, its influence could be substantial given that the variable spans a range of over 300 m³/ha in our dataset.

Before detailing the results for AB and TC, we note that our supplementary regression analyses for FL, FI, CFA, and ROS revealed a similar pattern of treatment effectiveness on AB and TC. The direction and significance of the main explanatory variables—particularly the interactions between treatment option, timber volume (*Bm*), and time since treatment (*τ*)—were consistent across all models. The full statistical results for these corroborating analyses are provided in S9–S12 Tables, and their visual trends are shown in S3 and S4 Figs.

### Effects of fuel treatments on area burned

The effectiveness of fuel treatments in reducing AB depended upon many factors. AB was significantly correlated with both the fuel treatment options and their interactions with other explanatory variables representing whether a fire spread out of or into the treatment area (*Bdtn* or *Bdnt*), how much timber was available (*Bm*), how far away the ignition location was from the treatment boundary (*d*), and how soon a fire occurred after treatment (*τ*).

The sums of coefficients associated with *Bdtn* and *PB*×*Bdtn*, *Bdtn* and *TFB*×*Bdtn*, *Bdnt* and *PB*×*Bdnt*, and *Bdnt* and *TFB*×*Bdnt* were all positive (Table 2). Consistent with expectations, the effectiveness of both PB and TFB declined when a fire burned across the treatment boundary. Our models further allowed us to quantify this reduction: the positive interaction terms (e.g., *PB*×*Bdtn*, *TFB*×*Bdtn*) show the specific penalty to effectiveness at the landscape level compared to the site level and reveal how this penalty varies by treatment option. The effectiveness of PB and TFB increased as *d* decreased (closer to the center of the treatment area on either side of the treatment boundary) given the positive coefficients associated with *PB* × *d* and *TFB* × *d*. TFB was less responsive to a change in *d* than PB.

**Fig 3:**
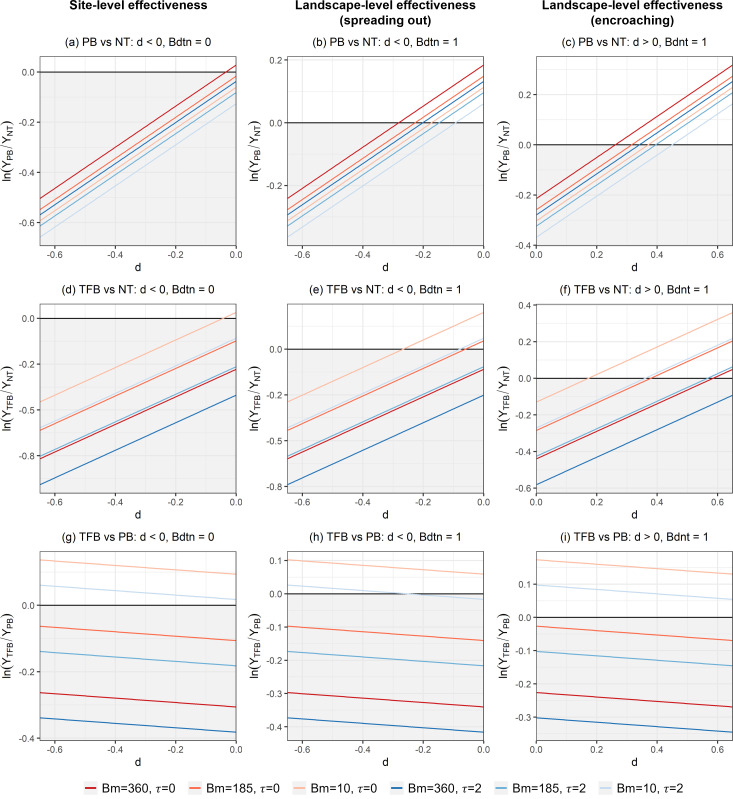
Effects of fuel treatments on area burned. Subplots are organized by columns representing site-level effectiveness (left; *d *< 0 and *Bdtn *= 0), landscape-level effectiveness (spreading out) (center; *d *< 0 and *Bdtn *= 1), and landscape-level effectiveness (encroaching fire) (right; *d *> 0 and *Bdnt *= 1). NT = non-treatment, PB = prescribed burning, and TFB = thinning from below. *Y* denotes area burned. The vertical axis represents the log-ratio of area burned between the compared treatment options. The shaded gray area below the horizontal line placed at the zero value of the vertical axis indicates where the treatment is effective in reducing area burned compared to the baseline. *d* represents the distance of the ignition location from the treatment boundary (*d* < 0 inside the treatment area, *d* > 0 outside the treatment area) (km), *Bm* depicts the volume of timber prior to fuel treatment (m^3^/ha), and *τ* indicates the number of years after treatment when the fire occurs.

An increase in *Bm* reduced AB and such an effect was more profound for TFB than for PB as indicated by the negative coefficient associated with *TFB* × *Bm*. The influence of *Bm* on treatment effectiveness also varied with *d*, *τ*, *Bdtn*, or *Bdnt*. Where a fire started and stayed in the treatment area (*d* < 0 and *Bdtn* = 0), *ceteris paribus*, PB was effective as long as *Bm* < 249.3 m^3^/ha and TFB was effective as long as *Bm* > 46.8 m^3^/ha. The effectiveness of PB or TFB increased as *d* decreased (closer to the center of the treatment area) or *τ* increased (Fig 3a-f). The positive coefficient associated with *τ* implies that AB increased over time without treatment; however, PB and TFB were more effective than NT given the negative coefficients of *PB* × *τ* and *TFB* × *τ*. For a 1-year delay in fire occurrence, AB increased by 15.2%, 11.9%, and 8.1% under NT, PB, and TFB, respectively (Table 2).

Overall, both PB and TFB were effective in reducing AB at both the site and landscape levels, although their effectiveness was lower at the landscape level. The effectiveness of TFB was positively correlated with *Bm* whereas that of PB was negatively correlated with *Bm* (Fig 3a-f). The effectiveness of PB and TFB was more evident where there was a longer delay in fire occurrence after treatment (larger *τ*) (Fig 3a-f) or when a fire starting in the non-treatment area burned across the treatment boundary (*d* > 0 and *Bdnt* = 1) (Fig 3c, 3f). TFB was more effective than PB as *τ*, *d*, and *Bm* increased or when a fire started in the treatment area and spread into the non-treatment area (*d* < 0 and *Bdtn* = 1) (Table 2, Fig 3g-i).

### Effects of fuel treatments on total cost

Likewise, we found complex relationships between fuel treatments and TC. Although the fuel treatments incurred costs, they could reduce timber losses by reducing fire risks and severity. The effect of a fuel treatment on TC also depended on not only the treatment itself but also its interactions with the crossing of the treatment boundary by a fire (*Bdtn* or *Bdnt*), quantity of timber prior to treatment (*Bm*), distance of the ignition location from the treatment boundary (*d*), and delay in fire occurrence after treatment (*τ*).

The sums of coefficients associated with *Bdtn* and *PB*×*Bdtn*, *Bdtn* and *TFB*×*Bdtn*, *Bdnt* and *PB*×*Bdnt*, and *Bdnt* and *TFB*×*Bdnt* were all positive (Table 3). This observation indicates that PB and TFB were less effective in mitigating TC at the landscape level than at the site level. The negative coefficient associated with *TFB*×*Bdtn* (*TFB*×*Bdnt*) was smaller than that with *PB*×*Bdtn (PB*×*Bdnt)*. Thus, TFB was more effective than PB where a fire burned across the treatment boundary regardless of its ignition location (i.e., at the landscape level).

**Fig 4:**
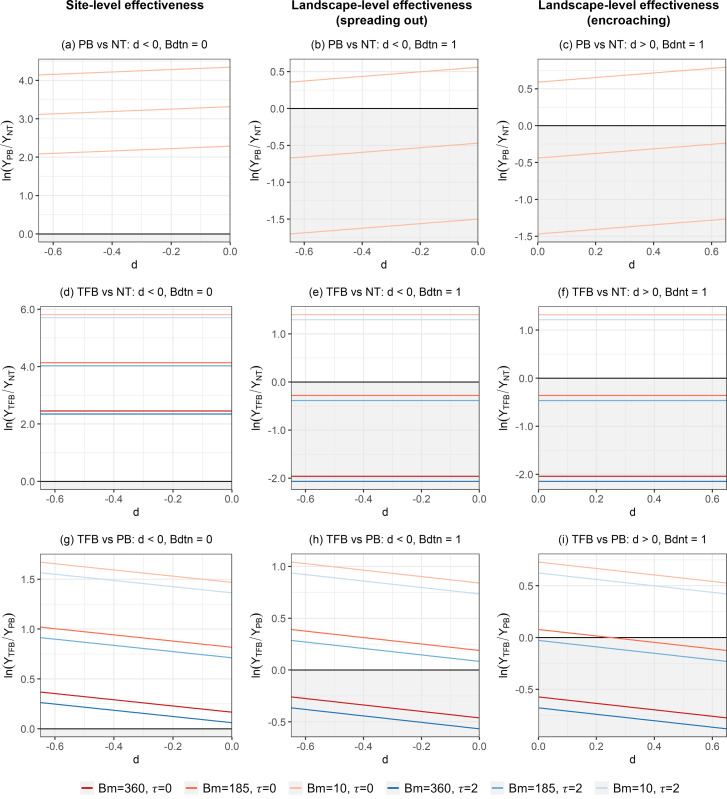
Effects of fuel treatments on total cost of treatment and timber damage. Subplots are organized by columns representing site-level effectiveness (left; *d *< 0 and *Bdtn *= 0), landscape-level effectiveness (spreading out) (center; *d *< 0 and *Bdtn *= 1), and landscape-level effectiveness (encroaching fire) (right; *d *> 0 and *Bdnt *= 1). NT = non-treatment, PB = prescribed burning, and TFB = thinning from below. *Y* denotes total cost. The vertical axis represents the log-ratio of area burned between the compared treatment options. The shaded gray area below the horizontal line placed at the zero value of the vertical axis indicates where the treatment is effective in reducing total cost compared to the baseline. *d* represents the distance of the ignition location from the treatment boundary (*d* < 0 inside the treatment area, *d* > 0 outside the treatment area) (km), *Bm* depicts the volume of timber prior to fuel treatment (m^3^/ha), and *τ* indicates the number of years after treatment when the fire occurs.

The coefficients associated with *PB* × *Bm* and *TFB* × *Bm* were negative (Table 3), implying the effectiveness of PB and TFB (relative to NT) improved as *Bm* increased. When a fire started and stayed in the treatment area (*d* < 0 and *Bdtn* = 0), PB and TFB were effective in reducing total cost where *Bm* > 748.8 m^3^/ha and *Bm* > 615.5 m^3^/ha, respectively. The minimum *Bm* threshold at which fuel treatments became effective was 105.3 m³/ha for PB and 147.4 m³/ha for TFB when fires spread from the treatment area to the non-treatment area. When fires spread in the opposite direction—from the non-treatment area to the treatment area—the thresholds were slightly higher: 110.4 m³/ha for PB and 155.9 m³/ha for TFB, respectively. Interestingly, while the effectiveness of PB decreased as *d* increased (Fig 4a-c), the effectiveness of TFB was independent of *d* (Fig 4d-f). This advantage of TFB over PB was also more evident with an increase in *τ*. For a 1-year delay in fire occurrence, total cost increased by 14.3% for the area treated with PB or untreated while it went up by 9.0% for the area treated with TFB (Table 3).

Overall, effectiveness of PB and TFB in mitigating total cost at both site and landscape levels largely depended on the amount of biomass (*Bm*), and both treatments would be effective with a sufficiently large *Bm* (Fig 4a-f). TFB outperformed PB with an increase in *Bm*, *d*, and *τ* or at the landscape level (Table 3, Fig 4g-i).

## Discussion

This study provides new insights into the effectiveness of fuel treatments such as prescribed burning (PB) and thinning from below (TFB) in reducing the area burned (AB) and the total cost (TC) of fuel treatment and timber loss caused by fires. We found the complex relationships of AB and TC with PB and TFB, especially their interactions with boundary crossing by a fire (*Bdtn* or *Bdnt*), timber volume (*Bm*), ignition location (*d*), and delay in fire occurrence after treatment (*τ*). These complex relationships help explain the existing mixed or inconclusive results on the effectiveness of fuel treatments [39,102]. The marked differences between our regression results and the simple mean comparisons (S3 and S4 Figs) also indicate that univariate analysis may not uncover the full spectrum of fuel treatment effectiveness.

In interpreting and applying our findings, however, please be reminded that our statistical analysis results are based on the data of simulated wildfire behaviors. Empirical data on wildfire behaviors under designed fuel treatments at the landscape level are limited largely due to high costs and liabilities associated with doing such in-field experiments. Data that measure multiple metrics of fire behaviors and allow for analyzing both site- and landscape-level effectiveness of fuel treatments are even rarer. As such, we had to use data derived from model simulations. Our findings and their implications should be treated differently from those derived from empirical or observation data. Nevertheless, the simulation models used in this study have been validated and widely used, and simulations enabled us to generate a large set of data covering a wide range of variations and scenarios. Hopefully, outcomes of this study can contribute to understanding the effects of fuel treatments on fire behaviors and timber loss at least from a theoretical perspective and offer some hints for designing and implementing fuel treatments as well as empirical studies of fuel treatments at the landscape level.

### Effectiveness by biomass level

To a large extent, whether PB and TFB were effective in reducing AB or TC depended on the amount of biomass or timber (*Bm*). However, *Bm* had a different effect on AB than on TC. As *Bm* increased, AB decreased due to a larger proportion of forestland relative to grassland and shrubland (fires tended to spread faster in grassland or shrubland than in forestland), while TC rose due to the higher timber volume that could potentially be lost to a fire. TFB became more effective than PB or NT in mitigating both AB and TC with an increase in *Bm*.

To achieve site-level effectiveness in reducing AB, *Bm* had to be below 249.3 m³/ha for PB and above 46.8 m³/ha for TFB. However, to attain effectiveness at the landscape level, the required maximum *Bm* for PB decreased, and the minimum *Bm* for TFB increased. While the *Bm* required for site-level effectiveness in reducing TC was relatively high, it dropped significantly once fires spread between treatment and non-treatment areas. Specifically, the minimum *Bm* threshold for effective cost mitigation was 105.3 m³/ha for PB and 147.4 m³/ha for TFB when fire spread from the treatment area to the non-treatment area. When fires spread in the opposite direction, the thresholds were slightly higher, 110.4 m³/ha for PB and 155.9 m³/ha for TFB, respectively. By contrast, the mean of *Bm* at the county level was 58.2 m^3^/ha with a range of 33.9–99.5 m^3^/ha across the 43 forest counties in East Texas, but *Bm* at the forest stand level was higher with an estimated mean of 118.04 m^3^/ha [49]. This gap in *Bm* suggests that, across most timberlands in our study area (where mean *Bm* = 58.2 m³/ha), both PB and TFB are likely to reduce AB. However, because treatment costs are high—especially for TFB—only a small subset of high‐volume stands (those exceeding the *Bm* thresholds) can be cost-effectively treated with these methods. Meanwhile, these timberlands also provide significant non-timber values ranging from carbon to wildlife habitat that are susceptible to wildfire as well [103,104]. If these non-timber benefits are accounted for, then PB and TFB may become more justifiable economically.

This finding is directly supported by our fire behavior analysis. The reduction in TC is driven by reduced timber loss, which was estimated by the FVS-FFE that defines timber loss and tree mortality as a function of fire severity. Our supplementary regression model for fireline intensity (S10 Table) confirms that the treatments were also effective at reducing fire intensity under high-biomass conditions. These findings suggest that fuel treatments lower fire intensity, reducing economic damage (timber loss).

### Effectiveness at the landscape level

Our results show that PB and TFB were less effective in reducing AB or TC at the landscape level than at the site level. This outcome was largely because fires burned across the treatment boundary. To lessen the chance for a fire to cross the treatment boundary, treatment area should be relatively large and/or placed in the location of high fire risk. It is beyond the scope of this study to explore the optimum size and locations of treatment areas [105]. Nevertheless, as described above, our findings reveal that TFB is a better choice than PB when there is little knowledge about where fires would occur, and that PB and TFB are effective even when a fire burns across the treatment boundary as long as the timber volume reaches its required threshold. Additionally, because large fires are more likely to burn across treatment boundaries, PB and TFB tend to be effective to mitigate AB and TC of these fires. This advantage is likely driven by TFB’s direct impact on forest structure. By removing ladder fuels, TFB is more effective than PB at reducing crown fire activity, especially in high-biomass stands (S11 Table), thereby limiting a primary driver of large-scale fire spread. As our findings in fireline intensity (S10 Table) indicates, PB and TFB can be effective in reducing burning severity, thus mitigating severe ecological impacts, as consistently supported by the literature [25,32,106].

It is challenging to precisely predict where a fire will hit. Our result shows that the effectiveness of TFB in mitigating AB and TC was less sensitive to fire ignition location (*d*) than that of PB. Hence, when it is highly uncertain about where a fire will start, TFB tends to be a better treatment option than PB. This pattern becomes more pronounced as timber volume (*Bm*) or fire occurrence year after treatment (*τ*) increases or when a fire burns at the landscape level (*Bdtn* = 1 or *Bdnt* = 1). However, we used a simplified spatial configuration by uniformly placing treatments at the center of each representative fuel landscape (RFL) and then simulating randomly distributed ignition points around the treatment boundary to assess marginal distance effects. While this controlled framework provided a consistent way to examine how effectiveness changes with ignition proximity, it does not capture the full range of real-world ignition scenarios, terrain constraints, or budget- and access-driven placement of treatments. Future studies should therefore incorporate spatial optimization of treatment locations, realistic implementation constraints, and a broader suite of ignition patterns to better reflect operational conditions.

Although this study emphasizes AB and TC, our modeling results for all metrics (AB, TC, FL, FI, CFA, and ROC) consistently reveal that PB and TFB become less effective when a fire burns across treatment boundaries, either from the treated area to the untreated area or from the opposite direction. This suggests that PB and TFB are less effective in mitigating area burned, fire intensity, and timber loss at the landscape level than at the site level.

### Effectiveness duration

Our results indicate that the relative effectiveness of fuel treatments, when compared to no treatment, grew as the time since treatment (*τ*) increased. This may seem counterintuitive, as vegetation and fuel loads regenerate over time. The model explains this dynamic through the interaction between the main effect of time and the treatment interaction terms. The main coefficient for *τ* is positive (Tables 2 and 3), confirming that in an untreated stand, AB and TC increase each year due to fuel accumulation. However, the negative coefficients for the interaction terms (*PB* × *τ* and *TFB* × *τ*) show that this increase is significantly dampened in treated areas. In other words, while fire risk increases in treated areas over time, this increase is at a much slower pace than in untreated areas. Consequently, the difference between treated and untreated areas widens over the 1–3 year period we studied, making the treatment appear more effective relative to NT in year 3 than in year 1. For a 1-year delay, TFB was 30% and 27% more effective in mitigating AB and TC than PB, respectively. This finding aligns with previous work demonstrating that TFB retains its effectiveness longer than PB [9,107].

The longer duration of TFB’s effectiveness is rooted in its impact on forest structure. By mechanically removing ladder fuels and increasing the distance between the ground and tree canopies, TFB fundamentally alters the pathways for fire to transition from the surface to the crown. These structural changes are far more persistent than the reduction of fine surface fuels achieved by PB [108,109]. Our supplementary regression analysis confirms that TFB provides a sustained mitigation of crown fire activity (S11 Table). This reduction in crown fire potential is strongly correlated with a smaller final area burned, as it limits fire spotting and the potential for rapid, uncontrollable fire growth. PB, however, is generally less effective at reducing crown bulk density and its impact on smaller surface fuels is relatively short-lived, with effectiveness generally considered highest in the first few years before fine fuels re-accumulate.

### Implication for fuel treatment operation

Our findings offer several suggestions for designing and implementing fuel treatments under real-world constraints in the southern United States. First, forest managers need to target high-biomass stands for cost effectiveness. Since both PB and TFB require minimum timber‐volume thresholds (105.3 m³/ha for PB; 147.4 m³/ha for TFB) to be economically justified at the landscape level, managers should prioritize treatments on stands exceeding these values. In regions where mean biomass is below these thresholds, PB may still effectively reduce area burned but offers limited cost savings unless non-timber benefits (e.g., carbon sequestration, wildlife habitat) are also quantified and credited in benefit–cost analyses.

Second, forest managers should prioritize treatment placement in areas with high-risk of fire. Because landscape-level effectiveness depends on intercepting large fire perimeters, treatments should be concentrated in locations where large wildfires are most likely or historically frequent. By targeting these “hot spot” zones, managers can maximize the chance that treatments will interrupt fire spread at scale—thereby improving returns on investment in both AB and TC reduction [15,24].

Third, forest managers can benefit from implementing hybrid treatment schedules. Combining an initial thinning treatment to remove ladder and canopy fuels with periodic PB can harness the structural longevity of TFB while using PB to curb fine-fuel accumulation at a lower ongoing cost. Re-treatment intervals should align with site-specific vegetation growth rates and fuel accumulation dynamics to sustain mitigation benefits over multiple years.

### Limitations and direction of future work

This study has several limitations, which suggest needs and directions for future research. We group these into three main areas: simulation framework, analytical scope and metrics, and economic simplifications.

Our simulation results are subject to the inherent assumptions of the FARSITE model, such as fuel homogeneity for each fuel type/model and the absence of fire-atmosphere interactions [77]. These assumptions may oversimplify real-world fuel variations and ignore the feedback effect of a fire on local weather conditions. Furthermore, we used a fixed circular treatment design rather than exploring multi-dimensional variations in treatment size, shape, and placement [22,110,111]. Future research can utilize optimization tools like the Treatment Optimization Model (TOM) to evaluate more complex spatial treatment patterns [24]. As this study focused on high-risk fire weather, future work can also consider a broader range of weather scenarios.

The analytical metrics of this study were primarily area burned and the total cost of fuel treatment and timber loss and, to a less extent, fire intensity. Future research can include more in-depth analysis of fuel treatment effects on fire severity and consider non-timber damages (e.g., infrastructure, housing, ecosystem services, and human health). While our use of 10 RFLs allowed for a statistically sound comparison of site- and landscape-level effectiveness, expanding this to larger or other ecosystems or accounting for spatial correlations between RFLs remains another area for future work. Additionally, as our study covered only a post-treatment period of three years, longer-term analysis is needed to identify when treatment effectiveness eventually fades out as fuels re-accumulate.

In estimating the total cost, we employed average per-hectare treatment costs and assumed the total loss of all mortality-affected timber without accounting for potential salvage revenue. In reality, treatment costs vary with stand structures and terrain conditions. Fuel treatments can also create suppression opportunities and lower suppression costs by reducing fire intensity [112,113]. Improving cost and damage estimations and linking treatments to fire suppression benefits warrant further investigation.

## Conclusions

We assessed the effectiveness of fuel treatments including PB and TFB in reducing area burned and total cost of treatment and fire-induced timber loss using a large set of fire simulation data generated from a wide range of vegetation covers, weather scenarios, and ignition locations in East Texas. We found complex relationships between fuel treatments and area burned or total cost. The effectiveness of fuel treatments in reducing the area burned and the total cost varied with treatment options and their interactions with vegetation characteristics and dynamics and fire ignition location. The effectiveness was influenced by whether fires crossed treatment boundaries as well as pre-existing timber quantity and the time lag between the treatment and fire occurrence. PB and TFB were less effective in reducing both area burned and total cost at the landscape level and at the site level where fires burn only inside the treatment area. TFB tended to outperform PB in reducing area burned and total cost with an increase in *Bm*, *d*, *τ*, and uncertainty of fire ignition location and in mitigating total cost at the landscape level.

Our findings advance the knowledge about the effectiveness of vegetation fuel treatments on mitigating wildfire risk and damage by uncovering the complex, quantitative relationships between area burned or total cost and a set of variables representing treatments and vegetation conditions as well as their interactions. Besides vegetation conditions, effectiveness of a treatment may vary with the treatment objective. These results can help improve the design and deployment of more effective fuel treatment strategies, especially at the landscape level.

## Supporting information

S1 File Wildfire simulation output and economic estimation data used for statistical analysis. (PDF)

S1 Table Descriptions of fuel models on the study site. (DOCX)

S2 Table Topography and vegetation composition in the selected representative fire locations (RFLs). (DOCX)

S3 Table Descriptions of Forest Vegetation Simulator settings for prescribed burning. (DOCX)

S4 Table Descriptions of Forest Vegetation Simulator settings for thinning from below. (DOCX)

S5 Table Descriptions of FARSITE settings. (DOCX)

S6 Table Descriptions of the Mayfield Fire and the Clear Lake Fire. (DOCX)

S7 Table Area burned based on the observed and simulated perimeters and Sorensen metrics for the Mayfield Fire and the Clear Lake Fire. (DOCX)

S8 Table Descriptions of additional variables included in the regression models for flame length, fireline intensity, crown fire activity, and rate of spread. (DOCX)

S9 Table The estimated regression model for flame length. (DOCX)

S10 Table The estimated regression model for fireline intensity. (DOCX)

S11 Table The estimated regression model for crown fire activity. (DOCX)

S12 Table The estimated regression model for rate of spread. (DOCX)

S1 Fig Observed (black) and simulated (yellow-red) fire perimeters of the Clear Lake Fire and the Mayfield Fire. (TIF)

S2 Fig Fire perimeters simulated under non-treatment (NT), prescribed burning (PB), and thinning from below (TFB) in three representative fire locations (RFLs 20, 33, and 43) after 24 hours of burning for a fire ignited inside (1st and 3rd rows) and outside (2nd and 4th rows) the treatment area one year (1st and 2nd rows) and three years (3rd and 4th rows) after treatment. (TIF)

S3 Fig Means and 95% confidence intervals (CIs) of area burned (AB) and total cost (TC = fuel treatment cost + timber loss), crown fire activity (CFA), and fireline intensity (FI) for non-treatment (NT), prescribed burning (PB), and thinning from below (TFB) after 24 hours of simulated fires in (a) all Representative Fire Locations (RFLs), (b) high-biomass RFLs (RFLs 20, 30, and 39; mean timber volume = 155.2 m3/ha), and (c) low-biomass RFLs (RFLs 16, 41, and 44; mean timber volume = 28.8 m3/ha). RFL numbers are shown in S2 Table.
(TIF)

S4 Fig Mean and 95% confidence intervals (CIs) of flame length (FL), heat per unit area (HPUA), rate of spread (ROS), and reaction intensity (RI) for non-treatment (NT), prescribed burning (PB), and thinning from below (TFB) after 24 hours of simulated fires in (a) all Representative Fire Locations (RFLs), (b) high-biomass RFLs (RFLs 20, 30, and 39; mean timber volume = 155.2 m3/ha), and (c) low-biomass RFLs (RFLs 16, 41, and 44; mean timber volume = 28.8 m3/ha). RFL numbers are shown in S2 Table.
(TIF)
