# Supplementary material for: Landscape-level effectiveness of fuel treatments in a forest-dominated ecosystem in the Southern United States
Source: PLoS One. 2026 Feb 13;21(2):e0342049. doi: 10.1371/journal.pone.0342049 (PMC12904393; doi:10.1371/journal.pone.0342049)
Supplement: S1 Table — (DOCX) [file pone.0342049.s002.docx]

**S1 Table. Descriptions of fuel models on the study site.**

| **Fuel model** | **Description** |
| --- | --- |
| FM 8 | COMPACT TIMBER LITTER. Fire usually burns slowly and lowly, but sometimes it can flare up when it meets a lot of fuel. Fire is dangerous only when the weather is very hot, dry, and windy. Fuel is mostly needles, leaves, and some twigs because there is not much undergrowth in the stand. |
| FM 9 | HARDWOOD LITTER. Fire burns faster and higher than FM 8 on the surface litter. This is common in both conifer and hardwood stands, especially oaks and hickories. Fire in fall is usually predicted in hardwoods, but it can spread faster than expected by spot fires caused by flying and rolling leaves in strong winds. Dead wood on the ground can make the fire shoot up and fly to other places. |
| FM 9 HWD | LIGHT HARDWOOD LITTER. Fire is lower for hardwood stands with a fluffy litter layer. The main difference from FM 9 is the absence of pine litter in the fuel bed component. |
| FM 9 PPL | DENSE PINE LITTER. Fire is elevated due to dense pine plantations or pine stands. This increases timber litter fuel bed depth as compared to FM 9 |
| GR 1 | SHORT, SPARSE DRY CLIMATE GRASS. Fire mainly burns sparse grass with small amounts of fine dead fuel. The grass in GR 1 is usually short and thin, naturally or by grazing. |
| GR 2 | LOW LOAD DRY CLIMATE GRASS. Fire mainly burns grass with small amounts of fine dead fuel. Fuel load is greater than in GR 1. Fuel may be more connected than in GR 1. |
| GR 3 | LOW LOAD VERY COARSE HUMID CLIMATE GRASS. Fire mainly burns wet-climate grass that is continuous and coarse. Grass and herb fuel load is relatively light and fuel bed depth is about two feet. |
| GS 2 | MODERATE LOAD DRY CLIMATE GRASS-SHRUB. Fire mainly burns grass and shrubs combined. The shrubs are 1-3 feet high; grass load is moderate. The spread rate is high, flame length moderate. Moisture level is low. |
| GS 3 | MODERATE LOAD HUMID CLIMATE GRASS-SHRUB. Fire mainly burns grass and shrubs combined. The vegetation is similar to GS 2, but moisture level is higher. |
| NB 91 | URBAN/SUBURBAN. This consists of urban and suburban development. |
| NB 93 | AGRICULTURAL FIELD. This is agricultural land under non-burnable condition; examples include irrigated annual crops, mowed or tilled orchards, and so forth. |
| NB 98 | OPEN WATER. Land covered by open bodies of water such as lakes, rivers, and oceans. |
| NB 99 | BARE GROUND. Land devoid of enough fuel to support wildland fire spread. Such areas may include gravel pits, arid deserts with little vegetation, sand dunes, rock outcroppings, beaches, and so forth. |

^a^ Sources: [41–43]
