# Supplementary material for: Landscape-level effectiveness of fuel treatments in a forest-dominated ecosystem in the Southern United States
Source: PLoS One. 2026 Feb 13;21(2):e0342049. doi: 10.1371/journal.pone.0342049 (PMC12904393; doi:10.1371/journal.pone.0342049)
Supplement: S2 Table — (DOCX) [file pone.0342049.s003.docx]

**S2 Table.** **Topography and vegetation composition in the selected representative fire locations (RFLs).**

| **RFL**^a^ | **Topography** | | **Vegetation type (%)** | | |
| --- | --- | --- | --- | --- | --- |
|  | **Elevation (m)** | **Slope (degree)** | **Forestland** | **Grassland** | **Non-burnable** |
| 10 | 153.8 | 21.7 | 62.9 | 28.2 | 8.9 |
| 16 | 157.6 | 21.8 | 34.1 | 36.9 | 28.9 |
| 20 | 106.2 | 21.6 | 61.9 | 33.1 | 4.9 |
| 30 | 80.2 | 20.0 | 54.0 | 36.0 | 10.0 |
| 32 | 113.5 | 21.7 | 52.3 | 31.8 | 15.9 |
| 33 | 139.3 | 21.6 | 43.1 | 43.5 | 13.4 |
| 39 | 73.3 | 11.8 | 63.9 | 24.6 | 11.6 |
| 41 | 82.4 | 15.2 | 40.7 | 52.0 | 7.3 |
| 43 | 148.7 | 18.3 | 8.4 | 78.7 | 12.9 |
| 44 | 116.9 | 20.3 | 28.0 | 65.7 | 6.3 |

^a^ RFL numbers and locations are shown in Fig 1.
