# Supplementary material for: Landscape-level effectiveness of fuel treatments in a forest-dominated ecosystem in the Southern United States
Source: PLoS One. 2026 Feb 13;21(2):e0342049. doi: 10.1371/journal.pone.0342049 (PMC12904393; doi:10.1371/journal.pone.0342049)
Supplement: S3 Table — (DOCX) [file pone.0342049.s004.docx]

**S3 Table. Descriptions of Forest Vegetation Simulator settings for prescribed burning.**

| **Variable** | **Selected value** |
| --- | --- |
| Wind speed at 20 feet (6.1 m) above the vegetation | 4.5 m/s |
| Moisture level for all fuels | 2 = Very dry |
| Temperature | 50 °F (10 °C) |
| Mortality code | 1 = FFE^a^ estimates mortality |
| Percentage of stand area burned | 70% |
| Season of this fire | 1 = Early Spring |

^a^ FFE stands for the Fire and Fuels Extension to the Forest Vegetation Simulator**.**
