# Supplementary material for: Landscape-level effectiveness of fuel treatments in a forest-dominated ecosystem in the Southern United States
Source: PLoS One. 2026 Feb 13;21(2):e0342049. doi: 10.1371/journal.pone.0342049 (PMC12904393; doi:10.1371/journal.pone.0342049)
Supplement: S4 Table — (DOCX) [file pone.0342049.s005.docx]

**S4 Table. Descriptions of Forest Vegetation Simulator settings for thinning from below.**

| **Variable** | **Selected value** |
| --- | --- |
| Residual density | 14 m^2^/ha |
| Density is in terms of | Basal area |
| Smallest DBH (Diameter at Breast Hight) to be considered for removal | No restriction |
| Largest DBH to be considered for removal | No restriction |
| Proportion of small trees left | 0% |
| Slash left | No slash left |
