# Supplementary material for: Landscape-level effectiveness of fuel treatments in a forest-dominated ecosystem in the Southern United States
Source: PLoS One. 2026 Feb 13;21(2):e0342049. doi: 10.1371/journal.pone.0342049 (PMC12904393; doi:10.1371/journal.pone.0342049)
Supplement: S5 Table — (DOCX) [file pone.0342049.s006.docx]

**S5 Table. Descriptions of FARSITE settings.**

| **Variable** | **Description** | **Set value** |
| --- | --- | --- |
| Timestep | Maximum amount of time that conditions at a given point are assumed to be constant so that the position of the fire front can be projected | 60 minutes |
| Distance resolution | Distance at which the FARSITE model checks for new fire characteristics within a time step | 30 m |
| Perimeter resolution | Distance between ignition vertices placed along the fire perimeter at each time step | 60 m |
| Minimum ignition vertex distance | Minimum distance between fire ignition vertices placed along the given ignition shapefile | 15 m |
| Spot grid resolution | Resolution of the background spotting grid | 15 m |
| Spot probability | Probability that a spot causes an ignition | 0.05 |
| Spot ignition delay | Time to delay an ignition caused by spotting | No delay |
| Minimum spot distance | Minimum distance a spot must travel to start a new fire | 30 m |
| Foliar moisture content | Fuel moisture content | 100% |
| Crown fire method | Methods to calculate crown fire potential | Finney |
