# Supplementary material for: Landscape-level effectiveness of fuel treatments in a forest-dominated ecosystem in the Southern United States
Source: PLoS One. 2026 Feb 13;21(2):e0342049. doi: 10.1371/journal.pone.0342049 (PMC12904393; doi:10.1371/journal.pone.0342049)
Supplement: S6 Table — (DOCX) [file pone.0342049.s007.docx]

**S6 Table. Descriptions of the Mayfield Fire and the Clear Lake Fire.**

| **Descriptor** | **Mayfield Fire** | **Clear Lake Fire** |
| --- | --- | --- |
| County | San Saba, Texas | Panola, Texas |
| Dominant vegetation type | Grassland | Forestland |
| Ignition location (longitude, latitude) | -94.084, 32.051 | -99.075, 31.039 |
| Fuel moisture | 1-hr: 8%  10-hr: 9%  100-hr: 13%  Herbaceous: 8%  Woody: 60% | 1-hr: 9%  10-hr: 10%  100-hr: 15%  Herbaceous: 30%  Woody: 70% |
| Ignition start time | May-12-2022 14:00 | Aug-20- 2023 10:00 |
| Perimeter record time | May-12-2022 20:00 | Aug-21-2023 7:00 |
| Area burned when recorded | 553.8 ha | 305.9 ha |
| Nearest RAWS name | Coleman | Henderson |
| Mean air temperature | 34.9 ℃ | 33.6 ℃ |
| Mean relative humidity | 21.0% | 45.3% |
| Mean wind speed | 20.0 km/h | 18.7 km/h |
