# Supplementary material for: Landscape-level effectiveness of fuel treatments in a forest-dominated ecosystem in the Southern United States
Source: PLoS One. 2026 Feb 13;21(2):e0342049. doi: 10.1371/journal.pone.0342049 (PMC12904393; doi:10.1371/journal.pone.0342049)
Supplement: S7 Table — (DOCX) [file pone.0342049.s008.docx]

**S7 Table. Area burned based on the observed and simulated perimeters and Sorensen metrics for the Mayfield Fire and the Clear Lake Fire.**

| **Variable** | **Mayfield Fire** | **Clear Lake Fire** |
| --- | --- | --- |
| Observed area burned (*A* + *B*) | 553.8 (100.0)^a^ | 305.9 (100.0) |
| Simulated area burned (*A* + *C*) | 557.8 (100.7) | 293.4 (95.9) |
| Correct estimate (*A*) | 425.6 (76.9) | 203.0 (66.4) |
| Underestimate (*B*) | 128.2 (23.1) | 102.9 (33.6) |
| Overestimate (*C*) | 132.2 (23.9) | 90.4 (30.0) |
| Sorensen metric ($2A/(2A+B+C)$) | 0.76 | 0.68 |

^a^ The unit for area burned is ha, and the values inside parentheses are percentage.
