# Supplementary material for: Landscape-level effectiveness of fuel treatments in a forest-dominated ecosystem in the Southern United States
Source: PLoS One. 2026 Feb 13;21(2):e0342049. doi: 10.1371/journal.pone.0342049 (PMC12904393; doi:10.1371/journal.pone.0342049)
Supplement: S8 Table — (DOCX) [file pone.0342049.s009.docx]

**S8 Table. Descriptions of additional variables included in the regression models for flame length, fireline intensity, crown fire activity, and rate of spread.**

| **Variable** | **Description** | **Min**^a^ | **Mean  (Std Dev)**^a^ | **Max**^a^ |
| --- | --- | --- | --- | --- |
| Response variables | |  |  |  |
| *FL* | Hourly average flame length, derived from FARSITE simulations (m) | 0 | 0.6 (0.6) | 7.9 |
| *FI* | Hourly average fireline intensity, derived from FARSITE simulations (kW/m) | 0 | 208.4 (408.5) | 6437.4 |
| *CFA* | Hourly cumulative active crown fire activity, derived from FARSITE simulations (case) | 0 | 86.0 (436.6) | 8793.0 |
| *ROS* | Hourly average rate of spread, derived from FARSITE simulations (m/min) | 0 | 1.2 (24.7) | 10886.5 |
