# Supplementary material for: Landscape-level effectiveness of fuel treatments in a forest-dominated ecosystem in the Southern United States
Source: PLoS One. 2026 Feb 13;21(2):e0342049. doi: 10.1371/journal.pone.0342049 (PMC12904393; doi:10.1371/journal.pone.0342049)
Supplement: S9 Table — (DOCX) [file pone.0342049.s010.docx]

**S9 Table. The estimated regression model for flame length.**

| **Variable**^a^ | **Estimated *β*** | **Std Error** | **p-value** | **VIF** |
| --- | --- | --- | --- | --- |
| *Intercept* | -6.406 | 0.200 | < 0.001 |  |
| *Fire duration (h)* | -0.029 | 0.001 | < 0.001 | 1.556 |
| *Relative humidity (RH)* | -0.066 | 0.000 | < 0.001 | 1.291 |
| *Wind speed (WS)* | 0.172 | 0.003 | < 0.001 | 1.181 |
| *Temperature (T)* | 0.028 | 0.001 | < 0.001 | 1.040 |
| *Prescribed burning (PB)* | -0.119 | 0.016 | < 0.001 | 2.889 |
| *Thinning from below (TFB)* | -0.071 | 0.018 | < 0.001 | 4.061 |
| *Fire spreading from the treatment area to the non-treatment area (Bdtn)* | 0.905 | 0.019 | < 0.001 | 4.027 |
| *Fire spreading from the non-treatment area to the treatment area (Bdnt)* | 0.915 | 0.020 | < 0.001 | 3.966 |
| *Timber volume (Bm)* | 0.000 | 0.000 | < 0.001 | 1.528 |
| *PB⨯Bdtn* | 0.365 | 0.026 | < 0.001 | 2.961 |
| *TFB⨯Bdtn* | 0.342 | 0.026 | < 0.001 | 2.953 |
| *PB⨯Bdnt* | -0.194 | 0.027 | < 0.001 | 3.215 |
| *TFB⨯Bdnt* | -0.133 | 0.027 | < 0.001 | 3.366 |
| *TFB⨯Bm* | 0.000 | 0.000 | 0.127 | 2.691 |
| *PB⨯d* | 0.917 | 0.025 | < 0.001 | 1.528 |
| *TFB⨯d* | 0.802 | 0.025 | < 0.001 | 1.587 |

^a^ All variables are described in Table 1 and S8 Table with ⨯ denoting the interaction between two variables.
The model was a significant improvement over the intercept-only model (Likelihood-ratio test: χ^2^ (16) = 120,676, p < 0.001). A Nagelkerke's pseudo-R² of 0.383 indicates a moderate model fit. VIF is the variance inflation factor.
