# Supplementary material for: Landscape-level effectiveness of fuel treatments in a forest-dominated ecosystem in the Southern United States
Source: PLoS One. 2026 Feb 13;21(2):e0342049. doi: 10.1371/journal.pone.0342049 (PMC12904393; doi:10.1371/journal.pone.0342049)
Supplement: S10 Table — (DOCX) [file pone.0342049.s011.docx]

**S10 Table. The estimated regression model for fireline intensity.**

| **Variable**^a^ | **Estimated *β*** | **Std Error** | **p-value** | **VIF** |
| --- | --- | --- | --- | --- |
| *Intercept* | -6.518 | 0.360 | < 0.001 |  |
| *Fire duration (h)* | -0.057 | 0.001 | < 0.001 | 1.557 |
| *Relative humidity (RH)* | -0.122 | 0.000 | < 0.001 | 1.291 |
| *Wind speed (WS)* | 0.320 | 0.005 | < 0.001 | 1.180 |
| *Temperature (T)* | 0.052 | 0.001 | < 0.001 | 1.040 |
| *Prescribed burning (PB)* | -0.216 | 0.028 | < 0.001 | 2.888 |
| *Thinning from below (TFB)* | -0.131 | 0.033 | < 0.001 | 4.061 |
| *Fire spreading from the treatment area to the non-treatment area (Bdtn)* | 1.738 | 0.034 | < 0.001 | 4.033 |
| *Fire spreading from the non-treatment area to the treatment area (Bdnt)* | 1.771 | 0.036 | < 0.001 | 3.974 |
| *Timber volume (Bm)* | 0.001 | 0.000 | < 0.001 | 1.529 |
| *Delay in fire occurrence after treatment (τ)* | 0.018 | 0.009 | 0.053 | 1.003 |
| *PB⨯Bdtn* | 0.591 | 0.047 | < 0.001 | 2.962 |
| *TFB⨯Bdtn* | 0.645 | 0.047 | < 0.001 | 2.957 |
| *PB⨯Bdnt* | -0.326 | 0.049 | < 0.001 | 3.219 |
| *TFB⨯Bdnt* | -0.210 | 0.049 | < 0.001 | 3.370 |
| *TFB⨯Bm* | 0.000 | 0.000 | 0.141 | 2.694 |
| *PB⨯d* | 1.619 | 0.044 | < 0.001 | 1.528 |
| *TFB⨯d* | 1.391 | 0.045 | < 0.001 | 1.588 |

^a^ All variables are described in Table 1 and S8 Table with ⨯ denoting the interaction between two variables.
The model was a significant improvement over the intercept-only model (Likelihood-ratio test: χ^2^ (17) = 126,984, p < 0.001). A Nagelkerke's pseudo-R² of 0.397 indicates a moderate model fit. VIF is the variance inflation factor.
