# Supplementary material for: Landscape-level effectiveness of fuel treatments in a forest-dominated ecosystem in the Southern United States
Source: PLoS One. 2026 Feb 13;21(2):e0342049. doi: 10.1371/journal.pone.0342049 (PMC12904393; doi:10.1371/journal.pone.0342049)
Supplement: S11 Table — (DOCX) [file pone.0342049.s012.docx]

**S11 Table. The estimated regression model for crown fire activity.**

| **Variable**^a^ | **Estimated *β*** | **Std Error** | **p-value** | **VIF** |
| --- | --- | --- | --- | --- |
| *Intercept* | 1.865 | 0.341 | < 0.001 |  |
| *Fire duration (h)* | 0.019 | 0.001 | < 0.001 | 1.557 |
| *Relative humidity (RH)* | -0.010 | 0.000 | < 0.001 | 1.291 |
| *Wind speed (WS)* | -0.130 | 0.005 | < 0.001 | 1.181 |
| *Temperature (T)* | -0.029 | 0.001 | < 0.001 | 1.040 |
| *Prescribed burning (PB)* | 0.122 | 0.034 | < 0.001 | 4.634 |
| *Fire spreading from the treatment area to the non-treatment area (Bdtn)* | 2.314 | 0.031 | < 0.001 | 3.836 |
| *Fire spreading from the non-treatment area to the treatment area (Bdnt)* | 2.749 | 0.034 | < 0.001 | 3.689 |
| *Timber volume (Bm)* | 0.005 | 0.000 | < 0.001 | 2.675 |
| *Delay in fire occurrence after treatment (τ)* | 0.879 | 0.014 | < 0.001 | 2.559 |
| *PB⨯Bdtn* | -0.934 | 0.044 | < 0.001 | 2.891 |
| *TFB⨯Bdtn* | -0.847 | 0.041 | < 0.001 | 2.649 |
| *PB⨯Bdnt* | -0.140 | 0.046 | 0.002 | 3.099 |
| *TFB⨯Bdnt* | 0.122 | 0.044 | 0.006 | 2.779 |
| *PB⨯Bm* | -0.003 | 0.000 | < 0.001 | 3.379 |
| *TFB⨯Bm* | -0.001 | 0.000 | < 0.001 | 2.732 |
| *PB⨯τ* | -0.203 | 0.021 | < 0.001 | 3.708 |
| *TFB⨯τ* | -0.167 | 0.018 | < 0.001 | 2.805 |
| *PB⨯d* | 0.273 | 0.042 | < 0.001 | 1.530 |
| *TFB⨯d* | -0.275 | 0.043 | < 0.001 | 1.578 |

^a^ All variables are described in Table 1 and S8 Table with ⨯ denoting the interaction between two variables.
The model was a significant improvement over the intercept-only model (Likelihood-ratio test: χ^2^ (19) = 40,578, p < 0.001). A Nagelkerke's pseudo-R² of 0.149 indicates a weak model fit. . VIF is the variance inflation factor.
