# Supplementary material for: Landscape-level effectiveness of fuel treatments in a forest-dominated ecosystem in the Southern United States
Source: PLoS One. 2026 Feb 13;21(2):e0342049. doi: 10.1371/journal.pone.0342049 (PMC12904393; doi:10.1371/journal.pone.0342049)
Supplement: S12 Table — (DOCX) [file pone.0342049.s013.docx]

**S12 Table. The estimated regression model for rate of spread.**

| **Variable**^a^ | **Estimated *β*** | **Std Error** | **p-value** | **VIF** |
| --- | --- | --- | --- | --- |
| *Intercept* | -8.547 | 0.215 | < 0.001 |  |
| *Fire duration (h)* | -0.041 | 0.001 | < 0.001 | 1.557 |
| *Relative humidity (RH)* | -0.073 | 0.000 | < 0.001 | 1.290 |
| *Wind speed (WS)* | 0.280 | 0.003 | < 0.001 | 1.180 |
| *Temperature (T)* | 0.038 | 0.001 | < 0.001 | 1.040 |
| *Prescribed burning (PB)* | -0.147 | 0.019 | < 0.001 | 3.942 |
| *Thinning from below (TFB)* | -0.081 | 0.017 | < 0.001 | 2.929 |
| *Fire spreading from the treatment area to the non-treatment area (Bdtn)* | 0.899 | 0.020 | < 0.001 | 4.033 |
| *Fire spreading from the non-treatment area to the treatment area (Bdnt)* | 0.938 | 0.022 | < 0.001 | 3.974 |
| *Timber volume (Bm)* | -0.002 | 0.000 | < 0.001 | 1.524 |
| *Delay in fire occurrence after treatment (τ)* | 0.010 | 0.006 | 0.071 | 1.003 |
| *PB⨯Bdtn* | 0.377 | 0.028 | < 0.001 | 2.970 |
| *TFB⨯Bdtn* | 0.326 | 0.028 | < 0.001 | 2.951 |
| *PB⨯Bdnt* | -0.216 | 0.029 | < 0.001 | 3.217 |
| *TFB⨯Bdnt* | -0.137 | 0.029 | < 0.001 | 3.373 |
| *PB⨯Bm* | 0.001 | 0.000 | < 0.001 | 2.696 |
| *PB⨯d* | 0.922 | 0.026 | < 0.001 | 1.528 |
| *TFB⨯d* | 0.808 | 0.027 | < 0.001 | 1.587 |

^a^ All variables are described in Table 1 and S8 Table with ⨯ denoting the interaction between two variables.
The model was a significant improvement over the intercept-only model (Likelihood-ratio test: χ^2^ (17) = 133,050, p < 0.001). A Nagelkerke's pseudo-R² of 0.414 indicates a moderate model fit. VIF is the variance inflation factor.
