# Supplementary figures and images for: Landscape-level effectiveness of fuel treatments in a forest-dominated ecosystem in the Southern United States
Source: PLoS One. 2026 Feb 13;21(2):e0342049. doi: 10.1371/journal.pone.0342049 (PMC12904393; doi:10.1371/journal.pone.0342049)

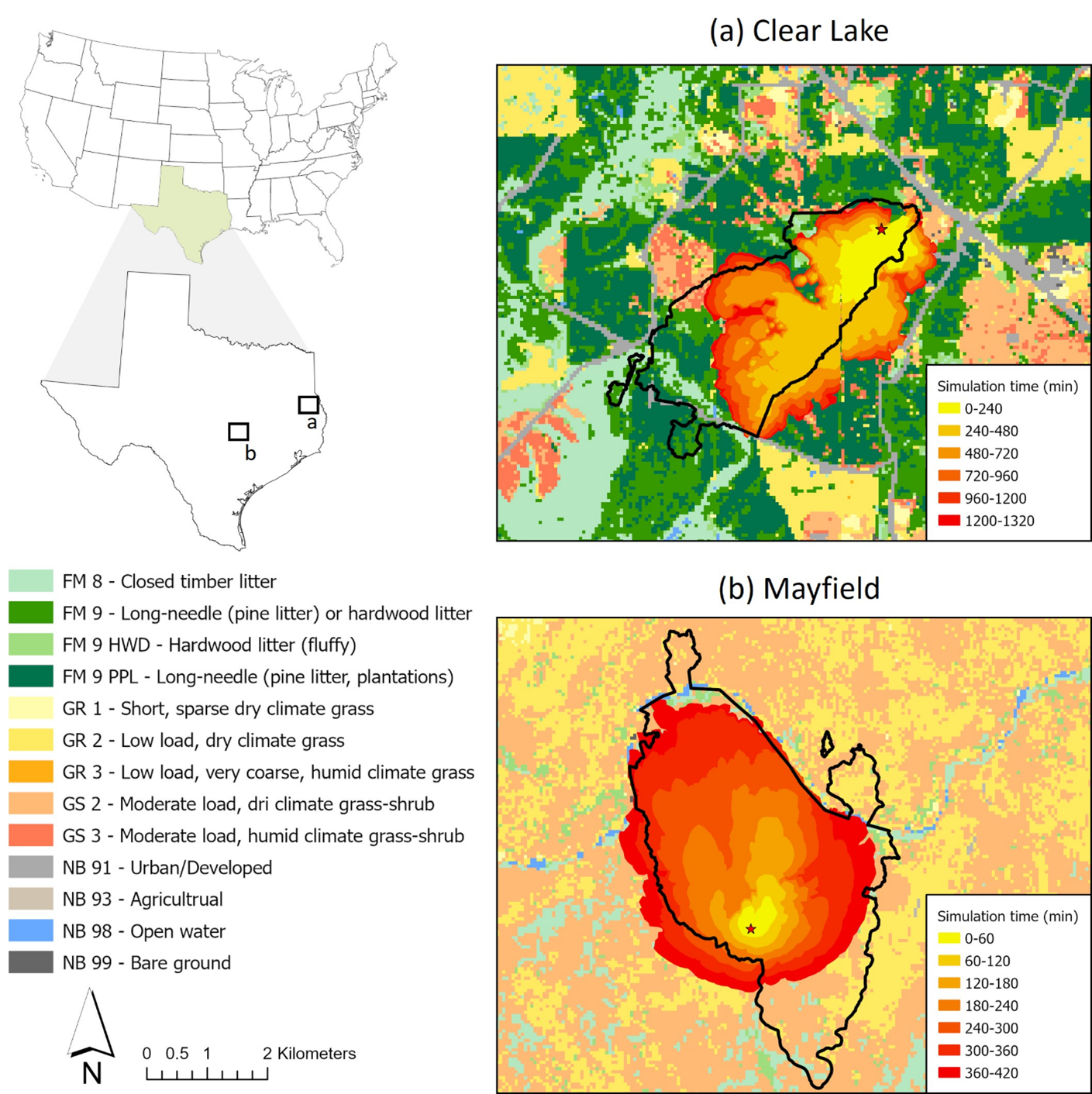

Supplement: S1 Fig — (TIF) [file pone.0342049.s014.tif]

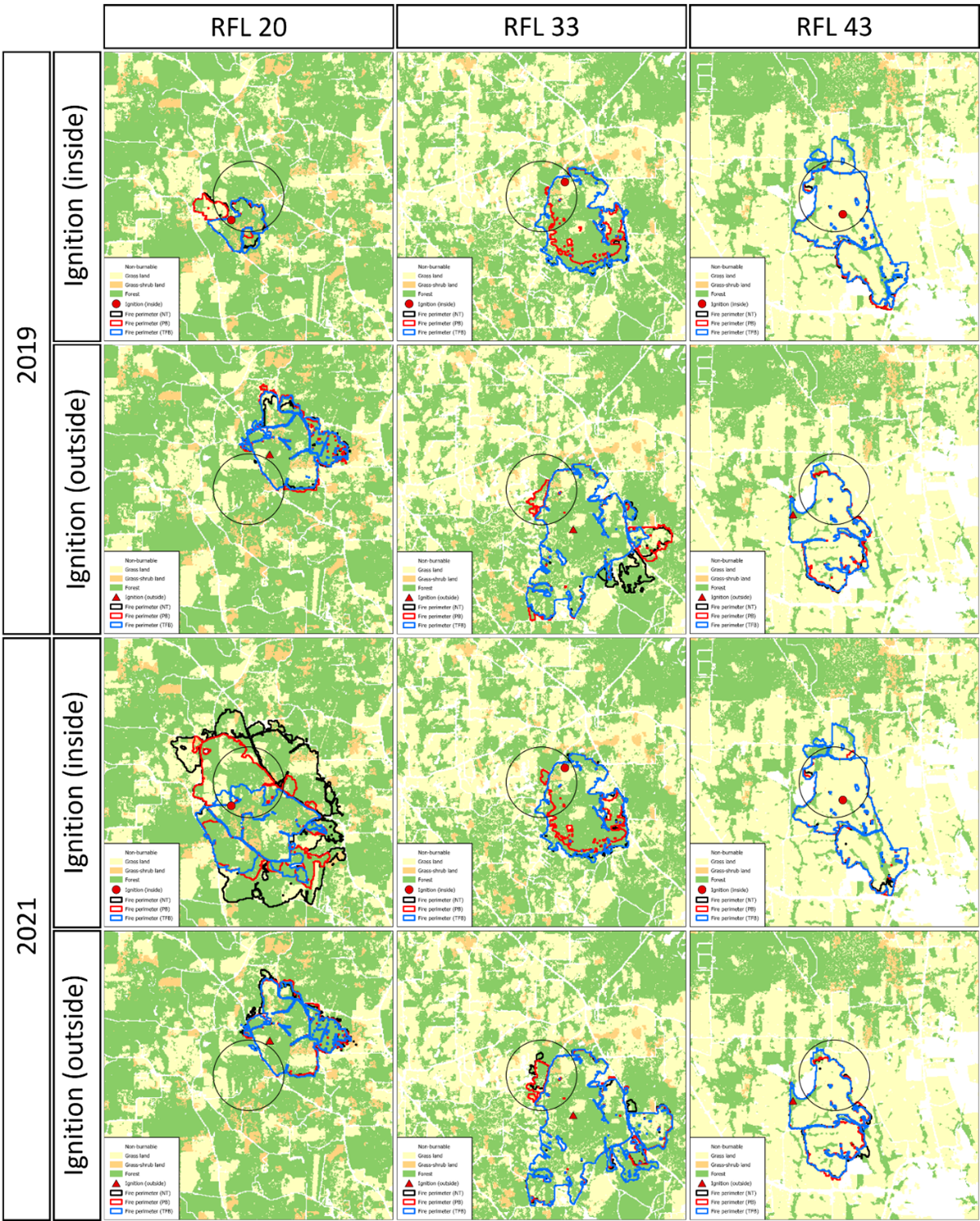

Supplement: S2 Fig — (TIF) [file pone.0342049.s015.tif]

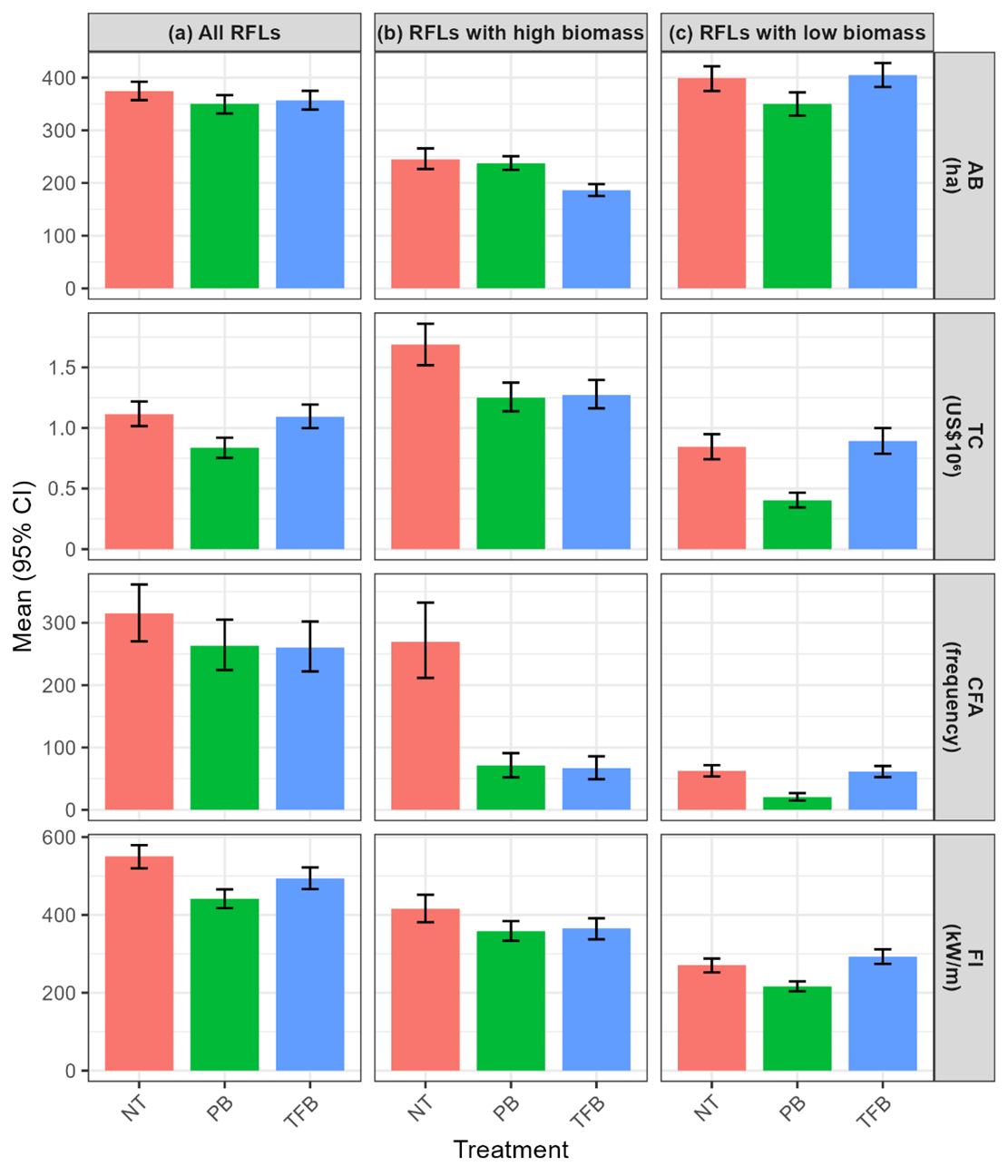

Supplement: S3 Fig — RFL numbers are shown in S2 Table. (TIF) [file pone.0342049.s016.tif]

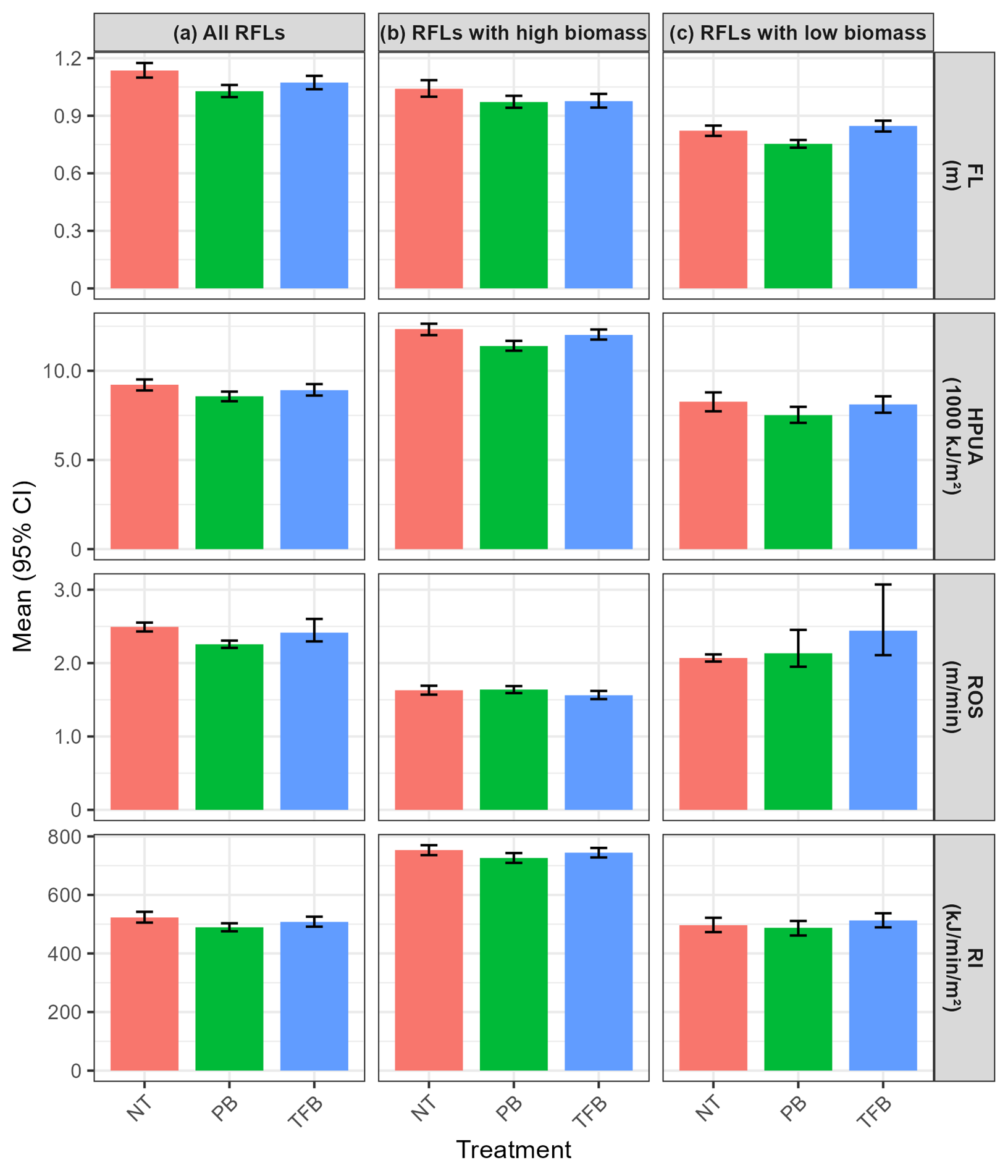

Supplement: S4 Fig — RFL numbers are shown in S2 Table. (TIF) [file pone.0342049.s017.tif]
