# Supplementary material for: Landscape-level effectiveness of fuel treatments in a forest-dominated ecosystem in the Southern United States
Source: PLoS One. 2026 Feb 13;21(2):e0342049. doi: 10.1371/journal.pone.0342049 (PMC12904393; doi:10.1371/journal.pone.0342049)
Supplement: S1 File — (PDF) [file pone.0342049.s001.pdf]

S1 File. Wildfire simulation output and economic estimation data used for statistical analysis

File name: Dataset.csv

This dataset was used for:

1) Generating summary statistics (see Table 1 of the manuscript)
2) Fitting Gaussian Generalized Linear Model to analyze the effects of fuel treatments, vegetation, ignition, and weather variables on:
Area burned (AB), Total cost (TC), Flame Length (FL), Fireline Intensity (FI), Rate of Spread (ROS), and Crown Fire Activity (CFA)
3) Ggenerating S3 and S4 Figs of supporting information

AB	TC	FL	FI	ROS	CFA	log_AB	log_TC	log_FL	log_FI	log_ROS	log_CFA	h	RH	WS	T_inK	T_inC	PB	TFB	Bdtn	Bdnt	Bm	tau	d
0.0216	471.980038	0.70962657	143.462721	1.97734169	0	-3.835062	-0.7487021	-0.3416082	4.96608219	0.68225897	-6.9077553	0	82	4.91742778	299.666667	26.66666666	0	0	0	0	30.519848	0	0.21141735
0.0702	1042.21998	0.4607627	58.319874	1.69561174	0	-2.656407	0.04231207	-0.7727042	4.06596007	0.52863317	-6.9077553	1	74	5.36446667	301.333333	28.33333333	0	0	0	0	30.519848	0	0.21141735
0.1386	2096.19366	0.58175222	97.5213302	1.54484016	0	-1.9761632	0.7406001	-0.5399932	4.58008138	0.43556756	-6.9077553	2	68	5.36446667	302.444444	29.44444444	0	0	0	0	30.519848	0	0.21141735
0.2421	2988.14786	0.59852195	107.519778	2.39876205	0	-1.4184044	1.09498835	-0.5116227	4.67768411	0.87536959	-6.9077553	3	63	5.81150556	303.555556	30.55555555	0	0	0	0	30.519848	0	0.21141735
0.3627	3783.48676	0.64131404	112.674885	2.17027283	0	-1.0141792	1.33091028	-0.4426779	4.72451542	0.77531355	-6.9077553	4	60	4.91742778	304.666667	31.66666666	0	0	0	1	30.519848	0	0.21141735
0.5526	9272.29483	0.79261773	184.725822	2.26017233	0	-0.5931209	2.22713875	-0.2311534	5.2188781	0.81588341	-6.9077553	5	55	4.91742778	306.888889	33.88888888	0	0	0	1	30.519848	0	0.21141735
0.8541	19291.5234	0.84259625	213.264482	2.95293151	0	-0.157707	2.95971763	-0.1700813	5.36253779	1.083137	-6.9077553	6	51	5.81150556	307.444444	34.4444444	0	0	0	1	30.519848	0	0.21141735
1.1988	29819.9731	0.71694683	141.476787	2.04744965	0	0.18132106	3.39521194	-0.3313598	4.95214272	0.71708324	-6.9077553	7	49	5.36446667	308.555556	35.5555556	0	0	0	1	30.519848	0	0.21141735
1.4355	38475.6439	0.69905804	135.553826	1.54123331	0	0.36151322	3.6500514	-0.356592	4.90937618	0.43323157	-6.9077553	8	52	4.47038889	306.888889	33.88888888	0	0	0	1	30.519848	0	0.21141735
1.6506	47016.7183	0.70020419	136.716544	1.36520194	0	0.50113886	3.85052452	-0.3549562	4.91791707	0.31203458	-6.9077553	9	52	4.47038889	307.444444	34.4444444	0	0	0	1	30.519848	0	0.21141735
1.8378	56199.0604	0.67815245	131.453651	1.09736303	0	0.6085692	4.02891783	-0.3869097	4.87866193	0.09382092	-6.9077553	10	54	3.57631111	306.888889	33.88888888	0	0	0	1	30.519848	0	0.21141735
1.9692	64542.1627	0.65386728	123.539303	0.93681011	0	0.67762737	4.16733419	-0.4233227	4.81656745	-0.0642078	-6.9077553	11	58	2.68223333	305.777778	32.7777778	0	0	0	1	30.519848	0	0.21141735
2.0628	73384.0925	0.70874346	134.60376	0.90510999	0	0.72406428	4.29572081	-0.3428517	4.90234278	-0.0985946	-6.9077553	12	65	1.78815556	304.111111	31.11111111	0	0	0	1	30.519848	0	0.21141735
2.133	80512.0758	0.74685879	149.770107	1.05868188	0	0.75752944	4.3884196	-0.2905411	5.00910818	0.05796875	-6.9077553	13	70	1.78815556	303	30	0	0	0	1	30.519848	0	0.21141735
2.1879	86520.6632	0.59965646	93.9909148	0.79194916	0	0.78294218	4.46039482	-0.5097321	4.54320877	-0.2319962	-6.9077553	14	77	0.44703889	301.888889	28.88888888	0	0	0	1	30.519848	0	0.21141735
2.2149	87661.1288	0.45886625	50.9011922	0.70422961	0	0.79520726	4.47348998	-0.7768196	3.92990599	-0.3492318	-6.9077553	15	75	0.89407778	297.444444	24.44444444	0	0	0	1	30.519848	0	0.21141735
2.2464	88845.4526	0.47067212	56.1246512	0.82025125	0	0.80932893	4.48690963	-0.7514712	4.02759295	-0.1969262	-6.9077553	16	88	0.89407778	295.222222	22.2222222	0	0	0	1	30.519848	0	0.21141735
2.2635	89428.3125	0.3618807	36.1155139	0.60455207	0	0.81691229	4.49344851	-1.0136811	3.58675021	-0.5016147	-6.9077553	17	46	2.23519444	296.888889	23.88888888	0	0	0	1	30.519848	0	0.21141735
2.5074	98747.8873	0.5951792	111.2364	1.46126891	0	0.91924636	4.59258014	-0.517214	4.71166666	0.37998928	-6.9077553	18	42	2.23519444	296.333333	23.33333333	0	0	0	1	30.519848	0	0.21141735
2.844	108086.651	0.64785807	138.103046	2.20866565	0	1.04521151	4.68294249	-0.4325413	4.92800735	0.79284122	-6.9077553	19	35	3.12927222	295.777778	22.7777778	0	0	0	1	30.519848	0	0.21141735
3.2256	119343.665	0.71865348	151.355432	2.3839595	0	1.17111898	4.78201565	-0.3289855	5.01963753	0.86918214	-6.9077553	20	36	4.91742778	294.666667	21.66666666	0	0	0	1	30.519848	0	0.21141735
3.5145	130384.41	0.62478077	111.867382	1.45140508	0	1.25689727	4.87049476	-0.4687552	4.71732302	0.37322086	-6.9077553	21	43	2.68223333	293	20	0	0	0	1	30.519848	0	0.21141735
3.6333	143490.379	0.5736592	97.2171076	0.7459785	0	1.29014133	4.96627495	-0.5539781	4.57695699	-0.2917189	-6.9077553	22	54	0.44703889	290.222222	17.2222222	0	0	0	1	30.519848	0	0.21141735
3.7404	154250.48	0.60186005	107.519966	0.79709606	0	1.31919256	5.03858426	-0.5060702	4.67768586	-0.2255263	-6.9077553	23	55	1.34111667	291.333333	18.33333333	0	0	0	1	30.519848	0	0.21141735
0.0324	1215.8595	0.99302063	277.123438	2.9633861	0	-3.4295969	0.19627336	-0.0059973	5.62446664	1.08666996	-6.9077553	0	82	4.91742778	299.666667	26.66666666	0	0	0	0	34.0724638	0	0.49277013
0.0918	3637.46773	0.7780812	160.712757	1.24610173	0	-2.388143	1.29156264	-0.24964	5.07962488	0.22082224	-6.9077553	1	74	5.36446667	301.333333	28.33333333	0	0	0	0	34.0724638	0	0.49277013
0.1683	7188.1518	0.85173816	189.33102	1.25685945	0	-1.7820072	1.9725732	-0.1593027	5.24350219	0.22941143	-6.9077553	2	68	5.36446667	302.444444	29.44444444	0	0	0	0	34.0724638	0	0.49277013
0.2808	11729.906	0.95212158	248.502094	2.2598457	0	-1.2701126	2.4622269	-0.0480128	5.5154553	0.81573895	-6.9077553	3	63	5.81150556	303.555556	30.55555555	0	0	0	1	34.0724638	0	0.49277013
0.5832	25597.6086	1.22377795	446.576823	3.55338978	0	-0.5392251	3.242538	0.20275956	6.10161368	1.2681834	-6.9077553	4	60	4.91742778	304.666667	31.66666666	0	0	0	1	34.0724638	0	0.49277013
1.2429	46342.9215	1.45458285	642.004221	5.09093889	1	0.21744736	3.83609014	0.3754064	6.46459644	1.62765868	0.0009995	5	55	4.91742778	306.888889	33.88888888	0	0	0	1	34.0724638	0	0.49277013
2.0835	65076.6378	1.35530355	578.500401	4.6709366	2	0.73404917	4.17558098	0.30476302	6.36044097	1.54157368	0.69364706	6	51	5.81150556	307.444444	34.4444444	0	0	0	1	34.0724638	0	0.49277013
2.7243	88078.9429	1.09382595	351.285156	2.76946919	3	1.00221151	4.47824484	0.0905954	5.86160115	1.01901669	1.09894557	7	49	5.36446667	308.555556	35.5555556	0	0	0	1	34.0724638	0	0.49277013
3.3993	114729.572	1.02101685	293.287396	2.56689893	3	1.22356953	4.74258653	0.02177797	5.68115641	0.94308803	1.09894557	8	52	4.47038889	306.888889	33.88888888	0	0	0	1	34.0724638	0	0.49277013
4.1445	139301.954	0.96367649	264.167686	2.86275228	3	1.42178215	4.93665109	-0.0359625	5.57658786	1.05213275	1.09894557	9	52	4.47038889	307.444444	34.4444444	0	0	0	1	34.0724638	0	0.49277013
4.6935	155370.601	0.82104297	195.611885	1.89980409	3	1.54617857	5.04581968	-0.1959626	5.27613763	0.642277	1.09894557	10	54	3.57631111	306.888889	33.88888888	0	0	0	1	34.0724638	0	0.49277013
5.0184	166392.254	0.74533681	165.598554	1.43862061	3	1.61311116	5.11435399	-0.2925783	5.10957255	0.36437962	1.09894557	11	58	2.68223333	305.777778	32.7777778	0	0	0	1	34.0724638	0	0.49277013
5.2362	174842.495	0.79275866	175.974206	1.27219258	3	1.65559604	5.16389126	-0.2309758	5.17034311	0.24152759	1.09894557	12	65	1.78815556	304.111111	31.11111111	0	0	0	1	34.0724638	0	0.49277013
5.409	181674.33	0.74499464	149.474752	1.2571346	3	1.68806423	5.20222119	-0.2930369	5.00713418	0.22963015	1.09894557	13	70	1.78815556	303	30	0	0	0	1	34.0724638	0	0.49277013
5.5575	187709.667	0.69683597	126.748248	1.11833126	3	1.71514837	5.23490177	-0.3597712	4.84221071	0.11273142	1.09894557	14	77	0.44703889	301.888889	28.88888888	0	0	0	1	34.0724638	0	0.49277013
5.6556	192008.316	0.48218267	58.253745	0.76916987	3	1.7326462	5.25754389	-0.7273605	4.06482555	-0.2611442	1.09894557	15	75	0.89407778	297.444444	24.44444444	0	0	0	1	34.0724638	0	0.49277013
5.7267	195210.372	0.41341352	42.7883857	0.65242231	3	1.74513945	5.27408293	-0.880891	3.75629007	-0.4255316	1.09894557	16	88	0.89407778	295.222222	22.2222222	0	0	0	1	34.0724638	0	0.49277013
5.7996	198509.851	0.41539326	58.3954053	0.6228243	3	1.75778895	5.29084376	-0.8761251	4.06725434	-0.4718865	1.09894557	17	46	2.23519444	296.888889	23.88888888	0	0	0	1	34.0724638	0	0.49277013
6.4557	216106.102	0.66476867	144.190533	1.47185116	3	1.86496346	5.37577413	-0.406813	4.9711425	0.38720008	1.09894557	18	42	2.23519444	296.333333	23.33333333	0	0	0	1	34.0724638	0	0.49277013
7.1667	236362.742	0.73705514	166.0107	1.65992138	3	1.9694453	5.4653719	-0.3037367	5.11205827	0.5073725	1.09894557	19	35	3.12927222	295.777778	22.7777778	0	0	0	1	34.0724638	0	0.49277013
8.1234	265385.577	0.87575464	228.813911	2.458918	3	2.09474879	5.58118754	-0.1315281	5.43291343	0.90012802	1.09894557	20	36	4.91742778	294.666667	21.66666666	0	0	0	1	34.0724638	0	0.49277013
8.8623	287333.545	0.74212438	168.469549	1.6228182	3	2.18180632	5.6606472	-0.2968918	5.12676095	0.48478029	1.09894557	21	43	2.68223333	293	20	0	0	0	1	34.0724638	0	0.49277013
9.4023	302730.774	0.65952118	132.958138	1.25603902	3	2.24095434	5.71284718	-0.4147261	4.89004185	0.22875897	1.09894557	22	54	0.44703889	290.222222	17.2222222	0	0	0	1	34.0724638	0	0.49277013
9.9108	322158.907	0.72099739	173.957923	1.40999183	3	2.29362507	5.77504803	-0.3257338	5.1588192	0.34429288	1.09894557	23	55	1.34111667	291.333333	18.33333333	0	0	0	1	34.0724638	0	0.49277013
0.0522	3723.21777	1.24313823	438.157792	2.87553325	0	-2.9526728	1.31485684	0.21844311	6.08258138	1.05658584	-6.9077553	0	82	4.91742778	299.666667	26.66666666	0	0	0	0	42.288408	0	-0.4111751
0.2115	10951.9061	1.05226446	316.287407	3.59039203	0	-1.5535303	2.39360482	0.05189435	5.75665448	1.27853988	-6.9077553	1	74	5.36446667	301.333333	28.33333333	0	0	0	0	42.288408	0	-0.4111751
0.4941	23571.5484	1.05121258	303.744452	3.0878846	0	-0.7050174	3.16008283	0.05089516	5.71619002	1.12781005	-6.9077553	2	68	5.36446667	302.444444	29.44444444	0	0	0	0	42.288408	0	-0.4111751
0.8172	37562.8268	0.95981251	247.700122	2.37504675	0	-0.2018714	3.62604153	-0.039976	5.51222287	0.86543808	-6.9077553	3	63	5.81150556	303.555556	30.55555555	0	0	0	0	42.288408	0	-0.4111751
1.2753	54743.625	0.93621502	244.38292	2.4795802	0	0.24318145	4.00267919	-0.0648425	5.49874043	0.90849249	-6.9077553	4	60	4.91742778	304.666667	31.66666666	0	0	0	0	42.288408	0	-0.4111751
1.8351	74785.3332	0.92276187	240.439284	2.63572295	0	0.60709898	4.31463516	-0.079301	5.48247176	0.96953684	-6.9077553	5	55	4.91742778	306.888889	33.88888888	0	0	1	0	42.288408	0	-0.4111751

2.322	95635.8174	0.92343574	231.856689	1.93158557	0	0.84242888	4.56055787	-0.0785717	5.44612377	0.65885878	-6.9077553	6	51	5.81150556	307.444444	34.4444444	0	0	1	0	42.288408	0	-0.4111751
2.9844	133139.414	1.08345471	336.566831	2.39225736	0	1.09339872	4.89140431	0.08107729	5.81879971	0.87265535	-6.9077553	7	49	5.36446667	308.555556	35.5555556	0	0	1	0	42.288408	0	-0.4111751
3.69	168617.713	1.05866958	318.354213	2.47547383	0	1.30562646	5.12764003	0.05795714	5.76316778	0.90683571	-6.9077553	8	52	4.47038889	306.888889	33.88888888	0	0	1	0	42.288408	0	-0.4111751
4.2921	193144.711	0.93217507	249.344731	2.21549972	0	1.45677612	5.26344488	-0.0691625	5.51884041	0.79592925	-6.9077553	9	52	4.47038889	307.444444	34.4444444	0	0	1	0	42.288408	0	-0.4111751
4.7871	211485.268	0.85856268	219.051875	1.82863703	0	1.5659248	5.35416007	-0.1513315	5.38931314	0.6041176	-6.9077553	10	54	3.57631111	306.888889	33.88888888	0	0	1	0	42.288408	0	-0.4111751
5.1777	227721.171	0.79255233	172.658158	1.25229979	0	1.64436094	5.42812634	-0.2312358	5.15131947	0.2257799	-6.9077553	11	58	2.68223333	305.777778	32.7777778	0	0	1	0	42.288408	0	-0.4111751
5.4855	241763.239	0.85577857	201.351311	1.42318387	0	1.70210825	5.48796303	-0.1545758	5.30505617	0.35359892	-6.9077553	12	65	1.78815556	304.111111	31.11111111	0	0	1	0	42.288408	0	-0.4111751
5.7438	253212.323	0.80823796	174.199409	1.36645837	0	1.74812101	5.53423231	-0.2116623	5.16020641	0.31295382	-6.9077553	13	70	1.78815556	303	30	0	0	1	0	42.288408	0	-0.4111751
5.9292	260573.219	0.6954182	128.346007	1.15829016	0	1.7798893	5.56288773	-0.3618049	4.85473758	0.14780789	-6.9077553	14	77	0.44703889	301.888889	28.88888888	0	0	1	0	42.288408	0	-0.4111751
6.0372	265003.446	0.46088845	54.1094076	0.77236176	0	1.79794033	5.5797466	-0.7724319	3.99102654	-0.2570084	-6.9077553	15	75	0.89407778	297.444444	24.44444444	0	0	1	0	42.288408	0	-0.4111751
6.1155	268030.051	0.44065405	49.3893117	0.70307366	0	1.81082653	5.59110283	-0.8172284	3.89975429	-0.3508723	-6.9077553	16	88	0.89407778	295.222222	22.2222222	0	0	1	0	42.288408	0	-0.4111751
6.2415	271991.162	0.51269984	82.9841797	0.99272167	0	1.83122054	5.60577325	-0.6661162	4.41866203	-0.0062981	-6.9077553	17	46	2.23519444	296.888889	23.88888888	0	0	1	0	42.288408	0	-0.4111751
6.8148	281705.704	0.536953	90.3077782	1.5421385	0	1.91909672	5.64086647	-0.6199841	4.50323467	0.43381833	-6.9077553	18	42	2.23519444	296.333333	23.33333333	0	0	1	0	42.288408	0	-0.4111751
7.4511	289071.645	0.56652504	100.424836	1.93232401	0	2.00836167	5.66667802	-0.5664704	4.60941951	0.65924081	-6.9077553	19	35	3.12927222	295.777778	22.7777778	0	0	1	0	42.288408	0	-0.4111751
8.262	298936.063	0.60538719	109.633099	2.49912397	0	2.11166669	5.70023306	-0.5002366	4.69714845	0.91634032	-6.9077553	20	36	4.91742778	294.666667	21.66666666	0	0	1	0	42.288408	0	-0.4111751
8.7147	308166.098	0.59590194	116.740906	1.30761452	0	2.16501125	5.73064216	-0.5160024	4.75996557	0.26896896	-6.9077553	21	43	2.68223333	293	20	0	0	1	0	42.288408	0	-0.4111751
9.1269	319629.095	0.65603571	146.312091	1.24370245	0	2.2112261	5.76716438	-0.4200169	4.98574878	0.21889651	-6.9077553	22	54	0.44703889	290.222222	17.2222222	0	0	1	0	42.288408	0	-0.4111751
9.5103	329254.403	0.63859379	124.258326	1.17279733	0	2.25237542	5.79683375	-0.446922	4.82237072	0.16024408	-6.9077553	23	55	1.34111667	291.333333	18.33333333	0	0	1	0	42.288408	0	-0.4111751
0.0243	0	0.68754306	131.572942	3.13551947	0	-3.7172789	-6.9077553	-0.3731774	4.87956899	1.14311374	-6.9077553	0	82	4.91742778	299.666667	26.66666666	0	0	0	0	18.9135952	0	-0.0428739
0.0936	372.53901	0.60521317	107.513329	2.18546483	0	-2.3687249	-0.9847328	-0.5005236	4.67762414	0.78228601	-6.9077553	1	74	5.36446667	301.333333	28.33333333	0	0	0	0	18.9135952	0	-0.0428739
0.3015	6432.24091	0.82705233	228.378179	2.52293389	0	-1.1989853	1.86147844	-0.1886789	5.43100731	0.92581875	-6.9077553	2	68	5.36446667	302.444444	29.44444444	0	0	1	0	18.9135952	0	-0.0428739
0.5463	13762.9991	0.82686632	232.388887	2.62844916	0	-0.604587	2.62205642	-0.1889036	5.44841651	0.96677438	-6.9077553	3	63	5.81150556	303.555556	30.55555555	0	0	1	0	18.9135952	0	-0.0428739
0.7434	18488.4008	0.79554526	209.217822	2.06920119	0	-0.296521	2.91719764	-0.2274713	5.3433807	0.72764579	-6.9077553	4	60	4.91742778	304.666667	31.66666666	0	0	1	0	18.9135952	0	-0.0428739
1.0107	28809.5571	0.90474549	258.206203	2.23882111	0	0.01064316	3.36074189	-0.0989969	5.55376237	0.806396	-6.9077553	5	55	4.91742778	306.888889	33.88888888	0	0	1	0	18.9135952	0	-0.0428739
1.4049	43410.8561	0.93463288	265.493721	2.57879611	0	0.33996613	3.77073259	-0.0665321	5.58159496	0.94771037	-6.9077553	6	51	5.81150556	307.444444	34.4444444	0	0	1	0	18.9135952	0	-0.0428739
1.7577	58151.2917	0.93066033	255.959782	2.13460338	0	0.56400614	4.06306529	-0.070787	5.54502424	0.75874922	-6.9077553	7	49	5.36446667	308.555556	35.5555556	0	0	1	0	18.9135952	0	-0.0428739
2.1042	70867.7122	0.85505244	215.512764	1.85180062	0	0.74393535	4.26082904	-0.1554236	5.37302478	0.61669834	-6.9077553	8	52	4.47038889	306.888889	33.88888888	0	0	1	0	18.9135952	0	-0.0428739
2.5209	88787.318	0.91401148	250.382391	2.0473501	0	0.92461598	4.48625509	-0.0888187	5.52299331	0.71703464	-6.9077553	9	52	4.47038889	307.444444	34.4444444	0	0	1	0	18.9135952	0	-0.0428739
2.9079	101157.009	0.80480893	193.571857	1.90712266	0	1.06743117	4.61668374	-0.2159086	5.26565396	0.64611986	-6.9077553	10	54	3.57631111	306.888889	33.88888888	0	0	1	0	18.9135952	0	-0.0428739
3.1824	110333.311	0.72834007	157.960912	1.32167468	0	1.15763563	4.70351495	-0.3156152	5.06235394	0.27965596	-6.9077553	11	58	2.68223333	305.777778	32.7777778	0	0	1	0	18.9135952	0	-0.0428739
3.3741	119471.321	0.84536306	192.656874	1.39146316	0	1.21612862	4.78308472	-0.1668069	5.26091594	0.33107424	-6.9077553	12	65	1.78815556	304.111111	31.11111111	0	0	1	0	18.9135952	0	-0.0428739
3.5658	128279	0.81411729	176.035523	1.37318141	0	1.27138843	4.85421538	-0.2044233	5.17069149	0.31785822	-6.9077553	13	70	1.78815556	303	30	0	0	1	0	18.9135952	0	-0.0428739
3.7269	135463.724	0.79078419	166.623963	1.4118325	0	1.31557679	4.90871126	-0.2334664	5.11574556	0.34559655	-6.9077553	14	77	0.44703889	301.888889	28.88888888	0	0	1	0	18.9135952	0	-0.0428739
3.8169	139148.274	0.49779766	61.7264502	0.93685928	0	1.33943857	4.93554727	-0.6955548	4.12272873	-0.0641554	-6.9077553	15	75	0.89407778	297.444444	24.44444444	0	0	1	0	18.9135952	0	-0.0428739
3.8898	141999.422	0.45537162	52.2958924	0.77546428	0	1.35835774	4.95583003	-0.7844478	3.95693695	-0.2530046	-6.9077553	16	88	0.89407778	295.222222	22.2222222	0	0	1	0	18.9135952	0	-0.0428739
3.9492	144449.627	0.48943554	80.2929392	0.79883026	0	1.37351303	4.97293777	-0.7124614	4.38569414	-0.2233557	-6.9077553	17	46	2.23519444	296.888889	23.88888888	0	0	1	0	18.9135952	0	-0.0428739
4.4262	155151.134	0.57004429	112.096027	1.4493847	0	1.48754143	5.04440615	-0.5602885	4.71936481	0.37182883	-6.9077553	18	42	2.23519444	296.333333	23.33333333	0	0	1	0	18.9135952	0	-0.0428739
4.9473	165509.696	0.60116656	115.96257	1.73625528	0	1.59884197	5.10903582	-0.5072212	4.75327609	0.55230644	-6.9077553	19	35	3.12927222	295.777778	22.7777778	0	0	1	0	18.9135952	0	-0.0428739
5.6637	183420.314	0.70891191	162.671806	2.28686968	0	1.73407739	5.21178577	-0.3426144	5.09174086	0.82762111	-6.9077553	20	36	4.91742778	294.666667	21.66666666	0	0	1	0	18.9135952	0	-0.0428739
6.1506	199013.76	0.73216098	169.090544	1.50397067	0	1.81654964	5.29337899	-0.31039	5.13044025	0.40877341	-6.9077553	21	43	2.68223333	293	20	0	0	1	0	18.9135952	0	-0.0428739
6.5673	214239.731	0.75278046	175.114572	1.39353149	0	1.88210279	5.36710029	-0.2826541	5.16544617	0.33255851	-6.9077553	22	54	0.44703889	290.222222	17.2222222	0	0	1	0	18.9135952	0	-0.0428739
7.1253	237241.425	0.82970552	209.240247	1.58774552	0	1.96365183	5.46908251	-0.1854799	5.34348788	0.46294472	-6.9077553	23	55	1.34111667	291.333333	18.33333333	0	0	1	0	18.9135952	0	-0.0428739
0.0243	527.831439	0.70848098	150.448749	2.332418	0	-3.7172789	-0.6370855	-0.3432216	5.01362914	0.84733415	-6.9077553	0	68	5.36446667	302.444444	29.44444444	0	0	0	0	30.519848	0	0.21141735
0.0981	1414.96181	0.54669008	80.6630443	2.05112401	0	-2.3217679	0.34780902	-0.6020457	4.39029293	0.71887536	-6.9077553	1	63	5.81150556	303.555556	30.55555555	0	0	0	0	30.519848	0	0.21141735
0.1665	2528.28857	0.61122086	106.537752	1.50923608	0	-1.79276	0.92793807	-0.4906622	4.66850879	0.41226599	-6.9077553	2	60	4.91742778	304.666667	31.66666666	0	0	0	0	30.519848	0	0.21141735
0.252	3124.21699	0.555576	85.4856343	1.61723296	0	-1.3783262	1.13950372	-0.5859515	4.44836004	0.48133479	-6.9077553	3	55	4.91742778	306.888889	33.88888888	0	0	0	0	30.519848	0	0.21141735
0.4527	6323.70489	0.81034242	202.247285	2.96371884	0	-0.7925256	1.84446338	-0.2090651	5.30949608	1.0867822	-6.9077553	4	51	5.81150556	307.444444	34.4444444	0	0	0	1	30.519848	0	0.21141735
0.7047	11319.5303	0.82209947	198.040206	2.71449084	0	-0.3499831	2.42661792	-0.1946782	5.28847512	0.99897272	-6.9077553	5	49	5.36446667	308.555556	35.5555556	0	0	0	1	30.519848	0	0.21141735
0.954	17366.3605	0.70572274	135.608021	1.81426616	0	-0.0470916	2.85459261	-0.3471169	4.9097759	0.5962321	-6.9077553	6	52	4.47038889	306.888889	33.88888888	0	0	0	1	30.519848	0	0.21141735
1.2249	24604.9517	0.65775134	120.248209	1.59805196	0	0.20285921	3.20298835	-0.4174091	4.78956633	0.46941093	-6.9077553	7	52	4.47038889	307.444444	34.4444444	0	0	0	1	30.519848	0	0.21141735
1.4238	33801.1302	0.6660982	130.662436	1.19951426	0	0.35332935	3.52052382	-0.404818	4.87262483	0.18275001	-6.9077553	8	54	3.57631111	306.888889	33.88888888	0	0	0	1	30.519848	0	0.21141735
1.5678	41655.0773	0.688626	144.984277	1.07679691	0	0.44967336	3.72944727	-0.3716059	4.9766322	0.07491906	-6.9077553	9	58	2.68223333	305.777778	32.7777778	0	0	0	1	30.519848	0	0.21141735
1.6569	49248.5853	0.7185437	141.591817	0.95165715	0	0.50494839	3.89690095	-0.329138	4.95295545	-0.0485002	-6.9077553	10	65	1.78815556	304.111111	31.11111111	0	0	0	1	30.519848	0	0.21141735
1.7244	55578.4677	0.72591578	141.40526	1.0159612	0	0.54487916	4.01781385	-0.3189447	4.95163703	0.01681897	-6.9077553	11	70	1.78815556	303	30	0	0	0	1	30.519848	0	0.21141735
1.7766	61943.2517	0.63349974	104.111791	0.86852948	0	0.57470143	4.12623482	-0.4549184	4.64547484	-0.139803	-6.9077553	12	77	0.44703889	301.888889	28.88888888	0	0	0	1	30.519848	0	0.21141735
1.8162	63829.3934	0.55699041	77.5286088	0.95957045	0	0.59674641	4.15622946	-0.5834135	4.35065991	-0.040228	-6.9077553	13	75	0.89407778	297.444444	24.44444444	0	0	0	1	30.519848	0	0.21141735
1.8468	65189.1709	0.48811122	59.1437952	0.82007262	0	0.61345441	4.1773087	-0.7151654	4.07998859	-0.1971437	-6.9077553	14	88	0.89407778	295.222222	22.2222222	0	0	0	1	30.519848	0	0.21141735
1.872	65953.7365	0.35936802	51.4713789	0.70658847	0	0.62700738	4.1889687	-1.0206295	3.94104533	-0.3458926	-6.9077553	15	46	2.23519444	296.888889	23.88888888	0	0	0	1	30.519848	0	0.21141735
2.0925	74554.8826	0.62667187	122.149689	1.45711296	0	0.73835952	4.31154895	-0.4657378	4.80525544	0.37714311	-6.9077553	16	42	2.23519444	296.333333	23.33333333	0	0	0	1	30.519848	0	0.21141735
2.3472	84436.4801	0.60489868	109.367043	1.47505819	0	0.85322313	4.43601138	-0.5010425	4.69471873	0.38937515	-6.9077553	17	35	3.12927222	295.777778	22.7777778	0	0	0	1	30.519848	0	0.21141735
2.907	94764.5398	0.86145952	229.646855	3.50055933	0	1.06712162	4.55140584	-0.1479671	5.43654707	1.25320839	-6.9077553	18	36	4.91742778	294.666667	21.66666666	0	0	0	1	30.519848	0	0.21141735
3.1743	106281.537	0.69175309	137.774516	1.55289487	0	1.15508714	4.66610099	-0.3670816	4.92562566	0.4407646	-6.9077553	19	43	2.68223333	293	20	0	0	0	1	30.519848	0	0.21141735

3.285	116723.011	0.53758965	89.057776	0.84420485	0	1.18936665	4.75981227	-0.6188013	4.48929656	-0.1681763	-6.9077553	20	54	0.44703889	290.222222	17.2222222	0	0	0	1	30.519848	0	0.21141735
3.3534	126135.072	0.49789549	72.5545719	0.58394593	0	1.20997476	4.83736126	-0.6953586	4.28435278	-0.5362359	-6.9077553	21	55	1.34111667	291.333333	18.33333333	0	0	0	1	30.519848	0	0.21141735
3.5838	138186.438	0.58344811	95.891983	1.85875535	0	1.27642369	4.92861101	-0.5370873	4.56323281	0.62044495	-6.9077553	22	46	3.12927222	294.111111	21.11111111	0	0	0	1	30.519848	0	0.21141735
4.2147	152472.413	0.83838918	208.139444	3.39681106	0	1.43857841	5.02699024	-0.1750808	5.33821306	1.22313142	-6.9077553	23	40	5.36446667	295.777778	22.7777778	0	0	0	1	30.519848	0	0.21141735
0.0432	1837.34488	1.14042891	368.389794	3.45766043	0	-3.1419148	0.60886564	0.13228091	5.90914431	1.24088136	-6.9077553	0	68	5.36446667	302.444444	29.44444444	0	0	0	0	34.0724638	0	0.49277013
0.1332	6041.08184	0.91412102	224.502598	1.46758011	0	-2.0159035	1.79874863	-0.088699	5.41389173	0.38429602	-6.9077553	1	63	5.81150556	303.555556	30.55555555	0	0	0	0	34.0724638	0	0.49277013
0.2178	9429.68071	0.88091911	204.305311	1.59007831	0	-1.5241781	2.24396828	-0.1256549	5.31962039	0.46441197	-6.9077553	2	60	4.91742778	304.666667	31.66666666	0	0	0	0	34.0724638	0	0.49277013
0.3609	15866.5997	1.02309029	303.632763	2.45554836	0	-1.0191544	2.76427928	0.0238047	5.71582225	0.89875726	-6.9077553	3	55	4.91742778	306.888889	33.88888888	0	0	0	1	34.0724638	0	0.49277013
0.9882	38664.2135	1.53735737	730.96866	5.66940032	1	-0.0118702	3.65494032	0.4307152	6.59437195	1.73525972	0.0009995	4	51	5.81150556	307.444444	34.4444444	0	0	0	1	34.0724638	0	0.49277013
1.7766	60165.7233	1.38104304	589.158426	4.77619551	2	0.57470143	4.09711943	0.32356287	6.37869682	1.56385366	0.69364706	5	49	5.36446667	308.555556	35.5555556	0	0	0	1	34.0724638	0	0.49277013
2.3499	79768.2971	1.05659751	315.468308	2.59580316	2	0.85437277	4.37913868	0.05599983	5.7540614	0.95428113	0.69364706	6	52	4.47038889	306.888889	33.88888888	0	0	0	1	34.0724638	0	0.49277013
2.9268	98094.9829	0.99246573	278.396524	2.32289293	2	1.07390968	4.58594642	-0.0065557	5.62905003	0.84324376	0.69364706	7	52	4.47038889	307.444444	34.4444444	0	0	0	1	34.0724638	0	0.49277013
3.4848	120773.3	0.94914551	251.049698	2.01846333	2	1.24841065	4.79392351	-0.0511401	5.5256549	0.7028318	0.69364706	8	54	3.57631111	306.888889	33.88888888	0	0	0	1	34.0724638	0	0.49277013
3.9987	138409.72	0.8555254	209.238781	1.93206563	2	1.38596931	4.9302255	-0.1548713	5.34348087	0.65910715	0.69364706	9	58	2.68223333	305.777778	32.7777778	0	0	0	1	34.0724638	0	0.49277013
4.2507	147692.36	0.82198345	186.284235	1.37483891	2	1.44708368	4.99513823	-0.1948192	5.22727902	0.31906366	0.69364706	10	65	1.78815556	304.111111	31.11111111	0	0	0	1	34.0724638	0	0.49277013
4.4262	155376.473	0.8279433	182.933153	1.43200402	2	1.48754143	5.04585747	-0.1876035	5.20912627	0.35977295	0.69364706	11	70	1.78815556	303	30	0	0	0	1	34.0724638	0	0.49277013
4.5738	160827.892	0.76063421	156.270925	1.39241567	2	1.52034437	5.08034102	-0.2722889	5.0515976	0.33175805	0.69364706	12	77	0.44703889	301.888889	28.88888888	0	0	0	1	34.0724638	0	0.49277013
4.6377	163284.265	0.47059255	54.7647722	0.75586835	2	1.53421855	5.09549876	-0.7516399	4.0030654	-0.278566	0.69364706	13	75	0.89407778	297.444444	24.44444444	0	0	0	1	34.0724638	0	0.49277013
4.6746	164819.485	0.42454045	42.3545339	0.58903806	2	1.5421436	5.10485691	-0.8543953	3.74609908	-0.5275682	0.69364706	14	88	0.89407778	295.222222	22.2222222	0	0	0	1	34.0724638	0	0.49277013
4.7331	166532.612	0.26240733	19.6482873	0.91548174	2	1.55458038	5.11519717	-1.3340537	2.97804107	-0.0872131	0.69364706	15	46	2.23519444	296.888889	23.88888888	0	0	0	1	34.0724638	0	0.49277013
5.2353	178596.598	0.61721517	120.940832	1.40293988	2	1.65542415	5.18513522	-0.4809187	4.7953097	0.33928249	0.69364706	16	42	2.23519444	296.333333	23.33333333	0	0	0	1	34.0724638	0	0.49277013
5.8032	192697.927	0.73049264	162.226835	1.60344492	2	1.75840949	5.26112901	-0.3126681	5.08900174	0.47277785	0.69364706	17	35	3.12927222	295.777778	22.7777778	0	0	0	1	34.0724638	0	0.49277013
6.5961	216869.989	0.82145561	197.676327	2.04360681	2	1.88647857	5.37930266	-0.1954608	5.28663604	0.7152055	0.69364706	18	36	4.91742778	294.666667	21.66666666	0	0	0	1	34.0724638	0	0.49277013
7.3062	240693.05	0.80940554	193.095493	1.68817054	2	1.9887233	5.48352662	-0.2102205	5.26319003	0.5242376	0.69364706	19	43	2.68223333	293	20	0	0	0	1	34.0724638	0	0.49277013
7.8993	260309.221	0.76776586	176.661585	1.45978959	2	2.06677415	5.56187408	-0.2629688	5.17424161	0.37897711	0.69364706	20	54	0.44703889	290.222222	17.2222222	0	0	0	1	34.0724638	0	0.49277013
8.4393	276966.124	0.71039179	152.622161	1.36719045	2	2.13289937	5.62389881	-0.340532	5.02797189	0.31348903	0.69364706	21	55	1.34111667	291.333333	18.33333333	0	0	0	1	34.0724638	0	0.49277013
9.234	296453.521	0.69792754	147.267978	1.65263821	2	2.22289232	5.69189382	-0.3582082	4.9922607	0.50297784	0.69364706	22	46	3.12927222	294.111111	21.11111111	0	0	0	1	34.0724638	0	0.49277013
10.3383	329860.758	0.84705346	219.83807	2.49970054	4	2.33585545	5.79867365	-0.1648116	5.39289578	0.91657091	1.38654433	23	40	5.36446667	295.777778	22.7777778	0	0	0	1	34.0724638	0	0.49277013
0.063	4668.56293	1.38134334	547.474751		0	-2.7646206	1.54106548	0.32378013	6.30531817		-6.9077553	0	68	5.36446667	302.444444	29.44444444	0	0	0	0	42.288408	0	-0.4111751
0.3024	15798.4778	1.17605683	390.446164		0	-1.1960046	2.75997689	0.16301711	5.96729266		-6.9077553	1	63	5.81150556	303.555556	30.55555555	0	0	0	0	42.288408	0	-0.4111751
0.6102	30621.506	1.04473181	300.876508		0	-0.4939685	3.42173523	0.04471694	5.70670323		-6.9077553	2	60	4.91742778	304.666667	31.66666666	0	0	0	0	42.288408	0	-0.4111751
0.9207	44896.9918	0.95930618	252.358877		0	-0.082621	3.80439307	-0.0405031	5.53085615		-6.9077553	3	55	4.91742778	306.888889	33.88888888	0	0	0	0	42.288408	0	-0.4111751
1.4265	63212.2264	0.96288107	262.289563		0	0.35522389	4.14651356	-0.0367874	5.56945291		-6.9077553	4	51	5.81150556	307.444444	34.4444444	0	0	1	0	42.288408	0	-0.4111751
2.0223	86643.5122	0.95109646	255.938491		0	0.70423548	4.46181368	-0.0490889	5.54494105		-6.9077553	5	49	5.36446667	308.555556	35.5555556	0	0	1	0	42.288408	0	-0.4111751
2.4957	107491.531	0.91950554	227.544395		0	0.91456925	4.67742137	-0.0828323	5.42734976		-6.9077553	6	52	4.47038889	306.888889	33.88888888	0	0	1	0	42.288408	0	-0.4111751
3.1356	141274.411	1.04137548	309.334256		0	1.14282054	4.95071125	0.04150223	5.73442566		-6.9077553	7	52	4.47038889	307.444444	34.4444444	0	0	1	0	42.288408	0	-0.4111751
3.7764	172358.13	0.99360237	279.866617		0	1.32877117	5.14958027	-0.0054123	5.63431669		-6.9077553	8	54	3.57631111	306.888889	33.88888888	0	0	1	0	42.288408	0	-0.4111751
4.2417	193232.424	0.88641806	232.635457		0	1.44496413	5.26389891	-0.1194391	5.44947696		-6.9077553	9	58	2.68223333	305.777778	32.7777778	0	0	1	0	42.288408	0	-0.4111751
4.5081	205524.31	0.8878446	222.46516		0	1.50587578	5.32556919	-0.1178329	5.404775		-6.9077553	10	65	1.78815556	304.111111	31.11111111	0	0	1	0	42.288408	0	-0.4111751
4.7538	216230.855	0.80148874	171.47599		0	1.5589443	5.37635124	-0.2200375	5.14444909		-6.9077553	11	70	1.78815556	303	30	0	0	1	0	42.288408	0	-0.4111751
4.9635	224594.581	0.72485004	139.674050		0	1.60211114	5.41430137	-0.3204118	4.93931869		-6.9077553	12	77	0.44703889	301.888889	28.88888888	0	0	1	0	42.288408	0	-0.4111751
5.0967	230165.294	0.52549032	68.2988017		0	1.62859327	5.43880207	-0.6415223	4.22390686		-6.9077553	13	75	0.89407778	297.444444	24.44444444	0	0	1	0	42.288408	0	-0.4111751
5.2092	234463.938	0.47589871	56.6459219		0	1.65042629	5.45730606	-0.7404512	4.03683765		-6.9077553	14	88	0.89407778	295.222222	22.2222222	0	0	1	0	42.288408	0	-0.4111751
5.283	237358.968	0.42047877	56.6195932		0	1.66449412	5.46957784	-0.8639859	4.03637276		-6.9077553	15	46	2.23519444	296.888889	23.88888888	0	0	1	0	42.288408	0	-0.4111751
5.9922	254086.538	0.62509961	121.393024		0	1.79045862	5.53767885	-0.4682458	4.79904165		-6.9077553	16	42	2.23519444	296.333333	23.33333333	0	0	1	0	42.288408	0	-0.4111751
6.6546	266935.514	0.62892694	120.531144		0	1.89530834	5.58701086	-0.4621514	4.79191647		-6.9077553	17	35	3.12927222	295.777778	22.7777778	0	0	1	0	42.288408	0	-0.4111751
7.4772	278774.691	0.6163674	115.342263		0	2.01185839	5.63040748	-0.482291	4.74791258		-6.9077553	18	36	4.91742778	294.666667	21.66666666	0	0	1	0	42.288408	0	-0.4111751
7.8948	288179.131	0.58778408	109.519312		0	2.06620431	5.66358574	-0.5296958	4.69611003		-6.9077553	19	43	2.68223333	293	20	0	0	1	0	42.288408	0	-0.4111751
8.217	297651.662	0.61554918	122.501768		0	2.10620518	5.69592724	-0.4836172	4.80813362		-6.9077553	20	54	0.44703889	290.222222	17.2222222	0	0	1	0	42.288408	0	-0.4111751
8.5464	307205.999	0.65340269	135.675194		0	2.14551014	5.72752179	-0.4240324	4.91027112		-6.9077553	21	55	1.34111667	291.333333	18.33333333	0	0	1	0	42.288408	0	-0.4111751
9.198	319518.427	0.69285356	149.306522		0	2.21898607	5.76681808	-0.3654944	5.00600808		-6.9077553	22	46	3.12927222	294.111111	21.11111111	0	0	1	0	42.288408	0	-0.4111751
10.3626	339218.292	0.81720263	209.860824		0	2.33820317	5.82664678	-0.2006453	5.34644933		-6.9077553	23	40	5.36446667	295.777778	22.7777778	0	0	1	0	42.288408	0	-0.4111751
0.0459	127.658457	0.77363831	176.755059	3.82374786	0	-3.0812902	-2.050594	-0.2553591	5.17477058	1.34149255	-6.9077553	0	68	5.36446667	302.444444	29.44444444	0	0	0	0	18.9135952	0	-0.0428739
0.2214	3447.43352	0.81301802	218.86855	3.06961075	0	-1.5077843	1.23792008	-0.2057728	5.38847589	1.12187648	-6.9077553	1	63	5.81150556	303.555556	30.55555555	0	0	0	0	18.9135952	0	-0.0428739
0.4833	12587.6184	0.8938886	266.093213	2.56996412	0	-0.7271177	2.5327931	-0.111056	5.58385043	0.94428097	-6.9077553	2	60	4.91742778	304.666667	31.66666666	0	0	1	0	18.9135952	0	-0.0428739
0.6615	17242.606	0.77957022	206.021682	1.77973445	0	-0.4132453	2.84744141	-0.2477306	5.32798627	0.57702589	-6.9077553	3	55	4.91742778	306.888889	33.88888888	0	0	1	0	18.9135952	0	-0.0428739
0.9252	26172.6555	0.90081068	257.650731	2.37982615	0	-0.0777453	3.26475339	-0.1033507	5.55160879	0.86744755	-6.9077553	4	51	5.81150556	307.444444	34.4444444	0	0	1	0	18.9135952	0	-0.0428739
1.2789	39017.6738	0.91535651	261.200282	2.60225998	0	0.24600033	3.66404035	-0.0873498	5.56529131	0.9567645	-6.9077553	5	49	5.36446667	308.555556	35.5555556	0	0	1	0	18.9135952	0	-0.0428739
1.6011	50946.0553	0.86461132	218.641149	1.83081941	0	0.47069089	3.93078696	-0.1443193	5.38743637	0.60530969	-6.9077553	6	52	4.47038889	306.888889	33.88888888	0	0	1	0	18.9135952	0	-0.0428739
1.9152	61750.2168	0.80993154	192.364703	1.64401429	0	0.64982206	4.12311368	-0.2095716	5.25939826	0.49774907	-6.9077553	7	52	4.47038889	307.444444	34.4444444	0	0	1	0	18.9135952	0	-0.0428739
2.2464	74997.6817	0.85156134	213.560186	1.7691045	0	0.80932893	4.31747054	-0.1595101	5.36392338	0.57103859	-6.9077553	8	54	3.57631111	306.888889	33.88888888	0	0	1	0	18.9135952	0	-0.0428739
2.5569	88228.5812	0.85143388	214.53673	1.58966082	0	0.93879559	4.47994229	-0.1596596	5.36848562	0.46414954	-6.9077553	9	58	2.68223333	305.777778	32.7777778	0	0	1	0	18.9135952	0	-0.0428739

2.7414	96173.3589	0.81098864	178.371913	1.32487198	0	1.00846874	4.56616278	-0.2082689	5.18387638	0.28207034	-6.9077553	10	65	1.78815556	304.111111	31.11111111	0	0	1	0	18.9135952	0	-0.0428739
2.9178	103956.443	0.86160278	202.414142	1.57838409	0	1.07082991	4.64398161	-0.147801	5.31032075	0.45703495	-6.9077553	11	70	1.78815556	303	30	0	0	1	0	18.9135952	0	-0.0428739
3.06	110294.812	0.69998536	128.07496	1.20168681	0	1.11841492	4.70316596	-0.3552683	4.85262353	0.18455806	-6.9077553	12	77	0.44703889	301.888889	28.88888888	0	0	1	0	18.9135952	0	-0.0428739
3.1635	114900.506	0.51115056	65.0633959	0.87080178	0	1.15167901	4.74407529	-0.6691366	4.17537749	-0.1371932	-6.9077553	13	75	0.89407778	297.444444	24.44444444	0	0	1	0	18.9135952	0	-0.0428739
3.2571	118892.117	0.51185403	65.3555533	0.86773315	0	1.18083723	4.77822491	-0.667764	4.17985771	-0.1407193	-6.9077553	14	88	0.89407778	295.222222	22.2222222	0	0	1	0	18.9135952	0	-0.0428739
3.3354	121747.048	0.30697042	29.9955942	0.65410995	0	1.20459261	4.80195373	-1.1777515	3.40108385	-0.4229522	-6.9077553	15	46	2.23519444	296.888889	23.88888888	0	0	1	0	18.9135952	0	-0.0428739
3.9078	136139.555	0.63293064	136.820116	1.53629385	0	1.36297456	4.91368784	-0.4558157	4.91867435	0.43002363	-6.9077553	16	42	2.23519444	296.333333	23.33333333	0	0	1	0	18.9135952	0	-0.0428739
4.3992	146956.981	0.63420737	128.885474	1.74934315	0	1.48142271	4.9901467	-0.4538038	4.85893197	0.55981185	-6.9077553	17	35	3.12927222	295.777778	22.7777778	0	0	1	0	18.9135952	0	-0.0428739
5.076	162690.103	0.69145024	151.81251	2.25816264	0	1.62452355	5.09185333	-0.3675189	5.02265286	0.81499423	-6.9077553	18	36	4.91742778	294.666667	21.66666666	0	0	1	0	18.9135952	0	-0.0428739
5.5656	179034.568	0.72631011	170.144617	1.48155639	0	1.7166048	5.18758449	-0.3184023	5.13665464	0.39376789	-6.9077553	19	43	2.68223333	293	20	0	0	1	0	18.9135952	0	-0.0428739
5.9544	193548.955	0.75703663	179.565394	1.42018269	0	1.78413044	5.26553565	-0.2770236	5.19054502	0.3514894	-6.9077553	20	54	0.44703889	290.222222	17.2222222	0	0	1	0	18.9135952	0	-0.0428739
6.3351	206137.428	0.70955368	163.245826	1.38825661	0	1.8461056	5.32854793	-0.3417108	5.09526333	0.32876879	-6.9077553	21	55	1.34111667	291.333333	18.33333333	0	0	1	0	18.9135952	0	-0.0428739
7.0272	227812.115	0.76376236	182.592104	1.86527596	0	1.94978833	5.42852562	-0.2681901	5.2072602	0.62394498	-6.9077553	22	46	3.12927222	294.111111	21.11111111	0	0	1	0	18.9135952	0	-0.0428739
8.1459	262158.432	0.89169447	237.285398	3.17243379	0	2.09751473	5.56895284	-0.1135109	5.46926784	1.15481422	-6.9077553	23	40	5.36446667	295.777778	22.7777778	0	0	1	0	18.9135952	0	-0.0428739
0.009	309.61065	0.90489292	220.48801	1.57805263	0	-4.7105307	-1.1692151	-0.0988342	5.39584785	0.45682506	-6.9077553	0	90	1.78815556	297.444444	24.44444444	0	0	0	0	30.519848	0	0.21141735
0.0162	660.520933	0.53686881	68.2898178	0.69199014	0	-4.122744	-0.4132137	-0.6201406	4.22377532	-0.3667395	-6.9077553	1	90	2.23519444	296.888889	23.88888888	0	0	0	0	30.519848	0	0.21141735
0.0297	1011.4312	0.63252792	101.670223	1.16060219	0	-3.5166082	0.01235456	-0.4564512	4.6217443	0.14980025	-6.9077553	2	84	1.78815556	296.888889	23.88888888	0	0	0	0	30.519848	0	0.21141735
0.0378	1318.47766	0.49965138	63.0838691	0.88749939	0	-3.2754462	0.27723594	-0.6918453	4.14448095	-0.1182213	-6.9077553	3	85	2.23519444	296.888889	23.88888888	0	0	0	0	30.519848	0	0.21141735
0.0414	1406.20526	0.39671221	42.9109459	0.73450154	0	-3.1844744	0.34160565	-0.9220266	3.75915025	-0.3072026	-6.9077553	4	81	2.23519444	296.888889	23.88888888	0	0	0	0	30.519848	0	0.21141735
0.0414	1406.20526	0	0	0	0	-3.1844744	0.34160565	-6.9077553	-6.9077553	-6.9077553	-6.9077553	5	78	2.23519444	296.333333	23.33333333	0	0	0	0	30.519848	0	0.21141735
0.0414	1406.20526	0	0	0	0	-3.1844744	0.34160565	-6.9077553	-6.9077553	-6.9077553	-6.9077553	6	76	2.68223333	295.777778	22.7777778	0	0	0	0	30.519848	0	0.21141735
0.0846	1713.25174	0.29504828	24.1602147	1.18096646	0	-2.469821	0.53897668	-1.2172327	3.18474865	0.16717954	-6.9077553	7	69	3.12927222	296.888889	23.88888888	0	0	0	0	30.519848	0	0.21141735
0.1611	2310.01895	0.43850138	53.849954	1.40746909	0	-1.82573	0.83768853	-0.8221144	3.98622012	0.34250336	-6.9077553	8	63	3.12927222	298	25	0	0	0	0	30.519848	0	0.21141735
0.3492	4628.48622	0.76081184	157.259009	3.01277299	0	-1.0521105	1.53244589	-0.2720557	5.05790054	1.10319278	-6.9077553	9	59	4.47038889	299.111111	26.11111111	0	0	0	1	30.519848	0	0.21141735
0.5787	8050.90488	0.68902863	127.841873	1.93645785	0	-0.5469711	2.08590869	-0.3710222	4.85080196	0.66137673	-6.9077553	10	54	3.57631111	300.777778	27.7777778	0	0	0	1	30.519848	0	0.21141735
1.0476	17344.9502	0.83887879	205.332084	2.8274535	1	0.04650183	2.85335906	-0.1744977	5.32463346	1.03973009	0.0009995	11	50	4.91742778	301.333333	28.33333333	0	0	0	1	30.519848	0	0.21141735
1.5507	27597.2394	0.83609495	212.679031	2.13561073	2	0.43870644	3.31775198	-0.1778178	5.35978883	0.7592208	0.69364706	12	49	4.02335	302.444444	29.44444444	0	0	0	1	30.519848	0	0.21141735
2.2419	37867.7512	0.96234298	267.286499	3.34332848	2	0.80732372	3.63412627	-0.0373458	5.58832485	1.20726592	0.69364706	13	46	4.47038889	303	30	0	0	0	1	30.519848	0	0.21141735
2.961	48895.3307	0.87570431	222.481227	2.60824352	2	1.08552705	3.88970236	-0.1315855	5.40484722	0.95906034	0.69364706	14	44	4.47038889	303.555556	30.55555555	0	0	0	1	30.519848	0	0.21141735
3.6792	59633.76	0.86140155	213.550556	2.34772631	2	1.30269534	4.08823863	-0.1480343	5.36387628	0.85387319	0.69364706	15	42	4.47038889	303.555556	30.55555555	0	0	0	1	30.519848	0	0.21141735
4.2975	73936.9471	0.91211488	237.265739	2.09826074	2	1.45803346	4.30322619	-0.0908936	5.46918499	0.74158525	0.69364706	16	41	4.47038889	303.555556	30.55555555	0	0	0	1	30.519848	0	0.21141735
4.8078	88399.9434	0.88910902	226.820202	1.73366734	2	1.5702396	4.48188264	-0.1164113	5.42416205	0.55081566	0.69364706	17	44	4.02335	302.444444	29.44444444	0	0	0	1	30.519848	0	0.21141735
5.3118	102681.673	0.87610495	222.134961	1.98866272	2	1.66993076	4.63164338	-0.1311286	5.40328963	0.68796514	0.69364706	18	47	3.12927222	301.888889	28.88888888	0	0	0	1	30.519848	0	0.21141735
5.7402	115280.654	0.79538823	185.735704	1.47946526	2	1.74749405	4.7473783	-0.2276685	5.2243301	0.3923564	0.69364706	19	52	2.23519444	300.222222	27.2222222	0	0	0	1	30.519848	0	0.21141735
5.985	122527.3	0.78330646	167.795582	1.09729879	2	1.78925634	4.80834202	-0.2429554	5.12275243	0.09376243	0.69364706	20	63	0.89407778	298	25	0	0	0	1	30.519848	0	0.21141735
6.1587	129297.782	0.81673981	181.484881	1.34067848	2	1.81786572	4.86212587	-0.2012111	5.20117786	0.29392142	0.69364706	21	76	0.44703889	295.222222	22.2222222	0	0	0	1	30.519848	0	0.21141735
6.1992	131183.914	0.38349868	36.303144	0.66923044	2	1.82442025	4.87660788	-0.9558149	3.59193189	-0.4001337	0.69364706	22	77	0.89407778	294.666667	21.66666666	0	0	0	1	30.519848	0	0.21141735
6.1992	131183.914	0	0	0	2	1.82442025	4.87660788	-6.9077553	-6.9077553	-6.9077553	0.69364706	23	77	0.89407778	294.666667	21.66666666	0	0	0	1	30.519848	0	0.21141735
0.0171	699.514108	0.93639882	243.114556	1.80239148	0	-4.0686768	-0.3559408	-0.0646464	5.49353687	0.58966905	-6.9077553	0	90	1.78815556	297.444444	24.44444444	0	0	0	0	34.0724638	0	0.49277013
0.027	1006.56059	0.45188678	51.2238215	0.70349953	0	-3.6119184	0.00753215	-0.7921131	3.93622421	-0.3502676	-6.9077553	1	90	2.23519444	296.888889	23.88888888	0	0	0	0	34.0724638	0	0.49277013
0.0387	1357.47083	0.46344783	50.4102689	0.62660577	0	-3.2519157	0.30635968	-0.766906	3.92021474	-0.4658431	-6.9077553	2	84	1.78815556	296.888889	23.88888888	0	0	0	0	34.0724638	0	0.49277013
0.0504	1796.10868	0.44163499	45.4755672	0.60816427	0	-2.9877641	0.58617908	-0.8150098	3.81719719	-0.4956673	-6.9077553	3	85	2.23519444	296.888889	23.88888888	0	0	0	0	34.0724638	0	0.49277013
0.0639	2234.74656	0.46627944	52.9203898	0.67493019	0	-2.7504359	0.8045752	-0.7608278	3.9688076	-0.3916655	-6.9077553	4	81	2.23519444	296.888889	23.88888888	0	0	0	0	34.0724638	0	0.49277013
0.0819	3024.29468	0.5279686	68.8407532	0.84351711	0	-2.5022563	1.1070085	-0.6368262	4.23181044	-0.1689903	-6.9077553	5	78	2.23519444	296.333333	23.33333333	0	0	0	0	34.0724638	0	0.49277013
0.1161	4637.37038	0.69868319	130.530222	1.0534413	0	-2.1533034	1.53436309	-0.3571276	4.87161245	0.05301105	-6.9077553	6	76	2.68223333	295.777778	22.7777778	0	0	0	0	34.0724638	0	0.49277013
0.1755	7474.4662	0.84104856	187.645685	1.33066848	0	-1.7401162	2.01162649	-0.1719176	5.23456086	0.28643265	-6.9077553	7	69	3.12927222	296.888889	23.88888888	0	0	0	0	34.0724638	0	0.49277013
0.2448	9991.40544	0.74562826	150.938096	1.18061224	0	-1.4073137	2.30182535	-0.2921879	5.01687642	0.16687981	-6.9077553	8	63	3.12927222	298	25	0	0	0	0	34.0724638	0	0.49277013
0.3168	12102.8782	0.78245549	163.140503	1.51576223	0	-1.1494846	2.49352592	-0.244041	5.09461794	0.41657795	-6.9077553	9	59	4.47038889	299.111111	26.11111111	0	0	0	1	34.0724638	0	0.49277013
0.4158	15955.607	0.90809201	231.348864	1.72170896	0	-0.8775509	2.76987297	-0.095309	5.44393113	0.54389803	-6.9077553	10	54	3.57631111	300.777778	27.7777778	0	0	0	1	34.0724638	0	0.49277013
0.5742	22628.0055	1.00224174	281.347967	2.04755887	0	-0.5547775	3.11923251	0.0032365	5.63959577	0.71713656	-6.9077553	11	50	4.91742778	301.333333	28.33333333	0	0	0	1	34.0724638	0	0.49277013
0.855	31847.7977	1.02362139	288.932943	2.30065683	0	-0.1566538	3.46099963	0.02432317	5.66619809	0.83362923	-6.9077553	12	49	4.02335	302.444444	29.44444444	0	0	0	1	34.0724638	0	0.49277013
1.1979	41487.2482	1.04181205	316.683071	2.38347828	0	0.18057002	3.72541021	0.04192095	5.75790466	0.86898035	-6.9077553	13	46	4.47038889	303	30	0	0	0	1	34.0724638	0	0.49277013
1.7514	53212.0865	1.07860415	348.517581	2.94692819	0	0.56041547	3.97430435	0.07659445	5.85369155	1.08110262	-6.9077553	14	44	4.47038889	303.555556	30.55555555	0	0	0	1	34.0724638	0	0.49277013
2.3652	68716.8105	0.99510269	287.183834	2.50268877	0	0.86086258	4.23000842	-0.0039049	5.66012603	0.91776515	-6.9077553	15	42	4.47038889	303.555556	30.55555555	0	0	0	1	34.0724638	0	0.49277013
3.0348	89088.2895	0.90331769	235.334509	2.11684065	0	1.11014552	4.48963912	-0.1005746	5.4610122	0.75039701	-6.9077553	16	41	4.47038889	303.555556	30.55555555	0	0	0	1	34.0724638	0	0.49277013
3.9951	115858.048	0.90444509	238.149807	2.44717387	0	1.38506861	4.75237435	-0.0993286	5.47290411	0.89534239	-6.9077553	17	44	4.02335	302.444444	29.44444444	0	0	0	1	34.0724638	0	0.49277013
4.8897	143389.353	0.83049426	200.319039	1.95821044	0	1.58713095	4.96557065	-0.1845309	5.29991628	0.67254156	-6.9077553	18	47	3.12927222	301.888889	28.88888888	0	0	0	1	34.0724638	0	0.49277013
5.6178	168078.861	0.79952524	188.717746	1.58133942	0	1.72594013	5.12443923	-0.2224872	5.24025779	0.4589044	-6.9077553	19	52	2.23519444	300.222222	27.2222222	0	0	0	1	34.0724638	0	0.49277013
6.1758	191509.443	0.86722648	216.200202	1.5655365	0	1.82063843	5.25494234	-0.1413027	5.37620946	0.44886713	-6.9077553	20	63	0.89407778	298	25	0	0	0	1	34.0724638	0	0.49277013
6.5331	207342.446	0.80049422	172.17217	1.4037225	0	1.87688156	5.33437658	-0.2212775	5.14850077	0.33983977	-6.9077553	21	76	0.44703889	295.222222	22.2222222	0	0	0	1	34.0724638	0	0.49277013
6.6366	211992.003	0.39016826	38.6236753	0.64548406	0	1.89259978	5.35655327	-0.9386175	3.65389133	-0.4362067	-6.9077553	22	77	0.89407778	294.666667	21.66666666	0	0	0	1	34.0724638	0	0.49277013
6.6366	211992.003	0	0	0	0	1.89259978	5.35655327	-6.9077553	-6.9077553	-6.9077553	-6.9077553	23	77	0.89407778	294.666667	21.66666666	0	0	0	1	34.0724638	0	0.49277013

0.0261	1434.16119	0.99875035	270.21242	1.82828467	0	-3.64582	0.36127717	-0.0002497	5.59921209	0.603925	-6.9077553	0	90	1.78815556	297.444444	24.44444444	0	0	0	0	42.288408	0	-0.4111751
0.0648	3320.36018	0.64773675	105.104844	1.05531329	0	-2.7364497	1.20037439	-0.4327283	4.65496788	0.05478482	-6.9077553	1	90	2.23519444	296.888889	23.88888888	0	0	0	0	42.288408	0	-0.4111751
0.108	5425.82108	0.61562538	93.3613688	1.00155258	0	-2.2256241	1.69135353	-0.4834936	4.53648836	0.00254933	-6.9077553	2	84	1.78815556	296.888889	23.88888888	0	0	0	0	42.288408	0	-0.4111751
0.1692	8189.23995	0.58661136	85.1189539	0.97527235	0	-1.7766738	2.1029432	-0.5316895	4.44406148	-0.0240137	-6.9077553	3	85	2.23519444	296.888889	23.88888888	0	0	0	0	42.288408	0	-0.4111751
0.2376	11479.0242	0.62270621	97.8939659	1.15324703	0	-1.4371667	2.4406085	-0.4720758	4.58389513	0.14344821	-6.9077553	4	81	2.23519444	296.888889	23.88888888	0	0	0	0	42.288408	0	-0.4111751
0.3231	15553.0323	0.73679528	143.232617	1.40233716	0	-1.1297934	2.74431992	-0.3040889	4.96447698	0.33885309	-6.9077553	5	78	2.23519444	296.333333	23.33333333	0	0	0	0	42.288408	0	-0.4111751
0.4644	23049.1441	0.92176479	231.873905	1.8144679	0	-0.767009	3.13767202	-0.0803809	5.44619802	0.59634323	-6.9077553	6	76	2.68223333	295.777778	22.7777778	0	0	1	0	42.288408	0	-0.4111751
0.7434	36045.3681	0.97795587	275.438785	2.32891491	0	-0.296521	3.58480611	-0.0212687	5.61836904	0.84583175	-6.9077553	7	69	3.12927222	296.888889	23.88888888	0	0	1	0	42.288408	0	-0.4111751
1.1079	46131.6815	0.81209837	188.803781	2.37226035	0	0.10246633	3.83152162	-0.2069032	5.24071358	0.86426469	-6.9077553	8	63	3.12927222	298	25	0	0	1	0	42.288408	0	-0.4111751
1.5165	60693.6486	0.85603373	198.663064	2.1213352	0	0.41640505	4.10585553	-0.154278	5.29161528	0.75251699	-6.9077553	9	59	4.47038889	299.111111	26.11111111	0	0	1	0	42.288408	0	-0.4111751
1.9908	78430.8167	0.87638568	204.042694	1.93609712	0	0.68853657	4.36222967	-0.1308086	5.31833416	0.66119052	-6.9077553	10	54	3.57631111	300.777778	27.7777778	0	0	1	0	42.288408	0	-0.4111751
2.6757	104997.784	0.94618279	247.467169	2.55432273	0	0.98421103	4.65394877	-0.0542632	5.51128196	0.93817853	-6.9077553	11	50	4.91742778	301.333333	28.33333333	0	0	1	0	42.288408	0	-0.4111751
3.5622	130060.554	0.87248089	217.924429	2.61903058	0	1.27037833	4.86800783	-0.135269	5.38415294	0.96318599	-6.9077553	12	49	4.02335	302.444444	29.44444444	0	0	1	0	42.288408	0	-0.4111751
4.7331	151073.4	0.77595148	178.374159	3.25960086	0	1.55458038	5.01777243	-0.2523774	5.18388897	1.18191149	-6.9077553	13	46	4.47038889	303	30	0	0	1	0	42.288408	0	-0.4111751
5.7816	173592.529	0.77106336	172.172921	2.62186368	0	1.75468046	5.15671653	-0.2586887	5.14850514	0.96426673	-6.9077553	14	44	4.47038889	303.555556	30.55555555	0	0	1	0	42.288408	0	-0.4111751
6.7815	202485.766	0.78170367	185.317971	2.14962898	0	1.91419832	5.31067453	-0.2450011	5.22207851	0.76576035	-6.9077553	15	42	4.47038889	303.555556	30.55555555	0	0	1	0	42.288408	0	-0.4111751
7.8336	239093.751	0.85236008	217.064639	2.25123156	0	2.05842217	5.47685992	-0.1585737	5.38019979	0.81192153	-6.9077553	16	41	4.47038889	303.555556	30.55555555	0	0	1	0	42.288408	0	-0.4111751
8.8605	278771.796	0.86152963	220.553654	1.97374451	0	2.1816032	5.6303971	-0.1478858	5.39614553	0.68043903	-6.9077553	17	44	4.02335	302.444444	29.44444444	0	0	1	0	42.288408	0	-0.4111751
9.756	315313.119	0.84536599	211.867918	1.62228182	0	2.27788248	5.75356934	-0.1668034	5.35596777	0.48444991	-6.9077553	18	47	3.12927222	301.888889	28.88888888	0	0	1	0	42.288408	0	-0.4111751
10.5876	350280.598	0.85770056	223.398235	1.65928458	0	2.35968351	5.8587374	-0.152335	5.40896046	0.50698902	-6.9077553	19	52	2.23519444	300.222222	27.2222222	0	0	1	0	42.288408	0	-0.4111751
11.1627	376851.058	0.89278234	227.209385	1.60759915	0	2.41257786	5.93185269	-0.112293	5.42587639	0.47536371	-6.9077553	20	63	0.89407778	298	25	0	0	1	0	42.288408	0	-0.4111751
11.4885	391605.136	0.84480808	192.626079	1.50914752	0	2.44134654	5.97025658	-0.1674628	5.26075609	0.41220734	-6.9077553	21	76	0.44703889	295.222222	22.2222222	0	0	1	0	42.288408	0	-0.4111751
11.5704	395289.684	0.43115348	49.9078114	0.75306601	0	2.44845011	5.9796214	-0.8389745	3.91019757	-0.2822754	-6.9077553	22	77	0.89407778	294.666667	21.66666666	0	0	1	0	42.288408	0	-0.4111751
11.5704	395289.684	0	0	0	0	2.44845011	5.9796214	-6.9077553	-6.9077553	-6.9077553	-6.9077553	23	77	0.89407778	294.666667	21.66666666	0	0	1	0	42.288408	0	-0.4111751
0.0009	0	0	0	0	0	-7.0131158	-6.9077553	-6.9077553	-6.9077553	-6.9077553	-6.9077553	0	90	1.78815556	297.444444	24.44444444	0	0	0	0	18.9135952	0	-0.0428739
0.0009	0	0	0	0	0	-7.0131158	-6.9077553	-6.9077553	-6.9077553	-6.9077553	-6.9077553	1	90	2.23519444	296.888889	23.88888888	0	0	0	0	18.9135952	0	-0.0428739
0.0009	0	0	0	0	0	-7.0131158	-6.9077553	-6.9077553	-6.9077553	-6.9077553	-6.9077553	2	84	1.78815556	296.888889	23.88888888	0	0	0	0	18.9135952	0	-0.0428739
0.0009	0	0	0	0	0	-7.0131158	-6.9077553	-6.9077553	-6.9077553	-6.9077553	-6.9077553	3	85	2.23519444	296.888889	23.88888888	0	0	0	0	18.9135952	0	-0.0428739
0.0009	0	0	0	0	0	-7.0131158	-6.9077553	-6.9077553	-6.9077553	-6.9077553	-6.9077553	4	81	2.23519444	296.888889	23.88888888	0	0	0	0	18.9135952	0	-0.0428739
0.0009	0	0	0	0	0	-7.0131158	-6.9077553	-6.9077553	-6.9077553	-6.9077553	-6.9077553	5	78	2.23519444	296.333333	23.33333333	0	0	0	0	18.9135952	0	-0.0428739
0.0009	0	0	0	0	0	-7.0131158	-6.9077553	-6.9077553	-6.9077553	-6.9077553	-6.9077553	6	76	2.68223333	295.777778	22.7777778	0	0	0	0	18.9135952	0	-0.0428739
0.0009	0	0	0	0	0	-7.0131158	-6.9077553	-6.9077553	-6.9077553	-6.9077553	-6.9077553	7	69	3.12927222	296.888889	23.88888888	0	0	0	0	18.9135952	0	-0.0428739
0.0162	0	0.37601342	43.2027265	1.42546878	0	-4.122744	-6.9077553	-0.9754745	3.76592675	0.35520201	-6.9077553	8	63	3.12927222	298	25	0	0	0	0	18.9135952	0	-0.0428739
0.1548	264.029904	0.63026002	107.707462	2.95480109	0	-1.8656213	-1.3279126	-0.4600374	4.67942816	1.08376971	-6.9077553	9	59	4.47038889	299.111111	26.11111111	0	0	1	0	18.9135952	0	-0.0428739
0.3195	1010.46443	0.52431375	76.1385118	1.8731446	0	-1.140998	0.01139921	-0.6437596	4.33256734	0.62815234	-6.9077553	10	54	3.57631111	300.777778	27.7777778	0	0	1	0	18.9135952	0	-0.0428739
0.6246	3899.47131	0.65238588	128.036044	3.21021516	0	-0.4706438	1.36109739	-0.4255874	4.85231962	1.16664942	-6.9077553	11	50	4.91742778	301.333333	28.33333333	0	0	1	0	18.9135952	0	-0.0428739
0.9963	9390.48186	0.67422175	138.739407	2.59096219	0	-0.0037069	2.23980309	-0.3927141	4.93260461	0.95241519	-6.9077553	12	49	4.02335	302.444444	29.44444444	0	0	1	0	18.9135952	0	-0.0428739
1.4625	15802.0153	0.67142006	138.691829	2.31215759	0	0.3801473	2.76020076	-0.396872	4.93226162	0.83861351	-6.9077553	13	46	4.47038889	303	30	0	0	1	0	18.9135952	0	-0.0428739
2.1609	27994.1955	0.75668461	175.213193	2.80151933	0	0.7705248	3.3320329	-0.2774881	5.16600918	1.03051878	-6.9077553	14	44	4.47038889	303.555556	30.55555555	0	0	1	0	18.9135952	0	-0.0428739
3.1464	58739.1247	0.89588355	249.703425	3.04171193	0	1.14625894	4.07312305	-0.1088292	5.52027792	1.1127492	-6.9077553	15	42	4.47038889	303.555556	30.55555555	0	0	1	0	18.9135952	0	-0.0428739
3.9933	95294.1573	0.96813161	281.344779	2.62535622	0	1.38461796	4.55697899	-0.0313549	5.63958444	0.96559742	-6.9077553	16	41	4.47038889	303.555556	30.55555555	0	0	1	0	18.9135952	0	-0.0428739
4.8969	137854.343	0.98925119	283.001183	2.34448923	0	1.58860235	4.92620489	-0.0097966	5.64545461	0.85249401	-6.9077553	17	44	4.02335	302.444444	29.44444444	0	0	1	0	18.9135952	0	-0.0428739
5.8104	182551.97	0.97542658	271.981404	2.08438005	0	1.75964942	5.20704038	-0.0238557	5.60573737	0.73495112	-6.9077553	18	47	3.12927222	301.888889	28.88888888	0	0	1	0	18.9135952	0	-0.0428739
6.6177	221539.769	0.94092647	250.830964	1.7942839	0	1.88974788	5.40060663	-0.0598281	5.52478325	0.58516317	-6.9077553	19	52	2.23519444	300.222222	27.2222222	0	0	1	0	18.9135952	0	-0.0428739
7.2567	253995.967	0.95016832	248.229137	1.73786501	0	1.98192518	5.53732232	-0.0500642	5.51435629	0.55323261	-6.9077553	20	63	0.89407778	298	25	0	0	1	0	18.9135952	0	-0.0428739
7.7049	275360.991	0.82851315	182.127777	1.41926323	0	2.04185649	5.61808656	-0.1869163	5.204714	0.35084223	-6.9077553	21	76	0.44703889	295.222222	22.2222222	0	0	1	0	18.9135952	0	-0.0428739
7.8318	281194.886	0.44670202	52.8899601	0.68950821	0	2.05819237	5.63905153	-0.8036274	3.96823244	-0.3703274	-6.9077553	22	77	0.89407778	294.666667	21.66666666	0	0	1	0	18.9135952	0	-0.0428739
7.8318	281194.886	0	0	0	0	2.05819237	5.63905153	-6.9077553	-6.9077553	-6.9077553	-6.9077553	23	77	0.89407778	294.666667	21.66666666	0	0	1	0	18.9135952	0	-0.0428739
0.0135	409.686525	0.81828363	194.329084	1.51690197	0	-4.3050656	-0.8899251	-0.1993249	5.26955818	0.4173291	-6.9077553	0	58	2.68223333	305.777778	32.7777778	0	0	0	0	30.519848	0	0.21141735
0.0297	1086.0021	0.84143798	189.636542	1.38011127	0	-3.5166082	0.08342354	-0.1714552	5.24511458	0.32288844	-6.9077553	1	65	1.78815556	304.111111	31.11111111	0	0	0	0	30.519848	0	0.21141735
0.0423	1350.80868	0.88764674	206.473371	1.67042351	0	-3.1629682	0.30144346	-0.1180555	5.33017629	0.51367566	-6.9077553	2	70	1.78815556	303	30	0	0	0	0	30.519848	0	0.21141735
0.0432	1350.80868	0.61928523	91.6537018	0.8939572	0	-3.1419148	0.30144346	-0.4775759	4.51802827	-0.1109794	-6.9077553	3	77	0.44703889	301.888889	28.88888888	0	0	0	0	30.519848	0	0.21141735
0.0432	1350.80868	0	0	0	0	-3.1419148	0.30144346	-6.9077553	-6.9077553	-6.9077553	-6.9077553	4	75	0.89407778	297.444444	24.44444444	0	0	0	0	30.519848	0	0.21141735
0.0432	1350.80868	0	0	0	0	-3.1419148	0.30144346	-6.9077553	-6.9077553	-6.9077553	-6.9077553	5	88	0.89407778	295.222222	22.2222222	0	0	0	0	30.519848	0	0.21141735
0.0432	1350.80868	0	0	0	0	-3.1419148	0.30144346	-6.9077553	-6.9077553	-6.9077553	-6.9077553	6	46	2.23519444	296.888889	23.88888888	0	0	0	0	30.519848	0	0.21141735
0.0882	1745.58272	0.33991642	34.7957422	1.23345589	0	-2.4281483	0.55766115	-1.0761179	3.54952377	0.2106303	-6.9077553	7	42	2.23519444	296.333333	23.33333333	0	0	0	0	30.519848	0	0.21141735
0.1584	2601.15637	0.44245461	61.0491975	1.24029688	0	-1.8426318	0.95634047	-0.8131598	4.11169644	0.2161567	-6.9077553	8	35	3.12927222	295.777778	22.7777778	0	0	0	0	30.519848	0	0.21141735
0.387	4157.01517	0.70951321	137.209092	3.42468214	0	-0.9493306	1.42503784	-0.3417677	4.92151327	1.23130062	-6.9077553	9	36	4.91742778	294.666667	21.66666666	0	0	0	1	30.519848	0	0.21141735
0.5283	5711.94528	0.52387981	76.6951072	1.26654646	0	-0.638091	1.7427347	-0.644586	4.33985095	0.23708311	-6.9077553	10	43	2.68223333	293	20	0	0	0	1	30.519848	0	0.21141735
0.6516	7487.05901	0.47229572	66.0207359	0.81227758	0	-0.4283244	2.01330962	-0.7480349	4.18998402	-0.2066828	-6.9077553	11	54	0.44703889	290.222222	17.2222222	0	0	0	1	30.519848	0	0.21141735
0.7776	9581.47792	0.50828716	73.701144	0.87606768	0	-0.251543	2.25993621	-0.6747433	4.30003189	-0.1311711	-6.9077553	12	55	1.34111667	291.333333	18.33333333	0	0	0	1	30.519848	0	0.21141735
1.0836	12429.884	0.69024272	154.11119	2.10449291	0	0.08028883	2.52018402	-0.3692643	5.03768085	0.7445496	-6.9077553	13	46	3.12927222	294.111111	21.11111111	0	0	0	1	30.519848	0	0.21141735

1.8738	20251.7384	0.95452269	269.710065	4.30320125	0	0.62796845	3.00829001	-0.0454968	5.59735126	1.45959158	-6.9077553	14	40	5.36446667	295.777778	22.7777778	0	0	0	1	30.519848	0	0.21141735
2.9088	32844.3486	0.96842561	281.086033	3.9296391	0	1.06774063	3.49181014	-0.0310515	5.63866435	1.36880203	-6.9077553	15	35	6.25854444	297.444444	24.44444444	0	0	0	1	30.519848	0	0.21141735
3.8889	51939.6757	0.97989348	280.081899	2.52704536	0	1.35812634	3.95010222	-0.0192914	5.63508563	0.92744642	-6.9077553	16	31	6.70558333	299.111111	26.11111111	0	0	0	1	30.519848	0	0.21141735
4.9572	66500.9001	1.0176966	303.78928	2.68384632	0	1.60084107	4.19723052	0.01852396	5.71633759	0.98762349	-6.9077553	17	27	6.25854444	300.777778	27.7777778	0	0	0	1	30.519848	0	0.21141735
6.1344	228873.113	1.41759881	550.413704	2.87624702	142	1.81391227	5.43317213	0.34966963	6.310672	1.05683394	4.9558341	18	24	7.59966111	301.888889	28.88888888	0	0	0	1	30.519848	0	0.21141735
7.3467	465254.977	1.5909142	647.039996	3.24468292	357	1.99425123	6.14258774	0.46493719	6.47240966	1.17732578	5.87773858	19	23	5.36446667	303.555556	30.55555555	0	0	0	1	30.519848	0	0.21141735
8.9577	944708.908	2.05742269	941.242458	4.44895824	800	2.1925135	6.8508779	0.72194001	6.84720183	1.49289471	6.68461298	20	23	5.81150556	303.555556	30.55555555	0	0	0	1	30.519848	0	0.21141735
10.8981	1727906.05	2.98916813	1630.2275	4.68464278	1605	2.38858846	7.45466616	1.09532962	7.39647547	1.54450311	7.38087966	21	22	6.25854444	304.111111	31.11111111	0	0	0	1	30.519848	0	0.21141735
12.6126	2230442.43	2.39116224	1240.28373	4.68820692	2105	2.53469631	7.70995569	0.87219766	7.12309625	1.54526347	7.65207122	22	23	5.36446667	304.111111	31.11111111	0	0	0	1	30.519848	0	0.21141735
13.5981	2264075.41	0.99217189	313.084817	2.72139631	2128	2.60993008	7.72492219	-0.0068515	5.74647733	1.00151249	7.66293832	23	24	4.91742778	303.555556	30.55555555	0	0	0	1	30.519848	0	0.21141735
0.0342	1269.82511	0.99604612	283.55862	2.58340351	0	-3.3755296	0.23966638	-0.0029583	5.6474224	0.94949473	-6.9077553	0	58	2.68223333	305.777778	32.7777778	0	0	0	0	34.0724638	0	0.49277013
0.0891	3566.91437	0.84307993	188.557793	1.28922309	0	-2.4179959	1.27198122	-0.1695081	5.23940986	0.25481514	-6.9077553	1	65	1.78815556	304.111111	31.11111111	0	0	0	0	34.0724638	0	0.49277013
0.1458	6300.39927	0.75663963	146.365963	1.05460418	0	-1.9255195	1.84077172	-0.2775474	4.98611691	0.05411329	-6.9077553	2	70	1.78815556	303	30	0	0	0	0	34.0724638	0	0.49277013
0.1935	8318.13411	0.66600022	108.641684	0.86138995	0	-1.6424778	2.11855818	-0.4049649	4.68806437	-0.1480477	-6.9077553	3	77	0.44703889	301.888889	28.88888888	0	0	0	0	34.0724638	0	0.49277013
0.2205	9458.59196	0.51187239	62.5166707	0.6694081	0	-1.5118576	2.24702925	-0.6677282	4.13544925	-0.3998686	-6.9077553	4	75	0.89407778	297.444444	24.44444444	0	0	0	0	34.0724638	0	0.49277013
0.2448	10467.4593	0.47287386	52.6517379	0.63185219	0	-1.4073137	2.34836686	-0.7468141	3.96371824	-0.4575184	-6.9077553	5	88	0.89407778	295.222222	22.2222222	0	0	0	0	34.0724638	0	0.49277013
0.2601	10906.0965	0.28836584	23.0337417	0.55762695	0	-1.3466891	2.38941363	-1.2400635	3.13700358	-0.5822734	-6.9077553	6	46	2.23519444	296.888889	23.88888888	0	0	0	0	34.0724638	0	0.49277013
0.3762	15062.0851	0.81272178	191.966267	1.64517756	0	-0.9776344	2.71224706	-0.2061368	5.25732487	0.49845597	-6.9077553	7	42	2.23519444	296.333333	23.33333333	0	0	0	0	34.0724638	0	0.49277013
0.5391	21610.0404	0.88168268	214.257532	1.65385648	0	-0.6178542	3.07320431	-0.1247895	5.36718338	0.50371428	-6.9077553	8	35	3.12927222	295.777778	22.7777778	0	0	0	1	34.0724638	0	0.49277013
0.7731	29733.4391	0.96053261	262.074369	2.24714731	0	-0.2573469	3.39230594	-0.0392268	5.56863213	0.81010646	-6.9077553	9	36	4.91742778	294.666667	21.66666666	0	0	0	1	34.0724638	0	0.49277013
1.0233	40429.3216	0.93064331	246.943402	1.86298222	0	0.0230327	3.69958004	-0.0708052	5.50916322	0.62271518	-6.9077553	10	43	2.68223333	293	20	0	0	0	1	34.0724638	0	0.49277013
1.2339	48037.1458	0.80983441	189.377003	1.42784914	0	0.21017988	3.8719954	-0.2096914	5.24374503	0.35686932	-6.9077553	11	54	0.44703889	290.222222	17.2222222	0	0	0	1	34.0724638	0	0.49277013
1.4211	53618.2209	0.7370896	165.707745	1.25112446	0	0.35143122	3.9819076	-0.3036901	5.1102317	0.22484167	-6.9077553	12	55	1.34111667	291.333333	18.33333333	0	0	0	1	34.0724638	0	0.49277013
1.7487	64110.6897	0.88777345	223.218664	1.85150381	0	0.55887265	4.16062671	-0.1179129	5.40815633	0.61653814	-6.9077553	13	46	3.12927222	294.111111	21.11111111	0	0	0	1	34.0724638	0	0.49277013
2.3211	86314.3109	1.0206034	300.709046	2.69637351	0	0.84204121	4.458007	0.02137335	5.7061465	0.99227853	-6.9077553	14	40	5.36446667	295.777778	22.7777778	0	0	0	1	34.0724638	0	0.49277013
3.1635	115963.747	1.07759172	327.723958	3.02306555	0	1.15167901	4.75328624	0.07565623	5.79217471	1.10660213	-6.9077553	15	35	6.25854444	297.444444	24.44444444	0	0	0	1	34.0724638	0	0.49277013
4.3839	161152.428	1.1137346	360.747926	3.47343188	1	1.47793874	5.08235688	0.10861635	5.88818222	1.24543098	0.0009995	16	31	6.70558333	299.111111	26.11111111	0	0	0	1	34.0724638	0	0.49277013
5.5593	200543.588	1.03580567	306.450182	2.84591661	1	1.7154722	5.30103661	0.03614452	5.72505847	1.04623652	0.0009995	17	27	6.25854444	300.777778	27.7777778	0	0	0	1	34.0724638	0	0.49277013
6.9714	260861.002	1.18394362	418.953314	3.84070641	3	1.94181607	5.56399154	0.1696952	6.03776188	1.34591665	1.09894557	18	24	7.59966111	301.888889	28.88888888	0	0	0	1	34.0724638	0	0.49277013
8.4699	313122.736	1.05326445	331.320796	3.41484492	3	2.1365187	5.74659844	0.05284332	5.8030901	1.22842488	1.09894557	19	23	5.36446667	303.555556	30.55555555	0	0	0	1	34.0724638	0	0.49277013
9.9936	363916.833	0.95060947	283.925724	3.25277741	3	2.30194489	5.89692811	-0.0496005	5.64871619	1.1798166	1.09894557	20	23	5.81150556	303.555556	30.55555555	0	0	0	1	34.0724638	0	0.49277013
11.5191	409310.81	0.97539855	305.066261	3.55803374	3	2.44400653	6.01447724	-0.0238844	5.72053228	1.26948909	1.09894557	21	22	6.25854444	304.111111	31.11111111	0	0	0	1	34.0724638	0	0.49277013
12.9555	457297.952	0.95452988	287.30038	3.12774735	3	2.56152041	6.12533734	-0.0454892	5.66053177	1.14063272	1.09894557	22	23	5.36446667	304.111111	31.11111111	0	0	0	1	34.0724638	0	0.49277013
14.4936	503907.057	0.91780404	269.003986	3.11099956	3	2.67370717	6.22239383	-0.0846824	5.59472992	1.13526546	1.09894557	23	24	4.91742778	303.555556	30.55555555	0	0	0	1	34.0724638	0	0.49277013
0.0414	2938.48121	1.23757765	426.245443	2.69312219	0	-3.1844744	1.07823311	0.21396367	6.05501768	0.99107243	-6.9077553	0	58	2.68223333	305.777778	32.7777778	0	0	0	0	42.288408	0	-0.4111751
0.1269	7802.90236	0.99456554	273.881086	1.92358736	0	-2.0643559	2.05462391	-0.0044443	5.61269767	0.65471158	-6.9077553	1	65	1.78815556	304.111111	31.11111111	0	0	0	0	42.288408	0	-0.4111751
0.2205	12781.8023	0.95013626	241.362192	1.79585736	0	-1.5118576	2.5481007	-0.0500979	5.48630282	0.58603923	-6.9077553	2	70	1.78815556	303	30	0	0	0	0	42.288408	0	-0.4111751
0.3186	17433.2595	0.87383865	201.164869	1.70482279	0	-1.1438189	2.85843721	-0.1337158	5.30412979	0.53404757	-6.9077553	3	77	0.44703889	301.888889	28.88888888	0	0	0	0	42.288408	0	-0.4111751
0.3942	20986.2251	0.67714582	116.274158	1.28089278	0	-0.9308969	3.04391392	-0.3883929	4.75595944	0.24833772	-6.9077553	4	75	0.89407778	297.444444	24.44444444	0	0	0	0	42.288408	0	-0.4111751
0.4635	24276.0093	0.54585806	76.2701654	0.93729381	0	-0.7689489	3.18952978	-0.603566	4.33429496	-0.0636922	-6.9077553	5	88	0.89407778	295.222222	22.2222222	0	0	0	0	42.288408	0	-0.4111751
0.5229	27068.4877	0.52820859	87.4848485	0.84188299	0	-0.648365	3.29840718	-0.6363726	4.47147705	-0.1709271	-6.9077553	6	46	2.23519444	296.888889	23.88888888	0	0	1	0	42.288408	0	-0.4111751
0.8559	40469.2703	0.86415092	230.97027	1.90116247	0	-0.1556017	3.70056764	-0.1448513	5.44229333	0.64299138	-6.9077553	7	42	2.23519444	296.333333	23.33333333	0	0	1	0	42.288408	0	-0.4111751
1.1925	52055.5496	0.84581165	199.564171	1.87469417	0	0.17605194	3.95233062	-0.166277	5.29614086	0.62897882	-6.9077553	8	35	3.12927222	295.777778	22.7777778	0	0	1	0	42.288408	0	-0.4111751
1.5795	65002.5918	0.81820622	178.147057	2.00593262	0	0.45710834	4.17444253	-0.1994194	5.18261498	0.6966075	-6.9077553	9	36	4.91742778	294.666667	21.66666666	0	0	1	0	42.288408	0	-0.4111751
1.9683	80669.4434	0.85070801	201.53776	1.63775592	0	0.67717023	4.39037226	-0.1605115	5.30598172	0.49393737	-6.9077553	10	43	2.68223333	293	20	0	0	1	0	42.288408	0	-0.4111751
2.2986	93742.718	0.76169796	161.649246	1.27340216	0	0.83230024	4.54056466	-0.2708932	5.08543503	0.24247717	-6.9077553	11	54	0.44703889	290.222222	17.2222222	0	0	1	0	42.288408	0	-0.4111751
2.7234	111100.603	0.86279756	209.775788	1.56905843	0	1.0018811	4.71044513	-0.1464168	5.34604405	0.45111284	-6.9077553	12	55	1.34111667	291.333333	18.33333333	0	0	1	0	42.288408	0	-0.4111751
3.4155	134449.412	0.83264763	200.53379	2.08289479	0	1.2283239	4.90119545	-0.1819445	5.30098775	0.73423864	-6.9077553	13	46	3.12927222	294.111111	21.11111111	0	0	1	0	42.288408	0	-0.4111751
4.6575	160546.418	0.81235741	199.94846	3.42534534	0	1.53847882	5.07858934	-0.2065847	5.29806464	1.23149419	-6.9077553	14	40	5.36446667	295.777778	22.7777778	0	0	1	0	42.288408	0	-0.4111751
6.183	190090.161	0.84323939	213.939712	4.00382271	0	1.82180359	5.24750375	-0.1693192	5.36569893	1.38749931	-6.9077553	15	35	6.25854444	297.444444	24.44444444	0	0	1	0	42.288408	0	-0.4111751
7.9848	250758.831	1.08709064	375.476024	4.69306767	0	2.07753973	5.52449563	0.08442446	5.92819728	1.54629951	-6.9077553	16	31	6.70558333	299.111111	26.11111111	0	0	1	0	42.288408	0	-0.4111751
9.4212	322199.012	1.19021782	433.669643	3.75724529	0	2.24296247	5.77517251	0.17497617	6.07228536	1.32395217	-6.9077553	17	27	6.25854444	300.777778	27.7777778	0	0	1	0	42.288408	0	-0.4111751
10.8837	399509.799	1.21991729	455.415885	3.81454387	0	2.38726626	5.9902408	0.19960246	6.12121323	1.33908322	-6.9077553	18	24	7.59966111	301.888889	28.88888888	0	0	1	0	42.288408	0	-0.4111751
12.3831	474988.267	1.18691676	419.347089	3.32732605	3	2.51633264	6.16329221	0.17220115	6.03870134	1.20246949	1.09894557	19	23	5.36446667	303.555556	30.55555555	0	0	1	0	42.288408	0	-0.4111751
14.0229	551121.292	1.18746141	421.938358	3.99983599	4	2.64069171	6.31195673	0.17265954	6.0448616	1.38650334	1.38654433	20	23	5.81150556	303.555556	30.55555555	0	0	1	0	42.288408	0	-0.4111751
15.5763	628471.379	1.18634479	432.156974	3.56738564	4	2.74575053	6.44329208	0.17171954	6.0687912	1.27211329	1.38654433	21	22	6.25854444	304.111111	31.11111111	0	0	1	0	42.288408	0	-0.4111751
17.0757	690819.018	1.05127319	326.715861	3.14334367	4	2.8376564	6.53787932	0.05095277	5.78909393	1.14560518	1.38654433	22	23	5.36446667	304.111111	31.11111111	0	0	1	0	42.288408	0	-0.4111751
18.4644	745542.711	1.03563077	319.507271	2.84021684	4	2.91584455	6.61411377	0.03597581	5.76678316	1.04423243	1.38654433	23	24	4.91742778	303.555556	30.55555555	0	0	1	0	42.288408	0	-0.4111751
0.0027	0	0.21041781	8.77035046	0.69165194	0	-5.9145035	-6.9077553	-1.553919	2.17149078	-0.3672277	-6.9077553	0	58	2.68223333	305.777778	32.7777778	0	0	0	0	18.9135952	0	-0.0428739
0.0036	0	0.06053963	0.5845502	0.09518638	0	-5.6268214	-6.9077553	-2.788074	-0.5352034	-2.3414676	-6.9077553	1	65	1.78815556	304.111111	31.11111111	0	0	0	0	18.9135952	0	-0.0428739
0.0036	0	0	0	0	0	-5.6268214	-6.9077553	-6.9077553	-6.9077553	-6.9077553	-6.9077553	2	70	1.78815556	303	30	0	0	0	0	18.9135952	0	-0.0428739
0.0036	0	0	0	0	0	-5.6268214	-6.9077553	-6.9077553	-6.9077553	-6.9077553	-6.9077553	3	77	0.44703889	301.888889	28.88888888	0	0	0	0	18.9135952	0	-0.0428739

0.0036	0	0	0	0	0	-5.6268214	-6.9077553	-6.9077553	-6.9077553	-6.9077553	-6.9077553	4	75	0.89407778	297.444444	24.44444444	0	0	0	0	18.9135952	0	-0.0428739
0.0036	0	0	0	0	0	-5.6268214	-6.9077553	-6.9077553	-6.9077553	-6.9077553	-6.9077553	5	88	0.89407778	295.222222	22.2222222	0	0	0	0	18.9135952	0	-0.0428739
0.0036	0	0	0	0	0	-5.6268214	-6.9077553	-6.9077553	-6.9077553	-6.9077553	-6.9077553	6	46	2.23519444	296.888889	23.88888888	0	0	0	0	18.9135952	0	-0.0428739
0.0396	0	0.41784134	45.2463715	1.57748966	0	-3.2289262	-6.9077553	-0.8702631	3.81214458	0.45646848	-6.9077553	7	42	2.23519444	296.333333	23.33333333	0	0	0	0	18.9135952	0	-0.0428739
0.1179	0	0.40626833	41.056433	1.66732876	0	-2.1379185	-6.9077553	-0.898283	3.71497189	0.51182238	-6.9077553	8	35	3.12927222	295.777778	22.7777778	0	0	0	0	18.9135952	0	-0.0428739
0.2475	131.59135	0.374614	35.3586561	1.60467667	0	-1.3963447	-2.0204834	-0.9791933	3.56557151	0.47354527	-6.9077553	9	36	4.91742778	294.666667	21.66666666	0	0	1	0	18.9135952	0	-0.0428739
0.342	789.548048	0.40047546	55.3185361	1.12942062	0	-1.0729445	-0.2350288	-0.9126089	4.01312612	0.12258979	-6.9077553	10	43	2.68223333	293	20	0	0	1	0	18.9135952	0	-0.0428739
0.4077	1863.67245	0.45577021	77.853315	0.81553524	0	-0.8972237	0.62308541	-0.7835748	4.35483932	-0.2026852	-6.9077553	11	54	0.44703889	290.222222	17.2222222	0	0	1	0	18.9135952	0	-0.0428739
0.495	4341.9899	0.61700361	130.091012	1.14069091	0	-0.7031975	1.46856303	-0.481261	4.86824198	0.13251042	-6.9077553	12	55	1.34111667	291.333333	18.33333333	0	0	1	0	18.9135952	0	-0.0428739
0.7011	7277.66283	0.56442294	107.619431	1.57613389	0	-0.3551047	1.98494717	-0.5701813	4.67861051	0.45560921	-6.9077553	13	46	3.12927222	294.111111	21.11111111	0	0	1	0	18.9135952	0	-0.0428739
1.1448	15551.2553	0.85060736	250.127472	3.52490284	0	0.13522995	2.74420566	-0.1606297	5.52197467	1.26013653	-6.9077553	14	40	5.36446667	295.777778	22.7777778	0	0	1	0	18.9135952	0	-0.0428739
1.7361	31280.6925	0.94409533	277.89512	4.19272022	0	0.55164122	3.44303302	-0.0564695	5.62724737	1.43358822	-6.9077553	15	35	6.25854444	297.444444	24.44444444	0	0	1	0	18.9135952	0	-0.0428739
2.5641	65728.4056	1.09317401	384.402344	4.09932622	0	0.94160754	4.1855464	0.08999975	5.95169238	1.41106654	-6.9077553	16	31	6.70558333	299.111111	26.11111111	0	0	1	0	18.9135952	0	-0.0428739
3.2949	96844.8714	1.05879008	343.563847	3.12425736	0	1.19237582	4.57312076	0.05807086	5.83937588	1.13951663	-6.9077553	17	27	6.25854444	300.777778	27.7777778	0	0	1	0	18.9135952	0	-0.0428739
4.3155	147945.943	1.2317323	476.229497	4.23528654	0	1.46221319	4.99685371	0.20923308	6.16590198	1.44368707	-6.9077553	18	24	7.59966111	301.888889	28.88888888	0	0	1	0	18.9135952	0	-0.0428739
5.2929	195124.608	1.1118553	366.866713	2.85780045	2	1.6663663	5.27364349	0.10692905	5.90500133	1.05040211	0.69364706	19	23	5.36446667	303.555556	30.55555555	0	0	1	0	18.9135952	0	-0.0428739
6.309	243169.114	1.08116249	351.72138	3.02356725	2	1.84197719	5.49376126	0.07896134	5.86284217	1.10676802	0.69364706	20	23	5.81150556	303.555556	30.55555555	0	0	1	0	18.9135952	0	-0.0428739
7.3899	294035.856	1.11248022	379.807452	3.38308734	2	2.0001142	5.68370512	0.10749044	5.93966705	1.21908425	0.69364706	21	22	6.25854444	304.111111	31.11111111	0	0	1	0	18.9135952	0	-0.0428739
8.352	339525.551	1.06376969	341.720621	2.68220519	2	2.12250103	5.82755215	0.06275852	5.83399643	0.98701204	0.69364706	22	23	5.36446667	304.111111	31.11111111	0	0	1	0	18.9135952	0	-0.0428739
9.2052	376535.489	0.97204094	268.957114	2.11929399	3	2.21976854	5.93101496	-0.0273291	5.59455566	0.75155475	1.09894557	23	24	4.91742778	303.555556	30.55555555	0	0	1	0	18.9135952	0	-0.0428739
0.0162	418.158655	0.78512085	177.57307	1.65930894	0	-4.122744	-0.8695058	-0.2406447	5.17938782	0.50700369	-6.9077553	0	71	3.57631111	302.444444	29.44444444	0	0	0	0	30.519848	0	0.21141735
0.0585	1454.63537	0.60298242	107.493995	1.32604534	0	-2.8387285	0.37544248	-0.5042102	4.67744429	0.28295492	-6.9077553	1	70	2.68223333	303	30	0	0	0	0	30.519848	0	0.21141735
0.1197	2447.81	0.62804357	122.047665	1.32696051	0	-2.1227667	0.89560219	-0.4635548	4.80441986	0.28364431	-6.9077553	2	67	3.12927222	303.555556	30.55555555	0	0	0	0	30.519848	0	0.21141735
0.1899	2976.12055	0.46179361	62.8257086	1.10491738	0	-1.6612577	1.09095657	-0.7704741	4.14038028	0.1006752	-6.9077553	3	65	2.68223333	303.555556	30.55555555	0	0	0	0	30.519848	0	0.21141735
0.3429	4176.50919	0.61409688	108.529044	2.06926718	0	-1.0703164	1.42971518	-0.4859755	4.68702704	0.72767767	-6.9077553	4	67	4.02335	303	30	0	0	0	1	30.519848	0	0.21141735
0.5382	7615.18897	0.74763004	169.615801	2.26831209	0	-0.619525	2.03027611	-0.2895104	5.13354178	0.81947674	-6.9077553	5	58	4.47038889	304.666667	31.66666666	0	0	0	1	30.519848	0	0.21141735
0.7776	14236.1327	0.77646895	173.15252	2.47178248	0	-0.251543	2.65585353	-0.2517116	5.1541786	0.90534403	-6.9077553	6	55	5.36446667	305.222222	32.2222222	0	0	0	1	30.519848	0	0.21141735
0.9999	22805.5914	0.69371669	134.569553	1.65655283	0	-0.0001	3.12704959	-0.3642512	4.90208862	0.50534231	-6.9077553	7	54	4.47038889	305.222222	32.2222222	0	0	0	1	30.519848	0	0.21141735
1.1376	31702.3052	0.68998379	141.520718	1.0593168	0	0.12892078	3.45642094	-0.3696389	4.95245319	0.05856773	-6.9077553	8	60	2.68223333	304.111111	31.11111111	0	0	0	1	30.519848	0	0.21141735
1.2447	39856.4273	0.81921951	182.446051	1.28336546	0	0.21889454	3.68530877	-0.1981833	5.20646	0.25026479	-6.9077553	9	67	1.34111667	302.444444	29.44444444	0	0	0	1	30.519848	0	0.21141735
1.3194	45056.2711	0.71700328	133.079819	1.09396776	0	0.27717709	3.80793437	-0.3312811	4.89095661	0.09072492	-6.9077553	10	73	2.23519444	300.777778	27.7777778	0	0	0	1	30.519848	0	0.21141735
1.3626	46898.55	0.56331976	80.0085653	0.81448428	0	0.30939464	3.84800808	-0.5721342	4.38214619	-0.2039731	-6.9077553	11	79	1.34111667	299.666667	26.66666666	0	0	0	1	30.519848	0	0.21141735
1.3815	47688.0973	0.3783845	33.7116845	0.52146081	0	0.32316987	3.8647028	-0.9692051	3.51787416	-0.6492053	-6.9077553	12	83	1.34111667	299.111111	26.11111111	0	0	0	1	30.519848	0	0.21141735
1.3986	48302.1898	0.24762811	13.2074063	0.34096454	0	0.33547174	3.8774976	-1.391797	2.58085347	-1.0730482	-6.9077553	13	85	2.68223333	298.555556	25.55555555	0	0	0	1	30.519848	0	0.21141735
1.4094	48784.6919	0.23340243	11.5816282	0.336133	0	0.34316408	3.88743707	-1.4507159	2.4495064	-1.0872778	-6.9077553	14	86	2.68223333	298	25	0	0	0	1	30.519848	0	0.21141735
1.4193	49223.3259	0.22336669	10.0535667	0.29352819	0	0.35016379	3.89638793	-1.4944735	2.30802693	-1.2223806	-6.9077553	15	90	2.23519444	298	25	0	0	0	1	30.519848	0	0.21141735
1.4265	49574.2388	0.20238042	8.3420887	0.25821525	0	0.35522389	3.90349149	-1.5926771	2.1214335	-1.3500965	-6.9077553	16	91	3.57631111	298	25	0	0	0	1	30.519848	0	0.21141735
1.4382	50144.4666	0.21042507	9.16694113	0.31784663	0	0.36339233	3.91492811	-1.5538846	2.21571274	-1.1430451	-6.9077553	17	90	3.12927222	298	25	0	0	0	1	30.519848	0	0.21141735
1.4463	50539.2426	0.21064084	8.80984667	0.26039235	0	0.36900857	3.9227699	-1.5528646	2.17598354	-1.3417328	-6.9077553	18	91	2.68223333	298	25	0	0	0	1	30.519848	0	0.21141735
1.4544	50934.016	0.21229666	9.19129859	0.2888204	0	0.37459344	3.93055062	-1.5450713	2.21836602	-1.2384939	-6.9077553	19	93	1.34111667	298	25	0	0	0	1	30.519848	0	0.21141735
1.4661	51504.2439	0.22817509	11.3860556	0.37581422	0	0.38260581	3.94168363	-1.473269	2.43247723	-0.976003	-6.9077553	20	93	2.23519444	298	25	0	0	0	1	30.519848	0	0.21141735
1.5183	52907.889	0.272881	24.9731151	0.56540193	0	0.41759129	3.96857136	-1.2950616	3.21783989	-0.5684513	-6.9077553	21	88	3.12927222	299.111111	26.11111111	0	0	0	1	30.519848	0	0.21141735
1.6479	57087.4196	0.43313842	57.6072591	0.96044498	0	0.49950175	4.04460129	-0.8343918	4.05366594	-0.0393179	-6.9077553	22	83	2.68223333	300.222222	27.2222222	0	0	0	1	30.519848	0	0.21141735
1.8396	62366.2846	0.53417256	76.9005144	1.38892676	0	0.60954816	4.13304085	-0.625166	4.34252557	0.32925106	-6.9077553	23	80	4.02335	300.777778	27.7777778	0	0	0	1	30.519848	0	0.21141735
0.0396	1540.8268	1.03219604	302.586255	3.11900188	0	-3.2289262	0.43296795	0.03265695	5.71236968	1.1378336	-6.9077553	0	71	3.57631111	302.444444	29.44444444	0	0	0	0	34.0724638	0	0.49277013
0.1134	4921.9484	0.90200406	219.067835	1.46852549	0	-2.1768339	1.59390762	-0.1020282	5.389386	0.38493955	-6.9077553	1	70	2.68223333	303	30	0	0	0	0	34.0724638	0	0.49277013
0.1926	8272.26353	0.84821961	184.226995	1.3101863	0	-1.6471398	2.11302905	-0.1634375	5.2161741	0.2709323	-6.9077553	2	67	3.12927222	303.555556	30.55555555	0	0	0	0	34.0724638	0	0.49277013
0.2853	11721.6174	0.86612035	205.393962	1.59739359	0	-1.254214	2.46152009	-0.1425775	5.32493477	0.46899912	-6.9077553	3	65	2.68223333	303.555556	30.55555555	0	0	0	1	34.0724638	0	0.49277013
0.5229	22393.2427	1.09608506	348.491418	2.98300864	0	-0.648365	3.10880391	0.09265671	5.85361647	1.09326758	-6.9077553	4	67	4.02335	303	30	0	0	0	1	34.0724638	0	0.49277013
1.0017	40333.8194	1.29460305	493.67675	4.04222807	0	0.00169856	3.6972151	0.25897626	6.20188298	1.3970434	-6.9077553	5	58	4.47038889	304.666667	31.66666666	0	0	0	1	34.0724638	0	0.49277013
1.7172	53728.9196	1.34508064	571.437028	4.65313636	1	0.54069506	3.98397001	0.29719714	6.34815604	1.53775636	0.0009995	6	55	5.36446667	305.222222	32.2222222	0	0	0	1	34.0724638	0	0.49277013
2.2707	66821.5699	0.97720372	274.659172	2.46274573	1	0.82008815	4.2020409	-0.0220373	5.61553459	0.90168285	0.0009995	7	54	4.47038889	305.222222	32.2222222	0	0	0	1	34.0724638	0	0.49277013
2.6829	82549.3917	0.89964108	227.940877	1.63383571	1	0.9868983	4.41340891	-0.1046485	5.42909067	0.49154231	0.0009995	8	60	2.68223333	304.111111	31.11111111	0	0	0	1	34.0724638	0	0.49277013
2.925	91775.5134	0.78523079	163.753137	1.218146	1	1.07329448	4.51935642	-0.2405049	5.09836614	0.19815061	0.0009995	9	67	1.34111667	302.444444	29.44444444	0	0	0	1	34.0724638	0	0.49277013
3.1815	102322.321	0.77548957	159.696848	1.31195347	1	1.15735278	4.62813762	-0.2529721	5.07328358	0.27227915	0.0009995	10	73	2.23519444	300.777778	27.7777778	0	0	0	1	34.0724638	0	0.49277013
3.3705	109867.764	0.63920637	105.529139	1.12324546	1	1.2150611	4.6992866	-0.4459647	4.65899659	0.11711211	0.0009995	11	79	1.34111667	299.666667	26.66666666	0	0	0	1	34.0724638	0	0.49277013
3.4605	113464.61	0.36278999	33.5661768	0.66436157	1	1.24141309	4.73149979	-1.0111785	3.51354871	-0.4074247	0.0009995	12	83	1.34111667	299.111111	26.11111111	0	0	0	1	34.0724638	0	0.49277013
3.5496	116929.841	0.31409393	29.2286044	0.5636241	1	1.26683492	4.76158266	-1.1548845	3.37518205	-0.5715951	0.0009995	13	85	2.68223333	298.555556	25.55555555	0	0	0	1	34.0724638	0	0.49277013
3.6153	119561.669	0.32684823	29.6966268	0.58318554	1	1.28517484	4.78384066	-1.1152045	3.39106714	-0.5375366	0.0009995	14	86	2.68223333	298	25	0	0	0	1	34.0724638	0	0.49277013
3.6909	122852.681	0.28614964	22.3748343	0.45930495	1	1.30587033	4.81099406	-1.2477518	3.10798155	-0.7758661	0.0009995	15	90	2.23519444	298	25	0	0	0	1	34.0724638	0	0.49277013
3.7467	125396.779	0.24603341	13.1621803	0.47080548	1	1.32087545	4.83149092	-1.3982317	2.57742356	-0.7511885	0.0009995	16	91	3.57631111	298	25	0	0	0	1	34.0724638	0	0.49277013
3.8133	128116.325	0.26088993	16.0862014	0.42687537	1	1.33849496	4.85294645	-1.339831	2.77802401	-0.8489233	0.0009995	17	90	3.12927222	298	25	0	0	0	1	34.0724638	0	0.49277013

3.8727	130660.436	0.2614325	16.92501	0.4880052	1	1.35395194	4.87260952	-1.3377614	2.82885149	-0.7153821	0.0009995	18	91	2.68223333	298	25	0	0	0	1	34.0724638	0	0.49277013
3.9384	133427.639	0.26801901	23.1185136	0.43624941	1	1.37077455	4.89356679	-1.3129732	3.140677	-0.8272515	0.0009995	19	93	1.34111667	298	25	0	0	0	1	34.0724638	0	0.49277013
3.9906	135533.087	0.28756737	21.6527647	0.44338532	1	1.3839416	4.90922318	-1.2428267	3.07517933	-0.8110633	0.0009995	20	93	2.23519444	298	25	0	0	0	1	34.0724638	0	0.49277013
4.0923	139262.848	0.48018326	70.8818532	0.85954379	1	1.40910716	4.93637032	-0.7315071	4.26102856	-0.1501908	0.0009995	21	88	3.12927222	299.111111	26.11111111	0	0	0	1	34.0724638	0	0.49277013
4.5027	148297.175	0.50837042	83.8709567	1.54958277	1	1.50467722	4.99922494	-0.6745798	4.42929131	0.43863084	0.0009995	22	83	2.68223333	300.222222	27.2222222	0	0	0	1	34.0724638	0	0.49277013
5.4306	164267.627	0.71340241	160.80762	2.32341174	1	1.69204963	5.10150306	-0.3363089	5.08021496	0.84346699	0.0009995	23	80	4.02335	300.777778	27.7777778	0	0	0	1	34.0724638	0	0.49277013
0.0504	3757.45094	1.30594621	481.682138	2.97241544	0	-2.9877641	1.32400689	0.26769328	6.17728651	1.08971127	-6.9077553	0	71	3.57631111	302.444444	29.44444444	0	0	0	0	42.288408	0	-0.4111751
0.2034	10173.0275	1.04508775	302.408502	3.31906702	0	-1.5925808	2.31983815	0.04505725	5.71178207	1.19998497	-6.9077553	1	70	2.68223333	303	30	0	0	0	0	42.288408	0	-0.4111751
0.4599	22931.7026	1.05943047	309.020175	2.59577551	0	-0.7767462	3.13256395	0.05867493	5.7334098	0.95427049	-6.9077553	2	67	3.12927222	303.555556	30.55555555	0	0	0	0	42.288408	0	-0.4111751
0.7443	37074.9427	0.98043591	265.408327	1.99534781	0	-0.2953111	3.61296832	-0.0187386	5.58127326	0.69131942	-6.9077553	3	65	2.68223333	303.555556	30.55555555	0	0	0	0	42.288408	0	-0.4111751
1.0458	48086.447	0.84858471	192.464401	1.74779618	0	0.04478214	3.87302117	-0.1630076	5.2599164	0.55892765	-6.9077553	4	67	4.02335	303	30	0	0	1	0	42.288408	0	-0.4111751
1.4058	63100.4281	0.86952682	199.689551	1.7350412	0	0.34060654	4.1447434	-0.1386567	5.29676892	0.55160735	-6.9077553	5	58	4.47038889	304.666667	31.66666666	0	0	1	0	42.288408	0	-0.4111751
1.9593	82855.586	0.89863827	229.668801	2.57038296	0	0.67258727	4.41711123	-0.1057625	5.43664263	0.94444387	-6.9077553	6	55	5.36446667	305.222222	32.2222222	0	0	1	0	42.288408	0	-0.4111751
2.4867	102481.537	0.83906623	198.468643	1.97921972	0	0.91095653	4.62969241	-0.1742745	5.29063616	0.68320781	-6.9077553	7	54	4.47038889	305.222222	32.2222222	0	0	1	0	42.288408	0	-0.4111751
2.9025	121625.738	0.88203767	213.801475	1.51154423	0	1.06557243	4.80095683	-0.1243874	5.36505257	0.41379315	-6.9077553	8	60	2.68223333	304.111111	31.11111111	0	0	1	0	42.288408	0	-0.4111751
3.3021	142041.579	0.94512486	245.789045	1.77032512	0	1.19455863	4.95612687	-0.0553807	5.5044777	0.57172792	-6.9077553	9	67	1.34111667	302.444444	29.44444444	0	0	1	0	42.288408	0	-0.4111751
3.7107	162418.515	0.91257319	223.501015	1.74468873	0	1.31122054	5.09018258	-0.0903918	5.40942043	0.55714917	-6.9077553	10	73	2.23519444	300.777778	27.7777778	0	0	1	0	42.288408	0	-0.4111751
4.0041	175545.162	0.68617904	121.722465	1.24220529	0	1.38731884	5.16790204	-0.3751604	4.80175179	0.21769295	-6.9077553	11	79	1.34111667	299.666667	26.66666666	0	0	1	0	42.288408	0	-0.4111751
4.1094	180107.005	0.36819533	40.096158	0.71094656	0	1.41327703	5.1935567	-0.9964294	3.69130546	-0.3397524	-6.9077553	12	83	1.34111667	299.111111	26.11111111	0	0	1	0	42.288408	0	-0.4111751
4.2021	184054.751	0.2845923	20.4536607	0.59770458	0	1.4355844	5.21523871	-1.25319	3.01821076	-0.512987	-6.9077553	13	85	2.68223333	298.555556	25.55555555	0	0	1	0	42.288408	0	-0.4111751
4.2777	187388.391	0.26167761	15.3051133	0.47882761	0	1.45341548	5.23318876	-1.3368278	2.72825231	-0.7343284	-6.9077553	14	86	2.68223333	298	25	0	0	1	0	42.288408	0	-0.4111751
4.3569	190678.178	0.24943432	13.7690624	0.42130158	0	1.4717608	5.25059232	-1.3845586	2.62249684	-0.8620356	-6.9077553	15	90	2.23519444	298	25	0	0	1	0	42.288408	0	-0.4111751
4.4172	193266.133	0.25820906	14.5275688	0.42270202	0	1.48550601	5.26407334	-1.3501204	2.67611697	-0.8587249	-6.9077553	16	91	3.57631111	298	25	0	0	1	0	42.288408	0	-0.4111751
4.4703	195415.466	0.23229851	11.4059965	0.33349218	0	1.49745552	5.275133	-1.4554365	2.4342269	-1.0951418	-6.9077553	17	90	3.12927222	298	25	0	0	1	0	42.288408	0	-0.4111751
4.5198	197520.916	0.21751799	10.377326	0.34606417	0	1.50846775	5.28584955	-1.5208869	2.33971959	-1.0582456	-6.9077553	18	91	2.68223333	298	25	0	0	1	0	42.288408	0	-0.4111751
4.5675	199450.931	0.23027312	11.50869	0.37309841	0	1.51896601	5.29557326	-1.4641559	2.44318929	-0.9832364	-6.9077553	19	93	1.34111667	298	25	0	0	1	0	42.288408	0	-0.4111751
4.6197	201687.979	0.23943839	12.9936913	0.36760248	0	1.53032977	5.3067268	-1.4252914	2.56454091	-0.9980365	-6.9077553	20	93	2.23519444	298	25	0	0	1	0	42.288408	0	-0.4111751
4.7619	205728.942	0.43706382	59.9742509	1.05174449	0	1.56064675	5.32656435	-0.8253907	4.09393199	0.05140055	-6.9077553	21	88	3.12927222	299.111111	26.11111111	0	0	1	0	42.288408	0	-0.4111751
5.1147	214821.647	0.52630794	92.4179787	1.17870276	0	1.63211875	5.36981279	-0.6399706	4.52633235	0.16526251	-6.9077553	22	83	2.68223333	300.222222	27.2222222	0	0	1	0	42.288408	0	-0.4111751
5.7375	230679.33	0.66530856	137.767409	1.69289156	0	1.74702358	5.4410329	-0.4060024	4.92557408	0.52702858	-6.9077553	23	80	4.02335	300.777778	27.7777778	0	0	1	0	42.288408	0	-0.4111751
0.0099	0	0.56151919	86.0996826	2.12463913	0	-4.6152205	-6.9077553	-0.57533	4.45551734	0.75407252	-6.9077553	0	71	3.57631111	302.444444	29.44444444	0	0	0	0	18.9135952	0	-0.0428739
0.045	0	0.45108203	53.722049	1.58175268	0	-3.1010928	-6.9077553	-0.7938916	3.98384213	0.45916553	-6.9077553	1	70	2.68223333	303	30	0	0	0	0	18.9135952	0	-0.0428739
0.1422	358.579694	0.5775712	92.8288032	1.77865855	0	-1.9505208	-1.0228194	-0.5471937	4.53076774	0.57642152	-6.9077553	2	67	3.12927222	303.555556	30.55555555	0	0	0	0	18.9135952	0	-0.0428739
0.2745	3089.79908	0.70861557	162.167849	1.71662768	0	-1.292804	1.12842966	-0.3430319	5.08863807	0.54094408	-6.9077553	3	65	2.68223333	303.555556	30.55555555	0	0	1	0	18.9135952	0	-0.0428739
0.4923	10848.1766	0.82598593	234.278458	2.16459971	0	-0.708667	2.38408919	-0.1899676	5.45651467	0.77269733	-6.9077553	4	67	4.02335	303	30	0	0	1	0	18.9135952	0	-0.0428739
0.6831	17499.3938	0.83489004	234.943986	2.03098312	0	-0.381114	2.86222338	-0.1792582	5.45935138	0.70901222	-6.9077553	5	58	4.47038889	304.666667	31.66666666	0	0	1	0	18.9135952	0	-0.0428739
0.9288	25582.2778	0.87599814	246.067193	2.36430074	0	-0.0738618	3.24193893	-0.1312504	5.50560871	0.86090518	-6.9077553	6	55	5.36446667	305.222222	32.2222222	0	0	1	0	18.9135952	0	-0.0428739
1.2177	35257.1206	0.83785165	211.639116	2.14254238	0	0.19696383	3.56269588	-0.1757214	5.35488727	0.76245978	-6.9077553	7	54	4.47038889	305.222222	32.2222222	0	0	1	0	18.9135952	0	-0.0428739
1.4337	44094.7062	0.85466652	211.603125	1.57188301	0	0.36025852	3.78636241	-0.1558746	5.35471719	0.45291025	-6.9077553	8	60	2.68223333	304.111111	31.11111111	0	0	1	0	18.9135952	0	-0.0428739
1.5804	51011.6509	0.86674565	201.086489	1.46104366	0	0.45767798	3.93207366	-0.1418566	5.30374008	0.37983522	-6.9077553	9	67	1.34111667	302.444444	29.44444444	0	0	1	0	18.9135952	0	-0.0428739
1.7073	56829.1916	0.78190959	160.267107	1.26753592	0	0.53491318	4.04006773	-0.2447381	5.07684808	0.23786342	-6.9077553	10	73	2.23519444	300.777778	27.7777778	0	0	1	0	18.9135952	0	-0.0428739
1.7991	60952.3834	0.60190638	89.8161669	0.9105826	0	0.58728654	4.11010937	-0.5059933	4.49777613	-0.0925731	-6.9077553	11	79	1.34111667	299.666667	26.66666666	0	0	1	0	18.9135952	0	-0.0428739
1.8495	63189.441	0.32082776	23.1241564	0.49769906	0	0.61491533	4.14615304	-1.1337388	3.14092105	-0.6957525	-6.9077553	12	83	1.34111667	299.111111	26.11111111	0	0	1	0	18.9135952	0	-0.0428739
1.908	65821.2684	0.30052927	19.8186392	0.5130887	0	0.64605557	4.18695821	-1.1988882	2.98667332	-0.6653595	-6.9077553	13	85	2.68223333	298.555556	25.55555555	0	0	1	0	18.9135952	0	-0.0428739
1.9638	68365.3623	0.29893734	19.5525198	0.5114891	0	0.67488137	4.22488092	-1.2041817	2.97315531	-0.6684758	-6.9077553	14	86	2.68223333	298	25	0	0	1	0	18.9135952	0	-0.0428739
2.0205	70997.1908	0.28042515	17.1153661	0.47003619	0	0.70334501	4.26265439	-1.2678888	2.84003509	-0.7528204	-6.9077553	15	90	2.23519444	298	25	0	0	1	0	18.9135952	0	-0.0428739
2.079	73672.8823	0.27679118	16.6713548	0.45994336	0	0.73188701	4.29964836	-1.2808856	2.81375195	-0.7744801	-6.9077553	16	91	3.57631111	298	25	0	0	1	0	18.9135952	0	-0.0428739
2.1348	76260.8457	0.27263623	16.2385421	0.46928409	0	0.75837297	4.33417276	-1.2959557	2.78744914	-0.7544183	-6.9077553	17	90	3.12927222	298	25	0	0	1	0	18.9135952	0	-0.0428739
2.1906	78804.9447	0.24818408	13.2186437	0.41215161	0	0.78417548	4.36698843	-1.3895634	2.58170388	-0.8839407	-6.9077553	18	91	2.68223333	298	25	0	0	1	0	18.9135952	0	-0.0428739
2.2356	80734.9493	0.23255684	11.4875317	0.358204	0	0.80450965	4.39118394	-1.4543298	2.4413493	-1.0238648	-6.9077553	19	93	1.34111667	298	25	0	0	1	0	18.9135952	0	-0.0428739
2.277	82401.7733	0.23153027	11.600781	0.33040741	0	0.82285879	4.41161909	-1.4587349	2.45115862	-1.1044068	-6.9077553	20	93	2.23519444	298	25	0	0	1	0	18.9135952	0	-0.0428739
2.3931	86586.2814	0.54604428	92.0899497	1.19185621	0	0.8725896	4.46115294	-0.6032255	4.52277667	0.17635061	-6.9077553	21	88	3.12927222	299.111111	26.11111111	0	0	1	0	18.9135952	0	-0.0428739
2.7504	97426.146	0.63729316	131.49693	1.52529592	0	1.01174636	4.57910488	-0.4489576	4.87899111	0.42284383	-6.9077553	22	83	2.68223333	300.222222	27.2222222	0	0	1	0	18.9135952	0	-0.0428739
3.2022	109515.37	0.71277427	154.56899	1.89951279	0	1.16383807	4.69607403	-0.3371885	5.040647	0.64212374	-6.9077553	23	80	4.02335	300.777778	27.7777778	0	0	1	0	18.9135952	0	-0.0428739
0.0117	361.053394	0.75148145	169.541911	1.37356567	0	-4.4481664	-1.0159636	-0.2843789	5.13310606	0.31813781	-6.9077553	0	68	2.68223333	303.555556	30.55555555	0	0	0	0	30.519848	0	0.21141735
0.0252	932.059522	0.71658484	133.542057	1.18591042	0	-3.6809113	-0.0692863	-0.3318641	4.89442395	0.17135364	-6.9077553	1	73	2.23519444	301.888889	28.88888888	0	0	0	0	30.519848	0	0.21141735
0.0396	1326.83367	0.68703789	126.52916	1.30980229	0	-3.2289262	0.28354879	-0.3739114	4.8404807	0.27063939	-6.9077553	2	77	1.78815556	300.777778	27.7777778	0	0	0	0	30.519848	0	0.21141735
0.0432	1458.4251	0.36612058	35.9827118	0.91665804	0	-3.1419148	0.37804259	-1.0020649	3.58306639	-0.0859305	-6.9077553	3	80	1.34111667	300.222222	27.2222222	0	0	0	0	30.519848	0	0.21141735
0.045	1458.42499	0.33855855	24.7481899	0.37199211	0	-3.1010928	0.37804251	-1.0801089	3.20879276	-0.986198	-6.9077553	4	84	2.68223333	299.666667	26.66666666	0	0	0	0	30.519848	0	0.21141735
0.0459	1502.28878	0.26214185	14.1419792	0.41642639	0	-3.0812902	0.40765523	-1.335062	2.64921833	-0.8736471	-6.9077553	5	88	1.78815556	299.111111	26.11111111	0	0	0	0	30.519848	0	0.21141735
0.0459	1502.28878	0	0	0	0	-3.0812902	0.40765523	-6.9077553	-6.9077553	-6.9077553	-6.9077553	6	89	2.68223333	299.666667	26.66666666	0	0	0	0	30.519848	0	0.21141735
0.0459	1502.28878	0	0	0	0	-3.0812902	0.40765523	-6.9077553	-6.9077553	-6.9077553	-6.9077553	7	86	4.02335	300.222222	27.2222222	0	0	0	0	30.519848	0	0.21141735

0.0459	1502.28878	0	0	0	0	-3.0812902	0.40765523	-6.9077553	-6.9077553	-6.9077553	-6.9077553	8	87	3.57631111	300.222222	27.2222222	0	0	0	0	30.519848	0	0.21141735
0.0459	1502.28878	0	0	0	0	-3.0812902	0.40765523	-6.9077553	-6.9077553	-6.9077553	-6.9077553	9	87	3.57631111	300.222222	27.2222222	0	0	0	0	30.519848	0	0.21141735
0.0459	1502.28878	0	0	0	0	-3.0812902	0.40765523	-6.9077553	-6.9077553	-6.9077553	-6.9077553	10	87	4.02335	300.222222	27.2222222	0	0	0	0	30.519848	0	0.21141735
0.0459	1502.28878	0	0	0	0	-3.0812902	0.40765523	-6.9077553	-6.9077553	-6.9077553	-6.9077553	11	88	2.68223333	300.222222	27.2222222	0	0	0	0	30.519848	0	0.21141735
0.0459	1502.28878	0	0	0	0	-3.0812902	0.40765523	-6.9077553	-6.9077553	-6.9077553	-6.9077553	12	84	3.57631111	300.777778	27.7777778	0	0	0	0	30.519848	0	0.21141735
0.0873	1633.88011	0.33451549	28.5363876	1.22843842	0	-2.4384048	0.49156948	-1.0920872	3.35121507	0.2065575	-6.9077553	13	78	3.57631111	301.888889	28.88888888	0	0	0	0	30.519848	0	0.21141735
0.1755	2197.95396	0.45152318	57.4818688	1.73621913	0	-1.7401162	0.78798178	-0.7929163	4.05148697	0.55228564	-6.9077553	14	76	4.02335	301.888889	28.88888888	0	0	0	0	30.519848	0	0.21141735
0.2799	3327.05978	0.57379729	91.2792716	1.93386012	0	-1.2733229	1.20238948	-0.5537378	4.51393468	0.66003504	-6.9077553	15	66	4.02335	304.111111	31.11111111	0	0	0	0	30.519848	0	0.21141735
0.3672	3979.59787	0.56828454	84.7607221	1.5995669	0	-1.0018486	1.38143203	-0.5633749	4.43984405	0.47035788	-6.9077553	16	63	3.57631111	304.666667	31.66666666	0	0	0	1	30.519848	0	0.21141735
0.567	8102.86343	0.78358906	184.066002	2.61037933	0	-0.567396	2.09234091	-0.2425952	5.21529983	0.95987856	-6.9077553	17	57	5.81150556	305.777778	32.7777778	0	0	0	1	30.519848	0	0.21141735
0.8379	18186.1323	0.85143272	217.581876	2.82678425	0	-0.1768565	2.90071433	-0.159661	5.38257981	1.03949346	-6.9077553	18	56	5.36446667	305.777778	32.7777778	0	0	0	1	30.519848	0	0.21141735
1.143	25868.8979	0.71303435	140.823193	2.21033003	0	0.13365638	3.25308005	-0.3368242	4.94751226	0.79359416	-6.9077553	19	55	5.81150556	306.333333	33.33333333	0	0	0	1	30.519848	0	0.21141735
1.3662	34490.0817	0.65716602	120.73291	1.33212846	0	0.31203316	3.54070079	-0.4182981	4.79358903	0.28752841	-6.9077553	20	59	3.12927222	305.777778	32.7777778	0	0	0	1	30.519848	0	0.21141735
1.5453	42906.6638	0.72438724	151.761562	1.26550592	0	0.43521807	3.75905045	-0.3210496	5.02231721	0.23626186	-6.9077553	21	59	3.12927222	305.222222	32.2222222	0	0	0	1	30.519848	0	0.21141735
1.6533	50099.6536	0.71351649	139.472852	0.99773788	0	0.50277329	3.91403405	-0.3361492	4.93787714	-0.0012629	-6.9077553	22	65	1.78815556	304.666667	31.66666666	0	0	0	1	30.519848	0	0.21141735
1.7325	56008.35	0.71716239	138.496183	0.97830356	0	0.54956545	4.02551864	-0.3310596	4.93084998	-0.0209136	-6.9077553	23	70	1.34111667	304.111111	31.11111111	0	0	0	1	30.519848	0	0.21141735
0.0225	958.567919	0.98404725	271.649597	2.09199079	0	-3.79424	-0.0412722	-0.0150657	5.60451667	0.73859404	-6.9077553	0	68	2.68223333	303.555556	30.55555555	0	0	0	0	34.0724638	0	0.49277013
0.0423	1616.52472	0.70425957	123.232089	0.95341102	0	-3.1629682	0.48089703	-0.3491894	4.81407759	-0.0466609	-6.9077553	1	73	2.23519444	301.888889	28.88888888	0	0	0	0	34.0724638	0	0.49277013
0.0558	2186.75389	0.52119376	69.5655762	0.71582305	0	-2.8859814	0.7828754	-0.6497166	4.24228422	-0.3329263	-6.9077553	2	77	1.78815556	300.777778	27.7777778	0	0	0	0	34.0724638	0	0.49277013
0.0738	2888.57428	0.41889009	44.847464	0.78533058	0	-2.6063965	1.06110918	-0.8677623	3.80328934	-0.240378	-6.9077553	3	80	1.34111667	300.222222	27.2222222	0	0	0	0	34.0724638	0	0.49277013
0.09	3590.39482	0.40707252	39.2164103	0.71972095	0	-2.4079456	1.27854066	-0.8963104	3.66912079	-0.3275033	-6.9077553	4	84	2.68223333	299.666667	26.66666666	0	0	0	0	34.0724638	0	0.49277013
0.1035	4248.35166	0.3129556	21.4160706	0.47270676	0	-2.2681837	1.44676642	-1.1585037	3.06418829	-0.7471668	-6.9077553	5	88	1.78815556	299.111111	26.11111111	0	0	0	0	34.0724638	0	0.49277013
0.1179	4862.44452	0.26149854	14.2583656	0.35246041	0	-2.1379185	1.58174694	-1.3375098	2.65741393	-1.0399838	-6.9077553	6	89	2.68223333	299.666667	26.66666666	0	0	0	0	34.0724638	0	0.49277013
0.1359	5695.85667	0.26268003	14.5029984	0.36307936	0	-1.995836	1.73991456	-1.3330189	2.67442436	-1.0103834	-6.9077553	7	86	4.02335	300.222222	27.2222222	0	0	0	0	34.0724638	0	0.49277013
0.1512	6397.67687	0.24953609	13.4721285	0.33933488	0	-1.8891518	1.85609123	-1.3841523	2.60069722	-1.0778252	-6.9077553	8	87	3.57631111	300.222222	27.2222222	0	0	0	0	34.0724638	0	0.49277013
0.1656	7055.63306	0.23256229	11.0045776	0.28038955	0	-1.79818	1.95396803	-1.4543065	2.3984022	-1.2680153	-6.9077553	9	87	3.57631111	300.222222	27.2222222	0	0	0	0	34.0724638	0	0.49277013
0.1809	7757.45468	0.22312	10.1394187	0.29184263	0	-1.7098109	2.04878318	-1.4955736	2.31652929	-1.2281199	-6.9077553	10	87	4.02335	300.222222	27.2222222	0	0	0	0	34.0724638	0	0.49277013
0.1944	8371.54689	0.2393669	11.7945964	0.3022096	0	-1.6378374	2.12495813	-1.4255888	2.46772627	-1.193331	-6.9077553	11	88	2.68223333	300.222222	27.2222222	0	0	0	0	34.0724638	0	0.49277013
0.2286	9720.5432	0.57141565	101.299548	1.00673485	0	-1.4757815	2.27434437	-0.5578899	4.61809182	0.00770509	-6.9077553	12	84	3.57631111	300.777778	27.7777778	0	0	0	0	34.0724638	0	0.49277013
0.3888	15689.4507	0.92934486	259.354218	2.172706	0	-0.9446902	2.75305229	-0.0721999	5.55819862	0.77643355	-6.9077553	13	78	3.57631111	301.888889	28.88888888	0	0	0	0	34.0724638	0	0.49277013
0.702	24450.0179	1.06593296	339.127986	3.05167573	0	-0.3538219	3.19667185	0.06478814	5.82638052	1.11601849	-6.9077553	14	76	4.02335	301.888889	28.88888888	0	0	0	1	34.0724638	0	0.49277013
1.215	33857.1342	1.26816567	483.933827	3.7552139	0	0.19474408	3.52217927	0.23835973	6.18195024	1.32341151	-6.9077553	15	66	4.02335	304.111111	31.11111111	0	0	0	1	34.0724638	0	0.49277013
1.7937	44852.8985	1.14500966	388.932494	3.179364	0	0.58428053	3.80341051	0.13628605	5.96340836	1.15699566	-6.9077553	16	63	3.57631111	304.666667	31.66666666	0	0	0	1	34.0724638	0	0.49277013
2.4435	65009.5565	1.128479	384.425641	3.1696703	1	0.89343144	4.17454966	0.12175647	5.95175298	1.15394302	0.0009995	17	57	5.81150556	305.777778	32.7777778	0	0	0	1	34.0724638	0	0.49277013
2.9619	82256.3444	0.91564676	231.531386	1.95137575	1	1.08583095	4.40985268	-0.0870331	5.44471976	0.66904696	0.0009995	18	56	5.36446667	305.777778	32.7777778	0	0	0	1	34.0724638	0	0.49277013
3.6252	108262.529	1.02261489	301.075432	2.86326734	1	1.28790946	4.68456834	0.02334037	5.70736416	1.05231259	0.0009995	19	55	5.81150556	306.333333	33.33333333	0	0	0	1	34.0724638	0	0.49277013
4.3848	132967.333	0.92470113	239.236337	2.59608611	1	1.47814401	4.890111	-0.0772038	5.4774561	0.95439009	0.0009995	20	59	3.12927222	305.777778	32.7777778	0	0	0	1	34.0724638	0	0.49277013
4.9491	147157.22	0.78232605	176.15002	1.8582606	1	1.59920574	4.99150833	-0.2442063	5.17134169	0.62017888	0.0009995	21	59	3.12927222	305.222222	32.2222222	0	0	0	1	34.0724638	0	0.49277013
5.2857	158279.382	0.75684899	160.815957	1.40226427	1	1.66500506	5.06436803	-0.2772711	5.08026681	0.33880115	0.0009995	22	65	1.78815556	304.666667	31.66666666	0	0	0	1	34.0724638	0	0.49277013
5.4918	166757.478	0.77298773	159.693027	1.23177488	1	1.70325607	5.11654653	-0.2561993	5.07325965	0.20926762	0.0009995	23	70	1.34111667	304.111111	31.11111111	0	0	0	1	34.0724638	0	0.49277013
0.0351	2321.22491	1.15807794	371.441997	2.41490736	0	-3.3495541	0.84252574	0.14762481	5.91739541	0.88207493	-6.9077553	0	68	2.68223333	303.555556	30.55555555	0	0	0	0	42.288408	0	-0.4111751
0.1053	6081.45166	0.92075573	230.752078	1.79338132	0	-2.2509419	1.80540785	-0.081475	5.44134821	0.5846603	-6.9077553	1	73	2.23519444	301.888889	28.88888888	0	0	0	0	42.288408	0	-0.4111751
0.1683	9064.29656	0.67697694	118.72433	1.22299892	0	-1.7820072	2.20445356	-0.388642	4.77681268	0.2021233	-6.9077553	2	77	1.78815556	300.777778	27.7777778	0	0	0	0	42.288408	0	-0.4111751
0.2241	11696.1241	0.5785827	82.5309035	1.06626991	0	-1.4956629	2.459343	-0.5454469	4.41318493	0.0651039	-6.9077553	3	80	1.34111667	300.222222	27.2222222	0	0	0	0	42.288408	0	-0.4111751
0.2583	13362.9472	0.44282677	45.742618	0.77493311	0	-1.3536336	2.59256058	-0.8123209	3.82305228	-0.253689	-6.9077553	4	84	2.68223333	299.666667	26.66666666	0	0	0	0	42.288408	0	-0.4111751
0.2943	15029.77	0.3116683	21.7918457	0.56365499	0	-1.2231556	2.71009943	-1.1626124	3.08158174	-0.5715404	-6.9077553	5	88	1.78815556	299.111111	26.11111111	0	0	0	0	42.288408	0	-0.4111751
0.3294	16608.8663	0.31331138	21.4700223	0.54455943	0	-1.1104825	2.80999687	-1.1573711	3.06670423	-0.6059435	-6.9077553	6	89	2.68223333	299.666667	26.66666666	0	0	0	0	42.288408	0	-0.4111751
0.3618	18144.0995	0.29526091	19.1643914	0.5036504	0	-1.0166637	2.89840053	-1.2165148	2.95310612	-0.6838894	-6.9077553	7	86	4.02335	300.222222	27.2222222	0	0	0	0	42.288408	0	-0.4111751
0.3906	19460.0135	0.27995542	16.9982758	0.45507073	0	-0.9400713	2.96841316	-1.2695593	2.83317074	-0.7851074	-6.9077553	8	87	3.57631111	300.222222	27.2222222	0	0	0	0	42.288408	0	-0.4111751
0.4284	21126.8378	0.24457355	13.0134583	0.37703737	0	-0.8476979	3.0505915	-1.4041588	2.56606091	-0.9727622	-6.9077553	9	87	3.57631111	300.222222	27.2222222	0	0	0	0	42.288408	0	-0.4111751
0.4563	22442.7512	0.26485957	15.5823187	0.43700621	0	-0.7846048	3.11101223	-1.324787	2.74620103	-0.8255222	-6.9077553	10	87	4.02335	300.222222	27.2222222	0	0	0	0	42.288408	0	-0.4111751
0.4968	24372.7559	0.3203412	28.5878038	0.48507699	0	-0.6995677	3.19350697	-1.1352518	3.35301517	-0.7213883	-6.9077553	11	88	2.68223333	300.222222	27.2222222	0	0	1	0	42.288408	0	-0.4111751
0.5913	28969.9281	0.70071774	141.513235	1.19196196	0	-0.5254318	3.36629285	-0.354224	4.95240031	0.17643926	-6.9077553	12	84	3.57631111	300.777778	27.7777778	0	0	1	0	42.288408	0	-0.4111751
0.8352	36169.0128	0.68367573	140.414971	1.65797182	0	-0.1800841	3.5882304	-0.3788099	4.94460924	0.50619803	-6.9077553	13	78	3.57631111	301.888889	28.88888888	0	0	1	0	42.288408	0	-0.4111751
1.2033	48133.7721	0.79038192	170.350436	1.89941988	0	0.18506778	3.87400483	-0.2339746	5.13786357	0.64207485	-6.9077553	14	76	4.02335	301.888889	28.88888888	0	0	1	0	42.288408	0	-0.4111751
1.5705	60303.3827	0.78484359	160.962201	1.56162606	0	0.45139404	4.09940478	-0.2409975	5.08117578	0.44636778	-6.9077553	15	66	4.02335	304.111111	31.11111111	0	0	1	0	42.288408	0	-0.4111751
1.944	75062.2785	0.81388072	173.529593	1.46247309	0	0.66474771	4.31833147	-0.2047135	5.15635391	0.38081244	-6.9077553	16	63	3.57631111	304.666667	31.66666666	0	0	1	0	42.288408	0	-0.4111751
2.5362	99092.3843	0.93978576	250.577341	2.43582088	0	0.9306669	4.59606268	-0.0610398	5.52377161	0.89069427	-6.9077553	17	57	5.81150556	305.777778	32.7777778	0	0	1	0	42.288408	0	-0.4111751
3.3642	131876.935	0.96079579	264.155299	2.72939798	0	1.21319019	4.88187676	-0.0389531	5.57654097	1.00444738	-6.9077553	18	56	5.36446667	305.777778	32.7777778	0	0	1	0	42.288408	0	-0.4111751
4.2246	173377.719	1.01338406	297.719208	2.51810088	0	1.44092458	5.15547833	0.0142816	5.69615414	0.92390205	-6.9077553	19	55	5.81150556	306.333333	33.33333333	0	0	1	0	42.288408	0	-0.4111751
5.0517	210204.445	0.97280077	273.109205	2.22119579	0	1.61972482	5.34808536	-0.0265485	5.60987539	0.7984958	-6.9077553	20	59	3.12927222	305.777778	32.7777778	0	0	1	0	42.288408	0	-0.4111751
5.6637	233177.759	0.83628064	203.414545	1.7259981	0	1.73407739	5.45180537	-0.177596	5.31525091	0.5463847	-6.9077553	21	59	3.12927222	305.222222	32.2222222	0	0	1	0	42.288408	0	-0.4111751

6.0426	246435.243	0.75809491	167.390922	1.31792329	0	1.79883438	5.50710331	-0.2756285	5.1203379	0.27681571	-6.9077553	22	65	1.78815556	304.666667	31.66666666	0	0	1	0	42.288408	0	-0.4111751
6.2172	253853.385	0.80466784	172.94352	1.30975342	0	1.82731964	5.53676082	-0.2160837	5.15297085	0.2706021	-6.9077553	23	70	1.34111667	304.111111	31.11111111	0	0	1	0	42.288408	0	-0.4111751
0.0009	0	0	0	0	0	-7.0131158	-6.9077553	-6.9077553	-6.9077553	-6.9077553	-6.9077553	0	68	2.68223333	303.555556	30.55555555	0	0	0	0	18.9135952	0	-0.0428739
0.0009	0	0	0	0	0	-7.0131158	-6.9077553	-6.9077553	-6.9077553	-6.9077553	-6.9077553	1	73	2.23519444	301.888889	28.88888888	0	0	0	0	18.9135952	0	-0.0428739
0.0009	0	0	0	0	0	-7.0131158	-6.9077553	-6.9077553	-6.9077553	-6.9077553	-6.9077553	2	77	1.78815556	300.777778	27.7777778	0	0	0	0	18.9135952	0	-0.0428739
0.0009	0	0	0	0	0	-7.0131158	-6.9077553	-6.9077553	-6.9077553	-6.9077553	-6.9077553	3	80	1.34111667	300.222222	27.2222222	0	0	0	0	18.9135952	0	-0.0428739
0.0009	0	0	0	0	0	-7.0131158	-6.9077553	-6.9077553	-6.9077553	-6.9077553	-6.9077553	4	84	2.68223333	299.666667	26.66666666	0	0	0	0	18.9135952	0	-0.0428739
0.0009	0	0	0	0	0	-7.0131158	-6.9077553	-6.9077553	-6.9077553	-6.9077553	-6.9077553	5	88	1.78815556	299.111111	26.11111111	0	0	0	0	18.9135952	0	-0.0428739
0.0009	0	0	0	0	0	-7.0131158	-6.9077553	-6.9077553	-6.9077553	-6.9077553	-6.9077553	6	89	2.68223333	299.666667	26.66666666	0	0	0	0	18.9135952	0	-0.0428739
0.0009	0	0	0	0	0	-7.0131158	-6.9077553	-6.9077553	-6.9077553	-6.9077553	-6.9077553	7	86	4.02335	300.222222	27.2222222	0	0	0	0	18.9135952	0	-0.0428739
0.0009	0	0	0	0	0	-7.0131158	-6.9077553	-6.9077553	-6.9077553	-6.9077553	-6.9077553	8	87	3.57631111	300.222222	27.2222222	0	0	0	0	18.9135952	0	-0.0428739
0.0009	0	0	0	0	0	-7.0131158	-6.9077553	-6.9077553	-6.9077553	-6.9077553	-6.9077553	9	87	3.57631111	300.222222	27.2222222	0	0	0	0	18.9135952	0	-0.0428739
0.0009	0	0	0	0	0	-7.0131158	-6.9077553	-6.9077553	-6.9077553	-6.9077553	-6.9077553	10	87	4.02335	300.222222	27.2222222	0	0	0	0	18.9135952	0	-0.0428739
0.0009	0	0	0	0	0	-7.0131158	-6.9077553	-6.9077553	-6.9077553	-6.9077553	-6.9077553	11	88	2.68223333	300.222222	27.2222222	0	0	0	0	18.9135952	0	-0.0428739
0.0009	0	0	0	0	0	-7.0131158	-6.9077553	-6.9077553	-6.9077553	-6.9077553	-6.9077553	12	84	3.57631111	300.777778	27.7777778	0	0	0	0	18.9135952	0	-0.0428739
0.0036	0	0.37611675	41.6883723	1.39623419	0	-5.6268214	-6.9077553	-0.9752005	3.73024624	0.33449471	-6.9077553	13	78	3.57631111	301.888889	28.88888888	0	0	0	0	18.9135952	0	-0.0428739
0.0423	60.8063135	0.60508617	108.732161	2.35884307	0	-3.1629682	-2.7837498	-0.5007331	4.68889681	0.85859512	-6.9077553	14	76	4.02335	301.888889	28.88888888	0	0	0	0	18.9135952	0	-0.0428739
0.117	762.896536	0.57106303	86.9790039	1.638791	0	-2.1455813	-0.2693229	-0.5585061	4.46567825	0.4945688	-6.9077553	15	66	4.02335	304.111111	31.11111111	0	0	0	0	18.9135952	0	-0.0428739
0.2556	3282.21662	0.67824307	139.852894	1.72228687	0	-1.3641416	1.18882362	-0.3867762	4.94059826	0.54423344	-6.9077553	16	63	3.57631111	304.666667	31.66666666	0	0	1	0	18.9135952	0	-0.0428739
0.5031	11881.3483	0.8756215	259.617614	2.69964844	0	-0.6869663	2.47505396	-0.13168	5.55921368	0.99349191	-6.9077553	17	57	5.81150556	305.777778	32.7777778	0	0	1	0	18.9135952	0	-0.0428739
0.7677	21706.8048	0.90167454	274.427136	2.44912242	0	-0.2643562	3.07767187	-0.1023932	5.61468943	0.89613799	-6.9077553	18	56	5.36446667	305.777778	32.7777778	0	0	1	0	18.9135952	0	-0.0428739
1.0926	30600.1548	0.86672712	235.362924	2.60681626	0	0.08856018	3.42103775	-0.141878	5.46113293	0.95851319	-6.9077553	19	55	5.81150556	306.333333	33.33333333	0	0	1	0	18.9135952	0	-0.0428739
1.3896	42357.4474	0.88170194	230.2616	1.87357437	0	0.32901594	3.74616787	-0.1247677	5.4392204	0.62838163	-6.9077553	20	59	3.12927222	305.777778	32.7777778	0	0	1	0	18.9135952	0	-0.0428739
1.6326	50845.8596	0.77936435	174.397164	1.54937428	0	0.49017384	3.92881836	-0.2479943	5.16134099	0.43849637	-6.9077553	21	59	3.12927222	305.222222	32.2222222	0	0	1	0	18.9135952	0	-0.0428739
1.8567	60003.5915	0.80307319	186.872176	1.44611723	0	0.61880072	4.09442108	-0.218065	5.23043018	0.36957346	-6.9077553	22	65	1.78815556	304.666667	31.66666666	0	0	1	0	18.9135952	0	-0.0428739
2.0592	69506.9073	0.87518161	202.953264	1.50501044	0	0.72231756	4.24144052	-0.1321819	5.31298065	0.40946406	-6.9077553	23	70	1.34111667	304.111111	31.11111111	0	0	1	0	18.9135952	0	-0.0428739
0.0063	223.988983	0.90985998	223.061788	1.43508021	0	-5.0672056	-1.4917038	-0.0933661	5.40745329	0.36191733	-6.9077553	0	88	1.78815556	299.111111	26.11111111	0	0	0	0	30.519848	0	0.21141735
0.0126	487.171654	0.49055658	59.7713623	0.7068822	0	-4.3740585	-0.7170882	-0.7101782	4.09054339	-0.3454776	-6.9077553	1	89	2.68223333	299.666667	26.66666666	0	0	0	0	30.519848	0	0.21141735
0.0225	925.809529	0.5815698	83.1426835	0.889444	0	-3.79424	-0.0760072	-0.5403063	4.42057024	-0.1160351	-6.9077553	2	86	4.02335	300.222222	27.2222222	0	0	0	0	30.519848	0	0.21141735
0.0342	1188.9922	0.60084145	87.2807242	0.94484278	0	-3.3755296	0.17394675	-0.5077612	4.4691411	-0.0556789	-6.9077553	3	87	3.57631111	300.222222	27.2222222	0	0	0	0	30.519848	0	0.21141735
0.0369	1276.7198	0.41765348	48.7250671	0.71683383	0	-3.2995437	0.24507708	-0.8707117	3.88621415	-0.3315172	-6.9077553	4	87	3.57631111	300.222222	27.2222222	0	0	0	0	30.519848	0	0.21141735
0.0396	1320.58361	0.4115266	41.3048223	0.87628619	0	-3.2289262	0.27883072	-0.8854546	3.72100347	-0.130922	-6.9077553	5	87	4.02335	300.222222	27.2222222	0	0	0	0	30.519848	0	0.21141735
0.0405	1320.58361	0.5918023	83.041153	0.91619909	0	-3.2064533	0.27883072	-0.5228943	4.41934835	-0.0864307	-6.9077553	6	88	2.68223333	300.222222	27.2222222	0	0	0	0	30.519848	0	0.21141735
0.0684	1627.62991	0.29942992	27.9563126	1.1472942	0	-2.6823825	0.48773912	-1.2025407	3.3306788	0.13827754	-6.9077553	7	84	3.57631111	300.777778	27.7777778	0	0	0	0	30.519848	0	0.21141735
0.1395	1797.17544	0.31901654	27.1035589	1.25378756	0	-1.9696907	0.58677251	-1.1393826	3.29970194	0.22696628	-6.9077553	8	78	3.57631111	301.888889	28.88888888	0	0	0	0	30.519848	0	0.21141735
0.2214	2567.83247	0.48743665	66.0786616	1.50593852	0	-1.5077843	0.9434515	-0.7165455	4.19086101	0.41008012	-6.9077553	9	76	4.02335	301.888889	28.88888888	0	0	0	0	30.519848	0	0.21141735
0.3024	3362.38116	0.581982	94.476747	1.59594811	0	-1.1960046	1.21294677	-0.539599	4.54836432	0.46809438	-6.9077553	10	66	4.02335	304.111111	31.11111111	0	0	0	0	30.519848	0	0.21141735
0.4266	4777.62	0.67793418	129.804065	2.03158923	0	-0.8519085	1.5641518	-0.3872311	4.86603382	0.70931046	-6.9077553	11	63	3.57631111	304.666667	31.66666666	0	0	0	1	30.519848	0	0.21141735
0.6867	11831.2508	0.85177876	220.221345	3.07491865	0	-0.3758578	2.47082892	-0.1592551	5.3946377	1.1236036	-6.9077553	12	57	5.81150556	305.777778	32.7777778	0	0	0	1	30.519848	0	0.21141735
0.9801	20787.3932	0.75631957	160.951675	2.41766713	0	-0.0201007	3.03439481	-0.27797	5.08111038	0.88321662	-6.9077553	13	56	5.36446667	305.777778	32.7777778	0	0	0	1	30.519848	0	0.21141735
1.2708	29397.4917	0.71335576	140.014561	1.81588887	0	0.23964662	3.38094337	-0.3363742	4.94175356	0.59712563	-6.9077553	14	55	5.81150556	306.333333	33.33333333	0	0	0	1	30.519848	0	0.21141735
1.4904	38815.8464	0.70162251	141.666768	1.38534421	0	0.39904454	3.65885434	-0.3529355	4.95348465	0.32667022	-6.9077553	15	59	3.12927222	305.777778	32.7777778	0	0	0	1	30.519848	0	0.21141735
1.6605	47072.0298	0.68316554	133.154514	1.21910168	0	0.50711876	3.85170022	-0.3795554	4.89151772	0.1989342	-6.9077553	16	59	3.12927222	305.222222	32.2222222	0	0	0	1	30.519848	0	0.21141735
1.7847	55713.4227	0.67197816	126.493603	0.92401444	0	0.57925033	4.02023905	-0.3960424	4.84019964	-0.0779459	-6.9077553	17	65	1.78815556	304.666667	31.66666666	0	0	0	1	30.519848	0	0.21141735
1.8549	62143.1739	0.72750977	141.130722	0.96192815	0	0.61783079	4.12945707	-0.3167542	4.94969365	-0.0377765	-6.9077553	18	70	1.34111667	304.111111	31.11111111	0	0	0	1	30.519848	0	0.21141735
1.917	69218.2003	0.63068329	104.29822	0.85983005	0	0.65076146	4.23727828	-0.4593671	4.64726388	-0.1498582	-6.9077553	19	73	1.78815556	303	30	0	0	0	1	30.519848	0	0.21141735
1.9458	70577.9799	0.52148771	66.455246	0.81245863	0	0.6656732	4.25673236	-0.6491538	4.19654378	-0.2064602	-6.9077553	20	76	2.68223333	301.888889	28.88888888	0	0	0	1	30.519848	0	0.21141735
1.9593	71235.9427	0.29367154	19.7026917	0.43159812	0	0.67258727	4.26601154	-1.221894	2.98080601	-0.8379461	-6.9077553	21	80	2.23519444	301.333333	28.33333333	0	0	0	1	30.519848	0	0.21141735
1.9719	71850.0334	0.24445137	12.7800293	0.32681254	0	0.67899754	4.27459499	-1.4046564	2.54796199	-1.1153134	-6.9077553	22	81	2.68223333	301.333333	28.33333333	0	0	0	1	30.519848	0	0.21141735
1.9719	71850.0334	0	0	0	0	0.67899754	4.27459499	-6.9077553	-6.9077553	-6.9077553	-6.9077553	23	83	2.68223333	300.777778	27.7777778	0	0	0	1	30.519848	0	0.21141735
0.0144	660.764682	0.97218793	262.398503	1.82137655	0	-4.2405271	-0.4128453	-0.0271781	5.56986816	0.60014144	-6.9077553	0	88	1.78815556	299.111111	26.11111111	0	0	0	0	34.0724638	0	0.49277013
0.0288	1143.26625	0.48737651	61.1322021	0.92989755	0	-3.5473799	0.13476361	-0.7166686	4.11305513	-0.0716061	-6.9077553	1	89	2.68223333	299.666667	26.66666666	0	0	0	0	34.0724638	0	0.49277013
0.0405	1625.76781	0.52784711	67.1028114	0.67133955	0	-3.2064533	0.48659511	-0.6370559	4.20624084	-0.3969918	-6.9077553	2	86	4.02335	300.222222	27.2222222	0	0	0	0	34.0724638	0	0.49277013
0.0567	2239.8607	0.60547521	94.6070557	1.08739154	0	-2.8699811	0.80686003	-0.5000914	4.54974263	0.08470095	-6.9077553	3	87	3.57631111	300.222222	27.2222222	0	0	0	0	34.0724638	0	0.49277013
0.0828	3292.5917	0.60851176	92.1654179	0.9529467	0	-2.4913272	1.19197867	-0.495097	4.52359583	-0.0471475	-6.9077553	4	87	3.57631111	300.222222	27.2222222	0	0	0	0	34.0724638	0	0.49277013
0.1197	5047.32246	0.67862799	116.059618	1.14697852	0	-2.1227667	1.619056	-0.3862097	4.75411262	0.13800259	-6.9077553	5	87	4.02335	300.222222	27.2222222	0	0	0	0	34.0724638	0	0.49277013
0.162	6977.32848	0.62631323	98.0862907	0.94910114	0	-1.8201589	1.94280941	-0.4663093	4.5858578	-0.0511868	-6.9077553	6	88	2.68223333	300.222222	27.2222222	0	0	0	0	34.0724638	0	0.49277013
0.2061	9051.07801	0.69068644	120.399045	1.03988258	0	-1.5793938	2.20299435	-0.3686225	4.79081991	0.04006899	-6.9077553	7	84	3.57631111	300.777778	27.7777778	0	0	0	0	34.0724638	0	0.49277013
0.3114	13392.0581	0.8854183	220.083951	1.9440498	0	-1.166677	2.59473652	-0.1205663	5.39401361	0.66528758	-6.9077553	8	78	3.57631111	301.888889	28.88888888	0	0	0	0	34.0724638	0	0.49277013
0.504	21329.055	1.04411715	328.426913	2.86624916	0	-0.685179	3.06011711	0.04412899	5.79431737	1.05335309	-6.9077553	9	76	4.02335	301.888889	28.88888888	0	0	0	1	34.0724638	0	0.49277013
0.8712	34044.7612	1.18416341	395.352022	3.2307458	0	-0.1378837	3.52770554	0.16988067	5.97977909	1.17302249	-6.9077553	10	66	4.02335	304.111111	31.11111111	0	0	0	1	34.0724638	0	0.49277013
1.3851	44996.1833	1.18581004	412.929674	3.22167413	0	0.32577234	3.80659989	0.17126907	6.02327972	1.17021149	-6.9077553	11	63	3.57631111	304.666667	31.66666666	0	0	0	1	34.0724638	0	0.49277013

2.0664	62197.9295	1.23357687	463.040621	3.92869642	0	0.72580796	4.13033779	0.21072829	6.13781694	1.36856218	-6.9077553	12	57	5.81150556	305.777778	32.7777778	0	0	0	1	34.0724638	0	0.49277013
2.6271	80496.8971	1.04765523	325.451244	2.46866829	1	0.96588058	4.38823106	0.04750861	5.78521574	0.90408385	0.0009995	13	56	5.36446667	305.777778	32.7777778	0	0	0	1	34.0724638	0	0.49277013
3.2994	106526.067	1.04165841	315.298988	2.76363004	1	1.19374063	4.6683991	0.04177362	5.75352453	1.01690683	0.0009995	14	55	5.81150556	306.333333	33.33333333	0	0	0	1	34.0724638	0	0.49277013
3.9402	128442.863	0.9262049	235.899359	2.32657238	1	1.37123148	4.85549195	-0.0755807	5.46340951	0.84482583	0.0009995	15	59	3.12927222	305.777778	32.7777778	0	0	0	1	34.0724638	0	0.49277013
4.4766	143669.096	0.83772467	198.272612	1.98214496	1	1.49886383	4.96751967	-0.1758728	5.28964795	0.68468395	0.0009995	16	59	3.12927222	305.222222	32.2222222	0	0	0	1	34.0724638	0	0.49277013
4.8096	152723.015	0.73743995	153.81136	1.42601351	1	1.57061392	5.02863247	-0.3032155	5.03573342	0.35558381	0.0009995	17	65	1.78815556	304.666667	31.66666666	0	0	0	1	34.0724638	0	0.49277013
5.0121	160012.608	0.82623942	180.698194	1.35768717	1	1.61185499	5.07525886	-0.1896611	5.19683374	0.30651892	0.0009995	18	70	1.34111667	304.111111	31.11111111	0	0	0	1	34.0724638	0	0.49277013
5.1714	165877.086	0.69145194	127.086379	1.20985542	1	1.64314344	5.1112531	-0.3675164	4.84487487	0.19132707	0.0009995	19	73	1.78815556	303	30	0	0	0	1	34.0724638	0	0.49277013
5.2614	168773.767	0.49147728	68.7748291	0.95491676	1	1.66039715	5.12856509	-0.708307	4.23085236	-0.0450844	0.0009995	20	76	2.68223333	301.888889	28.88888888	0	0	0	1	34.0724638	0	0.49277013
5.3172	170879.237	0.3189489	26.4616817	0.66476059	1	1.67094685	5.14096294	-1.139594	3.2757355	-0.4068251	0.0009995	21	80	2.23519444	301.333333	28.33333333	0	0	0	1	34.0724638	0	0.49277013
5.3496	172282.874	0.30031114	25.5063239	0.42215676	1	1.67702179	5.14914355	-1.1996119	3.23896562	-0.8600126	0.0009995	22	81	2.68223333	301.333333	28.33333333	0	0	0	1	34.0724638	0	0.49277013
5.3496	172282.874	0	0	0	1	1.67702179	5.14914355	-6.9077553	-6.9077553	-6.9077553	0.0009995	23	83	2.68223333	300.777778	27.7777778	0	0	0	1	34.0724638	0	0.49277013
0.0297	1737.50037	1.05720925	304.348328	2.0876646	0	-3.5166082	0.55302288	0.0565781	5.71817615	0.73652492	-6.9077553	0	88	1.78815556	299.111111	26.11111111	0	0	0	0	42.288408	0	-0.4111751
0.0774	4076.56599	0.72176325	136.199901	1.38296379	0	-2.5587685	1.40550024	-0.3246736	4.91413101	0.3249517	-6.9077553	1	89	2.68223333	299.666667	26.66666666	0	0	0	0	42.288408	0	-0.4111751
0.1377	6930.60626	0.69356674	124.062048	1.2896158	0	-1.9826779	1.93609157	-0.364467	4.82078989	0.25511947	-6.9077553	2	86	4.02335	300.222222	27.2222222	0	0	0	0	42.288408	0	-0.4111751
0.2052	10178.6478	0.69501078	125.203685	1.32440369	0	-1.5837702	2.32039042	-0.3623901	4.82994988	0.28171708	-6.9077553	3	87	3.57631111	300.222222	27.2222222	0	0	0	0	42.288408	0	-0.4111751
0.2745	13469.4629	0.71150321	129.169554	1.30573104	0	-1.292804	2.60049935	-0.3389709	4.86113366	0.26752863	-6.9077553	4	87	3.57631111	300.222222	27.2222222	0	0	0	0	42.288408	0	-0.4111751
0.3663	17726.4031	0.70932366	129.130764	1.3281983	0	-1.0043026	2.87511164	-0.3420346	4.86083331	0.28457598	-6.9077553	5	87	4.02335	300.222222	27.2222222	0	0	0	0	42.288408	0	-0.4111751
0.4509	21545.7884	0.66874825	118.023541	1.18500032	0	-0.7965097	3.07022678	-0.4008534	4.77089258	0.17058657	-6.9077553	6	88	2.68223333	300.222222	27.2222222	0	0	0	0	42.288408	0	-0.4111751
0.5778	27230.6696	0.68924654	134.631178	1.22832906	0	-0.5485275	3.30438062	-0.3707064	4.90254645	0.20646854	-6.9077553	7	84	3.57631111	300.777778	27.7777778	0	0	1	0	42.288408	0	-0.4111751
0.8739	36396.7068	0.75668358	165.52187	1.9679597	0	-0.1347893	3.59450577	-0.2774894	5.10910937	0.67750533	-6.9077553	8	78	3.57631111	301.888889	28.88888888	0	0	1	0	42.288408	0	-0.4111751
1.2321	49151.4869	0.84791304	195.41047	1.94213637	0	0.20872003	3.89492744	-0.1637985	5.27510744	0.66430335	-6.9077553	9	76	4.02335	301.888889	28.88888888	0	0	1	0	42.288408	0	-0.4111751
1.602	62126.3542	0.82364374	177.365154	1.5957358	0	0.47125285	4.12918638	-0.1928038	5.17821626	0.46796142	-6.9077553	10	66	4.02335	304.111111	31.11111111	0	0	1	0	42.288408	0	-0.4111751
2.0169	78060.6285	0.83312578	184.778928	1.59346892	0	0.70156168	4.35749862	-0.1813711	5.21916554	0.46654072	-6.9077553	11	63	3.57631111	304.666667	31.66666666	0	0	1	0	42.288408	0	-0.4111751
2.6739	104577.993	0.94807137	258.370826	2.53853559	0	0.98353808	4.6499427	-0.0522713	5.55439973	0.93198123	-6.9077553	12	57	5.81150556	305.777778	32.7777778	0	0	1	0	42.288408	0	-0.4111751
3.5172	139598.507	1.00133834	288.496198	2.8703371	0	1.25766522	4.93877766	0.00233561	5.66468537	1.05477781	-6.9077553	13	56	5.36446667	305.777778	32.7777778	0	0	1	0	42.288408	0	-0.4111751
4.4325	185117.896	1.06787662	341.861664	2.79897648	0	1.48896376	5.2209983	0.0666082	5.83440909	1.02961102	-6.9077553	14	55	5.81150556	306.333333	33.33333333	0	0	1	0	42.288408	0	-0.4111751
5.2083	219914.417	0.95786493	264.862311	2.12051385	0	1.65025351	5.393243	-0.0420051	5.57921389	0.75212991	-6.9077553	15	59	3.12927222	305.777778	32.7777778	0	0	1	0	42.288408	0	-0.4111751
5.7672	239065.548	0.81224883	188.79178	1.65787819	0	1.75218669	5.47674196	-0.2067181	5.24065001	0.50614159	-6.9077553	16	59	3.12927222	305.222222	32.2222222	0	0	1	0	42.288408	0	-0.4111751
6.1191	248718.72	0.68330895	139.361233	1.21113676	0	1.81141503	5.51632664	-0.3793458	4.93707654	0.19238472	-6.9077553	17	65	1.78815556	304.666667	31.66666666	0	0	1	0	42.288408	0	-0.4111751
6.3036	256669.336	0.78363097	164.341387	1.23223341	0	1.8411209	5.54779252	-0.2425418	5.10195198	0.20963951	-6.9077553	18	70	1.34111667	304.111111	31.11111111	0	0	1	0	42.288408	0	-0.4111751
6.4431	262546.642	0.6917732	128.782989	1.12423932	0	1.86300979	5.57043256	-0.3670526	4.8581365	0.11799575	-6.9077553	19	73	1.78815556	303	30	0	0	1	0	42.288408	0	-0.4111751
6.5295	266275.068	0.47667988	60.0110016	0.86132654	0	1.87633037	5.58453362	-0.7388145	4.09454457	-0.1481213	-6.9077553	20	76	2.68223333	301.888889	28.88888888	0	0	1	0	42.288408	0	-0.4111751
6.5925	268819.185	0.29458953	20.1194144	0.49324161	0	1.88593264	5.5940427	-1.2187835	3.00173494	-0.7047308	-6.9077553	21	80	2.23519444	301.333333	28.33333333	0	0	1	0	42.288408	0	-0.4111751
6.6285	270266.681	0.2960108	34.21073	0.53347912	0	1.89137853	5.59941288	-1.2139868	3.53256857	-0.6264626	-6.9077553	22	81	2.68223333	301.333333	28.33333333	0	0	1	0	42.288408	0	-0.4111751
6.6285	270266.681	0	0	0	0	1.89137853	5.59941288	-6.9077553	-6.9077553	-6.9077553	-6.9077553	23	83	2.68223333	300.777778	27.7777778	0	0	1	0	42.288408	0	-0.4111751
0.0009	0	0	0	0	0	-7.0131158	-6.9077553	-6.9077553	-6.9077553	-6.9077553	-6.9077553	0	88	1.78815556	299.111111	26.11111111	0	0	0	0	18.9135952	0	-0.0428739
0.0009	0	0	0	0	0	-7.0131158	-6.9077553	-6.9077553	-6.9077553	-6.9077553	-6.9077553	1	89	2.68223333	299.666667	26.66666666	0	0	0	0	18.9135952	0	-0.0428739
0.0009	0	0	0	0	0	-7.0131158	-6.9077553	-6.9077553	-6.9077553	-6.9077553	-6.9077553	2	86	4.02335	300.222222	27.2222222	0	0	0	0	18.9135952	0	-0.0428739
0.0009	0	0	0	0	0	-7.0131158	-6.9077553	-6.9077553	-6.9077553	-6.9077553	-6.9077553	3	87	3.57631111	300.222222	27.2222222	0	0	0	0	18.9135952	0	-0.0428739
0.0009	0	0	0	0	0	-7.0131158	-6.9077553	-6.9077553	-6.9077553	-6.9077553	-6.9077553	4	87	3.57631111	300.222222	27.2222222	0	0	0	0	18.9135952	0	-0.0428739
0.0009	0	0	0	0	0	-7.0131158	-6.9077553	-6.9077553	-6.9077553	-6.9077553	-6.9077553	5	87	4.02335	300.222222	27.2222222	0	0	0	0	18.9135952	0	-0.0428739
0.0009	0	0	0	0	0	-7.0131158	-6.9077553	-6.9077553	-6.9077553	-6.9077553	-6.9077553	6	88	2.68223333	300.222222	27.2222222	0	0	0	0	18.9135952	0	-0.0428739
0.0009	0	0	0	0	0	-7.0131158	-6.9077553	-6.9077553	-6.9077553	-6.9077553	-6.9077553	7	84	3.57631111	300.777778	27.7777778	0	0	0	0	18.9135952	0	-0.0428739
0.0117	0	0.4582967	57.3482463	1.63751539	0	-4.4481664	-6.9077553	-0.7780589	4.0491597	0.49379058	-6.9077553	8	78	3.57631111	301.888889	28.88888888	0	0	0	0	18.9135952	0	-0.0428739
0.0603	192.069487	0.64480068	119.243679	2.36815742	0	-2.8084232	-1.6447051	-0.4372644	4.78117751	0.86253437	-6.9077553	9	76	4.02335	301.888889	28.88888888	0	0	0	0	18.9135952	0	-0.0428739
0.1611	1283.57654	0.63414049	108.705619	1.87212263	0	-1.82573	0.25042913	-0.4539091	4.68865268	0.62760689	-6.9077553	10	66	4.02335	304.111111	31.11111111	0	0	1	0	18.9135952	0	-0.0428739
0.3204	5499.88931	0.74766282	186.450609	1.87965195	0	-1.1381851	1.70490977	-0.2894666	5.22817174	0.6316185	-6.9077553	11	63	3.57631111	304.666667	31.66666666	0	0	1	0	18.9135952	0	-0.0428739
0.5949	15331.089	0.87678703	260.845389	2.72081519	0	-0.519362	2.72994795	-0.1303513	5.56393169	1.00129901	-6.9077553	12	57	5.81150556	305.777778	32.7777778	0	0	1	0	18.9135952	0	-0.0428739
0.8712	24839.4696	0.93229253	293.817462	2.63385904	0	-0.1378837	3.21247416	-0.0690366	5.6829621	0.96882969	-6.9077553	13	56	5.36446667	305.777778	32.7777778	0	0	1	0	18.9135952	0	-0.0428739
1.251	37645.861	0.9186789	258.261922	2.65375826	0	0.22394323	3.62824958	-0.0837307	5.55397815	0.9763536	-6.9077553	14	55	5.81150556	306.333333	33.33333333	0	0	1	0	18.9135952	0	-0.0428739
1.5417	49449.4359	0.8826619	229.951093	1.76656075	0	0.4328857	3.90097087	-0.1236808	5.437871	0.56960049	-6.9077553	15	59	3.12927222	305.777778	32.7777778	0	0	1	0	18.9135952	0	-0.0428739
1.8351	60098.0921	0.81103516	195.109675	1.65566193	0	0.60709898	4.09599474	-0.2082116	5.27356696	0.50480469	-6.9077553	16	59	3.12927222	305.222222	32.2222222	0	0	1	0	18.9135952	0	-0.0428739
2.1186	71904.4117	0.84244394	206.08807	1.59635262	0	0.75075549	4.27535153	-0.1702618	5.32830846	0.46834764	-6.9077553	17	65	1.78815556	304.666667	31.66666666	0	0	1	0	18.9135952	0	-0.0428739
2.3634	83707.2821	0.90011081	219.586081	1.62557759	0	0.86010126	4.42733792	-0.104127	5.39174888	0.48647817	-6.9077553	18	70	1.34111667	304.111111	31.11111111	0	0	1	0	18.9135952	0	-0.0428739
2.5317	91447.3058	0.78154514	161.922251	1.40029434	0	0.92889101	4.51577385	-0.2452037	5.08712246	0.33739634	-6.9077553	19	73	1.78815556	303	30	0	0	1	0	18.9135952	0	-0.0428739
2.6226	95526.6346	0.47934103	56.9557163	0.88749287	0	0.96416619	4.55941557	-0.7332589	4.04229162	-0.1182287	-6.9077553	20	76	2.68223333	301.888889	28.88888888	0	0	1	0	18.9135952	0	-0.0428739
2.6766	97851.4246	0.33089059	25.3639262	0.5606383	0	0.98454733	4.58346047	-1.1029499	3.23336736	-0.5768972	-6.9077553	21	80	2.23519444	301.333333	28.33333333	0	0	1	0	18.9135952	0	-0.0428739
2.7243	100132.329	0.28165911	17.6226185	0.45495163	0	1.00221151	4.60650258	-1.2635137	2.86923996	-0.7853686	-6.9077553	22	81	2.68223333	301.333333	28.33333333	0	0	1	0	18.9135952	0	-0.0428739
2.7243	100132.329	0	0	0	0	1.00221151	4.60650258	-6.9077553	-6.9077553	-6.9077553	-6.9077553	23	83	2.68223333	300.777778	27.7777778	0	0	1	0	18.9135952	0	-0.0428739
0.0081	319.617505	0.84602535	192.986069	1.65757453	0	-4.8158912	-1.1375064	-0.1660247	5.26262319	0.50595852	-6.9077553	0	83	4.02335	299.666667	26.66666666	0	0	0	0	30.519848	0	0.21141735
0.0117	495.072674	0.51112497	65.5432129	0.74109179	0	-4.4481664	-0.7010328	-0.6691866	4.18272492	-0.2982823	-6.9077553	1	87	4.02335	299.111111	26.11111111	0	0	0	0	30.519848	0	0.21141735

0.0243	1021.43809	0.62018394	93.330767	1.02085229	0	-3.7172789	0.02219006	-0.476128	4.53616053	0.02161695	-6.9077553	2	89	3.57631111	298.555556	25.55555555	0	0	0	0	30.519848	0	0.21141735
0.0342	1240.75693	0.5861343	84.2380926	0.95609604	0	-3.3755296	0.21652725	-0.5325017	4.4336591	-0.0438515	-6.9077553	3	92	4.02335	298.555556	25.55555555	0	0	0	0	30.519848	0	0.21141735
0.0396	1328.48452	0.43319913	44.2984314	0.83866954	0	-3.2289262	0.28479128	-0.834252	3.79097184	-0.1747469	-6.9077553	4	88	4.91742778	298.555556	25.55555555	0	0	0	0	30.519848	0	0.21141735
0.0423	1416.21212	0.46944543	53.9144694	0.67672507	0	-3.1629682	0.34869165	-0.7540753	3.98741744	-0.3890136	-6.9077553	5	87	4.02335	298.555556	25.55555555	0	0	0	0	30.519848	0	0.21141735
0.0963	1662.32001	0.3194945	27.0559448	1.61864789	0	-2.340287	0.50881561	-1.1378902	3.29794371	0.48220878	-6.9077553	6	82	4.91742778	299.666667	26.66666666	0	0	0	0	30.519848	0	0.21141735
0.1863	1934.33622	0.43613972	52.6589258	2.04557983	0	-1.680397	0.66028107	-0.8275024	3.96385474	0.71617003	-6.9077553	7	74	5.36446667	301.333333	28.33333333	0	0	0	0	30.519848	0	0.21141735
0.2754	2906.23287	0.61019639	106.898714	1.95240229	0	-1.2895307	1.06720172	-0.4923369	4.67189114	0.66957262	-6.9077553	8	68	5.36446667	302.444444	29.44444444	0	0	0	0	30.519848	0	0.21141735
0.3744	6067.21676	0.61601139	101.610174	1.52988933	0	-0.9824305	1.80306478	-0.4828678	4.62115351	0.42584883	-6.9077553	9	63	5.81150556	303.555556	30.55555555	0	0	0	0	30.519848	0	0.21141735
0.5544	13281.3106	0.6921933	133.322939	1.82733246	0	-0.5898688	2.58643312	-0.3664464	4.8927818	0.60340433	-6.9077553	10	60	4.91742778	304.666667	31.66666666	0	0	0	1	30.519848	0	0.21141735
0.8397	23770.594	0.79154068	187.630447	2.45057327	0	-0.1747106	3.16849134	-0.2325114	5.23447965	0.89672997	-6.9077553	11	55	4.91742778	306.888889	33.88888888	0	0	0	1	30.519848	0	0.21141735
1.1592	32320.4699	0.79057325	184.104291	2.82316069	0	0.14773011	3.47573171	-0.2337329	5.21550783	1.03821122	-6.9077553	12	51	5.81150556	307.444444	34.4444444	0	0	0	1	30.519848	0	0.21141735
1.4634	41687.3121	0.71603692	139.339324	1.78049446	0	0.3807625	3.7302208	-0.332628	4.93691931	0.5774526	-6.9077553	13	49	5.36446667	308.555556	35.5555556	0	0	0	1	30.519848	0	0.21141735
1.7316	55511.5865	0.73332465	151.691511	1.35810739	0	0.54904584	4.01660978	-0.308804	5.02185552	0.30682816	-6.9077553	14	52	4.47038889	306.888889	33.88888888	0	0	0	1	30.519848	0	0.21141735
2.0331	70604.1634	0.77748204	172.497598	1.66887353	0	0.70956172	4.25710328	-0.2504094	5.15038911	0.51274789	-6.9077553	15	52	4.47038889	307.444444	34.4444444	0	0	0	1	30.519848	0	0.21141735
2.3067	84616.226	0.80603539	214.936202	1.90715368	0	0.83581793	4.43813786	-0.2143878	5.3703459	0.64613612	-6.9077553	16	54	3.57631111	306.888889	33.88888888	0	0	0	1	30.519848	0	0.21141735
2.4831	99719.9524	0.58411793	98.971301	0.91103869	0	0.90950778	4.60237581	-0.5359419	4.59484002	-0.0920729	-6.9077553	17	58	2.68223333	305.777778	32.7777778	0	0	0	1	30.519848	0	0.21141735
2.5839	113442.593	0.74923018	153.496547	1.00156573	0	0.94929989	4.73130573	-0.2873752	5.03368459	0.00256244	-6.9077553	18	65	1.78815556	304.111111	31.11111111	0	0	0	1	30.519848	0	0.21141735
2.673	126243.448	0.69414227	126.351365	0.8771387	0	0.98320144	4.83822009	-0.3636387	4.83907455	-0.1299507	-6.9077553	19	70	1.78815556	303	30	0	0	0	1	30.519848	0	0.21141735
2.7486	135407.263	0.66132795	116.214948	0.96399643	0	1.01109169	4.90829439	-0.4119944	4.75545008	-0.0356309	-6.9077553	20	77	0.44703889	301.888889	28.88888888	0	0	0	1	30.519848	0	0.21141735
2.7693	136109.081	0.51578091	65.6139606	0.84385026	0	1.01859458	4.91346398	-0.6601363	4.18380373	-0.1685959	-6.9077553	21	75	0.89407778	297.444444	24.44444444	0	0	0	1	30.519848	0	0.21141735
2.7864	136604.098	0.43518699	47.6652029	0.71602781	0	1.02475044	4.91709427	-0.8296843	3.86422261	-0.3326406	-6.9077553	22	88	0.89407778	295.222222	22.2222222	0	0	0	1	30.519848	0	0.21141735
2.7927	136779.564	0.27782682	16.8496508	0.64462464	0	1.02700887	4.91837792	-1.2771644	2.82438928	-0.437537	-6.9077553	23	46	2.23519444	296.888889	23.88888888	0	0	0	1	30.519848	0	0.21141735
0.0171	753.001661	0.98393451	276.14369	2.12426588	0	-4.0686768	-0.2823607	-0.0151801	5.62092497	0.75389692	-6.9077553	0	83	4.02335	299.666667	26.66666666	0	0	0	0	34.0724638	0	0.49277013
0.0333	1367.09468	0.54727109	73.8950467	0.89384757	0	-3.4021979	0.31341903	-0.6009854	4.30265933	-0.1111019	-6.9077553	1	87	4.02335	299.111111	26.11111111	0	0	0	0	34.0724638	0	0.49277013
0.0486	1893.45994	0.51914484	65.979801	0.79477114	0	-3.0241317	0.63893381	-0.653648	4.18936381	-0.2284436	-6.9077553	2	89	3.57631111	298.555556	25.55555555	0	0	0	0	34.0724638	0	0.49277013
0.0765	3077.78216	0.59420918	91.2660818	1.0204733	0	-2.5704645	1.12453411	-0.5188424	4.51379017	0.02124599	-6.9077553	3	92	4.02335	298.555556	25.55555555	0	0	0	0	34.0724638	0	0.49277013
0.1188	4920.06089	0.64244806	104.06196	1.07894946	0	-2.1303139	1.59352414	-0.440914	4.6449961	0.07691425	-6.9077553	4	88	4.91742778	298.555556	25.55555555	0	0	0	0	34.0724638	0	0.49277013
0.162	6810.55342	0.60063299	92.1674398	0.89527376	0	-1.8201589	1.9186202	-0.5081077	4.52361777	-0.1095094	-6.9077553	5	87	4.02335	298.555556	25.55555555	0	0	0	0	34.0724638	0	0.49277013
0.2403	10503.5183	0.85453112	208.695066	1.66510045	0	-1.4258671	2.35180548	-0.1560328	5.34087896	0.51048584	-6.9077553	6	82	4.91742778	299.666667	26.66666666	0	0	0	0	34.0724638	0	0.49277013
0.5769	19591.7635	1.17354221	424.99014	3.79928475	0	-0.5500863	2.97516029	0.16087846	6.05206832	1.335076	-6.9077553	7	74	5.36446667	301.333333	28.33333333	0	0	0	1	34.0724638	0	0.49277013
1.2618	32551.4297	1.42668162	646.630626	5.21168436	1	0.23253927	3.48285201	0.35605188	6.47177678	1.65109496	0.0009995	8	68	5.36446667	302.444444	29.44444444	0	0	0	1	34.0724638	0	0.49277013
2.0286	61284.8431	1.26981006	469.152289	4.02199902	4	0.7073459	4.11554887	0.23965454	6.15092956	1.39202765	1.38654433	9	63	5.81150556	303.555556	30.55555555	0	0	0	1	34.0724638	0	0.49277013
2.5848	78148.9688	0.98795356	280.272831	2.12921241	4	0.94964814	4.35862966	-0.0111079	5.63576709	0.7562217	1.38654433	10	60	4.91742778	304.666667	31.66666666	0	0	0	1	34.0724638	0	0.49277013
3.1698	99052.6374	0.9818611	271.856683	2.2903756	4	1.15366849	4.5956615	-0.0172875	5.6052787	0.82915234	1.38654433	11	55	4.91742778	306.888889	33.88888888	0	0	0	1	34.0724638	0	0.49277013
4.0239	127090.498	1.04295151	315.723195	3.66084314	4	1.39225158	4.84490728	0.04301305	5.75486903	1.29796661	1.38654433	12	51	5.81150556	307.444444	34.4444444	0	0	0	1	34.0724638	0	0.49277013
4.7601	146686.516	0.91926467	247.564849	2.75765073	4	1.56026868	4.98830459	-0.083094	5.51167661	1.0147417	1.38654433	13	49	5.36446667	308.555556	35.5555556	0	0	0	1	34.0724638	0	0.49277013
5.4297	166767.999	0.93308767	248.717112	2.31936219	4	1.69188388	5.11660961	-0.068185	5.51632017	0.84172329	1.38654433	14	52	4.47038889	306.888889	33.88888888	0	0	0	1	34.0724638	0	0.49277013
6.1146	183241.411	0.84902469	209.235299	2.64537467	4	1.81067935	5.21080993	-0.1624899	5.34346423	0.97319066	1.38654433	15	52	4.47038889	307.444444	34.4444444	0	0	0	1	34.0724638	0	0.49277013
6.5916	196936.702	0.70080152	142.25513	1.40784855	4	1.88579611	5.28288745	-0.3541047	4.95762916	0.34277274	1.38654433	16	54	3.57631111	306.888889	33.88888888	0	0	0	1	34.0724638	0	0.49277013
6.9552	208439.344	0.70903363	148.528707	1.19346014	4	1.93948958	5.33965288	-0.3424429	5.00078499	0.17769432	1.38654433	17	58	2.68223333	305.777778	32.7777778	0	0	0	1	34.0724638	0	0.49277013
7.173	216962.028	0.81408118	185.354468	1.37059967	4	1.97032398	5.37972696	-0.2044676	5.22227543	0.3159777	1.38654433	18	65	1.78815556	304.111111	31.11111111	0	0	0	1	34.0724638	0	0.49277013
7.3521	224489.077	0.7741325	160.058123	1.29821287	4	1.99498599	5.41383151	-0.2547213	5.07554326	0.26175859	1.38654433	19	70	1.78815556	303	30	0	0	0	1	34.0724638	0	0.49277013
7.5168	231617.565	0.74283904	145.893805	1.27872013	4	2.01714052	5.4450919	-0.2959306	4.98288585	0.2466414	1.38654433	20	77	0.44703889	301.888889	28.88888888	0	0	0	1	34.0724638	0	0.49277013
7.5816	234468.692	0.38783895	34.5042521	0.55216471	4	2.02572426	5.45732633	-0.94459	3.54111155	-0.5920995	1.38654433	21	75	0.89407778	297.444444	24.44444444	0	0	0	1	34.0724638	0	0.49277013
7.6338	236398.709	0.37884558	34.4814579	0.55022651	4	2.03258576	5.46552406	-0.9679905	3.54045073	-0.5956095	1.38654433	22	88	0.89407778	295.222222	22.2222222	0	0	0	1	34.0724638	0	0.49277013
7.7094	239075.711	0.30388957	33.0107102	0.56156576	4	2.04244036	5.47678447	-1.1878056	3.49686235	-0.5752472	1.38654433	23	46	2.23519444	296.888889	23.88888888	0	0	0	1	34.0724638	0	0.49277013
0.0369	2382.4081	1.13261671	355.117017	2.46964149	0	-3.2995437	0.86853144	0.12541315	5.87245018	0.90447783	-6.9077553	0	83	4.02335	299.666667	26.66666666	0	0	0	0	42.288408	0	-0.4111751
0.0864	4807.00201	0.73494915	141.797283	1.43478324	0	-2.4487676	1.57028162	-0.3065943	4.95440551	0.36171051	-6.9077553	1	87	4.02335	299.111111	26.11111111	0	0	0	0	42.288408	0	-0.4111751
0.1467	7658.30425	0.66850195	115.818651	1.27855557	0	-1.9193656	2.03592115	-0.4012212	4.75203425	0.24651281	-6.9077553	2	89	3.57631111	298.555556	25.55555555	0	0	0	0	42.288408	0	-0.4111751
0.2079	10597.3305	0.67858073	118.425781	1.24987557	0	-1.5706981	2.36069649	-0.3862792	4.77429489	0.22384376	-6.9077553	3	92	4.02335	298.555556	25.55555555	0	0	0	0	42.288408	0	-0.4111751
0.2826	14110.0037	0.69592152	123.968868	1.31954221	0	-1.2637228	2.6469549	-0.3610825	4.82003853	0.27804242	-6.9077553	4	88	4.91742778	298.555556	25.55555555	0	0	0	0	42.288408	0	-0.4111751
0.3816	18617.2029	0.74744394	146.879492	1.34464541	0	-0.9633823	2.92413975	-0.289759	4.98961928	0.29687376	-6.9077553	5	87	4.02335	298.555556	25.55555555	0	0	0	0	42.288408	0	-0.4111751
0.5139	23627.7067	0.67551007	130.091544	1.5423993	0	-0.6657266	3.16246236	-0.3908079	4.86824608	0.43398732	-6.9077553	6	82	4.91742778	299.666667	26.66666666	0	0	0	0	42.288408	0	-0.4111751
0.8325	34322.4422	0.85604014	209.156118	2.54071045	0	-0.1833221	3.53582857	-0.1542705	5.34308573	0.93283726	-6.9077553	7	74	5.36446667	301.333333	28.33333333	0	0	1	0	42.288408	0	-0.4111751
1.2069	48117.1395	0.86343384	200.989671	2.03900293	0	0.18805509	3.87365923	-0.1456805	5.30325849	0.71295125	-6.9077553	8	68	5.36446667	302.444444	29.44444444	0	0	1	0	42.288408	0	-0.4111751
1.629	65939.0609	0.89825888	218.966501	1.85931383	0	0.48796633	4.18874616	-0.1061843	5.38892332	0.6207452	-6.9077553	9	63	5.81150556	303.555556	30.55555555	0	0	1	0	42.288408	0	-0.4111751
2.2311	86862.9617	0.90857663	235.625677	2.5060059	0	0.80249474	4.46434324	-0.094776	5.46224868	0.91908917	-6.9077553	10	60	4.91742778	304.666667	31.66666666	0	0	1	0	42.288408	0	-0.4111751
2.9412	117269.631	0.97618283	270.736771	2.40661281	0	1.07881766	4.76448435	-0.0230815	5.60115072	0.87863572	-6.9077553	11	55	4.91742778	306.888889	33.88888888	0	0	1	0	42.288408	0	-0.4111751
3.7476	157355.086	1.05835451	323.539446	2.6867169	0	1.32111564	5.0585113	0.05765977	5.77932413	0.9886921	-6.9077553	12	51	5.81150556	307.444444	34.4444444	0	0	1	0	42.288408	0	-0.4111751
4.635	201057.536	1.08766283	356.090834	2.93860358	0	1.5336362	5.30359609	0.08495018	5.87518866	1.07827474	-6.9077553	13	49	5.36446667	308.555556	35.5555556	0	0	1	0	42.288408	0	-0.4111751
5.2821	225733.51	0.8881111	225.598596	2.04909807	0	1.66432375	5.41935957	-0.1175331	5.41876173	0.71788763	-6.9077553	14	52	4.47038889	306.888889	33.88888888	0	0	1	0	42.288408	0	-0.4111751
5.8473	244486.98	0.79464722	180.389792	1.76845167	0	1.76598002	5.49916615	-0.2285994	5.19512556	0.57066971	-6.9077553	15	52	4.47038889	307.444444	34.4444444	0	0	1	0	42.288408	0	-0.4111751

6.3027	257386.464	0.7320702	160.144608	1.50729141	0	1.84097811	5.55058259	-0.3105138	5.07608345	0.41097749	-6.9077553	16	54	3.57631111	306.888889	33.88888888	0	0	1	0	42.288408	0	-0.4111751
6.6276	268111.725	0.7106614	154.30261	1.19554988	0	1.89124275	5.59140751	-0.340153	5.03892215	0.17944231	-6.9077553	17	58	2.68223333	305.777778	32.7777778	0	0	1	0	42.288408	0	-0.4111751
6.768	274242.177	0.80538598	170.305777	1.19367746	0	1.91220562	5.61401522	-0.2151928	5.13760138	0.17787624	-6.9077553	18	65	1.78815556	304.111111	31.11111111	0	0	1	0	42.288408	0	-0.4111751
6.8877	279420.675	0.77233302	155.262189	1.18449046	0	1.92973721	5.63272202	-0.2570455	5.04512167	0.17015658	-6.9077553	19	70	1.78815556	303	30	0	0	1	0	42.288408	0	-0.4111751
7.0002	283771.48	0.69057294	122.96825	1.0748949	0	1.94593872	5.64817279	-0.3687867	4.81193432	0.07315278	-6.9077553	20	77	0.44703889	301.888889	28.88888888	0	0	1	0	42.288408	0	-0.4111751
7.0596	285789.199	0.41907484	46.0999867	0.69965634	0	1.95438839	5.65525797	-0.8673224	3.83083435	-0.3557378	-6.9077553	21	75	0.89407778	297.444444	24.44444444	0	0	1	0	42.288408	0	-0.4111751
7.1046	287368.328	0.42218895	46.5718262	0.6372863	0	1.96074246	5.66076825	-0.8599365	3.84101724	-0.4489683	-6.9077553	22	88	0.89407778	295.222222	22.2222222	0	0	1	0	42.288408	0	-0.4111751
7.1658	289538.712	0.62565461	114.560971	0.95286818	0	1.96931971	5.66829246	-0.4673597	4.7411159	-0.0472298	-6.9077553	23	46	2.23519444	296.888889	23.88888888	0	0	1	0	42.288408	0	-0.4111751
0.0009	0	0	0	0	0	-7.0131158	-6.9077553	-6.9077553	-6.9077553	-6.9077553	-6.9077553	0	83	4.02335	299.666667	26.66666666	0	0	0	0	18.9135952	0	-0.0428739
0.0009	0	0	0	0	0	-7.0131158	-6.9077553	-6.9077553	-6.9077553	-6.9077553	-6.9077553	1	87	4.02335	299.111111	26.11111111	0	0	0	0	18.9135952	0	-0.0428739
0.0009	0	0	0	0	0	-7.0131158	-6.9077553	-6.9077553	-6.9077553	-6.9077553	-6.9077553	2	89	3.57631111	298.555556	25.55555555	0	0	0	0	18.9135952	0	-0.0428739
0.0009	0	0	0	0	0	-7.0131158	-6.9077553	-6.9077553	-6.9077553	-6.9077553	-6.9077553	3	92	4.02335	298.555556	25.55555555	0	0	0	0	18.9135952	0	-0.0428739
0.0009	0	0	0	0	0	-7.0131158	-6.9077553	-6.9077553	-6.9077553	-6.9077553	-6.9077553	4	88	4.91742778	298.555556	25.55555555	0	0	0	0	18.9135952	0	-0.0428739
0.0009	0	0	0	0	0	-7.0131158	-6.9077553	-6.9077553	-6.9077553	-6.9077553	-6.9077553	5	87	4.02335	298.555556	25.55555555	0	0	0	0	18.9135952	0	-0.0428739
0.0009	0	0	0	0	0	-7.0131158	-6.9077553	-6.9077553	-6.9077553	-6.9077553	-6.9077553	6	82	4.91742778	299.666667	26.66666666	0	0	0	0	18.9135952	0	-0.0428739
0.0468	66.7588747	0.6807346	129.287885	3.08944612	0	-3.0618721	-2.6917998	-0.3831149	4.86204932	1.12831546	-6.9077553	7	74	5.36446667	301.333333	28.33333333	0	0	0	0	18.9135952	0	-0.0428739
0.2052	3817.08267	0.82971504	226.679177	2.67219439	0	-1.5837702	1.33974838	-0.1854685	5.42354011	0.98327416	-6.9077553	8	68	5.36446667	302.444444	29.44444444	0	0	0	0	18.9135952	0	-0.0428739
0.4752	12310.4775	0.87365896	251.595521	2.86012065	0	-0.7440195	2.51053196	-0.1339212	5.5278267	1.05121338	-6.9077553	9	63	5.81150556	303.555556	30.55555555	0	0	1	0	18.9135952	0	-0.0428739
0.6624	16366.5957	0.76565647	192.799429	2.02743765	0	-0.4118857	2.79530351	-0.2657165	5.26165561	0.70726587	-6.9077553	10	60	4.91742778	304.666667	31.66666666	0	0	1	0	18.9135952	0	-0.0428739
0.8793	23439.6287	0.79842532	195.670967	1.80853639	0	-0.1286291	3.15447078	-0.2238622	5.27643962	0.59307067	-6.9077553	11	55	4.91742778	306.888889	33.88888888	0	0	1	0	18.9135952	0	-0.0428739
1.224	35396.5085	0.90666362	252.959285	2.65228718	0	0.20212418	3.56664144	-0.0968814	5.5332325	0.97579932	-6.9077553	12	51	5.81150556	307.444444	34.4444444	0	0	1	0	18.9135952	0	-0.0428739
1.6002	51307.1357	0.94222278	266.703574	2.19899753	0	0.47012862	3.93784933	-0.0584528	5.58614158	0.78845624	-6.9077553	13	49	5.36446667	308.555556	35.5555556	0	0	1	0	18.9135952	0	-0.0428739
1.9026	61828.3902	0.82426607	197.380673	1.69424965	0	0.64322137	4.12437882	-0.1920494	5.28513928	0.52783002	-6.9077553	14	52	4.47038889	306.888889	33.88888888	0	0	1	0	18.9135952	0	-0.0428739
2.2572	75251.6772	0.85465453	216.690256	1.94739141	0	0.81412511	4.32085148	-0.1558886	5.37847356	0.66700412	-6.9077553	15	52	4.47038889	307.444444	34.4444444	0	0	1	0	18.9135952	0	-0.0428739
2.6253	89931.8609	0.85864153	216.1603	1.68943581	0	0.96519518	4.4990634	-0.1512398	5.37602489	0.52498637	-6.9077553	16	54	3.57631111	306.888889	33.88888888	0	0	1	0	18.9135952	0	-0.0428739
2.8908	98565.0027	0.73849218	160.573014	1.42821345	0	1.06153328	4.5907264	-0.3017916	5.07875498	0.35712426	-6.9077553	17	58	2.68223333	305.777778	32.7777778	0	0	1	0	18.9135952	0	-0.0428739
3.0762	107711.887	0.90124927	227.336468	1.63008799	0	1.12369507	4.67946923	-0.1028644	5.42643556	0.48924727	-6.9077553	18	65	1.78815556	304.111111	31.11111111	0	0	1	0	18.9135952	0	-0.0428739
3.2472	115743.054	0.80914034	176.242023	1.40913343	0	1.17779309	4.75138132	-0.2105478	5.17186386	0.34368433	-6.9077553	19	70	1.78815556	303	30	0	0	1	0	18.9135952	0	-0.0428739
3.4011	122750.801	0.76986659	156.458767	1.3706011	0	1.22409891	4.81016444	-0.26024	5.0527989	0.31597874	-6.9077553	20	77	0.44703889	301.888889	28.88888888	0	0	1	0	18.9135952	0	-0.0428739
3.51	127356.498	0.47618979	55.7401375	0.90081081	0	1.25561604	4.84699807	-0.739841	4.02071843	-0.1033505	-6.9077553	21	75	0.89407778	297.444444	24.44444444	0	0	1	0	18.9135952	0	-0.0428739
3.5892	130865.606	0.46009168	53.1105402	0.8213136	0	1.27792934	4.87417853	-0.7741584	3.97239423	-0.1956335	-6.9077553	22	88	0.89407778	295.222222	22.2222222	0	0	1	0	18.9135952	0	-0.0428739
3.6513	133577.805	0.49916873	85.5192128	0.82071006	0	1.29508327	4.8946916	-0.6928098	4.44875276	-0.1963677	-6.9077553	23	46	2.23519444	296.888889	23.88888888	0	0	1	0	18.9135952	0	-0.0428739
0.0153	408.859309	0.87743521	210.260681	1.65558851	0	-4.1799025	-0.8919413	-0.1296131	5.34835286	0.50476037	-6.9077553	0	62	1.34111667	304.111111	31.11111111	0	0	0	0	30.519848	0	0.21141735
0.0315	1123.37512	0.80002064	169.589749	1.31635793	0	-3.4577677	0.11722743	-0.2218686	5.13338817	0.27562816	-6.9077553	1	71	1.78815556	301.333333	28.33333333	0	0	0	0	30.519848	0	0.21141735
0.0414	1342.69396	0.67721549	120.722257	1.45931348	0	-3.1844744	0.29542251	-0.3882902	4.79350079	0.37865112	-6.9077553	2	75	1.78815556	300.222222	27.2222222	0	0	0	0	30.519848	0	0.21141735
0.0459	1430.42156	0.33119791	28.7907257	1.04837027	0	-3.0812902	0.35866805	-1.1020244	3.36008804	0.04819024	-6.9077553	3	82	1.34111667	299.111111	26.11111111	0	0	0	0	30.519848	0	0.21141735
0.0477	1474.28527	0.22720048	12.9556694	0.76003766	0	-3.0428239	0.38885138	-1.4775307	2.56161067	-0.2730724	-6.9077553	4	89	1.34111667	298	25	0	0	0	0	30.519848	0	0.21141735
0.0486	1474.28527	0.14451735	3.87525773	0.67661148	0	-3.0241317	0.38885138	-1.92746	1.35487019	-0.3891812	-6.9077553	5	90	1.34111667	298	25	0	0	0	0	30.519848	0	0.21141735
0.0486	1474.28527	0	0	0	0	-3.0241317	0.38885138	-6.9077553	-6.9077553	-6.9077553	-6.9077553	6	91	1.34111667	297.444444	24.44444444	0	0	0	0	30.519848	0	0.21141735
0.0486	1474.28527	0	0	0	0	-3.0241317	0.38885138	-6.9077553	-6.9077553	-6.9077553	-6.9077553	7	93	1.34111667	297.444444	24.44444444	0	0	0	0	30.519848	0	0.21141735
0.0486	1474.28527	0	0	0	0	-3.0241317	0.38885138	-6.9077553	-6.9077553	-6.9077553	-6.9077553	8	93	1.78815556	297.444444	24.44444444	0	0	0	0	30.519848	0	0.21141735
0.0486	1474.28527	0	0	0	0	-3.0241317	0.38885138	-6.9077553	-6.9077553	-6.9077553	-6.9077553	9	93	2.23519444	297.444444	24.44444444	0	0	0	0	30.519848	0	0.21141735
0.0486	1474.28527	0	0	0	0	-3.0241317	0.38885138	-6.9077553	-6.9077553	-6.9077553	-6.9077553	10	94	1.34111667	297.444444	24.44444444	0	0	0	0	30.519848	0	0.21141735
0.0486	1474.28527	0	0	0	0	-3.0241317	0.38885138	-6.9077553	-6.9077553	-6.9077553	-6.9077553	11	92	1.34111667	298	25	0	0	0	0	30.519848	0	0.21141735
0.0486	1474.28527	0	0	0	0	-3.0241317	0.38885138	-6.9077553	-6.9077553	-6.9077553	-6.9077553	12	88	2.68223333	299.111111	26.11111111	0	0	0	0	30.519848	0	0.21141735
0.0774	1662.79947	0.3042936	27.6921997	1.14062512	0	-2.5587685	0.50910382	-1.1864813	3.32118689	0.13245279	-6.9077553	13	83	3.57631111	300.222222	27.2222222	0	0	0	0	30.519848	0	0.21141735
0.1719	2136.45077	0.48788765	69.9118908	1.58237668	0	-1.7608424	0.75961389	-0.7156226	4.24725005	0.45955971	-6.9077553	14	73	3.57631111	302.444444	29.44444444	0	0	0	0	30.519848	0	0.21141735
0.3294	3343.87074	0.55387242	85.3205804	1.82605905	0	-1.1104825	1.20742805	-0.5890171	4.44642742	0.6027076	-6.9077553	15	63	3.12927222	304.111111	31.11111111	0	0	0	0	30.519848	0	0.21141735
0.5004	4632.06733	0.54768343	82.6610095	1.60696636	0	-0.6923475	1.53321914	-0.6002336	4.41476012	0.47497025	-6.9077553	16	55	2.68223333	306.333333	33.33333333	0	0	0	0	30.519848	0	0.21141735
0.6696	6233.01022	0.58912691	94.8173724	1.6065057	0	-0.4010748	1.83001982	-0.5274177	4.55196319	0.47468372	-6.9077553	17	50	3.12927222	307.444444	34.4444444	0	0	0	1	30.519848	0	0.21141735
0.945	8889.75665	0.75258612	162.897799	2.32351186	0	-0.0565704	2.18501216	-0.282912	5.09312914	0.84351006	-6.9077553	18	47	3.57631111	307.444444	34.4444444	0	0	0	1	30.519848	0	0.21141735
1.2915	13823.3187	0.78709978	168.205276	1.87876405	0	0.25580433	2.62642927	-0.2381306	5.12519106	0.63114626	-6.9077553	19	44	2.23519444	308.555556	35.5555556	0	0	0	1	30.519848	0	0.21141735
1.629	19738.8552	0.8309786	196.403083	1.96655111	0	0.48796633	2.9826397	-0.1839486	5.28017419	0.67678968	-6.9077553	20	43	2.23519444	309.111111	36.11111111	0	0	0	1	30.519848	0	0.21141735
1.9971	24819.9957	0.84037441	200.472303	3.53659453	0	0.69169613	3.2116899	-0.1727185	5.30068109	1.26344699	-6.9077553	21	88	5.81150556	296.333333	23.33333333	0	0	0	1	30.519848	0	0.21141735
2.0484	26691.544	0.51251823	106.545145	1.10131461	0	0.717059	3.28438427	-0.6664697	4.66857817	0.09741216	-6.9077553	22	90	2.23519444	296.888889	23.88888888	0	0	0	1	30.519848	0	0.21141735
2.0646	27393.3621	0.26030143	14.5876329	0.38648878	0	0.7249365	3.31033723	-1.3420806	2.68024266	-0.9480684	-6.9077553	23	91	1.34111667	296.888889	23.88888888	0	0	0	1	30.519848	0	0.21141735
0.0261	933.175439	0.97840752	268.065604	2.06247861	0	-3.64582	-0.068091	-0.0208075	5.59123547	0.7243932	-6.9077553	0	62	1.34111667	304.111111	31.11111111	0	0	0	0	34.0724638	0	0.49277013
0.0585	2312.37916	0.80884621	175.191786	1.32582442	0	-2.8387285	0.8387093	-0.2109109	5.165887	0.28278843	-6.9077553	1	71	1.78815556	301.333333	28.33333333	0	0	0	0	34.0724638	0	0.49277013
0.0927	3806.17429	0.70939059	127.577984	1.08818958	0	-2.3783868	1.33688726	-0.3419403	4.84873565	0.08543391	-6.9077553	2	75	1.78815556	300.222222	27.2222222	0	0	0	0	34.0724638	0	0.49277013
0.1233	5297.54354	0.50017301	61.2266846	0.69240531	0	-2.0931349	1.66743198	-0.6908039	4.11459945	-0.3661406	-6.9077553	3	82	1.34111667	299.111111	26.11111111	0	0	0	0	34.0724638	0	0.49277013
0.1413	6087.09163	0.35587518	28.4389771	0.44922552	0	-1.95687	1.80633467	-1.0303692	3.3477958	-0.7980067	-6.9077553	4	89	1.34111667	298	25	0	0	0	0	34.0724638	0	0.49277013
0.1602	6964.36713	0.23109622	11.4649259	0.30545403	0	-1.8313322	1.94095032	-1.4606032	2.43937967	-1.1826875	-6.9077553	5	90	1.34111667	298	25	0	0	0	0	34.0724638	0	0.49277013

0.1728	7578.45882	0.24361507	12.2876489	0.30588167	0	-1.7556204	2.0254418	-1.4080695	2.50867598	-1.181293	-6.9077553	6	91	1.34111667	297.444444	24.44444444	0	0	0	0	34.0724638	0	0.49277013
0.1863	8148.68834	0.22838998	10.9651754	0.29104023	0	-1.680397	2.09797969	-1.4723318	2.39481557	-1.2308637	-6.9077553	7	93	1.34111667	297.444444	24.44444444	0	0	0	0	34.0724638	0	0.49277013
0.1989	8587.32667	0.21092514	8.84654835	0.22961099	0	-1.6149531	2.15040392	-1.5515222	2.1801404	-1.467023	-6.9077553	8	93	1.78815556	297.444444	24.44444444	0	0	0	0	34.0724638	0	0.49277013
0.207	8938.23702	0.20045226	8.1609887	0.24745078	0	-1.5750365	2.19045024	-1.6022028	2.09948785	-1.3925105	-6.9077553	9	93	2.23519444	297.444444	24.44444444	0	0	0	0	34.0724638	0	0.49277013
0.2169	9333.01148	0.20547535	8.68052049	0.25221835	0	-1.5283189	2.23366488	-1.5775742	2.16119669	-1.3735031	-6.9077553	10	94	1.34111667	297.444444	24.44444444	0	0	0	0	34.0724638	0	0.49277013
0.2277	9771.64981	0.22420408	10.420119	0.28085204	0	-1.4797263	2.27958765	-1.4907483	2.34383442	-1.266373	-6.9077553	11	92	1.34111667	298	25	0	0	0	0	34.0724638	0	0.49277013
0.2439	10473.4707	0.37266893	41.4034932	0.57005988	0	-1.410997	2.34894093	-0.9843851	3.72338941	-0.5602612	-6.9077553	12	88	2.68223333	299.111111	26.11111111	0	0	0	0	34.0724638	0	0.49277013
0.342	13355.3285	0.70939356	149.319927	1.77758299	0	-1.0729445	2.59199032	-0.3419361	5.00609786	0.57581697	-6.9077553	13	83	3.57631111	300.222222	27.2222222	0	0	0	1	34.0724638	0	0.49277013
0.4833	20273.5492	0.92423652	248.973676	2.08088111	0	-0.7271177	3.00936636	-0.0777059	5.51735119	0.73327186	-6.9077553	14	73	3.57631111	302.444444	29.44444444	0	0	0	1	34.0724638	0	0.49277013
0.7614	34545.538	1.11086384	338.616303	2.80884658	0	-0.2725964	3.54230734	0.10603774	5.82487057	1.03312988	-6.9077553	15	63	3.12927222	304.111111	31.11111111	0	0	0	1	34.0724638	0	0.49277013
1.0746	47888.4509	1.03904408	300.804351	2.34346868	0	0.0719485	3.86889525	0.0392631	5.70646338	0.8520588	-6.9077553	16	55	2.68223333	306.333333	33.33333333	0	0	0	1	34.0724638	0	0.49277013
1.3968	56133.5353	0.93186917	241.018506	1.99933066	0	0.33418391	4.02775122	-0.0694903	5.48487787	0.6933125	-6.9077553	17	50	3.12927222	307.444444	34.4444444	0	0	0	1	34.0724638	0	0.49277013
1.8234	71640.2083	0.96634916	259.308314	2.34838713	0	0.60070289	4.27167044	-0.0331958	5.55802161	0.8541545	-6.9077553	18	47	3.57631111	307.444444	34.4444444	0	0	0	1	34.0724638	0	0.49277013
2.3553	90899.5198	0.95994772	253.233952	1.94574935	0	0.85666811	4.50976572	-0.0398353	5.53431772	0.66616098	-6.9077553	19	44	2.23519444	308.555556	35.5555556	0	0	0	1	34.0724638	0	0.49277013
3.0357	114977.122	0.98879391	273.456453	2.33353113	0	1.11044204	4.74474186	-0.0102585	5.61114604	0.84781107	-6.9077553	20	43	2.23519444	309.111111	36.11111111	0	0	0	1	34.0724638	0	0.49277013
3.5865	132482.951	0.95622413	277.780535	2.39203758	0	1.2771768	4.88646152	-0.0437177	5.62683496	0.87256351	-6.9077553	21	88	5.81150556	296.333333	23.33333333	0	0	0	1	34.0724638	0	0.49277013
3.7233	136430.687	0.56851452	118.423661	1.13384428	0	1.31461037	4.91582403	-0.562971	4.77427699	0.12649544	-6.9077553	22	90	2.23519444	296.888889	23.88888888	0	0	0	1	34.0724638	0	0.49277013
3.8088	139632.746	0.32043696	30.5851177	0.55234271	0	1.33731418	4.93902289	-1.1349538	3.42054624	-0.5917777	-6.9077553	23	91	1.34111667	296.888889	23.88888888	0	0	0	1	34.0724638	0	0.49277013
0.0315	2009.18867	1.14159606	356.008301	2.25822067	0	-3.4577677	0.69822858	0.13330292	5.87495686	0.81501992	-6.9077553	0	62	1.34111667	304.111111	31.11111111	0	0	0	0	42.288408	0	-0.4111751
0.1107	6451.15438	0.97893325	260.815496	1.8715073	0	-2.2009314	1.86441409	-0.0202708	5.56381708	0.62727833	-6.9077553	1	71	1.78815556	301.333333	28.33333333	0	0	0	0	42.288408	0	-0.4111751
0.1872	10156.1716	0.82049228	176.465659	1.53748043	0	-1.6755777	2.31818002	-0.1966327	5.17313196	0.4307952	-6.9077553	2	75	1.78815556	300.222222	27.2222222	0	0	0	0	42.288408	0	-0.4111751
0.2484	13051.181	0.59128374	87.8669649	1.11000835	0	-1.3927149	2.56895525	-0.5237695	4.47583529	0.10526803	-6.9077553	3	82	1.34111667	299.111111	26.11111111	0	0	0	0	42.288408	0	-0.4111751
0.2871	14805.732	0.4450794	47.0360051	0.83565725	0	-1.2479247	2.69508194	-0.8072583	3.85093463	-0.1783408	-6.9077553	4	89	1.34111667	298	25	0	0	0	0	42.288408	0	-0.4111751
0.3267	16648.0106	0.32987109	24.2771329	0.56743175	0	-1.118713	2.81235079	-1.1060264	3.18957606	-0.564874	-6.9077553	5	90	1.34111667	298	25	0	0	0	0	42.288408	0	-0.4111751
0.3609	18270.9716	0.3084372	20.9406015	0.55464323	0	-1.0191544	2.90536828	-1.1730001	3.04173769	-0.5876289	-6.9077553	6	91	1.34111667	297.444444	24.44444444	0	0	0	0	42.288408	0	-0.4111751
0.3915	19674.6122	0.2693112	16.1832652	0.43892238	0	-0.9377698	2.97937991	-1.3081814	2.78403949	-0.821157	-6.9077553	7	93	1.34111667	297.444444	24.44444444	0	0	0	0	42.288408	0	-0.4111751
0.4275	21253.7096	0.23926704	13.0447144	0.42433281	0	-0.849801	3.0565785	-1.4260043	2.56845968	-0.8548833	-6.9077553	8	93	1.78815556	297.444444	24.44444444	0	0	0	0	42.288408	0	-0.4111751
0.4635	22876.6698	0.25255911	13.7508667	0.41729946	0	-0.7689489	3.13016132	-1.3721583	2.62117457	-0.8715577	-6.9077553	9	93	2.23519444	297.444444	24.44444444	0	0	0	0	42.288408	0	-0.4111751
0.5004	24543.4915	0.25550179	13.8492923	0.38784778	0	-0.6923475	3.20048745	-1.3606196	2.62830634	-0.9445673	-6.9077553	10	94	1.34111667	297.444444	24.44444444	0	0	1	0	42.288408	0	-0.4111751
0.54	26473.4969	0.27797541	17.6862835	0.44392421	0	-0.6161861	3.27618189	-1.2766316	2.87284593	-0.8098513	-6.9077553	11	92	1.34111667	298	25	0	0	1	0	42.288408	0	-0.4111751
0.6237	30334.4838	0.54820617	85.3936807	0.87442451	0	-0.4720858	3.41231811	-0.5992814	4.44728381	-0.1330464	-6.9077553	12	88	2.68223333	299.111111	26.11111111	0	0	1	0	42.288408	0	-0.4111751
0.8748	37151.4406	0.63599541	121.997284	1.69543523	0	-0.13376	3.61502947	-0.4509928	4.80400698	0.52852912	-6.9077553	13	83	3.57631111	300.222222	27.2222222	0	0	1	0	42.288408	0	-0.4111751
1.2177	49160.086	0.79485299	175.321686	1.76084968	0	0.19696383	3.89510238	-0.2283408	5.1666282	0.56636421	-6.9077553	14	73	3.57631111	302.444444	29.44444444	0	0	1	0	42.288408	0	-0.4111751
1.5579	60468.5322	0.76744984	154.42821	1.46482502	0	0.44333876	4.10213964	-0.26338	5.0397358	0.38241824	-6.9077553	15	63	3.12927222	304.111111	31.11111111	0	0	1	0	42.288408	0	-0.4111751
1.9503	76081.4848	0.86730649	207.7958	1.7088861	0	0.66798321	4.33181808	-0.1412105	5.33656068	0.53642676	-6.9077553	16	55	2.68223333	306.333333	33.33333333	0	0	1	0	42.288408	0	-0.4111751
2.4588	94905.5415	0.88659663	215.926576	2.04125631	0	0.89967343	4.55289263	-0.1192379	5.37494306	0.71405523	-6.9077553	17	50	3.12927222	307.444444	34.4444444	0	0	1	0	42.288408	0	-0.4111751
3.1104	118399.876	0.88910688	221.86108	2.33169218	0	1.13475134	4.77407612	-0.1164137	5.40205593	0.84702304	-6.9077553	18	47	3.57631111	307.444444	34.4444444	0	0	1	0	42.288408	0	-0.4111751
3.8799	147417.198	0.8955687	238.820413	2.12702365	0	1.35580938	4.99327343	-0.1091804	5.47571605	0.75519369	-6.9077553	19	44	2.23519444	308.555556	35.5555556	0	0	1	0	42.288408	0	-0.4111751
4.7898	174879.428	0.84593024	216.594167	2.17153837	0	1.56648866	5.16410247	-0.1661369	5.37803002	0.77589624	-6.9077553	20	43	2.23519444	309.111111	36.11111111	0	0	1	0	42.288408	0	-0.4111751
5.3496	188699.925	0.72393082	152.86975	2.32009466	0	1.67702179	5.24016335	-0.321679	5.02959279	0.84203891	-6.9077553	21	88	5.81150556	296.333333	23.33333333	0	0	1	0	42.288408	0	-0.4111751
5.4567	193524.951	0.43654511	53.3466263	0.73074527	0	1.69684421	5.26541162	-0.8265755	3.97682948	-0.3123228	-6.9077553	22	90	2.23519444	296.888889	23.88888888	0	0	1	0	42.288408	0	-0.4111751
5.5134	196156.767	0.30763033	21.6887304	0.60202456	0	1.70718149	5.27891927	-1.1756111	3.07683889	-0.5057973	-6.9077553	23	91	1.34111667	296.888889	23.88888888	0	0	1	0	42.288408	0	-0.4111751
0.0036	0	0.18133275	6.37634977	0.52180731	0	-5.6268214	-6.9077553	-1.701922	1.85275261	-0.6485423	-6.9077553	0	62	1.34111667	304.111111	31.11111111	0	0	0	0	18.9135952	0	-0.0428739
0.0036	0	0	0	0	0	-5.6268214	-6.9077553	-6.9077553	-6.9077553	-6.9077553	-6.9077553	1	71	1.78815556	301.333333	28.33333333	0	0	0	0	18.9135952	0	-0.0428739
0.0036	0	0	0	0	0	-5.6268214	-6.9077553	-6.9077553	-6.9077553	-6.9077553	-6.9077553	2	75	1.78815556	300.222222	27.2222222	0	0	0	0	18.9135952	0	-0.0428739
0.0036	0	0	0	0	0	-5.6268214	-6.9077553	-6.9077553	-6.9077553	-6.9077553	-6.9077553	3	82	1.34111667	299.111111	26.11111111	0	0	0	0	18.9135952	0	-0.0428739
0.0036	0	0	0	0	0	-5.6268214	-6.9077553	-6.9077553	-6.9077553	-6.9077553	-6.9077553	4	89	1.34111667	298	25	0	0	0	0	18.9135952	0	-0.0428739
0.0036	0	0	0	0	0	-5.6268214	-6.9077553	-6.9077553	-6.9077553	-6.9077553	-6.9077553	5	90	1.34111667	298	25	0	0	0	0	18.9135952	0	-0.0428739
0.0036	0	0	0	0	0	-5.6268214	-6.9077553	-6.9077553	-6.9077553	-6.9077553	-6.9077553	6	91	1.34111667	297.444444	24.44444444	0	0	0	0	18.9135952	0	-0.0428739
0.0036	0	0	0	0	0	-5.6268214	-6.9077553	-6.9077553	-6.9077553	-6.9077553	-6.9077553	7	93	1.34111667	297.444444	24.44444444	0	0	0	0	18.9135952	0	-0.0428739
0.0036	0	0	0	0	0	-5.6268214	-6.9077553	-6.9077553	-6.9077553	-6.9077553	-6.9077553	8	93	1.78815556	297.444444	24.44444444	0	0	0	0	18.9135952	0	-0.0428739
0.0036	0	0	0	0	0	-5.6268214	-6.9077553	-6.9077553	-6.9077553	-6.9077553	-6.9077553	9	93	2.23519444	297.444444	24.44444444	0	0	0	0	18.9135952	0	-0.0428739
0.0036	0	0	0	0	0	-5.6268214	-6.9077553	-6.9077553	-6.9077553	-6.9077553	-6.9077553	10	94	1.34111667	297.444444	24.44444444	0	0	0	0	18.9135952	0	-0.0428739
0.0036	0	0	0	0	0	-5.6268214	-6.9077553	-6.9077553	-6.9077553	-6.9077553	-6.9077553	11	92	1.34111667	298	25	0	0	0	0	18.9135952	0	-0.0428739
0.0036	0	0	0	0	0	-5.6268214	-6.9077553	-6.9077553	-6.9077553	-6.9077553	-6.9077553	12	88	2.68223333	299.111111	26.11111111	0	0	0	0	18.9135952	0	-0.0428739
0.0036	0	0	0	0	0	-5.6268214	-6.9077553	-6.9077553	-6.9077553	-6.9077553	-6.9077553	13	83	3.57631111	300.222222	27.2222222	0	0	0	0	18.9135952	0	-0.0428739
0.0504	0	0.61398902	105.15718	3.00008334	0	-2.9877641	-6.9077553	-0.4861509	4.65546569	1.09897334	-6.9077553	14	73	3.57631111	302.444444	29.44444444	0	0	0	0	18.9135952	0	-0.0428739
0.1647	0	0.51267375	72.0564561	2.35651572	0	-1.8036296	-6.9077553	-0.666167	4.2774638	0.8576084	-6.9077553	15	63	3.12927222	304.111111	31.11111111	0	0	0	0	18.9135952	0	-0.0428739
0.2826	504.636359	0.46756302	67.2178927	1.40029534	0	-1.2637228	-0.6819375	-0.7580847	4.20795435	0.33739706	-6.9077553	16	55	2.68223333	306.333333	33.33333333	0	0	1	0	18.9135952	0	-0.0428739
0.4689	6296.92431	0.79159597	224.049819	1.98361412	0	-0.7573658	1.8402201	-0.2324417	5.4118729	0.6854245	-6.9077553	17	50	3.12927222	307.444444	34.4444444	0	0	1	0	18.9135952	0	-0.0428739
0.6471	11687.8055	0.74627578	190.23041	1.76792892	0	-0.4352544	2.45863158	-0.291321	5.24824128	0.57037424	-6.9077553	18	47	3.57631111	307.444444	34.4444444	0	0	1	0	18.9135952	0	-0.0428739
0.8208	15540.1785	0.63508005	121.181367	1.43826721	0	-0.1974758	2.74349318	-0.4524309	4.79729658	0.3641341	-6.9077553	19	44	2.23519444	308.555556	35.5555556	0	0	1	0	18.9135952	0	-0.0428739

1.0269	20284.0395	0.69295052	140.116173	1.47089049	0	0.02654456	3.00988364	-0.3653546	4.94247902	0.38654763	-6.9077553	20	43	2.23519444	309.111111	36.11111111	0	0	1	0	18.9135952	0	-0.0428739
1.2375	25229.6851	0.8145439	250.525341	2.93954729	0	0.21309322	3.22806091	-0.2039	5.52356407	1.07859572	-6.9077553	21	88	5.81150556	296.333333	23.33333333	0	0	1	0	18.9135952	0	-0.0428739
1.2789	27159.6922	0.40873494	42.9047958	0.77915747	0	0.24600033	3.30177079	-0.8922448	3.75900692	-0.2482595	-6.9077553	22	90	2.23519444	296.888889	23.88888888	0	0	1	0	18.9135952	0	-0.0428739
1.3086	28431.7418	0.27399202	16.8589237	0.72560085	0	0.26895786	3.34754136	-1.2910132	2.82493943	-0.319378	-6.9077553	23	91	1.34111667	296.888889	23.88888888	0	0	1	0	18.9135952	0	-0.0428739
0.0063	223.175983	0.9190921	227.84729	1.51014105	0	-5.0672056	-1.4953239	-0.0832815	5.42868001	0.41286503	-6.9077553	0	85	1.34111667	298.555556	25.55555555	0	0	0	0	30.519848	0	0.21141735
0.0135	530.222526	0.49115813	59.9924088	0.84749234	0	-4.3050656	-0.6325743	-0.7089552	4.0942347	-0.1642942	-6.9077553	1	88	1.34111667	298.555556	25.55555555	0	0	0	0	30.519848	0	0.21141735
0.0279	1012.72415	0.61862034	94.702858	1.05630159	0	-3.5791286	0.01363083	-0.4786483	4.55075474	0.05572	-6.9077553	2	92	1.34111667	298	25	0	0	0	0	30.519848	0	0.21141735
0.036	1144.31545	0.63273483	98.0148383	1.17234887	0	-3.3242363	0.1356801	-0.4561247	4.58512908	0.15986194	-6.9077553	3	95	1.34111667	297.444444	24.44444444	0	0	0	0	30.519848	0	0.21141735
0.0396	1232.04296	0.4158451	40.448082	0.76347607	0	-3.2289262	0.20948506	-0.8750406	3.70004395	-0.2685646	-6.9077553	4	96	2.23519444	297.444444	24.44444444	0	0	0	0	30.519848	0	0.21141735
0.0405	1275.90675	0.50577933	59.019928	0.71241373	0	-3.2064533	0.24444055	-0.6796796	4.07789209	-0.3376938	-6.9077553	5	98	1.78815556	297.444444	24.44444444	0	0	0	0	30.519848	0	0.21141735
0.0405	1275.90675	0	0	0	0	-3.2064533	0.24444055	-6.9077553	-6.9077553	-6.9077553	-6.9077553	6	93	3.12927222	298.555556	25.55555555	0	0	0	0	30.519848	0	0.21141735
0.0963	1683.59364	0.34222664	31.4437453	1.43895955	0	-2.340287	0.52152437	-1.0693643	3.44823189	0.36461502	-6.9077553	7	80	4.02335	300.777778	27.7777778	0	0	0	0	30.519848	0	0.21141735
0.2331	2306.67445	0.55588672	90.9941214	2.52540468	0	-1.4562877	0.83624029	-0.5853934	4.51080589	0.92679722	-6.9077553	8	72	4.47038889	302.444444	29.44444444	0	0	0	0	30.519848	0	0.21141735
0.3186	3466.6727	0.55563909	85.9433645	1.27630993	0	-1.1438189	1.24348368	-0.5858382	4.45370016	0.24475625	-6.9077553	9	62	3.57631111	304.111111	31.11111111	0	0	0	0	30.519848	0	0.21141735
0.4257	4271.23502	0.59432054	93.8098657	1.76511479	0	-0.8540204	1.45213711	-0.5186553	4.54128069	0.5687821	-6.9077553	10	55	3.12927222	305.777778	32.7777778	0	0	0	1	30.519848	0	0.21141735
0.5976	5737.02669	0.62394714	104.411772	1.84333937	0	-0.5148336	1.74711537	-0.4700882	4.648352	0.61212115	-6.9077553	11	50	3.57631111	306.888889	33.88888888	0	0	0	1	30.519848	0	0.21141735
0.8055	8965.35449	0.66876894	124.667174	1.73422017	0	-0.2162921	2.19347918	-0.4008225	4.8256556	0.5511343	-6.9077553	12	46	2.68223333	307.444444	34.4444444	0	0	0	1	30.519848	0	0.21141735
1.0593	14803.8479	0.77917935	171.457225	1.97998891	0	0.05760831	2.69495469	-0.2482315	5.14433965	0.68359617	-6.9077553	13	41	2.68223333	309.111111	36.11111111	0	0	0	1	30.519848	0	0.21141735
1.3086	19809.2189	0.80294282	184.707102	1.98418617	0	0.26895786	2.98619791	-0.2182271	5.21877675	0.6857127	-6.9077553	14	38	3.12927222	309.666667	36.66666666	0	0	0	1	30.519848	0	0.21141735
1.611	24909.8407	0.73135312	148.162307	1.7788551	0	0.4768551	3.21530308	-0.3114925	4.99831509	0.57653195	-6.9077553	15	36	3.12927222	309.111111	36.11111111	0	0	0	1	30.519848	0	0.21141735
1.8837	30514.7212	0.76953951	169.982596	1.63542321	0	0.63323793	3.418242	-0.2606643	5.13570194	0.49251289	-6.9077553	16	38	3.12927222	308.555556	35.5555556	0	0	0	1	30.519848	0	0.21141735
2.1474	36721.7976	0.7602587	167.194366	1.51774873	0	0.76425781	3.60339775	-0.272782	5.11916298	0.41788679	-6.9077553	17	42	1.34111667	308	35	0	0	0	1	30.519848	0	0.21141735
2.3751	42386.5567	0.7158033	151.88553	1.36855006	0	0.86503954	3.74685485	-0.3329538	5.02313373	0.31448226	-6.9077553	18	54	1.34111667	304.666667	31.66666666	0	0	0	1	30.519848	0	0.21141735
2.5011	49519.1176	0.72318606	156.485728	1.1310961	0	0.91673064	3.902379	-0.3227069	5.0529712	0.12407087	-6.9077553	19	61	0.89407778	303	30	0	0	0	1	30.519848	0	0.21141735
2.5695	55916.4503	0.78803785	170.549394	1.14891494	0	0.94371133	4.0238765	-0.236941	5.13903082	0.13968797	-6.9077553	20	66	1.34111667	301.888889	28.88888888	0	0	0	1	30.519848	0	0.21141735
2.6019	60965.9665	0.67767043	119.377333	0.79235093	0	0.95624195	4.11033219	-0.3876197	4.78229772	-0.2314896	-6.9077553	21	66	1.34111667	301.333333	28.33333333	0	0	0	1	30.519848	0	0.21141735
2.6388	66756.1537	0.66411256	118.599681	0.85379131	0	0.97032427	4.20106146	-0.407799	4.77576223	-0.1568979	-6.9077553	22	69	1.34111667	300.777778	27.7777778	0	0	0	1	30.519848	0	0.21141735
2.6577	71482.1689	0.53046644	68.6900577	0.42229752	0	0.97746109	4.26946202	-0.6321152	4.22961903	-0.85968	-6.9077553	23	72	1.78815556	299.666667	26.66666666	0	0	0	1	30.519848	0	0.21141735
0.0153	711.405165	0.99216515	271.093506	1.80635417	0	-4.1799025	-0.3391085	-0.0068583	5.60246749	0.59186399	-6.9077553	0	85	1.34111667	298.555556	25.55555555	0	0	0	0	34.0724638	0	0.49277013
0.0243	1018.45179	0.50966396	61.5369324	0.84228697	0	-3.7172789	0.01926502	-0.6720435	4.11965377	-0.170448	-6.9077553	1	88	1.34111667	298.555556	25.55555555	0	0	0	0	34.0724638	0	0.49277013
0.0414	1632.54471	0.51104189	65.7239733	0.78829258	0	-3.1844744	0.49075232	-0.6693488	4.18547896	-0.2366182	-6.9077553	2	92	1.34111667	298	25	0	0	0	0	34.0724638	0	0.49277013
0.0558	2246.63777	0.55138183	73.3718185	0.77716672	0	-2.8859814	0.80987978	-0.5935158	4.29555355	-0.2508145	-6.9077553	3	95	1.34111667	297.444444	24.44444444	0	0	0	0	34.0724638	0	0.49277013
0.0855	3518.68718	0.60417805	90.9032241	0.99568246	0	-2.4592389	1.25837212	-0.5022326	4.50980647	-0.0033231	-6.9077553	4	96	2.23519444	297.444444	24.44444444	0	0	0	0	34.0724638	0	0.49277013
0.1251	5229.37456	0.56623121	77.8030007	0.83106102	0	-2.0786419	1.65448289	-0.5669883	4.35419285	-0.1838495	-6.9077553	5	98	1.78815556	297.444444	24.44444444	0	0	0	0	34.0724638	0	0.49277013
0.1647	7115.51731	0.61209609	91.5379028	0.91735268	0	-1.8036296	1.96241846	-0.4892336	4.51676405	-0.0851738	-6.9077553	6	93	3.12927222	298.555556	25.55555555	0	0	0	0	34.0724638	0	0.49277013
0.2187	9333.2477	0.75389131	148.104769	1.37065366	0	-1.5200544	2.23369018	-0.2811815	4.99792667	0.31601706	-6.9077553	7	80	4.02335	300.777778	27.7777778	0	0	0	0	34.0724638	0	0.49277013
0.3681	15464.4407	0.97223654	273.35754	2.77641113	0	-0.9994006	2.7386079	-0.0271281	5.61078427	1.02151924	-6.9077553	8	72	4.47038889	302.444444	29.44444444	0	0	0	1	34.0724638	0	0.49277013
0.6012	28044.5582	1.18549503	397.781371	3.10820756	1	-0.5088276	3.33383027	0.17100361	5.98590505	1.13436789	0.0009995	9	62	3.57631111	304.111111	31.11111111	0	0	0	1	34.0724638	0	0.49277013
0.9216	43604.456	1.0988929	333.719189	2.67740948	1	-0.081644	3.77518228	0.09521281	5.81030288	0.98522314	0.0009995	10	55	3.12927222	305.777778	32.7777778	0	0	0	1	34.0724638	0	0.49277013
1.2771	53250.9512	1.05472458	316.523536	2.46757318	1	0.24459188	3.97503445	0.05422734	5.75740076	0.90364032	0.0009995	11	50	3.57631111	306.888889	33.88888888	0	0	0	1	34.0724638	0	0.49277013
1.6731	65746.4564	0.97361665	263.051953	2.01903118	1	0.51467819	4.18582098	-0.0257111	5.57235535	0.70311295	0.0009995	12	46	2.68223333	307.444444	34.4444444	0	0	0	1	34.0724638	0	0.49277013
2.214	85146.0295	1.06578402	318.830153	2.54227126	1	0.79480083	4.44437952	0.06464853	5.76466166	0.93345115	0.0009995	13	41	2.68223333	309.111111	36.11111111	0	0	0	1	34.0724638	0	0.49277013
2.88	110096.389	1.04879266	311.639379	2.54487965	1	1.05779029	4.70136533	0.04859268	5.74184989	0.93447623	0.0009995	14	38	3.12927222	309.666667	36.66666666	0	0	0	1	34.0724638	0	0.49277013
3.4965	125357.082	1.00735505	295.938412	2.27598316	1	1.25176247	4.8311743	0.00832034	5.69015475	0.82285139	0.0009995	15	36	3.12927222	309.111111	36.11111111	0	0	0	1	34.0724638	0	0.49277013
4.0437	141462.525	0.95520592	255.602385	1.90050808	1	1.39716011	4.95204191	-0.044782	5.54362697	0.6426473	0.0009995	16	38	3.12927222	308.555556	35.5555556	0	0	0	1	34.0724638	0	0.49277013
4.4577	153061.89	0.83218928	192.671094	1.48076039	1	1.49463294	5.03084888	-0.1824944	5.26098975	0.39323083	0.0009995	17	42	1.34111667	308	35	0	0	0	1	34.0724638	0	0.49277013
4.8978	165860.501	0.79954332	178.937724	1.65578281	1	1.58878612	5.11115311	-0.2224646	5.18704342	0.50487766	0.0009995	18	54	1.34111667	304.666667	31.66666666	0	0	0	1	34.0724638	0	0.49277013
5.1813	177440.397	0.80851	183.358085	1.34977708	1	1.64505599	5.1786404	-0.2113262	5.21144645	0.30068004	0.0009995	19	61	0.89407778	303	30	0	0	0	1	34.0724638	0	0.49277013
5.3892	186701.92	0.76813841	153.958807	1.12588118	1	1.68439695	5.22951869	-0.2624843	5.03669157	0.1194538	0.0009995	20	66	1.34111667	301.888889	28.88888888	0	0	0	1	34.0724638	0	0.49277013
5.5899	195402.674	0.75365705	149.131481	1.18858248	1	1.7209614	5.27506754	-0.2814919	5.00483505	0.17360239	0.0009995	21	66	1.34111667	301.333333	28.33333333	0	0	0	1	34.0724638	0	0.49277013
5.7654	203315.706	0.77521519	160.740084	1.25712695	1	1.75187454	5.31476489	-0.2533255	5.0797949	0.22962407	0.0009995	22	69	1.34111667	300.777778	27.7777778	0	0	0	1	34.0724638	0	0.49277013
5.913	209688.259	0.73741894	141.831936	1.13160157	1	1.77715332	5.34562672	-0.3032439	4.95464986	0.12451726	0.0009995	23	72	1.78815556	299.666667	26.66666666	0	0	0	1	34.0724638	0	0.49277013
0.027	1537.86052	1.04865357	297.08129	2.0293003	0	-3.6119184	0.43104222	0.04846018	5.69400917	0.70818371	-6.9077553	0	85	1.34111667	298.555556	25.55555555	0	0	0	0	42.288408	0	-0.4111751
0.0711	3692.62122	0.71199908	130.464605	1.27005815	0	-2.6436679	1.30660734	-0.3382752	4.87110963	0.23984974	-6.9077553	1	88	1.34111667	298.555556	25.55555555	0	0	0	0	42.288408	0	-0.4111751
0.1242	6192.8576	0.62146953	97.3429721	1.07576816	0	-2.0858621	1.82355809	-0.4740606	4.57825081	0.07396411	-6.9077553	2	92	1.34111667	298	25	0	0	0	0	42.288408	0	-0.4111751
0.1863	9175.59441	0.64125785	103.629161	1.14411827	0	-1.680397	2.21665616	-0.4427654	4.64082842	0.13550792	-6.9077553	3	95	1.34111667	297.444444	24.44444444	0	0	0	0	42.288408	0	-0.4111751
0.2538	12333.7868	0.64574946	104.862096	1.16439443	0	-1.3712087	2.51242347	-0.4357963	4.65265566	0.1530596	-6.9077553	4	96	2.23519444	297.444444	24.44444444	0	0	0	0	42.288408	0	-0.4111751
0.333	16106.0722	0.69528172	122.202282	1.30305689	0	-1.0996128	2.77925844	-0.3620009	4.8056859	0.26548009	-6.9077553	5	98	1.78815556	297.444444	24.44444444	0	0	0	0	42.288408	0	-0.4111751
0.4266	20497.9595	0.68523796	123.775128	1.22840456	0	-0.8519085	3.02037413	-0.3765308	4.81847451	0.20652995	-6.9077553	6	93	3.12927222	298.555556	25.55555555	0	0	0	0	42.288408	0	-0.4111751
0.6543	28097.0122	0.72684434	155.101532	2.15162436	0	-0.4241893	3.33569883	-0.3176681	5.04408639	0.76668773	-6.9077553	7	80	4.02335	300.777778	27.7777778	0	0	1	0	42.288408	0	-0.4111751
1.0116	40891.9017	0.85703937	202.69671	2.09213065	0	0.01153324	3.71095649	-0.1531053	5.31171575	0.73866086	-6.9077553	8	72	4.47038889	302.444444	29.44444444	0	0	1	0	42.288408	0	-0.4111751
1.3536	54096.4309	0.85472974	192.504934	1.62318726	0	0.30276771	3.9907867	-0.1558007	5.26012698	0.48500754	-6.9077553	9	62	3.57631111	304.111111	31.11111111	0	0	1	0	42.288408	0	-0.4111751

1.7568	71151.5407	0.87624107	208.945801	1.55912181	0	0.56349397	4.26482603	-0.1309734	5.34207968	0.4447639	-6.9077553	10	55	3.12927222	305.777778	32.7777778	0	0	1	0	42.288408	0	-0.4111751
2.3157	92290.8211	0.9237721	241.575257	2.18093572	0	0.83971202	4.52495552	-0.0782079	5.48718519	0.78021243	-6.9077553	11	50	3.57631111	306.888889	33.8888889	0	0	1	0	42.288408	0	-0.4111751
2.9835	117636.496	0.9086488	236.387235	2.14992044	0	1.09309711	4.76760783	-0.0946967	5.46547552	0.76589586	-6.9077553	12	46	2.68223333	307.444444	34.4444444	0	0	1	0	42.288408	0	-0.4111751
3.7656	151085.533	0.97760249	279.671551	2.38930344	0	1.32590721	5.01785274	-0.0216298	5.63361946	0.87142032	-6.9077553	13	41	2.68223333	309.111111	36.1111111	0	0	1	0	42.288408	0	-0.4111751
4.5711	185322.481	0.98498385	284.718401	2.30420276	0	1.51975388	5.22210285	-0.0141153	5.65150414	0.83516864	-6.9077553	14	38	3.12927222	309.666667	36.6666667	0	0	1	0	42.288408	0	-0.4111751
5.2938	218569.825	1.00060024	291.381032	2.25093704	0	1.66653632	5.3871101	0.00159896	5.67463523	0.81179075	-6.9077553	15	36	3.12927222	309.111111	36.1111111	0	0	1	0	42.288408	0	-0.4111751
5.9373	241602.372	0.8590746	227.060402	1.95046814	0	1.78125448	5.48729742	-0.1507361	5.42522047	0.66858198	-6.9077553	16	38	3.12927222	308.555556	35.5555556	0	0	1	0	42.288408	0	-0.4111751
6.4134	253165.653	0.66205134	139.912187	1.42101982	0	1.85838955	5.53404798	-0.4109029	4.94102214	0.35207827	-6.9077553	17	42	1.34111667	308	35	0	0	1	0	42.288408	0	-0.4111751
6.8004	263655.955	0.63073716	135.654515	1.25933937	0	1.91698143	5.57464885	-0.4592819	4.91011869	0.23138103	-6.9077553	18	54	1.34111667	304.666667	31.6666667	0	0	1	0	42.288408	0	-0.4111751
7.0074	271455.839	0.74817757	164.693937	1.21163861	0	1.94696673	5.60380315	-0.2887792	5.10409489	0.19279865	-6.9077553	19	61	0.89407778	303	30	0	0	1	0	42.288408	0	-0.4111751
7.146	277252.269	0.75814557	153.596046	1.17357229	0	1.96655276	5.62493142	-0.2755617	5.03433259	0.16090408	-6.9077553	20	66	1.34111667	301.888889	28.8888889	0	0	1	0	42.288408	0	-0.4111751
7.2549	282178.529	0.77159478	153.479985	1.18125966	0	1.9816771	5.64254349	-0.2580006	5.03357668	0.16742757	-6.9077553	21	66	1.34111667	301.333333	28.3333333	0	0	1	0	42.288408	0	-0.4111751
7.3719	286813.874	0.7569333	149.607302	1.19126575	0	1.99767547	5.65883697	-0.2771599	5.00802056	0.17585549	-6.9077553	22	69	1.34111667	300.777778	27.7777778	0	0	1	0	42.288408	0	-0.4111751
7.4799	290890.421	0.74775976	148.608822	1.29358851	0	2.01221942	5.67295007	-0.2893371	5.00132422	0.25819289	-6.9077553	23	72	1.78815556	299.666667	26.6666667	0	0	1	0	42.288408	0	-0.4111751
0.0009	0	0	0	0	0	-7.0131158	-6.9077553	-6.9077553	-6.9077553	-6.9077553	-6.9077553	0	85	1.34111667	298.555556	25.5555556	0	0	0	0	18.9135952	0	-0.0428739
0.0009	0	0	0	0	0	-7.0131158	-6.9077553	-6.9077553	-6.9077553	-6.9077553	-6.9077553	1	88	1.34111667	298.555556	25.5555556	0	0	0	0	18.9135952	0	-0.0428739
0.0009	0	0	0	0	0	-7.0131158	-6.9077553	-6.9077553	-6.9077553	-6.9077553	-6.9077553	2	92	1.34111667	298	25	0	0	0	0	18.9135952	0	-0.0428739
0.0009	0	0	0	0	0	-7.0131158	-6.9077553	-6.9077553	-6.9077553	-6.9077553	-6.9077553	3	95	1.34111667	297.444444	24.4444444	0	0	0	0	18.9135952	0	-0.0428739
0.0009	0	0	0	0	0	-7.0131158	-6.9077553	-6.9077553	-6.9077553	-6.9077553	-6.9077553	4	96	2.23519444	297.444444	24.4444444	0	0	0	0	18.9135952	0	-0.0428739
0.0009	0	0	0	0	0	-7.0131158	-6.9077553	-6.9077553	-6.9077553	-6.9077553	-6.9077553	5	98	1.78815556	297.444444	24.4444444	0	0	0	0	18.9135952	0	-0.0428739
0.0009	0	0	0	0	0	-7.0131158	-6.9077553	-6.9077553	-6.9077553	-6.9077553	-6.9077553	6	93	3.12927222	298.555556	25.5555556	0	0	0	0	18.9135952	0	-0.0428739
0.0009	0	0	0	0	0	-7.0131158	-6.9077553	-6.9077553	-6.9077553	-6.9077553	-6.9077553	7	80	4.02335	300.777778	27.7777778	0	0	0	0	18.9135952	0	-0.0428739
0.0333	0	0.55429681	86.8930393	2.53492567	0	-3.4021979	-6.9077553	-0.5882525	4.46468944	0.93055873	-6.9077553	8	72	4.47038889	302.444444	29.4444444	0	0	0	0	18.9135952	0	-0.0428739
0.1449	272.844815	0.58060726	90.8488297	2.27351626	0	-1.9317114	-1.2951937	-0.5419599	4.50920792	0.82176739	-6.9077553	9	62	3.57631111	304.111111	31.1111111	0	0	0	0	18.9135952	0	-0.0428739
0.3429	3325.13823	0.69025588	157.219673	2.32027005	0	-1.0703164	1.20181194	-0.3692452	5.05765038	0.84211447	-6.9077553	10	55	3.12927222	305.777778	32.7777778	0	0	1	0	18.9135952	0	-0.0428739
0.5373	11200.4009	0.90332342	282.998644	2.31371957	0	-0.6211987	2.41603885	-0.1005682	5.64544564	0.83928855	-6.9077553	11	50	3.57631111	306.888889	33.8888889	0	0	1	0	18.9135952	0	-0.0428739
0.6831	15598.5389	0.75513793	186.946361	1.45290139	0	-0.381114	2.74724135	-0.2795315	5.23082709	0.37425056	-6.9077553	12	46	2.68223333	307.444444	34.4444444	0	0	1	0	18.9135952	0	-0.0428739
0.8595	19895.4706	0.69946328	147.181352	1.50485619	0	-0.1514045	2.99054236	-0.3560133	4.99167231	0.40936163	-6.9077553	13	41	2.68223333	309.111111	36.1111111	0	0	1	0	18.9135952	0	-0.0428739
1.1331	30160.2388	0.91429028	253.403526	2.16682595	0	0.12495724	3.40655762	-0.088514	5.53498713	0.7737248	-6.9077553	14	38	3.12927222	309.666667	36.6666667	0	0	1	0	18.9135952	0	-0.0428739
1.4571	41188.6829	0.90573764	244.708377	2.29618361	0	0.37644816	3.71818781	-0.0979021	5.50007129	0.83168385	-6.9077553	15	36	3.12927222	309.111111	36.1111111	0	0	1	0	18.9135952	0	-0.0428739
1.7514	52280.1039	0.86885383	225.31379	1.74837146	0	0.56041547	3.956635	-0.1394301	5.41749849	0.55925656	-6.9077553	16	38	3.12927222	308.555556	35.5555556	0	0	1	0	18.9135952	0	-0.0428739
2.0682	63854.304	0.79490245	196.038441	1.60177682	0	0.72667866	4.15661965	-0.2282786	5.27831587	0.47173764	-6.9077553	17	42	1.34111667	308	35	0	0	1	0	18.9135952	0	-0.0428739
2.4543	78959.3715	0.85383844	217.297895	1.79109331	0	0.89784159	4.3689461	-0.1568428	5.3812738	0.58338438	-6.9077553	18	54	1.34111667	304.666667	31.6666667	0	0	1	0	18.9135952	0	-0.0428739
2.7414	92265.2352	0.89866232	228.420553	1.62599034	0	1.00846874	4.52467826	-0.1057358	5.43119284	0.48673189	-6.9077553	19	61	0.89407778	303	30	0	0	1	0	18.9135952	0	-0.0428739
2.9412	101559.978	0.8519316	197.492047	1.48099305	0	1.07881766	4.62065939	-0.1590759	5.28570338	0.39338784	-6.9077553	20	66	1.34111667	301.888889	28.8888889	0	0	1	0	18.9135952	0	-0.0428739
3.1041	109114.646	0.81887505	179.378086	1.38790725	0	1.13272382	4.69240829	-0.1986033	5.18950137	0.32851729	-6.9077553	21	66	1.34111667	301.333333	28.3333333	0	0	1	0	18.9135952	0	-0.0428739
3.2733	117124.941	0.87163024	208.454413	1.67544263	0	1.18579865	4.76324977	-0.1362434	5.33972517	0.51667407	-6.9077553	22	69	1.34111667	300.777778	27.7777778	0	0	1	0	18.9135952	0	-0.0428739
3.4542	125634.689	0.89951184	216.399448	1.74650701	0	1.23959088	4.83338637	-0.104792	5.37713062	0.55793266	-6.9077553	23	72	1.78815556	299.666667	26.6666667	0	0	1	0	18.9135952	0	-0.0428739
0.009	363.276291	0.84376552	191.820557	1.56177266	0	-4.7105307	-1.0098427	-0.1686962	5.25656555	0.44646159	-6.9077553	0	88	4.02335	299.111111	26.1111111	0	0	0	0	30.519848	0	0.21141735
0.0135	582.595285	0.44735041	51.8142944	0.80028448	0	-4.3050656	-0.5385475	-0.8021802	3.94768537	-0.2215392	-6.9077553	1	89	3.57631111	298.555556	25.5555556	0	0	0	0	30.519848	0	0.21141735
0.0225	977.369277	0.59900417	86.457843	0.93674965	0	-3.79424	-0.0218681	-0.5108187	4.4596685	-0.0642723	-6.9077553	2	90	4.02335	298.555556	25.5555556	0	0	0	0	30.519848	0	0.21141735
0.0351	1284.41574	0.61240673	93.0480347	1.05605711	0	-3.3495541	0.2510822	-0.4887271	4.53312661	0.05548874	-6.9077553	3	89	3.57631111	298.555556	25.5555556	0	0	0	0	30.519848	0	0.21141735
0.0666	1635.32598	0.33960988	35.0994908	1.1425257	0	-2.7090507	0.49245347	-1.0770175	3.55821511	0.13411621	-6.9077553	4	84	4.02335	299.666667	26.6666667	0	0	0	0	30.519848	0	0.21141735
0.1611	1806.49601	0.47091075	64.0098958	2.31610819	0	-1.82573	0.59194247	-0.7509654	4.15905332	0.84031994	-6.9077553	5	76	4.91742778	301.333333	28.3333333	0	0	0	0	30.519848	0	0.21141735
0.2664	2571.70979	0.58145676	101.45285	2.343148	0	-1.3227563	0.94495974	-0.5405003	4.61960401	0.85192201	-6.9077553	6	73	5.36446667	301.888889	28.8888889	0	0	0	0	30.519848	0	0.21141735
0.351	3498.99005	0.54029063	80.2120751	1.33201826	0	-1.0469691	1.25276012	-0.6137989	4.38468653	0.28744574	-6.9077553	7	63	6.25854444	304.111111	31.1111111	0	0	0	0	30.519848	0	0.21141735
0.5238	4256.33419	0.60329413	95.7498983	2.06061729	0	-0.6466453	1.44864319	-0.5036942	4.56175001	0.72349077	-6.9077553	8	58	5.81150556	305.222222	32.2222222	0	0	0	1	30.519848	0	0.21141735
0.7488	9310.63597	0.64378723	116.524531	1.81691138	0	-0.2892834	2.2312648	-0.4388349	4.7581104	0.59768825	-6.9077553	9	55	4.47038889	306.333333	33.3333333	0	0	0	1	30.519848	0	0.21141735
1.0611	24166.0264	0.78981497	183.240353	2.32404075	0	0.05930611	3.18498916	-0.2346913	5.21080415	0.84373756	-6.9077553	10	51	5.36446667	306.888889	33.8888889	0	0	0	1	30.519848	0	0.21141735
1.3959	40836.6547	0.74313797	158.673051	1.97459034	0	0.33353937	3.70960457	-0.2955288	5.0668521	0.68086726	-6.9077553	11	52	4.47038889	307.444444	34.4444444	0	0	0	1	30.519848	0	0.21141735
1.7928	57113.5226	0.72482134	152.86887	1.88845666	0	0.58377864	4.04505842	-0.3204514	5.02958704	0.63628931	-6.9077553	12	50	4.02335	308	35	0	0	0	1	30.519848	0	0.21141735
2.1978	76534.6884	0.83360745	224.723802	2.20402398	0	0.78745686	4.33775715	-0.1807938	5.41487655	0.79073838	-6.9077553	13	51	3.57631111	308	35	0	0	0	1	30.519848	0	0.21141735
2.5632	102840.042	0.71994409	139.56478	1.67900709	0	0.94125648	4.63318452	-0.3271937	4.93853603	0.51879802	-6.9077553	14	51	4.02335	307.444444	34.4444444	0	0	0	1	30.519848	0	0.21141735
2.8089	130933.518	0.70614948	140.68853	1.00809331	0	1.03279295	4.87469733	-0.3465132	4.94655555	0.00905221	-6.9077553	15	54	2.68223333	306.888889	33.8888889	0	0	0	1	30.519848	0	0.21141735
2.9673	153776.363	0.71972197	146.425004	0.96187505	0	1.08765245	5.03550586	-0.3275018	4.98652021	-0.0378316	-6.9077553	16	61	0.89407778	304.666667	31.6666667	0	0	0	1	30.519848	0	0.21141735
3.0798	173520.301	0.69853296	130.083969	0.85424438	0	1.12486466	5.15630037	-0.3573424	4.86818784	-0.156368	-6.9077553	17	72	2.68223333	303	30	0	0	0	1	30.519848	0	0.21141735
3.1122	176039.772	0.55075545	75.5750529	0.75710678	0	1.13532987	5.17071563	-0.5946504	4.32513947	-0.276931	-6.9077553	18	74	3.57631111	301.888889	28.8888889	0	0	0	1	30.519848	0	0.21141735
3.1311	176741.593	0.42586313	41.8017375	0.65020034	0	1.14138438	5.1746944	-0.8512919	3.73296183	-0.4289379	-6.9077553	19	78	2.68223333	300.777778	27.7777778	0	0	0	1	30.519848	0	0.21141735
3.141	177224.095	0.31567203	21.4908461	0.46494181	0	1.14454122	5.17742065	-1.1498886	3.06767361	-0.7636945	-6.9077553	20	80	2.68223333	300.222222	27.2222222	0	0	0	1	30.519848	0	0.21141735
3.1518	177618.866	0.2413426	12.4658394	0.37412592	0	1.14797372	5.17964568	-1.4174028	2.52307227	-0.9804935	-6.9077553	21	82	2.68223333	299.666667	26.6666667	0	0	0	1	30.519848	0	0.21141735
3.1599	177882.053	0.24720645	12.5960439	0.32701421	0	1.15054038	5.18112633	-1.3934944	2.53346218	-1.1146984	-6.9077553	22	85	1.78815556	298.555556	25.5555556	0	0	0	1	30.519848	0	0.21141735
3.1698	178276.82	0.24788369	12.7885895	0.37101473	0	1.15366849	5.18334312	-1.3907696	2.54863152	-0.9888218	-6.9077553	23	89	2.23519444	298.555556	25.5555556	0	0	0	1	30.519848	0	0.21141735

0.0171	756.987415	0.9890319	280.075901	1.99409231	0	-4.0686768	-0.2770885	-0.0100181	5.63506421	0.69069032	-6.9077553	0	88	4.02335	299.111111	26.11111111	0	0	0	0	34.0724638	0	0.49277013
0.0324	1327.21664	0.54118835	71.8409567	0.72815216	0	-3.4295969	0.28383717	-0.6121418	4.27446866	-0.3158728	-6.9077553	1	89	3.57631111	298.555556	25.55555555	0	0	0	0	34.0724638	0	0.49277013
0.0522	1897.44572	0.56450974	84.1597124	1.0168151	0	-2.9526728	0.64103551	-0.5700278	4.43272821	0.01765827	-6.9077553	2	90	4.02335	298.555556	25.55555555	0	0	0	0	34.0724638	0	0.49277013
0.0756	2994.04028	0.59647501	88.3455717	0.91626982	0	-2.582299	1.09695768	-0.5150428	4.48126739	-0.0863536	-6.9077553	3	89	3.57631111	298.555556	25.55555555	0	0	0	0	34.0724638	0	0.49277013
0.1152	4822.27889	0.72585102	137.935913	1.21873084	0	-2.1610855	1.57345396	-0.3190338	4.92679643	0.19863021	-6.9077553	4	84	4.02335	299.666667	26.66666666	0	0	0	0	34.0724638	0	0.49277013
0.1845	8128.33349	0.89142698	214.030261	1.85959814	0	-1.6901058	2.09547894	-0.1138106	5.36612208	0.62089801	-6.9077553	5	76	4.91742778	301.333333	28.33333333	0	0	0	0	34.0724638	0	0.49277013
0.3114	13680.4172	1.02119024	293.53258	2.70935448	0	-1.166677	2.6160385	0.02194762	5.68199204	0.99707943	-6.9077553	6	73	5.36446667	301.888889	28.88888888	0	0	0	0	34.0724638	0	0.49277013
0.6255	30759.9977	1.28275996	490.422949	4.13563726	0	-0.4692039	3.42624758	0.24979324	6.19527022	1.4198832	-6.9077553	7	63	6.25854444	304.111111	31.11111111	0	0	0	1	34.0724638	0	0.49277013
1.1007	49163.7174	1.24006214	455.965436	3.76691737	0	0.09594634	3.89517624	0.21596757	6.1224192	1.32652243	-6.9077553	8	58	5.81150556	305.222222	32.2222222	0	0	0	1	34.0724638	0	0.49277013
1.602	64816.5437	1.12885398	364.284672	2.94336376	0	0.47125285	4.1715763	0.1220884	5.89793837	1.07989275	-6.9077553	9	55	4.47038889	306.333333	33.33333333	0	0	0	1	34.0724638	0	0.49277013
2.2311	85100.64	1.16182035	396.217275	3.43215196	0	0.80249474	4.44384631	0.15084839	5.98196526	1.23347878	-6.9077553	10	51	5.36446667	306.888889	33.88888888	0	0	0	1	34.0724638	0	0.49277013
2.7801	104313.375	1.03188987	299.503586	2.58821321	0	1.0224869	4.64740918	0.03236057	5.70212973	0.95135405	-6.9077553	11	52	4.47038889	307.444444	34.4444444	0	0	0	1	34.0724638	0	0.49277013
3.2526	119266.797	0.93555914	239.836384	1.97486072	0	1.17945468	4.78137136	-0.0655426	5.47996113	0.68100411	-6.9077553	12	50	4.02335	308	35	0	0	0	1	34.0724638	0	0.49277013
3.7719	136485.153	0.95512858	253.990305	2.20095905	0	1.32757885	4.91622317	-0.0448629	5.53730004	0.78934744	-6.9077553	13	51	3.57631111	308	35	0	0	0	1	34.0724638	0	0.49277013
4.2723	149409.608	0.84436013	205.528791	1.9535966	0	1.45215232	5.00669828	-0.1679926	5.32559099	0.67018383	-6.9077553	14	51	4.02335	307.444444	34.4444444	0	0	0	1	34.0724638	0	0.49277013
4.6296	159051.68	0.71584868	144.963112	1.40228564	0	1.53247047	5.06923547	-0.3328905	4.97648621	0.33881637	-6.9077553	15	54	2.68223333	306.888889	33.88888888	0	0	0	1	34.0724638	0	0.49277013
4.8537	166664.093	0.74339392	156.709086	1.16233587	0	1.5797413	5.11598637	-0.2951849	5.05439751	0.15129163	-6.9077553	16	61	0.89407778	304.666667	31.66666666	0	0	0	1	34.0724638	0	0.49277013
5.0004	172045.088	0.72019547	135.682923	1.09151978	0	1.60951791	5.14776239	-0.3268451	4.91032808	0.08848676	-6.9077553	17	72	2.68223333	303	30	0	0	0	1	34.0724638	0	0.49277013
5.0958	175422.606	0.55335434	83.3068065	0.9000802	0	1.62841667	5.16720365	-0.5899512	4.42254226	-0.104161	-6.9077553	18	74	3.57631111	301.888889	28.88888888	0	0	0	1	34.0724638	0	0.49277013
5.1678	178054.43	0.41390791	54.9017639	0.77821741	0	1.64244707	5.18209491	-0.8796987	4.00556369	-0.2494652	-6.9077553	19	78	2.68223333	300.777778	27.7777778	0	0	0	1	34.0724638	0	0.49277013
5.2092	179896.71	0.2860158	20.2620398	0.46666187	0	1.65042629	5.19238841	-1.248218	3.00879853	-0.7600097	-6.9077553	20	80	2.68223333	300.222222	27.2222222	0	0	0	1	34.0724638	0	0.49277013
5.2596	181870.576	0.28290108	21.8061523	0.49500544	0	1.66005498	5.20330081	-1.2591294	3.08223801	-0.7011684	-6.9077553	21	82	2.68223333	299.666667	26.66666666	0	0	0	1	34.0724638	0	0.49277013
5.301	183405.804	0.23573096	12.4398193	0.47770048	0	1.66789548	5.21170666	-1.440831	2.52098295	-0.7366802	-6.9077553	22	85	1.78815556	298.555556	25.55555555	0	0	0	1	34.0724638	0	0.49277013
5.3496	185291.944	0.27343734	20.6431885	0.42511216	0	1.67702179	5.22193805	-1.2930323	3.02743385	-0.8530527	-6.9077553	23	89	2.23519444	298.555556	25.55555555	0	0	0	1	34.0724638	0	0.49277013
0.036	2347.98329	1.14306093	362.164338	2.50110568	0	-3.3242363	0.85398259	0.13458415	5.89210084	0.91713265	-6.9077553	0	88	4.02335	299.111111	26.11111111	0	0	0	0	42.288408	0	-0.4111751
0.0855	4726.64831	0.71764603	134.627388	1.37762299	0	-2.4592389	1.55342789	-0.3303864	4.9025183	0.32108516	-6.9077553	1	89	3.57631111	298.555556	25.55555555	0	0	0	0	42.288408	0	-0.4111751
0.1413	7358.48889	0.65044711	109.048426	1.23800302	0	-1.95687	1.99599049	-0.4285591	4.69180123	0.21430704	-6.9077553	2	90	4.02335	298.555556	25.55555555	0	0	0	0	42.288408	0	-0.4111751
0.2043	10385.3713	0.70714188	128.793499	1.25848618	0	-1.5881658	2.3404945	-0.3451108	4.8582181	0.23070384	-6.9077553	3	89	3.57631111	298.555556	25.55555555	0	0	0	0	42.288408	0	-0.4111751
0.3024	15382.3491	0.87578828	211.521413	1.68930558	0	-1.1960046	2.7332857	-0.1314897	5.35433096	0.52490933	-6.9077553	4	84	4.02335	299.666667	26.66666666	0	0	0	0	42.288408	0	-0.4111751
0.5976	26361.3343	0.88130318	223.035585	2.61161841	0	-0.5148336	3.27193626	-0.1252195	5.40733582	0.96035294	-6.9077553	5	76	4.91742778	301.333333	28.33333333	0	0	0	0	42.288408	0	-0.4111751
0.9396	38607.7387	0.88769917	213.45588	2.24109915	0	-0.062301	3.65347864	-0.1179965	5.36343484	0.80741255	-6.9077553	6	73	5.36446667	301.888889	28.88888888	0	0	1	0	42.288408	0	-0.4111751
1.3725	55595.5117	0.93320311	242.639423	2.41778019	0	0.31663389	4.01812046	-0.0680614	5.49158061	0.88326336	-6.9077553	7	63	6.25854444	304.111111	31.11111111	0	0	1	0	42.288408	0	-0.4111751
1.9197	75938.4964	0.90735064	227.509509	2.46335341	0	0.65216892	4.32993692	-0.0961248	5.42719643	0.90192946	-6.9077553	8	58	5.81150556	305.222222	32.2222222	0	0	1	0	42.288408	0	-0.4111751
2.5632	103309.892	0.96527876	260.258304	2.35643507	0	0.94125648	4.63774281	-0.0343029	5.56167846	0.85757419	-6.9077553	9	55	4.47038889	306.333333	33.33333333	0	0	1	0	42.288408	0	-0.4111751
3.2274	133725.85	0.98643101	276.71369	2.16623661	0	1.17167686	4.89579929	-0.0126486	5.62298697	0.7734529	-6.9077553	10	51	5.36446667	306.888889	33.88888888	0	0	1	0	42.288408	0	-0.4111751
4.0887	175593.662	1.07673745	346.0481	2.79351979	0	1.40822707	5.16817828	0.07486389	5.84658067	1.02766028	-6.9077553	11	52	4.47038889	307.444444	34.4444444	0	0	1	0	42.288408	0	-0.4111751
4.7601	201628.794	0.93457391	253.046184	2.33193438	0	1.56026868	5.30643332	-0.0665951	5.53357597	0.84712687	-6.9077553	12	50	4.02335	308	35	0	0	1	0	42.288408	0	-0.4111751
5.3325	221755.815	0.81260998	189.378476	1.85524495	0	1.67382017	5.40158135	-0.2062742	5.24375281	0.61855561	-6.9077553	13	51	3.57631111	308	35	0	0	1	0	42.288408	0	-0.4111751
5.8131	238334.503	0.78822698	174.521872	1.54113907	0	1.76011399	5.47367936	-0.2367013	5.16205581	0.43317046	-6.9077553	14	51	4.02335	307.444444	34.4444444	0	0	1	0	42.288408	0	-0.4111751
6.2046	251581.705	0.77085087	177.714224	1.42207845	0	1.82529095	5.52777178	-0.2589639	5.1801824	0.35282245	-6.9077553	15	54	2.68223333	306.888889	33.88888888	0	0	1	0	42.288408	0	-0.4111751
6.4647	261594.024	0.78295476	181.771167	1.34253901	0	1.86635661	5.5667976	-0.243404	5.20275407	0.29530719	-6.9077553	16	61	0.89407778	304.666667	31.66666666	0	0	1	0	42.288408	0	-0.4111751
6.6069	267312.78	0.73160302	143.928847	1.18570294	0	1.88811456	5.58842317	-0.3111513	4.96932601	0.17117882	-6.9077553	17	72	2.68223333	303	30	0	0	1	0	42.288408	0	-0.4111751
6.7059	271268.029	0.61615247	110.94834	1.06463672	0	1.90298774	5.60311105	-0.4826392	4.7090737	0.06357248	-6.9077553	18	74	3.57631111	301.888889	28.88888888	0	0	1	0	42.288408	0	-0.4111751
6.7662	273899.864	0.38367382	34.6560678	0.62914128	0	1.91193963	5.61276623	-0.9553595	3.54550168	-0.4618112	-6.9077553	19	78	2.68223333	300.777778	27.7777778	0	0	1	0	42.288408	0	-0.4111751
6.8076	275829.88	0.24750415	12.8210118	0.35791107	0	1.91803964	5.61978793	-1.3922957	2.55116336	-1.0246806	-6.9077553	20	80	2.68223333	300.222222	27.2222222	0	0	1	0	42.288408	0	-0.4111751
6.8382	277145.777	0.22023794	10.13634	0.28038771	0	1.92252454	5.62454725	-1.5085165	2.31622563	-1.2680218	-6.9077553	21	82	2.68223333	299.666667	26.66666666	0	0	1	0	42.288408	0	-0.4111751
6.876	278593.302	0.26053533	18.8190264	0.37383706	0	1.92803709	5.62975661	-1.3411859	2.93492154	-0.9812638	-6.9077553	22	85	1.78815556	298.555556	25.55555555	0	0	1	0	42.288408	0	-0.4111751
6.912	279777.616	0.29152613	30.5897705	0.48685331	0	1.93325903	5.63399863	-1.2292013	3.42069835	-0.7177405	-6.9077553	23	89	2.23519444	298.555556	25.55555555	0	0	1	0	42.288408	0	-0.4111751
0.0009	0	0	0	0	0	-7.0131158	-6.9077553	-6.9077553	-6.9077553	-6.9077553	-6.9077553	0	88	4.02335	299.111111	26.11111111	0	0	0	0	18.9135952	0	-0.0428739
0.0009	0	0	0	0	0	-7.0131158	-6.9077553	-6.9077553	-6.9077553	-6.9077553	-6.9077553	1	89	3.57631111	298.555556	25.55555555	0	0	0	0	18.9135952	0	-0.0428739
0.0009	0	0	0	0	0	-7.0131158	-6.9077553	-6.9077553	-6.9077553	-6.9077553	-6.9077553	2	90	4.02335	298.555556	25.55555555	0	0	0	0	18.9135952	0	-0.0428739
0.0009	0	0	0	0	0	-7.0131158	-6.9077553	-6.9077553	-6.9077553	-6.9077553	-6.9077553	3	89	3.57631111	298.555556	25.55555555	0	0	0	0	18.9135952	0	-0.0428739
0.0009	0	0	0	0	0	-7.0131158	-6.9077553	-6.9077553	-6.9077553	-6.9077553	-6.9077553	4	84	4.02335	299.666667	26.66666666	0	0	0	0	18.9135952	0	-0.0428739
0.0288	0	0.49159542	66.2981036	2.15039555	0	-3.5473799	-6.9077553	-0.7080671	4.19417638	0.76611672	-6.9077553	5	76	4.91742778	301.333333	28.33333333	0	0	0	0	18.9135952	0	-0.0428739
0.1863	2766.87858	0.77569903	194.171808	2.72481166	0	-1.680397	1.01808117	-0.2527024	5.26874853	1.00276624	-6.9077553	6	73	5.36446667	301.888889	28.88888888	0	0	0	0	18.9135952	0	-0.0428739
0.45	11346.3547	0.90897554	275.556234	3.08195063	0	-0.7985077	2.42898465	-0.0943376	5.61879535	1.12588714	-6.9077553	7	63	6.25854444	304.111111	31.11111111	0	0	1	0	18.9135952	0	-0.0428739
0.6147	15126.0812	0.72365407	178.959539	1.64019242	0	-0.4866209	2.7164866	-0.3220609	5.18716533	0.49542306	-6.9077553	8	58	5.81150556	305.222222	32.2222222	0	0	1	0	18.9135952	0	-0.0428739
0.8055	19990.805	0.71904064	157.788104	1.70619605	0	-0.2162921	2.99532244	-0.3284476	5.06125936	0.53485229	-6.9077553	9	55	4.47038889	306.333333	33.33333333	0	0	1	0	18.9135952	0	-0.0428739
1.0584	28724.2053	0.83654997	216.855121	1.88969058	0	0.05675833	3.35777497	-0.1772744	5.3792341	0.63694215	-6.9077553	10	51	5.36446667	306.888889	33.88888888	0	0	1	0	18.9135952	0	-0.0428739
1.3635	38521.6457	0.86095129	221.612832	2.20034979	0	0.31005492	3.65124627	-0.1485565	5.40093637	0.78907071	-6.9077553	11	52	4.47038889	307.444444	34.4444444	0	0	1	0	18.9135952	0	-0.0428739
1.656	48437.9992	0.84599797	206.754567	1.74473013	0	0.50440506	3.88030526	-0.166057	5.33153726	0.55717288	-6.9077553	12	50	4.02335	308	35	0	0	1	0	18.9135952	0	-0.0428739
1.926	57960.0158	0.79700984	190.360495	1.55622742	0	0.65544531	4.05977064	-0.2256344	5.24892487	0.44290694	-6.9077553	13	51	3.57631111	308	35	0	0	1	0	18.9135952	0	-0.0428739

2.2365	68364.8065	0.79096352	185.579008	1.62869112	0	0.80491214	4.22487279	-0.2332399	5.2234861	0.4883905	-6.9077553	14	51	4.02335	307.444444	34.4444444	0	0	1	0	18.9135952	0	-0.0428739
2.5866	83176.1877	0.86241248	224.672196	1.72143727	0	0.95034427	4.42097312	-0.1468627	5.41464689	0.54374031	-6.9077553	15	54	2.68223333	306.888889	33.88888888	0	0	1	0	18.9135952	0	-0.0428739
2.8368	93878.5445	0.82825058	194.718104	1.4353057	0	1.04267666	4.54201252	-0.1872329	5.27155803	0.36207433	-6.9077553	16	61	0.89407778	304.666667	31.66666666	0	0	1	0	18.9135952	0	-0.0428739
2.997	101318.381	0.83934655	190.099697	1.51174961	0	1.09761179	4.61827772	-0.1739409	5.24755392	0.41392893	-6.9077553	17	72	2.68223333	303	30	0	0	1	0	18.9135952	0	-0.0428739
3.0987	105979.093	0.63203356	102.80442	1.10395813	0	1.13098267	4.66325128	-0.4572318	4.63283807	0.09980744	-6.9077553	18	74	3.57631111	301.888889	28.88888888	0	0	1	0	18.9135952	0	-0.0428739
3.1626	108654.787	0.37870789	34.3262131	0.66247064	0	1.15139447	4.68818497	-0.968353	3.53593843	-0.4102707	-6.9077553	19	78	2.68223333	300.777778	27.7777778	0	0	1	0	18.9135952	0	-0.0428739
3.1977	110014.569	0.26966978	18.2513475	0.4547459	0	1.1624318	4.70062189	-1.3068557	2.9042937	-0.7858199	-6.9077553	20	80	2.68223333	300.222222	27.2222222	0	0	1	0	18.9135952	0	-0.0428739
3.2418	112032.29	0.24887019	13.76135	0.42147531	0	1.17612873	4.71879606	-1.3868137	2.62193661	-0.8616243	-6.9077553	21	82	2.68223333	299.666667	26.66666666	0	0	1	0	18.9135952	0	-0.0428739
3.285	113918.429	0.26310237	14.9485359	0.42047258	0	1.18936665	4.73549144	-1.3314185	2.70468026	-0.8640006	-6.9077553	22	85	1.78815556	298.555556	25.55555555	0	0	1	0	18.9135952	0	-0.0428739
3.3291	116023.901	0.24368617	12.9405044	0.4086287	0	1.202702	4.75380483	-1.4077788	2.56043954	-0.8925041	-6.9077553	23	89	2.23519444	298.555556	25.55555555	0	0	1	0	18.9135952	0	-0.0428739
0.0126	406.654966	0.89291411	216.614746	1.63371937	0	-4.3740585	-0.8973341	-0.1121456	5.37812503	0.49147115	-6.9077553	0	94	1.34111667	276.888889	3.888888888	0	0	0	0	30.519848	0	0.21141735
0.0324	1209.87518	0.72578044	147.540461	1.24924252	0	-3.4295969	0.19134339	-0.3191309	4.99410923	0.22333755	-6.9077553	1	58	1.78815556	284.666667	11.66666666	0	0	0	0	30.519848	0	0.21141735
0.0927	1800.74933	0.57755467	106.55976	1.37609875	0	-2.3783868	0.58875804	-0.5472222	4.66871534	0.31997893	-6.9077553	2	36	3.12927222	290.222222	17.2222222	0	0	0	0	30.519848	0	0.21141735
0.1791	2795.32842	0.50452034	76.4025981	1.49455579	0	-1.719811	1.02830728	-0.682167	4.33602979	0.4024979	-6.9077553	3	31	3.57631111	293.555556	20.55555555	0	0	0	0	30.519848	0	0.21141735
0.4239	4892.58073	0.7516631	164.404096	3.50146888	0	-0.8582577	1.58792429	-0.2841376	5.10233348	1.25346811	-6.9077553	4	22	5.36446667	296.333333	23.33333333	0	0	0	1	30.519848	0	0.21141735
0.7659	12537.6537	0.7727172	168.845806	3.09638415	0	-0.2667037	2.52881617	-0.2565488	5.12899183	1.13055793	-6.9077553	5	20	5.81150556	297.444444	24.44444444	0	0	0	1	30.519848	0	0.21141735
1.1457	26099.0271	0.84836445	210.662267	2.71672977	0	0.1360158	3.26193635	-0.1632669	5.35026097	0.99979689	-6.9077553	6	18	4.91742778	298.555556	25.55555555	0	0	0	1	30.519848	0	0.21141735
1.6182	45402.1643	0.8377875	206.817232	2.73728423	0	0.48131442	3.8155818	-0.1757979	5.3318403	1.00733153	-6.9077553	7	16	5.36446667	299.111111	26.11111111	0	0	0	1	30.519848	0	0.21141735
2.1186	68777.763	0.93889096	284.84434	2.76126691	0	0.75075549	4.23089502	-0.0619914	5.65194637	1.01605169	-6.9077553	8	18	5.36446667	298.555556	25.55555555	0	0	0	1	30.519848	0	0.21141735
2.4804	96634.2846	0.78283312	170.091107	1.55393473	0	0.90841984	4.57094394	-0.2435591	5.1363401	0.44143357	-6.9077553	9	25	2.68223333	295.222222	22.2222222	0	0	0	1	30.519848	0	0.21141735
2.7801	125240.149	0.79239845	173.210328	1.30097389	0	1.0224869	4.83024107	-0.2314297	5.1545124	0.26388149	-6.9077553	10	29	1.34111667	293	20	0	0	0	1	30.519848	0	0.21141735
3.0582	151774.581	0.75005521	154.806938	1.33723247	0	1.11782651	5.02240299	-0.2862761	5.04218524	0.29134969	-6.9077553	11	31	1.34111667	290.777778	17.7777778	0	0	0	1	30.519848	0	0.21141735
3.2625	176358.387	0.78308468	172.62235	1.21641614	0	1.18249377	5.17252389	-0.2432382	5.15111205	0.1967307	-6.9077553	12	33	2.68223333	290.222222	17.2222222	0	0	0	1	30.519848	0	0.21141735
3.4416	202033.264	0.76017232	164.27492	1.13421823	0	1.23593648	5.30843731	-0.2728955	5.10154745	0.1268249	-6.9077553	13	35	3.12927222	290.222222	17.2222222	0	0	0	1	30.519848	0	0.21141735
3.6378	232110.258	0.7111134	135.31138	1.50599432	0	1.2913791	5.44721681	-0.3395181	4.90758603	0.41011715	-6.9077553	14	36	3.57631111	290.222222	17.2222222	0	0	0	1	30.519848	0	0.21141735
3.7998	262180.214	0.66775182	118.428375	1.04190742	0	1.33494843	5.56903592	-0.4023423	4.77431679	0.04201241	-6.9077553	15	40	3.57631111	289.111111	16.11111111	0	0	0	1	30.519848	0	0.21141735
3.9267	293112.246	0.65744506	116.200923	0.65056248	0	1.36779938	5.68055904	-0.4178742	4.75532939	-0.428382	-6.9077553	16	44	3.57631111	288	15	0	0	0	1	30.519848	0	0.21141735
4.0419	324851.758	0.6253013	103.021988	0.63347173	0	1.39671488	5.78337203	-0.4679237	4.63495215	-0.4549626	-6.9077553	17	44	4.91742778	286.888889	13.88888888	0	0	0	1	30.519848	0	0.21141735
4.14	357228.855	0.63002308	100.773966	0.53296728	0	1.42069579	5.87837943	-0.4604128	4.61288997	-0.6274207	-6.9077553	18	51	4.02335	285.777778	12.77777777	0	0	0	1	30.519848	0	0.21141735
4.2282	388938.392	0.61455894	93.7806023	0.42697085	0	1.44177637	5.96342353	-0.4852246	4.5409687	-0.8487002	-6.9077553	19	63	2.23519444	284.666667	11.66666666	0	0	0	1	30.519848	0	0.21141735
4.2921	412637.115	0.59428674	85.6614423	0.40148681	0	1.45677612	6.02257097	-0.5187121	4.45041448	-0.9100929	-6.9077553	20	71	2.23519444	284.666667	11.66666666	0	0	0	1	30.519848	0	0.21141735
4.3641	439006.087	0.52376208	65.0476807	0.33357649	0	1.47341198	6.08451556	-0.6448103	4.17513592	-1.0948898	-6.9077553	21	73	2.68223333	284.666667	11.66666666	0	0	0	1	30.519848	0	0.21141735
4.4199	459033.174	0.50911325	61.8318541	0.34896703	0	1.48611707	6.12912466	-0.6731225	4.12443484	-1.0499163	-6.9077553	22	79	3.57631111	284.666667	11.66666666	0	0	0	1	30.519848	0	0.21141735
4.4568	472384.563	0.45612484	49.4880609	0.33886865	0	1.49443102	6.15779552	-0.7827988	3.90175165	-1.0791961	-6.9077553	23	83	4.47038889	286.333333	13.33333333	0	0	0	1	30.519848	0	0.21141735
0.0135	610.414007	0.97674097	264.593855	1.81012739	0	-4.3050656	-0.491981	-0.0225105	5.57819981	0.59394952	-6.9077553	0	94	1.34111667	276.888889	3.888888888	0	0	0	0	34.0724638	0	0.49277013
0.0252	1049.05191	0.55391777	80.2234732	0.95211609	0	-3.6809113	0.0488396	-0.5889353	4.38482862	-0.0480186	-6.9077553	1	58	1.78815556	284.666667	11.66666666	0	0	0	0	34.0724638	0	0.49277013
0.0936	3981.79549	0.92474436	241.944901	2.15732795	0	-2.3687249	1.38198396	-0.0771572	5.48871415	0.76933383	-6.9077553	2	36	3.12927222	290.222222	17.2222222	0	0	0	0	34.0724638	0	0.49277013
0.1791	8035.50264	0.9862222	259.043195	1.73573753	0	-1.719811	2.08399399	-0.0128601	5.55699868	0.55200837	-6.9077553	3	31	3.57631111	293.555556	20.55555555	0	0	0	0	34.0724638	0	0.49277013
0.2952	12386.5342	1.00755854	269.958212	2.48468467	0	-1.2201022	2.51669066	0.00852213	5.59827088	0.91054814	-6.9077553	4	22	5.36446667	296.333333	23.33333333	0	0	0	1	34.0724638	0	0.49277013
0.6093	33059.2143	1.49859427	658.175233	5.55947797	0	-0.4954445	3.49833058	0.40519458	6.48947273	1.71568407	-6.9077553	5	20	5.81150556	297.444444	24.44444444	0	0	0	1	34.0724638	0	0.49277013
1.0152	56305.2647	1.36144911	543.626905	3.93173211	1	0.01508564	4.0308058	0.3092839	6.29826502	1.36933438	0.0009995	6	18	4.91742778	298.555556	25.55555555	0	0	0	1	34.0724638	0	0.49277013
1.6794	76083.0163	1.31839775	528.547044	4.26947303	1	0.51843659	4.33183821	0.27717538	6.27013371	1.4517246	0.0009995	7	16	5.36446667	299.111111	26.11111111	0	0	0	1	34.0724638	0	0.49277013
2.4309	104263.654	1.21216734	432.729192	3.3069412	1	0.88826156	4.64693242	0.19323458	6.07011442	1.196326	0.0009995	8	18	5.36446667	298.555556	25.55555555	0	0	0	1	34.0724638	0	0.49277013
3.0465	127906.139	1.02684601	301.710526	2.26774981	1	1.11399339	4.85130452	0.02746536	5.70947135	0.81922893	0.0009995	9	25	2.68223333	295.222222	22.2222222	0	0	0	1	34.0724638	0	0.49277013
3.5658	144422.632	0.9542249	259.819757	2.00482188	1	1.27138843	4.97275087	-0.0458085	5.559992	0.69605389	0.0009995	10	29	1.34111667	293	20	0	0	0	1	34.0724638	0	0.49277013
3.9852	158011.477	0.87030239	210.794226	1.54847658	1	1.3825875	5.062674	-0.1377662	5.35088717	0.43791719	0.0009995	11	31	1.34111667	290.777778	17.7777778	0	0	0	1	34.0724638	0	0.49277013
4.4829	170052.125	0.87181789	214.309858	2.13397978	1	1.50027016	5.13611089	-0.1360283	5.36742757	0.75845718	0.0009995	12	33	2.68223333	290.222222	17.2222222	0	0	0	1	34.0724638	0	0.49277013
4.9725	180768.895	0.80277903	179.993624	1.80828835	1	1.60392273	5.19722492	-0.2184309	5.19292698	0.59293359	0.0009995	13	35	3.12927222	290.222222	17.2222222	0	0	0	1	34.0724638	0	0.49277013
5.4693	194863.812	0.78271175	172.395126	1.5816186	1	1.69915064	5.27230605	-0.243714	5.14979489	0.45908082	0.0009995	14	36	3.57631111	290.222222	17.2222222	0	0	0	1	34.0724638	0	0.49277013
5.9832	210675.468	0.79061179	170.19129	1.61929626	1	1.78895554	5.35032363	-0.2336842	5.13692892	0.48260901	0.0009995	15	40	3.57631111	289.111111	16.11111111	0	0	0	1	34.0724638	0	0.49277013
6.471	225753.758	0.76108807	159.023985	1.56165249	1	1.86733066	5.41944927	-0.2716931	5.06906133	0.44638469	0.0009995	16	44	3.57631111	288	15	0	0	0	1	34.0724638	0	0.49277013
7.0353	243417.782	0.81289026	192.301311	1.83357981	1	1.95094033	5.49478334	-0.2059297	5.25906867	0.60681547	0.0009995	17	44	4.91742778	286.888889	13.88888888	0	0	0	1	34.0724638	0	0.49277013
7.6104	260488.805	0.75582986	167.159539	1.86172624	1	2.02951573	5.56256372	-0.2786168	5.11895466	0.62204113	0.0009995	18	51	4.02335	285.777778	12.77777777	0	0	0	1	34.0724638	0	0.49277013
7.9812	276279.522	0.84083216	202.061628	1.4930417	1	2.07708878	5.62141673	-0.1721746	5.30857769	0.401485	0.0009995	19	63	2.23519444	284.666667	11.66666666	0	0	0	1	34.0724638	0	0.49277013
8.2305	286730.644	0.82895217	182.735306	1.39427874	1	2.10784677	5.65854674	-0.1863872	5.20804416	0.33309421	0.0009995	20	71	2.23519444	284.666667	11.66666666	0	0	0	1	34.0724638	0	0.49277013
8.4438	295648.5	0.78611015	161.78425	1.28430433	1	2.13343244	5.68917463	-0.2393871	5.08626984	0.25099553	0.0009995	21	73	2.68223333	284.666667	11.66666666	0	0	0	1	34.0724638	0	0.49277013
8.6031	302305.125	0.7171499	136.063592	1.20107998	1	2.1521226	5.71144016	-0.331077	4.91312972	0.18405337	0.0009995	22	79	3.57631111	284.666667	11.66666666	0	0	0	1	34.0724638	0	0.49277013
8.6967	306472.191	0.59840664	89.6634991	0.88457738	1	2.16294364	5.72513028	-0.511815	4.49607492	-0.1215154	0.0009995	23	83	4.47038889	286.333333	13.33333333	0	0	0	1	34.0724638	0	0.49277013
0.0315	1916.22953	1.0822543	320.902229	2.14249128	0	-3.4577677	0.65088119	0.07996975	5.77113961	0.76243594	-6.9077553	0	94	1.34111667	276.888889	3.888888888	0	0	0	0	42.288408	0	-0.4111751
0.117	6386.86662	0.99214453	269.545724	2.13911213	0	-2.1455813	1.85440035	-0.0068791	5.59674175	0.76085823	-6.9077553	1	58	1.78815556	284.666667	11.66666666	0	0	0	0	42.288408	0	-0.4111751
0.3654	17427.0693	1.0947949	333.558169	3.31529855	0	-1.0067626	2.85808208	0.09148003	5.80982027	1.19884927	-6.9077553	2	36	3.12927222	290.222222	17.2222222	0	0	0	0	42.288408	0	-0.4111751
0.6606	32397.4944	1.05647473	303.933046	2.22807442	0	-0.4146068	3.47811195	0.05588374	5.71681072	0.80158644	-6.9077553	3	31	3.57631111	293.555556	20.55555555	0	0	0	0	42.288408	0	-0.4111751

1.0197	46953.489	0.97720008	257.448739	2.36060901	0	0.01950847	3.84917881	-0.022041	5.55082451	0.85934317	-6.9077553	4	22	5.36446667	296.333333	23.33333333	0	0	0	0	42.288408	0	-0.4111751
1.5912	71026.6124	1.08309586	338.309473	3.2570151	0	0.46448845	4.26306871	0.08074633	5.82396403	1.18111815	-6.9077553	5	20	5.81150556	297.444444	24.44444444	0	0	1	0	42.288408	0	-0.4111751
2.3571	108015.256	1.09424244	344.515974	2.85523666	0	0.85743205	4.68228173	0.09097725	5.84214336	1.0495049	-6.9077553	6	18	4.91742778	298.555556	25.55555555	0	0	1	0	42.288408	0	-0.4111751
3.1266	146245.516	1.12419141	369.928509	2.70012907	0	1.13994615	4.98529367	0.11795316	5.91331247	0.99366986	-6.9077553	7	16	5.36446667	299.111111	26.11111111	0	0	1	0	42.288408	0	-0.4111751
3.924	182132.326	1.10111321	369.95958	2.9612125	1	1.36711154	5.20473898	0.09722944	5.91339646	1.08593646	0.0009995	8	18	5.36446667	298.555556	25.55555555	0	0	1	0	42.288408	0	-0.4111751
4.5135	208393.626	0.96263148	275.651026	2.05237413	1	1.50707291	5.33943352	-0.0370463	5.61913929	0.71948436	0.0009995	9	25	2.68223333	295.222222	22.2222222	0	0	1	0	42.288408	0	-0.4111751
5.1138	233798.288	0.91943616	248.675084	1.98519527	1	1.63194277	5.454463	-0.0829076	5.51615118	0.68622088	0.0009995	10	29	1.34111667	293	20	0	0	1	0	42.288408	0	-0.4111751
5.6727	253466.637	0.83436906	207.212711	1.67667348	1	1.73566519	5.53523615	-0.1798817	5.33375068	0.517408	0.0009995	11	31	1.34111667	290.777778	17.7777778	0	0	1	0	42.288408	0	-0.4111751
6.0867	266877.99	0.77030096	176.280554	1.42868931	1	1.80610606	5.58679533	-0.2596766	5.17208245	0.35745715	0.0009995	12	33	2.68223333	290.222222	17.2222222	0	0	1	0	42.288408	0	-0.4111751
6.498	280445.055	0.78694109	192.662199	1.54108267	1	1.87149444	5.63638139	-0.2383319	5.26094358	0.43313389	0.0009995	13	35	3.12927222	290.222222	17.2222222	0	0	1	0	42.288408	0	-0.4111751
6.9093	291093.937	0.68569465	152.846092	1.39027295	1	1.93286833	5.67364946	-0.3758655	5.02943802	0.33021912	0.0009995	14	36	3.57631111	290.222222	17.2222222	0	0	1	0	42.288408	0	-0.4111751
7.3296	300916.412	0.6746472	146.079196	1.34874417	1	1.99192094	5.70683585	-0.3920842	4.98415576	0.29991507	0.0009995	15	40	3.57631111	289.111111	16.11111111	0	0	1	0	42.288408	0	-0.4111751
7.7139	308949.394	0.66214547	139.349797	1.32961586	1	2.0430239	5.73318073	-0.4107609	4.93699447	0.28564189	0.0009995	16	44	3.57631111	288	15	0	0	1	0	42.288408	0	-0.4111751
8.1702	317162.006	0.70921226	173.5962	1.87183171	1	2.10049339	5.75941586	-0.3421914	5.15673767	0.62745157	0.0009995	17	44	4.91742778	286.888889	13.88888888	0	0	1	0	42.288408	0	-0.4111751
8.5608	326071.775	0.76685618	208.188814	1.72137029	1	2.14719364	5.78712059	-0.2641528	5.33845023	0.54370142	0.0009995	18	51	4.02335	285.777778	12.77777777	0	0	1	0	42.288408	0	-0.4111751
8.7156	331198.681	0.71292416	156.838549	1.16298338	1	2.16511452	5.80272146	-0.3369785	5.0552233	0.15184807	0.0009995	19	63	2.23519444	284.666667	11.66666666	0	0	1	0	42.288408	0	-0.4111751
8.811	335288.405	0.78895677	164.193691	1.27027087	1	2.17600094	5.81499405	-0.2357771	5.10105286	0.24001708	0.0009995	20	71	2.23519444	284.666667	11.66666666	0	0	1	0	42.288408	0	-0.4111751
8.9316	340449.132	0.83414687	186.488398	1.52365522	1	2.18959555	5.83026866	-0.1801477	5.22837439	0.4217683	0.0009995	21	73	2.68223333	284.666667	11.66666666	0	0	1	0	42.288408	0	-0.4111751
9.0558	345439.148	0.79821357	182.761124	1.77050483	1	2.20340544	5.8448194	-0.2241271	5.20818544	0.57182937	0.0009995	22	79	3.57631111	284.666667	11.66666666	0	0	1	0	42.288408	0	-0.4111751
9.1224	348147.101	0.6807084	148.766074	1.48151398	1	2.21073293	5.85262797	-0.3831533	5.00238182	0.39373928	0.0009995	23	83	4.47038889	286.333333	13.33333333	0	0	1	0	42.288408	0	-0.4111751
0.0018	0	0.16748893	5.34041262	0.42339128	0	-6.3199686	-6.9077553	-1.7808852	1.67549015	-0.8570994	-6.9077553	0	94	1.34111667	276.888889	3.888888888	0	0	0	0	18.9135952	0	-0.0428739
0.0018	0	0	0	0	0	-6.3199686	-6.9077553	-6.9077553	-6.9077553	-6.9077553	-6.9077553	1	58	1.78815556	284.666667	11.66666666	0	0	0	0	18.9135952	0	-0.0428739
0.0252	0	0.4354755	51.5123385	1.57020672	0	-3.6809113	-6.9077553	-0.829023	3.94184077	0.45184393	-6.9077553	2	36	3.12927222	290.222222	17.2222222	0	0	0	0	18.9135952	0	-0.0428739
0.1584	91.6690391	0.64519903	113.503814	2.89360005	0	-1.8426318	-2.3787209	-0.4366477	4.73184525	1.06284695	-6.9077553	3	31	3.57631111	293.555556	20.55555555	0	0	1	0	18.9135952	0	-0.0428739
0.3852	4332.98215	0.7907423	228.094184	3.09972055	0	-0.9539926	1.46648679	-0.2335193	5.42976302	1.13163452	-6.9077553	4	22	5.36446667	296.333333	23.33333333	0	0	1	0	18.9135952	0	-0.0428739
0.6273	14239.1096	0.99737662	358.00212	2.78728545	0	-0.4663304	2.6560626	-0.0016247	5.8805417	1.02542687	-6.9077553	5	20	5.81150556	297.444444	24.44444444	0	0	1	0	18.9135952	0	-0.0428739
0.8451	19624.758	0.82276917	213.075333	1.81687271	0	-0.1683003	2.97684289	-0.1938649	5.36165047	0.59766698	-6.9077553	6	18	4.91742778	298.555556	25.55555555	0	0	1	0	18.9135952	0	-0.0428739
1.1565	31207.3977	1.00014765	319.210802	2.78044067	0	0.1453982	3.44068722	0.00114699	5.76585484	1.02296902	-6.9077553	7	16	5.36446667	299.111111	26.11111111	0	0	1	0	18.9135952	0	-0.0428739
1.5669	46089.8692	0.98011418	293.941749	2.61760618	0	0.44909915	3.83061487	-0.0190664	5.68338502	0.96264218	-6.9077553	8	18	5.36446667	298.555556	25.55555555	0	0	1	0	18.9135952	0	-0.0428739
1.8927	59259.4667	0.8957979	242.480706	1.77812035	0	0.63800438	4.08194242	-0.1089248	5.49092627	0.57611905	-6.9077553	9	25	2.68223333	295.222222	22.2222222	0	0	1	0	18.9135952	0	-0.0428739
2.2347	71527.2648	0.8161756	200.499959	1.63025384	0	0.80410699	4.27009268	-0.2019013	5.30081903	0.48934895	-6.9077553	10	29	1.34111667	293	20	0	0	1	0	18.9135952	0	-0.0428739
2.6928	89199.4151	0.87960102	233.422719	1.88841287	0	0.99058154	4.49088569	-0.1271506	5.45285534	0.63626613	-6.9077553	11	31	1.34111667	290.777778	17.7777778	0	0	1	0	18.9135952	0	-0.0428739
3.1221	105204.349	0.82056982	204.962756	1.80143396	0	1.13850585	4.65591415	-0.1965384	5.32283316	0.58913795	-6.9077553	12	33	2.68223333	290.222222	17.2222222	0	0	1	0	18.9135952	0	-0.0428739
3.5811	120475.583	0.81291354	200.261397	1.95940444	0	1.27567002	4.79145541	-0.2059011	5.29962849	0.6731508	-6.9077553	13	35	3.12927222	290.222222	17.2222222	0	0	1	0	18.9135952	0	-0.0428739
4.0059	135189.367	0.79279715	184.461666	1.60447977	0	1.38776827	4.90668391	-0.2309273	5.21744709	0.47342264	-6.9077553	14	36	3.57631111	290.222222	17.2222222	0	0	1	0	18.9135952	0	-0.0428739
4.4901	152316.829	0.81480232	196.153259	1.7810222	0	1.50187497	5.02596932	-0.2035832	5.27890138	0.57774878	-6.9077553	15	40	3.57631111	289.111111	16.11111111	0	0	1	0	18.9135952	0	-0.0428739
4.9545	170199.587	0.82377441	203.592312	1.78778123	0	1.60029625	5.13697767	-0.1926454	5.31612443	0.58153451	-6.9077553	16	44	3.57631111	288	15	0	0	1	0	18.9135952	0	-0.0428739
5.3892	183997.005	0.7367519	159.333447	1.66360227	0	1.68439695	5.21492492	-0.3041477	5.07100543	0.50958622	-6.9077553	17	44	4.91742778	286.888889	13.88888888	0	0	1	0	18.9135952	0	-0.0428739
5.7726	196523.972	0.71661649	147.523685	1.29862181	0	1.75312259	5.28078951	-0.33182	4.99399552	0.26207331	-6.9077553	18	51	4.02335	285.777778	12.77777777	0	0	1	0	18.9135952	0	-0.0428739
6.1191	211754.727	0.84562632	195.258766	1.38523472	0	1.81141503	5.35543338	-0.1664959	5.27433081	0.32659124	-6.9077553	19	63	2.23519444	284.666667	11.66666666	0	0	1	0	18.9135952	0	-0.0428739
6.4953	230308.022	0.94126461	238.400942	1.73279057	0	1.87107884	5.43942198	-0.0594691	5.47395808	0.55031009	-6.9077553	20	71	2.23519444	284.666667	11.66666666	0	0	1	0	18.9135952	0	-0.0428739
6.8805	249530.124	0.89081352	214.876314	1.6456912	0	1.92869132	5.51958365	-0.1144982	5.37006723	0.49876794	-6.9077553	21	73	2.68223333	284.666667	11.66666666	0	0	1	0	18.9135952	0	-0.0428739
7.1865	264165.144	0.80133048	171.38619	1.38435525	0	1.97220427	5.57657824	-0.2202347	5.14392527	0.32595661	-6.9077553	22	79	3.57631111	284.666667	11.66666666	0	0	1	0	18.9135952	0	-0.0428739
7.3584	271990.221	0.6435455	108.230469	1.05413595	0	1.99584252	5.60576979	-0.4392099	4.68427216	0.05366962	-6.9077553	23	83	4.47038889	286.333333	13.33333333	0	0	1	0	18.9135952	0	-0.0428739
0.0081	321.932985	0.83863604	189.677216	1.72050416	0	-4.8158912	-1.1303105	-0.1747868	5.24532903	0.54319842	-6.9077553	0	93	4.47038889	293.555556	20.55555555	0	0	0	0	30.519848	0	0.21141735
0.0108	453.524351	0.49128477	61.2883301	0.75739964	0	-4.5282091	-0.7885038	-0.7086979	4.11560577	-0.2765448	-6.9077553	1	92	2.68223333	293.555556	20.55555555	0	0	0	0	30.519848	0	0.21141735
0.0225	980.673628	0.58320918	90.3315524	1.05282541	0	-3.79424	-0.0184964	-0.5374962	4.50349789	0.0524268	-6.9077553	2	91	2.68223333	294.111111	21.11111111	0	0	0	0	30.519848	0	0.21141735
0.0477	1375.89494	0.46165609	69.0843201	1.09065914	0	-3.0428239	0.31983092	-0.7707713	4.23534226	0.08769869	-6.9077553	3	91	2.68223333	294.666667	21.66666666	0	0	0	0	30.519848	0	0.21141735
0.0918	1814.53274	0.36092922	36.3579351	1.03319853	0	-2.388143	0.59637894	-1.0163066	3.59343998	0.03362676	-6.9077553	4	85	3.57631111	296.333333	23.33333333	0	0	0	0	30.519848	0	0.21141735
0.1953	2644.27569	0.49366094	68.4375679	2.00256839	0	-1.6332184	0.97277529	-0.7038827	4.22593652	0.69492978	-6.9077553	5	71	5.36446667	298.555556	25.55555555	0	0	0	0	30.519848	0	0.21141735
0.3348	3664.00758	0.64745414	119.261542	2.44836445	0	-1.0942219	1.2988304	-0.433164	4.7813273	0.89582858	-6.9077553	6	67	5.81150556	299.666667	26.66666666	0	0	0	0	30.519848	0	0.21141735
0.495	7282.2689	0.76424537	168.74425	2.48763832	0	-0.7031975	1.98557979	-0.2675588	5.12839018	0.9117357	-6.9077553	7	55	6.25854444	301.888889	28.88888888	0	0	0	1	30.519848	0	0.21141735
0.7893	20655.2362	0.85416228	214.313623	2.84998753	0	-0.2366088	3.02801727	-0.156464	5.36744514	1.04766544	-6.9077553	8	50	5.81150556	303.555556	30.55555555	0	0	0	1	30.519848	0	0.21141735
1.098	29629.1466	0.72059912	145.673469	2.17909388	0	0.09349034	3.38879231	-0.3262855	4.98137447	0.77936794	-6.9077553	9	45	5.36446667	304.111111	31.11111111	0	0	0	1	30.519848	0	0.21141735
1.3572	38346.0722	0.70832464	144.683729	1.55921173	0	0.30542375	3.64667818	-0.343442	4.9745571	0.44482154	-6.9077553	10	46	3.57631111	304.111111	31.11111111	0	0	0	1	30.519848	0	0.21141735
1.5795	48339.6725	0.71063115	146.592912	1.36954786	0	0.45710834	3.87827329	-0.3401955	4.98766626	0.31521055	-6.9077553	11	44	4.02335	304.666667	31.66666666	0	0	0	1	30.519848	0	0.21141735
1.7748	59372.8923	0.69454401	140.692864	1.23326371	0	0.57368774	4.08385461	-0.363061	4.94658636	0.21047461	-6.9077553	12	49	3.12927222	303	30	0	0	0	1	30.519848	0	0.21141735
1.9125	69313.4709	0.64872418	122.94927	0.93970125	0	0.64841129	4.2386537	-0.4312074	4.81177996	-0.0611297	-6.9077553	13	53	3.12927222	301.333333	28.33333333	0	0	0	1	30.519848	0	0.21141735
2.0106	78008.7136	0.65839638	124.069193	0.85533982	0	0.69843318	4.35683335	-0.4164304	4.82084747	-0.155088	-6.9077553	14	58	1.78815556	299.111111	26.11111111	0	0	0	1	30.519848	0	0.21141735
2.1114	89179.1291	0.72077029	149.792794	0.98188332	0	0.74735123	4.49065825	-0.3260483	5.00925964	-0.0172649	-6.9077553	15	51	2.68223333	298.555556	25.55555555	0	0	0	1	30.519848	0	0.21141735
2.2509	100591.604	0.63276057	116.999975	0.89702778	0	0.81133014	4.61107873	-0.456084	4.76218227	-0.1075543	-6.9077553	16	53	2.23519444	297.444444	24.44444444	0	0	0	1	30.519848	0	0.21141735
2.3526	111534.822	0.72292632	151.520586	1.03816034	0	0.8555211	4.71434582	-0.3230657	5.02072809	0.03841302	-6.9077553	17	62	2.68223333	296.333333	23.33333333	0	0	0	1	30.519848	0	0.21141735

2.4147	120484.466	0.71172172	133.233384	1.02109362	0	0.88157506	4.79152913	-0.3386642	4.89210987	0.02185309	-6.9077553	18	73	3.12927222	295.777778	22.7777778	0	0	0	1	30.519848	0	0.21141735
2.4552	126916.97	0.53358574	75.6457086	0.65498331	0	0.89820822	4.84354097	-0.6262632	4.32607393	-0.4216199	-6.9077553	19	80	2.23519444	295.222222	22.2222222	0	0	0	1	30.519848	0	0.21141735
2.4597	127048.559	0.31248236	22.6140335	0.4649662	0	0.90003939	4.84457724	-1.1600122	3.11861488	-0.7636422	-6.9077553	20	84	3.12927222	295.222222	22.2222222	0	0	0	1	30.519848	0	0.21141735
2.4624	127092.422	0.2063609	8.44528135	0.23621901	0	0.90113648	4.84492243	-1.5732945	2.13372627	-1.4387715	-6.9077553	21	88	2.23519444	294.666667	21.66666666	0	0	0	1	30.519848	0	0.21141735
2.466	127180.149	0.16002184	6.56729031	0.54118437	0	0.9025974	4.84561244	-1.8262153	1.88225357	-0.6121492	-6.9077553	22	90	2.23519444	294.666667	21.66666666	0	0	0	1	30.519848	0	0.21141735
2.4678	127224.012	0.19964334	7.90138245	0.24374872	0	0.90332707	4.84595727	-1.6062264	2.06716429	-1.4075232	-6.9077553	23	89	3.12927222	295.222222	22.2222222	0	0	0	1	30.519848	0	0.21141735
0.0162	713.495356	1.01237286	286.872616	2.17124243	0	-4.122744	-0.3361788	0.01328423	5.65904176	0.77576001	-6.9077553	0	93	4.47038889	293.555556	20.55555555	0	0	0	0	34.0724638	0	0.49277013
0.0315	1327.58829	0.45674905	54.017334	0.89364271	0	-3.4577677	0.28411694	-0.7814342	3.98932351	-0.1113309	-6.9077553	1	92	2.68223333	293.555556	20.55555555	0	0	0	0	34.0724638	0	0.49277013
0.0459	1766.22605	0.44351926	45.2685165	0.60408843	0	-3.0812902	0.56941111	-0.8107619	3.81263388	-0.5023807	-6.9077553	2	91	2.68223333	294.111111	21.11111111	0	0	0	0	34.0724638	0	0.49277013
0.0684	2511.91036	0.54294899	78.2006689	1.19149567	0	-2.6823825	0.92144159	-0.6088998	4.35929099	0.17604831	-6.9077553	3	91	2.68223333	294.666667	21.66666666	0	0	0	0	34.0724638	0	0.49277013
0.1278	5008.71116	0.75421825	154.370798	1.39598268	0	-2.0572887	1.61137826	-0.2807485	5.03936396	0.33431469	-6.9077553	4	85	3.57631111	296.333333	23.33333333	0	0	0	0	34.0724638	0	0.49277013
0.1935	8172.29496	0.88603973	208.104532	1.43183178	0	-1.6424778	2.10087213	-0.1198655	5.33804532	0.35965275	-6.9077553	5	71	5.36446667	298.555556	25.55555555	0	0	0	0	34.0724638	0	0.49277013
0.3807	15662.2241	1.10783584	376.416316	3.12115156	0	-0.9657436	2.75131555	0.10331067	5.93069841	1.13852237	-6.9077553	6	67	5.81150556	299.666667	26.66666666	0	0	0	1	34.0724638	0	0.49277013
1.0485	32947.3856	1.56126285	780.298096	6.08740563	0	0.04736057	3.49494226	0.44613531	6.6596773	1.80638625	-6.9077553	7	55	6.25854444	301.888889	28.88888888	0	0	0	1	34.0724638	0	0.49277013
1.881	58243.0253	1.31519426	511.939865	4.45676362	0	0.63180355	4.06464152	0.27474443	6.23820912	1.49464721	-6.9077553	8	50	5.81150556	303.555556	30.55555555	0	0	0	1	34.0724638	0	0.49277013
2.5047	79291.3542	1.08236655	342.372249	2.75540949	1	0.91816897	4.37314171	0.08007337	5.83590151	1.01392892	0.0009995	9	45	5.36446667	304.111111	31.11111111	0	0	0	1	34.0724638	0	0.49277013
3.0051	96930.2567	0.93235318	239.187078	1.80380578	1	1.10031085	4.57400203	-0.0689716	5.47725018	0.59045299	0.0009995	10	46	3.57631111	304.111111	31.11111111	0	0	0	1	34.0724638	0	0.49277013
3.7269	121386.703	0.99543644	277.811039	2.86648248	1	1.31557679	4.79898958	-0.0035699	5.62694477	1.05343446	0.0009995	11	44	4.02335	304.666667	31.66666666	0	0	0	1	34.0724638	0	0.49277013
4.329	140883.852	0.8677755	215.074178	2.04485473	1	1.46533657	4.9479429	-0.1406705	5.37098763	0.71581566	0.0009995	12	49	3.12927222	303	30	0	0	0	1	34.0724638	0	0.49277013
4.7916	153491.891	0.7552381	168.15704	1.7410859	1	1.56686438	5.03365426	-0.279399	5.12490425	0.55508319	0.0009995	13	53	3.12927222	301.333333	28.33333333	0	0	0	1	34.0724638	0	0.49277013
5.1309	164965.1	0.7716712	178.051807	1.33900431	1	1.63528108	5.10574	-0.2579017	5.18208017	0.29267283	0.0009995	14	58	1.78815556	299.111111	26.11111111	0	0	0	1	34.0724638	0	0.49277013
5.4108	173667.349	0.65766347	141.248556	1.16800771	1	1.68839696	5.15714744	-0.4175425	4.95052822	0.15615527	0.0009995	15	51	2.68223333	298.555556	25.55555555	0	0	0	1	34.0724638	0	0.49277013
5.7888	184747.046	0.67803425	148.95506	1.24384402	1	1.75592502	5.21899299	-0.3870837	5.00365136	0.21901024	0.0009995	16	53	2.23519444	297.444444	24.44444444	0	0	0	1	34.0724638	0	0.49277013
6.0201	193418.312	0.72328988	153.034959	1.16239686	1	1.79510387	5.26486043	-0.3225636	5.03067292	0.15134405	0.0009995	17	62	2.68223333	296.333333	23.33333333	0	0	0	1	34.0724638	0	0.49277013
6.1956	200459.444	0.75589662	153.590375	1.27353062	1	1.82383936	5.30061694	-0.2785286	5.03429566	0.24257797	0.0009995	18	73	3.12927222	295.777778	22.7777778	0	0	0	1	34.0724638	0	0.49277013
6.3324	206082.475	0.64265131	108.364412	1.0927888	1	1.84567931	5.32828131	-0.4405981	4.68550896	0.08964763	0.0009995	19	80	2.23519444	295.222222	22.2222222	0	0	0	1	34.0724638	0	0.49277013
6.3765	207924.752	0.29568172	20.2148736	0.54405991	1	1.85261936	5.33718105	-1.2150954	3.00646812	-0.6068596	0.0009995	20	84	3.12927222	295.222222	22.2222222	0	0	0	1	34.0724638	0	0.49277013
6.4233	209986.348	0.22077355	10.4069214	0.36484784	1	1.859932	5.34704728	-1.5060985	2.34256719	-1.0055378	0.0009995	21	88	2.23519444	294.666667	21.66666666	0	0	0	1	34.0724638	0	0.49277013
6.4611	211653.178	0.18327967	6.86769685	0.24998876	1	1.86579958	5.35495371	-1.6913007	1.9269744	-1.3823471	0.0009995	22	90	2.23519444	294.666667	21.66666666	0	0	0	1	34.0724638	0	0.49277013
6.4917	212969.1	0.18617885	7.0820142	0.2882967	1	1.87052444	5.36115178	-1.6756907	1.95769955	-1.2403025	0.0009995	23	89	3.12927222	295.222222	22.2222222	0	0	0	1	34.0724638	0	0.49277013
0.0369	2412.22585	1.13732729	361.002466	2.50996666	0	-3.2995437	0.88096438	0.1295599	5.88888756	0.9206678	-6.9077553	0	93	4.47038889	293.555556	20.55555555	0	0	0	0	42.288408	0	-0.4111751
0.0783	4435.7082	0.62900705	103.166642	1.15738686	0	-2.5472077	1.48991271	-0.4620243	4.63635526	0.1470284	-6.9077553	1	92	2.68223333	293.555556	20.55555555	0	0	0	0	42.288408	0	-0.4111751
0.1242	6587.07276	0.61738583	98.0304936	1.10980329	0	-2.0858621	1.88526086	-0.4806427	4.58528879	0.10508344	-6.9077553	2	91	2.68223333	294.111111	21.11111111	0	0	0	0	42.288408	0	-0.4111751
0.2691	11730.0729	0.7823239	173.644725	2.02621631	0	-1.3126722	2.46224113	-0.244209	5.15701716	0.70666357	-6.9077553	3	91	2.68223333	294.666667	21.66666666	0	0	0	0	42.288408	0	-0.4111751
0.5094	22675.2686	0.92584046	241.68712	2.09745417	0	-0.6745217	3.12131894	-0.0759738	5.48764813	0.74120096	-6.9077553	4	85	3.57631111	296.333333	23.33333333	0	0	0	0	42.288408	0	-0.4111751
0.8028	34376.2991	0.88546622	211.312716	2.06653843	0	-0.2196497	3.53739644	-0.1205123	5.35334383	0.72635874	-6.9077553	5	71	5.36446667	298.555556	25.55555555	0	0	0	0	42.288408	0	-0.4111751
1.1484	46959.7982	0.85752376	197.392782	1.98807144	0	0.13836967	3.84931317	-0.1525409	5.28520062	0.68766792	-6.9077553	6	67	5.81150556	299.666667	26.66666666	0	0	1	0	42.288408	0	-0.4111751
1.6245	64627.4728	0.89672673	226.642663	2.47739415	0	0.48520008	4.16865507	-0.1078896	5.42337902	0.90761083	-6.9077553	7	55	6.25854444	301.888889	28.88888888	0	0	1	0	42.288408	0	-0.4111751
2.295	92228.7628	0.99560211	284.470344	3.05206651	0	0.83073284	4.52428289	-0.0034037	5.65063252	1.1161465	-6.9077553	8	50	5.81150556	303.555556	30.55555555	0	0	1	0	42.288408	0	-0.4111751
3.0186	128184.723	1.04351974	315.583664	2.50305722	0	1.10479315	4.85348017	0.0435572	5.75442699	0.91791231	-6.9077553	9	45	5.36446667	304.111111	31.11111111	0	0	1	0	42.288408	0	-0.4111751
3.7638	163855.992	1.02092385	294.924177	2.34708821	0	1.32542909	5.09899404	0.02168698	5.68672169	0.85360147	-6.9077553	10	46	3.57631111	304.111111	31.11111111	0	0	1	0	42.288408	0	-0.4111751
4.5225	199351.447	1.00255269	289.659883	2.29378834	0	1.50906494	5.29507435	0.00354639	5.66871087	0.83064061	-6.9077553	11	44	4.02335	304.666667	31.66666666	0	0	1	0	42.288408	0	-0.4111751
5.0436	220814.391	0.85554839	208.277229	1.55716896	0	1.61812011	5.39732702	-0.1548445	5.33887483	0.44351139	-6.9077553	12	49	3.12927222	303	30	0	0	1	0	42.288408	0	-0.4111751
5.4918	240625.325	0.85181013	208.882938	1.55626731	0	1.70325607	5.48324521	-0.1592183	5.34177878	0.44293256	-6.9077553	13	53	3.12927222	301.333333	28.33333333	0	0	1	0	42.288408	0	-0.4111751
5.8365	254380.991	0.7923592	178.09377	1.29333799	0	1.7641313	5.53883704	-0.2314792	5.18231583	0.25799936	-6.9077553	14	58	1.78815556	299.111111	26.11111111	0	0	1	0	42.288408	0	-0.4111751
6.1092	264023.112	0.7664172	170.596096	1.24222537	0	1.80979583	5.57604043	-0.2647247	5.13930461	0.21770911	-6.9077553	15	51	2.68223333	298.555556	25.55555555	0	0	1	0	42.288408	0	-0.4111751
6.4116	273280.904	0.67289148	148.145264	1.14917891	0	1.85810885	5.61050388	-0.3946862	4.99820005	0.13991751	-6.9077553	16	53	2.23519444	297.444444	24.44444444	0	0	1	0	42.288408	0	-0.4111751
6.5754	279529.611	0.78075157	172.746952	1.24676774	0	1.88333541	5.63311181	-0.2462183	5.15183361	0.22135614	-6.9077553	17	62	2.68223333	296.333333	23.33333333	0	0	1	0	42.288408	0	-0.4111751
6.6636	283405.815	0.76101638	151.026666	1.16149381	0	1.89665988	5.64688337	-0.2717872	5.01746304	0.15056753	-6.9077553	18	73	3.12927222	295.777778	22.7777778	0	0	1	0	42.288408	0	-0.4111751
6.7392	286301.279	0.61428987	110.165144	1.09596407	0	1.90794122	5.65704817	-0.4856618	4.70198962	0.09254643	-6.9077553	19	80	2.23519444	295.222222	22.2222222	0	0	1	0	42.288408	0	-0.4111751
6.7635	287310.14	0.29353228	31.8906431	0.6748032	0	1.91154051	5.66056574	-1.2223667	3.462344	-0.3918534	-6.9077553	20	84	3.12927222	295.222222	22.2222222	0	0	1	0	42.288408	0	-0.4111751
6.7914	288538.327	0.22728482	11.1621773	0.40033036	0	1.91565711	5.66483139	-1.4771612	2.41262062	-0.9129704	-6.9077553	21	88	2.23519444	294.666667	21.66666666	0	0	1	0	42.288408	0	-0.4111751
6.8166	289459.483	0.21416581	10.0743757	0.41740196	0	1.91936081	5.66801879	-1.5363464	2.3100944	-0.8713127	-6.9077553	22	90	2.23519444	294.666667	21.66666666	0	0	1	0	42.288408	0	-0.4111751
6.8373	290161.305	0.17981419	6.84740017	0.30899678	0	1.92239292	5.67044044	-1.7102853	1.92401507	-1.1711934	-6.9077553	23	89	3.12927222	295.222222	22.2222222	0	0	1	0	42.288408	0	-0.4111751
0.0018	0	0.45057252	45.9067039	2.08759046	0	-6.3199686	-6.9077553	-0.7950193	3.82663295	0.73648942	-6.9077553	0	93	4.47038889	293.555556	20.55555555	0	0	0	0	18.9135952	0	-0.0428739
0.0018	0	0	0	0	0	-6.3199686	-6.9077553	-6.9077553	-6.9077553	-6.9077553	-6.9077553	1	92	2.68223333	293.555556	20.55555555	0	0	0	0	18.9135952	0	-0.0428739
0.0018	0	0	0	0	0	-6.3199686	-6.9077553	-6.9077553	-6.9077553	-6.9077553	-6.9077553	2	91	2.68223333	294.111111	21.11111111	0	0	0	0	18.9135952	0	-0.0428739
0.0018	0	0	0	0	0	-6.3199686	-6.9077553	-6.9077553	-6.9077553	-6.9077553	-6.9077553	3	91	2.68223333	294.666667	21.66666666	0	0	0	0	18.9135952	0	-0.0428739
0.0072	0	0.29284527	24.8875732	1.16351612	0	-4.9336743	-6.9077553	-1.2247019	3.21440879	0.15230566	-6.9077553	4	85	3.57631111	296.333333	23.33333333	0	0	0	0	18.9135952	0	-0.0428739
0.0756	194.594311	0.59383212	101.049406	2.46385153	0	-2.582299	-1.6317126	-0.5194761	4.61561947	0.90213157	-6.9077553	5	71	5.36446667	298.555556	25.55555555	0	0	0	0	18.9135952	0	-0.0428739
0.2871	6418.43315	0.87553873	258.19011	2.81247714	0	-1.2479247	1.85932982	-0.1317744	5.55370005	1.03442114	-6.9077553	6	67	5.81150556	299.666667	26.66666666	0	0	1	0	18.9135952	0	-0.0428739
0.5337	13717.9055	0.85687072	255.111941	2.69542098	0	-0.6279214	2.61877485	-0.1533019	5.54170635	0.99192533	-6.9077553	7	55	6.25854444	301.888889	28.88888888	0	0	1	0	18.9135952	0	-0.0428739

0.7344	18602.0072	0.77967684	195.43228	2.03321544	0	-0.3087014	2.92332324	-0.247594	5.27521904	0.71011021	-6.9077553	8	50	5.81150556	303.555556	30.55555555	0	0	1	0	18.9135952	0	-0.0428739
0.9981	28445.3681	0.84945043	228.788343	1.90374652	0	-0.0019018	3.34802049	-0.1619891	5.43280168	0.64434894	-6.9077553	9	45	5.36446667	304.111111	31.11111111	0	0	1	0	18.9135952	0	-0.0428739
1.3104	38974.1004	0.84943401	215.930656	2.11161466	0	0.27033243	3.66292299	-0.1620085	5.37496195	0.74792635	-6.9077553	10	46	3.57631111	304.111111	31.11111111	0	0	1	0	18.9135952	0	-0.0428739
1.6182	51888.0897	0.9039262	243.228436	1.90877778	0	0.48131442	3.94910855	-0.0999019	5.49400518	0.64698689	-6.9077553	11	44	4.02335	304.666667	31.66666666	0	0	1	0	18.9135952	0	-0.0428739
1.8855	62016.026	0.82935228	199.921875	1.60560233	0	0.63419304	4.12740896	-0.1859052	5.29793167	0.47412159	-6.9077553	12	49	3.12927222	303	30	0	0	1	0	18.9135952	0	-0.0428739
2.2176	75889.9532	0.86534057	222.234502	1.80330139	0	0.79642553	4.32929748	-0.1434772	5.40373764	0.59017348	-6.9077553	13	53	3.12927222	301.333333	28.33333333	0	0	1	0	18.9135952	0	-0.0428739
2.511	89058.7133	0.87697288	220.554927	1.58197377	0	0.92068108	4.48930708	-0.1301396	5.3961513	0.45930521	-6.9077553	14	58	1.78815556	299.111111	26.11111111	0	0	1	0	18.9135952	0	-0.0428739
2.7567	99121.973	0.79978345	189.793441	1.50575731	0	1.01403431	4.59636123	-0.2221647	5.2459416	0.40995987	-6.9077553	15	51	2.68223333	298.555556	25.55555555	0	0	1	0	18.9135952	0	-0.0428739
3.0996	111267.878	0.75831624	180.917282	1.63075909	0	1.13127307	4.7119496	-0.2753369	5.19804545	0.48965863	-6.9077553	16	53	2.23519444	297.444444	24.44444444	0	0	1	0	18.9135952	0	-0.0428739
3.3435	122658.131	0.89344703	224.480656	1.6447308	0	1.20701816	4.80940922	-0.1115496	5.41379399	0.49818454	-6.9077553	17	62	2.68223333	296.333333	23.33333333	0	0	1	0	18.9135952	0	-0.0428739
3.5487	132609.942	0.90473349	225.483553	1.73350605	0	1.26658134	4.8874196	-0.0990102	5.41825165	0.55072268	-6.9077553	18	73	3.12927222	295.777778	22.7777778	0	0	1	0	18.9135952	0	-0.0428739
3.6675	137007.727	0.69523696	129.934925	1.27770141	0	1.29951023	4.92004462	-0.3620652	4.86704145	0.24584504	-6.9077553	19	80	2.23519444	295.222222	22.2222222	0	0	1	0	18.9135952	0	-0.0428739
3.7044	138542.953	0.30344828	27.1660275	0.56180619	0	1.3095213	4.93118763	-1.1892541	3.30200401	-0.57482	-6.9077553	20	84	3.12927222	295.222222	22.2222222	0	0	1	0	18.9135952	0	-0.0428739
3.7341	139727.269	0.23066581	11.7176708	0.36537722	0	1.31750683	4.9396996	-1.4624594	2.46118336	-1.0040918	-6.9077553	21	88	2.23519444	294.666667	21.66666666	0	0	1	0	18.9135952	0	-0.0428739
3.7593	140911.586	0.21410808	9.76948983	0.32291899	0	1.32423277	4.94813974	-1.5366147	2.2793666	-1.1272618	-6.9077553	22	90	2.23519444	294.666667	21.66666666	0	0	1	0	18.9135952	0	-0.0428739
3.7728	141525.682	0.19564896	7.68715464	0.24248536	0	1.32781743	4.95248827	-1.6263351	2.03968079	-1.4126984	-6.9077553	23	89	3.12927222	295.222222	22.2222222	0	0	1	0	18.9135952	0	-0.0428739
0.0072	225.354111	0.94741453	245.4149	1.73939419	0	-4.9336743	-1.4856546	-0.0529636	5.50295432	0.55411163	-6.9077553	0	76	3.12927222	297.444444	24.44444444	0	0	0	0	30.519848	0	0.21141735
0.0117	444.673008	0.41652408	40.4067017	0.51698303	0	-4.4481664	-0.8081698	-0.8734131	3.6990204	-0.6578128	-6.9077553	1	79	3.12927222	296.888889	23.88888888	0	0	0	0	30.519848	0	0.21141735
0.0153	576.264374	0.35599366	28.6836624	0.49473536	0	-4.1799025	-0.5494549	-1.0300373	3.35636257	-0.701713	-6.9077553	2	82	2.68223333	296.333333	23.33333333	0	0	0	0	30.519848	0	0.21141735
0.0207	839.447159	0.25933675	13.9492404	0.33534316	0	-3.8776216	-0.1738212	-1.3457793	2.63549674	-1.0896233	-6.9077553	3	86	2.23519444	295.777778	22.7777778	0	0	0	0	30.519848	0	0.21141735
0.0243	971.038496	0.28328782	16.8140984	0.42333922	0	-3.7172789	-0.0283599	-1.2577681	2.82227719	-0.8572221	-6.9077553	4	86	2.68223333	296.333333	23.33333333	0	0	0	0	30.519848	0	0.21141735
0.0315	1102.62973	0.24240822	13.119339	0.43809712	0	-3.4577677	0.0986045	-1.4130153	2.57416362	-0.8230347	-6.9077553	5	86	1.34111667	296.333333	23.33333333	0	0	0	0	30.519848	0	0.21141735
0.036	1278.08496	0.19236407	7.96016388	0.33566239	0	-3.3242363	0.24614494	-1.6431805	2.07457521	-1.0886747	-6.9077553	6	86	2.23519444	296.333333	23.33333333	0	0	0	0	30.519848	0	0.21141735
0.0378	1321.94865	0.15606046	5.89998817	0.45682842	0	-3.2754462	0.27986307	-1.8511245	1.77511982	-0.7812608	-6.9077553	7	87	1.34111667	296.333333	23.33333333	0	0	0	0	30.519848	0	0.21141735
0.0405	1365.81248	0.17056334	6.3067557	0.30221482	0	-3.2064533	0.31248137	-1.7628028	1.84177994	-1.1933137	-6.9077553	8	88	0.89407778	295.777778	22.7777778	0	0	0	0	30.519848	0	0.21141735
0.0405	1365.81248	0	0	0	0	-3.2064533	0.31248137	-6.9077553	-6.9077553	-6.9077553	-6.9077553	9	88	2.23519444	295.777778	22.7777778	0	0	0	0	30.519848	0	0.21141735
0.0405	1365.81248	0	0	0	0	-3.2064533	0.31248137	-6.9077553	-6.9077553	-6.9077553	-6.9077553	10	88	2.68223333	295.777778	22.7777778	0	0	0	0	30.519848	0	0.21141735
0.0477	1585.1314	0.31207153	25.5005035	0.82470107	0	-3.0428239	0.46129797	-1.1613236	3.23873741	-0.1915225	-6.9077553	11	86	2.68223333	296.333333	23.33333333	0	0	0	0	30.519848	0	0.21141735
0.0855	1716.72278	0.28734763	22.6315249	1.07764716	0	-2.4592389	0.54099945	-1.2435885	3.11938803	0.07570763	-6.9077553	12	82	3.57631111	296.888889	23.88888888	0	0	0	0	30.519848	0	0.21141735
0.1404	2168.77627	0.37568962	42.1115883	1.11319307	0	-1.9632598	0.77462406	-0.9763337	3.7403467	0.10813044	-6.9077553	13	75	3.57631111	298.555556	25.55555555	0	0	0	0	30.519848	0	0.21141735
0.243	3141.41543	0.51714449	73.6627176	1.74154998	0	-1.4146938	1.14499175	-0.6575011	4.29951038	0.55534954	-6.9077553	14	69	4.47038889	299.111111	26.11111111	0	0	0	0	30.519848	0	0.21141735
0.3816	4188.40165	0.64405501	116.672839	2.16612125	0	-0.9633823	1.43255792	-0.4384197	4.75938234	0.77339967	-6.9077553	15	64	4.91742778	300.777778	27.7777778	0	0	0	1	30.519848	0	0.21141735
0.6102	8702.02275	0.84127573	209.588106	2.97545978	0	-0.4939685	2.16367041	-0.1716478	5.34514898	1.0907346	-6.9077553	16	61	4.47038889	301.333333	28.33333333	0	0	0	1	30.519848	0	0.21141735
0.8892	14357.5799	0.80733249	187.772681	2.8296113	0	-0.1174331	2.66434767	-0.2127818	5.23523742	1.0404927	-6.9077553	17	59	4.47038889	301.333333	28.33333333	0	0	0	1	30.519848	0	0.21141735
1.1475	24856.7219	0.75779065	162.670541	1.78160781	0	0.13758566	3.21316844	-0.2760294	5.09173308	0.57807735	-6.9077553	18	54	6.25854444	301.888889	28.88888888	0	0	0	1	30.519848	0	0.21141735
1.3608	34082.7311	0.70257066	151.366413	1.44211949	0	0.30807276	3.52882018	-0.351587	5.01971008	0.36680708	-6.9077553	19	54	4.02335	301.888889	28.88888888	0	0	0	1	30.519848	0	0.21141735
1.5003	40828.4443	0.63666894	122.867603	1.15267472	0	0.40566509	3.7094035	-0.449936	4.81111552	0.14295225	-6.9077553	20	54	3.12927222	301.888889	28.88888888	0	0	0	1	30.519848	0	0.21141735
1.5921	46764.5317	0.64476806	122.112764	0.90123996	0	0.4650539	3.84514643	-0.4373149	4.8049531	-0.1028748	-6.9077553	21	55	3.12927222	300.777778	27.7777778	0	0	0	1	30.519848	0	0.21141735
1.6542	52376.2962	0.74739782	147.058693	0.96017887	0	0.50331751	3.95847322	-0.2898206	4.99083858	-0.0395948	-6.9077553	22	62	2.68223333	299.666667	26.66666666	0	0	0	1	30.519848	0	0.21141735
1.71	58266.1753	0.69739133	126.912716	0.90625849	0	0.53649337	4.0650389	-0.3589757	4.84350745	-0.0973279	-6.9077553	23	63	3.12927222	298.555556	25.55555555	0	0	0	1	30.519848	0	0.21141735
0.0135	580.611009	0.9674293	248.952323	1.70266928	0	-4.3050656	-0.5419534	-0.0320798	5.51726542	0.53278433	-6.9077553	0	76	3.12927222	297.444444	24.44444444	0	0	0	0	34.0724638	0	0.49277013
0.0225	931.521281	0.45650482	48.5060211	0.71196995	0	-3.79424	-0.0698633	-0.7819679	3.88170855	-0.338316	-6.9077553	1	79	3.12927222	296.888889	23.88888888	0	0	0	0	34.0724638	0	0.49277013
0.027	1063.11263	0.38584414	33.2255127	0.55446539	0	-3.6119184	0.06214124	-0.9497334	3.50334813	-0.587949	-6.9077553	2	82	2.68223333	296.333333	23.33333333	0	0	0	0	34.0724638	0	0.49277013
0.0342	1370.15918	0.21973462	9.72495842	0.37433431	0	-3.3755296	0.3156565	-1.5107941	2.27479844	-0.9799382	-6.9077553	3	86	2.23519444	295.777778	22.7777778	0	0	0	0	34.0724638	0	0.49277013
0.0387	1545.61419	0.18574932	7.46759109	0.46555185	0	-3.2519157	0.43606815	-1.6779881	2.01070637	-0.7623861	-6.9077553	4	86	2.68223333	296.333333	23.33333333	0	0	0	0	34.0724638	0	0.49277013
0.0423	1633.34171	0.21658568	9.89876747	0.33695939	0	-3.1629682	0.4912401	-1.5251626	2.29251127	-1.0848295	-6.9077553	5	86	1.34111667	296.333333	23.33333333	0	0	0	0	34.0724638	0	0.49277013
0.0486	1940.38833	0.24605651	12.5316206	0.36468547	0	-3.0241317	0.66340335	-1.3981382	2.52833489	-1.0059817	-6.9077553	6	86	2.23519444	296.333333	23.33333333	0	0	0	0	34.0724638	0	0.49277013
0.0567	2247.43476	0.2106197	9.63039907	0.33030997	0	-2.8699811	0.81023431	-1.5529645	2.2650285	-1.1047009	-6.9077553	7	87	1.34111667	296.333333	23.33333333	0	0	0	0	34.0724638	0	0.49277013
0.0603	2422.88984	0.23694383	12.0561934	0.35239872	0	-2.8084232	0.88537362	-1.4357207	2.48966144	-1.0401583	-6.9077553	8	88	0.89407778	295.777778	22.7777778	0	0	0	0	34.0724638	0	0.49277013
0.0675	2729.93647	0.20747319	8.70946312	0.26382694	0	-2.6956277	1.00464458	-1.5679448	2.16452496	-1.3286787	-6.9077553	9	88	2.23519444	295.777778	22.7777778	0	0	0	0	34.0724638	0	0.49277013
0.0729	2993.11894	0.21618364	9.38001696	0.28561304	0	-2.6186666	1.09665001	-1.527012	2.23868818	-1.2496223	-6.9077553	10	88	2.68223333	295.777778	22.7777778	0	0	0	0	34.0724638	0	0.49277013
0.0792	3256.30184	0.20874408	8.71983392	0.25973875	0	-2.535779	1.1808992	-1.5618671	2.16571487	-1.3442363	-6.9077553	11	86	2.68223333	296.333333	23.33333333	0	0	0	0	34.0724638	0	0.49277013
0.1116	4709.27058	0.66377825	124.601305	0.99081188	0	-2.1928342	1.54974535	-0.4083017	4.8251271	-0.0082218	-6.9077553	12	82	3.57631111	296.888889	23.88888888	0	0	0	0	34.0724638	0	0.49277013
0.2304	9443.71806	0.94728718	270.350586	2.24084796	0	-1.4679384	2.24545565	-0.0530979	5.59972328	0.80730051	-6.9077553	13	75	3.57631111	298.555556	25.55555555	0	0	0	0	34.0724638	0	0.49277013
0.5328	17615.6878	1.1827933	411.519671	3.39437648	0	-0.6296092	2.86884662	0.16872394	6.01985925	1.22241465	-6.9077553	14	69	4.47038889	299.111111	26.11111111	0	0	0	0	34.0724638	0	0.49277013
1.2357	27622.2257	1.45001103	668.693342	5.25542424	0	0.21163761	3.31865693	0.37226058	6.50532707	1.65945099	-6.9077553	15	64	4.91742778	300.777778	27.7777778	0	0	0	0	34.0724638	0	0.49277013
1.8432	42589.6444	1.11184995	360.528079	2.96795862	3	0.61150319	3.75163461	0.10692425	5.88757262	1.08821126	1.09894557	16	61	4.47038889	301.333333	28.33333333	0	0	0	1	34.0724638	0	0.49277013
2.4147	60125.1471	0.996256	279.68312	2.46856103	3	0.88157506	4.09644481	-0.0027478	5.63366082	0.90404041	1.09894557	17	59	4.47038889	301.333333	28.33333333	0	0	0	1	34.0724638	0	0.49277013
2.898	78405.3996	0.94314172	253.184619	2.0604514	3	1.06402084	4.36190555	-0.057479	5.53412289	0.7234103	1.09894557	18	54	6.25854444	301.888889	28.88888888	0	0	0	1	34.0724638	0	0.49277013
3.6765	101887.456	0.96991821	262.598555	2.8994039	3	1.30196121	4.62387865	-0.0295131	5.57063027	1.06485	1.09894557	19	54	4.02335	301.888889	28.88888888	0	0	0	1	34.0724638	0	0.49277013
4.3578	119228.173	0.81343576	194.49773	2.08334742	3	1.47196734	4.78104746	-0.2052597	5.27042563	0.73445582	1.09894557	20	54	3.12927222	301.888889	28.88888888	0	0	0	1	34.0724638	0	0.49277013
4.7853	131649.866	0.76423738	171.754737	1.56186344	3	1.56554872	4.88015346	-0.2675692	5.14607333	0.44651967	1.09894557	21	55	3.12927222	300.777778	27.7777778	0	0	0	1	34.0724638	0	0.49277013

5.0598	143552.212	0.83300981	190.494057	1.36867476	3	1.62132696	4.96670578	-0.1815101	5.24962625	0.31457331	1.09894557	22	62	2.68223333	299.666667	26.66666666	0	0	0	1	34.0724638	0	0.49277013
5.3055	154380.294	0.85639283	198.088341	1.52930593	3	1.66874402	5.03942548	-0.1538591	5.28871815	0.42546767	1.09894557	23	63	3.12927222	298.555556	25.55555555	0	0	0	1	34.0724638	0	0.49277013
0.0252	1374.101	0.9889768	265.171694	1.85725869	0	-3.6809113	0.31852718	-0.0100738	5.58038129	0.61963986	-6.9077553	0	76	3.12927222	297.444444	24.44444444	0	0	0	0	42.288408	0	-0.4111751
0.0576	2954.11354	0.60615269	93.362264	1.11967553	0	-2.8542327	1.08353707	-0.498975	4.53649795	0.11393166	-6.9077553	1	79	3.12927222	296.888889	23.88888888	0	0	0	0	42.288408	0	-0.4111751
0.0792	4006.84357	0.39535256	36.5117747	0.65897489	0	-2.535779	1.38825333	-0.9254512	3.59766219	-0.4155535	-6.9077553	2	82	2.68223333	296.333333	23.33333333	0	0	0	0	42.288408	0	-0.4111751
0.1044	5235.03016	0.27456333	16.5595463	0.46547624	0	-2.2595256	1.65556361	-1.2889378	2.80702314	-0.7625482	-6.9077553	3	86	2.23519444	295.777778	22.7777778	0	0	0	0	42.288408	0	-0.4111751
0.1314	6463.21636	0.28278446	17.7007467	0.50854893	0	-2.0295092	1.86628179	-1.2595403	2.87366332	-0.6742294	-6.9077553	4	86	2.68223333	296.333333	23.33333333	0	0	0	0	42.288408	0	-0.4111751
0.1602	7822.99288	0.27783	16.5895615	0.45984742	0	-1.8313322	2.05719502	-1.277153	2.80883395	-0.7746883	-6.9077553	5	86	1.34111667	296.333333	23.33333333	0	0	0	0	42.288408	0	-0.4111751
0.1827	8919.58805	0.26683708	15.453645	0.45977135	0	-1.6999098	2.18836187	-1.3173764	2.73790961	-0.7748534	-6.9077553	6	86	2.23519444	296.333333	23.33333333	0	0	0	0	42.288408	0	-0.4111751
0.2079	10103.9099	0.26908192	15.6698685	0.47111961	0	-1.5706981	2.31302143	-1.30903	2.75180348	-0.7505229	-6.9077553	7	87	1.34111667	296.333333	23.33333333	0	0	0	0	42.288408	0	-0.4111751
0.2376	11551.4154	0.25168682	13.5618721	0.42439758	0	-1.4371667	2.44689454	-1.3756044	2.60733607	-0.8547311	-6.9077553	8	88	0.89407778	295.777778	22.7777778	0	0	0	0	42.288408	0	-0.4111751
0.2628	12779.6001	0.26268937	14.6332823	0.45680472	0	-1.336362	2.5479284	-1.3329835	2.68336688	-0.7813126	-6.9077553	9	88	2.23519444	295.777778	22.7777778	0	0	0	0	42.288408	0	-0.4111751
0.2979	14492.7647	0.38578769	46.1761412	0.68659548	0	-1.2109974	2.67371854	-0.9498793	3.8324849	-0.3745546	-6.9077553	10	88	2.68223333	295.777778	22.7777778	0	0	0	0	42.288408	0	-0.4111751
0.3987	19342.1605	0.83148629	195.680873	1.64070783	0	-0.919546	2.96233889	-0.1833385	5.27649025	0.49573706	-6.9077553	11	86	2.68223333	296.333333	23.33333333	0	0	0	0	42.288408	0	-0.4111751
0.6084	26982.9905	0.73104596	160.897197	1.82805349	0	-0.4969227	3.29524375	-0.311912	5.08077185	0.60379861	-6.9077553	12	82	3.57631111	296.888889	23.88888888	0	0	1	0	42.288408	0	-0.4111751
0.8532	36271.8566	0.81046379	184.837632	1.55264215	0	-0.1587613	3.59106971	-0.2089155	5.21948319	0.44060195	-6.9077553	13	75	3.57631111	298.555556	25.55555555	0	0	1	0	42.288408	0	-0.4111751
1.2267	48315.8965	0.83484975	188.041905	1.96565265	0	0.20432764	3.87778132	-0.1793064	5.23667016	0.67633293	-6.9077553	14	69	4.47038889	299.111111	26.11111111	0	0	1	0	42.288408	0	-0.4111751
1.5939	61122.7217	0.80598951	168.08475	1.6671717	0	0.46618384	4.11290004	-0.2144446	5.12447427	0.51172824	-6.9077553	15	64	4.91742778	300.777778	27.7777778	0	0	1	0	42.288408	0	-0.4111751
2.0709	80058.2262	0.87655271	209.727756	2.03697118	0	0.7279833	4.38276669	-0.1306183	5.34581506	0.71195479	-6.9077553	16	61	4.47038889	301.333333	28.33333333	0	0	1	0	42.288408	0	-0.4111751
2.655	102036.822	0.85600697	203.635352	1.95442658	0	0.97644465	4.62534355	-0.1543092	5.31633582	0.67060837	-6.9077553	17	59	4.47038889	301.333333	28.33333333	0	0	1	0	42.288408	0	-0.4111751
3.4155	136218.248	1.00125899	290.797393	2.75024992	0	1.2283239	4.91426571	0.00225644	5.67263022	1.01205532	-6.9077553	18	54	6.25854444	301.888889	28.88888888	0	0	1	0	42.288408	0	-0.4111751
4.2309	173312.534	0.97448232	271.367964	2.31986221	0	1.44241474	5.15510229	-0.0248232	5.60347938	0.84193876	-6.9077553	19	54	4.02335	301.888889	28.88888888	0	0	1	0	42.288408	0	-0.4111751
4.9284	205010.617	0.90418945	236.60254	1.87253339	0	1.59501439	5.32306665	-0.099611	5.46638591	0.62782616	-6.9077553	20	54	3.12927222	301.888889	28.88888888	0	0	1	0	42.288408	0	-0.4111751
5.436	227078.528	0.84800298	206.093362	1.53790738	0	1.6930435	5.4253003	-0.1636926	5.32833413	0.43107267	-6.9077553	21	55	3.12927222	300.777778	27.7777778	0	0	1	0	42.288408	0	-0.4111751
5.7564	241304.029	0.87483833	209.690177	1.4929477	0	1.75031228	5.48606181	-0.1325738	5.34563586	0.40142208	-6.9077553	22	62	2.68223333	299.666667	26.66666666	0	0	1	0	42.288408	0	-0.4111751
5.9868	251315.55	0.83299285	191.808319	1.47504091	0	1.78955705	5.5267133	-0.1815304	5.25650175	0.38936344	-6.9077553	23	63	3.12927222	298.555556	25.55555555	0	0	1	0	42.288408	0	-0.4111751
0.0009	0	0	0	0	0	-7.0131158	-6.9077553	-6.9077553	-6.9077553	-6.9077553	-6.9077553	0	76	3.12927222	297.444444	24.44444444	0	0	0	0	18.9135952	0	-0.0428739
0.0009	0	0	0	0	0	-7.0131158	-6.9077553	-6.9077553	-6.9077553	-6.9077553	-6.9077553	1	79	3.12927222	296.888889	23.88888888	0	0	0	0	18.9135952	0	-0.0428739
0.0009	0	0	0	0	0	-7.0131158	-6.9077553	-6.9077553	-6.9077553	-6.9077553	-6.9077553	2	82	2.68223333	296.333333	23.33333333	0	0	0	0	18.9135952	0	-0.0428739
0.0009	0	0	0	0	0	-7.0131158	-6.9077553	-6.9077553	-6.9077553	-6.9077553	-6.9077553	3	86	2.23519444	295.777778	22.7777778	0	0	0	0	18.9135952	0	-0.0428739
0.0009	0	0	0	0	0	-7.0131158	-6.9077553	-6.9077553	-6.9077553	-6.9077553	-6.9077553	4	86	2.68223333	296.333333	23.33333333	0	0	0	0	18.9135952	0	-0.0428739
0.0009	0	0	0	0	0	-7.0131158	-6.9077553	-6.9077553	-6.9077553	-6.9077553	-6.9077553	5	86	1.34111667	296.333333	23.33333333	0	0	0	0	18.9135952	0	-0.0428739
0.0009	0	0	0	0	0	-7.0131158	-6.9077553	-6.9077553	-6.9077553	-6.9077553	-6.9077553	6	86	2.23519444	296.333333	23.33333333	0	0	0	0	18.9135952	0	-0.0428739
0.0009	0	0	0	0	0	-7.0131158	-6.9077553	-6.9077553	-6.9077553	-6.9077553	-6.9077553	7	87	1.34111667	296.333333	23.33333333	0	0	0	0	18.9135952	0	-0.0428739
0.0009	0	0	0	0	0	-7.0131158	-6.9077553	-6.9077553	-6.9077553	-6.9077553	-6.9077553	8	88	0.89407778	295.777778	22.7777778	0	0	0	0	18.9135952	0	-0.0428739
0.0009	0	0	0	0	0	-7.0131158	-6.9077553	-6.9077553	-6.9077553	-6.9077553	-6.9077553	9	88	2.23519444	295.777778	22.7777778	0	0	0	0	18.9135952	0	-0.0428739
0.0009	0	0	0	0	0	-7.0131158	-6.9077553	-6.9077553	-6.9077553	-6.9077553	-6.9077553	10	88	2.68223333	295.777778	22.7777778	0	0	0	0	18.9135952	0	-0.0428739
0.0009	0	0	0	0	0	-7.0131158	-6.9077553	-6.9077553	-6.9077553	-6.9077553	-6.9077553	11	86	2.68223333	296.333333	23.33333333	0	0	0	0	18.9135952	0	-0.0428739
0.0009	0	0	0	0	0	-7.0131158	-6.9077553	-6.9077553	-6.9077553	-6.9077553	-6.9077553	12	82	3.57631111	296.888889	23.88888888	0	0	0	0	18.9135952	0	-0.0428739
0.0261	0	0.60977772	112.668117	2.88861166	0	-3.64582	-6.9077553	-0.4930222	4.72445535	1.06112212	-6.9077553	13	75	3.57631111	298.555556	25.55555555	0	0	0	0	18.9135952	0	-0.0428739
0.0927	508.76599	0.67308343	132.531435	2.4278583	0	-2.3783868	-0.6738035	-0.3944014	4.88682741	0.88742131	-6.9077553	14	69	4.47038889	299.111111	26.11111111	0	0	1	0	18.9135952	0	-0.0428739
0.2151	1999.59672	0.62534792	104.200066	1.98650831	0	-1.5366522	0.6934455	-0.4678493	4.64632236	0.68688175	-6.9077553	15	64	4.91742778	300.777778	27.7777778	0	0	1	0	18.9135952	0	-0.0428739
0.4104	7503.13089	0.76307524	187.847098	1.93289902	0	-0.890623	2.01545365	-0.269089	5.23563365	0.65953818	-6.9077553	16	61	4.47038889	301.333333	28.33333333	0	0	1	0	18.9135952	0	-0.0428739
0.6984	18528.9329	0.88251696	253.709424	2.40761547	0	-0.3589633	2.91938742	-0.1238448	5.53619355	0.87905209	-6.9077553	17	59	4.47038889	301.333333	28.33333333	0	0	1	0	18.9135952	0	-0.0428739
1.1061	32458.8653	1.00737394	322.550014	3.48879998	0	0.10084031	3.48000441	0.00833907	5.77626131	1.24984442	-6.9077553	18	54	6.25854444	301.888889	28.88888888	0	0	1	0	18.9135952	0	-0.0428739
1.449	45266.5012	0.88417714	228.681184	2.15367275	0	0.37087366	3.81258936	-0.1219675	5.4323332	0.76763885	-6.9077553	19	54	4.02335	301.888889	28.88888888	0	0	1	0	18.9135952	0	-0.0428739
1.8351	61642.9912	0.89632112	231.574811	1.95689396	0	0.60709898	4.12137576	-0.1083415	5.4449073	0.67186938	-6.9077553	20	54	3.12927222	301.888889	28.88888888	0	0	1	0	18.9135952	0	-0.0428739
2.2014	78631.8005	0.90747055	239.738444	1.75916532	0	0.78909352	4.36478892	-0.0959928	5.47955269	0.56540774	-6.9077553	21	55	3.12927222	300.777778	27.7777778	0	0	1	0	18.9135952	0	-0.0428739
2.4543	91608.1723	0.93579199	239.465275	1.69586594	0	0.89784159	4.5175314	-0.065294	5.47841259	0.52878299	-6.9077553	22	62	2.68223333	299.666667	26.66666666	0	0	1	0	18.9135952	0	-0.0428739
2.7099	105011.898	0.95214221	249.339706	1.85798495	0	0.99691173	4.65408319	-0.0479912	5.51882026	0.62003061	-6.9077553	23	63	3.12927222	298.555556	25.55555555	0	0	1	0	18.9135952	0	-0.0428739
0.0135	409.686525	0.81828363	194.329084	1.51690197	0	-4.3050656	-0.8899251	-0.1993249	5.26955818	0.4173291	-6.9077553	0	58	2.68223333	305.777778	32.7777778	0	0	0	0	30.519848	0	0.21141735
0.0297	1086.0021	0.84143798	189.636542	1.38011127	0	-3.5166082	0.08342354	-0.1714552	5.24511458	0.32288844	-6.9077553	1	65	1.78815556	304.111111	31.11111111	0	0	0	0	30.519848	0	0.21141735
0.0423	1350.80868	0.88764674	206.473371	1.67042351	0	-3.1629682	0.30144346	-0.1180555	5.33017629	0.51367566	-6.9077553	2	70	1.78815556	303	30	0	0	0	0	30.519848	0	0.21141735
0.0432	1350.80868	0.61928523	91.6537018	0.8939572	0	-3.1419148	0.30144346	-0.4775759	4.51802827	-0.1109794	-6.9077553	3	77	0.44703889	301.888889	28.88888888	0	0	0	0	30.519848	0	0.21141735
0.0432	1350.80868	0	0	0	0	-3.1419148	0.30144346	-6.9077553	-6.9077553	-6.9077553	-6.9077553	4	75	0.89407778	297.444444	24.44444444	0	0	0	0	30.519848	0	0.21141735
0.0432	1350.80868	0	0	0	0	-3.1419148	0.30144346	-6.9077553	-6.9077553	-6.9077553	-6.9077553	5	88	0.89407778	295.222222	22.2222222	0	0	0	0	30.519848	0	0.21141735
0.0432	1350.80868	0	0	0	0	-3.1419148	0.30144346	-6.9077553	-6.9077553	-6.9077553	-6.9077553	6	46	2.23519444	296.888889	23.88888888	0	0	0	0	30.519848	0	0.21141735
0.0882	1745.58272	0.33991642	34.7957422	1.23345589	0	-2.4281483	0.55766115	-1.0761179	3.54952377	0.2106303	-6.9077553	7	42	2.23519444	296.333333	23.33333333	0	0	0	0	30.519848	0	0.21141735
0.1584	2601.15637	0.44245461	61.0491975	1.24029688	0	-1.8426318	0.95634047	-0.8131598	4.11169644	0.2161567	-6.9077553	8	35	3.12927222	295.777778	22.7777778	0	0	0	0	30.519848	0	0.21141735
0.3663	4112.49105	0.69320824	131.924919	3.27231482	0	-1.0043026	1.41427207	-0.3649833	4.88224054	1.18580318	-6.9077553	9	36	4.91742778	294.666667	21.66666666	0	0	0	1	30.519848	0	0.21141735
0.5022	5610.04591	0.53415816	78.7769104	1.3183044	0	-0.6887568	1.72473714	-0.6251929	4.36663263	0.27710463	-6.9077553	10	43	2.68223333	293	20	0	0	0	1	30.519848	0	0.21141735
0.621	7482.58233	0.46196519	65.2629173	0.77242499	0	-0.4764242	2.0127116	-0.7701034	4.17843931	-0.2569266	-6.9077553	11	54	0.44703889	290.222222	17.2222222	0	0	0	1	30.519848	0	0.21141735

0.7479	9447.64816	0.52532504	78.7032774	0.87316148	0	-0.290486	2.24587168	-0.6418363	4.3656975	-0.1344902	-6.9077553	12	55	1.34111667	291.333333	18.33333333	0	0	0	1	30.519848	0	0.21141735
1.0611	12328.6902	0.65184929	131.130253	1.93807124	0	0.05930611	2.51201019	-0.426409	4.87619875	0.66220912	-6.9077553	13	46	3.12927222	294.111111	21.11111111	0	0	0	1	30.519848	0	0.21141735
1.8045	20262.2745	0.95581853	273.250757	3.85025431	0	0.59028355	3.00881011	-0.0441415	5.61039356	1.34839889	-6.9077553	14	40	5.36446667	295.777778	22.7777778	0	0	0	1	30.519848	0	0.21141735
2.8332	32706.6916	1.01947529	312.582021	4.63423761	1	1.04140681	3.48761027	0.02026849	5.7448701	1.53368746	0.0009995	15	35	6.25854444	297.444444	24.44444444	0	0	0	1	30.519848	0	0.21141735
3.7413	51189.6536	0.9500586	261.222869	2.35871638	1	1.31943314	3.93555697	-0.0501796	5.56537778	0.85854143	0.0009995	16	31	6.70558333	299.111111	26.11111111	0	0	0	1	30.519848	0	0.21141735
4.6863	65255.084	0.9779827	273.038631	2.36435524	1	1.54464336	4.17831928	-0.0212413	5.60961695	0.86092822	0.0009995	17	27	6.25854444	300.777778	27.7777778	0	0	0	1	30.519848	0	0.21141735
5.8257	166653.898	1.27099378	458.039346	3.03788191	84	1.76227916	5.11592519	0.24058557	6.12695727	1.11148965	4.4308287	18	24	7.59966111	301.888889	28.88888888	0	0	0	1	30.519848	0	0.21141735
6.741	307938.976	1.40039219	531.706121	2.64851432	207	1.90820828	5.72990488	0.33746616	6.27609281	0.97437635	5.33272362	19	23	5.36446667	303.555556	30.55555555	0	0	0	1	30.519848	0	0.21141735
8.0109	813759.508	2.49735961	1220.61729	3.4710959	692	2.08080311	6.70166611	0.91563436	7.10711281	1.24475842	6.5395874	20	23	5.81150556	303.555556	30.55555555	0	0	0	1	30.519848	0	0.21141735
9.9081	1512102.44	2.64136854	1343.66319	4.97394042	1414	2.2933526	7.32125697	0.97167569	7.20315563	1.60441339	7.25417855	21	22	6.25854444	304.111111	31.11111111	0	0	0	1	30.519848	0	0.21141735
11.8836	2155507.04	2.74421982	1505.04157	4.91457325	2080	2.4751593	7.67578172	1.00986115	7.31657646	1.59240838	7.64012365	22	23	5.36446667	304.111111	31.11111111	0	0	0	1	30.519848	0	0.21141735
13.0995	2236033.97	1.04828915	338.465697	2.67408293	2137	2.57257406	7.71245947	0.04811294	5.8244257	0.98398038	7.66715872	23	24	4.91742778	303.555556	30.55555555	0	0	0	1	30.519848	0	0.21141735
0.0342	1269.82511	0.99604612	283.55862	2.58340351	0	-3.3755296	0.23966638	-0.0029583	5.6474224	0.94949473	-6.9077553	0	58	2.68223333	305.777778	32.7777778	0	0	0	0	34.0724638	0	0.49277013
0.0891	3566.91437	0.84307993	188.557793	1.28922309	0	-2.4179959	1.27198122	-0.1695081	5.23940986	0.25481514	-6.9077553	1	65	1.78815556	304.111111	31.11111111	0	0	0	0	34.0724638	0	0.49277013
0.1458	6300.39927	0.75663963	146.365963	1.05460418	0	-1.9255195	1.84077172	-0.2775474	4.98611691	0.05411329	-6.9077553	2	70	1.78815556	303	30	0	0	0	0	34.0724638	0	0.49277013
0.1935	8318.13411	0.66600022	108.641684	0.86138995	0	-1.6424778	2.11855818	-0.4049649	4.68806437	-0.1480477	-6.9077553	3	77	0.44703889	301.888889	28.88888888	0	0	0	0	34.0724638	0	0.49277013
0.2205	9458.59196	0.51187239	62.5166707	0.6694081	0	-1.5118576	2.24702925	-0.6677282	4.13544925	-0.3998686	-6.9077553	4	75	0.89407778	297.444444	24.44444444	0	0	0	0	34.0724638	0	0.49277013
0.2448	10467.4593	0.47287386	52.6517379	0.63185219	0	-1.4073137	2.34836686	-0.7468141	3.96371824	-0.4575184	-6.9077553	5	88	0.89407778	295.222222	22.2222222	0	0	0	0	34.0724638	0	0.49277013
0.2601	10906.0965	0.28836584	23.0337417	0.55762695	0	-1.3466891	2.38941363	-1.2400635	3.13700358	-0.5822734	-6.9077553	6	46	2.23519444	296.888889	23.88888888	0	0	0	0	34.0724638	0	0.49277013
0.3762	15062.0851	0.81272178	191.966267	1.64517756	0	-0.9776344	2.71224706	-0.2061368	5.25732487	0.49845597	-6.9077553	7	42	2.23519444	296.333333	23.33333333	0	0	0	0	34.0724638	0	0.49277013
0.5391	21610.0404	0.88168268	214.257532	1.65385648	0	-0.6178542	3.07320431	-0.1247895	5.36718338	0.50371428	-6.9077553	8	35	3.12927222	295.777778	22.7777778	0	0	0	1	34.0724638	0	0.49277013
0.7731	29733.4391	0.96053261	262.074369	2.24714731	0	-0.2573469	3.39230594	-0.0392268	5.56863213	0.81010646	-6.9077553	9	36	4.91742778	294.666667	21.66666666	0	0	0	1	34.0724638	0	0.49277013
1.0233	40429.3216	0.93064331	246.943402	1.86298222	0	0.0230327	3.69958004	-0.0708052	5.50916322	0.62271518	-6.9077553	10	43	2.68223333	293	20	0	0	0	1	34.0724638	0	0.49277013
1.2339	48037.1458	0.80983441	189.377003	1.42784914	0	0.21017988	3.8719954	-0.2096914	5.24374503	0.35686932	-6.9077553	11	54	0.44703889	290.222222	17.2222222	0	0	0	1	34.0724638	0	0.49277013
1.4211	53618.2209	0.7370896	165.707745	1.25112446	0	0.35143122	3.9819076	-0.3036901	5.1102317	0.22484167	-6.9077553	12	55	1.34111667	291.333333	18.33333333	0	0	0	1	34.0724638	0	0.49277013
1.7487	64110.6897	0.88777345	223.218664	1.85150381	0	0.55887265	4.16062671	-0.1179129	5.40815633	0.61653814	-6.9077553	13	46	3.12927222	294.111111	21.11111111	0	0	0	1	34.0724638	0	0.49277013
2.3211	86314.3109	1.0206034	300.709046	2.69637351	0	0.84204121	4.458007	0.02137335	5.7061465	0.99227853	-6.9077553	14	40	5.36446667	295.777778	22.7777778	0	0	0	1	34.0724638	0	0.49277013
3.1635	115963.747	1.07759172	327.723958	3.02306555	0	1.15167901	4.75328624	0.07565623	5.79217471	1.10660213	-6.9077553	15	35	6.25854444	297.444444	24.44444444	0	0	0	1	34.0724638	0	0.49277013
4.3839	161152.428	1.1137346	360.747926	3.47343188	1	1.47793874	5.08235688	0.10861635	5.88818222	1.24543098	0.0009995	16	31	6.70558333	299.111111	26.11111111	0	0	0	1	34.0724638	0	0.49277013
5.5593	200543.588	1.03580567	306.450182	2.84591661	1	1.7154722	5.30103661	0.03614452	5.72505847	1.04623652	0.0009995	17	27	6.25854444	300.777778	27.7777778	0	0	0	1	34.0724638	0	0.49277013
6.9714	260861.002	1.18394362	418.953314	3.84070641	3	1.94181607	5.56399154	0.1696952	6.03776188	1.34591665	1.09894557	18	24	7.59966111	301.888889	28.88888888	0	0	0	1	34.0724638	0	0.49277013
8.4699	313122.736	1.05326445	331.320796	3.41484492	3	2.1365187	5.74659844	0.05284332	5.8030901	1.22842488	1.09894557	19	23	5.36446667	303.555556	30.55555555	0	0	0	1	34.0724638	0	0.49277013
9.9936	363916.833	0.95060947	283.925724	3.25277741	3	2.30194489	5.89692811	-0.0496005	5.64871619	1.1798166	1.09894557	20	23	5.81150556	303.555556	30.55555555	0	0	0	1	34.0724638	0	0.49277013
11.5191	409310.81	0.97539855	305.066261	3.55803374	3	2.44400653	6.01447724	-0.0238844	5.72053228	1.26948909	1.09894557	21	22	6.25854444	304.111111	31.11111111	0	0	0	1	34.0724638	0	0.49277013
12.9555	457297.952	0.95452988	287.30038	3.12774735	3	2.56152041	6.12533734	-0.0454892	5.66053177	1.14063272	1.09894557	22	23	5.36446667	304.111111	31.11111111	0	0	0	1	34.0724638	0	0.49277013
14.4936	503907.057	0.91780404	269.003986	3.11099956	3	2.67370717	6.22239383	-0.0846824	5.59472992	1.13526546	1.09894557	23	24	4.91742778	303.555556	30.55555555	0	0	0	1	34.0724638	0	0.49277013
0.0414	2938.48121	1.23757765	426.245443	2.69312219	0	-3.1844744	1.07823311	0.21396367	6.05501768	0.99107243	-6.9077553	0	58	2.68223333	305.777778	32.7777778	0	0	0	0	42.288408	0	-0.4111751
0.1269	7802.90236	0.99456554	273.881086	1.92358736	0	-2.0643559	2.05462391	-0.0044443	5.61269767	0.65471158	-6.9077553	1	65	1.78815556	304.111111	31.11111111	0	0	0	0	42.288408	0	-0.4111751
0.2205	12781.8023	0.95013626	241.362192	1.79585736	0	-1.5118576	2.5481007	-0.0500979	5.48630282	0.58603923	-6.9077553	2	70	1.78815556	303	30	0	0	0	0	42.288408	0	-0.4111751
0.3186	17433.2595	0.87383865	201.164869	1.70482279	0	-1.1438189	2.85843721	-0.1337158	5.30412979	0.53404757	-6.9077553	3	77	0.44703889	301.888889	28.88888888	0	0	0	0	42.288408	0	-0.4111751
0.3942	20986.2251	0.67714582	116.274158	1.28089278	0	-0.9308969	3.04391392	-0.3883929	4.75595944	0.24833772	-6.9077553	4	75	0.89407778	297.444444	24.44444444	0	0	0	0	42.288408	0	-0.4111751
0.4635	24276.0093	0.54585806	76.2701654	0.93729381	0	-0.7689489	3.18952978	-0.603566	4.33429496	-0.0636922	-6.9077553	5	88	0.89407778	295.222222	22.2222222	0	0	0	0	42.288408	0	-0.4111751
0.5229	27068.4877	0.52820859	87.4848485	0.84188299	0	-0.648365	3.29840718	-0.6363726	4.47147705	-0.1709271	-6.9077553	6	46	2.23519444	296.888889	23.88888888	0	0	1	0	42.288408	0	-0.4111751
0.8559	40469.2703	0.86415092	230.97027	1.90116247	0	-0.1556017	3.70056764	-0.1448513	5.44229333	0.64299138	-6.9077553	7	42	2.23519444	296.333333	23.33333333	0	0	1	0	42.288408	0	-0.4111751
1.1925	52055.5496	0.84581165	199.564171	1.87469417	0	0.17605194	3.95233062	-0.166277	5.29614086	0.62897882	-6.9077553	8	35	3.12927222	295.777778	22.7777778	0	0	1	0	42.288408	0	-0.4111751
1.5795	65002.5918	0.81820622	178.147057	2.00593262	0	0.45710834	4.17444253	-0.1994194	5.18261498	0.6966075	-6.9077553	9	36	4.91742778	294.666667	21.66666666	0	0	1	0	42.288408	0	-0.4111751
1.9683	80669.4434	0.85070801	201.53776	1.63775592	0	0.67717023	4.39037226	-0.1605115	5.30598172	0.49393737	-6.9077553	10	43	2.68223333	293	20	0	0	1	0	42.288408	0	-0.4111751
2.2986	93742.718	0.76169796	161.649246	1.27340216	0	0.83230024	4.54056466	-0.2708932	5.08543503	0.24247717	-6.9077553	11	54	0.44703889	290.222222	17.2222222	0	0	1	0	42.288408	0	-0.4111751
2.7234	111100.603	0.86279756	209.775788	1.56905843	0	1.0018811	4.71044513	-0.1464168	5.34604405	0.45111284	-6.9077553	12	55	1.34111667	291.333333	18.33333333	0	0	1	0	42.288408	0	-0.4111751
3.4155	134449.412	0.83264763	200.53379	2.08289479	0	1.2283239	4.90119545	-0.1819445	5.30098775	0.73423864	-6.9077553	13	46	3.12927222	294.111111	21.11111111	0	0	1	0	42.288408	0	-0.4111751
4.6575	160546.418	0.81235741	199.94846	3.42534534	0	1.53847882	5.07858934	-0.2065847	5.29806464	1.23149419	-6.9077553	14	40	5.36446667	295.777778	22.7777778	0	0	1	0	42.288408	0	-0.4111751
6.183	190090.161	0.84323939	213.939712	4.00382271	0	1.82180359	5.24750375	-0.1693192	5.36569893	1.38749931	-6.9077553	15	35	6.25854444	297.444444	24.44444444	0	0	1	0	42.288408	0	-0.4111751
7.9848	250758.831	1.08709064	375.476024	4.69306767	0	2.07753973	5.52449563	0.08442446	5.92819728	1.54629951	-6.9077553	16	31	6.70558333	299.111111	26.11111111	0	0	1	0	42.288408	0	-0.4111751
9.4212	322199.012	1.19021782	433.669643	3.75724529	0	2.24296247	5.77517251	0.17497617	6.07228536	1.32395217	-6.9077553	17	27	6.25854444	300.777778	27.7777778	0	0	1	0	42.288408	0	-0.4111751
10.8837	399509.799	1.21991729	455.415885	3.81454387	0	2.38726626	5.9902408	0.19960246	6.12121323	1.33908322	-6.9077553	18	24	7.59966111	301.888889	28.88888888	0	0	1	0	42.288408	0	-0.4111751
12.3831	474988.267	1.18691676	419.347089	3.32732605	3	2.51633264	6.16329221	0.17220115	6.03870134	1.20246949	1.09894557	19	23	5.36446667	303.555556	30.55555555	0	0	1	0	42.288408	0	-0.4111751
14.0229	551121.292	1.18746141	421.938358	3.99983599	4	2.64069171	6.31195673	0.17265954	6.0448616	1.38650334	1.38654433	20	23	5.81150556	303.555556	30.55555555	0	0	1	0	42.288408	0	-0.4111751
15.5763	628471.379	1.18634479	432.156974	3.56738564	4	2.74575053	6.44329208	0.17171954	6.0687912	1.27211329	1.38654433	21	22	6.25854444	304.111111	31.11111111	0	0	1	0	42.288408	0	-0.4111751
17.0775	690848.745	1.05110604	326.605516	3.14071165	4	2.83776181	6.53792235	0.05079391	5.78875613	1.14476776	1.38654433	22	23	5.36446667	304.111111	31.11111111	0	0	1	0	42.288408	0	-0.4111751
18.5121	745923.29	1.03761297	321.571008	2.83988357	4	2.91842457	6.61462411	0.03788614	5.7732215	1.04411512	1.38654433	23	24	4.91742778	303.555556	30.55555555	0	0	1	0	42.288408	0	-0.4111751
0.0027	0	0.21041781	8.77035046	0.69165194	0	-5.9145035	-6.9077553	-1.553919	2.17149078	-0.3672277	-6.9077553	0	58	2.68223333	305.777778	32.7777778	0	0	0	0	18.9135952	0	-0.0428739
0.0036	0	0.06053963	0.5845502	0.09518638	0	-5.6268214	-6.9077553	-2.788074	-0.5352034	-2.3414676	-6.9077553	1	65	1.78815556	304.111111	31.11111111	0	0	0	0	18.9135952	0	-0.0428739

0.0036	0	0	0	0	0	-5.6268214	-6.9077553	-6.9077553	-6.9077553	-6.9077553	-6.9077553	2	70	1.78815556	303	30	0	0	0	0	18.9135952	0	-0.0428739
0.0036	0	0	0	0	0	-5.6268214	-6.9077553	-6.9077553	-6.9077553	-6.9077553	-6.9077553	3	77	0.44703889	301.888889	28.88888888	0	0	0	0	18.9135952	0	-0.0428739
0.0036	0	0	0	0	0	-5.6268214	-6.9077553	-6.9077553	-6.9077553	-6.9077553	-6.9077553	4	75	0.89407778	297.444444	24.44444444	0	0	0	0	18.9135952	0	-0.0428739
0.0036	0	0	0	0	0	-5.6268214	-6.9077553	-6.9077553	-6.9077553	-6.9077553	-6.9077553	5	88	0.89407778	295.222222	22.2222222	0	0	0	0	18.9135952	0	-0.0428739
0.0036	0	0	0	0	0	-5.6268214	-6.9077553	-6.9077553	-6.9077553	-6.9077553	-6.9077553	6	46	2.23519444	296.888889	23.88888888	0	0	0	0	18.9135952	0	-0.0428739
0.0396	0	0.41784134	45.2463715	1.57748966	0	-3.2289262	-6.9077553	-0.8702631	3.81214458	0.45646848	-6.9077553	7	42	2.23519444	296.333333	23.33333333	0	0	0	0	18.9135952	0	-0.0428739
0.1179	0	0.40626833	41.056433	1.66732876	0	-2.1379185	-6.9077553	-0.898283	3.71497189	0.51182238	-6.9077553	8	35	3.12927222	295.777778	22.7777778	0	0	0	0	18.9135952	0	-0.0428739
0.2475	131.59135	0.374614	35.3586561	1.60467667	0	-1.3963447	-2.0204834	-0.9791933	3.56557151	0.47354527	-6.9077553	9	36	4.91742778	294.666667	21.66666666	0	0	1	0	18.9135952	0	-0.0428739
0.342	789.548048	0.40047546	55.3185361	1.12942062	0	-1.0729445	-0.2350288	-0.9126089	4.01312612	0.12258979	-6.9077553	10	43	2.68223333	293	20	0	0	1	0	18.9135952	0	-0.0428739
0.4077	1863.67245	0.45577021	77.853315	0.81553524	0	-0.8972237	0.62308541	-0.7835748	4.35483932	-0.2026852	-6.9077553	11	54	0.44703889	290.222222	17.2222222	0	0	1	0	18.9135952	0	-0.0428739
0.495	4341.9899	0.61700361	130.091012	1.14069091	0	-0.7031975	1.46856303	-0.481261	4.86824198	0.13251042	-6.9077553	12	55	1.34111667	291.333333	18.33333333	0	0	1	0	18.9135952	0	-0.0428739
0.7011	7277.66283	0.56442294	107.619431	1.57613389	0	-0.3551047	1.98494717	-0.5701813	4.67861051	0.45560921	-6.9077553	13	46	3.12927222	294.111111	21.11111111	0	0	1	0	18.9135952	0	-0.0428739
1.1448	15551.2553	0.85060736	250.127472	3.52490284	0	0.13522995	2.74420566	-0.1606297	5.52197467	1.26013653	-6.9077553	14	40	5.36446667	295.777778	22.7777778	0	0	1	0	18.9135952	0	-0.0428739
1.7361	31280.6925	0.94409533	277.89512	4.19272022	0	0.55164122	3.44303302	-0.0564695	5.62724737	1.43358822	-6.9077553	15	35	6.25854444	297.444444	24.44444444	0	0	1	0	18.9135952	0	-0.0428739
2.5641	65728.4056	1.09317401	384.402344	4.09932622	0	0.94160754	4.1855464	0.08999975	5.95169238	1.41106654	-6.9077553	16	31	6.70558333	299.111111	26.11111111	0	0	1	0	18.9135952	0	-0.0428739
3.2949	96844.8714	1.05879008	343.563847	3.12425736	0	1.19237582	4.57312076	0.05807086	5.83937588	1.13951663	-6.9077553	17	27	6.25854444	300.777778	27.7777778	0	0	1	0	18.9135952	0	-0.0428739
4.3155	147945.943	1.2317323	476.229497	4.23528654	0	1.46221319	4.99685371	0.20923308	6.16590198	1.44368707	-6.9077553	18	24	7.59966111	301.888889	28.88888888	0	0	1	0	18.9135952	0	-0.0428739
5.2929	195124.608	1.1118553	366.866713	2.85780045	2	1.6663663	5.27364349	0.10692905	5.90500133	1.05040211	0.69364706	19	23	5.36446667	303.555556	30.55555555	0	0	1	0	18.9135952	0	-0.0428739
6.309	243169.114	1.08116249	351.72138	3.02356725	2	1.84197719	5.49376126	0.07896134	5.86284217	1.10676802	0.69364706	20	23	5.81150556	303.555556	30.55555555	0	0	1	0	18.9135952	0	-0.0428739
7.3899	294035.856	1.11248022	379.807452	3.38308734	2	2.0001142	5.68370512	0.10749044	5.93966705	1.21908425	0.69364706	21	22	6.25854444	304.111111	31.11111111	0	0	1	0	18.9135952	0	-0.0428739
8.352	339525.551	1.06376969	341.720621	2.68220519	2	2.12250103	5.82755215	0.06275852	5.83399643	0.98701204	0.69364706	22	23	5.36446667	304.111111	31.11111111	0	0	1	0	18.9135952	0	-0.0428739
9.2052	376535.489	0.97204094	268.957114	2.11929399	3	2.21976854	5.93101496	-0.0273291	5.59455566	0.75155475	1.09894557	23	24	4.91742778	303.555556	30.55555555	0	0	1	0	18.9135952	0	-0.0428739
0.0081	319.68896	0.84287256	191.519974	1.73260558	0	-4.8158912	-1.1372836	-0.1697538	5.25499733	0.55020339	-6.9077553	0	87	4.02335	300.222222	27.2222222	0	0	0	0	30.519848	0	0.21141735
0.0126	539.007931	0.49056225	60.8081421	0.85839157	0	-4.3740585	-0.6161715	-0.7101667	4.10774014	-0.1515306	-6.9077553	1	88	2.68223333	300.222222	27.2222222	0	0	0	0	30.519848	0	0.21141735
0.0486	1373.61529	0.57954683	101.606921	1.25867062	0	-3.0241317	0.3181739	-0.5437848	4.6211215	0.23085027	-6.9077553	2	84	3.57631111	300.777778	27.7777778	0	0	0	0	30.519848	0	0.21141735
0.0891	1724.52556	0.43198149	57.7110135	1.14542448	0	-2.4179959	0.54553168	-0.8370603	4.05546536	0.13664795	-6.9077553	3	78	3.57631111	301.888889	28.88888888	0	0	0	0	30.519848	0	0.21141735
0.1647	2514.0737	0.46513308	62.4879732	1.48952548	0	-1.8036296	0.92230211	-0.7632841	4.13499011	0.39912873	-6.9077553	4	76	4.02335	301.888889	28.88888888	0	0	0	0	30.519848	0	0.21141735
0.243	3166.24772	0.5430037	81.663737	1.47353775	0	-1.4146938	1.15286298	-0.6087992	4.40262229	0.38834455	-6.9077553	5	66	4.02335	304.111111	31.11111111	0	0	0	0	30.519848	0	0.21141735
0.3798	4603.22303	0.64487026	116.920667	1.91109266	0	-0.9681105	1.52697393	-0.4371566	4.7615042	0.64819827	-6.9077553	6	63	3.57631111	304.666667	31.66666666	0	0	0	1	30.519848	0	0.21141735
0.6372	9830.00177	0.88321542	235.955201	3.31417911	0	-0.4506717	2.28554084	-0.1230546	5.4636462	1.19851165	-6.9077553	7	57	5.81150556	305.777778	32.7777778	0	0	0	1	30.519848	0	0.21141735
0.9171	14669.8111	0.8006004	179.055165	2.61341039	0	-0.0865388	2.68585988	-0.221145	5.18769953	0.9610386	-6.9077553	8	56	5.36446667	305.777778	32.7777778	0	0	0	1	30.519848	0	0.21141735
1.2321	20974.4243	0.72567195	144.169308	2.19130685	0	0.20872003	3.04335148	-0.3192801	4.9709953	0.78495435	-6.9077553	9	55	5.81150556	306.333333	33.33333333	0	0	0	1	30.519848	0	0.21141735
1.4391	30628.5875	0.71962095	149.629925	1.34697239	0	0.36401792	3.42196645	-0.327642	5.00817176	0.29860153	-6.9077553	10	59	3.12927222	305.777778	32.7777778	0	0	0	1	30.519848	0	0.21141735
1.6209	38896.2511	0.67893091	132.80374	1.25973805	0	0.48298155	3.66092358	-0.3857641	4.88887993	0.23169731	-6.9077553	11	59	3.12927222	305.222222	32.2222222	0	0	0	1	30.519848	0	0.21141735
1.737	45305.192	0.68526287	130.224019	1.02334607	0	0.55215949	3.81344371	-0.3764945	4.86926387	0.02405442	-6.9077553	12	65	1.78815556	304.666667	31.66666666	0	0	0	1	30.519848	0	0.21141735
1.8045	51832.1978	0.7231251	136.983906	0.98429209	0	0.59028355	3.94803083	-0.3227911	4.91987075	-0.0148171	-6.9077553	13	70	1.34111667	304.111111	31.11111111	0	0	0	1	30.519848	0	0.21141735
1.863	56966.2548	0.65237703	110.412538	0.96407242	0	0.62218809	4.04247663	-0.4256009	4.70423275	-0.0355521	-6.9077553	14	73	1.78815556	303	30	0	0	0	1	30.519848	0	0.21141735
1.8873	58106.7146	0.49050052	57.8644884	0.74666285	0	0.63514724	4.06229844	-0.7102923	4.05812115	-0.2908031	-6.9077553	15	76	2.68223333	301.888889	28.88888888	0	0	0	1	30.519848	0	0.21141735
1.9044	58896.2617	0.29695415	19.3602986	0.46391838	0	0.644167	4.0757946	-1.2108156	2.96327615	-0.7658934	-6.9077553	16	80	2.23519444	301.333333	28.33333333	0	0	0	1	30.519848	0	0.21141735
1.9206	59685.8114	0.25197185	13.204695	0.31670515	0	0.65263764	4.08911108	-1.374477	2.58064817	-1.1466315	-6.9077553	17	81	2.68223333	301.333333	28.33333333	0	0	0	1	30.519848	0	0.21141735
1.9296	60124.4479	0.22580657	10.8257813	0.30423241	0	0.65731273	4.09643318	-1.4836577	2.38202281	-1.1866818	-6.9077553	18	83	2.68223333	300.777778	27.7777778	0	0	0	1	30.519848	0	0.21141735
1.9431	60606.95	0.2353821	11.4152802	0.30096556	0	0.66428464	4.10442607	-1.4423057	2.43504042	-1.1974423	-6.9077553	19	84	2.68223333	300.777778	27.7777778	0	0	0	1	30.519848	0	0.21141735
1.9548	61133.3125	0.23291375	11.0826029	0.29191806	0	0.67028789	4.11307329	-1.4528028	2.4054668	-1.2278624	-6.9077553	20	85	2.23519444	300.222222	27.2222222	0	0	0	1	30.519848	0	0.21141735
1.9629	61440.362	0.24418836	12.2789705	0.33590905	0	0.67442297	4.11808326	-1.4057285	2.50796952	-1.0879423	-6.9077553	21	84	3.57631111	300.222222	27.2222222	0	0	0	1	30.519848	0	0.21141735
1.9764	61966.7293	0.2339705	11.5616811	0.36018337	0	0.68127701	4.12661375	-1.4482953	2.44778277	-1.0183695	-6.9077553	22	84	2.68223333	300.222222	27.2222222	0	0	0	1	30.519848	0	0.21141735
1.9872	62361.5027	0.21541429	9.70314407	0.35527583	0	0.68672661	4.13296418	-1.5305607	2.27255302	-1.03205	-6.9077553	23	83	3.57631111	300.222222	27.2222222	0	0	0	1	30.519848	0	0.21141735
0.0171	753.207511	0.98456489	276.495741	2.1259471	0	-4.0686768	-0.2820877	-0.0145403	5.62219903	0.75468767	-6.9077553	0	87	4.02335	300.222222	27.2222222	0	0	0	0	34.0724638	0	0.49277013
0.0324	1323.43673	0.54699264	73.6081687	0.89039303	0	-3.4295969	0.28098726	-0.6014934	4.29876959	-0.1149698	-6.9077553	1	88	2.68223333	300.222222	27.2222222	0	0	0	0	34.0724638	0	0.49277013
0.0504	1937.52975	0.55913196	77.2013062	0.82677221	0	-2.9877641	0.66192982	-0.5795829	4.34642933	-0.1890173	-6.9077553	2	84	3.57631111	300.777778	27.7777778	0	0	0	0	34.0724638	0	0.49277013
0.1035	4128.19226	0.78294883	168.197017	1.59923372	0	-2.2681837	1.41808181	-0.2434115	5.12514196	0.4701497	-6.9077553	3	78	3.57631111	301.888889	28.88888888	0	0	0	0	34.0724638	0	0.49277013
0.1701	7191.03016	0.87927122	210.033691	1.45701403	0	-1.7713688	1.97297349	-0.1275252	5.34727271	0.37707526	-6.9077553	4	76	4.02335	301.888889	28.88888888	0	0	0	0	34.0724638	0	0.49277013
0.2808	12546.4569	1.02394402	312.141927	2.35614311	0	-1.2701126	2.52951801	0.02463799	5.74346118	0.85745034	-6.9077553	5	66	4.02335	304.111111	31.11111111	0	0	0	0	34.0724638	0	0.49277013
0.5778	22396.8982	1.15070301	388.357363	3.12603871	0	-0.5485275	3.10896712	0.14124173	5.96192853	1.14008646	-6.9077553	6	63	3.57631111	304.666667	31.66666666	0	0	0	1	34.0724638	0	0.49277013
1.3752	37290.0817	1.59874055	824.044441	6.32771828	2	0.31859918	3.6187542	0.46984146	6.71422568	1.84509773	0.69364706	7	57	5.81150556	305.777778	32.7777778	0	0	0	1	34.0724638	0	0.49277013
2.1582	63605.4699	1.22860262	445.045187	3.87137493	3	0.76927454	4.15271519	0.20669104	6.09817807	1.353868	1.09894557	8	56	5.36446667	305.777778	32.7777778	0	0	0	1	34.0724638	0	0.49277013
2.7666	85757.8208	1.01267355	294.275125	2.38398354	3	1.01761913	4.45153895	0.01358091	5.68451853	0.86919222	1.09894557	9	55	5.81150556	306.333333	33.33333333	0	0	0	1	34.0724638	0	0.49277013
3.2994	106698.297	0.95073288	250.783493	1.97646476	3	1.19374063	4.67001457	-0.0494709	5.52459398	0.6818156	1.09894557	10	59	3.12927222	305.777778	32.7777778	0	0	0	1	34.0724638	0	0.49277013
3.9249	126376.028	0.91925637	234.673561	2.49386417	3	1.36734087	4.83926973	-0.083103	5.45819971	0.91423429	1.09894557	11	59	3.12927222	305.222222	32.2222222	0	0	0	1	34.0724638	0	0.49277013
4.302	138933.43	0.80085685	182.853073	1.52395666	3	1.45908003	4.93400209	-0.2208252	5.20868842	0.42196599	1.09894557	12	65	1.78815556	304.666667	31.66666666	0	0	0	1	34.0724638	0	0.49277013
4.5126	147769.659	0.79084543	168.835036	1.26717292	3	1.50687348	4.99566147	-0.2333891	5.12892804	0.23757722	1.09894557	13	70	1.34111667	304.111111	31.11111111	0	0	0	1	34.0724638	0	0.49277013
4.689	154273.211	0.71520482	132.958377	1.2306921	3	1.54521934	5.03873161	-0.3337891	4.89004364	0.20838892	1.09894557	14	73	1.78815556	303	30	0	0	0	1	34.0724638	0	0.49277013
4.7745	157255.951	0.48942333	61.1781301	0.84343487	3	1.56328926	5.0578811	-0.7124863	4.11380612	-0.1690877	1.09894557	15	76	2.68223333	301.888889	28.88888888	0	0	0	1	34.0724638	0	0.49277013

4.8312	159493.212	0.33397444	29.8058888	0.62955105	3	1.57509488	5.07200763	-1.093701	3.39473954	-0.4611612	1.09894557	16	80	2.23519444	301.333333	28.33333333	0	0	0	1	34.0724638	0	0.49277013
4.8744	161247.76	0.28552739	18.1070824	0.50358105	3	1.58399702	5.08294827	-1.2499212	2.89635838	-0.6840268	1.09894557	17	81	2.68223333	301.333333	28.33333333	0	0	0	1	34.0724638	0	0.49277013
4.9347	163704.132	0.27150049	16.5708737	0.48470204	3	1.59629188	5.09806683	-1.3001149	2.8077069	-0.7221599	1.09894557	18	83	2.68223333	300.777778	27.7777778	0	0	0	1	34.0724638	0	0.49277013
4.9689	165327.087	0.25757898	14.4687709	0.401493	3	1.60319849	5.1079319	-1.3525541	2.67206171	-0.9100776	1.09894557	19	84	2.68223333	300.777778	27.7777778	0	0	0	1	34.0724638	0	0.49277013
5.013	167169.373	0.22846366	11.5834326	0.39378727	3	1.61203454	5.11901349	-1.4720106	2.44966218	-0.9294082	1.09894557	20	85	2.23519444	300.222222	27.2222222	0	0	0	1	34.0724638	0	0.49277013
5.0382	168178.237	0.19507335	8.06595285	0.28845089	3	1.61704888	5.1250303	-1.6292665	2.08777582	-1.2397696	1.09894557	21	84	3.57631111	300.222222	27.2222222	0	0	0	1	34.0724638	0	0.49277013
5.0733	169669.61	0.21671603	9.8974625	0.30148638	3	1.62399149	5.13385897	-1.5245637	2.29237944	-1.195719	1.09894557	22	84	2.68223333	300.222222	27.2222222	0	0	0	1	34.0724638	0	0.49277013
5.1039	171117.111	0.20303383	8.81320729	0.3080322	3	1.63000495	5.14235403	-1.5894695	2.17636488	-1.1743098	1.09894557	23	83	3.57631111	300.222222	27.2222222	0	0	0	1	34.0724638	0	0.49277013
0.0369	2388.37446	1.13504887	356.657935	2.47648296	0	-3.2995437	0.8710316	0.12755634	5.87677996	0.90724311	-6.9077553	0	87	4.02335	300.222222	27.2222222	0	0	0	0	42.288408	0	-0.4111751
0.0837	4674.69595	0.72378144	136.25508	1.32320624	0	-2.4805163	1.54237802	-0.3218851	4.91453606	0.28081322	-6.9077553	1	88	2.68223333	300.222222	27.2222222	0	0	0	0	42.288408	0	-0.4111751
0.1692	8847.04528	0.80149359	181.345888	1.62603182	0	-1.7766738	2.18019656	-0.2200314	5.20041171	0.48675738	-6.9077553	2	84	3.57631111	300.777778	27.7777778	0	0	0	0	42.288408	0	-0.4111751
0.3879	17743.3729	0.92509103	242.707948	2.57158997	0	-0.9470077	2.87606844	-0.0767827	5.49186298	0.94491316	-6.9077553	3	78	3.57631111	301.888889	28.88888888	0	0	0	0	42.288408	0	-0.4111751
0.6696	30493.459	0.95531349	252.146765	2.25196736	0	-0.4010748	3.41754499	-0.0446695	5.53001529	0.81224817	-6.9077553	4	76	4.02335	301.888889	28.88888888	0	0	0	0	42.288408	0	-0.4111751
0.9585	42154.3479	0.863961	198.895347	1.72748544	0	-0.0423857	3.74136155	-0.1450709	5.29278382	0.54724556	-6.9077553	5	66	4.02335	304.111111	31.11111111	0	0	1	0	42.288408	0	-0.4111751
1.3149	56459.0168	0.87278293	202.505051	1.68268963	0	0.27376062	4.03353272	-0.1349233	5.31076977	0.52098759	-6.9077553	6	63	3.57631111	304.666667	31.66666666	0	0	1	0	42.288408	0	-0.4111751
1.8171	75796.9788	0.94035476	252.822693	2.59228297	0	0.59724182	4.32807163	-0.0604352	5.53269238	0.95292463	-6.9077553	7	57	5.81150556	305.777778	32.7777778	0	0	1	0	42.288408	0	-0.4111751
2.4201	95063.3236	0.88800204	220.664039	2.52558084	0	0.88380886	4.55455375	-0.1176558	5.39664589	0.92686694	-6.9077553	8	56	5.36446667	305.777778	32.7777778	0	0	1	0	42.288408	0	-0.4111751
3.0879	127243.618	1.01373809	297.500695	2.46783694	0	1.12749125	4.84611136	0.01463054	5.69541992	0.90374717	-6.9077553	9	55	5.81150556	306.333333	33.33333333	0	0	1	0	42.288408	0	-0.4111751
3.7899	163432.011	1.02812664	297.855629	2.22481157	0	1.33233963	5.09640319	0.02871052	5.69661226	0.8001216	-6.9077553	10	59	3.12927222	305.777778	32.7777778	0	0	1	0	42.288408	0	-0.4111751
4.4739	196232.705	0.9695747	265.004461	2.11192145	0	1.49826051	5.27930632	-0.0298669	5.57975043	0.74807156	-6.9077553	11	59	3.12927222	305.222222	32.2222222	0	0	1	0	42.288408	0	-0.4111751
4.968	218639.085	0.90337295	229.876082	1.69967279	0	1.60301734	5.38742693	-0.1005134	5.43754474	0.53102393	-6.9077553	12	65	1.78815556	304.666667	31.66666666	0	0	1	0	42.288408	0	-0.4111751
5.2515	230695.516	0.79710291	172.209648	1.30582382	0	1.65851375	5.44110306	-0.2255177	5.14871842	0.26759963	-6.9077553	13	70	1.34111667	304.111111	31.11111111	0	0	1	0	42.288408	0	-0.4111751
5.454	239597.08	0.7094637	133.945729	1.20053263	0	1.69634928	5.47896285	-0.3418374	4.89744218	0.18359794	-6.9077553	14	73	1.78815556	303	30	0	0	1	0	42.288408	0	-0.4111751
5.5611	244115.063	0.45213578	49.5677767	0.70708568	0	1.71579593	5.49764378	-0.7915635	3.90336113	-0.3451902	-6.9077553	15	76	2.68223333	301.888889	28.88888888	0	0	1	0	42.288408	0	-0.4111751
5.6421	247448.723	0.30728493	22.8638428	0.55634477	0	1.73025634	5.51120742	-1.1767308	3.12960048	-0.5845712	-6.9077553	16	80	2.23519444	301.333333	28.33333333	0	0	1	0	42.288408	0	-0.4111751
5.6988	249685.751	0.26726883	15.9835825	0.46286692	0	1.74025563	5.52020714	-1.3157657	2.77162466	-0.7681576	-6.9077553	17	81	2.68223333	301.333333	28.33333333	0	0	1	0	42.288408	0	-0.4111751
5.7573	251878.926	0.27300292	25.8530611	0.47425244	0	1.75046861	5.52895249	-1.2946165	3.25246769	-0.7439092	-6.9077553	18	83	2.68223333	300.777778	27.7777778	0	0	1	0	42.288408	0	-0.4111751
5.805	253765.069	0.23120078	11.138855	0.30935464	0	1.75871962	5.53641285	-1.4601529	2.41052922	-1.1700396	-6.9077553	19	84	2.68223333	300.777778	27.7777778	0	0	1	0	42.288408	0	-0.4111751
5.8554	255563.487	0.22299872	10.6003527	0.32252724	0	1.76736431	5.54347477	-1.4961149	2.36098161	-1.128472	-6.9077553	20	85	2.23519444	300.222222	27.2222222	0	0	1	0	42.288408	0	-0.4111751
5.9031	257493.516	0.31320255	43.9263801	0.58696041	0	1.77547764	5.55099842	-1.1577174	3.78253782	-0.5310957	-6.9077553	21	84	3.57631111	300.222222	27.2222222	0	0	1	0	42.288408	0	-0.4111751
5.9508	259379.656	0.26275874	24.3590825	0.4096693	0	1.78352566	5.5582967	-1.3327205	3.19294583	-0.889967	-6.9077553	22	84	2.68223333	300.222222	27.2222222	0	0	1	0	42.288408	0	-0.4111751
5.9841	260739.43	0.23898581	16.7523408	0.44124077	0	1.78910595	5.56352539	-1.4271755	2.81859769	-0.8159008	-6.9077553	23	83	3.57631111	300.222222	27.2222222	0	0	1	0	42.288408	0	-0.4111751
0.0009	0	0	0	0	0	-7.0131158	-6.9077553	-6.9077553	-6.9077553	-6.9077553	-6.9077553	0	87	4.02335	300.222222	27.2222222	0	0	0	0	18.9135952	0	-0.0428739
0.0009	0	0	0	0	0	-7.0131158	-6.9077553	-6.9077553	-6.9077553	-6.9077553	-6.9077553	1	88	2.68223333	300.222222	27.2222222	0	0	0	0	18.9135952	0	-0.0428739
0.0009	0	0	0	0	0	-7.0131158	-6.9077553	-6.9077553	-6.9077553	-6.9077553	-6.9077553	2	84	3.57631111	300.777778	27.7777778	0	0	0	0	18.9135952	0	-0.0428739
0.0144	0	0.43947652	50.2750081	1.55957031	0	-4.2405271	-6.9077553	-0.8198981	3.91752799	0.44505134	-6.9077553	3	78	3.57631111	301.888889	28.88888888	0	0	0	0	18.9135952	0	-0.0428739
0.0657	250.747096	0.65504369	125.633224	2.15338536	0	-2.7226564	-1.3793303	-0.4215279	4.8333747	0.76750547	-6.9077553	4	76	4.02335	301.888889	28.88888888	0	0	0	0	18.9135952	0	-0.0428739
0.1737	1355.22987	0.62351042	107.300081	1.87711945	0	-1.7504256	0.3047087	-0.4707873	4.67563873	0.63027098	-6.9077553	5	66	4.02335	304.111111	31.11111111	0	0	1	0	18.9135952	0	-0.0428739
0.3447	6946.62714	0.82591585	223.704688	2.01749091	0	-1.0650808	1.93840018	-0.1900523	5.41033129	0.70235016	-6.9077553	6	63	3.57631111	304.666667	31.66666666	0	0	1	0	18.9135952	0	-0.0428739
0.6264	17120.6291	0.87865665	260.655252	2.70377026	0	-0.4677661	2.84034252	-0.1282236	5.5632025	0.99501698	-6.9077553	7	57	5.81150556	305.777778	32.7777778	0	0	1	0	18.9135952	0	-0.0428739
0.9459	27332.8582	0.93962901	282.366351	2.83016856	0	-0.0556184	3.30812616	-0.0612065	5.64320889	1.04068955	-6.9077553	8	56	5.36446667	305.777778	32.7777778	0	0	1	0	18.9135952	0	-0.0428739
1.3194	41614.6256	0.94439352	271.968694	2.48628577	0	0.27717709	3.72847571	-0.056154	5.60569064	0.91119206	-6.9077553	9	55	5.81150556	306.333333	33.33333333	0	0	1	0	18.9135952	0	-0.0428739
1.6092	53182.4367	0.86802853	224.930318	1.79512886	0	0.47573716	3.97374701	-0.1403793	5.4157951	0.58563372	-6.9077553	10	59	3.12927222	305.777778	32.7777778	0	0	1	0	18.9135952	0	-0.0428739
1.9314	65462.2429	0.8400422	207.735052	1.86392331	0	0.65824513	4.18148881	-0.1731134	5.33626829	0.62321993	-6.9077553	11	59	3.12927222	305.222222	32.2222222	0	0	1	0	18.9135952	0	-0.0428739
2.2653	80568.6759	0.90786348	236.319828	1.7711843	0	0.8177072	4.38912235	-0.0955604	5.46519032	0.57221285	-6.9077553	12	65	1.78815556	304.666667	31.66666666	0	0	1	0	18.9135952	0	-0.0428739
2.4507	89477.1395	0.87336153	206.105184	1.57477014	0	0.8963737	4.49399434	-0.1342613	5.32839149	0.45474413	-6.9077553	13	70	1.34111667	304.111111	31.11111111	0	0	1	0	18.9135952	0	-0.0428739
2.5974	96159.9981	0.76174131	152.656262	1.32514888	0	0.95451094	4.56602385	-0.2708363	5.02819529	0.28227916	-6.9077553	14	73	1.78815556	303	30	0	0	1	0	18.9135952	0	-0.0428739
2.6802	99932.2833	0.48921855	58.9499565	0.84216474	0	0.98589142	4.6045028	-0.712904	4.07670585	-0.1705929	-6.9077553	15	76	2.68223333	301.888889	28.88888888	0	0	1	0	18.9135952	0	-0.0428739
2.7441	102783.424	0.34138688	26.9914877	0.6065057	0	1.00945315	4.63263382	-1.071814	3.2955586	-0.4983937	-6.9077553	16	80	2.23519444	301.333333	28.33333333	0	0	1	0	18.9135952	0	-0.0428739
2.7918	104932.761	0.28513354	18.8243212	0.52527744	0	1.02668655	4.6533293	-1.2512966	2.93520284	-0.6419267	-6.9077553	17	81	2.68223333	301.333333	28.33333333	0	0	1	0	18.9135952	0	-0.0428739
2.8386	107125.948	0.24945257	13.7008479	0.38758128	0	1.04331097	4.67401456	-1.3844857	2.61753071	-0.9452529	-6.9077553	18	83	2.68223333	300.777778	27.7777778	0	0	1	0	18.9135952	0	-0.0428739
2.8836	108924.359	0.22738165	11.4408032	0.3710873	0	1.05903951	4.69066287	-1.4767372	2.4372736	-0.9886268	-6.9077553	19	84	2.68223333	300.777778	27.7777778	0	0	1	0	18.9135952	0	-0.0428739
2.9223	110635.044	0.2450581	12.955318	0.37917332	0	1.07237098	4.70624593	-1.4021876	2.56158355	-0.967128	-6.9077553	20	85	2.23519444	300.222222	27.2222222	0	0	1	0	18.9135952	0	-0.0428739
2.9682	112565.059	0.26158004	17.5057433	0.43584652	0	1.08795571	4.72354024	-1.3371993	2.86258614	-0.8281734	-6.9077553	21	84	3.57631111	300.222222	27.2222222	0	0	1	0	18.9135952	0	-0.0428739
3.0213	114977.566	0.25203425	13.8081975	0.40802419	0	1.1056872	4.74474573	-1.3742304	2.62533486	-0.893981	-6.9077553	22	84	2.68223333	300.222222	27.2222222	0	0	1	0	18.9135952	0	-0.0428739
3.0663	116951.433	0.23919292	13.0507837	0.42416702	0	1.12047162	4.7617673	-1.4263128	2.56892481	-0.8552732	-6.9077553	23	83	3.57631111	300.222222	27.2222222	0	0	1	0	18.9135952	0	-0.0428739
0.0108	407.553541	0.87770228	207.729825	1.51571343	0	-4.5282091	-0.8951323	-0.1293091	5.33624313	0.41654578	-6.9077553	0	70	1.78815556	303	30	0	0	0	0	30.519848	0	0.21141735
0.0216	892.420829	0.77064069	152.126953	1.31470942	0	-3.835062	-0.1126976	-0.2592363	5.02472196	0.27437601	-6.9077553	1	77	0.44703889	301.888889	28.88888888	0	0	0	0	30.519848	0	0.21141735
0.0351	1243.33109	0.65669174	111.681104	1.28433558	0	-3.3495541	0.21859811	-0.4190189	4.71565647	0.25101984	-6.9077553	2	75	0.89407778	297.444444	24.44444444	0	0	0	0	30.519848	0	0.21141735
0.0387	1374.92242	0.55032092	71.5361938	0.89202547	0	-3.2519157	0.31912436	-0.5954382	4.27021751	-0.1131402	-6.9077553	3	88	0.89407778	295.222222	22.2222222	0	0	0	0	30.519848	0	0.21141735
0.0405	1418.78621	0.21500586	9.28873062	0.70394522	0	-3.2064533	0.35050631	-1.5324497	2.22890956	-0.3496352	-6.9077553	4	46	2.23519444	296.888889	23.88888888	0	0	0	0	30.519848	0	0.21141735
0.0891	1769.69639	0.35393906	40.9282633	1.21875226	0	-2.4179959	0.57137291	-1.0358092	3.71184529	0.19864777	-6.9077553	5	42	2.23519444	296.333333	23.33333333	0	0	0	0	30.519848	0	0.21141735

0.1566	2530.88696	0.43602895	55.3811328	1.33096873	0	-1.8540605	0.92896486	-0.8277558	4.01425703	0.2866581	-6.9077553	6	35	3.12927222	295.777778	22.7777778	0	0	0	0	30.519848	0	0.21141735
0.3942	4053.38462	0.71007168	136.880238	3.34101775	0	-0.9308969	1.39979892	-0.340982	4.91911367	1.20657474	-6.9077553	7	36	4.91742778	294.666667	21.66666666	0	0	0	0	30.519848	0	0.21141735
0.5508	6034.11397	0.56092624	85.9071255	1.28975914	0	-0.5963835	1.79759474	-0.5763847	4.45327842	0.25523053	-6.9077553	8	43	2.68223333	293	20	0	0	0	1	30.519848	0	0.21141735
0.6831	7717.78225	0.50369958	74.4250505	0.85352279	0	-0.381114	2.04365661	-0.6837919	4.30980602	-0.1572121	-6.9077553	9	54	0.44703889	290.222222	17.2222222	0	0	0	1	30.519848	0	0.21141735
0.8019	9405.5008	0.45094265	63.2853486	0.81535166	0	-0.2207714	2.24140103	-0.7942	4.14766964	-0.2029101	-6.9077553	10	55	1.34111667	291.333333	18.33333333	0	0	0	1	30.519848	0	0.21141735
1.1142	12467.2437	0.70052731	157.306601	2.12062904	0	0.10813666	2.5231849	-0.3544954	5.05820313	0.75218421	-6.9077553	11	46	3.12927222	294.111111	21.11111111	0	0	0	1	30.519848	0	0.21141735
1.8702	19962.7869	0.97136972	277.4351	4.13523065	0	0.62604538	2.99391998	-0.0280192	5.62559064	1.4197849	-6.9077553	12	40	5.36446667	295.777778	22.7777778	0	0	0	1	30.519848	0	0.21141735
2.7999	29833.871	0.99047863	299.327868	4.36487844	0	1.0295837	3.39567788	-0.0085579	5.70154286	1.47381942	-6.9077553	13	35	6.25854444	297.444444	24.44444444	0	0	0	1	30.519848	0	0.21141735
3.618	48296.7672	0.97536812	276.797236	2.53226846	0	1.28592139	3.87738533	-0.0239156	5.62328885	0.92951035	-6.9077553	14	31	6.70558333	299.111111	26.11111111	0	0	0	1	30.519848	0	0.21141735
4.4955	63762.8302	0.95791279	257.355064	1.88179838	0	1.5030769	4.15518611	-0.0419552	5.55046059	0.63275917	-6.9077553	15	27	6.25854444	300.777778	27.7777778	0	0	0	1	30.519848	0	0.21141735
5.7492	91494.3339	1.11035034	392.307968	3.84668039	14	1.74906071	4.51628798	0.10557579	5.97204971	1.34747047	2.63912876	16	24	7.59966111	301.888889	28.88888888	0	0	0	1	30.519848	0	0.21141735
6.813	272520.533	1.52430542	618.590366	2.91655265	175	1.91883255	5.60771763	0.42219466	6.4274449	1.07074513	5.16479169	17	23	5.36446667	303.555556	30.55555555	0	0	0	1	30.519848	0	0.21141735
8.1648	670579.248	2.0549505	950.640812	3.7395998	548	2.09983223	6.50814338	0.72073827	6.85713735	1.31924597	6.30627711	18	23	5.81150556	303.555556	30.55555555	0	0	0	1	30.519848	0	0.21141735
10.0323	1396806.49	2.56558782	1293.00795	4.67103021	1248	2.30580989	7.24194455	0.94257732	7.1647273	1.54159371	7.12929835	19	22	6.25854444	304.111111	31.11111111	0	0	0	1	30.519848	0	0.21141735
12.393	2141138.5	2.52119453	1296.03431	4.64089008	2027	2.5171318	7.66909344	0.92512937	7.16706512	1.53512163	7.61431264	20	23	5.36446667	304.111111	31.11111111	0	0	0	1	30.519848	0	0.21141735
13.572	2265286.43	1.28350886	493.224857	3.01461881	2135	2.60800885	7.72545693	0.25037644	6.2009672	1.10380505	7.66622239	21	24	4.91742778	303.555556	30.55555555	0	0	0	1	30.519848	0	0.21141735
14.3577	2366575.14	1.29128493	473.129367	2.41730988	2214	2.66428638	7.76919952	0.25641192	6.15937097	0.8830689	7.70255656	22	27	3.57631111	302.444444	29.44444444	0	0	0	1	30.519848	0	0.21141735
14.9508	2500000.4	1.52917518	591.043722	2.28358286	2328	2.70476481	7.82404657	0.42538222	6.38189169	0.82618345	7.75276524	23	46	0.44703889	299.111111	26.11111111	0	0	0	1	30.519848	0	0.21141735
0.0198	936.319094	1.00630097	282.592029	1.99136062	0	-3.9220733	-0.0647315	0.00727445	5.6440078	0.68932018	-6.9077553	0	70	1.78815556	303	30	0	0	0	0	34.0724638	0	0.49277013
0.0342	1374.95689	0.67255586	112.821335	0.94502842	0	-3.3755296	0.31914941	-0.3951843	4.72581432	-0.0554827	-6.9077553	1	77	0.44703889	301.888889	28.88888888	0	0	0	0	34.0724638	0	0.49277013
0.0477	1901.32219	0.45272439	49.6411621	0.65690479	0	-3.0428239	0.64307535	-0.7902653	3.90484052	-0.418695	-6.9077553	2	75	0.89407778	297.444444	24.44444444	0	0	0	0	34.0724638	0	0.49277013
0.0621	2427.68746	0.52438992	70.0994263	0.82976282	0	-2.7790093	0.88735097	-0.6436146	4.24992887	-0.1854109	-6.9077553	3	88	0.89407778	295.222222	22.2222222	0	0	0	0	34.0724638	0	0.49277013
0.0747	2954.05293	0.36158752	32.3636693	0.44997321	0	-2.5942752	1.08351656	-1.0144894	3.47706737	-0.7963473	-6.9077553	4	46	2.23519444	296.888889	23.88888888	0	0	0	0	34.0724638	0	0.49277013
0.1467	6283.04372	0.88907757	222.308057	1.72644615	0	-1.9193656	1.83801368	-0.1164467	5.40406856	0.5466441	-6.9077553	5	42	2.23519444	296.333333	23.33333333	0	0	0	0	34.0724638	0	0.49277013
0.2169	9666.53588	0.91312047	224.518204	1.47710918	0	-1.5283189	2.26877346	-0.0897929	5.41396125	0.39076369	-6.9077553	6	35	3.12927222	295.777778	22.7777778	0	0	0	0	34.0724638	0	0.49277013
0.3267	13185.195	0.90791177	218.731814	2.1200464	0	-1.118713	2.57917045	-0.0955073	5.38785096	0.75190955	-6.9077553	7	36	4.91742778	294.666667	21.66666666	0	0	0	1	34.0724638	0	0.49277013
0.4797	20233.2215	0.96797665	263.224449	1.93162429	0	-0.7345944	3.00737531	-0.0315148	5.57301088	0.65887881	-6.9077553	8	43	2.68223333	293	20	0	0	0	1	34.0724638	0	0.49277013
0.6579	28276.3544	0.9205407	235.121094	1.66189267	0	-0.4187023	3.34206129	-0.0817083	5.46010493	0.50855866	-6.9077553	9	54	0.44703889	290.222222	17.2222222	0	0	0	1	34.0724638	0	0.49277013
0.8748	37689.1867	0.91497157	238.342745	1.70850546	0	-0.13376	3.62939976	-0.08777	5.47371394	0.53620413	-6.9077553	10	55	1.34111667	291.333333	18.33333333	0	0	0	1	34.0724638	0	0.49277013
1.2006	47561.9924	0.92143098	240.701226	2.00070443	0	0.18282143	3.86205499	-0.0807427	5.48356059	0.69399903	-6.9077553	11	46	3.12927222	294.111111	21.11111111	0	0	0	1	34.0724638	0	0.49277013
1.5849	59952.5517	0.98601223	281.135209	2.70813989	0	0.46052131	4.09357012	-0.0130728	5.63883928	0.9966312	-6.9077553	12	40	5.36446667	295.777778	22.7777778	0	0	0	1	34.0724638	0	0.49277013
2.1924	82564.6175	1.07912543	341.998773	2.93409686	0	0.78499683	4.41359334	0.07707718	5.83481007	1.07674045	-6.9077553	13	35	6.25854444	297.444444	24.44444444	0	0	0	1	34.0724638	0	0.49277013
3.0609	117971.233	1.10685124	346.704858	2.92060395	0	1.11870899	4.77044929	0.10242232	5.84847675	1.07213276	-6.9077553	14	31	6.70558333	299.111111	26.11111111	0	0	0	1	34.0724638	0	0.49277013
4.1742	155901.28	1.08668374	338.780214	3.39332022	0	1.42892272	5.0492294	0.08405042	5.82535451	1.22210351	-6.9077553	15	27	6.25854444	300.777778	27.7777778	0	0	0	1	34.0724638	0	0.49277013
5.6232	219366.076	1.18242407	409.025893	3.49057678	0	1.7269009	5.39074647	0.16841199	6.01378091	1.25035343	-6.9077553	16	24	7.59966111	301.888889	28.88888888	0	0	0	1	34.0724638	0	0.49277013
7.1721	279023.122	1.09878581	354.949884	3.30885619	0	1.9701985	5.63129824	0.09511544	5.87197942	1.19690474	-6.9077553	17	23	5.36446667	303.555556	30.55555555	0	0	0	1	34.0724638	0	0.49277013
8.8155	336483.233	1.07149844	346.634789	3.64188287	1	2.17651154	5.81855129	0.06999092	5.84827463	1.29277537	0.0009995	18	23	5.81150556	303.555556	30.55555555	0	0	0	1	34.0724638	0	0.49277013
10.4508	385689.096	0.91501111	253.417102	3.18390279	1	2.34667853	5.95503419	-0.0877268	5.5350407	1.15842177	0.0009995	19	22	6.25854444	304.111111	31.11111111	0	0	0	1	34.0724638	0	0.49277013
11.8656	425322.744	0.90357952	260.519482	3.04047889	1	2.47364346	6.05285063	-0.1002851	5.56268148	1.11234387	0.0009995	20	23	5.36446667	304.111111	31.11111111	0	0	0	1	34.0724638	0	0.49277013
13.4082	467894.579	0.8887646	253.837478	3.13926385	1	2.59586646	6.14824515	-0.1167983	5.53669815	1.14430683	0.0009995	21	24	4.91742778	303.555556	30.55555555	0	0	0	1	34.0724638	0	0.49277013
14.715	511029.915	0.89704731	250.80826	2.56153605	1	2.68886738	6.23643009	-0.1075325	5.52469273	0.94099741	0.0009995	22	27	3.57631111	302.444444	29.44444444	0	0	0	1	34.0724638	0	0.49277013
15.8211	549342.259	0.85019956	213.856031	1.89434015	1	2.76134449	6.30872349	-0.1611087	5.36530771	0.63939832	0.0009995	23	46	0.44703889	299.111111	26.11111111	0	0	0	1	34.0724638	0	0.49277013
0.0324	2067.76496	1.13486546	353.292746	2.31601497	0	-3.4295969	0.72695179	0.12739488	5.86729985	0.84027971	-6.9077553	0	70	1.78815556	303	30	0	0	0	0	42.288408	0	-0.4111751
0.0963	5210.88936	0.86054407	197.931104	1.56295271	0	-2.340287	1.65094243	-0.1490291	5.28792406	0.44721641	-6.9077553	1	77	0.44703889	301.888889	28.88888888	0	0	0	0	42.288408	0	-0.4111751
0.1548	7974.30748	0.62058129	96.1458158	1.06994793	0	-1.8656213	2.0763502	-0.4754886	4.56587635	0.06854417	-6.9077553	2	75	0.89407778	297.444444	24.44444444	0	0	0	0	42.288408	0	-0.4111751
0.2115	10693.8628	0.61989702	97.7813043	1.06214711	0	-1.5535303	2.36976352	-0.47659	4.58274362	0.06123348	-6.9077553	3	88	0.89407778	295.222222	22.2222222	0	0	0	0	42.288408	0	-0.4111751
0.2484	12413.5831	0.45318948	64.6698861	0.83166839	0	-1.3927149	2.51887184	-0.7892408	4.16931112	-0.1831198	-6.9077553	4	46	2.23519444	296.888889	23.88888888	0	0	0	0	42.288408	0	-0.4111751
0.4572	22529.9715	0.9449724	261.495252	1.91727671	0	-0.7826343	3.11489087	-0.0555419	5.56641995	0.65142724	-6.9077553	5	42	2.23519444	296.333333	23.33333333	0	0	0	0	42.288408	0	-0.4111751
0.7722	37889.1855	0.99803737	286.099955	2.11660209	0	-0.2585117	3.63469212	-0.0009631	5.65634474	0.75028436	-6.9077553	6	35	3.12927222	295.777778	22.7777778	0	0	1	0	42.288408	0	-0.4111751
1.1943	53122.2209	0.9155127	234.498801	2.31091335	0	0.17756024	3.97261414	-0.0871794	5.45745474	0.83807547	-6.9077553	7	36	4.91742778	294.666667	21.66666666	0	0	1	0	42.288408	0	-0.4111751
1.5066	65481.3627	0.80239384	173.066778	1.42211668	0	0.40985546	4.18178084	-0.2189102	5.1536833	0.35284931	-6.9077553	8	43	2.68223333	293	20	0	0	1	0	42.288408	0	-0.4111751
1.8405	78377.9035	0.79414865	173.788494	1.35420889	0	0.61003727	4.3615548	-0.2292262	5.15784476	0.30395561	-6.9077553	9	54	0.44703889	290.222222	17.2222222	0	0	1	0	42.288408	0	-0.4111751
2.1465	90106.2039	0.73977419	151.884214	1.19370979	0	0.76383861	4.50100012	-0.3000594	5.02312507	0.1779033	-6.9077553	10	55	1.34111667	291.333333	18.33333333	0	0	1	0	42.288408	0	-0.4111751
2.6532	107547.533	0.8146294	185.686293	1.94307534	0	0.97576646	4.67794222	-0.2037952	5.22406404	0.66478646	-6.9077553	11	46	3.12927222	294.111111	21.11111111	0	0	1	0	42.288408	0	-0.4111751
3.6108	135997.578	0.90620204	243.613692	3.24546504	0	1.28392935	4.91264443	-0.0973901	5.49558785	1.17756673	-6.9077553	12	40	5.36446667	295.777778	22.7777778	0	0	1	0	42.288408	0	-0.4111751
5.0778	166696.858	0.84587507	222.212117	3.84337016	0	1.6248781	5.11618294	-0.1662021	5.40363691	1.34660978	-6.9077553	13	35	6.25854444	297.444444	24.44444444	0	0	1	0	42.288408	0	-0.4111751
6.2037	197269.966	0.97308384	302.905426	4.32683502	1	1.82514589	5.28457825	-0.0262579	5.71342393	1.46506742	0.0009995	14	31	6.70558333	299.111111	26.11111111	0	0	1	0	42.288408	0	-0.4111751
6.2037	197269.966	0	0	0	1	1.82514589	5.28457825	-6.9077553	-6.9077553	-6.9077553	0.0009995	15	27	6.25854444	300.777778	27.7777778	0	0	1	0	42.288408	0	-0.4111751
6.2037	197269.966	0	0	0	1	1.82514589	5.28457825	-6.9077553	-6.9077553	-6.9077553	0.0009995	16	24	7.59966111	301.888889	28.88888888	0	0	1	0	42.288408	0	-0.4111751
6.2037	197269.966	0	0	0	1	1.82514589	5.28457825	-6.9077553	-6.9077553	-6.9077553	0.0009995	17	23	5.36446667	303.555556	30.55555555	0	0	1	0	42.288408	0	-0.4111751
6.2037	197269.966	0	0	0	1	1.82514589	5.28457825	-6.9077553	-6.9077553	-6.9077553	0.0009995	18	23	5.81150556	303.555556	30.55555555	0	0	1	0	42.288408	0	-0.4111751
6.2037	197269.966	0	0	0	1	1.82514589	5.28457825	-6.9077553	-6.9077553	-6.9077553	0.0009995	19	22	6.25854444	304.111111	31.11111111	0	0	1	0	42.288408	0	-0.4111751

6.2037	197269.966	0	0	0	1	1.82514589	5.28457825	-6.9077553	-6.9077553	-6.9077553	0.0009995	20	23	5.36446667	304.111111	31.11111111	0	0	1	0	42.288408	0	-0.4111751
6.2037	197269.966	0	0	0	1	1.82514589	5.28457825	-6.9077553	-6.9077553	-6.9077553	0.0009995	21	24	4.91742778	303.555556	30.55555555	0	0	1	0	42.288408	0	-0.4111751
6.2037	197269.966	0	0	0	1	1.82514589	5.28457825	-6.9077553	-6.9077553	-6.9077553	0.0009995	22	27	3.57631111	302.444444	29.44444444	0	0	1	0	42.288408	0	-0.4111751
6.2037	197269.966	0	0	0	1	1.82514589	5.28457825	-6.9077553	-6.9077553	-6.9077553	0.0009995	23	46	0.44703889	299.111111	26.11111111	0	0	1	0	42.288408	0	-0.4111751
0.0018	0	0.1061632	1.98204601	0.29212055	0	-6.3199686	-6.9077553	-2.2334023	0.68463405	-1.2271713	-6.9077553	0	70	1.78815556	303	30	0	0	0	0	18.9135952	0	-0.0428739
0.0018	0	0	0	0	0	-6.3199686	-6.9077553	-6.9077553	-6.9077553	-6.9077553	-6.9077553	1	77	0.44703889	301.888889	28.88888888	0	0	0	0	18.9135952	0	-0.0428739
0.0018	0	0	0	0	0	-6.3199686	-6.9077553	-6.9077553	-6.9077553	-6.9077553	-6.9077553	2	75	0.89407778	297.444444	24.44444444	0	0	0	0	18.9135952	0	-0.0428739
0.0018	0	0	0	0	0	-6.3199686	-6.9077553	-6.9077553	-6.9077553	-6.9077553	-6.9077553	3	88	0.89407778	295.222222	22.2222222	0	0	0	0	18.9135952	0	-0.0428739
0.0018	0	0	0	0	0	-6.3199686	-6.9077553	-6.9077553	-6.9077553	-6.9077553	-6.9077553	4	46	2.23519444	296.888889	23.88888888	0	0	0	0	18.9135952	0	-0.0428739
0.0171	0	0.36484831	37.8692663	1.41049351	0	-4.0686768	-6.9077553	-1.0055365	3.63416627	0.34464837	-6.9077553	5	42	2.23519444	296.333333	23.33333333	0	0	0	0	18.9135952	0	-0.0428739
0.0972	0	0.47608151	57.4275259	2.00378418	0	-2.3309846	-6.9077553	-0.7400679	4.05054115	0.69553641	-6.9077553	6	35	3.12927222	295.777778	22.7777778	0	0	0	0	18.9135952	0	-0.0428739
0.2178	131.59135	0.39098455	39.1004183	1.70950431	0	-1.5241781	-2.0204834	-0.9365329	3.66615874	0.53678825	-6.9077553	7	36	4.91742778	294.666667	21.66666666	0	0	1	0	18.9135952	0	-0.0428739
0.3123	526.365399	0.37641322	46.0201497	1.18669099	0	-1.163791	-0.6398616	-0.9744146	3.82910107	0.17201108	-6.9077553	8	43	2.68223333	293	20	0	0	1	0	18.9135952	0	-0.0428739
0.3744	1327.37809	0.42741773	69.5366989	0.78618622	0	-0.9824305	0.28395871	-0.8476566	4.24186904	-0.2392904	-6.9077553	9	54	0.44703889	290.222222	17.2222222	0	0	1	0	18.9135952	0	-0.0428739
0.4536	3400.53219	0.56979015	118.847934	1.03794609	0	-0.7905395	1.22422598	-0.5607337	4.77785322	0.03820683	-6.9077553	10	55	1.34111667	291.333333	18.33333333	0	0	1	0	18.9135952	0	-0.0428739
0.6759	6237.75413	0.61135833	127.763727	1.88533842	0	-0.3917101	1.8307805	-0.4904377	4.8501905	0.6346376	-6.9077553	11	46	3.12927222	294.111111	21.11111111	0	0	1	0	18.9135952	0	-0.0428739
1.1034	13267.8004	0.83370156	239.748289	3.33771973	0	0.09839632	2.58541545	-0.180681	5.47959375	1.20558742	-6.9077553	12	40	5.36446667	295.777778	22.7777778	0	0	1	0	18.9135952	0	-0.0428739
1.7343	30812.7788	0.97247985	295.802247	4.49681747	0	0.55060387	3.42796195	-0.0268782	5.68969453	1.50359227	-6.9077553	13	35	6.25854444	297.444444	24.44444444	0	0	1	0	18.9135952	0	-0.0428739
2.5389	65505.9894	1.09205208	387.21057	3.89139593	0	0.93173092	4.18215685	0.08897385	5.95897124	1.35902489	-6.9077553	14	31	6.70558333	299.111111	26.11111111	0	0	1	0	18.9135952	0	-0.0428739
3.2175	94906.866	1.0577459	341.447986	3.05337613	0	1.16860466	4.55290659	0.05708509	5.83319828	1.11657536	-6.9077553	15	27	6.25854444	300.777778	27.7777778	0	0	1	0	18.9135952	0	-0.0428739
4.2426	144008.341	1.21020626	461.20698	4.09185646	0	1.44517629	4.96987817	0.19161677	6.13384909	1.40924313	-6.9077553	16	24	7.59966111	301.888889	28.88888888	0	0	1	0	18.9135952	0	-0.0428739
5.2263	191501.891	1.11871355	375.076853	2.98018775	1	1.65370357	5.2549029	0.11307289	5.92713361	1.0923218	0.0009995	17	23	5.36446667	303.555556	30.55555555	0	0	1	0	18.9135952	0	-0.0428739
6.1938	237118.972	1.10157999	367.454826	3.32431459	1	1.82354879	5.46856622	0.09765288	5.90660311	1.20156428	0.0009995	18	23	5.81150556	303.555556	30.55555555	0	0	1	0	18.9135952	0	-0.0428739
7.2603	287777.754	1.12507407	388.540269	3.39197859	1	1.98242115	5.66219197	0.11873731	5.96239939	1.22170818	0.0009995	19	22	6.25854444	304.111111	31.11111111	0	0	1	0	18.9135952	0	-0.0428739
8.2017	331890.974	1.05686913	337.215165	2.59322064	1	2.10434145	5.80480954	0.05625663	5.82072416	0.95328614	0.0009995	20	23	5.36446667	304.111111	31.11111111	0	0	1	0	18.9135952	0	-0.0428739
9.0468	369250.465	0.96826315	266.28308	2.0986748	1	2.2024111	5.91147789	-0.0312191	5.58456371	0.74178248	0.0009995	21	24	4.91742778	303.555556	30.55555555	0	0	1	0	18.9135952	0	-0.0428739
9.9027	409628.24	0.97325312	261.435972	1.86041035	1	2.29280745	6.01525246	-0.0260841	5.56619323	0.62133445	0.0009995	22	27	3.57631111	302.444444	29.44444444	0	0	1	0	18.9135952	0	-0.0428739
10.8189	451695.935	0.95526315	256.367341	1.95890271	1	2.3812946	6.11301146	-0.0447221	5.54661524	0.67289484	0.0009995	23	46	0.44703889	299.111111	26.11111111	0	0	1	0	18.9135952	0	-0.0428739
0.0135	364.502375	0.82562453	184.660086	1.70548194	0	-4.3050656	-1.0064825	-0.1904047	5.21852218	0.53443391	-6.9077553	0	89	3.57631111	291.888889	18.88888888	0	0	0	0	30.519848	0	0.21141735
0.054	1026.33236	0.49193442	66.1624891	1.71937748	0	-2.9187712	0.0269655	-0.7073791	4.19212879	0.54254373	-6.9077553	1	79	5.36446667	294.111111	21.11111111	0	0	0	0	30.519848	0	0.21141735
0.1611	1858.2972	0.57797177	99.7575335	2.50441582	0	-1.82573	0.62019857	-0.5465016	4.6027526	0.91845472	-6.9077553	2	73	6.70558333	295.222222	22.2222222	0	0	0	0	30.519848	0	0.21141735
0.2736	2919.51951	0.6480545	123.141156	2.61685474	0	-1.2960881	1.07176152	-0.4322386	4.81333943	0.96235518	-6.9077553	3	66	4.91742778	297.444444	24.44444444	0	0	0	0	30.519848	0	0.21141735
0.3996	3851.82347	0.600263	98.1736049	2.0106611	0	-0.9172912	1.34880625	-0.5087228	4.58674758	0.6989608	-6.9077553	4	64	5.36446667	298	25	0	0	0	0	30.519848	0	0.21141735
0.5868	5801.6395	0.59086649	96.8517503	1.79510483	0	-0.5330712	1.7583129	-0.5244742	4.57319179	0.58562034	-6.9077553	5	59	4.91742778	299.111111	26.11111111	0	0	0	1	30.519848	0	0.21141735
0.8577	15727.9736	0.69460147	134.397347	2.28770614	0	-0.1535009	2.75550446	-0.3629784	4.90080813	0.82798665	-6.9077553	6	53	6.25854444	300.222222	27.2222222	0	0	0	1	30.519848	0	0.21141735
1.152	31283.9061	0.76039142	168.266449	2.18628378	0	0.14149956	3.44313575	-0.2726077	5.12555467	0.78266049	-6.9077553	7	50	6.25854444	300.777778	27.7777778	0	0	0	1	30.519848	0	0.21141735
1.4103	48086.8946	0.68868216	129.47233	1.44605141	0	0.34380245	3.87303047	-0.3715244	4.86347491	0.36952797	-6.9077553	8	53	4.91742778	300.222222	27.2222222	0	0	0	1	30.519848	0	0.21141735
1.638	63438.7406	0.61944851	110.284052	1.11590914	0	0.49347599	4.15009049	-0.4773127	4.7030684	0.11056517	-6.9077553	9	53	4.91742778	299.666667	26.66666666	0	0	0	1	30.519848	0	0.21141735
1.8009	75593.5553	0.57163066	99.711477	0.85549851	0	0.58828654	4.32538426	-0.5575143	4.60229081	-0.1549027	-6.9077553	10	57	3.57631111	298	25	0	0	0	1	30.519848	0	0.21141735
1.908	90432.5418	0.70551153	131.027418	0.84760893	0	0.64605557	4.50461524	-0.3474158	4.87541423	-0.1641568	-6.9077553	11	67	1.78815556	295.777778	22.7777778	0	0	0	1	30.519848	0	0.21141735
2.0151	102384.58	0.70089952	132.458123	1.01727353	0	0.70066882	4.62874588	-0.353965	4.88627409	0.01810857	-6.9077553	12	70	1.78815556	295.222222	22.2222222	0	0	0	1	30.519848	0	0.21141735
2.1177	113868.499	0.73248619	143.444739	1.13735119	0	0.75033059	4.73505304	-0.3099465	4.96595684	0.12958089	-6.9077553	13	75	1.78815556	294.111111	21.11111111	0	0	0	1	30.519848	0	0.21141735
2.1771	117080.367	0.60325848	95.2950772	1.01472936	0	0.77799372	4.76286914	-0.5037532	4.55698865	0.01560693	-6.9077553	14	79	2.23519444	293.555556	20.55555555	0	0	0	1	30.519848	0	0.21141735
2.1996	118045.375	0.28168407	17.5265186	0.39848793	0	0.78827553	4.77107756	-1.2634253	2.86377213	-0.9175717	-6.9077553	15	83	3.12927222	293	20	0	0	0	1	30.519848	0	0.21141735
2.2113	118571.732	0.19741522	7.78841811	0.24422055	0	0.79358058	4.77552655	-1.6173934	2.05276616	-1.4055973	-6.9077553	16	87	3.57631111	293	20	0	0	0	1	30.519848	0	0.21141735
2.2239	119141.963	0.18595406	6.79531479	0.22005643	0	0.79926241	4.78032414	-1.6768924	1.91638052	-1.5093373	-6.9077553	17	91	3.57631111	292.444444	19.44444444	0	0	0	1	30.519848	0	0.21141735
2.2374	119756.072	0.16013306	5.16371511	0.21783978	0	0.80531448	4.78546529	-1.8255248	1.64184994	-1.5194154	-6.9077553	18	94	3.12927222	291.888889	18.88888888	0	0	0	1	30.519848	0	0.21141735
2.2473	120106.977	0.17052393	5.65415261	0.21668915	0	0.8097295	4.78839114	-1.7630325	1.7325671	-1.5246871	-6.9077553	19	93	4.02335	291.888889	18.88888888	0	0	0	1	30.519848	0	0.21141735
2.2554	120501.747	0.1436966	3.9093043	0.19617142	0	0.81332734	4.79167255	-1.9331161	1.3636152	-1.6236818	-6.9077553	20	90	4.91742778	291.888889	18.88888888	0	0	0	1	30.519848	0	0.21141735
2.2626	120764.92	0.14469275	3.96148181	0.17037094	0	0.81651459	4.79385413	-1.9262553	1.37687055	-1.7639249	-6.9077553	21	90	3.57631111	291.888889	18.88888888	0	0	0	1	30.519848	0	0.21141735
2.2716	121115.842	0.15396161	4.49748917	0.20145242	0	0.82048443	4.79675572	-1.8645779	1.5037416	-1.5972504	-6.9077553	22	89	4.47038889	291.888889	18.88888888	0	0	0	1	30.519848	0	0.21141735
2.2788	121422.883	0.1512315	4.54074764	0.19016662	0	0.82364899	4.79928759	-1.8823529	1.51331188	-1.6546099	-6.9077553	23	90	4.47038889	291.888889	18.88888888	0	0	0	1	30.519848	0	0.21141735
0.0243	911.598202	0.95891314	263.051082	2.60165376	0	-3.7172789	-0.0914596	-0.0409125	5.57235204	0.9565316	-6.9077553	0	89	3.57631111	291.888889	18.88888888	0	0	0	0	34.0724638	0	0.49277013
0.0702	2687.86811	0.73320426	151.375574	1.442777	0	-2.656407	0.98912033	-0.308968	5.0197706	0.36726259	-6.9077553	1	79	5.36446667	294.111111	21.11111111	0	0	0	0	34.0724638	0	0.49277013
0.1746	6869.84139	0.93108874	231.680445	2.2498924	0	-1.7452576	1.92728667	-0.0703273	5.44536335	0.81132676	-6.9077553	2	73	6.70558333	295.222222	22.2222222	0	0	0	1	34.0724638	0	0.49277013
0.2781	10747.4541	0.85653978	191.383084	1.50619016	0	-1.2797745	2.37476194	-0.1536877	5.25428232	0.4102471	-6.9077553	3	66	4.91742778	297.444444	24.44444444	0	0	0	1	34.0724638	0	0.49277013
0.4608	20988.9237	1.20142218	413.381927	3.97811574	0	-0.7747912	3.0440425	0.18433801	6.02437435	1.38105962	-6.9077553	4	64	5.36446667	298	25	0	0	0	1	34.0724638	0	0.49277013
0.8334	41526.9973	1.19042571	403.248113	3.27656504	0	-0.1822416	3.72636783	0.17515066	5.99955452	1.18710078	-6.9077553	5	59	4.91742778	299.111111	26.11111111	0	0	0	1	34.0724638	0	0.49277013
1.4319	60636.561	1.31616229	534.60188	4.52279356	2	0.35900223	4.10491452	0.27547964	6.28152419	1.50935092	0.69364706	6	53	6.25854444	300.222222	27.2222222	0	0	0	1	34.0724638	0	0.49277013
2.2203	81615.4867	1.22369078	459.460688	4.09063498	2	0.79764232	4.40203128	0.20268839	6.13005556	1.40894464	0.69364706	7	50	6.25854444	300.777778	27.7777778	0	0	0	1	34.0724638	0	0.49277013
2.8017	101062.347	0.98959861	281.893624	2.40317519	2	1.03022638	4.61574752	-0.0094459	5.64153333	0.87720689	0.69364706	8	53	4.91742778	300.222222	27.2222222	0	0	0	1	34.0724638	0	0.49277013
3.348	122654.843	0.9269187	256.877986	2.16474605	2	1.20836315	4.80938241	-0.0748112	5.5486051	0.7727649	0.69364706	9	53	4.91742778	299.666667	26.66666666	0	0	0	1	34.0724638	0	0.49277013

3.6549	135258.196	0.82270486	197.375137	1.43723393	2	1.29606873	4.90719291	-0.193943	5.28511124	0.36341593	0.69364706	10	57	3.57631111	298	25	0	0	0	1	34.0724638	0	0.49277013
3.8736	144120.962	0.82991323	191.236272	1.41384398	2	1.35418431	4.9706599	-0.1852299	5.25351492	0.34701926	0.69364706	11	67	1.78815556	295.777778	22.7777778	0	0	0	1	34.0724638	0	0.49277013
4.0374	149551.596	0.78546998	167.22242	1.43121858	2	1.39560092	5.00764814	-0.2402007	5.11933076	0.35922469	0.69364706	12	70	1.78815556	295.222222	22.2222222	0	0	0	1	34.0724638	0	0.49277013
4.1859	154296.323	0.75308163	152.453527	1.47514852	2	1.43172173	5.03888141	-0.2822547	5.02686637	0.38943634	0.69364706	13	75	1.78815556	294.111111	21.1111111	0	0	0	1	34.0724638	0	0.49277013
4.2768	157147.482	0.59907154	108.951655	1.17948566	2	1.45320507	5.0571911	-0.5107064	4.69091343	0.16592593	0.69364706	14	79	2.23519444	293.555556	20.5555555	0	0	0	1	34.0724638	0	0.49277013
4.3371	158507.266	0.53395092	118.568622	1.3688917	2	1.46720592	5.06580674	-0.6255803	4.77550031	0.31473169	0.69364706	15	83	3.12927222	293	20	0	0	0	1	34.0724638	0	0.49277013
4.3632	159296.795	0.3105814	37.8214995	0.77005893	2	1.47320573	5.07077538	-1.1660946	3.63290415	-0.2599905	0.69364706	16	87	3.57631111	293	20	0	0	0	1	34.0724638	0	0.49277013
4.3956	160305.677	0.33920836	50.5268419	0.66797839	2	1.48060404	5.07708871	-1.078197	3.92252451	-0.4020035	0.69364706	17	91	3.57631111	292.444444	19.4444444	0	0	0	1	34.0724638	0	0.49277013
4.4388	161358.403	0.50069431	117.35616	1.30926887	2	1.49038407	5.0836342	-0.6897643	4.76522194	0.27023236	0.69364706	18	94	3.12927222	291.888889	18.8888888	0	0	0	1	34.0724638	0	0.49277013
4.4649	162279.545	0.27336675	30.8413928	0.42887375	2	1.49624682	5.0893266	-1.2932896	3.42889013	-0.8442637	0.69364706	19	93	4.02335	291.888889	18.8888888	0	0	0	1	34.0724638	0	0.49277013
4.4883	163288.406	0.15654393	4.88222357	0.20921538	2	1.50147401	5.09552412	-1.8480509	1.58580557	-1.5596226	0.69364706	20	90	4.91742778	291.888889	18.8888888	0	0	0	1	34.0724638	0	0.49277013
4.5108	164341.141	0.15440642	4.91611328	0.22416445	2	1.50647452	5.10195048	-1.8617115	1.59272163	-1.4909243	0.69364706	21	90	3.57631111	291.888889	18.8888888	0	0	0	1	34.0724638	0	0.49277013
4.5288	165130.693	0.17494659	7.98389511	0.23881617	2	1.510457	5.10674329	-1.7375748	2.07755165	-1.4278826	0.69364706	22	89	4.47038889	291.888889	18.8888888	0	0	0	1	34.0724638	0	0.49277013
4.5657	166271.145	0.36948986	50.225056	0.58274613	2	1.51857184	5.11362588	-0.9929292	3.91653394	-0.5382891	0.69364706	23	90	4.47038889	291.888889	18.8888888	0	0	0	1	34.0724638	0	0.49277013
0.0414	2850.52037	1.19582587	399.876302	2.62945947	0	-3.1844744	1.04785231	0.17967295	5.99115776	0.96715853	-6.9077553	0	89	3.57631111	291.888889	18.8888888	0	0	0	0	42.288408	0	-0.4111751
0.171	8200.73504	1.00826465	293.63482	3.57541105	0	-1.7660917	2.10434572	0.00922199	5.68234029	1.2743598	-6.9077553	1	79	5.36446667	294.111111	21.1111111	0	0	0	0	42.288408	0	-0.4111751
0.4086	19269.1427	1.03323341	298.002308	2.58051971	0	-0.8950186	2.95855689	0.03366049	5.69710459	0.94837826	-6.9077553	2	73	6.70558333	295.222222	22.2222222	0	0	0	0	42.288408	0	-0.4111751
0.6885	30146.5041	0.93180744	230.392886	2.27432143	0	-0.37324	3.40610214	-0.0695565	5.43979039	0.82212133	-6.9077553	3	66	4.91742778	297.444444	24.4444444	0	0	0	0	42.288408	0	-0.4111751
1.0143	43728.5953	0.88386991	206.353872	1.76481198	0	0.01419872	3.77802511	-0.1223146	5.32959736	0.56861063	-6.9077553	4	64	5.36446667	298	25	0	0	0	0	42.288408	0	-0.4111751
1.4679	59837.6017	0.90924993	228.731414	2.51847573	0	0.38383281	4.09165097	-0.0940361	5.43255282	0.92405084	-6.9077553	5	59	4.91742778	299.111111	26.1111111	0	0	0	0	42.288408	0	-0.4111751
2.1024	88026.8671	1.00356333	291.593484	2.85019497	0	0.74307955	4.47765344	0.00455295	5.67536408	1.04773819	-6.9077553	6	53	6.25854444	300.222222	27.2222222	0	0	1	0	42.288408	0	-0.4111751
2.7225	119910.531	1.03112527	306.103728	2.29689219	0	1.00155058	4.78675423	0.03162005	5.72392729	0.83199226	-6.9077553	7	50	6.25854444	300.777778	27.7777778	0	0	1	0	42.288408	0	-0.4111751
3.3534	147222.475	0.97997559	284.415723	2.42376822	0	1.20997476	4.99195167	-0.0192077	5.6504405	0.88573594	-6.9077553	8	53	4.91742778	300.222222	27.2222222	0	0	1	0	42.288408	0	-0.4111751
3.8331	167472.628	0.8778071	224.459897	1.78248204	0	1.34367388	5.12082589	-0.1291899	5.41370151	0.57856766	-6.9077553	9	53	4.91742778	299.666667	26.6666666	0	0	1	0	42.288408	0	-0.4111751
4.2057	186418.958	0.94787509	262.489998	1.8639258	0	1.43644075	5.22800197	-0.0524781	5.57021679	0.62322127	-6.9077553	10	57	3.57631111	298	25	0	0	1	0	42.288408	0	-0.4111751
4.3857	195260.627	0.87522743	210.119453	1.4791507	0	1.47834925	5.27434034	-0.1321296	5.34768095	0.3921439	-6.9077553	11	67	1.78815556	295.777778	22.7777778	0	0	1	0	42.288408	0	-0.4111751
4.5585	202911.748	0.82613571	181.966105	1.40350342	0	1.51699362	5.31277608	-0.1897865	5.20382593	0.3396838	-6.9077553	12	70	1.78815556	295.222222	22.2222222	0	0	1	0	42.288408	0	-0.4111751
4.7475	211228.952	0.77345683	158.629167	1.33666382	0	1.55761816	5.35294736	-0.2555934	5.0665755	0.29092467	-6.9077553	13	75	1.78815556	294.111111	21.1111111	0	0	1	0	42.288408	0	-0.4111751
4.8843	216843.542	0.59891108	93.9103747	1.01895041	0	1.58602598	5.3791807	-0.5109738	4.54235152	0.01975401	-6.9077553	14	79	2.23519444	293.555556	20.5555555	0	0	1	0	42.288408	0	-0.4111751
4.9329	218905.117	0.22301727	10.4735537	0.34317002	0	1.59592705	5.38864295	-1.4960321	2.34894886	-1.0666195	-6.9077553	15	83	3.12927222	293	20	0	0	1	0	42.288408	0	-0.4111751
4.9761	220659.674	0.19122744	7.71351878	0.29394313	0	1.60464645	5.39662611	-1.649076	2.04310411	-1.2209727	-6.9077553	16	87	3.57631111	293	20	0	0	1	0	42.288408	0	-0.4111751
5.0166	222414.23	0.17862284	6.61872355	0.29588983	0	1.61275241	5.40454604	-1.716896	1.89005361	-1.2143941	-6.9077553	17	91	3.57631111	292.444444	19.4444444	0	0	1	0	42.288408	0	-0.4111751
5.0616	224256.517	0.17547636	6.1935321	0.25444695	0	1.62168264	5.41279502	-1.7345683	1.82366698	-1.3647405	-6.9077553	18	94	3.12927222	291.888889	18.8888888	0	0	1	0	42.288408	0	-0.4111751
5.1039	225923.331	0.18154367	6.87450458	0.29706362	0	1.63000495	5.42020012	-1.7007658	1.92796503	-1.2104483	-6.9077553	19	93	4.02335	291.888889	18.8888888	0	0	1	0	42.288408	0	-0.4111751
5.1381	227414.705	0.18460749	7.29923208	0.32312556	0	1.63668336	5.42677964	-1.6841211	1.98790614	-1.1266243	-6.9077553	20	90	4.91742778	291.888889	18.8888888	0	0	1	0	42.288408	0	-0.4111751
5.1822	229388.57	0.16545566	5.88777565	0.29336649	0	1.64522968	5.43542174	-1.7930263	1.77304811	-1.2229297	-6.9077553	21	90	3.57631111	291.888889	18.8888888	0	0	1	0	42.288408	0	-0.4111751
5.2209	231143.126	0.1619426	5.51177943	0.24908647	0	1.6526698	5.44304144	-1.8143573	1.70706893	-1.3859485	-6.9077553	22	89	4.47038889	291.888889	18.8888888	0	0	1	0	42.288408	0	-0.4111751
5.2623	232853.793	0.17670169	8.94511613	0.27248076	0	1.66056819	5.45041505	-1.727649	2.19121949	-1.296524	-6.9077553	23	90	4.47038889	291.888889	18.8888888	0	0	1	0	42.288408	0	-0.4111751
0.0081	0	0.42014515	46.4625969	1.70399749	0	-4.8158912	-6.9077553	-0.8647777	3.83866914	0.53356364	-6.9077553	0	89	3.57631111	291.888889	18.8888888	0	0	0	0	18.9135952	0	-0.0428739
0.0081	0	0	0	0	0	-4.8158912	-6.9077553	-6.9077553	-6.9077553	-6.9077553	-6.9077553	1	79	5.36446667	294.111111	21.1111111	0	0	0	0	18.9135952	0	-0.0428739
0.0756	0	0.52301219	74.0915039	2.77609558	0	-2.582299	-6.9077553	-0.6462403	4.30531437	1.02140563	-6.9077553	2	73	6.70558333	295.222222	22.2222222	0	0	0	0	18.9135952	0	-0.0428739
0.2907	3786.88636	0.77706545	194.517897	2.88806918	0	-1.2354635	1.33180818	-0.2509446	5.27052931	1.06093437	-6.9077553	3	66	4.91742778	297.444444	24.4444444	0	0	1	0	18.9135952	0	-0.0428739
0.4887	10803.8993	0.84912324	247.51843	2.33078058	0	-0.7160065	2.37999967	-0.1623739	5.51148909	0.84663217	-6.9077553	4	64	5.36446667	298	25	0	0	1	0	18.9135952	0	-0.0428739
0.6417	14131.9622	0.72343176	182.132617	1.64851792	0	-0.4436344	2.64850981	-0.3223677	5.20474058	0.50048308	-6.9077553	5	59	4.91742778	299.111111	26.1111111	0	0	1	0	18.9135952	0	-0.0428739
0.8271	18556.9472	0.73725987	159.443454	1.83341106	0	-0.1898297	2.92089812	-0.3034594	5.07169561	0.60672348	-6.9077553	6	53	6.25854444	300.222222	27.2222222	0	0	1	0	18.9135952	0	-0.0428739
1.0449	25804.433	0.78187019	183.6788	1.56422904	0	0.04392119	3.25058505	-0.2447884	5.21319402	0.44803216	-6.9077553	7	50	6.25854444	300.777778	27.7777778	0	0	1	0	18.9135952	0	-0.0428739
1.3122	35805.6642	0.84117871	216.956821	1.85522173	0	0.27170512	3.57813403	-0.171763	5.37970296	0.6185431	-6.9077553	8	53	4.91742778	300.222222	27.2222222	0	0	1	0	18.9135952	0	-0.0428739
1.5183	42820.0612	0.76114025	173.470268	1.498579	0	0.41759129	3.75703007	-0.2716247	5.15601198	0.4051844	-6.9077553	9	53	4.91742778	299.666667	26.6666666	0	0	1	0	18.9135952	0	-0.0428739
1.728	51209.7555	0.85150756	208.615477	1.54647094	0	0.54696467	3.93594958	-0.1595732	5.34049753	0.43662194	-6.9077553	10	57	3.57631111	298	25	0	0	1	0	18.9135952	0	-0.0428739
1.8918	58589.6727	0.90402993	221.834607	1.61761709	0	0.63752876	4.07057552	-0.0997873	5.4019366	0.48157214	-6.9077553	11	67	1.78815556	295.777778	22.7777778	0	0	1	0	18.9135952	0	-0.0428739
2.0151	64089.1369	0.8046792	174.250171	1.41564173	0	0.70066882	4.16029048	-0.2160696	5.16049777	0.34828909	-6.9077553	12	70	1.78815556	295.222222	22.2222222	0	0	1	0	18.9135952	0	-0.0428739
2.1339	69421.7872	0.75405225	155.062574	1.34844335	0	0.75795129	4.24021516	-0.2809683	5.04383519	0.29969217	-6.9077553	13	75	1.78815556	294.111111	21.1111111	0	0	1	0	18.9135952	0	-0.0428739
2.2131	72930.8887	0.57349066	84.4498624	0.92002002	0	0.79439425	4.28952597	-0.5542714	4.43616986	-0.0822735	-6.9077553	14	79	2.23519444	293.555556	20.5555555	0	0	1	0	18.9135952	0	-0.0428739
2.2482	74466.1226	0.2338539	11.8970157	0.45128852	0	0.8101299	4.31035772	-1.4487916	2.47637164	-0.793435	-6.9077553	15	83	3.12927222	293	20	0	0	1	0	18.9135952	0	-0.0428739
2.2896	76352.2692	0.2229274	10.5996989	0.38512707	0	0.82837713	4.33537085	-1.4964334	2.36091994	-0.9515888	-6.9077553	16	87	3.57631111	293	20	0	0	1	0	18.9135952	0	-0.0428739
2.3238	77755.9043	0.20563783	9.11031462	0.3659151	0	0.84320378	4.35358735	-1.5767876	2.20951701	-1.0026248	-6.9077553	17	91	3.57631111	292.444444	19.4444444	0	0	1	0	18.9135952	0	-0.0428739
2.3508	78984.0904	0.20179553	8.33013255	0.30921011	0	0.8547557	4.36925911	-1.595557	2.11999941	-1.1705054	-6.9077553	18	94	3.12927222	291.888889	18.8888888	0	0	1	0	18.9135952	0	-0.0428739
2.3841	80431.5959	0.19248854	7.52532299	0.30811769	0	0.86882169	4.38741952	-1.642537	2.01840661	-1.1740332	-6.9077553	19	93	4.02335	291.888889	18.8888888	0	0	1	0	18.9135952	0	-0.0428739
2.4066	81484.3276	0.17176411	6.09691589	0.2644029	0	0.87821496	4.40042297	-1.7558282	1.80794706	-1.3265062	-6.9077553	20	90	4.91742778	291.888889	18.8888888	0	0	1	0	18.9135952	0	-0.0428739
2.439	82887.9678	0.15045947	4.74156359	0.2695806	0	0.89158812	4.41750197	-1.8874372	1.55657783	-1.3071852	-6.9077553	21	90	3.57631111	291.888889	18.8888888	0	0	1	0	18.9135952	0	-0.0428739
2.4552	83633.6548	0.16654139	5.49315304	0.24074697	0	0.89820822	4.42645796	-1.7865248	1.70368444	-1.4198637	-6.9077553	22	89	4.47038889	291.888889	18.8888888	0	0	1	0	18.9135952	0	-0.0428739
2.4777	84554.7939	0.15787263	5.08249634	0.22092155	0	0.90733071	4.4374116	-1.8396525	1.62599928	-1.5054313	-6.9077553	23	90	4.47038889	291.888889	18.8888888	0	0	1	0	18.9135952	0	-0.0428739

0.0162	503.018212	0.83153164	189.753547	1.71323754	0	-4.122744	-0.6851429	-0.1832841	5.24573138	0.5389684	-6.9077553	0	36	3.12927222	290.222222	17.2222222	0	0	0	0	30.519848	0	0.21141735
0.072	1853.07823	0.75397135	175.044087	1.70155507	0	-2.6310892	0.61738766	-0.2810755	5.16504358	0.5321301	-6.9077553	1	31	3.57631111	293.555556	20.5555555	0	0	0	0	30.519848	0	0.21141735
0.2187	2908.58173	0.6425529	122.186182	3.15261672	0	-1.5200544	1.06800934	-0.4407511	4.80555415	1.14854996	-6.9077553	2	22	5.36446667	296.333333	23.3333333	0	0	0	0	30.519848	0	0.21141735
0.3852	3983.97711	0.78055123	179.9697	3.43354294	0	-0.9539926	1.38253157	-0.2464746	5.19279406	1.23388386	-6.9077553	3	20	5.81150556	297.444444	24.4444444	0	0	0	0	30.519848	0	0.21141735
0.6534	6182.41441	0.73723372	154.850737	2.62938245	0	-0.4255658	1.82187061	-0.3034948	5.04246812	0.96712925	-6.9077553	4	18	4.91742778	298.555556	25.5555555	0	0	0	1	30.519848	0	0.21141735
0.9999	17563.1752	0.87725307	236.713677	2.80607688	0	-0.0001	2.86586133	-0.1298205	5.46685552	1.03214369	-6.9077553	5	16	5.36446667	299.111111	26.1111111	0	0	0	1	30.519848	0	0.21141735
1.3599	30262.0108	0.77675941	164.477793	2.2095726	0	0.30741117	3.4099262	-0.251338	5.10278164	0.79325158	-6.9077553	6	18	5.36446667	298.555556	25.5555555	0	0	0	1	30.519848	0	0.21141735
1.6803	44002.6991	0.72767991	150.441011	1.41724044	0	0.51897235	3.7842737	-0.3165207	5.0135777	0.34941698	-6.9077553	7	25	2.68223333	295.222222	22.2222222	0	0	0	1	30.519848	0	0.21141735
1.9782	61054.4009	0.7757088	174.988682	1.50343235	0	0.68218734	4.11178166	-0.2526898	5.16472702	0.40841565	-6.9077553	8	29	1.34111667	293	20	0	0	0	1	30.519848	0	0.21141735
2.2626	76975.5938	0.74677016	156.410033	1.34839746	0	0.81651459	4.3435014	-0.2906596	5.05248737	0.29965817	-6.9077553	9	31	1.34111667	290.777778	17.7777778	0	0	0	1	30.519848	0	0.21141735
2.5263	94566.7432	0.74786028	166.934727	1.44914105	0	0.92675578	4.54931644	-0.2892029	5.11760887	0.37166083	-6.9077553	10	33	2.68223333	290.222222	17.2222222	0	0	0	1	30.519848	0	0.21141735
2.7585	113175.868	0.69006868	132.889838	1.13305593	0	1.01468705	4.7289518	-0.3695161	4.88952802	0.12580053	-6.9077553	11	35	3.12927222	290.222222	17.2222222	0	0	0	1	30.519848	0	0.21141735
2.9934	135378.932	0.72022279	139.036069	1.2430607	0	1.09640987	4.90808514	-0.3268072	4.93474058	0.21838079	-6.9077553	12	36	3.57631111	290.222222	17.2222222	0	0	0	1	30.519848	0	0.21141735
3.2238	157217.115	0.71448994	139.61525	1.42789543	0	1.17056079	5.05763411	-0.3347877	4.93889758	0.35690172	-6.9077553	13	40	3.57631111	289.111111	16.1111111	0	0	0	1	30.519848	0	0.21141735
3.4074	185341.926	0.67717384	125.812376	1.124353	0	1.22594954	5.22220776	-0.3883516	4.83479966	0.11809676	-6.9077553	14	44	3.57631111	288	15	0	0	0	1	30.519848	0	0.21141735
3.6045	213852.732	0.66921649	121.222763	1.51952052	0	1.28218306	5.36529229	-0.4001545	4.79763812	0.41905272	-6.9077553	15	44	4.91742778	286.888889	13.8888888	0	0	0	1	30.519848	0	0.21141735
3.7845	243835.599	0.62994591	105.313779	0.93380928	0	1.33091378	5.49649832	-0.4605351	4.65695376	-0.0674127	-6.9077553	16	51	4.02335	285.777778	12.7777777	0	0	0	1	30.519848	0	0.21141735
3.9258	272171.735	0.62979748	102.743058	0.54304913	0	1.36757015	5.60643692	-0.4607704	4.63224103	-0.6087157	-6.9077553	17	63	2.23519444	284.666667	11.6666666	0	0	0	1	30.519848	0	0.21141735
4.0464	298418.772	0.6140295	93.5103049	0.44675147	0	1.3978276	5.69850113	-0.486085	4.53808234	-0.803517	-6.9077553	18	71	2.23519444	284.666667	11.6666666	0	0	0	1	30.519848	0	0.21141735
4.14	320558.423	0.5788769	81.9554725	0.43191484	0	1.42069579	5.77006767	-0.5449394	4.40618828	-0.8372142	-6.9077553	19	73	2.68223333	284.666667	11.6666666	0	0	0	1	30.519848	0	0.21141735
4.23	342334.946	0.54150749	72.5856543	0.41560509	0	1.44220199	5.83579255	-0.6115534	4.28478108	-0.8756165	-6.9077553	20	79	3.57631111	284.666667	11.6666666	0	0	0	1	30.519848	0	0.21141735
4.2822	357819.022	0.43657277	45.1153901	0.32702032	0	1.4544669	5.88003013	-0.8265123	3.8092456	-1.1146797	-6.9077553	21	83	4.47038889	286.333333	13.3333333	0	0	0	1	30.519848	0	0.21141735
4.2822	357819.022	0	0	0	0	1.4544669	5.88003013	-6.9077553	-6.9077553	-6.9077553	-6.9077553	22	84	3.12927222	287.444444	14.4444444	0	0	0	1	30.519848	0	0.21141735
4.3119	367498.735	0.3159847	25.9222005	0.47013973	0	1.46137864	5.9067226	-1.1489018	3.25513834	-0.7526006	-6.9077553	23	83	4.91742778	289.111111	16.1111111	0	0	0	1	30.519848	0	0.21141735
0.0441	1792.67413	1.1197931	362.977946	3.20243327	0	-3.1212955	0.5842661	0.11403656	5.89434483	1.16422313	-6.9077553	0	36	3.12927222	290.222222	17.2222222	0	0	0	0	34.0724638	0	0.49277013
0.1278	5879.38044	0.95160921	244.384514	1.50931623	0	-2.0572887	1.77162146	-0.0485505	5.49874695	0.41231905	-6.9077553	1	31	3.57631111	293.555556	20.5555555	0	0	0	0	34.0724638	0	0.49277013
0.2565	11053.3357	1.01863226	277.949628	2.54775531	0	-1.3606266	2.40282272	0.01944203	5.6274435	0.93560513	-6.9077553	2	22	5.36446667	296.333333	23.3333333	0	0	0	1	34.0724638	0	0.49277013
0.4815	24818.3851	1.38765015	569.334	4.57157861	0	-0.730849	3.21162501	0.32833216	6.34446901	1.52007729	-6.9077553	3	20	5.81150556	297.444444	24.4444444	0	0	0	1	34.0724638	0	0.49277013
0.8622	49821.1211	1.43342219	597.461584	4.38160623	2	-0.148268	3.90845908	0.36076211	6.39269166	1.47764358	0.69364706	4	18	4.91742778	298.555556	25.5555555	0	0	0	1	34.0724638	0	0.49277013
1.3806	67328.9774	1.36968157	562.774631	4.37847307	5	0.32251819	4.20960557	0.31530811	6.33288102	1.47692841	1.60963789	5	16	5.36446667	299.111111	26.1111111	0	0	0	1	34.0724638	0	0.49277013
2.133	93067.1467	1.26983146	477.404381	3.8090207	5	0.75752944	4.53333199	0.23967138	6.16836599	1.33763462	1.60963789	6	18	5.36446667	298.555556	25.5555555	0	0	0	1	34.0724638	0	0.49277013
2.6829	113315.803	0.99856602	285.366893	2.17072982	5	0.9868983	4.73018746	-0.0004341	5.6537792	0.775524	1.60963789	7	25	2.68223333	295.222222	22.2222222	0	0	0	1	34.0724638	0	0.49277013
3.249	133812.268	0.97532482	268.409206	2.00199582	5	1.17834726	4.89644531	-0.0239599	5.59251643	0.69464397	1.60963789	8	29	1.34111667	293	20	0	0	0	1	34.0724638	0	0.49277013
3.7188	147882.983	0.89636248	226.576164	1.71568094	5	1.31340104	4.99642806	-0.1082954	5.42308557	0.54039274	1.60963789	9	31	1.34111667	290.777778	17.7777778	0	0	0	1	34.0724638	0	0.49277013
4.1805	160368.363	0.85619629	203.794895	1.74444759	5	1.43043086	5.07747967	-0.1540883	5.31711898	0.55701102	1.60963789	10	33	2.68223333	290.222222	17.2222222	0	0	0	1	34.0724638	0	0.49277013
4.6989	172530.274	0.87704817	223.322822	2.14247364	5	1.54732844	5.15057852	-0.1300538	5.40862283	0.76242771	1.60963789	11	35	3.12927222	290.222222	17.2222222	0	0	0	1	34.0724638	0	0.49277013
5.1813	184977.518	0.78042978	169.314468	1.5913382	5	1.64505599	5.2202397	-0.24663	5.13176365	0.4652035	1.60963789	12	36	3.57631111	290.222222	17.2222222	0	0	0	1	34.0724638	0	0.49277013
5.6763	199962.044	0.80259327	178.919375	1.58100309	5	1.73629961	5.29813257	-0.218662	5.18694087	0.45869182	1.60963789	13	40	3.57631111	289.111111	16.1111111	0	0	0	1	34.0724638	0	0.49277013
6.1425	215333.21	0.78578799	167.412464	1.54094292	5	1.81523183	5.37219129	-0.2397965	5.12046658	0.43304326	1.60963789	14	44	3.57631111	288	15	0	0	0	1	34.0724638	0	0.49277013
6.6861	230631.522	0.78153547	170.524291	1.88513911	5	1.90003074	5.44082563	-0.245216	5.13888362	0.63453194	1.60963789	15	44	4.91742778	286.888889	13.8888888	0	0	0	1	34.0724638	0	0.49277013
7.2216	248236.674	0.81152944	196.092686	1.74356946	5	1.97707654	5.51438665	-0.2076031	5.27859254	0.5565078	1.60963789	16	51	4.02335	285.777778	12.7777777	0	0	0	1	34.0724638	0	0.49277013
7.5735	263116.345	0.8032602	181.036925	1.37147339	5	2.02465531	5.57260011	-0.2178324	5.19870654	0.3166145	1.60963789	17	63	2.23519444	284.666667	11.6666666	0	0	0	1	34.0724638	0	0.49277013
7.8633	276466.799	0.87769635	207.754125	1.55364289	5	2.06220637	5.62209435	-0.1293159	5.3363601	0.44124587	1.60963789	18	71	2.23519444	284.666667	11.6666666	0	0	0	1	34.0724638	0	0.49277013
8.1153	286780.307	0.80047575	171.710756	1.40523998	5	2.09375117	5.65871993	-0.2213006	5.14581723	0.34091946	1.60963789	19	73	2.68223333	284.666667	11.6666666	0	0	0	1	34.0724638	0	0.49277013
8.2719	293650.529	0.73675344	140.921258	1.17668424	5	2.11286423	5.68239379	-0.3041456	4.94820837	0.16355	1.60963789	20	79	3.57631111	284.666667	11.6666666	0	0	0	1	34.0724638	0	0.49277013
8.3628	297422.809	0.60192225	91.7266882	0.91492001	5	2.1237933	5.69515809	-0.505967	4.51882428	-0.0878262	1.60963789	21	83	4.47038889	286.333333	13.3333333	0	0	0	1	34.0724638	0	0.49277013
8.3988	299089.639	0.19804523	8.02194519	0.28614435	5	2.12808884	5.70074667	-1.6142232	2.08230559	-1.2477702	1.60963789	22	84	3.12927222	287.444444	14.4444444	0	0	0	1	34.0724638	0	0.49277013
8.4798	302598.735	0.47803786	63.5248372	0.68747491	5	2.13768686	5.71241092	-0.7359757	4.15144671	-0.3732764	1.60963789	23	83	4.91742778	289.111111	16.1111111	0	0	0	1	34.0724638	0	0.49277013
0.0558		1.34411071			0	-2.8859814		0.29647632			-6.9077553	0	36	3.12927222	290.222222	17.2222222	0	0	0	0	42.288408	0	-0.4111751
0.2619		1.19288389			0	-1.3397925		0.17721176			-6.9077553	1	31	3.57631111	293.555556	20.5555555	0	0	0	0	42.288408	0	-0.4111751
0.5535		1.11452673			0	-0.5914935		0.10932669			-6.9077553	2	22	5.36446667	296.333333	23.3333333	0	0	0	0	42.288408	0	-0.4111751
0.9495		1.06925021			0	-0.0518197		0.06789246			-6.9077553	3	20	5.81150556	297.444444	24.4444444	0	0	0	0	42.288408	0	-0.4111751
1.4166		1.04355088			0	0.34825963		0.04358701			-6.9077553	4	18	4.91742778	298.555556	25.5555555	0	0	1	0	42.288408	0	-0.4111751
2.2365		1.18077421			1	0.80491214		0.16701687			0.0009995	5	16	5.36446667	299.111111	26.1111111	0	0	1	0	42.288408	0	-0.4111751
2.9907		1.13132289			1	1.09550747		0.12427118			0.0009995	6	18	5.36446667	298.555556	25.5555555	0	0	1	0	42.288408	0	-0.4111751
3.6486		1.00632161			1	1.29434353		0.00729493			0.0009995	7	25	2.68223333	295.222222	22.2222222	0	0	1	0	42.288408	0	-0.4111751
4.1661		0.91354609			1	1.42698035		-0.0893274			0.0009995	8	29	1.34111667	293	20	0	0	1	0	42.288408	0	-0.4111751
4.6755		0.92578211			1	1.54233611		-0.0760368			0.0009995	9	31	1.34111667	290.777778	17.7777778	0	0	1	0	42.288408	0	-0.4111751
5.202		0.86472304			1	1.64904317		-0.1441902			0.0009995	10	33	2.68223333	290.222222	17.2222222	0	0	1	0	42.288408	0	-0.4111751
5.6979		0.82263859			1	1.74009769		-0.1940234			0.0009995	11	35	3.12927222	290.222222	17.2222222	0	0	1	0	42.288408	0	-0.4111751
6.1524		0.6933804			1	1.81684225		-0.3647353			0.0009995	12	36	3.57631111	290.222222	17.2222222	0	0	1	0	42.288408	0	-0.4111751
6.5601		0.67159866			1	1.88100585		-0.3966065			0.0009995	13	40	3.57631111	289.111111	16.1111111	0	0	1	0	42.288408	0	-0.4111751

6.9336		0.64026511			1	1.93637916		-0.4443123			0.0009995	14	44	3.57631111	288	15	0	0	1	0	42.288408	0	-0.4111751
7.3161		0.65397059			1	1.9900774		-0.4231649			0.0009995	15	44	4.91742778	286.888889	13.88888888	0	0	1	0	42.288408	0	-0.4111751
7.6437		0.7123901			1	2.03388178		-0.3377269			0.0009995	16	51	4.02335	285.777778	12.77777777	0	0	1	0	42.288408	0	-0.4111751
7.821		0.67566878			1	2.05681242		-0.3905734			0.0009995	17	63	2.23519444	284.666667	11.66666666	0	0	1	0	42.288408	0	-0.4111751
7.9272		0.83464412			1	2.07029988		-0.1795524			0.0009995	18	71	2.23519444	284.666667	11.66666666	0	0	1	0	42.288408	0	-0.4111751
7.992		0.76163022			1	2.07844104		-0.270982			0.0009995	19	73	2.68223333	284.666667	11.66666666	0	0	1	0	42.288408	0	-0.4111751
8.0658		0.74919073			1	2.0876329		-0.2874278			0.0009995	20	79	3.57631111	284.666667	11.66666666	0	0	1	0	42.288408	0	-0.4111751
8.1063		0.57892439			1	2.09264154		-0.5448575			0.0009995	21	83	4.47038889	286.333333	13.33333333	0	0	1	0	42.288408	0	-0.4111751
8.118		0.27261818			1	2.09408382		-1.2960216			0.0009995	22	84	3.12927222	287.444444	14.44444444	0	0	1	0	42.288408	0	-0.4111751
8.2026		0.64646299			1	2.10445118		-0.4346936			0.0009995	23	83	4.91742778	289.111111	16.11111111	0	0	1	0	42.288408	0	-0.4111751
0.0117	0	0.51139458	72.3967692	2.07208411	0	-4.4481664	-6.9077553	-0.6686603	4.28217549	0.72903741	-6.9077553	0	36	3.12927222	290.222222	17.2222222	0	0	0	0	18.9135952	0	-0.0428739
0.1116	0	0.6100811	99.4621692	2.91157765	0	-2.1928342	-6.9077553	-0.4925256	4.59978742	1.06903848	-6.9077553	1	31	3.57631111	293.555556	20.55555555	0	0	0	0	18.9135952	0	-0.0428739
0.351	3334.92124	0.78901902	214.285494	3.49614721	0	-1.0469691	1.20474887	-0.2356983	5.36731388	1.25194755	-6.9077553	2	22	5.36446667	296.333333	23.33333333	0	0	1	0	18.9135952	0	-0.0428739
0.585	13080.0139	1.02808321	380.461418	2.84256169	0	-0.5361434	2.57116186	0.02866832	5.9413874	1.04505738	-6.9077553	3	20	5.81150556	297.444444	24.44444444	0	0	1	0	18.9135952	0	-0.0428739
0.7821	17877.9219	0.81343963	209.22271	1.85136476	0	-0.2457727	2.88362247	-0.205255	5.34340406	0.61646307	-6.9077553	4	18	4.91742778	298.555556	25.55555555	0	0	1	0	18.9135952	0	-0.0428739
1.0566	26568.536	0.91228167	259.714395	2.28660789	0	0.05505621	3.2797653	-0.0907109	5.5595864	0.82750668	-6.9077553	5	16	5.36446667	299.111111	26.11111111	0	0	1	0	18.9135952	0	-0.0428739
1.4175	41429.1836	1.01495909	320.317	2.58843127	0	0.34889476	3.72400969	0.01583309	5.76931425	0.95143826	-6.9077553	6	18	5.36446667	298.555556	25.55555555	0	0	1	0	18.9135952	0	-0.0428739
1.7487	52807.411	0.87889215	231.218219	1.87589911	0	0.55887265	3.96667048	-0.1279559	5.44336626	0.629621	-6.9077553	7	25	2.68223333	295.222222	22.2222222	0	0	1	0	18.9135952	0	-0.0428739
2.0583	64509.8112	0.82141353	205.774164	1.60685765	0	0.7218804	4.16683283	-0.1955119	5.32678414	0.47490264	-6.9077553	8	29	1.34111667	293	20	0	0	1	0	18.9135952	0	-0.0428739
2.4426	78015.543	0.83985747	215.892107	1.8029738	0	0.89306305	4.35692089	-0.1733331	5.37478341	0.5899919	-6.9077553	9	31	1.34111667	290.777778	17.7777778	0	0	1	0	18.9135952	0	-0.0428739
2.8764	95174.2761	0.85957811	225.017862	1.89779245	0	1.05653951	4.5557202	-0.1501509	5.41618423	0.64121813	-6.9077553	10	33	2.68223333	290.222222	17.2222222	0	0	1	0	18.9135952	0	-0.0428739
3.3183	109697.387	0.81995362	199.452138	1.99373613	0	1.1994526	4.69773467	-0.1972887	5.29557931	0.69051177	-6.9077553	11	35	3.12927222	290.222222	17.2222222	0	0	1	0	18.9135952	0	-0.0428739
3.771	125240.978	0.8014024	190.381943	1.74558872	0	1.32734022	4.83024769	-0.2201451	5.24903753	0.55766458	-6.9077553	12	36	3.57631111	290.222222	17.2222222	0	0	1	0	18.9135952	0	-0.0428739
4.1832	139770.776	0.79319923	181.883955	1.53753476	0	1.4310765	4.94001092	-0.2304209	5.20337437	0.43083051	-6.9077553	13	40	3.57631111	289.111111	16.11111111	0	0	1	0	18.9135952	0	-0.0428739
4.6881	159492.841	0.83666252	210.185773	1.78462489	0	1.54502738	5.07200531	-0.17714	5.34799653	0.57976843	-6.9077553	14	44	3.57631111	288	15	0	0	1	0	18.9135952	0	-0.0428739
5.1525	176137.652	0.79710258	192.329639	1.8222836	0	1.63948203	5.17127148	-0.2255181	5.25921597	0.60063905	-6.9077553	15	44	4.91742778	286.888889	13.88888888	0	0	1	0	18.9135952	0	-0.0428739
5.5386	188470.58	0.71618396	144.436316	1.37081063	0	1.71174176	5.23894722	-0.3324229	4.97284562	0.31613149	-6.9077553	16	51	4.02335	285.777778	12.77777777	0	0	1	0	18.9135952	0	-0.0428739
5.8365	201498.905	0.8266998	183.519673	1.28449888	0	1.7641313	5.30578891	-0.1891047	5.21232732	0.25114687	-6.9077553	17	63	2.23519444	284.666667	11.66666666	0	0	1	0	18.9135952	0	-0.0428739
6.1596	216701.333	0.89646482	215.646675	1.57818042	0	1.81801184	5.37852468	-0.1081814	5.37364594	0.45690599	-6.9077553	18	71	2.23519444	284.666667	11.66666666	0	0	1	0	18.9135952	0	-0.0428739
6.4854	232688.941	0.89377843	215.369346	1.62394706	0	1.8695535	5.44970684	-0.1111792	5.37235908	0.48547524	-6.9077553	19	73	2.68223333	284.666667	11.66666666	0	0	1	0	18.9135952	0	-0.0428739
6.7986	248026.708	0.82033206	182.701172	1.50561997	0	1.91671671	5.51354047	-0.1968278	5.20785735	0.40986871	-6.9077553	20	79	3.57631111	284.666667	11.66666666	0	0	1	0	18.9135952	0	-0.0428739
7.0263	258587.016	0.66207837	116.371619	1.15615688	0	1.94966025	5.55523612	-0.4108621	4.75679727	0.14596603	-6.9077553	21	83	4.47038889	286.333333	13.33333333	0	0	1	0	18.9135952	0	-0.0428739
7.1262	263061.096	0.27699964	21.9332396	0.47365381	0	1.96377813	5.57239011	-1.2801355	3.08804887	-0.7451696	-6.9077553	22	84	3.12927222	287.444444	14.44444444	0	0	1	0	18.9135952	0	-0.0428739
7.3809	274046.847	0.65070464	128.385932	1.21863009	0	1.99889558	5.61330271	-0.4281638	4.85504861	0.1985476	-6.9077553	23	83	4.91742778	289.111111	16.11111111	0	0	1	0	18.9135952	0	-0.0428739
0.0063	223.152687	0.91843629	227.561198	1.50977008	0	-5.0672056	-1.4954278	-0.0839945	5.4274236	0.4126195	-6.9077553	0	92	1.34111667	298	25	0	0	0	0	30.519848	0	0.21141735
0.0135	530.199164	0.46302772	57.2659149	0.83521116	0	-4.3050656	-0.6326183	-0.767811	4.04772306	-0.1788741	-6.9077553	1	95	1.34111667	297.444444	24.44444444	0	0	0	0	30.519848	0	0.21141735
0.0288	1056.56467	0.62535252	94.6400218	1.04718309	0	-3.5473799	0.05596879	-0.4678419	4.55009102	0.04705828	-6.9077553	2	96	2.23519444	297.444444	24.44444444	0	0	0	0	30.519848	0	0.21141735
0.0387	1275.8835	0.5558932	80.5139549	1.09108569	0	-3.2519157	0.24442234	-0.5853818	4.38844294	0.08808935	-6.9077553	3	98	1.78815556	297.444444	24.44444444	0	0	0	0	30.519848	0	0.21141735
0.0486	1582.92986	0.34625877	33.7020125	0.99206266	0	-3.0241317	0.45990901	-1.057685	3.51758722	-0.0069615	-6.9077553	4	93	3.12927222	298.555556	25.55555555	0	0	0	0	30.519848	0	0.21141735
0.0972	1742.08326	0.34201679	33.227374	1.35754522	0	-2.3309846	0.55565553	-1.0699759	3.50340415	0.30641443	-6.9077553	5	80	4.02335	300.777778	27.7777778	0	0	0	0	30.519848	0	0.21141735
0.2412	2347.32848	0.54267216	85.6148499	2.35109406	0	-1.4221288	0.85370379	-0.6094089	4.44987043	0.85530602	-6.9077553	6	72	4.47038889	302.444444	29.44444444	0	0	0	0	30.519848	0	0.21141735
0.3267	5397.29426	0.60968395	103.306229	1.5496166	0	-1.118713	1.68608303	-0.4931757	4.63770736	0.43865266	-6.9077553	7	62	3.57631111	304.111111	31.11111111	0	0	0	0	30.519848	0	0.21141735
0.4617	10597.8963	0.60566111	97.5447135	1.50329519	0	-0.7728399	2.36074987	-0.4997849	4.58032112	0.40832447	-6.9077553	8	55	3.12927222	305.777778	32.7777778	0	0	0	1	30.519848	0	0.21141735
0.6345	16496.2769	0.62589395	107.75415	1.6341265	0	-0.454918	2.80319533	-0.4669779	4.67986153	0.49172017	-6.9077553	9	50	3.57631111	306.888889	33.88888888	0	0	0	1	30.519848	0	0.21141735
0.864	23962.0159	0.66249382	118.474632	1.70272253	0	-0.1461825	3.17651164	-0.410229	4.77470731	0.53281558	-6.9077553	10	46	2.68223333	307.444444	34.4444444	0	0	0	1	30.519848	0	0.21141735
1.1232	33652.2949	0.78406355	174.073622	1.8434817	0	0.11618175	3.51611097	-0.2419906	5.15948407	0.61219832	-6.9077553	11	41	2.68223333	309.111111	36.11111111	0	0	0	1	30.519848	0	0.21141735
1.3995	43287.3133	0.79269414	177.639353	1.89779057	0	0.33611503	3.7678827	-0.2310571	5.17976102	0.64121714	-6.9077553	12	38	3.12927222	309.666667	36.66666666	0	0	0	1	30.519848	0	0.21141735
1.7271	55763.6145	0.74287029	151.778031	1.69577043	0	0.5464437	4.02113952	-0.2958886	5.02242572	0.5287267	-6.9077553	13	36	3.12927222	309.111111	36.11111111	0	0	0	1	30.519848	0	0.21141735
2.0115	68359.857	0.81395219	187.086976	1.55598054	0	0.69888071	4.22480039	-0.2046258	5.23157897	0.44274839	-6.9077553	14	38	3.12927222	308.555556	35.5555556	0	0	0	1	30.519848	0	0.21141735
2.268	80662.5742	0.78452491	174.230921	1.42243063	0	0.81889839	4.3902871	-0.2414031	5.16038729	0.3530699	-6.9077553	15	42	1.34111667	308	35	0	0	0	1	30.519848	0	0.21141735
2.4741	93066.7818	0.72868374	152.911947	1.25755583	0	0.90587669	4.53332806	-0.3151441	5.02986879	0.2299649	-6.9077553	16	54	1.34111667	304.666667	31.66666666	0	0	0	1	30.519848	0	0.21141735
2.6001	103297.409	0.72413341	153.752651	1.0756255	0	0.95554991	4.63762197	-0.3213996	5.03535165	0.07383161	-6.9077553	17	61	0.89407778	303	30	0	0	0	1	30.519848	0	0.21141735
2.6793	114019.759	0.77669057	162.731268	1.06841634	0	0.98555557	4.73638053	-0.2514266	5.09210632	0.06711302	-6.9077553	18	66	1.34111667	301.888889	28.88888888	0	0	0	1	30.519848	0	0.21141735
2.7162	121400.31	0.63144223	102.404904	0.64187138	0	0.99923384	4.79910167	-0.4581664	4.62894437	-0.4418106	-6.9077553	19	66	1.34111667	301.333333	28.33333333	0	0	0	1	30.519848	0	0.21141735
2.7522	129499.505	0.62553487	101.515247	0.73022175	0	1.01240059	4.86368478	-0.4675509	4.62021885	-0.3130385	-6.9077553	20	69	1.34111667	300.777778	27.7777778	0	0	0	1	30.519848	0	0.21141735
2.772	133614.715	0.54010569	77.3858254	0.60060328	0	1.01956908	4.89496788	-0.6141407	4.34881655	-0.5081571	-6.9077553	21	72	1.78815556	299.666667	26.66666666	0	0	0	1	30.519848	0	0.21141735
2.7954	139378.74	0.40383082	37.4923025	0.27115992	0	1.02797521	4.93720215	-0.904286	3.62416232	-1.3013654	-6.9077553	22	78	2.23519444	299.666667	26.66666666	0	0	0	1	30.519848	0	0.21141735
2.7954	139378.74	0	0	0	0	1.02797521	4.93720215	-6.9077553	-6.9077553	-6.9077553	-6.9077553	23	83	2.23519444	299.111111	26.11111111	0	0	0	1	30.519848	0	0.21141735
0.0135	613.354524	0.98885209	269.55528	1.78391457	0	-4.3050656	-0.4871831	-0.0101997	5.5967772	0.57937055	-6.9077553	0	92	1.34111667	298	25	0	0	0	0	34.0724638	0	0.49277013
0.0252	964.264794	0.53721479	67.7680335	0.74615743	0	-3.6809113	-0.0353528	-0.6194976	4.21610536	-0.2914794	-6.9077553	1	95	1.34111667	297.444444	24.44444444	0	0	0	0	34.0724638	0	0.49277013
0.0378	1490.63018	0.52216561	68.2047294	0.83112117	0	-3.2754462	0.3998696	-0.6478572	4.22252857	-0.1837772	-6.9077553	2	96	2.23519444	297.444444	24.44444444	0	0	0	0	34.0724638	0	0.49277013
0.0495	1973.13165	0.51269744	65.5048781	0.7498268	0	-3.0057826	0.68012863	-0.6661208	4.18213988	-0.2865803	-6.9077553	3	98	1.78815556	297.444444	24.44444444	0	0	0	0	34.0724638	0	0.49277013

0.0783	3157.45403	0.61117291	94.4201279	1.0749681	0	-2.5472077	1.15008268	-0.4907405	4.54776486	0.07322081	-6.9077553	4	93	3.12927222	298.555556	25.55555555	0	0	0	0	34.0724638	0	0.49277013
0.1413	6029.86553	0.78150564	168.838909	1.43867667	0	-1.95687	1.79689054	-0.2452541	5.12895098	0.36441855	-6.9077553	5	80	4.02335	300.777778	27.7777778	0	0	0	0	34.0724638	0	0.49277013
0.2376	10166.693	0.88753046	214.97304	1.80318658	0	-1.4371667	2.31921534	-0.1181863	5.37051727	0.59010984	-6.9077553	6	72	4.47038889	302.444444	29.44444444	0	0	0	0	34.0724638	0	0.49277013
0.4536	19345.7604	1.06005319	315.496517	2.63896942	0	-0.7905395	2.96252498	0.05926199	5.75415081	0.97076733	-6.9077553	7	62	3.57631111	304.111111	31.11111111	0	0	0	1	34.0724638	0	0.49277013
0.7569	32577.9063	1.13585393	374.140579	2.87884467	0	-0.2785241	3.48366503	0.12826474	5.92463428	1.05773636	-6.9077553	8	55	3.12927222	305.777778	32.7777778	0	0	0	1	34.0724638	0	0.49277013
1.2033	49548.2942	1.15827647	384.246724	3.03726344	0	0.18506778	3.90296802	0.14779607	5.95128746	1.11128612	-6.9077553	9	50	3.57631111	306.888889	33.88888888	0	0	0	1	34.0724638	0	0.49277013
1.593	58782.913	0.99039098	275.897283	2.12781579	0	0.46561903	4.07386823	-0.0086463	5.62003226	0.75556586	-6.9077553	10	46	2.68223333	307.444444	34.4444444	0	0	0	1	34.0724638	0	0.49277013
2.052	69041.3555	1.01196971	286.156189	2.11038651	0	0.71881493	4.23472016	0.01288633	5.65654127	0.74734484	-6.9077553	11	41	2.68223333	309.111111	36.11111111	0	0	0	1	34.0724638	0	0.49277013
2.6811	87080.0018	1.06835396	327.648359	2.50721804	0	0.98622716	4.46683874	0.06705469	5.79194401	0.91957256	-6.9077553	12	38	3.12927222	309.666667	36.66666666	0	0	0	1	34.0724638	0	0.49277013
3.4641	114513.194	1.10439088	346.065661	2.75650368	0	1.24245286	4.74069878	0.10019901	5.84663142	1.01432581	-6.9077553	13	36	3.12927222	309.111111	36.11111111	0	0	0	1	34.0724638	0	0.49277013
4.0896	134908.36	0.93421596	247.535567	1.94432979	0	1.40844717	4.90460315	-0.0669778	5.51155831	0.66543152	-6.9077553	14	38	3.12927222	308.555556	35.5555556	0	0	0	1	34.0724638	0	0.49277013
4.5981	154074.928	0.8971152	221.757107	1.6422063	0	1.52564317	5.03744552	-0.1074569	5.40158718	0.49664939	-6.9077553	15	42	1.34111667	308	35	0	0	0	1	34.0724638	0	0.49277013
5.076	169632.039	0.81885639	187.80223	1.78411911	0	1.62452355	5.13363751	-0.1986261	5.23539477	0.57948514	-6.9077553	16	54	1.34111667	304.666667	31.66666666	0	0	0	1	34.0724638	0	0.49277013
5.3568	180700.2	0.74628351	156.744341	1.22847366	0	1.67836678	5.19684484	-0.2913106	5.05462246	0.20658616	-6.9077553	17	61	0.89407778	303	30	0	0	0	1	34.0724638	0	0.49277013
5.5872	190635.242	0.87552351	206.341064	1.52961135	0	1.72047827	5.25036712	-0.1317918	5.3295353	0.42566723	-6.9077553	18	66	1.34111667	301.888889	28.88888888	0	0	0	1	34.0724638	0	0.49277013
5.7852	198922.115	0.77042944	153.898047	1.21373083	0	1.75530293	5.29291839	-0.2595101	5.03629685	0.19452251	-6.9077553	19	66	1.34111667	301.333333	28.33333333	0	0	0	1	34.0724638	0	0.49277013
5.9742	207165.876	0.77967006	160.049219	1.27006022	0	1.7874502	5.33352463	-0.2476027	5.07548763	0.23985137	-6.9077553	20	69	1.34111667	300.777778	27.7777778	0	0	0	1	34.0724638	0	0.49277013
6.1659	215596.07	0.76620584	154.621479	1.26686089	0	1.81903411	5.37341125	-0.2650001	5.04098653	0.23733114	-6.9077553	21	72	1.78815556	299.666667	26.66666666	0	0	0	1	34.0724638	0	0.49277013
6.3189	221874.222	0.66146689	114.067394	1.0854617	0	1.84354514	5.40211516	-0.4117847	4.73679822	0.08292626	-6.9077553	22	78	2.23519444	299.666667	26.66666666	0	0	0	1	34.0724638	0	0.49277013
6.3675	223891.965	0.27574534	20.7275571	0.54409818	0	1.85120693	5.4111681	-1.2846575	3.03151232	-0.6067893	-6.9077553	23	83	2.23519444	299.111111	26.11111111	0	0	0	1	34.0724638	0	0.49277013
0.0261	1455.7842	1.02318096	283.515102	1.93222264	0	-3.64582	0.3762314	0.02389323	5.64726892	0.65918837	-6.9077553	0	92	1.34111667	298	25	0	0	0	0	42.288408	0	-0.4111751
0.0693	3565.62184	0.70820292	128.5837	1.27600352	0	-2.6693104	1.27161888	-0.3436136	4.85658783	0.24451633	-6.9077553	1	95	1.34111667	297.444444	24.44444444	0	0	0	0	42.288408	0	-0.4111751
0.1206	5934.26678	0.59030105	89.4124178	1.07662	0	-2.115276	1.78091198	-0.52543	4.49327076	0.07475491	-6.9077553	2	96	2.23519444	297.444444	24.44444444	0	0	0	0	42.288408	0	-0.4111751
0.1809	8829.276	0.64831675	105.780703	1.15266384	0	-1.7098109	2.17818627	-0.4318339	4.66137757	0.14294283	-6.9077553	3	98	1.78815556	297.444444	24.44444444	0	0	0	0	42.288408	0	-0.4111751
0.2601	12618.759	0.75485628	147.95166	1.37956533	0	-1.3466891	2.53526376	-0.279904	4.99689236	0.32249307	-6.9077553	4	93	3.12927222	298.555556	25.55555555	0	0	0	0	42.288408	0	-0.4111751
0.4887	20906.0071	0.82699897	199.099379	2.40442447	0	-0.7160065	3.04008437	-0.1887434	5.29380911	0.87772638	-6.9077553	5	80	4.02335	300.777778	27.7777778	0	0	0	0	42.288408	0	-0.4111751
0.7938	33103.1338	0.91924194	232.23903	2.28315559	0	-0.2309237	3.49965816	-0.0831187	5.44777145	0.82599641	-6.9077553	6	72	4.47038889	302.444444	29.44444444	0	0	1	0	42.288408	0	-0.4111751
1.1331	46353.8411	0.86762713	200.615841	1.6790221	0	0.12495724	3.83632573	-0.1408413	5.30139682	0.51880695	-6.9077553	7	62	3.57631111	304.111111	31.11111111	0	0	1	0	42.288408	0	-0.4111751
1.4967	61513.9887	0.88175572	207.127011	1.59572307	0	0.40326268	4.11928086	-0.1247068	5.33333701	0.46795345	-6.9077553	8	55	3.12927222	305.777778	32.7777778	0	0	1	0	42.288408	0	-0.4111751
2.0061	82022.9243	0.93849166	249.190923	2.03392476	0	0.69619254	4.40701096	-0.0624163	5.51822338	0.71045885	-6.9077553	9	50	3.57631111	306.888889	33.88888888	0	0	1	0	42.288408	0	-0.4111751
2.6316	106055.588	0.93319215	248.517963	2.32354007	0	0.96759203	4.6639728	-0.0680731	5.51551915	0.8435222	-6.9077553	10	46	2.68223333	307.444444	34.4444444	0	0	1	0	42.288408	0	-0.4111751
3.3201	134812.387	0.95365812	262.234967	2.22919587	0	1.1999949	4.90389151	-0.046402	5.56924474	0.80208941	-6.9077553	11	41	2.68223333	309.111111	36.11111111	0	0	1	0	42.288408	0	-0.4111751
4.0779	169265.441	1.02657421	307.587775	2.34242172	0	1.40558215	5.13147405	0.02720089	5.72876374	0.85161214	-6.9077553	12	38	3.12927222	309.666667	36.66666666	0	0	1	0	42.288408	0	-0.4111751
4.8105	204750.393	0.99819744	289.834344	2.15855389	0	1.57080103	5.32179652	-0.0008029	5.66931298	0.76990167	-6.9077553	13	36	3.12927222	309.111111	36.11111111	0	0	1	0	42.288408	0	-0.4111751
5.5305	236384.857	0.97640106	281.775449	2.27402405	0	1.71027823	5.46546546	-0.0228582	5.64111402	0.82199062	-6.9077553	14	38	3.12927222	308.555556	35.5555556	0	0	1	0	42.288408	0	-0.4111751
6.0606	251101.17	0.756133	177.177393	1.59866349	0	1.80180881	5.52585991	-0.2782164	5.17715709	0.46979329	-6.9077553	15	42	1.34111667	308	35	0	0	1	0	42.288408	0	-0.4111751
6.4386	260544.197	0.62001067	123.659877	1.27765735	0	1.86231113	5.56277635	-0.476407	4.81754296	0.24581059	-6.9077553	16	54	1.34111667	304.666667	31.66666666	0	0	1	0	42.288408	0	-0.4111751
6.6627	268974.566	0.73853232	167.047957	1.22403922	0	1.89652481	5.59462054	-0.3017373	5.11828693	0.20297286	-6.9077553	17	61	0.89407778	303	30	0	0	1	0	42.288408	0	-0.4111751
6.8247	275886.762	0.79224396	168.805577	1.27279816	0	1.92054838	5.61999412	-0.2316245	5.12875355	0.24200311	-6.9077553	18	66	1.34111667	301.888889	28.88888888	0	0	1	0	42.288408	0	-0.4111751
6.957	281641.28	0.74602742	143.878574	1.15366846	0	1.93974835	5.64063776	-0.2916534	4.96897666	0.14381326	-6.9077553	19	66	1.34111667	301.333333	28.33333333	0	0	1	0	42.288408	0	-0.4111751
7.0749	286639.363	0.76943451	155.192062	1.16593502	0	1.95655331	5.65822834	-0.2608006	5.0446699	0.15438067	-6.9077553	20	69	1.34111667	300.777778	27.7777778	0	0	1	0	42.288408	0	-0.4111751
7.1667	290152.439	0.67777125	119.467917	1.06578027	0	1.9694453	5.67040988	-0.3874711	4.78305623	0.06464501	-6.9077553	21	72	1.78815556	299.666667	26.66666666	0	0	1	0	42.288408	0	-0.4111751
7.2405	292915.857	0.62972506	101.059844	0.96183544	0	1.97969026	5.6798888	-0.4608852	4.61572275	-0.0378728	-6.9077553	22	78	2.23519444	299.666667	26.66666666	0	0	1	0	42.288408	0	-0.4111751
7.2684	293924.739	0.29746576	28.1126985	0.52540152	0	1.98353618	5.68332715	-1.2091001	3.33625695	-0.641691	-6.9077553	23	83	2.23519444	299.111111	26.11111111	0	0	1	0	42.288408	0	-0.4111751
0.0009	0	0	0	0	0	-7.0131158	-6.9077553	-6.9077553	-6.9077553	-6.9077553	-6.9077553	0	92	1.34111667	298	25	0	0	0	0	18.9135952	0	-0.0428739
0.0009	0	0	0	0	0	-7.0131158	-6.9077553	-6.9077553	-6.9077553	-6.9077553	-6.9077553	1	95	1.34111667	297.444444	24.44444444	0	0	0	0	18.9135952	0	-0.0428739
0.0009	0	0	0	0	0	-7.0131158	-6.9077553	-6.9077553	-6.9077553	-6.9077553	-6.9077553	2	96	2.23519444	297.444444	24.44444444	0	0	0	0	18.9135952	0	-0.0428739
0.0009	0	0	0	0	0	-7.0131158	-6.9077553	-6.9077553	-6.9077553	-6.9077553	-6.9077553	3	98	1.78815556	297.444444	24.44444444	0	0	0	0	18.9135952	0	-0.0428739
0.0009	0	0	0	0	0	-7.0131158	-6.9077553	-6.9077553	-6.9077553	-6.9077553	-6.9077553	4	93	3.12927222	298.555556	25.55555555	0	0	0	0	18.9135952	0	-0.0428739
0.0009	0	0	0	0	0	-7.0131158	-6.9077553	-6.9077553	-6.9077553	-6.9077553	-6.9077553	5	80	4.02335	300.777778	27.7777778	0	0	0	0	18.9135952	0	-0.0428739
0.0333	0	0.55434338	86.9034153	2.53521983	0	-3.4021979	-6.9077553	-0.5881686	4.46480884	0.93067472	-6.9077553	6	72	4.47038889	302.444444	29.44444444	0	0	0	0	18.9135952	0	-0.0428739
0.144	272.838201	0.57908835	90.3740711	2.26185893	0	-1.937942	-1.2952179	-0.5445749	4.50396847	0.81662903	-6.9077553	7	62	3.57631111	304.111111	31.11111111	0	0	0	0	18.9135952	0	-0.0428739
0.342	3319.51382	0.69403589	158.097994	2.33073287	0	-1.0729445	1.20011954	-0.3637918	5.06322138	0.84661171	-6.9077553	8	55	3.12927222	305.777778	32.7777778	0	0	1	0	18.9135952	0	-0.0428739
0.5364	11186.3436	0.90278696	282.425926	2.30919887	0	-0.6228751	2.4147831	-0.1011616	5.64341985	0.83733361	-6.9077553	9	50	3.57631111	306.888889	33.88888888	0	0	1	0	18.9135952	0	-0.0428739
0.6822	15662.1455	0.76788891	192.746853	1.4894202	0	-0.3824324	2.75131053	-0.2628088	5.26138288	0.39905809	-6.9077553	10	46	2.68223333	307.444444	34.4444444	0	0	1	0	18.9135952	0	-0.0428739
0.8559	19884.6066	0.71614209	156.31588	1.49516937	0	-0.1556017	2.98999619	-0.3324813	5.05188523	0.40290809	-6.9077553	11	41	2.68223333	309.111111	36.11111111	0	0	1	0	18.9135952	0	-0.0428739
1.125	29986.5097	0.91123906	253.16712	2.09389003	0	0.11778304	3.40078095	-0.0918532	5.53405377	0.73950106	-6.9077553	12	38	3.12927222	309.666667	36.66666666	0	0	1	0	18.9135952	0	-0.0428739
1.4589	40846.0793	0.9086015	247.751937	2.36800119	0	0.37768273	3.70983532	-0.0947487	5.51243203	0.86246843	-6.9077553	13	36	3.12927222	309.111111	36.11111111	0	0	1	0	18.9135952	0	-0.0428739
1.7559	52081.3381	0.86177627	222.715814	1.79015632	0	0.56298155	3.95282589	-0.1475999	5.40590107	0.5828614	-6.9077553	14	38	3.12927222	308.555556	35.5555556	0	0	1	0	18.9135952	0	-0.0428739
2.061	63324.5198	0.80299301	197.106379	1.59801598	0	0.7231913	4.1482884	-0.2181647	5.28374865	0.46938843	-6.9077553	15	42	1.34111667	308	35	0	0	1	0	18.9135952	0	-0.0428739
2.4588	78782.9947	0.85764323	217.479673	1.7691979	0	0.89967343	4.36670986	-0.1524018	5.38210999	0.57109135	-6.9077553	16	54	1.34111667	304.666667	31.66666666	0	0	1	0	18.9135952	0	-0.0428739
2.7405	92040.7371	0.91781275	236.309829	1.6807093	0	1.00814039	4.52224214	-0.0846729	5.46514801	0.51981072	-6.9077553	17	61	0.89407778	303	30	0	0	1	0	18.9135952	0	-0.0428739

2.9421	101287.231	0.84470858	192.829136	1.43660218	0	1.07912361	4.61797023	-0.1675804	5.26180968	0.36297658	-6.9077553	18	66	1.34111667	301.888889	28.88888888	0	0	1	0	18.9135952	0	-0.0428739
3.1104	108904.955	0.78901876	166.36794	1.30814186	0	1.13475134	4.69048471	-0.2356986	5.11420785	0.26937185	-6.9077553	19	66	1.34111667	301.333333	28.33333333	0	0	1	0	18.9135952	0	-0.0428739
3.2904	117201.517	0.87410416	207.329258	1.66416107	0	1.19100914	4.76390336	-0.1334124	5.33431297	0.50992186	-6.9077553	20	69	1.34111667	300.777778	27.7777778	0	0	1	0	18.9135952	0	-0.0428739
3.5055	127253.257	0.84969141	196.411055	1.64374622	0	1.25433316	4.84618711	-0.1617058	5.28021477	0.4975861	-6.9077553	21	72	1.78815556	299.666667	26.66666666	0	0	1	0	18.9135952	0	-0.0428739
3.6351	132680.541	0.72729132	140.11225	1.31065157	0	1.29063662	4.88795183	-0.3170541	4.94245103	0.27128708	-6.9077553	22	78	2.23519444	299.666667	26.66666666	0	0	1	0	18.9135952	0	-0.0428739
3.6819	134654.409	0.3739299	35.0182589	0.63743063	0	1.30342892	4.90271899	-0.9810162	3.55589816	-0.4487423	-6.9077553	23	83	2.23519444	299.111111	26.11111111	0	0	1	0	18.9135952	0	-0.0428739
0.0198	445.443739	0.81224105	187.01409	2.09560031	0	-3.9220733	-0.8064419	-0.2067277	5.23118931	0.74031713	-6.9077553	0	87	2.23519444	299.111111	26.11111111	0	0	0	0	30.519848	0	0.21141735
0.0477	1284.43389	0.62379603	117.642948	1.16780767	0	-3.0428239	0.25109632	-0.47033	4.76766268	0.15598414	-6.9077553	1	79	2.68223333	300.777778	27.7777778	0	0	0	0	30.519848	0	0.21141735
0.1044	2433.04453	0.63631718	120.875109	1.29074545	0	-2.2595256	0.88955429	-0.4504878	4.79476612	0.25599436	-6.9077553	2	71	2.68223333	302.444444	29.44444444	0	0	0	0	30.519848	0	0.21141735
0.2124	3238.41789	0.65255496	129.054696	2.52062047	0	-1.549284	1.17539365	-0.4253287	4.86024406	0.92490174	-6.9077553	3	65	4.02335	303.555556	30.55555555	0	0	0	1	30.519848	0	0.21141735
0.405	5783.77817	0.75161986	168.370455	2.90563651	0	-0.9038682	1.75523001	-0.284195	5.12617258	1.06699657	-6.9077553	4	61	3.12927222	304.666667	31.66666666	0	0	0	1	30.519848	0	0.21141735
0.6921	11585.8514	0.87797886	237.543887	2.83491272	0	-0.3680248	2.44987096	-0.1289944	5.4703566	1.04236383	-6.9077553	5	54	3.57631111	305.777778	32.7777778	0	0	0	1	30.519848	0	0.21141735
1.0035	19653.7285	0.83834204	206.136922	2.43838007	0	0.00349389	2.97831795	-0.175137	5.32854547	0.89174394	-6.9077553	6	49	4.02335	307.444444	34.4444444	0	0	0	1	30.519848	0	0.21141735
1.2258	27352.4769	0.81876463	204.409745	1.77640623	0	0.20359369	3.30884365	-0.198738	5.32013143	0.57515513	-6.9077553	7	51	4.02335	306.333333	33.33333333	0	0	0	1	30.519848	0	0.21141735
1.3599	30314.36	0.64547202	117.505165	1.19004279	0	0.30741117	3.41165452	-0.4362254	4.7664908	0.17482922	-6.9077553	8	50	2.68223333	306.333333	33.33333333	0	0	0	1	30.519848	0	0.21141735
1.4814	33008.8868	0.62225014	111.206301	1.18119371	0	0.39298759	3.49680712	-0.4728073	4.71139603	0.16737179	-6.9077553	9	52	3.12927222	306.333333	33.33333333	0	0	0	1	30.519848	0	0.21141735
1.539	34984.3268	0.6742503	126.351082	0.96181965	0	0.43113285	3.55492874	-0.3926718	4.83907231	-0.0378892	-6.9077553	10	81	2.23519444	300.222222	27.2222222	0	0	0	1	30.519848	0	0.21141735
1.5606	36037.0589	0.45747232	52.0570475	0.70660098	0	0.44507036	3.58457557	-0.7798554	3.95235939	-0.3458749	-6.9077553	11	89	1.34111667	298	25	0	0	0	1	30.519848	0	0.21141735
1.5795	36914.333	0.30745474	20.7664766	0.46513312	0	0.45710834	3.60862699	-1.1761801	3.03338814	-0.763284	-6.9077553	12	94	2.23519444	296.888889	23.88888888	0	0	0	1	30.519848	0	0.21141735
1.5939	37572.29	0.25957519	14.0343704	0.40159318	0	0.46618384	3.62629343	-1.3448638	2.6415806	-0.9098287	-6.9077553	13	94	1.34111667	297.444444	24.44444444	0	0	0	1	30.519848	0	0.21141735
1.6083	38098.6548	0.26113471	14.2528868	0.34459054	0	0.47517772	3.64020522	-1.3388967	2.65702963	-1.0625006	-6.9077553	14	95	2.23519444	296.888889	23.88888888	0	0	0	1	30.519848	0	0.21141735
1.6209	38625.0222	0.23950124	11.8877814	0.31635118	0	0.48298155	3.6539262	-1.42503	2.47559522	-1.1477463	-6.9077553	15	93	2.23519444	296.888889	23.88888888	0	0	0	1	30.519848	0	0.21141735
1.6299	38932.0699	0.23830826	11.8094383	0.31658072	0	0.48851866	3.66184402	-1.4300028	2.46898374	-1.1470232	-6.9077553	16	95	1.78815556	296.333333	23.33333333	0	0	0	1	30.519848	0	0.21141735
1.6407	39458.4326	0.24579221	12.3597997	0.31765515	0	0.49512298	3.67527312	-1.3992086	2.51453015	-1.1436458	-6.9077553	17	94	2.68223333	296.888889	23.88888888	0	0	0	1	30.519848	0	0.21141735
1.6479	39809.3445	0.25253052	13.6160927	0.37239549	0	0.49950175	3.68412679	-1.3722711	2.61132582	-0.9851171	-6.9077553	18	94	2.23519444	296.888889	23.88888888	0	0	0	1	30.519848	0	0.21141735
1.6605	40379.5739	0.25247559	14.2263184	0.4167519	0	0.50711876	3.69834882	-1.3724878	2.65516394	-0.8728676	-6.9077553	19	89	3.57631111	298	25	0	0	0	1	30.519848	0	0.21141735
1.674	40993.6663	0.29104916	18.2907776	0.46814906	0	0.51521597	3.71344197	-1.2308331	2.90645165	-0.7568347	-6.9077553	20	91	4.47038889	298	25	0	0	0	1	30.519848	0	0.21141735
1.6902	41695.4845	0.30102173	20.4157918	0.53961849	0	0.52484687	3.73041682	-1.1972563	3.01635769	-0.6150414	-6.9077553	21	93	4.47038889	296.888889	23.88888888	0	0	0	1	30.519848	0	0.21141735
1.7082	42485.0355	0.331826	24.7033081	0.65751052	0	0.53544018	3.74917545	-1.1001355	3.20697765	-0.4177748	-6.9077553	22	94	4.91742778	296.333333	23.33333333	0	0	0	1	30.519848	0	0.21141735
1.7487	43809.1876	0.48648542	80.9640408	1.23284607	0	0.55887265	3.77986638	-0.7184949	4.39401747	0.21013618	-6.9077553	23	94	4.47038889	295.777778	22.7777778	0	0	0	1	30.519848	0	0.21141735
0.018	820.492065	0.91433666	230.868601	1.58171312	0	-4.0173835	-0.196633	-0.0884634	5.44185305	0.45914054	-6.9077553	0	87	2.23519444	299.111111	26.11111111	0	0	0	0	34.0724638	0	0.49277013
0.0324	1259.12986	0.54777062	83.9050903	0.729581	0	-3.4295969	0.23121478	-0.6000747	4.4296982	-0.3139152	-6.9077553	1	79	2.68223333	300.777778	27.7777778	0	0	0	0	34.0724638	0	0.49277013
0.0693	2497.75676	0.68060284	125.270615	1.0932887	0	-2.6693104	0.91579331	-0.3833081	4.8304843	0.09010456	-6.9077553	2	71	2.68223333	302.444444	29.44444444	0	0	0	0	34.0724638	0	0.49277013
0.1215	4655.92819	0.90040128	210.011584	1.38185067	0	-2.107841	1.53835605	-0.1038047	5.34716745	0.32414707	-6.9077553	3	65	4.02335	303.555556	30.55555555	0	0	0	0	34.0724638	0	0.49277013
0.1773	7292.32677	0.87926539	200.024682	1.29290255	0	-1.7299121	1.98695979	-0.1275318	5.29844577	0.25766288	-6.9077553	4	61	3.12927222	304.666667	31.66666666	0	0	0	0	34.0724638	0	0.49277013
0.2637	11456.9864	0.9431893	252.486898	1.80278397	0	-1.3329432	2.43868699	-0.0574286	5.53136332	0.58988666	-6.9077553	5	54	3.57631111	305.777778	32.7777778	0	0	0	0	34.0724638	0	0.49277013
0.5292	17077.7421	1.18104186	425.275159	3.55361328	0	-0.6363888	2.83783454	0.16724333	6.05273874	1.26824628	-6.9077553	6	49	4.02335	307.444444	34.4444444	0	0	0	1	34.0724638	0	0.49277013
1.0089	24555.08	1.28702578	510.581262	3.79251253	0	0.00886063	3.20095948	0.25311064	6.23555176	1.33329238	-6.9077553	7	51	4.02335	306.333333	33.33333333	0	0	0	1	34.0724638	0	0.49277013
1.4085	33222.5115	0.98178376	269.860959	2.14283986	0	0.34252531	3.5032578	-0.0173662	5.59791056	0.76259855	-6.9077553	8	50	2.68223333	306.333333	33.33333333	0	0	0	1	34.0724638	0	0.49277013
1.8243	42814.4962	0.91283757	234.157096	1.93906313	0	0.60119635	3.7569001	-0.0901024	5.45599651	0.66272052	-6.9077553	9	52	3.12927222	306.333333	33.33333333	0	0	0	1	34.0724638	0	0.49277013
2.0286	50078.9629	0.81651629	181.424129	1.37469482	0	0.7073459	3.91362099	-0.2014845	5.20084306	0.31895893	-6.9077553	10	81	2.23519444	300.222222	27.2222222	0	0	0	1	34.0724638	0	0.49277013
2.1276	53588.0643	0.44788208	52.8564453	0.83077954	0	0.75499458	3.98134502	-0.8009951	3.96759858	-0.1841878	-6.9077553	11	89	1.34111667	298	25	0	0	0	1	34.0724638	0	0.49277013
2.1888	56088.2994	0.29248268	19.971283	0.50368747	0	0.78335345	4.02694505	-1.2259366	2.99434546	-0.6838159	-6.9077553	12	94	2.23519444	296.888889	23.88888888	0	0	0	1	34.0724638	0	0.49277013
2.2464	58325.3569	0.29391652	22.8936615	0.45639229	0	0.80932893	4.06605408	-1.2210629	3.13090376	-0.7822139	-6.9077553	13	94	1.34111667	297.444444	24.44444444	0	0	0	1	34.0724638	0	0.49277013
2.3031	60211.4975	0.28369922	23.2682505	0.4921842	0	0.83425604	4.09787993	-1.256322	3.14713277	-0.7068725	-6.9077553	14	95	2.23519444	296.888889	23.88888888	0	0	0	1	34.0724638	0	0.49277013
2.3517	61790.5944	0.3049244	28.8131352	0.58264552	0	0.85513847	4.12376734	-1.1844173	3.36086607	-0.5384615	-6.9077553	15	93	2.23519444	296.888889	23.88888888	0	0	0	1	34.0724638	0	0.49277013
2.3913	63150.3675	0.35938252	46.4971286	0.5562302	0	0.87183715	4.1455345	-1.0205893	3.83941207	-0.5847768	-6.9077553	16	95	1.78815556	296.333333	23.33333333	0	0	0	1	34.0724638	0	0.49277013
2.4453	64422.4226	0.50556231	90.4576497	1.00948118	0	0.89416781	4.16547727	-0.680108	4.50489284	0.01042663	-6.9077553	17	94	2.68223333	296.888889	23.88888888	0	0	0	1	34.0724638	0	0.49277013
2.502	65562.8806	0.5745347	117.434981	1.38988543	0	0.91709041	4.18302494	-0.5524558	4.76589334	0.32994055	-6.9077553	18	94	2.23519444	296.888889	23.88888888	0	0	0	1	34.0724638	0	0.49277013
2.5515	67098.1079	0.32590093	33.343002	0.65532192	0	0.93668142	4.20617075	-1.1180981	3.50687791	-0.4211039	-6.9077553	19	89	3.57631111	298	25	0	0	0	1	34.0724638	0	0.49277013
2.5803	68282.4361	0.27878168	21.4465637	0.47671905	0	0.94790567	4.22366722	-1.2737457	3.06561106	-0.7387325	-6.9077553	20	91	4.47038889	298	25	0	0	0	1	34.0724638	0	0.49277013
2.6199	69729.9416	0.29801525	28.327867	0.52832803	0	0.96313615	4.24464415	-1.2072607	3.34388132	-0.6361469	-6.9077553	21	93	4.47038889	296.888889	23.88888888	0	0	0	1	34.0724638	0	0.49277013
2.6559	71396.7617	0.26984818	16.4076065	0.45383615	0	0.97678358	4.26826652	-1.3061968	2.79780598	-0.787818	-6.9077553	22	94	4.91742778	296.333333	23.33333333	0	0	0	1	34.0724638	0	0.49277013
2.7306	74229.3141	0.41130457	66.3308606	0.91686938	0	1.00452137	4.30717261	-0.885993	4.19467033	-0.0857002	-6.9077553	23	94	4.47038889	295.777778	22.7777778	0	0	0	1	34.0724638	0	0.49277013
0.0252	1460.6615	1.05755326	303.59252	1.99438109	0	-3.6809113	0.3795738	0.05690312	5.7156897	0.69083506	-6.9077553	0	87	2.23519444	299.111111	26.11111111	0	0	0	0	42.288408	0	-0.4111751
0.1098	6097.6139	0.99158113	266.453457	1.91223145	0	-2.2090947	1.80806152	-0.0074465	5.58520334	0.64879367	-6.9077553	1	79	2.68223333	300.777778	27.7777778	0	0	0	0	42.288408	0	-0.4111751
0.3015	16274.2641	1.0541399	316.689921	2.48658745	0	-1.1989853	2.78964641	0.05367337	5.75792628	0.91131335	-6.9077553	2	71	2.68223333	302.444444	29.44444444	0	0	0	0	42.288408	0	-0.4111751
0.5958	31472.973	1.08935948	343.656154	2.8607118	0	-0.5178502	3.44916095	0.08650744	5.83964452	1.05141998	-6.9077553	3	65	4.02335	303.555556	30.55555555	0	0	1	0	42.288408	0	-0.4111751
0.8928	47733.5088	1.01583844	292.85161	2.0021551	0	-0.1133927	3.86565459	0.01669824	5.67966944	0.69472349	-6.9077553	4	61	3.12927222	304.666667	31.66666666	0	0	1	0	42.288408	0	-0.4111751
1.278	61138.5237	0.92671246	231.20568	2.31308525	0	0.24529636	4.11315853	-0.0750334	5.44331203	0.83901447	-6.9077553	5	54	3.57631111	305.777778	32.7777778	0	0	1	0	42.288408	0	-0.4111751
1.6956	75031.6881	0.84160311	187.484762	1.9416054	0	0.52803666	4.31792386	-0.1712592	5.23370291	0.66403006	-6.9077553	6	49	4.02335	307.444444	34.4444444	0	0	1	0	42.288408	0	-0.4111751
2.1195	92309.3593	0.8571708	196.423666	1.63640386	0	0.75118021	4.52515637	-0.1529521	5.28027898	0.49311197	-6.9077553	7	51	4.02335	306.333333	33.33333333	0	0	1	0	42.288408	0	-0.4111751

2.5398	109248.674	0.80746754	175.10168	1.35111761	0	0.93208534	4.69363585	-0.2126147	5.16537254	0.30167196	-6.9077553	8	50	2.68223333	306.333333	33.33333333	0	0	1	0	42.288408	0	-0.4111751
3.0231	128811.499	0.83359236	197.191152	1.61513936	0	1.10628279	4.85835785	-0.1808119	5.28417864	0.48004019	-6.9077553	9	52	3.12927222	306.333333	33.33333333	0	0	1	0	42.288408	0	-0.4111751
3.3219	141575.908	0.81905659	188.893814	1.4043489	0	1.20053691	4.95284309	-0.1983819	5.24119032	0.3402856	-6.9077553	10	81	2.23519444	300.222222	27.2222222	0	0	1	0	42.288408	0	-0.4111751
3.4479	147102.748	0.45887353	53.659668	0.75708035	0	1.23776535	4.99113811	-0.7768038	3.98268029	-0.2769659	-6.9077553	11	89	1.34111667	298	25	0	0	1	0	42.288408	0	-0.4111751
3.5379	150962.757	0.32637062	24.454585	0.53970482	0	1.26353333	5.01703979	-1.1166624	3.19685861	-0.6148818	-6.9077553	12	94	2.23519444	296.888889	23.88888888	0	0	1	0	42.288408	0	-0.4111751
3.6261	154603.455	0.28568636	18.1799802	0.47871703	0	1.28815769	5.04086995	-1.2493665	2.900376	-0.7345589	-6.9077553	13	94	1.34111667	297.444444	24.44444444	0	0	1	0	42.288408	0	-0.4111751
3.7197	158507.327	0.273291	16.617819	0.43900314	0	1.31364302	5.06580713	-1.2935657	2.81053573	-0.8209734	-6.9077553	14	95	2.23519444	296.888889	23.88888888	0	0	1	0	42.288408	0	-0.4111751
3.7953	161621.648	0.26552196	15.6261713	0.43730372	0	1.33376346	5.08526428	-1.3222986	2.74901115	-0.8248432	-6.9077553	15	93	2.23519444	296.888889	23.88888888	0	0	1	0	42.288408	0	-0.4111751
3.8799	165043.026	0.26676348	15.620473	0.43870593	0	1.35580938	5.10621226	-1.3176512	2.74864644	-0.8216491	-6.9077553	16	95	1.78815556	296.333333	23.33333333	0	0	1	0	42.288408	0	-0.4111751
3.9438	167894.174	0.25685721	14.3108933	0.40224333	0	1.37214473	5.12333982	-1.3553493	2.66109089	-0.9082151	-6.9077553	17	94	2.68223333	296.888889	23.88888888	0	0	1	0	42.288408	0	-0.4111751
4.0149	171052.372	0.26204273	14.7092826	0.39510415	0	1.39001244	5.14197562	-1.3354388	2.68854675	-0.9260781	-6.9077553	18	94	2.23519444	296.888889	23.88888888	0	0	1	0	42.288408	0	-0.4111751
4.0761	173684.196	0.25229549	13.7344809	0.37061245	0	1.40514065	5.15724444	-1.3731985	2.61998233	-0.9899038	-6.9077553	19	89	3.57631111	298	25	0	0	1	0	42.288408	0	-0.4111751
4.14	176359.877	0.23877155	12.3987745	0.34177431	0	1.42069579	5.17253233	-1.4280687	2.51767829	-1.070683	-6.9077553	20	91	4.47038889	298	25	0	0	1	0	42.288408	0	-0.4111751
4.1967	178903.977	0.25164152	13.4273178	0.34686691	0	1.4342985	5.18685481	-1.3757837	2.59736574	-1.0559353	-6.9077553	21	93	4.47038889	296.888889	23.88888888	0	0	1	0	42.288408	0	-0.4111751
4.2534	181228.768	0.23929926	12.6745644	0.36486871	0	1.44771866	5.19976567	-1.4258702	2.53967608	-1.0054807	-6.9077553	22	94	4.91742778	296.333333	23.33333333	0	0	1	0	42.288408	0	-0.4111751
4.3272	184079.916	0.29181164	22.7006523	0.48122806	0	1.46492068	5.21537542	-1.2282257	3.12243771	-0.7293381	-6.9077553	23	94	4.47038889	295.777778	22.7777778	0	0	1	0	42.288408	0	-0.4111751
0.0072	0	0.50907769	95.335859	2.53406143	0	-4.9336743	-6.9077553	-0.6731922	4.5574165	0.93021787	-6.9077553	0	87	2.23519444	299.111111	26.11111111	0	0	0	0	18.9135952	0	-0.0428739
0.0477	43.8637829	0.47246051	57.8488444	1.62756432	0	-3.0428239	-3.1041244	-0.7476868	4.05785076	0.48769884	-6.9077553	1	79	2.68223333	300.777778	27.7777778	0	0	1	0	18.9135952	0	-0.0428739
0.0963	438.759496	0.52927925	77.565927	1.16983181	0	-2.340287	-0.8215273	-0.6343515	4.35114114	0.15771444	-6.9077553	2	71	2.68223333	302.444444	29.44444444	0	0	1	0	18.9135952	0	-0.0428739
0.2349	1973.99199	0.65352799	115.922953	2.10505577	0	-1.4485954	0.68056434	-0.4238409	4.7529344	0.7448169	-6.9077553	3	65	4.02335	303.555556	30.55555555	0	0	1	0	18.9135952	0	-0.0428739
0.4086	5843.74937	0.67574599	137.630768	1.75750701	0	-0.8950186	1.76554371	-0.3904593	4.92458177	0.56446516	-6.9077553	4	61	3.12927222	304.666667	31.66666666	0	0	1	0	18.9135952	0	-0.0428739
0.6318	12241.5213	0.78722234	187.759514	1.90114372	0	-0.4591824	2.50491524	-0.2379751	5.23516729	0.64298152	-6.9077553	5	54	3.57631111	305.777778	32.7777778	0	0	1	0	18.9135952	0	-0.0428739
0.9783	22712.3101	0.84635129	222.718019	2.43208927	0	-0.0219389	3.1229511	-0.1656399	5.40591097	0.88916176	-6.9077553	6	49	4.02335	307.444444	34.4444444	0	0	1	0	18.9135952	0	-0.0428739
1.4562	39156.0918	0.87204445	232.817208	2.33252781	0	0.3758303	3.66758155	-0.1357688	5.45025793	0.8473812	-6.9077553	7	51	4.02335	306.333333	33.33333333	0	0	1	0	18.9135952	0	-0.0428739
1.9503	58133.7413	0.85971686	227.67862	1.97481305	0	0.66798321	4.06276344	-0.1499897	5.42793947	0.68097998	-6.9077553	8	50	2.68223333	306.333333	33.33333333	0	0	1	0	18.9135952	0	-0.0428739
2.4633	76240.3093	0.8213441	200.750137	1.87173344	0	0.90150191	4.33390343	-0.1955964	5.30206602	0.6273991	-6.9077553	9	52	3.12927222	306.333333	33.33333333	0	0	1	0	18.9135952	0	-0.0428739
2.7405	88199.2803	0.80353323	177.165673	1.44797169	0	1.00814039	4.47961014	-0.217493	5.17709095	0.37085413	-6.9077553	10	81	2.23519444	300.222222	27.2222222	0	0	1	0	18.9135952	0	-0.0428739
2.8422	92980.4308	0.48464557	58.8704974	0.78622707	0	1.0445784	4.5323998	-0.7222762	4.07535706	-0.2392386	-6.9077553	11	89	1.34111667	298	25	0	0	1	0	18.9135952	0	-0.0428739
2.9043	95919.3071	0.30855198	21.490965	0.47466383	0	1.0661924	4.56351771	-1.1726292	3.06767915	-0.7430439	-6.9077553	12	94	2.23519444	296.888889	23.88888888	0	0	1	0	18.9135952	0	-0.0428739
2.9727	99121.3556	0.26427482	14.812349	0.36585434	0	1.08947063	4.596355	-1.3269889	2.69552873	-1.0027904	-6.9077553	13	94	1.34111667	297.444444	24.44444444	0	0	1	0	18.9135952	0	-0.0428739
3.0303	101709.32	0.26547846	15.3256369	0.40092337	0	1.10866162	4.62212877	-1.3224619	2.72959229	-0.9114938	-6.9077553	14	95	2.23519444	296.888889	23.88888888	0	0	1	0	18.9135952	0	-0.0428739
3.0978	104648.196	0.27162875	15.8422201	0.40407585	0	1.13069218	4.65061377	-1.2996443	2.76274165	-0.9036809	-6.9077553	15	93	2.23519444	296.888889	23.88888888	0	0	1	0	18.9135952	0	-0.0428739
3.1653	107762.525	0.26090103	14.7854199	0.39914246	0	1.15224784	4.67993924	-1.3397886	2.69370919	-0.9159347	-6.9077553	16	95	1.78815556	296.333333	23.33333333	0	0	1	0	18.9135952	0	-0.0428739
3.2202	110218.899	0.26400829	15.189224	0.42097348	0	1.16944347	4.70247745	-1.3279942	2.72065206	-0.8628128	-6.9077553	17	94	2.68223333	296.888889	23.88888888	0	0	1	0	18.9135952	0	-0.0428739
3.2859	113245.503	0.27273646	16.2572373	0.42707352	0	1.18964059	4.72956689	-1.2955895	2.78859969	-0.8484603	-6.9077553	18	94	2.23519444	296.888889	23.88888888	0	0	1	0	18.9135952	0	-0.0428739
3.3498	116272.103	0.27300891	16.7543017	0.44173996	0	1.20890064	4.75594176	-1.2945947	2.81871473	-0.8147727	-6.9077553	19	89	3.57631111	298	25	0	0	1	0	18.9135952	0	-0.0428739
3.4191	119386.424	0.30155469	20.4830544	0.52020313	0	1.22937736	4.78237387	-1.1954932	3.01964675	-0.6516154	-6.9077553	20	91	4.47038889	298	25	0	0	1	0	18.9135952	0	-0.0428739
3.483	122193.709	0.26998251	16.3492174	0.42255622	0	1.24789399	4.80561574	-1.305701	2.79424119	-0.859069	-6.9077553	21	93	4.47038889	296.888889	23.88888888	0	0	1	0	18.9135952	0	-0.0428739
3.5199	123772.808	0.24547609	13.0857216	0.38119244	0	1.25843258	4.81845577	-1.4004903	2.5715981	-0.961831	-6.9077553	22	94	4.91742778	296.333333	23.33333333	0	0	1	0	18.9135952	0	-0.0428739
3.6216	127080.421	0.35924787	43.1291093	1.04244806	0	1.28691592	4.84482799	-1.020963	3.76422135	0.04253067	-6.9077553	23	94	4.47038889	295.777778	22.7777778	0	0	1	0	18.9135952	0	-0.0428739
0.0009	0	0	0	0	0	-7.0131158	-6.9077553	-6.9077553	-6.9077553	-6.9077553	-6.9077553	0	76	2.68223333	301.888889	28.88888888	0	0	0	0	30.519848	0	0.21141735
0.0036	87.7275657	0.59429808	87.8032837	0.92521032	0	-5.6268214	-2.4221847	-0.518693	4.47511029	-0.0766539	-6.9077553	1	78	2.68223333	301.333333	28.33333333	0	0	0	0	30.519848	0	0.21141735
0.0063	219.318913	0.47113339	50.695516	0.58402729	0	-5.0672056	-1.5126792	-0.7504937	3.92585719	-0.5360968	-6.9077553	2	81	3.12927222	300.777778	27.7777778	0	0	0	0	30.519848	0	0.21141735
0.0108	438.637827	0.34868817	26.5892365	0.47067385	0	-4.5282091	-0.821804	-1.0507135	3.2805441	-0.7514675	-6.9077553	3	84	3.12927222	300.777778	27.7777778	0	0	0	0	30.519848	0	0.21141735
0.0135	570.229192	0.34810122	26.5503794	0.50629282	0	-4.3050656	-0.5599648	-1.0523934	3.2790817	-0.6786669	-6.9077553	4	86	4.02335	300.222222	27.2222222	0	0	0	0	30.519848	0	0.21141735
0.0198	833.411938	0.23826449	12.9215448	0.44205175	0	-3.9220733	-0.1810281	-1.4301857	2.55897344	-0.8140687	-6.9077553	5	88	3.12927222	300.222222	27.2222222	0	0	0	0	30.519848	0	0.21141735
0.0261	1096.5947	0.29029696	17.798342	0.43775082	0	-3.64582	0.09312115	-1.233412	2.87916149	-0.8238236	-6.9077553	6	90	2.23519444	299.666667	26.66666666	0	0	0	0	30.519848	0	0.21141735
0.0324	1184.32221	0.25640382	14.5471791	0.45618606	0	-3.4295969	0.17001465	-1.3571091	2.67746584	-0.7826648	-6.9077553	7	90	2.68223333	299.666667	26.66666666	0	0	0	0	30.519848	0	0.21141735
0.0342	1272.04973	0.20213276	9.35655594	0.44941628	0	-3.3755296	0.24141539	-1.5938955	2.23618414	-0.7975831	-6.9077553	8	89	2.68223333	299.666667	26.66666666	0	0	0	0	30.519848	0	0.21141735
0.0369	1315.91353	0.25405025	13.2250481	0.36545122	0	-3.2995437	0.27529076	-1.3662947	2.58218822	-1.0038899	-6.9077553	9	88	3.12927222	300.222222	27.2222222	0	0	0	0	30.519848	0	0.21141735
0.0783	1636.23667	0.31129014	25.3366328	1.34427377	0	-2.5472077	0.49300986	-1.1638226	3.23229075	0.29659754	-6.9077553	10	81	4.02335	301.888889	28.88888888	0	0	0	0	30.519848	0	0.21141735
0.198	1998.63496	0.45722571	58.7782285	2.23927089	0	-1.6194882	0.69296464	-0.7803934	4.07378854	0.80659679	-6.9077553	11	75	5.81150556	301.888889	28.88888888	0	0	0	0	30.519848	0	0.21141735
0.2781	3417.85101	0.60979222	103.447386	1.63861324	0	-1.2797745	1.22930453	-0.4929984	4.63907281	0.49446038	-6.9077553	12	65	4.91742778	304.111111	31.11111111	0	0	0	0	30.519848	0	0.21141735
0.3789	7425.49371	0.61675753	107.321062	1.54933711	0	-0.970483	2.00505384	-0.4816592	4.67583424	0.4384724	-6.9077553	13	57	6.25854444	305.222222	32.2222222	0	0	0	0	30.519848	0	0.21141735
0.5805	16692.2165	0.69489847	141.68698	1.99040917	0	-0.5438655	2.81500244	-0.3625515	4.95361179	0.68884251	-6.9077553	14	52	5.81150556	306.888889	33.88888888	0	0	0	1	30.519848	0	0.21141735
0.8316	25759.7006	0.75384418	172.805836	2.25801945	0	-0.1844037	3.2488501	-0.2812439	5.15217441	0.81493085	-6.9077553	15	50	4.47038889	308	35	0	0	0	1	30.519848	0	0.21141735
1.1304	33639.4278	0.7159865	144.560688	2.19428446	0	0.12257155	3.51572855	-0.3326983	4.97370632	0.78631163	-6.9077553	16	46	5.36446667	308.555556	35.5555556	0	0	0	1	30.519848	0	0.21141735
1.3995	43887.5008	0.66738698	123.404656	1.43099486	0	0.33611503	3.78165234	-0.402888	4.81547695	0.35906848	-6.9077553	17	48	2.68223333	308.555556	35.5555556	0	0	0	1	30.519848	0	0.21141735
1.602	53141.3275	0.68954678	134.353516	1.20224867	0	0.47125285	3.97297374	-0.3702716	4.90048195	0.18502512	-6.9077553	18	49	3.12927222	307.444444	34.4444444	0	0	0	1	30.519848	0	0.21141735
1.7838	63884.1346	0.72330044	151.463664	1.17052966	0	0.57874592	4.1570867	-0.322549	5.02035236	0.1583103	-6.9077553	19	52	1.34111667	307.444444	34.4444444	0	0	0	1	30.519848	0	0.21141735
1.9116	74092.8058	0.77414623	166.880667	1.16700197	0	0.64794059	4.30533194	-0.2547036	5.11728498	0.15529457	-6.9077553	20	70	3.57631111	301.333333	28.33333333	0	0	0	1	30.519848	0	0.21141735
1.9737	78716.6825	0.64078671	107.190748	0.93292281	0	0.67990995	4.36586781	-0.4434993	4.67461927	-0.0683615	-6.9077553	21	84	2.68223333	299.111111	26.11111111	0	0	0	1	30.519848	0	0.21141735

1.9935	79637.8211	0.35712036	30.7204035	0.70724877	0	0.68989189	4.37750168	-1.0268861	3.42495959	-0.3449599	-6.9077553	22	87	0.44703889	299.111111	26.11111111	0	0	0	1	30.519848	0	0.21141735
2.0106	80383.5117	0.26416322	15.248601	0.4038373	0	0.69843318	4.38682152	-1.3274097	2.72455334	-0.90427	-6.9077553	23	83	0.89407778	300.222222	27.2222222	0	0	0	1	30.519848	0	0.21141735
0.0009	0	0	0	0	0	-7.0131158	-6.9077553	-6.9077553	-6.9077553	-6.9077553	-6.9077553	0	76	2.68223333	301.888889	28.88888888	0	0	0	0	34.0724638	0	0.49277013
0.0072	263.182695	0.67316375	111.930847	1.19354956	0	-4.9336743	-1.3311144	-0.3942822	4.71789018	0.17776918	-6.9077553	1	78	2.68223333	301.333333	28.33333333	0	0	0	0	34.0724638	0	0.49277013
0.0171	701.820524	0.5216563	65.5483676	0.81337643	0	-4.0686768	-0.3526537	-0.6488312	4.18280356	-0.2053326	-6.9077553	2	81	3.12927222	300.777778	27.7777778	0	0	0	0	34.0724638	0	0.49277013
0.0252	1008.867	0.38638796	33.1204597	0.50722233	0	-3.6809113	0.00981864	-0.9483286	3.5001814	-0.6768363	-6.9077553	3	84	3.12927222	300.777778	27.7777778	0	0	0	0	34.0724638	0	0.49277013
0.0333	1228.18602	0.3276217	23.9060957	0.52951972	0	-3.4021979	0.20635218	-1.112848	3.17417531	-0.6338982	-6.9077553	4	86	4.02335	300.222222	27.2222222	0	0	0	0	34.0724638	0	0.49277013
0.0414	1491.36867	0.20399936	8.89666239	0.21310653	0	-3.1844744	0.40036457	-1.5847484	2.18578859	-1.5412816	-6.9077553	5	88	3.12927222	300.222222	27.2222222	0	0	0	0	34.0724638	0	0.49277013
0.0441	1622.95993	0.2459419	12.3733978	0.33741943	0	-3.1212955	0.48486757	-1.3986022	2.51562965	-1.0834692	-6.9077553	6	90	2.23519444	299.666667	26.66666666	0	0	0	0	34.0724638	0	0.49277013
0.0522	1973.87015	0.25166469	13.7215432	0.41163333	0	-2.9526728	0.68050265	-1.375692	2.61903997	-0.8851959	-6.9077553	7	90	2.68223333	299.666667	26.66666666	0	0	0	0	34.0724638	0	0.49277013
0.0612	2368.6442	0.28680232	17.8246796	0.45301223	0	-2.7936081	0.86273982	-1.2454814	2.88064009	-0.7896311	-6.9077553	8	89	2.68223333	299.666667	26.66666666	0	0	0	0	34.0724638	0	0.49277013
0.0702	2763.41825	0.2767385	17.013324	0.37252479	0	-2.656407	1.01683021	-1.2810753	2.83405558	-0.9847709	-6.9077553	9	88	3.12927222	300.222222	27.2222222	0	0	0	0	34.0724638	0	0.49277013
0.1071	4353.53886	0.69014321	136.178389	1.29235365	0	-2.2339923	1.47121872	-0.3694082	4.91397306	0.25723858	-6.9077553	10	81	4.02335	301.888889	28.88888888	0	0	0	0	34.0724638	0	0.49277013
0.1809	7629.78978	0.8379458	194.115889	1.65245447	0	-1.7098109	2.03219135	-0.1756092	5.2684605	0.50286672	-6.9077553	11	75	5.81150556	301.888889	28.88888888	0	0	0	0	34.0724638	0	0.49277013
0.2907	13156.5471	1.00752777	291.641265	2.27224156	0	-1.2354635	2.57699552	0.00849162	5.67552793	0.82120681	-6.9077553	12	65	4.91742778	304.111111	31.11111111	0	0	0	0	34.0724638	0	0.49277013
0.5526	26213.7481	1.2593583	481.156035	3.89471415	0	-0.5931209	3.26632216	0.23139605	6.17619369	1.35987701	-6.9077553	13	57	6.25854444	305.222222	32.2222222	0	0	0	1	34.0724638	0	0.49277013
1.1943	47594.5779	1.36188232	542.545407	4.91642173	3	0.17756024	3.86273986	0.30960181	6.29627362	1.59278435	1.09894557	14	52	5.81150556	306.888889	33.88888888	0	0	0	1	34.0724638	0	0.49277013
1.71	64556.4002	1.12307894	361.441154	2.74512102	3	0.53649337	4.16755475	0.11696398	5.89010201	1.01018938	1.09894557	15	50	4.47038889	308	35	0	0	0	1	34.0724638	0	0.49277013
2.2869	81413.0809	1.04974232	317.890918	2.81934774	3	0.82719719	4.39954824	0.04949689	5.76171144	1.03686019	1.09894557	16	46	5.36446667	308.555556	35.5555556	0	0	0	1	34.0724638	0	0.49277013
2.7729	99020.8841	0.97236667	263.628964	2.11392732	3	1.0198937	4.59534088	-0.0269944	5.57454647	0.74902045	1.09894557	17	48	2.68223333	308.555556	35.5555556	0	0	0	1	34.0724638	0	0.49277013
3.2616	115189.679	0.93223979	240.046933	1.96578524	3	1.18221787	4.74658883	-0.0690931	5.48083862	0.67640035	1.09894557	18	49	3.12927222	307.444444	34.4444444	0	0	0	1	34.0724638	0	0.49277013
3.7242	129576.777	0.8535538	201.137464	1.70949973	3	1.31485206	4.86428129	-0.1571758	5.30399354	0.53678557	1.09894557	19	52	1.34111667	307.444444	34.4444444	0	0	0	1	34.0724638	0	0.49277013
3.9843	138275.889	0.89658459	238.76722	1.91028846	3	1.38236164	4.92925812	-0.1080479	5.47549329	0.6477776	1.09894557	20	70	3.57631111	301.333333	28.33333333	0	0	0	1	34.0724638	0	0.49277013
4.1409	144315.698	0.7096512	140.610453	1.37330759	3	1.42091316	4.97201018	-0.3415735	4.94600043	0.31795003	1.09894557	21	84	2.68223333	299.111111	26.11111111	0	0	0	1	34.0724638	0	0.49277013
4.1976	146070.236	0.38501576	57.8819251	0.90007637	3	1.43451293	4.98409442	-0.9518771	4.05842244	-0.1041653	1.09894557	22	87	0.44703889	299.111111	26.11111111	0	0	0	1	34.0724638	0	0.49277013
4.2417	147824.795	0.31028101	30.0619146	0.57940935	3	1.44496413	4.99603452	-1.1670592	3.40329234	-0.5440217	1.09894557	23	83	0.89407778	300.222222	27.2222222	0	0	0	1	34.0724638	0	0.49277013
0.0009	0	0	0	0	0	-7.0131158	-6.9077553	-6.9077553	-6.9077553	-6.9077553	-6.9077553	0	76	2.68223333	301.888889	28.88888888	0	0	0	0	42.288408	0	-0.4111751
0.009	394.774045	0.69149865	119.37972	1.27151373	0	-4.7105307	-0.9269118	-0.367449	4.78231771	0.24099426	-6.9077553	1	78	2.68223333	301.333333	28.33333333	0	0	0	0	42.288408	0	-0.4111751
0.0369	1754.55131	0.6413309	102.882143	1.21831992	0	-3.2995437	0.56278294	-0.4426517	4.63359381	0.19829326	-6.9077553	2	81	3.12927222	300.777778	27.7777778	0	0	0	0	42.288408	0	-0.4111751
0.072	3465.23818	0.51596426	64.6963767	0.94450525	0	-2.6310892	1.24306991	-0.6597815	4.16972066	-0.0560358	-6.9077553	3	84	3.12927222	300.777778	27.7777778	0	0	0	0	42.288408	0	-0.4111751
0.0981	4693.42419	0.36092863	30.2508671	0.67403346	0	-2.3217679	1.54637546	-1.0163082	3.40955791	-0.392993	-6.9077553	4	86	4.02335	300.222222	27.2222222	0	0	0	0	42.288408	0	-0.4111751
0.1206	5790.01933	0.29752689	19.8220154	0.49091194	0	-2.115276	1.75630833	-1.2088953	2.98684366	-0.7094556	-6.9077553	5	88	3.12927222	300.222222	27.2222222	0	0	0	0	42.288408	0	-0.4111751
0.1431	6886.61289	0.29000221	18.9888928	0.51849667	0	-1.9442116	1.92972456	-1.2344244	2.94390688	-0.6548949	-6.9077553	6	90	2.23519444	299.666667	26.66666666	0	0	0	0	42.288408	0	-0.4111751
0.1728	8290.25388	0.29120838	18.4864724	0.4830439	0	-1.7556204	2.11520121	-1.2302881	2.91709333	-0.7255797	-6.9077553	7	90	2.68223333	299.666667	26.66666666	0	0	0	0	42.288408	0	-0.4111751
0.1962	9430.71358	0.29844352	19.3490413	0.49564549	0	-1.6286207	2.2440778	-1.2058295	2.96269455	-0.6998788	-6.9077553	8	89	2.68223333	299.666667	26.66666666	0	0	0	0	42.288408	0	-0.4111751
0.2394	11514.1021	0.51792399	84.3011271	0.9287014	0	-1.4296195	2.4436594	-0.6559979	4.4344071	-0.0728918	-6.9077553	9	88	3.12927222	300.222222	27.2222222	0	0	0	0	42.288408	0	-0.4111751
0.4329	18760.9517	0.85206242	206.72669	2.44845822	0	-0.8372485	2.93183098	-0.1589226	5.33140242	0.89586687	-6.9077553	10	81	4.02335	301.888889	28.88888888	0	0	0	0	42.288408	0	-0.4111751
0.7002	30229.8076	0.89588964	230.351957	2.20380009	0	-0.3563893	3.40886152	-0.1088225	5.43961273	0.79063684	-6.9077553	11	75	5.81150556	301.888889	28.88888888	0	0	0	0	42.288408	0	-0.4111751
1.0377	41740.7947	0.8330957	184.997938	1.92869206	0	0.03700673	3.7315029	-0.1814071	5.22035008	0.65736043	-6.9077553	12	65	4.91742778	304.111111	31.11111111	0	0	1	0	42.288408	0	-0.4111751
1.3914	57070.1724	0.87711464	205.828165	1.79038198	0	0.33031043	4.04429913	-0.1299781	5.32704653	0.58298738	-6.9077553	13	57	6.25854444	305.222222	32.2222222	0	0	1	0	42.288408	0	-0.4111751
2.0034	78718.5453	0.93915405	253.27739	2.94327823	0	0.69484574	4.36589148	-0.0617115	5.53448924	1.07986371	-6.9077553	14	52	5.81150556	306.888889	33.88888888	0	0	1	0	42.288408	0	-0.4111751
2.6334	104669.862	0.94364598	250.226339	2.33879569	0	0.96827579	4.65082078	-0.056945	5.52236986	0.85006361	-6.9077553	15	50	4.47038889	308	35	0	0	1	0	42.288408	0	-0.4111751
3.3309	137933.22	1.00884695	291.645968	2.31486297	0	1.20324254	4.92677691	0.00979878	5.67554406	0.83978239	-6.9077553	16	46	5.36446667	308.555556	35.5555556	0	0	1	0	42.288408	0	-0.4111751
4.0347	174241.997	1.01435442	294.05219	2.14495178	0	1.39493195	5.16045086	0.01523774	5.68376067	0.76358318	-6.9077553	17	48	2.68223333	308.555556	35.5555556	0	0	1	0	42.288408	0	-0.4111751
4.7286	204736.833	0.97814451	277.503121	2.28111368	0	1.55362918	5.3217303	-0.021076	5.62583578	0.82510207	-6.9077553	18	49	3.12927222	307.444444	34.4444444	0	0	1	0	42.288408	0	-0.4111751
5.1606	222473.364	0.82754745	191.885938	1.47125066	0	1.64105285	5.40481188	-0.1880812	5.25690633	0.38679229	-6.9077553	19	52	1.34111667	307.444444	34.4444444	0	0	1	0	42.288408	0	-0.4111751
5.4621	235890.585	0.83454116	191.097201	1.44178039	0	1.69783333	5.46337231	-0.1796757	5.25278744	0.36657208	-6.9077553	20	70	3.57631111	301.333333	28.33333333	0	0	1	0	42.288408	0	-0.4111751
5.6511	243485.047	0.70985333	136.787435	1.25102888	0	1.73185022	5.49505964	-0.3412892	4.91843546	0.22476534	-6.9077553	21	84	2.68223333	299.111111	26.11111111	0	0	1	0	42.288408	0	-0.4111751
5.7051	245678.247	0.34084291	27.7531637	0.50425962	0	1.74136051	5.50402681	-1.073404	3.32338587	-0.6826829	-6.9077553	22	87	0.44703889	299.111111	26.11111111	0	0	1	0	42.288408	0	-0.4111751
5.7483	247608.243	0.23917838	12.224528	0.36242684	0	1.74890416	5.51185187	-1.4263734	2.50352622	-1.0121773	-6.9077553	23	83	0.89407778	300.222222	27.2222222	0	0	1	0	42.288408	0	-0.4111751
0.0009	0	0	0	0	0	-7.0131158	-6.9077553	-6.9077553	-6.9077553	-6.9077553	-6.9077553	0	76	2.68223333	301.888889	28.88888888	0	0	0	0	18.9135952	0	-0.0428739
0.0009	0	0	0	0	0	-7.0131158	-6.9077553	-6.9077553	-6.9077553	-6.9077553	-6.9077553	1	78	2.68223333	301.333333	28.33333333	0	0	0	0	18.9135952	0	-0.0428739
0.0009	0	0	0	0	0	-7.0131158	-6.9077553	-6.9077553	-6.9077553	-6.9077553	-6.9077553	2	81	3.12927222	300.777778	27.7777778	0	0	0	0	18.9135952	0	-0.0428739
0.0009	0	0	0	0	0	-7.0131158	-6.9077553	-6.9077553	-6.9077553	-6.9077553	-6.9077553	3	84	3.12927222	300.777778	27.7777778	0	0	0	0	18.9135952	0	-0.0428739
0.0009	0	0	0	0	0	-7.0131158	-6.9077553	-6.9077553	-6.9077553	-6.9077553	-6.9077553	4	86	4.02335	300.222222	27.2222222	0	0	0	0	18.9135952	0	-0.0428739
0.0009	0	0	0	0	0	-7.0131158	-6.9077553	-6.9077553	-6.9077553	-6.9077553	-6.9077553	5	88	3.12927222	300.222222	27.2222222	0	0	0	0	18.9135952	0	-0.0428739
0.0009	0	0	0	0	0	-7.0131158	-6.9077553	-6.9077553	-6.9077553	-6.9077553	-6.9077553	6	90	2.23519444	299.666667	26.66666666	0	0	0	0	18.9135952	0	-0.0428739
0.0009	0	0	0	0	0	-7.0131158	-6.9077553	-6.9077553	-6.9077553	-6.9077553	-6.9077553	7	90	2.68223333	299.666667	26.66666666	0	0	0	0	18.9135952	0	-0.0428739
0.0009	0	0	0	0	0	-7.0131158	-6.9077553	-6.9077553	-6.9077553	-6.9077553	-6.9077553	8	89	2.68223333	299.666667	26.66666666	0	0	0	0	18.9135952	0	-0.0428739
0.0009	0	0	0	0	0	-7.0131158	-6.9077553	-6.9077553	-6.9077553	-6.9077553	-6.9077553	9	88	3.12927222	300.222222	27.2222222	0	0	0	0	18.9135952	0	-0.0428739
0.0009	0	0	0	0	0	-7.0131158	-6.9077553	-6.9077553	-6.9077553	-6.9077553	-6.9077553	10	81	4.02335	301.888889	28.88888888	0	0	0	0	18.9135952	0	-0.0428739
0.0171	0	0.65739176	114.137939	3.56400977	0	-4.0686768	-6.9077553	-0.4179551	4.73741647	1.2711668	-6.9077553	11	75	5.81150556	301.888889	28.88888888	0	0	0	0	18.9135952	0	-0.0428739

0.0837	149.445886	0.58594235	100.733603	2.3327579	0	-2.4805163	-1.8941518	-0.5328287	4.61248937	0.8474798	-6.9077553	12	65	4.91742778	304.111111	31.11111111	0	0	0	0	18.9135952	0	-0.0428739
0.306	7290.91451	0.89668119	271.856655	2.80475965	0	-1.1841702	1.98676613	-0.1079403	5.6052786	1.03167432	-6.9077553	13	57	6.25854444	305.222222	32.2222222	0	0	1	0	18.9135952	0	-0.0428739
0.5427	14014.9866	0.84990921	248.107593	2.67301146	0	-0.6111986	2.64019858	-0.1614498	5.51386652	0.98357976	-6.9077553	14	52	5.81150556	306.888889	33.88888888	0	0	1	0	18.9135952	0	-0.0428739
0.7263	18601.2139	0.73057953	173.053041	1.67994361	0	-0.3197921	2.9232806	-0.3125493	5.15360392	0.51935531	-6.9077553	15	50	4.47038889	308	35	0	0	1	0	18.9135952	0	-0.0428739
0.9774	27971.4819	0.85634731	230.90009	1.94960159	0	-0.0228593	3.33122124	-0.1539122	5.44198944	0.66813783	-6.9077553	16	46	5.36446667	308.555556	35.5555556	0	0	1	0	18.9135952	0	-0.0428739
1.233	35261.6375	0.77355388	174.533038	1.87635932	0	0.20945022	3.56282398	-0.2554681	5.16211978	0.62986617	-6.9077553	17	48	2.68223333	308.555556	35.5555556	0	0	1	0	18.9135952	0	-0.0428739
1.5408	47706.4754	0.88872889	233.438094	1.85649386	0	0.43230176	3.8650881	-0.1168385	5.4529212	0.61922819	-6.9077553	18	49	3.12927222	307.444444	34.4444444	0	0	1	0	18.9135952	0	-0.0428739
1.7703	56097.0557	0.77462481	177.20648	1.3713039	0	0.57114902	4.02710115	-0.2540864	5.17732125	0.31649101	-6.9077553	19	52	1.34111667	307.444444	34.4444444	0	0	1	0	18.9135952	0	-0.0428739
1.9467	63892.6937	0.82955014	190.641821	1.48338178	0	0.66613563	4.15722067	-0.185667	5.25040163	0.39499837	-6.9077553	20	70	3.57631111	301.333333	28.33333333	0	0	1	0	18.9135952	0	-0.0428739
2.0952	70333.3532	0.73966101	144.599349	1.29758939	0	0.73964901	4.25326035	-0.3002122	4.97397372	0.26127859	-6.9077553	21	84	2.68223333	299.111111	26.11111111	0	0	1	0	18.9135952	0	-0.0428739
2.1393	72307.2262	0.24922161	13.2993525	0.42489729	0	0.76047867	4.2809379	-1.3854083	2.58779054	-0.8535571	-6.9077553	22	87	0.44703889	299.111111	26.11111111	0	0	1	0	18.9135952	0	-0.0428739
2.187	74412.6862	0.25405252	13.9445801	0.41381667	0	0.78253074	4.30963988	-1.3662858	2.63516262	-0.8799186	-6.9077553	23	83	0.89407778	300.222222	27.2222222	0	0	1	0	18.9135952	0	-0.0428739
0.0171	492.64356	0.81154357	181.87363	1.73146354	0	-4.0686768	-0.7059416	-0.2075857	5.2033176	0.54954441	-6.9077553	0	60	3.57631111	304.666667	31.66666666	0	0	0	0	30.519848	0	0.21141735
0.1332	2641.30621	0.88128751	231.131526	3.95428987	0	-2.0159035	0.9716521	-0.1252373	5.44299125	1.37505389	-6.9077553	1	57	4.91742778	304.111111	31.11111111	0	0	0	0	30.519848	0	0.21141735
0.2349	4639.87681	0.65089417	120.71754	1.45074909	0	-1.4485954	1.53490332	-0.4278731	4.79346172	0.3727691	-6.9077553	2	70	1.78815556	303	30	0	0	0	0	30.519848	0	0.21141735
0.5571	6606.00688	0.7831455	172.831398	3.70643795	0	-0.5850105	1.88813074	-0.2431607	5.15232233	1.31034106	-6.9077553	3	51	4.47038889	306.888889	33.88888888	0	0	0	1	30.519848	0	0.21141735
1.0143	10386.8069	0.81422364	203.333338	2.79405825	0	0.01419872	2.3406327	-0.2042928	5.31485161	1.02785295	-6.9077553	4	50	3.57631111	306.888889	33.88888888	0	0	0	1	30.519848	0	0.21141735
1.62	17636.0083	0.81839683	198.176495	2.5520941	0	0.48242615	2.86999944	-0.1991868	5.28916307	0.93730599	-6.9077553	5	52	3.57631111	306.888889	33.88888888	0	0	0	1	30.519848	0	0.21141735
2.0961	25531.9944	0.79800825	178.911389	2.34274044	0	0.74007848	3.23997151	-0.224384	5.18689624	0.85174813	-6.9077553	6	43	4.02335	308	35	0	0	0	1	30.519848	0	0.21141735
2.3895	31032.5149	0.76312261	164.550949	1.78358862	0	0.87108414	3.43506775	-0.269027	5.10322632	0.57918792	-6.9077553	7	48	3.12927222	307.444444	34.4444444	0	0	0	1	30.519848	0	0.21141735
2.7108	39548.8715	0.76310546	172.290682	1.44981269	0	0.99724379	3.67756245	-0.2690495	5.14918887	0.37212387	-6.9077553	8	50	2.23519444	306.888889	33.88888888	0	0	0	1	30.519848	0	0.21141735
2.9367	45740.0237	0.79293665	200.117421	1.48747405	0	1.0772865	3.82299557	-0.2307516	5.2989093	0.39775147	-6.9077553	9	57	0.44703889	304.666667	31.66666666	0	0	0	1	30.519848	0	0.21141735
3.0303	47647.0939	0.66507706	122.056537	0.7910449	0	1.10866162	3.86384263	-0.4063499	4.80449255	-0.2331372	-6.9077553	10	73	0.89407778	301.333333	28.33333333	0	0	0	1	30.519848	0	0.21141735
3.0492	48173.4611	0.55156762	80.3364781	0.98824755	0	1.11487926	3.87482903	-0.5931795	4.38623624	-0.0108107	-6.9077553	11	76	0.44703889	300.222222	27.2222222	0	0	0	1	30.519848	0	0.21141735
3.0627	48699.826	0.42191346	42.2675496	0.63115247	0	1.11929688	3.88569599	-0.8605877	3.7440433	-0.4586247	-6.9077553	12	75	1.78815556	300.777778	27.7777778	0	0	0	1	30.519848	0	0.21141735
3.0681	48919.144	0.34895515	26.7910817	0.4847405	0	1.12105848	3.89018925	-1.0499503	3.28810639	-0.7220808	-6.9077553	13	76	2.23519444	300.222222	27.2222222	0	0	0	1	30.519848	0	0.21141735
3.069	48919.144	0.19532011	7.45945454	0.17690368	0	1.12135178	3.89018925	-1.6280087	2.00961634	-1.726513	-6.9077553	14	79	3.12927222	300.222222	27.2222222	0	0	0	1	30.519848	0	0.21141735
3.069	48919.144	0	0	0	0	1.12135178	3.89018925	-6.9077553	-6.9077553	-6.9077553	-6.9077553	15	83	4.02335	299.666667	26.66666666	0	0	0	1	30.519848	0	0.21141735
3.069	48919.144	0	0	0	0	1.12135178	3.89018925	-6.9077553	-6.9077553	-6.9077553	-6.9077553	16	87	4.02335	299.111111	26.11111111	0	0	0	1	30.519848	0	0.21141735
3.069	48919.144	0	0	0	0	1.12135178	3.89018925	-6.9077553	-6.9077553	-6.9077553	-6.9077553	17	89	3.57631111	298.555556	25.55555555	0	0	0	1	30.519848	0	0.21141735
3.069	48919.144	0	0	0	0	1.12135178	3.89018925	-6.9077553	-6.9077553	-6.9077553	-6.9077553	18	92	4.02335	298.555556	25.55555555	0	0	0	1	30.519848	0	0.21141735
3.069	48919.144	0	0	0	0	1.12135178	3.89018925	-6.9077553	-6.9077553	-6.9077553	-6.9077553	19	88	4.91742778	298.555556	25.55555555	0	0	0	1	30.519848	0	0.21141735
3.069	48919.144	0	0	0	0	1.12135178	3.89018925	-6.9077553	-6.9077553	-6.9077553	-6.9077553	20	87	4.02335	298.555556	25.55555555	0	0	0	1	30.519848	0	0.21141735
3.0906	48975.0119	0.14791028	6.25762304	0.82456454	0	1.12836525	3.89133063	-1.9044113	1.8339602	-0.1916878	-6.9077553	21	82	4.91742778	299.666667	26.66666666	0	0	0	1	30.519848	0	0.21141735
3.2562	49457.0433	0.43085869	57.89123	1.42872271	0	1.18056087	3.9011247	-0.8396568	4.05858318	0.35748052	-6.9077553	22	74	5.36446667	301.333333	28.33333333	0	0	0	1	30.519848	0	0.21141735
3.6531	52203.472	0.56077628	88.1229539	1.58441522	0	1.29557612	3.95516816	-0.5766516	4.47874439	0.46084634	-6.9077553	23	68	5.36446667	302.444444	29.44444444	0	0	0	1	30.519848	0	0.21141735
0.0441	2135.12292	1.14288298	380.988973		0	-3.1212955	0.75899247	0.1344286	5.94277306		-6.9077553	0	60	3.57631111	304.666667	31.66666666	0	0	0	0	34.0724638	0	0.49277013
0.1017	4760.55719	0.88604295	210.260605		0	-2.285728	1.56057476	-0.1198619	5.34835249		-6.9077553	1	57	4.91742778	304.111111	31.11111111	0	0	0	0	34.0724638	0	0.49277013
0.1782	7538.98757	0.84217637	185.274494		0	-1.7248488	2.02022053	-0.1705791	5.22184388		-6.9077553	2	70	1.78815556	303	30	0	0	0	0	34.0724638	0	0.49277013
0.2673	10348.3598	0.85515171	191.801708		0	-1.3193837	2.33692466	-0.1553077	5.25646728		-6.9077553	3	51	4.47038889	306.888889	33.88888888	0	0	0	1	34.0724638	0	0.49277013
0.378	16287.2104	1.00416608	279.36706		0	-0.9728611	2.79044156	0.00515279	5.63253013		-6.9077553	4	50	3.57631111	306.888889	33.88888888	0	0	0	1	34.0724638	0	0.49277013
0.612	27877.7904	1.09111551	335.926022		0	-0.491023	3.3278662	0.08811665	5.81689394		-6.9077553	5	52	3.57631111	306.888889	33.88888888	0	0	0	1	34.0724638	0	0.49277013
0.9342	42029.5224	1.01534565	282.118933		0	-0.0680647	3.73839608	0.0162135	5.64233228		-6.9077553	6	43	4.02335	308	35	0	0	0	1	34.0724638	0	0.49277013
1.3527	62233.4252	1.04161561	301.66129		0	0.3021026	4.13090831	0.04173256	5.70930815		-6.9077553	7	48	3.12927222	307.444444	34.4444444	0	0	0	1	34.0724638	0	0.49277013
1.8144	78998.2974	0.91048237	230.086714		0	0.59575483	4.36943896	-0.092683	5.4384606		-6.9077553	8	50	2.23519444	306.888889	33.88888888	0	0	0	1	34.0724638	0	0.49277013
2.1825	92754.303	0.80310036	179.928484		0	0.78047101	4.52996488	-0.2180312	5.19256502		-6.9077553	9	57	0.44703889	304.666667	31.66666666	0	0	0	1	34.0724638	0	0.49277013
2.4345	103078.181	0.79869036	176.967271		0	0.8897414	4.63549744	-0.2235307	5.17597046		-6.9077553	10	73	0.89407778	301.333333	28.33333333	0	0	0	1	34.0724638	0	0.49277013
2.5695	107289.094	0.55371892	79.3074089		0	0.94371133	4.67553632	-0.5892937	4.37334416		-6.9077553	11	76	0.44703889	300.222222	27.2222222	0	0	0	1	34.0724638	0	0.49277013
2.6775	110666.622	0.46675142	59.4358236		0	0.98488352	4.70653131	-0.7598183	4.08491396		-6.9077553	12	75	1.78815556	300.777778	27.7777778	0	0	0	1	34.0724638	0	0.49277013
2.7558	113210.709	0.46945506	71.1887235		0	1.01370778	4.7292596	-0.7540548	4.26534848		-6.9077553	13	76	2.23519444	300.222222	27.2222222	0	0	0	1	34.0724638	0	0.49277013
2.8476	115891.642	0.58882698	147.030609		0	1.04647653	4.75266426	-0.527926	4.99064759		-6.9077553	14	79	3.12927222	300.222222	27.2222222	0	0	0	1	34.0724638	0	0.49277013
2.9457	118611.194	0.62155305	171.214073		0	1.08034648	4.7758593	-0.4739264	5.1429205		-6.9077553	15	83	4.02335	299.666667	26.66666666	0	0	0	1	34.0724638	0	0.49277013
3.0069	120935.975	0.34913226	45.4911499		0	1.10090965	4.79526954	-1.0494443	3.81753978		-6.9077553	16	87	4.02335	299.111111	26.11111111	0	0	0	1	34.0724638	0	0.49277013
3.0888	123699.395	0.43404208	89.754577		0	1.12778267	4.81786247	-0.8323125	4.49709016		-6.9077553	17	89	3.57631111	298.555556	25.55555555	0	0	0	1	34.0724638	0	0.49277013
3.1671	126111.904	0.4651049	86.4225148		0	1.15281634	4.83717757	-0.7633446	4.4592598		-6.9077553	18	92	4.02335	298.555556	25.55555555	0	0	0	1	34.0724638	0	0.49277013
3.2463	128862.346	0.46742075	101.72462		0	1.17751589	4.85875251	-0.7583883	4.62227919		-6.9077553	19	88	4.91742778	298.555556	25.55555555	0	0	0	1	34.0724638	0	0.49277013
3.3498	131976.676	0.68908526	195.147809		0	1.20890064	4.88263279	-0.3709401	5.27376239		-6.9077553	20	87	4.02335	298.555556	25.55555555	0	0	0	1	34.0724638	0	0.49277013
3.6567	140872.776	0.63045737	139.43498		0	1.2965611	4.94786429	-0.4597249	4.93760557		-6.9077553	21	82	4.91742778	299.666667	26.66666666	0	0	0	1	34.0724638	0	0.49277013
4.4919	156358.19	0.74067116	179.530257		0	1.50227577	5.05215586	-0.2988493	5.19034932		-6.9077553	22	74	5.36446667	301.333333	28.33333333	0	0	0	1	34.0724638	0	0.49277013
5.5773	182884.971	0.91023783	258.311515		1	1.71870479	5.20886285	-0.0929513	5.55417015		0.0009995	23	68	5.36446667	302.444444	29.44444444	0	0	0	1	34.0724638	0	0.49277013
0.045	3113.02022	1.21840458	411.326291	2.47235092	0	-3.1010928	1.13591457	0.19836269	6.01938923	0.90557388	-6.9077553	0	60	3.57631111	304.666667	31.66666666	0	0	0	0	42.288408	0	-0.4111751
0.1386	7434.6327	0.96978862	256.123761	2.15236928	0	-1.9761632	2.00628367	-0.0296465	5.54566467	0.76703372	-6.9077553	1	57	4.91742778	304.111111	31.11111111	0	0	0	0	42.288408	0	-0.4111751

0.3294	16855.8234	1.00528372	283.992685	2.08804408	0	-1.1104825	2.82475553	0.00626406	5.648952	0.73670658	-6.9077553	2	70	1.78815556	303	30	0	0	0	0	42.288408	0	-0.4111751
0.6579	30227.3268	1.0190586	284.417188	2.60201115	0	-0.4187023	3.40877946	0.01986008	5.65044565	0.95666891	-6.9077553	3	51	4.47038889	306.888889	33.88888888	0	0	1	0	42.288408	0	-0.4111751
1.0359	48880.305	1.01129492	280.804483	2.0811599	0	0.03527061	3.88939501	0.01221995	5.6376622	0.73340577	-6.9077553	4	50	3.57631111	306.888889	33.88888888	0	0	1	0	42.288408	0	-0.4111751
1.4922	66527.4488	0.97537436	261.770741	2.54890421	0	0.40025154	4.19762966	-0.0239092	5.56747291	0.93605579	-6.9077553	5	52	3.57631111	306.888889	33.88888888	0	0	1	0	42.288408	0	-0.4111751
2.0205	83983.9994	0.86928313	214.984109	2.52243307	0	0.70334501	4.4306382	-0.1389367	5.37056876	0.9256203	-6.9077553	6	43	4.02335	308	35	0	0	1	0	42.288408	0	-0.4111751
2.5623	102324.557	0.79728349	186.591129	1.96468599	0	0.94090529	4.62815946	-0.2252915	5.2289251	0.67584129	-6.9077553	7	48	3.12927222	307.444444	34.4444444	0	0	1	0	42.288408	0	-0.4111751
3.1167	124162.853	0.83006004	209.504845	1.74746189	0	1.13677475	4.82160209	-0.1850532	5.34475164	0.55873648	-6.9077553	8	50	2.23519444	306.888889	33.88888888	0	0	1	0	42.288408	0	-0.4111751
3.5784	141085.348	0.77450068	177.227629	1.46600282	0	1.27491577	4.9493721	-0.2542464	5.17744059	0.38322142	-6.9077553	9	57	0.44703889	304.666667	31.66666666	0	0	1	0	42.288408	0	-0.4111751
3.8943	154901.247	0.82623178	190.220998	1.42879345	0	1.35951395	5.04279425	-0.1896704	5.2481918	0.35752999	-6.9077553	10	73	0.89407778	301.333333	28.33333333	0	0	1	0	42.288408	0	-0.4111751
4.0311	160778.999	0.55191632	75.0632645	0.87983904	0	1.39403929	5.08003697	-0.5925486	4.31834461	-0.1268804	-6.9077553	11	76	0.44703889	300.222222	27.2222222	0	0	1	0	42.288408	0	-0.4111751
4.1472	165691.741	0.42786759	43.1670906	0.66468427	0	1.42243341	5.11013511	-0.846607	3.76510158	-0.4069398	-6.9077553	12	75	1.78815556	300.777778	27.7777778	0	0	1	0	42.288408	0	-0.4111751
4.2156	168499.024	0.35051266	33.9550267	0.60357766	0	1.43879193	5.12693589	-1.0455096	3.52506636	-0.5032251	-6.9077553	13	76	2.23519444	300.222222	27.2222222	0	0	1	0	42.288408	0	-0.4111751
4.2651	170560.62	0.25996489	14.6221424	0.39346022	0	1.45046563	5.13909664	-1.3433694	2.68260537	-0.930237	-6.9077553	14	79	3.12927222	300.222222	27.2222222	0	0	1	0	42.288408	0	-0.4111751
4.32	173016.992	0.25408757	14.1664139	0.39809618	0	1.4632554	5.15339559	-1.3661484	2.65094453	-0.9185528	-6.9077553	15	83	4.02335	299.666667	26.66666666	0	0	1	0	42.288408	0	-0.4111751
4.3749	175385.625	0.26964275	16.0178513	0.46658585	0	1.47588366	5.16699282	-1.3069556	2.77376623	-0.7601723	-6.9077553	16	87	4.02335	299.111111	26.11111111	0	0	1	0	42.288408	0	-0.4111751
4.4388	178149.055	0.25045011	14.1611603	0.43662797	0	1.49038407	5.1826262	-1.3805107	2.65057364	-0.8263861	-6.9077553	17	89	3.57631111	298.555556	25.55555555	0	0	1	0	42.288408	0	-0.4111751
4.4955	180605.427	0.25582849	14.6652386	0.47098983	0	1.5030769	5.19632023	-1.3593468	2.68554816	-0.7507978	-6.9077553	18	92	4.02335	298.555556	25.55555555	0	0	1	0	42.288408	0	-0.4111751
4.5549	183017.936	0.2633161	15.8359615	0.50840846	0	1.51620358	5.20958962	-1.3306096	2.76234655	-0.6745051	-6.9077553	19	88	4.91742778	298.555556	25.55555555	0	0	1	0	42.288408	0	-0.4111751
4.6152	185781.356	0.28692772	21.2152938	0.51143219	0	1.5293552	5.22457586	-1.2450458	3.05476946	-0.6685869	-6.9077553	20	87	4.02335	298.555556	25.55555555	0	0	1	0	42.288408	0	-0.4111751
4.8402	191866.402	0.5473634	106.074859	1.36631934	0	1.57695604	5.25680452	-0.6008171	4.66415449	0.31285213	-6.9077553	21	82	4.91742778	299.666667	26.66666666	0	0	1	0	42.288408	0	-0.4111751
5.4648	205446.566	0.60336515	122.227024	1.84702426	0	1.69832752	5.32519085	-0.5035767	4.80588835	0.6141171	-6.9077553	22	74	5.36446667	301.333333	28.33333333	0	0	1	0	42.288408	0	-0.4111751
6.2181	223897.129	0.71817217	175.544224	2.11597526	0	1.82746439	5.41119117	-0.3296545	5.1678967	0.7499883	-6.9077553	23	68	5.36446667	302.444444	29.44444444	0	0	1	0	42.288408	0	-0.4111751
0.0432	0	0.79738114	182.947183	4.43872265	0	-3.1419148	-6.9077553	-0.2251692	5.20920296	1.49059191	-6.9077553	0	60	3.57631111	304.666667	31.66666666	0	0	0	0	18.9135952	0	-0.0428739
0.2106	47.0606856	0.52511896	74.1319094	2.84993555	0	-1.5577947	-3.0352908	-0.6422279	4.30585956	1.0476472	-6.9077553	1	57	4.91742778	304.111111	31.11111111	0	0	1	0	18.9135952	0	-0.0428739
0.3312	310.243378	0.41459012	48.9793391	1.32418368	0	-1.1050329	-1.1671801	-0.8780558	3.89141898	0.28155107	-6.9077553	2	70	1.78815556	303	30	0	0	1	0	18.9135952	0	-0.0428739
0.5796	1718.01225	0.61725838	136.716542	2.60171266	0	-0.5454171	0.54174985	-0.4808488	4.91791706	0.95655423	-6.9077553	3	51	4.47038889	306.888889	33.88888888	0	0	1	0	18.9135952	0	-0.0428739
0.8568	7798.06897	0.78820979	200.821568	2.16393131	0	-0.1545508	2.05400436	-0.2367231	5.30242177	0.77238864	-6.9077553	4	50	3.57631111	306.888889	33.88888888	0	0	1	0	18.9135952	0	-0.0428739
1.1439	14932.2591	0.77593913	175.863796	1.97966324	0	0.13444348	2.70359088	-0.2523933	5.16971549	0.68343176	-6.9077553	5	52	3.57631111	306.888889	33.88888888	0	0	1	0	18.9135952	0	-0.0428739
1.4958	23201.8327	0.7632131	171.866868	2.55557228	0	0.40266118	3.14427437	-0.2689086	5.14672597	0.93866741	-6.9077553	6	43	4.02335	308	35	0	0	1	0	18.9135952	0	-0.0428739
1.8009	35732.5551	0.88030637	230.063237	1.80859627	0	0.58828654	3.57609016	-0.12635	5.43835856	0.59310377	-6.9077553	7	48	3.12927222	307.444444	34.4444444	0	0	1	0	18.9135952	0	-0.0428739
2.1033	46860.4466	0.81711796	201.388253	1.56720007	0	0.74350754	3.8471953	-0.2007487	5.30523962	0.44992851	-6.9077553	8	50	2.23519444	306.888889	33.88888888	0	0	1	0	18.9135952	0	-0.0428739
2.4219	58548.4783	0.80827573	202.329714	1.6219291	0	0.88455236	4.06987218	-0.2116156	5.30990356	0.48423261	-6.9077553	9	57	0.44703889	304.666667	31.66666666	0	0	1	0	18.9135952	0	-0.0428739
2.6361	68745.6959	0.85545029	201.898503	1.50245346	0	0.96930055	4.23042868	-0.154959	5.30777006	0.40776477	-6.9077553	10	73	0.89407778	301.333333	28.33333333	0	0	1	0	18.9135952	0	-0.0428739
2.7855	75500.711	0.58378169	85.082796	0.97356047	0	1.02442739	4.32415532	-0.5365167	4.44363661	-0.0257687	-6.9077553	11	76	0.44703889	300.222222	27.2222222	0	0	1	0	18.9135952	0	-0.0428739
2.8935	80413.459	0.45388568	48.9456177	0.72746894	0	1.06246684	4.387194	-0.7877091	3.89073027	-0.3168103	-6.9077553	12	75	1.78815556	300.777778	27.7777778	0	0	1	0	18.9135952	0	-0.0428739
2.9799	84405.0638	0.37010415	32.2180634	0.651009	0	1.09188974	4.43563924	-0.9912725	3.47255831	-0.4276969	-6.9077553	13	76	2.23519444	300.222222	27.2222222	0	0	1	0	18.9135952	0	-0.0428739
3.0591	88045.7575	0.28996966	19.065463	0.47598639	0	1.11812076	4.47786801	-1.2345363	2.94793093	-0.7402673	-6.9077553	14	79	3.12927222	300.222222	27.2222222	0	0	1	0	18.9135952	0	-0.0428739
3.1293	91247.8122	0.27655249	16.653721	0.42532099	0	1.14080934	4.51358998	-1.2817452	2.81269372	-0.8525627	-6.9077553	15	83	4.02335	299.666667	26.66666666	0	0	1	0	18.9135952	0	-0.0428739
3.186	93704.1881	0.25277813	13.7326815	0.39080008	0	1.15876621	4.54015356	-1.3712949	2.61985132	-0.9370036	-6.9077553	16	87	4.02335	299.111111	26.11111111	0	0	1	0	18.9135952	0	-0.0428739
3.2355	96072.8256	0.24221807	12.4191761	0.36226103	0	1.17418348	4.56511691	-1.4137968	2.51932226	-1.0126336	-6.9077553	17	89	3.57631111	298.555556	25.55555555	0	0	1	0	18.9135952	0	-0.0428739
3.303	99055.5702	0.25163864	20.8595817	0.44447372	0	1.19483115	4.5956911	-1.3757951	3.03786134	-0.808617	-6.9077553	18	92	4.02335	298.555556	25.55555555	0	0	1	0	18.9135952	0	-0.0428739
3.3714	102257.624	0.23964603	12.6634722	0.39253511	0	1.21532809	4.62750513	-1.4244282	2.53880061	-0.932585	-6.9077553	19	88	4.91742778	298.555556	25.55555555	0	0	1	0	18.9135952	0	-0.0428739
3.4533	106029.905	0.31366401	26.8509293	0.46952941	0	1.2393303	4.66373061	-1.1562499	3.29033767	-0.7538968	-6.9077553	20	87	4.02335	298.555556	25.55555555	0	0	1	0	18.9135952	0	-0.0428739
3.7746	119587.008	0.69322102	144.205488	1.40182999	0	1.32829442	4.78405257	-0.3649649	4.97124622	0.33849162	-6.9077553	21	82	4.91742778	299.666667	26.66666666	0	0	1	0	18.9135952	0	-0.0428739
4.5207	143195.493	0.76631457	179.235487	2.41301923	0	1.50866685	4.96421777	-0.2648584	5.18870609	0.88129309	-6.9077553	22	74	5.36446667	301.333333	28.33333333	0	0	1	0	18.9135952	0	-0.0428739
5.3685	173214.227	0.85351141	213.809647	2.26802572	0	1.68054854	5.15453491	-0.1572254	5.3650908	0.81935054	-6.9077553	23	68	5.36446667	302.444444	29.44444444	0	0	1	0	18.9135952	0	-0.0428739
0.009	316.587898	0.83120759	188.886	1.32515293	0	-4.7105307	-1.1470007	-0.1836734	5.24114895	0.28228221	-6.9077553	0	97	3.12927222	293	20	0	0	0	0	30.519848	0	0.21141735
0.0117	448.179249	0.43795494	46.4011536	0.79950301	0	-4.4481664	-0.8003333	-0.8233585	3.83734587	-0.222515	-6.9077553	1	97	2.68223333	293	20	0	0	0	0	30.519848	0	0.21141735
0.0216	886.817219	0.45204284	47.4444525	0.70148715	0	-3.835062	-0.1189894	-0.7917686	3.85958068	-0.3531282	-6.9077553	2	93	4.47038889	293.555556	20.55555555	0	0	0	0	30.519848	0	0.21141735
0.0315	1149.99979	0.51464415	62.5768488	0.86796405	0	-3.4577677	0.14063095	-0.6623384	4.13641136	-0.1404535	-6.9077553	3	92	2.68223333	293.555556	20.55555555	0	0	0	0	30.519848	0	0.21141735
0.036	1281.59121	0.43381453	45.3322296	0.75351858	0	-3.3242363	0.24888241	-0.8328357	3.81404031	-0.2816754	-6.9077553	4	91	2.68223333	294.111111	21.11111111	0	0	0	0	30.519848	0	0.21141735
0.0585	1632.50127	0.32473682	30.7236719	0.9787468	0	-2.8387285	0.49072573	-1.1216655	3.42506598	-0.0204611	-6.9077553	5	91	2.68223333	294.666667	21.66666666	0	0	0	0	30.519848	0	0.21141735
0.1125	1922.07767	0.36595821	36.3991781	1.20059166	0	-2.1848021	0.65392686	-1.0025073	3.59457367	0.18364706	-6.9077553	6	85	3.57631111	296.333333	23.33333333	0	0	0	0	30.519848	0	0.21141735
0.2277	2853.74205	0.56694478	93.8840637	2.33711863	0	-1.4797263	1.04898149	-0.5657311	4.54207131	0.8493466	-6.9077553	7	71	5.36446667	298.555556	25.55555555	0	0	0	0	30.519848	0	0.21141735
0.3573	3910.83241	0.62366189	107.817885	2.24732908	0	-1.0291795	1.36400591	-0.4705448	4.68045283	0.81018731	-6.9077553	8	67	5.81150556	299.666667	26.66666666	0	0	0	0	30.519848	0	0.21141735
0.5499	11379.0354	0.73206636	155.317173	2.16338798	0	-0.5980188	2.43186054	-0.310519	5.04547574	0.77213763	-6.9077553	9	55	6.25854444	301.888889	28.88888888	0	0	0	1	30.519848	0	0.21141735
0.8478	22358.2811	0.84409962	211.74476	2.88485856	0	-0.1651105	3.1072415	-0.1683008	5.35538631	1.05982245	-6.9077553	10	50	5.81150556	303.555556	30.55555555	0	0	0	1	30.519848	0	0.21141735
1.1691	32582.6358	0.72288304	144.572019	2.18996534	0	0.15623422	3.48381019	-0.3231255	4.9737847	0.78434224	-6.9077553	11	45	5.36446667	304.111111	31.11111111	0	0	0	1	30.519848	0	0.21141735
1.4184	41583.0052	0.6918779	137.046029	1.46954191	0	0.34952948	3.7277156	-0.3669015	4.92032414	0.38563098	-6.9077553	12	46	3.57631111	304.111111	31.11111111	0	0	0	1	30.519848	0	0.21141735
1.6695	55756.6192	0.7098532	145.189292	1.40730313	0	0.51252418	4.02101407	-0.3412893	4.97804524	0.34238553	-6.9077553	13	44	4.02335	304.666667	31.66666666	0	0	0	1	30.519848	0	0.21141735
1.8828	70909.8235	0.68046654	131.153943	1.11988438	0	0.63276003	4.26142308	-0.3835081	4.87637939	0.114118	-6.9077553	14	49	3.12927222	303	30	0	0	0	1	30.519848	0	0.21141735
2.0412	84523.8334	0.63479541	116.226984	0.87360339	0	0.71353787	4.43704538	-0.4528785	4.75555364	-0.1339848	-6.9077553	15	53	3.12927222	301.333333	28.33333333	0	0	0	1	30.519848	0	0.21141735

2.16	96334.8437	0.67965496	128.910763	0.84404893	0	0.77010822	4.56784046	-0.3846998	4.85912816	-0.1683607	-6.9077553	16	58	1.78815556	299.111111	26.11111111	0	0	0	1	30.519848	0	0.21141735
2.2905	110785.622	0.65483262	127.804337	0.93328058	0	0.82877013	4.70760603	-0.4218497	4.8505083	-0.0679785	-6.9077553	17	51	2.68223333	298.555556	25.55555555	0	0	0	1	30.519848	0	0.21141735
2.4525	127176.873	0.62997259	115.736643	0.91654239	0	0.89710791	4.84558668	-0.4604929	4.75132593	-0.0860565	-6.9077553	18	53	2.23519444	297.444444	24.44444444	0	0	0	1	30.519848	0	0.21141735
2.5587	141884.273	0.71531955	143.182369	0.92895605	0	0.93949932	4.9550188	-0.3336289	4.96412611	-0.072618	-6.9077553	19	62	2.68223333	296.333333	23.33333333	0	0	0	1	30.519848	0	0.21141735
2.6388	152800.319	0.71394211	137.387399	1.01876068	0	0.97032427	5.02913851	-0.3355537	4.92281194	0.01956797	-6.9077553	20	73	3.12927222	295.777778	22.7777778	0	0	0	1	30.519848	0	0.21141735
2.6757	158588.453	0.54809394	80.1262326	0.73039995	0	0.98421103	5.06631881	-0.5994857	4.38361578	-0.3127948	-6.9077553	21	80	2.23519444	295.222222	22.2222222	0	0	0	1	30.519848	0	0.21141735
2.6784	158644.914	0.27502582	17.407107	0.57275605	0	0.9852196	5.06667476	-1.2872608	2.85693602	-0.555551	-6.9077553	22	84	3.12927222	295.222222	22.2222222	0	0	0	1	30.519848	0	0.21141735
2.6802	158688.776	0.21848074	9.74872684	0.54958749	0	0.98589142	5.0669512	-1.5164908	2.27723927	-0.5967694	-6.9077553	23	88	2.23519444	294.666667	21.66666666	0	0	0	1	30.519848	0	0.21141735
0.0135	688.016834	1.01525797	284.045898	1.91829245	0	-4.3050656	-0.3724896	0.01612723	5.64913936	0.6519566	-6.9077553	0	97	3.12927222	293	20	0	0	0	0	34.0724638	0	0.49277013
0.0243	1082.79075	0.4735562	53.490977	0.68822122	0	-3.7172789	0.08046485	-0.7453752	3.97953168	-0.372193	-6.9077553	1	97	2.68223333	293	20	0	0	0	0	34.0724638	0	0.49277013
0.0387	1565.29241	0.38551682	36.0522232	0.69075167	0	-3.2519157	0.4487113	-0.9505799	3.58499627	-0.3685282	-6.9077553	2	93	4.47038889	293.555556	20.55555555	0	0	0	0	34.0724638	0	0.49277013
0.0477	1960.06649	0.40688963	39.3886993	0.62388597	0	-3.0428239	0.67348845	-0.8967587	3.67350434	-0.4701861	-6.9077553	3	92	2.68223333	293.555556	20.55555555	0	0	0	0	34.0724638	0	0.49277013
0.0675	2661.88697	0.50464353	62.5923795	0.83859921	0	-2.6956277	0.97941086	-0.6819234	4.13665951	-0.1748306	-6.9077553	4	91	2.68223333	294.111111	21.11111111	0	0	0	0	34.0724638	0	0.49277013
0.1017	4067.20912	0.6574971	113.142899	1.18225489	0	-2.285728	1.40320288	-0.4177952	4.72866045	0.16826902	-6.9077553	5	91	2.68223333	294.666667	21.66666666	0	0	0	0	34.0724638	0	0.49277013
0.1701	6969.62215	0.77991506	165.779772	1.42159914	0	-1.7713688	1.94170448	-0.2472889	5.11066627	0.35248558	-6.9077553	6	85	3.57631111	296.333333	23.33333333	0	0	0	0	34.0724638	0	0.49277013
0.2295	9205.24002	0.83001726	179.906886	1.70207399	0	-1.4718522	2.21988152	-0.1851047	5.19244498	0.53243485	-6.9077553	7	71	5.36446667	298.555556	25.55555555	0	0	0	0	34.0724638	0	0.49277013
0.4023	17528.1404	1.08575583	340.190735	3.05114873	0	-0.9105572	2.86386466	0.08319695	5.82950938	1.11584585	-6.9077553	8	67	5.81150556	299.666667	26.66666666	0	0	0	1	34.0724638	0	0.49277013
0.8226	38336.1653	1.37164124	549.8392	4.79844858	0	-0.1952852	3.6464198	0.31673679	6.30962769	1.56850103	-6.9077553	9	55	6.25854444	301.888889	28.88888888	0	0	0	1	34.0724638	0	0.49277013
1.4715	57496.0701	1.29104923	495.404646	4.07106693	2	0.38628229	4.05173399	0.25622951	6.20537691	1.40415072	0.69364706	10	50	5.81150556	303.555556	30.55555555	0	0	0	1	34.0724638	0	0.49277013
2.0547	74312.9579	1.11992805	371.014371	3.12257348	2	0.72012985	4.30829879	0.11415696	5.91624349	1.13897769	0.69364706	11	45	5.36446667	304.111111	31.11111111	0	0	0	1	34.0724638	0	0.49277013
2.601	92529.3464	1.0010563	282.730591	2.29879394	2	0.95589599	4.52753666	0.00205419	5.64449801	0.83281953	0.69364706	12	46	3.57631111	304.111111	31.11111111	0	0	0	1	34.0724638	0	0.49277013
3.2022	114224.079	1.0243702	294.806372	2.47973048	2	1.16383807	4.73817088	0.02505372	5.68632217	0.90855307	0.69364706	13	44	4.02335	304.666667	31.66666666	0	0	0	1	34.0724638	0	0.49277013
3.7161	129268.332	0.87599356	212.879173	1.94548566	2	1.31267473	4.86189808	-0.1312556	5.36072944	0.66602552	0.69364706	14	49	3.12927222	303	30	0	0	0	1	34.0724638	0	0.49277013
4.1436	142447.381	0.78195441	182.749572	1.70884637	2	1.42156498	4.95897969	-0.2446808	5.20812223	0.53640353	0.69364706	15	53	3.12927222	301.333333	28.33333333	0	0	0	1	34.0724638	0	0.49277013
4.401	150503.751	0.71541281	154.947798	1.23770504	2	1.48183179	5.01399465	-0.3334987	5.04309473	0.21406651	0.69364706	16	58	1.78815556	299.111111	26.11111111	0	0	0	1	34.0724638	0	0.49277013
4.6728	159609.837	0.714358	152.365532	1.19209118	2	1.54175846	5.07273858	-0.3349722	5.02628901	0.17654757	0.69364706	17	51	2.68223333	298.555556	25.55555555	0	0	0	1	34.0724638	0	0.49277013
5.0103	166817.079	0.64997009	131.057646	1.17529997	2	1.61149579	5.11690387	-0.4292916	4.8756449	0.16237389	0.69364706	18	53	2.23519444	297.444444	24.44444444	0	0	0	1	34.0724638	0	0.49277013
5.2209	173047.103	0.74394487	161.520299	1.23720974	2	1.6526698	5.15356961	-0.2944451	5.08463702	0.21366658	0.69364706	19	62	2.68223333	296.333333	23.33333333	0	0	0	1	34.0724638	0	0.49277013
5.3991	180420.121	0.79081456	171.842645	1.44697463	2	1.68623227	5.19529368	-0.2334281	5.14658502	0.37016577	0.69364706	20	73	3.12927222	295.777778	22.7777778	0	0	0	1	34.0724638	0	0.49277013
5.5377	186095.794	0.64053335	107.455091	1.06856359	2	1.71157925	5.22626694	-0.4438941	4.67708231	0.0672507	0.69364706	21	80	2.23519444	295.222222	22.2222222	0	0	0	1	34.0724638	0	0.49277013
5.5962	188376.71	0.30203948	29.5681434	0.51690785	2	1.7220878	5.23844904	-1.1938922	3.38673137	-0.6579579	0.69364706	22	84	3.12927222	295.222222	22.2222222	0	0	0	1	34.0724638	0	0.49277013
5.6376	189999.677	0.31012465	42.1955195	0.58222066	2	1.72945844	5.24702763	-1.1675617	3.74233774	-0.5391897	0.69364706	23	88	2.23519444	294.666667	21.66666666	0	0	0	1	34.0724638	0	0.49277013
0.0306	1886.90322	1.09929218	330.859819	2.28536456	0	-3.4867553	0.63546681	0.09557576	5.8016978	0.82696303	-6.9077553	0	97	3.12927222	293	20	0	0	0	0	42.288408	0	-0.4111751
0.0675	3686.18129	0.61579974	97.4494153	1.12475456	0	-2.6956277	1.30486229	-0.4832109	4.57934369	0.11845353	-6.9077553	1	97	2.68223333	293	20	0	0	0	0	42.288408	0	-0.4111751
0.117	5967.09833	0.55145597	76.7466619	1.06949095	0	-2.1455813	1.78642834	-0.5933815	4.34052292	0.06811738	-6.9077553	2	93	4.47038889	293.555556	20.55555555	0	0	0	0	42.288408	0	-0.4111751
0.1674	8423.47001	0.55566778	76.4846104	1.00420148	0	-1.7873691	2.13114057	-0.5857867	4.33710262	0.005188	-6.9077553	3	92	2.68223333	293.555556	20.55555555	0	0	0	0	42.288408	0	-0.4111751
0.234	11608.6463	0.67422367	124.834011	1.2964658	0	-1.4524342	2.45183633	-0.3927113	4.82699295	0.26041297	-6.9077553	4	91	2.68223333	294.111111	21.11111111	0	0	0	0	42.288408	0	-0.4111751
0.4266	18731.8744	0.79636305	190.902088	1.98315202	0	-0.8519085	2.93027997	-0.2264452	5.25176591	0.68519163	-6.9077553	5	91	2.68223333	294.666667	21.66666666	0	0	0	0	42.288408	0	-0.4111751
0.6543	28111.3953	0.84301758	198.665792	1.83868951	0	-0.4241893	3.33621059	-0.169582	5.29162901	0.60959681	-6.9077553	6	85	3.57631111	296.333333	23.33333333	0	0	1	0	42.288408	0	-0.4111751
0.9891	39763.8193	0.85362982	196.827537	2.11261757	0	-0.0109598	3.68298258	-0.1570869	5.28233298	0.74840097	-6.9077553	7	71	5.36446667	298.555556	25.55555555	0	0	1	0	42.288408	0	-0.4111751
1.3779	55868.115	0.8906315	212.224537	2.01883556	0	0.3205606	4.02301172	-0.1147023	5.35764956	0.7030161	-6.9077553	8	67	5.81150556	299.666667	26.66666666	0	0	1	0	42.288408	0	-0.4111751
2.0241	78165.3809	0.92305711	245.374108	2.99027314	0	0.70512516	4.35883964	-0.0789814	5.50278809	1.0956991	-6.9077553	9	55	6.25854444	301.888889	28.88888888	0	0	1	0	42.288408	0	-0.4111751
2.6703	105774.162	0.94271366	251.057408	2.16787014	0	0.98219083	4.66131573	-0.0579325	5.52568561	0.77420636	-6.9077553	10	50	5.81150556	303.555556	30.55555555	0	0	1	0	42.288408	0	-0.4111751
3.4029	141943.261	1.02905281	306.449881	2.37586349	0	1.22462801	4.95543445	0.02961007	5.72505748	0.86578176	-6.9077553	11	45	5.36446667	304.111111	31.11111111	0	0	1	0	42.288408	0	-0.4111751
4.2372	185739.11	1.06715816	323.834614	2.51000081	0	1.44390267	5.22434844	0.06593582	5.78023602	0.9206814	-6.9077553	12	46	3.57631111	304.111111	31.11111111	0	0	1	0	42.288408	0	-0.4111751
4.8942	212965.59	0.95348084	264.073801	2.23410126	0	1.58805083	5.3611353	-0.0465877	5.5762324	0.80428653	-6.9077553	13	44	4.02335	304.666667	31.66666666	0	0	1	0	42.288408	0	-0.4111751
5.3433	231007.227	0.84089168	195.541411	1.49144064	0	1.67584344	5.44245333	-0.1721039	5.27577729	0.4004128	-6.9077553	14	49	3.12927222	303	30	0	0	1	0	42.288408	0	-0.4111751
5.7708	248545.511	0.81479075	195.99477	1.49886873	0	1.75281072	5.51562999	-0.2035974	5.27809308	0.40537759	-6.9077553	15	53	3.12927222	301.333333	28.33333333	0	0	1	0	42.288408	0	-0.4111751
6.0876	261423.01	0.81487612	189.345925	1.33172226	0	1.80625392	5.56614365	-0.2034927	5.24358091	0.28722366	-6.9077553	16	58	1.78815556	299.111111	26.11111111	0	0	1	0	42.288408	0	-0.4111751
6.3594	271447.732	0.74259191	162.029232	1.20649972	0	1.84993403	5.60377329	-0.2962629	5.08778294	0.18855187	-6.9077553	17	51	2.68223333	298.555556	25.55555555	0	0	1	0	42.288408	0	-0.4111751
6.642	279066	0.59695551	116.857832	0.9610793	0	1.89341312	5.63145189	-0.5142389	4.76096665	-0.0386584	-6.9077553	18	53	2.23519444	297.444444	24.44444444	0	0	1	0	42.288408	0	-0.4111751
6.8103	284882.6	0.75362678	162.852826	1.17483782	0	1.91843617	5.65208068	-0.281532	5.09285303	0.16198093	-6.9077553	19	62	2.68223333	296.333333	23.33333333	0	0	1	0	42.288408	0	-0.4111751
6.8787	287427.134	0.73273413	143.149183	1.13363878	0	1.92842968	5.66097286	-0.3096085	4.96389431	0.12631435	-6.9077553	20	73	3.12927222	295.777778	22.7777778	0	0	1	0	42.288408	0	-0.4111751
6.912	288655.325	0.54475207	73.3886191	0.81489218	0	1.93325903	5.66523679	-0.6055905	4.2957825	-0.2034731	-6.9077553	21	80	2.23519444	295.222222	22.2222222	0	0	1	0	42.288408	0	-0.4111751
6.9273	289313.294	0.25505627	15.1496708	0.44724066	0	1.93547013	5.66751362	-1.3623581	2.71804481	-0.802425	-6.9077553	22	84	3.12927222	295.222222	22.2222222	0	0	1	0	42.288408	0	-0.4111751
6.9399	289839.639	0.22390912	10.6349466	0.31928863	0	1.93728737	5.66933125	-1.4920589	2.36423945	-1.1385327	-6.9077553	23	88	2.23519444	294.666667	21.66666666	0	0	1	0	42.288408	0	-0.4111751
0.0009	0	0	0	0	0	-7.0131158	-6.9077553	-6.9077553	-6.9077553	-6.9077553	-6.9077553	0	97	3.12927222	293	20	0	0	0	0	18.9135952	0	-0.0428739
0.0009	0	0	0	0	0	-7.0131158	-6.9077553	-6.9077553	-6.9077553	-6.9077553	-6.9077553	1	97	2.68223333	293	20	0	0	0	0	18.9135952	0	-0.0428739
0.0009	0	0	0	0	0	-7.0131158	-6.9077553	-6.9077553	-6.9077553	-6.9077553	-6.9077553	2	93	4.47038889	293.555556	20.55555555	0	0	0	0	18.9135952	0	-0.0428739
0.0009	0	0	0	0	0	-7.0131158	-6.9077553	-6.9077553	-6.9077553	-6.9077553	-6.9077553	3	92	2.68223333	293.555556	20.55555555	0	0	0	0	18.9135952	0	-0.0428739
0.0009	0	0	0	0	0	-7.0131158	-6.9077553	-6.9077553	-6.9077553	-6.9077553	-6.9077553	4	91	2.68223333	294.111111	21.11111111	0	0	0	0	18.9135952	0	-0.0428739
0.0009	0	0	0	0	0	-7.0131158	-6.9077553	-6.9077553	-6.9077553	-6.9077553	-6.9077553	5	91	2.68223333	294.666667	21.66666666	0	0	0	0	18.9135952	0	-0.0428739

0.0009	0	0	0	0	0	-7.0131158	-6.9077553	-6.9077553	-6.9077553	-6.9077553	-6.9077553	6	85	3.57631111	296.333333	23.33333333	0	0	0	0	18.9135952	0	-0.0428739
0.036	0	0.65629729	122.381598	3.22588661	0	-3.3242363	-6.9077553	-0.4196189	4.80715219	1.17151778	-6.9077553	7	71	5.36446667	298.555556	25.55555555	0	0	0	0	18.9135952	0	-0.0428739
0.2043	3879.14953	0.85401925	244.313252	2.96809852	0	-1.5881658	1.35587369	-0.1566313	5.49845531	1.08825838	-6.9077553	8	67	5.81150556	299.666667	26.66666666	0	0	0	0	18.9135952	0	-0.0428739
0.459	12286.4073	0.88543858	261.956824	2.78791448	0	-0.7787051	2.50857494	-0.1205434	5.56818351	1.02565244	-6.9077553	9	55	6.25854444	301.888889	28.88888888	0	0	1	0	18.9135952	0	-0.0428739
0.6588	16705.5808	0.77384255	206.443623	2.13192722	0	-0.4173353	2.8158027	-0.2550954	5.33003221	0.75749532	-6.9077553	10	50	5.81150556	303.555556	30.55555555	0	0	1	0	18.9135952	0	-0.0428739
0.9063	25251.7599	0.83904619	221.34875	1.97213468	0	-0.0983849	3.22893545	-0.1742984	5.39974403	0.67962348	-6.9077553	11	45	5.36446667	304.111111	31.11111111	0	0	1	0	18.9135952	0	-0.0428739
1.1871	34160.0571	0.81088492	196.934395	1.95794032	0	0.17151336	3.53108631	-0.2083967	5.28287573	0.67240367	-6.9077553	12	46	3.57631111	304.111111	31.11111111	0	0	1	0	18.9135952	0	-0.0428739
1.512	47500.1419	0.91379824	249.808042	2.03027073	0	0.41343328	3.86075375	-0.0890517	5.52069679	0.70866157	-6.9077553	13	44	4.02335	304.666667	31.66666666	0	0	1	0	18.9135952	0	-0.0428739
1.7586	57412.7529	0.82718725	196.92973	1.59149214	0	0.56451804	4.05028387	-0.188516	5.28285204	0.46530018	-6.9077553	14	49	3.12927222	303	30	0	0	1	0	18.9135952	0	-0.0428739
2.0574	69298.7618	0.84096159	213.311653	1.71921622	0	0.72144305	4.23844147	-0.1720209	5.36275894	0.54244999	-6.9077553	15	53	3.12927222	301.333333	28.33333333	0	0	1	0	18.9135952	0	-0.0428739
2.3589	83148.5985	0.8793202	227.677659	1.66178616	0	0.85819541	4.42064138	-0.1274696	5.42793524	0.50849461	-6.9077553	16	58	1.78815556	299.111111	26.11111111	0	0	1	0	18.9135952	0	-0.0428739
2.61	93894.341	0.82159555	192.47421	1.49225335	0	0.95935022	4.54218077	-0.1952906	5.25996737	0.40095719	-6.9077553	17	51	2.68223333	298.555556	25.55555555	0	0	1	0	18.9135952	0	-0.0428739
2.9484	105948.926	0.76132624	181.616273	1.5951017	0	1.08126265	4.66296659	-0.2713807	5.20190158	0.46756422	-6.9077553	18	53	2.23519444	297.444444	24.44444444	0	0	1	0	18.9135952	0	-0.0428739
3.1707	115886.884	0.84928647	205.474016	1.5350107	0	1.15395238	4.75262321	-0.162182	5.32532445	0.4291886	-6.9077553	19	62	2.68223333	296.333333	23.33333333	0	0	1	0	18.9135952	0	-0.0428739
3.3732	125967.953	0.90625468	226.451406	1.72822374	0	1.21586185	4.83603547	-0.0973321	5.4225348	0.5476726	-6.9077553	20	73	3.12927222	295.777778	22.7777778	0	0	1	0	18.9135952	0	-0.0428739
3.5118	132101.225	0.70265485	133.373833	1.33311898	0	1.25612873	4.88357605	-0.3514673	4.89316346	0.28827114	-6.9077553	21	80	2.23519444	295.222222	22.2222222	0	0	1	0	18.9135952	0	-0.0428739
3.5505	133504.872	0.32317849	36.4165124	0.61030202	0	1.26708844	4.89414547	-1.126461	3.59504977	-0.4921641	-6.9077553	22	84	3.12927222	295.222222	22.2222222	0	0	1	0	18.9135952	0	-0.0428739
3.591	134908.51	0.31937764	33.8099555	0.50149502	0	1.27843072	4.90460426	-1.1382548	3.52078488	-0.6881695	-6.9077553	23	88	2.23519444	294.666667	21.66666666	0	0	1	0	18.9135952	0	-0.0428739
0.0009	0	0	0	0	0	-7.0131158	-6.9077553	-6.9077553	-6.9077553	-6.9077553	-6.9077553	0	82	2.68223333	296.333333	23.33333333	0	0	0	0	30.519848	0	0.21141735
0.0027	43.8637829	0.48428315	54.546566	0.82146746	0	-5.9145035	-3.1041244	-0.7230227	3.99907309	-0.1954464	-6.9077553	1	86	2.23519444	295.777778	22.7777778	0	0	0	0	30.519848	0	0.21141735
0.0063	219.318913	0.30334371	19.8744087	0.37363535	0	-5.0672056	-1.5126792	-1.1895976	2.98948322	-0.9818021	-6.9077553	2	86	2.68223333	296.333333	23.33333333	0	0	0	0	30.519848	0	0.21141735
0.0072	263.182695	0.23636454	11.2923412	0.26155218	0	-4.9336743	-1.3311144	-1.4381582	2.42421328	-1.3373054	-6.9077553	3	86	1.34111667	296.333333	23.33333333	0	0	0	0	30.519848	0	0.21141735
0.0081	307.046481	0.17773458	6.07618427	0.36811104	0	-4.8158912	-1.1775046	-1.7218533	1.80454148	-0.9966577	-6.9077553	4	86	2.23519444	296.333333	23.33333333	0	0	0	0	30.519848	0	0.21141735
0.0117	482.501588	0.23101315	10.9549475	0.27769071	0	-4.4481664	-0.7267007	-1.4609612	2.39388246	-1.2776527	-6.9077553	5	87	1.34111667	296.333333	23.33333333	0	0	0	0	30.519848	0	0.21141735
0.0153	614.092962	0.23485905	12.1503124	0.41829014	0	-4.1799025	-0.4859819	-1.4445209	2.49743718	-0.8691921	-6.9077553	6	88	0.89407778	295.777778	22.7777778	0	0	0	0	30.519848	0	0.21141735
0.0216	921.139558	0.28269621	16.8431516	0.43468574	0	-3.835062	-0.0810587	-1.2598513	2.82400351	-0.8308341	-6.9077553	7	88	2.23519444	295.777778	22.7777778	0	0	0	0	30.519848	0	0.21141735
0.027	1008.867	0.26642849	14.8763669	0.41651841	0	-3.6119184	0.00981864	-1.3189031	2.69984106	-0.8734266	-6.9077553	8	88	2.68223333	295.777778	22.7777778	0	0	0	0	30.519848	0	0.21141735
0.0396	1359.77726	0.35482921	38.9820731	0.7429281	0	-3.2289262	0.30805605	-1.0333044	3.66312753	-0.2958109	-6.9077553	9	86	2.68223333	296.333333	23.33333333	0	0	0	0	30.519848	0	0.21141735
0.0819	1798.4151	0.32789372	32.6012924	0.99978808	0	-2.5022563	0.58746167	-1.1120206	3.4843826	0.00078777	-6.9077553	10	82	3.57631111	296.888889	23.88888888	0	0	0	0	30.519848	0	0.21141735
0.144	2412.50806	0.43882434	57.0129961	1.2057734	0	-1.937942	0.88108131	-0.8213799	4.04329678	0.18795018	-6.9077553	11	75	3.57631111	298.555556	25.55555555	0	0	0	0	30.519848	0	0.21141735
0.2448	3137.84134	0.52920519	79.7798549	1.776968	0	-1.4073137	1.14385373	-0.6344912	4.37928356	0.57547114	-6.9077553	12	69	4.47038889	299.111111	26.11111111	0	0	0	0	30.519848	0	0.21141735
0.4338	5148.68949	0.72048107	149.094689	2.63858439	0	-0.8351717	1.63893642	-0.3264491	5.00458831	0.97062148	-6.9077553	13	64	4.91742778	300.777778	27.7777778	0	0	0	1	30.519848	0	0.21141735
0.7092	11016.9184	0.88601784	230.868234	3.3193495	0	-0.3436177	2.39952289	-0.1198902	5.44185146	1.20007005	-6.9077553	14	61	4.47038889	301.333333	28.33333333	0	0	0	1	30.519848	0	0.21141735
0.9837	17362.3463	0.77841076	166.339805	2.2824639	0	-0.0164343	2.85436145	-0.2492171	5.11403873	0.82569354	-6.9077553	15	59	4.47038889	301.333333	28.33333333	0	0	0	1	30.519848	0	0.21141735
1.2348	27988.9103	0.7469005	163.079791	1.65731024	0	0.21090901	3.3318441	-0.2904853	5.09424573	0.50579916	-6.9077553	16	54	6.25854444	301.888889	28.88888888	0	0	0	1	30.519848	0	0.21141735
1.4355	37064.2276	0.67278423	134.269724	1.39085901	0	0.36151322	3.61267927	-0.3948453	4.89985809	0.33064027	-6.9077553	17	54	4.02335	301.888889	28.88888888	0	0	0	1	30.519848	0	0.21141735
1.5615	43799.9679	0.64469812	120.224023	1.01842368	0	0.4456469	3.77965592	-0.4374232	4.78936518	0.01923745	-6.9077553	18	54	3.12927222	301.888889	28.88888888	0	0	0	1	30.519848	0	0.21141735
1.6569	50046.4847	0.64344399	117.172953	0.89431137	0	0.50494839	3.91297225	-0.4393674	4.76365961	-0.1105837	-6.9077553	19	55	3.12927222	300.777778	27.7777778	0	0	0	1	30.519848	0	0.21141735
1.7271	54424.0474	0.67485535	125.337164	0.8411699	0	0.5464437	3.99682448	-0.3917762	4.8310154	-0.1717735	-6.9077553	20	62	2.68223333	299.666667	26.66666666	0	0	0	1	30.519848	0	0.21141735
1.7856	61199.9197	0.74723018	147.933128	1.03090938	0	0.57975449	4.11416222	-0.2900446	4.99676709	0.03141085	-6.9077553	21	63	3.12927222	298.555556	25.55555555	0	0	0	1	30.519848	0	0.21141735
1.8486	67759.2105	0.75403192	151.034794	1.04773767	0	0.6144286	4.21597516	-0.2809953	5.01751685	0.04758722	-6.9077553	22	64	2.23519444	298.555556	25.55555555	0	0	0	1	30.519848	0	0.21141735
1.9125	75075.3979	0.70578997	130.245241	0.97054796	0	0.64841129	4.31850623	-0.3470217	4.86942682	-0.0288646	-6.9077553	23	67	2.68223333	298	25	0	0	0	1	30.519848	0	0.21141735
0.0009	0	0	0	0	0	-7.0131158	-6.9077553	-6.9077553	-6.9077553	-6.9077553	-6.9077553	0	82	2.68223333	296.333333	23.33333333	0	0	0	0	34.0724638	0	0.49277013
0.0036	131.59135	0.45980628	48.5574799	0.75124431	0	-5.6268214	-2.0204834	-0.7747775	3.88276884	-0.2846941	-6.9077553	1	86	2.23519444	295.777778	22.7777778	0	0	0	0	34.0724638	0	0.49277013
0.0108	438.637827	0.32322451	23.8873711	0.5065257	0	-4.5282091	-0.821804	-1.1263191	3.17339177	-0.6782079	-6.9077553	2	86	2.68223333	296.333333	23.33333333	0	0	0	0	34.0724638	0	0.49277013
0.0135	526.365391	0.25369138	14.1977844	0.47891931	0	-4.3050656	-0.6398616	-1.3677028	2.65315636	-0.7341373	-6.9077553	3	86	1.34111667	296.333333	23.33333333	0	0	0	0	34.0724638	0	0.49277013
0.0171	657.956723	0.23253699	10.9227934	0.26260075	0	-4.0686768	-0.4170974	-1.4544148	2.39094329	-1.3333196	-6.9077553	4	86	2.23519444	296.333333	23.33333333	0	0	0	0	34.0724638	0	0.49277013
0.0234	877.275669	0.21982981	9.67095075	0.2798171	0	-3.7550193	-0.1297948	-1.5103629	2.26923002	-1.2700517	-6.9077553	5	87	1.34111667	296.333333	23.33333333	0	0	0	0	34.0724638	0	0.49277013
0.0288	1052.73091	0.21495205	10.5826848	0.36878578	0	-3.5473799	0.05233711	-1.5326989	2.35931365	-0.9948314	-6.9077553	6	88	0.89407778	295.777778	22.7777778	0	0	0	0	34.0724638	0	0.49277013
0.036	1315.91355	0.21389194	9.80202866	0.27793312	0	-3.3242363	0.27529078	-1.53762	2.28269138	-1.2767832	-6.9077553	7	88	2.23519444	295.777778	22.7777778	0	0	0	0	34.0724638	0	0.49277013
0.0387	1403.64105	0.17927581	7.17475891	0.18636898	0	-3.2519157	0.33978179	-1.7132673	1.97070853	-1.6746754	-6.9077553	8	88	2.68223333	295.777778	22.7777778	0	0	0	0	34.0724638	0	0.49277013
0.045	1666.82372	0.27629311	18.0378113	0.36732381	0	-3.1010928	0.51151962	-1.2826802	2.89252562	-0.9987928	-6.9077553	9	86	2.68223333	296.333333	23.33333333	0	0	0	0	34.0724638	0	0.49277013
0.0819	3186.80916	0.73618117	147.55545	1.35717001	0	-2.5022563	1.1593339	-0.3049216	4.99421081	0.30613821	-6.9077553	10	82	3.57631111	296.888889	23.88888888	0	0	0	0	34.0724638	0	0.49277013
0.1467	5889.82954	0.79324108	173.571126	1.37522909	0	-1.9193656	1.77339683	-0.2303682	5.15659323	0.31934722	-6.9077553	11	75	3.57631111	298.555556	25.55555555	0	0	0	0	34.0724638	0	0.49277013
0.2277	9839.70593	0.95847151	260.128125	1.89957767	0	-1.4797263	2.28652745	-0.0413727	5.56117814	0.64215788	-6.9077553	12	69	4.47038889	299.111111	26.11111111	0	0	0	0	34.0724638	0	0.49277013
0.5418	18490.7646	1.26500739	489.334482	3.99752187	0	-0.6128583	2.91732548	0.23586816	6.19304831	1.38592476	-6.9077553	13	64	4.91742778	300.777778	27.7777778	0	0	0	0	34.0724638	0	0.49277013
1.1781	27113.6835	1.39640581	618.357408	4.75316002	0	0.16390297	3.30007541	0.33461753	6.42706824	1.55902003	-6.9077553	14	61	4.47038889	301.333333	28.33333333	0	0	0	1	34.0724638	0	0.49277013
1.8036	44083.2706	1.15462761	389.21214	3.15966797	0	0.58978467	3.78610304	0.14464358	5.96412711	1.15078339	-6.9077553	15	59	4.47038889	301.333333	28.33333333	0	0	0	1	34.0724638	0	0.49277013
2.4561	67470.6434	1.12369444	378.03931	3.26193292	1	0.89857473	4.21170741	0.11751139	5.93500083	1.18262646	0.0009995	16	54	6.25854444	301.888889	28.88888888	0	0	0	1	34.0724638	0	0.49277013
2.9457	83564.8813	0.89576665	218.388787	1.74905261	1	1.08034648	4.42563532	-0.1089596	5.38628148	0.55964585	0.0009995	17	54	4.02335	301.888889	28.88888888	0	0	0	1	34.0724638	0	0.49277013
3.5442	103134.416	0.90106798	224.517857	2.18753745	1	1.26531246	4.63604284	-0.1030654	5.4139597	0.78323349	0.0009995	18	54	3.12927222	301.888889	28.88888888	0	0	0	1	34.0724638	0	0.49277013
4.1103	119574.438	0.81000144	194.012818	2.01652898	1	1.41349602	4.78394745	-0.2094854	5.26792938	0.70187349	0.0009995	19	55	3.12927222	300.777778	27.7777778	0	0	0	1	34.0724638	0	0.49277013

4.3767	130945.522	0.84220082	198.827228	1.45751644	1	1.47629502	4.87478901	-0.1705501	5.29244127	0.37741978	0.0009995	20	62	2.68223333	299.666667	26.66666666	0	0	0	1	34.0724638	0	0.49277013
4.5891	140155.628	0.86573494	203.494008	1.59486337	1	1.52368393	4.94276057	-0.1430221	5.31564147	0.46741489	0.0009995	21	63	3.12927222	298.555556	25.55555555	0	0	0	1	34.0724638	0	0.49277013
4.8177	150628.11	0.88093182	208.982806	1.63483819	1	1.57229664	5.01482059	-0.1256405	5.34225677	0.49215533	0.0009995	22	64	2.23519444	298.555556	25.55555555	0	0	0	1	34.0724638	0	0.49277013
5.0859	162952.946	0.88194306	208.826526	1.62548122	1	1.626472	5.09346762	-0.1244946	5.34150868	0.48641892	0.0009995	23	67	2.68223333	298	25	0	0	0	1	34.0724638	0	0.49277013
0.0009	0	0	0	0	0	-7.0131158	-6.9077553	-6.9077553	-6.9077553	-6.9077553	-6.9077553	0	82	2.68223333	296.333333	23.33333333	0	0	0	0	42.288408	0	-0.4111751
0.0045	175.455131	0.4687959	51.0834618	0.80759859	0	-5.4036779	-1.7346886	-0.7554569	3.93348038	-0.2124527	-6.9077553	1	86	2.23519444	295.777778	22.7777778	0	0	0	0	42.288408	0	-0.4111751
0.0126	570.229192	0.40968172	37.8496501	0.73053439	0	-4.3740585	-0.5599648	-0.8899368	3.63364815	-0.312611	-6.9077553	2	86	2.68223333	296.333333	23.33333333	0	0	0	0	42.288408	0	-0.4111751
0.0306	1447.50486	0.31599672	22.0838898	0.52107	0	-3.4867553	0.3705319	-1.1488639	3.09489365	-0.6499536	-6.9077553	3	86	1.34111667	296.333333	23.33333333	0	0	0	0	42.288408	0	-0.4111751
0.0423	2017.73407	0.33501159	24.6232112	0.59399671	0	-3.1629682	0.70247062	-1.0906096	3.20373015	-0.5191994	-6.9077553	4	86	2.23519444	296.333333	23.33333333	0	0	0	0	42.288408	0	-0.4111751
0.0639	3070.46484	0.30505286	20.4765498	0.50367649	0	-2.7504359	1.1221546	-1.1839975	3.01932915	-0.6838377	-6.9077553	5	87	1.34111667	296.333333	23.33333333	0	0	0	0	42.288408	0	-0.4111751
0.0828	3991.60371	0.27081962	16.123929	0.4288682	0	-2.4913272	1.38444358	-1.3026166	2.78036646	-0.8442766	-6.9077553	6	88	0.89407778	295.777778	22.7777778	0	0	0	0	42.288408	0	-0.4111751
0.1017	4868.88004	0.25515722	14.0001032	0.37956369	0	-2.285728	1.58306931	-1.3619639	2.63913612	-0.9661017	-6.9077553	7	88	2.23519444	295.777778	22.7777778	0	0	0	0	42.288408	0	-0.4111751
0.1269	6097.0653	0.29622599	20.991939	0.46765024	0	-2.0643559	1.80797156	-1.2132625	3.04418614	-0.7578985	-6.9077553	8	88	2.68223333	295.777778	22.7777778	0	0	0	0	42.288408	0	-0.4111751
0.2133	10655.1235	0.87063535	217.636556	1.77213128	0	-1.5450557	2.3661347	-0.1373841	5.38283109	0.57274707	-6.9077553	9	86	2.68223333	296.333333	23.33333333	0	0	0	0	42.288408	0	-0.4111751
0.4446	20530.9567	0.90215008	239.751186	2.46408283	0	-0.8105803	3.02198254	-0.1018665	5.47960583	0.90222541	-6.9077553	10	82	3.57631111	296.888889	23.88888888	0	0	0	0	42.288408	0	-0.4111751
0.7128	31536.7147	0.86692001	211.697384	1.91234573	0	-0.3385544	3.45118412	-0.1416557	5.35516254	0.6488534	-6.9077553	11	75	3.57631111	298.555556	25.55555555	0	0	1	0	42.288408	0	-0.4111751
1.017	43140.1389	0.85845071	194.579373	1.84731532	0	0.01685712	3.76447704	-0.1514618	5.27084531	0.61427459	-6.9077553	12	69	4.47038889	299.111111	26.11111111	0	0	1	0	42.288408	0	-0.4111751
1.3797	56615.6699	0.82401626	178.993448	1.72090001	0	0.32186608	4.03630346	-0.1923522	5.18735479	0.54342834	-6.9077553	13	64	4.91742778	300.777778	27.7777778	0	0	1	0	42.288408	0	-0.4111751
1.8657	75426.6749	0.89736594	217.929948	2.11042119	0	0.62363632	4.32317425	-0.1071778	5.38417826	0.74736127	-6.9077553	14	61	4.47038889	301.333333	28.33333333	0	0	1	0	42.288408	0	-0.4111751
2.4606	94751.3073	0.84530552	198.191069	2.19351598	0	0.90040522	4.55126619	-0.1668749	5.28923661	0.78596151	-6.9077553	15	59	4.47038889	301.333333	28.33333333	0	0	1	0	42.288408	0	-0.4111751
3.2256	130825.956	1.0190118	306.291342	2.83189108	0	1.17111898	4.87387551	0.0198142	5.72454001	1.04129777	-6.9077553	16	54	6.25854444	301.888889	28.88888888	0	0	1	0	42.288408	0	-0.4111751
3.9924	168991.914	1.0141224	286.971721	2.30407199	0	1.38439255	5.12985678	0.0150092	5.65938716	0.83511191	-6.9077553	17	54	4.02335	301.888889	28.88888888	0	0	1	0	42.288408	0	-0.4111751
4.6728	201031.9	0.94437469	249.960545	1.9099226	0	1.54175846	5.30346858	-0.0561739	5.52130709	0.64758616	-6.9077553	18	54	3.12927222	301.888889	28.88888888	0	0	1	0	42.288408	0	-0.4111751
5.2083	224596.51	0.86766747	218.685675	1.65017869	0	1.65025351	5.41430995	-0.1407949	5.38764	0.50148939	-6.9077553	19	55	3.12927222	300.777778	27.7777778	0	0	1	0	42.288408	0	-0.4111751
5.5179	237911.239	0.84206337	196.640739	1.41516131	0	1.70799735	5.47190186	-0.1707131	5.28138349	0.34794991	-6.9077553	20	62	2.68223333	299.666667	26.66666666	0	0	1	0	42.288408	0	-0.4111751
5.7564	248111.196	0.81310252	181.713945	1.37724529	0	1.75031228	5.51388105	-0.205669	5.20243922	0.32081116	-6.9077553	21	63	3.12927222	298.555556	25.55555555	0	0	1	0	42.288408	0	-0.4111751
5.9814	257529.953	0.81411096	175.074313	1.34918042	0	1.78865465	5.55113992	-0.204431	5.16521624	0.30023823	-6.9077553	22	64	2.23519444	298.555556	25.55555555	0	0	1	0	42.288408	0	-0.4111751
6.1578	264769.875	0.80347116	170.841916	1.35577517	0	1.81771957	5.57886483	-0.2175702	5.14074452	0.30511069	-6.9077553	23	67	2.68223333	298	25	0	0	1	0	42.288408	0	-0.4111751
0.0009	0	0	0	0	0	-7.0131158	-6.9077553	-6.9077553	-6.9077553	-6.9077553	-6.9077553	0	82	2.68223333	296.333333	23.33333333	0	0	0	0	18.9135952	0	-0.0428739
0.0009	0	0	0	0	0	-7.0131158	-6.9077553	-6.9077553	-6.9077553	-6.9077553	-6.9077553	1	86	2.23519444	295.777778	22.7777778	0	0	0	0	18.9135952	0	-0.0428739
0.0009	0	0	0	0	0	-7.0131158	-6.9077553	-6.9077553	-6.9077553	-6.9077553	-6.9077553	2	86	2.68223333	296.333333	23.33333333	0	0	0	0	18.9135952	0	-0.0428739
0.0009	0	0	0	0	0	-7.0131158	-6.9077553	-6.9077553	-6.9077553	-6.9077553	-6.9077553	3	86	1.34111667	296.333333	23.33333333	0	0	0	0	18.9135952	0	-0.0428739
0.0009	0	0	0	0	0	-7.0131158	-6.9077553	-6.9077553	-6.9077553	-6.9077553	-6.9077553	4	86	2.23519444	296.333333	23.33333333	0	0	0	0	18.9135952	0	-0.0428739
0.0009	0	0	0	0	0	-7.0131158	-6.9077553	-6.9077553	-6.9077553	-6.9077553	-6.9077553	5	87	1.34111667	296.333333	23.33333333	0	0	0	0	18.9135952	0	-0.0428739
0.0009	0	0	0	0	0	-7.0131158	-6.9077553	-6.9077553	-6.9077553	-6.9077553	-6.9077553	6	88	0.89407778	295.777778	22.7777778	0	0	0	0	18.9135952	0	-0.0428739
0.0009	0	0	0	0	0	-7.0131158	-6.9077553	-6.9077553	-6.9077553	-6.9077553	-6.9077553	7	88	2.23519444	295.777778	22.7777778	0	0	0	0	18.9135952	0	-0.0428739
0.0009	0	0	0	0	0	-7.0131158	-6.9077553	-6.9077553	-6.9077553	-6.9077553	-6.9077553	8	88	2.68223333	295.777778	22.7777778	0	0	0	0	18.9135952	0	-0.0428739
0.0009	0	0	0	0	0	-7.0131158	-6.9077553	-6.9077553	-6.9077553	-6.9077553	-6.9077553	9	86	2.68223333	296.333333	23.33333333	0	0	0	0	18.9135952	0	-0.0428739
0.0009	0	0	0	0	0	-7.0131158	-6.9077553	-6.9077553	-6.9077553	-6.9077553	-6.9077553	10	82	3.57631111	296.888889	23.88888888	0	0	0	0	18.9135952	0	-0.0428739
0.0171	0	0.50952042	70.1668498	1.96600829	0	-4.0686768	-6.9077553	-0.6723246	4.25089023	0.67651376	-6.9077553	11	75	3.57631111	298.555556	25.55555555	0	0	0	0	18.9135952	0	-0.0428739
0.0963	415.934759	0.68621826	133.869651	2.63137921	0	-2.340287	-0.8748255	-0.3751033	4.89687404	0.96788808	-6.9077553	12	69	4.47038889	299.111111	26.11111111	0	0	0	0	18.9135952	0	-0.0428739
0.2781	5077.14284	0.81090145	207.294825	2.22275959	0	-1.2797745	1.62494561	-0.2083763	5.33414688	0.79919927	-6.9077553	13	64	4.91742778	300.777778	27.7777778	0	0	1	0	18.9135952	0	-0.0428739
0.4968	13309.0117	0.86105033	240.883857	2.18530173	0	-0.6995677	2.58851651	-0.1484416	5.48431905	0.78221141	-6.9077553	14	61	4.47038889	301.333333	28.33333333	0	0	1	0	18.9135952	0	-0.0428739
0.7713	21435.8773	0.8540001	245.563268	2.36626997	0	-0.2596779	3.06511268	-0.1566537	5.5035587	0.86173738	-6.9077553	15	59	4.47038889	301.333333	28.33333333	0	0	1	0	18.9135952	0	-0.0428739
1.1682	34744.3514	0.96071384	286.154301	3.26624283	0	0.1554641	3.54804579	-0.0390383	5.65653467	1.18394646	-6.9077553	16	54	6.25854444	301.888889	28.88888888	0	0	1	0	18.9135952	0	-0.0428739
1.5021	48119.7415	0.85773314	219.043021	1.84949537	0	0.40686413	3.8737133	-0.1522971	5.38927272	0.61545337	-6.9077553	17	54	4.02335	301.888889	28.88888888	0	0	1	0	18.9135952	0	-0.0428739
1.8792	65072.5577	0.93036333	247.456146	2.03286066	0	0.63084615	4.17551829	-0.0711058	5.51123742	0.70993579	-6.9077553	18	54	3.12927222	301.888889	28.88888888	0	0	1	0	18.9135952	0	-0.0428739
2.2266	80578.4466	0.88850411	226.626194	1.69584885	0	0.80047576	4.38924361	-0.1170912	5.42330635	0.52877291	-6.9077553	19	55	3.12927222	300.777778	27.7777778	0	0	1	0	18.9135952	0	-0.0428739
2.4813	93467.9796	0.91506947	236.75519	1.66619269	0	0.90878262	4.53762961	-0.0876631	5.46703088	0.51114119	-6.9077553	20	62	2.68223333	299.666667	26.66666666	0	0	1	0	18.9135952	0	-0.0428739
2.7045	105091.867	0.91437124	231.637065	1.72709422	0	0.99491705	4.65484441	-0.0884256	5.44517609	0.54701919	-6.9077553	21	63	3.12927222	298.555556	25.55555555	0	0	1	0	18.9135952	0	-0.0428739
2.8917	113773.736	0.82671811	182.519851	1.42411657	0	1.06184456	4.73422049	-0.1890826	5.20686442	0.35425362	-6.9077553	22	64	2.23519444	298.555556	25.55555555	0	0	1	0	18.9135952	0	-0.0428739
3.0798	123078.775	0.8815469	213.226843	1.65570609	0	1.12486466	4.81283272	-0.1249434	5.36236128	0.50483135	-6.9077553	23	67	2.68223333	298	25	0	0	1	0	18.9135952	0	-0.0428739
0.0144	369.997058	0.77678922	170.47002	1.84596812	0	-4.2405271	-0.9915611	-0.2512997	5.13856531	0.61354544	-6.9077553	0	92	4.02335	298	25	0	0	0	0	30.519848	0	0.21141735
0.0477	1028.89476	0.48303785	67.5147276	1.23489689	0	-3.0428239	0.02945662	-0.7255922	4.21236057	0.21179693	-6.9077553	1	81	4.02335	299.666667	26.66666666	0	0	0	0	30.519848	0	0.21141735
0.1134	1869.24382	0.543982	90.0145882	1.39843175	0	-2.1768339	0.62606881	-0.6070025	4.49998286	0.33606626	-6.9077553	2	75	4.02335	300.777778	27.7777778	0	0	0	0	30.519848	0	0.21141735
0.1872	2760.57308	0.49808344	71.4086795	1.44411943	0	-1.6755777	1.01580047	-0.694982	4.26843343	0.36819197	-6.9077553	3	63	4.02335	303.555556	30.55555555	0	0	0	0	30.519848	0	0.21141735
0.2799	3312.00873	0.53781621	79.7932342	1.54906338	0	-1.2733229	1.19785676	-0.6183808	4.37945125	0.43829582	-6.9077553	4	58	3.12927222	304.666667	31.66666666	0	0	0	0	30.519848	0	0.21141735
0.4275	4535.08269	0.65417397	116.197849	2.0528531	0	-0.849801	1.5120638	-0.4228545	4.75530294	0.71971759	-6.9077553	5	51	3.12927222	305.777778	32.7777778	0	0	0	1	30.519848	0	0.21141735
0.5904	7748.83162	0.72782932	151.044307	1.7370243	0	-0.526955	2.04767112	-0.3163157	5.01757984	0.55274901	-6.9077553	6	52	2.68223333	305.777778	32.7777778	0	0	0	1	30.519848	0	0.21141735
0.8208	12846.9754	0.7671752	168.169327	2.02499819	0	-0.1974758	2.55318624	-0.2637374	5.12497732	0.70606251	-6.9077553	7	58	1.78815556	305.222222	32.2222222	0	0	0	1	30.519848	0	0.21141735
1.0494	16730.6106	0.66806943	129.435562	1.52457554	0	0.04821857	2.81729978	-0.4018674	4.86319089	0.42237174	-6.9077553	8	51	1.78815556	306.333333	33.33333333	0	0	0	1	30.519848	0	0.21141735
1.3563	21829.8239	0.75221525	159.688096	2.4492379	0	0.3047604	3.08332291	-0.2834042	5.07322877	0.89618512	-6.9077553	9	48	4.91742778	306.333333	33.33333333	0	0	0	1	30.519848	0	0.21141735

1.5579	27463.8472	0.72732326	155.188389	1.44715009	0	0.44333876	3.3129069	-0.3170103	5.04464624	0.37028694	-6.9077553	10	50	4.02335	305.777778	32.7777778	0	0	0	1	30.519848	0	0.21141735
1.7082	31647.6212	0.68680184	133.908402	1.2395406	0	0.53544018	3.45469458	-0.3742545	4.89716347	0.21554725	-6.9077553	11	59	5.81150556	301.333333	28.33333333	0	0	0	1	30.519848	0	0.21141735
1.7856	34613.3623	0.78814804	163.801815	1.13621503	0	0.57975449	3.54426869	-0.2368013	5.09866335	0.12858232	-6.9077553	12	70	2.23519444	299.666667	26.66666666	0	0	0	1	30.519848	0	0.21141735
1.845	37067.4656	0.74664827	146.858014	1.17780107	0	0.61247928	3.61276663	-0.2908226	4.98947304	0.16449788	-6.9077553	13	71	1.34111667	298.555556	25.55555555	0	0	0	1	30.519848	0	0.21141735
1.881	38647.2432	0.67428989	116.633606	1.01851597	0	0.63180355	3.65450132	-0.3926132	4.75904602	0.01932797	-6.9077553	14	77	1.34111667	297.444444	24.44444444	0	0	0	1	30.519848	0	0.21141735
1.8954	39217.4747	0.29640752	19.4184303	0.39921212	0	0.6394299	3.66914793	-1.212652	2.96627413	-0.9157606	-6.9077553	15	82	0.44703889	296.333333	23.33333333	0	0	0	1	30.519848	0	0.21141735
1.908	39743.8421	0.24933287	12.9796949	0.36234093	0	0.64605557	3.68248007	-1.3849638	2.56346325	-1.0124137	-6.9077553	16	88	0.44703889	295.777778	22.7777778	0	0	0	1	30.519848	0	0.21141735
1.9197	40226.3401	0.20789289	9.40466191	0.37239269	0	0.65216892	3.69454687	-1.5659336	2.24131184	-0.9851246	-6.9077553	17	93	0.44703889	295.777778	22.7777778	0	0	0	1	30.519848	0	0.21141735
1.9332	40752.7033	0.20954657	9.23308309	0.31902583	0	0.65917666	3.70754671	-1.5580484	2.22290132	-1.1393536	-6.9077553	18	95	0.89407778	295.777778	22.7777778	0	0	0	1	30.519848	0	0.21141735
1.9431	41147.4794	0.23295608	11.0581512	0.33073633	0	0.66428464	3.71718697	-1.4526219	2.40325825	-1.1034148	-6.9077553	19	95	1.78815556	296.333333	23.33333333	0	0	0	1	30.519848	0	0.21141735
1.9494	41410.663	0.22929588	11.1896994	0.38674034	0	0.66752163	3.72356256	-1.4683903	2.41508303	-0.9474194	-6.9077553	20	93	1.34111667	297.444444	24.44444444	0	0	0	1	30.519848	0	0.21141735
1.9593	41893.1662	0.21273065	9.66058766	0.33778373	0	0.67258727	3.73514659	-1.5430387	2.26815799	-1.0823933	-6.9077553	21	91	1.34111667	297.444444	24.44444444	0	0	0	1	30.519848	0	0.21141735
1.9647	42156.3446	0.24091546	11.8276863	0.35004739	0	0.67533956	3.74140892	-1.419167	2.47052762	-1.0468341	-6.9077553	22	93	0.89407778	297.444444	24.44444444	0	0	0	1	30.519848	0	0.21141735
1.971	42463.3925	0.22490675	10.4569953	0.33010095	0	0.67854103	3.7486659	-1.487633	2.34736678	-1.105332	-6.9077553	23	94	2.23519444	297.444444	24.44444444	0	0	0	1	30.519848	0	0.21141735
0.0216	874.048111	1.03914186	300.783713	2.42214817	0	-3.835062	-0.1334764	0.03935711	5.70639477	0.88506759	-6.9077553	0	92	4.02335	298	25	0	0	0	0	34.0724638	0	0.49277013
0.0504	1864.23638	0.70405912	133.140167	1.13432479	0	-2.9877641	0.62338779	-0.3494736	4.89140997	0.12691877	-6.9077553	1	81	4.02335	299.666667	26.66666666	0	0	0	0	34.0724638	0	0.49277013
0.1206	5061.09857	0.84920981	194.258526	1.45764767	0	-2.115276	1.62178113	-0.1622721	5.26919503	0.37750975	-6.9077553	2	75	4.02335	300.777778	27.7777778	0	0	0	0	34.0724638	0	0.49277013
0.1908	8383.50227	0.91625243	218.424003	1.42685611	0	-1.6565295	2.12638503	-0.0863726	5.38644272	0.3561741	-6.9077553	3	63	4.02335	303.555556	30.55555555	0	0	0	0	34.0724638	0	0.49277013
0.2934	13010.3957	0.98011017	269.904965	2.12471611	0	-1.2262184	2.56582557	-0.0190705	5.59807362	0.75410874	-6.9077553	4	58	3.12927222	304.666667	31.66666666	0	0	0	1	34.0724638	0	0.49277013
0.4734	21276.7526	1.06678787	322.539961	2.66670746	0	-0.7478146	3.05766205	0.0655891	5.77623014	0.98121947	-6.9077553	5	51	3.12927222	305.777778	32.7777778	0	0	0	1	34.0724638	0	0.49277013
0.7884	38437.619	1.22277222	421.234509	3.238253	0	-0.2377497	3.64906266	0.20193807	6.04319208	1.17534275	-6.9077553	6	52	2.68223333	305.777778	32.7777778	0	0	0	1	34.0724638	0	0.49277013
1.0647	48375.9884	0.9668937	262.035907	2.03118002	0	0.06269307	3.87902426	-0.032633	5.56848536	0.70910912	-6.9077553	7	58	1.78815556	305.222222	32.2222222	0	0	0	1	34.0724638	0	0.49277013
1.2798	54148.5262	0.82063172	181.398601	1.39242362	0	0.24670382	3.99174922	-0.196463	5.20070234	0.33176376	-6.9077553	8	51	1.78815556	306.333333	33.33333333	0	0	0	1	34.0724638	0	0.49277013
1.9746	63820.9711	1.3572802	584.972555	4.68228829	0	0.68036585	4.15609751	0.30621934	6.37156664	1.54400049	-6.9077553	9	48	4.91742778	306.333333	33.33333333	0	0	0	1	34.0724638	0	0.49277013
2.5605	80909.7446	1.00098875	281.634601	2.40564115	0	0.94020255	4.39334663	0.00198678	5.64061404	0.87823206	-6.9077553	10	50	4.02335	305.777778	32.7777778	0	0	0	1	34.0724638	0	0.49277013
3.1878	105372.624	0.98302697	275.687993	2.42722718	0	1.15933102	4.65751236	-0.016102	5.61927339	0.88716144	-6.9077553	11	59	5.81150556	301.333333	28.33333333	0	0	0	1	34.0724638	0	0.49277013
3.5046	118976.94	0.82784401	185.386919	1.37546297	0	1.25407639	4.7789381	-0.1877233	5.22245049	0.31951714	-6.9077553	12	70	2.23519444	299.666667	26.66666666	0	0	0	1	34.0724638	0	0.49277013
3.735	127684.334	0.77012688	155.83905	1.31220508	0	1.31774782	4.84956891	-0.2599024	5.04883016	0.27247077	-6.9077553	13	71	1.34111667	298.555556	25.55555555	0	0	0	1	34.0724638	0	0.49277013
3.9087	133418.493	0.71046171	134.560881	1.23248924	0	1.36320484	4.89349825	-0.3404337	4.90202417	0.20984693	-6.9077553	14	77	1.34111667	297.444444	24.44444444	0	0	0	1	34.0724638	0	0.49277013
3.978	135699.407	0.31402551	31.3713379	0.67264805	0	1.38077918	4.91044956	-1.1551017	3.44592655	-0.3950475	-6.9077553	15	82	0.44703889	296.333333	23.33333333	0	0	0	1	34.0724638	0	0.49277013
4.0527	138155.781	0.33301583	38.0707096	0.64483408	0	1.39938333	4.92838913	-1.0965669	3.63947148	-0.4372126	-6.9077553	16	88	0.44703889	295.777778	22.7777778	0	0	0	1	34.0724638	0	0.49277013
4.1004	139691.02	0.37467446	52.5336546	0.71703634	0	1.41108453	4.93944014	-0.9790323	3.96147304	-0.3312351	-6.9077553	17	93	0.44703889	295.777778	22.7777778	0	0	0	1	34.0724638	0	0.49277013
4.1535	141971.935	0.22799131	14.2661588	0.35553567	0	1.42395135	4.95563644	-1.4740712	2.65796031	-1.031321	-6.9077553	18	95	0.89407778	295.777778	22.7777778	0	0	0	1	34.0724638	0	0.49277013
4.1841	143243.979	0.17496542	6.58383089	0.36287555	0	1.43129163	4.9645563	-1.7374678	1.88476865	-1.0109434	-6.9077553	19	95	1.78815556	296.333333	23.33333333	0	0	0	1	34.0724638	0	0.49277013
4.2255	144998.538	0.18783837	7.41859768	0.32232907	0	1.4411376	4.97673055	-1.6668638	2.00412483	-1.1290847	-6.9077553	20	93	1.34111667	297.444444	24.44444444	0	0	0	1	34.0724638	0	0.49277013
4.2705	146840.803	0.25978582	22.9853833	0.40554359	0	1.45173092	4.98935584	-1.3440558	3.13490201	-0.9000641	-6.9077553	21	91	1.34111667	297.444444	24.44444444	0	0	0	1	34.0724638	0	0.49277013
4.3065	148244.451	0.21611745	13.1288025	0.37302959	0	1.46012551	4.99886935	-1.5273168	2.57488465	-0.9834204	-6.9077553	22	93	0.89407778	297.444444	24.44444444	0	0	0	1	34.0724638	0	0.49277013
4.3416	149823.553	0.19857352	8.14409774	0.28528546	0	1.46824294	5.00946497	-1.6115726	2.09741624	-1.2507659	-6.9077553	23	94	2.23519444	297.444444	24.44444444	0	0	0	1	34.0724638	0	0.49277013
0.0378	2471.90431	1.14304761	363.636028	2.50282697	0	-3.2754462	0.9053933	0.13457251	5.89615619	0.91782035	-6.9077553	0	92	4.02335	298	25	0	0	0	0	42.288408	0	-0.4111751
0.135	6478.33926	0.90340162	225.812753	2.79050615	0	-2.0024805	1.86861854	-0.1004817	5.41971056	1.02658129	-6.9077553	1	81	4.02335	299.666667	26.66666666	0	0	0	0	42.288408	0	-0.4111751
0.3672	16595.5318	1.02995348	294.400375	3.02964132	0	-1.0018486	2.80919374	0.03048408	5.68494406	1.10877425	-6.9077553	2	75	4.02335	300.777778	27.7777778	0	0	0	0	42.288408	0	-0.4111751
0.6426	29962.3441	0.99198535	264.849724	2.10274331	0	-0.4422328	3.39997477	-0.0070394	5.57916636	0.74371829	-6.9077553	3	63	4.02335	303.555556	30.55555555	0	0	0	0	42.288408	0	-0.4111751
0.9441	42177.2222	0.8926889	215.020336	1.83729266	0	-0.0575232	3.74190403	-0.1123976	5.37073726	0.60883724	-6.9077553	4	58	3.12927222	304.666667	31.66666666	0	0	0	0	42.288408	0	-0.4111751
1.2978	57309.2195	0.89684422	217.848064	1.71539291	0	0.26067052	4.04847896	-0.1077587	5.38380245	0.54022494	-6.9077553	5	51	3.12927222	305.777778	32.7777778	0	0	1	0	42.288408	0	-0.4111751
1.7631	74866.7998	0.90714351	229.83757	2.11782807	0	0.56707362	4.31572389	-0.0963529	5.43737719	0.75086314	-6.9077553	6	52	2.68223333	305.777778	32.7777778	0	0	1	0	42.288408	0	-0.4111751
2.2761	93827.2965	0.87078204	211.102878	1.89992676	0	0.82246345	4.54146648	-0.1372158	5.35235033	0.64234153	-6.9077553	7	58	1.78815556	305.222222	32.2222222	0	0	1	0	42.288408	0	-0.4111751
2.7828	112994.074	0.86725265	209.792712	1.77980294	0	1.02345762	4.72734422	-0.1412725	5.34612472	0.57706435	-6.9077553	8	51	1.78815556	306.333333	33.33333333	0	0	1	0	42.288408	0	-0.4111751
3.4155	141300.194	0.96540557	263.947457	2.20566972	0	1.2283239	4.95089374	-0.0341717	5.57575385	0.79148447	-6.9077553	9	48	4.91742778	306.333333	33.33333333	0	0	1	0	42.288408	0	-0.4111751
4.1229	178304.774	1.01170617	296.339396	2.19672671	0	1.4165568	5.18349991	0.01262612	5.69150878	0.78742351	-6.9077553	10	50	4.02335	305.777778	32.7777778	0	0	1	0	42.288408	0	-0.4111751
4.7286	208994.667	1.00756809	305.19219	2.36569218	0	1.55362918	5.34231352	0.00853159	5.72094499	0.86149328	-6.9077553	11	59	5.81150556	301.333333	28.33333333	0	0	1	0	42.288408	0	-0.4111751
5.0184	222310.314	0.83985588	189.354256	1.40313579	0	1.61311116	5.40407872	-0.173335	5.24362491	0.33942201	-6.9077553	12	70	2.23519444	299.666667	26.66666666	0	0	1	0	42.288408	0	-0.4111751
5.274	233066.125	0.77476475	162.720978	1.38058321	0	1.66278909	5.4513265	-0.253906	5.09204309	0.3232301	-6.9077553	13	71	1.34111667	298.555556	25.55555555	0	0	1	0	42.288408	0	-0.4111751
5.4504	239629.46	0.71192776	133.76423	1.20182232	0	1.695689	5.47909799	-0.3383752	4.89608625	0.18467073	-6.9077553	14	77	1.34111667	297.444444	24.44444444	0	0	1	0	42.288408	0	-0.4111751
5.4819	240857.631	0.36322032	34.7293841	0.63899302	0	1.70145176	5.48421017	-1.0099963	3.54761493	-0.446298	-6.9077553	15	82	0.44703889	296.333333	23.33333333	0	0	1	0	42.288408	0	-0.4111751
5.5161	242305.152	0.21523837	9.93086162	0.38082236	0	1.70767109	5.49020202	-1.5313739	2.29574794	-0.9627998	-6.9077553	16	88	0.44703889	295.777778	22.7777778	0	0	1	0	42.288408	0	-0.4111751
5.5386	243226.266	0.20373108	8.63183899	0.36465759	0	1.71174176	5.49399626	-1.586058	2.15557342	-1.0060579	-6.9077553	17	93	0.44703889	295.777778	22.7777778	0	0	1	0	42.288408	0	-0.4111751
5.5656	244410.6	0.20025433	8.38344879	0.30227197	0	1.7166048	5.49885369	-1.6031859	2.12637866	-1.1931253	-6.9077553	18	95	0.89407778	295.777778	22.7777778	0	0	1	0	42.288408	0	-0.4111751
5.5962	245682.653	0.20755974	8.97409237	0.29115652	0	1.7220878	5.50404474	-1.5675298	2.19445323	-1.2304656	-6.9077553	19	95	1.78815556	296.333333	23.33333333	0	0	1	0	42.288408	0	-0.4111751
5.6205	246735.383	0.21545375	9.85430908	0.31086537	0	1.72642063	5.50832049	-1.5303784	2.2880103	-1.1651837	-6.9077553	20	93	1.34111667	297.444444	24.44444444	0	0	1	0	42.288408	0	-0.4111751
5.6529	247919.701	0.2181988	10.1117079	0.3248761	0	1.73216869	5.51310894	-1.5177762	2.31379284	-1.121238	-6.9077553	21	91	1.34111667	297.444444	24.44444444	0	0	1	0	42.288408	0	-0.4111751
5.6835	249367.201	0.21683464	10.2181423	0.32630693	0	1.73756724	5.51893052	-1.524019	2.32426266	-1.1168569	-6.9077553	22	93	0.89407778	297.444444	24.44444444	0	0	1	0	42.288408	0	-0.4111751
5.7114	250507.662	0.21926621	10.4310687	0.31260604	0	1.74246418	5.5234935	-1.5129184	2.34488459	-1.1596177	-6.9077553	23	94	2.23519444	297.444444	24.44444444	0	0	1	0	42.288408	0	-0.4111751

0.0099	0	0.59813786	97.2446716	2.6484211	0	-4.6152205	-6.9077553	-0.5122636	4.57724047	0.97434116	-6.9077553	0	92	4.02335	298	25	0	0	0	0	18.9135952	0	-0.0428739
0.0396	89.1066444	0.4548639	65.3154667	1.6722797	0	-3.2289262	-2.4067614	-0.785561	4.17924417	0.5147856	-6.9077553	1	81	4.02335	299.666667	26.66666666	0	0	0	0	18.9135952	0	-0.0428739
0.1323	704.244776	0.57490432	91.8413039	1.86706365	0	-2.0226832	-0.3492103	-0.5518137	4.52007302	0.62490241	-6.9077553	2	75	4.02335	300.777778	27.7777778	0	0	0	0	18.9135952	0	-0.0428739
0.3438	6973.56108	0.84626419	233.108344	2.55942071	0	-1.0676952	1.9422694	-0.1657427	5.45150763	0.94017159	-6.9077553	3	63	4.02335	303.555556	30.55555555	0	0	1	0	18.9135952	0	-0.0428739
0.5526	14151.8021	0.8015436	221.961965	2.01057224	0	-0.5931209	2.64991264	-0.2199691	5.40251054	0.69891662	-6.9077553	4	58	3.12927222	304.666667	31.66666666	0	0	1	0	18.9135952	0	-0.0428739
0.7119	18219.7511	0.7297715	167.492033	1.46082819	0	-0.3398178	2.90256111	-0.3136545	5.12094176	0.37968784	-6.9077553	5	51	3.12927222	305.777778	32.7777778	0	0	1	0	18.9135952	0	-0.0428739
0.9369	25424.8039	0.81279626	206.179344	1.78811694	0	-0.0651787	3.23576456	-0.2060452	5.32875124	0.58172217	-6.9077553	6	52	2.68223333	305.777778	32.7777778	0	0	1	0	18.9135952	0	-0.0428739
1.1898	33694.6401	0.82868349	204.498179	1.86507625	0	0.17378523	3.51736846	-0.186711	5.32056396	0.62383796	-6.9077553	7	58	1.78815556	305.222222	32.2222222	0	0	1	0	18.9135952	0	-0.0428739
1.4697	42353.0216	0.79322849	187.200274	1.6875263	0	0.3850583	3.74606338	-0.2303841	5.23218437	0.52385614	-6.9077553	8	51	1.78815556	306.333333	33.33333333	0	0	1	0	18.9135952	0	-0.0428739
1.8207	57446.3601	0.9393032	275.116827	2.30747321	0	0.59922104	4.05086905	-0.0615529	5.61719947	0.83658636	-6.9077553	9	48	4.91742778	306.333333	33.33333333	0	0	1	0	18.9135952	0	-0.0428739
2.1978	72385.5617	0.87476475	225.026328	1.95238355	0	0.78745686	4.28202067	-0.1326578	5.41622185	0.66956302	-6.9077553	10	50	4.02335	305.777778	32.7777778	0	0	1	0	18.9135952	0	-0.0428739
2.5362	89220.3895	0.96824143	272.417429	2.04312637	0	0.9306669	4.4911208	-0.0312415	5.60733922	0.7149705	-6.9077553	11	59	5.81150556	301.333333	28.33333333	0	0	1	0	18.9135952	0	-0.0428739
2.7126	97885.1648	0.87031649	205.434869	1.50586342	0	0.99790758	4.58380522	-0.13775	5.32513391	0.41003029	-6.9077553	12	70	2.23519444	299.666667	26.66666666	0	0	1	0	18.9135952	0	-0.0428739
2.8728	105363.207	0.82142948	180.459028	1.47452708	0	1.05528716	4.65742298	-0.1954925	5.1955093	0.38901527	-6.9077553	13	71	1.34111667	298.555556	25.55555555	0	0	1	0	18.9135952	0	-0.0428739
3.0249	112283.113	0.75894942	153.863651	1.34954545	0	1.10687803	4.72103238	-0.2745034	5.03607333	0.30050855	-6.9077553	14	77	1.34111667	297.444444	24.44444444	0	0	1	0	18.9135952	0	-0.0428739
3.0726	114388.572	0.27335473	16.717626	0.49777549	0	1.12252411	4.73960992	-1.2933334	2.81652343	-0.6955992	-6.9077553	15	82	0.44703889	296.333333	23.33333333	0	0	1	0	18.9135952	0	-0.0428739
3.1167	116362.431	0.25942269	14.7469981	0.49995913	0	1.13677475	4.75671832	-1.3454492	2.69110735	-0.6912308	-6.9077553	16	88	0.44703889	295.777778	22.7777778	0	0	1	0	18.9135952	0	-0.0428739
3.1617	118336.32	0.24516878	13.6552588	0.47739243	0	1.15110986	4.77353919	-1.4017379	2.61419794	-0.7373239	-6.9077553	17	93	0.44703889	295.777778	22.7777778	0	0	1	0	18.9135952	0	-0.0428739
3.2085	120441.779	0.24540371	13.253783	0.43341222	0	1.16580354	4.79117477	-1.400784	2.58435847	-0.8337614	-6.9077553	18	95	0.89407778	295.777778	22.7777778	0	0	1	0	18.9135952	0	-0.0428739
3.2607	122722.694	0.23454248	12.0696106	0.41303582	0	1.1819419	4.80993544	-1.445864	2.49077362	-0.8818028	-6.9077553	19	95	1.78815556	296.333333	23.33333333	0	0	1	0	18.9135952	0	-0.0428739
3.3066	124740.427	0.22270543	10.9814681	0.36851311	0	1.19592047	4.82624301	-1.4974251	2.39630019	-0.9955691	-6.9077553	20	93	1.34111667	297.444444	24.44444444	0	0	1	0	18.9135952	0	-0.0428739
3.3453	126538.84	0.24412812	12.6699346	0.38813968	0	1.20755638	4.8405572	-1.4059743	2.53931076	-0.9438169	-6.9077553	21	91	1.34111667	297.444444	24.44444444	0	0	1	0	18.9135952	0	-0.0428739
3.3831	128117.93	0.22088089	10.6312786	0.35459151	0	1.21879245	4.85295897	-1.5056146	2.36389453	-1.0339727	-6.9077553	22	93	0.89407778	297.444444	24.44444444	0	0	1	0	18.9135952	0	-0.0428739
3.4173	129609.306	0.21831465	10.5613098	0.35287388	0	1.22885077	4.8645323	-1.5172478	2.35729199	-1.0388147	-6.9077553	23	94	2.23519444	297.444444	24.44444444	0	0	1	0	18.9135952	0	-0.0428739
0.0153	413.393575	0.84109455	194.070328	1.6302247	0	-4.1799025	-0.8809391	-0.171863	5.26822576	0.48933109	-6.9077553	0	98	2.68223333	289.666667	16.66666666	0	0	0	0	30.519848	0	0.21141735
0.036	1159.89803	0.61313973	112.706214	1.06061927	0	-3.3242363	0.14919387	-0.4875328	4.72479343	0.05979536	-6.9077553	1	100	1.78815556	289.666667	16.66666666	0	0	0	0	30.519848	0	0.21141735
0.0891	1818.16046	0.54049049	86.898512	1.18290335	0	-2.4179959	0.59837511	-0.6134298	4.46475242	0.16881691	-6.9077553	2	93	3.57631111	292.444444	19.44444444	0	0	0	0	30.519848	0	0.21141735
0.1845	2592.19541	0.45611281	59.2856215	1.6565035	0	-1.6901058	0.95289086	-0.7828251	4.08238367	0.50531255	-6.9077553	3	76	4.47038889	294.111111	21.11111111	0	0	0	0	30.519848	0	0.21141735
0.3555	3759.98132	0.71904329	151.531086	3.45424773	0	-1.03423	1.32467991	-0.3284439	5.02079739	1.23989416	-6.9077553	4	63	5.36446667	294.111111	21.11111111	0	0	0	0	30.519848	0	0.21141735
0.5436	4795.10111	0.63416447	106.127448	2.36131236	0	-0.6095416	1.56780332	-0.4538713	4.66465014	0.85964095	-6.9077553	5	55	5.36446667	295.222222	22.2222222	0	0	0	1	30.519848	0	0.21141735
0.8883	8243.1703	0.72160232	143.962141	2.9873952	0	-0.1184458	2.10950632	-0.3248963	4.9695573	1.09473652	-6.9077553	6	42	5.81150556	296.333333	23.33333333	0	0	0	1	30.519848	0	0.21141735
1.5345	20773.6585	1.05635288	363.838854	5.31933186	0	0.4282046	3.0337339	0.0557685	5.89671381	1.67153568	-6.9077553	7	30	7.59966111	295.777778	22.7777778	0	0	0	1	30.519848	0	0.21141735
2.2338	37174.2673	0.81960309	197.267535	2.5546853	0	0.80370417	3.61564368	-0.1977157	5.28456592	0.93832041	-6.9077553	8	28	7.59966111	296.333333	23.33333333	0	0	0	1	30.519848	0	0.21141735
2.9421	56072.9241	0.79307345	186.077787	2.23776024	0	1.07912361	4.02667089	-0.2305793	5.22617017	0.80592225	-6.9077553	9	34	8.49373889	294.111111	21.11111111	0	0	0	1	30.519848	0	0.21141735
3.8268	79367.2552	0.80200108	201.388606	2.22621082	0	1.34202894	4.37409848	-0.2193992	5.30524137	0.80075005	-6.9077553	10	40	8.0467	290.777778	17.7777778	0	0	0	1	30.519848	0	0.21141735
4.7052	108695.907	0.75704206	171.147637	1.72076541	0	1.54866828	4.68856334	-0.2770164	5.1425324	0.54335017	-6.9077553	11	47	6.70558333	288	15	0	0	0	1	30.519848	0	0.21141735
5.607	135065.795	0.69949146	138.698026	1.36014787	0	1.72401582	4.90576944	-0.3559731	4.9323063	0.30832836	-6.9077553	12	50	7.15262222	286.888889	13.88888888	0	0	0	1	30.519848	0	0.21141735
6.3324	156307.274	0.677807	136.51232	1.22231798	0	1.84567931	5.05183017	-0.3874184	4.91642219	0.20156682	-6.9077553	13	53	7.59966111	285.222222	12.22222222	0	0	0	1	30.519848	0	0.21141735
6.8166	176793.231	0.68308698	143.731238	0.92203604	0	1.91936081	5.17498652	-0.3796702	4.96795211	-0.080087	-6.9077553	14	57	4.47038889	283.555556	10.55555555	0	0	0	1	30.519848	0	0.21141735
7.0515	191779.914	0.72838841	138.930705	0.72028383	0	1.95324036	5.25635365	-0.3155489	4.93398248	-0.3267226	-6.9077553	15	64	5.81150556	282.444444	9.444444444	0	0	0	1	30.519848	0	0.21141735
7.245	207545.341	0.69843452	134.060511	0.77762394	0	1.98031158	5.33535464	-0.3574831	4.89829873	-0.2502271	-6.9077553	16	66	6.25854444	282.444444	9.444444444	0	0	0	1	30.519848	0	0.21141735
7.4196	223419.557	0.68666037	124.691185	0.68585748	0	2.00412515	5.4090559	-0.3744602	4.82584818	-0.3756285	-6.9077553	17	64	5.81150556	281.888889	8.888888888	0	0	0	1	30.519848	0	0.21141735
7.5735	238378.742	0.74467615	149.096149	0.75493162	0	2.02465531	5.47386496	-0.2934639	5.0045981	-0.2798044	-6.9077553	18	62	8.0467	281.888889	8.888888888	0	0	0	1	30.519848	0	0.21141735
7.7283	253469.345	0.687206	122.245117	0.58714756	0	2.04488892	5.53524684	-0.3736671	4.80603637	-0.5307774	-6.9077553	19	63	5.81150556	281.888889	8.888888888	0	0	0	1	30.519848	0	0.21141735
7.8705	265744.912	0.68484941	122.318384	0.63639479	0	2.06312159	5.58254063	-0.3770972	4.80663553	-0.4503661	-6.9077553	20	65	5.81150556	281.888889	8.888888888	0	0	0	1	30.519848	0	0.21141735
7.9857	279261.752	0.62545294	99.8540497	0.49609411	0	2.07765244	5.6321531	-0.4676816	4.60371963	-0.6989759	-6.9077553	21	66	5.36446667	281.333333	8.333333333	0	0	0	1	30.519848	0	0.21141735
8.118	293575.048	0.61913865	99.1084715	0.61161171	0	2.09408382	5.68213671	-0.4778122	4.59622501	-0.490024	-6.9077553	22	67	4.91742778	281.333333	8.333333333	0	0	0	1	30.519848	0	0.21141735
9.0009	308787.827	0.70278959	154.867546	3.24706384	0	2.19732457	5.73265764	-0.3512759	5.04257667	1.17805908	-6.9077553	23	65	5.81150556	281.333333	8.333333333	0	0	0	1	30.519848	0	0.21141735
0.0135	658.886336	0.98276751	262.467547	1.7698337	0	-4.3050656	-0.4156877	-0.0163657	5.57013125	0.57145045	-6.9077553	0	98	2.68223333	289.666667	16.66666666	0	0	0	0	34.0724638	0	0.49277013
0.027	1141.38798	0.57505487	85.5840658	0.70477829	0	-3.6119184	0.13312079	-0.5515524	4.4495108	-0.3484541	-6.9077553	1	100	1.78815556	289.666667	16.66666666	0	0	0	0	34.0724638	0	0.49277013
0.0819	3153.94741	0.77640602	171.834641	1.92915057	0	-2.5022563	1.14897183	-0.2517925	5.14653844	0.65759801	-6.9077553	2	93	3.57631111	292.444444	19.44444444	0	0	0	0	34.0724638	0	0.49277013
0.1584	6789.75313	0.8911429	209.167233	1.4418895	0	-1.8426318	1.91556185	-0.114129	5.34313887	0.3666477	-6.9077553	3	76	4.47038889	294.111111	21.11111111	0	0	0	0	34.0724638	0	0.49277013
0.2646	10613.8497	0.92415192	224.397527	2.38986827	0	-1.329536	2.36225393	-0.0777973	5.41342361	0.87165659	-6.9077553	4	63	5.36446667	294.111111	21.11111111	0	0	0	1	34.0724638	0	0.49277013
0.4374	20341.4433	1.1930453	410.184326	3.47732862	0	-0.8269072	3.01270951	0.17734695	6.01660907	1.2465519	-6.9077553	5	55	5.36446667	295.222222	22.2222222	0	0	0	1	34.0724638	0	0.49277013
0.7614	41421.2975	1.3535731	522.96237	4.37313232	0	-0.2725964	3.72381932	0.30348635	6.25951142	1.47570817	-6.9077553	6	42	5.81150556	296.333333	23.33333333	0	0	0	1	34.0724638	0	0.49277013
1.1385	61280.5112	1.19290999	414.323277	3.93885064	0	0.12971161	4.11547819	0.17723362	6.02664895	1.37114281	-6.9077553	7	30	7.59966111	295.777778	22.7777778	0	0	0	1	34.0724638	0	0.49277013
1.7658	86988.3303	1.14700033	391.788379	3.84516581	0	0.56860385	4.46578547	0.13802158	5.9707244	1.34707676	-6.9077553	8	28	7.59966111	296.333333	23.33333333	0	0	0	1	34.0724638	0	0.49277013
2.5164	121176.159	1.12936701	384.358288	3.45794692	0	0.92282931	4.7972536	0.12254237	5.95157776	1.24096419	-6.9077553	9	34	8.49373889	294.111111	21.11111111	0	0	0	1	34.0724638	0	0.49277013
3.2067	147014.888	0.997956	281.716917	3.02510542	0	1.16524237	4.99054066	-0.0010445	5.64090627	1.10727645	-6.9077553	10	40	8.0467	290.777778	17.7777778	0	0	0	1	34.0724638	0	0.49277013
3.6621	165289.194	0.94514954	257.130435	2.11315387	0	1.29803675	5.10770268	-0.0553546	5.54958737	0.74865467	-6.9077553	11	47	6.70558333	288	15	0	0	0	1	34.0724638	0	0.49277013
4.1175	182435.581	0.90834805	242.692225	2.22891006	0	1.41524618	5.20640261	-0.0950274	5.4917982	0.80196125	-6.9077553	12	50	7.15262222	286.888889	13.88888888	0	0	0	1	34.0724638	0	0.49277013
4.4784	195816.164	0.84716584	204.204878	1.56087351	0	1.49926584	5.27718139	-0.1646791	5.31912869	0.44588607	-6.9077553	13	53	7.59966111	285.222222	12.22222222	0	0	0	1	34.0724638	0	0.49277013

4.7358	205835.845	0.75382398	158.046943	1.12718937	0	1.55515067	5.32708384	-0.2812707	5.06289843	0.12061402	-6.9077553	14	57	4.47038889	283.555556	10.55555555	0	0	0	1	34.0724638	0	0.49277013
4.9662	215565.966	0.83558744	184.666916	1.29433334	0	1.60265496	5.37327161	-0.1784242	5.21855916	0.25876807	-6.9077553	15	64	5.81150556	282.444444	9.444444444	0	0	0	1	34.0724638	0	0.49277013
5.2164	227627.512	0.96150702	263.825596	1.99183161	0	1.65180751	5.42771496	-0.0382139	5.57529205	0.68955655	-6.9077553	16	66	6.25854444	282.444444	9.444444444	0	0	0	1	34.0724638	0	0.49277013
5.4657	240060.962	0.92222491	235.231851	1.78644912	0	1.6984922	5.48089706	-0.0798824	5.46057588	0.58078953	-6.9077553	17	64	5.81150556	281.888889	8.888888888	0	0	0	1	34.0724638	0	0.49277013
5.7744	256047.129	1.02176749	290.006423	2.11898457	0	1.75343435	5.54536543	0.02251217	5.66990652	0.75140881	-6.9077553	18	62	8.0467	281.888889	8.888888888	0	0	0	1	34.0724638	0	0.49277013
6.0642	269892.237	0.98174736	266.604425	1.99582554	0	1.80240263	5.59802646	-0.0174032	5.58576976	0.69155869	-6.9077553	19	63	5.81150556	281.888889	8.888888888	0	0	0	1	34.0724638	0	0.49277013
6.228	276591.38	0.83679669	189.479932	1.46131176	0	1.82905525	5.62254487	-0.1769798	5.2442884	0.38001858	-6.9077553	20	65	5.81150556	281.888889	8.888888888	0	0	0	1	34.0724638	0	0.49277013
6.3567	282329.492	0.78723635	170.898465	1.40300937	0	1.84950937	5.64307834	-0.2379573	5.14107546	0.33933198	-6.9077553	21	66	5.36446667	281.333333	8.333333333	0	0	0	1	34.0724638	0	0.49277013
6.4989	287288.705	0.70239982	153.901849	1.37640033	0	1.87163293	5.66049113	-0.3518298	5.03632155	0.32019791	-6.9077553	22	67	4.91742778	281.333333	8.333333333	0	0	0	1	34.0724638	0	0.49277013
7.0911	298193.672	0.65526291	138.282556	2.57533204	0	1.95884048	5.69774654	-0.4211938	4.92930633	0.9463667	-6.9077553	23	65	5.81150556	281.333333	8.333333333	0	0	0	1	34.0724638	0	0.49277013
0.0324	2058.42499	1.11857943	347.006752	2.30825871	0	-3.4295969	0.72242681	0.1129531	5.84934712	0.83692657	-6.9077553	0	98	2.68223333	289.666667	16.66666666	0	0	0	0	42.288408	0	-0.4111751
0.1161	6330.42532	0.96779575	257.06082	1.98954379	0	-2.1533034	1.84552538	-0.0317015	5.5493166	0.68840786	-6.9077553	1	100	1.78815556	289.666667	16.66666666	0	0	0	0	42.288408	0	-0.4111751
0.3402	15325.3356	1.02251241	288.33189	3.39435795	0	-1.0782216	2.72957263	0.02324025	5.66411568	1.22240919	-6.9077553	2	93	3.57631111	292.444444	19.44444444	0	0	0	0	42.288408	0	-0.4111751
0.567	25126.9668	0.91659485	222.841456	1.80514284	0	-0.567396	3.22398144	-0.0859993	5.40646505	0.59119354	-6.9077553	3	76	4.47038889	294.111111	21.11111111	0	0	0	0	42.288408	0	-0.4111751
0.8649	36120.249	0.91123497	220.976917	2.41349677	0	-0.1451414	3.58688131	-0.0918577	5.39806277	0.88149089	-6.9077553	4	63	5.36446667	294.111111	21.11111111	0	0	0	0	42.288408	0	-0.4111751
1.2087	50347.1743	0.86601185	199.43613	1.70833312	0	0.1895454	3.91896236	-0.1427026	5.29549905	0.53610331	-6.9077553	5	55	5.36446667	295.222222	22.2222222	0	0	0	0	42.288408	0	-0.4111751
1.7649	71593.6551	0.98546277	285.285093	3.00304228	0	0.56809403	4.27102042	-0.0136297	5.65349251	1.09995881	-6.9077553	6	42	5.81150556	296.333333	23.33333333	0	0	1	0	42.288408	0	-0.4111751
2.5731	109853.6	1.12314414	375.989908	3.68512004	0	0.9451114	4.69915767	0.11702198	5.92956496	1.30457442	-6.9077553	7	30	7.59966111	295.777778	22.7777778	0	0	1	0	42.288408	0	-0.4111751
3.5613	152808.629	1.12019923	399.092213	3.86470429	0	1.27012565	5.02919289	0.11439886	5.98919501	1.35214389	-6.9077553	8	28	7.59966111	296.333333	23.33333333	0	0	1	0	42.288408	0	-0.4111751
4.1571	169612.581	0.86444184	226.408705	2.74700282	0	1.42481772	5.1335228	-0.1445151	5.42234621	1.0108744	-6.9077553	9	34	8.49373889	294.111111	21.11111111	0	0	1	0	42.288408	0	-0.4111751
4.6476	181167.614	0.73296632	156.663073	1.96146229	0	1.53635096	5.19942817	-0.3092921	5.05410385	0.67419996	-6.9077553	10	40	8.0467	290.777778	17.7777778	0	0	1	0	42.288408	0	-0.4111751
5.0193	192575.862	0.69557415	144.882396	1.37423211	0	1.61329048	5.26049536	-0.361581	4.97592926	0.31862252	-6.9077553	11	47	6.70558333	288	15	0	0	1	0	42.288408	0	-0.4111751
5.4081	205579.332	0.70428629	149.003707	1.27591931	0	1.68789783	5.32583687	-0.3491515	5.0039779	0.24445039	-6.9077553	12	50	7.15262222	286.888889	13.88888888	0	0	1	0	42.288408	0	-0.4111751
5.7456	216438.843	0.67039014	134.510875	1.15244906	0	1.74843434	5.37731265	-0.3984049	4.90165249	0.14275663	-6.9077553	13	53	7.59966111	285.222222	12.22222222	0	0	1	0	42.288408	0	-0.4111751
6.0813	228865.64	0.74942974	163.922064	1.24053276	0	1.80521849	5.43313948	-0.2871093	5.09939719	0.21634671	-6.9077553	14	57	4.47038889	283.555556	10.55555555	0	0	1	0	42.288408	0	-0.4111751
6.2838	237968.092	0.80907484	172.055295	1.19875326	0	1.83797489	5.4721408	-0.2106286	5.14782172	0.18211591	-6.9077553	15	64	5.81150556	282.444444	9.444444444	0	0	1	0	42.288408	0	-0.4111751
6.462	245794.033	0.75064688	151.245502	1.23522325	0	1.86593887	5.50449799	-0.2854886	5.01891097	0.21206097	-6.9077553	16	66	6.25854444	282.444444	9.444444444	0	0	1	0	42.288408	0	-0.4111751
6.6303	253352.03	0.76187574	154.47792	1.19856107	0	1.89165005	5.53478389	-0.2706601	5.04005765	0.18195572	-6.9077553	17	64	5.81150556	281.888889	8.888888888	0	0	1	0	42.288408	0	-0.4111751
6.8436	263688.863	0.89883668	227.817741	1.67206815	0	1.92331391	5.57477365	-0.105542	5.42855032	0.51465916	-6.9077553	18	62	8.0467	281.888889	8.888888888	0	0	1	0	42.288408	0	-0.4111751
7.0659	274798.351	0.89590491	223.85403	1.67106598	0	1.9552804	5.6160412	-0.1088054	5.41099865	0.51405997	-6.9077553	19	63	5.81150556	281.888889	8.888888888	0	0	1	0	42.288408	0	-0.4111751
7.3062	286616.245	0.87580169	206.43058	1.53382605	0	1.9887233	5.65814769	-0.1314744	5.32996903	0.42841705	-6.9077553	20	65	5.81150556	281.888889	8.888888888	0	0	1	0	42.288408	0	-0.4111751
7.6014	301266.108	0.90304584	225.321313	1.66757686	0	2.02833244	5.70799727	-0.1008752	5.41753188	0.51197108	-6.9077553	21	66	5.36446667	281.333333	8.333333333	0	0	1	0	42.288408	0	-0.4111751
7.9281	316987.933	0.84430251	202.077652	1.56186224	0	2.07041341	5.75886686	-0.1680607	5.30865699	0.44651891	-6.9077553	22	67	4.91742778	281.333333	8.333333333	0	0	1	0	42.288408	0	-0.4111751
8.7894	338233.483	0.67720496	145.234244	2.3365717	0	2.17354645	5.82373939	-0.3883057	4.9783548	0.84911266	-6.9077553	23	65	5.81150556	281.333333	8.333333333	0	0	1	0	42.288408	0	-0.4111751
0.0018	0	0.18587095	6.69716311	0.50842071	0	-6.3199686	-6.9077553	-1.677337	1.90183333	-0.6744811	-6.9077553	0	98	2.68223333	289.666667	16.66666666	0	0	0	0	18.9135952	0	-0.0428739
0.0036	0	0.11431472	3.30488801	0.37079287	0	-5.6268214	-6.9077553	-2.1600902	1.19570512	-0.9894184	-6.9077553	1	100	1.78815556	289.666667	16.66666666	0	0	0	0	18.9135952	0	-0.0428739
0.0324	0	0.43157792	49.1256638	1.58667588	0	-3.4295969	-6.9077553	-0.8379928	3.89440194	0.46227124	-6.9077553	2	93	3.57631111	292.444444	19.44444444	0	0	0	0	18.9135952	0	-0.0428739
0.1557	219.611775	0.58384727	90.3210538	2.6889243	0	-1.8598242	-1.5113508	-0.5364045	4.50338166	0.98951305	-6.9077553	3	76	4.47038889	294.111111	21.11111111	0	0	1	0	18.9135952	0	-0.0428739
0.3843	4099.40148	0.71947431	178.145531	2.80280406	0	-0.9563318	1.41108489	-0.3278455	5.18260642	1.03097709	-6.9077553	4	63	5.36446667	294.111111	21.11111111	0	0	1	0	18.9135952	0	-0.0428739
0.5877	12070.5733	0.88287245	272.325204	2.36307924	0	-0.5315387	2.49085338	-0.1234425	5.60700063	0.86038862	-6.9077553	5	55	5.36446667	295.222222	22.2222222	0	0	1	0	18.9135952	0	-0.0428739
0.7821	16422.9475	0.72469867	163.519097	1.80479205	0	-0.2457727	2.79874048	-0.3206204	5.0969359	0.5909993	-6.9077553	6	42	5.81150556	296.333333	23.33333333	0	0	1	0	18.9135952	0	-0.0428739
1.0575	21650.4344	0.78642946	197.854167	2.5466169	0	0.05590763	3.0750717	-0.2389815	5.28753528	0.93515837	-6.9077553	7	30	7.59966111	295.777778	22.7777778	0	0	1	0	18.9135952	0	-0.0428739
1.3428	30165.1579	0.78609339	197.136263	1.90756967	0	0.29475699	3.4067207	-0.2394084	5.28390025	0.6463541	-6.9077553	8	28	7.59966111	296.333333	23.33333333	0	0	1	0	18.9135952	0	-0.0428739
1.6461	37074.4305	0.73356796	163.641158	2.27978498	0	0.49840885	3.6129545	-0.3084728	5.09768208	0.82451967	-6.9077553	9	34	8.49373889	294.111111	21.11111111	0	0	1	0	18.9135952	0	-0.0428739
1.9908	44413.3236	0.72275219	159.191835	2.33922926	0	0.68853657	3.79356202	-0.3233062	5.07011626	0.8502489	-6.9077553	10	40	8.0467	290.777778	17.7777778	0	0	1	0	18.9135952	0	-0.0428739
2.3085	56344.7485	0.79632326	201.814669	1.82113483	0	0.83659796	4.03150679	-0.2264951	5.30735475	0.6000088	-6.9077553	11	47	6.70558333	288	15	0	0	1	0	18.9135952	0	-0.0428739
2.637	70136.1989	0.85057641	225.117123	1.84627084	0	0.96964191	4.25045331	-0.1606661	5.41662526	0.61370933	-6.9077553	12	50	7.15262222	286.888889	13.88888888	0	0	1	0	18.9135952	0	-0.0428739
2.9853	87253.0343	0.92730713	262.877463	1.90635031	0	1.09370024	4.4688238	-0.0743926	5.57169181	0.645715	-6.9077553	13	53	7.59966111	285.222222	12.22222222	0	0	1	0	18.9135952	0	-0.0428739
3.3093	102537.612	0.88917414	224.995747	1.59137065	0	1.19673669	4.63023943	-0.1163382	5.41608594	0.46522388	-6.9077553	14	57	4.47038889	283.555556	10.55555555	0	0	1	0	18.9135952	0	-0.0428739
3.5892	116629.59	0.88432287	212.656551	1.4867747	0	1.27792934	4.75901159	-0.1218029	5.35968313	0.39728152	-6.9077553	15	64	5.81150556	282.444444	9.444444444	0	0	1	0	18.9135952	0	-0.0428739
3.8835	132205.233	0.90213442	224.36229	1.60293943	0	1.35673681	4.88436308	-0.1018839	5.41326657	0.47246275	-6.9077553	16	66	6.25854444	282.444444	9.444444444	0	0	1	0	18.9135952	0	-0.0428739
4.1859	147602.494	0.88083049	210.98763	1.56806437	0	1.43172173	4.99452959	-0.1257554	5.35180425	0.4504795	-6.9077553	17	64	5.81150556	281.888889	8.888888888	0	0	1	0	18.9135952	0	-0.0428739
4.545	167660.259	0.99715728	281.72061	2.02138188	0	1.51402773	5.12194563	-0.0018444	5.64091939	0.70427597	-6.9077553	18	62	8.0467	281.888889	8.888888888	0	0	1	0	18.9135952	0	-0.0428739
4.9005	186526.82	0.96413049	263.550178	1.9024924	0	1.58933724	5.22858039	-0.035492	5.57424757	0.6436903	-6.9077553	19	63	5.81150556	281.888889	8.888888888	0	0	1	0	18.9135952	0	-0.0428739
5.3046	210215.816	1.01924721	298.38283	2.14670473	0	1.66857437	5.34813946	0.02004496	5.69838068	0.7643997	-6.9077553	20	65	5.81150556	281.888889	8.888888888	0	0	1	0	18.9135952	0	-0.0428739
5.6682	229618.14	0.97284223	264.794748	1.92833249	0	1.73487161	5.43642202	-0.026506	5.57895877	0.65717408	-6.9077553	21	66	5.36446667	281.333333	8.333333333	0	0	1	0	18.9135952	0	-0.0428739
6.039	247226.249	0.87590968	225.306394	1.76008947	0	1.79823844	5.51030795	-0.1313513	5.41746567	0.56593263	-6.9077553	22	67	4.91742778	281.333333	8.333333333	0	0	1	0	18.9135952	0	-0.0428739
6.8931	265002.902	0.62361388	118.471146	2.11472062	0	1.93052091	5.57974455	-0.4706216	4.77467787	0.74939548	-6.9077553	23	65	5.81150556	281.333333	8.333333333	0	0	1	0	18.9135952	0	-0.0428739
0.0081	322.040679	0.81249166	182.690735	1.76276875	0	-4.8158912	-1.129977	-0.2064196	5.20780022	0.56745285	-6.9077553	0	89	4.47038889	295.222222	22.2222222	0	0	0	0	30.519848	0	0.21141735
0.0108	453.632025	0.41583176	44.0034587	0.71760146	0	-4.5282091	-0.7882669	-0.8750726	3.78429096	-0.3304484	-6.9077553	1	88	4.91742778	295.222222	22.2222222	0	0	0	0	30.519848	0	0.21141735
0.0216	936.133797	0.53443503	69.1541087	0.82201385	0	-3.835062	-0.0649292	-0.6246757	4.23635193	-0.1947822	-6.9077553	2	86	5.36446667	295.222222	22.2222222	0	0	0	0	30.519848	0	0.21141735
0.0495	1331.02144	0.51659541	87.0421024	1.19498197	0	-3.0057826	0.28669767	-0.6585614	4.46640343	0.17896758	-6.9077553	3	98	2.68223333	289.666667	16.66666666	0	0	0	0	30.519848	0	0.21141735

0.0792	1857.38691	0.39217232	45.2402196	0.97431587	0	-2.535779	0.61970886	-0.9335073	3.81200861	-0.0249939	-6.9077553	4	100	1.78815556	289.666667	16.66666666	0	0	0	0	30.519848	0	0.21141735
0.1422	2501.20604	0.44264494	59.451702	1.19523686	0	-1.9505208	0.91717276	-0.8127307	4.08518107	0.17918068	-6.9077553	5	93	3.57631111	292.444444	19.44444444	0	0	0	0	30.519848	0	0.21141735
0.3132	3483.49087	0.57953997	99.9439145	2.56133102	0	-1.1609133	1.24832194	-0.5437966	4.60461918	0.9409174	-6.9077553	6	76	4.47038889	294.111111	21.11111111	0	0	0	0	30.519848	0	0.21141735
0.4716	4486.1125	0.62260194	102.340254	2.1461355	0	-0.7516241	1.5012094	-0.472243	4.62831286	0.76413463	-6.9077553	7	63	5.36446667	294.111111	21.11111111	0	0	0	1	30.519848	0	0.21141735
0.738	7205.07969	0.6682099	122.97845	2.31177706	0	-0.3038115	1.97492507	-0.4016575	4.81201727	0.83844899	-6.9077553	8	55	5.36446667	295.222222	22.2222222	0	0	0	1	30.519848	0	0.21141735
1.1061	16568.3515	0.74835429	156.209849	2.58042732	0	0.10084031	2.80755469	-0.2885434	5.05120669	0.94834247	-6.9077553	9	42	5.81150556	296.333333	23.33333333	0	0	0	1	30.519848	0	0.21141735
1.854	31001.8393	1.04347312	352.425955	4.95919826	0	0.61734547	3.43407879	0.04351256	5.86484338	1.60144571	-6.9077553	10	30	7.59966111	295.777778	22.7777778	0	0	0	1	30.519848	0	0.21141735
2.502	46656.9356	0.80885688	197.690148	2.46349521	0	0.91709041	3.84284302	-0.2108977	5.28670595	0.901987	-6.9077553	11	28	7.59966111	296.333333	23.33333333	0	0	0	1	30.519848	0	0.21141735
3.2283	66338.2572	0.77114827	182.856645	2.12058429	0	1.17195568	4.19478184	-0.2585787	5.20870795	0.75216312	-6.9077553	12	34	8.49373889	294.111111	21.11111111	0	0	0	1	30.519848	0	0.21141735
4.0428	89001.6921	0.80486069	196.415521	2.24926974	0	1.39693752	4.48866662	-0.2158444	5.28023751	0.81105009	-6.9077553	13	40	8.0467	290.777778	17.7777778	0	0	0	1	30.519848	0	0.21141735
4.9059	109837.151	0.74811147	165.101799	1.57548693	0	1.59043856	4.69900793	-0.2888675	5.1065683	0.45519891	-6.9077553	14	47	6.70558333	288	15	0	0	0	1	30.519848	0	0.21141735
5.625	129120.319	0.68974325	137.722915	1.57442638	0	1.72722095	4.86075242	-0.3699871	4.92525107	0.45452595	-6.9077553	15	50	7.15262222	286.888889	13.88888888	0	0	0	1	30.519848	0	0.21141735
6.2469	149236.099	0.70348419	152.577582	1.18136831	0	1.83208534	5.00553631	-0.3502894	5.02767976	0.16751947	-6.9077553	16	53	7.59966111	285.222222	12.22222222	0	0	0	1	30.519848	0	0.21141735
6.6033	166698.124	0.66712705	128.702494	0.71541511	0	1.88756952	5.11619053	-0.4032769	4.85751126	-0.3334955	-6.9077553	17	57	4.47038889	283.555556	10.55555555	0	0	0	1	30.519848	0	0.21141735
6.7959	183366.437	0.72405956	140.835992	0.72343866	0	1.91631949	5.21149199	-0.3215015	4.94760314	-0.3223582	-6.9077553	18	64	5.81150556	282.444444	9.444444444	0	0	0	1	30.519848	0	0.21141735
6.9777	196871.063	0.71209128	134.834429	0.76663495	0	1.94271935	5.28255409	-0.3381458	4.90405499	-0.264441	-6.9077553	19	66	6.25854444	282.444444	9.444444444	0	0	0	1	30.519848	0	0.21141735
7.1793	212349.595	0.70165621	127.43764	0.72522681	0	1.97120189	5.35823866	-0.3528875	4.84763499	-0.3198929	-6.9077553	20	64	5.81150556	281.888889	8.888888888	0	0	0	1	30.519848	0	0.21141735
7.4097	230708.821	0.75711131	158.798492	0.96853399	0	2.00278995	5.44116074	-0.2769251	5.06764235	-0.0309397	-6.9077553	21	62	8.0467	281.888889	8.888888888	0	0	0	1	30.519848	0	0.21141735
7.6293	249569.18	0.75532457	152.697938	0.95106988	0	2.0319961	5.51974016	-0.2792847	5.02846826	-0.0491168	-6.9077553	22	63	5.81150556	281.888889	8.888888888	0	0	0	1	30.519848	0	0.21141735
7.8246	265610.506	0.71132762	135.006948	0.81957234	0	2.05727262	5.58203474	-0.3392173	4.90533365	-0.1977532	-6.9077553	23	65	5.81150556	281.888889	8.888888888	0	0	0	1	30.519848	0	0.21141735
0.0171	742.733102	0.98531967	267.124051	1.96006478	0	-4.0686768	-0.296073	-0.0137748	5.5877169	0.67348758	-6.9077553	0	89	4.47038889	295.222222	22.2222222	0	0	0	0	34.0724638	0	0.49277013
0.0315	1312.9624	0.50045651	62.2999992	0.90719795	0	-3.4577677	0.27304731	-0.6902384	4.13197746	-0.0962929	-6.9077553	1	88	4.91742778	295.222222	22.2222222	0	0	0	0	34.0724638	0	0.49277013
0.0486	1883.19146	0.51273813	63.9651264	0.82944674	0	-3.0241317	0.6334988	-0.6660416	4.15835367	-0.1857915	-6.9077553	2	86	5.36446667	295.222222	22.2222222	0	0	0	0	34.0724638	0	0.49277013
0.0702	2717.33088	0.54898655	79.6882833	0.90352225	0	-2.656407	1.00001804	-0.5978614	4.37813511	-0.1003484	-6.9077553	3	98	2.68223333	289.666667	16.66666666	0	0	0	0	34.0724638	0	0.49277013
0.1161	4700.12786	0.7277835	142.138117	1.22505076	0	-2.1533034	1.54780245	-0.3163786	4.95680627	0.20379824	-6.9077553	4	100	1.78815556	289.666667	16.66666666	0	0	0	0	34.0724638	0	0.49277013
0.1818	7586.51311	0.83858689	187.147608	1.56850548	0	-1.7048481	2.02650388	-0.1748453	5.231903	0.45076059	-6.9077553	5	93	3.57631111	292.444444	19.44444444	0	0	0	0	34.0724638	0	0.49277013
0.3033	12961.6913	0.95727245	250.415567	2.21995488	0	-1.1930329	2.56207533	-0.0426231	5.5231258	0.79793723	-6.9077553	6	76	4.47038889	294.111111	21.11111111	0	0	0	1	34.0724638	0	0.49277013
0.5697	25514.7565	1.15946651	383.235563	3.47491373	0	-0.5626454	3.23929616	0.14882209	5.94865246	1.24585739	-6.9077553	7	63	5.36446667	294.111111	21.11111111	0	0	0	1	34.0724638	0	0.49277013
1.0728	48250.9843	1.29377334	491.574799	4.06447584	1	0.07027205	3.87643695	0.25833566	6.19761615	1.40253079	0.0009995	8	55	5.36446667	295.222222	22.2222222	0	0	0	1	34.0724638	0	0.49277013
1.6389	63907.4629	1.16085442	399.843974	3.54702793	1	0.49402529	4.15745179	0.15001737	5.99107691	1.26639194	0.0009995	9	42	5.81150556	296.333333	23.33333333	0	0	0	1	34.0724638	0	0.49277013
2.4093	100383	1.24822442	461.616092	3.77063644	1	0.87933625	4.60900283	0.22252289	6.13473574	1.32750898	0.0009995	10	30	7.59966111	295.777778	22.7777778	0	0	0	1	34.0724638	0	0.49277013
3.2337	130411.836	1.11141155	366.688319	3.60806714	1	1.17362699	4.87070508	0.10653023	5.90451495	1.28344933	0.0009995	11	28	7.59966111	296.333333	23.33333333	0	0	0	1	34.0724638	0	0.49277013
3.7008	147086.385	1.00959299	302.361964	2.75960028	1	1.30854901	4.99102687	0.01053727	5.71162816	1.01544815	0.0009995	12	34	8.49373889	294.111111	21.11111111	0	0	0	1	34.0724638	0	0.49277013
3.762	147086.388	0.74188749	147.09352	1.16031119	1	1.32495073	4.99102688	-0.2972107	4.99107537	0.14954971	0.0009995	13	40	8.0467	290.777778	17.7777778	0	0	0	1	34.0724638	0	0.49277013
3.8259	147174.116	0.70524372	145.955738	1.21303537	1	1.34179373	4.99162314	-0.3477949	4.98331027	0.19394983	0.0009995	14	47	6.70558333	288	15	0	0	0	1	34.0724638	0	0.49277013
3.9051	147523.964	0.68867215	171.141224	1.34677297	1	1.36228339	4.99399741	-0.3715389	5.14249493	0.29845358	0.0009995	15	50	7.15262222	286.888889	13.88888888	0	0	0	1	34.0724638	0	0.49277013
3.9717	148600.23	0.67075647	156.505002	1.30147986	1	1.37919421	5.00126641	-0.3978594	5.05309436	0.26427003	0.0009995	16	53	7.59966111	285.222222	12.22222222	0	0	0	1	34.0724638	0	0.49277013
4.0356	150121.27	0.68298431	142.900239	1.03797225	1	1.39515499	5.01145009	-0.3798203	4.96215376	0.038232	0.0009995	17	57	4.47038889	283.555556	10.55555555	0	0	0	1	34.0724638	0	0.49277013
4.0851	151756.402	0.72632564	145.881978	1.03329759	1	1.40734621	5.0222832	-0.318381	4.98280478	0.03372254	0.0009995	18	64	5.81150556	282.444444	9.444444444	0	0	0	1	34.0724638	0	0.49277013
4.158	154998.616	0.84825869	196.635272	1.54431388	1	1.42503419	5.04342264	-0.1633914	5.28135569	0.43522705	0.0009995	19	66	6.25854444	282.444444	9.444444444	0	0	0	1	34.0724638	0	0.49277013
4.2444	158859.538	0.90599648	228.648356	1.81994549	1	1.44560047	5.0680267	-0.0976167	5.43218963	0.59935587	0.0009995	20	64	5.81150556	281.888889	8.888888888	0	0	0	1	34.0724638	0	0.49277013
4.2975	161081.213	0.85256247	199.303959	1.59020725	1	1.45803346	5.08191487	-0.1583365	5.29483661	0.464493	0.0009995	21	62	8.0467	281.888889	8.888888888	0	0	0	1	34.0724638	0	0.49277013
4.3623	164241.45	0.96164407	268.817654	2.01346885	1	1.47299944	5.10134369	-0.0380715	5.594037	0.70035556	0.0009995	22	63	5.81150556	281.888889	8.888888888	0	0	0	1	34.0724638	0	0.49277013
4.437	167547.034	0.82037354	180.940065	1.36714586	1	1.48997847	5.12127008	-0.1967773	5.19817137	0.31345644	0.0009995	23	65	5.81150556	281.888889	8.888888888	0	0	0	1	34.0724638	0	0.49277013
0.036					0	-3.3242363					-6.9077553	0	89	4.47038889	295.222222	22.2222222	0	0	0	0	42.288408	0	-0.4111751
0.0873					0	-2.4384048					-6.9077553	1	88	4.91742778	295.222222	22.2222222	0	0	0	0	42.288408	0	-0.4111751
0.1512					0	-1.8891518					-6.9077553	2	86	5.36446667	295.222222	22.2222222	0	0	0	0	42.288408	0	-0.4111751
0.3114					0	-1.166677					-6.9077553	3	98	2.68223333	289.666667	16.66666666	0	0	0	0	42.288408	0	-0.4111751
0.5229					0	-0.648365					-6.9077553	4	100	1.78815556	289.666667	16.66666666	0	0	0	0	42.288408	0	-0.4111751
0.7632					0	-0.2702352					-6.9077553	5	93	3.57631111	292.444444	19.44444444	0	0	1	0	42.288408	0	-0.4111751
1.0467					0	0.04564236					-6.9077553	6	76	4.47038889	294.111111	21.11111111	0	0	1	0	42.288408	0	-0.4111751
1.4085					0	0.34252531					-6.9077553	7	63	5.36446667	294.111111	21.11111111	0	0	1	0	42.288408	0	-0.4111751
1.9593					0	0.67258727					-6.9077553	8	55	5.36446667	295.222222	22.2222222	0	0	1	0	42.288408	0	-0.4111751
2.7036					0	0.99458422					-6.9077553	9	42	5.81150556	296.333333	23.33333333	0	0	1	0	42.288408	0	-0.4111751
3.7359					1	1.31798875					0.0009995	10	30	7.59966111	295.777778	22.7777778	0	0	1	0	42.288408	0	-0.4111751
4.5909					2	1.52407608					0.69364706	11	28	7.59966111	296.333333	23.33333333	0	0	1	0	42.288408	0	-0.4111751
5.1138					2	1.63194277					0.69364706	12	34	8.49373889	294.111111	21.11111111	0	0	1	0	42.288408	0	-0.4111751
5.6241					2	1.72706094					0.69364706	13	40	8.0467	290.777778	17.7777778	0	0	1	0	42.288408	0	-0.4111751
6.1038					2	1.80891153					0.69364706	14	47	6.70558333	288	15	0	0	1	0	42.288408	0	-0.4111751
6.6033					2	1.88756952					0.69364706	15	50	7.15262222	286.888889	13.88888888	0	0	1	0	42.288408	0	-0.4111751
6.9975					2	1.94555294					0.69364706	16	53	7.59966111	285.222222	12.22222222	0	0	1	0	42.288408	0	-0.4111751
7.3053					2	1.98860011					0.69364706	17	57	4.47038889	283.555556	10.55555555	0	0	1	0	42.288408	0	-0.4111751

7.5366					2	2.01977115					0.69364706	18	64	5.81150556	282.444444	9.444444444	0	0	1	0	42.288408	0	-0.4111751
7.7877					2	2.05254557					0.69364706	19	66	6.25854444	282.444444	9.444444444	0	0	1	0	42.288408	0	-0.4111751
8.0262					2	2.08271119					0.69364706	20	64	5.81150556	281.888889	8.888888888	0	0	1	0	42.288408	0	-0.4111751
8.3736					2	2.1250839					0.69364706	21	62	8.0467	281.888889	8.888888888	0	0	1	0	42.288408	0	-0.4111751
8.7309					2	2.16686846					0.69364706	22	63	5.81150556	281.888889	8.888888888	0	0	1	0	42.288408	0	-0.4111751
9.0432					2	2.20201309					0.69364706	23	65	5.81150556	281.888889	8.888888888	0	0	1	0	42.288408	0	-0.4111751
0.0018	0	0.58418107	80.7339172	2.82491279	0	-6.3199686	-6.9077553	-0.535834	4.39117116	1.03883142	-6.9077553	0	89	4.47038889	295.222222	22.2222222	0	0	0	0	18.9135952	0	-0.0428739
0.0018	0	0	0	0	0	-6.3199686	-6.9077553	-6.9077553	-6.9077553	-6.9077553	-6.9077553	1	88	4.91742778	295.222222	22.2222222	0	0	0	0	18.9135952	0	-0.0428739
0.0018	0	0	0	0	0	-6.3199686	-6.9077553	-6.9077553	-6.9077553	-6.9077553	-6.9077553	2	86	5.36446667	295.222222	22.2222222	0	0	0	0	18.9135952	0	-0.0428739
0.0018	0	0	0	0	0	-6.3199686	-6.9077553	-6.9077553	-6.9077553	-6.9077553	-6.9077553	3	98	2.68223333	289.666667	16.66666666	0	0	0	0	18.9135952	0	-0.0428739
0.0027	0	0.13822962	3.51805902	0.3471278	0	-5.9145035	-6.9077553	-1.9716307	1.25819363	-1.0551856	-6.9077553	4	100	1.78815556	289.666667	16.66666666	0	0	0	0	18.9135952	0	-0.0428739
0.0198	0	0.31914322	25.190971	1.10843377	0	-3.9220733	-6.9077553	-1.1389868	3.22652533	0.10384977	-6.9077553	5	93	3.57631111	292.444444	19.44444444	0	0	0	0	18.9135952	0	-0.0428739
0.1251	43.8919331	0.57809077	91.9385433	2.84445973	0	-2.0786419	-3.1034972	-0.5462961	4.52113122	1.04572465	-6.9077553	6	76	4.47038889	294.111111	21.11111111	0	0	0	0	18.9135952	0	-0.0428739
0.333	2375.99466	0.68310904	156.676458	2.91781022	0	-1.0996128	0.86583694	-0.379638	5.05418928	1.07117607	-6.9077553	7	63	5.36446667	294.111111	21.11111111	0	0	1	0	18.9135952	0	-0.0428739
0.5319	10798.7816	0.93883766	308.14865	2.49402877	0	-0.6312998	2.37952591	-0.0620481	5.73058554	0.91430026	-6.9077553	8	55	5.36446667	295.222222	22.2222222	0	0	1	0	18.9135952	0	-0.0428739
0.7101	15423.2249	0.74308052	173.369042	1.89490825	0	-0.3423495	2.73593932	-0.295606	5.15542828	0.63969801	-6.9077553	9	42	5.81150556	296.333333	23.33333333	0	0	1	0	18.9135952	0	-0.0428739
0.9306	19590.3897	0.72240862	171.995759	1.94097477	0	-0.0719257	2.97509017	-0.323781	5.14747563	0.66370538	-6.9077553	10	30	7.59966111	295.777778	22.7777778	0	0	1	0	18.9135952	0	-0.0428739
1.2132	27502.005	0.81755056	204.119103	2.14561511	0	0.1932615	3.31429527	-0.2002201	5.31870856	0.76389223	-6.9077553	11	28	7.59966111	296.333333	23.33333333	0	0	1	0	18.9135952	0	-0.0428739
1.485	34738.8733	0.72791664	165.097695	1.75155781	0	0.39541477	3.54788811	-0.3161959	5.10654345	0.56107633	-6.9077553	12	34	8.49373889	294.111111	21.11111111	0	0	1	0	18.9135952	0	-0.0428739
1.773	41168.9035	0.68006539	139.088086	2.02823563	0	0.57267303	3.71770749	-0.384097	4.93511463	0.70765918	-6.9077553	13	40	8.0467	290.777778	17.7777778	0	0	1	0	18.9135952	0	-0.0428739
2.0601	47942.2583	0.67609714	139.17899	1.86437395	0	0.72275453	3.87001819	-0.3899405	4.93576798	0.62346154	-6.9077553	14	47	6.70558333	288	15	0	0	1	0	18.9135952	0	-0.0428739
2.3796	61258.2397	0.82632051	229.990361	1.95836147	0	0.86693241	4.11511469	-0.1895631	5.43804175	0.67261864	-6.9077553	15	50	7.15262222	286.888889	13.88888888	0	0	1	0	18.9135952	0	-0.0428739
2.6865	74781.1597	0.86208444	224.101494	1.70323812	0	0.98823923	4.31457935	-0.1472427	5.41210351	0.53311816	-6.9077553	16	53	7.59966111	285.222222	12.22222222	0	0	1	0	18.9135952	0	-0.0428739
3.0069	90605.1896	0.91214615	240.336025	1.6706543	0	1.10090965	4.50652253	-0.0908593	5.48204221	0.51381373	-6.9077553	17	57	4.47038889	283.555556	10.55555555	0	0	1	0	18.9135952	0	-0.0428739
3.2913	105140.574	0.92472762	235.92766	1.72702915	0	1.19128262	4.65530777	-0.0771752	5.46352947	0.54698154	-6.9077553	18	64	5.81150556	282.444444	9.444444444	0	0	1	0	18.9135952	0	-0.0428739
3.5802	119627.223	0.88888036	214.262607	1.57014708	0	1.27541866	4.7843888	-0.1166682	5.36720707	0.45180598	-6.9077553	19	66	6.25854444	282.444444	9.444444444	0	0	1	0	18.9135952	0	-0.0428739
3.8592	133804.222	0.86853126	203.540877	1.5157391	0	1.35045991	4.89638518	-0.139801	5.31587177	0.4165627	-6.9077553	20	64	5.81150556	281.888889	8.888888888	0	0	1	0	18.9135952	0	-0.0428739
4.2246	153606.662	0.9845315	271.785791	1.98112751	0	1.44092458	5.0344017	-0.0145742	5.6050179	0.68417077	-6.9077553	21	62	8.0467	281.888889	8.888888888	0	0	1	0	18.9135952	0	-0.0428739
4.5783	173076.097	0.96771613	268.385536	1.96530221	0	1.52132775	5.15373714	-0.0317837	5.59242824	0.67615473	-6.9077553	22	63	5.81150556	281.888889	8.888888888	0	0	1	0	18.9135952	0	-0.0428739
4.9329	193363.783	0.99030548	279.268817	1.99092358	0	1.59592705	5.26457847	-0.0087325	5.6321784	0.68910079	-6.9077553	23	65	5.81150556	281.888889	8.888888888	0	0	1	0	18.9135952	0	-0.0428739
0.0135	401.190314	0.80384132	179.214896	1.47251783	0	-4.3050656	-0.9108299	-0.2171101	5.1885912	0.38765262	-6.9077553	0	92	1.78815556	295.777778	22.7777778	0	0	0	0	30.519848	0	0.21141735
0.0315	1059.14713	0.56792259	88.7475647	0.94147949	0	-3.4577677	0.0584077	-0.5640109	4.48580726	-0.0592411	-6.9077553	1	90	2.68223333	296.888889	23.88888888	0	0	0	0	30.519848	0	0.21141735
0.0729	1629.55196	0.58527354	106.659562	1.25176761	0	-2.6186666	0.48891858	-0.5339688	4.66965147	0.2253552	-6.9077553	2	83	3.12927222	298.555556	25.55555555	0	0	0	0	30.519848	0	0.21141735
0.153	2419.09996	0.46398797	60.2478165	1.50090918	0	-1.8773174	0.88380884	-0.7657437	4.09848293	0.40673709	-6.9077553	3	77	4.47038889	299.111111	26.11111111	0	0	0	0	30.519848	0	0.21141735
0.2394	3116.45877	0.53945279	79.5783183	1.86562443	0	-1.4296195	1.13701817	-0.615348	4.37675424	0.62413168	-6.9077553	4	72	4.47038889	300.222222	27.2222222	0	0	0	0	30.519848	0	0.21141735
0.396	4670.9239	0.69739585	144.089125	2.53675228	0	-0.9263411	1.54157096	-0.3589692	4.97043897	0.93127876	-6.9077553	5	63	6.25854444	301.333333	28.33333333	0	0	0	1	30.519848	0	0.21141735
0.6462	9542.7195	0.85637692	222.262323	3.04844479	0	-0.4366462	2.25588329	-0.1538776	5.40386282	1.11495954	-6.9077553	6	59	6.25854444	302.444444	29.44444444	0	0	0	1	30.519848	0	0.21141735
0.8856	13907.4663	0.72817224	144.594264	1.97359748	0	-0.1214899	2.63249774	-0.3158453	4.97393856	0.68036457	-6.9077553	7	55	4.91742778	304.111111	31.11111111	0	0	0	1	30.519848	0	0.21141735
1.1637	18604.1214	0.73404637	152.114963	1.96454193	0	0.15160458	2.92343689	-0.3078217	5.02464314	0.675768	-6.9077553	8	54	4.02335	305.222222	32.2222222	0	0	0	1	30.519848	0	0.21141735
1.395	27321.4134	0.72836375	154.152572	1.49097072	0	0.33289442	3.30770737	-0.3155827	5.03794932	0.40009787	-6.9077553	9	53	4.02335	305.777778	32.7777778	0	0	0	1	30.519848	0	0.21141735
1.5669	35234.9774	0.71801151	152.875859	1.32096663	0	0.44909915	3.56206765	-0.3298779	5.02963275	0.2791205	-6.9077553	10	59	4.47038889	304.111111	31.11111111	0	0	0	1	30.519848	0	0.21141735
1.6713	41919.1097	0.65874626	125.325835	0.99467074	0	0.51360177	3.73576566	-0.4159	4.83092501	-0.0043387	-6.9077553	11	59	4.47038889	304.111111	31.11111111	0	0	0	1	30.519848	0	0.21141735
1.7514	47897.4651	0.71522912	133.953344	0.91604014	0	0.56041547	3.86908346	-0.3337552	4.89749903	-0.086604	-6.9077553	12	63	3.57631111	302.444444	29.44444444	0	0	0	1	30.519848	0	0.21141735
1.8135	54280.9375	0.72818966	136.458631	0.97326395	0	0.59525868	3.99419153	-0.3158214	4.91602882	-0.026073	-6.9077553	13	69	1.78815556	301.333333	28.33333333	0	0	0	1	30.519848	0	0.21141735
1.8756	59509.272	0.688728	127.583079	0.97700191	0	0.62892861	4.08614894	-0.371458	4.84877559	-0.0222437	-6.9077553	14	69	2.68223333	300.777778	27.7777778	0	0	0	1	30.519848	0	0.21141735
1.9341	65268.0749	0.63784796	104.832121	0.86119297	0	0.6596421	4.17851834	-0.4480888	4.65236977	-0.1482762	-6.9077553	15	73	3.57631111	300.222222	27.2222222	0	0	0	1	30.519848	0	0.21141735
1.9674	67251.5586	0.57082006	83.603001	0.87306925	0	0.67671287	4.20845506	-0.5589309	4.42609138	-0.1345957	-6.9077553	16	75	3.57631111	299.666667	26.66666666	0	0	0	1	30.519848	0	0.21141735
1.98	67734.0607	0.25167741	14.0179716	0.55339302	0	0.68309684	4.21560393	-1.3756417	2.64041153	-0.5898814	-6.9077553	17	77	4.02335	299.111111	26.11111111	0	0	0	1	30.519848	0	0.21141735
1.9917	68260.4249	0.2571521	13.8549089	0.37443333	0	0.68898855	4.22334482	-1.3542063	2.62871177	-0.9796744	-6.9077553	18	79	1.78815556	298.555556	25.55555555	0	0	0	1	30.519848	0	0.21141735
2.0016	68655.2009	0.2122951	10.3051272	0.56470737	0	0.69394686	4.22911146	-1.5450786	2.3327386	-0.5696784	-6.9077553	19	80	1.78815556	298.555556	25.55555555	0	0	0	1	30.519848	0	0.21141735
2.0151	69225.4287	0.21281573	9.73221436	0.30614052	0	0.70066882	4.23738271	-1.5426407	2.2755442	-1.1804499	-6.9077553	20	83	1.34111667	298	25	0	0	0	1	30.519848	0	0.21141735
2.0205	69400.8852	0.23994867	11.7234472	0.36403795	0	0.70334501	4.23991403	-1.4231714	2.46167616	-1.007754	-6.9077553	21	86	0.89407778	297.444444	24.44444444	0	0	0	1	30.519848	0	0.21141735
2.0277	69664.0673	0.21975765	9.72532082	0.31163281	0	0.70690215	4.24369901	-1.5106898	2.2748357	-1.1627259	-6.9077553	22	87	1.34111667	297.444444	24.44444444	0	0	0	1	30.519848	0	0.21141735
2.0358	70014.9758	0.18602777	7.31632487	0.31700895	0	0.71088886	4.24872344	-1.6764982	1.99024481	-1.1456758	-6.9077553	23	90	2.23519444	296.888889	23.88888888	0	0	0	1	30.519848	0	0.21141735
0.0153	704.947684	0.94231623	249.075836	1.81922638	0	-4.1799025	-0.3482141	-0.0583537	5.51776143	0.59896088	-6.9077553	0	92	1.78815556	295.777778	22.7777778	0	0	0	0	34.0724638	0	0.49277013
0.0261	1055.85796	0.51482352	62.5890554	0.73811754	0	-3.64582	0.05530032	-0.6619906	4.13660641	-0.3022983	-6.9077553	1	90	2.68223333	296.888889	23.88888888	0	0	0	0	34.0724638	0	0.49277013
0.0531	1985.23239	0.58496946	97.2018717	1.28810145	0	-2.9355784	0.68623957	-0.5344876	4.57680026	0.25394543	-6.9077553	2	83	3.12927222	298.555556	25.55555555	0	0	0	0	34.0724638	0	0.49277013
0.1206	5207.87122	0.97773102	263.199818	1.99497843	0	-2.115276	1.65036318	-0.0214984	5.57291731	0.69113437	-6.9077553	3	77	4.47038889	299.111111	26.11111111	0	0	0	0	34.0724638	0	0.49277013
0.1836	8292.42161	0.89583713	213.455608	1.40657283	0	-1.6949958	2.11546262	-0.108881	5.36343357	0.34186683	-6.9077553	4	72	4.47038889	300.222222	27.2222222	0	0	0	0	34.0724638	0	0.49277013
0.3141	14371.4759	1.03213333	314.496794	2.6723938	0	-1.1580439	2.66531498	0.03259625	5.75097706	0.98334875	-6.9077553	5	63	6.25854444	301.333333	28.33333333	0	0	0	1	34.0724638	0	0.49277013
0.6273	30317.5072	1.3395726	534.60044	4.22856473	0	-0.4663304	3.41175832	0.29309684	6.2815215	1.44209909	-6.9077553	6	59	6.25854444	302.444444	29.44444444	0	0	0	1	34.0724638	0	0.49277013
1.2402	51596.7579	1.29567608	487.555984	4.33079513	0	0.21527266	3.94347822	0.25980413	6.18940717	1.46598204	-6.9077553	7	55	4.91742778	304.111111	31.11111111	0	0	0	1	34.0724638	0	0.49277013

1.791	67165.9562	1.12515568	372.73052	2.91295449	0	0.58277412	4.2071814	0.11880978	5.92085837	1.06951109	-6.9077553	8	54	4.02335	305.222222	32.2222222	0	0	0	1	34.0724638	0	0.49277013
2.3481	81692.9429	1.06444001	330.198329	2.73826114	0	0.85360649	4.40297986	0.06338787	5.7996965	1.00768823	-6.9077553	9	53	4.02335	305.777778	32.7777778	0	0	0	1	34.0724638	0	0.49277013
2.9511	100621.951	1.04226894	318.323134	2.53476289	1	1.08217798	4.61138038	0.042359	5.76307015	0.93049454	0.0009995	10	59	4.47038889	304.111111	31.11111111	0	0	0	1	34.0724638	0	0.49277013
3.4218	116606.359	0.92150599	238.841509	1.95843611	1	1.23016673	4.75881239	-0.0806614	5.47580438	0.67265673	0.0009995	11	59	4.47038889	304.111111	31.11111111	0	0	0	1	34.0724638	0	0.49277013
3.7035	129204.842	0.85892357	204.311714	1.50358762	1	1.30927832	4.86140681	-0.1509118	5.31965173	0.40851885	0.0009995	12	63	3.57631111	302.444444	29.44444444	0	0	0	1	34.0724638	0	0.49277013
3.9312	138865.487	0.87331285	207.004848	1.61315098	1	1.36894472	4.93351295	-0.134317	5.33274704	0.47880911	0.0009995	13	69	1.78815556	301.333333	28.33333333	0	0	0	1	34.0724638	0	0.49277013
4.068	144258.276	0.813435	179.660799	1.4991963	1	1.40315148	4.97161221	-0.2052607	5.19107619	0.40559597	0.0009995	14	69	2.68223333	300.777778	27.7777778	0	0	0	1	34.0724638	0	0.49277013
4.1562	147504.585	0.72242776	137.318897	1.20331862	1	1.4246012	4.99386604	-0.3237546	4.92231322	0.18591394	0.0009995	15	73	3.57631111	300.222222	27.2222222	0	0	0	1	34.0724638	0	0.49277013
4.2345	149698.759	0.62976219	113.61794	1.203921	1	1.44326526	5.00863168	-0.4608264	4.73285021	0.186414	0.0009995	16	75	3.57631111	299.666667	26.66666666	0	0	0	1	34.0724638	0	0.49277013
4.2903	150356.709	0.70191599	172.060106	1.7833674	1	1.45635666	5.01301718	-0.3525179	5.14784968	0.57906396	0.0009995	17	77	4.02335	299.111111	26.11111111	0	0	0	1	34.0724638	0	0.49277013
4.3137	151277.851	0.27226323	22.315324	0.52647631	1	1.461796	5.01912483	-1.2973197	3.10531843	-0.6396513	0.0009995	18	79	1.78815556	298.555556	25.55555555	0	0	0	1	34.0724638	0	0.49277013
4.3398	152155.119	0.3467253	50.2517006	0.69377386	1	1.46782826	5.02490709	-1.0563425	3.91706429	-0.3641689	0.0009995	19	80	1.78815556	298.555556	25.55555555	0	0	0	1	34.0724638	0	0.49277013
4.3605	152900.808	0.20333417	9.04819853	0.33602352	1	1.47258673	5.02979593	-1.5879986	2.20267619	-1.0876026	0.0009995	20	83	1.34111667	298	25	0	0	0	1	34.0724638	0	0.49277013
4.3884	153997.395	0.26095021	21.0677175	0.33746836	1	1.4789647	5.03694218	-1.3396008	3.04778936	-1.0833247	0.0009995	21	86	0.89407778	297.444444	24.44444444	0	0	0	1	34.0724638	0	0.49277013
4.4118	154962.404	0.24307341	17.6524177	0.3659873	1	1.48428277	5.04318898	-1.4102862	2.8709294	-1.002428	0.0009995	22	87	1.34111667	297.444444	24.44444444	0	0	0	1	34.0724638	0	0.49277013
4.4343	155795.819	0.2219545	17.1704749	0.33512871	1	1.48936977	5.04855271	-1.5007876	2.84324957	-1.0902611	0.0009995	23	90	2.23519444	296.888889	23.88888888	0	0	0	1	34.0724638	0	0.49277013
0.0279	1616.79106	1.0449192	297.637109	2.03319931	0	-3.5791286	0.48106167	0.04489612	5.69587835	0.71010228	-6.9077553	0	92	1.78815556	295.777778	22.7777778	0	0	0	0	42.288408	0	-0.4111751
0.0783	4133.14638	0.77824402	163.138777	1.55219323	0	-2.5472077	1.41928087	-0.249431	5.09460736	0.44031296	-6.9077553	1	90	2.68223333	296.888889	23.88888888	0	0	0	0	42.288408	0	-0.4111751
0.2196	9965.378	0.90068453	230.838749	2.50900191	0	-1.5159476	2.29921723	-0.1034906	5.44172374	0.92028351	-6.9077553	2	83	3.12927222	298.555556	25.55555555	0	0	0	0	42.288408	0	-0.4111751
0.5067	23481.7936	1.04577742	304.386266	2.88166336	0	-0.6798362	3.15626797	0.04571632	5.71830079	1.05871464	-6.9077553	3	77	4.47038889	299.111111	26.11111111	0	0	0	0	42.288408	0	-0.4111751
0.8046	36208.8975	0.92754076	232.704919	2.02328242	0	-0.21741	3.58933249	-0.074141	5.44977551	0.70521528	-6.9077553	4	72	4.47038889	300.222222	27.2222222	0	0	0	0	42.288408	0	-0.4111751
1.1979	50658.1492	0.93827558	244.781518	2.84132843	0	0.18057002	3.92511985	-0.0626464	5.50037014	1.04462358	-6.9077553	5	63	6.25854444	301.333333	28.33333333	0	0	1	0	42.288408	0	-0.4111751
1.7559	71543.1976	0.91082705	233.379688	2.57330618	0	0.56298155	4.27031541	-0.0923049	5.45267097	0.94558005	-6.9077553	6	59	6.25854444	302.444444	29.44444444	0	0	1	0	42.288408	0	-0.4111751
2.3265	94103.674	0.9205045	235.260351	2.31890465	0	0.84436499	4.54440772	-0.0817476	5.46069703	0.84152608	-6.9077553	7	55	4.91742778	304.111111	31.11111111	0	0	1	0	42.288408	0	-0.4111751
2.9529	126228.836	1.00018346	281.121992	2.00258277	0	1.08278774	4.83810434	0.00118276	5.63879227	0.69493696	-6.9077553	8	54	4.02335	305.222222	32.2222222	0	0	1	0	42.288408	0	-0.4111751
3.6846	163871.43	1.04828722	313.279501	2.45971364	0	1.30416197	5.09908826	0.04811109	5.74709896	0.90045141	-6.9077553	9	53	4.02335	305.777778	32.7777778	0	0	1	0	42.288408	0	-0.4111751
4.3398	192747.115	0.95894489	260.792497	2.30510351	0	1.46782826	5.26138424	-0.0408794	5.56372889	0.83555931	-6.9077553	10	59	4.47038889	304.111111	31.11111111	0	0	1	0	42.288408	0	-0.4111751
4.8384	213758.754	0.88665105	226.010196	1.7746083	0	1.57658409	5.36485274	-0.1191766	5.42058454	0.57414307	-6.9077553	11	59	4.47038889	304.111111	31.11111111	0	0	1	0	42.288408	0	-0.4111751
5.1903	230536.229	0.86388177	207.930507	1.45999028	0	1.6467915	5.44041236	-0.1451625	5.33720873	0.37911448	-6.9077553	12	63	3.57631111	302.444444	29.44444444	0	0	1	0	42.288408	0	-0.4111751
5.4387	241820.784	0.81456204	175.489413	1.31590691	0	1.69354006	5.48820103	-0.2038778	5.16758442	0.27528574	-6.9077553	13	69	1.78815556	301.333333	28.33333333	0	0	1	0	42.288408	0	-0.4111751
5.6565	251096.421	0.75387649	148.538837	1.2112027	0	1.73280533	5.52584099	-0.2812011	5.00085318	0.19243912	-6.9077553	14	69	2.68223333	300.777778	27.7777778	0	0	1	0	42.288408	0	-0.4111751
5.8536	259154.291	0.7300082	145.373965	1.24359047	0	1.76705686	5.55742746	-0.3133306	4.97931637	0.21880654	-6.9077553	15	73	3.57631111	300.222222	27.2222222	0	0	1	0	42.288408	0	-0.4111751
5.9652	263805.141	0.57614505	85.9278919	0.99325937	0	1.78594258	5.57521452	-0.5496617	4.45352012	-0.0057572	-6.9077553	16	75	3.57631111	299.666667	26.66666666	0	0	1	0	42.288408	0	-0.4111751
6.0084	265735.154	0.23329822	11.316625	0.36909707	0	1.79315849	5.58250391	-1.4511605	2.42636124	-0.9939899	-6.9077553	17	77	4.02335	299.111111	26.11111111	0	0	1	0	42.288408	0	-0.4111751
6.0507	267401.968	0.22965378	10.9537665	0.34975717	0	1.80017397	5.58875676	-1.4668375	2.39377466	-1.0476611	-6.9077553	18	79	1.78815556	298.555556	25.55555555	0	0	1	0	42.288408	0	-0.4111751
6.0858	268674.007	0.25652538	22.3613892	0.39033816	0	1.80595819	5.5935025	-1.356637	3.10738049	-0.9381832	-6.9077553	19	80	1.78815556	298.555556	25.55555555	0	0	1	0	42.288408	0	-0.4111751
6.1173	269814.473	0.26857477	26.2961077	0.49601724	0	1.81112082	5.59773829	-1.3109095	3.26945896	-0.6991306	-6.9077553	20	83	1.34111667	298	25	0	0	1	0	42.288408	0	-0.4111751
6.1443	270998.786	0.19743263	9.81162109	0.2981911	0	1.81552482	5.60211803	-1.6173056	2.28366942	-1.2066728	-6.9077553	21	86	0.89407778	297.444444	24.44444444	0	0	1	0	42.288408	0	-0.4111751
6.174	272314.728	0.17444613	6.33657328	0.34529394	0	1.82034693	5.60696216	-1.7404232	1.84649593	-1.0604673	-6.9077553	22	87	1.34111667	297.444444	24.44444444	0	0	1	0	42.288408	0	-0.4111751
6.2001	273542.919	0.17999378	6.82464968	0.25217998	0	1.82456542	5.61146219	-1.7092926	1.92068753	-1.3736546	-6.9077553	23	90	2.23519444	296.888889	23.88888888	0	0	1	0	42.288408	0	-0.4111751
0.0018	0	0.1506348	4.24074936	0.4177871	0	-6.3199686	-6.9077553	-1.8862803	1.44497577	-0.8703926	-6.9077553	0	92	1.78815556	295.777778	22.7777778	0	0	0	0	18.9135952	0	-0.0428739
0.0018	0	0	0	0	0	-6.3199686	-6.9077553	-6.9077553	-6.9077553	-6.9077553	-6.9077553	1	90	2.68223333	296.888889	23.88888888	0	0	0	0	18.9135952	0	-0.0428739
0.0117	0	0.34732021	32.2107516	1.03138984	0	-4.4481664	-6.9077553	-1.0546331	3.47233134	0.03187635	-6.9077553	2	83	3.12927222	298.555556	25.55555555	0	0	0	0	18.9135952	0	-0.0428739
0.0693	143.021072	0.65984416	123.984703	2.45380282	0	-2.6693104	-1.9377957	-0.4142372	4.82016626	0.89804644	-6.9077553	3	77	4.47038889	299.111111	26.11111111	0	0	0	0	18.9135952	0	-0.0428739
0.2079	1899.19458	0.68162304	141.836901	2.28002454	0	-1.5706981	0.64195629	-0.3818125	4.95468487	0.8246247	-6.9077553	4	72	4.47038889	300.222222	27.2222222	0	0	1	0	18.9135952	0	-0.0428739
0.477	11784.0083	0.93303127	293.478418	3.00279169	0	-0.7402388	2.46682824	-0.0682454	5.68180751	1.09987539	-6.9077553	5	63	6.25854444	301.333333	28.33333333	0	0	1	0	18.9135952	0	-0.0428739
0.6921	18825.4978	0.87106304	265.581933	2.23115623	0	-0.3680248	2.93526534	-0.1368936	5.58192716	0.80296804	-6.9077553	6	59	6.25854444	302.444444	29.44444444	0	0	1	0	18.9135952	0	-0.0428739
0.9729	27640.0453	0.86184908	231.651542	2.29248575	0	-0.027474	3.31930182	-0.1475155	5.44523859	0.83007282	-6.9077553	7	55	4.91742778	304.111111	31.11111111	0	0	1	0	18.9135952	0	-0.0428739
1.3239	39755.928	0.88525062	232.761098	2.25220572	0	0.28058193	3.68278412	-0.1207555	5.45001689	0.81235397	-6.9077553	8	54	4.02335	305.222222	32.2222222	0	0	1	0	18.9135952	0	-0.0428739
1.6497	52721.4004	0.88427903	231.102879	1.91298174	0	0.50059345	3.96504042	-0.1218524	5.4428673	0.64918575	-6.9077553	9	53	4.02335	305.777778	32.7777778	0	0	1	0	18.9135952	0	-0.0428739
2.0466	69234.8323	0.91513497	245.599738	2.0927405	0	0.71617988	4.23751854	-0.0875916	5.5037072	0.73895218	-6.9077553	10	59	4.47038889	304.111111	31.11111111	0	0	1	0	18.9135952	0	-0.0428739
2.4435	88914.8609	0.96598543	273.388659	2.0255837	0	0.89343144	4.48769054	-0.0335719	5.6108981	0.70635147	-6.9077553	11	59	4.47038889	304.111111	31.11111111	0	0	1	0	18.9135952	0	-0.0428739
2.6775	100546.057	0.91363267	230.795162	1.64854783	0	0.98488352	4.61062585	-0.0892327	5.44153491	0.50050121	-6.9077553	12	63	3.57631111	302.444444	29.44444444	0	0	1	0	18.9135952	0	-0.0428739
2.8755	109849.497	0.85839969	202.418537	1.56116666	0	1.05622657	4.69912032	-0.1515212	5.31034246	0.44607374	-6.9077553	13	69	1.78815556	301.333333	28.33333333	0	0	1	0	18.9135952	0	-0.0428739
3.033	116964.141	0.749582	148.399955	1.27637068	0	1.10955223	4.76187595	-0.2869064	4.99991777	0.24480381	-6.9077553	14	69	2.68223333	300.777778	27.7777778	0	0	1	0	18.9135952	0	-0.0428739
3.2175	125749.076	0.84149721	191.792912	1.63258563	0	1.16860466	4.83429641	-0.1713849	5.25642142	0.49077737	-6.9077553	15	73	3.57631111	300.222222	27.2222222	0	0	1	0	18.9135952	0	-0.0428739
3.3327	130970.106	0.64253348	108.962936	1.20934784	0	1.20378279	4.87497673	-0.4407812	4.69101697	0.19090779	-6.9077553	16	75	3.57631111	299.666667	26.66666666	0	0	1	0	18.9135952	0	-0.0428739
3.375	132768.517	0.2782917	17.4196232	0.50695736	0	1.21639532	4.88861467	-1.2754985	2.85765475	-0.6773578	-6.9077553	17	77	4.02335	299.111111	26.11111111	0	0	1	0	18.9135952	0	-0.0428739
3.4353	135358.73	0.29139089	22.292455	0.50050331	0	1.23410426	4.9079359	-1.2296637	3.10429314	-0.6901451	-6.9077553	18	79	1.78815556	298.555556	25.55555555	0	0	1	0	18.9135952	0	-0.0428739
3.4704	136806.231	0.2467759	13.1987023	0.42122992	0	1.24426986	4.91857286	-1.3952306	2.58019428	-0.8622053	-6.9077553	19	80	1.78815556	298.555556	25.55555555	0	0	1	0	18.9135952	0	-0.0428739
3.5055	138078.285	0.22941523	11.7932434	0.41908416	0	1.25433316	4.92782805	-1.4678722	2.46761157	-0.8673002	-6.9077553	20	83	1.34111667	298	25	0	0	1	0	18.9135952	0	-0.0428739
3.528	139043.291	0.2331398	11.667373	0.35994347	0	1.26073114	4.93479252	-1.4518369	2.45688202	-1.0190339	-6.9077553	21	86	0.89407778	297.444444	24.44444444	0	0	1	0	18.9135952	0	-0.0428739

3.5514	139876.696	0.25621865	19.202598	0.54029751	0	1.26734189	4.94076844	-1.3578288	2.95509766	-0.6137862	-6.9077553	22	87	1.34111667	297.444444	24.44444444	0	0	1	0	18.9135952	0	-0.0428739
3.5685	140666.248	0.20318887	8.9591723	0.30456044	0	1.27214534	4.94639716	-1.5887099	2.19278946	-1.1856077	-6.9077553	23	90	2.23519444	296.888889	23.88888888	0	0	1	0	18.9135952	0	-0.0428739
0.0081	322.159322	0.83546883	189.192535	1.75908506	0	-4.8158912	-1.1296098	-0.178566	5.24277049	0.56536214	-6.9077553	0	85	4.47038889	295.222222	22.2222222	0	0	0	0	30.519848	0	0.21141735
0.0117	497.614491	0.39771649	37.3409882	0.61284208	0	-4.4481664	-0.695922	-0.9195047	3.62011838	-0.4880176	-6.9077553	1	89	5.36446667	294.666667	21.66666666	0	0	0	0	30.519848	0	0.21141735
0.018	760.797228	0.51905009	63.542511	0.74314117	0	-4.0173835	-0.2720749	-0.6538302	4.15172488	-0.2955245	-6.9077553	2	90	4.47038889	294.111111	21.11111111	0	0	0	0	30.519848	0	0.21141735
0.0297	1155.57122	0.52748669	64.8990385	0.78952965	0	-3.5166082	0.14545978	-0.6377377	4.17284822	-0.2350521	-6.9077553	3	94	3.57631111	293.555556	20.55555555	0	0	0	0	30.519848	0	0.21141735
0.0351	1287.16264	0.4662447	50.2057495	0.79712828	0	-3.3495541	0.25321689	-0.7609022	3.91614947	-0.2254859	-6.9077553	4	97	3.12927222	293	20	0	0	0	0	30.519848	0	0.21141735
0.0351	1287.16264	0	0	0	0	-3.3495541	0.25321689	-6.9077553	-6.9077553	-6.9077553	-6.9077553	5	97	2.68223333	293	20	0	0	0	0	30.519848	0	0.21141735
0.0378	1331.02635	0.32680166	23.5641505	0.58644136	0	-3.2754462	0.28670135	-1.1153466	3.15976895	-0.5319789	-6.9077553	6	93	4.47038889	293.555556	20.55555555	0	0	0	0	30.519848	0	0.21141735
0.0378	1331.02635	0	0	0	0	-3.2754462	0.28670135	-6.9077553	-6.9077553	-6.9077553	-6.9077553	7	92	2.68223333	293.555556	20.55555555	0	0	0	0	30.519848	0	0.21141735
0.0378	1331.02635	0	0	0	0	-3.2754462	0.28670135	-6.9077553	-6.9077553	-6.9077553	-6.9077553	8	91	2.68223333	294.111111	21.11111111	0	0	0	0	30.519848	0	0.21141735
0.0648	1638.07289	0.23164392	13.3932332	1.14992383	0	-2.7364497	0.49413077	-1.4582463	2.59482426	0.14056495	-6.9077553	9	91	2.68223333	294.666667	21.66666666	0	0	0	0	30.519848	0	0.21141735
0.1116	1959.62316	0.35597156	39.9100342	1.17245806	0	-2.1928342	0.67326236	-1.0300992	3.68665283	0.159955	-6.9077553	10	85	3.57631111	296.333333	23.33333333	0	0	0	0	30.519848	0	0.21141735
0.2502	3083.46917	0.59848558	105.126471	2.64523751	0	-1.3854947	1.12637957	-0.5116834	4.65517363	0.97313883	-6.9077553	11	71	5.36446667	298.555556	25.55555555	0	0	0	0	30.519848	0	0.21141735
0.3609	3970.69476	0.62943975	108.414332	2.22614865	0	-1.0191544	1.3791929	-0.4613377	4.68596952	0.80072214	-6.9077553	12	67	5.81150556	299.666667	26.66666666	0	0	0	1	30.519848	0	0.21141735
0.5454	8635.66073	0.73642801	158.047485	2.22674352	0	-0.6062358	2.15601602	-0.3045868	5.06290185	0.8009892	-6.9077553	13	55	6.25854444	301.888889	28.88888888	0	0	0	1	30.519848	0	0.21141735
0.8424	19253.7604	0.8513892	218.623248	2.86792529	0	-0.1715003	2.95775832	-0.1597121	5.38735449	1.05393749	-6.9077553	14	50	5.81150556	303.555556	30.55555555	0	0	0	1	30.519848	0	0.21141735
1.1421	27774.425	0.72447108	145.093985	2.20729739	0	0.13286867	3.32415164	-0.3209341	4.97738859	0.7922218	-6.9077553	15	45	5.36446667	304.111111	31.11111111	0	0	0	1	30.519848	0	0.21141735
1.4031	37112.4792	0.68966733	132.540814	1.53715515	0	0.33868407	3.61398022	-0.370097	4.88689817	0.43058374	-6.9077553	16	46	3.57631111	304.111111	31.11111111	0	0	0	1	30.519848	0	0.21141735
1.6362	48220.7214	0.72334437	151.200139	1.36020852	0	0.49237648	3.87580957	-0.3224884	5.018611	0.30837292	-6.9077553	17	44	4.02335	304.666667	31.66666666	0	0	0	1	30.519848	0	0.21141735
1.8135	57467.9135	0.69751875	138.322811	1.12297081	0	0.59525868	4.05124417	-0.3587933	4.92959739	0.11686778	-6.9077553	18	49	3.12927222	303	30	0	0	0	1	30.519848	0	0.21141735
1.9395	66760.2254	0.66522642	125.929464	0.90862296	0	0.66243021	4.20112245	-0.4061257	4.83572988	-0.0947251	-6.9077553	19	53	3.12927222	301.333333	28.33333333	0	0	0	1	30.519848	0	0.21141735
2.0502	76350.9743	0.70219316	139.798749	0.95702709	0	0.71793735	4.33535389	-0.3521237	4.94021103	-0.0428792	-6.9077553	20	58	1.78815556	299.111111	26.11111111	0	0	0	1	30.519848	0	0.21141735
2.1609	85520.5391	0.67438954	132.680775	0.95088723	0	0.7705248	4.44876826	-0.3924657	4.88795359	-0.0493087	-6.9077553	21	51	2.68223333	298.555556	25.55555555	0	0	0	1	30.519848	0	0.21141735
2.304	96911.8869	0.65385754	124.734486	0.95555057	0	0.83464674	4.5738125	-0.4233376	4.82619538	-0.0444216	-6.9077553	22	53	2.23519444	297.444444	24.44444444	0	0	0	1	30.519848	0	0.21141735
2.3967	106175.434	0.7140317	147.840014	0.98999283	0	0.87409279	4.66510218	-0.3354284	4.99613747	-0.009048	-6.9077553	23	62	2.68223333	296.333333	23.33333333	0	0	0	1	30.519848	0	0.21141735
0.0171	753.86788	0.99250889	274.549127	2.06497765	0	-4.0686768	-0.2812125	-0.0065123	5.61513385	0.72560355	-6.9077553	0	85	4.47038889	295.222222	22.2222222	0	0	0	0	34.0724638	0	0.49277013
0.0324	1367.9608	0.47841908	59.5126917	0.95091393	0	-3.4295969	0.31405192	-0.7351802	4.0862064	-0.0492807	-6.9077553	1	89	5.36446667	294.666667	21.66666666	0	0	0	0	34.0724638	0	0.49277013
0.0486	1850.46228	0.4658725	50.7576057	0.66177013	0	-3.0241317	0.61597575	-0.7616991	3.92708117	-0.4113271	-6.9077553	2	90	4.47038889	294.111111	21.11111111	0	0	0	0	34.0724638	0	0.49277013
0.0639	2464.5552	0.50788717	62.5330523	0.79802603	0	-2.7504359	0.90241702	-0.675529	4.13571125	-0.2243618	-6.9077553	3	94	3.57631111	293.555556	20.55555555	0	0	0	0	34.0724638	0	0.49277013
0.0828	3341.83096	0.52152034	64.9456729	0.81001218	0	-2.4913272	1.20681804	-0.6490914	4.17356652	-0.2094722	-6.9077553	4	97	3.12927222	293	20	0	0	0	0	34.0724638	0	0.49277013
0.1017	4219.1067	0.47865413	54.3249628	0.74524825	0	-2.285728	1.43986041	-0.73469	3.99500225	-0.292697	-6.9077553	5	97	2.68223333	293	20	0	0	0	0	34.0724638	0	0.49277013
0.1233	5184.10972	0.49273467	58.3023936	0.73995622	0	-2.0931349	1.645791	-0.705757	4.0656603	-0.2998137	-6.9077553	6	93	4.47038889	293.555556	20.55555555	0	0	0	0	34.0724638	0	0.49277013
0.1512	6412.29576	0.45966056	50.5564871	0.72525997	0	-1.8891518	1.8583733	-0.7750938	3.92311105	-0.3198472	-6.9077553	7	92	2.68223333	293.555556	20.55555555	0	0	0	0	34.0724638	0	0.49277013
0.1971	8524.05174	0.59967067	95.7659409	0.98599901	0	-1.6240441	2.14300909	-0.5097085	4.56191754	-0.0130862	-6.9077553	8	91	2.68223333	294.111111	21.11111111	0	0	0	0	34.0724638	0	0.49277013
0.3366	13953.974	0.85388331	213.640071	1.8884555	0	-1.08886	2.635836	-0.1567903	5.36429737	0.63628869	-6.9077553	9	91	2.68223333	294.666667	21.66666666	0	0	0	0	34.0724638	0	0.49277013
0.5697	21683.5922	0.9619044	287.817326	2.53588561	0	-0.5626454	3.07660197	-0.0378011	5.66232947	0.93093719	-6.9077553	10	85	3.57631111	296.333333	23.33333333	0	0	0	0	34.0724638	0	0.49277013
1.1484	33641.5218	1.29363416	513.721374	4.42970762	0	0.13836967	3.5157908	0.25822815	6.24168299	1.48855931	-6.9077553	11	71	5.36446667	298.555556	25.55555555	0	0	0	1	34.0724638	0	0.49277013
1.9467	53506.4913	1.32661749	543.343116	4.60738169	1	0.66613563	3.97982167	0.28338598	6.29774285	1.52787675	0.0009995	12	67	5.81150556	299.666667	26.66666666	0	0	0	1	34.0724638	0	0.49277013
2.5749	73685.9953	1.09116886	349.593705	2.87137532	2	0.9458107	4.29982633	0.0881655	5.8567745	1.05513932	0.69364706	13	55	6.25854444	301.888889	28.88888888	0	0	0	1	34.0724638	0	0.49277013
3.141	93084.9201	0.98556232	282.659728	2.26443574	2	1.14454122	4.53352294	-0.0135288	5.64424734	0.81776712	0.69364706	14	50	5.81150556	303.555556	30.55555555	0	0	0	1	34.0724638	0	0.49277013
3.8448	118472.511	1.05795671	320.611453	3.35187283	2	1.34672159	4.7746894	0.05728418	5.77023308	1.20981754	0.69364706	15	45	5.36446667	304.111111	31.11111111	0	0	0	1	34.0724638	0	0.49277013
4.5873	139515.154	0.88087432	218.964716	2.53346976	2	1.52329162	4.93818039	-0.1257057	5.38891517	0.92998445	0.69364706	16	46	3.57631111	304.111111	31.11111111	0	0	0	1	34.0724638	0	0.49277013
5.2182	156095.549	0.85766993	217.04984	2.11154667	2	1.65215251	5.05047472	-0.1523707	5.38013161	0.74789417	0.69364706	17	44	4.02335	304.666667	31.66666666	0	0	0	1	34.0724638	0	0.49277013
5.7357	170656.99	0.81480639	195.507038	1.95172554	2	1.7467098	5.13966149	-0.2035782	5.27560149	0.66922611	0.69364706	18	49	3.12927222	303	30	0	0	0	1	34.0724638	0	0.49277013
6.1209	183376.882	0.70622688	149.411553	1.25324171	2	1.81170914	5.21154895	-0.3464038	5.00671129	0.22653117	0.69364706	19	53	3.12927222	301.333333	28.33333333	0	0	0	1	34.0724638	0	0.49277013
6.4809	197879.91	0.8442659	205.257266	1.49701996	2	1.86885939	5.28766538	-0.168104	5.32426902	0.40414421	0.69364706	20	58	1.78815556	299.111111	26.11111111	0	0	0	1	34.0724638	0	0.49277013
6.8049	207218.827	0.62568626	125.476204	1.1496816	2	1.91764294	5.3337802	-0.4673092	4.8321241	0.14035446	0.69364706	21	51	2.68223333	298.555556	25.55555555	0	0	0	1	34.0724638	0	0.49277013
7.2297	218563.08	0.58145012	127.009997	1.21521436	3	1.97819754	5.38707924	-0.5405117	4.84427368	0.19574305	1.09894557	22	53	2.23519444	297.444444	24.44444444	0	0	0	1	34.0724638	0	0.49277013
7.4862	229396.545	0.76304792	170.967791	1.27413351	3	2.01306133	5.4354565	-0.2691248	5.14148103	0.24305089	1.09894557	23	62	2.68223333	296.333333	23.33333333	0	0	0	1	34.0724638	0	0.49277013
0.0369	2409.12633	1.13301277	358.47876	2.49469204	0	-3.2995437	0.87967917	0.12576247	5.8818722	0.91456606	-6.9077553	0	85	4.47038889	295.222222	22.2222222	0	0	0	0	42.288408	0	-0.4111751
0.0837	4655.54385	0.68563945	125.416307	1.43145444	0	-2.4805163	1.53827351	-0.3759459	4.83164663	0.35938936	-6.9077553	1	89	5.36446667	294.666667	21.66666666	0	0	0	0	42.288408	0	-0.4111751
0.1341	7111.91614	0.59391969	89.5088937	1.14690072	0	-2.0091695	1.96191231	-0.5193289	4.49434916	0.13793482	-6.9077553	2	90	4.47038889	294.111111	21.11111111	0	0	0	0	42.288408	0	-0.4111751
0.1836	9480.56112	0.56218189	78.6140803	1.04103574	0	-1.6949958	2.24934898	-0.5741526	4.36456354	0.04117624	-6.9077553	3	94	3.57631111	293.555556	20.55555555	0	0	0	0	42.288408	0	-0.4111751
0.234	11893.0687	0.55121606	75.1620571	1.03452526	0	-1.4524342	2.47603985	-0.5938159	4.31965985	0.03490879	-6.9077553	4	97	3.12927222	293	20	0	0	0	0	42.288408	0	-0.4111751
0.3006	15007.3962	0.54985134	74.9226536	1.02096599	0	-1.2019748	2.70860979	-0.5962903	4.31646964	0.02172821	-6.9077553	5	97	2.68223333	293	20	0	0	0	0	42.288408	0	-0.4111751
0.3681	17990.1339	0.54051854	70.6097266	0.93441355	0	-0.9994006	2.88987908	-0.613378	4.25718207	-0.0667665	-6.9077553	6	93	4.47038889	293.555556	20.55555555	0	0	0	0	42.288408	0	-0.4111751
0.4293	20753.5547	0.49150181	59.8483851	0.82678924	0	-0.8455993	3.03276573	-0.7082571	4.09183116	-0.1889967	-6.9077553	7	92	2.68223333	293.555556	20.55555555	0	0	0	0	42.288408	0	-0.4111751
0.5058	24401.9223	0.58228271	88.1504653	0.98282713	0	-0.6816139	3.19470289	-0.5390833	4.47905653	-0.0163051	-6.9077553	8	91	2.68223333	294.111111	21.11111111	0	0	1	0	42.288408	0	-0.4111751
0.6966	30939.1458	0.62541379	116.604879	1.47032482	0	-0.3615439	3.43205456	-0.4677441	4.75879969	0.38616323	-6.9077553	9	91	2.68223333	294.666667	21.66666666	0	0	1	0	42.288408	0	-0.4111751
0.9207	38637.7806	0.74528577	155.686935	1.57777105	0	-0.082621	3.65425645	-0.2926467	5.04785359	0.45664672	-6.9077553	10	85	3.57631111	296.333333	23.33333333	0	0	1	0	42.288408	0	-0.4111751
1.3266	52071.6288	0.83541342	190.890625	2.26584167	0	0.28261928	3.95263945	-0.1786323	5.25170586	0.81838753	-6.9077553	11	71	5.36446667	298.555556	25.55555555	0	0	1	0	42.288408	0	-0.4111751

1.7496	67479.1713	0.85160029	193.599967	1.91264051	0	0.55938719	4.2118338	-0.1594644	5.26579917	0.64900745	-6.9077553	12	67	5.81150556	299.666667	26.66666666	0	0	1	0	42.288408	0	-0.4111751
2.3481	91357.8906	0.91464293	239.7625	2.41375852	0	0.85360649	4.5147956	-0.0881288	5.47965302	0.88159929	-6.9077553	13	55	6.25854444	301.888889	28.88888888	0	0	1	0	42.288408	0	-0.4111751
3.1437	123903.253	1.0160568	297.919895	3.09090317	0	1.14540045	4.81950911	0.01691297	5.696828	1.12878681	-6.9077553	14	50	5.81150556	303.555556	30.55555555	0	0	1	0	42.288408	0	-0.4111751
4.0392	171027.726	1.06424873	328.301539	2.47686406	0	1.39604665	5.14183153	0.06320832	5.79393556	0.90739692	-6.9077553	15	45	5.36446667	304.111111	31.11111111	0	0	1	0	42.288408	0	-0.4111751
4.8861	210970.789	1.01789874	304.364107	2.40048506	0	1.58639444	5.35172442	0.01872238	5.71822799	0.87608732	-6.9077553	16	46	3.57631111	304.111111	31.11111111	0	0	1	0	42.288408	0	-0.4111751
5.4774	233466.132	0.85922097	211.607496	1.85809131	0	1.70063054	5.4530413	-0.150566	5.35473785	0.62008783	-6.9077553	17	44	4.02335	304.666667	31.66666666	0	0	1	0	42.288408	0	-0.4111751
5.9427	247893.496	0.75864486	164.798537	1.40463475	0	1.78216358	5.51300323	-0.2749042	5.10472981	0.34048898	-6.9077553	18	49	3.12927222	303	30	0	0	1	0	42.288408	0	-0.4111751
6.3036	259836.982	0.71100401	155.854933	1.23836222	0	1.8411209	5.56005829	-0.3396717	5.04893207	0.21459691	-6.9077553	19	53	3.12927222	301.333333	28.33333333	0	0	1	0	42.288408	0	-0.4111751
6.5628	269350.816	0.70957226	154.395264	1.12418143	0	1.88141734	5.59601839	-0.3416846	5.03952244	0.11794429	-6.9077553	20	58	1.78815556	299.111111	26.11111111	0	0	1	0	42.288408	0	-0.4111751
6.8022	277724.475	0.69567114	149.187823	1.11631626	0	1.91724609	5.62663313	-0.3614418	5.00521277	0.11092961	-6.9077553	21	51	2.68223333	298.555556	25.55555555	0	0	1	0	42.288408	0	-0.4111751
7.0866	285216.704	0.55863788	105.32904	0.88160474	0	1.95820568	5.65325276	-0.5804653	4.65709866	-0.1248778	-6.9077553	22	53	2.23519444	297.444444	24.44444444	0	0	1	0	42.288408	0	-0.4111751
7.245	291289.24	0.69710584	140.089078	1.01830942	0	1.98031158	5.67432016	-0.3593846	4.94228563	0.01912536	-6.9077553	23	62	2.68223333	296.333333	23.33333333	0	0	1	0	42.288408	0	-0.4111751
0.0018	0	0.45023254	45.8314285	1.99160612	0	-6.3199686	-6.9077553	-0.7957725	3.82499189	0.68944339	-6.9077553	0	85	4.47038889	295.222222	22.2222222	0	0	0	0	18.9135952	0	-0.0428739
0.0018	0	0	0	0	0	-6.3199686	-6.9077553	-6.9077553	-6.9077553	-6.9077553	-6.9077553	1	89	5.36446667	294.666667	21.66666666	0	0	0	0	18.9135952	0	-0.0428739
0.0018	0	0	0	0	0	-6.3199686	-6.9077553	-6.9077553	-6.9077553	-6.9077553	-6.9077553	2	90	4.47038889	294.111111	21.11111111	0	0	0	0	18.9135952	0	-0.0428739
0.0018	0	0	0	0	0	-6.3199686	-6.9077553	-6.9077553	-6.9077553	-6.9077553	-6.9077553	3	94	3.57631111	293.555556	20.55555555	0	0	0	0	18.9135952	0	-0.0428739
0.0018	0	0	0	0	0	-6.3199686	-6.9077553	-6.9077553	-6.9077553	-6.9077553	-6.9077553	4	97	3.12927222	293	20	0	0	0	0	18.9135952	0	-0.0428739
0.0018	0	0	0	0	0	-6.3199686	-6.9077553	-6.9077553	-6.9077553	-6.9077553	-6.9077553	5	97	2.68223333	293	20	0	0	0	0	18.9135952	0	-0.0428739
0.0018	0	0	0	0	0	-6.3199686	-6.9077553	-6.9077553	-6.9077553	-6.9077553	-6.9077553	6	93	4.47038889	293.555556	20.55555555	0	0	0	0	18.9135952	0	-0.0428739
0.0018	0	0	0	0	0	-6.3199686	-6.9077553	-6.9077553	-6.9077553	-6.9077553	-6.9077553	7	92	2.68223333	293.555556	20.55555555	0	0	0	0	18.9135952	0	-0.0428739
0.0018	0	0	0	0	0	-6.3199686	-6.9077553	-6.9077553	-6.9077553	-6.9077553	-6.9077553	8	91	2.68223333	294.111111	21.11111111	0	0	0	0	18.9135952	0	-0.0428739
0.0018	0	0	0	0	0	-6.3199686	-6.9077553	-6.9077553	-6.9077553	-6.9077553	-6.9077553	9	91	2.68223333	294.666667	21.66666666	0	0	0	0	18.9135952	0	-0.0428739
0.0072	0	0.27243833	21.7677333	1.03961921	0	-4.9336743	-6.9077553	-1.2966792	3.08047469	0.03981593	-6.9077553	10	85	3.57631111	296.333333	23.33333333	0	0	0	0	18.9135952	0	-0.0428739
0.0576	278.021041	0.61070088	106.097796	2.35984394	0	-2.8542327	-1.2764681	-0.4915119	4.6643707	0.85901915	-6.9077553	11	71	5.36446667	298.555556	25.55555555	0	0	0	0	18.9135952	0	-0.0428739
0.2331	3336.01494	0.78996003	202.764042	2.94423202	0	-1.4562877	1.20507668	-0.2345078	5.31204788	1.0801876	-6.9077553	12	67	5.81150556	299.666667	26.66666666	0	0	1	0	18.9135952	0	-0.0428739
0.5076	13243.5677	0.91798196	285.870133	2.8168385	0	-0.6780615	2.58358748	-0.0844888	5.65554113	1.0359701	-6.9077553	13	55	6.25854444	301.888889	28.88888888	0	0	1	0	18.9135952	0	-0.0428739
0.7029	18349.6963	0.77077586	198.726617	1.88487307	0	-0.3525406	2.90966752	-0.2590611	5.29193513	0.63439088	-6.9077553	14	50	5.81150556	303.555556	30.55555555	0	0	1	0	18.9135952	0	-0.0428739
0.9612	27264.5004	0.88141098	247.620481	2.20355863	0	-0.0395728	3.30562218	-0.1250974	5.5119013	0.79052732	-6.9077553	15	45	5.36446667	304.111111	31.11111111	0	0	1	0	18.9135952	0	-0.0428739
1.2888	38226.0433	0.86062924	222.797261	2.18966457	0	0.25371155	3.6435432	-0.1489302	5.4062667	0.78420495	-6.9077553	16	46	3.57631111	304.111111	31.11111111	0	0	1	0	18.9135952	0	-0.0428739
1.6011	51078.1437	0.902841	240.396299	1.90826821	0	0.47069089	3.93337627	-0.1011018	5.48229297	0.64672003	-6.9077553	17	44	4.02335	304.666667	31.66666666	0	0	1	0	18.9135952	0	-0.0428739
1.8693	61369.1964	0.81873695	194.954803	1.57420994	0	0.62556403	4.11692432	-0.1987718	5.27277288	0.45438856	-6.9077553	18	49	3.12927222	303	30	0	0	1	0	18.9135952	0	-0.0428739
2.2185	76210.6788	0.87437722	227.486308	1.78030742	0	0.79683129	4.33351472	-0.1331004	5.42709445	0.5773476	-6.9077553	19	53	3.12927222	301.333333	28.33333333	0	0	1	0	18.9135952	0	-0.0428739
2.5119	89761.7113	0.90323157	231.219014	1.64134731	0	0.92103944	4.49716965	-0.1006698	5.4433697	0.49612651	-6.9077553	20	58	1.78815556	299.111111	26.11111111	0	0	1	0	18.9135952	0	-0.0428739
2.7666	99976.5117	0.77723004	180.174636	1.43589745	0	1.01761913	4.60494528	-0.2507331	5.19393213	0.36248624	-6.9077553	21	51	2.68223333	298.555556	25.55555555	0	0	1	0	18.9135952	0	-0.0428739
3.0888	111630.408	0.76018008	179.817857	1.60560241	0	1.12778267	4.71520244	-0.2728853	5.19195	0.47412165	-6.9077553	22	53	2.23519444	297.444444	24.44444444	0	0	1	0	18.9135952	0	-0.0428739
3.3156	122140.698	0.8709629	212.026135	1.57853069	0	1.1986386	4.80518183	-0.1370084	5.35671426	0.45712777	-6.9077553	23	62	2.68223333	296.333333	23.33333333	0	0	1	0	18.9135952	0	-0.0428739
0.0198	469.48091	0.7293391	151.441313	1.88625354	0	-3.9220733	-0.7539999	-0.3142463	5.02020478	0.63512262	-6.9077553	0	82	4.91742778	299.666667	26.66666666	0	0	0	0	30.519848	1	0.21141735
0.0702	1039.81451	0.45857818	57.8504028	1.71447318	0	-2.656407	0.04000358	-0.7774462	4.0578777	0.53968895	-6.9077553	1	74	5.36446667	301.333333	28.33333333	0	0	0	0	30.519848	1	0.21141735
0.1395	2138.09619	0.57207439	93.336838	1.54621857	0	-1.9696907	0.7603834	-0.5567397	4.53622558	0.43645885	-6.9077553	2	68	5.36446667	302.444444	29.44444444	0	0	0	0	30.519848	1	0.21141735
0.2304	2954.21524	0.56175379	91.6266631	2.00673094	0	-1.4679384	1.08357149	-0.5749131	4.51773322	0.6970052	-6.9077553	3	63	5.81150556	303.555556	30.55555555	0	0	0	0	30.519848	1	0.21141735
0.3645	3959.29543	0.71144022	147.948105	2.87760362	0	-1.0092287	1.37631863	-0.3390593	4.99686833	1.05730532	-6.9077553	4	60	4.91742778	304.666667	31.66666666	0	0	0	1	30.519848	1	0.21141735
0.5463	7074.07536	0.77807119	175.954015	2.28711315	0	-0.604587	1.95657809	-0.2496529	5.17022836	0.82772753	-6.9077553	5	55	4.91742778	306.888889	33.88888888	0	0	0	1	30.519848	1	0.21141735
0.8559	17197.6215	0.84073435	210.383766	3.06924864	0	-0.1556017	2.84482923	-0.1722908	5.34893807	1.12175855	-6.9077553	6	51	5.81150556	307.444444	34.4444444	0	0	0	1	30.519848	1	0.21141735
1.179	26840.6113	0.71403822	140.120582	1.94565848	0	0.16466662	3.28995334	-0.3354193	4.94251049	0.6661143	-6.9077553	7	49	5.36446667	308.555556	35.5555556	0	0	0	1	30.519848	1	0.21141735
1.4373	35641.3061	0.69162663	133.356435	1.57729858	0	0.36276635	3.57353331	-0.3672642	4.89303301	0.45634742	-6.9077553	8	52	4.47038889	306.888889	33.88888888	0	0	0	1	30.519848	1	0.21141735
1.6497	45251.1925	0.69390462	136.132134	1.35722144	0	0.50059345	3.81225112	-0.3639807	4.91363333	0.30617608	-6.9077553	9	52	4.47038889	307.444444	34.4444444	0	0	0	1	30.519848	1	0.21141735
1.8198	55608.3477	0.65638285	122.990916	1.0467363	0	0.5987266	4.01835131	-0.4194887	4.81211863	0.04663193	-6.9077553	10	54	3.57631111	306.888889	33.88888888	0	0	0	1	30.519848	1	0.21141735
1.9395	64587.3839	0.66107866	122.434769	0.89464856	0	0.66243021	4.16803458	-0.4123709	4.80758655	-0.1102072	-6.9077553	11	58	2.68223333	305.777778	32.7777778	0	0	0	1	30.519848	1	0.21141735
2.0304	74357.068	0.67400149	121.150942	0.78880401	0	0.70823282	4.30889218	-0.3930404	4.79704547	-0.2359705	-6.9077553	12	65	1.78815556	304.111111	31.11111111	0	0	0	1	30.519848	1	0.21141735
2.0997	82144.7983	0.74161559	149.42781	1.09214218	0	0.74179448	4.4084957	-0.2975767	5.0068201	0.08905628	-6.9077553	13	70	1.78815556	303	30	0	0	0	1	30.519848	1	0.21141735
2.1519	89164.6837	0.62250275	100.416117	0.79987585	0	0.76635117	4.49049625	-0.4724021	4.60933268	-0.2220493	-6.9077553	14	77	0.44703889	301.888889	28.88888888	0	0	0	1	30.519848	1	0.21141735
2.1762	90261.2755	0.44716432	47.5642316	0.63341388	0	0.77758024	4.5027196	-0.8025953	3.86210207	-0.4550537	-6.9077553	15	75	0.89407778	297.444444	24.44444444	0	0	0	1	30.519848	1	0.21141735
2.2005	91138.5536	0.47379162	53.6779876	0.78663939	0	0.78868461	4.51239189	-0.7448793	3.98302163	-0.2387149	-6.9077553	16	88	0.89407778	295.222222	22.2222222	0	0	0	1	30.519848	1	0.21141735
2.2194	91874.1489	0.52608876	89.9453532	0.85722396	0	0.79723689	4.52043058	-0.6403863	4.49921342	-0.1528902	-6.9077553	17	46	2.23519444	296.888889	23.88888888	0	0	0	1	30.519848	1	0.21141735
2.4534	101491.705	0.65597053	141.603696	1.54830322	0	0.89747482	4.61998693	-0.4201161	4.95303934	0.4378053	-6.9077553	18	42	2.23519444	296.333333	23.33333333	0	0	0	1	30.519848	1	0.21141735
2.8107	112482.534	0.70969773	161.417034	2.43287885	0	1.03343356	4.72280685	-0.3415081	5.08399748	0.88948622	-6.9077553	19	35	3.12927222	295.777778	22.7777778	0	0	0	1	30.519848	1	0.21141735
3.2238	123020.354	0.73189344	157.9343	2.63561187	0	1.17056079	4.81235795	-0.310755	5.06218546	0.96949471	-6.9077553	20	36	4.91742778	294.666667	21.66666666	0	0	0	1	30.519848	1	0.21141735
3.537	136709.815	0.61160918	107.048637	1.33309226	0	1.26327891	4.91786786	-0.4900281	4.67329263	0.28825111	-6.9077553	21	43	2.68223333	293	20	0	0	0	1	30.519848	1	0.21141735
3.6936	151012.266	0.53649025	82.48103	0.74188329	0	1.30660159	5.01736769	-0.6208447	4.41258045	-0.2972163	-6.9077553	22	54	0.44703889	290.222222	17.2222222	0	0	0	1	30.519848	1	0.21141735
3.7899	161997.06	0.54282262	83.6265607	0.67268421	0	1.33233963	5.08758436	-0.6091322	4.42637314	-0.3949938	-6.9077553	23	55	1.34111667	291.333333	18.33333333	0	0	0	1	30.519848	1	0.21141735
0.0315	1162.85756	0.98261115	272.045726	2.9683324	0	-3.4577677	0.15173997	-0.0165246	5.60597384	1.08833715	-6.9077553	0	82	4.91742778	299.666667	26.66666666	0	0	0	0	34.0724638	1	0.49277013
0.0891	3538.85236	0.78663504	163.676529	1.25187123	0	-2.4179959	1.26408502	-0.2387204	5.09789821	0.2254379	-6.9077553	1	74	5.36446667	301.333333	28.33333333	0	0	0	0	34.0724638	1	0.49277013

0.1665	7122.61474	0.85917974	191.873705	1.2751118	0	-1.79276	1.96341529	-0.1506139	5.25684258	0.2438178	-6.9077553	2	68	5.36446667	302.444444	29.44444444	0	0	0	0	34.0724638	1	0.49277013
0.2853	12009.5552	0.98051915	266.384411	2.61629879	0	-1.254214	2.48578586	-0.0186538	5.58494417	0.96214279	-6.9077553	3	63	5.81150556	303.555556	30.55555555	0	0	0	1	34.0724638	1	0.49277013
0.5049	24457.8415	1.17026557	396.768443	3.10343258	0	-0.6833949	3.19699177	0.15808485	5.98335536	1.13283095	-6.9077553	4	60	4.91742778	304.666667	31.66666666	0	0	0	1	34.0724638	1	0.49277013
0.9873	44583.2075	1.33657962	515.066465	4.38514095	0	-0.0127813	3.7973797	0.29086173	6.24429789	1.47844978	-6.9077553	5	55	4.91742778	306.888889	33.88888888	0	0	0	1	34.0724638	1	0.49277013
1.7091	60173.1446	1.36777852	585.945449	4.5917433	1	0.53596692	4.09724277	0.31391875	6.3732284	1.52447751	0.0009995	6	51	5.81150556	307.444444	34.4444444	0	0	0	1	34.0724638	1	0.49277013
2.3562	79193.5112	1.12742112	372.914943	3.24598407	1	0.85705015	4.37190699	0.12081941	5.92135304	1.17772659	0.0009995	7	49	5.36446667	308.555556	35.5555556	0	0	0	1	34.0724638	1	0.49277013
3.0168	104665.424	1.02347642	293.113334	2.40831755	1	1.10419667	4.65077838	0.02418167	5.68056275	0.87934353	0.0009995	8	52	4.47038889	306.888889	33.88888888	0	0	0	1	34.0724638	1	0.49277013
3.5955	124942.099	0.98049552	270.338817	2.22770233	1	1.27968306	4.82785843	-0.0186778	5.59967975	0.8014195	0.0009995	9	52	4.47038889	307.444444	34.4444444	0	0	0	1	34.0724638	1	0.49277013
4.1319	142283.47	0.8507057	202.678626	2.03913869	1	1.41873735	4.95782837	-0.1605142	5.31162653	0.71301779	0.0009995	10	54	3.57631111	306.888889	33.88888888	0	0	0	1	34.0724638	1	0.49277013
4.5135	155157.039	0.77686907	176.745909	1.60348669	1	1.50707291	5.0444442	-0.2511971	5.17471882	0.47280389	0.0009995	11	58	2.68223333	305.777778	32.7777778	0	0	0	1	34.0724638	1	0.49277013
4.7169	163207.87	0.82663193	184.141126	1.32554424	1	1.5511518	5.09503079	-0.1891867	5.21570788	0.28257724	0.0009995	12	65	1.78815556	304.111111	31.11111111	0	0	0	1	34.0724638	1	0.49277013
4.8501	167995.316	0.74840484	151.545674	1.25769765	1	1.57899932	5.12394205	-0.2884759	5.02089366	0.23007757	0.0009995	13	70	1.78815556	303	30	0	0	0	1	34.0724638	1	0.49277013
4.9653	171251.904	0.67977142	123.438782	1.17418373	1	1.60247372	5.14314144	-0.3845287	4.81575344	0.1614245	0.0009995	14	77	0.44703889	301.888889	28.88888888	0	0	0	1	34.0724638	1	0.49277013
5.0355	174015.322	0.43812439	47.9377316	0.72702193	1	1.61651283	5.1591491	-0.8229726	3.86992377	-0.3174241	0.0009995	15	75	0.89407778	297.444444	24.44444444	0	0	0	1	34.0724638	1	0.49277013
5.0796	175901.461	0.39057518	40.531818	0.6457379	1	1.62523252	5.16992965	-0.9375778	3.70211197	-0.4358142	0.0009995	16	88	0.89407778	295.222222	22.2222222	0	0	0	1	34.0724638	1	0.49277013
5.1291	178107.272	0.43656984	58.7412598	0.64124527	1	1.63493021	5.18239164	-0.826519	4.0731594	-0.442785	0.0009995	17	46	2.23519444	296.888889	23.88888888	0	0	0	1	34.0724638	1	0.49277013
5.607	189924.175	0.59425794	117.851217	1.22399994	1	1.72401582	5.24663018	-0.5187605	4.76943144	0.2029408	0.0009995	18	42	2.23519444	296.333333	23.33333333	0	0	0	1	34.0724638	1	0.49277013
6.2091	205734.763	0.73281705	160.394245	1.53177642	1	1.82601596	5.32659264	-0.3094955	5.07764105	0.42708074	0.0009995	19	35	3.12927222	295.777778	22.7777778	0	0	0	1	34.0724638	1	0.49277013
7.0452	232942.387	0.89458335	235.275582	2.43604725	1	1.95234653	5.45079545	-0.11028	5.46076177	0.89078716	0.0009995	20	36	4.91742778	294.666667	21.66666666	0	0	0	1	34.0724638	1	0.49277013
7.7085	256728.883	0.82090761	194.311652	1.6439426	1	2.04232362	5.54802449	-0.1961273	5.26946847	0.49770549	0.0009995	21	43	2.68223333	293	20	0	0	0	1	34.0724638	1	0.49277013
8.2656	277917.408	0.78497546	179.104377	1.38062263	1	2.11210232	5.62732757	-0.2408297	5.18797433	0.32325863	0.0009995	22	54	0.44703889	290.222222	17.2222222	0	0	0	1	34.0724638	1	0.49277013
8.8443	299186.787	0.80099236	197.456296	1.55190532	1	2.17977318	5.70107143	-0.2206562	5.28552234	0.44012758	0.0009995	23	55	1.34111667	291.333333	18.33333333	0	0	0	1	34.0724638	1	0.49277013
0.0477	3396.24985	1.23828653	433.736929	2.85373834	0	-3.0428239	1.22296624	0.21453584	6.0724405	1.04898019	-6.9077553	0	82	4.91742778	299.666667	26.66666666	0	0	0	0	42.288408	1	-0.4111751
0.2016	10404.9011	1.03492282	306.985152	3.44846991	0	-1.6014697	2.34237306	0.03529264	5.72680264	1.23822057	-6.9077553	1	74	5.36446667	301.333333	28.33333333	0	0	0	0	42.288408	1	-0.4111751
0.4734	22606.2162	1.05497913	308.2366	3.21060322	0	-0.7478146	3.11826916	0.05446843	5.73087091	1.16677026	-6.9077553	2	68	5.36446667	302.444444	29.44444444	0	0	0	0	42.288408	1	-0.4111751
0.7857	36467.6815	0.95689502	245.705827	2.21534681	0	-0.2411802	3.59645385	-0.0430171	5.50413906	0.79586026	-6.9077553	3	63	5.81150556	303.555556	30.55555555	0	0	0	0	42.288408	1	-0.4111751
1.2267	52912.8256	0.92950751	241.737372	2.39707131	0	0.20432764	3.96866466	-0.0720251	5.48785604	0.87466479	-6.9077553	4	60	4.91742778	304.666667	31.66666666	0	0	0	0	42.288408	1	-0.4111751
1.728	70334.8304	0.87131992	207.569681	2.20877618	0	0.54696467	4.25328135	-0.136599	5.33547191	0.79289123	-6.9077553	5	55	4.91742778	306.888889	33.88888888	0	0	1	0	42.288408	1	-0.4111751
2.2365	91390.5193	0.94424746	248.047511	2.3115068	0	0.80491214	4.51515269	-0.0563085	5.51362434	0.83833213	-6.9077553	6	51	5.81150556	307.444444	34.4444444	0	0	1	0	42.288408	1	-0.4111751
2.8269	121661.322	1.00510648	283.749095	2.09264876	0	1.0391807	4.80124936	0.00608792	5.6480939	0.73890836	-6.9077553	7	49	5.36446667	308.555556	35.5555556	0	0	1	0	42.288408	1	-0.4111751
3.5361	158845.152	1.0474089	311.139197	2.30975683	0	1.26302442	5.06793614	0.04727368	5.74024361	0.8375751	-6.9077553	8	52	4.47038889	306.888889	33.88888888	0	0	1	0	42.288408	1	-0.4111751
4.1796	187195.16	0.97315759	270.794493	2.29318626	0	1.43021555	5.23215705	-0.0261822	5.6013639	0.83037821	-6.9077553	9	52	4.47038889	307.444444	34.4444444	0	0	1	0	42.288408	1	-0.4111751
4.7241	206669.123	0.86365795	220.321333	1.95513745	0	1.55267707	5.33112391	-0.1454213	5.39509162	0.67097184	-6.9077553	10	54	3.57631111	306.888889	33.88888888	0	0	1	0	42.288408	1	-0.4111751
5.1057	222026.044	0.79327328	172.982846	1.2917063	0	1.63035756	5.4027992	-0.2303277	5.15319821	0.25673793	-6.9077553	11	58	2.68223333	305.777778	32.7777778	0	0	1	0	42.288408	1	-0.4111751
5.4009	235294.779	0.83393283	189.859601	1.3639464	0	1.68656561	5.46084336	-0.180404	5.24629013	0.31111516	-6.9077553	12	65	1.78815556	304.111111	31.11111111	0	0	1	0	42.288408	1	-0.4111751
5.6439	246309.622	0.80875109	173.818027	1.32765311	0	1.73057532	5.50659343	-0.2110284	5.15801468	0.28416573	-6.9077553	13	70	1.78815556	303	30	0	0	1	0	42.288408	1	-0.4111751
5.8437	254555.805	0.71611607	133.514288	1.19112293	0	1.76536416	5.53952401	-0.3325176	4.89421599	0.17573569	-6.9077553	14	77	0.44703889	301.888889	28.88888888	0	0	1	0	42.288408	1	-0.4111751
5.958	259205.366	0.44363493	48.4262542	0.73720352	0	1.78473485	5.55762453	-0.8105017	3.88006276	-0.3035357	-6.9077553	15	75	0.89407778	297.444444	24.44444444	0	0	1	0	42.288408	1	-0.4111751
6.03	262144.24	0.4270937	46.1210144	0.66634445	0	1.79674701	5.5688987	-0.8484132	3.83129037	-0.4044489	-6.9077553	16	88	0.89407778	295.222222	22.2222222	0	0	1	0	42.288408	1	-0.4111751
6.1659	266653.62	0.56106385	95.0001164	1.07053097	0	1.81903411	5.58595426	-0.5761398	4.55388864	0.06908844	-6.9077553	17	46	2.23519444	296.888889	23.88888888	0	0	1	0	42.288408	1	-0.4111751
6.7761	277805.282	0.54967055	96.3902563	1.53959831	0	1.91340172	5.62692404	-0.5966186	4.56841549	0.43217086	-6.9077553	18	42	2.23519444	296.333333	23.33333333	0	0	1	0	42.288408	1	-0.4111751
7.3746	284706.48	0.54918975	93.1844161	1.86746487	0	1.99804166	5.65146227	-0.5974921	4.53459123	0.62511717	-6.9077553	19	35	3.12927222	295.777778	22.7777778	0	0	1	0	42.288408	1	-0.4111751
8.1612	293644.232	0.60103993	107.365543	2.52883541	0	2.09939122	5.68237235	-0.5074315	4.67624861	0.92815425	-6.9077553	20	36	4.91742778	294.666667	21.66666666	0	0	1	0	42.288408	1	-0.4111751
8.5977	303201.215	0.59300738	111.596746	1.30444273	0	2.15149473	5.71439996	-0.5208635	4.71490085	0.26654224	-6.9077553	21	43	2.68223333	293	20	0	0	1	0	42.288408	1	-0.4111751
9.018	316217.242	0.71012052	165.662289	1.32085959	0	2.19922258	5.75643261	-0.3409134	5.10995735	0.27903953	-6.9077553	22	54	0.44703889	290.222222	17.2222222	0	0	1	0	42.288408	1	-0.4111751
9.4329	326030.715	0.6338311	126.808348	1.19942436	0	2.24420358	5.78699466	-0.4543963	4.84268476	0.18267513	-6.9077553	23	55	1.34111667	291.333333	18.33333333	0	0	1	0	42.288408	1	-0.4111751
0.0243	0	0.68746251	131.543438	3.13539036	0	-3.7172789	-6.9077553	-0.3732944	4.87934473	1.14307257	-6.9077553	0	82	4.91742778	299.666667	26.66666666	0	0	0	0	18.9135952	1	-0.0428739
0.0936	370.950217	0.6046691	107.125228	2.18197216	0	-2.3687249	-0.9889953	-0.5014215	4.67400784	0.78068732	-6.9077553	1	74	5.36446667	301.333333	28.33333333	0	0	0	0	18.9135952	1	-0.0428739
0.3015	6353.97083	0.82660797	225.422822	2.53249131	0	-1.1989853	1.84923731	-0.1892157	5.41798228	0.92959832	-6.9077553	2	68	5.36446667	302.444444	29.44444444	0	0	1	0	18.9135952	1	-0.0428739
0.5427	13345.5865	0.80109679	220.158815	2.50956885	0	-0.6111986	2.59126066	-0.220526	5.39435372	0.92050936	-6.9077553	3	63	5.81150556	303.555556	30.55555555	0	0	1	0	18.9135952	1	-0.0428739
0.738	17951.3328	0.78538492	199.763663	2.03176978	0	-0.3038115	2.88772007	-0.2403089	5.29713999	0.70939929	-6.9077553	4	60	4.91742778	304.666667	31.66666666	0	0	1	0	18.9135952	1	-0.0428739
1.0035	27990.3706	0.89558499	250.826615	2.19069328	0	0.00349389	3.33189627	-0.1091622	5.52476591	0.78467443	-6.9077553	5	55	4.91742778	306.888889	33.88888888	0	0	1	0	18.9135952	1	-0.0428739
1.3815	41304.5004	0.9194413	254.163821	2.64047503	0	0.32316987	3.72099567	-0.0829021	5.53798296	0.97133748	-6.9077553	6	51	5.81150556	307.444444	34.4444444	0	0	1	0	18.9135952	1	-0.0428739
1.7397	56972.0317	0.94815861	270.841473	2.11636475	0	0.55371268	4.04257803	-0.0521794	5.60153737	0.75017227	-6.9077553	7	49	5.36446667	308.555556	35.5555556	0	0	1	0	18.9135952	1	-0.0428739
2.0799	69515.5223	0.85656407	217.980076	1.90201581	0	0.73231982	4.24156446	-0.1536594	5.38440825	0.64343989	-6.9077553	8	52	4.47038889	306.888889	33.88888888	0	0	1	0	18.9135952	1	-0.0428739
2.4921	87077.1775	0.90208415	245.015898	1.97781372	0	0.91312573	4.46680631	-0.1019395	5.50132718	0.68249753	-6.9077553	9	52	4.47038889	307.444444	34.4444444	0	0	1	0	18.9135952	1	-0.0428739
2.8593	98377.3485	0.7617937	169.593252	1.71153319	0	1.05057684	4.58882074	-0.2707677	5.13340883	0.53797367	-6.9077553	10	54	3.57631111	306.888889	33.88888888	0	0	1	0	18.9135952	1	-0.0428739
3.1554	109389.226	0.7712804	179.900123	1.51050742	0	1.14911527	4.69492154	-0.2584076	5.19240739	0.41310744	-6.9077553	11	58	2.68223333	305.777778	32.7777778	0	0	1	0	18.9135952	1	-0.0428739
3.3552	118599.134	0.83328281	189.260557	1.32889715	0	1.21051138	4.77575761	-0.1811828	5.24312996	0.28510161	-6.9077553	12	65	1.78815556	304.111111	31.11111111	0	0	1	0	18.9135952	1	-0.0428739
3.5541	127814.009	0.8498172	191.603825	1.46748898	0	1.26810187	4.85058397	-0.161558	5.25543505	0.38423396	-6.9077553	13	70	1.78815556	303	30	0	0	1	0	18.9135952	1	-0.0428739
3.7188	135194.988	0.77299441	160.736424	1.44654021	0	1.31340104	4.90672549	-0.2561906	5.07977213	0.36986571	-6.9077553	14	77	0.44703889	301.888889	28.88888888	0	0	1	0	18.9135952	1	-0.0428739
3.8025	138791.813	0.51945245	69.2116778	0.95796827	0	1.33565875	4.93298227	-0.6530567	4.23718405	-0.0418973	-6.9077553	15	75	0.89407778	297.444444	24.44444444	0	0	1	0	18.9135952	1	-0.0428739

3.8619	141248.187	0.47301391	57.0597257	0.80894898	0	1.35115929	4.95052562	-0.7465186	4.04411606	-0.210784	-6.9077553	16	88	0.89407778	295.222222	22.2222222	0	0	1	0	18.9135952	1	-0.0428739
3.9105	143237.342	0.46400667	69.6552418	0.77801606	0	1.36366524	4.96450997	-0.7657035	4.24357231	-0.2497236	-6.9077553	17	46	2.23519444	296.888889	23.8888888	0	0	1	0	18.9135952	1	-0.0428739
4.3623	152499.023	0.55646372	103.152997	1.40638788	0	1.47299944	5.02716475	-0.5843579	4.63622299	0.34173542	-6.9077553	18	42	2.23519444	296.333333	23.3333333	0	0	1	0	18.9135952	1	-0.0428739
4.8834	163806.592	0.62098907	122.369616	1.69713396	0	1.5858417	5.09869252	-0.4748328	4.80705428	0.52952998	-6.9077553	19	35	3.12927222	295.777778	22.7777778	0	0	1	0	18.9135952	1	-0.0428739
5.5854	182144.511	0.73403234	173.053065	2.32001499	0	1.72015605	5.20480588	-0.3078408	5.15360406	0.84200459	-6.9077553	20	36	4.91742778	294.666667	21.6666666	0	0	1	0	18.9135952	1	-0.0428739
6.057	196267.136	0.70742099	159.7039	1.47913425	0	1.80121463	5.27948177	-0.3447167	5.07332774	0.39213279	-6.9077553	21	43	2.68223333	293	20	0	0	1	0	18.9135952	1	-0.0428739
6.471	210608.452	0.72862721	166.673132	1.33532144	0	1.86733066	5.35000548	-0.3152215	5.1160406	0.28992065	-6.9077553	22	54	0.44703889	290.222222	17.2222222	0	0	1	0	18.9135952	1	-0.0428739
6.9822	229953.678	0.79310264	191.20351	1.50539087	0	1.94336405	5.43788224	-0.2305426	5.25334359	0.40971663	-6.9077553	23	55	1.34111667	291.333333	18.3333333	0	0	1	0	18.9135952	1	-0.0428739
0.0243	525.596683	0.71300925	146.520339	2.47417919	0	-3.7172789	-0.6413203	-0.3368594	4.98717108	0.9063128	-6.9077553	0	68	5.36446667	302.444444	29.4444444	0	0	0	0	30.519848	1	0.21141735
0.0927	1324.94181	0.54042144	77.35694	2.05305019	0	-2.3783868	0.282123	-0.6135573	4.34844322	0.71981355	-6.9077553	1	63	5.81150556	303.555556	30.5555555	0	0	0	0	30.519848	1	0.21141735
0.162	2522.7006	0.62322091	109.805727	1.57980991	0	-1.8201589	0.92572632	-0.4712509	4.6987218	0.45793732	-6.9077553	2	60	4.91742778	304.666667	31.6666666	0	0	0	0	30.519848	1	0.21141735
0.2493	3117.71943	0.55538925	85.7296734	1.5826243	0	-1.3890983	1.13742248	-0.5862871	4.45121068	0.45971608	-6.9077553	3	55	4.91742778	306.888889	33.8888888	0	0	0	0	30.519848	1	0.21141735
0.4248	6259.87029	0.79902391	201.375441	2.66550168	0	-0.8561368	1.8343192	-0.2231137	5.305176	0.98076738	-6.9077553	4	51	5.81150556	307.444444	34.4444444	0	0	0	1	30.519848	1	0.21141735
0.6939	10992.3862	0.81449344	192.183163	2.72325129	0	-0.3654274	2.39729384	-0.2039619	5.25845409	1.00219364	-6.9077553	5	49	5.36446667	308.555556	35.5555556	0	0	0	1	30.519848	1	0.21141735
0.9234	14855.1077	0.71519422	137.510141	1.82992685	0	-0.0796928	2.69841108	-0.3338039	4.92370494	0.60482232	-6.9077553	6	52	4.47038889	306.888889	33.8888888	0	0	0	1	30.519848	1	0.21141735
1.1772	18719.988	0.66889239	123.956145	1.67505781	0	0.16313874	2.92964525	-0.4006382	4.8199359	0.51644449	-6.9077553	7	52	4.47038889	307.444444	34.4444444	0	0	0	1	30.519848	1	0.21141735
1.3455	23269.4452	0.68263547	136.186581	1.30924849	0	0.29676569	3.14718411	-0.3803304	4.91403321	0.27021681	-6.9077553	8	54	3.57631111	306.888889	33.8888888	0	0	0	1	30.519848	1	0.21141735
1.476	27408.8704	0.70845658	148.862365	1.11476956	0	0.38933573	3.31090318	-0.343256	5.00302887	0.10954436	-6.9077553	9	58	2.68223333	305.777778	32.7777778	0	0	0	1	30.519848	1	0.21141735
1.5642	30743.2268	0.7900544	169.391283	1.18744994	0	0.44737451	3.42570223	-0.2343885	5.13221722	0.17264989	-6.9077553	10	65	1.78815556	304.111111	31.1111111	0	0	0	1	30.519848	1	0.21141735
1.6182	32689.5651	0.70276076	132.56897	1.09266586	0	0.48131442	3.48708651	-0.3513168	4.88711058	0.08953523	-6.9077553	11	70	1.78815556	303	30	0	0	0	1	30.519848	1	0.21141735
1.6605	34692.7234	0.72494166	134.120741	1.15988906	0	0.50711876	3.54655879	-0.3202856	4.8987479	0.14918614	-6.9077553	12	77	0.44703889	301.888889	28.8888888	0	0	0	1	30.519848	1	0.21141735
1.6947	36315.6842	0.57737822	81.7219238	0.97027206	0	0.52750573	3.59227726	-0.5475273	4.40333455	-0.0291487	-6.9077553	13	75	0.89407778	297.444444	24.4444444	0	0	0	1	30.519848	1	0.21141735
1.7208	37500.0067	0.47771543	54.8161284	0.72879259	0	0.5427893	3.62436778	-0.736649	4.00400271	-0.3149949	-6.9077553	14	88	0.89407778	295.222222	22.2222222	0	0	0	1	30.519848	1	0.21141735
1.7415	38114.0991	0.29878007	26.1586357	0.57389085	0	0.55474681	3.64061051	-1.2047062	3.2642176	-0.5535751	-6.9077553	15	46	2.23519444	296.888889	23.8888888	0	0	0	1	30.519848	1	0.21141735
1.9467	41683.8674	0.61867108	119.873818	1.53824214	0	0.66613563	3.73013817	-0.4785665	4.78644801	0.43129018	-6.9077553	16	42	2.23519444	296.333333	23.3333333	0	0	0	1	30.519848	1	0.21141735
2.16	44909.7804	0.63706333	125.449548	1.61769188	0	0.77010822	3.80467786	-0.4493177	4.83191164	0.48161834	-6.9077553	17	35	3.12927222	295.777778	22.7777778	0	0	0	1	30.519848	1	0.21141735
2.6937	47982.34	0.91859186	254.696617	3.93177855	0	0.99091571	3.87085387	-0.0838253	5.54007702	1.36934619	-6.9077553	18	36	4.91742778	294.666667	21.6666666	0	0	0	1	30.519848	1	0.21141735
2.9151	49268.4238	0.66820049	125.75813	1.56140335	0	1.06990412	3.89730368	-0.4016716	4.83436841	0.44622525	-6.9077553	19	43	2.68223333	293	20	0	0	0	1	30.519848	1	0.21141735
2.9691	49473.9682	0.52122316	87.6039714	0.72003625	0	1.08825888	3.90146685	-0.6496603	4.47283775	-0.3270659	-6.9077553	20	54	0.44703889	290.222222	17.2222222	0	0	0	1	30.519848	1	0.21141735
3.0114	49617.6989	0.43128139	61.8666005	0.69770781	0	1.10240509	3.90436776	-0.8386785	4.12499663	-0.3585226	-6.9077553	21	55	1.34111667	291.333333	18.3333333	0	0	0	1	30.519848	1	0.21141735
3.1473	50047.9705	0.66917157	123.985591	1.77735103	0	1.14654494	3.91300194	-0.4002215	4.82017342	0.57568654	-6.9077553	22	46	3.12927222	294.111111	21.1111111	0	0	0	1	30.519848	1	0.21141735
3.618	52736.2862	0.96987232	273.276052	4.67779693	0	1.28592139	3.96532272	-0.0295603	5.61048612	1.54304101	-6.9077553	23	40	5.36446667	295.777778	22.7777778	0	0	0	1	30.519848	1	0.21141735
0.0414	1709.17729	1.1312934	362.36862	3.50159064	0	-3.1844744	0.53659705	0.12424514	5.89266474	1.25350288	-6.9077553	0	68	5.36446667	302.444444	29.4444444	0	0	0	0	34.0724638	1	0.49277013
0.1314	6072.16744	0.91977875	230.789883	1.51819168	0	-2.0295092	1.80388029	-0.0825355	5.44151203	0.4181784	-6.9077553	1	63	5.81150556	303.555556	30.5555555	0	0	0	0	34.0724638	1	0.49277013
0.2205	9656.91301	0.8806046	201.291055	1.66947367	0	-1.5118576	2.26777758	-0.1260116	5.30475686	0.51310722	-6.9077553	2	60	4.91742778	304.666667	31.6666666	0	0	0	0	34.0724638	1	0.49277013
0.3411	14138.1342	0.99389671	285.477933	2.87242081	0	-1.0755796	2.64894643	-0.0051164	5.65416824	1.05550324	-6.9077553	3	55	4.91742778	306.888889	33.8888888	0	0	0	1	34.0724638	1	0.49277013
0.6453	34494.0672	1.3802306	560.780325	4.04997371	0	-0.43804	3.54081633	0.32297484	6.32933104	1.39895727	-6.9077553	4	51	5.81150556	307.444444	34.4444444	0	0	0	1	34.0724638	1	0.49277013
1.1727	53430.7519	1.32642726	517.524531	4.49867931	1	0.15930878	3.97840517	0.28324268	6.24905886	1.50400613	0.0009995	5	49	5.36446667	308.555556	35.5555556	0	0	0	1	34.0724638	1	0.49277013
1.6893	68429.2981	1.11668593	357.435649	2.91015668	1	0.52431424	4.22581568	0.11126041	5.87895814	1.06855049	0.0009995	6	52	4.47038889	306.888889	33.8888888	0	0	0	1	34.0724638	1	0.49277013
2.25	85768.6981	1.04971867	312.745812	2.82487809	1	0.81093022	4.45166578	0.04947438	5.74539395	1.03881914	0.0009995	7	52	4.47038889	307.444444	34.4444444	0	0	0	1	34.0724638	1	0.49277013
2.8035	106222.849	0.98175873	273.916159	2.28104834	1	1.03086864	4.66554865	-0.0173916	5.61282572	0.82507343	0.0009995	8	54	3.57631111	306.888889	33.8888888	0	0	0	1	34.0724638	1	0.49277013
3.1725	119509.945	0.84119129	202.711509	1.52315599	1	1.15451992	4.78340795	-0.1717481	5.31178876	0.42144081	0.0009995	9	58	2.68223333	305.777778	32.7777778	0	0	0	1	34.0724638	1	0.49277013
3.3912	128421.853	0.84682524	196.915927	1.38773249	1	1.22118384	4.85532836	-0.1650807	5.28278195	0.32839145	0.0009995	10	65	1.78815556	304.111111	31.1111111	0	0	0	1	34.0724638	1	0.49277013
3.5604	135707.973	0.83302453	187.780751	1.48235613	1	1.2698729	4.91051269	-0.1814925	5.23528039	0.39430718	0.0009995	11	70	1.78815556	303	30	0	0	0	1	34.0724638	1	0.49277013
3.6801	139864.151	0.72242588	138.626469	1.29421997	1	1.30293993	4.94067875	-0.3237572	4.93179025	0.25868054	0.0009995	12	77	0.44703889	301.888889	28.8888888	0	0	0	1	34.0724638	1	0.49277013
3.7413	141881.883	0.50446477	68.4307071	0.97288648	1	1.31943314	4.95500195	-0.6822769	4.22583627	-0.0264605	0.0009995	13	75	0.89407778	297.444444	24.4444444	0	0	0	1	34.0724638	1	0.49277013
3.7962	143504.84	0.45465075	58.2057265	0.80644758	1	1.33400057	4.96637573	-0.7860287	4.06400092	-0.2138771	0.0009995	14	88	0.89407778	295.222222	22.2222222	0	0	0	1	34.0724638	1	0.49277013
3.9321	146752.035	0.63111574	135.279103	1.47540687	1	1.36917363	4.98875114	-0.4586828	4.90734746	0.38961135	0.0009995	15	46	2.23519444	296.888889	23.8888888	0	0	0	1	34.0724638	1	0.49277013
4.4289	158396.364	0.66103761	138.603063	1.46429554	1	1.48815125	5.06510684	-0.4124329	4.9316214	0.38205696	0.0009995	16	42	2.23519444	296.333333	23.3333333	0	0	0	1	34.0724638	1	0.49277013
4.9401	170594.634	0.77468389	183.095401	1.59919438	1	1.59738557	5.13929604	-0.2540102	5.21001279	0.47012511	0.0009995	17	35	3.12927222	295.777778	22.7777778	0	0	0	1	34.0724638	1	0.49277013
5.6079	189415.826	0.86104434	228.171896	2.18464451	1	1.72417632	5.24395002	-0.1484486	5.43010366	0.78191075	0.0009995	18	36	4.91742778	294.666667	21.6666666	0	0	0	1	34.0724638	1	0.49277013
6.1173	206967.458	0.78419602	176.375193	1.51743557	1	1.81112082	5.3325664	-0.2418219	5.17261918	0.41768057	0.0009995	19	43	2.68223333	293	20	0	0	0	1	34.0724638	1	0.49277013
6.5799	222129.813	0.75894569	172.069553	1.39349852	1	1.88401955	5.40326646	-0.2745083	5.14790458	0.33253487	0.0009995	20	54	0.44703889	290.222222	17.2222222	0	0	0	1	34.0724638	1	0.49277013
7.0713	236703.246	0.73266618	157.03238	1.42719948	1	1.95604434	5.46681146	-0.3097011	5.0564584	0.35641455	0.0009995	21	55	1.34111667	291.333333	18.3333333	0	0	0	1	34.0724638	1	0.49277013
7.7832	259353.159	0.79941047	186.474972	1.76252247	1	2.05196756	5.55819454	-0.2226306	5.2283024	0.56731321	0.0009995	22	46	3.12927222	294.111111	21.1111111	0	0	0	1	34.0724638	1	0.49277013
8.8632	294785.606	0.91314056	244.442448	2.51392598	1	2.18190787	5.68625172	-0.0897709	5.49898399	0.92224337	0.0009995	23	40	5.36446667	295.777778	22.7777778	0	0	0	1	34.0724638	1	0.49277013
0.063	4585.62191	1.37112106	537.340523	3.68376093	0	-2.7646206	1.52314379	0.31635776	6.28663388	1.30420565	-6.9077553	0	68	5.36446667	302.444444	29.4444444	0	0	0	0	42.288408	1	-0.4111751
0.2943	15421.462	1.17242016	388.692364	4.35145468	0	-1.2231556	2.73582502	0.1599227	5.96279076	1.47073998	-6.9077553	1	63	5.81150556	303.555556	30.5555555	0	0	0	0	42.288408	1	-0.4111751
0.5931	29751.5213	1.04397215	299.292922	2.6967597	0	-0.5223923	3.39291388	0.04399024	5.70142611	0.99242169	-6.9077553	2	60	4.91742778	304.666667	31.6666666	0	0	0	0	42.288408	1	-0.4111751
0.9207	44964.9995	0.96097657	251.60328	1.98215426	0	-0.082621	3.80590664	-0.0387652	5.52785753	0.68468864	-6.9077553	3	55	4.91742778	306.888889	33.8888888	0	0	0	0	42.288408	1	-0.4111751
1.4517	63330.4202	0.9780081	273.196292	3.25265782	0	0.37273528	4.14838158	-0.0212154	5.61019422	1.17977985	-6.9077553	4	51	5.81150556	307.444444	34.4444444	0	0	1	0	42.288408	1	-0.4111751
2.0169	86690.5906	0.95743468	260.42093	2.54607105	0	0.70156168	4.46235689	-0.0424539	5.56230312	0.93494409	-6.9077553	5	49	5.36446667	308.555556	35.5555556	0	0	1	0	42.288408	1	-0.4111751

2.4912	107793.821	0.92083167	230.251809	1.70007174	0	0.91276452	4.68022962	-0.0813926	5.43917787	0.53125849	-6.9077553	6	52	4.47038889	306.888889	33.88888888	0	0	1	0	42.288408	1	-0.4111751
3.1338	140743.347	1.02797145	298.769564	2.27402802	0	1.14224633	4.9469451	0.02855972	5.69967593	0.82199237	-6.9077553	7	52	4.47038889	307.444444	34.4444444	0	0	1	0	42.288408	1	-0.4111751
3.7701	171403.948	0.99224266	280.421986	2.11970004	0	1.32710153	5.14402887	-0.0067803	5.63629913	0.75174624	-6.9077553	8	54	3.57631111	306.888889	33.88888888	0	0	1	0	42.288408	1	-0.4111751
4.2489	192099.463	0.8775364	226.156456	1.77577336	0	1.44666013	5.25801848	-0.1294979	5.42123146	0.574799	-6.9077553	9	58	2.68223333	305.777778	32.7777778	0	0	1	0	42.288408	1	-0.4111751
4.5459	206226.184	0.88234188	217.423769	1.52896645	0	1.51422573	5.3289784	-0.124043	5.3818529	0.42524581	-6.9077553	10	65	1.78815556	304.111111	31.11111111	0	0	1	0	42.288408	1	-0.4111751
4.8033	218060.141	0.81216681	174.229786	1.35323531	0	1.56930318	5.38477549	-0.206819	5.16038078	0.30323695	-6.9077553	11	70	1.78815556	303	30	0	0	1	0	42.288408	1	-0.4111751
5.0301	227335.208	0.71047865	132.552719	1.18087672	0	1.61543986	5.42643002	-0.3404099	4.88698799	0.16710361	-6.9077553	12	77	0.44703889	301.888889	28.88888888	0	0	1	0	42.288408	1	-0.4111751
5.1507	232511.135	0.50464602	63.7449277	0.84179619	0	1.63913263	5.44894242	-0.6819184	4.1549053	-0.1710301	-6.9077553	13	75	0.89407778	297.444444	24.44444444	0	0	1	0	42.288408	1	-0.4111751
5.2452	236239.536	0.49898006	62.2666109	0.8109367	0	1.65731337	5.46485051	-0.6931871	4.1314414	-0.2083329	-6.9077553	14	88	0.89407778	295.222222	22.2222222	0	0	1	0	42.288408	1	-0.4111751
5.3325	239277.54	0.45220066	65.9942161	0.78670407	0	1.67382017	5.47762831	-0.7914203	4.18958226	-0.2386328	-6.9077553	15	46	2.23519444	296.888889	23.88888888	0	0	1	0	42.288408	1	-0.4111751
6.0129	254811.846	0.62536758	121.405434	1.7113557	0	1.79390716	5.54052934	-0.4678179	4.79914387	0.53787003	-6.9077553	16	42	2.23519444	296.333333	23.33333333	0	0	1	0	42.288408	1	-0.4111751
6.6762	268392.649	0.63558066	123.666554	1.74903073	0	1.89854896	5.59245474	-0.4516442	4.81759695	0.55963335	-6.9077553	17	35	3.12927222	295.777778	22.7777778	0	0	1	0	42.288408	1	-0.4111751
7.515	280205.584	0.62136933	118.541619	2.45717768	0	2.01690102	5.63552713	-0.4742216	4.77527255	0.8994203	-6.9077553	18	36	4.91742778	294.666667	21.66666666	0	0	1	0	42.288408	1	-0.4111751
7.9218	288898.809	0.5738682	104.442322	1.22949003	0	2.06961845	5.66607995	-0.5536145	4.64864455	0.20741249	-6.9077553	19	43	2.68223333	293	20	0	0	1	0	42.288408	1	-0.4111751
8.2035	295584.182	0.55833791	105.229757	0.97776907	0	2.10456089	5.68895706	-0.5810015	4.65615563	-0.0214596	-6.9077553	20	54	0.44703889	290.222222	17.2222222	0	0	1	0	42.288408	1	-0.4111751
8.5014	303572.097	0.61726218	127.478864	1.10136409	0	2.14023086	5.71562243	-0.4808427	4.84795842	0.09745705	-6.9077553	21	55	1.34111667	291.333333	18.33333333	0	0	1	0	42.288408	1	-0.4111751
9.0288	311654.075	0.61732983	112.616348	1.57847163	0	2.20041947	5.74189705	-0.4807333	4.72399577	0.45709038	-6.9077553	22	46	3.12927222	294.111111	21.11111111	0	0	1	0	42.288408	1	-0.4111751
10.206	334516.185	0.84963812	224.585531	3.42255777	0	2.32297578	5.81268825	-0.1617685	5.41426107	1.23068029	-6.9077553	23	40	5.36446667	295.777778	22.7777778	0	0	1	0	42.288408	1	-0.4111751
0.0459	125.224544	0.77256798	176.007988	3.81893127	0	-3.0812902	-2.0696929	-0.2567417	5.17053506	1.34023243	-6.9077553	0	68	5.36446667	302.444444	29.44444444	0	0	0	0	18.9135952	1	-0.0428739
0.2178	3179.66068	0.79859757	210.153428	3.01948268	0	-1.5241781	1.15708893	-0.2236467	5.34784263	1.10541665	-6.9077553	1	63	5.81150556	303.555556	30.55555555	0	0	0	0	18.9135952	1	-0.0428739
0.4797	12404.3451	0.89242019	262.254671	2.5535444	0	-0.7345944	2.51812744	-0.1126983	5.56931987	0.93787389	-6.9077553	2	60	4.91742778	304.666667	31.66666666	0	0	1	0	18.9135952	1	-0.0428739
0.6615	16977.684	0.80165606	228.594736	2.13267321	0	-0.4132453	2.83195868	-0.219829	5.4319551	0.757845	-6.9077553	3	55	4.91742778	306.888889	33.88888888	0	0	1	0	18.9135952	1	-0.0428739
0.927	26453.0261	0.9015384	259.633978	2.33257064	0	-0.0758017	3.27540836	-0.102544	5.55927671	0.84739956	-6.9077553	4	51	5.81150556	307.444444	34.4444444	0	0	1	0	18.9135952	1	-0.0428739
1.269	37879.9072	0.89245742	239.650164	2.47756573	0	0.23822919	3.63444722	-0.1126566	5.47918438	0.90768006	-6.9077553	5	49	5.36446667	308.555556	35.5555556	0	0	1	0	18.9135952	1	-0.0428739
1.5993	50837.7699	0.89251559	236.820972	1.9961473	0	0.46956603	3.92865925	-0.1125915	5.46730869	0.69171981	-6.9077553	6	52	4.47038889	306.888889	33.88888888	0	0	1	0	18.9135952	1	-0.0428739
1.9044	63097.3131	0.86381472	222.858453	1.76680213	0	0.644167	4.14469404	-0.14524	5.40654132	0.56973704	-6.9077553	7	52	4.47038889	307.444444	34.4444444	0	0	1	0	18.9135952	1	-0.0428739
2.2374	76589.8504	0.88606204	237.197382	1.9801126	0	0.80531448	4.33847762	-0.1198404	5.46889684	0.68365861	-6.9077553	8	54	3.57631111	306.888889	33.88888888	0	0	1	0	18.9135952	1	-0.0428739
2.5452	89826.8354	0.86511409	217.507584	1.62946984	0	0.93420923	4.4978949	-0.1437386	5.38223832	0.48886822	-6.9077553	9	58	2.68223333	305.777778	32.7777778	0	0	1	0	18.9135952	1	-0.0428739
2.7144	97230.9273	0.81679024	180.110996	1.30216818	0	0.99857093	4.57709913	-0.2011494	5.19357886	0.26479836	-6.9077553	10	65	1.78815556	304.111111	31.11111111	0	0	1	0	18.9135952	1	-0.0428739
2.8791	104797.437	0.85841762	202.049116	1.56475396	0	1.05747775	4.65203886	-0.1515003	5.30851577	0.44836747	-6.9077553	11	70	1.78815556	303	30	0	0	1	0	18.9135952	1	-0.0428739
3.0015	110271.747	0.69322827	126.071978	1.11852466	0	1.09911216	4.70295682	-0.3649544	4.83686093	0.11290419	-6.9077553	12	77	0.44703889	301.888889	28.88888888	0	0	1	0	18.9135952	1	-0.0428739
3.0843	113956.298	0.4858512	57.8539614	0.81252363	0	1.12632473	4.7358238	-0.7197967	4.05793921	-0.2063803	-6.9077553	13	75	0.89407778	297.444444	24.44444444	0	0	1	0	18.9135952	1	-0.0428739
3.1671	117728.579	0.50586477	63.4986891	0.86688995	0	1.15281634	4.76839029	-0.679511	4.15103501	-0.1416904	-6.9077553	14	88	0.89407778	295.222222	22.2222222	0	0	1	0	18.9135952	1	-0.0428739
3.2418	120538.024	0.28225862	23.6770873	0.61328635	0	1.17612873	4.79197355	-1.2613949	3.16455003	-0.4872941	-6.9077553	15	46	2.23519444	296.888889	23.88888888	0	0	1	0	18.9135952	1	-0.0428739
3.78	135515.485	0.69741403	163.99237	1.64340057	0	1.32972401	4.90909329	-0.3589432	5.099826	0.49737592	-6.9077553	16	42	2.23519444	296.333333	23.33333333	0	0	1	0	18.9135952	1	-0.0428739
4.3254	147164.301	0.6326447	129.373904	1.74865259	0	1.46450462	4.99155645	-0.4562669	4.86271442	0.55941725	-6.9077553	17	35	3.12927222	295.777778	22.7777778	0	0	1	0	18.9135952	1	-0.0428739
4.9977	162158.458	0.68362267	148.212674	2.24810391	0	1.60897781	5.08858016	-0.3788874	4.99865497	0.81053187	-6.9077553	18	36	4.91742778	294.666667	21.66666666	0	0	1	0	18.9135952	1	-0.0428739
5.4756	178051.181	0.73070375	172.386167	1.55095711	0	1.70030186	5.18207666	-0.3123796	5.14974292	0.43951679	-6.9077553	19	43	2.68223333	293	20	0	0	1	0	18.9135952	1	-0.0428739
5.8743	192292.915	0.73073542	169.188347	1.36700288	0	1.7705869	5.25902501	-0.3123363	5.13101848	0.31335192	-6.9077553	20	54	0.44703889	290.222222	17.2222222	0	0	1	0	18.9135952	1	-0.0428739
6.246	204873.953	0.7069009	156.694357	1.33321397	0	1.83194126	5.32239981	-0.3454512	5.05430352	0.28834233	-6.9077553	21	55	1.34111667	291.333333	18.33333333	0	0	1	0	18.9135952	1	-0.0428739
6.9138	225255.649	0.75664855	179.157893	1.90110429	0	1.93351941	5.41724041	-0.2775356	5.18827308	0.6429608	-6.9077553	22	46	3.12927222	294.111111	21.11111111	0	0	1	0	18.9135952	1	-0.0428739
7.9929	259394.715	0.88749831	235.074176	3.08526779	0	2.07855365	5.55835475	-0.1182225	5.45990536	1.12696253	-6.9077553	23	40	5.36446667	295.777778	22.7777778	0	0	1	0	18.9135952	1	-0.0428739
0.009	309.591941	0.91256449	223.668443	1.58950933	0	-4.7105307	-1.1692753	-0.0904013	5.41016926	0.4640543	-6.9077553	0	90	1.78815556	297.444444	24.44444444	0	0	0	0	30.519848	1	0.21141735
0.0162	660.502203	0.54176921	69.7721329	0.71074253	0	-4.122744	-0.413242	-0.6110711	4.24524902	-0.340039	-6.9077553	1	90	2.23519444	296.888889	23.88888888	0	0	0	0	30.519848	1	0.21141735
0.0306	1055.27628	0.59516597	93.4500351	1.13786221	0	-3.4867553	0.05474978	-0.5172362	4.53743761	0.1300297	-6.9077553	2	84	1.78815556	296.888889	23.88888888	0	0	0	0	30.519848	1	0.21141735
0.0387	1362.32274	0.49880123	62.8334215	0.88824542	0	-3.2519157	0.30992491	-0.6935448	4.14050304	-0.117382	-6.9077553	3	85	2.23519444	296.888889	23.88888888	0	0	0	0	30.519848	1	0.21141735
0.0432	1450.05024	0.44473042	51.1922852	0.80127907	0	-3.1419148	0.3722876	-0.8080409	3.93560837	-0.2202988	-6.9077553	4	81	2.23519444	296.888889	23.88888888	0	0	0	0	30.519848	1	0.21141735
0.0441	1493.91412	0.24566619	12.2807646	0.65691119	0	-3.1212955	0.40206876	-1.3997193	2.50811561	-0.4186853	-6.9077553	5	78	2.23519444	296.333333	23.33333333	0	0	0	0	30.519848	1	0.21141735
0.0441	1493.91412	0	0	0	0	-3.1212955	0.40206876	-6.9077553	-6.9077553	-6.9077553	-6.9077553	6	76	2.68223333	295.777778	22.7777778	0	0	0	0	30.519848	1	0.21141735
0.0864	1669.3692	0.29222511	23.6204964	1.11256815	0	-2.4487676	0.51304468	-1.2268147	3.16215716	0.1075694	-6.9077553	7	69	3.12927222	296.888889	23.88888888	0	0	0	0	30.519848	1	0.21141735
0.1683	2367.90879	0.47679612	66.235813	1.56809092	0	-1.7820072	0.86242942	-0.7385712	4.1932364	0.45049642	-6.9077553	8	63	3.12927222	298	25	0	0	0	0	30.519848	1	0.21141735
0.3726	5086.71676	0.75031741	154.103662	2.84495323	0	-0.9872498	1.62682916	-0.2859271	5.03763199	1.04589806	-6.9077553	9	59	4.47038889	299.111111	26.11111111	0	0	0	1	30.519848	1	0.21141735
0.6219	8483.18205	0.6821299	124.5263	2.03541053	0	-0.474976	2.13820349	-0.3810703	4.82452497	0.71118871	-6.9077553	10	54	3.57631111	300.777778	27.7777778	0	0	0	1	30.519848	1	0.21141735
1.0431	16576.5005	0.81601187	191.914029	2.58673017	1	0.04219705	2.80804638	-0.2021017	5.25705272	0.95078111	0.0009995	11	50	4.91742778	301.333333	28.33333333	0	0	0	1	30.519848	1	0.21141735
1.5543	27130.8826	0.85624684	225.225655	2.20267755	2	0.44102528	3.30070952	-0.1540294	5.41710725	0.79012758	0.69364706	12	49	4.02335	302.444444	29.44444444	0	0	0	1	30.519848	1	0.21141735
2.304	38885.2627	0.9499286	260.286427	3.26645209	2	0.83464674	3.66064105	-0.0503163	5.56178651	1.1840105	0.69364706	13	46	4.47038889	303	30	0	0	0	1	30.519848	1	0.21141735
3.1032	50313.3019	0.88431281	228.377534	2.68405921	2	1.13243384	3.91828937	-0.1218142	5.43100449	0.98770278	0.69364706	14	44	4.47038889	303.555556	30.55555555	0	0	0	1	30.519848	1	0.21141735
3.7971	61048.1451	0.86848753	219.078571	2.34886508	2	1.33423762	4.1116792	-0.1398513	5.389435	0.85435791	0.69364706	15	42	4.47038889	303.555556	30.55555555	0	0	0	1	30.519848	1	0.21141735
4.4649	76869.311	0.90059775	234.386877	1.92477672	2	1.49624682	4.34211973	-0.1035868	5.45697734	0.65532938	0.69364706	16	41	4.47038889	303.555556	30.55555555	0	0	0	1	30.519848	1	0.21141735
5.0661	91875.6861	0.87162077	216.206353	1.97530963	2	1.62257129	4.52044731	-0.1362542	5.37623791	0.68123128	0.69364706	17	44	4.02335	302.444444	29.44444444	0	0	0	1	30.519848	1	0.21141735
5.6241	106493.395	0.82637024	202.191633	2.06826605	2	1.72706094	4.66809236	-0.189503	5.30922087	0.72719398	0.69364706	18	47	3.12927222	301.888889	28.88888888	0	0	0	1	30.519848	1	0.21141735
6.03	118720.712	0.76638388	183.658623	1.4314425	2	1.79674701	4.7767822	-0.2647681	5.21308417	0.35938103	0.69364706	19	52	2.23519444	300.222222	27.2222222	0	0	0	1	30.519848	1	0.21141735

6.2631	125923.295	0.77137161	169.182809	1.10896637	2	1.83467527	4.83568089	-0.2582895	5.13098575	0.10432972	0.69364706	20	63	0.89407778	298	25	0	0	0	1	30.519848	1	0.21141735
6.444	133403.954	0.81901664	179.677627	1.30108506	2	1.86314947	4.89338927	-0.1984306	5.19116985	0.26396687	0.69364706	21	76	0.44703889	295.222222	22.2222222	0	0	0	1	30.519848	1	0.21141735
6.4818	135246.241	0.39033249	36.9283593	0.66463916	2	1.86899825	4.90710452	-0.9381977	3.60900688	-0.4070076	0.69364706	22	77	0.89407778	294.666667	21.66666666	0	0	0	1	30.519848	1	0.21141735
6.4818	135246.241	0	0	0	2	1.86899825	4.90710452	-6.9077553	-6.9077553	-6.9077553	0.69364706	23	77	0.89407778	294.666667	21.66666666	0	0	0	1	30.519848	1	0.21141735
0.0171	699.638207	0.94434526	247.059814	1.80258751	0	-4.0686768	-0.3557636	-0.0562051	5.50963452	0.58977774	-6.9077553	0	90	1.78815556	297.444444	24.44444444	0	0	0	0	34.0724638	1	0.49277013
0.027	1006.68476	0.45204743	51.2221347	0.70309665	0	-3.6119184	0.00765538	-0.7917584	3.93619128	-0.3508396	-6.9077553	1	90	2.23519444	296.888889	23.88888888	0	0	0	0	34.0724638	1	0.49277013
0.0405	1445.32262	0.45662839	48.802889	0.65429516	0	-3.2064533	0.36902421	-0.7816978	3.88781	-0.4226695	-6.9077553	2	84	1.78815556	296.888889	23.88888888	0	0	0	0	34.0724638	1	0.49277013
0.0504	1796.23282	0.45395825	47.5694303	0.5675771	0	-2.9877641	0.58624816	-0.7875496	3.86221136	-0.5646184	-6.9077553	3	85	2.23519444	296.888889	23.88888888	0	0	0	0	34.0724638	1	0.49277013
0.063	2234.87057	0.47340768	55.690652	0.6625279	0	-2.7646206	0.80463067	-0.7456882	4.01983026	-0.4101844	-6.9077553	4	81	2.23519444	296.888889	23.88888888	0	0	0	0	34.0724638	1	0.49277013
0.0828	3068.28233	0.53930278	71.8615889	0.87545352	0	-2.4913272	1.12144377	-0.6156256	4.27475581	-0.1318716	-6.9077553	5	78	2.23519444	296.333333	23.33333333	0	0	0	0	34.0724638	1	0.49277013
0.1152	4596.9567	0.70412678	132.753974	1.0942457	0	-2.1610855	1.52561201	-0.3493777	4.88850513	0.09097872	-6.9077553	6	76	2.68223333	295.777778	22.7777778	0	0	0	0	34.0724638	1	0.49277013
0.1755	7459.753	0.81291728	178.927268	1.28014431	0	-1.7401162	2.00965635	-0.2058965	5.18698499	0.24775367	-6.9077553	7	69	3.12927222	296.888889	23.88888888	0	0	0	0	34.0724638	1	0.49277013
0.2592	10592.4314	0.79248621	173.337198	1.38787661	0	-1.3501553	2.36023413	-0.2313191	5.15524459	0.32849523	-6.9077553	8	63	3.12927222	298	25	0	0	0	0	34.0724638	1	0.49277013
0.3861	15077.2151	0.91151991	235.527427	1.98929309	0	-0.9516589	2.71325099	-0.0915454	5.46183161	0.68828191	-6.9077553	9	59	4.47038889	299.111111	26.11111111	0	0	0	1	34.0724638	1	0.49277013
0.6255	21470.6771	1.01767886	297.148966	2.3084033	0	-0.4692039	3.06673472	0.01850655	5.69423695	0.83698918	-6.9077553	10	54	3.57631111	300.777778	27.7777778	0	0	0	1	34.0724638	1	0.49277013
0.9387	28929.2757	1.0680563	336.395856	2.4492854	2	-0.0632593	3.36488865	0.0667763	5.81829158	0.89620451	0.69364706	11	50	4.91742778	301.333333	28.33333333	0	0	0	1	34.0724638	1	0.49277013
1.3428	37649.4325	1.02867674	302.235941	2.39374717	2	0.29475699	3.62834444	0.0292449	5.71121128	0.87327766	0.69364706	12	49	4.02335	302.444444	29.44444444	0	0	0	1	34.0724638	1	0.49277013
1.7946	49413.259	0.96672696	275.722983	2.37690692	2	0.58478216	3.90023903	-0.0328053	5.6194003	0.86622066	0.69364706	13	46	4.47038889	303	30	0	0	0	1	34.0724638	1	0.49277013
2.4174	64421.4718	0.90479027	237.186958	2.52324487	2	0.88269258	4.16546251	-0.0989475	5.4688529	0.92594196	0.69364706	14	44	4.47038889	303.555556	30.55555555	0	0	0	1	34.0724638	1	0.49277013
3.348	88682.2801	0.9187285	250.374547	2.84307448	2	1.20836315	4.48507137	-0.0836768	5.52296198	1.0452377	0.69364706	15	42	4.47038889	303.555556	30.55555555	0	0	0	1	34.0724638	1	0.49277013
4.2867	118044.487	0.91113667	245.70287	2.43232132	2	1.45551721	4.77107004	-0.0919654	5.50412703	0.88925712	0.69364706	16	41	4.47038889	303.555556	30.55555555	0	0	0	1	34.0724638	1	0.49277013
5.4657	153585.354	0.92123725	258.364122	2.52087179	2	1.6984922	5.03426298	-0.0809528	5.55437379	0.9250014	0.69364706	17	44	4.02335	302.444444	29.44444444	0	0	0	1	34.0724638	1	0.49277013
6.5223	186137.399	0.86494313	222.337095	2.04521218	2	1.87522707	5.22649048	-0.143936	5.40419918	0.71599037	0.69364706	18	47	3.12927222	301.888889	28.88888888	0	0	0	1	34.0724638	1	0.49277013
7.2594	212960.448	0.80701399	190.360844	1.54407707	2	1.98229718	5.36111115	-0.2131759	5.2489267	0.43507379	0.69364706	19	52	2.23519444	300.222222	27.2222222	0	0	0	1	34.0724638	1	0.49277013
7.7814	235649.718	0.84190179	197.180132	1.41685086	2	2.05173627	5.4623507	-0.1709048	5.28412276	0.34914225	0.69364706	20	63	0.89407778	298	25	0	0	0	1	34.0724638	1	0.49277013
8.1108	250748.304	0.80456084	173.955836	1.39047967	2	2.09319651	5.52445365	-0.2162165	5.1588072	0.33036769	0.69364706	21	76	0.44703889	295.222222	22.2222222	0	0	0	1	34.0724638	1	0.49277013
8.199	254389.005	0.32637822	28.4033228	0.58390972	2	2.1040122	5.53886854	-1.1166391	3.34654134	-0.5362978	0.69364706	22	77	0.89407778	294.666667	21.66666666	0	0	0	1	34.0724638	1	0.49277013
8.199	254389.005	0	0	0	2	2.1040122	5.53886854	-6.9077553	-6.9077553	-6.9077553	0.69364706	23	77	0.89407778	294.666667	21.66666666	0	0	0	1	34.0724638	1	0.49277013
0.0279	1522.24241	0.99208775	266.108317	1.82351189	0	-3.5791286	0.42084123	-0.0069363	5.58390719	0.60131249	-6.9077553	0	90	1.78815556	297.444444	24.44444444	0	0	0	0	42.288408	1	-0.4111751
0.0639	3276.79363	0.6410162	101.966486	1.04014606	0	-2.7504359	1.18717052	-0.4431417	4.62465399	0.04032209	-6.9077553	1	90	2.23519444	296.888889	23.88888888	0	0	0	0	42.288408	1	-0.4111751
0.1089	5469.98331	0.61328705	92.5659961	0.99215767	0	-2.2173252	1.69945836	-0.487293	4.52793266	-0.0068658	-6.9077553	2	84	1.78815556	296.888889	23.88888888	0	0	0	0	42.288408	1	-0.4111751
0.1683	8189.53719	0.60052299	87.9676995	0.9784539	0	-1.7820072	2.10297949	-0.5082905	4.47698106	-0.0207601	-6.9077553	3	85	2.23519444	296.888889	23.88888888	0	0	0	0	42.288408	1	-0.4111751
0.2349	11391.5936	0.62847653	99.4690535	1.14978192	0	-1.4485954	2.43296346	-0.4628667	4.59985663	0.14044164	-6.9077553	4	81	2.23519444	296.888889	23.88888888	0	0	0	0	42.288408	1	-0.4111751
0.3213	15502.5977	0.73190721	141.686707	1.40527948	0	-1.13538	2.74107211	-0.3107362	4.95362539	0.34094755	-6.9077553	5	78	2.23519444	296.333333	23.33333333	0	0	0	0	42.288408	1	-0.4111751
0.4581	22661.7559	0.9051861	222.636976	1.75540482	0	-0.7806678	3.12072287	-0.0985106	5.40554703	0.56326901	-6.9077553	6	76	2.68223333	295.777778	22.7777778	0	0	1	0	42.288408	1	-0.4111751
0.7272	35781.5354	0.96074827	270.329327	2.21545124	0	-0.3185537	3.57745994	-0.0390025	5.59964464	0.79590738	-6.9077553	7	69	3.12927222	296.888889	23.88888888	0	0	1	0	42.288408	1	-0.4111751
1.0845	45432.7741	0.81076596	187.380648	2.49945945	0	0.08111905	3.81625575	-0.2085432	5.23314743	0.91647449	-6.9077553	8	63	3.12927222	298	25	0	0	1	0	42.288408	1	-0.4111751
1.4958	59126.8662	0.83198531	186.393018	2.02725988	0	0.40266118	4.07970232	-0.1827393	5.22786281	0.70717822	-6.9077553	9	59	4.47038889	299.111111	26.11111111	0	0	1	0	42.288408	1	-0.4111751
1.9557	76189.1331	0.86896421	200.151052	1.86688877	0	0.67074819	4.33323197	-0.1393032	5.29907734	0.6248088	-6.9077553	10	54	3.57631111	300.777778	27.7777778	0	0	1	0	42.288408	1	-0.4111751
2.5992	102820.547	0.95964287	255.067657	2.42630282	0	0.95520371	4.63299493	-0.0401526	5.54153275	0.88678069	-6.9077553	11	50	4.91742778	301.333333	28.33333333	0	0	1	0	42.288408	1	-0.4111751
3.3777	127341.086	0.8724162	218.84729	2.45916614	0	1.217195	4.84687705	-0.1353431	5.38837875	0.90022888	-6.9077553	12	49	4.02335	302.444444	29.44444444	0	0	1	0	42.288408	1	-0.4111751
4.4964	147132.276	0.81858928	199.271747	3.50572974	0	1.50327708	4.99133881	-0.1989519	5.29467447	1.25468391	-6.9077553	13	46	4.47038889	303	30	0	0	1	0	42.288408	1	-0.4111751
5.5467	168641.374	0.77728685	176.819449	2.90753284	0	1.71320316	5.12778034	-0.2506601	5.1751348	1.06764877	-6.9077553	14	44	4.47038889	303.555556	30.55555555	0	0	1	0	42.288408	1	-0.4111751
6.5529	193579.58	0.76670278	172.555847	2.29403812	0	1.8799077	5.26569386	-0.2643526	5.15072673	0.83074945	-6.9077553	15	42	4.47038889	303.555556	30.55555555	0	0	1	0	42.288408	1	-0.4111751
7.5843	229205.551	0.82835529	207.400933	2.10029195	0	2.02608032	5.43462357	-0.1871066	5.33465861	0.74255237	-6.9077553	16	41	4.47038889	303.555556	30.55555555	0	0	1	0	42.288408	1	-0.4111751
8.6346	270270.022	0.87075526	224.530688	1.97176713	0	2.15577739	5.59942524	-0.1372466	5.41401684	0.67943719	-6.9077553	17	44	4.02335	302.444444	29.44444444	0	0	1	0	42.288408	1	-0.4111751
9.5202	305643.065	0.83132935	204.249635	1.60251351	0	2.25341586	5.72242124	-0.1835271	5.31934784	0.47219716	-6.9077553	18	47	3.12927222	301.888889	28.88888888	0	0	1	0	42.288408	1	-0.4111751
10.3113	339270.899	0.86316383	222.920293	1.62392748	0	2.33324038	5.82680185	-0.1459929	5.40681876	0.48546318	-6.9077553	19	52	2.23519444	300.222222	27.2222222	0	0	1	0	42.288408	1	-0.4111751
10.9125	367168.75	0.89979827	230.030735	1.62230002	0	2.38990892	5.90582428	-0.1044739	5.43821728	0.48446112	-6.9077553	20	63	0.89407778	298	25	0	0	1	0	42.288408	1	-0.4111751
11.2482	382840.814	0.85578489	196.97539	1.52931553	0	2.42020812	5.94762189	-0.1545684	5.28308387	0.42547394	-6.9077553	21	76	0.44703889	295.222222	22.2222222	0	0	1	0	42.288408	1	-0.4111751
11.34	386920.133	0.44613449	52.4917232	0.722202	0	2.4283363	5.95822088	-0.8048959	3.96067455	-0.3240667	-6.9077553	22	77	0.89407778	294.666667	21.66666666	0	0	1	0	42.288408	1	-0.4111751
11.34	386920.133	0	0	0	0	2.4283363	5.95822088	-6.9077553	-6.9077553	-6.9077553	-6.9077553	23	77	0.89407778	294.666667	21.66666666	0	0	1	0	42.288408	1	-0.4111751
0.0009	0	0	0	0	0	-7.0131158	-6.9077553	-6.9077553	-6.9077553	-6.9077553	-6.9077553	0	90	1.78815556	297.444444	24.44444444	0	0	0	0	18.9135952	1	-0.0428739
0.0009	0	0	0	0	0	-7.0131158	-6.9077553	-6.9077553	-6.9077553	-6.9077553	-6.9077553	1	90	2.23519444	296.888889	23.88888888	0	0	0	0	18.9135952	1	-0.0428739
0.0009	0	0	0	0	0	-7.0131158	-6.9077553	-6.9077553	-6.9077553	-6.9077553	-6.9077553	2	84	1.78815556	296.888889	23.88888888	0	0	0	0	18.9135952	1	-0.0428739
0.0009	0	0	0	0	0	-7.0131158	-6.9077553	-6.9077553	-6.9077553	-6.9077553	-6.9077553	3	85	2.23519444	296.888889	23.88888888	0	0	0	0	18.9135952	1	-0.0428739
0.0009	0	0	0	0	0	-7.0131158	-6.9077553	-6.9077553	-6.9077553	-6.9077553	-6.9077553	4	81	2.23519444	296.888889	23.88888888	0	0	0	0	18.9135952	1	-0.0428739
0.0009	0	0	0	0	0	-7.0131158	-6.9077553	-6.9077553	-6.9077553	-6.9077553	-6.9077553	5	78	2.23519444	296.333333	23.33333333	0	0	0	0	18.9135952	1	-0.0428739
0.0009	0	0	0	0	0	-7.0131158	-6.9077553	-6.9077553	-6.9077553	-6.9077553	-6.9077553	6	76	2.68223333	295.777778	22.7777778	0	0	0	0	18.9135952	1	-0.0428739
0.0009	0	0	0	0	0	-7.0131158	-6.9077553	-6.9077553	-6.9077553	-6.9077553	-6.9077553	7	69	3.12927222	296.888889	23.88888888	0	0	0	0	18.9135952	1	-0.0428739
0.0162	0	0.37601342	43.2027265	1.42546878	0	-4.122744	-6.9077553	-0.9754745	3.76592675	0.35520201	-6.9077553	8	63	3.12927222	298	25	0	0	0	0	18.9135952	1	-0.0428739
0.1557	264.066862	0.63213403	108.463029	2.97198801	0	-1.8598242	-1.3277732	-0.4570731	4.68641859	1.08956751	-6.9077553	9	59	4.47038889	299.111111	26.11111111	0	0	1	0	18.9135952	1	-0.0428739

0.3249	966.671107	0.52395727	75.9270123	1.92860218	0	-1.1242378	-0.032863	-0.6444384	4.32978569	0.65731386	-6.9077553	10	54	3.57631111	300.777778	27.7777778	0	0	1	0	18.9135952	1	-0.0428739
0.6471	3897.32961	0.65240031	128.317585	3.22589589	0	-0.4352544	1.36054816	-0.4255653	4.85451611	1.17152065	-6.9077553	11	50	4.91742778	301.333333	28.33333333	0	0	1	0	18.9135952	1	-0.0428739
1.0161	9505.81083	0.67417811	137.62722	2.55794291	0	0.01597177	2.25200847	-0.3927788	4.92455599	0.93959425	-6.9077553	12	49	4.02335	302.444444	29.44444444	0	0	1	0	18.9135952	1	-0.0428739
1.494	15771.6353	0.65587327	132.117585	2.27707892	0	0.40145709	2.7582765	-0.4202642	4.88369989	0.82333251	-6.9077553	13	46	4.47038889	303	30	0	0	1	0	18.9135952	1	-0.0428739
2.1969	27797.8521	0.75581733	174.113556	2.7311052	0	0.78704728	3.32499473	-0.2786334	5.15971345	1.00507245	-6.9077553	14	44	4.47038889	303.555556	30.55555555	0	0	1	0	18.9135952	1	-0.0428739
3.1896	58471.441	0.90229312	251.106584	3.2076955	0	1.15989552	4.06855555	-0.1017082	5.52588147	1.16586447	-6.9077553	15	42	4.47038889	303.555556	30.55555555	0	0	1	0	18.9135952	1	-0.0428739
4.014	95063.9614	0.98242874	287.748669	2.68339545	0	1.38978825	4.55456046	-0.0167101	5.6620909	0.98745555	-6.9077553	16	41	4.47038889	303.555556	30.55555555	0	0	1	0	18.9135952	1	-0.0428739
4.9311	138239.996	0.98749696	282.198878	2.388928	0	1.59556209	4.92899851	-0.0115697	5.64261561	0.87126324	-6.9077553	17	44	4.02335	302.444444	29.44444444	0	0	1	0	18.9135952	1	-0.0428739
5.8167	182929.59	0.99993078	281.291032	2.17121453	0	1.76073309	5.20910679	0.00093034	5.63939339	0.77574717	-6.9077553	18	47	3.12927222	301.888889	28.88888888	0	0	1	0	18.9135952	1	-0.0428739
6.5898	219160.811	0.91712818	236.209601	1.70183296	0	1.885523	5.38981032	-0.0854183	5.46472378	0.53229331	-6.9077553	19	52	2.23519444	300.222222	27.2222222	0	0	1	0	18.9135952	1	-0.0428739
7.2216	251080.873	0.94529346	245.874332	1.71816451	0	1.97707654	5.52577907	-0.0552025	5.50482463	0.54183843	-6.9077553	20	63	0.89407778	298	25	0	0	1	0	18.9135952	1	-0.0428739
7.6896	273635.027	0.85017935	192.454718	1.48654104	0	2.03986877	5.61179885	-0.1611324	5.25986609	0.39712445	-6.9077553	21	76	0.44703889	295.222222	22.2222222	0	0	1	0	18.9135952	1	-0.0428739
7.8264	279819.81	0.44064652	50.2677002	0.72737006	0	2.05750263	5.63414944	-0.8172454	3.91738262	-0.316946	-6.9077553	22	77	0.89407778	294.666667	21.66666666	0	0	1	0	18.9135952	1	-0.0428739
7.8264	279819.81	0	0	0	0	2.05750263	5.63414944	-6.9077553	-6.9077553	-6.9077553	-6.9077553	23	77	0.89407778	294.666667	21.66666666	0	0	1	0	18.9135952	1	-0.0428739
0.0135	405.913009	0.73587445	164.059344	1.4021537	0	-4.3050656	-0.8991559	-0.3053378	5.10023431	0.33872234	-6.9077553	0	58	2.68223333	305.777778	32.7777778	0	0	0	0	30.519848	1	0.21141735
0.0333	1193.01289	0.90635716	222.109686	1.57342408	0	-3.4021979	0.17731981	-0.0972191	5.40317584	0.45388954	-6.9077553	1	65	1.78815556	304.111111	31.11111111	0	0	0	0	30.519848	1	0.21141735
0.0432	1456.30939	0.69909893	133.435736	1.52033875	0	-3.1419148	0.37659185	-0.3565336	4.89362747	0.41959071	-6.9077553	2	70	1.78815556	303	30	0	0	0	0	30.519848	1	0.21141735
0.0441	1500.1731	0.59729022	84.7243042	1.03055334	0	-3.1212955	0.40624687	-0.5136793	4.43941431	0.03106576	-6.9077553	3	77	0.44703889	301.888889	28.88888888	0	0	0	0	30.519848	1	0.21141735
0.0441	1500.1731	0	0	0	0	-3.1212955	0.40624687	-6.9077553	-6.9077553	-6.9077553	-6.9077553	4	75	0.89407778	297.444444	24.44444444	0	0	0	0	30.519848	1	0.21141735
0.0441	1500.1731	0	0	0	0	-3.1212955	0.40624687	-6.9077553	-6.9077553	-6.9077553	-6.9077553	5	88	0.89407778	295.222222	22.2222222	0	0	0	0	30.519848	1	0.21141735
0.0441	1500.1731	0	0	0	0	-3.1212955	0.40624687	-6.9077553	-6.9077553	-6.9077553	-6.9077553	6	46	2.23519444	296.888889	23.88888888	0	0	0	0	30.519848	1	0.21141735
0.0891	1763.35575	0.33974792	37.835293	1.14640816	0	-2.4179959	0.56778561	-1.0766123	3.63326877	0.13750562	-6.9077553	7	42	2.23519444	296.333333	23.33333333	0	0	0	0	30.519848	1	0.21141735
0.153	2465.17629	0.39423883	45.083613	1.18236907	0	-1.8773174	0.90266889	-0.9282651	3.80854101	0.16836552	-6.9077553	8	35	3.12927222	295.777778	22.7777778	0	0	0	0	30.519848	1	0.21141735
0.3348	3815.84589	0.67623463	125.384369	3.01195594	0	-1.0942219	1.3394244	-0.3897375	4.83139195	1.10292164	-6.9077553	9	36	4.91742778	294.666667	21.66666666	0	0	0	0	30.519848	1	0.21141735
0.4761	5349.75585	0.52789025	76.053854	1.35595023	0	-0.7421274	1.67723783	-0.6369743	4.33145484	0.3052397	-6.9077553	10	43	2.68223333	293	20	0	0	0	1	30.519848	1	0.21141735
0.5823	7053.33275	0.46898392	64.9421428	0.83158862	0	-0.5407695	1.953642	-0.7550568	4.17351216	-0.1832156	-6.9077553	11	54	0.44703889	290.222222	17.2222222	0	0	0	1	30.519848	1	0.21141735
0.6921	8646.73651	0.48924718	70.050429	0.91072658	0	-0.3680248	2.15729761	-0.7128456	4.24922967	-0.0924151	-6.9077553	12	55	1.34111667	291.333333	18.33333333	0	0	0	1	30.519848	1	0.21141735
0.9594	11285.4055	0.5957096	100.243792	1.77018352	0	-0.0414472	2.42359895	-0.5163247	4.60761512	0.57164798	-6.9077553	13	46	3.12927222	294.111111	21.11111111	0	0	0	1	30.519848	1	0.21141735
1.7406	18450.7425	0.99890545	304.850446	4.51212518	0	0.55422988	2.91515881	-9.46E-05	5.7198246	1.50698986	-6.9077553	14	40	5.36446667	295.777778	22.7777778	0	0	0	1	30.519848	1	0.21141735
2.7432	28917.1438	0.96211032	270.430319	4.03726454	0	1.00912512	3.36446921	-0.0375873	5.60001816	1.39581503	-6.9077553	15	35	6.25854444	297.444444	24.44444444	0	0	0	1	30.519848	1	0.21141735
3.8349	48343.4026	1.02293976	314.649938	3.19131467	0	1.34414336	3.87835045	0.0236577	5.75146389	1.16074626	-6.9077553	16	31	6.70558333	299.111111	26.11111111	0	0	0	1	30.519848	1	0.21141735
4.9896	131440.27	1.17014851	384.974474	2.3125293	60	1.60735575	4.87856014	0.1579849	5.95317963	0.8387742	4.09436123	17	27	6.25854444	300.777778	27.7777778	0	0	0	1	30.519848	1	0.21141735
6.1191	224399.741	1.21567393	423.540886	3.16095832	138	1.81141503	5.41343347	0.19612084	6.04865241	1.15119156	4.92726093	18	24	7.59966111	301.888889	28.88888888	0	0	0	1	30.519848	1	0.21141735
7.1388	454707.34	1.75509797	775.734775	3.108011	341	1.96554469	6.1196562	0.56309429	6.65381197	1.13430467	5.83188541	19	23	5.36446667	303.555556	30.55555555	0	0	0	1	30.519848	1	0.21141735
8.811	1275787.47	2.8717857	1463.58315	4.32613745	1119	2.17600094	7.15131967	1.05528219	7.28864361	1.46490623	7.0201916	20	23	5.81150556	303.555556	30.55555555	0	0	0	1	30.519848	1	0.21141735
11.3805	2042225.78	2.51354292	1308.13161	6.11870759	1912	2.43190136	7.62179605	0.92209105	7.17635591	1.81151432	7.55590562	21	22	6.25854444	304.111111	31.11111111	0	0	0	1	30.519848	1	0.21141735
12.6261	2225913.16	1.67938197	751.995033	2.85765155	2109	2.5357661	7.70792297	0.51902113	6.62273105	1.05035003	7.65396965	22	23	5.36446667	304.111111	31.11111111	0	0	0	1	30.519848	1	0.21141735
13.6458	2240555.69	0.91999966	258.575905	2.31187855	2109	2.61343178	7.71447964	-0.0822956	5.55519315	0.83849287	7.65396965	23	24	4.91742778	303.555556	30.55555555	0	0	0	1	30.519848	1	0.21141735
0.0351	1304.83236	0.97841855	272.076455	2.61131487	0	-3.3495541	0.26684066	-0.0207962	5.60608679	0.96023675	-6.9077553	0	58	2.68223333	305.777778	32.7777778	0	0	0	0	34.0724638	1	0.49277013
0.0882	3621.50722	0.84407043	193.157591	1.32504557	0	-2.4281483	1.28716639	-0.1683353	5.26351156	0.28220126	-6.9077553	1	65	1.78815556	304.111111	31.11111111	0	0	0	0	34.0724638	1	0.49277013
0.1521	6609.71318	0.7565817	145.464706	1.05146661	0	-1.8832171	1.88869154	-0.2776239	4.97994036	0.05113656	-6.9077553	2	70	1.78815556	303	30	0	0	0	0	34.0724638	1	0.49277013
0.1953	8364.26469	0.64807947	103.648631	0.85381222	0	-1.6332184	2.12408798	-0.4322001	4.64101628	-0.1568735	-6.9077553	3	77	0.44703889	301.888889	28.88888888	0	0	0	0	34.0724638	1	0.49277013
0.2241	9636.31452	0.52296758	64.5176392	0.68596601	0	-1.4956629	2.26564249	-0.6463255	4.16695416	-0.3754705	-6.9077553	4	75	0.89407778	297.444444	24.44444444	0	0	0	0	34.0724638	1	0.49277013
0.2448	10250.4074	0.44102636	46.2090746	0.59086825	0	-1.4073137	2.327415	-0.8163858	3.83319784	-0.5244712	-6.9077553	5	88	0.89407778	295.222222	22.2222222	0	0	0	0	34.0724638	1	0.49277013
0.2664	11039.9549	0.30070571	26.8159205	0.51622951	0	-1.3227563	2.40161153	-1.1983032	3.28903305	-0.6592686	-6.9077553	6	46	2.23519444	296.888889	23.88888888	0	0	0	0	34.0724638	1	0.49277013
0.3717	14813.5799	0.83080742	202.484375	1.65751257	0	-0.9896682	2.69561183	-0.1841543	5.31066766	0.50592116	-6.9077553	7	42	2.23519444	296.333333	23.33333333	0	0	0	1	34.0724638	1	0.49277013
0.5121	20697.5571	0.88738388	218.155749	1.64298796	0	-0.6692354	3.03006399	-0.1183513	5.38521384	0.49712497	-6.9077553	8	35	3.12927222	295.777778	22.7777778	0	0	0	1	34.0724638	1	0.49277013
0.774	29525.9903	0.95289869	259.226106	2.25354885	0	-0.2561834	3.38530477	-0.0471978	5.55770454	0.81294989	-6.9077553	9	36	4.91742778	294.666667	21.66666666	0	0	0	1	34.0724638	1	0.49277013
1.0332	41032.9089	0.9406431	249.094293	1.85213958	0	0.03266078	3.71439877	-0.060129	5.51783552	0.61688127	-6.9077553	10	43	2.68223333	293	20	0	0	0	1	34.0724638	1	0.49277013
1.2726	49954.3232	0.85229194	209.637453	1.56832037	0	0.24106205	3.91112907	-0.1586535	5.34538439	0.45064264	-6.9077553	11	54	0.44703889	290.222222	17.2222222	0	0	0	1	34.0724638	1	0.49277013
1.467	55622.8952	0.69049694	138.300239	1.14762243	0	0.3832195	4.01861288	-0.3688966	4.9294342	0.13856333	-6.9077553	12	55	1.34111667	291.333333	18.33333333	0	0	0	1	34.0724638	1	0.49277013
1.8387	67739.5481	0.88616397	221.415727	1.85986033	0	0.6090588	4.21568494	-0.1197255	5.40004657	0.62103892	-6.9077553	13	46	3.12927222	294.111111	21.11111111	0	0	0	1	34.0724638	1	0.49277013
2.4417	91478.8852	1.02363864	296.987873	2.6105671	0	0.89269452	4.51611911	0.02434001	5.69369467	0.95995046	-6.9077553	14	40	5.36446667	295.777778	22.7777778	0	0	0	1	34.0724638	1	0.49277013
3.3318	122652.333	1.06305493	317.94344	3.00882658	0	1.2035127	4.80936195	0.06208702	5.76187665	1.10188246	-6.9077553	15	35	6.25854444	297.444444	24.44444444	0	0	0	1	34.0724638	1	0.49277013
4.5036	164958.848	1.06476308	327.28749	3.11242207	0	1.50487708	5.1057021	0.06369105	5.79084202	1.13572247	-6.9077553	16	31	6.70558333	299.111111	26.11111111	0	0	0	1	34.0724638	1	0.49277013
5.7681	207646.071	1.05530645	318.738901	3.14238073	0	1.75234274	5.33583987	0.05477834	5.76437541	1.14529889	-6.9077553	17	27	6.25854444	300.777778	27.7777778	0	0	0	1	34.0724638	1	0.49277013
7.0659	265555.603	1.15094578	397.553571	3.27552573	1	1.9552804	5.58182801	0.1414525	5.98533221	1.18678363	0.0009995	18	24	7.59966111	301.888889	28.88888888	0	0	0	1	34.0724638	1	0.49277013
8.496	315315.297	1.08291824	348.118156	3.57913168	1	2.13959546	5.75357625	0.08058247	5.85254482	1.27539958	0.0009995	19	23	5.36446667	303.555556	30.55555555	0	0	0	1	34.0724638	1	0.49277013
10.0971	368096.227	0.96370655	285.706805	3.35919522	1	2.31224825	5.90834711	-0.0359313	5.65496963	1.21199907	0.0009995	20	23	5.81150556	303.555556	30.55555555	0	0	0	1	34.0724638	1	0.49277013
11.691	415994.501	0.95885493	292.046196	3.51530463	1	2.45881932	6.03067444	-0.0409731	5.67691542	1.25741062	0.0009995	21	22	6.25854444	304.111111	31.11111111	0	0	0	1	34.0724638	1	0.49277013
13.1859	455171.371	0.89762887	255.83248	3.20975575	1	2.57914808	6.12067618	-0.1068852	5.54452677	1.16650634	0.0009995	22	23	5.36446667	304.111111	31.11111111	0	0	0	1	34.0724638	1	0.49277013
14.7483	507017.711	0.95233773	282.641381	3.15101195	2	2.69112782	6.22854791	-0.0477861	5.64418243	1.14804096	0.69364706	23	24	4.91742778	303.555556	30.55555555	0	0	0	1	34.0724638	1	0.49277013

0.0405	2863.92042	1.23404494	423.64138	2.67524234	0	-3.2064533	1.05254057	0.21110736	6.04888966	0.9844137	-6.9077553	0	58	2.68223333	305.777778	32.7777778	0	0	0	0	42.288408	1	-0.4111751
0.126	7712.91099	0.98620067	270.814947	1.9204797	0	-2.0714734	2.04302532	-0.0128819	5.60143943	0.65309557	-6.9077553	1	65	1.78815556	304.111111	31.11111111	0	0	0	0	42.288408	1	-0.4111751
0.2205	12709.1105	0.94986914	242.257496	1.80819179	0	-1.5118576	2.54239778	-0.0503788	5.49000532	0.59288022	-6.9077553	2	70	1.78815556	303	30	0	0	0	0	42.288408	1	-0.4111751
0.3204	17502.6786	0.87128888	201.433347	1.71661487	0	-1.1381851	2.86241106	-0.1366346	5.30546351	0.54093662	-6.9077553	3	77	0.44703889	301.888889	28.88888888	0	0	0	0	42.288408	1	-0.4111751
0.3942	20967.919	0.68023561	117.138934	1.28216227	0	-0.9308969	3.04304129	-0.3838471	4.76336924	0.24932756	-6.9077553	4	75	0.89407778	297.444444	24.44444444	0	0	0	0	42.288408	1	-0.4111751
0.4617	24126.1117	0.56705205	81.7321549	0.9940033	0	-0.7728399	3.18333617	-0.5655422	4.40345973	-0.0050092	-6.9077553	5	88	0.89407778	295.222222	22.2222222	0	0	1	0	42.288408	1	-0.4111751
0.5373	27656.5357	0.54998875	102.969285	1.00054023	0	-0.6211987	3.31989823	-0.5960409	4.63444045	0.00153905	-6.9077553	6	46	2.23519444	296.888889	23.88888888	0	0	1	0	42.288408	1	-0.4111751
0.8721	41063.52	0.8689147	228.8771	1.84485175	0	-0.1368512	3.71514449	-0.1393601	5.43318955	0.61294082	-6.9077553	7	42	2.23519444	296.333333	23.33333333	0	0	1	0	42.288408	1	-0.4111751
1.2231	52877.3288	0.81882465	188.317268	1.83496062	0	0.20138862	3.96799359	-0.1986648	5.23813344	0.60756785	-6.9077553	8	35	3.12927222	295.777778	22.7777778	0	0	1	0	42.288408	1	-0.4111751
1.6074	65820.1013	0.79241507	166.304267	1.89375795	0	0.47461797	4.18694048	-0.2314088	5.11382506	0.6390911	-6.9077553	9	36	4.91742778	294.666667	21.66666666	0	0	1	0	42.288408	1	-0.4111751
2.0043	80997.2936	0.84069907	197.117382	1.72260006	0	0.69529487	4.39442809	-0.1723327	5.28380447	0.54441516	-6.9077553	10	43	2.68223333	293	20	0	0	1	0	42.288408	1	-0.4111751
2.3157	93266.8829	0.75261322	157.406668	1.24134148	0	0.83971202	4.53547581	-0.282876	5.05883905	0.21699789	-6.9077553	11	54	0.44703889	290.222222	17.2222222	0	0	1	0	42.288408	1	-0.4111751
2.7009	109453.249	0.81765662	186.801749	1.38217962	0	0.99358505	4.69550664	-0.2000906	5.23005324	0.32438492	-6.9077553	12	55	1.34111667	291.333333	18.33333333	0	0	1	0	42.288408	1	-0.4111751
3.3678	133071.016	0.85558703	206.830634	2.04145218	0	1.21425971	4.89089045	-0.1547994	5.3319051	0.71415113	-6.9077553	13	46	3.12927222	294.111111	21.11111111	0	0	1	0	42.288408	1	-0.4111751
4.5612	158811.433	0.8078732	196.433494	3.21707526	0	1.51758575	5.06772384	-0.2121131	5.28032901	1.16878344	-6.9077553	14	40	5.36446667	295.777778	22.7777778	0	0	1	0	42.288408	1	-0.4111751
6.0876	188339.802	0.85000481	219.318765	4.0911491	0	1.80625392	5.2382531	-0.1613375	5.39053078	1.40907028	-6.9077553	15	35	6.25854444	297.444444	24.44444444	0	0	1	0	42.288408	1	-0.4111751
7.8534	241730.507	1.03299868	337.833142	4.45679831	0	2.06094656	5.48782764	0.0334335	5.82255507	1.49465499	-6.9077553	16	31	6.70558333	299.111111	26.11111111	0	0	1	0	42.288408	1	-0.4111751
9.3168	312262.483	1.19449269	437.678083	4.20274543	0	2.23181922	5.74384733	0.17855839	6.08148595	1.4359759	-6.9077553	17	27	6.25854444	300.777778	27.7777778	0	0	1	0	42.288408	1	-0.4111751
10.7982	388043.413	1.20626397	443.668666	3.85527296	0	2.37937945	5.9611198	0.18835662	6.09508029	1.34970116	-6.9077553	18	24	7.59966111	301.888889	28.88888888	0	0	1	0	42.288408	1	-0.4111751
12.3246	467267.552	1.21145472	435.469782	3.48250594	1	2.51159727	6.14690415	0.19264699	6.0764277	1.24803924	0.0009995	19	23	5.36446667	303.555556	30.55555555	0	0	1	0	42.288408	1	-0.4111751
14.0499	550406.806	1.20325618	435.115089	3.90704062	3	2.64261528	6.31065947	0.1858621	6.07561287	1.36303613	1.09894557	20	23	5.81150556	303.555556	30.55555555	0	0	1	0	42.288408	1	-0.4111751
15.5592	623101.111	1.14186004	393.845483	3.31183207	3	2.7446521	6.43471041	0.13353393	5.9759612	1.19780343	1.09894557	21	22	6.25854444	304.111111	31.11111111	0	0	1	0	42.288408	1	-0.4111751
17.0145	681340.084	1.03156193	314.433615	2.88187519	4	2.83406592	6.52406304	0.03204303	5.75077615	1.05878813	1.38654433	22	23	5.36446667	304.111111	31.11111111	0	0	1	0	42.288408	1	-0.4111751
18.3843	734010.514	1.01674417	309.057695	2.92554834	4	2.91149704	6.59852471	0.01758858	5.73353121	1.07382369	1.38654433	23	24	4.91742778	303.555556	30.55555555	0	0	1	0	42.288408	1	-0.4111751
0.0027	0	0.21041781	8.77035046	0.69165194	0	-5.9145035	-6.9077553	-1.553919	2.17149078	-0.3672277	-6.9077553	0	58	2.68223333	305.777778	32.7777778	0	0	0	0	18.9135952	1	-0.0428739
0.0036	0	0.06053963	0.5845502	0.09518638	0	-5.6268214	-6.9077553	-2.788074	-0.5352034	-2.3414676	-6.9077553	1	65	1.78815556	304.111111	31.11111111	0	0	0	0	18.9135952	1	-0.0428739
0.0036	0	0	0	0	0	-5.6268214	-6.9077553	-6.9077553	-6.9077553	-6.9077553	-6.9077553	2	70	1.78815556	303	30	0	0	0	0	18.9135952	1	-0.0428739
0.0036	0	0	0	0	0	-5.6268214	-6.9077553	-6.9077553	-6.9077553	-6.9077553	-6.9077553	3	77	0.44703889	301.888889	28.88888888	0	0	0	0	18.9135952	1	-0.0428739
0.0036	0	0	0	0	0	-5.6268214	-6.9077553	-6.9077553	-6.9077553	-6.9077553	-6.9077553	4	75	0.89407778	297.444444	24.44444444	0	0	0	0	18.9135952	1	-0.0428739
0.0036	0	0	0	0	0	-5.6268214	-6.9077553	-6.9077553	-6.9077553	-6.9077553	-6.9077553	5	88	0.89407778	295.222222	22.2222222	0	0	0	0	18.9135952	1	-0.0428739
0.0036	0	0	0	0	0	-5.6268214	-6.9077553	-6.9077553	-6.9077553	-6.9077553	-6.9077553	6	46	2.23519444	296.888889	23.88888888	0	0	0	0	18.9135952	1	-0.0428739
0.0396	0	0.41784134	45.2463715	1.57748966	0	-3.2289262	-6.9077553	-0.8702631	3.81214458	0.45646848	-6.9077553	7	42	2.23519444	296.333333	23.33333333	0	0	0	0	18.9135952	1	-0.0428739
0.1179	0	0.40626833	41.056433	1.66732876	0	-2.1379185	-6.9077553	-0.898283	3.71497189	0.51182238	-6.9077553	8	35	3.12927222	295.777778	22.7777778	0	0	0	0	18.9135952	1	-0.0428739
0.2484	175.455131	0.37389316	35.2373653	1.59876099	0	-1.3927149	-1.7346886	-0.9811142	3.56213541	0.46985423	-6.9077553	9	36	4.91742778	294.666667	21.66666666	0	0	1	0	18.9135952	1	-0.0428739
0.3411	745.684337	0.39291845	52.9767853	1.1290801	0	-1.0755796	-0.2921128	-0.9316114	3.96987268	0.12228851	-6.9077553	10	43	2.68223333	293	20	0	0	1	0	18.9135952	1	-0.0428739
0.4077	1831.41779	0.45117481	77.4083879	0.8129091	0	-0.8972237	0.60563629	-0.7936864	4.34910806	-0.2059066	-6.9077553	11	54	0.44703889	290.222222	17.2222222	0	0	1	0	18.9135952	1	-0.0428739
0.4941	4275.9398	0.61794285	128.343465	1.1212546	0	-0.7050174	1.45323775	-0.4797423	4.85471778	0.1153397	-6.9077553	12	55	1.34111667	291.333333	18.33333333	0	0	1	0	18.9135952	1	-0.0428739
0.7056	7309.48918	0.58115299	116.480086	1.62069261	0	-0.3487068	1.98931019	-0.541022	4.75772891	0.48347042	-6.9077553	13	46	3.12927222	294.111111	21.11111111	0	0	1	0	18.9135952	1	-0.0428739
1.1439	15419.2914	0.85301945	243.476819	3.37915641	0	0.13444348	2.73568426	-0.1578013	5.49502585	1.21792198	-6.9077553	14	40	5.36446667	295.777778	22.7777778	0	0	1	0	18.9135952	1	-0.0428739
1.7442	31689.1009	0.936345	273.585317	4.17994152	0	0.556296	3.45600436	-0.0647039	5.61161717	1.43053647	-6.9077553	15	35	6.25854444	297.444444	24.44444444	0	0	1	0	18.9135952	1	-0.0428739
2.5488	64507.1363	1.06836224	367.209522	3.92278821	0	0.93562266	4.16679136	0.06706244	5.90593531	1.36705757	-6.9077553	16	31	6.70558333	299.111111	26.11111111	0	0	1	0	18.9135952	1	-0.0428739
3.2553	94250.2383	1.04797332	338.25625	3.06566263	0	1.18028444	4.54596396	0.0478119	5.8238067	1.12058988	-6.9077553	17	27	6.25854444	300.777778	27.7777778	0	0	1	0	18.9135952	1	-0.0428739
4.2651	143280.687	1.20429283	455.526014	4.0976519	0	1.45046563	4.96481253	0.18672255	6.12145502	1.41065811	-6.9077553	18	24	7.59966111	301.888889	28.88888888	0	0	1	0	18.9135952	1	-0.0428739
5.2434	188789.015	1.09826458	356.891099	2.84312314	2	1.65697014	5.24063536	0.09464139	5.87743349	1.04525481	0.69364706	19	23	5.36446667	303.555556	30.55555555	0	0	1	0	18.9135952	1	-0.0428739
6.1965	234490.958	1.07039296	342.801759	2.94858523	2	1.82398462	5.45742129	0.06895963	5.83715523	1.08166456	0.69364706	20	23	5.81150556	303.555556	30.55555555	0	0	1	0	18.9135952	1	-0.0428739
7.2027	280888.595	1.09619512	367.417319	3.29848637	2	1.97445596	5.63796169	0.09275703	5.90650103	1.19376681	0.69364706	21	22	6.25854444	304.111111	31.11111111	0	0	1	0	18.9135952	1	-0.0428739
8.1549	323802.127	1.03285399	316.240784	2.6280243	2	2.09861897	5.7801357	0.03329356	5.75650706	0.96661279	0.69364706	22	23	5.36446667	304.111111	31.11111111	0	0	1	0	18.9135952	1	-0.0428739
9.0387	364080.374	1.01220566	295.098523	2.33331808	2	2.20151536	5.8973774	0.01311923	5.68731267	0.84771981	0.69364706	23	24	4.91742778	303.555556	30.55555555	0	0	1	0	18.9135952	1	-0.0428739
0.0171	417.674456	0.77956883	174.106839	1.64940113	0	-4.0686768	-0.8706616	-0.2477324	5.15967487	0.50101837	-6.9077553	0	71	3.57631111	302.444444	29.44444444	0	0	0	0	30.519848	1	0.21141735
0.0585	1451.36265	0.61607502	110.710651	1.32379126	0	-2.8387285	0.37319165	-0.4827647	4.70692908	0.2812549	-6.9077553	1	70	2.68223333	303	30	0	0	0	0	30.519848	1	0.21141735
0.1233	2533.62031	0.61942138	118.760851	1.31647873	0	-2.0931349	0.93004385	-0.4773564	4.77712023	0.27571986	-6.9077553	2	67	3.12927222	303.555556	30.55555555	0	0	0	0	30.519848	1	0.21141735
0.1908	2989.09943	0.4613118	62.2108073	1.12819285	0	-1.6565295	1.09530664	-0.7715157	4.13054481	0.12150309	-6.9077553	3	65	2.68223333	303.555556	30.55555555	0	0	0	0	30.519848	1	0.21141735
0.3456	4187.72091	0.59289342	100.877521	2.0576564	0	-1.0624732	1.43239542	-0.5210554	4.61391703	0.72205354	-6.9077553	4	67	4.02335	303	30	0	0	0	1	30.519848	1	0.21141735
0.5427	7969.2842	0.74716612	162.815693	2.13472103	0	-0.6111986	2.07572015	-0.2901302	5.09262498	0.75880431	-6.9077553	5	58	4.47038889	304.666667	31.66666666	0	0	0	1	30.519848	1	0.21141735
0.783	14276.9418	0.7856363	176.031016	2.63862524	0	-0.2446226	2.65871581	-0.2399893	5.17066589	0.97063695	-6.9077553	6	55	5.36446667	305.222222	32.2222222	0	0	0	1	30.519848	1	0.21141735
1.0044	21874.2104	0.70241305	139.080062	1.766708	0	0.00439035	3.08535405	-0.351811	4.93505694	0.56968379	-6.9077553	7	54	4.47038889	305.222222	32.2222222	0	0	0	1	30.519848	1	0.21141735
1.1403	30267.5814	0.68601848	141.560974	1.07398522	0	0.13129139	3.41011026	-0.3753941	4.9527376	0.07230691	-6.9077553	8	60	2.68223333	304.111111	31.11111111	0	0	0	1	30.519848	1	0.21141735
1.2456	38338.4899	0.80920925	175.908287	1.2768539	0	0.21961734	3.64648043	-0.2104627	5.16996845	0.24518203	-6.9077553	9	67	1.34111667	302.444444	29.44444444	0	0	0	1	30.519848	1	0.21141735
1.3194	44021.574	0.70430444	128.638291	1.0305628	0	0.27717709	3.78470255	-0.3491257	4.85701229	0.03107493	-6.9077553	10	73	2.23519444	300.777778	27.7777778	0	0	0	1	30.519848	1	0.21141735
1.3617	45819.9923	0.58704129	85.8677693	0.87291515	0	0.30873392	3.82474233	-0.5309581	4.45282019	-0.134772	-6.9077553	11	79	1.34111667	299.666667	26.66666666	0	0	0	1	30.519848	1	0.21141735
1.3851	46828.864	0.37008447	31.9687218	0.5179092	0	0.32577234	3.84652112	-0.9913256	3.46478926	-0.6560264	-6.9077553	12	83	1.34111667	299.111111	26.11111111	0	0	0	1	30.519848	1	0.21141735
1.3968	47223.6348	0.25254556	13.4461306	0.33946745	0	0.33418391	3.85491568	-1.3722118	2.59876575	-1.0774357	-6.9077553	13	85	2.68223333	298.555556	25.55555555	0	0	0	1	30.519848	1	0.21141735

1.4067	47574.5478	0.23060283	10.8264812	0.27602603	0	0.34124654	3.86231893	-1.4627313	2.38208746	-1.2836438	-6.9077553	14	86	2.68223333	298	25	0	0	0	1	30.519848	1	0.21141735
1.4184	48100.9105	0.21063557	9.45334743	0.26581968	0	0.34952948	3.8733219	-1.5528895	2.24647468	-1.3211822	-6.9077553	15	90	2.23519444	298	25	0	0	0	1	30.519848	1	0.21141735
1.4265	48495.6865	0.20984205	8.92454274	0.27950785	0	0.35522389	3.88149548	-1.556646	2.18891714	-1.2711536	-6.9077553	16	91	3.57631111	298	25	0	0	0	1	30.519848	1	0.21141735
1.4364	48978.1881	0.21184065	9.2400152	0.29220358	0	0.36213998	3.89139548	-1.5472115	2.22365175	-1.2268881	-6.9077553	17	90	3.12927222	298	25	0	0	0	1	30.519848	1	0.21141735
1.4454	49372.9636	0.19791912	9.19120941	0.33528938	0	0.3683861	3.89942323	-1.614857	2.21835632	-1.0897832	-6.9077553	18	91	2.68223333	298	25	0	0	0	1	30.519848	1	0.21141735
1.458	49987.0566	0.21384694	9.83789171	0.29639738	0	0.37706563	3.91178411	-1.5378294	2.28634307	-1.2126861	-6.9077553	19	93	1.34111667	298	25	0	0	0	1	30.519848	1	0.21141735
1.4697	50557.2848	0.22331181	10.9896733	0.34359484	0	0.3850583	3.92312683	-1.4947182	2.39704703	-1.0653859	-6.9077553	20	93	2.23519444	298	25	0	0	0	1	30.519848	1	0.21141735
1.4859	51171.3755	0.24946186	14.0031213	0.47169012	0	0.39602065	3.93519984	-1.3844486	2.63935166	-0.7493152	-6.9077553	21	88	3.12927222	299.111111	26.11111111	0	0	0	1	30.519848	1	0.21141735
1.6227	55748.6656	0.45270558	60.4300409	1.03821724	0	0.48409143	4.02087141	-0.7903068	4.10150289	0.03846778	-6.9077553	22	83	2.68223333	300.222222	27.2222222	0	0	0	1	30.519848	1	0.21141735
1.8207	61133.4231	0.53629563	78.3614169	1.44538172	0	0.59922104	4.1130751	-0.6212068	4.36134444	0.36906507	-6.9077553	23	80	4.02335	300.777778	27.7777778	0	0	0	1	30.519848	1	0.21141735
0.0378	1415.95716	1.03362823	301.403773	3.01411568	0	-3.2754462	0.34851172	0.03404217	5.70845412	1.1036382	-6.9077553	0	71	3.57631111	302.444444	29.44444444	0	0	0	0	34.0724638	1	0.49277013
0.1116	4772.54596	0.88718656	212.992854	1.43390293	0	-2.1928342	1.56308942	-0.1185735	5.36126331	0.3610972	-6.9077553	1	70	2.68223333	303	30	0	0	0	0	34.0724638	1	0.49277013
0.1854	8083.17866	0.85966808	188.90781	1.25835279	0	-1.6852396	2.0899089	-0.1500464	5.24126441	0.23059793	-6.9077553	2	67	3.12927222	303.555556	30.55555555	0	0	0	0	34.0724638	1	0.49277013
0.2646	10861.3619	0.79986832	167.929166	1.3319768	0	-1.329536	2.38530377	-0.2220587	5.12354821	0.28741464	-6.9077553	3	65	2.68223333	303.555556	30.55555555	0	0	0	1	34.0724638	1	0.49277013
0.4185	17885.1459	1.04033407	308.12098	2.74920404	0	-0.8710784	2.88402644	0.04050265	5.73049574	1.01167511	-6.9077553	4	67	4.02335	303	30	0	0	0	1	34.0724638	1	0.49277013
0.6966	33580.8709	1.22460938	424.757332	3.40738063	0	-0.3615439	3.51398636	0.20343817	6.05152038	1.22623729	-6.9077553	5	58	4.47038889	304.666667	31.66666666	0	0	0	1	34.0724638	1	0.49277013
1.1727	47734.6174	1.21031999	436.579631	3.84607787	0	0.15930878	3.86567782	0.19171067	6.07897308	1.34731387	-6.9077553	6	55	5.36446667	305.222222	32.2222222	0	0	0	1	34.0724638	1	0.49277013
1.6389	57174.8829	1.01188819	311.06992	2.60578477	0	0.49402529	4.04613218	0.01280584	5.74002093	0.95811757	-6.9077553	7	54	4.47038889	305.222222	32.2222222	0	0	0	1	34.0724638	1	0.49277013
1.9332	69788.669	0.8866887	224.271168	1.60173702	0	0.65917666	4.24548599	-0.1191342	5.41286035	0.4717128	-6.9077553	8	60	2.68223333	304.111111	31.11111111	0	0	0	1	34.0724638	1	0.49277013
2.1123	77084.843	0.84197477	189.925094	1.44236088	0	0.7477774	4.34491965	-0.1708183	5.24663502	0.36697434	-6.9077553	9	67	1.34111667	302.444444	29.44444444	0	0	0	1	34.0724638	1	0.49277013
2.3022	84954.94	0.75279041	151.20082	1.29734115	0	0.83386519	4.44213277	-0.2826409	5.0186155	0.26108741	-6.9077553	10	73	2.23519444	300.777778	27.7777778	0	0	0	1	34.0724638	1	0.49277013
2.4624	91579.0977	0.62001483	99.5102704	1.07648648	0	0.90113648	4.51721397	-0.4764003	4.60027091	0.074631	-6.9077553	11	79	1.34111667	299.666667	26.66666666	0	0	0	1	34.0724638	1	0.49277013
2.5479	95000.4772	0.3401468	28.6217722	0.57983354	0	0.93526949	4.55389244	-1.0754424	3.35420263	-0.5432911	-6.9077553	12	83	1.34111667	299.111111	26.11111111	0	0	0	1	34.0724638	1	0.49277013
2.6199	98290.2632	0.27771623	16.9421326	0.4759409	0	0.96313615	4.58793514	-1.2775611	2.82986259	-0.7403627	-6.9077553	13	85	2.68223333	298.555556	25.55555555	0	0	0	1	34.0724638	1	0.49277013
2.6991	101843.228	0.27019982	16.5194328	0.48462577	0	0.99291838	4.62344447	-1.3048994	2.80459796	-0.722317	-6.9077553	14	86	2.68223333	298	25	0	0	0	1	34.0724638	1	0.49277013
2.7756	105089.147	0.2657093	15.7269818	0.46366559	0	1.02086694	4.65481852	-1.321596	2.75544141	-0.7664373	-6.9077553	15	90	2.23519444	298	25	0	0	0	1	34.0724638	1	0.49277013
2.8431	108028.021	0.25977514	15.9705941	0.44066503	0	1.04489501	4.6823999	-1.3440968	2.77081177	-0.8172035	-6.9077553	16	91	3.57631111	298	25	0	0	0	1	34.0724638	1	0.49277013
2.916	110747.577	0.39759671	60.9665678	0.75725711	0	1.07021281	4.70726256	-0.9198051	4.11034205	-0.2767328	-6.9077553	17	90	3.12927222	298	25	0	0	0	1	34.0724638	1	0.49277013
2.9844	113467.132	0.27862152	22.1489499	0.49717973	0	1.09339872	4.73152202	-1.2743183	3.09783523	-0.6967944	-6.9077553	18	91	2.68223333	298	25	0	0	0	1	34.0724638	1	0.49277013
3.0393	115397.137	0.28941955	25.1074439	0.45435893	0	1.11162723	4.74838821	-1.2364287	3.2232042	-0.7866693	-6.9077553	19	93	1.34111667	298	25	0	0	0	1	34.0724638	1	0.49277013
3.0897	117195.56	0.25640282	17.3121338	0.45309819	0	1.128074	4.76385253	-1.357113	2.85146539	-0.7894418	-6.9077553	20	93	2.23519444	298	25	0	0	0	1	34.0724638	1	0.49277013
3.1797	120617.274	0.32843975	33.0605933	0.63107609	0	1.15678685	4.7926308	-1.1103618	3.49837228	-0.4587455	-6.9077553	21	88	3.12927222	299.111111	26.11111111	0	0	0	1	34.0724638	1	0.49277013
3.6369	130462.264	0.53799925	93.9067344	1.52942784	0	1.29113167	4.87109169	-0.6180411	4.54231275	0.42554733	-6.9077553	22	83	2.68223333	300.222222	27.2222222	0	0	0	1	34.0724638	1	0.49277013
4.4325	143191.178	0.70440239	152.469775	2.07130439	0	1.48896376	4.96418763	-0.3489869	5.02697294	0.72866122	-6.9077553	23	80	4.02335	300.777778	27.7777778	0	0	0	1	34.0724638	1	0.49277013
0.0477	3500.83836	1.28558335	465.295898	2.87464553	0	-3.0428239	1.25328808	0.25199014	6.14267569	1.05627718	-6.9077553	0	71	3.57631111	302.444444	29.44444444	0	0	0	0	42.288408	1	-0.4111751
0.2016	9915.72365	1.03829813	300.786001	3.23367934	0	-1.6014697	2.29422259	0.03854561	5.70640238	1.1739298	-6.9077553	1	70	2.68223333	303	30	0	0	0	0	42.288408	1	-0.4111751
0.4671	22741.4074	1.05893937	307.447775	2.70453729	0	-0.7612119	3.12423135	0.05821171	5.72830849	0.99530052	-6.9077553	2	67	3.12927222	303.555556	30.55555555	0	0	0	0	42.288408	1	-0.4111751
0.7452	36671.3946	0.9788929	264.505234	2.02841038	0	-0.2941026	3.60202428	-0.020312	5.57786482	0.7077453	-6.9077553	3	65	2.68223333	303.555556	30.55555555	0	0	0	0	42.288408	1	-0.4111751
1.0575	48122.1388	0.84970445	192.603003	1.77706584	0	0.05590763	3.87376312	-0.1616905	5.26063629	0.57552617	-6.9077553	4	67	4.02335	303	30	0	0	1	0	42.288408	1	-0.4111751
1.431	63497.2616	0.87683105	203.678257	1.82030073	0	0.3583735	4.15101253	-0.1303011	5.31654649	0.59955093	-6.9077553	5	58	4.47038889	304.666667	31.66666666	0	0	1	0	42.288408	1	-0.4111751
1.9836	82818.5699	0.89127193	226.606321	2.57733552	0	0.68491338	4.41666439	-0.1139843	5.42321866	0.94714404	-6.9077553	6	55	5.36446667	305.222222	32.2222222	0	0	1	0	42.288408	1	-0.4111751
2.4822	101524.733	0.84268084	198.630768	1.93533226	0	0.90914526	4.6203123	-0.169981	5.2914527	0.6607956	-6.9077553	7	54	4.47038889	305.222222	32.2222222	0	0	1	0	42.288408	1	-0.4111751
2.8854	119947.98	0.87575422	213.127616	1.5190803	0	1.05966354	4.78706648	-0.1315286	5.36189581	0.41876316	-6.9077553	8	60	2.68223333	304.111111	31.11111111	0	0	1	0	42.288408	1	-0.4111751
3.2787	140522.085	0.94566223	245.630167	1.75053577	0	1.187447	4.94537178	-0.0548129	5.50383109	0.56049299	-6.9077553	9	67	1.34111667	302.444444	29.44444444	0	0	1	0	42.288408	1	-0.4111751
3.6972	161208.962	0.91132274	221.705074	1.73854114	0	1.30757578	5.08270763	-0.0917615	5.40135251	0.55362137	-6.9077553	10	73	2.23519444	300.777778	27.7777778	0	0	1	0	42.288408	1	-0.4111751
3.9726	173454.556	0.67642561	119.155637	1.23578659	0	1.37942079	5.15592141	-0.3894555	4.78043891	0.21251655	-6.9077553	11	79	1.34111667	299.666667	26.66666666	0	0	1	0	42.288408	1	-0.4111751
4.0797	178279.582	0.33804616	29.1722751	0.63223029	0	1.40602346	5.18335861	-1.081619	3.37325305	-0.4569211	-6.9077553	12	83	1.34111667	299.111111	26.11111111	0	0	1	0	42.288408	1	-0.4111751
4.185	182534.357	0.34023304	44.837177	0.68334041	0	1.4315067	5.20694389	-1.0751896	3.80305994	-0.3792998	-6.9077553	13	85	2.68223333	298.555556	25.55555555	0	0	1	0	42.288408	1	-0.4111751
4.2552	185692.542	0.26384963	15.1277481	0.43848874	0	1.44814176	5.22409769	-1.3285931	2.71659678	-0.8221432	-6.9077553	14	86	2.68223333	298	25	0	0	1	0	42.288408	1	-0.4111751
4.3308	189026.203	0.25807808	14.5005668	0.41953064	0	1.46575228	5.24189094	-1.3506258	2.6742567	-0.8662379	-6.9077553	15	90	2.23519444	298	25	0	0	1	0	42.288408	1	-0.4111751
4.392	191570.305	0.24431916	13.1751574	0.38664793	0	1.4797847	5.25526009	-1.4051952	2.57840894	-0.9476578	-6.9077553	16	91	3.57631111	298	25	0	0	1	0	42.288408	1	-0.4111751
4.4505	194114.401	0.22038919	10.4871244	0.34798082	0	1.49301645	5.26845283	-1.5078331	2.35024361	-1.0527383	-6.9077553	17	90	3.12927222	298	25	0	0	1	0	42.288408	1	-0.4111751
4.4919	195649.63	0.22712046	11.1608117	0.33565498	0	1.50227577	5.27633057	-1.4778814	2.41249828	-1.0886967	-6.9077553	18	91	2.68223333	298	25	0	0	1	0	42.288408	1	-0.4111751
4.5279	197272.587	0.22208786	10.3116562	0.29911394	0	1.51025826	5.28459153	-1.5001896	2.3333719	-1.2035931	-6.9077553	19	93	1.34111667	298	25	0	0	1	0	42.288408	1	-0.4111751
4.5684	199071	0.24602746	12.7732259	0.36778302	0	1.51916303	5.29366657	-1.3982558	2.54742954	-0.9975468	-6.9077553	20	93	2.23519444	298	25	0	0	1	0	42.288408	1	-0.4111751
4.6926	202758.949	0.41119086	50.3634334	1.00571453	0	1.5459868	5.31202276	-0.8862688	3.91928524	0.00669209	-6.9077553	21	88	3.12927222	299.111111	26.11111111	0	0	1	0	42.288408	1	-0.4111751
5.0148	210560.3	0.53061659	86.5584519	1.17541981	0	1.61239354	5.34977682	-0.6318327	4.46083148	0.16247577	-6.9077553	22	83	2.68223333	300.222222	27.2222222	0	0	1	0	42.288408	1	-0.4111751
5.6079	223798.674	0.62481782	117.002561	1.57793865	0	1.72417632	5.41075134	-0.468696	4.76220437	0.45675288	-6.9077553	23	80	4.02335	300.777778	27.7777778	0	0	1	0	42.288408	1	-0.4111751
0.0099	0	0.56151919	86.0996826	2.12463913	0	-4.6152205	-6.9077553	-0.57533	4.45551734	0.75407252	-6.9077553	0	71	3.57631111	302.444444	29.44444444	0	0	0	0	18.9135952	1	-0.0428739
0.045	0	0.45108203	53.722049	1.58175268	0	-3.1010928	-6.9077553	-0.7938916	3.98384213	0.45916553	-6.9077553	1	70	2.68223333	303	30	0	0	0	0	18.9135952	1	-0.0428739
0.1422	356.82022	0.57643258	92.2156937	1.77445447	0	-1.9505208	-1.0277246	-0.5491636	4.52414117	0.57405643	-6.9077553	2	67	3.12927222	303.555556	30.55555555	0	0	0	0	18.9135952	1	-0.0428739
0.2745	3071.37179	0.70569688	160.209516	1.70850523	0	-1.292804	1.12244983	-0.3471534	5.07648867	0.53620399	-6.9077553	3	65	2.68223333	303.555556	30.55555555	0	0	1	0	18.9135952	1	-0.0428739

0.4896	10608.9857	0.81694133	227.473065	2.1319197	0	-0.7141665	2.36179561	-0.2009647	5.42703623	0.75749179	-6.9077553	4	67	4.02335	303	30	0	0	1	0	18.9135952	1	-0.0428739
0.6759	17100.8559	0.8157785	223.247585	1.977021	0	-0.3917101	2.83918699	-0.2023873	5.40828588	0.68209685	-6.9077553	5	58	4.47038889	304.666667	31.66666666	0	0	1	0	18.9135952	1	-0.0428739
0.9126	24769.6229	0.84443485	226.696204	2.16421544	0	-0.0914576	3.20965839	-0.1679042	5.42361522	0.77251987	-6.9077553	6	55	5.36446667	305.222222	32.2222222	0	0	1	0	18.9135952	1	-0.0428739
1.1961	34179.3185	0.83397023	211.828348	2.25484735	0	0.17906626	3.53165	-0.1803592	5.35578099	0.81352567	-6.9077553	7	54	4.47038889	305.222222	32.2222222	0	0	1	0	18.9135952	1	-0.0428739
1.4067	43306.613	0.85772079	217.300948	1.59469631	0	0.34124654	3.76832844	-0.1523114	5.38128785	0.4673102	-6.9077553	8	60	2.68223333	304.111111	31.11111111	0	0	1	0	18.9135952	1	-0.0428739
1.5399	49395.9809	0.87656784	203.15749	1.44478318	0	0.43171748	3.89988931	-0.130601	5.31398642	0.36865117	-6.9077553	9	67	1.34111667	302.444444	29.44444444	0	0	1	0	18.9135952	1	-0.0428739
1.6398	53979.101	0.78012161	159.679283	1.29525111	0	0.49457428	3.98861548	-0.2470244	5.07317358	0.25947633	-6.9077553	10	73	2.23519444	300.777778	27.7777778	0	0	1	0	18.9135952	1	-0.0428739
1.7199	57488.2025	0.61626096	96.4143368	1.00899523	0	0.54226615	4.05159715	-0.4824634	4.56866528	0.00994561	-6.9077553	11	79	1.34111667	299.666667	26.66666666	0	0	1	0	18.9135952	1	-0.0428739
1.764	59418.2123	0.33553256	25.5725671	0.53575173	0	0.56758396	4.08461761	-1.0890604	3.24155928	-0.6222196	-6.9077553	12	83	1.34111667	299.111111	26.11111111	0	0	1	0	18.9135952	1	-0.0428739
1.8054	61304.358	0.28061792	17.1534278	0.42496843	0	0.59078217	4.11586725	-1.267204	2.84225632	-0.85339	-6.9077553	13	85	2.68223333	298.555556	25.55555555	0	0	1	0	18.9135952	1	-0.0428739
1.8558	63585.2723	0.27722679	16.8381064	0.4560817	0	0.61831587	4.1523976	-1.2793187	2.82370394	-0.7828931	-6.9077553	14	86	2.68223333	298	25	0	0	1	0	18.9135952	1	-0.0428739
1.8963	65471.4139	0.26392687	15.0676419	0.4077702	0	0.63990462	4.18162889	-1.3283015	2.71261589	-0.8946021	-6.9077553	15	90	2.23519444	298	25	0	0	1	0	18.9135952	1	-0.0428739
1.9503	67927.7848	0.26879692	15.6139821	0.44172335	0	0.66798321	4.21845987	-1.3100857	2.74823085	-0.8148102	-6.9077553	16	91	3.57631111	298	25	0	0	1	0	18.9135952	1	-0.0428739
2.0079	70735.0686	0.27091739	15.9357109	0.46108758	0	0.6970894	4.25895561	-1.302257	2.76862531	-0.7720008	-6.9077553	17	90	3.12927222	298	25	0	0	1	0	18.9135952	1	-0.0428739
2.0583	73147.5765	0.27304876	16.0431148	0.46525628	0	0.7218804	4.29249267	-1.2944492	2.7753421	-0.7630198	-6.9077553	18	91	2.68223333	298	25	0	0	1	0	18.9135952	1	-0.0428739
2.1096	75560.0834	0.24904134	13.3289538	0.4033072	0	0.74649836	4.32494138	-1.386129	2.59001367	-0.9055803	-6.9077553	19	93	1.34111667	298	25	0	0	1	0	18.9135952	1	-0.0428739
2.1627	77928.7281	0.26609369	15.2435148	0.43951154	0	0.77135744	4.3558075	-1.3201558	2.72421975	-0.8198186	-6.9077553	20	93	2.23519444	298	25	0	0	1	0	18.9135952	1	-0.0428739
2.2599	81631.6695	0.47055425	68.895128	1.05619028	0	0.81532056	4.40222954	-0.7517211	4.23259998	0.05561471	-6.9077553	21	88	3.12927222	299.111111	26.11111111	0	0	1	0	18.9135952	1	-0.0428739
2.6487	93266.6302	0.65635526	136.709021	1.65694894	0	0.97406895	4.5354731	-0.4195307	4.91786204	0.50558126	-6.9077553	22	83	2.68223333	300.222222	27.2222222	0	0	1	0	18.9135952	1	-0.0428739
3.1005	106585.317	0.74623819	169.168918	1.91322357	0	1.13156339	4.66895515	-0.2913713	5.13090364	0.64931209	-6.9077553	23	80	4.02335	300.777778	27.7777778	0	0	1	0	18.9135952	1	-0.0428739
0.0108	360.925687	0.80924485	185.408481	1.45987025	0	-4.5282091	-1.0163164	-0.2104188	5.22256679	0.37903232	-6.9077553	0	68	2.68223333	303.555556	30.55555555	0	0	0	0	30.519848	1	0.21141735
0.0243	932.093096	0.71799068	134.247941	1.18816299	0	-3.7172789	-0.0692503	-0.3299069	4.89969585	0.17324969	-6.9077553	1	73	2.23519444	301.888889	28.88888888	0	0	0	0	30.519848	1	0.21141735
0.0378	1283.00337	0.69784005	131.352995	1.35557963	0	-3.2754462	0.24998283	-0.3583334	4.87789593	0.30496655	-6.9077553	2	77	1.78815556	300.777778	27.7777778	0	0	0	0	30.519848	1	0.21141735
0.0414	1414.5947	0.37046331	36.7980194	0.93465137	0	-3.1844744	0.34754973	-0.9903052	3.6054712	-0.0665123	-6.9077553	3	80	1.34111667	300.222222	27.2222222	0	0	0	0	30.519848	1	0.21141735
0.0432	1414.5947	0.34132832	25.1736546	0.37508568	0	-3.1419148	0.34754973	-1.071985	3.22583772	-0.9779383	-6.9077553	4	84	2.68223333	299.666667	26.66666666	0	0	0	0	30.519848	1	0.21141735
0.0441	1458.4585	0.24662316	12.3849993	0.40572742	0	-3.1212955	0.37806547	-1.3958472	2.51656674	-0.8996121	-6.9077553	5	88	1.78815556	299.111111	26.11111111	0	0	0	0	30.519848	1	0.21141735
0.0441	1458.4585	0	0	0	0	-3.1212955	0.37806547	-6.9077553	-6.9077553	-6.9077553	-6.9077553	6	89	2.68223333	299.666667	26.66666666	0	0	0	0	30.519848	1	0.21141735
0.0441	1458.4585	0	0	0	0	-3.1212955	0.37806547	-6.9077553	-6.9077553	-6.9077553	-6.9077553	7	86	4.02335	300.222222	27.2222222	0	0	0	0	30.519848	1	0.21141735
0.0441	1458.4585	0	0	0	0	-3.1212955	0.37806547	-6.9077553	-6.9077553	-6.9077553	-6.9077553	8	87	3.57631111	300.222222	27.2222222	0	0	0	0	30.519848	1	0.21141735
0.0441	1458.4585	0	0	0	0	-3.1212955	0.37806547	-6.9077553	-6.9077553	-6.9077553	-6.9077553	9	87	3.57631111	300.222222	27.2222222	0	0	0	0	30.519848	1	0.21141735
0.0441	1458.4585	0	0	0	0	-3.1212955	0.37806547	-6.9077553	-6.9077553	-6.9077553	-6.9077553	10	87	4.02335	300.222222	27.2222222	0	0	0	0	30.519848	1	0.21141735
0.0441	1458.4585	0	0	0	0	-3.1212955	0.37806547	-6.9077553	-6.9077553	-6.9077553	-6.9077553	11	88	2.68223333	300.222222	27.2222222	0	0	0	0	30.519848	1	0.21141735
0.0459	1502.3223	0.00558748	0.00346298	0.54897469	0	-3.0812902	0.40767753	-5.022584	-5.4119376	-0.597883	-6.9077553	12	84	3.57631111	300.777778	27.7777778	0	0	0	0	30.519848	1	0.21141735
0.09	1734.98991	0.35752246	33.8081129	1.40120495	0	-2.4079456	0.55157781	-1.025764	3.52073038	0.33804596	-6.9077553	13	78	3.57631111	301.888889	28.88888888	0	0	0	0	30.519848	1	0.21141735
0.1746	2301.11732	0.42166109	48.8334234	1.57376634	0	-1.7452576	0.83382927	-0.8611846	3.88843546	0.45410691	-6.9077553	14	76	4.02335	301.888889	28.88888888	0	0	0	0	30.519848	1	0.21141735
0.2628	3241.97346	0.55254745	85.4218949	1.58335534	0	-1.336362	1.17649064	-0.5914078	4.44761416	0.4601776	-6.9077553	15	66	4.02335	304.111111	31.11111111	0	0	0	0	30.519848	1	0.21141735
0.3609	3946.5354	0.58330529	88.7779709	1.70551545	0	-1.0191544	1.37309144	-0.5373317	4.48614981	0.53445354	-6.9077553	16	63	3.57631111	304.666667	31.66666666	0	0	0	1	30.519848	1	0.21141735
0.5571	8498.55843	0.80505217	192.006182	2.46089158	0	-0.5850105	2.14001421	-0.2156068	5.25753278	0.90092999	-6.9077553	17	57	5.81150556	305.777778	32.7777778	0	0	0	1	30.519848	1	0.21141735
0.8244	17402.4121	0.84796256	211.874934	2.88423131	0	-0.1930994	2.85666629	-0.1637402	5.35600089	1.05960507	-6.9077553	18	56	5.36446667	305.777778	32.7777778	0	0	0	1	30.519848	1	0.21141735
1.1493	24466.0168	0.71098416	139.298238	2.10266976	0	0.13915306	3.19732596	-0.3396996	4.93662441	0.74368332	-6.9077553	19	55	5.81150556	306.333333	33.33333333	0	0	0	1	30.519848	1	0.21141735
1.3689	32362.6732	0.65769439	122.74278	1.30746085	0	0.3140075	3.4770366	-0.4174956	4.81009909	0.26885152	-6.9077553	20	59	3.12927222	305.777778	32.7777778	0	0	0	1	30.519848	1	0.21141735
1.5417	41273.4652	0.67892241	136.428762	1.12973102	0	0.4328857	3.72024403	-0.3857766	4.91580992	0.12286434	-6.9077553	21	59	3.12927222	305.222222	32.2222222	0	0	0	1	30.519848	1	0.21141735
1.6551	49329.1108	0.7445844	150.776708	1.09736403	0	0.50386143	3.89853466	-0.2935869	5.01580662	0.09382183	-6.9077553	22	65	1.78815556	304.666667	31.66666666	0	0	0	1	30.519848	1	0.21141735
1.7325	56456.2932	0.72249501	140.054881	1.01632948	0	0.54956545	4.03348448	-0.3236616	4.94204149	0.01718103	-6.9077553	23	70	1.34111667	304.111111	31.11111111	0	0	0	1	30.519848	1	0.21141735
0.0225	957.235778	0.97718422	270.306946	2.03247356	0	-3.79424	-0.0426614	-0.0220573	5.59956185	0.70974545	-6.9077553	0	68	2.68223333	303.555556	30.55555555	0	0	0	0	34.0724638	1	0.49277013
0.045	1659.05632	0.70564995	125.492803	0.93811218	0	-3.1010928	0.50685153	-0.3472199	4.83225638	-0.0628203	-6.9077553	1	73	2.23519444	301.888889	28.88888888	0	0	0	0	34.0724638	1	0.49277013
0.0621	2404.74059	0.53265386	69.6840306	0.82060252	0	-2.7790093	0.87785779	-0.6280079	4.24398553	-0.1964986	-6.9077553	2	77	1.78815556	300.777778	27.7777778	0	0	0	0	34.0724638	1	0.49277013
0.0828	3282.01625	0.46934916	52.6173149	0.72213977	0	-2.4913272	1.18876259	-0.75428	3.96306425	-0.3241528	-6.9077553	3	80	1.34111667	300.222222	27.2222222	0	0	0	0	34.0724638	1	0.49277013
0.0999	3983.83643	0.35326293	28.0547196	0.4781318	0	-2.3035856	1.38249627	-1.0377159	3.33419252	-0.7357796	-6.9077553	4	84	2.68223333	299.666667	26.66666666	0	0	0	0	34.0724638	1	0.49277013
0.1143	4685.65725	0.28425384	17.2267742	0.38270032	0	-2.1689287	1.54471959	-1.2543758	2.84652286	-0.9578934	-6.9077553	5	88	1.78815556	299.111111	26.11111111	0	0	0	0	34.0724638	1	0.49277013
0.1314	5475.2053	0.24665157	12.5395604	0.30983978	0	-2.0295092	1.7004124	-1.3957325	2.52896822	-1.1684777	-6.9077553	6	89	2.68223333	299.666667	26.66666666	0	0	0	0	34.0724638	1	0.49277013
0.1485	6264.75314	0.24710028	12.9703482	0.32485144	0	-1.9071703	1.8350988	-1.3939223	2.56274294	-1.1213137	-6.9077553	7	86	4.02335	300.222222	27.2222222	0	0	0	0	34.0724638	1	0.49277013
0.1665	7098.16529	0.27025285	15.6898682	0.39512289	0	-1.79276	1.95997721	-1.3047039	2.7530789	-0.9260308	-6.9077553	8	87	3.57631111	300.222222	27.2222222	0	0	0	0	34.0724638	1	0.49277013
0.18	7668.39495	0.22991254	11.1332265	0.28383751	0	-1.7147984	2.03723773	-1.4657163	2.41002383	-1.2558364	-6.9077553	9	87	3.57631111	300.222222	27.2222222	0	0	0	0	34.0724638	1	0.49277013
0.1935	8194.75996	0.21075773	9.24473165	0.25902405	0	-1.6424778	2.10361694	-1.5523124	2.224162	-1.3469812	-6.9077553	10	87	4.02335	300.222222	27.2222222	0	0	0	0	34.0724638	1	0.49277013
0.2124	9072.0353	0.22973946	12.0737305	0.33589324	0	-1.549284	2.20530686	-1.4664661	2.49111488	-1.0879892	-6.9077553	11	88	2.68223333	300.222222	27.2222222	0	0	0	0	34.0724638	1	0.49277013
0.2646	11321.1411	0.69134443	135.514808	1.20898687	0	-1.329536	2.4267602	-0.3676717	4.9090883	0.19060951	-6.9077553	12	84	3.57631111	300.777778	27.7777778	0	0	0	0	34.0724638	1	0.49277013
0.4644	18051.5034	0.94667961	276.682714	2.46602651	0	-0.767009	2.89328437	-0.0537388	5.62287503	0.90301358	-6.9077553	13	78	3.57631111	301.888889	28.88888888	0	0	0	0	34.0724638	1	0.49277013
0.7956	26397.3338	1.0616674	338.891262	3.15775797	0	-0.2286587	3.27330089	0.06078216	5.82568225	1.1501789	-6.9077553	14	76	4.02335	301.888889	28.88888888	0	0	0	1	34.0724638	1	0.49277013
1.359	35733.6643	1.27777353	500.006739	3.87309368	0	0.30674914	3.57612121	0.24590144	6.21462358	1.35431175	-6.9077553	15	66	4.02335	304.111111	31.11111111	0	0	0	1	34.0724638	1	0.49277013
1.9404	47752.5051	1.11457017	359.848684	2.98682094	0	0.66289414	3.86605247	0.10936564	5.8856864	1.09454434	-6.9077553	16	63	3.57631111	304.666667	31.66666666	0	0	0	1	34.0724638	1	0.49277013
2.5542	66944.7326	1.07252426	343.048983	2.85714408	1	0.93773906	4.20388233	0.07094694	5.83787616	1.05017249	0.0009995	17	57	5.81150556	305.777778	32.7777778	0	0	0	1	34.0724638	1	0.49277013

3.0852	84175.214	0.91687301	232.410593	2.04886413	1	1.12661649	4.43291239	-0.0856962	5.44850991	0.71777351	0.0009995	18	56	5.36446667	305.777778	32.7777778	0	0	0	1	34.0724638	1	0.49277013
3.843	114070.402	1.06484978	331.397417	3.32750392	1	1.34625331	4.73682458	0.0637724	5.80332133	1.20252293	0.0009995	19	55	5.81150556	306.333333	33.33333333	0	0	0	1	34.0724638	1	0.49277013
4.608	136532.279	0.88641343	221.029779	2.50536707	1	1.52779392	4.91656839	-0.1194443	5.39830197	0.91883432	0.0009995	20	59	3.12927222	305.777778	32.7777778	0	0	0	1	34.0724638	1	0.49277013
5.1543	151113.517	0.76944085	171.642375	1.72000107	1	1.63983132	5.01803794	-0.2607924	5.14541893	0.54290614	0.0009995	21	59	3.12927222	305.222222	32.2222222	0	0	0	1	34.0724638	1	0.49277013
5.4846	162699.496	0.75694479	161.172918	1.29976394	1	1.70194416	5.09191106	-0.2771447	5.08248402	0.26295174	0.0009995	22	65	1.78815556	304.666667	31.66666666	0	0	0	1	34.0724638	1	0.49277013
5.7195	173015.004	0.80218775	173.699054	1.32287504	1	1.74388139	5.1533841	-0.2191668	5.15732998	0.28056307	0.0009995	23	70	1.34111667	304.111111	31.11111111	0	0	0	1	34.0724638	1	0.49277013
0.0351	2315.9711	1.15640429	369.909591	2.40590889	0	-3.3495541	0.84026077	0.14617982	5.91326133	0.8783433	-6.9077553	0	68	2.68223333	303.555556	30.55555555	0	0	0	0	42.288408	1	-0.4111751
0.1044	6007.32182	0.91396639	227.222479	1.78517924	0	-2.2595256	1.79314548	-0.0888679	5.42593402	0.58007883	-6.9077553	1	73	2.23519444	301.888889	28.88888888	0	0	0	0	42.288408	1	-0.4111751
0.1674	8990.08989	0.6880285	121.325363	1.20234811	0	-1.7873691	2.19623408	-0.3724726	4.79848413	0.18510776	-6.9077553	2	77	1.78815556	300.777778	27.7777778	0	0	0	0	42.288408	1	-0.4111751
0.2214	11534.1895	0.57694918	82.1584961	1.06467527	0	-1.5077843	2.44540232	-0.5482693	4.40866243	0.06360865	-6.9077553	3	80	1.34111667	300.222222	27.2222222	0	0	0	0	42.288408	1	-0.4111751
0.2583	13332.6018	0.43812128	44.8658447	0.7583453	0	-1.3536336	2.5902873	-0.8229796	3.8036991	-0.2752987	-6.9077553	4	84	2.68223333	299.666667	26.66666666	0	0	0	0	42.288408	1	-0.4111751
0.2925	14911.7005	0.32274718	23.1176533	0.56720498	0	-1.2292906	2.70221323	-1.1277924	3.14063979	-0.565273	-6.9077553	5	88	1.78815556	299.111111	26.11111111	0	0	0	0	42.288408	1	-0.4111751
0.3285	16578.5235	0.30874829	20.8496826	0.5199337	0	-1.1132184	2.80816841	-1.1719953	3.03738669	-0.6521325	-6.9077553	6	89	2.68223333	299.666667	26.66666666	0	0	0	0	42.288408	1	-0.4111751
0.3582	17938.3013	0.29390575	19.1531686	0.51837545	0	-1.0266638	2.88699391	-1.2210995	2.95252038	-0.6551282	-6.9077553	7	86	4.02335	300.222222	27.2222222	0	0	0	0	42.288408	1	-0.4111751
0.3861	19254.215	0.28339472	17.4436606	0.46524285	0	-0.9516589	2.95778193	-1.2573921	2.85903362	-0.7630486	-6.9077553	8	87	3.57631111	300.222222	27.2222222	0	0	0	0	42.288408	1	-0.4111751
0.4239	20964.9021	0.25139182	13.9527312	0.38971955	0	-0.8582577	3.04289741	-1.3767726	2.63574694	-0.9397652	-6.9077553	9	87	3.57631111	300.222222	27.2222222	0	0	0	0	42.288408	1	-0.4111751
0.4536	22324.6807	0.26068919	15.0366535	0.43279006	0	-0.7905395	3.10573762	-1.3405978	2.71055729	-0.8351946	-6.9077553	10	87	4.02335	300.222222	27.2222222	0	0	0	0	42.288408	1	-0.4111751
0.4923	24166.9584	0.29418928	21.550689	0.43512504	0	-0.708667	3.18502772	-1.2201385	3.07045419	-0.8298263	-6.9077553	11	88	2.68223333	300.222222	27.2222222	0	0	1	0	42.288408	1	-0.4111751
0.5805	28353.2253	0.67405708	129.422363	1.1311644	0	-0.5438655	3.34477606	-0.392958	4.86308892	0.12413119	-6.9077553	12	84	3.57631111	300.777778	27.7777778	0	0	1	0	42.288408	1	-0.4111751
0.8352	36345.6961	0.6791618	141.285653	1.63027706	0	-0.1800841	3.59310331	-0.3854246	4.95079083	0.48936318	-6.9077553	13	78	3.57631111	301.888889	28.88888888	0	0	1	0	42.288408	1	-0.4111751
1.2051	48040.4205	0.79945244	173.695769	1.99709675	0	0.18656255	3.87206357	-0.2225782	5.15731107	0.6921951	-6.9077553	14	76	4.02335	301.888889	28.88888888	0	0	1	0	42.288408	1	-0.4111751
1.5732	60405.8883	0.79006786	163.027583	1.66956999	0	0.45311176	4.10110314	-0.2343715	5.09392554	0.51316488	-6.9077553	15	66	4.02335	304.111111	31.11111111	0	0	1	0	42.288408	1	-0.4111751
1.944	75176.9696	0.81290562	173.872269	1.4501722	0	0.66474771	4.31985823	-0.2059109	5.1583267	0.37237165	-6.9077553	16	63	3.57631111	304.666667	31.66666666	0	0	1	0	42.288408	1	-0.4111751
2.5416	98565.5903	0.9344749	243.40865	2.62540978	0	0.9327938	4.59073236	-0.066701	5.49474583	0.96561781	-6.9077553	17	57	5.81150556	305.777778	32.7777778	0	0	1	0	42.288408	1	-0.4111751
3.3165	128923.553	0.95144841	259.674361	2.71790586	0	1.19891001	4.85922737	-0.0487193	5.55943224	1.00022954	-6.9077553	18	56	5.36446667	305.777778	32.7777778	0	0	1	0	42.288408	1	-0.4111751
4.1166	167785.115	0.97840612	274.272146	2.13588403	0	1.41502758	5.12269004	-0.0208089	5.61412449	0.75934871	-6.9077553	19	55	5.81150556	306.333333	33.33333333	0	0	1	0	42.288408	1	-0.4111751
4.9509	205962.281	0.9815155	280.905812	2.22139841	0	1.59956938	5.32769791	-0.0176392	5.63802298	0.79858698	-6.9077553	20	59	3.12927222	305.777778	32.7777778	0	0	1	0	42.288408	1	-0.4111751
5.5755	229278.426	0.86080572	216.709699	1.86245947	0	1.718382	5.43494146	-0.1487254	5.37856328	0.62243469	-6.9077553	21	59	3.12927222	305.222222	32.2222222	0	0	1	0	42.288408	1	-0.4111751
5.9499	241381.478	0.71589932	149.659396	1.23174447	0	1.78337441	5.48638272	-0.3328199	5.0083687	0.20924296	-6.9077553	22	65	1.78815556	304.666667	31.66666666	0	0	1	0	42.288408	1	-0.4111751
6.111	248413.952	0.7793805	161.946174	1.22872567	0	1.81009043	5.51510054	-0.2479737	5.0872702	0.20679111	-6.9077553	23	70	1.34111667	304.111111	31.11111111	0	0	1	0	42.288408	1	-0.4111751
0.0009	0	0	0	0	0	-7.0131158	-6.9077553	-6.9077553	-6.9077553	-6.9077553	-6.9077553	0	68	2.68223333	303.555556	30.55555555	0	0	0	0	18.9135952	1	-0.0428739
0.0009	0	0	0	0	0	-7.0131158	-6.9077553	-6.9077553	-6.9077553	-6.9077553	-6.9077553	1	73	2.23519444	301.888889	28.88888888	0	0	0	0	18.9135952	1	-0.0428739
0.0009	0	0	0	0	0	-7.0131158	-6.9077553	-6.9077553	-6.9077553	-6.9077553	-6.9077553	2	77	1.78815556	300.777778	27.7777778	0	0	0	0	18.9135952	1	-0.0428739
0.0009	0	0	0	0	0	-7.0131158	-6.9077553	-6.9077553	-6.9077553	-6.9077553	-6.9077553	3	80	1.34111667	300.222222	27.2222222	0	0	0	0	18.9135952	1	-0.0428739
0.0009	0	0	0	0	0	-7.0131158	-6.9077553	-6.9077553	-6.9077553	-6.9077553	-6.9077553	4	84	2.68223333	299.666667	26.66666666	0	0	0	0	18.9135952	1	-0.0428739
0.0009	0	0	0	0	0	-7.0131158	-6.9077553	-6.9077553	-6.9077553	-6.9077553	-6.9077553	5	88	1.78815556	299.111111	26.11111111	0	0	0	0	18.9135952	1	-0.0428739
0.0009	0	0	0	0	0	-7.0131158	-6.9077553	-6.9077553	-6.9077553	-6.9077553	-6.9077553	6	89	2.68223333	299.666667	26.66666666	0	0	0	0	18.9135952	1	-0.0428739
0.0009	0	0	0	0	0	-7.0131158	-6.9077553	-6.9077553	-6.9077553	-6.9077553	-6.9077553	7	86	4.02335	300.222222	27.2222222	0	0	0	0	18.9135952	1	-0.0428739
0.0009	0	0	0	0	0	-7.0131158	-6.9077553	-6.9077553	-6.9077553	-6.9077553	-6.9077553	8	87	3.57631111	300.222222	27.2222222	0	0	0	0	18.9135952	1	-0.0428739
0.0009	0	0	0	0	0	-7.0131158	-6.9077553	-6.9077553	-6.9077553	-6.9077553	-6.9077553	9	87	3.57631111	300.222222	27.2222222	0	0	0	0	18.9135952	1	-0.0428739
0.0009	0	0	0	0	0	-7.0131158	-6.9077553	-6.9077553	-6.9077553	-6.9077553	-6.9077553	10	87	4.02335	300.222222	27.2222222	0	0	0	0	18.9135952	1	-0.0428739
0.0009	0	0	0	0	0	-7.0131158	-6.9077553	-6.9077553	-6.9077553	-6.9077553	-6.9077553	11	88	2.68223333	300.222222	27.2222222	0	0	0	0	18.9135952	1	-0.0428739
0.0009	0	0	0	0	0	-7.0131158	-6.9077553	-6.9077553	-6.9077553	-6.9077553	-6.9077553	12	84	3.57631111	300.777778	27.7777778	0	0	0	0	18.9135952	1	-0.0428739
0.0036	0	0.37611675	41.6883723	1.39623419	0	-5.6268214	-6.9077553	-0.9752005	3.73024624	0.33449471	-6.9077553	13	78	3.57631111	301.888889	28.88888888	0	0	0	0	18.9135952	1	-0.0428739
0.0423	59.4318761	0.60432443	108.254122	2.35345956	0	-3.1629682	-2.8062386	-0.5019907	4.68449068	0.85631121	-6.9077553	14	76	4.02335	301.888889	28.88888888	0	0	0	0	18.9135952	1	-0.0428739
0.1161	717.393731	0.56781029	85.8580709	1.64444547	0	-2.1533034	-0.3307375	-0.5642083	4.45270724	0.49801115	-6.9077553	15	66	4.02335	304.111111	31.11111111	0	0	0	0	18.9135952	1	-0.0428739
0.2556	3163.4274	0.66737159	134.551235	1.70614742	0	-1.3641416	1.15197212	-0.402911	4.90195249	0.5348238	-6.9077553	16	63	3.57631111	304.666667	31.66666666	0	0	1	0	18.9135952	1	-0.0428739
0.4995	11334.284	0.84684607	244.447273	2.58094591	0	-0.6941477	2.42792034	-0.1650562	5.49900372	0.94854334	-6.9077553	17	57	5.81150556	305.777778	32.7777778	0	0	1	0	18.9135952	1	-0.0428739
0.7641	21279.2804	0.89738693	273.463701	2.42527345	0	-0.2690566	3.05778084	-0.1071544	5.61117255	0.88635652	-6.9077553	18	56	5.36446667	305.777778	32.7777778	0	0	1	0	18.9135952	1	-0.0428739
1.0926	29977.6727	0.87131281	231.937949	2.71543353	0	0.08856018	3.40048622	-0.1366072	5.44647419	0.99931982	-6.9077553	19	55	5.81150556	306.333333	33.33333333	0	0	1	0	18.9135952	1	-0.0428739
1.3977	42338.2648	0.88594798	232.829116	1.8670901	0	0.33482803	3.7457149	-0.1199689	5.45030907	0.62491657	-6.9077553	20	59	3.12927222	305.777778	32.7777778	0	0	1	0	18.9135952	1	-0.0428739
1.6596	51836.8201	0.79287447	182.317145	1.58185926	0	0.50657661	3.94812	-0.2308299	5.20575321	0.45923287	-6.9077553	21	59	3.12927222	305.222222	32.2222222	0	0	1	0	18.9135952	1	-0.0428739
1.8963	61604.7325	0.81502843	187.292048	1.46110227	0	0.63990462	4.12075493	-0.2033061	5.23267449	0.37987531	-6.9077553	22	65	1.78815556	304.666667	31.66666666	0	0	1	0	18.9135952	1	-0.0428739
2.097	71062.6211	0.88946218	209.681089	1.5793613	0	0.74050775	4.26357555	-0.1160146	5.34559252	0.45765349	-6.9077553	23	70	1.34111667	304.111111	31.11111111	0	0	1	0	18.9135952	1	-0.0428739
0.0063	223.85653	0.91111724	223.695943		0	-5.0672056	-1.4922927	-0.0919867	5.4102922		-6.9077553	0	88	1.78815556	299.111111	26.11111111	0	0	0	0	30.519848	1	0.21141735
0.0126	487.03927	0.48754709	58.8762861		0	-4.3740585	-0.7173594	-0.7163194	4.07545538		-6.9077553	1	89	2.68223333	299.666667	26.66666666	0	0	0	0	30.519848	1	0.21141735
0.0225	925.677063	0.58471207	84.1390381		0	-3.79424	-0.0761501	-0.534927	4.43248253		-6.9077553	2	86	4.02335	300.222222	27.2222222	0	0	0	0	30.519848	1	0.21141735
0.0342	1188.85973	0.60382601	88.3748967		0	-3.3755296	0.17383543	-0.5028145	4.48159927		-6.9077553	3	87	3.57631111	300.222222	27.2222222	0	0	0	0	30.519848	1	0.21141735
0.0378	1276.58732	0.42514953	48.6114197		0	-3.2754462	0.24497339	-0.852965	3.88387905		-6.9077553	4	87	3.57631111	300.222222	27.2222222	0	0	0	0	30.519848	1	0.21141735
0.0405	1320.45111	0.44402393	49.5247294		0	-3.2064533	0.27873046	-0.8096272	3.90249232		-6.9077553	5	87	4.02335	300.222222	27.2222222	0	0	0	0	30.519848	1	0.21141735
0.0414	1320.45111	0.57503301	78.0107651		0	-3.1844744	0.27873046	-0.5515903	4.35685965		-6.9077553	6	88	2.68223333	300.222222	27.2222222	0	0	0	0	30.519848	1	0.21141735
0.0711	1627.49757	0.25588576	17.0491259		0	-2.6436679	0.48765785	-1.3591238	2.83615759		-6.9077553	7	84	3.57631111	300.777778	27.7777778	0	0	0	0	30.519848	1	0.21141735

0.1215	1770.28462	0.33138136	28.7656032		0	-2.107841	0.57170506	-1.1014723	3.3592151		-6.9077553	8	78	3.57631111	301.888889	28.88888888	0	0	0	0	30.519848	1	0.21141735
0.2385	2669.96887	0.54907338	85.7086163		0	-1.433386	0.98244128	-0.5977036	4.45096503		-6.9077553	9	76	4.02335	301.888889	28.88888888	0	0	0	0	30.519848	1	0.21141735
0.3186	3450.55834	0.60221996	98.3981742		0	-1.1438189	1.23882582	-0.5054734	4.58903241		-6.9077553	10	66	4.02335	304.111111	31.11111111	0	0	0	0	30.519848	1	0.21141735
0.4338	4551.4137	0.62346882	107.554688		0	-0.8351717	1.51565758	-0.4708539	4.67800874		-6.9077553	11	63	3.57631111	304.666667	31.66666666	0	0	0	1	30.519848	1	0.21141735
0.6642	8977.35622	0.80572063	197.682755		0	-0.409172	2.19481682	-0.2147779	5.28666855		-6.9077553	12	57	5.81150556	305.777778	32.7777778	0	0	0	1	30.519848	1	0.21141735
0.9666	14325.6464	0.80348451	190.755871		0	-0.0339705	2.66212118	-0.2175536	5.25099969		-6.9077553	13	56	5.36446667	305.777778	32.7777778	0	0	0	1	30.519848	1	0.21141735
1.2537	18642.4792	0.71876746	141.608959		0	0.22609918	2.92549644	-0.3288271	4.95307651		-6.9077553	14	55	5.81150556	306.333333	33.33333333	0	0	0	1	30.519848	1	0.21141735
1.4535	23980.787	0.71567467	146.094551		0	0.37397444	3.17729467	-0.3331333	4.98426086		-6.9077553	15	59	3.12927222	305.777778	32.7777778	0	0	0	1	30.519848	1	0.21141735
1.6083	28589.9647	0.71840198	145.626749		0	0.47517772	3.35309075	-0.329335	4.9810537		-6.9077553	16	59	3.12927222	305.222222	32.2222222	0	0	0	1	30.519848	1	0.21141735
1.7127	31893.7427	0.74710537	153.931186		0	0.53807107	3.46244119	-0.2902114	5.03651215		-6.9077553	17	65	1.78815556	304.666667	31.66666666	0	0	0	1	30.519848	1	0.21141735
1.7739	34041.9591	0.71976774	136.471163		0	0.57318051	3.52762323	-0.3274383	4.91612066		-6.9077553	18	70	1.34111667	304.111111	31.11111111	0	0	0	1	30.519848	1	0.21141735
1.8288	36492.8071	0.655595	112.710329		0	0.60366001	3.59714258	-0.4206879	4.72482994		-6.9077553	19	73	1.78815556	303	30	0	0	0	1	30.519848	1	0.21141735
1.8549	37589.4012	0.49032438	58.8021577		0	0.61783079	3.62674873	-0.7106507	4.07419556		-6.9077553	20	76	2.68223333	301.888889	28.88888888	0	0	0	1	30.519848	1	0.21141735
1.8693	38247.3578	0.28799927	17.9221916		0	0.62556403	3.64410063	-1.2413311	2.8860955		-6.9077553	21	80	2.23519444	301.333333	28.33333333	0	0	0	1	30.519848	1	0.21141735
1.8801	38773.723	0.25011214	13.0247714		0	0.63132497	3.65776856	-1.3818557	2.56692981		-6.9077553	22	81	2.68223333	301.333333	28.33333333	0	0	0	1	30.519848	1	0.21141735
1.8801	38773.723	0	0		0	0.63132497	3.65776856	-6.9077553	-6.9077553		-6.9077553	23	83	2.68223333	300.777778	27.7777778	0	0	0	1	30.519848	1	0.21141735
0.0144	660.640138	0.97281698	262.922428	1.82121607	0	-4.2405271	-0.4130335	-0.0265319	5.57186284	0.60005338	-6.9077553	0	88	1.78815556	299.111111	26.11111111	0	0	0	0	34.0724638	1	0.49277013
0.0288	1143.1418	0.49596089	63.0018692	0.95555633	0	-3.5473799	0.13465484	-0.6992439	4.14318027	-0.0444156	-6.9077553	1	89	2.68223333	299.666667	26.66666666	0	0	0	0	34.0724638	1	0.49277013
0.0441	1757.23466	0.56964796	80.2782629	0.85704478	0	-3.1212955	0.56431027	-0.5609828	4.38551134	-0.153099	-6.9077553	2	86	4.02335	300.222222	27.2222222	0	0	0	0	34.0724638	1	0.49277013
0.0603	2459.05517	0.59174527	88.8156874	1.09091949	0	-2.8084232	0.90018378	-0.5229905	4.48657455	0.08793715	-6.9077553	3	87	3.57631111	300.222222	27.2222222	0	0	0	0	34.0724638	1	0.49277013
0.0891	3687.24101	0.61998546	95.1455078	1.0235883	0	-2.4179959	1.30514965	-0.4764476	4.55541789	0.02429087	-6.9077553	4	87	3.57631111	300.222222	27.2222222	0	0	0	0	34.0724638	1	0.49277013
0.1278	5397.92852	0.62236666	97.4009584	0.95129696	0	-2.0572887	1.68620051	-0.4726204	4.57884632	-0.0488784	-6.9077553	5	87	4.02335	300.222222	27.2222222	0	0	0	0	34.0724638	1	0.49277013
0.1665	7240.20737	0.59873213	86.9994379	0.84342575	0	-1.79276	1.97978796	-0.5112722	4.46591315	-0.1690985	-6.9077553	6	88	2.68223333	300.222222	27.2222222	0	0	0	0	34.0724638	1	0.49277013
0.2025	8907.03139	0.62266865	94.2999695	0.84210262	0	-1.5970154	2.18695327	-0.4721361	4.54649147	-0.1706666	-6.9077553	7	84	3.57631111	300.777778	27.7777778	0	0	0	0	34.0724638	1	0.49277013
0.3105	12964.244	0.81622454	196.144092	1.86963984	0	-1.1695714	2.56227224	-0.2018414	5.27885465	0.62628053	-6.9077553	8	78	3.57631111	301.888889	28.88888888	0	0	0	0	34.0724638	1	0.49277013
0.4986	21172.5217	1.07304492	337.33676	2.93782766	0	-0.6959511	3.05275142	0.07143182	5.82108468	1.07801075	-6.9077553	9	76	4.02335	301.888889	28.88888888	0	0	0	1	34.0724638	1	0.49277013
0.8667	33556.4483	1.18453853	397.840694	3.28772898	0	-0.1430624	3.51325884	0.17019712	5.98605417	1.19050116	-6.9077553	10	66	4.02335	304.111111	31.11111111	0	0	0	1	34.0724638	1	0.49277013
1.368	43923.5915	1.17208254	407.471611	3.15455285	0	0.31334982	3.78247434	0.15963494	6.00997372	1.14916371	-6.9077553	11	63	3.57631111	304.666667	31.66666666	0	0	0	1	34.0724638	1	0.49277013
2.0493	60042.4244	1.23198487	466.978988	3.85861613	0	0.71749827	4.09506804	0.20943796	6.1462864	1.35056773	-6.9077553	12	57	5.81150556	305.777778	32.7777778	0	0	0	1	34.0724638	1	0.49277013
2.6703	79776.6079	1.06691169	337.324547	2.70079434	0	0.98219083	4.37924286	0.06570505	5.82104848	0.99391612	-6.9077553	13	56	5.36446667	305.777778	32.7777778	0	0	0	1	34.0724638	1	0.49277013
3.3147	104914.804	1.02312295	299.482847	2.70010171	0	1.19836712	4.65315816	0.02383659	5.70206048	0.99365973	-6.9077553	14	55	5.81150556	306.333333	33.33333333	0	0	0	1	34.0724638	1	0.49277013
3.9726	128226.35	0.94305954	250.333896	2.31809152	0	1.37942079	4.85380486	-0.057566	5.52279961	0.84117552	-6.9077553	15	59	3.12927222	305.777778	32.7777778	0	0	0	1	34.0724638	1	0.49277013
4.5162	143413.836	0.82904972	193.330324	2.00593941	0	1.50767093	4.96574138	-0.1862697	5.26440542	0.69661088	-6.9077553	16	59	3.12927222	305.222222	32.2222222	0	0	0	1	34.0724638	1	0.49277013
4.8501	153917.874	0.75869134	161.949366	1.38673125	0	1.57899932	5.03642567	-0.2748431	5.08728991	0.32767022	-6.9077553	17	65	1.78815556	304.666667	31.66666666	0	0	0	1	34.0724638	1	0.49277013
5.0733	162265.136	0.82277519	185.398343	1.42971457	0	1.62399149	5.0892378	-0.1938576	5.22251211	0.35817402	-6.9077553	18	70	1.34111667	304.111111	31.11111111	0	0	0	1	34.0724638	1	0.49277013
5.2092	167127.154	0.70219295	129.448831	1.21094073	0	1.65042629	5.11876091	-0.352124	4.8632934	0.19222299	-6.9077553	19	73	1.78815556	303	30	0	0	0	1	34.0724638	1	0.49277013
5.2677	169320.354	0.41622646	42.0775879	0.67500481	0	1.66159383	5.13179841	-0.8741261	3.73953901	-0.3915551	-6.9077553	20	76	2.68223333	301.888889	28.88888888	0	0	0	1	34.0724638	1	0.49277013
5.3046	170811.707	0.29363386	24.7435154	0.50280622	0	1.66857437	5.14056767	-1.2220219	3.20860386	-0.6855636	-6.9077553	21	80	2.23519444	301.333333	28.33333333	0	0	0	1	34.0724638	1	0.49277013
5.3343	171952.18	0.33452323	39.399107	0.57151245	0	1.67415767	5.14722223	-1.0920641	3.67376853	-0.5577208	-6.9077553	22	81	2.68223333	301.333333	28.33333333	0	0	0	1	34.0724638	1	0.49277013
5.3343	171952.18	0	0	0	0	1.67415767	5.14722223	-6.9077553	-6.9077553	-6.9077553	-6.9077553	23	83	2.68223333	300.777778	27.7777778	0	0	0	1	34.0724638	1	0.49277013
0.0288	1682.30165	1.05551271	303.526147	2.07385894	0	-3.5473799	0.52075713	0.05497358	5.71547105	0.72989317	-6.9077553	0	88	1.78815556	299.111111	26.11111111	0	0	0	0	42.288408	1	-0.4111751
0.0765	4026.35447	0.73182297	139.372734	1.38738553	0	-2.5704645	1.3931097	-0.3108511	4.93715906	0.32814158	-6.9077553	1	89	2.68223333	299.666667	26.66666666	0	0	0	0	42.288408	1	-0.4111751
0.1368	6879.71072	0.69415226	124.167561	1.30486172	0	-1.9892353	1.92872195	-0.3636244	4.82164	0.26686315	-6.9077553	2	86	4.02335	300.222222	27.2222222	0	0	0	0	42.288408	1	-0.4111751
0.2052	10171.1564	0.69953999	126.337981	1.30584034	0	-1.5837702	2.31965423	-0.3559038	4.83896862	0.26761227	-6.9077553	3	87	3.57631111	300.222222	27.2222222	0	0	0	0	42.288408	1	-0.4111751
0.2727	13373.6599	0.7148705	129.96138	1.29586965	0	-1.299383	2.59336187	-0.334256	4.86724503	0.2599534	-6.9077553	4	87	3.57631111	300.222222	27.2222222	0	0	0	0	42.288408	1	-0.4111751
0.3636	17586.6614	0.71095132	131.918936	1.37369892	0	-1.0117009	2.8671976	-0.3397457	4.88219519	0.31823474	-6.9077553	5	87	4.02335	300.222222	27.2222222	0	0	0	0	42.288408	1	-0.4111751
0.45	21404.0154	0.64748355	109.98114	1.13405267	0	-0.7985077	3.06362526	-0.4331186	4.70031799	0.12667906	-6.9077553	6	88	2.68223333	300.222222	27.2222222	0	0	0	0	42.288408	1	-0.4111751
0.5823	27101.754	0.65855377	124.648517	1.33979974	0	-0.5407695	3.29963535	-0.4161918	4.82550594	0.29326626	-6.9077553	7	84	3.57631111	300.777778	27.7777778	0	0	1	0	42.288408	1	-0.4111751
0.8469	35573.5803	0.75003778	160.67044	1.84023197	0	-0.1661727	3.57163135	-0.2862993	5.07936153	0.61043489	-6.9077553	8	78	3.57631111	301.888889	28.88888888	0	0	1	0	42.288408	1	-0.4111751
1.2375	48697.2776	0.83666627	188.838098	2.02022359	0	0.21309322	3.88564366	-0.1771355	5.24089532	0.70370307	-6.9077553	9	76	4.02335	301.888889	28.88888888	0	0	1	0	42.288408	1	-0.4111751
1.6281	62092.9588	0.82034759	177.680534	1.66476623	0	0.48741369	4.1286487	-0.1968089	5.17999281	0.51028522	-6.9077553	10	66	4.02335	304.111111	31.11111111	0	0	1	0	42.288408	1	-0.4111751
2.0538	79334.8712	0.83357197	182.78087	1.55084293	0	0.71969174	4.37369037	-0.1808363	5.20829348	0.43944321	-6.9077553	11	63	3.57631111	304.666667	31.66666666	0	0	1	0	42.288408	1	-0.4111751
2.7603	106510.196	0.97258752	271.025119	2.99501611	0	1.01533937	4.66825011	-0.0267676	5.6022152	1.09728344	-6.9077553	12	57	5.81150556	305.777778	32.7777778	0	0	1	0	42.288408	1	-0.4111751
3.5946	142852.329	1.00202404	288.394856	2.67365114	0	1.27943272	4.96181843	0.00301947	5.66433404	0.98381896	-6.9077553	13	56	5.36446667	305.777778	32.7777778	0	0	1	0	42.288408	1	-0.4111751
4.4694	185571.306	1.02878624	309.667631	2.53082853	0	1.49725417	5.2234446	0.02935125	5.73550279	0.92894178	-6.9077553	14	55	5.81150556	306.333333	33.33333333	0	0	1	0	42.288408	1	-0.4111751
5.2308	218991.313	0.9463967	259.890219	2.02985456	0	1.65456423	5.38903663	-0.0540374	5.56026315	0.70845667	-6.9077553	15	59	3.12927222	305.777778	32.7777778	0	0	1	0	42.288408	1	-0.4111751
5.7708	237433.64	0.80588994	187.599206	1.63313059	0	1.75281072	5.46989239	-0.214568	5.23431313	0.49111092	-6.9077553	16	59	3.12927222	305.222222	32.2222222	0	0	1	0	42.288408	1	-0.4111751
6.1416	248493.846	0.70799596	148.392938	1.25260244	0	1.81508529	5.5154221	-0.3439054	4.99987048	0.22602136	-6.9077553	17	65	1.78815556	304.666667	31.66666666	0	0	1	0	42.288408	1	-0.4111751
6.3027	255381.774	0.78395848	164.226988	1.23798682	0	1.84097811	5.54276349	-0.2421245	5.10125563	0.21429396	-6.9077553	18	70	1.34111667	304.111111	31.11111111	0	0	1	0	42.288408	1	-0.4111751
6.4422	261142.942	0.70099689	127.067036	1.10846794	0	1.8628701	5.56507176	-0.3538263	4.84472266	0.10388057	-6.9077553	19	73	1.78815556	303	30	0	0	1	0	42.288408	1	-0.4111751
6.5268	264695.915	0.46964694	55.6787213	0.72951232	0	1.87591678	5.57858546	-0.7536471	4.01961601	-0.3140092	-6.9077553	20	76	2.68223333	301.888889	28.88888888	0	0	1	0	42.288408	1	-0.4111751
6.5727	266713.637	0.3073798	21.858633	0.4658683	0	1.88292471	5.58617931	-1.1764231	3.08464169	-0.7617081	-6.9077553	21	80	2.23519444	301.333333	28.33333333	0	0	1	0	42.288408	1	-0.4111751

6.6141	268037.781	0.44513134	77.6641209	0.86960079	0	1.88920373	5.59113167	-0.8071419	4.35240626	-0.1385717	-6.9077553	22	81	2.68223333	301.333333	28.33333333	0	0	1	0	42.288408	1	-0.4111751
6.6141	268037.781	0	0	0	0	1.88920373	5.59113167	-6.9077553	-6.9077553	-6.9077553	-6.9077553	23	83	2.68223333	300.777778	27.7777778	0	0	1	0	42.288408	1	-0.4111751
0.0009	0	0	0	0	0	-7.0131158	-6.9077553	-6.9077553	-6.9077553	-6.9077553	-6.9077553	0	88	1.78815556	299.111111	26.11111111	0	0	0	0	18.9135952	1	-0.0428739
0.0009	0	0	0	0	0	-7.0131158	-6.9077553	-6.9077553	-6.9077553	-6.9077553	-6.9077553	1	89	2.68223333	299.666667	26.66666666	0	0	0	0	18.9135952	1	-0.0428739
0.0009	0	0	0	0	0	-7.0131158	-6.9077553	-6.9077553	-6.9077553	-6.9077553	-6.9077553	2	86	4.02335	300.222222	27.2222222	0	0	0	0	18.9135952	1	-0.0428739
0.0009	0	0	0	0	0	-7.0131158	-6.9077553	-6.9077553	-6.9077553	-6.9077553	-6.9077553	3	87	3.57631111	300.222222	27.2222222	0	0	0	0	18.9135952	1	-0.0428739
0.0009	0	0	0	0	0	-7.0131158	-6.9077553	-6.9077553	-6.9077553	-6.9077553	-6.9077553	4	87	3.57631111	300.222222	27.2222222	0	0	0	0	18.9135952	1	-0.0428739
0.0009	0	0	0	0	0	-7.0131158	-6.9077553	-6.9077553	-6.9077553	-6.9077553	-6.9077553	5	87	4.02335	300.222222	27.2222222	0	0	0	0	18.9135952	1	-0.0428739
0.0009	0	0	0	0	0	-7.0131158	-6.9077553	-6.9077553	-6.9077553	-6.9077553	-6.9077553	6	88	2.68223333	300.222222	27.2222222	0	0	0	0	18.9135952	1	-0.0428739
0.0009	0	0	0	0	0	-7.0131158	-6.9077553	-6.9077553	-6.9077553	-6.9077553	-6.9077553	7	84	3.57631111	300.777778	27.7777778	0	0	0	0	18.9135952	1	-0.0428739
0.0117	0	0.4582967	57.3482463	1.63751539	0	-4.4481664	-6.9077553	-0.7780589	4.0491597	0.49379058	-6.9077553	8	78	3.57631111	301.888889	28.88888888	0	0	0	0	18.9135952	1	-0.0428739
0.0594	146.824491	0.64208013	118.391638	2.39296564	0	-2.8234611	-1.9117296	-0.4414859	4.77400654	0.87295125	-6.9077553	9	76	4.02335	301.888889	28.88888888	0	0	0	0	18.9135952	1	-0.0428739
0.1575	1201.53771	0.62132137	101.646986	1.83179425	0	-1.8483298	0.18443408	-0.4742986	4.62151573	0.60584171	-6.9077553	10	66	4.02335	304.111111	31.11111111	0	0	1	0	18.9135952	1	-0.0428739
0.3231	5773.31201	0.77306333	200.506135	2.00768147	0	-1.1297934	1.75341912	-0.2561016	5.30084983	0.69747852	-6.9077553	11	63	3.57631111	304.666667	31.66666666	0	0	1	0	18.9135952	1	-0.0428739
0.5931	15142.2553	0.86235484	253.335573	2.62108236	0	-0.5223923	2.71755524	-0.1469295	5.53471893	0.96396879	-6.9077553	12	57	5.81150556	305.777778	32.7777778	0	0	1	0	18.9135952	1	-0.0428739
0.855	24059.6325	0.89016755	264.671526	2.38424169	0	-0.1566538	3.180577	-0.1152228	5.57849331	0.86930046	-6.9077553	13	56	5.36446667	305.777778	32.7777778	0	0	1	0	18.9135952	1	-0.0428739
1.2249	35603.3171	0.90590361	247.157333	2.69555248	0	0.20285921	3.5724669	-0.0977191	5.51002916	0.9919741	-6.9077553	14	55	5.81150556	306.333333	33.33333333	0	0	1	0	18.9135952	1	-0.0428739
1.5255	47786.3861	0.88303179	230.755473	1.82150689	0	0.42232223	3.86676171	-0.1232623	5.44136293	0.60021296	-6.9077553	15	59	3.12927222	305.777778	32.7777778	0	0	1	0	18.9135952	1	-0.0428739
1.8027	57983.6722	0.80488537	192.48265	1.67426647	0	0.58928554	4.0601787	-0.2158138	5.26001122	0.51597224	-6.9077553	16	59	3.12927222	305.222222	32.2222222	0	0	1	0	18.9135952	1	-0.0428739
2.0934	69543.1094	0.821814	195.351091	1.56646114	0	0.73878954	4.24196122	-0.1950251	5.27480352	0.4494572	-6.9077553	17	65	1.78815556	304.666667	31.66666666	0	0	1	0	18.9135952	1	-0.0428739
2.3346	81377.8441	0.91345295	223.274123	1.64174504	0	0.84784057	4.39911534	-0.0894293	5.40840474	0.49636865	-6.9077553	18	70	1.34111667	304.111111	31.11111111	0	0	1	0	18.9135952	1	-0.0428739
2.5128	89462.5864	0.76335329	154.895942	1.39803152	0	0.92139767	4.49383169	-0.2687252	5.04276	0.33578023	-6.9077553	19	73	1.78815556	303	30	0	0	1	0	18.9135952	1	-0.0428739
2.5983	93366.4652	0.47862629	57.2938117	0.86008823	0	0.95485739	4.53654295	-0.734748	4.04821007	-0.1495583	-6.9077553	20	76	2.68223333	301.888889	28.88888888	0	0	1	0	18.9135952	1	-0.0428739
2.6406	95340.3378	0.33138095	25.1259714	0.52075934	0	0.97100616	4.55746348	-1.1014735	3.22394183	-0.6505488	-6.9077553	21	80	2.23519444	301.333333	28.33333333	0	0	1	0	18.9135952	1	-0.0428739
2.6919	97752.8404	0.27354985	16.3540949	0.41227193	0	0.99024726	4.58245249	-1.2926224	2.79453946	-0.8836495	-6.9077553	22	81	2.68223333	301.333333	28.33333333	0	0	1	0	18.9135952	1	-0.0428739
2.6919	97752.8404	0	0	0	0	0.99024726	4.58245249	-6.9077553	-6.9077553	-6.9077553	-6.9077553	23	83	2.68223333	300.777778	27.7777778	0	0	1	0	18.9135952	1	-0.0428739
0.009	363.014982	0.85538605	196.563002	1.62470288	0	-4.7105307	-1.0105603	-0.155034	5.28098809	0.48594026	-6.9077553	0	83	4.02335	299.666667	26.66666666	0	0	0	0	30.519848	1	0.21141735
0.0135	582.333993	0.45712867	54.372644	0.84110222	0	-4.3050656	-0.5389954	-0.7806052	3.99587955	-0.1718539	-6.9077553	1	87	4.02335	299.111111	26.11111111	0	0	0	0	30.519848	1	0.21141735
0.0261	1064.83561	0.63504212	97.9498291	1.02893932	0	-3.64582	0.0637591	-0.4524905	4.58446561	0.02949988	-6.9077553	2	89	3.57631111	298.555556	25.55555555	0	0	0	0	30.519848	1	0.21141735
0.0351	1240.29064	0.55142641	75.6119019	0.97449207	0	-3.3495541	0.21615168	-0.593435	4.32562693	-0.0248132	-6.9077553	3	92	4.02335	298.555556	25.55555555	0	0	0	0	30.519848	1	0.21141735
0.0423	1371.88206	0.39470023	40.0940514	0.87624097	0	-3.1629682	0.31691222	-0.9270984	3.69125292	-0.1309736	-6.9077553	4	88	4.91742778	298.555556	25.55555555	0	0	0	0	30.519848	1	0.21141735
0.0441	1415.74577	0.52579826	64.2198486	0.64548671	0	-3.1212955	0.34836253	-0.6409376	4.1623279	-0.4362026	-6.9077553	5	87	4.02335	298.555556	25.55555555	0	0	0	0	30.519848	1	0.21141735
0.0909	1649.20929	0.30207779	24.5875549	1.47486452	0	-2.3979953	0.50090212	-1.1937658	3.20228109	0.38924393	-6.9077553	6	82	4.91742778	299.666667	26.66666666	0	0	0	0	30.519848	1	0.21141735
0.2025	2026.0079	0.51986368	80.2912755	2.29456305	0	-1.5970154	0.70656077	-0.6522669	4.38567342	0.83097815	-6.9077553	7	74	5.36446667	301.333333	28.33333333	0	0	0	0	30.519848	1	0.21141735
0.2889	3679.88346	0.58211859	96.9496257	1.77938747	0	-1.2416747	1.3031528	-0.5393647	4.57420184	0.57683102	-6.9077553	8	68	5.36446667	302.444444	29.44444444	0	0	0	0	30.519848	1	0.21141735
0.3942	8003.4761	0.59306114	96.3787143	1.47453139	0	-0.9308969	2.0800009	-0.520773	4.56829575	0.38901819	-6.9077553	9	63	5.81150556	303.555556	30.55555555	0	0	0	0	30.519848	1	0.21141735
0.5832	14566.2155	0.65853279	123.887202	1.98821062	0	-0.5392251	2.67877349	-0.4162236	4.81937957	0.68773789	-6.9077553	10	60	4.91742778	304.666667	31.66666666	0	0	0	1	30.519848	1	0.21141735
0.8622	25234.2742	0.74451117	167.668674	2.25054183	0	-0.148268	3.22824278	-0.2936852	5.12199582	0.81161524	-6.9077553	11	55	4.91742778	306.888889	33.88888888	0	0	0	1	30.519848	1	0.21141735
1.1997	35680.1184	0.79764583	184.35725	2.91563542	0	0.18207153	3.57462165	-0.2248377	5.21688088	1.0704307	-6.9077553	12	51	5.81150556	307.444444	34.4444444	0	0	0	1	30.519848	1	0.21141735
1.5102	48526.4575	0.72582309	145.483152	1.67696374	0	0.41224209	3.88212977	-0.3190722	4.98006716	0.517581	-6.9077553	13	49	5.36446667	308.555556	35.5555556	0	0	0	1	30.519848	1	0.21141735
1.7739	63240.6165	0.73845759	151.791049	1.42617683	0	0.57318051	4.14696257	-0.3018384	5.02251149	0.35569825	-6.9077553	14	52	4.47038889	306.888889	33.88888888	0	0	0	1	30.519848	1	0.21141735
2.1213	79464.726	0.82503324	227.024996	2.08397947	0	0.75202911	4.37532581	-0.1911203	5.42506453	0.73475901	-6.9077553	15	52	4.47038889	307.444444	34.4444444	0	0	0	1	30.519848	1	0.21141735
2.412	96276.7521	0.67690227	134.533512	1.62007335	0	0.88045628	4.56723727	-0.3887521	4.90182076	0.48308849	-6.9077553	16	54	3.57631111	306.888889	33.88888888	0	0	0	1	30.519848	1	0.21141735
2.5767	111718.3	0.63902612	113.136303	0.96244203	0	0.94650951	4.71598948	-0.4462463	4.72860215	-0.037243	-6.9077553	17	58	2.68223333	305.777778	32.7777778	0	0	0	1	30.519848	1	0.21141735
2.6802	128195.898	0.72462609	143.340031	0.90584849	0	0.98589142	4.85356735	-0.3207204	4.96522662	-0.0977799	-6.9077553	18	65	1.78815556	304.111111	31.11111111	0	0	0	1	30.519848	1	0.21141735
2.7684	141296.629	0.69531125	128.337313	0.89152924	0	1.01826954	4.95086851	-0.3619585	4.85466984	-0.113696	-6.9077553	19	70	1.78815556	303	30	0	0	0	1	30.519848	1	0.21141735
2.8476	152903.771	0.61507221	103.449996	0.86989741	0	1.04647653	5.02981532	-0.4843911	4.63909803	-0.1382311	-6.9077553	20	77	0.44703889	301.888889	28.88888888	0	0	0	1	30.519848	1	0.21141735
2.8701	153824.902	0.50894524	62.3393848	0.80514259	0	1.05434687	5.03582146	-0.6734519	4.13260945	-0.2154946	-6.9077553	21	75	0.89407778	297.444444	24.44444444	0	0	0	1	30.519848	1	0.21141735
2.8845	154307.403	0.4651691	50.8031082	0.70099103	0	1.05935157	5.03895322	-0.7632068	3.92797722	-0.3538346	-6.9077553	22	88	0.89407778	295.222222	22.2222222	0	0	0	1	30.519848	1	0.21141735
2.8926	154658.316	0.25379329	13.7970479	0.43092842	0	1.06215575	5.04122474	-1.3673027	2.62452713	-0.8394954	-6.9077553	23	46	2.23519444	296.888889	23.88888888	0	0	0	1	30.519848	1	0.21141735
0.0171		0.99409019			0	-4.0686768		-0.0049219			-6.9077553	0	83	4.02335	299.666667	26.66666666	0	0	0	0	34.0724638	1	0.49277013
0.0324		0.54136355			0	-3.4295969		-0.6118187			-6.9077553	1	87	4.02335	299.111111	26.11111111	0	0	0	0	34.0724638	1	0.49277013
0.0513		0.50130844			0	-2.9700645		-0.6885409			-6.9077553	2	89	3.57631111	298.555556	25.55555555	0	0	0	0	34.0724638	1	0.49277013
0.0756		0.59941589			0	-2.582299		-0.5101327			-6.9077553	3	92	4.02335	298.555556	25.55555555	0	0	0	0	34.0724638	1	0.49277013
0.1134		0.59760239			0	-2.1768339		-0.5131577			-6.9077553	4	88	4.91742778	298.555556	25.55555555	0	0	0	0	34.0724638	1	0.49277013
0.1539		0.59418644			0	-1.8714522		-0.5188806			-6.9077553	5	87	4.02335	298.555556	25.55555555	0	0	0	0	34.0724638	1	0.49277013
0.1899		0.62024946			0	-1.6612577		-0.4760226			-6.9077553	6	82	4.91742778	299.666667	26.66666666	0	0	0	0	34.0724638	1	0.49277013
0.288		0.91713232			0	-1.2447948		-0.0854138			-6.9077553	7	74	5.36446667	301.333333	28.33333333	0	0	0	1	34.0724638	1	0.49277013
0.5067		1.12383933			0	-0.6798362		0.11764021			-6.9077553	8	68	5.36446667	302.444444	29.44444444	0	0	0	1	34.0724638	1	0.49277013
1.044		1.35904294			2	0.04305949		0.30751627			0.69364706	9	63	5.81150556	303.555556	30.55555555	0	0	0	1	34.0724638	1	0.49277013
1.6578		1.24200708			2	0.50549142		0.21753351			0.69364706	10	60	4.91742778	304.666667	31.66666666	0	0	0	1	34.0724638	1	0.49277013
2.2392		1.09112483			2	0.80611866		0.08812518			0.69364706	11	55	4.91742778	306.888889	33.88888888	0	0	0	1	34.0724638	1	0.49277013

2.889		1.13876301			3	1.06091042		0.13082035			1.09894557	12	51	5.81150556	307.444444	34.4444444	0	0	0	1	34.0724638	1	0.49277013
3.5361		1.01226323			3	1.26302442		0.01317604			1.09894557	13	49	5.36446667	308.555556	35.5555556	0	0	0	1	34.0724638	1	0.49277013
4.1319		0.88610969			3	1.41873735		-0.1197866			1.09894557	14	52	4.47038889	306.888889	33.88888888	0	0	0	1	34.0724638	1	0.49277013
4.6692		0.85417035			3	1.54098775		-0.1564546			1.09894557	15	52	4.47038889	307.444444	34.4444444	0	0	0	1	34.0724638	1	0.49277013
5.1201		0.77982499			3	1.63317397		-0.2474042			1.09894557	16	54	3.57631111	306.888889	33.88888888	0	0	0	1	34.0724638	1	0.49277013
5.4846		0.71835094			3	1.70194416		-0.329406			1.09894557	17	58	2.68223333	305.777778	32.7777778	0	0	0	1	34.0724638	1	0.49277013
5.6952		0.79127111			3	1.73962371		-0.2328516			1.09894557	18	65	1.78815556	304.111111	31.11111111	0	0	0	1	34.0724638	1	0.49277013
5.8896		0.8047958			3	1.77318808		-0.2159249			1.09894557	19	70	1.78815556	303	30	0	0	0	1	34.0724638	1	0.49277013
6.0525		0.72604235			3	1.80047141		-0.3187705			1.09894557	20	77	0.44703889	301.888889	28.88888888	0	0	0	1	34.0724638	1	0.49277013
6.1389		0.39296778			3	1.81464557		-0.9314861			1.09894557	21	75	0.89407778	297.444444	24.44444444	0	0	0	1	34.0724638	1	0.49277013
6.21		0.40109825			3	1.8261609		-0.9110588			1.09894557	22	88	0.89407778	295.222222	22.2222222	0	0	0	1	34.0724638	1	0.49277013
6.2901		0.26814825			3	1.83897697		-1.3124929			1.09894557	23	46	2.23519444	296.888889	23.88888888	0	0	0	1	34.0724638	1	0.49277013
0.0351	2265.73398	1.13087172	354.089921	2.44504266	0	-3.3495541	0.81834002	0.12387265	5.86955372	0.89447148	-6.9077553	0	83	4.02335	299.666667	26.66666666	0	0	0	0	42.288408	1	-0.4111751
0.0837	4647.38804	0.73338629	141.355378	1.4281992	0	-2.4805163	1.5365205	-0.3087201	4.95128421	0.35711429	-6.9077553	1	87	4.02335	299.111111	26.11111111	0	0	0	0	42.288408	1	-0.4111751
0.1449	7586.44491	0.67231779	117.673677	1.29837732	0	-1.9317114	2.0264949	-0.3955379	4.76792385	0.26188516	-6.9077553	2	89	3.57631111	298.555556	25.55555555	0	0	0	0	42.288408	1	-0.4111751
0.2079	10613.1491	0.67471395	116.785519	1.28225741	0	-1.5706981	2.36218793	-0.3919854	4.76034764	0.24940169	-6.9077553	3	92	4.02335	298.555556	25.55555555	0	0	0	0	42.288408	1	-0.4111751
0.2835	14124.25	0.69633152	123.853713	1.28653354	0	-1.2605432	2.64796398	-0.3604943	4.81910921	0.2527284	-6.9077553	4	88	4.91742778	298.555556	25.55555555	0	0	0	0	42.288408	1	-0.4111751
0.3798	18527.7168	0.74457186	144.491412	1.3610402	0	-0.9681105	2.91932179	-0.2936038	4.97322699	0.30898373	-6.9077553	5	87	4.02335	298.555556	25.55555555	0	0	0	0	42.288408	1	-0.4111751
0.5247	23837.7329	0.68225036	131.46892	1.67973261	0	-0.6449286	3.17131169	-0.3808939	4.87877808	0.51922978	-6.9077553	6	82	4.91742778	299.666667	26.66666666	0	0	0	0	42.288408	1	-0.4111751
0.8496	35064.0334	0.84143219	201.121797	2.48597223	0	-0.1629896	3.55720443	-0.1714621	5.30391565	0.911066	-6.9077553	7	74	5.36446667	301.333333	28.33333333	0	0	1	0	42.288408	1	-0.4111751
1.2357	49297.6206	0.87742768	207.590727	2.17941334	0	0.21163761	3.8978961	-0.1296217	5.3355733	0.77951446	-6.9077553	8	68	5.36446667	302.444444	29.44444444	0	0	1	0	42.288408	1	-0.4111751
1.6938	67503.2095	0.8992065	218.601348	2.10960523	0	0.52697453	4.21218996	-0.1051311	5.38725432	0.74697475	-6.9077553	9	63	5.81150556	303.555556	30.55555555	0	0	1	0	42.288408	1	-0.4111751
2.3499	90883.8028	0.90789971	231.002379	2.57284115	0	0.85437277	4.5095928	-0.0955205	5.44243234	0.94539939	-6.9077553	10	60	4.91742778	304.666667	31.66666666	0	0	1	0	42.288408	1	-0.4111751
3.0753	121876.414	0.98419841	275.608367	2.56422534	0	1.12340246	4.80301574	-0.0149122	5.61898453	0.94204633	-6.9077553	11	55	4.91742778	306.888889	33.88888888	0	0	1	0	42.288408	1	-0.4111751
3.8871	163952.531	1.07547146	334.259354	2.64680917	0	1.35766338	5.09958304	0.07368853	5.81192019	0.97373257	-6.9077553	12	51	5.81150556	307.444444	34.4444444	0	0	1	0	42.288408	1	-0.4111751
4.7592	206291.198	1.08277451	350.605392	2.8977396	1	1.56007959	5.3292936	0.08044986	5.8596642	1.06427602	0.0009995	13	49	5.36446667	308.555556	35.5555556	0	0	1	0	42.288408	1	-0.4111751
5.3622	228266.478	0.8638149	211.072271	1.93781593	1	1.67937434	5.43051809	-0.1452398	5.35220533	0.66207744	0.0009995	14	52	4.47038889	306.888889	33.88888888	0	0	1	0	42.288408	1	-0.4111751
5.9544	247883.337	0.79435605	183.953137	1.85225188	1	1.78413044	5.51296225	-0.2289654	5.21468647	0.61694187	0.0009995	15	52	4.47038889	307.444444	34.4444444	0	0	1	0	42.288408	1	-0.4111751
6.444	262610.763	0.74533059	164.191478	1.44906078	1	1.86314947	5.57067676	-0.2925866	5.10103939	0.37160547	0.0009995	16	54	3.57631111	306.888889	33.88888888	0	0	1	0	42.288408	1	-0.4111751
6.7563	271881.011	0.67006923	139.78886	1.11228499	1	1.9104754	5.60536819	-0.398883	4.94014029	0.10731509	0.0009995	17	58	2.68223333	305.777778	32.7777778	0	0	1	0	42.288408	1	-0.4111751
6.9075	278122.283	0.78406815	166.284319	1.16885994	1	1.93260778	5.62806448	-0.2419847	5.1137051	0.15688403	0.0009995	18	65	1.78815556	304.111111	31.11111111	0	0	1	0	42.288408	1	-0.4111751
7.0416	284142.76	0.76233893	150.908164	1.14643358	1	1.95183542	5.64948031	-0.2700531	5.01667809	0.13752778	0.0009995	19	70	1.78815556	303	30	0	0	1	0	42.288408	1	-0.4111751
7.1748	289284.743	0.67039742	117.411252	1.07207303	1	1.97057489	5.66741493	-0.398394	4.76569126	0.07052653	0.0009995	20	77	0.44703889	301.888889	28.88888888	0	0	1	0	42.288408	1	-0.4111751
7.2225	291127.013	0.39961117	38.4459182	0.65889589	1	1.97720115	5.67376308	-0.914764	3.64927854	-0.4156732	0.0009995	21	75	0.89407778	297.444444	24.44444444	0	0	1	0	42.288408	1	-0.4111751
7.2675	292837.695	0.34929138	30.5794116	0.5222102	1	1.98341235	5.67962193	-1.04899	3.42035966	-0.647772	0.0009995	22	88	0.89407778	295.222222	22.2222222	0	0	1	0	42.288408	1	-0.4111751
7.3566	296191.772	0.52056079	83.8268328	0.91032672	1	1.99559787	5.6910105	-0.6509294	4.42876509	-0.0928538	0.0009995	23	46	2.23519444	296.888889	23.88888888	0	0	1	0	42.288408	1	-0.4111751
0.0009	0	0	0	0	0	-7.0131158	-6.9077553	-6.9077553	-6.9077553	-6.9077553	-6.9077553	0	83	4.02335	299.666667	26.66666666	0	0	0	0	18.9135952	1	-0.0428739
0.0009	0	0	0	0	0	-7.0131158	-6.9077553	-6.9077553	-6.9077553	-6.9077553	-6.9077553	1	87	4.02335	299.111111	26.11111111	0	0	0	0	18.9135952	1	-0.0428739
0.0009	0	0	0	0	0	-7.0131158	-6.9077553	-6.9077553	-6.9077553	-6.9077553	-6.9077553	2	89	3.57631111	298.555556	25.55555555	0	0	0	0	18.9135952	1	-0.0428739
0.0009	0	0	0	0	0	-7.0131158	-6.9077553	-6.9077553	-6.9077553	-6.9077553	-6.9077553	3	92	4.02335	298.555556	25.55555555	0	0	0	0	18.9135952	1	-0.0428739
0.0009	0	0	0	0	0	-7.0131158	-6.9077553	-6.9077553	-6.9077553	-6.9077553	-6.9077553	4	88	4.91742778	298.555556	25.55555555	0	0	0	0	18.9135952	1	-0.0428739
0.0009	0	0	0	0	0	-7.0131158	-6.9077553	-6.9077553	-6.9077553	-6.9077553	-6.9077553	5	87	4.02335	298.555556	25.55555555	0	0	0	0	18.9135952	1	-0.0428739
0.0009	0	0	0	0	0	-7.0131158	-6.9077553	-6.9077553	-6.9077553	-6.9077553	-6.9077553	6	82	4.91742778	299.666667	26.66666666	0	0	0	0	18.9135952	1	-0.0428739
0.0468	65.3945018	0.67982371	128.679142	3.08706725	0	-3.0618721	-2.712141	-0.3844519	4.8573298	1.12754541	-6.9077553	7	74	5.36446667	301.333333	28.33333333	0	0	0	0	18.9135952	1	-0.0428739
0.2025	3667.60365	0.81978479	221.147918	2.65244783	0	-1.5970154	1.29981111	-0.1974943	5.39883631	0.97585986	-6.9077553	8	68	5.36446667	302.444444	29.44444444	0	0	0	0	18.9135952	1	-0.0428739
0.4716	12088.4037	0.86030533	243.863503	2.79841905	0	-0.7516241	2.49232934	-0.1493062	5.49661276	1.02941191	-6.9077553	9	63	5.81150556	303.555556	30.55555555	0	0	1	0	18.9135952	1	-0.0428739
0.6462	16127.6982	0.77399681	208.90325	1.97877581	0	-0.4366462	2.78060018	-0.2548964	5.34187601	0.68298361	-6.9077553	10	60	4.91742778	304.666667	31.66666666	0	0	1	0	18.9135952	1	-0.0428739
0.8667	23133.5987	0.81931688	206.938409	2.00482414	0	-0.1430624	3.14132928	-0.1980646	5.33242604	0.69605502	-6.9077553	11	55	4.91742778	306.888889	33.88888888	0	0	1	0	18.9135952	1	-0.0428739
1.2024	34068.347	0.89537331	247.885661	2.75683201	0	0.18431956	3.52839807	-0.1093983	5.51297163	1.01444487	-6.9077553	12	51	5.81150556	307.444444	34.4444444	0	0	1	0	18.9135952	1	-0.0428739
1.5696	49774.8843	0.94975984	270.676145	2.17031606	0	0.45082081	3.90753062	-0.0504938	5.60092676	0.77533346	-6.9077553	13	49	5.36446667	308.555556	35.5555556	0	0	1	0	18.9135952	1	-0.0428739
1.8657	61103.602	0.86310334	219.024791	1.821728	0	0.62363632	4.11258718	-0.1460629	5.38918949	0.60033428	-6.9077553	14	52	4.47038889	306.888889	33.88888888	0	0	1	0	18.9135952	1	-0.0428739
2.2293	75713.6142	0.89044869	239.114287	2.02397987	0	0.80168763	4.3269712	-0.1149074	5.47694581	0.70555976	-6.9077553	15	52	4.47038889	307.444444	34.4444444	0	0	1	0	18.9135952	1	-0.0428739
2.5839	90513.8839	0.86700641	221.685636	1.75293759	0	0.94929989	4.5055143	-0.1415562	5.40126484	0.56186331	-6.9077553	16	54	3.57631111	306.888889	33.88888888	0	0	1	0	18.9135952	1	-0.0428739
2.853	99451.2562	0.72227845	155.815518	1.38424958	0	1.04837107	4.59967769	-0.323961	5.04867915	0.32588033	-6.9077553	17	58	2.68223333	305.777778	32.7777778	0	0	1	0	18.9135952	1	-0.0428739
3.0564	108825.557	0.90024249	225.957757	1.62276492	0	1.11723775	4.68975539	-0.1039809	5.42035249	0.48474748	-6.9077553	18	65	1.78815556	304.111111	31.11111111	0	0	1	0	18.9135952	1	-0.0428739
3.2013	115583.912	0.78767547	164.108307	1.37504682	0	1.16355698	4.75000543	-0.2374004	5.10053271	0.31921476	-6.9077553	19	70	1.78815556	303	30	0	0	1	0	18.9135952	1	-0.0428739
3.3525	122494.762	0.76282892	154.119187	1.34729594	0	1.20970634	4.80807643	-0.2694114	5.03773273	0.29884153	-6.9077553	20	77	0.44703889	301.888889	28.88888888	0	0	1	0	18.9135952	1	-0.0428739
3.4416	126398.644	0.52096816	67.978491	0.93384282	0	1.23593648	4.83944867	-0.6501487	4.21920606	-0.0673769	-6.9077553	21	75	0.89407778	297.444444	24.44444444	0	0	1	0	18.9135952	1	-0.0428739
3.5208	130039.334	0.4608404	52.7269232	0.82843642	0	1.25868824	4.86784466	-0.7725359	3.96514517	-0.1870088	-6.9077553	22	88	0.89407778	295.222222	22.2222222	0	0	1	0	18.9135952	1	-0.0428739
3.5901	132633.067	0.41723767	56.0027648	0.70288527	0	1.27818006	4.88759396	-0.8717054	4.02541892	-0.3511399	-6.9077553	23	46	2.23519444	296.888889	23.88888888	0	0	1	0	18.9135952	1	-0.0428739
0.0153	409.20374	0.87856716	211.063995	1.65889704	0	-4.1799025	-0.8911013	-0.1283254	5.35216612	0.50675558	-6.9077553	0	62	1.34111667	304.111111	31.11111111	0	0	0	0	30.519848	1	0.21141735
0.0306	1117.35997	0.77225719	157.05743	1.23981083	0	-3.4867553	0.1118633	-0.2571436	5.0566179	0.21576506	-6.9077553	1	71	1.78815556	301.333333	28.33333333	0	0	0	0	30.519848	1	0.21141735

0.0414	1380.54262	0.71717453	135.058075	1.46041775	0	-3.1844744	0.32320071	-0.3310427	4.90571227	0.37940702	-6.9077553	2	75	1.78815556	300.222222	27.2222222	0	0	0	0	30.519848	1	0.21141735
0.0441	1424.40645	0.39757208	35.8098602	0.74242592	0	-3.1212955	0.354457	-0.9198669	3.57825121	-0.2964862	-6.9077553	3	82	1.34111667	299.111111	26.11111111	0	0	0	0	30.519848	1	0.21141735
0.0441	1424.40645	0	0	0	0	-3.1212955	0.354457	-6.9077553	-6.9077553	-6.9077553	-6.9077553	4	89	1.34111667	298	25	0	0	0	0	30.519848	1	0.21141735
0.0441	1424.40645	0	0	0	0	-3.1212955	0.354457	-6.9077553	-6.9077553	-6.9077553	-6.9077553	5	90	1.34111667	298	25	0	0	0	0	30.519848	1	0.21141735
0.0441	1424.40645	0	0	0	0	-3.1212955	0.354457	-6.9077553	-6.9077553	-6.9077553	-6.9077553	6	91	1.34111667	297.444444	24.44444444	0	0	0	0	30.519848	1	0.21141735
0.0441	1424.40645	0	0	0	0	-3.1212955	0.354457	-6.9077553	-6.9077553	-6.9077553	-6.9077553	7	93	1.34111667	297.444444	24.44444444	0	0	0	0	30.519848	1	0.21141735
0.0441	1424.40645	0	0	0	0	-3.1212955	0.354457	-6.9077553	-6.9077553	-6.9077553	-6.9077553	8	93	1.78815556	297.444444	24.44444444	0	0	0	0	30.519848	1	0.21141735
0.0441	1424.40645	0	0	0	0	-3.1212955	0.354457	-6.9077553	-6.9077553	-6.9077553	-6.9077553	9	93	2.23519444	297.444444	24.44444444	0	0	0	0	30.519848	1	0.21141735
0.0441	1424.40645	0	0	0	0	-3.1212955	0.354457	-6.9077553	-6.9077553	-6.9077553	-6.9077553	10	94	1.34111667	297.444444	24.44444444	0	0	0	0	30.519848	1	0.21141735
0.0441	1424.40645	0	0	0	0	-3.1212955	0.354457	-6.9077553	-6.9077553	-6.9077553	-6.9077553	11	92	1.34111667	298	25	0	0	0	0	30.519848	1	0.21141735
0.0441	1424.40645	0	0	0	0	-3.1212955	0.354457	-6.9077553	-6.9077553	-6.9077553	-6.9077553	12	88	2.68223333	299.111111	26.11111111	0	0	0	0	30.519848	1	0.21141735
0.0783	1657.52643	0.32598902	31.1293463	1.20721024	0	-2.5472077	0.50592951	-1.1178287	3.43818311	0.18914012	-6.9077553	13	83	3.57631111	300.222222	27.2222222	0	0	0	0	30.519848	1	0.21141735
0.2097	2174.48794	0.53604628	82.3778896	1.9983554	0	-1.5620773	0.77725298	-0.621671	4.41132921	0.69282483	-6.9077553	14	73	3.57631111	302.444444	29.44444444	0	0	0	0	30.519848	1	0.21141735
0.3645	3305.01579	0.52511375	80.9783453	1.77357873	0	-1.0092287	1.19574378	-0.6422378	4.39419413	0.57356306	-6.9077553	15	63	3.12927222	304.111111	31.11111111	0	0	0	0	30.519848	1	0.21141735
0.5247	4691.23417	0.56074352	85.8873332	1.54667818	0	-0.6449286	1.54590884	-0.5767099	4.453048	0.43675586	-6.9077553	16	55	2.68223333	306.333333	33.33333333	0	0	0	0	30.519848	1	0.21141735
0.6984	6261.33659	0.6258052	115.68498	1.69329834	0	-0.3589633	1.83455337	-0.4671195	4.75087945	0.5272687	-6.9077553	17	50	3.12927222	307.444444	34.4444444	0	0	0	1	30.519848	1	0.21141735
0.9765	8813.72024	0.77557946	172.002731	2.40576567	0	-0.0237805	2.17642308	-0.2528563	5.14751617	0.8782838	-6.9077553	18	47	3.57631111	307.444444	34.4444444	0	0	0	1	30.519848	1	0.21141735
1.2672	13669.4463	0.77086817	159.358141	1.80396226	0	0.23680974	2.6152363	-0.2589415	5.07116041	0.59053968	-6.9077553	19	44	2.23519444	308.555556	35.5555556	0	0	0	1	30.519848	1	0.21141735
1.5714	19153.9495	0.84389934	206.324565	2.0530679	0	0.45196694	2.95256114	-0.1685378	5.32945534	0.71982217	-6.9077553	20	43	2.23519444	309.111111	36.11111111	0	0	0	1	30.519848	1	0.21141735
1.9575	24473.5733	0.79869275	179.528919	3.27995162	0	0.67166815	3.19763475	-0.2235277	5.19034187	1.18813351	-6.9077553	21	88	5.81150556	296.333333	23.33333333	0	0	0	1	30.519848	1	0.21141735
2.0025	26084.2081	0.49566544	99.7557813	0.9702597	0	0.6943964	3.26136841	-0.6998386	4.60273504	-0.0291614	-6.9077553	22	90	2.23519444	296.888889	23.88888888	0	0	0	1	30.519848	1	0.21141735
2.0232	26873.757	0.22064576	10.3866378	0.40826926	0	0.70468042	3.29118744	-1.5066748	2.34061643	-0.893382	-6.9077553	23	91	1.34111667	296.888889	23.88888888	0	0	0	1	30.519848	1	0.21141735
0.0261	933.039486	0.97959811	268.572248	2.06864847	0	-3.64582	-0.0682366	-0.0195926	5.59312368	0.72737877	-6.9077553	0	62	1.34111667	304.111111	31.11111111	0	0	0	0	34.0724638	1	0.49277013
0.0585	2312.93842	0.80771393	174.73919	1.32383188	0	-2.8387285	0.83895102	-0.21231	5.16330024	0.28128557	-6.9077553	1	71	1.78815556	301.333333	28.33333333	0	0	0	0	34.0724638	1	0.49277013
0.0927	3807.1765	0.71061691	128.063734	1.09104438	0	-2.3783868	1.33715047	-0.3402156	4.85253587	0.08805151	-6.9077553	2	75	1.78815556	300.222222	27.2222222	0	0	0	0	34.0724638	1	0.49277013
0.1233	5298.54549	0.49849252	60.7448012	0.6889548	0	-2.0931349	1.66762106	-0.6941627	4.10669796	-0.3711292	-6.9077553	3	82	1.34111667	299.111111	26.11111111	0	0	0	0	34.0724638	1	0.49277013
0.1422	6088.09359	0.35206901	28.2989211	0.44809019	0	-1.9505208	1.80649924	-1.0410917	3.34285902	-0.8005315	-6.9077553	4	89	1.34111667	298	25	0	0	0	0	34.0724638	1	0.49277013
0.1593	6921.50504	0.24449429	12.3659813	0.29278431	0	-1.8369661	1.9347777	-1.4044816	2.51503012	-1.2249094	-6.9077553	5	90	1.34111667	298	25	0	0	0	0	34.0724638	1	0.49277013
0.1737	7623.32573	0.24187946	12.7397766	0.34354797	0	-1.7504256	2.03134389	-1.41519	2.54480761	-1.065522	-6.9077553	6	91	1.34111667	297.444444	24.44444444	0	0	0	0	34.0724638	1	0.49277013
0.1872	8149.69088	0.23065831	11.11291	0.28342215	0	-1.6755777	2.09810269	-1.4624918	2.40819747	-1.2572957	-6.9077553	7	93	1.34111667	297.444444	24.44444444	0	0	0	0	34.0724638	1	0.49277013
0.198	8544.46523	0.21301009	9.03127988	0.2344145	0	-1.6194882	2.14540076	-1.5417321	2.20080481	-1.4464075	-6.9077553	8	93	1.78815556	297.444444	24.44444444	0	0	0	0	34.0724638	1	0.49277013
0.207	8895.37572	0.19985569	8.07402649	0.24153397	0	-1.5750365	2.18564397	-1.6051686	2.08877615	-1.4166135	-6.9077553	9	93	2.23519444	297.444444	24.44444444	0	0	0	0	34.0724638	1	0.49277013
0.2178	9334.01402	0.18820331	7.63809903	0.24537057	0	-1.5241781	2.23377228	-1.6649331	2.03327967	-1.4009185	-6.9077553	10	94	1.34111667	297.444444	24.44444444	0	0	0	0	34.0724638	1	0.49277013
0.2286	9772.65157	0.20663917	8.82377942	0.24942501	0	-1.4757815	2.27969015	-1.5719535	2.17756361	-1.3845957	-6.9077553	11	92	1.34111667	298	25	0	0	0	0	34.0724638	1	0.49277013
0.2412	10299.0174	0.34332149	32.8940386	0.48979473	0	-1.4221288	2.33214558	-1.0661795	3.49332184	-0.7117293	-6.9077553	12	88	2.68223333	299.111111	26.11111111	0	0	0	0	34.0724638	1	0.49277013
0.3294	12876.179	0.6796506	136.58531	1.73499407	0	-1.1104825	2.55545668	-0.3847062	4.91695672	0.5515802	-6.9077553	13	83	3.57631111	300.222222	27.2222222	0	0	0	1	34.0724638	1	0.49277013
0.4491	18199.7468	0.8891157	227.050253	2.01752673	0	-0.8005097	2.90146263	-0.1164038	5.42517577	0.7023679	-6.9077553	14	73	3.57631111	302.444444	29.44444444	0	0	0	1	34.0724638	1	0.49277013
0.6885	30539.6827	1.09312129	325.609874	2.74055171	0	-0.37324	3.41905965	0.08995157	5.78570303	1.00852408	-6.9077553	15	63	3.12927222	304.111111	31.11111111	0	0	0	1	34.0724638	1	0.49277013
0.981	42875.5492	1.04199801	300.325264	2.45729286	0	-0.0191828	3.75832504	0.04209927	5.70486943	0.89946715	-6.9077553	16	55	2.68223333	306.333333	33.33333333	0	0	0	1	34.0724638	1	0.49277013
1.2546	49844.1931	0.93945905	249.62207	1.92794539	0	0.2268168	3.90892206	-0.0613872	5.51995206	0.65697342	-6.9077553	17	50	3.12927222	307.444444	34.4444444	0	0	0	1	34.0724638	1	0.49277013
1.629	62568.789	0.96942146	260.260479	2.37648069	0	0.48796633	4.13628256	-0.0300248	5.56168682	0.86604139	-6.9077553	18	47	3.57631111	307.444444	34.4444444	0	0	0	1	34.0724638	1	0.49277013
2.0844	78379.8761	0.93757491	242.327569	1.91672453	0	0.73448104	4.36157997	-0.0633926	5.49029453	0.65113934	-6.9077553	19	44	2.23519444	308.555556	35.5555556	0	0	0	1	34.0724638	1	0.49277013
2.7036	100859.86	0.98800517	270.15139	2.17585541	0	0.99458422	4.61374195	-0.0110557	5.59898621	0.77788137	-6.9077553	20	43	2.23519444	309.111111	36.11111111	0	0	0	1	34.0724638	1	0.49277013
3.2391	117897.126	0.97013134	284.653414	2.44871139	0	1.17529551	4.76982091	-0.0292936	5.65127586	0.89597022	-6.9077553	21	88	5.81150556	296.333333	23.33333333	0	0	0	1	34.0724638	1	0.49277013
3.3768	121406.232	0.63223107	145.141748	1.44443567	0	1.21692852	4.79915045	-0.4569199	4.97771773	0.36841078	-6.9077553	22	90	2.23519444	296.888889	23.88888888	0	0	0	1	34.0724638	1	0.49277013
3.4452	123687.148	0.31752413	31.1605096	0.51546037	0	1.23698196	4.81776346	-1.144057	3.43918367	-0.6607567	-6.9077553	23	91	1.34111667	296.888889	23.88888888	0	0	0	1	34.0724638	1	0.49277013
0.0315	2009.22613	1.14183101	356.056095	2.25830706	0	-3.4577677	0.69824722	0.13350852	5.8750911	0.81505816	-6.9077553	0	62	1.34111667	304.111111	31.11111111	0	0	0	0	42.288408	1	-0.4111751
0.1071	6239.69752	0.97327069	258.000907	1.84912763	0	-2.2339923	1.83109196	-0.0260661	5.55296698	0.61525463	-6.9077553	1	71	1.78815556	301.333333	28.33333333	0	0	0	0	42.288408	1	-0.4111751
0.1854	10057.1137	0.8166475	176.134889	1.54784525	0	-1.6852396	2.30837964	-0.201324	5.17125579	0.43750965	-6.9077553	2	75	1.78815556	300.222222	27.2222222	0	0	0	0	42.288408	1	-0.4111751
0.2484	13083.7144	0.58366514	84.6131696	1.12246813	0	-1.3927149	2.57144471	-0.536716	4.43810174	0.11642045	-6.9077553	3	82	1.34111667	299.111111	26.11111111	0	0	0	0	42.288408	1	-0.4111751
0.2862	14750.5387	0.43743379	45.0969413	0.81314741	0	-1.2510644	2.6913474	-0.8245465	3.8088366	-0.2056138	-6.9077553	4	89	1.34111667	298	25	0	0	0	0	42.288408	1	-0.4111751
0.3267	16680.5454	0.32058135	23.3176866	0.58796671	0	-1.118713	2.81430304	-1.1345047	3.14925504	-0.5293856	-6.9077553	5	90	1.34111667	298	25	0	0	0	0	42.288408	1	-0.4111751
0.3627	18347.3691	0.30824971	20.8379608	0.57454662	0	-1.0141792	2.90954069	-1.1736062	3.03682435	-0.552435	-6.9077553	6	91	1.34111667	297.444444	24.44444444	0	0	0	0	42.288408	1	-0.4111751
0.3888	19531.6897	0.25700043	15.1647844	0.45515902	0	-0.9446902	2.97208946	-1.354794	2.71904186	-0.7849138	-6.9077553	7	93	1.34111667	297.444444	24.44444444	0	0	0	0	42.288408	1	-0.4111751
0.4212	21023.0606	0.26182244	14.6806861	0.40555803	0	-0.8646475	3.04566753	-1.3362766	2.68660087	-0.9000286	-6.9077553	8	93	1.78815556	297.444444	24.44444444	0	0	0	0	42.288408	1	-0.4111751
0.4563	22733.7477	0.23171447	11.6693945	0.36988601	0	-0.7846048	3.12389449	-1.457943	2.45705525	-0.9918605	-6.9077553	9	93	2.23519444	297.444444	24.44444444	0	0	0	0	42.288408	1	-0.4111751
0.4896	24268.9787	0.25315496	13.6766341	0.38682981	0	-0.7141665	3.18924014	-1.3698111	2.61576195	-0.9471887	-6.9077553	10	94	1.34111667	297.444444	24.44444444	0	0	1	0	42.288408	1	-0.4111751
0.5238	25848.075	0.27383644	15.9981561	0.42242246	0	-0.6466453	3.25227482	-1.2915791	2.77253598	-0.8593849	-6.9077553	11	92	1.34111667	298	25	0	0	1	0	42.288408	1	-0.4111751
0.5769	28348.3641	0.36974661	40.6522196	0.59598987	0	-0.5500863	3.3446046	-0.9922364	3.70507804	-0.5158551	-6.9077553	12	88	2.68223333	299.111111	26.11111111	0	0	1	0	42.288408	1	-0.4111751
0.8217	35395.0738	0.6413618	123.236917	1.66018542	0	-0.1963799	3.56660091	-0.4426036	4.81411677	0.50753146	-6.9077553	13	83	3.57631111	300.222222	27.2222222	0	0	1	0	42.288408	1	-0.4111751
1.1592	46814.896	0.77757983	166.452042	1.66988216	0	0.14773011	3.8462228	-0.2502837	5.11471324	0.51335173	-6.9077553	14	73	3.57631111	302.444444	29.44444444	0	0	1	0	42.288408	1	-0.4111751
1.5048	59392.3499	0.80222877	170.479818	1.48368963	0	0.40866	4.08418227	-0.2191157	5.13862278	0.39520574	-6.9077553	15	63	3.12927222	304.111111	31.11111111	0	0	1	0	42.288408	1	-0.4111751

1.9161	73983.6858	0.85385533	199.923807	2.00166398	0	0.65029187	4.30385812	-0.156823	5.29794133	0.69447828	-6.9077553	16	55	2.68223333	306.333333	33.33333333	0	0	1	0	42.288408	1	-0.4111751
2.3805	90352.6899	0.83898571	194.275375	1.8168012	0	0.86731055	4.50373186	-0.1743704	5.26928176	0.59762764	-6.9077553	17	50	3.12927222	307.444444	34.4444444	0	0	1	0	42.288408	1	-0.4111751
3.0825	116122.877	0.9029433	233.629587	2.46665352	0	1.12574096	4.75465753	-0.1009886	5.45374118	0.90326771	-6.9077553	18	47	3.57631111	307.444444	34.4444444	0	0	1	0	42.288408	1	-0.4111751
3.8133	142114.094	0.84729395	213.403883	1.99612607	0	1.33849496	4.95663725	-0.1645281	5.36319122	0.69170918	-6.9077553	19	44	2.23519444	308.555556	35.5555556	0	0	1	0	42.288408	1	-0.4111751
4.6773	169266.576	0.84831855	216.167057	2.12005603	0	1.54272102	5.13148075	-0.163321	5.37605615	0.75191409	-6.9077553	20	43	2.23519444	309.111111	36.11111111	0	0	1	0	42.288408	1	-0.4111751
5.1822	181870.758	0.7099129	149.442123	2.05135657	0	1.64522968	5.20330181	-0.3412054	5.00691588	0.71898868	-6.9077553	21	88	5.81150556	296.333333	23.33333333	0	0	1	0	42.288408	1	-0.4111751
5.2695	185511.446	0.41444857	48.3719243	0.63591688	0	1.66193548	5.22312197	-0.8783964	3.87894024	-0.4511161	-6.9077553	22	90	2.23519444	296.888889	23.88888888	0	0	1	0	42.288408	1	-0.4111751
5.337	188581.901	0.27355222	16.4006755	0.44145315	0	1.6746637	5.2395377	-1.2926138	2.79738349	-0.8154207	-6.9077553	23	91	1.34111667	296.888889	23.88888888	0	0	1	0	42.288408	1	-0.4111751
0.0036	0	0.18133275	6.37634977	0.52180731	0	-5.6268214	-6.9077553	-1.701922	1.85275261	-0.6485423	-6.9077553	0	62	1.34111667	304.111111	31.11111111	0	0	0	0	18.9135952	1	-0.0428739
0.0036	0	0	0	0	0	-5.6268214	-6.9077553	-6.9077553	-6.9077553	-6.9077553	-6.9077553	1	71	1.78815556	301.333333	28.33333333	0	0	0	0	18.9135952	1	-0.0428739
0.0036	0	0	0	0	0	-5.6268214	-6.9077553	-6.9077553	-6.9077553	-6.9077553	-6.9077553	2	75	1.78815556	300.222222	27.2222222	0	0	0	0	18.9135952	1	-0.0428739
0.0036	0	0	0	0	0	-5.6268214	-6.9077553	-6.9077553	-6.9077553	-6.9077553	-6.9077553	3	82	1.34111667	299.111111	26.11111111	0	0	0	0	18.9135952	1	-0.0428739
0.0036	0	0	0	0	0	-5.6268214	-6.9077553	-6.9077553	-6.9077553	-6.9077553	-6.9077553	4	89	1.34111667	298	25	0	0	0	0	18.9135952	1	-0.0428739
0.0036	0	0	0	0	0	-5.6268214	-6.9077553	-6.9077553	-6.9077553	-6.9077553	-6.9077553	5	90	1.34111667	298	25	0	0	0	0	18.9135952	1	-0.0428739
0.0036	0	0	0	0	0	-5.6268214	-6.9077553	-6.9077553	-6.9077553	-6.9077553	-6.9077553	6	91	1.34111667	297.444444	24.44444444	0	0	0	0	18.9135952	1	-0.0428739
0.0036	0	0	0	0	0	-5.6268214	-6.9077553	-6.9077553	-6.9077553	-6.9077553	-6.9077553	7	93	1.34111667	297.444444	24.44444444	0	0	0	0	18.9135952	1	-0.0428739
0.0036	0	0	0	0	0	-5.6268214	-6.9077553	-6.9077553	-6.9077553	-6.9077553	-6.9077553	8	93	1.78815556	297.444444	24.44444444	0	0	0	0	18.9135952	1	-0.0428739
0.0036	0	0	0	0	0	-5.6268214	-6.9077553	-6.9077553	-6.9077553	-6.9077553	-6.9077553	9	93	2.23519444	297.444444	24.44444444	0	0	0	0	18.9135952	1	-0.0428739
0.0036	0	0	0	0	0	-5.6268214	-6.9077553	-6.9077553	-6.9077553	-6.9077553	-6.9077553	10	94	1.34111667	297.444444	24.44444444	0	0	0	0	18.9135952	1	-0.0428739
0.0036	0	0	0	0	0	-5.6268214	-6.9077553	-6.9077553	-6.9077553	-6.9077553	-6.9077553	11	92	1.34111667	298	25	0	0	0	0	18.9135952	1	-0.0428739
0.0036	0	0	0	0	0	-5.6268214	-6.9077553	-6.9077553	-6.9077553	-6.9077553	-6.9077553	12	88	2.68223333	299.111111	26.11111111	0	0	0	0	18.9135952	1	-0.0428739
0.0036	0	0	0	0	0	-5.6268214	-6.9077553	-6.9077553	-6.9077553	-6.9077553	-6.9077553	13	83	3.57631111	300.222222	27.2222222	0	0	0	0	18.9135952	1	-0.0428739
0.0504	0	0.61398902	105.15718	3.00008334	0	-2.9877641	-6.9077553	-0.4861509	4.65546569	1.09897334	-6.9077553	14	73	3.57631111	302.444444	29.44444444	0	0	0	0	18.9135952	1	-0.0428739
0.1647	0	0.51267375	72.0564561	2.35651596	0	-1.8036296	-6.9077553	-0.666167	4.2774638	0.85760851	-6.9077553	15	63	3.12927222	304.111111	31.11111111	0	0	0	0	18.9135952	1	-0.0428739
0.2826	504.167754	0.46695575	66.935532	1.39892275	0	-1.2637228	-0.6828647	-0.7593815	4.20374489	0.33641706	-6.9077553	16	55	2.68223333	306.333333	33.33333333	0	0	1	0	18.9135952	1	-0.0428739
0.4617	5870.44673	0.76510229	208.178588	1.90204471	0	-0.7728399	1.77010106	-0.2664396	5.33840111	0.64345508	-6.9077553	17	50	3.12927222	307.444444	34.4444444	0	0	1	0	18.9135952	1	-0.0428739
0.6435	11514.5626	0.73927745	188.58586	1.69605791	0	-0.4408333	2.44369939	-0.3007302	5.2395587	0.52889611	-6.9077553	18	47	3.57631111	307.444444	34.4444444	0	0	1	0	18.9135952	1	-0.0428739
0.8163	15347.1668	0.64912216	125.287557	1.55685933	0	-0.2029733	2.73099604	-0.430595	4.83061953	0.44331266	-6.9077553	19	44	2.23519444	308.555556	35.5555556	0	0	1	0	18.9135952	1	-0.0428739
1.026	19847.1989	0.6664969	129.612728	1.43644184	0	0.02566775	2.98811327	-0.4042205	4.8645587	0.36286503	-6.9077553	20	43	2.23519444	309.111111	36.11111111	0	0	1	0	18.9135952	1	-0.0428739
1.2393	24754.0531	0.80297594	245.927083	2.81505678	0	0.2145467	3.20902963	-0.2181859	5.50503915	1.0353376	-6.9077553	21	88	5.81150556	296.333333	23.33333333	0	0	1	0	18.9135952	1	-0.0428739
1.2825	26771.7849	0.41493273	43.4263	0.97120714	0	0.2488113	3.28738588	-0.8772317	3.77108828	-0.0281864	-6.9077553	22	90	2.23519444	296.888889	23.88888888	0	0	1	0	18.9135952	1	-0.0428739
1.3113	28043.8344	0.29319701	19.2545776	0.55972826	0	0.27101901	3.33380446	-1.2235056	2.95780077	-0.5785189	-6.9077553	23	91	1.34111667	296.888889	23.88888888	0	0	1	0	18.9135952	1	-0.0428739
0.0063	223.098442	0.91984145	228.266357	1.51162052	0	-5.0672056	-1.4956698	-0.0824674	5.43051756	0.41384359	-6.9077553	0	85	1.34111667	298.555556	25.55555555	0	0	0	0	30.519848	1	0.21141735
0.0135	530.144985	0.47957906	59.1758766	0.84896135	0	-4.3050656	-0.6327203	-0.7327635	4.08053087	-0.1625644	-6.9077553	1	88	1.34111667	298.555556	25.55555555	0	0	0	0	30.519848	1	0.21141735
0.0279	1012.64661	0.62531263	96.9991837	1.08293819	0	-3.5791286	0.01355434	-0.4679056	4.57471287	0.08060089	-6.9077553	2	92	1.34111667	298	25	0	0	0	0	30.519848	1	0.21141735
0.0378	1144.23791	0.60108033	93.5868919	1.20219864	0	-3.2754462	0.1356124	-0.5073644	4.53890101	0.18498354	-6.9077553	3	95	1.34111667	297.444444	24.44444444	0	0	0	0	30.519848	1	0.21141735
0.0414	1231.96544	0.41982788	41.5153885	0.7634173	0	-3.1844744	0.20942219	-0.8655314	3.72608825	-0.2686414	-6.9077553	4	96	2.23519444	297.444444	24.44444444	0	0	0	0	30.519848	1	0.21141735
0.0423	1275.82923	0.54580736	69.6479034	0.75004858	0	-3.1629682	0.24437984	-0.6036587	4.24346696	-0.2862849	-6.9077553	5	98	1.78815556	297.444444	24.44444444	0	0	0	0	30.519848	1	0.21141735
0.0423	1275.82923	0	0	0	0	-3.1629682	0.24437984	-6.9077553	-6.9077553	-6.9077553	-6.9077553	6	93	3.12927222	298.555556	25.55555555	0	0	0	0	30.519848	1	0.21141735
0.1062	1754.53987	0.37454506	38.0443751	1.60538483	0	-2.2424312	0.56277643	-0.9793768	3.63877953	0.4739862	-6.9077553	7	80	4.02335	300.777778	27.7777778	0	0	0	0	30.519848	1	0.21141735
0.2304	2449.70647	0.53262429	83.7433693	2.26238129	0	-1.4679384	0.89637634	-0.6280633	4.42776894	0.81685984	-6.9077553	8	72	4.47038889	302.444444	29.44444444	0	0	0	0	30.519848	1	0.21141735
0.3249	3556.3012	0.61772926	109.450995	1.95103309	0	-1.1242378	1.26900217	-0.4800875	4.69548605	0.66887144	-6.9077553	9	62	3.57631111	304.111111	31.11111111	0	0	0	0	30.519848	1	0.21141735
0.4428	4489.35153	0.5899607	91.6014134	1.6439916	0	-0.8146371	1.50193099	-0.5260058	4.51745762	0.49773528	-6.9077553	10	55	3.12927222	305.777778	32.7777778	0	0	0	1	30.519848	1	0.21141735
0.6219	5932.78125	0.61125375	98.0305139	1.82728216	0	-0.474976	1.78066166	-0.4906085	4.585289	0.60337682	-6.9077553	11	50	3.57631111	306.888889	33.88888888	0	0	0	1	30.519848	1	0.21141735
0.8523	9661.55611	0.67881346	130.155807	1.78078818	0	-0.1598167	2.26825822	-0.3859368	4.86873994	0.57761746	-6.9077553	12	46	2.68223333	307.444444	34.4444444	0	0	0	1	30.519848	1	0.21141735
1.0917	15670.5494	0.80252001	183.715167	1.93988221	0	0.08773611	2.75184693	-0.2187532	5.213392	0.66314262	-6.9077553	13	41	2.68223333	309.111111	36.11111111	0	0	0	1	30.519848	1	0.21141735
1.35	20447.265	0.78793516	176.262821	1.91409738	0	0.30010459	3.01789804	-0.2370711	5.17198186	0.64976847	-6.9077553	14	38	3.12927222	309.666667	36.66666666	0	0	0	1	30.519848	1	0.21141735
1.6533	26053.9394	0.76179446	159.86881	1.77747361	0	0.50277329	3.26020736	-0.2707667	5.0743598	0.57575547	-6.9077553	15	36	3.12927222	309.111111	36.11111111	0	0	0	1	30.519848	1	0.21141735
1.8999	31764.2054	0.83735646	195.316235	1.67643838	0	0.64180125	3.45837152	-0.1763119	5.27462508	0.51726786	-6.9077553	16	38	3.12927222	308.555556	35.5555556	0	0	0	1	30.519848	1	0.21141735
2.1231	37629.3162	0.82960369	190.182523	1.53745097	0	0.75287728	3.62781001	-0.1856025	5.24798951	0.43077605	-6.9077553	17	42	1.34111667	308	35	0	0	0	1	30.519848	1	0.21141735
2.3049	42677.8107	0.74742769	162.59083	1.40857221	0	0.83503729	3.75370256	-0.2897807	5.09124295	0.34328626	-6.9077553	18	54	1.34111667	304.666667	31.66666666	0	0	0	1	30.519848	1	0.21141735
2.4138	46015.4547	0.78844862	184.115832	1.30814759	0	0.88120227	3.82899904	-0.2364205	5.21557051	0.26937623	-6.9077553	19	61	0.89407778	303	30	0	0	0	1	30.519848	1	0.21141735
2.4615	47461.7112	0.81926288	183.577425	1.31049217	0	0.90077092	3.85994438	-0.1981304	5.21264196	0.27116555	-6.9077553	20	66	1.34111667	301.888889	28.88888888	0	0	0	1	30.519848	1	0.21141735
2.4813	47787.2878	0.6457129	111.251653	0.81171296	0	0.90878262	3.86678058	-0.4358528	4.71180377	-0.2073773	-6.9077553	21	66	1.34111667	301.333333	28.33333333	0	0	0	1	30.519848	1	0.21141735
2.5002	48175.6997	0.72674869	154.360689	1.2660052	0	0.91637073	3.8748755	-0.3177995	5.03929848	0.236656	-6.9077553	22	69	1.34111667	300.777778	27.7777778	0	0	0	1	30.519848	1	0.21141735
2.5065	48228.2471	0.53079176	67.398904	0.46007875	0	0.91888736	3.87596562	-0.6315033	4.21064359	-0.7741864	-6.9077553	23	72	1.78815556	299.666667	26.66666666	0	0	0	1	30.519848	1	0.21141735
0.0135	613.265092	0.99068437	270.562587	1.7887828	0	-4.3050656	-0.4873287	-0.0083504	5.60050714	0.58209427	-6.9077553	0	85	1.34111667	298.555556	25.55555555	0	0	0	0	34.0724638	1	0.49277013
0.0252	964.17537	0.54417229	69.7142193	0.75489565	0	-3.6809113	-0.0354455	-0.6066534	4.24441865	-0.2798519	-6.9077553	1	88	1.34111667	298.555556	25.55555555	0	0	0	0	34.0724638	1	0.49277013
0.0378	1446.67694	0.5115052	66.6936515	0.79582541	0	-3.2754462	0.36996016	-0.6684444	4.20012476	-0.2271197	-6.9077553	2	92	1.34111667	298	25	0	0	0	0	34.0724638	1	0.49277013
0.0504	1973.04225	0.54511244	71.8411298	0.79495375	0	-2.9877641	0.68008334	-0.6049304	4.27447107	-0.2282142	-6.9077553	3	95	1.34111667	297.444444	24.44444444	0	0	0	0	34.0724638	1	0.49277013
0.0693	2806.45417	0.59504709	86.7081124	1.00007139	0	-2.6693104	1.03227808	-0.5174356	4.46255898	0.00107082	-6.9077553	4	96	2.23519444	297.444444	24.44444444	0	0	0	0	34.0724638	1	0.49277013
0.0882	3683.73006	0.56635598	78.2814825	0.81006114	0	-2.4281483	1.30419727	-0.5667683	4.36032386	-0.2094118	-6.9077553	5	98	1.78815556	297.444444	24.44444444	0	0	0	0	34.0724638	1	0.49277013

0.117	5043.50715	0.61407185	92.2731171	0.92494166	0	-2.1455813	1.61829996	-0.4860162	4.52476368	-0.076944	-6.9077553	6	93	3.12927222	298.555556	25.55555555	0	0	0	0	34.0724638	1	0.49277013
0.1602	7164.60482	0.72578239	138.911092	1.16815575	0	-1.8313322	1.96929247	-0.3191282	4.9338413	0.15628191	-6.9077553	7	80	4.02335	300.777778	27.7777778	0	0	0	0	34.0724638	1	0.49277013
0.306	13504.3015	0.96091179	259.75598	2.53573119	0	-1.1841702	2.60308231	-0.0388325	5.5597465	0.93087632	-6.9077553	8	72	4.47038889	302.444444	29.44444444	0	0	0	0	34.0724638	1	0.49277013
0.5256	24145.4386	1.08972331	333.973297	2.9112874	0	-0.6432148	3.1841369	0.08684106	5.81106403	1.06893882	-6.9077553	9	62	3.57631111	304.111111	31.11111111	0	0	0	1	34.0724638	1	0.49277013
0.8424	39128.2319	1.07972648	323.76092	2.61370104	0	-0.1715003	3.66686981	0.07763348	5.78000843	0.96114977	-6.9077553	10	55	3.12927222	305.777778	32.7777778	0	0	0	1	34.0724638	1	0.49277013
1.1817	49137.8084	1.07240712	329.587015	2.48327281	0	0.16695408	3.89464912	0.07083782	5.79784344	0.90997998	-6.9077553	11	50	3.57631111	306.888889	33.88888888	0	0	0	1	34.0724638	1	0.49277013
1.5264	58808.9055	0.94727535	249.087875	1.94106044	0	0.42291202	4.0743103	-0.0531104	5.51780976	0.66374949	-6.9077553	12	46	2.68223333	307.444444	34.4444444	0	0	0	1	34.0724638	1	0.49277013
1.962	71785.7048	1.01012717	285.135395	2.21383062	0	0.67396436	4.27369929	0.01106572	5.65296765	0.79517593	-6.9077553	13	41	2.68223333	309.111111	36.11111111	0	0	0	1	34.0724638	1	0.49277013
2.5974	95485.9081	1.0952477	341.761066	2.60495644	0	0.95451094	4.55898915	0.09189316	5.83411478	0.95779976	-6.9077553	14	38	3.12927222	309.666667	36.66666666	0	0	0	1	34.0724638	1	0.49277013
3.2598	114647.124	1.0527843	318.478261	2.51275718	0	1.18166584	4.74186764	0.05238778	5.76355736	0.92177852	-6.9077553	15	36	3.12927222	309.111111	36.11111111	0	0	0	1	34.0724638	1	0.49277013
3.8484	131647.991	0.97211757	267.823633	2.04487409	0	1.34765748	4.88013922	-0.0272504	5.59033241	0.71582513	-6.9077553	16	38	3.12927222	308.555556	35.5555556	0	0	0	1	34.0724638	1	0.49277013
4.2939	145845.946	0.88388567	214.290215	1.62053149	0	1.45719541	4.98255775	-0.1222968	5.36733591	0.48337106	-6.9077553	17	42	1.34111667	308	35	0	0	0	1	34.0724638	1	0.49277013
4.6989	157168.061	0.77914049	171.020313	1.5094873	0	1.54732844	5.05732205	-0.2482813	5.14178818	0.41243232	-6.9077553	18	54	1.34111667	304.666667	31.66666666	0	0	0	1	34.0724638	1	0.49277013
4.9833	168053.158	0.79092687	176.722743	1.35160374	0	1.60609232	5.12428629	-0.2332862	5.17458774	0.30203143	-6.9077553	19	61	0.89407778	303	30	0	0	0	1	34.0724638	1	0.49277013
5.2029	178292.611	0.85369523	196.471696	1.44946727	0	1.64921616	5.18343169	-0.1570103	5.28052347	0.37188576	-6.9077553	20	66	1.34111667	301.888889	28.88888888	0	0	0	1	34.0724638	1	0.49277013
5.3847	186642.071	0.71875446	133.156385	1.05736338	0	1.6835616	5.22919808	-0.3288452	4.89153178	0.05672373	-6.9077553	21	66	1.34111667	301.333333	28.33333333	0	0	0	1	34.0724638	1	0.49277013
5.571	194706.19	0.74307664	143.802961	1.13548109	0	1.71757457	5.27149684	-0.2956112	4.96845099	0.12793673	-6.9077553	22	69	1.34111667	300.777778	27.7777778	0	0	0	1	34.0724638	1	0.49277013
5.7906	204093.814	0.77682232	160.303759	1.3236967	0	1.75623591	5.31858466	-0.2512572	5.07707675	0.28118353	-6.9077553	23	72	1.78815556	299.666667	26.66666666	0	0	0	1	34.0724638	1	0.49277013
0.027	1535.51197	1.04791279	296.558223	2.02634667	0	-3.6119184	0.42951489	0.04775419	5.69224694	0.70672788	-6.9077553	0	85	1.34111667	298.555556	25.55555555	0	0	0	0	42.288408	1	-0.4111751
0.0711	3689.32003	0.71079511	129.847856	1.26582578	0	-2.6436679	1.30571318	-0.3399652	4.86637112	0.23651438	-6.9077553	1	88	1.34111667	298.555556	25.55555555	0	0	0	0	42.288408	1	-0.4111751
0.1287	6408.87486	0.64110494	103.624924	1.09186125	0	-2.0502712	1.85783975	-0.4430035	4.64078753	0.08879926	-6.9077553	2	92	1.34111667	298	25	0	0	0	0	42.288408	1	-0.4111751
0.2007	9874.11451	0.67288346	114.31991	1.21772757	0	-1.605944	2.29001791	-0.3946981	4.73900949	0.19780734	-6.9077553	3	95	1.34111667	297.444444	24.44444444	0	0	0	0	42.288408	1	-0.4111751
0.2763	13470.9446	0.68332372	117.187709	1.27217184	0	-1.286268	2.60060935	-0.3793242	4.76378554	0.2415113	-6.9077553	4	96	2.23519444	297.444444	24.44444444	0	0	0	0	42.288408	1	-0.4111751
0.3609	17418.6857	0.6775031	117.990775	1.30843889	0	-1.0191544	2.85760093	-0.3878662	4.77061492	0.26959872	-6.9077553	5	98	1.78815556	297.444444	24.44444444	0	0	0	0	42.288408	1	-0.4111751
0.4482	21367.3173	0.62481768	102.969928	1.1335955	0	-0.8025157	3.06190933	-0.4686962	4.6344467	0.1262762	-6.9077553	6	93	3.12927222	298.555556	25.55555555	0	0	0	0	42.288408	1	-0.4111751
0.6597	28681.806	0.75030725	161.369215	2.04543847	0	-0.4159701	3.35629785	-0.2859406	5.0837012	0.71610095	-6.9077553	7	80	4.02335	300.777778	27.7777778	0	0	1	0	42.288408	1	-0.4111751
1.0314	41447.6051	0.84648845	198.283898	2.15293652	0	0.0309171	3.72445423	-0.1654781	5.28970488	0.76729711	-6.9077553	8	72	4.47038889	302.444444	29.44444444	0	0	1	0	42.288408	1	-0.4111751
1.368	54582.5375	0.85110482	189.685223	1.56615166	0	0.31334982	3.99973233	-0.1600457	5.24537125	0.44925975	-6.9077553	9	62	3.57631111	304.111111	31.11111111	0	0	1	0	42.288408	1	-0.4111751
1.7622	70637.063	0.86430923	201.904484	1.53551661	0	0.56656303	4.25756913	-0.1446683	5.30779969	0.42951791	-6.9077553	10	55	3.12927222	305.777778	32.7777778	0	0	1	0	42.288408	1	-0.4111751
2.3193	91250.6159	0.9251705	241.902565	2.20819437	0	0.84126542	4.5136207	-0.0766969	5.48853915	0.79262791	-6.9077553	11	50	3.57631111	306.888889	33.88888888	0	0	1	0	42.288408	1	-0.4111751
2.952	115430.274	0.8945408	227.638313	2.05683858	0	1.08248291	4.74867532	-0.1103275	5.42776242	0.7216562	-6.9077553	12	46	2.68223333	307.444444	34.4444444	0	0	1	0	42.288408	1	-0.4111751
3.7053	148562.978	0.99002305	281.13215	2.35424353	0	1.30976423	5.00101569	-0.0090175	5.6388284	0.85664413	-6.9077553	13	41	2.68223333	309.111111	36.11111111	0	0	1	0	42.288408	1	-0.4111751
4.4766	181706.412	0.98461886	282.925649	2.23660332	0	1.49886383	5.20239777	-0.0144856	5.64518767	0.80540535	-6.9077553	14	38	3.12927222	309.666667	36.66666666	0	0	1	0	42.288408	1	-0.4111751
5.2191	214462.304	0.98120975	279.948049	2.19816909	0	1.65232497	5.36813864	-0.0179504	5.63460762	0.7880796	-6.9077553	15	36	3.12927222	309.111111	36.11111111	0	0	1	0	42.288408	1	-0.4111751
5.8635	237390.631	0.85227174	226.847765	1.89275748	0	1.76874669	5.46971123	-0.1586772	5.42428356	0.63856294	-6.9077553	16	38	3.12927222	308.555556	35.5555556	0	0	1	0	42.288408	1	-0.4111751
6.3342	247690.749	0.62355179	123.263549	1.35934337	0	1.84596352	5.51218503	-0.470721	4.81433285	0.30773715	-6.9077553	17	42	1.34111667	308	35	0	0	1	0	42.288408	1	-0.4111751
6.6987	256477.616	0.58931418	112.792978	1.12245054	0	1.90191348	5.54704529	-0.5271004	4.72556295	0.11640479	-6.9077553	18	54	1.34111667	304.666667	31.66666666	0	0	1	0	42.288408	1	-0.4111751
6.9102	264249.836	0.72694105	156.995263	1.14604077	0	1.93299858	5.57689879	-0.3175352	5.056222	0.13718538	-6.9077553	19	61	0.89407778	303	30	0	0	1	0	42.288408	1	-0.4111751
7.0434	269974.325	0.79380489	167.034615	1.21067181	0	1.95209101	5.59833057	-0.2296586	5.11820705	0.19200107	-6.9077553	20	66	1.34111667	301.888889	28.88888888	0	0	1	0	42.288408	1	-0.4111751
7.1775	275833.984	0.76029856	153.045656	1.15170196	0	1.97095113	5.6198028	-0.2727297	5.03074282	0.14210872	-6.9077553	21	66	1.34111667	301.333333	28.33333333	0	0	1	0	42.288408	1	-0.4111751
7.3008	281216.268	0.77258223	155.441748	1.21375325	0	1.98798393	5.63912757	-0.2567233	5.04627749	0.19454097	-6.9077553	22	69	1.34111667	300.777778	27.7777778	0	0	1	0	42.288408	1	-0.4111751
7.3935	285143.563	0.7117671	131.765815	1.1084331	0	2.00060124	5.65299629	-0.3386006	4.8810338	0.10384917	-6.9077553	23	72	1.78815556	299.666667	26.66666666	0	0	1	0	42.288408	1	-0.4111751
0.0009	0	0	0	0	0	-7.0131158	-6.9077553	-6.9077553	-6.9077553	-6.9077553	-6.9077553	0	85	1.34111667	298.555556	25.55555555	0	0	0	0	18.9135952	1	-0.0428739
0.0009	0	0	0	0	0	-7.0131158	-6.9077553	-6.9077553	-6.9077553	-6.9077553	-6.9077553	1	88	1.34111667	298.555556	25.55555555	0	0	0	0	18.9135952	1	-0.0428739
0.0009	0	0	0	0	0	-7.0131158	-6.9077553	-6.9077553	-6.9077553	-6.9077553	-6.9077553	2	92	1.34111667	298	25	0	0	0	0	18.9135952	1	-0.0428739
0.0009	0	0	0	0	0	-7.0131158	-6.9077553	-6.9077553	-6.9077553	-6.9077553	-6.9077553	3	95	1.34111667	297.444444	24.44444444	0	0	0	0	18.9135952	1	-0.0428739
0.0009	0	0	0	0	0	-7.0131158	-6.9077553	-6.9077553	-6.9077553	-6.9077553	-6.9077553	4	96	2.23519444	297.444444	24.44444444	0	0	0	0	18.9135952	1	-0.0428739
0.0009	0	0	0	0	0	-7.0131158	-6.9077553	-6.9077553	-6.9077553	-6.9077553	-6.9077553	5	98	1.78815556	297.444444	24.44444444	0	0	0	0	18.9135952	1	-0.0428739
0.0009	0	0	0	0	0	-7.0131158	-6.9077553	-6.9077553	-6.9077553	-6.9077553	-6.9077553	6	93	3.12927222	298.555556	25.55555555	0	0	0	0	18.9135952	1	-0.0428739
0.0009	0	0	0	0	0	-7.0131158	-6.9077553	-6.9077553	-6.9077553	-6.9077553	-6.9077553	7	80	4.02335	300.777778	27.7777778	0	0	0	0	18.9135952	1	-0.0428739
0.0333	0	0.55429681	86.8930393	2.53492567	0	-3.4021979	-6.9077553	-0.5882525	4.46468944	0.93055873	-6.9077553	8	72	4.47038889	302.444444	29.44444444	0	0	0	0	18.9135952	1	-0.0428739
0.1449	272.401006	0.58022228	90.6641255	2.27185502	0	-1.9317114	-1.2968157	-0.542622	4.50717278	0.82103676	-6.9077553	9	62	3.57631111	304.111111	31.11111111	0	0	0	0	18.9135952	1	-0.0428739
0.3429	3325.12166	0.6864606	155.364684	2.30464699	0	-1.0703164	1.20180696	-0.3747508	5.04578159	0.83536133	-6.9077553	10	55	3.12927222	305.777778	32.7777778	0	0	1	0	18.9135952	1	-0.0428739
0.5355	10988.1513	0.89190674	274.373686	2.27006802	0	-0.6245544	2.39690854	-0.1132731	5.61449464	0.82025021	-6.9077553	11	50	3.57631111	306.888889	33.88888888	0	0	1	0	18.9135952	1	-0.0428739
0.6804	15567.8764	0.75274033	183.865974	1.42194456	0	-0.3850744	2.74527382	-0.2827074	5.21421253	0.35272836	-6.9077553	12	46	2.68223333	307.444444	34.4444444	0	0	1	0	18.9135952	1	-0.0428739
0.8469	19674.8441	0.69640948	151.397804	1.35863285	0	-0.1661727	2.9793917	-0.3603825	5.01991744	0.3072147	-6.9077553	13	41	2.68223333	309.111111	36.11111111	0	0	1	0	18.9135952	1	-0.0428739
1.1214	29601.6436	0.90634095	248.809887	2.28730509	0	0.1145779	3.38786367	-0.097237	5.51669312	0.82781141	-6.9077553	14	38	3.12927222	309.666667	36.66666666	0	0	1	0	18.9135952	1	-0.0428739
1.449	40504.5998	0.90341019	244.914749	2.32323514	0	0.37087366	3.70144023	-0.1004723	5.50091427	0.84339102	-6.9077553	15	36	3.12927222	309.111111	36.11111111	0	0	1	0	18.9135952	1	-0.0428739
1.7424	51249.0765	0.85464609	216.954491	1.77676111	0	0.55526347	3.93671711	-0.1558984	5.37969222	0.57535477	-6.9077553	16	38	3.12927222	308.555556	35.5555556	0	0	1	0	18.9135952	1	-0.0428739
2.0484	61854.92	0.78282058	188.053033	1.52511094	0	0.717059	4.12480781	-0.2435751	5.23672933	0.42272263	-6.9077553	17	42	1.34111667	308	35	0	0	1	0	18.9135952	1	-0.0428739
2.4363	76400.2984	0.82412628	205.085611	1.72876656	0	0.89048049	4.33599969	-0.1922188	5.32343238	0.54798646	-6.9077553	18	54	1.34111667	304.666667	31.66666666	0	0	1	0	18.9135952	1	-0.0428739
2.7225	89841.6895	0.92209064	237.492384	1.67695531	0	1.00155058	4.49806025	-0.0800278	5.47013977	0.51757598	-6.9077553	19	61	0.89407778	303	30	0	0	1	0	18.9135952	1	-0.0428739

2.9295	99625.8968	0.86345142	202.710088	1.5253146	0	1.07483176	4.60143218	-0.1456602	5.31178176	0.42285607	-6.9077553	20	66	1.34111667	301.888889	28.88888888	0	0	1	0	18.9135952	1	-0.0428739
3.1041	108001.166	0.82290327	180.037532	1.37986692	0	1.13272382	4.68215129	-0.1937021	5.1931709	0.32271151	-6.9077553	21	66	1.34111667	301.333333	28.33333333	0	0	1	0	18.9135952	1	-0.0428739
3.267	115546.897	0.87816085	206.608684	1.60487796	0	1.18387213	4.74968514	-0.1287874	5.33083143	0.47367062	-6.9077553	22	69	1.34111667	300.777778	27.7777778	0	0	1	0	18.9135952	1	-0.0428739
3.4533	124176.785	0.87662923	207.134209	1.69908444	0	1.2393303	4.82171429	-0.1305311	5.33337176	0.53067792	-6.9077553	23	72	1.78815556	299.666667	26.66666666	0	0	1	0	18.9135952	1	-0.0428739
0.009	362.53523	0.83758672	189.96445	1.533208	0	-4.7105307	-1.0118791	-0.1760373	5.24684222	0.42801429	-6.9077553	0	88	4.02335	299.111111	26.11111111	0	0	0	0	30.519848	1	0.21141735
0.0135	581.8542	0.45634594	52.5851135	0.78426037	0	-4.3050656	-0.5398182	-0.7823152	3.96245208	-0.2417399	-6.9077553	1	89	3.57631111	298.555556	25.55555555	0	0	0	0	30.519848	1	0.21141735
0.0252	1064.35581	0.6123768	90.6658466	0.9681709	0	-3.6809113	0.06330884	-0.4887759	4.50719176	-0.0313143	-6.9077553	2	90	4.02335	298.555556	25.55555555	0	0	0	0	30.519848	1	0.21141735
0.036	1327.53855	0.59426403	87.7328898	1.0328091	0	-3.3242363	0.2840795	-0.5187502	4.47430825	0.03325013	-6.9077553	3	89	3.57631111	298.555556	25.55555555	0	0	0	0	30.519848	1	0.21141735
0.0639	1634.58479	0.32210042	32.9293489	1.07200081	0	-2.7504359	0.49200041	-1.1297921	3.49439469	0.07045922	-6.9077553	4	84	4.02335	299.666667	26.66666666	0	0	0	0	30.519848	1	0.21141735
0.135	1765.04663	0.42686404	49.5480061	2.02549299	0	-2.0024805	0.56874351	-0.8489498	3.9029622	0.70630671	-6.9077553	5	76	4.91742778	301.333333	28.33333333	0	0	0	0	30.519848	1	0.21141735
0.2718	2669.07936	0.6493072	132.083419	3.24983757	0	-1.3026888	0.98210819	-0.4303104	4.88344126	1.17891268	-6.9077553	6	73	5.36446667	301.888889	28.88888888	0	0	0	0	30.519848	1	0.21141735
0.3465	3540.77411	0.56985023	89.3396967	1.28170767	0	-1.0598725	1.26462776	-0.5606284	4.49245711	0.24897321	-6.9077553	7	63	6.25854444	304.111111	31.11111111	0	0	0	0	30.519848	1	0.21141735
0.5121	4398.79731	0.63851124	108.984301	2.20928408	0	-0.6692354	1.48155847	-0.4470511	4.69121302	0.79312105	-6.9077553	8	58	5.81150556	305.222222	32.2222222	0	0	0	1	30.519848	1	0.21141735
0.72	6816.20001	0.68375796	128.811299	2.16150986	0	-0.3285041	1.91944883	-0.3786898	4.8583563	0.77126952	-6.9077553	9	55	4.47038889	306.333333	33.33333333	0	0	0	1	30.519848	1	0.21141735
0.9909	12399.1903	0.83479086	204.558438	2.6904159	0	-0.0091417	2.51771182	-0.1793769	5.32085858	0.99006741	-6.9077553	10	51	5.36446667	306.888889	33.88888888	0	0	0	1	30.519848	1	0.21141735
1.2348	17016.5418	0.7483998	149.962019	1.83651497	0	0.21090901	2.83424468	-0.2884827	5.01038872	0.6084141	-6.9077553	11	52	4.47038889	307.444444	34.4444444	0	0	0	1	30.519848	1	0.21141735
1.5084	21512.1791	0.78991659	177.123188	1.79512144	0	0.41104949	3.06866573	-0.2345628	5.17685111	0.58562959	-6.9077553	12	50	4.02335	308	35	0	0	0	1	30.519848	1	0.21141735
1.8369	26081.503	0.86754468	248.627033	2.39310972	0	0.60807937	3.26126471	-0.1409362	5.51595794	0.87301144	-6.9077553	13	51	3.57631111	308	35	0	0	0	1	30.519848	1	0.21141735
2.0745	31593.3747	0.72071243	141.268643	1.56154101	0	0.72972016	3.45297909	-0.3261285	4.95067043	0.44631335	-6.9077553	14	51	4.02335	307.444444	34.4444444	0	0	0	1	30.519848	1	0.21141735
2.2266	35896.0165	0.7316244	151.700629	1.13321465	0	0.80047576	3.58065419	-0.3111221	5.02191562	0.12594047	-6.9077553	15	54	2.68223333	306.888889	33.88888888	0	0	0	1	30.519848	1	0.21141735
2.3391	39997.3954	0.77212866	174.227313	1.21527039	0	0.84976624	3.68883934	-0.2573098	5.16036658	0.19578912	-6.9077553	16	61	0.89407778	304.666667	31.66666666	0	0	0	1	30.519848	1	0.21141735
2.4003	42413.9072	0.75961551	154.512394	1.09524502	0	0.87559373	3.74749989	-0.2736273	5.04028078	0.09189073	-6.9077553	17	72	2.68223333	303	30	0	0	0	1	30.519848	1	0.21141735
2.43	43554.3676	0.58923502	85.4710582	0.86700422	0	0.88789126	3.77403295	-0.5272345	4.44818952	-0.1415587	-6.9077553	18	74	3.57631111	301.888889	28.88888888	0	0	0	1	30.519848	1	0.21141735
2.4489	44343.914	0.43678275	44.2575655	0.66756407	0	0.89563894	3.79199802	-0.8260325	3.79004892	-0.402623	-6.9077553	19	78	2.68223333	300.777778	27.7777778	0	0	0	1	30.519848	1	0.21141735
2.4606	44870.2813	0.30591906	20.8952191	0.51073221	0	0.90040522	3.80379797	-1.1811712	3.03956824	-0.6699538	-6.9077553	20	80	2.68223333	300.222222	27.2222222	0	0	0	1	30.519848	1	0.21141735
2.4714	45177.3265	0.2735576	15.6569455	0.38542509	0	0.90478479	3.81061747	-1.2925942	2.75097849	-0.9508172	-6.9077553	21	82	2.68223333	299.666667	26.66666666	0	0	0	1	30.519848	1	0.21141735
2.4795	45484.3717	0.24485159	12.6131761	0.38449118	0	0.90805693	3.81739077	-1.4030272	2.53482127	-0.953237	-6.9077553	22	85	1.78815556	298.555556	25.55555555	0	0	0	1	30.519848	1	0.21141735
2.4885	45703.6918	0.19816	9.72469711	0.38175983	0	0.91168012	3.82220096	-1.6136467	2.27477157	-0.9603476	-6.9077553	23	89	2.23519444	298.555556	25.55555555	0	0	0	1	30.519848	1	0.21141735
0.0171	754.91517	0.98955875	278.500787	1.97465791	0	-4.0686768	-0.2798261	-0.0094861	5.62942447	0.68090146	-6.9077553	0	88	4.02335	299.111111	26.11111111	0	0	0	0	34.0724638	1	0.49277013
0.0324	1325.14445	0.53884607	70.8015711	0.72271605	0	-3.4295969	0.28227582	-0.6164712	4.25989532	-0.3233562	-6.9077553	1	89	3.57631111	298.555556	25.55555555	0	0	0	0	34.0724638	1	0.49277013
0.0513	1895.37338	0.51428032	66.8605957	0.86539486	0	-2.9700645	0.63994332	-0.6630442	4.20262475	-0.1434145	-6.9077553	2	90	4.02335	298.555556	25.55555555	0	0	0	0	34.0724638	1	0.49277013
0.0819	3167.42338	0.63763209	101.721421	1.11467575	0	-2.5022563	1.15323411	-0.4484268	4.62224774	0.10946027	-6.9077553	3	89	3.57631111	298.555556	25.55555555	0	0	0	0	34.0724638	1	0.49277013
0.1278	5229.02083	0.67388759	114.459549	1.04731077	0	-2.0572887	1.65441526	-0.3932091	4.74023022	0.04718008	-6.9077553	4	84	4.02335	299.666667	26.66666666	0	0	0	0	34.0724638	1	0.49277013
0.189	7999.52084	0.82033898	174.747386	1.49443155	0	-1.6660083	2.07950665	-0.1968194	5.16334715	0.40241483	-6.9077553	5	76	4.91742778	301.333333	28.33333333	0	0	0	0	34.0724638	1	0.49277013
0.3213	13104.6141	0.97740495	266.206314	2.75819221	0	-1.13538	2.5730407	-0.0218316	5.58427538	1.01493796	-6.9077553	6	73	5.36446667	301.888889	28.88888888	0	0	0	1	34.0724638	1	0.49277013
0.5742	28021.0436	1.23325226	436.929743	3.5938182	0	-0.5547775	3.33299147	0.21046533	6.0797747	1.27949342	-6.9077553	7	63	6.25854444	304.111111	31.11111111	0	0	0	1	34.0724638	1	0.49277013
1.0584	49111.9892	1.31511839	506.906482	4.26256644	2	0.05675833	3.89412355	0.27468679	6.22832851	1.450106	0.69364706	8	58	5.81150556	305.222222	32.2222222	0	0	0	1	34.0724638	1	0.49277013
1.5723	61824.6017	1.08604845	335.787325	2.94324586	2	0.45253952	4.12431755	0.08346618	5.81648098	1.07985271	0.69364706	9	55	4.47038889	306.333333	33.33333333	0	0	0	1	34.0724638	1	0.49277013
2.2122	82149.1762	1.18165608	411.072961	3.35057426	2	0.7939875	4.40854899	0.16776282	6.01877315	1.20943016	0.69364706	10	51	5.36446667	306.888889	33.88888888	0	0	0	1	34.0724638	1	0.49277013
2.8854	105285.191	1.08320381	336.310599	2.84703284	2	1.05966354	4.65668227	0.0808459	5.81803811	1.04662853	0.69364706	11	52	4.47038889	307.444444	34.4444444	0	0	0	1	34.0724638	1	0.49277013
3.3912	121811.378	0.94154244	246.884953	2.02882467	2	1.22118384	4.80248198	-0.0591743	5.5089265	0.70794942	0.69364706	12	50	4.02335	308	35	0	0	0	1	34.0724638	1	0.49277013
3.9051	138341.865	0.92261843	234.422477	1.96388429	2	1.36228339	4.92973513	-0.0794562	5.45712921	0.67543336	0.69364706	13	51	3.57631111	308	35	0	0	0	1	34.0724638	1	0.49277013
4.4037	151894.79	0.81873016	189.543716	1.8775811	2	1.4824451	5.02319469	-0.1987801	5.24462497	0.63051676	0.69364706	14	51	4.02335	307.444444	34.4444444	0	0	0	1	34.0724638	1	0.49277013
4.7817	161982.088	0.72674393	151.634496	1.46405451	2	1.56479613	5.08749194	-0.317806	5.02147959	0.38189245	0.69364706	15	54	2.68223333	306.888889	33.88888888	0	0	0	1	34.0724638	1	0.49277013
5.0157	170523.47	0.7544396	156.242638	1.14804077	2	1.61257299	5.1388788	-0.2804554	5.05141657	0.13892748	0.69364706	16	61	0.89407778	304.666667	31.66666666	0	0	0	1	34.0724638	1	0.49277013
5.1957	176405.536	0.72180252	138.824658	1.10936386	2	1.64783136	5.17279119	-0.3246192	4.93321889	0.10468776	0.69364706	17	72	2.68223333	303	30	0	0	0	1	34.0724638	1	0.49277013
5.3127	181055.095	0.55105074	80.3904447	0.90175723	2	1.67010018	5.1988069	-0.5941153	4.38690776	-0.1023016	0.69364706	18	74	3.57631111	301.888889	28.88888888	0	0	0	1	34.0724638	1	0.49277013
5.3901	184125.571	0.35404141	32.0259499	0.68487819	2	1.68456394	5.21562341	-1.0355208	3.46657773	-0.3770552	0.69364706	19	78	2.68223333	300.777778	27.7777778	0	0	0	1	34.0724638	1	0.49277013
5.4513	186450.331	0.33713992	34.3128626	0.52133465	2	1.69585411	5.22817024	-1.0842955	3.53554943	-0.6494468	0.69364706	20	80	2.68223333	300.222222	27.2222222	0	0	0	1	34.0724638	1	0.49277013
5.5026	188643.536	0.27296699	18.9495764	0.44298654	2	1.70522071	5.23986448	-1.2947477	2.94183435	-0.811961	0.69364706	21	82	2.68223333	299.666667	26.66666666	0	0	0	1	34.0724638	1	0.49277013
5.5584	191143.765	0.22712385	10.9952875	0.30747731	2	1.7153103	5.25303107	-1.4778666	2.39755772	-1.176107	0.69364706	22	85	1.78815556	298.555556	25.55555555	0	0	0	1	34.0724638	1	0.49277013
5.6034	192942.174	0.21941431	11.5207471	0.40841331	2	1.72337356	5.26239571	-1.5122463	2.4442363	-0.8930301	0.69364706	23	89	2.23519444	298.555556	25.55555555	0	0	0	1	34.0724638	1	0.49277013
0.0342	2199.8633	1.13091577	353.980654	2.43670263	0	-3.3755296	0.78884969	0.12391157	5.86924509	0.89105605	-6.9077553	0	88	4.02335	299.111111	26.11111111	0	0	0	0	42.288408	1	-0.4111751
0.0819	4538.02495	0.72551972	138.080161	1.40082507	0	-2.5022563	1.51271222	-0.3194897	4.92784164	0.33777501	-6.9077553	1	89	3.57631111	298.555556	25.55555555	0	0	0	0	42.288408	1	-0.4111751
0.1404	7301.62886	0.66693755	115.187064	1.24196777	0	-1.9632598	1.9882344	-0.4035606	4.74656613	0.21750189	-6.9077553	2	90	4.02335	298.555556	25.55555555	0	0	0	0	42.288408	1	-0.4111751
0.1998	10108.9278	0.67403828	116.699248	1.21139365	0	-1.6104384	2.31351789	-0.3929859	4.75960867	0.19259662	-6.9077553	3	89	3.57631111	298.555556	25.55555555	0	0	0	0	42.288408	1	-0.4111751
0.2916	14587.9315	0.84517363	196.289101	1.6289987	0	-1.2323723	2.68026313	-0.1670307	5.27959367	0.48857922	-6.9077553	4	84	4.02335	299.666667	26.66666666	0	0	0	0	42.288408	1	-0.4111751
0.5742	25332.6914	0.89906204	226.617163	2.55602911	0	-0.5547775	3.23213518	-0.1052916	5.4232665	0.93884608	-6.9077553	5	76	4.91742778	301.333333	28.33333333	0	0	0	0	42.288408	1	-0.4111751
0.9144	37406.8272	0.88451212	211.802042	2.33567011	0	-0.0894872	3.62187996	-0.1215891	5.3556568	0.84872689	-6.9077553	6	73	5.36446667	301.888889	28.88888888	0	0	1	0	42.288408	1	-0.4111751
1.3086	53145.9511	0.89488701	216.354613	2.1331174	0	0.26895786	3.97306074	-0.109941	5.37692341	0.75805316	-6.9077553	7	63	6.25854444	304.111111	31.11111111	0	0	1	0	42.288408	1	-0.4111751
1.9044	74489.0623	0.94426153	254.910404	2.90370552	0	0.644167	4.31066572	-0.0562936	5.54091605	1.06633201	-6.9077553	8	58	5.81150556	305.222222	32.2222222	0	0	1	0	42.288408	1	-0.4111751
2.5596	103567.149	0.97444304	265.22345	2.27566528	0	0.939851	4.64022984	-0.0248635	5.58057645	0.82271178	-6.9077553	9	55	4.47038889	306.333333	33.33333333	0	0	1	0	42.288408	1	-0.4111751

3.2148	134374.231	0.9924464	279.981628	2.13985871	0	1.16776515	4.90063612	-0.0065752	5.63472756	0.76120701	-6.9077553	10	51	5.36446667	306.888889	33.88888888	0	0	1	0	42.288408	1	-0.4111751
4.0446	172640.584	1.05701187	332.677671	2.77032325	0	1.39738266	5.15121768	0.05639155	5.80717707	1.01932491	-6.9077553	11	52	4.47038889	307.444444	34.4444444	0	0	1	0	42.288408	1	-0.4111751
4.6935	196597.727	0.90271639	235.589112	2.29043152	0	1.54617857	5.28116473	-0.1012397	5.46209348	0.82917674	-6.9077553	12	50	4.02335	308	35	0	0	1	0	42.288408	1	-0.4111751
5.2785	217304.312	0.82460834	194.169002	1.9157666	0	1.66364197	5.38130333	-0.1916348	5.26873408	0.6506397	-6.9077553	13	51	3.57631111	308	35	0	0	1	0	42.288408	1	-0.4111751
5.7555	233645.695	0.77052324	169.128744	1.51829304	0	1.75015592	5.45381012	-0.2593885	5.13066614	0.41824512	-6.9077553	14	51	4.02335	307.444444	34.4444444	0	0	1	0	42.288408	1	-0.4111751
6.1479	247607.027	0.78342795	183.06655	1.41791625	0	1.81611056	5.51184696	-0.2428006	5.20985521	0.34989338	-6.9077553	15	54	2.68223333	306.888889	33.88888888	0	0	1	0	42.288408	1	-0.4111751
6.4251	258566.621	0.81524282	195.201819	1.40882755	0	1.86021219	5.55515725	-0.2030434	5.27403911	0.34346739	-6.9077553	16	61	0.89407778	304.666667	31.66666666	0	0	1	0	42.288408	1	-0.4111751
6.5565	264299.519	0.75183586	157.103984	1.2340623	0	1.88045692	5.57708679	-0.2839081	5.05691427	0.21112141	-6.9077553	17	72	2.68223333	303	30	0	0	1	0	42.288408	1	-0.4111751
6.6474	268159.549	0.58487399	92.8432182	0.94029349	0	1.8942258	5.59158587	-0.5346505	4.53092302	-0.0605003	-6.9077553	18	74	3.57631111	301.888889	28.88888888	0	0	1	0	42.288408	1	-0.4111751
6.7167	270879.094	0.39500469	42.7411158	0.64838419	0	1.90459696	5.60167627	-0.9263292	3.75518475	-0.4317308	-6.9077553	19	78	2.68223333	300.777778	27.7777778	0	0	1	0	42.288408	1	-0.4111751
6.7608	272809.111	0.25335526	15.69992	0.4043669	0	1.91114123	5.60877599	-1.3690233	2.75371931	-0.9029627	-6.9077553	20	80	2.68223333	300.222222	27.2222222	0	0	1	0	42.288408	1	-0.4111751
6.795	274475.92	0.24011707	12.0458912	0.30716377	0	1.91618705	5.61486718	-1.4224727	2.48880664	-1.1771239	-6.9077553	21	82	2.68223333	299.666667	26.66666666	0	0	1	0	42.288408	1	-0.4111751
6.8256	275835.69	0.22564706	10.6684337	0.29770492	0	1.92068025	5.61980899	-1.4843613	2.36738299	-1.2082991	-6.9077553	22	85	1.78815556	298.555556	25.55555555	0	0	1	0	42.288408	1	-0.4111751
6.8652	277327.068	0.24902784	21.0408214	0.3907798	0	1.92646517	5.62520117	-1.386183	3.04651195	-0.9370553	-6.9077553	23	89	2.23519444	298.555556	25.55555555	0	0	1	0	42.288408	1	-0.4111751
0.0009	0	0	0	0	0	-7.0131158	-6.9077553	-6.9077553	-6.9077553	-6.9077553	-6.9077553	0	88	4.02335	299.111111	26.11111111	0	0	0	0	18.9135952	1	-0.0428739
0.0009	0	0	0	0	0	-7.0131158	-6.9077553	-6.9077553	-6.9077553	-6.9077553	-6.9077553	1	89	3.57631111	298.555556	25.55555555	0	0	0	0	18.9135952	1	-0.0428739
0.0009	0	0	0	0	0	-7.0131158	-6.9077553	-6.9077553	-6.9077553	-6.9077553	-6.9077553	2	90	4.02335	298.555556	25.55555555	0	0	0	0	18.9135952	1	-0.0428739
0.0009	0	0	0	0	0	-7.0131158	-6.9077553	-6.9077553	-6.9077553	-6.9077553	-6.9077553	3	89	3.57631111	298.555556	25.55555555	0	0	0	0	18.9135952	1	-0.0428739
0.0009	0	0	0	0	0	-7.0131158	-6.9077553	-6.9077553	-6.9077553	-6.9077553	-6.9077553	4	84	4.02335	299.666667	26.66666666	0	0	0	0	18.9135952	1	-0.0428739
0.0288	0	0.49159511	66.2979776	2.15039259	0	-3.5473799	-6.9077553	-0.7080677	4.19417448	0.76611535	-6.9077553	5	76	4.91742778	301.333333	28.33333333	0	0	0	0	18.9135952	1	-0.0428739
0.1845	2587.98649	0.76291784	186.290835	2.69617193	0	-1.6901058	0.95126648	-0.269295	5.22731445	0.99220379	-6.9077553	6	73	5.36446667	301.888889	28.88888888	0	0	0	0	18.9135952	1	-0.0428739
0.4491	11136.3321	0.89868911	267.353369	3.08312282	0	-0.8005097	2.41030272	-0.105706	5.588575	1.12626728	-6.9077553	7	63	6.25854444	304.111111	31.11111111	0	0	1	0	18.9135952	1	-0.0428739
0.6093	14844.0428	0.72154373	173.45363	1.61601309	0	-0.4954445	2.69766599	-0.3249773	5.15591607	0.48058067	-6.9077553	8	58	5.81150556	305.222222	32.2222222	0	0	1	0	18.9135952	1	-0.0428739
0.8046	19931.7679	0.73328523	165.142515	1.68378614	0	-0.21741	2.99236501	-0.3088577	5.10681488	0.52163863	-6.9077553	9	55	4.47038889	306.333333	33.33333333	0	0	1	0	18.9135952	1	-0.0428739
1.0476	28437.5919	0.83477472	214.261834	1.85624616	0	0.04650183	3.34774709	-0.1793962	5.36720346	0.61909483	-6.9077553	10	51	5.36446667	306.888889	33.88888888	0	0	1	0	18.9135952	1	-0.0428739
1.3698	38064.707	0.86303029	221.319352	2.29995642	0	0.31466474	3.6393138	-0.1461475	5.39961121	0.83332487	-6.9077553	11	52	4.47038889	307.444444	34.4444444	0	0	1	0	18.9135952	1	-0.0428739
1.6569	48054.9337	0.83027137	201.354722	1.71880893	0	0.50494839	3.87236562	-0.184799	5.3050731	0.5422132	-6.9077553	12	50	4.02335	308	35	0	0	1	0	18.9135952	1	-0.0428739
1.9296	57385.39	0.79297016	188.807047	1.56806125	0	0.65731273	4.04980717	-0.2307094	5.24073087	0.45047751	-6.9077553	13	51	3.57631111	308	35	0	0	1	0	18.9135952	1	-0.0428739
2.2581	68618.4673	0.78040771	180.979238	1.53164547	0	0.81452375	4.22857628	-0.2466582	5.19838784	0.42699531	-6.9077553	14	51	4.02335	307.444444	34.4444444	0	0	1	0	18.9135952	1	-0.0428739
2.6082	83013.1941	0.8607082	222.732065	1.73039469	0	0.95866033	4.41901161	-0.1488386	5.40597404	0.54892726	-6.9077553	15	54	2.68223333	306.888889	33.88888888	0	0	1	0	18.9135952	1	-0.0428739
2.8566	94021.2717	0.82280809	192.3781	1.38890474	0	1.04963211	4.54353169	-0.1938177	5.2594679	0.32923521	-6.9077553	16	61	0.89407778	304.666667	31.66666666	0	0	1	0	18.9135952	1	-0.0428739
3.0141	101276.823	0.85599261	197.814018	1.51551723	0	1.10330128	4.61786746	-0.154326	5.28733234	0.41641641	-6.9077553	17	72	2.68223333	303	30	0	0	1	0	18.9135952	1	-0.0428739
3.1122	105799.065	0.63555292	104.967505	1.1087524	0	1.13532987	4.66155114	-0.4516877	4.65366035	0.10413693	-6.9077553	18	74	3.57631111	301.888889	28.88888888	0	0	1	0	18.9135952	1	-0.0428739
3.1662	108123.846	0.39554965	37.913623	0.66408037	0	1.15253213	4.68328654	-0.924954	3.63533687	-0.4078474	-6.9077553	19	78	2.68223333	300.777778	27.7777778	0	0	1	0	18.9135952	1	-0.0428739
3.2004	109659.085	0.26367963	15.0785362	0.41570119	0	1.1632758	4.69738544	-1.3292351	2.71333861	-0.8753859	-6.9077553	20	80	2.68223333	300.222222	27.2222222	0	0	1	0	18.9135952	1	-0.0428739
3.2481	111808.407	0.25615998	14.7036536	0.45449397	0	1.17807021	4.7167957	-1.3580569	2.68816401	-0.7863728	-6.9077553	21	82	2.68223333	299.666667	26.66666666	0	0	1	0	18.9135952	1	-0.0428739
3.2958	113957.731	0.25938407	14.830495	0.44464576	0	1.19264893	4.73583637	-1.3455976	2.69675296	-0.8082309	-6.9077553	22	85	1.78815556	298.555556	25.55555555	0	0	1	0	18.9135952	1	-0.0428739
3.339	115931.599	0.25731989	14.3970006	0.45351116	0	1.20567136	4.75300898	-1.3535566	2.66708935	-0.7885328	-6.9077553	23	89	2.23519444	298.555556	25.55555555	0	0	1	0	18.9135952	1	-0.0428739
0.0117	406.822788	0.911484	225.253092	1.60287031	0	-4.4481664	-0.8969225	-0.0915847	5.41722906	0.47241965	-6.9077553	0	94	1.34111667	276.888889	3.888888888	0	0	0	0	30.519848	1	0.21141735
0.0324	1210.47169	0.69485329	139.955779	1.20835321	0	-3.4295969	0.19183589	-0.3626164	4.94133365	0.19008568	-6.9077553	1	58	1.78815556	284.666667	11.66666666	0	0	0	0	30.519848	1	0.21141735
0.09	1801.26534	0.58396709	108.874077	1.38924062	0	-2.4079456	0.5890444	-0.5361997	4.69020114	0.32947684	-6.9077553	2	36	3.12927222	290.222222	17.2222222	0	0	0	0	30.519848	1	0.21141735
0.18	2840.46859	0.51756073	81.6830273	1.51016037	0	-1.7147984	1.04432103	-0.6566981	4.40285848	0.41287781	-6.9077553	3	31	3.57631111	293.555556	20.55555555	0	0	0	0	30.519848	1	0.21141735
0.4005	4186.27036	0.74414063	162.782207	3.58390715	0	-0.9150415	1.43204906	-0.2941823	5.09241929	1.27673257	-6.9077553	4	22	5.36446667	296.333333	23.33333333	0	0	0	1	30.519848	1	0.21141735
0.675	6337.3827	0.78526171	170.516624	3.13271604	0	-0.3930426	1.84662364	-0.2404656	5.13883866	1.14221953	-6.9077553	5	20	5.81150556	297.444444	24.44444444	0	0	0	1	30.519848	1	0.21141735
1.0197	13068.1442	0.8210795	194.76893	2.59551635	0	0.01950847	2.57025405	-0.1959182	5.27181901	0.95417068	-6.9077553	6	18	4.91742778	298.555556	25.55555555	0	0	0	1	30.519848	1	0.21141735
1.4706	25822.8383	0.84624458	213.515797	2.95713773	0	0.38567048	3.25129803	-0.1657659	5.3637155	1.08455992	-6.9077553	7	16	5.36446667	299.111111	26.11111111	0	0	0	1	30.519848	1	0.21141735
1.9872	45326.943	0.89249295	261.62647	2.75196121	0	0.68672661	3.81392368	-0.1126168	5.56692162	1.01267713	-6.9077553	8	18	5.36446667	298.555556	25.55555555	0	0	0	1	30.519848	1	0.21141735
2.349	70776.1409	0.7967735	178.112484	1.6220527	0	0.85398971	4.25953608	-0.2259306	5.1824209	0.48430876	-6.9077553	9	25	2.68223333	295.222222	22.2222222	0	0	0	1	30.519848	1	0.21141735
2.6379	95867.9387	0.78425047	168.089491	1.35117214	0	0.96998315	4.56298204	-0.2417525	5.12450247	0.30171229	-6.9077553	10	29	1.34111667	293	20	0	0	0	1	30.519848	1	0.21141735
2.8908	121808.09	0.7571573	159.06788	1.1737723	0	1.06153328	4.80245498	-0.2768644	5.06933731	0.16107434	-6.9077553	11	31	1.34111667	290.777778	17.7777778	0	0	0	1	30.519848	1	0.21141735
3.141	147697.459	0.76430989	161.42699	1.43758123	0	1.14454122	4.99517276	-0.2674744	5.08405916	0.36365737	-6.9077553	12	33	2.68223333	290.222222	17.2222222	0	0	0	1	30.519848	1	0.21141735
3.3147	170345.006	0.77687935	170.488787	1.1170867	0	1.19836712	5.1378317	-0.2511839	5.1386754	0.11161892	-6.9077553	13	35	3.12927222	290.222222	17.2222222	0	0	0	1	30.519848	1	0.21141735
3.4605	193235.182	0.69858438	138.063561	1.0529156	0	1.24141309	5.26391318	-0.3572689	4.92772141	0.05251237	-6.9077553	14	36	3.57631111	290.222222	17.2222222	0	0	0	1	30.519848	1	0.21141735
3.6477	220873.022	0.70277126	132.430382	1.46716939	0	1.29409683	5.3975925	-0.3513019	4.88606464	0.38401632	-6.9077553	15	40	3.57631111	289.111111	16.11111111	0	0	0	1	30.519848	1	0.21141735
3.7962	251427.015	0.6861538	127.658902	1.02911728	0	1.33400057	5.52715672	-0.3751971	4.84936971	0.02967266	-6.9077553	16	44	3.57631111	288	15	0	0	0	1	30.519848	1	0.21141735
3.9267	285200.203	0.66823404	121.185062	1.05936942	0	1.36779938	5.65319491	-0.4016214	4.79732707	0.05861736	-6.9077553	17	44	4.91742778	286.888889	13.88888888	0	0	0	1	30.519848	1	0.21141735
4.0338	319913.78	0.64692611	107.821724	0.53113421	0	1.39470886	5.76805465	-0.4339786	4.68048843	-0.6308595	-6.9077553	18	51	4.02335	285.777778	12.77777777	0	0	0	1	30.519848	1	0.21141735
4.1238	351289.569	0.64333839	103.134209	0.4656776	0	1.41677507	5.86161371	-0.4395312	4.63604084	-0.7621166	-6.9077553	19	63	2.23519444	284.666667	11.66666666	0	0	0	1	30.519848	1	0.21141735
4.1994	378993.669	0.59682755	86.3300549	0.41253113	0	1.43494166	5.93752214	-0.5144529	4.45818938	-0.8830225	-6.9077553	20	71	2.23519444	284.666667	11.66666666	0	0	0	1	30.519848	1	0.21141735
4.2705	404695.079	0.53522245	68.3285577	0.36055645	0	1.45173092	6.00313636	-0.6232062	4.22434244	-1.0173371	-6.9077553	21	73	2.68223333	284.666667	11.66666666	0	0	0	1	30.519848	1	0.21141735
4.3245	424388.377	0.51060692	61.3010946	0.3332029	0	1.46429653	6.05065137	-0.6701987	4.11581401	-1.096007	-6.9077553	22	79	3.57631111	284.666667	11.66666666	0	0	0	1	30.519848	1	0.21141735
4.3605	437405.994	0.38894112	34.8464355	0.25813522	0	1.47258673	6.0808641	-0.9417595	3.55097955	-1.3504053	-6.9077553	23	83	4.47038889	286.333333	13.33333333	0	0	0	1	30.519848	1	0.21141735

0.0144	654.148517	0.95934703	255.44235	1.74980799	0	-4.2405271	-0.4228933	-0.0404606	5.54300066	0.56007739	-6.9077553	0	94	1.34111667	276.888889	3.888888888	0	0	0	0	34.0724638	1	0.49277013
0.0252	1048.92269	0.54313187	77.0979207	0.96425319	0	-3.6809113	0.04871653	-0.6085637	4.34508928	-0.0353648	-6.9077553	1	58	1.78815556	284.666667	11.66666666	0	0	0	0	34.0724638	1	0.49277013
0.0909	3810.26016	0.91528205	237.22873	2.1548834	0	-2.3979953	1.33795989	-0.087431	5.469029	0.76820057	-6.9077553	2	36	3.12927222	290.222222	17.2222222	0	0	0	0	34.0724638	1	0.49277013
0.1773	7749.98808	0.97997562	254.955688	1.80714258	0	-1.7299121	2.04782033	-0.0192077	5.54109368	0.59230012	-6.9077553	3	31	3.57631111	293.555556	20.55555555	0	0	0	0	34.0724638	1	0.49277013
0.3024	12769.4896	1.01107722	275.265569	2.44634571	0	-1.1960046	2.54713701	0.01200487	5.61773997	0.89500405	-6.9077553	4	22	5.36446667	296.333333	23.33333333	0	0	0	1	34.0724638	1	0.49277013
0.6381	34503.6833	1.44877645	612.390457	5.10393976	0	-0.4492603	3.54109506	0.37140937	6.41737171	1.63020865	-6.9077553	5	20	5.81150556	297.444444	24.44444444	0	0	0	1	34.0724638	1	0.49277013
1.0242	56504.0631	1.33003004	522.872742	3.63947771	0	0.02391182	4.03433025	0.28595311	6.25934002	1.29211491	-6.9077553	6	18	4.91742778	298.555556	25.55555555	0	0	0	1	34.0724638	1	0.49277013
1.7433	78153.5711	1.3434533	537.394634	4.47109302	2	0.55577987	4.35868855	0.29598746	6.28673457	1.49785654	0.69364706	7	16	5.36446667	299.111111	26.11111111	0	0	0	1	34.0724638	1	0.49277013
2.4768	103347.804	1.20335259	430.158972	3.2587648	2	0.9069674	4.63810972	0.18594215	6.06415717	1.18165504	0.69364706	8	18	5.36446667	298.555556	25.55555555	0	0	0	1	34.0724638	1	0.49277013
3.1104	127825.432	1.03613073	308.163108	2.37307809	2	1.13475134	4.85067335	0.03645799	5.73063246	0.86460919	0.69364706	9	25	2.68223333	295.222222	22.2222222	0	0	0	1	34.0724638	1	0.49277013
3.618	145301.1	0.96149699	261.627715	1.91847045	2	1.28592139	4.97881502	-0.0382243	5.56692638	0.65204934	0.69364706	10	29	1.34111667	293	20	0	0	0	1	34.0724638	1	0.49277013
4.0608	159589.911	0.8762542	214.470878	1.63736284	2	1.40138	5.07261374	-0.1309585	5.36817862	0.49369748	0.69364706	11	31	1.34111667	290.777778	17.7777778	0	0	0	1	34.0724638	1	0.49277013
4.5603	171378.191	0.85909231	209.625676	2.1074927	2	1.51738841	5.14387859	-0.1507156	5.34532821	0.74597333	0.69364706	12	33	2.68223333	290.222222	17.2222222	0	0	0	1	34.0724638	1	0.49277013
5.0499	183973.13	0.79938165	178.929084	1.72854884	2	1.61936844	5.21479515	-0.2226666	5.18699514	0.54786059	0.69364706	13	35	3.12927222	290.222222	17.2222222	0	0	0	1	34.0724638	1	0.49277013
5.5521	198143.641	0.79604222	178.765233	1.5870206	2	1.71417623	5.28899727	-0.2268476	5.18607899	0.46248834	0.69364706	14	36	3.57631111	290.222222	17.2222222	0	0	0	1	34.0724638	1	0.49277013
6.0453	213691.738	0.79534545	171.92236	1.58433622	2	1.79928111	5.36453918	-0.2277222	5.14704879	0.46079651	0.69364706	15	40	3.57631111	289.111111	16.11111111	0	0	0	1	34.0724638	1	0.49277013
6.5448	228903.192	0.76533467	159.244144	1.5802576	2	1.87867084	5.43330354	-0.2661363	5.0704448	0.45822048	0.69364706	16	44	3.57631111	288	15	0	0	0	1	34.0724638	1	0.49277013
7.1316	246341.756	0.81215101	191.134131	1.91685626	2	1.96453561	5.50672388	-0.2068384	5.25298067	0.65120803	0.69364706	17	44	4.91742778	286.888889	13.88888888	0	0	0	1	34.0724638	1	0.49277013
7.6806	263823.227	0.75947201	168.513589	1.76342873	2	2.03869767	5.57528308	-0.273816	5.12702233	0.56782698	0.69364706	18	51	4.02335	285.777778	12.77777777	0	0	0	1	34.0724638	1	0.49277013
8.0577	279479.41	0.84403821	201.282816	1.51492987	2	2.08662816	5.6329322	-0.1683734	5.30471593	0.41602903	0.69364706	19	63	2.23519444	284.666667	11.66666666	0	0	0	1	34.0724638	1	0.49277013
8.3124	289809.582	0.8016655	171.804826	1.35245031	2	2.11774838	5.66922754	-0.2198172	5.14636492	0.30265712	0.69364706	20	71	2.23519444	284.666667	11.66666666	0	0	0	1	34.0724638	1	0.49277013
8.5167	298940.469	0.79460863	165.426315	1.30857358	2	2.14202894	5.7002478	-0.2286479	5.10853191	0.26970158	0.69364706	21	73	2.68223333	284.666667	11.66666666	0	0	0	1	34.0724638	1	0.49277013
8.6706	305414.372	0.67878161	120.260874	1.0867986	2	2.15993799	5.72167272	-0.3859837	4.78967164	0.08415602	0.69364706	22	79	3.57631111	284.666667	11.66666666	0	0	0	1	34.0724638	1	0.49277013
8.766	309713.016	0.55492408	78.316351	0.86507502	2	2.1708806	5.73564934	-0.5871235	4.36076917	-0.1437837	0.69364706	23	83	4.47038889	286.333333	13.33333333	0	0	0	1	34.0724638	1	0.49277013
0.0306	1845.17392	1.07603501	316.928089	2.11538835	0	-3.4867553	0.61311535	0.0742119	5.75867806	0.74971103	-6.9077553	0	94	1.34111667	276.888889	3.888888888	0	0	0	0	42.288408	1	-0.4111751
0.1143	6199.70432	0.98772726	267.182796	2.12823093	0	-2.1689287	1.82466289	-0.0113368	5.58793679	0.75576085	-6.9077553	1	58	1.78815556	284.666667	11.66666666	0	0	0	0	42.288408	1	-0.4111751
0.3555	17120.0891	1.0956307	332.27335	3.2765963	0	-1.03423	2.84031098	0.09224248	5.80596098	1.18711032	-6.9077553	2	36	3.12927222	290.222222	17.2222222	0	0	0	0	42.288408	1	-0.4111751
0.6516	32133.0803	1.05539024	304.029944	2.18098519	0	-0.4283244	3.46991716	0.05485766	5.71712949	0.7802351	-6.9077553	3	31	3.57631111	293.555556	20.55555555	0	0	0	0	42.288408	1	-0.4111751
1.0233	46991.2354	0.9810007	260.273343	2.46773945	0	0.0230327	3.84998238	-0.0181633	5.56173624	0.90370768	-6.9077553	4	22	5.36446667	296.333333	23.33333333	0	0	0	0	42.288408	1	-0.4111751
1.5381	69017.7246	1.04282214	308.693837	2.83753978	0	0.43054789	4.23437784	0.04288911	5.73235321	1.04328976	-6.9077553	5	20	5.81150556	297.444444	24.44444444	0	0	1	0	42.288408	1	-0.4111751
2.3238	106264.106	1.10996934	353.944337	3.11440855	0	0.84320378	4.66593697	0.10523291	5.86914249	1.1363603	-6.9077553	6	18	4.91742778	298.555556	25.55555555	0	0	1	0	42.288408	1	-0.4111751
3.0906	144035.001	1.11318569	358.895613	2.72494321	0	1.12836525	4.97006328	0.10812381	5.88303436	1.0028145	-6.9077553	7	16	5.36446667	299.111111	26.11111111	0	0	1	0	42.288408	1	-0.4111751
3.9123	181258.036	1.10937065	378.292716	3.04419106	0	1.36412544	5.19992715	0.10469388	5.93567092	1.11356364	-6.9077553	8	18	5.36446667	298.555556	25.55555555	0	0	1	0	42.288408	1	-0.4111751
4.5027	207543.388	0.96225711	275.296327	2.07762258	0	1.50467722	5.33534523	-0.0374349	5.6178517	0.73170545	-6.9077553	9	25	2.68223333	295.222222	22.2222222	0	0	1	0	42.288408	1	-0.4111751
5.0913	233103.603	0.92397931	253.45604	1.97642195	0	1.6275332	5.45148729	-0.0779839	5.53519434	0.68179395	-6.9077553	10	29	1.34111667	293	20	0	0	1	0	42.288408	1	-0.4111751
5.6637	253626.693	0.85726007	215.806358	1.74140709	0	1.73407739	5.53586742	-0.1528481	5.37438615	0.55526754	-6.9077553	11	31	1.34111667	290.777778	17.7777778	0	0	1	0	42.288408	1	-0.4111751
6.0858	267521.358	0.75653467	171.843317	1.42730309	0	1.80595819	5.58920314	-0.277686	5.14658893	0.35648709	-6.9077553	12	33	2.68223333	290.222222	17.2222222	0	0	1	0	42.288408	1	-0.4111751
6.4926	280495.832	0.75574163	180.095461	1.47793079	0	1.87066307	5.63656243	-0.2787334	5.1934926	0.39131939	-6.9077553	13	35	3.12927222	290.222222	17.2222222	0	0	1	0	42.288408	1	-0.4111751
6.894	291106.745	0.69121487	151.633934	1.39997241	0	1.93065147	5.67369346	-0.3678589	5.02147588	0.33716657	-6.9077553	14	36	3.57631111	290.222222	17.2222222	0	0	1	0	42.288408	1	-0.4111751
7.3107	300745.279	0.66154292	139.761744	1.33171408	0	1.98933903	5.70626698	-0.4116699	4.9399463	0.28721752	-6.9077553	15	40	3.57631111	289.111111	16.11111111	0	0	1	0	42.288408	1	-0.4111751
7.7103	309298.448	0.66316691	139.910429	1.33414672	0	2.0425571	5.7343099	-0.4092218	4.94100957	0.28904119	-6.9077553	16	44	3.57631111	288	15	0	0	1	0	42.288408	1	-0.4111751
8.1639	317523.667	0.70679117	169.325164	1.77557082	0	2.099722	5.76055551	-0.3456062	5.13182682	0.574685	-6.9077553	17	44	4.91742778	286.888889	13.88888888	0	0	1	0	42.288408	1	-0.4111751
8.5383	325710.036	0.74166496	194.08806	1.61049887	0	2.14456192	5.7860106	-0.2975103	5.26831712	0.47716472	-6.9077553	18	51	4.02335	285.777778	12.77777777	0	0	1	0	42.288408	1	-0.4111751
8.7012	330574.109	0.74064206	164.82929	1.20810721	0	2.16346095	5.80083389	-0.2988885	5.1049164	0.18988224	-6.9077553	19	63	2.23519444	284.666667	11.66666666	0	0	1	0	42.288408	1	-0.4111751
8.7705	333206.02	0.7065075	127.958934	1.01689178	0	2.17139382	5.80876398	-0.3460071	4.8517172	0.0177336	-6.9077553	20	71	2.23519444	284.666667	11.66666666	0	0	1	0	42.288408	1	-0.4111751
8.8407	335891.966	0.71128229	131.689804	1.13178194	0	2.17936606	5.81679255	-0.339281	4.88045678	0.12467651	-6.9077553	21	73	2.68223333	284.666667	11.66666666	0	0	1	0	42.288408	1	-0.4111751
8.9208	339463.61	0.77061342	167.942493	1.39874028	0	2.18838563	5.8273697	-0.2592716	5.12362757	0.3362867	-6.9077553	22	79	3.57631111	284.666667	11.66666666	0	0	1	0	42.288408	1	-0.4111751
8.9883	342156.698	0.70422877	161.080065	1.76916707	0	2.19592373	5.83527174	-0.349233	5.08190775	0.57107393	-6.9077553	23	83	4.47038889	286.333333	13.33333333	0	0	1	0	42.288408	1	-0.4111751
0.0018	0	0.16748893	5.34041262	0.42339128	0	-6.3199686	-6.9077553	-1.7808852	1.67549015	-0.8570994	-6.9077553	0	94	1.34111667	276.888889	3.888888888	0	0	0	0	18.9135952	1	-0.0428739
0.0018	0	0	0	0	0	-6.3199686	-6.9077553	-6.9077553	-6.9077553	-6.9077553	-6.9077553	1	58	1.78815556	284.666667	11.66666666	0	0	0	0	18.9135952	1	-0.0428739
0.0252	0	0.4354755	51.5123385	1.57020672	0	-3.6809113	-6.9077553	-0.829023	3.94184077	0.45184393	-6.9077553	2	36	3.12927222	290.222222	17.2222222	0	0	0	0	18.9135952	1	-0.0428739
0.1584	91.5282212	0.64509237	113.444336	2.89320992	0	-1.8426318	-2.3802416	-0.4368128	4.7313211	1.06271216	-6.9077553	3	31	3.57631111	293.555556	20.55555555	0	0	1	0	18.9135952	1	-0.0428739
0.3843	4207.88617	0.78496889	222.597765	3.07966231	0	-0.9563318	1.43719805	-0.2408381	5.40537089	1.12514461	-6.9077553	4	22	5.36446667	296.333333	23.33333333	0	0	1	0	18.9135952	1	-0.0428739
0.6255	13916.8631	0.98004219	345.137331	2.75079756	0	-0.4692039	2.63317313	-0.0191398	5.8439453	1.01225436	-6.9077553	5	20	5.81150556	297.444444	24.44444444	0	0	1	0	18.9135952	1	-0.0428739
0.8424	19124.9338	0.81802976	214.739643	1.88626922	0	-0.1715003	2.95104521	-0.1996349	5.36943099	0.63513093	-6.9077553	6	18	4.91742778	298.555556	25.55555555	0	0	1	0	18.9135952	1	-0.0428739
1.1592	30634.19	0.97819987	301.157582	2.64434814	0	0.14773011	3.42214935	-0.0210195	5.70763698	0.97280268	-6.9077553	7	16	5.36446667	299.111111	26.11111111	0	0	1	0	18.9135952	1	-0.0428739
1.5471	44074.3157	0.96210963	282.503752	2.55176404	0	0.43638221	3.78589989	-0.037588	5.64369537	0.93717671	-6.9077553	8	18	5.36446667	298.555556	25.55555555	0	0	1	0	18.9135952	1	-0.0428739
1.8801	57249.8309	0.88770125	239.805173	1.78361948	0	0.63132497	4.04744216	-0.1179942	5.47983099	0.57920522	-6.9077553	9	25	2.68223333	295.222222	22.2222222	0	0	1	0	18.9135952	1	-0.0428739
2.2014	69099.958	0.81446902	201.071647	1.59419947	0	0.78909352	4.2355686	-0.2039918	5.30366627	0.46699879	-6.9077553	10	29	1.34111667	293	20	0	0	1	0	18.9135952	1	-0.0428739
2.6604	86208.246	0.87630508	234.479105	1.9805992	0	0.97847649	4.45677743	-0.1309005	5.45737075	0.68390419	-6.9077553	11	31	1.34111667	290.777778	17.7777778	0	0	1	0	18.9135952	1	-0.0428739
3.0717	102569.103	0.85458314	221.207809	1.80871235	0	1.12223115	4.6305465	-0.155972	5.39910709	0.59316791	-6.9077553	12	33	2.68223333	290.222222	17.2222222	0	0	1	0	18.9135952	1	-0.0428739
3.5262	116536.662	0.79692214	188.11564	1.91478078	0	1.2602208	4.7582145	-0.2257443	5.2370622	0.65012526	-6.9077553	13	35	3.12927222	290.222222	17.2222222	0	0	1	0	18.9135952	1	-0.0428739

3.9933	132771.891	0.80681879	193.191203	1.64965385	0	1.38461796	4.88864008	-0.2134175	5.26368556	0.50117148	-6.9077553	14	36	3.57631111	290.222222	17.2222222	0	0	1	0	18.9135952	1	-0.0428739
4.4577	150476.101	0.81326945	194.498774	1.61476703	0	1.49463294	5.01381092	-0.205464	5.270431	0.47980979	-6.9077553	15	40	3.57631111	289.111111	16.1111111	0	0	1	0	18.9135952	1	-0.0428739
4.95	169091.418	0.83028388	208.085552	1.88471117	0	1.59938758	5.13044542	-0.1847839	5.33795411	0.63430503	-6.9077553	16	44	3.57631111	288	15	0	0	1	0	18.9135952	1	-0.0428739
5.3928	182841.964	0.73390161	158.798892	1.66216235	0	1.68506473	5.20862766	-0.3080187	5.06764487	0.50872082	-6.9077553	17	44	4.91742778	286.888889	13.8888888	0	0	1	0	18.9135952	1	-0.0428739
5.778	195454.176	0.71099304	145.435237	1.26791553	0	1.7540576	5.27533107	-0.3396871	4.97973775	0.23816262	-6.9077553	18	51	4.02335	285.777778	12.7777777	0	0	1	0	18.9135952	1	-0.0428739
6.102	209654.426	0.83111623	192.343229	1.39032474	0	1.80861659	5.34546535	-0.1837831	5.25928663	0.33025634	-6.9077553	19	63	2.23519444	284.666667	11.6666666	0	0	1	0	18.9135952	1	-0.0428739
6.4494	227138.616	0.92330102	231.476886	1.69213124	0	1.8639871	5.42556488	-0.0787175	5.44448435	0.52657962	-6.9077553	20	71	2.23519444	284.666667	11.6666666	0	0	1	0	18.9135952	1	-0.0428739
6.7995	244300.997	0.88535487	210.303382	1.61464269	0	1.91684908	5.49840515	-0.1206379	5.34855592	0.47973283	-6.9077553	21	73	2.68223333	284.666667	11.6666666	0	0	1	0	18.9135952	1	-0.0428739
7.1343	260554.654	0.79786633	171.44065	1.42754553	0	1.96491414	5.56281648	-0.2245616	5.14424298	0.35665681	-6.9077553	22	79	3.57631111	284.666667	11.6666666	0	0	1	0	18.9135952	1	-0.0428739
7.3593	270780.222	0.66870117	118.685539	1.15916943	0	1.99596482	5.6013112	-0.4009237	4.77648589	0.14856606	-6.9077553	23	83	4.47038889	286.333333	13.3333333	0	0	1	0	18.9135952	1	-0.0428739
0.0081	320.894375	0.84135509	190.713745	1.69075966	0	-4.8158912	-1.1335318	-0.1715536	5.25077883	0.52576921	-6.9077553	0	93	4.47038889	293.555556	20.5555555	0	0	0	0	30.519848	1	0.21141735
0.0108	452.485762	0.45106272	54.8309123	0.74355086	0	-4.5282091	-0.7907914	-0.7939343	4.00427237	-0.2949741	-6.9077553	1	92	2.68223333	293.555556	20.5555555	0	0	0	0	30.519848	1	0.21141735
0.0225	979.210667	0.56892648	86.1075346	1.03902611	0	-3.79424	-0.0199878	-0.5622479	4.45560853	0.03924582	-6.9077553	2	91	2.68223333	294.111111	21.1111111	0	0	0	0	30.519848	1	0.21141735
0.0495	1374.1955	0.46611468	67.4026123	1.05682901	0	-3.0057826	0.31859591	-0.7611805	4.21069861	0.0562187	-6.9077553	3	91	2.68223333	294.666667	21.6666666	0	0	0	0	30.519848	1	0.21141735
0.0873	1812.8333	0.34316079	32.4077148	1.0325598	0	-2.4384048	0.59544245	-1.0666463	3.47842736	0.03300896	-6.9077553	4	85	3.57631111	296.333333	23.3333333	0	0	0	0	30.519848	1	0.21141735
0.189	2600.37835	0.50526486	72.9838305	2.10302532	0	-1.6660083	0.95604144	-0.6806953	4.29025162	0.74385233	-6.9077553	5	71	5.36446667	298.555556	25.5555555	0	0	0	0	30.519848	1	0.21141735
0.3276	3629.55341	0.64108564	116.994585	2.57015773	0	-1.1159619	1.28938509	-0.4430336	4.76213619	0.94435628	-6.9077553	6	67	5.81150556	299.666667	26.6666666	0	0	0	0	30.519848	1	0.21141735
0.4941	6009.51958	0.7144269	145.583868	2.29764668	0	-0.7050174	1.7935112	-0.3348759	4.9807592	0.83232055	-6.9077553	7	55	6.25854444	301.888889	28.8888888	0	0	0	1	30.519848	1	0.21141735
0.7668	16119.7633	0.85914556	220.7533	2.84990972	0	-0.2655293	2.78010809	-0.1506536	5.39705032	1.04763814	-6.9077553	8	50	5.81150556	303.555556	30.5555555	0	0	0	1	30.519848	1	0.21141735
1.0827	25023.8641	0.72547873	145.41177	2.18102019	0	0.07945792	3.21986989	-0.3195461	4.97957639	0.78025114	-6.9077553	9	45	5.36446667	304.111111	31.1111111	0	0	0	1	30.519848	1	0.21141735
1.3392	33276.6615	0.69817815	136.788816	1.53688183	0	0.29207242	3.50488635	-0.3578497	4.91844556	0.43040604	-6.9077553	10	46	3.57631111	304.111111	31.1111111	0	0	0	1	30.519848	1	0.21141735
1.557	42623.3341	0.71092413	146.46055	1.37660457	0	0.44276089	3.75242531	-0.3397839	4.98676294	0.32034617	-6.9077553	11	44	4.02335	304.666667	31.6666666	0	0	0	1	30.519848	1	0.21141735
1.7379	52658.9498	0.72718059	149.858287	1.22365802	0	0.55267749	3.9638552	-0.3172062	5.00969677	0.20266163	-6.9077553	12	49	3.12927222	303	30	0	0	0	1	30.519848	1	0.21141735
1.8603	60620.6152	0.64549306	121.447395	0.91408241	0	0.62073777	4.10465152	-0.4361928	4.79948944	-0.0887411	-6.9077553	13	53	3.12927222	301.333333	28.3333333	0	0	0	1	30.519848	1	0.21141735
1.9656	67465.0582	0.68768637	133.617513	0.93424883	0	0.67579754	4.21162463	-0.3729693	4.89498882	-0.0669427	-6.9077553	14	58	1.78815556	299.111111	26.1111111	0	0	0	1	30.519848	1	0.21141735
2.0691	75623.9338	0.68850681	138.46723	1.0092926	0	0.72711373	4.32578604	-0.3717787	4.93064091	0.01023999	-6.9077553	15	51	2.68223333	298.555556	25.5555555	0	0	0	1	30.519848	1	0.21141735
2.1897	84248.4745	0.6182768	114.446967	0.9109763	0	0.78376455	4.43378233	-0.4792029	4.74012028	-0.0921413	-6.9077553	16	53	2.23519444	297.444444	24.4444444	0	0	0	1	30.519848	1	0.21141735
2.2815	92678.2498	0.74218922	154.052016	1.01843591	0	0.82483312	4.52914461	-0.2968046	5.0372968	0.01924944	-6.9077553	17	62	2.68223333	296.333333	23.3333333	0	0	0	1	30.519848	1	0.21141735
2.349	101348.266	0.711701	133.011901	0.97152171	0	0.85398971	4.61857263	-0.3386933	4.89044612	-0.0278629	-6.9077553	18	73	3.12927222	295.777778	22.7777778	0	0	0	1	30.519848	1	0.21141735
2.394	107286.96	0.63197598	114.445498	0.99018021	0	0.87296561	4.67551644	-0.4573228	4.74010745	-0.0088589	-6.9077553	19	80	2.23519444	295.222222	22.2222222	0	0	0	1	30.519848	1	0.21141735
2.4039	107345.008	0.74357388	183.819236	2.00900789	0	0.87709242	4.67605733	-0.2949432	5.2139583	0.69813865	-6.9077553	20	84	3.12927222	295.222222	22.2222222	0	0	0	1	30.519848	1	0.21141735
2.4084	107400.959	0.36267369	42.9002502	1.16377811	0	0.87896263	4.67657842	-1.0114983	3.75890097	0.15253061	-6.9077553	21	88	2.23519444	294.666667	21.6666666	0	0	0	1	30.519848	1	0.21141735
2.412	107444.822	0.26981509	20.2910576	0.36663026	0	0.88045628	4.67698674	-1.306319	3.01022956	-1.0006776	-6.9077553	22	90	2.23519444	294.666667	21.6666666	0	0	0	1	30.519848	1	0.21141735
2.4129	107488.685	0.17671391	6.00058508	0.18715119	0	0.88082934	4.67739489	-1.7275802	1.79202361	-1.6705094	-6.9077553	23	89	3.12927222	295.222222	22.2222222	0	0	0	1	30.519848	1	0.21141735
0.0162	711.41297	1.0147235	286.025563	2.16277111	0	-4.122744	-0.3390975	0.01560116	5.65608468	0.77185258	-6.9077553	0	93	4.47038889	293.555556	20.5555555	0	0	0	0	34.0724638	1	0.49277013
0.0315	1325.50591	0.45035197	51.4235158	0.86782461	0	-3.4577677	0.28254835	-0.7955078	3.94011502	-0.140614	-6.9077553	1	92	2.68223333	293.555556	20.5555555	0	0	0	0	34.0724638	1	0.49277013
0.0441	1676.41625	0.43786389	44.2046116	0.61188105	0	-3.1212955	0.51725466	-0.8235659	3.78885174	-0.4895844	-6.9077553	2	91	2.68223333	294.111111	21.1111111	0	0	0	0	34.0724638	1	0.49277013
0.0621	2334.3729	0.50191102	65.6222961	1.05648594	0	-2.7790093	0.84817158	-0.687342	4.18393076	0.05589434	-6.9077553	3	91	2.68223333	294.666667	21.6666666	0	0	0	0	34.0724638	1	0.49277013
0.1215	4786.60411	0.7455994	149.905495	1.45403533	0	-2.107841	1.5660301	-0.2922265	5.01001174	0.37503018	-6.9077553	4	85	3.57631111	296.333333	23.3333333	0	0	0	0	34.0724638	1	0.49277013
0.1791	7505.73427	0.86943746	204.276276	1.42441595	0	-1.719811	2.01580052	-0.1387594	5.31947827	0.35446366	-6.9077553	5	71	5.36446667	298.555556	25.5555555	0	0	0	0	34.0724638	1	0.49277013
0.2979	12887.0656	0.99857608	287.091264	2.57702984	0	-1.2109974	2.55630173	-0.000424	5.65980364	0.94702548	-6.9077553	6	67	5.81150556	299.666667	26.6666666	0	0	0	1	34.0724638	1	0.49277013
0.576	26729.4163	1.28319088	497.381877	4.01454493	0	-0.5516476	3.2858021	0.25012885	6.20936011	1.39017306	-6.9077553	7	55	6.25854444	301.888889	28.8888888	0	0	0	1	34.0724638	1	0.49277013
1.233	49222.4658	1.35515421	541.179195	4.75578914	0	0.20945022	3.89637046	0.30465291	6.2937523	1.55957289	-6.9077553	8	50	5.81150556	303.555556	30.5555555	0	0	0	1	34.0724638	1	0.49277013
1.845	67745.2723	1.15699203	397.899632	3.36273445	0	0.61247928	4.21576944	0.14668749	5.98620231	1.2130518	-6.9077553	9	45	5.36446667	304.111111	31.1111111	0	0	0	1	34.0724638	1	0.49277013
2.3454	83316.774	1.00092277	282.03288	2.21333104	0	0.85245596	4.4226619	0.00192093	5.64202721	0.79495035	-6.9077553	10	46	3.57631111	304.111111	31.1111111	0	0	0	1	34.0724638	1	0.49277013
2.8971	102307.341	1.00779468	284.930796	2.38494047	0	1.06371024	4.62799121	0.00875623	5.65224984	0.86959337	-6.9077553	11	44	4.02335	304.666667	31.6666666	0	0	0	1	34.0724638	1	0.49277013
3.447	119960.179	0.92868746	237.496573	2.13455545	0	1.23750429	4.78716819	-0.0729068	5.47015741	0.75872678	-6.9077553	12	49	3.12927222	303	30	0	0	0	1	34.0724638	1	0.49277013
3.8844	132392.179	0.79952017	190.936182	1.63260428	0	1.35696853	4.88577612	-0.2224935	5.25194448	0.49078879	-6.9077553	13	53	3.12927222	301.333333	28.3333333	0	0	0	1	34.0724638	1	0.49277013
4.1877	143104.983	0.74816732	167.279604	1.28477048	0	1.43215166	4.96358549	-0.2887929	5.11967267	0.25135813	-6.9077553	14	58	1.78815556	299.111111	26.1111111	0	0	0	1	34.0724638	1	0.49277013
4.4721	152359.261	0.70232749	152.516416	1.26359568	0	1.4978581	5.02624786	-0.3519327	5.02727879	0.23475245	-6.9077553	15	51	2.68223333	298.555556	25.5555555	0	0	0	1	34.0724638	1	0.49277013
4.8348	162595.568	0.68788635	143.343992	1.21814942	0	1.57583976	5.09127209	-0.372679	4.96525426	0.19815342	-6.9077553	16	53	2.23519444	297.444444	24.4444444	0	0	0	1	34.0724638	1	0.49277013
5.0301	167242.939	0.69650719	143.450218	1.14693243	0	1.61543986	5.11945346	-0.3602425	4.96599503	0.13796244	-6.9077553	17	62	2.68223333	296.333333	23.3333333	0	0	0	1	34.0724638	1	0.49277013
5.1777	172892.521	0.77586095	163.804283	1.34035008	0	1.64436094	5.15267592	-0.2524939	5.09867842	0.29367663	-6.9077553	18	73	3.12927222	295.777778	22.7777778	0	0	0	1	34.0724638	1	0.49277013
5.2983	177892.989	0.61911147	100.497449	1.05148361	0	1.66738601	5.18118781	-0.477856	4.6101423	0.05115271	-6.9077553	19	80	2.23519444	295.222222	22.2222222	0	0	0	1	34.0724638	1	0.49277013
5.3442	179954.61	0.27162062	17.5364966	0.47194197	0	1.67601186	5.19271021	-1.2996741	2.86434125	-0.7487826	-6.9077553	20	84	3.12927222	295.222222	22.2222222	0	0	0	1	34.0724638	1	0.49277013
5.3928	182147.774	0.21613232	10.0930639	0.34495085	0	1.68506473	5.20482379	-1.5272483	2.31194752	-1.0614586	-6.9077553	21	88	2.23519444	294.666667	21.6666666	0	0	0	1	34.0724638	1	0.49277013
5.4351	183770.744	0.26046654	24.616572	0.44208575	0	1.69287792	5.21369446	-1.341449	3.2034605	-0.813992	-6.9077553	22	90	2.23519444	294.666667	21.6666666	0	0	0	1	34.0724638	1	0.49277013
5.4729	185525.291	0.1743073	5.97898465	0.22841481	0	1.69980864	5.2231966	-1.7412148	1.788418	-1.4722235	-6.9077553	23	89	3.12927222	295.222222	22.2222222	0	0	0	1	34.0724638	1	0.49277013
0.0369	2389.01059	1.12993679	355.276001	2.47897625	0	-3.2995437	0.8712978	0.12304631	5.87289777	0.90824898	-6.9077553	0	93	4.47038889	293.555556	20.5555555	0	0	0	0	42.288408	1	-0.4111751
0.0783	4364.01957	0.61484229	96.9875488	1.11610346	0	-2.5472077	1.47362267	-0.4847644	4.57459292	0.11073914	-6.9077553	1	92	2.68223333	293.555556	20.5555555	0	0	0	0	42.288408	1	-0.4111751
0.1233	6513.3458	0.60359936	92.3819629	1.0657222	0	-2.0931349	1.87400679	-0.5031893	4.52594258	0.06459058	-6.9077553	2	91	2.68223333	294.111111	21.1111111	0	0	0	0	42.288408	1	-0.4111751
0.2655	11516.3388	0.77896558	169.544329	2.13058394	0	-1.3261404	2.44385362	-0.2485055	5.13312032	0.75686534	-6.9077553	3	91	2.68223333	294.666667	21.6666666	0	0	0	0	42.288408	1	-0.4111751

0.4725	20830.0034	0.92543295	238.203431	2.15229758	0	-0.7497175	3.03644243	-0.0764136	5.47312926	0.76700042	-6.9077553	4	85	3.57631111	296.333333	23.33333333	0	0	0	0	42.288408	1	-0.4111751
0.7596	32651.6776	0.90515596	224.89136	2.23337777	0	-0.2749633	3.48592686	-0.0985438	5.41562189	0.80396279	-6.9077553	5	71	5.36446667	298.555556	25.55555555	0	0	0	0	42.288408	1	-0.4111751
1.1475	46495.5214	0.89938673	219.496701	2.43382883	0	0.13758566	3.8393775	-0.1049309	5.39134176	0.88987646	-6.9077553	6	67	5.81150556	299.666667	26.66666666	0	0	1	0	42.288408	1	-0.4111751
1.6704	65621.18	0.8882565	221.835521	2.33134757	0	0.51306312	4.18391375	-0.1173696	5.40194072	0.8468753	-6.9077553	7	55	6.25854444	301.888889	28.88888888	0	0	1	0	42.288408	1	-0.4111751
2.2131	84754.6292	0.87936174	209.590433	2.2408842	0	0.79439425	4.43977217	-0.1274224	5.34516008	0.80731667	-6.9077553	8	50	5.81150556	303.555556	30.55555555	0	0	1	0	42.288408	1	-0.4111751
2.8584	115868.604	1.00517956	285.502005	2.24902293	0	1.05026203	4.75246546	0.00616055	5.65425255	0.81094041	-6.9077553	9	45	5.36446667	304.111111	31.11111111	0	0	1	0	42.288408	1	-0.4111751
3.6099	154560.419	1.03256924	301.883402	2.19354635	0	1.28368007	5.04059155	0.03301809	5.71004417	0.78597535	-6.9077553	10	46	3.57631111	304.111111	31.11111111	0	0	1	0	42.288408	1	-0.4111751
4.3362	189496.136	1.01600982	297.272692	2.41281941	0	1.46699839	5.24437391	0.01686677	5.69465324	0.88121031	-6.9077553	11	44	4.02335	304.666667	31.66666666	0	0	1	0	42.288408	1	-0.4111751
4.9176	211581.445	0.88334566	220.763932	1.8065212	0	1.59282061	5.35461473	-0.1229073	5.39709848	0.5919564	-6.9077553	12	49	3.12927222	303	30	0	0	1	0	42.288408	1	-0.4111751
5.3568	230686.676	0.8198316	196.174741	1.47221875	0	1.67836678	5.44106474	-0.1974373	5.27901089	0.38744963	-6.9077553	13	53	3.12927222	301.333333	28.33333333	0	0	1	0	42.288408	1	-0.4111751
5.6943	245318.682	0.82549414	191.948063	1.35515658	0	1.73946567	5.50256219	-0.1905625	5.25723004	0.30465465	-6.9077553	14	58	1.78815556	299.111111	26.11111111	0	0	1	0	42.288408	1	-0.4111751
5.9904	256747.203	0.78271772	178.238614	1.29334628	0	1.79015819	5.54809585	-0.2437064	5.18312879	0.25800576	-6.9077553	15	51	2.68223333	298.555556	25.55555555	0	0	1	0	42.288408	1	-0.4111751
6.3027	266535.413	0.68042142	144.342906	1.1084184	0	1.84097811	5.58551087	-0.3835743	4.97219869	0.10383592	-6.9077553	16	53	2.23519444	297.444444	24.44444444	0	0	1	0	42.288408	1	-0.4111751
6.4998	274038.469	0.79719209	180.688873	1.29347347	0	1.87177141	5.61327214	-0.225406	5.19678216	0.25810403	-6.9077553	17	62	2.68223333	296.333333	23.33333333	0	0	1	0	42.288408	1	-0.4111751
6.6114	278258.927	0.72101439	140.890247	1.32213223	0	1.88879543	5.62855567	-0.3257102	4.94798829	0.28000183	-6.9077553	18	73	3.12927222	295.777778	22.7777778	0	0	1	0	42.288408	1	-0.4111751
6.6897	281329.384	0.57972893	84.563948	0.91490357	0	1.90056903	5.63952972	-0.5434712	4.43751985	-0.0878442	-6.9077553	19	80	2.23519444	295.222222	22.2222222	0	0	1	0	42.288408	1	-0.4111751
6.7176	282338.266	0.23228781	12.0086562	0.33555855	0	1.90473095	5.64310942	-1.4554824	2.48571101	-1.0889831	-6.9077553	20	84	3.12927222	295.222222	22.2222222	0	0	1	0	42.288408	1	-0.4111751
6.7428	283434.854	0.22250928	10.641474	0.38406621	0	1.90847527	5.64698583	-1.4983023	2.36485298	-0.95434	-6.9077553	21	88	2.23519444	294.666667	21.66666666	0	0	1	0	42.288408	1	-0.4111751
6.7653	284136.675	0.18420561	7.29355408	0.30667084	0	1.91180661	5.64945889	-1.6862887	1.98712805	-1.1787248	-6.9077553	22	90	2.23519444	294.666667	21.66666666	0	0	1	0	42.288408	1	-0.4111751
6.7806	284706.897	0.16627275	5.61502748	0.23002397	0	1.91406559	5.65146373	-1.7881296	1.72562456	-1.4652338	-6.9077553	23	89	3.12927222	295.222222	22.2222222	0	0	1	0	42.288408	1	-0.4111751
0.0018	0	0.45057252	45.9067039	2.08759046	0	-6.3199686	-6.9077553	-0.7950193	3.82663295	0.73648942	-6.9077553	0	93	4.47038889	293.555556	20.55555555	0	0	0	0	18.9135952	1	-0.0428739
0.0018	0	0	0	0	0	-6.3199686	-6.9077553	-6.9077553	-6.9077553	-6.9077553	-6.9077553	1	92	2.68223333	293.555556	20.55555555	0	0	0	0	18.9135952	1	-0.0428739
0.0018	0	0	0	0	0	-6.3199686	-6.9077553	-6.9077553	-6.9077553	-6.9077553	-6.9077553	2	91	2.68223333	294.111111	21.11111111	0	0	0	0	18.9135952	1	-0.0428739
0.0018	0	0	0	0	0	-6.3199686	-6.9077553	-6.9077553	-6.9077553	-6.9077553	-6.9077553	3	91	2.68223333	294.666667	21.66666666	0	0	0	0	18.9135952	1	-0.0428739
0.0072	0	0.29284527	24.8875732	1.16351612	0	-4.9336743	-6.9077553	-1.2247019	3.21440879	0.15230566	-6.9077553	4	85	3.57631111	296.333333	23.33333333	0	0	0	0	18.9135952	1	-0.0428739
0.0738	105.066622	0.58995788	100.013843	2.50551708	0	-2.6063965	-2.2436879	-0.5260105	4.60531861	0.91889417	-6.9077553	5	71	5.36446667	298.555556	25.55555555	0	0	0	0	18.9135952	1	-0.0428739
0.2835	6008.37459	0.85185432	240.490477	2.7043313	0	-1.2605432	1.79332068	-0.1591665	5.48268465	0.99522438	-6.9077553	6	67	5.81150556	299.666667	26.66666666	0	0	1	0	18.9135952	1	-0.0428739
0.5256	13476.0787	0.86735881	264.468721	2.77126066	0	-0.6432148	2.60099037	-0.1411503	5.57772677	1.01966311	-6.9077553	7	55	6.25854444	301.888889	28.88888888	0	0	1	0	18.9135952	1	-0.0428739
0.7281	18100.9916	0.78562615	200.973333	2.12239855	0	-0.3173169	2.89602197	-0.2400022	5.30317721	0.75301789	-6.9077553	8	50	5.81150556	303.555556	30.55555555	0	0	1	0	18.9135952	1	-0.0428739
0.9855	27565.8603	0.84592801	226.677229	1.97697289	0	-0.0146062	3.31661434	-0.1661396	5.42353152	0.68207253	-6.9077553	9	45	5.36446667	304.111111	31.11111111	0	0	1	0	18.9135952	1	-0.0428739
1.2834	37025.2147	0.82139555	200.601539	2.00867326	0	0.24951281	3.61162617	-0.1955338	5.30132553	0.69797215	-6.9077553	10	46	3.57631111	304.111111	31.11111111	0	0	1	0	18.9135952	1	-0.0428739
1.611	51135.141	0.91674201	251.996781	1.95284783	0	0.4768551	3.93449151	-0.085839	5.52942028	0.66980067	-6.9077553	11	44	4.02335	304.666667	31.66666666	0	0	1	0	18.9135952	1	-0.0428739
1.8783	61495.8701	0.8395415	204.741688	1.64379862	0	0.63036711	4.11898628	-0.1737089	5.32175401	0.49761796	-6.9077553	12	49	3.12927222	303	30	0	0	1	0	18.9135952	1	-0.0428739
2.2077	74763.6094	0.85009465	215.698002	1.81011763	0	0.79195125	4.31434464	-0.1612319	5.37388393	0.59394413	-6.9077553	13	53	3.12927222	301.333333	28.33333333	0	0	1	0	18.9135952	1	-0.0428739
2.5047	88288.974	0.89653228	228.263589	1.60541696	0	0.91816897	4.48062656	-0.1081062	5.43050543	0.47400621	-6.9077553	14	58	1.78815556	299.111111	26.11111111	0	0	1	0	18.9135952	1	-0.0428739
2.7486	98217.3726	0.79240451	184.248155	1.43905246	0	1.01109169	4.58719329	-0.2314221	5.21628894	0.36467954	-6.9077553	15	51	2.68223333	298.555556	25.55555555	0	0	1	0	18.9135952	1	-0.0428739
3.0942	110479.809	0.76621151	183.703023	1.6101532	0	1.12952939	4.70484183	-0.2649928	5.21332589	0.47695019	-6.9077553	16	53	2.23519444	297.444444	24.44444444	0	0	1	0	18.9135952	1	-0.0428739
3.3408	121338.207	0.8951421	220.996079	1.59237515	0	1.2062103	4.79858999	-0.1096563	5.39814949	0.4658545	-6.9077553	17	62	2.68223333	296.333333	23.33333333	0	0	1	0	18.9135952	1	-0.0428739
3.5388	130937.834	0.88200115	214.114382	1.69796919	0	1.26378769	4.8747303	-0.1244288	5.36651504	0.53002171	-6.9077553	18	73	3.12927222	295.777778	22.7777778	0	0	1	0	18.9135952	1	-0.0428739
3.654	135867.386	0.6901077	128.254272	1.26641321	0	1.29582246	4.91168667	-0.3694596	4.85402259	0.23697798	-6.9077553	19	80	2.23519444	295.222222	22.2222222	0	0	1	0	18.9135952	1	-0.0428739
3.6945	137490.356	0.26934861	17.3343845	0.51916339	0	1.30684523	4.92356105	-1.308043	2.85274976	-0.6536123	-6.9077553	20	84	3.12927222	295.222222	22.2222222	0	0	1	0	18.9135952	1	-0.0428739
3.7287	138937.854	0.2193263	10.4964463	0.40086455	0	1.31605965	4.93403394	-1.5126457	2.35113202	-0.9116402	-6.9077553	21	88	2.23519444	294.666667	21.66666666	0	0	1	0	18.9135952	1	-0.0428739
3.7602	140209.908	0.20886621	9.57923148	0.40531148	0	1.32447215	4.94314778	-1.561285	2.25970175	-0.9006352	-6.9077553	22	90	2.23519444	294.666667	21.66666666	0	0	1	0	18.9135952	1	-0.0428739
3.7935	141701.274	0.24656074	18.4670064	0.6233388	0	1.33328908	4.95372819	-1.3960993	2.91603985	-0.4710621	-6.9077553	23	89	3.12927222	295.222222	22.2222222	0	0	1	0	18.9135952	1	-0.0428739
0.0072	225.089031	0.9509761	247.02002	1.76086344	0	-4.9336743	-1.4868264	-0.0492154	5.50947343	0.56637202	-6.9077553	0	76	3.12927222	297.444444	24.44444444	0	0	0	0	30.519848	1	0.21141735
0.0117	444.407949	0.41279736	39.6307251	0.61892071	0	-4.4481664	-0.8087647	-0.8823789	3.67962994	-0.4781637	-6.9077553	1	79	3.12927222	296.888889	23.88888888	0	0	0	0	30.519848	1	0.21141735
0.0162	619.863074	0.30044537	19.6647278	0.664608	0	-4.122744	-0.4766447	-1.1991665	2.97887741	-0.4070544	-6.9077553	2	82	2.68223333	296.333333	23.33333333	0	0	0	0	30.519848	1	0.21141735
0.0207	839.1821	0.25928321	13.9985321	0.35595965	0	-3.8776216	-0.1741366	-1.345985	2.63902391	-1.0301325	-6.9077553	3	86	2.23519444	295.777778	22.7777778	0	0	0	0	30.519848	1	0.21141735
0.0243	970.773435	0.28469825	16.9949284	0.42743775	0	-3.7172789	-0.0286326	-1.2528191	2.83297381	-0.8476098	-6.9077553	4	86	2.68223333	296.333333	23.33333333	0	0	0	0	30.519848	1	0.21141735
0.0324	1146.22848	0.23813497	12.5742026	0.41307216	0	-3.4295969	0.13734902	-1.4307272	2.53172683	-0.881715	-6.9077553	5	86	1.34111667	296.333333	23.33333333	0	0	0	0	30.519848	1	0.21141735
0.0378	1321.68361	0.19446961	7.90645472	0.40517139	0	-3.2754462	0.27966271	-1.6323504	2.06780595	-0.9009801	-6.9077553	6	86	2.23519444	296.333333	23.33333333	0	0	0	0	30.519848	1	0.21141735
0.0396	1321.68361	0.25962871	13.9156923	0.40144676	0	-3.2289262	0.27966271	-1.3446584	2.63308901	-0.9101925	-6.9077553	7	87	1.34111667	296.333333	23.33333333	0	0	0	0	30.519848	1	0.21141735
0.0405	1321.68361	0.21880442	9.5477457	0.30106032	0	-3.2064533	0.27966271	-1.5150171	2.25640981	-1.1971285	-6.9077553	8	88	0.89407778	295.777778	22.7777778	0	0	0	0	30.519848	1	0.21141735
0.0414	1365.5474	0.18256532	6.44093704	0.19045322	0	-3.1844744	0.31228742	-1.6951847	1.86282928	-1.6531118	-6.9077553	9	88	2.23519444	295.777778	22.7777778	0	0	0	0	30.519848	1	0.21141735
0.0414	1365.5474	0	0	0	0	-3.1844744	0.31228742	-6.9077553	-6.9077553	-6.9077553	-6.9077553	10	88	2.68223333	295.777778	22.7777778	0	0	0	0	30.519848	1	0.21141735
0.0414	1365.5474	0	0	0	0	-3.1844744	0.31228742	-6.9077553	-6.9077553	-6.9077553	-6.9077553	11	86	2.68223333	296.333333	23.33333333	0	0	0	0	30.519848	1	0.21141735
0.0855	1672.59387	0.29085698	20.658943	1.19718404	0	-2.4592389	0.51497333	-1.2314914	3.02819671	0.18080711	-6.9077553	12	82	3.57631111	296.888889	23.88888888	0	0	0	0	30.519848	1	0.21141735
0.1485	2242.39246	0.41280046	50.9392648	1.27506943	0	-1.9071703	0.80798921	-0.8823714	3.93065367	0.24378459	-6.9077553	13	75	3.57631111	298.555556	25.55555555	0	0	0	0	30.519848	1	0.21141735
0.2547	3153.19067	0.55072435	84.3464397	1.97509533	0	-1.3676689	1.14873194	-0.5947067	4.43494446	0.68112284	-6.9077553	14	69	4.47038889	299.111111	26.11111111	0	0	0	0	30.519848	1	0.21141735
0.378	3992.12204	0.6400181	117.21396	2.20069896	0	-0.9728611	1.38457339	-0.4446976	4.76400951	0.78922932	-6.9077553	15	64	4.91742778	300.777778	27.7777778	0	0	0	1	30.519848	1	0.21141735
0.6039	8348.45062	0.84861038	216.554376	2.96621181	0	-0.5043467	2.12219574	-0.1629774	5.37784629	1.08762273	-6.9077553	16	61	4.47038889	301.333333	28.33333333	0	0	0	1	30.519848	1	0.21141735
0.8964	14006.0062	0.79695369	179.548317	2.78771935	0	-0.1093685	2.63955765	-0.2257047	5.19044992	1.02558248	-6.9077553	17	59	4.47038889	301.333333	28.33333333	0	0	0	1	30.519848	1	0.21141735

1.143	23675.5483	0.77398815	174.216184	1.75067361	0	0.13365638	3.16448504	-0.2549075	5.1603027	0.56057168	-6.9077553	18	54	6.25854444	301.888889	28.88888888	0	0	0	1	30.519848	1	0.21141735
1.3536	31715.3058	0.68765109	146.259298	1.41057385	0	0.30276771	3.45683093	-0.3730205	4.9853879	0.34470528	-6.9077553	19	54	4.02335	301.888889	28.88888888	0	0	0	1	30.519848	1	0.21141735
1.4976	38478.4242	0.63096557	123.728259	1.17376957	0	0.40386383	3.65012366	-0.4589204	4.81809579	0.16107202	-6.9077553	20	54	3.12927222	301.888889	28.88888888	0	0	0	1	30.519848	1	0.21141735
1.5939	45383.9004	0.63862517	118.671902	0.90478216	0	0.46618384	3.81517946	-0.4468729	4.77637099	-0.0989564	-6.9077553	21	55	3.12927222	300.777778	27.7777778	0	0	0	1	30.519848	1	0.21141735
1.6596	51544.0901	0.75772701	152.969205	1.03151139	0	0.50657661	3.94245696	-0.2761132	5.03024316	0.03199407	-6.9077553	22	62	2.68223333	299.666667	26.66666666	0	0	0	1	30.519848	1	0.21141735
1.719	56908.5826	0.69267758	125.423207	0.93923944	0	0.54174273	4.04146374	-0.365748	4.83170165	-0.0616207	-6.9077553	23	63	3.12927222	298.555556	25.55555555	0	0	0	1	30.519848	1	0.21141735
0.0135	581.243668	0.96865777	249.803938	1.71873052	0	-4.3050656	-0.5408662	-0.0308121	5.52068036	0.5421676	-6.9077553	0	76	3.12927222	297.444444	24.44444444	0	0	0	0	34.0724638	1	0.49277013
0.0225	932.153922	0.46326308	50.0433197	0.71507807	0	-3.79424	-0.0691851	-0.7673039	3.91290901	-0.3339661	-6.9077553	1	79	3.12927222	296.888889	23.88888888	0	0	0	0	34.0724638	1	0.49277013
0.027	1063.74537	0.3838485	32.8254456	0.54880142	0	-3.6119184	0.06273568	-0.9549055	3.49123446	-0.5981981	-6.9077553	2	82	2.68223333	296.333333	23.33333333	0	0	0	0	34.0724638	1	0.49277013
0.0342	1370.79192	0.20635231	8.93094158	0.35316193	0	-3.3755296	0.31611785	-1.573336	2.18963379	-1.038001	-6.9077553	3	86	2.23519444	295.777778	22.7777778	0	0	0	0	34.0724638	1	0.49277013
0.0396	1590.11075	0.19452977	8.23429298	0.45038887	0	-3.2289262	0.46443236	-1.6320426	2.10842894	-0.7954261	-6.9077553	4	86	2.68223333	296.333333	23.33333333	0	0	0	0	34.0724638	1	0.49277013
0.0432	1677.83818	0.22022839	10.287261	0.33381161	0	-3.1419148	0.51810199	-1.5085597	2.33100354	-1.0941873	-6.9077553	5	86	1.34111667	296.333333	23.33333333	0	0	0	0	34.0724638	1	0.49277013
0.0495	1984.88477	0.24729841	12.6854521	0.36130861	0	-3.0057826	0.68606454	-1.393124	2.54053466	-1.0152589	-6.9077553	6	86	2.23519444	296.333333	23.33333333	0	0	0	0	34.0724638	1	0.49277013
0.0576	2291.93123	0.21232891	9.68595293	0.32887684	0	-2.8542327	0.82983101	-1.5449201	2.27077992	-1.1090359	-6.9077553	7	87	1.34111667	296.333333	23.33333333	0	0	0	0	34.0724638	1	0.49277013
0.0612	2467.38647	0.23626608	11.9819527	0.35022756	0	-2.7936081	0.90356469	-1.4385731	2.48348503	-1.0463209	-6.9077553	8	88	0.89407778	295.777778	22.7777778	0	0	0	0	34.0724638	1	0.49277013
0.0684	2774.4329	0.20482385	8.57018089	0.25959131	0	-2.6823825	1.02080674	-1.5807346	2.14840552	-1.344802	-6.9077553	9	88	2.23519444	295.777778	22.7777778	0	0	0	0	34.0724638	1	0.49277013
0.0738	3037.61541	0.21674653	9.42776299	0.28711339	0	-2.6063965	1.11140195	-1.5244236	2.24376491	-1.2444012	-6.9077553	10	88	2.68223333	295.777778	22.7777778	0	0	0	0	34.0724638	1	0.49277013
0.0801	3300.79804	0.19199613	7.54165213	0.23036911	0	-2.5244794	1.19446718	-1.6450851	2.02057386	-1.463741	-6.9077553	11	86	2.68223333	296.333333	23.33333333	0	0	0	0	34.0724638	1	0.49277013
0.1143	4828.72021	0.70566333	139.941239	1.13331333	0	-2.1689287	1.57478854	-0.3472009	4.94122976	0.12602747	-6.9077553	12	82	3.57631111	296.888889	23.88888888	0	0	0	0	34.0724638	1	0.49277013
0.2196	9326.42973	0.88427741	223.600177	1.88377002	0	-1.5159476	2.23295949	-0.1218542	5.409864	0.63380581	-6.9077553	13	75	3.57631111	298.555556	25.55555555	0	0	0	0	34.0724638	1	0.49277013
0.5094	17548.8137	1.18179606	414.778241	3.4844584	0	-0.6745217	2.86504333	0.16788117	6.02774643	1.24859957	-6.9077553	14	69	4.47038889	299.111111	26.11111111	0	0	0	0	34.0724638	1	0.49277013
1.1853	27735.2758	1.46769087	692.998086	5.28504507	0	0.16999591	3.32274115	0.38437144	6.54102868	1.66507034	-6.9077553	15	64	4.91742778	300.777778	27.7777778	0	0	0	0	34.0724638	1	0.49277013
1.7919	42752.964	1.13468766	381.829609	3.12612363	0	0.58327651	3.75546192	0.12723833	5.94497708	1.14011361	-6.9077553	16	61	4.47038889	301.333333	28.33333333	0	0	0	1	34.0724638	1	0.49277013
2.3499	59131.4274	1.00071549	284.64753	2.54768007	0	0.85437277	4.07977946	0.00171402	5.65125519	0.93557561	-6.9077553	17	59	4.47038889	301.333333	28.33333333	0	0	0	1	34.0724638	1	0.49277013
2.8575	77353.2382	0.97989974	277.33832	2.31249913	0	1.04994712	4.34839537	-0.019285	5.62524174	0.83876116	-6.9077553	18	54	6.25854444	301.888889	28.88888888	0	0	0	1	34.0724638	1	0.49277013
3.6081	100038.847	0.94395026	249.48638	2.66693832	0	1.28318132	4.60556857	-0.056623	5.51940833	0.98130601	-6.9077553	19	54	4.02335	301.888889	28.88888888	0	0	0	1	34.0724638	1	0.49277013
4.2813	116716.082	0.79488271	182.436268	2.16532253	0	1.4542567	4.7597529	-0.2283035	5.20640637	0.77303104	-6.9077553	20	54	3.12927222	301.888889	28.88888888	0	0	0	1	34.0724638	1	0.49277013
4.7016	130756.654	0.79590595	190.978068	1.6093188	0	1.54790288	4.87334564	-0.2270186	5.25216383	0.47643217	-6.9077553	21	55	3.12927222	300.777778	27.7777778	0	0	0	1	34.0724638	1	0.49277013
4.9716	142403.787	0.8306192	189.422135	1.36582153	0	1.60374172	4.95867362	-0.1843806	5.24398332	0.312488	-6.9077553	22	62	2.68223333	299.666667	26.66666666	0	0	0	1	34.0724638	1	0.49277013
5.2461	154628.053	0.8916664	214.803983	1.62030079	0	1.65748494	5.04102904	-0.1135423	5.36973056	0.48322879	-6.9077553	23	63	3.12927222	298.555556	25.55555555	0	0	0	1	34.0724638	1	0.49277013
0.0252	1376.05496	0.99385805	267.233362	1.8674055	0	-3.6809113	0.31994713	-0.0051552	5.58812603	0.62508539	-6.9077553	0	76	3.12927222	297.444444	24.44444444	0	0	0	0	42.288408	1	-0.4111751
0.0585	2999.66065	0.60010193	91.3308765	1.09897201	0	-2.8387285	1.09883248	-0.5089908	4.51449987	0.09528474	-6.9077553	1	79	3.12927222	296.888889	23.88888888	0	0	0	0	42.288408	1	-0.4111751
0.0801	4052.39158	0.404682	37.8613332	0.64394454	0	-2.5244794	1.39955396	-0.9021857	3.63395677	-0.4385909	-6.9077553	2	82	2.68223333	296.333333	23.33333333	0	0	0	0	42.288408	1	-0.4111751
0.1062	5236.71347	0.26744997	15.6777323	0.49843032	0	-2.2424312	1.65588504	-1.3150907	2.75230516	-0.6942872	-6.9077553	3	86	2.23519444	295.777778	22.7777778	0	0	0	0	42.288408	1	-0.4111751
0.1341	6596.49029	0.29028779	18.7177222	0.50574776	0	-2.0091695	1.88668932	-1.2334435	2.92952421	-0.6797419	-6.9077553	4	86	2.68223333	296.333333	23.33333333	0	0	0	0	42.288408	1	-0.4111751
0.162	7912.40411	0.28557544	17.6056105	0.4861046	0	-1.8201589	2.06855804	-1.2497535	2.86827443	-0.7192764	-6.9077553	5	86	1.34111667	296.333333	23.33333333	0	0	0	0	42.288408	1	-0.4111751
0.1863	9096.7266	0.25802045	14.3341291	0.44127295	0	-1.680397	2.20802456	-1.3508482	2.6627131	-0.8158281	-6.9077553	6	86	2.23519444	296.333333	23.33333333	0	0	0	0	42.288408	1	-0.4111751
0.2106	10281.0495	0.26054246	14.6759429	0.4593698	0	-1.5577947	2.33039961	-1.3411586	2.68627775	-0.7757252	-6.9077553	7	87	1.34111667	296.333333	23.33333333	0	0	0	0	42.288408	1	-0.4111751
0.2331	11377.6435	0.25297247	13.6614783	0.41785824	0	-1.4562877	2.43173822	-1.3705294	2.61465326	-0.8702227	-6.9077553	8	88	0.89407778	295.777778	22.7777778	0	0	0	0	42.288408	1	-0.4111751
0.2601	12649.6926	0.24191705	12.6423177	0.44250898	0	-1.3466891	2.53771196	-1.4150352	2.53712883	-0.8130372	-6.9077553	9	88	2.23519444	295.777778	22.7777778	0	0	0	0	42.288408	1	-0.4111751
0.2961	14407.5224	0.37051537	41.9030273	0.66277518	0	-1.217058	2.66781987	-0.990165	3.73538194	-0.4098118	-6.9077553	10	88	2.68223333	295.777778	22.7777778	0	0	0	0	42.288408	1	-0.4111751
0.3951	19032.5761	0.83216213	194.975923	1.67679624	0	-0.9286164	2.94620458	-0.182527	5.27288121	0.51748117	-6.9077553	11	86	2.68223333	296.333333	23.33333333	0	0	0	0	42.288408	1	-0.4111751
0.6084	26634.7084	0.72687811	159.49227	1.81838345	0	-0.4969227	3.28225274	-0.3176217	5.07200173	0.59849768	-6.9077553	12	82	3.57631111	296.888889	23.88888888	0	0	1	0	42.288408	1	-0.4111751
0.8802	37052.2875	0.82055982	187.942156	1.68454161	0	-0.1276061	3.61235708	-0.1965505	5.23613956	0.52208694	-6.9077553	13	75	3.57631111	298.555556	25.55555555	0	0	1	0	42.288408	1	-0.4111751
1.2393	48615.544	0.81354326	173.287457	1.86881419	0	0.2145467	3.88396388	-0.2051277	5.15495759	0.62583906	-6.9077553	14	69	4.47038889	299.111111	26.11111111	0	0	1	0	42.288408	1	-0.4111751
1.6326	62670.7908	0.82122782	177.508259	1.78053182	0	0.49017384	4.13791144	-0.1957378	5.17902277	0.57747357	-6.9077553	15	64	4.91742778	300.777778	27.7777778	0	0	1	0	42.288408	1	-0.4111751
2.0781	79899.0794	0.84368921	191.075379	1.90785664	0	0.73145401	4.38077685	-0.1687865	5.25267324	0.64650445	-6.9077553	16	61	4.47038889	301.333333	28.33333333	0	0	1	0	42.288408	1	-0.4111751
2.6874	101722.234	0.88052391	214.134255	2.19961706	0	0.98857418	4.62225574	-0.1261032	5.36660785	0.7887378	-6.9077553	17	59	4.47038889	301.333333	28.33333333	0	0	1	0	42.288408	1	-0.4111751
3.4335	134697.455	0.97673684	276.117894	2.63443461	0	1.23358015	4.90303861	-0.0225147	5.62083155	0.9690481	-6.9077553	18	54	6.25854444	301.888889	28.88888888	0	0	1	0	42.288408	1	-0.4111751
4.2156	171210.524	0.96179466	261.566132	2.04440915	0	1.43879193	5.14289978	-0.0379151	5.56669097	0.71559784	-6.9077553	19	54	4.02335	301.888889	28.88888888	0	0	1	0	42.288408	1	-0.4111751
4.9095	203457.346	0.93314299	249.911783	2.00171896	0	1.5911721	5.3154613	-0.0681258	5.52111199	0.69450574	-6.9077553	20	54	3.12927222	301.888889	28.88888888	0	0	1	0	42.288408	1	-0.4111751
5.4261	226179.913	0.86569225	217.592865	1.66089612	0	1.69122064	5.42133518	-0.1430713	5.38263032	0.50795919	-6.9077553	21	55	3.12927222	300.777778	27.7777778	0	0	1	0	42.288408	1	-0.4111751
5.7339	238808.47	0.83441001	189.287578	1.35873484	0	1.74639593	5.47566604	-0.1798326	5.24327272	0.30728971	-6.9077553	22	62	2.68223333	299.666667	26.66666666	0	0	1	0	42.288408	1	-0.4111751
5.9742	249586.331	0.81273945	180.809545	1.38825492	0	1.7874502	5.51980888	-0.206115	5.19744977	0.32876757	-6.9077553	23	63	3.12927222	298.555556	25.55555555	0	0	1	0	42.288408	1	-0.4111751
0.0009	0	0	0	0	0	-7.0131158	-6.9077553	-6.9077553	-6.9077553	-6.9077553	-6.9077553	0	76	3.12927222	297.444444	24.44444444	0	0	0	0	18.9135952	1	-0.0428739
0.0009	0	0	0	0	0	-7.0131158	-6.9077553	-6.9077553	-6.9077553	-6.9077553	-6.9077553	1	79	3.12927222	296.888889	23.88888888	0	0	0	0	18.9135952	1	-0.0428739
0.0009	0	0	0	0	0	-7.0131158	-6.9077553	-6.9077553	-6.9077553	-6.9077553	-6.9077553	2	82	2.68223333	296.333333	23.33333333	0	0	0	0	18.9135952	1	-0.0428739
0.0009	0	0	0	0	0	-7.0131158	-6.9077553	-6.9077553	-6.9077553	-6.9077553	-6.9077553	3	86	2.23519444	295.777778	22.7777778	0	0	0	0	18.9135952	1	-0.0428739
0.0009	0	0	0	0	0	-7.0131158	-6.9077553	-6.9077553	-6.9077553	-6.9077553	-6.9077553	4	86	2.68223333	296.333333	23.33333333	0	0	0	0	18.9135952	1	-0.0428739
0.0009	0	0	0	0	0	-7.0131158	-6.9077553	-6.9077553	-6.9077553	-6.9077553	-6.9077553	5	86	1.34111667	296.333333	23.33333333	0	0	0	0	18.9135952	1	-0.0428739
0.0009	0	0	0	0	0	-7.0131158	-6.9077553	-6.9077553	-6.9077553	-6.9077553	-6.9077553	6	86	2.23519444	296.333333	23.33333333	0	0	0	0	18.9135952	1	-0.0428739
0.0009	0	0	0	0	0	-7.0131158	-6.9077553	-6.9077553	-6.9077553	-6.9077553	-6.9077553	7	87	1.34111667	296.333333	23.33333333	0	0	0	0	18.9135952	1	-0.0428739

0.0009	0	0	0	0	0	-7.0131158	-6.9077553	-6.9077553	-6.9077553	-6.9077553	-6.9077553	8	88	0.89407778	295.777778	22.7777778	0	0	0	0	18.9135952	1	-0.0428739
0.0009	0	0	0	0	0	-7.0131158	-6.9077553	-6.9077553	-6.9077553	-6.9077553	-6.9077553	9	88	2.23519444	295.777778	22.7777778	0	0	0	0	18.9135952	1	-0.0428739
0.0009	0	0	0	0	0	-7.0131158	-6.9077553	-6.9077553	-6.9077553	-6.9077553	-6.9077553	10	88	2.68223333	295.777778	22.7777778	0	0	0	0	18.9135952	1	-0.0428739
0.0009	0	0	0	0	0	-7.0131158	-6.9077553	-6.9077553	-6.9077553	-6.9077553	-6.9077553	11	86	2.68223333	296.333333	23.33333333	0	0	0	0	18.9135952	1	-0.0428739
0.0009	0	0	0	0	0	-7.0131158	-6.9077553	-6.9077553	-6.9077553	-6.9077553	-6.9077553	12	82	3.57631111	296.888889	23.88888888	0	0	0	0	18.9135952	1	-0.0428739
0.0261	0	0.60977772	112.668117	2.88861166	0	-3.64582	-6.9077553	-0.4930222	4.72445535	1.06112212	-6.9077553	13	75	3.57631111	298.555556	25.55555555	0	0	0	0	18.9135952	1	-0.0428739
0.0927	505.956897	0.6733449	132.342826	2.4304659	0	-2.3783868	-0.6793293	-0.3940136	4.88540328	0.88849433	-6.9077553	14	69	4.47038889	299.111111	26.11111111	0	0	1	0	18.9135952	1	-0.0428739
0.2178	2031.57007	0.62631511	105.730139	1.94894343	0	-1.5241781	0.70930104	-0.4663063	4.66089944	0.66780036	-6.9077553	15	64	4.91742778	300.777778	27.7777778	0	0	1	0	18.9135952	1	-0.0428739
0.414	8125.21954	0.78640278	205.093714	1.99478065	0	-0.8818893	2.09509581	-0.2390154	5.32347189	0.69103528	-6.9077553	16	61	4.47038889	301.333333	28.33333333	0	0	1	0	18.9135952	1	-0.0428739
0.7065	19510.3401	0.89910889	259.971875	2.38695463	0	-0.3474321	2.97099584	-0.1052395	5.5605773	0.8704372	-6.9077553	17	59	4.47038889	301.333333	28.33333333	0	0	1	0	18.9135952	1	-0.0428739
1.1088	32540.1206	0.99304588	320.698406	3.49517781	0	0.10327835	3.48250454	-0.0059719	5.77050425	1.25167032	-6.9077553	18	54	6.25854444	301.888889	28.88888888	0	0	1	0	18.9135952	1	-0.0428739
1.44	44368.677	0.86073237	216.627972	1.94314111	0	0.36464311	3.79255628	-0.1488105	5.37818608	0.66482029	-6.9077553	19	54	4.02335	301.888889	28.88888888	0	0	1	0	18.9135952	1	-0.0428739
1.8099	59887.709	0.88819948	226.966393	1.95316064	0	0.5932716	4.09248799	-0.1174337	5.42480636	0.66996076	-6.9077553	20	54	3.12927222	301.888889	28.88888888	0	0	1	0	18.9135952	1	-0.0428739
2.1735	76687.4045	0.8942896	228.024946	1.70862678	0	0.77633877	4.33975052	-0.110608	5.42945942	0.53627509	-6.9077553	21	55	3.12927222	300.777778	27.7777778	0	0	1	0	18.9135952	1	-0.0428739
2.4417	89958.4941	0.91475806	229.568818	1.61873314	0	0.89269452	4.4993595	-0.0880031	5.4362072	0.48226141	-6.9077553	22	62	2.68223333	299.666667	26.66666666	0	0	1	0	18.9135952	1	-0.0428739
2.7036	103475.808	0.93069773	239.631497	1.83208285	0	0.99458422	4.63934751	-0.0707468	5.47910649	0.60599917	-6.9077553	23	63	3.12927222	298.555556	25.55555555	0	0	1	0	18.9135952	1	-0.0428739
0.0135	405.913009	0.73587445	164.059344	1.4021537	0	-4.3050656	-0.8991559	-0.3053378	5.10023431	0.33872234	-6.9077553	0	58	2.68223333	305.777778	32.7777778	0	0	0	0	30.519848	1	0.21141735
0.0333	1193.01289	0.90635716	222.109686	1.57342408	0	-3.4021979	0.17731981	-0.0972191	5.40317584	0.45388954	-6.9077553	1	65	1.78815556	304.111111	31.11111111	0	0	0	0	30.519848	1	0.21141735
0.0432	1456.30939	0.69909893	133.435736	1.52033875	0	-3.1419148	0.37659185	-0.3565336	4.89362747	0.41959071	-6.9077553	2	70	1.78815556	303	30	0	0	0	0	30.519848	1	0.21141735
0.0441	1500.1731	0.59729022	84.7243042	1.03055334	0	-3.1212955	0.40624687	-0.5136793	4.43941431	0.03106576	-6.9077553	3	77	0.44703889	301.888889	28.88888888	0	0	0	0	30.519848	1	0.21141735
0.0441	1500.1731	0	0	0	0	-3.1212955	0.40624687	-6.9077553	-6.9077553	-6.9077553	-6.9077553	4	75	0.89407778	297.444444	24.44444444	0	0	0	0	30.519848	1	0.21141735
0.0441	1500.1731	0	0	0	0	-3.1212955	0.40624687	-6.9077553	-6.9077553	-6.9077553	-6.9077553	5	88	0.89407778	295.222222	22.2222222	0	0	0	0	30.519848	1	0.21141735
0.0441	1500.1731	0	0	0	0	-3.1212955	0.40624687	-6.9077553	-6.9077553	-6.9077553	-6.9077553	6	46	2.23519444	296.888889	23.88888888	0	0	0	0	30.519848	1	0.21141735
0.0891	1763.35575	0.33974792	37.835293	1.14640816	0	-2.4179959	0.56778561	-1.0766123	3.63326877	0.13750562	-6.9077553	7	42	2.23519444	296.333333	23.33333333	0	0	0	0	30.519848	1	0.21141735
0.153	2465.17629	0.39423883	45.083613	1.18236907	0	-1.8773174	0.90266889	-0.9282651	3.80854101	0.16836552	-6.9077553	8	35	3.12927222	295.777778	22.7777778	0	0	0	0	30.519848	1	0.21141735
0.3456	3815.84589	0.68750955	128.811752	1.12672952	0	-1.0624732	1.3394244	-0.3732261	4.85835981	1.14030735	-6.9077553	9	36	4.91742778	294.666667	21.66666666	0	0	0	0	30.519848	1	0.21141735
0.4896	5401.823	0.52510066	75.5046082	1.33080587	0	-0.7141665	1.6869216	-0.6422627	4.32420693	0.28653582	-6.9077553	10	43	2.68223333	293	20	0	0	0	1	30.519848	1	0.21141735
0.594	7206.47212	0.48388573	69.2640507	0.78624824	0	-0.520876	1.97511828	-0.723842	4.23794046	-0.2392117	-6.9077553	11	54	0.44703889	290.222222	17.2222222	0	0	0	1	30.519848	1	0.21141735
0.7119	8771.20757	0.50397334	72.2720062	0.94853292	0	-0.3398178	2.17158849	-0.6832496	4.2804507	-0.0517851	-6.9077553	12	55	1.34111667	291.333333	18.33333333	0	0	0	1	30.519848	1	0.21141735
1.0215	11743.1001	0.62020368	111.31979	1.86148834	0	0.02127214	2.463351	-0.4760963	4.71241603	0.62191341	-6.9077553	13	46	3.12927222	294.111111	21.11111111	0	0	0	1	30.519848	1	0.21141735
1.7865	19187.2287	0.99687313	304.873824	4.1477292	0	0.5802584	2.954297	-0.0021291	5.71990128	1.42280207	-6.9077553	14	40	5.36446667	295.777778	22.7777778	0	0	0	1	30.519848	1	0.21141735
2.7378	29099.2199	1.00951929	306.205682	4.57641301	0	1.00715468	3.37074573	0.01046435	5.72426031	1.52113399	-6.9077553	15	35	6.25854444	297.444444	24.44444444	0	0	0	1	30.519848	1	0.21141735
3.618	47331.4133	0.93302222	250.911235	2.31106112	0	1.28592139	3.85719533	-0.0682551	5.52510322	0.83813939	-6.9077553	16	31	6.70558333	299.111111	26.11111111	0	0	0	1	30.519848	1	0.21141735
4.5594	63732.3282	0.98218567	272.106836	2.09814196	0	1.51719104	4.15470763	-0.0169573	5.60619844	0.74152867	-6.9077553	17	27	6.25854444	300.777778	27.7777778	0	0	0	1	30.519848	1	0.21141735
5.7744	107206.354	1.12054688	379.739669	3.20850369	24	1.75343435	4.67476485	0.11470887	5.93963872	1.16611631	3.1780955	18	24	7.59966111	301.888889	28.88888888	0	0	0	1	30.519848	1	0.21141735
6.7869	267242.687	1.34770356	488.431944	2.65066319	155	1.91499428	5.58816093	0.2991438	6.19120219	0.97518706	5.04343157	19	23	5.36446667	303.555556	30.55555555	0	0	0	1	30.519848	1	0.21141735
8.1306	647820.676	2.09413085	991.740623	4.02438823	514	2.09563472	6.47361547	0.73961601	6.89946261	1.39262136	6.24222521	20	23	5.81150556	303.555556	30.55555555	0	0	0	1	30.519848	1	0.21141735
9.9018	1272050.64	2.31607031	1119.62424	4.37550416	1109	2.29271656	7.14838634	0.84030359	7.0207493	1.47625027	7.01121489	21	22	6.25854444	304.111111	31.11111111	0	0	0	1	30.519848	1	0.21141735
11.9853	1987901.63	2.63241049	1386.49773	4.55807699	1842	2.4836809	7.59483541	0.96827977	7.23453695	1.51712019	7.51860776	22	23	5.36446667	304.111111	31.11111111	0	0	0	1	30.519848	1	0.21141735
13.4514	2269316.98	1.7834672	823.220918	3.9261925	2109	2.59908319	7.72723462	0.57911988	6.71322581	1.36792479	7.65396965	23	24	4.91742778	303.555556	30.55555555	0	0	0	1	30.519848	1	0.21141735
0.0351	1304.83236	0.97841855	272.076455	2.61131487	0	-3.3495541	0.26684066	-0.0207962	5.60608679	0.96023675	-6.9077553	0	58	2.68223333	305.777778	32.7777778	0	0	0	0	34.0724638	1	0.49277013
0.0882	3621.50722	0.84407043	193.157591	1.32504557	0	-2.4281483	1.28716639	-0.1683353	5.26351156	0.28220126	-6.9077553	1	65	1.78815556	304.111111	31.11111111	0	0	0	0	34.0724638	1	0.49277013
0.1521	6609.71318	0.7565817	145.464706	1.05146661	0	-1.8832171	1.88869154	-0.2776239	4.97994036	0.05113656	-6.9077553	2	70	1.78815556	303	30	0	0	0	0	34.0724638	1	0.49277013
0.1953	8364.26469	0.64807947	103.648631	0.85381222	0	-1.6332184	2.12408798	-0.4322001	4.64101628	-0.1568735	-6.9077553	3	77	0.44703889	301.888889	28.88888888	0	0	0	0	34.0724638	1	0.49277013
0.2241	9636.31452	0.52296758	64.5176392	0.68596601	0	-1.4956629	2.26564249	-0.6463255	4.16695416	-0.3754705	-6.9077553	4	75	0.89407778	297.444444	24.44444444	0	0	0	0	34.0724638	1	0.49277013
0.2448	10250.4074	0.44102636	46.2090746	0.59086825	0	-1.4073137	2.327415	-0.8163858	3.83319784	-0.5244712	-6.9077553	5	88	0.89407778	295.222222	22.2222222	0	0	0	0	34.0724638	1	0.49277013
0.2664	11039.9549	0.30070571	26.8159205	0.51622951	0	-1.3227563	2.40161153	-1.1983032	3.28903305	-0.6592686	-6.9077553	6	46	2.23519444	296.888889	23.88888888	0	0	0	0	34.0724638	1	0.49277013
0.3717	14813.5799	0.83080742	202.484375	1.65751257	0	-0.9896682	2.69561183	-0.1841543	5.31066766	0.50592116	-6.9077553	7	42	2.23519444	296.333333	23.33333333	0	0	0	1	34.0724638	1	0.49277013
0.5121	20697.5571	0.88738388	218.155749	1.64298796	0	-0.6692354	3.03006399	-0.1183513	5.38521384	0.49712497	-6.9077553	8	35	3.12927222	295.777778	22.7777778	0	0	0	1	34.0724638	1	0.49277013
0.774	29525.9903	0.95289869	259.226106	2.25354885	0	-0.2561834	3.38530477	-0.0471978	5.55770454	0.81294989	-6.9077553	9	36	4.91742778	294.666667	21.66666666	0	0	0	1	34.0724638	1	0.49277013
1.0332	41032.9089	0.9406431	249.094293	1.85213958	0	0.03266078	3.71439877	-0.060129	5.51783552	0.61688127	-6.9077553	10	43	2.68223333	293	20	0	0	0	1	34.0724638	1	0.49277013
1.2726	49954.3232	0.85229194	209.637453	1.56832037	0	0.24106205	3.91112907	-0.1586535	5.34538439	0.45064264	-6.9077553	11	54	0.44703889	290.222222	17.2222222	0	0	0	1	34.0724638	1	0.49277013
1.467	55622.8952	0.69049694	138.300239	1.14762243	0	0.3832195	4.01861288	-0.3688966	4.9294342	0.13856333	-6.9077553	12	55	1.34111667	291.333333	18.33333333	0	0	0	1	34.0724638	1	0.49277013
1.8387	67739.5481	0.88616397	221.415727	1.85986033	0	0.6090588	4.21568494	-0.1197255	5.40004657	0.62103892	-6.9077553	13	46	3.12927222	294.111111	21.11111111	0	0	0	1	34.0724638	1	0.49277013
2.4417	91478.8852	1.02363864	296.987873	2.6105671	0	0.89269452	4.51611911	0.02434001	5.69369467	0.95995046	-6.9077553	14	40	5.36446667	295.777778	22.7777778	0	0	0	1	34.0724638	1	0.49277013
3.3318	122652.333	1.06305493	317.94344	3.00882658	0	1.2035127	4.80936195	0.06208702	5.76187665	1.10188246	-6.9077553	15	35	6.25854444	297.444444	24.44444444	0	0	0	1	34.0724638	1	0.49277013
4.5036	164958.848	1.06476308	327.28749	3.11242207	0	1.50487708	5.1057021	0.06369105	5.79084202	1.13572247	-6.9077553	16	31	6.70558333	299.111111	26.11111111	0	0	0	1	34.0724638	1	0.49277013
5.7681	207646.071	1.05530645	318.738901	3.14238073	0	1.75234274	5.33583987	0.05477834	5.76437541	1.14529889	-6.9077553	17	27	6.25854444	300.777778	27.7777778	0	0	0	1	34.0724638	1	0.49277013
7.0659	265555.603	1.15094578	397.553571	3.27552573	1	1.9552804	5.58182801	0.1414525	5.98533221	1.18678363	0.0009995	18	24	7.59966111	301.888889	28.88888888	0	0	0	1	34.0724638	1	0.49277013
8.496	315315.297	1.08291824	348.118156	3.57913168	1	2.13959546	5.75357625	0.08058247	5.85254482	1.27539958	0.0009995	19	23	5.36446667	303.555556	30.55555555	0	0	0	1	34.0724638	1	0.49277013
10.0971	368096.227	0.96370655	285.706805	3.35919522	1	2.31224825	5.90834711	-0.0359313	5.65496963	1.21199907	0.0009995	20	23	5.81150556	303.555556	30.55555555	0	0	0	1	34.0724638	1	0.49277013
11.691	415994.501	0.95885493	292.046196	3.51530463	1	2.45881932	6.03067444	-0.0409731	5.67691542	1.25741062	0.0009995	21	22	6.25854444	304.111111	31.11111111	0	0	0	1	34.0724638	1	0.49277013

13.1859	455171.371	0.89762887	255.83248	3.20975575	1	2.57914808	6.12067618	-0.1068852	5.54452677	1.16650634	0.0009995	22	23	5.36446667	304.111111	31.1111111	0	0	0	1	34.0724638	1	0.49277013
14.7483	507017.711	0.95233773	282.641381	3.15101195	2	2.69112782	6.22854791	-0.0477861	5.64418243	1.14804096	0.69364706	23	24	4.91742778	303.555556	30.5555556	0	0	0	1	34.0724638	1	0.49277013
0.0405	2863.92042	1.23404494	423.64138	2.67524234	0	-3.2064533	1.05254057	0.21110736	6.04888966	0.9844137	-6.9077553	0	58	2.68223333	305.777778	32.7777778	0	0	0	0	42.288408	1	-0.4111751
0.126	7712.91099	0.98620067	270.814947	1.9204797	0	-2.0714734	2.04302532	-0.0128819	5.60143943	0.65309557	-6.9077553	1	65	1.78815556	304.111111	31.1111111	0	0	0	0	42.288408	1	-0.4111751
0.2205	12709.1105	0.94986914	242.257496	1.80819179	0	-1.5118576	2.54239778	-0.0503788	5.49000532	0.59288022	-6.9077553	2	70	1.78815556	303	30	0	0	0	0	42.288408	1	-0.4111751
0.3204	17502.6786	0.87128888	201.433347	1.71661487	0	-1.1381851	2.86241106	-0.1366346	5.30546351	0.54093662	-6.9077553	3	77	0.44703889	301.888889	28.8888889	0	0	0	0	42.288408	1	-0.4111751
0.3942	20967.919	0.68023561	117.138934	1.28216227	0	-0.9308969	3.04304129	-0.3838471	4.76336924	0.24932756	-6.9077553	4	75	0.89407778	297.444444	24.4444444	0	0	0	0	42.288408	1	-0.4111751
0.4617	24126.1117	0.56705205	81.7321549	0.9940033	0	-0.7728399	3.18333617	-0.5655422	4.40345973	-0.0050092	-6.9077553	5	88	0.89407778	295.222222	22.2222222	0	0	1	0	42.288408	1	-0.4111751
0.5373	27656.5357	0.54998875	102.969285	1.00054023	0	-0.6211987	3.31989823	-0.5960409	4.63444045	0.00153905	-6.9077553	6	46	2.23519444	296.888889	23.8888889	0	0	1	0	42.288408	1	-0.4111751
0.8721	41063.52	0.8689147	228.8771	1.84485175	0	-0.1368512	3.71514449	-0.1393601	5.43318955	0.61294082	-6.9077553	7	42	2.23519444	296.333333	23.3333333	0	0	1	0	42.288408	1	-0.4111751
1.2231	52877.3288	0.81882465	188.317268	1.83496062	0	0.20138862	3.96799359	-0.1986648	5.23813344	0.60756785	-6.9077553	8	35	3.12927222	295.777778	22.7777778	0	0	1	0	42.288408	1	-0.4111751
1.6074	65820.1013	0.79241507	166.304267	1.89375795	0	0.47461797	4.18694048	-0.2314088	5.11382506	0.6390911	-6.9077553	9	36	4.91742778	294.666667	21.6666666	0	0	1	0	42.288408	1	-0.4111751
2.0043	80997.2936	0.84069907	197.117382	1.72260006	0	0.69529487	4.39442809	-0.1723327	5.28380447	0.54441516	-6.9077553	10	43	2.68223333	293	20	0	0	1	0	42.288408	1	-0.4111751
2.3157	93266.8829	0.75261322	157.406668	1.24134148	0	0.83971202	4.53547581	-0.282876	5.05883905	0.21699789	-6.9077553	11	54	0.44703889	290.222222	17.2222222	0	0	1	0	42.288408	1	-0.4111751
2.7009	109453.249	0.81765662	186.801749	1.38217962	0	0.99358505	4.69550664	-0.2000906	5.23005324	0.32438492	-6.9077553	12	55	1.34111667	291.333333	18.3333333	0	0	1	0	42.288408	1	-0.4111751
3.3678	133071.016	0.85558703	206.830634	2.04145218	0	1.21425971	4.89089045	-0.1547994	5.3319051	0.71415113	-6.9077553	13	46	3.12927222	294.111111	21.1111111	0	0	1	0	42.288408	1	-0.4111751
4.5612	158811.433	0.8078732	196.433494	3.21707526	0	1.51758575	5.06772384	-0.2121131	5.28032901	1.16878344	-6.9077553	14	40	5.36446667	295.777778	22.7777778	0	0	1	0	42.288408	1	-0.4111751
6.0876	188339.802	0.85000481	219.318765	4.0911491	0	1.80625392	5.2382531	-0.1613375	5.39053078	1.40907028	-6.9077553	15	35	6.25854444	297.444444	24.4444444	0	0	1	0	42.288408	1	-0.4111751
7.8534	241730.507	1.03299868	337.833142	4.45679831	0	2.06094656	5.48782764	0.0334335	5.82255507	1.49465499	-6.9077553	16	31	6.70558333	299.111111	26.1111111	0	0	1	0	42.288408	1	-0.4111751
9.3168	312262.483	1.19449269	437.678083	4.20274543	0	2.23181922	5.74384733	0.17855839	6.08148595	1.4359759	-6.9077553	17	27	6.25854444	300.777778	27.7777778	0	0	1	0	42.288408	1	-0.4111751
10.7982	388043.413	1.20626397	443.668666	3.85527296	0	2.37937945	5.9611198	0.18835662	6.09508029	1.34970116	-6.9077553	18	24	7.59966111	301.888889	28.8888889	0	0	1	0	42.288408	1	-0.4111751
12.3246	467267.552	1.21145472	435.469782	3.48250594	1	2.51159727	6.14690415	0.19264699	6.0764277	1.24803924	0.0009995	19	23	5.36446667	303.555556	30.5555556	0	0	1	0	42.288408	1	-0.4111751
14.0499	550406.806	1.20325618	435.115089	3.90704062	3	2.64261528	6.31065947	0.1858621	6.07561287	1.36303613	1.09894557	20	23	5.81150556	303.555556	30.5555556	0	0	1	0	42.288408	1	-0.4111751
15.5592	623101.111	1.14186004	393.845483	3.31183207	3	2.7446521	6.43471041	0.13353393	5.9759612	1.19780343	1.09894557	21	22	6.25854444	304.111111	31.1111111	0	0	1	0	42.288408	1	-0.4111751
17.0145	681340.084	1.03156193	314.433615	2.88187519	4	2.83406592	6.52406304	0.03204303	5.75077615	1.05878813	1.38654433	22	23	5.36446667	304.111111	31.1111111	0	0	1	0	42.288408	1	-0.4111751
18.4392	734286.73	1.02353759	314.061118	2.98441016	4	2.91447883	6.59890095	0.02424138	5.74959079	1.09373715	1.38654433	23	24	4.91742778	303.555556	30.5555556	0	0	1	0	42.288408	1	-0.4111751
0.0027	0	0.21041781	8.77035046	0.69165194	0	-5.9145035	-6.9077553	-1.553919	2.17149078	-0.3672277	-6.9077553	0	58	2.68223333	305.777778	32.7777778	0	0	0	0	18.9135952	1	-0.0428739
0.0036	0	0.06053963	0.5845502	0.09518638	0	-5.6268214	-6.9077553	-2.788074	-0.5352034	-2.3414676	-6.9077553	1	65	1.78815556	304.111111	31.1111111	0	0	0	0	18.9135952	1	-0.0428739
0.0036	0	0	0	0	0	-5.6268214	-6.9077553	-6.9077553	-6.9077553	-6.9077553	-6.9077553	2	70	1.78815556	303	30	0	0	0	0	18.9135952	1	-0.0428739
0.0036	0	0	0	0	0	-5.6268214	-6.9077553	-6.9077553	-6.9077553	-6.9077553	-6.9077553	3	77	0.44703889	301.888889	28.8888889	0	0	0	0	18.9135952	1	-0.0428739
0.0036	0	0	0	0	0	-5.6268214	-6.9077553	-6.9077553	-6.9077553	-6.9077553	-6.9077553	4	75	0.89407778	297.444444	24.4444444	0	0	0	0	18.9135952	1	-0.0428739
0.0036	0	0	0	0	0	-5.6268214	-6.9077553	-6.9077553	-6.9077553	-6.9077553	-6.9077553	5	88	0.89407778	295.222222	22.2222222	0	0	0	0	18.9135952	1	-0.0428739
0.0036	0	0	0	0	0	-5.6268214	-6.9077553	-6.9077553	-6.9077553	-6.9077553	-6.9077553	6	46	2.23519444	296.888889	23.8888889	0	0	0	0	18.9135952	1	-0.0428739
0.0396	0	0.41784134	45.2463715	1.57748966	0	-3.2289262	-6.9077553	-0.8702631	3.81214458	0.45646848	-6.9077553	7	42	2.23519444	296.333333	23.3333333	0	0	0	0	18.9135952	1	-0.0428739
0.1179	0	0.40626833	41.056433	1.66732876	0	-2.1379185	-6.9077553	-0.898283	3.71497189	0.51182238	-6.9077553	8	35	3.12927222	295.777778	22.7777778	0	0	0	0	18.9135952	1	-0.0428739
0.2484	175.455131	0.37389316	35.2373653	1.59876099	0	-1.3927149	-1.7346886	-0.9811142	3.56213541	0.46985423	-6.9077553	9	36	4.91742778	294.666667	21.6666666	0	0	1	0	18.9135952	1	-0.0428739
0.3411	745.684337	0.39291845	52.9767853	1.1290801	0	-1.0755796	-0.2921128	-0.9316114	3.96987268	0.12228851	-6.9077553	10	43	2.68223333	293	20	0	0	1	0	18.9135952	1	-0.0428739
0.4077	1831.41779	0.45117481	77.4083879	0.8129091	0	-0.8972237	0.60563629	-0.7936864	4.34910806	-0.2059066	-6.9077553	11	54	0.44703889	290.222222	17.2222222	0	0	1	0	18.9135952	1	-0.0428739
0.4941	4275.9398	0.61794285	128.343465	1.1212546	0	-0.7050174	1.45323775	-0.4797423	4.85471778	0.1153397	-6.9077553	12	55	1.34111667	291.333333	18.3333333	0	0	1	0	18.9135952	1	-0.0428739
0.7056	7309.48918	0.58115299	116.480086	1.62069261	0	-0.3487068	1.98931019	-0.541022	4.75772891	0.48347042	-6.9077553	13	46	3.12927222	294.111111	21.1111111	0	0	1	0	18.9135952	1	-0.0428739
1.1439	15419.2914	0.85301945	243.476819	3.37915641	0	0.13444348	2.73568426	-0.1578013	5.49502585	1.21792198	-6.9077553	14	40	5.36446667	295.777778	22.7777778	0	0	1	0	18.9135952	1	-0.0428739
1.7442	31689.1009	0.936345	273.585317	4.17994152	0	0.556296	3.45600436	-0.0647039	5.61161717	1.43053647	-6.9077553	15	35	6.25854444	297.444444	24.4444444	0	0	1	0	18.9135952	1	-0.0428739
2.5488	64507.1363	1.06836224	367.209522	3.92278821	0	0.93562266	4.16679136	0.06706244	5.90593531	1.36705757	-6.9077553	16	31	6.70558333	299.111111	26.1111111	0	0	1	0	18.9135952	1	-0.0428739
3.2553	94250.2383	1.04797332	338.25625	3.06566263	0	1.18028444	4.54596396	0.0478119	5.8238067	1.12058988	-6.9077553	17	27	6.25854444	300.777778	27.7777778	0	0	1	0	18.9135952	1	-0.0428739
4.2651	143280.687	1.20429283	455.526014	4.0976519	0	1.45046563	4.96481253	0.18672255	6.12145502	1.41065811	-6.9077553	18	24	7.59966111	301.888889	28.8888889	0	0	1	0	18.9135952	1	-0.0428739
5.2434	188789.015	1.09826458	356.891099	2.84312314	2	1.65697014	5.24063536	0.09464139	5.87743349	1.04525481	0.69364706	19	23	5.36446667	303.555556	30.5555556	0	0	1	0	18.9135952	1	-0.0428739
6.1965	234490.958	1.07039296	342.801759	2.94858523	2	1.82398462	5.45742129	0.06895963	5.83715523	1.08166456	0.69364706	20	23	5.81150556	303.555556	30.5555556	0	0	1	0	18.9135952	1	-0.0428739
7.2027	280888.595	1.09619512	367.417319	3.29848637	2	1.97445596	5.63796169	0.09275703	5.90650103	1.19376681	0.69364706	21	22	6.25854444	304.111111	31.1111111	0	0	1	0	18.9135952	1	-0.0428739
8.1549	323802.127	1.03285399	316.240784	2.6280243	2	2.09861897	5.7801357	0.03329356	5.75650706	0.96661279	0.69364706	22	23	5.36446667	304.111111	31.1111111	0	0	1	0	18.9135952	1	-0.0428739
9.0387	364080.374	1.01220566	295.098523	2.33331808	2	2.20151536	5.8973774	0.01311923	5.68731267	0.84771981	0.69364706	23	24	4.91742778	303.555556	30.5555556	0	0	1	0	18.9135952	1	-0.0428739
0.0081	318.86975	0.84193701	191.277115	1.71963394	0	-4.8158912	-1.1398414	-0.1708631	5.25372847	0.54269279	-6.9077553	0	87	4.02335	300.222222	27.2222222	0	0	0	0	30.519848	1	0.21141735
0.0126	538.18872	0.48754315	59.7573303	0.852633	0	-4.3740585	-0.6176896	-0.7163275	4.0903086	-0.1582539	-6.9077553	1	88	2.68223333	300.222222	27.2222222	0	0	0	0	30.519848	1	0.21141735
0.0477	1372.2962	0.57590607	99.9253994	1.2506307	0	-3.0428239	0.31721383	-0.5500758	4.60443391	0.22444726	-6.9077553	2	84	3.57631111	300.777778	27.7777778	0	0	0	0	30.519848	1	0.21141735
0.09	1767.07017	0.45773664	63.997356	1.16059778	0	-2.4079456	0.56988865	-0.779279	4.1588574	0.14979645	-6.9077553	3	78	3.57631111	301.888889	28.8888889	0	0	0	0	30.519848	1	0.21141735
0.1656	2512.75451	0.45609415	60.4689069	1.46813493	0	-1.79818	0.92177745	-0.7828659	4.10214584	0.38467374	-6.9077553	4	76	4.02335	301.888889	28.8888889	0	0	0	0	30.519848	1	0.21141735
0.2601	3204.82842	0.54199695	81.2409226	1.6290988	0	-1.3466891	1.16497054	-0.6106516	4.3974314	0.48864063	-6.9077553	5	66	4.02335	304.111111	31.1111111	0	0	0	0	30.519848	1	0.21141735
0.3951	4480.15735	0.65362559	119.381654	1.94403687	0	-0.9286164	1.49988135	-0.4236918	4.78233391	0.66528093	-6.9077553	6	63	3.57631111	304.666667	31.6666666	0	0	0	1	30.519848	1	0.21141735
0.6624	10203.5801	0.87523695	230.006892	3.2041104	0	-0.4118857	2.32283665	-0.1321187	5.43811362	1.16474654	-6.9077553	7	57	5.81150556	305.777778	32.7777778	0	0	0	1	30.519848	1	0.21141735
0.9504	18771.2515	0.77094417	167.480029	2.42926311	0	-0.0508723	2.93237979	-0.2588431	5.12087009	0.88799953	-6.9077553	8	56	5.36446667	305.777778	32.7777778	0	0	0	1	30.519848	1	0.21141735
1.2717	29300.1211	0.71702477	139.161743	1.94410137	0	0.24035459	3.37762578	-0.3312512	4.93564406	0.66531409	-6.9077553	9	55	5.81150556	306.333333	33.3333333	0	0	0	1	30.519848	1	0.21141735
1.476	39415.4123	0.69678595	138.818239	1.21542802	0	0.38933573	3.67418228	-0.3598429	4.93317265	0.19591871	-6.9077553	10	59	3.12927222	305.777778	32.7777778	0	0	0	1	30.519848	1	0.21141735
1.6542	48108.4846	0.67755921	131.664753	1.2161893	0	0.50331751	3.87347934	-0.3877835	4.88026654	0.19654435	-6.9077553	11	59	3.12927222	305.222222	32.2222222	0	0	0	1	30.519848	1	0.21141735

1.7685	55078.5536	0.69879391	135.099117	1.0357487	0	0.57013173	4.00877857	-0.3569694	4.90601611	0.03608957	-6.9077553	12	65	1.78815556	304.666667	31.66666666	0	0	0	1	30.519848	1	0.21141735
1.8342	61486.9159	0.70736992	131.793972	0.93201635	0	0.60660842	4.11884067	-0.3447888	4.88124747	-0.0693326	-6.9077553	13	70	1.34111667	304.111111	31.11111111	0	0	0	1	30.519848	1	0.21141735
1.8972	68132.6909	0.65461834	110.427539	0.94411	0	0.64037912	4.22147182	-0.4221765	4.70436861	-0.056454	-6.9077553	14	73	1.78815556	303	30	0	0	0	1	30.519848	1	0.21141735
1.9215	69229.2834	0.49287305	60.1753111	0.75495353	0	0.65310613	4.23743839	-0.7054768	4.09727877	-0.2797754	-6.9077553	15	76	2.68223333	301.888889	28.88888888	0	0	0	1	30.519848	1	0.21141735
1.9359	69931.1001	0.2914333	18.2695541	0.38451028	0	0.66057233	4.24752477	-1.2295187	2.9052907	-0.9531874	-6.9077553	16	80	2.23519444	301.333333	28.33333333	0	0	0	1	30.519848	1	0.21141735
1.9494	70545.1931	0.2389703	12.2921743	0.32569745	0	0.66752163	4.25626772	-1.4272401	2.50904417	-1.1187208	-6.9077553	17	81	2.68223333	301.333333	28.33333333	0	0	0	1	30.519848	1	0.21141735
1.9647	71159.2857	0.22853198	11.5664547	0.33020353	0	0.67533956	4.26493488	-1.4717129	2.44819553	-1.1050222	-6.9077553	18	83	2.68223333	300.777778	27.7777778	0	0	0	1	30.519848	1	0.21141735
1.9719	71422.4656	0.25555259	13.6341448	0.34858924	0	0.67899754	4.26862647	-1.3604216	2.61265063	-1.0509964	-6.9077553	19	84	2.68223333	300.777778	27.7777778	0	0	0	1	30.519848	1	0.21141735
1.9845	71992.6958	0.23014232	10.9142521	0.29923763	0	0.68536699	4.27657856	-1.4647217	2.39016109	-1.203181	-6.9077553	20	85	2.23519444	300.222222	27.2222222	0	0	0	1	30.519848	1	0.21141735
1.9917	72212.015	0.20314068	9.13863754	0.2908456	0	0.68898855	4.27962029	-1.5889459	2.21262073	-1.2315304	-6.9077553	21	84	3.57631111	300.222222	27.2222222	0	0	0	1	30.519848	1	0.21141735
2.0052	72650.6544	0.2335734	11.4510518	0.31748063	0	0.69574381	4.28567616	-1.4499867	2.43816891	-1.1441936	-6.9077553	22	84	2.68223333	300.222222	27.2222222	0	0	0	1	30.519848	1	0.21141735
2.0187	73264.7448	0.22372084	11.3401703	0.37470086	0	0.70245374	4.29409317	-1.4928964	2.42843949	-0.978962	-6.9077553	23	83	3.57631111	300.222222	27.2222222	0	0	0	1	30.519848	1	0.21141735
0.0171	751.315065	0.99475087	277.288303	2.1168588	0	-4.0686768	-0.2846001	-0.0042582	5.62506138	0.75040558	-6.9077553	0	87	4.02335	300.222222	27.2222222	0	0	0	0	34.0724638	1	0.49277013
0.0324	1321.54437	0.54172583	71.8819077	0.87436261	0	-3.4295969	0.27955743	-0.611151	4.27503851	-0.1331171	-6.9077553	1	88	2.68223333	300.222222	27.2222222	0	0	0	0	34.0724638	1	0.49277013
0.0513	1935.63728	0.58646724	85.3214402	0.91198685	0	-2.9700645	0.66095311	-0.5319348	4.44643749	-0.0910338	-6.9077553	2	84	3.57631111	300.777778	27.7777778	0	0	0	0	34.0724638	1	0.49277013
0.1044	4114.72733	0.77821725	168.407276	1.60865978	0	-2.2595256	1.41481557	-0.2494654	5.12639125	0.47602284	-6.9077553	3	78	3.57631111	301.888889	28.88888888	0	0	0	0	34.0724638	1	0.49277013
0.1692	7120.20919	0.88105106	205.183729	1.3845822	0	-1.7766738	1.96307754	-0.1255053	5.32391069	0.32612041	-6.9077553	4	76	4.02335	301.888889	28.88888888	0	0	0	0	34.0724638	1	0.49277013
0.2313	9877.10047	0.8642195	199.684415	1.39590266	0	-1.4640397	2.29032023	-0.144772	5.2967432	0.3342574	-6.9077553	5	66	4.02335	304.111111	31.11111111	0	0	0	0	34.0724638	1	0.49277013
0.369	15942.4506	0.99447402	283.346814	2.42412732	0	-0.9969586	2.76904812	-0.0045362	5.64667517	0.88588402	-6.9077553	6	63	3.57631111	304.666667	31.66666666	0	0	0	1	34.0724638	1	0.49277013
0.7659	31771.1443	1.35947714	549.256236	4.60364877	1	-0.2667037	3.45858994	0.30783548	6.30856689	1.52706639	0.0009995	7	57	5.81150556	305.777778	32.7777778	0	0	0	1	34.0724638	1	0.49277013
1.4778	49988.3209	1.35482645	559.980128	4.46357829	1	0.3905545	3.9118094	0.3044112	6.32790308	1.49617476	0.0009995	8	56	5.36446667	305.777778	32.7777778	0	0	0	1	34.0724638	1	0.49277013
2.1096	69091.2745	1.12138249	368.423923	3.22753037	1	0.74649836	4.23544292	0.11545365	5.90923695	1.17202704	0.0009995	9	55	5.81150556	306.333333	33.33333333	0	0	0	1	34.0724638	1	0.49277013
2.601	86667.699	0.95842634	253.866529	1.96502976	1	0.95589599	4.46209279	-0.0414197	5.53681259	0.67601616	0.0009995	10	59	3.12927222	305.777778	32.7777778	0	0	0	1	34.0724638	1	0.49277013
3.096	103005.197	0.96162276	257.254545	2.09019975	1	1.13011096	4.63478915	-0.0380937	5.55006993	0.73773795	0.0009995	11	59	3.12927222	305.222222	32.2222222	0	0	0	1	34.0724638	1	0.49277013
3.4722	115736.046	0.82892474	187.918885	1.55402721	1	1.2447884	4.75132078	-0.1864203	5.23601573	0.44149304	0.0009995	12	65	1.78815556	304.666667	31.66666666	0	0	0	1	34.0724638	1	0.49277013
3.7098	125993.624	0.77730439	163.742395	1.31241064	1	1.31097797	4.83623924	-0.2506376	5.09830054	0.2726273	0.0009995	13	70	1.34111667	304.111111	31.11111111	0	0	0	1	34.0724638	1	0.49277013
3.8862	133428.863	0.70698734	131.208486	1.1485693	1	1.35743182	4.89357597	-0.3453291	4.87679518	0.13938735	0.0009995	14	73	1.78815556	303	30	0	0	0	1	34.0724638	1	0.49277013
3.978	136937.969	0.46903767	55.1338657	0.82642207	1	1.38077918	4.91953535	-0.7549424	4.00978229	-0.1894403	0.0009995	15	76	2.68223333	301.888889	28.88888888	0	0	0	1	34.0724638	1	0.49277013
4.0365	139262.75	0.33313757	27.3207782	0.57780292	1	1.39537798	4.93636962	-1.0962025	3.30768412	-0.5467932	0.0009995	16	80	2.23519444	301.333333	28.33333333	0	0	0	1	34.0724638	1	0.49277013
4.086	140973.433	0.30695589	27.7503351	0.50870691	1	1.4075665	4.94857855	-1.1777987	3.32328395	-0.6739194	0.0009995	17	81	2.68223333	301.333333	28.33333333	0	0	0	1	34.0724638	1	0.49277013
4.1346	142771.846	0.33655439	35.7853258	0.58236528	1	1.41939059	4.96125488	-1.0860286	3.57756586	-0.5389417	0.0009995	18	83	2.68223333	300.777778	27.7777778	0	0	0	1	34.0724638	1	0.49277013
4.1814	144482.529	0.31548225	37.6677622	0.57716252	1	1.43064612	4.97316552	-1.1504881	3.62883116	-0.5479003	0.0009995	19	84	2.68223333	300.777778	27.7777778	0	0	0	1	34.0724638	1	0.49277013
4.2273	145973.905	0.37653826	46.4819192	0.64599557	1	1.44156349	4.98343472	-0.9740834	3.83908492	-0.4354158	0.0009995	20	85	2.23519444	300.222222	27.2222222	0	0	0	1	34.0724638	1	0.49277013
4.2588	146587.999	0.46196115	76.8155552	0.82639536	1	1.44898743	4.98763274	-0.7701121	4.34142018	-0.1894726	0.0009995	21	84	3.57631111	300.222222	27.2222222	0	0	0	1	34.0724638	1	0.49277013
4.2849	147333.677	0.28571461	24.882562	0.47047539	1	1.45509721	4.99270671	-1.2492679	3.21420743	-0.7518884	0.0009995	22	84	2.68223333	300.222222	27.2222222	0	0	0	1	34.0724638	1	0.49277013
4.3218	148386.412	0.40100377	58.363841	0.72696216	1	1.46367198	4.9998265	-0.9112938	4.06671367	-0.3175062	0.0009995	23	83	3.57631111	300.222222	27.2222222	0	0	0	1	34.0724638	1	0.49277013
0.036	2316.74145	1.12960013	352.965971	2.44723393	0	-3.3242363	0.8405932	0.12274858	5.86637448	0.89536692	-6.9077553	0	87	4.02335	300.222222	27.2222222	0	0	0	0	42.288408	1	-0.4111751
0.0837	4648.18213	0.72443505	136.581349	1.33114019	0	-2.4805163	1.53669132	-0.3209837	4.91692773	0.28678682	-6.9077553	1	88	2.68223333	300.222222	27.2222222	0	0	0	0	42.288408	1	-0.4111751
0.1638	8469.72737	0.78043991	170.011258	1.53673613	0	-1.8091091	2.13661638	-0.246617	5.13587054	0.43031129	-6.9077553	2	84	3.57631111	300.777778	27.7777778	0	0	0	0	42.288408	1	-0.4111751
0.3825	17233.8552	0.91481859	239.065458	2.56187508	0	-0.9610266	2.8469338	-0.087937	5.47674158	0.94112971	-6.9077553	3	78	3.57631111	301.888889	28.88888888	0	0	0	0	42.288408	1	-0.4111751
0.6462	29596.3263	0.9636646	255.493974	2.17150837	0	-0.4366462	3.38768403	-0.0359748	5.54320274	0.77588243	-6.9077553	4	76	4.02335	301.888889	28.88888888	0	0	0	0	42.288408	1	-0.4111751
0.9432	41218.4222	0.874641	203.91321	1.88985115	0	-0.0584769	3.71890956	-0.1327991	5.31769937	0.63702707	-6.9077553	5	66	4.02335	304.111111	31.11111111	0	0	1	0	42.288408	1	-0.4111751
1.2915	55317.9121	0.85593598	196.438368	1.55313307	0	0.25580433	4.01311484	-0.1543921	5.28035382	0.44091788	-6.9077553	6	63	3.57631111	304.666667	31.66666666	0	0	1	0	42.288408	1	-0.4111751
1.7775	73478.811	0.91679761	230.803791	2.46798955	0	0.57520788	4.29701069	-0.0857784	5.44157229	0.90380898	-6.9077553	7	57	5.81150556	305.777778	32.7777778	0	0	1	0	42.288408	1	-0.4111751
2.3796	92747.1969	0.87153678	212.036435	2.43980018	0	0.86693241	4.52988826	-0.1363505	5.35676284	0.89232593	-6.9077553	8	56	5.36446667	305.777778	32.7777778	0	0	1	0	42.288408	1	-0.4111751
3.0699	125170.762	1.00432633	291.953899	2.45927308	0	1.12164499	4.82968689	0.0053122	5.67659934	0.90027235	-6.9077553	9	55	5.81150556	306.333333	33.33333333	0	0	1	0	42.288408	1	-0.4111751
3.7818	159903.734	0.99336214	276.326723	2.14048904	0	1.33020009	5.07457822	-0.0056538	5.62158756	0.7615014	-6.9077553	10	59	3.12927222	305.777778	32.7777778	0	0	1	0	42.288408	1	-0.4111751
4.4928	195099.358	0.9794252	269.323972	2.06449299	0	1.50247612	5.27351408	-0.0197689	5.59591872	0.72536893	-6.9077553	11	59	3.12927222	305.222222	32.2222222	0	0	1	0	42.288408	1	-0.4111751
4.9995	217554.171	0.89080138	225.503261	1.66471893	0	1.60933791	5.38245248	-0.1145118	5.41833905	0.51025682	-6.9077553	12	65	1.78815556	304.666667	31.66666666	0	0	1	0	42.288408	1	-0.4111751
5.2749	229756.836	0.80919253	175.749285	1.3129801	0	1.66295972	5.43702587	-0.2104834	5.16906415	0.27306078	-6.9077553	13	70	1.34111667	304.111111	31.11111111	0	0	1	0	42.288408	1	-0.4111751
5.4702	238176.702	0.71030437	133.534058	1.18190009	0	1.69931518	5.47301704	-0.3406549	4.89436405	0.16796913	-6.9077553	14	73	1.78815556	303	30	0	0	1	0	42.288408	1	-0.4111751
5.5836	243001.716	0.49120758	62.5254953	0.79834644	0	1.71983373	5.49307262	-0.7088547	4.13559039	-0.2239608	-6.9077553	15	76	2.68223333	301.888889	28.88888888	0	0	1	0	42.288408	1	-0.4111751
5.661	246028.882	0.33808211	32.2401123	0.60096364	0	1.73360056	5.505453	-1.081513	3.47324242	-0.5075582	-6.9077553	16	80	2.23519444	301.333333	28.33333333	0	0	1	0	42.288408	1	-0.4111751
5.7123	248309.786	0.25308263	13.5091499	0.37491524	0	1.74262174	5.51468113	-1.3700958	2.60344125	-0.9783916	-6.9077553	17	81	2.68223333	301.333333	28.33333333	0	0	1	0	42.288408	1	-0.4111751
5.7627	250327.513	0.23993155	12.2119053	0.31770311	0	1.75140611	5.52277411	-1.4232424	2.50249321	-1.1434953	-6.9077553	18	83	2.68223333	300.777778	27.7777778	0	0	1	0	42.288408	1	-0.4111751
5.8167	252389.13	0.22507251	10.6999512	0.32039515	0	1.76073309	5.53097602	-1.4868995	2.37033263	-1.1350839	-6.9077553	19	84	2.68223333	300.777778	27.7777778	0	0	1	0	42.288408	1	-0.4111751
5.8599	254055.939	0.22687213	10.8510666	0.34458383	0	1.76813254	5.53755841	-1.4789707	2.38435553	-1.06252	-6.9077553	20	85	2.23519444	300.222222	27.2222222	0	0	1	0	42.288408	1	-0.4111751
5.9022	255722.77	0.21142621	9.86627912	0.32487279	0	1.77532516	5.54409784	-1.5491606	2.28922414	-1.1212482	-6.9077553	21	84	3.57631111	300.222222	27.2222222	0	0	1	0	42.288408	1	-0.4111751
5.9571	257916.735	0.35604236	53.5691118	0.6248653	0	1.78458379	5.55264068	-1.0299008	3.9809913	-0.4686201	-6.9077553	22	84	2.68223333	300.222222	27.2222222	0	0	1	0	42.288408	1	-0.4111751
6.0093	259846.727	0.31968038	38.1387645	0.65677926	0	1.79330827	5.56009579	-1.1373104	3.64125743	-0.4188859	-6.9077553	23	83	3.57631111	300.222222	27.2222222	0	0	1	0	42.288408	1	-0.4111751
0.0009	0	0	0	0	0	-7.0131158	-6.9077553	-6.9077553	-6.9077553	-6.9077553	-6.9077553	0	87	4.02335	300.222222	27.2222222	0	0	0	0	18.9135952	1	-0.0428739
0.0009	0	0	0	0	0	-7.0131158	-6.9077553	-6.9077553	-6.9077553	-6.9077553	-6.9077553	1	88	2.68223333	300.222222	27.2222222	0	0	0	0	18.9135952	1	-0.0428739

0.0009	0	0	0	0	0	-7.0131158	-6.9077553	-6.9077553	-6.9077553	-6.9077553	-6.9077553	2	84	3.57631111	300.777778	27.7777778	0	0	0	0	18.9135952	1	-0.0428739
0.0144	0	0.43947652	50.2750081	1.55957031	0	-4.2405271	-6.9077553	-0.8198981	3.91752799	0.44505134	-6.9077553	3	78	3.57631111	301.888889	28.88888888	0	0	0	0	18.9135952	1	-0.0428739
0.0657	239.684166	0.65098819	123.245169	2.13030042	0	-2.7226564	-1.4242697	-0.4277288	4.81418373	0.75673232	-6.9077553	4	76	4.02335	301.888889	28.88888888	0	0	0	0	18.9135952	1	-0.0428739
0.1755	1373.9283	0.63545133	112.286093	1.87581797	0	-1.7401162	0.31840158	-0.4518473	4.72105892	0.62957777	-6.9077553	5	66	4.02335	304.111111	31.11111111	0	0	1	0	18.9135952	1	-0.0428739
0.3492	7163.48609	0.82111031	220.275057	1.9871453	0	-1.0521105	1.96913633	-0.1958807	5.39488156	0.68720219	-6.9077553	6	63	3.57631111	304.666667	31.66666666	0	0	1	0	18.9135952	1	-0.0428739
0.6264	17090.3824	0.86815079	254.994141	2.57573333	0	-0.4677661	2.83857439	-0.1402387	5.54124449	0.94652245	-6.9077553	7	57	5.81150556	305.777778	32.7777778	0	0	1	0	18.9135952	1	-0.0428739
0.927	26277.5082	0.93003672	281.620603	2.80266219	0	-0.0758017	3.26875143	-0.0714566	5.64056433	1.03092649	-6.9077553	8	56	5.36446667	305.777778	32.7777778	0	0	1	0	18.9135952	1	-0.0428739
1.2816	38935.1703	0.89701309	246.247898	2.33323538	0	0.2481093	3.66192365	-0.1075706	5.50634281	0.84768438	-6.9077553	9	55	5.81150556	306.333333	33.33333333	0	0	1	0	18.9135952	1	-0.0428739
1.5705	49953.272	0.85323669	214.827298	1.73508841	0	0.45139404	3.91110803	-0.157547	5.36983909	0.55163454	-6.9077553	10	59	3.12927222	305.777778	32.7777778	0	0	1	0	18.9135952	1	-0.0428739
1.872	60878.1826	0.80777269	192.352903	1.71233165	0	0.62700738	4.10889129	-0.2122374	5.25933692	0.53843981	-6.9077553	11	59	3.12927222	305.222222	32.2222222	0	0	1	0	18.9135952	1	-0.0428739
2.1852	74042.9269	0.85439002	209.915185	1.66511781	0	0.78170736	4.30465852	-0.1561977	5.34670833	0.51049626	-6.9077553	12	65	1.78815556	304.666667	31.66666666	0	0	1	0	18.9135952	1	-0.0428739
2.421	85423.7007	0.89769063	214.665225	1.61563576	0	0.88418068	4.44763529	-0.1068164	5.36908438	0.4803473	-6.9077553	13	70	1.34111667	304.111111	31.11111111	0	0	1	0	18.9135952	1	-0.0428739
2.5938	93613.6971	0.76297235	156.969472	1.43333594	0	0.95312398	4.53918739	-0.2692237	5.05605771	0.36070198	-6.9077553	14	73	1.78815556	303	30	0	0	1	0	18.9135952	1	-0.0428739
2.6802	97385.9834	0.47863928	55.9847158	0.81956228	0	0.98589142	4.57869256	-0.7347209	4.02509658	-0.1977655	-6.9077553	15	76	2.68223333	301.888889	28.88888888	0	0	1	0	18.9135952	1	-0.0428739
2.7369	100017.805	0.34007027	26.295197	0.58192862	0	1.00682589	4.60535822	-1.0756668	3.26942433	-0.5396905	-6.9077553	16	80	2.23519444	301.333333	28.33333333	0	0	1	0	18.9135952	1	-0.0428739
2.7873	102254.858	0.26572534	15.4067928	0.40730783	0	1.02507339	4.62747808	-1.3215358	2.73487341	-0.8957339	-6.9077553	17	81	2.68223333	301.333333	28.33333333	0	0	1	0	18.9135952	1	-0.0428739
2.8251	104053.282	0.24994253	13.6711033	0.42718179	0	1.03854376	4.6449127	-1.3825313	2.6153575	-0.8482074	-6.9077553	18	83	2.68223333	300.777778	27.7777778	0	0	1	0	18.9135952	1	-0.0428739
2.8638	105763.965	0.24317777	12.8245367	0.39499984	0	1.05214941	4.66121932	-1.4098588	2.55143824	-0.9263415	-6.9077553	19	84	2.68223333	300.777778	27.7777778	0	0	1	0	18.9135952	1	-0.0428739
2.9007	107299.193	0.24283712	12.6820351	0.39074344	0	1.06495209	4.67563045	-1.4112548	2.54026528	-0.9371481	-6.9077553	20	85	2.23519444	300.222222	27.2222222	0	0	1	0	18.9135952	1	-0.0428739
2.9358	108834.432	0.25505709	15.4953973	0.39113282	0	1.07697999	4.68983694	-1.3623549	2.74060757	-0.9361547	-6.9077553	21	84	3.57631111	300.222222	27.2222222	0	0	1	0	18.9135952	1	-0.0428739
2.9799	110764.447	0.24069619	12.5202114	0.3887094	0	1.09188974	4.70741488	-1.4200738	2.52742411	-0.9423539	-6.9077553	22	84	2.68223333	300.222222	27.2222222	0	0	1	0	18.9135952	1	-0.0428739
3.0222	112738.315	0.25039488	13.7658613	0.47568256	0	1.10598504	4.7250782	-1.3807303	2.62226435	-0.7409045	-6.9077553	23	83	3.57631111	300.222222	27.2222222	0	0	1	0	18.9135952	1	-0.0428739
0.0108	407.720003	0.87667188	207.55218	1.51726376	0	-4.5282091	-0.8947249	-0.1304825	5.33538759	0.41756742	-6.9077553	0	70	1.78815556	303	30	0	0	0	0	30.519848	1	0.21141735
0.0216	892.516413	0.77161598	152.40449	1.31393528	0	-3.835062	-0.1125906	-0.2579731	5.02654467	0.27378745	-6.9077553	1	77	0.44703889	301.888889	28.88888888	0	0	0	0	30.519848	1	0.21141735
0.036	1243.42669	0.64178622	107.211838	1.30420256	0	-3.3242363	0.21867493	-0.4419431	4.674816	0.26635824	-6.9077553	2	75	0.89407778	297.444444	24.44444444	0	0	0	0	30.519848	1	0.21141735
0.0396	1375.01792	0.54828393	70.9243698	0.88686806	0	-3.2289262	0.31919376	-0.5991398	4.26162819	-0.1189321	-6.9077553	3	88	0.89407778	295.222222	22.2222222	0	0	0	0	30.519848	1	0.21141735
0.0414	1418.88171	0.2144724	9.22966766	0.70517886	0	-3.1844744	0.35057357	-1.5349225	2.22253138	-0.3478867	-6.9077553	4	46	2.23519444	296.888889	23.88888888	0	0	0	0	30.519848	1	0.21141735
0.0891	1769.79197	0.35919373	41.7573979	1.21222931	0	-2.4179959	0.57142689	-1.0211133	3.73190058	0.19328566	-6.9077553	5	42	2.23519444	296.333333	23.33333333	0	0	0	0	30.519848	1	0.21141735
0.1557	2530.14621	0.44062238	56.8973818	1.29131998	0	-1.8598242	0.92867225	-0.8173001	4.0412669	0.25643903	-6.9077553	6	35	3.12927222	295.777778	22.7777778	0	0	0	0	30.519848	1	0.21141735
0.3555	4199.47028	0.6945941	133.754883	3.13412338	0	-1.03423	1.43519649	-0.362989	4.89601637	1.14266853	-6.9077553	7	36	4.91742778	294.666667	21.66666666	0	0	0	1	30.519848	1	0.21141735
0.504	5702.4654	0.54463538	81.3704427	1.39442287	0	-0.685179	1.74107396	-0.6058043	4.39902438	0.3331975	-6.9077553	8	43	2.68223333	293	20	0	0	0	1	30.519848	1	0.21141735
0.6354	7438.58598	0.48010886	67.6952924	0.92403381	0	-0.4535006	2.0068152	-0.7316617	4.21503141	-0.077925	-6.9077553	9	54	0.44703889	290.222222	17.2222222	0	0	0	1	30.519848	1	0.21141735
0.7443	9184.70034	0.48399583	71.9849642	0.82554261	0	-0.2953111	2.21764796	-0.723615	4.27647116	-0.1905038	-6.9077553	10	55	1.34111667	291.333333	18.33333333	0	0	0	1	30.519848	1	0.21141735
1.0449	12231.7474	0.62984654	113.605071	1.81871024	0	0.04392119	2.50411657	-0.4606926	4.73273695	0.59867728	-6.9077553	11	46	3.12927222	294.111111	21.11111111	0	0	0	1	30.519848	1	0.21141735
1.7514	19276.2661	0.97894707	296.805096	4.10243643	0	0.56041547	2.95892648	-0.0202567	5.69307905	1.41182478	-6.9077553	12	40	5.36446667	295.777778	22.7777778	0	0	0	1	30.519848	1	0.21141735
2.8035	30020.1958	0.99765999	300.571509	4.45979135	0	1.03086864	3.40190366	-0.0013409	5.70568902	1.49532618	-6.9077553	13	35	6.25854444	297.444444	24.44444444	0	0	0	1	30.519848	1	0.21141735
3.645	48140.4968	0.95689051	262.493349	2.36220076	0	1.29335637	3.87414452	-0.0430218	5.57022955	0.86001696	-6.9077553	14	31	6.70558333	299.111111	26.11111111	0	0	0	1	30.519848	1	0.21141735
4.5792	63359.4566	0.94491642	251.37643	1.98914868	0	1.52152431	4.14883995	-0.0556011	5.52695552	0.68820935	-6.9077553	15	27	6.25854444	300.777778	27.7777778	0	0	0	1	30.519848	1	0.21141735
5.9157	120478.397	1.1309073	384.494444	3.63999369	41	1.77760983	4.79147876	0.12390408	5.95193194	1.29225664	3.71359646	16	24	7.59966111	301.888889	28.88888888	0	0	0	1	30.519848	1	0.21141735
6.9822	243139.614	1.28843515	465.209177	2.66147152	146	1.94336405	5.49363993	0.25420426	6.1424893	0.97925483	4.98361347	17	23	5.36446667	303.555556	30.55555555	0	0	0	1	30.519848	1	0.21141735
8.3088	612830.146	2.02817721	941.75848	3.35562246	486	2.11731519	6.41808944	0.70763039	6.84774991	1.21093525	6.18621068	18	23	5.81150556	303.555556	30.55555555	0	0	0	1	30.519848	1	0.21141735
10.4994	1354854.4	2.28953714	1091.82621	4.62292731	1187	2.35131811	7.21145002	0.82878635	6.99560791	1.53124441	7.07918524	19	22	6.25854444	304.111111	31.11111111	0	0	0	1	30.519848	1	0.21141735
13.0194	2040022.11	2.24839634	1112.76723	4.4623877	1871	2.56644055	7.62071642	0.81066189	7.01460609	1.49590805	7.53422886	20	23	5.36446667	304.111111	31.11111111	0	0	0	1	30.519848	1	0.21141735
14.2677	2245941.52	1.83416955	892.756579	3.34980089	2101	2.65799824	7.71688054	0.60713687	6.79431508	1.20919939	7.65016918	21	24	4.91742778	303.555556	30.55555555	0	0	0	1	30.519848	1	0.21141735
15.0471	2266811.71	0.92082676	250.831752	2.25161933	2101	2.71118528	7.72613003	-0.081398	5.52478639	0.81209369	7.65016918	22	27	3.57631111	302.444444	29.44444444	0	0	0	1	30.519848	1	0.21141735
15.5817	2283534.5	0.87262795	224.972775	1.77069893	2101	2.74609715	7.73348018	-0.1351007	5.41598384	0.57193894	7.65016918	23	46	0.44703889	299.111111	26.11111111	0	0	0	1	30.519848	1	0.21141735
0.0198	934.850149	1.00215294	281.741374	1.98845872	0	-3.9220733	-0.0662999	0.00314798	5.64099308	0.6878626	-6.9077553	0	70	1.78815556	303	30	0	0	0	0	34.0724638	1	0.49277013
0.0342	1373.48803	0.67160475	112.462082	0.93991214	0	-3.3755296	0.31808132	-0.3965974	4.72262501	-0.0609055	-6.9077553	1	77	0.44703889	301.888889	28.88888888	0	0	0	0	34.0724638	1	0.49277013
0.0477	1899.85349	0.45241731	49.6927409	0.65564899	0	-3.0428239	0.64230299	-0.7909424	3.90587899	-0.4206057	-6.9077553	2	75	0.89407778	297.444444	24.44444444	0	0	0	0	34.0724638	1	0.49277013
0.0621	2426.21879	0.52853107	71.1097031	0.84131122	0	-2.7790093	0.88674607	-0.6357634	4.26423786	-0.1716057	-6.9077553	3	88	0.89407778	295.222222	22.2222222	0	0	0	0	34.0724638	1	0.49277013
0.0747	2952.58413	0.37705074	35.6891501	0.46959135	0	-2.5942752	1.08301939	-0.9727269	3.57487474	-0.7537652	-6.9077553	4	46	2.23519444	296.888889	23.88888888	0	0	0	0	34.0724638	1	0.49277013
0.1494	6403.10114	0.89398809	225.58269	1.69358844	0	-1.901128	1.85693859	-0.1109449	5.41869122	0.5274399	-6.9077553	5	42	2.23519444	296.333333	23.33333333	0	0	0	0	34.0724638	1	0.49277013
0.2196	9743.81555	0.92968995	230.101237	1.51460511	0	-1.5159476	2.2767354	-0.0718291	5.43852372	0.41581477	-6.9077553	6	35	3.12927222	295.777778	22.7777778	0	0	0	0	34.0724638	1	0.49277013
0.3303	13342.9954	0.88644601	209.193105	2.10307101	0	-1.1077539	2.5910665	-0.1194076	5.34326255	0.74387404	-6.9077553	7	36	4.91742778	294.666667	21.66666666	0	0	0	1	34.0724638	1	0.49277013
0.4824	20273.3345	0.96099501	257.731463	1.95315931	0	-0.7289816	3.00935578	-0.038746	5.55192208	0.66996008	-6.9077553	8	43	2.68223333	293	20	0	0	0	1	34.0724638	1	0.49277013
0.675	28598.531	0.91745765	234.223751	1.67327396	0	-0.3930426	3.35339032	-0.0850595	5.45628113	0.51537961	-6.9077553	9	54	0.44703889	290.222222	17.2222222	0	0	0	1	34.0724638	1	0.49277013
0.8793	37106.7187	0.8869766	227.090274	1.66242342	0	-0.1286291	3.613825	-0.1188099	5.42535202	0.50887778	-6.9077553	10	55	1.34111667	291.333333	18.33333333	0	0	0	1	34.0724638	1	0.49277013
1.1979	46804.2353	0.90460231	230.841256	1.93491428	0	0.18057002	3.84599506	-0.099155	5.4417346	0.66057971	-6.9077553	11	46	3.12927222	294.111111	21.11111111	0	0	0	1	34.0724638	1	0.49277013
1.5408	57145.3057	0.93365895	249.331221	2.30644294	0	0.43230176	4.04561475	-0.0675736	5.51878623	0.83613996	-6.9077553	12	40	5.36446667	295.777778	22.7777778	0	0	0	1	34.0724638	1	0.49277013
2.1249	79029.9628	1.09496468	353.662197	2.95743715	0	0.75372474	4.36983971	0.09163497	5.86834504	1.08466114	-6.9077553	13	35	6.25854444	297.444444	24.44444444	0	0	0	1	34.0724638	1	0.49277013
2.9412	112354.825	1.10801836	345.009613	2.84091425	0	1.07881766	4.72167085	0.10347527	5.84357518	1.04447786	-6.9077553	14	31	6.70558333	299.111111	26.11111111	0	0	0	1	34.0724638	1	0.49277013
3.987	147418.783	1.08317264	337.174699	3.27195375	1	1.38303907	4.99328419	0.08081715	5.82060416	1.18569286	0.0009995	15	27	6.25854444	300.777778	27.7777778	0	0	0	1	34.0724638	1	0.49277013

5.3676	203871.776	1.17020676	399.619948	3.65467184	1	1.68038088	5.31749615	0.15803464	5.99051647	1.29627989	0.0009995	16	24	7.59966111	301.888889	28.88888888	0	0	0	1	34.0724638	1	0.49277013
6.7365	256545.217	1.07507669	336.921844	3.04979762	2	1.9075405	5.54730883	0.07332174	5.81985396	1.11540307	0.69364706	17	23	5.36446667	303.555556	30.55555555	0	0	0	1	34.0724638	1	0.49277013
8.2386	312309.755	1.10530711	366.476483	3.65817387	2	2.10883043	5.7439987	0.10102754	5.90393708	1.2972374	0.69364706	18	23	5.81150556	303.555556	30.55555555	0	0	0	1	34.0724638	1	0.49277013
9.8253	366903.876	0.97085331	288.280594	3.32495713	2	2.28496069	5.90510262	-0.0285504	5.66393776	1.20175749	0.69364706	19	22	6.25854444	304.111111	31.11111111	0	0	0	1	34.0724638	1	0.49277013
11.2212	407343.643	0.89690143	248.314676	2.92612975	2	2.41780485	6.00965962	-0.107695	5.51470082	1.07402234	0.69364706	20	23	5.36446667	304.111111	31.11111111	0	0	0	1	34.0724638	1	0.49277013
12.6324	450076.824	0.91939809	270.101204	2.94894471	2	2.53626494	6.10942051	-0.082949	5.59880042	1.08178643	0.69364706	21	24	4.91742778	303.555556	30.55555555	0	0	0	1	34.0724638	1	0.49277013
13.9356	489831.97	0.86738376	238.465642	2.49662621	2	2.63444672	6.19406445	-0.1411215	5.47422944	0.91534076	0.69364706	22	27	3.57631111	302.444444	29.44444444	0	0	0	1	34.0724638	1	0.49277013
14.994	520661.108	0.7891636	191.096248	1.80895726	2	2.70765012	6.25510129	-0.2355153	5.25278245	0.59330323	0.69364706	23	46	0.44703889	299.111111	26.11111111	0	0	0	1	34.0724638	1	0.49277013
0.0324	2063.75503	1.1336537	352.266713	2.31022971	0	-3.4295969	0.72501159	0.12632749	5.86439144	0.83777972	-6.9077553	0	70	1.78815556	303	30	0	0	0	0	42.288408	1	-0.4111751
0.0963	5201.0554	0.85876733	196.771154	1.55689873	0	-2.340287	1.64905382	-0.1510935	5.28204648	0.44333795	-6.9077553	1	77	0.44703889	301.888889	28.88888888	0	0	0	0	42.288408	1	-0.4111751
0.1539	7920.61001	0.62137365	96.5384598	1.07626808	0	-1.8714522	2.06959447	-0.4742146	4.56995183	0.07442828	-6.9077553	2	75	0.89407778	297.444444	24.44444444	0	0	0	0	42.288408	1	-0.4111751
0.2115	10684.0292	0.61947179	97.4957275	1.05745316	0	-1.5535303	2.36884362	-0.4772751	4.57981881	0.05680856	-6.9077553	3	88	0.89407778	295.222222	22.2222222	0	0	0	0	42.288408	1	-0.4111751
0.2475	12402.7763	0.45942802	64.7832642	0.83356981	0	-1.3963447	2.51800096	-0.7755987	4.17106274	-0.1808389	-6.9077553	4	46	2.23519444	296.888889	23.88888888	0	0	0	0	42.288408	1	-0.4111751
0.4572	22556.7218	0.94409448	261.654758	1.92089163	0	-0.7826343	3.11607744	-0.0564704	5.56702974	0.65330992	-6.9077553	5	42	2.23519444	296.333333	23.33333333	0	0	0	0	42.288408	1	-0.4111751
0.7704	37661.2668	0.99749072	283.874461	2.10501221	0	-0.2608454	3.62865871	-0.0015104	5.64853563	0.74479621	-6.9077553	6	35	3.12927222	295.777778	22.7777778	0	0	1	0	42.288408	1	-0.4111751
1.1943	53151.5986	0.91437182	233.148554	2.38000618	0	0.17756024	3.973167	-0.0884249	5.45168011	0.86752316	-6.9077553	7	36	4.91742778	294.666667	21.66666666	0	0	1	0	42.288408	1	-0.4111751
1.5066	65704.1502	0.80889945	177.061172	1.38615571	0	0.40985546	4.18517731	-0.2108452	5.17650092	0.3272554	-6.9077553	8	43	2.68223333	293	20	0	0	1	0	42.288408	1	-0.4111751
1.8423	78980.8147	0.79324619	173.063945	1.37088414	0	0.61101479	4.36921763	-0.2303618	5.15366693	0.31618508	-6.9077553	9	54	0.44703889	290.222222	17.2222222	0	0	1	0	42.288408	1	-0.4111751
2.1492	90416.6746	0.72800927	144.384783	1.17371214	0	0.76509568	4.50443976	-0.3160688	4.97248876	0.16102313	-6.9077553	10	55	1.34111667	291.333333	18.33333333	0	0	1	0	42.288408	1	-0.4111751
2.6577	107392.322	0.78833834	174.532826	1.83728697	0	0.97746109	4.676498	-0.2365602	5.16211857	0.60883414	-6.9077553	11	46	3.12927222	294.111111	21.11111111	0	0	1	0	42.288408	1	-0.4111751
3.6306	135768.916	0.89260105	236.1495	3.21667943	0	1.28939792	4.91096166	-0.1124959	5.46446931	1.16866043	-6.9077553	12	40	5.36446667	295.777778	22.7777778	0	0	1	0	42.288408	1	-0.4111751
5.0418	165078.956	0.83354452	210.438178	3.76282112	0	1.61776316	5.10642994	-0.1808692	5.34919667	1.3254347	-6.9077553	13	35	6.25854444	297.444444	24.44444444	0	0	1	0	42.288408	1	-0.4111751
6.8886	216923.063	0.99935645	313.966557	4.56221541	0	1.92986787	5.37954735	0.00035639	5.74928966	1.51802751	-6.9077553	14	31	6.70558333	299.111111	26.11111111	0	0	1	0	42.288408	1	-0.4111751
8.4069	283715.115	1.17072058	419.976512	4.30260586	0	2.1290528	5.64797414	0.15847325	6.04020117	1.45945324	-6.9077553	15	27	6.25854444	300.777778	27.7777778	0	0	1	0	42.288408	1	-0.4111751
9.7434	352877.461	1.23326519	463.287879	3.94507444	0	2.27659013	5.86612369	0.2104758	6.13835079	1.37272127	-6.9077553	16	24	7.59966111	301.888889	28.88888888	0	0	1	0	42.288408	1	-0.4111751
11.0781	420528.869	1.13952974	389.88423	3.02253798	0	2.40497019	6.04151551	0.13149284	5.96585241	1.10642766	-6.9077553	17	23	5.36446667	303.555556	30.55555555	0	0	1	0	42.288408	1	-0.4111751
12.5352	488856.611	1.14612359	393.822074	3.40067171	3	2.52854069	6.19207126	0.13725759	5.97590176	1.22426699	1.09894557	18	23	5.81150556	303.555556	30.55555555	0	0	1	0	42.288408	1	-0.4111751
14.0994	560518.798	1.15915433	397.879064	3.82892232	4	2.64613224	6.32886456	0.14855304	5.98615061	1.34284452	1.38654433	19	22	6.25854444	304.111111	31.11111111	0	0	1	0	42.288408	1	-0.4111751
15.498	630954.06	1.15395062	398.33816	3.16076644	4	2.74071098	6.44723464	0.14405759	5.9873038	1.15113087	1.38654433	20	23	5.36446667	304.111111	31.11111111	0	0	1	0	42.288408	1	-0.4111751
16.9398	688344.282	1.04180504	325.650964	3.15767888	6	2.82966588	6.53429058	0.04191424	5.78582921	1.15015386	1.79192612	21	24	4.91742778	303.555556	30.55555555	0	0	1	0	42.288408	1	-0.4111751
18.2646	761921.923	1.04121996	318.07993	2.33433317	27	2.90496476	6.6358454	0.04135302	5.76230585	0.84815457	3.2958739	22	27	3.57631111	302.444444	29.44444444	0	0	1	0	42.288408	1	-0.4111751
19.2708	844879.351	0.98805502	289.734543	1.81486563	70	2.958591	6.73919502	-0.0110053	5.66896859	0.59656229	4.24850953	23	46	0.44703889	299.111111	26.11111111	0	0	1	0	42.288408	1	-0.4111751
0.0018	0	0.1061632	1.98204601	0.29212055	0	-6.3199686	-6.9077553	-2.2334023	0.68463405	-1.2271713	-6.9077553	0	70	1.78815556	303	30	0	0	0	0	18.9135952	1	-0.0428739
0.0018	0	0	0	0	0	-6.3199686	-6.9077553	-6.9077553	-6.9077553	-6.9077553	-6.9077553	1	77	0.44703889	301.888889	28.88888888	0	0	0	0	18.9135952	1	-0.0428739
0.0018	0	0	0	0	0	-6.3199686	-6.9077553	-6.9077553	-6.9077553	-6.9077553	-6.9077553	2	75	0.89407778	297.444444	24.44444444	0	0	0	0	18.9135952	1	-0.0428739
0.0018	0	0	0	0	0	-6.3199686	-6.9077553	-6.9077553	-6.9077553	-6.9077553	-6.9077553	3	88	0.89407778	295.222222	22.2222222	0	0	0	0	18.9135952	1	-0.0428739
0.0018	0	0	0	0	0	-6.3199686	-6.9077553	-6.9077553	-6.9077553	-6.9077553	-6.9077553	4	46	2.23519444	296.888889	23.88888888	0	0	0	0	18.9135952	1	-0.0428739
0.0171	0	0.36484831	37.8692663	1.41049351	0	-4.0686768	-6.9077553	-1.0055365	3.63416627	0.34464837	-6.9077553	5	42	2.23519444	296.333333	23.33333333	0	0	0	0	18.9135952	1	-0.0428739
0.0972	0	0.47608151	57.4275259	2.00378418	0	-2.3309846	-6.9077553	-0.7400679	4.05054115	0.69553641	-6.9077553	6	35	3.12927222	295.777778	22.7777778	0	0	0	0	18.9135952	1	-0.0428739
0.2178	131.59135	0.39130991	39.2192346	1.71010874	0	-1.5241781	-2.0204834	-0.9357032	3.6691928	0.53714155	-6.9077553	7	36	4.91742778	294.666667	21.66666666	0	0	1	0	18.9135952	1	-0.0428739
0.3123	526.366262	0.37681296	46.2126535	1.18831845	0	-1.163791	-0.63986	-0.973356	3.83327528	0.17338041	-6.9077553	8	43	2.68223333	293	20	0	0	1	0	18.9135952	1	-0.0428739
0.3735	1328.519	0.41416715	64.6742446	0.76525755	0	-0.9848373	0.28481722	-0.8790741	4.16937851	-0.2662369	-6.9077553	9	54	0.44703889	290.222222	17.2222222	0	0	1	0	18.9135952	1	-0.0428739
0.4518	3292.65893	0.55234063	111.833143	1.00426194	0	-0.7945157	1.19199908	-0.5917815	4.7170169	0.00524814	-6.9077553	10	55	1.34111667	291.333333	18.33333333	0	0	1	0	18.9135952	1	-0.0428739
0.6696	6215.57026	0.61493784	129.230194	1.87070005	0	-0.4010748	1.82721835	-0.4846092	4.861603	0.62684713	-6.9077553	11	46	3.12927222	294.111111	21.11111111	0	0	1	0	18.9135952	1	-0.0428739
1.1169	13973.4016	0.8354203	234.34911	3.44432857	0	0.11055699	2.6372272	-0.178624	5.45681619	1.23701928	-6.9077553	12	40	5.36446667	295.777778	22.7777778	0	0	1	0	18.9135952	1	-0.0428739
1.7334	31356.7809	0.96129186	287.576779	4.15664918	0	0.5500848	3.44546243	-0.0384375	5.66149336	1.42494981	-6.9077553	13	35	6.25854444	297.444444	24.44444444	0	0	1	0	18.9135952	1	-0.0428739
2.5047	64075.2195	1.10588154	395.31735	4.19378954	0	0.91816897	4.1600733	0.10154664	5.97969139	1.43384317	-6.9077553	14	31	6.70558333	299.111111	26.11111111	0	0	1	0	18.9135952	1	-0.0428739
3.1914	93515.8093	1.03013009	326.093422	2.9322377	0	1.16045969	4.5381412	0.03065537	5.78718698	1.07610683	-6.9077553	15	27	6.25854444	300.777778	27.7777778	0	0	1	0	18.9135952	1	-0.0428739
4.2057	143471.445	1.21803989	467.391249	4.20836958	0	1.43644075	4.96614299	0.19806357	6.14716884	1.43731289	-6.9077553	16	24	7.59966111	301.888889	28.88888888	0	0	1	0	18.9135952	1	-0.0428739
5.1813	189330.794	1.10734485	364.590348	3.00770214	1	1.64505599	5.243501	0.10286778	5.89877713	1.1015088	0.0009995	17	23	5.36446667	303.555556	30.55555555	0	0	1	0	18.9135952	1	-0.0428739
6.1848	235808.239	1.09365169	358.409809	3.26519671	1	1.82209467	5.46302317	0.09043622	5.88167984	1.18362622	0.0009995	18	23	5.81150556	303.555556	30.55555555	0	0	1	0	18.9135952	1	-0.0428739
7.2963	289703.423	1.12887393	388.587551	3.34862411	1	1.98736737	5.66886117	0.12210606	5.96252107	1.20884813	0.0009995	19	22	6.25854444	304.111111	31.11111111	0	0	1	0	18.9135952	1	-0.0428739
8.2143	332350.822	1.03265189	319.897917	2.48196998	1	2.10587654	5.80619411	0.03309806	5.76800506	0.90945541	0.0009995	20	23	5.36446667	304.111111	31.11111111	0	0	1	0	18.9135952	1	-0.0428739
9.0567	369502.845	0.98324031	275.549245	2.11255587	1	2.20350482	5.91216115	-0.0158852	5.61876999	0.74837178	0.0009995	21	24	4.91742778	303.555556	30.55555555	0	0	1	0	18.9135952	1	-0.0428739
9.8955	408035.324	0.95957207	253.829533	1.82911649	1	2.29208011	6.0113562	-0.0402263	5.53666685	0.60437962	0.0009995	22	27	3.57631111	302.444444	29.44444444	0	0	1	0	18.9135952	1	-0.0428739
10.8288	450450.778	0.96547882	261.73656	1.99897776	1	2.38220925	6.11025103	-0.0340959	5.56734232	0.69313606	0.0009995	23	46	0.44703889	299.111111	26.11111111	0	0	1	0	18.9135952	1	-0.0428739
0.0135	363.84732	0.82379791	184.33517	1.70469448	0	-4.3050656	-1.0082763	-0.1926169	5.2167611	0.53397234	-6.9077553	0	89	3.57631111	291.888889	18.88888888	0	0	0	0	30.519848	1	0.21141735
0.0549	1074.69099	0.50427955	71.7747484	1.73709438	0	-2.9022419	0.07296323	-0.6826434	4.27354665	0.55278933	-6.9077553	1	79	5.36446667	294.111111	21.11111111	0	0	0	0	30.519848	1	0.21141735
0.1665	1950.74867	0.60199984	110.231004	2.79040011	0	-1.79276	0.66872573	-0.5058384	4.70258727	1.0265433	-6.9077553	2	73	6.70558333	295.222222	22.2222222	0	0	0	0	30.519848	1	0.21141735
0.279	3349.46668	0.62971667	117.177086	2.52860352	0	-1.2765435	1.20909964	-0.4608985	4.76369488	0.92806258	-6.9077553	3	66	4.91742778	297.444444	24.44444444	0	0	0	0	30.519848	1	0.21141735
0.3825	7412.66877	0.60510267	101.627531	1.64825824	0	-0.9610266	2.00332543	-0.5007059	4.62132431	0.50032564	-6.9077553	4	64	5.36446667	298	25	0	0	0	0	30.519848	1	0.21141735
0.5742	13555.8806	0.59371701	96.8983366	1.73299985	0	-0.5547775	2.60689422	-0.5196696	4.57367267	0.55043079	-6.9077553	5	59	4.91742778	299.111111	26.11111111	0	0	0	1	30.519848	1	0.21141735

0.8631	25184.478	0.67388783	127.473666	2.10494948	0	-0.1472247	3.22626756	-0.3932088	4.84791765	0.74476642	-6.9077553	6	53	6.25854444	300.222222	27.2222222	0	0	0	1	30.519848	1	0.21141735
1.1574	39081.149	0.72728998	154.698848	2.16678762	0	0.14617611	3.66566582	-0.317056	5.04148678	0.77370712	-6.9077553	7	50	6.25854444	300.777778	27.7777778	0	0	0	1	30.519848	1	0.21141735
1.4058	56149.1352	0.66958618	126.680225	1.33423338	0	0.34060654	4.02802909	-0.3996031	4.84167389	0.28910609	-6.9077553	8	53	4.91742778	300.222222	27.2222222	0	0	0	1	30.519848	1	0.21141735
1.62	72087.9934	0.6420842	116.270746	1.10687717	0	0.48242615	4.27790137	-0.4414796	4.75593009	0.10244573	-6.9077553	9	53	4.91742778	299.666667	26.66666666	0	0	0	1	30.519848	1	0.21141735
1.8018	88769.7306	0.61165237	113.06952	0.91083255	0	0.58878617	4.48605699	-0.4899576	4.72801169	-0.0922989	-6.9077553	10	57	3.57631111	298	25	0	0	0	1	30.519848	1	0.21141735
1.8999	101095.331	0.69094394	127.889111	0.84564636	0	0.64180125	4.61607383	-0.3682503	4.85117139	-0.1664722	-6.9077553	11	67	1.78815556	295.777778	22.7777778	0	0	0	1	30.519848	1	0.21141735
2.0007	113510.718	0.71994441	139.623448	1.0355023	0	0.69349712	4.73190607	-0.3271932	4.9389563	0.03585187	-6.9077553	12	70	1.78815556	295.222222	22.2222222	0	0	0	1	30.519848	1	0.21141735
2.0979	124742.59	0.73547667	143.206688	1.13903575	0	0.74093684	4.82626035	-0.3058777	4.96429594	0.13105963	-6.9077553	13	75	1.78815556	294.111111	21.11111111	0	0	0	1	30.519848	1	0.21141735
2.1573	128244.18	0.59998547	94.1345289	1.02073878	0	0.76885744	4.8539439	-0.5091845	4.54473554	0.02150586	-6.9077553	14	79	2.23519444	293.555556	20.55555555	0	0	0	1	30.519848	1	0.21141735
2.1798	129165.32	0.23561747	11.4559839	0.30378626	0	0.77923313	4.86110088	-1.4413105	2.43859949	-1.1881445	-6.9077553	15	83	3.12927222	293	20	0	0	0	1	30.519848	1	0.21141735
2.1933	129691.685	0.19209436	7.5532486	0.34515085	0	0.78540726	4.86516769	-1.6445763	2.02211013	-1.0608806	-6.9077553	16	87	3.57631111	293	20	0	0	0	1	30.519848	1	0.21141735
2.2068	130261.922	0.19420908	7.50657552	0.24642148	0	0.7915435	4.86955489	-1.6336841	2.01591258	-1.396662	-6.9077553	17	91	3.57631111	292.444444	19.44444444	0	0	0	1	30.519848	1	0.21141735
2.223	130876.008	0.15849724	5.17143589	0.2641942	0	0.79885763	4.87425801	-1.8357287	1.64334374	-1.3272929	-6.9077553	18	94	3.12927222	291.888889	18.88888888	0	0	0	1	30.519848	1	0.21141735
2.2329	131270.784	0.15944625	5.03850798	0.18560325	0	0.80330119	4.87726986	-1.8297963	1.61730845	-1.6787706	-6.9077553	19	93	4.02335	291.888889	18.88888888	0	0	0	1	30.519848	1	0.21141735
2.2419	131621.692	0.16235778	5.18022346	0.27829959	0	0.80732372	4.87993944	-1.8118125	1.64504122	-1.2754703	-6.9077553	20	90	4.91742778	291.888889	18.88888888	0	0	0	1	30.519848	1	0.21141735
2.25	132016.459	0.14496117	3.95078744	0.15861362	0	0.81093022	4.88293418	-1.9244146	1.37416799	-1.8349993	-6.9077553	21	90	3.57631111	291.888889	18.88888888	0	0	0	1	30.519848	1	0.21141735
2.2572	132279.646	0.14549114	4.01800442	0.16717854	0	0.81412511	4.88492577	-1.9207903	1.39103421	-1.7827291	-6.9077553	22	89	4.47038889	291.888889	18.88888888	0	0	0	1	30.519848	1	0.21141735
2.2617	132455.099	0.14825031	4.1271534	0.17058854	0	0.81611674	4.88625126	-1.9021305	1.41783019	-1.7626559	-6.9077553	23	90	4.47038889	291.888889	18.88888888	0	0	0	1	30.519848	1	0.21141735
0.0243	907.059243	0.96511283	263.625451	2.45865323	0	-3.7172789	-0.0964457	-0.0344746	5.57453314	0.90002038	-6.9077553	0	89	3.57631111	291.888889	18.88888888	0	0	0	0	34.0724638	1	0.49277013
0.0675	2634.45981	0.76296997	167.687653	1.53753249	0	-2.6956277	0.96905767	-0.2692268	5.122109	0.43082903	-6.9077553	1	79	5.36446667	294.111111	21.11111111	0	0	0	0	34.0724638	1	0.49277013
0.1611	6885.69405	0.95971562	246.195989	2.0612523	0	-1.82573	1.92959115	-0.0400768	5.50613198	0.72379874	-6.9077553	2	73	6.70558333	295.222222	22.2222222	0	0	0	0	34.0724638	1	0.49277013
0.2628	10397.4155	0.83365111	181.812673	1.81044911	0	-1.336362	2.34165344	-0.1807415	5.20298239	0.59412714	-6.9077553	3	66	4.91742778	297.444444	24.44444444	0	0	0	1	34.0724638	1	0.49277013
0.3906	17488.2054	1.11544273	356.565994	3.00221618	0	-0.9400713	2.86158386	0.1101475	5.87652214	1.09968377	-6.9077553	4	64	5.36446667	298	25	0	0	0	1	34.0724638	1	0.49277013
0.6903	34757.4064	1.25406654	442.824794	4.13116253	0	-0.370629	3.54842145	0.22718859	6.09317645	1.41880089	-6.9077553	5	59	4.91742778	299.111111	26.11111111	0	0	0	1	34.0724638	1	0.49277013
1.0332	51709.4825	1.19942268	425.578289	3.47035966	0	0.03266078	3.94566052	0.18267373	6.05345127	1.24454635	-6.9077553	6	53	6.25854444	300.222222	27.2222222	0	0	0	1	34.0724638	1	0.49277013
1.6029	67802.8974	1.16370805	406.044925	3.96309118	0	0.47181449	4.21661968	0.15247046	6.00646627	1.37727662	-6.9077553	7	50	6.25854444	300.777778	27.7777778	0	0	0	1	34.0724638	1	0.49277013
2.1681	88091.0855	1.03313879	309.860072	2.49905415	0	0.77385121	4.47838269	0.03356899	5.73612404	0.91631239	-6.9077553	8	53	4.91742778	300.222222	27.2222222	0	0	0	1	34.0724638	1	0.49277013
2.6379	105343.172	0.90854887	248.824743	2.1627997	0	0.96998315	4.65723281	-0.0948065	5.51675282	0.7718658	-6.9077553	9	53	4.91742778	299.666667	26.66666666	0	0	0	1	34.0724638	1	0.49277013
2.9511	117930.669	0.85966527	223.34375	1.68433722	0	1.08217798	4.77010538	-0.1500496	5.40871654	0.52196568	-6.9077553	10	57	3.57631111	298	25	0	0	0	1	34.0724638	1	0.49277013
3.1113	124970.552	0.83377881	186.936995	1.33801732	0	1.13504065	4.82808613	-0.1805885	5.23077699	0.291936	-6.9077553	11	67	1.78815556	295.777778	22.7777778	0	0	0	1	34.0724638	1	0.49277013
3.2724	131367.834	0.74931719	151.928105	1.30507451	0	1.18552366	4.87800889	-0.2872592	5.023414	0.26702608	-6.9077553	12	70	1.78815556	295.222222	22.2222222	0	0	0	1	34.0724638	1	0.49277013
3.4011	136483.992	0.7138053	136.87022	1.18855883	0	1.22409891	4.91621466	-0.3357451	4.91904048	0.17358251	-6.9077553	13	75	1.78815556	294.111111	21.11111111	0	0	0	1	34.0724638	1	0.49277013
3.4974	140476.145	0.57022715	91.3118064	1.05783088	0	1.25201984	4.94504481	-0.5599683	4.51429104	0.05716536	-6.9077553	14	79	2.23519444	293.555556	20.55555555	0	0	0	1	34.0724638	1	0.49277013
3.519	141353.428	0.24093713	26.687055	0.49272458	0	1.25817686	4.95127041	-1.4190774	3.28421609	-0.7057775	-6.9077553	15	83	3.12927222	293	20	0	0	0	1	34.0724638	1	0.49277013
3.5415	142142.965	0.1824353	6.76238281	0.42585468	0	1.26455037	4.95684039	-1.6958932	1.91152318	-0.8513116	-6.9077553	16	87	3.57631111	293	20	0	0	0	1	34.0724638	1	0.49277013
3.5676	143371.159	0.15629119	4.89943248	0.29051583	0	1.2718931	4.96544376	-1.8496565	1.58932346	-1.232661	-6.9077553	17	91	3.57631111	292.444444	19.44444444	0	0	0	1	34.0724638	1	0.49277013
3.5883	143941.389	0.25747618	23.0702541	0.35128245	0	1.27767855	4.96941314	-1.3529517	3.13858743	-1.043322	-6.9077553	18	94	3.12927222	291.888889	18.88888888	0	0	0	1	34.0724638	1	0.49277013
3.6144	144476.57	0.52699737	122.828706	1.20612427	0	1.28492587	4.97312427	-0.638664	4.81079889	0.1882409	-6.9077553	19	93	4.02335	291.888889	18.88888888	0	0	0	1	34.0724638	1	0.49277013
3.6378	145002.927	0.38934058	68.9729098	0.78021732	0	1.2913791	4.97676082	-0.9407356	4.23372831	-0.2469019	-6.9077553	20	90	4.91742778	291.888889	18.88888888	0	0	0	1	34.0724638	1	0.49277013
3.6594	145529.305	0.48339725	107.446838	1.14187439	0	1.2972992	4.98038434	-0.72485	4.67700551	0.13354649	-6.9077553	21	90	3.57631111	291.888889	18.88888888	0	0	0	1	34.0724638	1	0.49277013
3.6738	146099.527	0.13064583	3.26731133	0.16261362	0	1.30122655	4.98429492	-2.0276401	1.18427344	-1.8102476	-6.9077553	22	89	4.47038889	291.888889	18.88888888	0	0	0	1	34.0724638	1	0.49277013
3.6837	146450.437	0.13527574	3.42676995	0.1653379	0	1.30391768	4.98669389	-1.9930749	1.23190989	-1.793734	-6.9077553	23	90	4.47038889	291.888889	18.88888888	0	0	0	1	34.0724638	1	0.49277013
0.0414	2817.58469	1.18299459	390.473438	2.58294	0	-3.1844744	1.03623488	0.16889397	5.96736251	0.94931536	-6.9077553	0	89	3.57631111	291.888889	18.88888888	0	0	0	0	42.288408	1	-0.4111751
0.1719	8088.40472	0.98491674	278.189063	3.56811818	0	-1.7608424	2.09055515	-0.0141834	5.62830456	1.27231856	-6.9077553	1	79	5.36446667	294.111111	21.11111111	0	0	0	0	42.288408	1	-0.4111751
0.4113	18664.674	1.01086976	285.009986	2.63250962	0	-0.8884324	2.92668622	0.01179987	5.65252773	0.96831741	-6.9077553	2	73	6.70558333	295.222222	22.2222222	0	0	0	0	42.288408	1	-0.4111751
0.6831	29549.0846	0.92281155	225.552928	2.13636022	0	-0.381114	3.38608661	-0.0792472	5.41855928	0.75957153	-6.9077553	3	66	4.91742778	297.444444	24.44444444	0	0	0	0	42.288408	1	-0.4111751
1.0206	43474.9468	0.88431991	207.262979	1.85075179	0	0.02039069	3.77220784	-0.1218062	5.33399324	0.6161321	-6.9077553	4	64	5.36446667	298	25	0	0	0	0	42.288408	1	-0.4111751
1.4283	58994.1508	0.90172302	228.585075	2.21417088	0	0.35648493	4.07745525	-0.1023395	5.43191284	0.79532955	-6.9077553	5	59	4.91742778	299.111111	26.11111111	0	0	0	0	42.288408	1	-0.4111751
2.0772	86805.8426	1.01648863	299.365378	3.04611346	0	0.73102083	4.46368545	0.01733747	5.70166817	1.11419474	-6.9077553	6	53	6.25854444	300.222222	27.2222222	0	0	1	0	42.288408	1	-0.4111751
2.7009	116591.005	0.99836059	286.116026	2.31913073	0	0.99358505	4.7586807	-0.0006396	5.65640091	0.84162353	-6.9077553	7	50	6.25854444	300.777778	27.7777778	0	0	1	0	42.288408	1	-0.4111751
3.3057	143876.861	0.9647086	270.421038	2.26655524	0	1.19564825	4.96896475	-0.0348931	5.59998384	0.81870226	-6.9077553	8	53	4.91742778	300.222222	27.2222222	0	0	1	0	42.288408	1	-0.4111751
3.7845	162828.68	0.88066635	233.89926	1.91144671	0	1.33091378	5.09270475	-0.1259416	5.45489478	0.64838342	-6.9077553	9	53	4.91742778	299.666667	26.66666666	0	0	1	0	42.288408	1	-0.4111751
4.176	182425.84	0.94762664	265.029849	1.91288866	0	1.42935385	5.20634922	-0.05274	5.57984623	0.64913712	-6.9077553	10	57	3.57631111	298	25	0	0	1	0	42.288408	1	-0.4111751
4.3938	193880.46	0.91699301	231.714166	1.62217221	0	1.48019446	5.26724694	-0.0855655	5.44550888	0.48438239	-6.9077553	11	67	1.78815556	295.777778	22.7777778	0	0	1	0	42.288408	1	-0.4111751
4.5765	201861.978	0.81053891	177.86815	1.38893526	0	1.52093451	5.30758914	-0.2088229	5.18104817	0.32925717	-6.9077553	12	70	1.78815556	295.222222	22.2222222	0	0	1	0	42.288408	1	-0.4111751
4.77	210406.746	0.76847719	156.502326	1.33845896	0	1.5623463	5.34904729	-0.262044	5.05307726	0.29226577	-6.9077553	13	75	1.78815556	294.111111	21.11111111	0	0	1	0	42.288408	1	-0.4111751
4.9095	216328.396	0.59082459	90.677224	1.00483428	0	1.5911721	5.37680223	-0.524545	4.50731724	0.00581733	-6.9077553	14	79	2.23519444	293.555556	20.55555555	0	0	1	0	42.288408	1	-0.4111751
4.9671	218477.718	0.21344903	9.79961109	0.38397458	0	1.60283617	5.38668861	-1.5396832	2.28244474	-0.954578	-6.9077553	15	83	3.12927222	293	20	0	0	1	0	42.288408	1	-0.4111751
5.0175	220627.048	0.19802049	8.43294634	0.31445353	0	1.6129318	5.39647824	-1.6143475	2.13226479	-1.1537439	-6.9077553	16	87	3.57631111	293	20	0	0	1	0	42.288408	1	-0.4111751
5.058	222293.874	0.1928034	7.92064073	0.33023879	0	1.62097115	5.40400476	-1.640911	2.06959835	-1.1049158	-6.9077553	17	91	3.57631111	292.444444	19.44444444	0	0	1	0	42.288408	1	-0.4111751
5.0967	224004.562	0.17991964	6.78980344	0.31057167	0	1.62859327	5.41167088	-1.7097023	1.91556926	-1.1661259	-6.9077553	18	94	3.12927222	291.888889	18.88888888	0	0	1	0	42.288408	1	-0.4111751
5.1453	226110.031	0.16513729	5.64997186	0.26055815	0	1.63808368	5.42102617	-1.7949408	1.73182754	-1.3410987	-6.9077553	19	93	4.02335	291.888889	18.88888888	0	0	1	0	42.288408	1	-0.4111751

5.1777	227382.075	0.1720238	6.38268238	0.30301078	0	1.64436094	5.42663615	-1.7543261	1.85374511	-1.1906921	-6.9077553	20	90	4.91742778	291.888889	18.88888888	0	0	1	0	42.288408	1	-0.4111751
5.211	228829.571	0.16264516	5.52036925	0.24848564	0	1.65077178	5.43298187	-1.8100549	1.70862588	-1.3883539	-6.9077553	21	90	3.57631111	291.888889	18.88888888	0	0	1	0	42.288408	1	-0.4111751
5.238	230013.893	0.15619844	5.12863871	0.23662841	0	1.65593975	5.43814406	-1.8502463	1.63503523	-1.4370471	-6.9077553	22	89	4.47038889	291.888889	18.88888888	0	0	1	0	42.288408	1	-0.4111751
5.2713	231592.993	0.15861411	5.07166888	0.21883624	0	1.66227701	5.44498581	-1.8349962	1.62386709	-1.5148724	-6.9077553	23	90	4.47038889	291.888889	18.88888888	0	0	1	0	42.288408	1	-0.4111751
0.0081	0	0.42014515	46.4625969	1.70399749	0	-4.8158912	-6.9077553	-0.8647777	3.83866914	0.53356364	-6.9077553	0	89	3.57631111	291.888889	18.88888888	0	0	1	0	18.9135952	1	-0.0428739
0.0081	0	0	0	0	0	-4.8158912	-6.9077553	-6.9077553	-6.9077553	-6.9077553	-6.9077553	1	79	5.36446667	294.111111	21.11111111	0	0	0	0	18.9135952	1	-0.0428739
0.0756	0	0.52301219	74.0915039	2.77609558	0	-2.582299	-6.9077553	-0.6462403	4.30531437	1.02140563	-6.9077553	2	73	6.70558333	295.222222	22.2222222	0	0	0	0	18.9135952	1	-0.0428739
0.2916	3805.70136	0.77409884	191.906966	2.86696269	0	-1.2323723	1.33676303	-0.2547647	5.25701591	1.05360191	-6.9077553	3	66	4.91742778	297.444444	24.44444444	0	0	1	0	18.9135952	1	-0.0428739
0.4869	10552.0552	0.84295654	243.560898	2.38705149	0	-0.7196965	2.35641541	-0.1696543	5.49537111	0.87047776	-6.9077553	4	64	5.36446667	298	25	0	0	1	0	18.9135952	1	-0.0428739
0.6363	14004.8007	0.69116615	158.825007	1.49628862	0	-0.4520851	2.63947158	-0.3679292	5.06780931	0.40365589	-6.9077553	5	59	4.91742778	299.111111	26.11111111	0	0	1	0	18.9135952	1	-0.0428739
0.8235	18416.9507	0.75789547	174.29248	1.99432799	0	-0.1941917	2.91332577	-0.2758912	5.16074055	0.69080844	-6.9077553	6	53	6.25854444	300.222222	27.2222222	0	0	1	0	18.9135952	1	-0.0428739
1.0341	24685.1179	0.75422395	169.345286	1.52257636	0	0.03353148	3.20624106	-0.280741	5.13194565	0.42106044	-6.9077553	7	50	6.25854444	300.777778	27.7777778	0	0	1	0	18.9135952	1	-0.0428739
1.3059	35537.1663	0.85300511	224.540589	1.90414934	0	0.26689246	3.57060723	-0.1578181	5.41406094	0.6445604	-6.9077553	8	53	4.91742778	300.222222	27.2222222	0	0	1	0	18.9135952	1	-0.0428739
1.5075	42339.1605	0.77165576	176.101946	1.5441449	0	0.41045265	3.74573606	-0.2579217	5.17106875	0.43511769	-6.9077553	9	53	4.91742778	299.666667	26.66666666	0	0	1	0	18.9135952	1	-0.0428739
1.7109	50224.5797	0.81167528	194.28787	1.45302643	0	0.53701955	3.91652445	-0.2074237	5.26934607	0.37433655	-6.9077553	10	57	3.57631111	298	25	0	0	1	0	18.9135952	1	-0.0428739
1.8864	58089.1288	0.91411368	225.83748	1.63256632	0	0.63467025	4.06199575	-0.088707	5.41982005	0.49076555	-6.9077553	11	67	1.78815556	295.777778	22.7777778	0	0	1	0	18.9135952	1	-0.0428739
2.0115	64048.2813	0.86265849	202.491134	1.59313174	0	0.69888071	4.15965281	-0.1465779	5.31070104	0.46632923	-6.9077553	12	70	1.78815556	295.222222	22.2222222	0	0	1	0	18.9135952	1	-0.0428739
2.115	68797.0306	0.7300695	142.6616	1.25788614	0	0.74905481	4.23117512	-0.3132467	4.9604824	0.23022731	-6.9077553	13	75	1.78815556	294.111111	21.11111111	0	0	1	0	18.9135952	1	-0.0428739
2.1969	72349.992	0.55382613	79.7348096	0.91681881	0	0.78704728	4.28152916	-0.5891005	4.37871879	-0.0857553	-6.9077553	14	79	2.23519444	293.555556	20.55555555	0	0	1	0	18.9135952	1	-0.0428739
2.2302	73841.3606	0.25790702	15.2146491	0.4987812	0	0.80209127	4.30193256	-1.3512863	2.72232444	-0.6935849	-6.9077553	15	83	3.12927222	293	20	0	0	1	0	18.9135952	1	-0.0428739
2.2626	75288.867	0.21673897	10.0682882	0.38951609	0	0.81651459	4.32134556	-1.5244583	2.30949002	-0.9402861	-6.9077553	16	87	3.57631111	293	20	0	0	1	0	18.9135952	1	-0.0428739
2.2896	76517.0594	0.20291564	8.83355713	0.33488696	0	0.82837713	4.33752678	-1.5900489	2.17867098	-1.0909806	-6.9077553	17	91	3.57631111	292.444444	19.44444444	0	0	1	0	18.9135952	1	-0.0428739
2.3229	78052.2868	0.18842384	7.42485583	0.31378885	0	0.84281641	4.35739176	-1.6637682	2.00496794	-1.1558532	-6.9077553	18	94	3.12927222	291.888889	18.88888888	0	0	1	0	18.9135952	1	-0.0428739
2.3499	79192.7446	0.18151671	6.5927536	0.26789316	0	0.85437277	4.37189731	-1.7009135	1.88612278	-1.3134412	-6.9077553	19	93	4.02335	291.888889	18.88888888	0	0	1	0	18.9135952	1	-0.0428739
2.3769	80464.7947	0.1812192	6.56245321	0.27727432	0	0.86579712	4.38783218	-1.7025449	1.88151687	-1.2791479	-6.9077553	20	90	4.91742778	291.888889	18.88888888	0	0	1	0	18.9135952	1	-0.0428739
2.4003	81473.66	0.15459374	4.85346956	0.24723253	0	0.87559373	4.40029205	-1.8605069	1.57989984	-1.3933894	-6.9077553	21	90	3.57631111	291.888889	18.88888888	0	0	1	0	18.9135952	1	-0.0428739
2.4237	82482.5254	0.15793857	4.96932162	0.21962611	0	0.8852953	4.41259858	-1.8392375	1.60348455	-1.5112858	-6.9077553	22	89	4.47038889	291.888889	18.88888888	0	0	1	0	18.9135952	1	-0.0428739
2.4453	83447.534	0.16152832	5.11425654	0.21888471	0	0.89416781	4.42423008	-1.816903	1.63222755	-1.5146519	-6.9077553	23	90	4.47038889	291.888889	18.88888888	0	0	1	0	18.9135952	1	-0.0428739
0.0171	502.523265	0.84101857	194.214817	1.72113959	0	-4.0686768	-0.6861254	-0.1719532	5.26897	0.54356747	-6.9077553	0	36	3.12927222	290.222222	17.2222222	0	0	0	0	30.519848	1	0.21141735
0.0711	1887.23114	0.76647383	182.396354	1.72390226	0	-2.6436679	0.63564049	-0.2646509	5.20618757	0.54517039	-6.9077553	1	31	3.57631111	293.555556	20.55555555	0	0	0	0	30.519848	1	0.21141735
0.2358	2976.19145	0.65421616	124.910188	3.26180847	0	-1.4447713	1.09098039	-0.4227901	4.82760299	1.18258832	-6.9077553	2	22	5.36446667	296.333333	23.33333333	0	0	0	0	30.519848	1	0.21141735
0.4005	4741.59582	0.77514065	175.029393	3.02089937	0	-0.9150415	1.55658463	-0.2534215	5.16495963	1.10588556	-6.9077553	3	20	5.81150556	297.444444	24.44444444	0	0	0	0	30.519848	1	0.21141735
0.6597	11774.267	0.78474236	172.946913	2.51016023	0	-0.4159701	2.46600132	-0.2411263	5.15299047	0.92074489	-6.9077553	4	18	4.91742778	298.555556	25.55555555	0	0	0	1	30.519848	1	0.21141735
0.9891	24306.3712	0.92151246	259.183829	3.04567964	0	-0.0109598	3.19077965	-0.0806544	5.55754143	1.11405236	-6.9077553	5	16	5.36446667	299.111111	26.11111111	0	0	0	1	30.519848	1	0.21141735
1.314	33827.4719	0.78802516	167.11037	2.11750231	0	0.27307592	3.52130281	-0.2369571	5.11866048	0.75070938	-6.9077553	6	18	5.36446667	298.555556	25.55555555	0	0	0	1	30.519848	1	0.21141735
1.5894	43173.7132	0.75789308	161.456419	1.40550182	0	0.46335659	3.76525498	-0.2758944	5.08424145	0.34110564	-6.9077553	7	25	2.68223333	295.222222	22.2222222	0	0	0	1	30.519848	1	0.21141735
1.8936	55098.1694	0.75345837	165.162063	1.45964177	0	0.63847978	4.00913464	-0.2817552	5.10693325	0.37887591	-6.9077553	8	29	1.34111667	293	20	0	0	0	1	30.519848	1	0.21141735
2.1762	65929.4211	0.73850518	157.627239	1.43451997	0	0.77758024	4.18859996	-0.301774	5.06023934	0.36152713	-6.9077553	9	31	1.34111667	290.777778	17.7777778	0	0	0	1	30.519848	1	0.21141735
2.4525	77034.8049	0.72169654	160.320236	1.55343698	0	0.89710791	4.34427031	-0.3247659	5.07717953	0.44111341	-6.9077553	10	33	2.68223333	290.222222	17.2222222	0	0	0	1	30.519848	1	0.21141735
2.6793	91112.0457	0.68714239	132.416729	1.33125886	0	0.98555557	4.512101	-0.3737595	4.88596154	0.2868759	-6.9077553	11	35	3.12927222	290.222222	17.2222222	0	0	0	1	30.519848	1	0.21141735
2.9214	112530.762	0.7135788	138.470783	1.25727476	0	1.07206295	4.72323551	-0.336062	4.93066657	0.22974154	-6.9077553	12	36	3.57631111	290.222222	17.2222222	0	0	0	1	30.519848	1	0.21141735
3.1131	132231.68	0.69999387	134.876357	1.16536236	0	1.13561901	4.8845631	-0.3552561	4.9043659	0.15388981	-6.9077553	13	40	3.57631111	289.111111	16.11111111	0	0	0	1	30.519848	1	0.21141735
3.2697	154834.417	0.68043115	124.924232	1.30854043	0	1.18469824	5.04236273	-0.3835601	4.82771542	0.26967626	-6.9077553	14	44	3.57631111	288	15	0	0	0	1	30.519848	1	0.21141735
3.4164	180023.659	0.65620413	119.980169	1.3535336	0	1.22858737	5.19309384	-0.4197606	4.78733481	0.30345719	-6.9077553	15	44	4.91742778	286.888889	13.88888888	0	0	0	1	30.519848	1	0.21141735
3.528	196588.084	0.60159763	97.4063996	1.08504978	0	1.26073114	5.28111568	-0.5065056	4.57890218	0.08254706	-6.9077553	16	51	4.02335	285.777778	12.77777777	0	0	0	1	30.519848	1	0.21141735
3.6162	215852.372	0.63350382	107.285585	0.71839858	0	1.28542375	5.37459934	-0.454912	4.67550362	-0.3293397	-6.9077553	17	63	2.23519444	284.666667	11.66666666	0	0	0	1	30.519848	1	0.21141735
3.6684	232222.838	0.63985811	101.670941	0.48322625	0	1.2997556	5.44770173	-0.4449472	4.62175136	-0.725203	-6.9077553	18	71	2.23519444	284.666667	11.66666666	0	0	0	1	30.519848	1	0.21141735
3.7044	245240.438	0.55766096	76.2556335	0.39588926	0	1.3095213	5.50224319	-0.5822215	4.33410441	-0.924098	-6.9077553	19	73	2.68223333	284.666667	11.66666666	0	0	0	1	30.519848	1	0.21141735
3.7449	259926.943	0.55478138	78.0751302	0.45712458	0	1.32039491	5.56040445	-0.5873803	4.35768438	-0.7806141	-6.9077553	20	79	3.57631111	284.666667	11.66666666	0	0	0	1	30.519848	1	0.21141735
3.7593	265267.512	0.47423112	53.6844406	0.34674287	0	1.32423277	5.58074257	-0.743954	3.98314184	-1.056292	-6.9077553	21	83	4.47038889	286.333333	13.33333333	0	0	0	1	30.519848	1	0.21141735
3.7593	265267.512	0	0	0	0	1.32423277	5.58074257	-6.9077553	-6.9077553	-6.9077553	-6.9077553	22	84	3.12927222	287.444444	14.44444444	0	0	0	1	30.519848	1	0.21141735
3.7755	269940.508	0.28558429	23.706031	0.39570318	0	1.32853282	5.5982053	-1.2497226	3.16577167	-0.9245669	-6.9077553	23	83	4.91742778	289.111111	16.11111111	0	0	0	1	30.519848	1	0.21141735
0.045	1837.22735	1.10980287	357.108179	3.13846261	0	-3.1010928	0.60880171	0.10508306	5.87804156	1.14405164	-6.9077553	0	36	3.12927222	290.222222	17.2222222	0	0	0	0	34.0724638	1	0.49277013
0.1278	5911.65061	0.95173073	244.061566	1.50701407	0	-2.0572887	1.77709423	-0.048423	5.49742461	0.4107936	-6.9077553	1	31	3.57631111	293.555556	20.55555555	0	0	0	0	34.0724638	1	0.49277013
0.2466	10623.8742	1.00509389	269.512932	2.33821383	0	-1.3999877	2.36319788	0.0060754	5.59662008	0.8498149	-6.9077553	2	22	5.36446667	296.333333	23.33333333	0	0	0	1	34.0724638	1	0.49277013
0.4437	22012.8264	1.3322871	523.769014	4.02832143	0	-0.8126066	3.09167073	0.28764739	6.26105268	1.39359798	-6.9077553	3	20	5.81150556	297.444444	24.44444444	0	0	0	1	34.0724638	1	0.49277013
0.8145	46625.6889	1.4209974	589.493704	4.36262646	0	-0.2051809	3.8421731	0.3520625	6.37926574	1.47330347	-6.9077553	4	18	4.91742778	298.555556	25.55555555	0	0	0	1	34.0724638	1	0.49277013
1.3257	64898.043	1.3774182	566.973261	4.3603593	1	0.28194062	4.17283288	0.32093661	6.34031391	1.47278377	0.0009995	5	16	5.36446667	299.111111	26.11111111	0	0	0	1	34.0724638	1	0.49277013
2.1069	90911.2242	1.27395475	480.980775	3.79873334	1	0.74521767	4.50989447	0.24291069	6.17582938	1.33493089	0.0009995	6	18	5.36446667	298.555556	25.55555555	0	0	0	1	34.0724638	1	0.49277013
2.6586	112290.01	1.02469305	302.716227	2.35376578	1	0.97779967	4.72109381	0.02536853	5.71279912	0.85643744	0.0009995	7	25	2.68223333	295.222222	22.2222222	0	0	0	1	34.0724638	1	0.49277013
3.2364	133049.351	0.96657137	263.87573	1.98882334	1	1.1744616	4.89072764	-0.0329661	5.57548206	0.68804586	0.0009995	8	29	1.34111667	293	20	0	0	0	1	34.0724638	1	0.49277013
3.7458	150059.646	0.93065479	244.648106	1.84442786	1	1.32063521	5.01103952	-0.0707929	5.49982496	0.61271115	0.0009995	9	31	1.34111667	290.777778	17.7777778	0	0	0	1	34.0724638	1	0.49277013

4.2399	162742.833	0.85291717	204.335496	1.9014539	1	1.44453968	5.09217739	-0.1579211	5.31976813	0.64314458	0.0009995	10	33	2.68223333	290.222222	17.2222222	0	0	0	1	34.0724638	1	0.49277013
4.7574	174150.115	0.85431025	211.474348	2.01358526	1	1.5597013	5.1599234	-0.156291	5.35410843	0.70041335	0.0009995	11	35	3.12927222	290.222222	17.2222222	0	0	0	1	34.0724638	1	0.49277013
5.229	186400.782	0.78154912	171.724535	1.55704096	1	1.65422006	5.22790446	-0.2451986	5.14589747	0.44342924	0.0009995	12	36	3.57631111	290.222222	17.2222222	0	0	0	1	34.0724638	1	0.49277013
5.7069	201218.099	0.78859726	173.033339	1.48088875	1	1.74167597	5.30439436	-0.2362323	5.15349007	0.39331746	0.0009995	13	40	3.57631111	289.111111	16.11111111	0	0	0	1	34.0724638	1	0.49277013
6.1749	217404.518	0.79942245	173.251998	1.47452322	1	1.82049269	5.38176436	-0.2226156	5.15475294	0.38901265	0.0009995	14	44	3.57631111	288	15	0	0	0	1	34.0724638	1	0.49277013
6.7185	232653.073	0.79774819	176.564052	1.94117707	1	1.90486492	5.44955269	-0.2247095	5.17368938	0.66380954	0.0009995	15	44	4.91742778	286.888889	13.88888888	0	0	0	1	34.0724638	1	0.49277013
7.3098	251461.03	0.8071526	190.899615	1.82288768	1	1.98921591	5.527292	-0.2130044	5.25175295	0.60097031	0.0009995	16	51	4.02335	285.777778	12.77777777	0	0	0	1	34.0724638	1	0.49277013
7.6779	267455.378	0.83810373	199.40136	1.51397332	1	2.03834607	5.58895648	-0.1754209	5.29532469	0.41539783	0.0009995	17	63	2.23519444	284.666667	11.66666666	0	0	0	1	34.0724638	1	0.49277013
7.9848	280490.324	0.86802186	201.88169	1.49686448	1	2.07753973	5.6365428	-0.140387	5.30768679	0.40404041	0.0009995	18	71	2.23519444	284.666667	11.66666666	0	0	0	1	34.0724638	1	0.49277013
8.2008	289622.577	0.77738152	159.688053	1.2895153	1	2.10423171	5.66858207	-0.2505385	5.07322851	0.2550416	0.0009995	19	73	2.68223333	284.666667	11.66666666	0	0	0	1	34.0724638	1	0.49277013
8.3547	296224.696	0.72437282	136.438151	1.13405703	1	2.12282425	5.69112165	-0.3210695	4.91587874	0.12668289	0.0009995	20	79	3.57631111	284.666667	11.66666666	0	0	0	1	34.0724638	1	0.49277013
8.4483	300216.284	0.58296842	86.2165903	0.88364748	1	2.13396524	5.70450649	-0.5379084	4.45687422	-0.122566	0.0009995	21	83	4.47038889	286.333333	13.33333333	0	0	0	1	34.0724638	1	0.49277013
8.4717	301312.897	0.20620606	8.77876575	0.33811232	1	2.1367312	5.70815257	-1.5740415	2.17244973	-1.0814239	0.0009995	22	84	3.12927222	287.444444	14.44444444	0	0	0	1	34.0724638	1	0.49277013
8.5968	306357.22	0.46630925	63.30428	0.89554514	1	2.15139004	5.72475507	-0.760764	4.14796874	-0.1092066	0.0009995	23	83	4.91742778	289.111111	16.11111111	0	0	0	1	34.0724638	1	0.49277013
0.0531	4001.40195	1.32962049	499.793979	2.94784388	0	-2.9355784	1.38689467	0.28564537	6.21419797	1.08141319	-6.9077553	0	36	3.12927222	290.222222	17.2222222	0	0	0	0	42.288408	1	-0.4111751
0.252	13712.5072	1.1771736	391.56045	3.73849391	0	-1.3783262	2.61838127	0.16396544	5.97014246	1.31895028	-6.9077553	1	31	3.57631111	293.555556	20.55555555	0	0	0	0	42.288408	1	-0.4111751
0.5544	28164.3468	1.12444124	348.919271	2.89642152	0	-0.5898688	3.33809239	0.11817517	5.85484345	1.06382121	-6.9077553	2	22	5.36446667	296.333333	23.33333333	0	0	0	0	42.288408	1	-0.4111751
0.9567	47067.1317	1.06029755	306.752901	2.6069904	0	-0.0442654	3.85159616	0.05949226	5.7260458	0.95857996	-6.9077553	3	20	5.81150556	297.444444	24.44444444	0	0	0	0	42.288408	1	-0.4111751
1.5885	74201.2775	1.09146405	342.473602	3.25059609	0	0.46279017	4.30679484	0.08843574	5.8361975	1.17914598	-6.9077553	4	18	4.91742778	298.555556	25.55555555	0	0	1	0	42.288408	1	-0.4111751
2.3022	109447.848	1.1037259	351.145571	2.78171673	0	0.83386519	4.6954573	0.09959725	5.86120372	1.02342769	-6.9077553	5	16	5.36446667	299.111111	26.11111111	0	0	1	0	42.288408	1	-0.4111751
3.0141	145843.585	1.11393396	360.667075	2.55092601	0	1.10330128	4.98254157	0.10879518	5.88795808	0.93684837	-6.9077553	6	18	5.36446667	298.555556	25.55555555	0	0	1	0	42.288408	1	-0.4111751
3.6873	174167.134	1.00040595	291.550092	2.27100948	0	1.30489448	5.16002112	0.00140496	5.67521526	0.82066467	-6.9077553	7	25	2.68223333	295.222222	22.2222222	0	0	1	0	42.288408	1	-0.4111751
4.2192	198255.74	0.93928954	256.151703	1.90767374	0	1.43964554	5.28956286	-0.0615674	5.54577376	0.64640862	-6.9077553	8	29	1.34111667	293	20	0	0	1	0	42.288408	1	-0.4111751
4.7142	218223.318	0.86323919	215.390923	1.74067904	0	1.55057923	5.38552352	-0.1459057	5.37245927	0.55484962	-6.9077553	9	31	1.34111667	290.777778	17.7777778	0	0	1	0	42.288408	1	-0.4111751
5.247	238644.458	0.85886239	215.562434	1.70474181	0	1.65765648	5.47497901	-0.1509829	5.37325522	0.5340001	-6.9077553	10	33	2.68223333	290.222222	17.2222222	0	0	1	0	42.288408	1	-0.4111751
5.7447	256486.063	0.82624064	200.278495	1.63631457	0	1.74827769	5.54707823	-0.1896597	5.29971387	0.49305744	-6.9077553	11	35	3.12927222	290.222222	17.2222222	0	0	1	0	42.288408	1	-0.4111751
6.183	268572.115	0.69107118	142.368872	1.34884625	0	1.82180359	5.59312318	-0.3680665	4.9584284	0.2999907	-6.9077553	12	36	3.57631111	290.222222	17.2222222	0	0	1	0	42.288408	1	-0.4111751
6.5862	279454.689	0.68039594	146.855225	1.39782579	0	1.88497655	5.63284374	-0.3836117	4.98945404	0.33563316	-6.9077553	13	40	3.57631111	289.111111	16.11111111	0	0	1	0	42.288408	1	-0.4111751
6.9489	289527.37	0.64087641	132.897681	1.22392193	0	1.93858337	5.66825329	-0.4433595	4.88958704	0.20287711	-6.9077553	14	44	3.57631111	288	15	0	0	1	0	42.288408	1	-0.4111751
7.3413	298078.583	0.68039941	161.162217	1.52507299	0	1.99351594	5.69736051	-0.3836066	5.08241762	0.42269776	-6.9077553	15	44	4.91742778	286.888889	13.88888888	0	0	1	0	42.288408	1	-0.4111751
7.6581	306619.198	0.65727338	140.736628	1.2206166	0	2.03576391	5.72560984	-0.418135	4.94689736	0.20017506	-6.9077553	16	51	4.02335	285.777778	12.77777777	0	0	1	0	42.288408	1	-0.4111751
7.8372	313463.638	0.75631399	164.381438	1.17278892	0	2.05888163	5.74768656	-0.2779773	5.10219565	0.16023691	-6.9077553	17	63	2.23519444	284.666667	11.66666666	0	0	1	0	42.288408	1	-0.4111751
7.9434	317884.493	0.81138785	178.101993	1.43449777	0	2.0723414	5.76169123	-0.2077774	5.18236199	0.36151167	-6.9077553	18	71	2.23519444	284.666667	11.66666666	0	0	1	0	42.288408	1	-0.4111751
8.0298	321500.773	0.77357785	162.502889	1.38987319	0	2.08315962	5.77300306	-0.2554371	5.09070193	0.32993174	-6.9077553	19	73	2.68223333	284.666667	11.66666666	0	0	1	0	42.288408	1	-0.4111751
8.0991	324138.323	0.7425606	152.021472	1.31754917	0	2.09175294	5.78117343	-0.296305	5.02402835	0.27653202	-6.9077553	20	79	3.57631111	284.666667	11.66666666	0	0	1	0	42.288408	1	-0.4111751
8.1522	326480.037	0.60663359	99.0098484	0.99916148	0	2.09828783	5.78837187	-0.4981832	4.59522942	0.00016146	-6.9077553	21	83	4.47038889	286.333333	13.33333333	0	0	1	0	42.288408	1	-0.4111751
8.1693	327138.01	0.16636331	5.84821842	0.42143039	0	2.10038323	5.79038519	-1.7875883	1.76630805	-0.8617306	-6.9077553	22	84	3.12927222	287.444444	14.44444444	0	0	1	0	42.288408	1	-0.4111751
8.2512	329977.636	0.45296026	69.7802842	0.94705318	0	2.11035864	5.79902791	-0.7897456	4.24536584	-0.0533447	-6.9077553	23	83	4.91742778	289.111111	16.11111111	0	0	1	0	42.288408	1	-0.4111751
0.0117	0	0.51139458	72.3967692	2.07208411	0	-4.4481664	-6.9077553	-0.6686603	4.28217549	0.72903741	-6.9077553	0	36	3.12927222	290.222222	17.2222222	0	0	0	0	18.9135952	1	-0.0428739
0.1116	0	0.61006914	99.4567409	2.91151332	0	-2.1928342	-6.9077553	-0.4925452	4.59973284	1.06901639	-6.9077553	1	31	3.57631111	293.555556	20.55555555	0	0	0	0	18.9135952	1	-0.0428739
0.3501	3269.26998	0.78544502	211.819428	3.48799381	0	-1.0495365	1.18487254	-0.2402325	5.35573888	1.24961339	-6.9077553	2	22	5.36446667	296.333333	23.33333333	0	0	1	0	18.9135952	1	-0.0428739
0.5868	13175.2319	1.03381557	380.422529	2.87886053	0	-0.5330712	2.57841459	0.03422321	5.94128518	1.05774187	-6.9077553	3	20	5.81150556	297.444444	24.44444444	0	0	1	0	18.9135952	1	-0.0428739
0.7803	17656.7799	0.82176883	213.208594	1.8570568	0	-0.2480768	2.87117647	-0.19508	5.36227569	0.61953121	-6.9077553	4	18	4.91742778	298.555556	25.55555555	0	0	1	0	18.9135952	1	-0.0428739
1.0557	26218.3217	0.89807937	253.274867	2.33638549	0	0.05420405	3.26649661	-0.106384	5.53447928	0.84903299	-6.9077553	5	16	5.36446667	299.111111	26.11111111	0	0	1	0	18.9135952	1	-0.0428739
1.4049	40315.0166	1.00150315	307.682426	2.59671398	0	0.33996613	3.69674882	0.00250002	5.72907142	0.95463182	-6.9077553	6	18	5.36446667	298.555556	25.55555555	0	0	1	0	18.9135952	1	-0.0428739
1.7397	51747.9544	0.89278092	239.447287	1.94853752	0	0.55371268	3.94640423	-0.1122946	5.47833747	0.66759217	-6.9077553	7	25	2.68223333	295.222222	22.2222222	0	0	1	0	18.9135952	1	-0.0428739
2.043	63113.0597	0.80896548	201.030276	1.56619199	0	0.71441932	4.14494356	-0.2107636	5.3034605	0.44928548	-6.9077553	8	29	1.34111667	293	20	0	0	1	0	18.9135952	1	-0.0428739
2.4003	75518.8766	0.82522929	208.602645	1.69614086	0	0.87559373	4.32439589	-0.190883	5.34043602	0.52894499	-6.9077553	9	31	1.34111667	290.777778	17.7777778	0	0	1	0	18.9135952	1	-0.0428739
2.8521	93158.0627	0.87091648	228.778215	1.95560665	0	1.04805557	4.53430838	-0.1370616	5.43275741	0.67121167	-6.9077553	10	33	2.68223333	290.222222	17.2222222	0	0	1	0	18.9135952	1	-0.0428739
3.285	107968.4	0.8310873	205.367236	1.89444598	0	1.18936665	4.68184785	-0.1838179	5.32480464	0.63945416	-6.9077553	11	35	3.12927222	290.222222	17.2222222	0	0	1	0	18.9135952	1	-0.0428739
3.735	122685.051	0.80231366	193.725906	1.83840979	0	1.31774782	4.80962866	-0.21901	5.26644947	0.60944475	-6.9077553	12	36	3.57631111	290.222222	17.2222222	0	0	1	0	18.9135952	1	-0.0428739
4.1796	138660.545	0.80450192	189.682771	1.59368612	0	1.43021555	4.93203604	-0.2162897	5.24535832	0.46667693	-6.9077553	13	40	3.57631111	289.111111	16.11111111	0	0	1	0	18.9135952	1	-0.0428739
4.6494	157267.26	0.83080236	205.735542	1.71046161	0	1.53673818	5.05795301	-0.1841604	5.32659643	0.53734775	-6.9077553	14	44	3.57631111	288	15	0	0	1	0	18.9135952	1	-0.0428739
5.1201	174517.467	0.81517545	199.489125	1.88080009	0	1.63317397	5.16203057	-0.2031259	5.29576474	0.63222881	-6.9077553	15	44	4.91742778	286.888889	13.88888888	0	0	1	0	18.9135952	1	-0.0428739
5.5395	187995.419	0.71265408	147.873525	1.41565269	0	1.71190424	5.23642291	-0.3373569	4.99636411	0.34829683	-6.9077553	16	51	4.02335	285.777778	12.77777777	0	0	1	0	18.9135952	1	-0.0428739
5.8221	199964.792	0.8135928	180.094285	1.26622728	0	1.76166102	5.29814631	-0.2050669	5.19348607	0.23683127	-6.9077553	17	63	2.23519444	284.666667	11.66666666	0	0	1	0	18.9135952	1	-0.0428739
6.1038	212849.534	0.85913973	196.700804	1.41951003	0	1.80891153	5.3605902	-0.1506604	5.2816889	0.35101599	-6.9077553	18	71	2.23519444	284.666667	11.66666666	0	0	1	0	18.9135952	1	-0.0428739
6.4251	228017.007	0.85554654	196.13511	1.51084152	0	1.86021219	5.42942461	-0.1548466	5.27880886	0.41332846	-6.9077553	19	73	2.68223333	284.666667	11.66666666	0	0	1	0	18.9135952	1	-0.0428739
6.7338	243046.26	0.82261849	180.480924	1.43075766	0	1.90713962	5.49325591	-0.1940479	5.19563063	0.35890282	-6.9077553	20	79	3.57631111	284.666667	11.66666666	0	0	1	0	18.9135952	1	-0.0428739
6.9723	254259.526	0.68652885	127.412323	1.23250974	0	1.94194516	5.53835943	-0.3746515	4.84743631	0.20986356	-6.9077553	21	83	4.47038889	286.333333	13.33333333	0	0	1	0	18.9135952	1	-0.0428739
7.0479	257637.783	0.30444593	30.4855986	0.53245667	0	1.9527297	5.55155854	-1.1859825	3.4172872	-0.6283774	-6.9077553	22	84	3.12927222	287.444444	14.44444444	0	0	1	0	18.9135952	1	-0.0428739
7.3305	271056.282	0.74128684	167.492598	1.45993499	0	1.99204373	5.60233017	-0.2980195	5.12094513	0.37907663	-6.9077553	23	83	4.91742778	289.111111	16.11111111	0	0	1	0	18.9135952	1	-0.0428739

0.0063	223.075007	0.91918349	227.976501	1.51119598	0	-5.0672056	-1.4957744	-0.0831822	5.42924695	0.41356288	-6.9077553	0	92	1.34111667	298	25	0	0	0	0	30.519848	1	0.21141735
0.0126	486.257718	0.52588674	65.3026908	0.87050281	0	-4.3740585	-0.7189621	-0.6407697	4.17904856	-0.1375362	-6.9077553	1	95	1.34111667	297.444444	24.44444444	0	0	0	0	30.519848	1	0.21141735
0.0288	1056.48696	0.58571815	87.7140842	0.98249679	0	-3.5473799	0.0558953	-0.5332107	4.47409388	-0.0166409	-6.9077553	2	96	2.23519444	297.444444	24.44444444	0	0	0	0	30.519848	1	0.21141735
0.036	1231.94207	0.61621583	95.9885788	1.15354276	0	-3.3242363	0.20940324	-0.4825365	4.56423963	0.14370438	-6.9077553	3	98	1.78815556	297.444444	24.44444444	0	0	0	0	30.519848	1	0.21141735
0.045	1582.85223	0.32800605	27.3068604	0.8789279	0	-3.1010928	0.45986	-1.1116791	3.30717459	-0.1279153	-6.9077553	4	93	3.12927222	298.555556	25.55555555	0	0	0	0	30.519848	1	0.21141735
0.0936	1670.57985	0.32622888	29.1766719	1.31070201	0	-2.3687249	0.5137692	-1.1170954	3.37340376	0.27132554	-6.9077553	5	80	4.02335	300.777778	27.7777778	0	0	0	0	30.519848	1	0.21141735
0.216	2237.11887	0.53256607	83.168378	2.34043166	0	-1.5324769	0.80563572	-0.6281724	4.42087923	0.85076256	-6.9077553	6	72	4.47038889	302.444444	29.44444444	0	0	0	0	30.519848	1	0.21141735
0.2997	3359.19236	0.63746504	117.202894	1.94448508	0	-1.2049733	1.21199822	-0.4486884	4.7639151	0.66551134	-6.9077553	7	62	3.57631111	304.111111	31.11111111	0	0	0	0	30.519848	1	0.21141735
0.4059	4232.35264	0.58420686	91.1134964	1.6238715	0	-0.9016485	1.44299427	-0.5357899	4.51211692	0.48542873	-6.9077553	8	55	3.12927222	305.777778	32.7777778	0	0	0	1	30.519848	1	0.21141735
0.5625	5441.51538	0.6177918	100.771496	1.76719981	0	-0.5753641	1.69424134	-0.4799864	4.61286546	0.56996197	-6.9077553	9	50	3.57631111	306.888889	33.88888888	0	0	0	1	30.519848	1	0.21141735
0.7614	8587.76641	0.66077627	124.949201	1.71683651	0	-0.2725964	2.15045512	-0.4128277	4.82791527	0.54106565	-6.9077553	10	46	2.68223333	307.444444	34.4444444	0	0	0	1	30.519848	1	0.21141735
1.008	14091.3742	0.75914013	165.239807	1.98464342	0	0.00796817	2.64563382	-0.2742525	5.10740384	0.685943	-6.9077553	11	41	2.68223333	309.111111	36.11111111	0	0	0	1	30.519848	1	0.21141735
1.2573	19114.7839	0.78658231	176.706044	1.89299942	0	0.22896656	2.95051438	-0.2387874	5.17449324	0.63869069	-6.9077553	12	38	3.12927222	309.666667	36.66666666	0	0	0	1	30.519848	1	0.21141735
1.53	24303.1046	0.76109913	161.51908	1.81676521	0	0.42526774	3.19064525	-0.2716786	5.08462947	0.59760784	-6.9077553	13	36	3.12927222	309.111111	36.11111111	0	0	0	1	30.519848	1	0.21141735
1.7667	30092.266	0.77924193	168.199026	1.57213543	0	0.5691134	3.40430142	-0.2481512	5.1251539	0.45307072	-6.9077553	14	38	3.12927222	308.555556	35.5555556	0	0	0	1	30.519848	1	0.21141735
1.9899	35981.7881	0.84316967	196.178553	1.57946457	0	0.68808439	3.58304072	-0.1694018	5.27903033	0.45771884	-6.9077553	15	42	1.34111667	308	35	0	0	0	1	30.519848	1	0.21141735
2.1708	41112.7682	0.78724792	174.495044	1.4144275	0	0.77509576	3.71634306	-0.2379426	5.16190207	0.3474316	-6.9077553	16	54	1.34111667	304.666667	31.66666666	0	0	0	1	30.519848	1	0.21141735
2.277	44447.5151	0.75011509	168.249636	1.23816616	0	0.82285879	3.79433156	-0.2861964	5.12545475	0.2144387	-6.9077553	17	61	0.89407778	303	30	0	0	0	1	30.519848	1	0.21141735
2.3373	46567.5268	0.8567268	200.079466	1.37160048	0	0.84899642	3.84092492	-0.1534696	5.29871962	0.3167071	-6.9077553	18	66	1.34111667	301.888889	28.88888888	0	0	0	1	30.519848	1	0.21141735
2.3679	47356.4542	0.77101298	165.525103	1.2691638	0	0.86200349	3.85772423	-0.2587539	5.10912891	0.23914587	-6.9077553	19	66	1.34111667	301.333333	28.33333333	0	0	0	1	30.519848	1	0.21141735
2.3832	47784.3476	0.71011285	137.763413	1.12105179	0	0.86844412	3.86671906	-0.3409241	4.92554508	0.11515896	-6.9077553	20	69	1.34111667	300.777778	27.7777778	0	0	0	1	30.519848	1	0.21141735
2.3895	47811.1745	0.45200665	52.9467904	0.69028786	0	0.87108414	3.8672803	-0.7918485	3.96930634	-0.369199	-6.9077553	21	72	1.78815556	299.666667	26.66666666	0	0	0	1	30.519848	1	0.21141735
2.394	47874.2053	0.37564061	32.8398163	0.26408572	0	0.87296561	3.86859774	-0.9764638	3.49167214	-1.327702	-6.9077553	22	78	2.23519444	299.666667	26.66666666	0	0	0	1	30.519848	1	0.21141735
2.394	47874.2053	0	0	0	0	0.87296561	3.86859774	-6.9077553	-6.9077553	-6.9077553	-6.9077553	23	83	2.23519444	299.111111	26.11111111	0	0	0	1	30.519848	1	0.21141735
0.0144	657.070401	0.97015572	260.146257	1.72698046	0	-4.2405271	-0.4184434	-0.0292685	5.56124784	0.54695336	-6.9077553	0	92	1.34111667	298	25	0	0	0	0	34.0724638	1	0.49277013
0.0243	1007.98075	0.50962275	60.6969993	0.70242778	0	-3.7172789	0.00894067	-0.6721242	4.10591074	-0.3517901	-6.9077553	1	95	1.34111667	297.444444	24.44444444	0	0	0	0	34.0724638	1	0.49277013
0.0405	1622.07364	0.49570963	61.7962307	0.77268738	0	-3.2064533	0.48432166	-0.6997497	4.12385855	-0.2565874	-6.9077553	2	96	2.23519444	297.444444	24.44444444	0	0	0	0	34.0724638	1	0.49277013
0.0558	2280.03036	0.54028887	71.1228602	0.84228443	0	-2.8859814	0.82462725	-0.6138022	4.26442287	-0.170451	-6.9077553	3	98	1.78815556	297.444444	24.44444444	0	0	0	0	34.0724638	1	0.49277013
0.0936	3859.12611	0.61200283	93.7037819	1.01172593	0	-2.3687249	1.35069985	-0.4893857	4.54014922	0.01264563	-6.9077553	4	93	3.12927222	298.555556	25.55555555	0	0	0	0	34.0724638	1	0.49277013
0.1512	6424.52638	0.73739326	143.875153	1.21453917	0	-1.8891518	1.86027855	-0.3032787	4.96895288	0.19518774	-6.9077553	5	80	4.02335	300.777778	27.7777778	0	0	0	0	34.0724638	1	0.49277013
0.2439	9756.18795	0.82682437	184.062064	1.90007301	0	-1.410997	2.27800424	-0.1889543	5.21527844	0.64241847	-6.9077553	6	72	4.47038889	302.444444	29.44444444	0	0	0	0	34.0724638	1	0.49277013
0.4374	18412.5642	1.0511124	311.017297	2.59438363	0	-0.8269072	2.91308758	0.05079995	5.73985174	0.95373434	-6.9077553	7	62	3.57631111	304.111111	31.11111111	0	0	0	1	34.0724638	1	0.49277013
0.7236	32525.9278	1.16296022	388.262308	2.90582141	0	-0.3235165	3.48206829	0.15182817	5.96168374	1.06706019	-6.9077553	8	55	3.12927222	305.777778	32.7777778	0	0	0	1	34.0724638	1	0.49277013
1.1655	49704.2063	1.16606062	389.420443	3.15784363	0	0.15315018	3.90610968	0.1544883	5.96466216	1.15020602	-6.9077553	9	50	3.57631111	306.888889	33.88888888	0	0	0	1	34.0724638	1	0.49277013
1.5597	60734.4223	0.98759795	275.085438	2.07947	0	0.4444935	4.10652709	-0.0114676	5.61708537	0.73259383	-6.9077553	10	46	2.68223333	307.444444	34.4444444	0	0	0	1	34.0724638	1	0.49277013
2.0403	72164.6777	1.02838112	298.935598	2.22640157	0	0.71309686	4.27896456	0.02895777	5.7002315	0.80083569	-6.9077553	11	41	2.68223333	309.111111	36.11111111	0	0	0	1	34.0724638	1	0.49277013
2.6865	87759.4426	1.0552505	319.452603	2.49046083	0	0.98823923	4.47461086	0.05472538	5.76661204	0.91286922	-6.9077553	12	38	3.12927222	309.666667	36.66666666	0	0	0	1	34.0724638	1	0.49277013
3.4569	113615.04	1.0675503	326.410193	2.50420914	0	1.24037223	4.73282469	0.06630287	5.78815791	0.91837222	-6.9077553	13	36	3.12927222	309.111111	36.11111111	0	0	0	1	34.0724638	1	0.49277013
4.194	140486.198	0.96780466	263.522321	2.11450016	0	1.43365493	4.94511636	-0.0316923	5.57414187	0.74929127	-6.9077553	14	38	3.12927222	308.555556	35.5555556	0	0	0	1	34.0724638	1	0.49277013
4.7232	158289.998	0.86449303	205.357422	1.49989516	0	1.55248654	5.0644351	-0.144456	5.32475685	0.4060617	-6.9077553	15	42	1.34111667	308	35	0	0	0	1	34.0724638	1	0.49277013
5.2083	176126.591	0.83307168	197.204154	1.63942785	0	1.65025351	5.17120868	-0.1814359	5.28424458	0.49495709	-6.9077553	16	54	1.34111667	304.666667	31.66666666	0	0	0	1	34.0724638	1	0.49277013
5.5296	189086.569	0.7689792	163.264279	1.24161331	0	1.71011548	5.24221024	-0.2613918	5.09537636	0.21721667	-6.9077553	17	61	0.89407778	303	30	0	0	0	1	34.0724638	1	0.49277013
5.7852	200200.86	0.82561439	180.001018	1.3536594	0	1.75530293	5.29932616	-0.190417	5.19296806	0.30355006	-6.9077553	18	66	1.34111667	301.888889	28.88888888	0	0	0	1	34.0724638	1	0.49277013
6.0075	209520.91	0.78298993	162.837835	1.31443607	0	1.79300869	5.34482832	-0.2433591	5.09276097	0.27416823	-6.9077553	19	66	1.34111667	301.333333	28.33333333	0	0	0	1	34.0724638	1	0.49277013
6.2037	218094.159	0.79611297	168.613711	1.33311112	0	1.82514589	5.38493148	-0.2267589	5.1276163	0.28826525	-6.9077553	20	69	1.34111667	300.777778	27.7777778	0	0	0	1	34.0724638	1	0.49277013
6.3513	224423.649	0.76231245	153.103682	1.27730021	0	1.84865952	5.41354001	-0.2700878	5.03112189	0.24553123	-6.9077553	21	72	1.78815556	299.666667	26.66666666	0	0	0	1	34.0724638	1	0.49277013
6.4908	229957.9	0.69779929	127.88028	1.22530222	0	1.87038579	5.4379006	-0.3583917	4.85110233	0.20400332	-6.9077553	22	78	2.23519444	299.666667	26.66666666	0	0	0	1	34.0724638	1	0.49277013
6.5448	231844.019	0.27041044	18.0227214	0.53216461	0	1.87867084	5.44606913	-1.3041231	2.89168874	-0.6289251	-6.9077553	23	83	2.23519444	299.111111	26.11111111	0	0	0	1	34.0724638	1	0.49277013
0.0243	1320.90814	0.98093693	261.903546	1.78676811	0	-3.7172789	0.27907625	-0.0182282	5.56798011	0.58096797	-6.9077553	0	92	1.34111667	298	25	0	0	0	0	42.288408	1	-0.4111751
0.0666	3386.0195	0.70264232	125.989694	1.25818748	0	-2.7090507	1.21995033	-0.3514851	4.83620805	0.23046665	-6.9077553	1	95	1.34111667	297.444444	24.44444444	0	0	0	0	42.288408	1	-0.4111751
0.1188	5842.39139	0.62924628	100.161882	1.15661897	0	-2.1303139	1.76531135	-0.4616446	4.60679768	0.14636529	-6.9077553	2	96	2.23519444	297.444444	24.44444444	0	0	0	0	42.288408	1	-0.4111751
0.1917	9307.62974	0.6700203	113.757186	1.25105116	0	-1.6518236	2.2309419	-0.3989559	4.73407502	0.22478314	-6.9077553	3	98	1.78815556	297.444444	24.44444444	0	0	0	0	42.288408	1	-0.4111751
0.2763	13354.9536	0.72598745	138.046054	1.35672135	0	-1.286268	2.59196225	-0.3188461	4.9275946	0.30580782	-6.9077553	4	93	3.12927222	298.555556	25.55555555	0	0	0	0	42.288408	1	-0.4111751
0.5031	21391.9792	0.81987672	193.257084	2.3603518	0	-0.6869663	3.06306279	-0.1973823	5.26402652	0.85923425	-6.9077553	5	80	4.02335	300.777778	27.7777778	0	0	0	0	42.288408	1	-0.4111751
0.7821	33000.0959	0.89806164	220.084652	2.02648237	0	-0.2457727	3.49654077	-0.1064037	5.3940168	0.70679481	-6.9077553	6	72	4.47038889	302.444444	29.44444444	0	0	1	0	42.288408	1	-0.4111751
1.1115	45226.0945	0.86717841	199.654201	1.76834757	0	0.10571045	3.81169634	-0.141358	5.29659188	0.57061087	-6.9077553	7	62	3.57631111	304.111111	31.11111111	0	0	1	0	42.288408	1	-0.4111751
1.4805	60091.6781	0.86331058	198.662233	1.56009015	0	0.39237987	4.09588801	-0.1458231	5.2916111	0.44538439	-6.9077553	8	55	3.12927222	305.777778	32.7777778	0	0	1	0	42.288408	1	-0.4111751
1.935	78364.6361	0.89710705	218.426903	1.83690379	0	0.66010733	4.36138552	-0.107466	5.386456	0.60862568	-6.9077553	9	50	3.57631111	306.888889	33.88888888	0	0	1	0	42.288408	1	-0.4111751
2.5731	100815.688	0.92391731	242.874008	2.39312391	0	0.9451114	4.6133039	-0.0780509	5.49254694	0.87301737	-6.9077553	10	46	2.68223333	307.444444	34.4444444	0	0	1	0	42.288408	1	-0.4111751
3.2499	128496.256	0.93585416	250.498462	2.10603365	0	1.17862423	4.85590755	-0.0652277	5.52345677	0.74528111	-6.9077553	11	41	2.68223333	309.111111	36.11111111	0	0	1	0	42.288408	1	-0.4111751
3.969	163892.562	1.01826135	299.473658	2.23091853	0	1.37851417	5.09921721	0.0190782	5.7020298	0.80286154	-6.9077553	12	38	3.12927222	309.666667	36.66666666	0	0	1	0	42.288408	1	-0.4111751
4.7403	199607.747	1.02502869	306.82807	2.34172537	0	1.55610042	5.29635919	0.02569571	5.72629082	0.85131494	-6.9077553	13	36	3.12927222	309.111111	36.11111111	0	0	1	0	42.288408	1	-0.4111751

5.4693	230910.178	0.96253376	274.319232	2.19039292	0	1.69915064	5.44203313	-0.0371478	5.61429615	0.78453738	-6.9077553	14	38	3.12927222	308.555556	35.5555556	0	0	1	0	42.288408	1	-0.4111751
6.0237	247160.625	0.77372603	186.869978	1.68817357	0	1.79570169	5.51004248	-0.2552458	5.23041842	0.5242394	-6.9077553	15	42	1.34111667	308	35	0	0	1	0	42.288408	1	-0.4111751
6.4179	256041.594	0.598628	113.509525	1.2096297	0	1.85909096	5.54534381	-0.5114458	4.73189556	0.19114064	-6.9077553	16	54	1.34111667	304.666667	31.66666666	0	0	1	0	42.288408	1	-0.4111751
6.6411	264172.711	0.73290979	164.621661	1.20378679	0	1.89327761	5.57660688	-0.3093692	5.10365595	0.18630261	-6.9077553	17	61	0.89407778	303	30	0	0	1	0	42.288408	1	-0.4111751
6.7995	271347.029	0.84208306	191.563654	1.40221162	0	1.91684908	5.60340224	-0.1706898	5.25522537	0.33876363	-6.9077553	18	66	1.34111667	301.888889	28.88888888	0	0	1	0	42.288408	1	-0.4111751
6.9327	277167.281	0.7475356	148.47556	1.17000683	0	1.93624935	5.62462484	-0.2896365	5.0004271	0.15786392	-6.9077553	19	66	1.34111667	301.333333	28.33333333	0	0	1	0	42.288408	1	-0.4111751
7.056	282475.803	0.78219682	157.839958	1.21200584	0	1.95387832	5.64359643	-0.2443712	5.06158793	0.19310144	-6.9077553	20	69	1.34111667	300.777778	27.7777778	0	0	1	0	42.288408	1	-0.4111751
7.1397	285933.504	0.69809903	127.892221	1.05834124	0	1.96567076	5.65576278	-0.3579629	4.85119571	0.05764724	-6.9077553	21	72	1.78815556	299.666667	26.66666666	0	0	1	0	42.288408	1	-0.4111751
7.2045	288214.43	0.61978886	97.5813056	0.93718592	0	1.97470583	5.66370822	-0.4767643	4.58069618	-0.0638071	-6.9077553	22	78	2.23519444	299.666667	26.66666666	0	0	1	0	42.288408	1	-0.4111751
7.2243	288960.089	0.3139286	27.9383351	0.54389355	0	1.97745034	5.66629204	-1.1554093	3.33003556	-0.6071648	-6.9077553	23	83	2.23519444	299.111111	26.11111111	0	0	1	0	42.288408	1	-0.4111751
0.0009	0	0	0	0	0	-7.0131158	-6.9077553	-6.9077553	-6.9077553	-6.9077553	-6.9077553	0	92	1.34111667	298	25	0	0	0	0	18.9135952	1	-0.0428739
0.0009	0	0	0	0	0	-7.0131158	-6.9077553	-6.9077553	-6.9077553	-6.9077553	-6.9077553	1	95	1.34111667	297.444444	24.44444444	0	0	0	0	18.9135952	1	-0.0428739
0.0009	0	0	0	0	0	-7.0131158	-6.9077553	-6.9077553	-6.9077553	-6.9077553	-6.9077553	2	96	2.23519444	297.444444	24.44444444	0	0	0	0	18.9135952	1	-0.0428739
0.0009	0	0	0	0	0	-7.0131158	-6.9077553	-6.9077553	-6.9077553	-6.9077553	-6.9077553	3	98	1.78815556	297.444444	24.44444444	0	0	0	0	18.9135952	1	-0.0428739
0.0009	0	0	0	0	0	-7.0131158	-6.9077553	-6.9077553	-6.9077553	-6.9077553	-6.9077553	4	93	3.12927222	298.555556	25.5555555	0	0	0	0	18.9135952	1	-0.0428739
0.0009	0	0	0	0	0	-7.0131158	-6.9077553	-6.9077553	-6.9077553	-6.9077553	-6.9077553	5	80	4.02335	300.777778	27.7777778	0	0	0	0	18.9135952	1	-0.0428739
0.0333	0	0.55434338	86.9034153	2.53521983	0	-3.4021979	-6.9077553	-0.5881686	4.46480884	0.93067472	-6.9077553	6	72	4.47038889	302.444444	29.44444444	0	0	0	0	18.9135952	1	-0.0428739
0.144	272.394292	0.57869658	90.1866187	2.26017699	0	-1.937942	-1.2968402	-0.5452504	4.50189215	0.81588547	-6.9077553	7	62	3.57631111	304.111111	31.11111111	0	0	0	0	18.9135952	1	-0.0428739
0.342	3306.23474	0.69003553	155.900355	2.31455272	0	-1.0729445	1.19611241	-0.369564	5.04922347	0.83964842	-6.9077553	8	55	3.12927222	305.777778	32.7777778	0	0	1	0	18.9135952	1	-0.0428739
0.5346	10897.8266	0.88717687	272.088475	2.25834584	0	-0.6262365	2.38865513	-0.1185844	5.60613096	0.81507532	-6.9077553	9	50	3.57631111	306.888889	33.88888888	0	0	1	0	18.9135952	1	-0.0428739
0.6795	15572.7677	0.75216926	183.018452	1.4359309	0	-0.386398	2.74558794	-0.2834653	5.20959244	0.36250952	-6.9077553	10	46	2.68223333	307.444444	34.4444444	0	0	1	0	18.9135952	1	-0.0428739
0.8442	19722.6817	0.70611922	152.989156	1.35847148	0	-0.1693658	2.98182003	-0.346556	5.03037358	0.30709601	-6.9077553	11	41	2.68223333	309.111111	36.11111111	0	0	1	0	18.9135952	1	-0.0428739
1.1088	29482.08	0.91843544	255.857302	2.31109827	0	0.10327835	3.38381654	-0.0839954	5.54462378	0.83815545	-6.9077553	12	38	3.12927222	309.666667	36.66666666	0	0	1	0	18.9135952	1	-0.0428739
1.44	40409.2029	0.89325548	240.993589	2.30238409	0	0.36464311	3.6990823	-0.1117638	5.48477448	0.83437938	-6.9077553	13	36	3.12927222	309.111111	36.11111111	0	0	1	0	18.9135952	1	-0.0428739
1.7451	51730.3391	0.86188266	222.267423	1.80566694	0	0.55681186	3.94606377	-0.1474766	5.40388576	0.59148368	-6.9077553	14	38	3.12927222	308.555556	35.5555556	0	0	1	0	18.9135952	1	-0.0428739
2.0601	62998.0844	0.7855031	188.389241	1.54186018	0	0.72275453	4.14312019	-0.2401586	5.23851566	0.43363795	-6.9077553	15	42	1.34111667	308	35	0	0	1	0	18.9135952	1	-0.0428739
2.4417	77426.8811	0.84561078	213.491524	1.72185329	0	0.89269452	4.34934694	-0.1665142	5.36360182	0.54398181	-6.9077553	16	54	1.34111667	304.666667	31.66666666	0	0	1	0	18.9135952	1	-0.0428739
2.7342	90965.6984	0.8914271	224.642716	1.59186805	0	1.00583889	4.51049349	-0.1138105	5.41451566	0.4655362	-6.9077553	17	61	0.89407778	303	30	0	0	1	0	18.9135952	1	-0.0428739
2.9421	100847.042	0.86396895	202.289654	1.50262042	0	1.07912361	4.61361485	-0.1450617	5.30970555	0.40787581	-6.9077553	18	66	1.34111667	301.888889	28.88888888	0	0	1	0	18.9135952	1	-0.0428739
3.105	108554.388	0.81171953	178.01092	1.41413719	0	1.13301372	4.68726053	-0.2073692	5.18185052	0.34722648	-6.9077553	19	66	1.34111667	301.333333	28.33333333	0	0	1	0	18.9135952	1	-0.0428739
3.2859	116820.743	0.8656308	205.508454	1.64186954	0	1.18964059	4.76064921	-0.1431422	5.32549204	0.49644443	-6.9077553	20	69	1.34111667	300.777778	27.7777778	0	0	1	0	18.9135952	1	-0.0428739
3.4812	125855.852	0.87159933	204.463314	1.6955828	0	1.24737706	4.83514516	-0.1362788	5.32039345	0.52861611	-6.9077553	21	72	1.78815556	299.666667	26.66666666	0	0	1	0	18.9135952	1	-0.0428739
3.6	131155.039	0.74370506	146.575373	1.3593609	0	1.28093385	4.87638775	-0.294767	4.98754661	0.30775003	-6.9077553	22	78	2.23519444	299.666667	26.66666666	0	0	1	0	18.9135952	1	-0.0428739
3.6432	132953.453	0.38838657	39.2355855	0.66537762	0	1.29286242	4.89000661	-0.9431827	3.66960962	-0.4058988	-6.9077553	23	83	2.23519444	299.111111	26.11111111	0	0	1	0	18.9135952	1	-0.0428739
0.0189	445.273215	0.83620605	194.383215	2.1457016	0	-3.9685934	-0.8068239	-0.1776851	5.26983669	0.76393252	-6.9077553	0	87	2.23519444	299.111111	26.11111111	0	0	0	0	30.519848	1	0.21141735
0.0459	1283.72998	0.63632806	119.261605	1.1907252	0	-3.0812902	0.25054856	-0.4504707	4.78132782	0.175402	-6.9077553	1	79	2.68223333	300.777778	27.7777778	0	0	0	0	30.519848	1	0.21141735
0.0999	2341.01676	0.64096921	122.609106	1.29843915	0	-2.3035856	0.85101242	-0.4432149	4.80900945	0.26193275	-6.9077553	2	71	2.68223333	302.444444	29.44444444	0	0	0	0	30.519848	1	0.21141735
0.2124	3242.21964	0.65832538	132.055594	2.58636255	0	-1.549284	1.17656655	-0.4165381	4.88323057	0.95063904	-6.9077553	3	65	4.02335	303.555556	30.5555555	0	0	0	1	30.519848	1	0.21141735
0.4176	5903.38357	0.76074098	171.325966	2.98740802	0	-0.8732312	1.77569505	-0.2721487	5.14357381	1.09474081	-6.9077553	4	61	3.12927222	304.666667	31.66666666	0	0	0	1	30.519848	1	0.21141735
0.6993	11775.9553	0.87035636	240.943865	2.72385141	0	-0.3576754	2.46614468	-0.1377043	5.48456813	1.0024139	-6.9077553	5	54	3.57631111	305.777778	32.7777778	0	0	0	1	30.519848	1	0.21141735
1.0107	19206.8477	0.79796305	185.020547	2.35988745	0	0.01064316	2.95531893	-0.2244406	5.22047229	0.85903759	-6.9077553	6	49	4.02335	307.444444	34.4444444	0	0	0	1	30.519848	1	0.21141735
1.2456	27343.9074	0.83154542	208.709201	1.81225446	0	0.21961734	3.30853031	-0.1832675	5.34094669	0.59512327	-6.9077553	7	51	4.02335	306.333333	33.33333333	0	0	0	1	30.519848	1	0.21141735
1.3833	30559.852	0.65775095	120.466682	1.25587114	0	0.32447195	3.41971984	-0.4174097	4.79138152	0.22862541	-6.9077553	8	50	2.68223333	306.333333	33.33333333	0	0	0	1	30.519848	1	0.21141735
1.5048	33173.4817	0.64156822	114.71679	1.23338883	0	0.40866	3.50178096	-0.4422823	4.74247511	0.21057597	-6.9077553	9	52	3.12927222	306.333333	33.33333333	0	0	0	1	30.519848	1	0.21141735
1.5642	35556.5121	0.72530983	139.246286	1.06284506	0	0.44737451	3.57115145	-0.3197786	4.93625139	0.06188976	-6.9077553	10	81	2.23519444	300.222222	27.2222222	0	0	0	1	30.519848	1	0.21141735
1.5876	36609.2416	0.48633245	59.3566049	0.76064983	0	0.46222344	3.60032803	-0.7188087	4.08358025	-0.2722684	-6.9077553	11	89	1.34111667	298	25	0	0	0	1	30.519848	1	0.21141735
1.6038	37311.0629	0.29864981	20.1432699	0.3907973	0	0.47237581	3.61931668	-1.2051408	3.00291987	-0.9370107	-6.9077553	12	94	2.23519444	296.888889	23.88888888	0	0	0	1	30.519848	1	0.21141735
1.6182	37969.02	0.24640667	12.8134403	0.33590591	0	0.48131442	3.6367969	-1.3967218	2.55057269	-1.0879516	-6.9077553	13	94	1.34111667	297.444444	24.44444444	0	0	0	1	30.519848	1	0.21141735
1.6299	38539.2462	0.25630716	13.6170279	0.3288897	0	0.48851866	3.65170305	-1.3574847	2.61139449	-1.1089969	-6.9077553	14	95	2.23519444	296.888889	23.88888888	0	0	0	1	30.519848	1	0.21141735
1.6443	39153.3419	0.23990434	12.0601597	0.32716405	0	0.49731476	3.66751132	-1.4233553	2.48999035	-1.1142416	-6.9077553	15	93	2.23519444	296.888889	23.88888888	0	0	0	1	30.519848	1	0.21141735
1.6551	39548.1153	0.25135346	13.0382716	0.326888	0	0.50386143	3.67754332	-1.3769245	2.56796569	-1.1150832	-6.9077553	16	95	1.78815556	296.333333	23.33333333	0	0	0	1	30.519848	1	0.21141735
1.6713	40206.0713	0.25507447	14.0383674	0.36819937	0	0.51360177	3.69404288	-1.362287	2.64186534	-0.9964185	-6.9077553	17	94	2.68223333	296.888889	23.88888888	0	0	0	1	30.519848	1	0.21141735
1.6839	40776.3001	0.24201718	12.2739955	0.38509451	0	0.52111253	3.70812556	-1.4146231	2.50756431	-0.9516731	-6.9077553	18	94	2.23519444	296.888889	23.88888888	0	0	0	1	30.519848	1	0.21141735
1.6992	41434.2573	0.2967559	18.8079295	0.48124498	0	0.53015755	3.72413214	-1.2114812	2.93433173	-0.729303	-6.9077553	19	89	3.57631111	298	25	0	0	0	1	30.519848	1	0.21141735
1.7154	42136.0754	0.33361578	24.991094	0.64215867	0	0.53964629	3.740928	-1.0947723	3.21855953	-0.4413638	-6.9077553	20	91	4.47038889	298	25	0	0	0	1	30.519848	1	0.21141735
1.7334	42969.4905	0.3401881	25.8545471	0.66170402	0	0.5500848	3.76051361	-1.0753213	3.25252517	-0.4114268	-6.9077553	21	93	4.47038889	296.888889	23.88888888	0	0	0	1	30.519848	1	0.21141735
1.7505	43759.0369	0.34634103	26.3409777	0.66904259	0	0.55990146	3.778721	-1.0574482	3.27116378	-0.400414	-6.9077553	22	94	4.91742778	296.333333	23.33333333	0	0	0	1	30.519848	1	0.21141735
1.7829	44090.3025	0.33354534	35.8100857	1.00444243	0	0.57824125	3.78626254	-1.0949829	3.5782575	0.00542767	-6.9077553	23	94	4.47038889	295.777778	22.7777778	0	0	0	1	30.519848	1	0.21141735
0.018	819.704435	0.9089228	229.032972	1.57173046	0	-4.0173835	-0.1975922	-0.0943955	5.43387034	0.45281326	-6.9077553	0	87	2.23519444	299.111111	26.11111111	0	0	0	0	34.0724638	1	0.49277013
0.0315	1214.47852	0.52913895	78.1497314	0.71394997	0	-3.4577677	0.19513784	-0.6346161	4.35863942	-0.3355427	-6.9077553	1	79	2.68223333	300.777778	27.7777778	0	0	0	0	34.0724638	1	0.49277013
0.0684	2411.5227	0.66240004	120.118605	1.07765756	0	-2.6823825	0.88067296	-0.4103771	4.78848795	0.07571727	-6.9077553	2	71	2.68223333	302.444444	29.44444444	0	0	0	0	34.0724638	1	0.49277013
0.1188	4514.61559	0.8646916	194.756313	1.2869151	0	-2.1303139	1.50754152	-0.1442266	5.27175423	0.25302471	-6.9077553	3	65	4.02335	303.555556	30.5555555	0	0	0	0	34.0724638	1	0.49277013

0.1773	7280.25739	0.8776626	200.532422	1.294589	0	-1.7299121	1.98530357	-0.1293543	5.30098093	0.25896542	-6.9077553	4	61	3.12927222	304.666667	31.66666666	0	0	0	0	34.0724638	1	0.49277013
0.2574	10842.196	0.88897928	213.574197	1.48487168	0	-1.357124	2.38353778	-0.1165571	5.36398898	0.39600159	-6.9077553	5	54	3.57631111	305.777778	32.7777778	0	0	0	0	34.0724638	1	0.49277013
0.4914	17108.6161	1.14676795	394.984075	3.25624624	0	-0.7104968	2.83964065	0.13781915	5.97884798	1.18088213	-6.9077553	6	49	4.02335	307.444444	34.4444444	0	0	0	1	34.0724638	1	0.49277013
0.9459	24155.2026	1.29291436	522.039728	3.7569452	0	-0.0556184	3.18454118	0.25767201	6.25774561	1.32387232	-6.9077553	7	51	4.02335	306.333333	33.33333333	0	0	0	1	34.0724638	1	0.49277013
1.3257	31240.3512	0.98593212	271.027918	2.18567444	0	0.28194062	3.44174258	-0.013154	5.60222552	0.78238187	-6.9077553	8	50	2.68223333	306.333333	33.33333333	0	0	0	1	34.0724638	1	0.49277013
1.7307	40768.2477	0.8937835	218.776181	1.81537516	0	0.54852595	3.70792806	-0.1111735	5.38805377	0.59684285	-6.9077553	9	52	3.12927222	306.333333	33.33333333	0	0	0	1	34.0724638	1	0.49277013
1.9431	48210.7812	0.80116944	175.3053	1.3690814	0	0.66428464	3.87560341	-0.2204354	5.16653473	0.31487015	-6.9077553	10	81	2.23519444	300.222222	27.2222222	0	0	0	1	34.0724638	1	0.49277013
2.0493	52115.3445	0.47809193	61.8206311	0.86814389	0	0.71749827	3.95347861	-0.7358628	4.12425332	-0.1402466	-6.9077553	11	89	1.34111667	298	25	0	0	0	1	34.0724638	1	0.49277013
2.1123	54310.7572	0.35767961	38.1023054	0.70810073	0	0.7477774	3.99474073	-1.0253257	3.64030103	-0.3437577	-6.9077553	12	94	2.23519444	296.888889	23.88888888	0	0	0	1	34.0724638	1	0.49277013
2.1753	56942.5846	0.27929679	18.1320923	0.49032582	0	0.77716659	4.04206103	-1.2719063	2.89773857	-0.7106478	-6.9077553	13	94	1.34111667	297.444444	24.44444444	0	0	0	1	34.0724638	1	0.49277013
2.2329	58740.9968	0.33922601	36.1227417	0.64513922	0	0.80330119	4.07315492	-1.0781452	3.58695031	-0.4367403	-6.9077553	14	95	2.23519444	296.888889	23.88888888	0	0	0	1	34.0724638	1	0.49277013
2.2896	60846.4621	0.31456784	31.4468781	0.52943439	0	0.82837713	4.10837011	-1.1533816	3.44833151	-0.634059	-6.9077553	15	93	2.23519444	296.888889	23.88888888	0	0	0	1	34.0724638	1	0.49277013
2.3409	61986.9208	0.40469789	58.995357	0.73484381	0	0.85053547	4.12693954	-0.9021465	4.0774757	-0.3067374	-6.9077553	16	95	1.78815556	296.333333	23.33333333	0	0	0	1	34.0724638	1	0.49277013
2.3706	62995.7852	0.39948463	52.052923	0.68272267	0	0.86314309	4.1430837	-0.9150799	3.95228016	-0.3802029	-6.9077553	17	94	2.68223333	296.888889	23.88888888	0	0	0	1	34.0724638	1	0.49277013
2.412	64092.3798	0.37873563	46.6030167	0.65377716	0	0.88045628	4.16034108	-0.96828	3.84168673	-0.4234603	-6.9077553	18	94	2.23519444	296.888889	23.88888888	0	0	0	1	34.0724638	1	0.49277013
2.4435	65320.5658	0.27571057	22.9885707	0.48828932	0	0.89343144	4.17932224	-1.2847832	3.13504067	-0.7148013	-6.9077553	19	89	3.57631111	298	25	0	0	0	1	34.0724638	1	0.49277013
2.4786	66680.3379	0.3270068	36.5119879	0.63364234	0	0.90769388	4.19992512	-1.1147209	3.59766803	-0.4546937	-6.9077553	20	91	4.47038889	298	25	0	0	0	1	34.0724638	1	0.49277013
2.5137	68215.5769	0.29484898	23.2624778	0.45316672	0	0.92175577	4.2226876	-1.2179062	3.14688465	-0.7892909	-6.9077553	21	93	4.47038889	296.888889	23.88888888	0	0	0	1	34.0724638	1	0.49277013
2.5659	70364.901	0.30071749	24.2748413	0.50824448	0	0.94230929	4.25370878	-1.1982642	3.18948167	-0.6748271	-6.9077553	22	94	4.91742778	296.333333	23.33333333	0	0	0	1	34.0724638	1	0.49277013
2.6289	73092.4033	0.37171917	43.08637	0.65515393	0	0.96656551	4.29173812	-0.98693	3.76322991	-0.4213599	-6.9077553	23	94	4.47038889	295.777778	22.7777778	0	0	0	1	34.0724638	1	0.49277013
0.0252	1455.5682	1.05431359	301.545103	1.98841702	0	-3.6809113	0.37608312	0.05383796	5.70892292	0.68784164	-6.9077553	0	87	2.23519444	299.111111	26.11111111	0	0	0	0	42.288408	1	-0.4111751
0.1044	5731.42912	0.98031495	259.925115	1.88740193	0	-2.2595256	1.74613937	-0.0188618	5.56039742	0.63573093	-6.9077553	1	79	2.68223333	300.777778	27.7777778	0	0	0	0	42.288408	1	-0.4111751
0.2817	15081.0589	1.03771438	305.332091	2.42094909	0	-1.2669126	2.71350588	0.03798378	5.72140328	0.88457263	-6.9077553	2	71	2.68223333	302.444444	29.44444444	0	0	0	0	42.288408	1	-0.4111751
0.5778	30526.6927	1.10199494	351.436479	2.94865311	0	-0.5485275	3.41863423	0.09802915	5.86203183	1.08168757	-6.9077553	3	65	4.02335	303.555556	30.55555555	0	0	1	0	42.288408	1	-0.4111751
0.8892	47204.8726	1.01316613	290.545385	2.04412171	0	-0.1174331	3.8545183	0.01406673	5.67176324	0.71545731	-6.9077553	4	61	3.12927222	304.666667	31.66666666	0	0	1	0	42.288408	1	-0.4111751
1.3023	60986.8178	0.90691215	221.375732	2.25073615	0	0.26413193	4.11067414	-0.0966077	5.39986592	0.81170154	-6.9077553	5	54	3.57631111	305.777778	32.7777778	0	0	1	0	42.288408	1	-0.4111751
1.7262	75935.6895	0.83269059	182.250299	1.88873265	0	0.54592246	4.32989996	-0.1818929	5.2053865	0.63643536	-6.9077553	6	49	4.02335	307.444444	34.4444444	0	0	1	0	42.288408	1	-0.4111751
2.1537	93240.1547	0.86572754	202.270855	1.66823872	0	0.76718729	4.5351892	-0.1430306	5.30961261	0.51236767	-6.9077553	7	51	4.02335	306.333333	33.33333333	0	0	1	0	42.288408	1	-0.4111751
2.5812	110130.773	0.80214028	174.137336	1.33189273	0	0.94825441	4.70167759	-0.2192259	5.15985002	0.28735157	-6.9077553	8	50	2.68223333	306.333333	33.33333333	0	0	1	0	42.288408	1	-0.4111751
3.0591	128917.669	0.81132016	186.670242	1.55862835	0	1.11812076	4.85918174	-0.2078607	5.229349	0.44444755	-6.9077553	9	52	3.12927222	306.333333	33.33333333	0	0	1	0	42.288408	1	-0.4111751
3.3768	142993.971	0.83526352	193.648438	1.43089774	0	1.21692852	4.96280946	-0.1788115	5.2660495	0.35900066	-6.9077553	10	81	2.23519444	300.222222	27.2222222	0	0	1	0	42.288408	1	-0.4111751
3.4902	147950.586	0.44747661	50.02572	0.69937975	0	1.24995904	4.9968851	-0.8018987	3.91255726	-0.3561326	-6.9077553	11	89	1.34111667	298	25	0	0	1	0	42.288408	1	-0.4111751
3.5784	151898.324	0.29623411	20.0456643	0.48931632	0	1.27491577	5.02321796	-1.2132352	2.99806277	-0.7127046	-6.9077553	12	94	2.23519444	296.888889	23.88888888	0	0	1	0	42.288408	1	-0.4111751
3.6702	155802.204	0.28366583	17.8433622	0.47394285	0	1.30024616	5.0485937	-1.2564393	2.88168762	-0.7445608	-6.9077553	13	94	1.34111667	297.444444	24.44444444	0	0	1	0	42.288408	1	-0.4111751
3.7575	159442.894	0.27860592	17.394759	0.48287323	0	1.32375384	5.0716921	-1.2743741	2.85622644	-0.7259323	-6.9077553	14	95	2.23519444	296.888889	23.88888888	0	0	1	0	42.288408	1	-0.4111751
3.8466	162820.399	0.27043859	16.150652	0.44873282	0	1.34718964	5.09265389	-1.3040194	2.78202234	-0.7991016	-6.9077553	15	93	2.23519444	296.888889	23.88888888	0	0	1	0	42.288408	1	-0.4111751
3.9195	166110.186	0.2673176	15.5237344	0.42181929	0	1.36596409	5.11265736	-1.3155839	2.74243452	-0.8608104	-6.9077553	16	95	1.78815556	296.333333	23.33333333	0	0	1	0	42.288408	1	-0.4111751
3.9816	168829.732	0.25681233	14.5228192	0.40846675	0	1.38168375	5.12889663	-1.3555234	2.67579	-0.8928996	-6.9077553	17	94	2.68223333	296.888889	23.88888888	0	0	1	0	42.288408	1	-0.4111751
4.0527	171768.606	0.2432207	12.8490647	0.37001846	0	1.39938333	5.14615408	-1.4096829	2.55334885	-0.9915034	-6.9077553	18	94	2.23519444	296.888889	23.88888888	0	0	1	0	42.288408	1	-0.4111751
4.1067	174005.659	0.24232512	12.9023224	0.3656209	0	1.41261979	5.15909357	-1.4133568	2.55748483	-1.0034269	-6.9077553	19	89	3.57631111	298	25	0	0	1	0	42.288408	1	-0.4111751
4.1787	177251.58	0.24404941	12.6767464	0.32669077	0	1.43000019	5.17757572	-1.4062954	2.5398482	-1.1156849	-6.9077553	20	91	4.47038889	298	25	0	0	1	0	42.288408	1	-0.4111751
4.2345	179532.496	0.25325398	13.5670678	0.35508039	0	1.44326526	5.1903618	-1.3694216	2.60771908	-1.0325988	-6.9077553	21	93	4.47038889	296.888889	23.88888888	0	0	1	0	42.288408	1	-0.4111751
4.2993	182208.19	0.23413489	12.0351308	0.34504684	0	1.45845222	5.20515542	-1.4475959	2.48791303	-1.0611811	-6.9077553	22	94	4.91742778	296.333333	23.33333333	0	0	1	0	42.288408	1	-0.4111751
4.3974	185629.558	0.29181571	23.3618634	0.67671855	0	1.48101346	5.22375845	-1.2282118	3.15114773	-0.3890232	-6.9077553	23	94	4.47038889	295.777778	22.7777778	0	0	1	0	42.288408	1	-0.4111751
0.0072	0	0.50907769	95.335859	2.53406143	0	-4.9336743	-6.9077553	-0.6731922	4.5574165	0.93021787	-6.9077553	0	87	2.23519444	299.111111	26.11111111	0	0	0	0	18.9135952	1	-0.0428739
0.0477	43.8637829	0.47246051	57.8488444	1.62756432	0	-3.0428239	-3.1041244	-0.7476868	4.05785076	0.48769884	-6.9077553	1	79	2.68223333	300.777778	27.7777778	0	0	1	0	18.9135952	1	-0.0428739
0.0963	438.699581	0.52608702	76.4262062	1.16431081	0	-2.340287	-0.8216636	-0.6403896	4.33633874	0.15298784	-6.9077553	2	71	2.68223333	302.444444	29.44444444	0	0	1	0	18.9135952	1	-0.0428739
0.234	1930.06816	0.65042563	114.930326	2.10323638	0	-1.4524342	0.6580733	-0.428592	4.74433478	0.74395264	-6.9077553	3	65	4.02335	303.555556	30.55555555	0	0	1	0	18.9135952	1	-0.0428739
0.4032	5736.75913	0.67532819	137.548548	1.72630245	0	-0.9083226	1.74706874	-0.3910768	4.9239842	0.54656091	-6.9077553	4	61	3.12927222	304.666667	31.66666666	0	0	1	0	18.9135952	1	-0.0428739
0.6291	11921.8859	0.76722827	178.763757	1.86651052	0	-0.4634651	2.47845974	-0.2636684	5.18607074	0.62460627	-6.9077553	5	54	3.57631111	305.777778	32.7777778	0	0	1	0	18.9135952	1	-0.0428739
0.9576	21623.1138	0.8238553	210.243001	2.264818	0	-0.0433251	3.07380907	-0.1925473	5.34826877	0.81793584	-6.9077553	6	49	4.02335	307.444444	34.4444444	0	0	1	0	18.9135952	1	-0.0428739
1.4211	37337.1816	0.85374074	225.014563	2.24907416	0	0.35143122	3.62001644	-0.1569571	5.41616957	0.81096318	-6.9077553	7	51	4.02335	306.333333	33.33333333	0	0	1	0	18.9135952	1	-0.0428739
1.8972	56239.6287	0.85243903	224.071391	1.92571191	0	0.64037912	4.02963943	-0.1584812	5.41196917	0.65581488	-6.9077553	8	50	2.68223333	306.333333	33.33333333	0	0	1	0	18.9135952	1	-0.0428739
2.4003	74131.6053	0.84152822	211.262508	1.98211703	0	0.87559373	4.30585545	-0.1713481	5.35310621	0.68466986	-6.9077553	9	52	3.12927222	306.333333	33.33333333	0	0	1	0	18.9135952	1	-0.0428739
2.6694	85042.5751	0.80419417	177.82026	1.42073748	0	0.98185373	4.44316377	-0.2166718	5.18077889	0.3518797	-6.9077553	10	81	2.23519444	300.222222	27.2222222	0	0	1	0	18.9135952	1	-0.0428739
2.7657	89648.2713	0.46075119	53.9067657	0.75247349	0	1.01729377	4.49590507	-0.7727291	3.98727454	-0.2830614	-6.9077553	11	89	1.34111667	298	25	0	0	1	0	18.9135952	1	-0.0428739
2.8188	92060.778	0.29505339	19.3316485	0.46836142	0	1.03631126	4.52245985	-1.2172155	2.9617953	-0.7563822	-6.9077553	12	94	2.23519444	296.888889	23.88888888	0	0	1	0	18.9135952	1	-0.0428739
2.8827	95087.3803	0.26631224	15.3737002	0.41937283	0	1.05872735	4.55480678	-1.3193379	2.73272331	-0.8666133	-6.9077553	13	94	1.34111667	297.444444	24.44444444	0	0	1	0	18.9135952	1	-0.0428739
2.943	97763.0687	0.27784091	16.6380306	0.44187933	0	1.07942947	4.58255711	-1.2771139	2.81175117	-0.8144579	-6.9077553	14	95	2.23519444	296.888889	23.88888888	0	0	1	0	18.9135952	1	-0.0428739
3.0168	101008.991	0.27630492	16.5103507	0.42587946	0	1.10419667	4.61521943	-1.2826376	2.80404806	-0.8512536	-6.9077553	15	93	2.23519444	296.888889	23.88888888	0	0	1	0	18.9135952	1	-0.0428739
3.0834	104079.458	0.2724022	16.2219585	0.43756537	0	1.12603288	4.64516424	-1.2968113	2.78642743	-0.8242464	-6.9077553	16	95	1.78815556	296.333333	23.33333333	0	0	1	0	18.9135952	1	-0.0428739
3.1446	106755.15	0.25603892	14.4370593	0.41845167	0	1.1456867	4.67054726	-1.3585278	2.66986773	-0.868807	-6.9077553	17	94	2.68223333	296.888889	23.88888888	0	0	1	0	18.9135952	1	-0.0428739

3.2175	109913.346	0.2688033	15.8913544	0.43468805	0	1.16860466	4.69970139	-1.3100621	2.76583814	-0.8308288	-6.9077553	18	94	2.23519444	296.888889	23.88888888	0	0	1	0	18.9135952	1	-0.0428739
3.2823	112896.083	0.27751149	17.0189226	0.44555738	0	1.1885444	4.72647663	-1.278296	2.83438458	-0.8061874	-6.9077553	19	89	3.57631111	298	25	0	0	1	0	18.9135952	1	-0.0428739
3.3417	115659.502	0.29665178	19.702596	0.50611843	0	1.20647966	4.7506592	-1.211831	2.98080116	-0.6790107	-6.9077553	20	91	4.47038889	298	25	0	0	1	0	18.9135952	1	-0.0428739
3.4065	118598.368	0.2716705	16.0468004	0.4129866	0	1.22568537	4.77575116	-1.2994912	2.77557179	-0.8819217	-6.9077553	21	93	4.47038889	296.888889	23.88888888	0	0	1	0	18.9135952	1	-0.0428739
3.4614	121098.603	0.26627766	15.6456739	0.41956473	0	1.24167313	4.79661337	-1.3194672	2.75025836	-0.8661569	-6.9077553	22	94	4.91742778	296.333333	23.33333333	0	0	1	0	18.9135952	1	-0.0428739
3.5532	124464.584	0.36306983	51.8041849	0.89657615	0	1.26784861	4.82402924	-1.0104096	3.94749024	-0.1080573	-6.9077553	23	94	4.47038889	295.777778	22.7777778	0	0	1	0	18.9135952	1	-0.0428739
0.0009	0	0	0	0	0	-7.0131158	-6.9077553	-6.9077553	-6.9077553	-6.9077553	-6.9077553	0	76	2.68223333	301.888889	28.88888888	0	0	0	0	30.519848	1	0.21141735
0.0027	43.8637829	0.66915679	109.426636	1.14286661	0	-5.9145035	-3.1041244	-0.4002436	4.69526347	0.13441429	-6.9077553	1	78	2.68223333	301.333333	28.33333333	0	0	0	0	30.519848	1	0.21141735
0.0063	219.318913	0.40768972	38.7048912	0.56892568	0	-5.0672056	-1.5126792	-0.894799	3.65599182	-0.5622493	-6.9077553	2	81	3.12927222	300.777778	27.7777778	0	0	0	0	30.519848	1	0.21141735
0.0108	438.637827	0.3505127	26.7011047	0.46723471	0	-4.5282091	-0.821804	-1.0455095	3.28474239	-0.7587856	-6.9077553	3	84	3.12927222	300.777778	27.7777778	0	0	0	0	30.519848	1	0.21141735
0.0144	570.229192	0.35474774	27.3886585	0.5443821	0	-4.2405271	-0.5599648	-1.0335334	3.31016552	-0.6062686	-6.9077553	4	86	4.02335	300.222222	27.2222222	0	0	0	0	30.519848	1	0.21141735
0.0198	789.548209	0.26849641	15.3594119	0.44802491	0	-3.9220733	-0.2350286	-1.3112002	2.73179354	-0.8006769	-6.9077553	5	88	3.12927222	300.222222	27.2222222	0	0	0	0	30.519848	1	0.21141735
0.027	1052.73091	0.28051791	17.1149731	0.47339839	0	-3.6119184	0.05233711	-1.2675592	2.84001213	-0.7457078	-6.9077553	6	90	2.23519444	299.666667	26.66666666	0	0	0	0	30.519848	1	0.21141735
0.0324	1140.4584	0.27548538	16.4970996	0.47422449	0	-3.4295969	0.13230674	-1.2855973	2.8032452	-0.743968	-6.9077553	7	90	2.68223333	299.666667	26.66666666	0	0	0	0	30.519848	1	0.21141735
0.036	1272.04984	0.20238841	9.07671547	0.3481383	0	-3.3242363	0.24141547	-1.5926378	2.20582256	-1.0522872	-6.9077553	8	89	2.68223333	299.666667	26.66666666	0	0	0	0	30.519848	1	0.21141735
0.036	1272.04984	0	0	0	0	-3.3242363	0.24141547	-6.9077553	-6.9077553	-6.9077553	-6.9077553	9	88	3.12927222	300.222222	27.2222222	0	0	0	0	30.519848	1	0.21141735
0.0702	1579.0961	0.33373368	31.4958046	1.30668981	0	-2.656407	0.45748567	-1.09442	3.4498861	0.26826208	-6.9077553	10	81	4.02335	301.888889	28.88888888	0	0	0	0	30.519848	1	0.21141735
0.1647	1926.59787	0.3999321	39.7552269	1.97917553	0	-1.8036296	0.6562746	-0.9139632	3.68276648	0.68318549	-6.9077553	11	75	5.81150556	301.888889	28.88888888	0	0	0	0	30.519848	1	0.21141735
0.2556	3045.049	0.60329845	108.600325	2.02321934	0	-1.3641416	1.11384534	-0.5036871	4.68768361	0.70518412	-6.9077553	12	65	4.91742778	304.111111	31.11111111	0	0	0	0	30.519848	1	0.21141735
0.3582	4179.96007	0.62493917	107.531498	2.0187054	0	-1.0266638	1.4305409	-0.4685021	4.67779311	0.70295166	-6.9077553	13	57	6.25854444	305.222222	32.2222222	0	0	0	1	30.519848	1	0.21141735
0.5256	8015.70445	0.70946642	144.601668	2.17695257	0	-0.6432148	2.08152742	-0.3418336	4.97398976	0.77838525	-6.9077553	14	52	5.81150556	306.888889	33.88888888	0	0	0	1	30.519848	1	0.21141735
0.7758	17616.2639	0.77265524	172.85263	2.27676282	0	-0.2538605	2.86887933	-0.2566289	5.15244517	0.82319374	-6.9077553	15	50	4.47038889	308	35	0	0	0	1	30.519848	1	0.21141735
1.0791	27807.7724	0.71149306	142.2205	2.0302153	0	0.07612736	3.32535152	-0.3389851	4.9573857	0.70863428	-6.9077553	16	46	5.36446667	308.555556	35.5555556	0	0	0	1	30.519848	1	0.21141735
1.332	38511.6267	0.68820641	135.328695	1.47940096	0	0.28668157	3.65098615	-0.3722145	4.90771399	0.39231297	-6.9077553	17	48	2.68223333	308.555556	35.5555556	0	0	0	1	30.519848	1	0.21141735
1.5507	48359.3644	0.64319162	116.864407	1.21750518	0	0.43870644	3.87868056	-0.4397591	4.7610229	0.19762485	-6.9077553	18	49	3.12927222	307.444444	34.4444444	0	0	0	1	30.519848	1	0.21141735
1.7307	56861.5753	0.72068359	154.713584	1.23415009	0	0.54852595	4.0406374	-0.3261685	5.04158203	0.21119249	-6.9077553	19	52	1.34111667	307.444444	34.4444444	0	0	0	1	30.519848	1	0.21141735
1.8531	65878.1083	0.76168453	161.670784	1.15025868	0	0.61685991	4.18782137	-0.2709108	5.08556825	0.14085585	-6.9077553	20	70	3.57631111	301.333333	28.33333333	0	0	0	1	30.519848	1	0.21141735
1.9197	70898.7909	0.61024398	98.0157966	0.91438788	0	0.65216892	4.26126748	-0.4922591	4.58513886	-0.0884074	-6.9077553	21	84	2.68223333	299.111111	26.11111111	0	0	0	1	30.519848	1	0.21141735
1.9404	71819.9363	0.36035525	29.5245255	0.56983857	0	0.66289414	4.27417603	-1.0178937	3.38525516	-0.5606488	-6.9077553	22	87	0.44703889	299.111111	26.11111111	0	0	0	1	30.519848	1	0.21141735
1.9602	72741.0628	0.26558976	15.0877103	0.39670298	0	0.67304651	4.2869198	-1.3220443	2.7139468	-0.9220498	-6.9077553	23	83	0.89407778	300.222222	27.2222222	0	0	0	1	30.519848	1	0.21141735
0.0009	0	0	0	0	0	-7.0131158	-6.9077553	-6.9077553	-6.9077553	-6.9077553	-6.9077553	0	76	2.68223333	301.888889	28.88888888	0	0	0	0	34.0724638	1	0.49277013
0.0072	263.182695	0.67307799	111.891078	1.19319943	0	-4.9336743	-1.3311144	-0.3944095	4.71753482	0.17747603	-6.9077553	1	78	2.68223333	301.333333	28.33333333	0	0	0	0	34.0724638	1	0.49277013
0.0171	701.820524	0.52155724	65.2426314	0.82146124	0	-4.0686768	-0.3526537	-0.6490207	4.17812844	-0.1954539	-6.9077553	2	81	3.12927222	300.777778	27.7777778	0	0	0	0	34.0724638	1	0.49277013
0.0252	1008.867	0.38574394	32.9922757	0.5063058	0	-3.6809113	0.00981864	-0.9499925	3.49630377	-0.6786413	-6.9077553	3	84	3.12927222	300.777778	27.7777778	0	0	0	0	34.0724638	1	0.49277013
0.0333	1228.18602	0.32169154	22.8542294	0.51100413	0	-3.4021979	0.20635218	-1.1310584	3.12917995	-0.6694226	-6.9077553	4	86	4.02335	300.222222	27.2222222	0	0	0	0	34.0724638	1	0.49277013
0.0396	1447.50485	0.20964594	8.98685455	0.21807476	0	-3.2289262	0.37053188	-1.5575766	2.19587417	-1.5183422	-6.9077553	5	88	3.12927222	300.222222	27.2222222	0	0	0	0	34.0724638	1	0.49277013
0.0432	1622.96006	0.2488503	12.7005272	0.32803589	0	-3.1419148	0.48486765	-1.3868933	2.54172224	-1.1115884	-6.9077553	6	90	2.23519444	299.666667	26.66666666	0	0	0	0	34.0724638	1	0.49277013
0.0513	1973.87015	0.2517439	13.6469667	0.40705649	0	-2.9700645	0.68050265	-1.3753786	2.61359055	-0.8963497	-6.9077553	7	90	2.68223333	299.666667	26.66666666	0	0	0	0	34.0724638	1	0.49277013
0.0603	2324.78056	0.26754208	16.0585815	0.48507571	0	-2.8084232	0.84405571	-1.3147477	2.77630565	-0.7213909	-6.9077553	8	89	2.68223333	299.666667	26.66666666	0	0	0	0	34.0724638	1	0.49277013
0.0675	2675.69079	0.25456548	13.4710712	0.37138629	0	-2.6956277	0.98458125	-1.3642766	2.60061875	-0.9878236	-6.9077553	9	88	3.12927222	300.222222	27.2222222	0	0	0	0	34.0724638	1	0.49277013
0.1035	4160.65216	0.68630419	134.371045	1.53283939	0	-2.2681837	1.42591215	-0.3749783	4.90061241	0.427774	-6.9077553	10	81	4.02335	301.888889	28.88888888	0	0	0	0	34.0724638	1	0.49277013
0.1773	7515.3922	0.86674137	204.712224	1.70343539	0	-1.7299121	2.01708626	-0.1418616	5.32161009	0.5332339	-6.9077553	11	75	5.81150556	301.888889	28.88888888	0	0	0	0	34.0724638	1	0.49277013
0.2844	12738.191	0.99120889	278.586988	2.26383754	0	-1.2573736	2.54468314	-0.0078216	5.62973394	0.81750303	-6.9077553	12	65	4.91742778	304.111111	31.11111111	0	0	0	0	34.0724638	1	0.49277013
0.5562	25780.6213	1.2512298	484.796978	4.07093457	0	-0.5866273	3.24966189	0.2249258	6.18373226	1.40411821	-6.9077553	13	57	6.25854444	305.222222	32.2222222	0	0	0	1	34.0724638	1	0.49277013
1.1781	47479.3721	1.33856069	527.708575	4.69281704	0	0.16390297	3.86031641	0.29234171	6.26854608	1.54624612	-6.9077553	14	52	5.81150556	306.888889	33.88888888	0	0	0	1	34.0724638	1	0.49277013
1.7091	65054.7617	1.1167735	359.701642	2.76317035	0	0.53596692	4.17524477	0.11133876	5.8852777	1.01674054	-6.9077553	15	50	4.47038889	308	35	0	0	0	1	34.0724638	1	0.49277013
2.2896	81963.4986	1.05487392	317.48251	2.74885197	0	0.82837713	4.40628621	0.05436878	5.76042588	1.01154708	-6.9077553	16	46	5.36446667	308.555556	35.5555556	0	0	0	1	34.0724638	1	0.49277013
2.772	99394.9968	0.98993284	273.805125	2.19363335	0	1.01956908	4.59911184	-0.0091085	5.61242028	0.78601499	-6.9077553	17	48	2.68223333	308.555556	35.5555556	0	0	0	1	34.0724638	1	0.49277013
3.2598	115051.723	0.91895995	234.621425	2.06939022	0	1.18166584	4.74539048	-0.0834251	5.45797752	0.7277371	-6.9077553	18	49	3.12927222	307.444444	34.4444444	0	0	0	1	34.0724638	1	0.49277013
3.7152	129252.771	0.83050682	192.373487	1.56061418	0	1.31243251	4.86177769	-0.1845158	5.25944393	0.44572002	-6.9077553	19	52	1.34111667	307.444444	34.4444444	0	0	0	1	34.0724638	1	0.49277013
3.9933	139556.138	0.90398953	235.057014	1.75452924	0	1.38461796	4.93847411	-0.0998319	5.45983235	0.56277037	-6.9077553	20	70	3.57631111	301.333333	28.33333333	0	0	0	1	34.0724638	1	0.49277013
4.1508	146279.321	0.69917319	129.922887	1.25214207	0	1.42330109	4.98552479	-0.3564276	4.86694856	0.22565406	-6.9077553	21	84	2.68223333	299.111111	26.11111111	0	0	0	1	34.0724638	1	0.49277013
4.1976	148253.2	0.23704411	12.0725638	0.43014112	0	1.43451293	4.99892837	-1.4352993	2.49101825	-0.8413198	-6.9077553	22	87	0.44703889	299.111111	26.11111111	0	0	0	1	34.0724638	1	0.49277013
4.2372	149876.156	0.25459723	13.8335211	0.4188867	0	1.44390267	5.009816	-1.3641524	2.62716699	-0.8677704	-6.9077553	23	83	0.89407778	300.222222	27.2222222	0	0	0	1	34.0724638	1	0.49277013
0.0009	0	0	0	0	0	-7.0131158	-6.9077553	-6.9077553	-6.9077553	-6.9077553	-6.9077553	0	76	2.68223333	301.888889	28.88888888	0	0	0	0	42.288408	1	-0.4111751
0.009	394.774045	0.69106706	119.156521	1.26937781	0	-4.7105307	-0.9269118	-0.3680724	4.78044633	0.23931435	-6.9077553	1	78	2.68223333	301.333333	28.33333333	0	0	0	0	42.288408	1	-0.4111751
0.0369	1754.55131	0.63910509	101.938225	1.20981942	0	-3.2995437	0.56278294	-0.4461229	4.6243768	0.19129734	-6.9077553	2	81	3.12927222	300.777778	27.7777778	0	0	0	0	42.288408	1	-0.4111751
0.072	3465.23818	0.51158211	63.4769444	0.92842034	0	-2.6310892	1.24306991	-0.6682944	4.15069251	-0.0731942	-6.9077553	3	84	3.12927222	300.777778	27.7777778	0	0	0	0	42.288408	1	-0.4111751
0.0963	4605.69687	0.35908826	29.9601237	0.67160868	0	-2.340287	1.52751109	-1.0214061	3.39990066	-0.3965916	-6.9077553	4	86	4.02335	300.222222	27.2222222	0	0	0	0	42.288408	1	-0.4111751
0.1197	5746.1554	0.28595326	18.9124263	0.50239948	0	-2.1227667	1.74870502	-1.2484359	2.93987206	-0.6863712	-6.9077553	5	88	3.12927222	300.222222	27.2222222	0	0	0	0	42.288408	1	-0.4111751
0.1395	6711.15823	0.28788168	18.7374767	0.49649083	0	-1.9696907	1.90392054	-1.2417381	2.93057899	-0.6981782	-6.9077553	6	90	2.23519444	299.666667	26.66666666	0	0	0	0	42.288408	1	-0.4111751
0.171	8246.39042	0.28626548	17.9731253	0.48816675	0	-1.7660917	2.10989684	-1.2473485	2.88893324	-0.7150518	-6.9077553	7	90	2.68223333	299.666667	26.66666666	0	0	0	0	42.288408	1	-0.4111751

0.1962	9430.71358	0.29480505	18.9669538	0.5154704	0	-1.6286207	2.2440778	-1.2180547	2.94275091	-0.6607373	-6.9077553	8	89	2.68223333	299.666667	26.66666666	0	0	0	0	42.288408	1	-0.4111751
0.2349	11242.169	0.47556726	73.4733376	0.86884201	0	-1.4485954	2.41976074	-0.7411464	4.2969362	-0.1394437	-6.9077553	9	88	3.12927222	300.222222	27.2222222	0	0	0	0	42.288408	1	-0.4111751
0.414	17690.8676	0.81401507	191.772103	2.34890916	0	-0.8818893	2.87310508	-0.2045487	5.25631292	0.85437667	-6.9077553	10	81	4.02335	301.888889	28.88888888	0	0	0	0	42.288408	1	-0.4111751
0.6444	27284.7312	0.86825716	212.99968	2.08515215	0	-0.4394356	3.3063639	-0.1401163	5.36129536	0.73532129	-6.9077553	11	75	5.81150556	301.888889	28.88888888	0	0	0	0	42.288408	1	-0.4111751
0.9747	39377.846	0.87154195	205.196015	2.06811158	0	-0.0256255	3.67322877	-0.1363445	5.32397057	0.72711932	-6.9077553	12	65	4.91742778	304.111111	31.11111111	0	0	1	0	42.288408	1	-0.4111751
1.4022	55756.6014	0.90965865	228.702845	2.45284411	0	0.33804243	4.02101375	-0.0935871	5.43242792	0.89765582	-6.9077553	13	57	6.25854444	305.222222	32.2222222	0	0	1	0	42.288408	1	-0.4111751
1.9476	75333.0137	0.86702494	207.799376	2.12989611	0	0.66659785	4.32193174	-0.1415348	5.33657789	0.7565426	-6.9077553	14	52	5.81150556	306.888889	33.88888888	0	0	1	0	42.288408	1	-0.4111751
2.5929	101673.067	0.95009699	252.003334	2.36627206	0	0.95277694	4.62177227	-0.0501392	5.52944629	0.86173826	-6.9077553	15	50	4.47038889	308	35	0	0	1	0	42.288408	1	-0.4111751
3.3453	137212.205	1.00999268	294.284054	2.41744192	0	1.20755638	4.92153596	0.0109327	5.68454887	0.8831235	-6.9077553	16	46	5.36446667	308.555556	35.5555556	0	0	1	0	42.288408	1	-0.4111751
4.1067	174777.539	0.99555937	280.137762	2.14735243	0	1.41261979	5.16351968	-0.0034466	5.63528506	0.76470124	-6.9077553	17	48	2.68223333	308.555556	35.5555556	0	0	1	0	42.288408	1	-0.4111751
4.7925	204915.118	0.95677173	262.251825	2.10174384	0	1.5670522	5.32260071	-0.0431458	5.56930902	0.74324308	-6.9077553	18	49	3.12927222	307.444444	34.4444444	0	0	1	0	42.288408	1	-0.4111751
5.2551	223898.59	0.81920271	188.056755	1.40263313	0	1.65919903	5.41119769	-0.1982038	5.23674912	0.33906397	-6.9077553	19	52	1.34111667	307.444444	34.4444444	0	0	1	0	42.288408	1	-0.4111751
5.5602	237138.937	0.84052639	195.627627	1.48092867	0	1.71563408	5.46865042	-0.1725379	5.2762181	0.3933444	-6.9077553	20	70	3.57631111	301.333333	28.33333333	0	0	1	0	42.288408	1	-0.4111751
5.7591	245038.962	0.69847528	129.658645	1.18374399	0	1.75078121	5.50142131	-0.3574248	4.8649129	0.16952671	-6.9077553	21	84	2.68223333	299.111111	26.11111111	0	0	1	0	42.288408	1	-0.4111751
5.8086	247144.431	0.23354534	11.9321112	0.39768954	0	1.75933958	5.50997695	-1.4501064	2.47931699	-0.9195723	-6.9077553	22	87	0.44703889	299.111111	26.11111111	0	0	1	0	42.288408	1	-0.4111751
5.8608	249381.479	0.2362504	12.1339143	0.34204582	0	1.76828611	5.51898778	-1.4386392	2.49608678	-1.0698913	-6.9077553	23	83	0.89407778	300.222222	27.2222222	0	0	1	0	42.288408	1	-0.4111751
0.0009	0	0	0	0	0	-7.0131158	-6.9077553	-6.9077553	-6.9077553	-6.9077553	-6.9077553	0	76	2.68223333	301.888889	28.88888888	0	0	0	0	18.9135952	1	-0.0428739
0.0009	0	0	0	0	0	-7.0131158	-6.9077553	-6.9077553	-6.9077553	-6.9077553	-6.9077553	1	78	2.68223333	301.333333	28.33333333	0	0	0	0	18.9135952	1	-0.0428739
0.0009	0	0	0	0	0	-7.0131158	-6.9077553	-6.9077553	-6.9077553	-6.9077553	-6.9077553	2	81	3.12927222	300.777778	27.7777778	0	0	0	0	18.9135952	1	-0.0428739
0.0009	0	0	0	0	0	-7.0131158	-6.9077553	-6.9077553	-6.9077553	-6.9077553	-6.9077553	3	84	3.12927222	300.777778	27.7777778	0	0	0	0	18.9135952	1	-0.0428739
0.0009	0	0	0	0	0	-7.0131158	-6.9077553	-6.9077553	-6.9077553	-6.9077553	-6.9077553	4	86	4.02335	300.222222	27.2222222	0	0	0	0	18.9135952	1	-0.0428739
0.0009	0	0	0	0	0	-7.0131158	-6.9077553	-6.9077553	-6.9077553	-6.9077553	-6.9077553	5	88	3.12927222	300.222222	27.2222222	0	0	0	0	18.9135952	1	-0.0428739
0.0009	0	0	0	0	0	-7.0131158	-6.9077553	-6.9077553	-6.9077553	-6.9077553	-6.9077553	6	90	2.23519444	299.666667	26.66666666	0	0	0	0	18.9135952	1	-0.0428739
0.0009	0	0	0	0	0	-7.0131158	-6.9077553	-6.9077553	-6.9077553	-6.9077553	-6.9077553	7	90	2.68223333	299.666667	26.66666666	0	0	0	0	18.9135952	1	-0.0428739
0.0009	0	0	0	0	0	-7.0131158	-6.9077553	-6.9077553	-6.9077553	-6.9077553	-6.9077553	8	89	2.68223333	299.666667	26.66666666	0	0	0	0	18.9135952	1	-0.0428739
0.0009	0	0	0	0	0	-7.0131158	-6.9077553	-6.9077553	-6.9077553	-6.9077553	-6.9077553	9	88	3.12927222	300.222222	27.2222222	0	0	0	0	18.9135952	1	-0.0428739
0.0009	0	0	0	0	0	-7.0131158	-6.9077553	-6.9077553	-6.9077553	-6.9077553	-6.9077553	10	81	4.02335	301.888889	28.88888888	0	0	0	0	18.9135952	1	-0.0428739
0.0171	0	0.65739176	114.137939	3.56400977	0	-4.0686768	-6.9077553	-0.4179551	4.73741647	1.2711668	-6.9077553	11	75	5.81150556	301.888889	28.88888888	0	0	0	0	18.9135952	1	-0.0428739
0.0846	192.316412	0.58654841	100.45	2.30456014	0	-2.469821	-1.643427	-0.5317966	4.60967005	0.83532366	-6.9077553	12	65	4.91742778	304.111111	31.11111111	0	0	0	0	18.9135952	1	-0.0428739
0.3051	7141.79128	0.88781016	265.408913	2.77521923	0	-1.1871157	1.96610363	-0.1178716	5.58127547	1.02109001	-6.9077553	13	57	6.25854444	305.222222	32.2222222	0	0	1	0	18.9135952	1	-0.0428739
0.5373	13823.2794	0.8377449	241.259569	2.59641331	0	-0.6211987	2.62642642	-0.1758487	5.48587755	0.95451607	-6.9077553	14	52	5.81150556	306.888889	33.88888888	0	0	1	0	18.9135952	1	-0.0428739
0.7092	18022.6302	0.73810086	172.405657	1.69603901	0	-0.3436177	2.89168369	-0.3023209	5.14985597	0.52888497	-6.9077553	15	50	4.47038889	308	35	0	0	1	0	18.9135952	1	-0.0428739
0.963	26916.512	0.8384315	218.632203	1.90556952	0	-0.0377019	3.29277708	-0.1750304	5.38739545	0.64530557	-6.9077553	16	46	5.36446667	308.555556	35.5555556	0	0	1	0	18.9135952	1	-0.0428739
1.2195	34090.2473	0.75593968	167.1267	1.81056015	0	0.19844094	3.52904068	-0.2784717	5.11875819	0.59418844	-6.9077553	17	48	2.68223333	308.555556	35.5555556	0	0	1	0	18.9135952	1	-0.0428739
1.5201	46217.0394	0.88915312	234.702096	1.90870182	0	0.41877612	3.83337019	-0.1163618	5.45832129	0.64694712	-6.9077553	18	49	3.12927222	307.444444	34.4444444	0	0	1	0	18.9135952	1	-0.0428739
1.7541	54916.3554	0.76288757	170.508534	1.34557448	0	0.5619559	4.00582943	-0.2693347	5.13879121	0.29756395	-6.9077553	19	52	1.34111667	307.444444	34.4444444	0	0	1	0	18.9135952	1	-0.0428739
1.9413	63069.8386	0.8457957	201.04049	1.5493949	0	0.66335785	4.14425852	-0.1662958	5.3035113	0.43850967	-6.9077553	20	70	3.57631111	301.333333	28.33333333	0	0	1	0	18.9135952	1	-0.0428739
2.0844	69259.9514	0.74189581	144.697155	1.28480808	0	0.73448104	4.23788128	-0.2971995	4.97464988	0.25138738	-6.9077553	21	84	2.68223333	299.111111	26.11111111	0	0	1	0	18.9135952	1	-0.0428739
2.1375	71672.4602	0.365838	34.0561213	0.62156509	0	0.75963692	4.27212053	-1.0028349	3.52803915	-0.4739071	-6.9077553	22	87	0.44703889	299.111111	26.11111111	0	0	1	0	18.9135952	1	-0.0428739
2.1762	73427.0135	0.24809908	13.2166606	0.42140491	0	0.77758024	4.29630552	-1.3899045	2.58155386	-0.8617909	-6.9077553	23	83	0.89407778	300.222222	27.2222222	0	0	1	0	18.9135952	1	-0.0428739
0.0171	494.230791	0.84927421	194.093614	1.82132106	0	-4.0686768	-0.7027314	-0.1621964	5.26834574	0.600111	-6.9077553	0	60	3.57631111	304.666667	31.66666666	0	0	0	0	30.519848	1	0.21141735
0.1296	2622.51047	0.89094641	233.959297	3.95503052	0	-2.0433025	0.96451329	-0.1143492	5.45515143	1.37524113	-6.9077553	1	57	4.91742778	304.111111	31.11111111	0	0	0	0	30.519848	1	0.21141735
0.2304	4582.71428	0.6543699	121.180743	1.55656392	0	-1.4679384	1.52250965	-0.4225555	4.79729142	0.44312301	-6.9077553	2	70	1.78815556	303	30	0	0	0	0	30.519848	1	0.21141735
0.5724	6934.85889	0.78519905	174.678701	3.66481323	0	-0.5579172	1.93670489	-0.2405453	5.16295401	1.2990502	-6.9077553	3	51	4.47038889	306.888889	33.88888888	0	0	0	1	30.519848	1	0.21141735
1.0602	10663.1113	0.83543728	211.845999	3.08807666	0	0.05845757	2.36688402	-0.1786037	5.35586431	1.12787223	-6.9077553	4	50	3.57631111	306.888889	33.88888888	0	0	0	1	30.519848	1	0.21141735
1.6479	17677.0154	0.78909091	180.686088	2.41063107	0	0.49950175	2.8723218	-0.2356073	5.19676674	0.88030331	-6.9077553	5	52	3.57631111	306.888889	33.88888888	0	0	0	1	30.519848	1	0.21141735
2.3256	26185.8097	0.8211571	195.047435	2.4745421	0	0.84397807	3.26525584	-0.1958238	5.27324791	0.9064594	-6.9077553	6	43	4.02335	308	35	0	0	0	1	30.519848	1	0.21141735
2.7036	32517.5571	0.76579299	165.009375	1.62015584	0	0.99458422	3.48181091	-0.2655384	5.10600835	0.48313938	-6.9077553	7	48	3.12927222	307.444444	34.4444444	0	0	0	1	30.519848	1	0.21141735
3.0447	40848.1668	0.74421817	163.681316	1.39574648	0	1.11340237	3.70988642	-0.2940783	5.09792745	0.33414559	-6.9077553	8	50	2.23519444	306.888889	33.88888888	0	0	0	1	30.519848	1	0.21141735
3.2742	46731.9816	0.7391428	180.394393	1.38593738	0	1.18607356	3.84445016	-0.3009121	5.19515107	0.32709799	-6.9077553	9	57	0.44703889	304.666667	31.66666666	0	0	0	1	30.519848	1	0.21141735
3.339	48319.8543	0.61652231	113.770549	0.74017848	0	1.20567136	3.87786323	-0.4820401	4.73419248	-0.2995138	-6.9077553	10	73	0.89407778	301.333333	28.33333333	0	0	0	1	30.519848	1	0.21141735
3.3489	48670.7635	0.43907907	47.9368064	0.55120624	0	1.20863193	3.88509906	-0.8208009	3.86990447	-0.5938337	-6.9077553	11	76	0.44703889	300.222222	27.2222222	0	0	0	1	30.519848	1	0.21141735
3.3597	49109.4005	0.43084137	44.3841146	0.63935518	0	1.21185168	3.89407084	-0.839697	3.79290416	-0.4457323	-6.9077553	12	75	1.78815556	300.777778	27.7777778	0	0	0	1	30.519848	1	0.21141735
3.3687	49504.174	0.33823161	25.5608337	0.53468175	0	1.21452691	3.90207719	-1.0810722	3.24110037	-0.624215	-6.9077553	13	76	2.23519444	300.222222	27.2222222	0	0	0	1	30.519848	1	0.21141735
3.3714	49548.0376	0.23745787	11.8807805	0.28243391	0	1.21532809	3.90296284	-1.4335626	2.47500618	-1.2607763	-6.9077553	14	79	3.12927222	300.222222	27.2222222	0	0	0	1	30.519848	1	0.21141735
3.3732	49635.7658	0.26294461	14.4922113	0.36173686	0	1.21586185	3.90473181	-1.332016	2.67368036	-1.0140776	-6.9077553	15	83	4.02335	299.666667	26.66666666	0	0	0	1	30.519848	1	0.21141735
3.3732	49635.7658	0	0	0	0	1.21586185	3.90473181	-6.9077553	-6.9077553	-6.9077553	-6.9077553	16	87	4.02335	299.111111	26.11111111	0	0	0	1	30.519848	1	0.21141735
3.3732	49635.7658	0	0	0	0	1.21586185	3.90473181	-6.9077553	-6.9077553	-6.9077553	-6.9077553	17	89	3.57631111	298.555556	25.55555555	0	0	0	1	30.519848	1	0.21141735
3.3732	49635.7658	0	0	0	0	1.21586185	3.90473181	-6.9077553	-6.9077553	-6.9077553	-6.9077553	18	92	4.02335	298.555556	25.55555555	0	0	0	1	30.519848	1	0.21141735
3.3732	49635.7658	0	0	0	0	1.21586185	3.90473181	-6.9077553	-6.9077553	-6.9077553	-6.9077553	19	88	4.91742778	298.555556	25.55555555	0	0	0	1	30.519848	1	0.21141735
3.3732	49635.7658	0	0	0	0	1.21586185	3.90473181	-6.9077553	-6.9077553	-6.9077553	-6.9077553	20	87	4.02335	298.555556	25.55555555	0	0	0	1	30.519848	1	0.21141735
3.4155	49822.0553	0.26902588	23.818936	1.15043713	0	1.2283239	3.90847783	-1.3092375	3.17052288	0.14101084	-6.9077553	21	82	4.91742778	299.666667	26.66666666	0	0	0	1	30.519848	1	0.21141735

3.6036	50426.8453	0.39556206	43.5307827	1.44953006	0	1.28193335	3.92054351	-0.9249227	3.77349131	0.37192905	-6.9077553	22	74	5.36446667	301.333333	28.33333333	0	0	0	1	30.519848	1	0.21141735
4.0275	52615.2168	0.56629144	87.6355494	1.55952003	0	1.39314584	3.96302438	-0.5668821	4.47319814	0.44501912	-6.9077553	23	68	5.36446667	302.444444	29.44444444	0	0	0	1	30.519848	1	0.21141735
0.0432	2052.4322	1.1320356	373.911569	3.31794934	0	-3.1419148	0.71951264	0.1249004	5.924022	1.19964827	-6.9077553	0	60	3.57631111	304.666667	31.66666666	0	0	0	0	34.0724638	1	0.49277013
0.099	4702.32662	0.89469405	217.542055	1.38688004	0	-2.3126354	1.54827005	-0.1101564	5.38239679	0.32777772	-6.9077553	1	57	4.91742778	304.111111	31.11111111	0	0	0	0	34.0724638	1	0.49277013
0.1737	7468.99215	0.84606695	188.455549	1.28629128	0	-1.7504256	2.01089395	-0.1659755	5.23886747	0.25254023	-6.9077553	2	70	1.78815556	303	30	0	0	0	0	34.0724638	1	0.49277013
0.2601	10274.1215	0.84405835	185.634135	1.91449865	0	-1.3466891	2.32972558	-0.1683496	5.22378311	0.64997798	-6.9077553	3	51	4.47038889	306.888889	33.88888888	0	0	0	1	34.0724638	1	0.49277013
0.3375	13849.307	0.91342713	218.8182	1.52514223	0	-1.0861898	2.6283074	-0.0894575	5.38824582	0.42274313	-6.9077553	4	50	3.57631111	306.888889	33.88888888	0	0	0	1	34.0724638	1	0.49277013
0.5238	23254.8753	1.05236522	309.484432	2.34960112	0	-0.6466453	3.1465578	0.05199001	5.73491102	0.85467109	-6.9077553	5	52	3.57631111	306.888889	33.88888888	0	0	0	1	34.0724638	1	0.49277013
0.819	36208.6662	1.06346447	309.647866	2.71370902	0	-0.1996712	3.5893261	0.06247182	5.73543896	0.99868477	-6.9077553	6	43	4.02335	308	35	0	0	0	1	34.0724638	1	0.49277013
1.206	54248.2608	1.04177885	302.78323	2.499646	0	0.1873091	3.99358937	0.04188912	5.71302044	0.9165491	-6.9077553	7	48	3.12927222	307.444444	34.4444444	0	0	0	1	34.0724638	1	0.49277013
1.6173	70020.4534	0.92292729	237.25106	1.85622374	0	0.48075809	4.24880167	-0.0791219	5.46912312	0.61908276	-6.9077553	8	50	2.23519444	306.888889	33.88888888	0	0	0	1	34.0724638	1	0.49277013
1.9773	82965.5119	0.80898132	184.128672	1.39404068	0	0.68173228	4.41843705	-0.2107441	5.21564025	0.33292358	-6.9077553	9	57	0.44703889	304.666667	31.66666666	0	0	0	1	34.0724638	1	0.49277013
2.1933	91944.5617	0.81128241	184.110173	1.3923762	0	0.78540726	4.52119668	-0.2079072	5.21553977	0.33172972	-6.9077553	10	73	0.89407778	301.333333	28.33333333	0	0	0	1	34.0724638	1	0.49277013
2.2914	95234.3436	0.52078779	69.1785721	0.74463493	0	0.82916298	4.55635113	-0.6504943	4.23670562	-0.2935192	-6.9077553	11	76	0.44703889	300.222222	27.2222222	0	0	0	1	34.0724638	1	0.49277013
2.3553	97559.1233	0.3743734	32.6684502	0.50848394	0	0.85666811	4.58046884	-0.979834	3.4864404	-0.6743569	-6.9077553	12	75	1.78815556	300.777778	27.7777778	0	0	0	1	34.0724638	1	0.49277013
2.4048	99357.5355	0.34579905	30.6924627	0.55932465	0	0.87746674	4.59873488	-1.0590098	3.42404969	-0.5792389	-6.9077553	13	76	2.23519444	300.222222	27.2222222	0	0	0	1	34.0724638	1	0.49277013
2.4489	101378.505	0.28878887	23.0850481	0.54340366	0	0.89563894	4.61887095	-1.2386026	3.13922846	-0.6080643	-6.9077553	14	79	3.12927222	300.222222	27.2222222	0	0	0	1	34.0724638	1	0.49277013
2.4975	103440.106	0.27617926	17.6168326	0.46957737	0	0.91529023	4.63900243	-1.2830908	2.8689116	-0.7537949	-6.9077553	15	83	4.02335	299.666667	26.66666666	0	0	0	1	34.0724638	1	0.49277013
2.5497	105589.434	0.3296862	33.760296	0.6708109	0	0.93597571	4.65956778	-1.1065854	3.51931506	-0.3977784	-6.9077553	16	87	4.02335	299.111111	26.11111111	0	0	0	1	34.0724638	1	0.49277013
2.5974	107694.895	0.24804891	13.9490172	0.47691018	0	0.95451094	4.67931146	-1.390106	2.63548074	-0.7383325	-6.9077553	17	89	3.57631111	298.555556	25.55555555	0	0	0	1	34.0724638	1	0.49277013
2.6487	109888.082	0.28812398	22.7958599	0.5714365	0	0.97406895	4.69947151	-1.2408997	3.1266228	-0.5578535	-6.9077553	18	92	4.02335	298.555556	25.55555555	0	0	0	1	34.0724638	1	0.49277013
2.7009	112168.997	0.25754937	16.3084191	0.49165183	0	0.99358505	4.72001555	-1.3526686	2.7917428	-0.7079526	-6.9077553	19	88	4.91742778	298.555556	25.55555555	0	0	0	1	34.0724638	1	0.49277013
2.7513	114581.506	0.26877846	18.0526308	0.38073097	0	1.01207353	4.74129514	-1.3101542	2.89334682	-0.9630392	-6.9077553	20	87	4.02335	298.555556	25.55555555	0	0	0	1	34.0724638	1	0.49277013
2.9151	121222.1	0.57171136	96.4943338	1.11121067	0	1.06990412	4.79763265	-0.5573734	4.56949465	0.10634963	-6.9077553	21	82	4.91742778	299.666667	26.66666666	0	0	0	1	34.0724638	1	0.49277013
3.5361	136330.247	0.82978923	235.476721	2.44208135	0	1.26302442	4.91508757	-0.1853792	5.46161631	0.89326009	-6.9077553	22	74	5.36446667	301.333333	28.33333333	0	0	0	1	34.0724638	1	0.49277013
4.6557	155029.218	1.13119556	432.326467	3.69247937	0	1.53809228	5.04362006	0.12415872	6.06918333	1.30656893	-6.9077553	23	68	5.36446667	302.444444	29.44444444	0	0	0	1	34.0724638	1	0.49277013
0.0432	2984.94944	1.21678162	410.240442	2.46962608	0	-3.1419148	1.09391777	0.19703086	6.01674587	0.90447159	-6.9077553	0	60	3.57631111	304.666667	31.66666666	0	0	0	0	42.288408	1	-0.4111751
0.1422	7650.31945	0.96800863	254.982564	2.03958116	0	-1.9505208	2.03487811	-0.0314818	5.54119909	0.71323465	-6.9077553	1	57	4.91742778	304.111111	31.11111111	0	0	0	0	42.288408	1	-0.4111751
0.3231	16535.5481	1.01034379	284.596043	2.07851307	0	-1.1297934	2.80557297	0.01127993	5.6510743	0.73213377	-6.9077553	2	70	1.78815556	303	30	0	0	0	0	42.288408	1	-0.4111751
0.6462	30440.1016	1.04240906	298.330475	2.70357741	0	-0.4366462	3.41579372	0.0424933	5.6982052	0.99494567	-6.9077553	3	51	4.47038889	306.888889	33.88888888	0	0	1	0	42.288408	1	-0.4111751
1.008	47954.4079	1.0101184	279.878498	2.11036492	0	0.00796817	3.87027158	0.01105704	5.63435915	0.74733462	-6.9077553	4	50	3.57631111	306.888889	33.88888888	0	0	1	0	42.288408	1	-0.4111751
1.4166	65681.192	0.94850421	247.318161	1.92683122	0	0.34825963	4.18482784	-0.0518153	5.51067965	0.65639565	-6.9077553	5	52	3.57631111	306.888889	33.88888888	0	0	1	0	42.288408	1	-0.4111751
1.9611	84296.665	0.91188269	235.464773	2.66360287	0	0.67350554	4.43435417	-0.0911479	5.46156556	0.98005503	-6.9077553	6	43	4.02335	308	35	0	0	1	0	42.288408	1	-0.4111751
2.5254	106193.446	0.87195944	218.30632	2.09443102	0	0.92639947	4.66527181	-0.1358662	5.38590379	0.73975927	-6.9077553	7	48	3.12927222	307.444444	34.4444444	0	0	1	0	42.288408	1	-0.4111751
3.1131	128256.679	0.8307883	205.639692	1.84675764	0	1.13561901	4.85404136	-0.1841773	5.32613043	0.61397282	-6.9077553	8	50	2.23519444	306.888889	33.88888888	0	0	1	0	42.288408	1	-0.4111751
3.5739	144468.278	0.76159596	173.156891	1.44898343	0	1.27365744	4.97306688	-0.2710269	5.15420384	0.37155213	-6.9077553	9	57	0.44703889	304.666667	31.66666666	0	0	1	0	42.288408	1	-0.4111751
3.8637	157398.12	0.84362793	197.742321	1.4779701	0	1.35162527	5.05878475	-0.1688591	5.28696983	0.39134597	-6.9077553	10	73	0.89407778	301.333333	28.33333333	0	0	1	0	42.288408	1	-0.4111751
4.0023	163582.908	0.51138177	63.7120409	0.8108739	0	1.3868692	5.09732606	-0.6686853	4.15438927	-0.2084102	-6.9077553	11	76	0.44703889	300.222222	27.2222222	0	0	1	0	42.288408	1	-0.4111751
4.0977	167574.519	0.42073779	42.9912478	0.69773267	0	1.41042584	5.12143411	-0.8633715	3.76101982	-0.3584871	-6.9077553	12	75	1.78815556	300.777778	27.7777778	0	0	1	0	42.288408	1	-0.4111751
4.1652	170175.153	0.35500964	33.1036914	0.5901501	0	1.42676429	5.13683409	-1.0327975	3.49967501	-0.5256853	-6.9077553	13	76	2.23519444	300.222222	27.2222222	0	0	1	0	42.288408	1	-0.4111751
4.2228	172719.251	0.27049181	16.1731987	0.4339397	0	1.44049842	5.15167324	-1.3038233	2.7834173	-0.8325479	-6.9077553	14	79	3.12927222	300.222222	27.2222222	0	0	1	0	42.288408	1	-0.4111751
4.2813	175298.789	0.28089151	24.5730394	0.55594987	0	1.4542567	5.16649759	-1.266233	3.20169058	-0.58528	-6.9077553	15	83	4.02335	299.666667	26.66666666	0	0	1	0	42.288408	1	-0.4111751
4.3416	177974.5	0.27707769	21.3242424	0.48044051	0	1.46824294	5.1816459	-1.2798547	3.05989146	-0.7309726	-6.9077553	16	87	4.02335	299.111111	26.11111111	0	0	1	0	42.288408	1	-0.4111751
4.3974	180255.407	0.25928359	14.9735836	0.45332703	0	1.48101346	5.19438032	-1.3459835	2.70635434	-0.788938	-6.9077553	17	89	3.57631111	298.555556	25.55555555	0	0	1	0	42.288408	1	-0.4111751
4.4559	182931.098	0.24575383	13.6292499	0.42383842	0	1.49422906	5.20911504	-1.3993641	2.61229158	-0.8560464	-6.9077553	18	92	4.02335	298.555556	25.55555555	0	0	1	0	42.288408	1	-0.4111751
4.5099	185212.014	0.2670181	16.470222	0.53269084	0	1.50627498	5.22150659	-1.3167008	2.80161473	-0.6279386	-6.9077553	19	88	4.91742778	298.555556	25.55555555	0	0	1	0	42.288408	1	-0.4111751
4.572	188019.294	0.27259782	19.5598445	0.47494855	0	1.51995075	5.23654991	-1.296096	2.97352984	-0.7424455	-6.9077553	20	87	4.02335	298.555556	25.55555555	0	0	1	0	42.288408	1	-0.4111751
4.8087	194385.433	0.52333086	94.8435124	1.20833712	0	1.57042678	5.2698481	-0.6456324	4.55223884	0.19007238	-6.9077553	21	82	4.91742778	299.666667	26.66666666	0	0	1	0	42.288408	1	-0.4111751
5.4783	209132.969	0.61229689	128.935788	1.94481519	0	1.70079483	5.34297505	-0.4889061	4.85932227	0.66568101	-6.9077553	22	74	5.36446667	301.333333	28.33333333	0	0	1	0	42.288408	1	-0.4111751
6.1659	224167.681	0.6641634	151.607146	2.0163702	0	1.81903411	5.41239881	-0.4077226	5.0212992	0.70179478	-6.9077553	23	68	5.36446667	302.444444	29.44444444	0	0	1	0	42.288408	1	-0.4111751
0.0432	0	0.79738114	182.947183	4.43872265	0	-3.1419148	-6.9077553	-0.2251692	5.20920296	1.49059191	-6.9077553	0	60	3.57631111	304.666667	31.66666666	0	0	0	0	18.9135952	1	-0.0428739
0.2106	47.0606856	0.5251222	74.1334005	2.84992308	0	-1.5577947	-3.0352908	-0.6422218	4.30587967	1.04764283	-6.9077553	1	57	4.91742778	304.111111	31.11111111	0	0	1	0	18.9135952	1	-0.0428739
0.3312	310.243378	0.41452334	48.948694	1.32401059	0	-1.1050329	-1.1671801	-0.8782165	3.89079312	0.28142045	-6.9077553	2	70	1.78815556	303	30	0	0	1	0	18.9135952	1	-0.0428739
0.5787	1718.40296	0.61716059	136.837287	2.60702526	0	-0.5469711	0.54197712	-0.481007	4.91879984	0.95859333	-6.9077553	3	51	4.47038889	306.888889	33.88888888	0	0	1	0	18.9135952	1	-0.0428739
0.855	7858.50227	0.78613649	198.954766	2.15953119	0	-0.1566538	2.06172328	-0.2393536	5.29308252	0.77035411	-6.9077553	4	50	3.57631111	306.888889	33.88888888	0	0	1	0	18.9135952	1	-0.0428739
1.1448	14887.9502	0.77220377	175.190314	1.94785797	0	0.13522995	2.70061934	-0.2572127	5.1658786	0.66724354	-6.9077553	5	52	3.57631111	306.888889	33.88888888	0	0	1	0	18.9135952	1	-0.0428739
1.5048	23400.25	0.76782097	175.436738	2.56160339	0	0.40866	3.15278944	-0.2628971	5.16728421	0.94102369	-6.9077553	6	43	4.02335	308	35	0	0	1	0	18.9135952	1	-0.0428739
1.8189	36871.5394	0.89132052	238.599122	1.85285141	0	0.59823192	3.60746708	-0.1139299	5.47478902	0.61726532	-6.9077553	7	48	3.12927222	307.444444	34.4444444	0	0	1	0	18.9135952	1	-0.0428739
2.1267	47765.1521	0.80261962	193.029742	1.55653515	0	0.75457148	3.86631727	-0.2186292	5.26284946	0.44310454	-6.9077553	8	50	2.23519444	306.888889	33.88888888	0	0	1	0	18.9135952	1	-0.0428739
2.448	59889.6304	0.81711679	209.201527	1.67728319	0	0.89527136	4.09252007	-0.2007502	5.34330281	0.51777136	-6.9077553	9	57	0.44703889	304.666667	31.66666666	0	0	1	0	18.9135952	1	-0.0428739
2.6694	70671.4533	0.88175617	213.606612	1.5732268	0	0.98185373	4.25805587	-0.1247063	5.36414074	0.45376423	-6.9077553	10	73	0.89407778	301.333333	28.33333333	0	0	1	0	18.9135952	1	-0.0428739
2.8188	77426.5728	0.59145015	89.393237	1.00780947	0	1.03631126	4.34934296	-0.5234885	4.49305622	0.00877089	-6.9077553	11	76	0.44703889	300.222222	27.2222222	0	0	1	0	18.9135952	1	-0.0428739

2.9223	81944.5503	0.4227959	42.5798786	0.67184462	0	1.07237098	4.40605501	-0.8585033	3.75140529	-0.3962409	-6.9077553	12	75	1.78815556	300.777778	27.7777778	0	0	1	0	18.9135952	1	-0.0428739
3.0087	85804.5584	0.36208129	29.8460108	0.56549378	0	1.10150809	4.45208379	-1.0131285	3.3960847	-0.5682892	-6.9077553	13	76	2.23519444	300.222222	27.2222222	0	0	1	0	18.9135952	1	-0.0428739
3.0735	88918.8899	0.28586117	17.9855821	0.46491464	0	1.12281698	4.48773585	-1.2487569	2.88962604	-0.7637528	-6.9077553	14	79	3.12927222	300.222222	27.2222222	0	0	1	0	18.9135952	1	-0.0428739
3.141	91989.3458	0.26727442	16.1101953	0.44603831	0	1.14454122	4.52168363	-1.3157449	2.77951439	-0.805111	-6.9077553	15	83	4.02335	299.666667	26.66666666	0	0	1	0	18.9135952	1	-0.0428739
3.204	94884.3569	0.26219036	14.9371896	0.40569507	0	1.16440003	4.55266939	-1.3348777	2.70392099	-0.8996916	-6.9077553	16	87	4.02335	299.111111	26.11111111	0	0	1	0	18.9135952	1	-0.0428739
3.2661	97691.6408	0.23633194	11.8149228	0.35595816	0	1.18359661	4.58182623	-1.4382955	2.46944801	-1.0301367	-6.9077553	17	89	3.57631111	298.555556	25.55555555	0	0	1	0	18.9135952	1	-0.0428739
3.3309	100630.512	0.26362281	24.5867089	0.45555475	0	1.20324254	4.61146545	-1.3294498	3.20224668	-0.7840467	-6.9077553	18	92	4.02335	298.555556	25.55555555	0	0	1	0	18.9135952	1	-0.0428739
3.4002	103744.848	0.23543615	12.3723525	0.39530853	0	1.22383425	4.64194414	-1.4420771	2.51554517	-0.9255623	-6.9077553	19	88	4.91742778	298.555556	25.55555555	0	0	1	0	18.9135952	1	-0.0428739
3.474	107253.944	0.31365841	26.7154481	0.46661247	0	1.24530667	4.67520865	-1.1562676	3.28527941	-0.7601154	-6.9077553	20	87	4.02335	298.555556	25.55555555	0	0	1	0	18.9135952	1	-0.0428739
3.7521	118766.753	0.64878203	127.596784	1.32535452	0	1.32231568	4.77716993	-0.4311183	4.848883	0.28243421	-6.9077553	21	82	4.91742778	299.666667	26.66666666	0	0	1	0	18.9135952	1	-0.0428739
4.4694	141358.816	0.76370266	173.937147	2.37344556	0	1.49725417	4.95130853	-0.2682682	5.15869976	0.86476396	-6.9077553	22	74	5.36446667	301.333333	28.33333333	0	0	1	0	18.9135952	1	-0.0428739
5.2875	171507	0.8523199	217.157625	2.2250097	0	1.66534554	5.14462991	-0.1586208	5.38062808	0.80021061	-6.9077553	23	68	5.36446667	302.444444	29.44444444	0	0	1	0	18.9135952	1	-0.0428739
0.009	316.282578	0.82984813	188.726576	1.31498517	0	-4.7105307	-1.1479625	-0.1853083	5.24030458	0.27458556	-6.9077553	0	97	3.12927222	293	20	0	0	0	0	30.519848	1	0.21141735
0.0126	491.737726	0.4261443	42.7233353	0.70759094	0	-4.3740585	-0.7077782	-0.8506334	3.75476867	-0.3444769	-6.9077553	1	97	2.68223333	293	20	0	0	0	0	30.519848	1	0.21141735
0.0216	886.511885	0.46616006	50.717157	0.76337647	0	-3.835062	-0.1193334	-0.7610833	3.92628397	-0.2686948	-6.9077553	2	93	4.47038889	293.555556	20.55555555	0	0	0	0	30.519848	1	0.21141735
0.0342	1237.42206	0.51583569	63.9463457	0.84443351	0	-3.3755296	0.21383804	-0.6600303	4.15806002	-0.1679058	-6.9077553	3	92	2.68223333	293.555556	20.55555555	0	0	0	0	30.519848	1	0.21141735
0.0387	1325.14958	0.4071938	41.6151947	0.86753769	0	-3.2519157	0.28227969	-0.8960132	3.72848939	-0.1409443	-6.9077553	4	91	2.68223333	294.111111	21.11111111	0	0	0	0	30.519848	1	0.21141735
0.0612	1632.19593	0.32631962	32.505127	1.00993568	0	-2.7936081	0.49053879	-1.1168182	3.48142859	0.01087632	-6.9077553	5	91	2.68223333	294.666667	21.66666666	0	0	0	0	30.519848	1	0.21141735
0.1098	1895.37879	0.34194501	33.0970459	1.17512731	0	-2.2090947	0.63994617	-1.0701852	3.49947424	0.1622271	-6.9077553	6	85	3.57631111	296.333333	23.33333333	0	0	0	0	30.519848	1	0.21141735
0.2313	2796.6995	0.52714934	84.4199074	2.30825263	0	-1.4640397	1.02879747	-0.6383762	4.43581509	0.83692394	-6.9077553	7	71	5.36446667	298.555556	25.55555555	0	0	0	0	30.519848	1	0.21141735
0.3591	3842.96349	0.64332731	117.819556	2.34694274	0	-1.0241544	1.34650399	-0.4395484	4.76916276	0.85353952	-6.9077553	8	67	5.81150556	299.666667	26.66666666	0	0	0	0	30.519848	1	0.21141735
0.5247	7228.45809	0.74357564	160.231806	2.28749333	0	-0.6449286	1.97816408	-0.2949408	5.0766278	0.82789367	-6.9077553	9	55	6.25854444	301.888889	28.88888888	0	0	0	1	30.519848	1	0.21141735
0.8154	18101.5031	0.83975739	212.801108	2.84307276	0	-0.2040765	2.89605022	-0.1734521	5.36036266	1.04523709	-6.9077553	10	50	5.81150556	303.555556	30.55555555	0	0	0	1	30.519848	1	0.21141735
1.1277	27994.6415	0.72450555	148.164839	2.1163717	0	0.12018016	3.33204884	-0.3208866	4.99833218	0.75017556	-6.9077553	11	45	5.36446667	304.111111	31.11111111	0	0	0	1	30.519848	1	0.21141735
1.3977	37864.8077	0.67976028	132.288021	1.52006368	0	0.33482803	3.63404853	-0.384545	4.88498908	0.41940988	-6.9077553	12	46	3.57631111	304.111111	31.11111111	0	0	0	1	30.519848	1	0.21141735
1.6182	48462.6987	0.72232243	151.723358	1.40070228	0	0.48131442	3.88081504	-0.3239002	5.02206544	0.33768741	-6.9077553	13	44	4.02335	304.666667	31.66666666	0	0	0	1	30.519848	1	0.21141735
1.8072	59513.4319	0.71830386	145.951497	1.21112736	0	0.59177869	4.08621884	-0.3294714	4.98328121	0.19237697	-6.9077553	14	49	3.12927222	303	30	0	0	0	1	30.519848	1	0.21141735
1.935	68899.6432	0.65157377	122.146787	0.91966011	0	0.66010733	4.23266551	-0.4268311	4.80523168	-0.0826644	-6.9077553	15	53	3.12927222	301.333333	28.33333333	0	0	0	1	30.519848	1	0.21141735
2.0502	79690.6649	0.64354122	116.849106	0.80399895	0	0.71793735	4.378165	-0.4392165	4.76089197	-0.2169143	-6.9077553	16	58	1.78815556	299.111111	26.11111111	0	0	0	1	30.519848	1	0.21141735
2.1492	88437.9178	0.67013737	133.708185	0.96305098	0	0.76509568	4.48231212	-0.3987814	4.89566718	-0.0366111	-6.9077553	17	51	2.68223333	298.555556	25.55555555	0	0	0	1	30.519848	1	0.21141735
2.2923	100548.004	0.62843193	116.901054	0.92908329	0	0.82955568	4.61064521	-0.4629376	4.76133644	-0.0724811	-6.9077553	18	53	2.23519444	297.444444	24.44444444	0	0	0	1	30.519848	1	0.21141735
2.394	110977.067	0.78160743	168.58191	1.1010834	0	0.87296561	4.70933258	-0.2451241	5.12742768	0.09720239	-6.9077553	19	62	2.68223333	296.333333	23.33333333	0	0	0	1	30.519848	1	0.21141735
2.4714	121236.477	0.69997765	132.201467	0.9545234	0	0.90478479	4.79775124	-0.3552793	4.88433459	-0.045496	-6.9077553	20	73	3.12927222	295.777778	22.7777778	0	0	0	1	30.519848	1	0.21141735
2.5083	125332	0.61440905	101.828065	1.01725825	0	0.91960523	4.83097419	-0.4854681	4.62329558	0.01809357	-6.9077553	21	80	2.23519444	295.222222	22.2222222	0	0	0	1	30.519848	1	0.21141735
2.5092	125375.863	0.28633571	17.1338959	0.66315514	0	0.91996398	4.8313241	-1.247104	2.84111708	-0.4092395	-6.9077553	22	84	3.12927222	295.222222	22.2222222	0	0	0	1	30.519848	1	0.21141735
2.5128	125419.726	0.20097604	8.02546597	0.22542451	0	0.92139767	4.83167389	-1.5996062	2.08274433	-1.4853437	-6.9077553	23	88	2.23519444	294.666667	21.66666666	0	0	0	1	30.519848	1	0.21141735
0.0135	686.812422	1.01415212	283.346906	1.91286005	0	-4.3050656	-0.3742391	0.01503847	5.64667549	0.64912217	-6.9077553	0	97	3.12927222	293	20	0	0	0	0	34.0724638	1	0.49277013
0.0243	1081.58645	0.4754773	54.1421509	0.68241517	0	-3.7172789	0.07935304	-0.7413352	3.99163148	-0.3806527	-6.9077553	1	97	2.68223333	293	20	0	0	0	0	34.0724638	1	0.49277013
0.0387	1564.08801	0.38323778	35.6231461	0.68629295	0	-3.2519157	0.44794206	-0.9564937	3.57302367	-0.3749947	-6.9077553	2	93	4.47038889	293.555556	20.55555555	0	0	0	0	34.0724638	1	0.49277013
0.0468	1914.99822	0.40077676	38.4067145	0.63055076	0	-3.0618721	0.65023875	-0.9118587	3.64825834	-0.459577	-6.9077553	3	92	2.68223333	293.555556	20.55555555	0	0	0	0	34.0724638	1	0.49277013
0.0657	2616.8189	0.49633185	60.677374	0.83429255	0	-2.7226564	0.96234149	-0.6984978	4.10558736	-0.1799733	-6.9077553	4	91	2.68223333	294.111111	21.11111111	0	0	0	0	34.0724638	1	0.49277013
0.1017	3992.47669	0.67238717	119.764392	1.34224529	0	-2.285728	1.3846622	-0.3954348	4.78553476	0.29508855	-6.9077553	5	91	2.68223333	294.666667	21.66666666	0	0	0	0	34.0724638	1	0.49277013
0.1692	6900.391	0.78796351	170.230833	1.43298604	0	-1.7766738	1.93172298	-0.2370352	5.13716123	0.36045801	-6.9077553	6	85	3.57631111	296.333333	23.33333333	0	0	0	0	34.0724638	1	0.49277013
0.2952	11820.0548	1.03676529	322.890653	2.51871403	0	-1.2201022	2.46988225	0.03706964	5.77731683	0.92414542	-6.9077553	7	71	5.36446667	298.555556	25.55555555	0	0	0	0	34.0724638	1	0.49277013
0.7497	22535.9237	1.36152646	583.360891	4.92888691	0	-0.2880822	3.11515502	0.30934067	6.36880773	1.59531605	-6.9077553	8	67	5.81150556	299.666667	26.66666666	0	0	0	1	34.0724638	1	0.49277013
1.6083	43747.6075	1.47200994	682.941038	5.57158285	0	0.47517772	3.77845978	0.38730789	6.52640999	1.71785865	-6.9077553	9	55	6.25854444	301.888889	28.88888888	0	0	0	1	34.0724638	1	0.49277013
2.3427	71753.6309	1.18592924	413.433019	3.54457511	1	0.85130411	4.2732524	0.1713695	6.02449794	1.26570038	0.0009995	10	50	5.81150556	303.555556	30.55555555	0	0	0	1	34.0724638	1	0.49277013
2.8638	88196.8962	0.93651606	246.285325	1.89121364	1	1.05214941	4.47958311	-0.0645214	5.50649478	0.63774738	0.0009995	11	45	5.36446667	304.111111	31.11111111	0	0	0	1	34.0724638	1	0.49277013
3.4794	111782.427	0.99312774	273.502421	2.51264748	1	1.24685987	4.71656331	-0.0058896	5.61131413	0.92173488	0.0009995	12	46	3.57631111	304.111111	31.11111111	0	0	0	1	34.0724638	1	0.49277013
4.2282	134176.945	0.94963044	254.930138	2.91455313	1	1.44177637	4.89916687	-0.0506299	5.54099346	1.07005955	0.0009995	13	44	4.02335	304.666667	31.66666666	0	0	0	1	34.0724638	1	0.49277013
4.8042	149076.175	0.81297741	189.505762	1.98217201	1	1.56949054	5.00446413	-0.2058227	5.24442471	0.68469759	0.0009995	14	49	3.12927222	303	30	0	0	0	1	34.0724638	1	0.49277013
5.2533	162309.751	0.73333202	159.766549	1.37419615	1	1.65885645	5.08951271	-0.308794	5.07371994	0.31859637	0.0009995	15	53	3.12927222	301.333333	28.33333333	0	0	0	1	34.0724638	1	0.49277013
5.553	173193.179	0.74688753	167.651933	1.26937573	1	1.71433832	5.15441339	-0.2905027	5.12189597	0.23931271	0.0009995	16	58	1.78815556	299.111111	26.11111111	0	0	0	1	34.0724638	1	0.49277013
5.8977	185314.56	0.71851558	159.884067	1.23799265	1	1.77456244	5.2220601	-0.3291771	5.07445523	0.21429867	0.0009995	17	51	2.68223333	298.555556	25.55555555	0	0	0	1	34.0724638	1	0.49277013
6.2802	195903.014	0.63258365	126.040368	1.24548311	1	1.83740183	5.27762482	-0.4563632	4.83661017	0.22032607	0.0009995	18	53	2.23519444	297.444444	24.44444444	0	0	0	1	34.0724638	1	0.49277013
6.5403	205585.22	0.77309874	170.758191	1.32765277	1	1.87798304	5.32586551	-0.2560558	5.14025432	0.28416547	0.0009995	19	62	2.68223333	296.333333	23.33333333	0	0	0	1	34.0724638	1	0.49277013
6.7194	213184.654	0.76622638	160.617129	1.28844785	1	1.90499886	5.3621634	-0.2649734	5.07902968	0.25421411	0.0009995	20	73	3.12927222	295.777778	22.7777778	0	0	0	1	34.0724638	1	0.49277013
6.84	218012.407	0.60985041	98.4827352	1.09657925	1	1.92278773	5.38455656	-0.4929032	4.58989141	0.09310708	0.0009995	21	80	2.23519444	295.222222	22.2222222	0	0	0	1	34.0724638	1	0.49277013
6.8868	220117.835	0.23098623	11.3238068	0.45631475	1	1.92960654	5.39416756	-1.4610773	2.42699561	-0.7823834	0.0009995	22	84	3.12927222	295.222222	22.2222222	0	0	0	1	34.0724638	1	0.49277013
6.9318	222047.852	0.19052868	7.45860352	0.31032431	1	1.93611952	5.40289741	-1.6527177	2.00950226	-1.1669201	0.0009995	23	88	2.23519444	294.666667	21.66666666	0	0	0	1	34.0724638	1	0.49277013
0.0306	1875.94935	1.09749707	329.05522	2.27513423	0	-3.4867553	0.62964778	0.09394295	5.79622862	0.82247849	-6.9077553	0	97	3.12927222	293	20	0	0	0	0	42.288408	1	-0.4111751
0.0693	3762.18477	0.60384262	92.7145372	1.11715689	0	-2.6693104	1.32526561	-0.502787	4.52953607	0.1116817	-6.9077553	1	97	2.68223333	293	20	0	0	0	0	42.288408	1	-0.4111751

0.117	5955.37447	0.55705751	77.534152	1.08203586	0	-2.1455813	1.78446199	-0.5832933	4.35073141	0.07976808	-6.9077553	2	93	4.47038889	293.555556	20.55555555	0	0	0	0	42.288408	1	-0.4111751
0.1647	8280.15437	0.5661552	79.0720537	0.98403679	0	-1.8036296	2.11398238	-0.5671223	4.37037216	-0.0150763	-6.9077553	3	92	2.68223333	293.555556	20.55555555	0	0	0	0	42.288408	1	-0.4111751
0.2313	11453.1507	0.66059994	117.556918	1.25630838	0	-1.4640397	2.43835217	-0.4130942	4.76693113	0.22897323	-6.9077553	4	91	2.68223333	294.111111	21.11111111	0	0	0	0	42.288408	1	-0.4111751
0.414	18306.8096	0.79485453	188.540025	1.98987508	0	-0.8818893	2.90732772	-0.2283389	5.23931562	0.68857428	-6.9077553	5	91	2.68223333	294.666667	21.66666666	0	0	0	0	42.288408	1	-0.4111751
0.63	27377.1892	0.85826995	204.263851	1.8944973	0	-0.4620355	3.30974668	-0.1516722	5.31941744	0.63948123	-6.9077553	6	85	3.57631111	296.333333	23.33333333	0	0	0	0	42.288408	1	-0.4111751
0.9594	38971.9953	0.84919489	192.727694	2.06590863	0	-0.0414472	3.66286898	-0.1622897	5.26128347	0.72605408	-6.9077553	7	71	5.36446667	298.555556	25.55555555	0	0	1	0	42.288408	1	-0.4111751
1.3464	54161.899	0.89077674	215.310683	2.18384215	0	0.29743436	3.99199615	-0.1145395	5.37208667	0.78154358	-6.9077553	8	67	5.81150556	299.666667	26.66666666	0	0	1	0	42.288408	1	-0.4111751
1.9404	75075.2247	0.9092742	233.732765	2.74290993	0	0.66289414	4.31850393	-0.0940094	5.45418271	1.00938389	-6.9077553	9	55	6.25854444	301.888889	28.88888888	0	0	1	0	42.288408	1	-0.4111751
2.556	100234.605	0.93175145	245.444399	2.26785617	0	0.93844354	4.60752347	-0.0696165	5.50307451	0.81927582	-6.9077553	10	50	5.81150556	303.555556	30.55555555	0	0	1	0	42.288408	1	-0.4111751
3.1941	130146.796	0.96788208	267.382228	2.02533243	0	1.16130536	4.8686707	-0.0316124	5.58868294	0.70622747	-6.9077553	11	45	5.36446667	304.111111	31.11111111	0	0	1	0	42.288408	1	-0.4111751
3.9573	171204.024	1.05426133	313.531324	2.2773472	0	1.37556197	5.14286181	0.05378845	5.74790246	0.82345027	-6.9077553	12	46	3.57631111	304.111111	31.11111111	0	0	1	0	42.288408	1	-0.4111751
4.6494	201077.569	0.97867754	283.697842	2.31620148	0	1.53673818	5.30369572	-0.0205318	5.64791326	0.8403602	-6.9077553	13	44	4.02335	304.666667	31.66666666	0	0	1	0	42.288408	1	-0.4111751
5.1309	220324.779	0.86165013	208.991048	1.78331504	0	1.63528108	5.39510726	-0.1477461	5.34229621	0.57903461	-6.9077553	14	49	3.12927222	303	30	0	0	1	0	42.288408	1	-0.4111751
5.5134	235159.864	0.78233571	171.204393	1.30909065	0	1.70718149	5.46026981	-0.2441939	5.14286397	0.27009633	-6.9077553	15	53	3.12927222	301.333333	28.33333333	0	0	1	0	42.288408	1	-0.4111751
5.8401	248413.412	0.81932383	192.162362	1.36456551	0	1.76474792	5.51509837	-0.1980561	5.25834586	0.31156864	-6.9077553	16	58	1.78815556	299.111111	26.11111111	0	0	1	0	42.288408	1	-0.4111751
6.1299	259889.986	0.78375737	182.933157	1.35656511	0	1.81317844	5.56026226	-0.2423807	5.20912629	0.30569273	-6.9077553	17	51	2.68223333	298.555556	25.55555555	0	0	1	0	42.288408	1	-0.4111751
6.4323	269018.447	0.65172513	136.833264	1.06835284	0	1.86133217	5.59478367	-0.4265992	4.91877044	0.06705364	-6.9077553	18	53	2.23519444	297.444444	24.44444444	0	0	1	0	42.288408	1	-0.4111751
6.6258	276345.076	0.77529567	172.264281	1.22479049	0	1.89097112	5.62165398	-0.2532218	5.14903562	0.20358594	-6.9077553	19	62	2.68223333	296.333333	23.33333333	0	0	1	0	42.288408	1	-0.4111751
6.7491	280959.387	0.75383196	153.100707	1.28245021	0	1.90940916	5.63821369	-0.2812601	5.03110245	0.24955193	-6.9077553	20	73	3.12927222	295.777778	22.7777778	0	0	1	0	42.288408	1	-0.4111751
6.8157	283284.17	0.55968151	82.1049013	0.90140245	0	1.91922877	5.64645406	-0.5786023	4.40800989	-0.1026947	-6.9077553	21	80	2.23519444	295.222222	22.2222222	0	0	1	0	42.288408	1	-0.4111751
6.8337	283854.387	0.27532079	27.7933685	0.57445741	0	1.92186625	5.64846491	-1.2861928	3.32483343	-0.5525901	-6.9077553	22	84	3.12927222	295.222222	22.2222222	0	0	1	0	42.288408	1	-0.4111751
6.8526	284600.087	0.18971591	7.85159011	0.42370174	0	1.92462814	5.6510885	-1.6569704	2.06084343	-0.8563681	-6.9077553	23	88	2.23519444	294.666667	21.66666666	0	0	1	0	42.288408	1	-0.4111751
0.0009	0	0	0	0	0	-7.0131158	-6.9077553	-6.9077553	-6.9077553	-6.9077553	-6.9077553	0	97	3.12927222	293	20	0	0	0	0	18.9135952	1	-0.0428739
0.0009	0	0	0	0	0	-7.0131158	-6.9077553	-6.9077553	-6.9077553	-6.9077553	-6.9077553	1	97	2.68223333	293	20	0	0	0	0	18.9135952	1	-0.0428739
0.0009	0	0	0	0	0	-7.0131158	-6.9077553	-6.9077553	-6.9077553	-6.9077553	-6.9077553	2	93	4.47038889	293.555556	20.55555555	0	0	0	0	18.9135952	1	-0.0428739
0.0009	0	0	0	0	0	-7.0131158	-6.9077553	-6.9077553	-6.9077553	-6.9077553	-6.9077553	3	92	2.68223333	293.555556	20.55555555	0	0	0	0	18.9135952	1	-0.0428739
0.0009	0	0	0	0	0	-7.0131158	-6.9077553	-6.9077553	-6.9077553	-6.9077553	-6.9077553	4	91	2.68223333	294.111111	21.11111111	0	0	0	0	18.9135952	1	-0.0428739
0.0009	0	0	0	0	0	-7.0131158	-6.9077553	-6.9077553	-6.9077553	-6.9077553	-6.9077553	5	91	2.68223333	294.666667	21.66666666	0	0	0	0	18.9135952	1	-0.0428739
0.0009	0	0	0	0	0	-7.0131158	-6.9077553	-6.9077553	-6.9077553	-6.9077553	-6.9077553	6	85	3.57631111	296.333333	23.33333333	0	0	0	0	18.9135952	1	-0.0428739
0.036	0	0.65597627	122.202173	3.22482241	0	-3.3242363	-6.9077553	-0.4201074	4.80568502	1.17118793	-6.9077553	7	71	5.36446667	298.555556	25.55555555	0	0	0	0	18.9135952	1	-0.0428739
0.2016	3595.85499	0.83708771	232.80645	2.91919211	0	-1.6014697	1.28005985	-0.1766325	5.45021172	1.0716494	-6.9077553	8	67	5.81150556	299.666667	26.66666666	0	0	0	0	18.9135952	1	-0.0428739
0.459	12157.9244	0.89518204	266.901879	2.84533008	0	-0.7787051	2.49806342	-0.1096117	5.58688484	1.04603048	-6.9077553	9	55	6.25854444	301.888889	28.88888888	0	0	1	0	18.9135952	1	-0.0428739
0.6606	16695.6192	0.77281734	201.840995	1.99571991	0	-0.4146068	2.81520626	-0.2564194	5.30748519	0.69150579	-6.9077553	10	50	5.81150556	303.555556	30.55555555	0	0	1	0	18.9135952	1	-0.0428739
0.9045	25048.0683	0.8138189	205.480699	1.85466358	0	-0.100373	3.22083663	-0.2047894	5.32535698	0.61824236	-6.9077553	11	45	5.36446667	304.111111	31.11111111	0	0	1	0	18.9135952	1	-0.0428739
1.1979	34385.3497	0.83513272	210.535612	2.17712627	0	0.18057002	3.53765967	-0.1789679	5.34965956	0.778465	-6.9077553	12	46	3.57631111	304.111111	31.11111111	0	0	1	0	18.9135952	1	-0.0428739
1.5237	48328.4499	0.92145332	252.706967	2.01519	0	0.42114159	3.8780411	-0.0807185	5.53223454	0.70120959	-6.9077553	13	44	4.02335	304.666667	31.66666666	0	0	1	0	18.9135952	1	-0.0428739
1.7829	59091.8145	0.84857861	209.328817	1.60847346	0	0.57824125	4.07910934	-0.1630148	5.34391108	0.47590708	-6.9077553	14	49	3.12927222	303	30	0	0	1	0	18.9135952	1	-0.0428739
2.0934	71955.5424	0.85741197	222.212772	1.69892401	0	0.73878954	4.27606236	-0.1526711	5.40363985	0.53058355	-6.9077553	15	53	3.12927222	301.333333	28.33333333	0	0	1	0	18.9135952	1	-0.0428739
2.3751	84403.1688	0.88035354	221.004268	1.62864963	0	0.86503954	4.43561679	-0.1262964	5.39818654	0.48836504	-6.9077553	16	58	1.78815556	299.111111	26.11111111	0	0	1	0	18.9135952	1	-0.0428739
2.6442	95806.4597	0.80447245	190.987772	1.49160409	0	0.97236856	4.56234055	-0.2163263	5.25221464	0.40052231	-6.9077553	17	51	2.68223333	298.555556	25.55555555	0	0	1	0	18.9135952	1	-0.0428739
2.9979	108182.366	0.76596947	182.560194	1.6030202	0	1.09791204	4.68382762	-0.2653083	5.20708543	0.4725131	-6.9077553	18	53	2.23519444	297.444444	24.44444444	0	0	1	0	18.9135952	1	-0.0428739
3.1977	117257.482	0.83939045	203.105856	1.48108419	0	1.1624318	4.76438075	-0.1738887	5.31373222	0.39344933	-6.9077553	19	62	2.68223333	296.333333	23.33333333	0	0	1	0	18.9135952	1	-0.0428739
3.3903	126976.267	0.91544264	231.437537	1.81946172	0	1.22091841	4.84400807	-0.0872558	5.44431434	0.59909016	-6.9077553	20	73	3.12927222	295.777778	22.7777778	0	0	1	0	18.9135952	1	-0.0428739
3.5082	132125.582	0.71923968	137.448503	1.29426016	0	1.25510309	4.88376041	-0.3281712	4.9232566	0.25871157	-6.9077553	21	80	2.23519444	295.222222	22.2222222	0	0	1	0	18.9135952	1	-0.0428739
3.5451	133660.802	0.26500055	15.644104	0.50556295	0	1.26556637	4.89531274	-1.3242569	2.75015802	-0.6801067	-6.9077553	22	84	3.12927222	295.222222	22.2222222	0	0	1	0	18.9135952	1	-0.0428739
3.5784	135152.165	0.22503734	10.8658472	0.38984064	0	1.27491577	4.90640869	-1.4870551	2.38571661	-0.9394554	-6.9077553	23	88	2.23519444	294.666667	21.66666666	0	0	1	0	18.9135952	1	-0.0428739
0.0009	0	0	0	0	0	-7.0131158	-6.9077553	-6.9077553	-6.9077553	-6.9077553	-6.9077553	0	82	2.68223333	296.333333	23.33333333	0	0	0	0	30.519848	1	0.21141735
0.0027	43.8637829	0.4835211	54.3825226	0.81935394	0	-5.9145035	-3.1041244	-0.7245943	3.99606121	-0.1980194	-6.9077553	1	86	2.23519444	295.777778	22.7777778	0	0	0	0	30.519848	1	0.21141735
0.0063	219.318913	0.3033171	19.8788147	0.37409225	0	-5.0672056	-1.5126792	-1.189685	2.98970488	-0.9805833	-6.9077553	2	86	2.68223333	296.333333	23.33333333	0	0	0	0	30.519848	1	0.21141735
0.0072	263.182695	0.23665272	11.3222923	0.26201463	0	-4.9336743	-1.3311144	-1.4369448	2.42686187	-1.3355456	-6.9077553	3	86	1.34111667	296.333333	23.33333333	0	0	0	0	30.519848	1	0.21141735
0.0081	307.046481	0.17839873	6.12565136	0.36969408	0	-4.8158912	-1.1775046	-1.7181444	1.81264833	-0.9923781	-6.9077553	4	86	2.23519444	296.333333	23.33333333	0	0	0	0	30.519848	1	0.21141735
0.0117	482.501588	0.23112667	10.9676552	0.27796924	0	-4.4481664	-0.7267007	-1.4604721	2.39504168	-1.2766538	-6.9077553	5	87	1.34111667	296.333333	23.33333333	0	0	0	0	30.519848	1	0.21141735
0.0153	614.092962	0.23366106	12.044158	0.41754198	0	-4.1799025	-0.4859819	-1.4496131	2.48866275	-0.8709781	-6.9077553	6	88	0.89407778	295.777778	22.7777778	0	0	0	0	30.519848	1	0.21141735
0.0216	921.139558	0.28322053	16.9160494	0.43654357	0	-3.835062	-0.0810587	-1.2580048	2.82832195	-0.826579	-6.9077553	7	88	2.23519444	295.777778	22.7777778	0	0	0	0	30.519848	1	0.21141735
0.027	1008.867	0.26764766	15.0273259	0.42045061	0	-3.6119184	0.00981864	-1.3143546	2.70993682	-0.8640527	-6.9077553	8	88	2.68223333	295.777778	22.7777778	0	0	0	0	30.519848	1	0.21141735
0.0387	1359.77726	0.35478581	37.2085055	0.78256226	0	-3.2519157	0.30805605	-1.0334264	3.61656425	-0.2439048	-6.9077553	9	86	2.68223333	296.333333	23.33333333	0	0	0	0	30.519848	1	0.21141735
0.0774	1710.68751	0.32797232	31.5872036	0.99862618	0	-2.5587685	0.53747973	-1.1117817	3.45278375	-0.0003739	-6.9077553	10	82	3.57631111	296.888889	23.88888888	0	0	0	0	30.519848	1	0.21141735
0.144	2324.78044	0.42835591	54.1323737	1.18453371	0	-1.937942	0.84405566	-0.8454691	3.99145088	0.17019306	-6.9077553	11	75	3.57631111	298.555556	25.55555555	0	0	0	0	30.519848	1	0.21141735
0.2394	3196.28045	0.51699063	75.1148382	1.65386833	0	-1.4296195	1.16230059	-0.6577981	4.31903143	0.50372145	-6.9077553	12	69	4.47038889	299.111111	26.11111111	0	0	0	0	30.519848	1	0.21141735
0.396	4690.44308	0.67746086	129.212745	2.21089646	0	-0.9263411	1.54574023	-0.3879285	4.86146797	0.79385027	-6.9077553	13	64	4.91742778	300.777778	27.7777778	0	0	0	1	30.519848	1	0.21141735
0.6696	10106.0728	0.85775054	215.977899	3.18719824	0	-0.4010748	2.31323546	-0.1522768	5.37518071	1.15945594	-6.9077553	14	61	4.47038889	301.333333	28.33333333	0	0	0	1	30.519848	1	0.21141735
0.9414	15507.6678	0.79053583	174.680593	2.59941576	0	-0.0603872	2.74139908	-0.2337801	5.16296485	0.95567134	-6.9077553	15	59	4.47038889	301.333333	28.33333333	0	0	0	1	30.519848	1	0.21141735

1.2006	26334.7752	0.74016884	158.381456	1.65107006	0	0.18282143	3.27092829	-0.2995268	5.06501272	0.50202909	-6.9077553	16	54	6.25854444	301.888889	28.88888888	0	0	0	1	30.519848	1	0.21141735
1.4121	35389.6029	0.71416665	154.824036	1.49754093	0	0.34507796	3.56644633	-0.3352397	5.04229568	0.40449192	-6.9077553	17	54	4.02335	301.888889	28.88888888	0	0	0	1	30.519848	1	0.21141735
1.5399	42768.2715	0.63987915	119.743054	1.06621713	0	0.43171748	3.75581989	-0.4449144	4.78535658	0.06505444	-6.9077553	18	54	3.12927222	301.888889	28.88888888	0	0	0	1	30.519848	1	0.21141735
1.6425	48807.7649	0.60788787	109.971106	0.84051547	0	0.49621947	3.8879099	-0.4961211	4.70022675	-0.1725509	-6.9077553	19	55	3.12927222	300.777778	27.7777778	0	0	0	1	30.519848	1	0.21141735
1.7055	54286.5825	0.69273529	130.337123	0.87063223	0	0.53385832	3.99429552	-0.3656648	4.87013202	-0.1373877	-6.9077553	20	62	2.68223333	299.666667	26.66666666	0	0	0	1	30.519848	1	0.21141735
1.7649	60732.269	0.72599584	139.467226	0.99126573	0	0.56809403	4.10649164	-0.3188345	4.9378368	-0.0077643	-6.9077553	21	63	3.12927222	298.555556	25.55555555	0	0	0	1	30.519848	1	0.21141735
1.8315	67097.7634	0.74366693	146.746107	1.01663641	0	0.6051353	4.20616562	-0.2948182	4.98871074	0.01748269	-6.9077553	22	64	2.23519444	298.555556	25.55555555	0	0	0	1	30.519848	1	0.21141735
1.8963	74265.0619	0.68549977	123.998115	0.8953602	0	0.63990462	4.30765408	-0.3761494	4.82027443	-0.1094129	-6.9077553	23	67	2.68223333	298	25	0	0	0	1	30.519848	1	0.21141735
0.0009	0	0	0	0	0	-7.0131158	-6.9077553	-6.9077553	-6.9077553	-6.9077553	-6.9077553	0	82	2.68223333	296.333333	23.33333333	0	0	0	0	34.0724638	1	0.49277013
0.0036	131.59135	0.46151511	48.9325104	0.7560811	0	-5.6268214	-2.0204834	-0.7710761	3.89046245	-0.2782849	-6.9077553	1	86	2.23519444	295.777778	22.7777778	0	0	0	0	34.0724638	1	0.49277013
0.0108	438.637827	0.32618561	24.2627182	0.50798911	0	-4.5282091	-0.821804	-1.1172276	3.18898216	-0.6753287	-6.9077553	2	86	2.68223333	296.333333	23.33333333	0	0	0	0	34.0724638	1	0.49277013
0.0135	526.365391	0.25618609	14.5985591	0.47923477	0	-4.3050656	-0.6398616	-1.3579554	2.68099133	-0.7334802	-6.9077553	3	86	1.34111667	296.333333	23.33333333	0	0	0	0	34.0724638	1	0.49277013
0.0171	657.956723	0.23262289	10.9306231	0.26276353	0	-4.0686768	-0.4170974	-1.454047	2.39165979	-1.3327023	-6.9077553	4	86	2.23519444	296.333333	23.33333333	0	0	0	0	34.0724638	1	0.49277013
0.0234	877.275669	0.21964904	9.65783582	0.2803999	0	-3.7550193	-0.1297948	-1.5111819	2.26787313	-1.2679785	-6.9077553	5	87	1.34111667	296.333333	23.33333333	0	0	0	0	34.0724638	1	0.49277013
0.0288	1052.73091	0.21631471	10.667127	0.36911015	0	-3.5473799	0.05233711	-1.5264087	2.36726051	-0.9939546	-6.9077553	6	88	0.89407778	295.777778	22.7777778	0	0	0	0	34.0724638	1	0.49277013
0.0351	1272.04984	0.20665901	9.15108599	0.25168295	0	-3.3495541	0.24141547	-1.5718579	2.21398183	-1.3756198	-6.9077553	7	88	2.23519444	295.777778	22.7777778	0	0	0	0	34.0724638	1	0.49277013
0.0387	1403.64105	0.20334229	9.2673378	0.24895802	0	-3.2519157	0.33978179	-1.5879588	2.22660405	-1.3864623	-6.9077553	8	88	2.68223333	295.777778	22.7777778	0	0	0	0	34.0724638	1	0.49277013
0.045	1666.82372	0.27219718	16.974872	0.36021144	0	-3.1010928	0.51151962	-1.2975615	2.83179304	-1.0182918	-6.9077553	9	86	2.68223333	296.333333	23.33333333	0	0	0	0	34.0724638	1	0.49277013
0.0801	3094.52665	0.72675945	143.330228	1.36210407	0	-2.5244794	1.12995805	-0.3177847	4.96515823	0.30976451	-6.9077553	10	82	3.57631111	296.888889	23.88888888	0	0	0	0	34.0724638	1	0.49277013
0.1413	5604.33629	0.77523153	165.867087	1.34247331	0	-1.95687	1.72371905	-0.2533044	5.11119282	0.29525828	-6.9077553	11	75	3.57631111	298.555556	25.55555555	0	0	0	0	34.0724638	1	0.49277013
0.2277	9840.08687	0.96745348	264.063924	1.79031579	0	-1.4797263	2.28656616	-0.0320548	5.576195	0.58295043	-6.9077553	12	69	4.47038889	299.111111	26.11111111	0	0	0	0	34.0724638	1	0.49277013
0.5238	19293.1091	1.26505806	488.988317	3.99844908	0	-0.6466453	2.95979982	0.23590818	6.19234064	1.38615662	-6.9077553	13	64	4.91742778	300.777778	27.7777778	0	0	0	0	34.0724638	1	0.49277013
1.1097	27538.7563	1.41621786	635.519441	4.867222	1	0.10408971	3.31563065	0.34869569	6.45444426	1.58272878	0.0009995	14	61	4.47038889	301.333333	28.33333333	0	0	0	1	34.0724638	1	0.49277013
1.7541	43887.6028	1.1905925	416.438984	3.39267462	1	0.5619559	3.78165467	0.17529065	6.03174236	1.22191329	0.0009995	15	59	4.47038889	301.333333	28.33333333	0	0	0	1	34.0724638	1	0.49277013
2.4102	66138.9641	1.12141877	374.144805	3.26146155	2	0.87970973	4.19177316	0.11548597	5.92464557	1.18248199	0.69364706	16	54	6.25854444	301.888889	28.88888888	0	0	0	1	34.0724638	1	0.49277013
2.9322	83330.5817	0.9085244	225.612311	1.80414345	2	1.07575299	4.42282761	-0.0948335	5.41882252	0.59064006	0.69364706	17	54	4.02335	301.888889	28.88888888	0	0	0	1	34.0724638	1	0.49277013
3.5262	103369.817	0.88858347	216.638447	2.11649429	2	1.2602208	4.63832269	-0.1170019	5.37823444	0.75023345	0.69364706	18	54	3.12927222	301.888889	28.88888888	0	0	0	1	34.0724638	1	0.49277013
4.0869	119773.019	0.78637323	180.618605	1.9071525	2	1.40778674	4.78560679	-0.2390529	5.19639319	0.6461355	0.69364706	19	55	3.12927222	300.777778	27.7777778	0	0	0	1	34.0724638	1	0.49277013
4.3722	131564.601	0.87003353	212.457413	1.54694316	2	1.47526631	4.87950559	-0.1380748	5.35874627	0.43692706	0.69364706	20	62	2.68223333	299.666667	26.66666666	0	0	0	1	34.0724638	1	0.49277013
4.5873	140867.177	0.85816247	201.340743	1.63011776	2	1.52329162	4.94782454	-0.1517972	5.30500368	0.48926552	0.69364706	21	63	3.12927222	298.555556	25.55555555	0	0	0	1	34.0724638	1	0.49277013
4.8105	150565.448	0.85892271	198.665071	1.5372691	2	1.57080103	5.0144045	-0.1509128	5.29162538	0.43065782	0.69364706	22	64	2.23519444	298.555556	25.55555555	0	0	0	1	34.0724638	1	0.49277013
5.0967	163650.047	0.89574466	216.514519	1.69666717	2	1.62859327	5.0977364	-0.1089841	5.37766223	0.52925506	0.69364706	23	67	2.68223333	298	25	0	0	0	1	34.0724638	1	0.49277013
0.0009	0	0	0	0	0	-7.0131158	-6.9077553	-6.9077553	-6.9077553	-6.9077553	-6.9077553	0	82	2.68223333	296.333333	23.33333333	0	0	0	0	42.288408	1	-0.4111751
0.0045	175.455131	0.46975738	51.2976685	0.80981678	0	-5.4036779	-1.7346886	-0.7534124	3.9376648	-0.2097132	-6.9077553	1	86	2.23519444	295.777778	22.7777778	0	0	0	0	42.288408	1	-0.4111751
0.0126	570.229192	0.41000891	37.9054226	0.73039352	0	-4.3740585	-0.5599648	-0.8891404	3.63512056	-0.3128036	-6.9077553	2	86	2.68223333	296.333333	23.33333333	0	0	0	0	42.288408	1	-0.4111751
0.0306	1447.50486	0.31638212	22.115274	0.5218575	0	-3.4867553	0.3705319	-1.1476488	3.09631372	-0.6484463	-6.9077553	3	86	1.34111667	296.333333	23.33333333	0	0	0	0	42.288408	1	-0.4111751
0.0423	2017.73407	0.33447306	24.5306796	0.59191884	0	-3.1629682	0.70247062	-1.0922136	3.19996533	-0.5226978	-6.9077553	4	86	2.23519444	296.333333	23.33333333	0	0	0	0	42.288408	1	-0.4111751
0.0639	3070.46484	0.30389216	20.2936312	0.49926384	0	-2.7504359	1.1221546	-1.1877972	3.01035638	-0.6926196	-6.9077553	5	87	1.34111667	296.333333	23.33333333	0	0	0	0	42.288408	1	-0.4111751
0.081	3903.87638	0.26784729	15.7729123	0.42196028	0	-2.5133061	1.36222613	-1.3136118	2.75835745	-0.860477	-6.9077553	6	88	0.89407778	295.777778	22.7777778	0	0	0	0	42.288408	1	-0.4111751
0.1017	4868.88004	0.2576856	14.3412316	0.38733586	0	-2.285728	1.58306931	-1.3521419	2.66320845	-0.9458847	-6.9077553	7	88	2.23519444	295.777778	22.7777778	0	0	0	0	42.288408	1	-0.4111751
0.1269	6097.0653	0.29587133	20.806754	0.46757221	0	-2.0643559	1.80797156	-1.2144565	3.0353257	-0.7580651	-6.9077553	8	88	2.68223333	295.777778	22.7777778	0	0	0	0	42.288408	1	-0.4111751
0.2088	10337.6643	0.84443916	204.034877	1.71263743	0	-1.5663784	2.33589068	-0.1678991	5.31829585	0.53861826	-6.9077553	9	86	2.68223333	296.333333	23.33333333	0	0	0	0	42.288408	1	-0.4111751
0.4383	19996.9289	0.89969494	237.740732	2.42638155	0	-0.8248517	2.99562872	-0.1045887	5.47118493	0.88681312	-6.9077553	10	82	3.57631111	296.888889	23.88888888	0	0	0	0	42.288408	1	-0.4111751
0.6966	30996.7729	0.88438628	219.133439	1.90440214	0	-0.3615439	3.43391536	-0.1217313	5.38968542	0.64469309	-6.9077553	11	75	3.57631111	298.555556	25.55555555	0	0	1	0	42.288408	1	-0.4111751
1.0098	42939.006	0.84966831	189.531003	1.79199394	0	0.00975229	3.75980393	-0.161733	5.24455789	0.58388682	-6.9077553	12	69	4.47038889	299.111111	26.11111111	0	0	1	0	42.288408	1	-0.4111751
1.3554	56380.9917	0.83061171	182.900004	1.56528155	0	0.30409661	4.03214981	-0.1843896	5.20894505	0.44870437	-6.9077553	13	64	4.91742778	300.777778	27.7777778	0	0	1	0	42.288408	1	-0.4111751
1.7919	72796.6268	0.86726351	200.06097	1.97002457	0	0.58327651	4.28768336	-0.14126	5.29862717	0.67855349	-6.9077553	14	61	4.47038889	301.333333	28.33333333	0	0	1	0	42.288408	1	-0.4111751
2.3949	92915.3712	0.8636951	209.568843	2.33735352	0	0.87334148	4.53169985	-0.1453783	5.34505706	0.84944706	-6.9077553	15	59	4.47038889	301.333333	28.33333333	0	0	1	0	42.288408	1	-0.4111751
3.1257	125112.116	0.98118298	283.994073	2.76598219	0	1.13965826	4.82921826	-0.0179777	5.64895689	1.01775726	-6.9077553	16	54	6.25854444	301.888889	28.88888888	0	0	1	0	42.288408	1	-0.4111751
3.8718	161021.099	0.99706582	276.550607	2.18623056	0	1.35371952	5.08154161	-0.0019361	5.62239745	0.78263616	-6.9077553	17	54	4.02335	301.888889	28.88888888	0	0	1	0	42.288408	1	-0.4111751
4.5909	196351.628	0.96358141	258.721644	1.9315155	0	1.52407608	5.27991216	-0.036061	5.55575662	0.65882252	-6.9077553	18	54	3.12927222	301.888889	28.88888888	0	0	1	0	42.288408	1	-0.4111751
5.1453	221269.22	0.88280556	224.93509	1.70115662	0	1.63808368	5.39938467	-0.1235182	5.41581632	0.53189604	-6.9077553	19	55	3.12927222	300.777778	27.7777778	0	0	1	0	42.288408	1	-0.4111751
5.4729	236210.002	0.86940841	209.039449	1.49782185	0	1.69980864	5.46472548	-0.1387927	5.34252777	0.40467937	-6.9077553	20	62	2.68223333	299.666667	26.66666666	0	0	1	0	42.288408	1	-0.4111751
5.733	247359.416	0.81237719	176.043212	1.34150416	0	1.74623895	5.51084645	-0.2065603	5.17073517	0.29453665	-6.9077553	21	63	3.12927222	298.555556	25.55555555	0	0	1	0	42.288408	1	-0.4111751
5.9589	256902.436	0.80595744	173.117981	1.37116111	0	1.7848859	5.54870028	-0.2144843	5.15397911	0.31638695	-6.9077553	22	64	2.23519444	298.555556	25.55555555	0	0	1	0	42.288408	1	-0.4111751
6.147	264217.899	0.80080907	172.232319	1.38578176	0	1.81596416	5.57677792	-0.2208848	5.14885006	0.32698578	-6.9077553	23	67	2.68223333	298	25	0	0	1	0	42.288408	1	-0.4111751
0.0009	0	0	0	0	0	-7.0131158	-6.9077553	-6.9077553	-6.9077553	-6.9077553	-6.9077553	0	82	2.68223333	296.333333	23.33333333	0	0	0	0	18.9135952	1	-0.0428739
0.0009	0	0	0	0	0	-7.0131158	-6.9077553	-6.9077553	-6.9077553	-6.9077553	-6.9077553	1	86	2.23519444	295.777778	22.7777778	0	0	0	0	18.9135952	1	-0.0428739
0.0009	0	0	0	0	0	-7.0131158	-6.9077553	-6.9077553	-6.9077553	-6.9077553	-6.9077553	2	86	2.68223333	296.333333	23.33333333	0	0	0	0	18.9135952	1	-0.0428739
0.0009	0	0	0	0	0	-7.0131158	-6.9077553	-6.9077553	-6.9077553	-6.9077553	-6.9077553	3	86	1.34111667	296.333333	23.33333333	0	0	0	0	18.9135952	1	-0.0428739
0.0009	0	0	0	0	0	-7.0131158	-6.9077553	-6.9077553	-6.9077553	-6.9077553	-6.9077553	4	86	2.23519444	296.333333	23.33333333	0	0	0	0	18.9135952	1	-0.0428739
0.0009	0	0	0	0	0	-7.0131158	-6.9077553	-6.9077553	-6.9077553	-6.9077553	-6.9077553	5	87	1.34111667	296.333333	23.33333333	0	0	0	0	18.9135952	1	-0.0428739

0.0009	0	0	0	0	0	-7.0131158	-6.9077553	-6.9077553	-6.9077553	-6.9077553	-6.9077553	6	88	0.89407778	295.777778	22.7777778	0	0	0	0	18.9135952	1	-0.0428739
0.0009	0	0	0	0	0	-7.0131158	-6.9077553	-6.9077553	-6.9077553	-6.9077553	-6.9077553	7	88	2.23519444	295.777778	22.7777778	0	0	0	0	18.9135952	1	-0.0428739
0.0009	0	0	0	0	0	-7.0131158	-6.9077553	-6.9077553	-6.9077553	-6.9077553	-6.9077553	8	88	2.68223333	295.777778	22.7777778	0	0	0	0	18.9135952	1	-0.0428739
0.0009	0	0	0	0	0	-7.0131158	-6.9077553	-6.9077553	-6.9077553	-6.9077553	-6.9077553	9	86	2.68223333	296.333333	23.33333333	0	0	0	0	18.9135952	1	-0.0428739
0.0009	0	0	0	0	0	-7.0131158	-6.9077553	-6.9077553	-6.9077553	-6.9077553	-6.9077553	10	82	3.57631111	296.888889	23.88888888	0	0	0	0	18.9135952	1	-0.0428739
0.0171	0	0.50952042	70.1668498	1.96600829	0	-4.0686768	-6.9077553	-0.6723246	4.25089023	0.67651376	-6.9077553	11	75	3.57631111	298.555556	25.55555555	0	0	0	0	18.9135952	1	-0.0428739
0.0963	414.266796	0.68508803	133.143932	2.62568769	0	-2.340287	-0.8788341	-0.3767493	4.89143825	0.96572362	-6.9077553	12	69	4.47038889	299.111111	26.11111111	0	0	0	0	18.9135952	1	-0.0428739
0.27	4634.83443	0.79837273	199.804364	2.20334511	0	-1.3093333	1.53381621	-0.2239279	5.29734371	0.79043046	-6.9077553	13	64	4.91742778	300.777778	27.7777778	0	0	1	0	18.9135952	1	-0.0428739
0.4896	12855.8492	0.84691908	233.768635	2.16756026	0	-0.7141665	2.55387668	-0.1649701	5.45433616	0.77406347	-6.9077553	14	61	4.47038889	301.333333	28.33333333	0	0	1	0	18.9135952	1	-0.0428739
0.7434	20325.9848	0.82781609	232.315187	2.16084885	0	-0.296521	3.0119493	-0.187757	5.44809932	0.7709638	-6.9077553	15	59	4.47038889	301.333333	28.33333333	0	0	1	0	18.9135952	1	-0.0428739
1.1502	33312.956	0.96438234	289.85533	3.46883399	0	0.13993584	3.50597641	-0.035231	5.66938539	1.24410675	-6.9077553	16	54	6.25854444	301.888889	28.88888888	0	0	1	0	18.9135952	1	-0.0428739
1.467	45929.8105	0.85406798	213.652455	1.86837942	0	0.3832195	3.82713614	-0.1565743	5.36435533	0.62560651	-6.9077553	17	54	4.02335	301.888889	28.88888888	0	0	1	0	18.9135952	1	-0.0428739
1.8315	62119.9144	0.9179469	243.048302	1.94333526	0	0.6051353	4.12908272	-0.0845269	5.49326431	0.66492015	-6.9077553	18	54	3.12927222	301.888889	28.88888888	0	0	1	0	18.9135952	1	-0.0428739
2.187	78383.6461	0.91349866	236.727888	1.73259339	0	0.78253074	4.36162807	-0.0893793	5.46691555	0.55019636	-6.9077553	19	55	3.12927222	300.777778	27.7777778	0	0	1	0	18.9135952	1	-0.0428739
2.4534	91631.0548	0.91842311	241.145851	1.70570456	0	0.89747482	4.51778115	-0.0840089	5.48540609	0.53456435	-6.9077553	20	62	2.68223333	299.666667	26.66666666	0	0	1	0	18.9135952	1	-0.0428739
2.6685	102584.755	0.91059512	228.476203	1.70991216	0	0.98151652	4.63069909	-0.0925593	5.43143644	0.53702666	-6.9077553	21	63	3.12927222	298.555556	25.55555555	0	0	1	0	18.9135952	1	-0.0428739
2.8611	112082.518	0.86710379	205.692593	1.57188173	0	1.05120617	4.71924429	-0.141444	5.32638765	0.45290943	-6.9077553	22	64	2.23519444	298.555556	25.55555555	0	0	1	0	18.9135952	1	-0.0428739
3.0573	122149.115	0.91525199	227.998351	1.71284555	0	1.11753217	4.80525074	-0.0874639	5.42934278	0.5387397	-6.9077553	23	67	2.68223333	298	25	0	0	1	0	18.9135952	1	-0.0428739
0.0144	368.242533	0.77091128	167.947721	1.83542849	0	-4.2405271	-0.9963016	-0.2588857	5.1236587	0.60782265	-6.9077553	0	92	4.02335	298	25	0	0	0	0	30.519848	1	0.21141735
0.0477	1027.35156	0.47903205	66.3632549	1.22798177	0	-3.0428239	0.02795709	-0.7339024	4.19515858	0.206186	-6.9077553	1	81	4.02335	299.666667	26.66666666	0	0	0	0	30.519848	1	0.21141735
0.1143	1867.12795	0.55113169	91.4542005	1.39422979	0	-2.1689287	0.62493683	-0.5939687	4.51584924	0.33305912	-6.9077553	2	75	4.02335	300.777778	27.7777778	0	0	0	0	30.519848	1	0.21141735
0.1899	2744.40373	0.51393168	76.1192511	1.61098026	0	-1.6612577	1.00992814	-0.663721	4.33231434	0.4774634	-6.9077553	3	63	4.02335	303.555556	30.55555555	0	0	0	0	30.519848	1	0.21141735
0.2988	3365.27201	0.54272852	78.3472931	1.64169349	0	-1.2079808	1.2138059	-0.6093052	4.36116418	0.49633727	-6.9077553	4	58	3.12927222	304.666667	31.66666666	0	0	0	0	30.519848	1	0.21141735
0.4311	4521.89875	0.64254657	111.409884	1.98887956	0	-0.8414152	1.5091531	-0.4407609	4.71322502	0.68807411	-6.9077553	5	51	3.12927222	305.777778	32.7777778	0	0	0	1	30.519848	1	0.21141735
0.5967	7400.077	0.71261248	138.846807	1.72645503	0	-0.5163408	2.00162553	-0.3374152	4.93337842	0.54664924	-6.9077553	6	52	2.68223333	305.777778	32.7777778	0	0	0	1	30.519848	1	0.21141735
0.8073	12534.257	0.77615552	172.546691	1.92454229	0	-0.2140599	2.52854523	-0.2521148	5.15067367	0.65520764	-6.9077553	7	58	1.78815556	305.222222	32.2222222	0	0	0	1	30.519848	1	0.21141735
1.0575	16369.7884	0.67459117	133.252487	1.6518131	0	0.05590763	2.79549855	-0.3921672	4.89225323	0.50247874	-6.9077553	8	51	1.78815556	306.333333	33.33333333	0	0	0	1	30.519848	1	0.21141735
1.3446	20880.0716	0.74230244	155.191614	2.44513743	0	0.29609657	3.03884308	-0.2966523	5.04466702	0.89451022	-6.9077553	9	48	4.91742778	306.333333	33.33333333	0	0	0	1	30.519848	1	0.21141735
1.5597	26720.066	0.71694934	148.310326	1.44421642	0	0.4444935	3.28545224	-0.3313563	4.99931362	0.36825908	-6.9077553	10	50	4.02335	305.777778	32.7777778	0	0	0	1	30.519848	1	0.21141735
1.7073	31041.0711	0.66618422	125.683379	1.2267056	0	0.53491318	3.43534342	-0.4046891	4.83377384	0.20514706	-6.9077553	11	59	5.81150556	301.333333	28.33333333	0	0	0	1	30.519848	1	0.21141735
1.782	33791.0519	0.76382979	152.422946	1.05862261	0	0.57773633	3.52022562	-0.268102	5.02666575	0.05791282	-6.9077553	12	70	2.23519444	299.666667	26.66666666	0	0	0	1	30.519848	1	0.21141735
1.8414	36213.8193	0.75578291	151.610485	1.20120586	0	0.61052615	3.58946841	-0.2786788	5.02132123	0.18415809	-6.9077553	13	71	1.34111667	298.555556	25.55555555	0	0	0	1	30.519848	1	0.21141735
1.8891	38319.007	0.64657463	115.914468	1.02046563	0	0.63610053	3.64597214	-0.4345212	4.7528612	0.02123849	-6.9077553	14	77	1.34111667	297.444444	24.44444444	0	0	0	1	30.519848	1	0.21141735
1.899	38713.7793	0.36413713	30.9523121	0.51027818	0	0.64132743	3.65622142	-1.0074823	3.43248001	-0.6708415	-6.9077553	15	82	0.44703889	296.333333	23.33333333	0	0	0	1	30.519848	1	0.21141735
1.9125	39327.8748	0.21864657	10.2488251	0.30926208	0	0.64841129	3.67195898	-1.5157355	2.32726064	-1.1703379	-6.9077553	16	88	0.44703889	295.777778	22.7777778	0	0	0	1	30.519848	1	0.21141735
1.9224	39722.6478	0.23028909	11.2410819	0.31673128	0	0.65357441	3.68194667	-1.4640869	2.41966405	-1.1465493	-6.9077553	17	93	0.44703889	295.777778	22.7777778	0	0	0	1	30.519848	1	0.21141735
1.9332	40117.4207	0.23389238	11.4024989	0.34641131	0	0.65917666	3.6918356	-1.4486278	2.43392023	-1.0572459	-6.9077553	18	95	0.89407778	295.777778	22.7777778	0	0	0	1	30.519848	1	0.21141735
1.9458	40687.6527	0.22575797	10.5482832	0.31509355	0	0.6656732	3.70594925	-1.483872	2.35605791	-1.1517171	-6.9077553	19	95	1.78815556	296.333333	23.33333333	0	0	0	1	30.519848	1	0.21141735
1.9557	41082.4255	0.20806798	9.2981935	0.3387073	0	0.67074819	3.71560477	-1.5650958	2.22992768	-1.0796709	-6.9077553	20	93	1.34111667	297.444444	24.44444444	0	0	0	1	30.519848	1	0.21141735
1.9647	41433.3349	0.20917587	9.81045151	0.32854609	0	0.67533956	3.72410988	-1.5598106	2.28355022	-1.110039	-6.9077553	21	91	1.34111667	297.444444	24.44444444	0	0	0	1	30.519848	1	0.21141735
1.971	41740.3828	0.22447337	10.4675184	0.33719383	0	0.67854103	3.73149303	-1.4895532	2.34837251	-1.0841361	-6.9077553	22	93	0.89407778	297.444444	24.44444444	0	0	0	1	30.519848	1	0.21141735
1.9791	42091.2894	0.22028524	10.0306108	0.29964124	0	0.6826422	3.73986458	-1.5083027	2.30574119	-1.2018376	-6.9077553	23	94	2.23519444	297.444444	24.44444444	0	0	0	1	30.519848	1	0.21141735
0.0198	869.661991	0.99756232	285.237398	2.36740258	0	-3.9220733	-0.1385014	-0.0014387	5.65332531	0.86221571	-6.9077553	0	92	4.02335	298	25	0	0	0	0	34.0724638	1	0.49277013
0.0387	1527.61871	0.59484423	89.0654529	0.9114626	0	-3.2519157	0.42436452	-0.517776	4.48938275	-0.0916082	-6.9077553	1	81	4.02335	299.666667	26.66666666	0	0	0	0	34.0724638	1	0.49277013
0.1035	4276.28616	0.88500637	214.310764	1.78095669	0	-2.2681837	1.45331873	-0.1210311	5.3674318	0.57771203	-6.9077553	2	75	4.02335	300.777778	27.7777778	0	0	0	0	34.0724638	1	0.49277013
0.1764	7685.92456	0.92706723	226.197651	1.48374176	0	-1.7350011	2.03952078	-0.0746511	5.4214136	0.39524086	-6.9077553	3	63	4.02335	303.555556	30.55555555	0	0	0	0	34.0724638	1	0.49277013
0.2781	11771.5369	0.89871256	217.908082	1.72163526	0	-1.2797745	2.46576943	-0.1056799	5.38407792	0.54385525	-6.9077553	4	58	3.12927222	304.666667	31.66666666	0	0	0	1	34.0724638	1	0.49277013
0.5049	21024.9567	1.08081781	332.296472	2.68145098	0	-0.6833949	3.04575771	0.07864279	5.80603057	0.98673092	-6.9077553	5	51	3.12927222	305.777778	32.7777778	0	0	0	1	34.0724638	1	0.49277013
0.8559	37415.3521	1.19451137	406.672035	3.0742344	0	-0.1556017	3.62210783	0.17857402	6.00800951	1.12338113	-6.9077553	6	52	2.68223333	305.777778	32.7777778	0	0	0	1	34.0724638	1	0.49277013
1.1574	48814.8599	0.98626499	272.333815	2.22342511	0	0.14617611	3.88805526	-0.0128168	5.60703225	0.79949851	-6.9077553	7	58	1.78815556	305.222222	32.2222222	0	0	0	1	34.0724638	1	0.49277013
1.4013	54852.8281	0.81593373	179.767153	1.3782575	0	0.33740038	4.00467198	-0.2021973	5.19166798	0.32154531	-6.9077553	8	51	1.78815556	306.333333	33.33333333	0	0	0	1	34.0724638	1	0.49277013
2.1771	63339.4926	1.40653776	634.191995	4.83126314	1	0.77799372	4.14852482	0.34184191	6.45235332	1.57531492	0.0009995	9	48	4.91742778	306.333333	33.33333333	0	0	0	1	34.0724638	1	0.49277013
2.8287	79442.2269	1.01699441	294.891316	2.64485969	1	1.03981724	4.37504264	0.01783443	5.68661026	0.97299604	0.0009995	10	50	4.02335	305.777778	32.7777778	0	0	0	1	34.0724638	1	0.49277013
3.4245	104722.353	0.98803766	288.010291	2.41161741	1	1.23095548	4.65132214	-0.0110229	5.66299968	0.88071222	0.0009995	11	59	5.81150556	301.333333	28.33333333	0	0	0	1	34.0724638	1	0.49277013
3.7485	119850.685	0.84441884	191.511914	1.40387726	1	1.32135576	4.78625502	-0.1679231	5.25495524	0.33994994	0.0009995	12	70	2.23519444	299.666667	26.66666666	0	0	0	1	34.0724638	1	0.49277013
4.0005	130648.547	0.7601944	150.353836	1.24971858	1	1.38641935	4.87251852	-0.2728665	5.01299808	0.22371825	0.0009995	13	71	1.34111667	298.555556	25.55555555	0	0	0	1	34.0724638	1	0.49277013
4.1796	138759.605	0.72847177	140.862339	1.28959341	1	1.43021555	4.93275018	-0.3154346	4.94779019	0.25510212	0.0009995	14	77	1.34111667	297.444444	24.44444444	0	0	0	1	34.0724638	1	0.49277013
4.248	141303.705	0.31938523	28.5485936	0.63252344	1	1.44644828	4.95091859	-1.1382312	3.3516427	-0.4564583	0.0009995	15	82	0.44703889	296.333333	23.33333333	0	0	0	1	34.0724638	1	0.49277013
4.2858	142751.203	0.27880957	21.7881571	0.52692681	1	1.45530723	4.96111028	-1.273646	3.08141247	-0.6387976	0.0009995	16	88	0.44703889	295.777778	22.7777778	0	0	0	1	34.0724638	1	0.49277013
4.3263	144461.889	0.25188175	15.1788425	0.36294746	1	1.46471267	4.97302265	-1.3748333	2.7199684	-1.0107458	0.0009995	17	93	0.44703889	295.777778	22.7777778	0	0	0	1	34.0724638	1	0.49277013
4.3614	145733.945	0.20191513	8.5437982	0.30693661	1	1.47279311	4.98178953	-1.5949675	2.1453227	-1.1778613	0.0009995	18	95	0.89407778	295.777778	22.7777778	0	0	0	1	34.0724638	1	0.49277013
4.392	147093.727	0.18198267	7.14699195	0.37707357	1	1.4797847	4.99107678	-1.6983638	1.96683147	-0.9726665	0.0009995	19	95	1.78815556	296.333333	23.33333333	0	0	0	1	34.0724638	1	0.49277013

4.4244	148585.08	0.26020734	22.0203196	0.38972863	1	1.48713468	5.00116446	-1.3424408	3.09201106	-0.939742	0.0009995	20	93	1.34111667	297.444444	24.44444444	0	0	0	1	34.0724638	1	0.49277013
4.4577	149813.271	0.21889983	12.0421687	0.31384445	1	1.49463294	5.00939634	-1.5145832	2.48849759	-1.1556766	0.0009995	21	91	1.34111667	297.444444	24.44444444	0	0	0	1	34.0724638	1	0.49277013
4.4883	150866.008	0.27111738	25.7583923	0.46235634	1	1.50147401	5.01639871	-1.3015218	3.24879931	-0.7692589	0.0009995	22	93	0.89407778	297.444444	24.44444444	0	0	0	1	34.0724638	1	0.49277013
4.5117	151918.741	0.22055582	14.0280644	0.32497879	1	1.50667402	5.02335236	-1.5070807	2.64113121	-1.120923	0.0009995	23	94	2.23519444	297.444444	24.44444444	0	0	0	1	34.0724638	1	0.49277013
0.036	2318.21844	1.13495822	357.203626	2.47004778	0	-3.3242363	0.84123025	0.12747654	5.8783088	0.90464226	-6.9077553	0	92	4.02335	298	25	0	0	0	0	42.288408	1	-0.4111751
0.1323	6276.1619	0.90490708	223.428793	2.71301412	0	-2.0226832	1.83691795	-0.0988185	5.40909724	0.99842876	-6.9077553	1	81	4.02335	299.666667	26.66666666	0	0	0	0	42.288408	1	-0.4111751
0.3636	16002.8314	1.01709056	288.058791	3.12880935	0	-1.0117009	2.77282816	0.01792887	5.66316807	1.14097209	-6.9077553	2	75	4.02335	300.777778	27.7777778	0	0	0	0	42.288408	1	-0.4111751
0.6408	28843.9356	0.98223897	259.195947	2.16072954	0	-0.4450379	3.36193444	-0.0169031	5.55758818	0.77090862	-6.9077553	3	63	4.02335	303.555556	30.55555555	0	0	0	0	42.288408	1	-0.4111751
0.9477	41719.8023	0.89192643	218.239003	1.74412183	0	-0.0537173	3.73099986	-0.1132511	5.38559539	0.55682437	-6.9077553	4	58	3.12927222	304.666667	31.66666666	0	0	0	0	42.288408	1	-0.4111751
1.332	57657.9849	0.92122348	233.282586	1.89861875	0	0.28668157	4.05454609	-0.0809677	5.45225482	0.64165321	-6.9077553	5	51	3.12927222	305.777778	32.7777778	0	0	1	0	42.288408	1	-0.4111751
1.8405	76349.9628	0.8822607	218.662804	2.12881378	0	0.61003727	4.33534064	-0.1241349	5.38753541	0.75603455	-6.9077553	6	52	2.68223333	305.777778	32.7777778	0	0	1	0	42.288408	1	-0.4111751
2.3346	94477.0927	0.87671249	210.699653	1.91929351	0	0.84784057	4.54836798	-0.1304362	5.35043842	0.65247805	-6.9077553	7	58	1.78815556	305.222222	32.2222222	0	0	1	0	42.288408	1	-0.4111751
2.7702	111464.827	0.84826868	196.083032	1.55788403	0	1.01891952	4.71371806	-0.1633797	5.2785433	0.4439702	-6.9077553	8	51	1.78815556	306.333333	33.33333333	0	0	1	0	42.288408	1	-0.4111751
3.3822	139054.699	0.96264756	261.258387	2.15386873	0	1.21852639	4.93487456	-0.0370297	5.56551373	0.76772981	-6.9077553	9	48	4.91742778	306.333333	33.33333333	0	0	1	0	42.288408	1	-0.4111751
4.0842	174162.477	0.98775181	279.185617	2.13600714	0	1.40712587	5.15999438	-0.0113119	5.63188044	0.75940632	-6.9077553	10	50	4.02335	305.777778	32.7777778	0	0	1	0	42.288408	1	-0.4111751
4.6728	203854.904	0.99028458	296.324016	2.25650407	0	1.54175846	5.31741339	-0.0087536	5.69145688	0.81425981	-6.9077553	11	59	5.81150556	301.333333	28.33333333	0	0	1	0	42.288408	1	-0.4111751
4.9374	216360.112	0.83155833	186.619207	1.35939161	0	1.59683888	5.37694883	-0.183252	5.22907557	0.30777261	-6.9077553	12	70	2.23519444	299.666667	26.66666666	0	0	1	0	42.288408	1	-0.4111751
5.1858	226900.353	0.7890092	166.522617	1.37273175	0	1.64592412	5.42451535	-0.2357107	5.11513714	0.31753094	-6.9077553	13	71	1.34111667	298.555556	25.55555555	0	0	1	0	42.288408	1	-0.4111751
5.391	234970.216	0.70836024	133.830404	1.25502777	0	1.6847309	5.45946302	-0.3433918	4.89658083	0.22795418	-6.9077553	14	77	1.34111667	297.444444	24.44444444	0	0	1	0	42.288408	1	-0.4111751
5.4432	236944.064	0.41729986	46.02007	0.64506518	0	1.69436712	5.46782832	-0.8715567	3.82909934	-0.4368549	-6.9077553	15	82	0.44703889	296.333333	23.33333333	0	0	1	0	42.288408	1	-0.4111751
5.4783	238172.251	0.21882766	10.5969935	0.45077916	0	1.70079483	5.47299835	-1.5149114	2.36066469	-0.7945618	-6.9077553	16	88	0.44703889	295.777778	22.7777778	0	0	1	0	42.288408	1	-0.4111751
5.5188	239663.65	0.20369956	8.60768229	0.31502402	0	1.70816045	5.47924065	-1.586212	2.15277126	-1.1519371	-6.9077553	17	93	0.44703889	295.777778	22.7777778	0	0	1	0	42.288408	1	-0.4111751
5.5431	240760.237	0.20312977	8.30730749	0.27809138	0	1.71255391	5.48380573	-1.5889994	2.11725592	-1.276216	-6.9077553	18	95	0.89407778	295.777778	22.7777778	0	0	1	0	42.288408	1	-0.4111751
5.5746	242120.032	0.19204584	7.52722953	0.27328404	0	1.71822057	5.48943773	-1.6448276	2.01865989	-1.2935911	-6.9077553	19	95	1.78815556	296.333333	23.33333333	0	0	1	0	42.288408	1	-0.4111751
5.6043	243304.329	0.19030532	7.48309927	0.28897309	0	1.72353416	5.49431715	-1.6538846	2.01278067	-1.2379672	-6.9077553	20	93	1.34111667	297.444444	24.44444444	0	0	1	0	42.288408	1	-0.4111751
5.6358	244707.973	0.19535141	7.76741246	0.2429984	0	1.72913911	5.50006964	-1.6278493	2.05006583	-1.4105936	-6.9077553	21	91	1.34111667	297.444444	24.44444444	0	0	1	0	42.288408	1	-0.4111751
5.6682	246155.473	0.19962545	8.19909075	0.25707139	0	1.73487161	5.5059674	-1.6063155	2.10414522	-1.354519	-6.9077553	22	93	0.89407778	297.444444	24.44444444	0	0	1	0	42.288408	1	-0.4111751
5.6988	247471.416	0.21280377	9.5048873	0.29252131	0	1.74025563	5.51129912	-1.5426966	2.25191132	-1.225805	-6.9077553	23	94	2.23519444	297.444444	24.44444444	0	0	1	0	42.288408	1	-0.4111751
0.0099	0	0.59813786	97.2446716	2.6484211	0	-4.6152205	-6.9077553	-0.5122636	4.57724047	0.97434116	-6.9077553	0	92	4.02335	298	25	0	0	0	0	18.9135952	1	-0.0428739
0.0396	88.7761357	0.45420667	64.9902122	1.6703905	0	-3.2289262	-2.4104361	-0.7870037	4.17425206	0.51365592	-6.9077553	1	81	4.02335	299.666667	26.66666666	0	0	0	0	18.9135952	1	-0.0428739
0.1323	703.587453	0.57354088	91.1921553	1.86086577	0	-2.0226832	-0.3501428	-0.554184	4.51297984	0.62157909	-6.9077553	2	75	4.02335	300.777778	27.7777778	0	0	0	0	18.9135952	1	-0.0428739
0.3429	6827.57469	0.83903008	227.789864	2.53856483	0	-1.0703164	1.92111597	-0.1743176	5.42842794	0.93199274	-6.9077553	3	63	4.02335	303.555556	30.55555555	0	0	1	0	18.9135952	1	-0.0428739
0.5481	13867.0199	0.79863639	219.2402	1.97774412	0	-0.6012975	2.62958546	-0.2235982	5.39017249	0.68246236	-6.9077553	4	58	3.12927222	304.666667	31.66666666	0	0	1	0	18.9135952	1	-0.0428739
0.7047	17935.2682	0.69102408	152.689206	1.36054651	0	-0.3499831	2.88682482	-0.3681345	5.02841107	0.30862119	-6.9077553	5	51	3.12927222	305.777778	32.7777778	0	0	1	0	18.9135952	1	-0.0428739
0.9099	24234.4494	0.79711258	199.773198	1.79352556	0	-0.0944206	3.18781641	-0.2255056	5.29718772	0.58474068	-6.9077553	6	52	2.68223333	305.777778	32.7777778	0	0	1	0	18.9135952	1	-0.0428739
1.1664	32426.0234	0.81877399	200.83051	1.87674068	0	0.15392208	3.47899213	-0.1987266	5.3024663	0.63006929	-6.9077553	7	58	1.78815556	305.222222	32.2222222	0	0	1	0	18.9135952	1	-0.0428739
1.4337	40658.3535	0.81050161	194.259838	1.73561666	0	0.36025852	3.70522891	-0.2088689	5.26920178	0.55193877	-6.9077553	8	51	1.78815556	306.333333	33.33333333	0	0	1	0	18.9135952	1	-0.0428739
1.7676	53918.2741	0.89631303	245.458853	2.21168292	0	0.56962269	3.987488	-0.1083505	5.5031334	0.79420577	-6.9077553	9	48	4.91742778	306.333333	33.33333333	0	0	1	0	18.9135952	1	-0.0428739
2.106	66275.9509	0.83197906	203.870512	1.76737716	0	0.74479041	4.19384219	-0.1827468	5.31748995	0.57006227	-6.9077553	10	50	4.02335	305.777778	32.7777778	0	0	1	0	18.9135952	1	-0.0428739
2.4462	83420.6141	0.97443483	277.261967	2.05720124	0	0.8945358	4.42390744	-0.0248719	5.62496639	0.72183242	-6.9077553	11	59	5.81150556	301.333333	28.33333333	0	0	1	0	18.9135952	1	-0.0428739
2.6361	92610.7648	0.8909706	211.892958	1.54551386	0	0.96930055	4.52841618	-0.1143221	5.35608595	0.43600327	-6.9077553	12	70	2.23519444	299.666667	26.66666666	0	0	1	0	18.9135952	1	-0.0428739
2.7837	99326.1967	0.77348151	162.870236	1.35874362	0	1.02378098	4.59841942	-0.2555615	5.09295993	0.30729617	-6.9077553	13	71	1.34111667	298.555556	25.55555555	0	0	1	0	18.9135952	1	-0.0428739
2.9142	105416.759	0.72608748	140.360399	1.28315093	0	1.06959534	4.65793112	-0.3187085	4.94422052	0.25009774	-6.9077553	14	77	1.34111667	297.444444	24.44444444	0	0	1	0	18.9135952	1	-0.0428739
2.9466	106776.534	0.23873568	12.2804269	0.44940699	0	1.08065196	4.67074754	-1.4282183	2.50808811	-0.7976037	-6.9077553	15	82	0.44703889	296.333333	23.33333333	0	0	1	0	18.9135952	1	-0.0428739
2.9808	108399.499	0.24538371	12.7515187	0.38165848	0	1.09219172	4.68583269	-1.4008651	2.54572879	-0.9606124	-6.9077553	16	88	0.44703889	295.777778	22.7777778	0	0	1	0	18.9135952	1	-0.0428739
3.0177	109846.999	0.23744318	12.3701537	0.40279314	0	1.10449495	4.69909758	-1.4336242	2.51536744	-0.9068526	-6.9077553	17	93	0.44703889	295.777778	22.7777778	0	0	1	0	18.9135952	1	-0.0428739
3.0528	111382.228	0.22814567	11.1850351	0.38110958	0	1.1160592	4.71297676	-1.4733974	2.41466614	-0.9620479	-6.9077553	18	95	0.89407778	295.777778	22.7777778	0	0	1	0	18.9135952	1	-0.0428739
3.087	112917.456	0.24068238	12.1857597	0.37521154	0	1.12719975	4.72666593	-1.4201309	2.50035009	-0.9776037	-6.9077553	19	95	1.78815556	296.333333	23.33333333	0	0	1	0	18.9135952	1	-0.0428739
3.1257	114672.015	0.24314346	12.7228394	0.43031125	0	1.13965826	4.74208473	-1.4099993	2.54347735	-0.8409253	-6.9077553	20	93	1.34111667	297.444444	24.44444444	0	0	1	0	18.9135952	1	-0.0428739
3.1608	116251.119	0.26112202	14.5507312	0.43502852	0	1.15082516	4.75576127	-1.3389452	2.67770997	-0.8300476	-6.9077553	21	91	1.34111667	297.444444	24.44444444	0	0	1	0	18.9135952	1	-0.0428739
3.2004	117917.928	0.23500414	12.3039787	0.4218981	0	1.1632758	4.76999734	-1.4439059	2.51000395	-0.860624	-6.9077553	22	93	0.89407778	297.444444	24.44444444	0	0	1	0	18.9135952	1	-0.0428739
3.24	119672.487	0.24665624	13.3217496	0.42791856	0	1.17557333	4.78476709	-1.3957136	2.58947307	-0.8464882	-6.9077553	23	94	2.23519444	297.444444	24.44444444	0	0	1	0	18.9135952	1	-0.0428739
0.0153	404.721177	0.82179838	183.453156	1.63174605	0	-4.1799025	-0.9020891	-0.1950441	5.2119648	0.49026329	-6.9077553	0	98	2.68223333	289.666667	16.66666666	0	0	0	0	30.519848	1	0.21141735
0.036	1150.73982	0.60215776	108.348166	1.0385889	0	-3.3242363	0.14127369	-0.5055765	4.68535903	0.03882534	-6.9077553	1	100	1.78815556	289.666667	16.66666666	0	0	0	0	30.519848	1	0.21141735
0.0882	1808.76509	0.51866143	78.9832974	1.18358007	0	-2.4281483	0.59319705	-0.6545778	4.36924907	0.16938834	-6.9077553	2	93	3.57631111	292.444444	19.44444444	0	0	0	0	30.519848	1	0.21141735
0.1809	2626.25064	0.44781816	56.5195502	1.55367138	0	-1.7098109	0.96593792	-0.8011375	4.03460429	0.44126419	-6.9077553	3	76	4.47038889	294.111111	21.11111111	0	0	0	0	30.519848	1	0.21141735
0.351	3621.59577	0.67273744	136.22493	3.13574025	0	-1.0469691	1.28719083	-0.3949148	4.91431476	1.14318412	-6.9077553	4	63	5.36446667	294.111111	21.11111111	0	0	0	0	30.519848	1	0.21141735
0.513	4639.09999	0.65215734	111.05472	2.20436096	0	-0.6674794	1.53473592	-0.4259372	4.71003206	0.7908912	-6.9077553	5	55	5.36446667	295.222222	22.2222222	0	0	0	1	30.519848	1	0.21141735
0.8559	7865.07903	0.7057085	137.6737	2.8363428	0	-0.1556017	2.06255972	-0.347137	4.92489366	1.04286798	-6.9077553	6	42	5.81150556	296.333333	23.33333333	0	0	0	1	30.519848	1	0.21141735
1.5129	17089.5375	1.07284387	380.705394	5.51065523	0	0.41402834	2.83852495	0.07124461	5.94202846	1.70686498	-6.9077553	7	30	7.59966111	295.777778	22.7777778	0	0	0	1	30.519848	1	0.21141735
2.1348	29264.348	0.80164422	183.846667	2.57154239	0	0.75837297	3.37640415	-0.2198437	5.21410752	0.94489467	-6.9077553	8	28	7.59966111	296.333333	23.33333333	0	0	0	1	30.519848	1	0.21141735
2.8629	46387.762	0.78659152	187.024258	2.25419053	0	1.0518351	3.83705723	-0.2387757	5.23124368	0.81323446	-6.9077553	9	34	8.49373889	294.111111	21.11111111	0	0	0	1	30.519848	1	0.21141735

3.7485	64632.0299	0.76644829	187.907886	2.38620963	0	1.32135576	4.16872558	-0.2646842	5.23595719	0.87012516	-6.9077553	10	40	8.0467	290.777778	17.7777778	0	0	0	1	30.519848	1	0.21141735
4.5423	87769.9003	0.72917262	154.86802	1.43735329	0	1.51343349	4.47473001	-0.3144743	5.04257973	0.36349891	-6.9077553	11	47	6.70558333	288	15	0	0	0	1	30.519848	1	0.21141735
5.3694	110494.255	0.72304883	156.811735	1.57097346	0	1.68071617	4.70497258	-0.3228964	5.05505232	0.45233181	-6.9077553	12	50	7.15262222	286.888889	13.88888888	0	0	0	1	30.519848	1	0.21141735
6.0624	132933.796	0.65514146	124.823245	1.03836781	0	1.80210576	4.88985875	-0.4213789	4.82690671	0.03861265	-6.9077553	13	53	7.59966111	285.222222	12.22222222	0	0	0	1	30.519848	1	0.21141735
6.516	153737.531	0.64319489	119.367622	0.76600393	0	1.87426069	5.03525331	-0.439754	4.78221637	-0.2652634	-6.9077553	14	57	4.47038889	283.555556	10.55555555	0	0	0	1	30.519848	1	0.21141735
6.7203	167271.083	0.71098798	137.800248	0.7818137	0	1.9051328	5.11962173	-0.3396942	4.92581241	-0.2448605	-6.9077553	15	64	5.81150556	282.444444	9.444444444	0	0	0	1	30.519848	1	0.21141735
6.8904	182127.664	0.66303964	116.759569	0.67956914	0	1.93012914	5.20471338	-0.4094134	4.76012542	-0.3848259	-6.9077553	16	66	6.25854444	282.444444	9.444444444	0	0	0	1	30.519848	1	0.21141735
7.0524	195293.956	0.73525594	162.74222	0.9648663	0	1.95336798	5.27451101	-0.3061775	5.09217362	-0.0347299	-6.9077553	17	64	5.81150556	281.888889	8.888888888	0	0	0	1	30.519848	1	0.21141735
7.1874	208590.479	0.69422282	129.731146	0.69857859	0	1.97232949	5.34037769	-0.3635229	4.86547191	-0.3572771	-6.9077553	18	62	8.0467	281.888889	8.888888888	0	0	0	1	30.519848	1	0.21141735
7.3602	223528.825	0.68636958	124.277425	0.64927538	0	1.99608711	5.40954485	-0.3748832	4.82252441	-0.4303593	-6.9077553	19	63	5.81150556	281.888889	8.888888888	0	0	0	1	30.519848	1	0.21141735
7.5285	239131.879	0.67771112	119.875272	0.61157851	0	2.01869582	5.47701938	-0.3875597	4.78646014	-0.4900782	-6.9077553	20	65	5.81150556	281.888889	8.888888888	0	0	0	1	30.519848	1	0.21141735
7.6824	252248.171	0.64389283	107.167009	0.58394565	0	2.038932	5.53041737	-0.4386711	4.67439778	-0.5362363	-6.9077553	21	66	5.36446667	281.333333	8.333333333	0	0	0	1	30.519848	1	0.21141735
7.8282	264788.432	0.58162435	88.2491922	0.49221816	0	2.0577326	5.57893491	-0.5402126	4.48017587	-0.7068037	-6.9077553	22	67	4.91742778	281.333333	8.333333333	0	0	0	1	30.519848	1	0.21141735
8.6742	282340.71	0.7201303	163.876363	3.27220978	0	2.1603531	5.64311808	-0.3269354	5.09911836	1.18577109	-6.9077553	23	65	5.81150556	281.333333	8.333333333	0	0	0	1	30.519848	1	0.21141735
0.0135		0.9801274			0	-4.3050656		-0.019053			-6.9077553	0	98	2.68223333	289.666667	16.66666666	0	0	0	0	34.0724638	1	0.49277013
0.0261		0.53252254			0	-3.64582		-0.628254			-6.9077553	1	100	1.78815556	289.666667	16.66666666	0	0	0	0	34.0724638	1	0.49277013
0.0765		0.76583365			0	-2.5704645		-0.2654854			-6.9077553	2	93	3.57631111	292.444444	19.44444444	0	0	0	0	34.0724638	1	0.49277013
0.1467		0.88821626			0	-1.9193656		-0.1174148			-6.9077553	3	76	4.47038889	294.111111	21.11111111	0	0	0	0	34.0724638	1	0.49277013
0.2556		0.89634017			0	-1.3641416		-0.1083203			-6.9077553	4	63	5.36446667	294.111111	21.11111111	0	0	0	1	34.0724638	1	0.49277013
0.3942		1.10876485			0	-0.9308969		0.10414814			-6.9077553	5	55	5.36446667	295.222222	22.2222222	0	0	0	1	34.0724638	1	0.49277013
0.702		1.29815781			0	-0.3538219		0.26171622			-6.9077553	6	42	5.81150556	296.333333	23.33333333	0	0	0	1	34.0724638	1	0.49277013
1.0521		1.22753184			0	0.05078817		0.20581983			-6.9077553	7	30	7.59966111	295.777778	22.7777778	0	0	0	1	34.0724638	1	0.49277013
1.6308		1.18497316			2	0.48907069		0.17056367			0.69364706	8	28	7.59966111	296.333333	23.33333333	0	0	0	1	34.0724638	1	0.49277013
2.3013		1.09722909			2	0.83347418		0.09369896			0.69364706	9	34	8.49373889	294.111111	21.11111111	0	0	0	1	34.0724638	1	0.49277013
3.0402		1.03698805			3	1.1119233		0.03728427			1.09894557	10	40	8.0467	290.777778	17.7777778	0	0	0	1	34.0724638	1	0.49277013
3.5415		0.90872154			3	1.26455037		-0.0946167			1.09894557	11	47	6.70558333	288	15	0	0	0	1	34.0724638	1	0.49277013
4.0023		0.91276133			3	1.3868692		-0.0901859			1.09894557	12	50	7.15262222	286.888889	13.88888888	0	0	0	1	34.0724638	1	0.49277013
4.3524		0.84760322			3	1.47072742		-0.1641636			1.09894557	13	53	7.59966111	285.222222	12.22222222	0	0	0	1	34.0724638	1	0.49277013
4.6197		0.77054275			3	1.53032977		-0.2593632			1.09894557	14	57	4.47038889	283.555556	10.55555555	0	0	0	1	34.0724638	1	0.49277013
4.8411		0.83719666			3	1.57714197		-0.1765025			1.09894557	15	64	5.81150556	282.444444	9.444444444	0	0	0	1	34.0724638	1	0.49277013
5.0841		0.88215818			3	1.62611802		-0.124251			1.09894557	16	66	6.25854444	282.444444	9.444444444	0	0	0	1	34.0724638	1	0.49277013
5.3631		0.96693105			3	1.67954217		-0.0325944			1.09894557	17	64	5.81150556	281.888889	8.888888888	0	0	0	1	34.0724638	1	0.49277013
5.6304		1.00617503			3	1.72818049		0.00714941			1.09894557	18	62	8.0467	281.888889	8.888888888	0	0	0	1	34.0724638	1	0.49277013
5.9184		1.00307245			3	1.77806614		0.00406418			1.09894557	19	63	5.81150556	281.888889	8.888888888	0	0	0	1	34.0724638	1	0.49277013
6.1155		0.88308486			3	1.81082653		-0.1232022			1.09894557	20	65	5.81150556	281.888889	8.888888888	0	0	0	1	34.0724638	1	0.49277013
6.2451		0.76868174			3	1.83179716		-0.2617782			1.09894557	21	66	5.36446667	281.333333	8.333333333	0	0	0	1	34.0724638	1	0.49277013
6.4008		0.78513314			3	1.85642298		-0.2406291			1.09894557	22	67	4.91742778	281.333333	8.333333333	0	0	0	1	34.0724638	1	0.49277013
7.029		0.69473844			3	1.95004445		-0.3627815			1.09894557	23	65	5.81150556	281.333333	8.333333333	0	0	0	1	34.0724638	1	0.49277013
0.0306	1951.98486	1.12528321	349.657049	2.33117213	0	-3.4867553	0.6693589	0.11892302	5.85695567	0.84680008	-6.9077553	0	98	2.68223333	289.666667	16.66666666	0	0	0	0	42.288408	1	-0.4111751
0.108	5882.08014	0.95299885	247.995208	1.9214765	0	-2.2256241	1.77208046	-0.0470928	5.51341346	0.6536142	-6.9077553	1	100	1.78815556	289.666667	16.66666666	0	0	0	0	42.288408	1	-0.4111751
0.3276	14736.8488	1.01124548	282.865715	3.23793355	0	-1.1159619	2.69041893	0.01217111	5.64497581	1.17524412	-6.9077553	2	93	3.57631111	292.444444	19.44444444	0	0	0	0	42.288408	1	-0.4111751
0.558	24511.8034	0.92092711	224.229752	1.89723969	0	-0.5833963	3.19919557	-0.0812891	5.41267566	0.64092698	-6.9077553	3	76	4.47038889	294.111111	21.11111111	0	0	0	0	42.288408	1	-0.4111751
0.8505	35445.7576	0.88497314	206.991202	2.00728328	0	-0.1619309	3.56803178	-0.1210686	5.33268112	0.69728027	-6.9077553	4	63	5.36446667	294.111111	21.11111111	0	0	0	0	42.288408	1	-0.4111751
1.1943	49327.9111	0.85932723	197.559759	1.80017985	0	0.17756024	3.89851034	-0.1504425	5.28604618	0.58844192	-6.9077553	5	55	5.36446667	295.222222	22.2222222	0	0	0	0	42.288408	1	-0.4111751
1.7253	67984.2674	0.90088542	231.737712	2.52781258	0	0.54540095	4.21929103	-0.1032678	5.4456105	0.92774986	-6.9077553	6	42	5.81150556	296.333333	23.33333333	0	0	1	0	42.288408	1	-0.4111751
2.5047	104549.861	1.14585295	390.785725	4.0708239	0	0.91816897	4.64967366	0.13702163	5.96816195	1.40409103	-6.9077553	7	30	7.59966111	295.777778	22.7777778	0	0	1	0	42.288408	1	-0.4111751
3.447	145588.15	1.09162584	381.617001	3.78699232	0	1.23750429	4.98078861	0.08858382	5.94442011	1.33183615	-6.9077553	8	28	7.59966111	296.333333	23.33333333	0	0	1	0	42.288408	1	-0.4111751
4.1553	168098.418	0.92078567	263.328324	2.88653324	0	1.42438463	5.12455558	-0.0814425	5.57340543	1.06040259	-6.9077553	9	34	8.49373889	294.111111	21.11111111	0	0	1	0	42.288408	1	-0.4111751
4.6386	179483.922	0.72562242	154.735044	2.01265326	0	1.5344126	5.19009121	-0.3193483	5.04172072	0.69995061	-6.9077553	10	40	8.0467	290.777778	17.7777778	0	0	1	0	42.288408	1	-0.4111751
5.013	190812.815	0.69176058	143.961501	1.34615531	0	1.61203454	5.25129816	-0.3670708	4.96955286	0.29799519	-6.9077553	11	47	6.70558333	288	15	0	0	1	0	42.288408	1	-0.4111751
5.4018	204353.143	0.71727767	153.037525	1.25694557	0	1.68673223	5.31985449	-0.3308991	5.03068969	0.22947989	-6.9077553	12	50	7.15262222	286.888889	13.88888888	0	0	1	0	42.288408	1	-0.4111751
5.7105	214448.71	0.66838336	134.267538	1.12437612	0	1.74230659	5.36807526	-0.4013983	4.89984181	0.11811731	-6.9077553	13	53	7.59966111	285.222222	12.22222222	0	0	1	0	42.288408	1	-0.4111751
6.0138	225935.045	0.75940657	169.681797	1.22183852	0	1.79405683	5.42025197	-0.273902	5.13393079	0.20117482	-6.9077553	14	57	4.47038889	283.555556	10.55555555	0	0	1	0	42.288408	1	-0.4111751
6.1911	233748.821	0.80055028	169.994369	1.20756298	0	1.82311278	5.4542514	-0.2212076	5.13577119	0.18943204	-6.9077553	15	64	5.81150556	282.444444	9.444444444	0	0	1	0	42.288408	1	-0.4111751
6.3333	239553.007	0.74662385	144.5279	1.13720413	0	1.84582143	5.47877889	-0.2908553	4.97347949	0.1294517	-6.9077553	16	66	6.25854444	282.444444	9.444444444	0	0	1	0	42.288408	1	-0.4111751
6.4584	244848.478	0.73178913	141.865642	1.20732907	0	1.86538161	5.50064365	-0.3108973	4.95488747	0.18923847	-6.9077553	17	64	5.81150556	281.888889	8.888888888	0	0	1	0	42.288408	1	-0.4111751
6.5979	250817.637	0.77052937	155.948853	1.21003438	0	1.88675142	5.52473012	-0.2593805	5.0495345	0.19147485	-6.9077553	18	62	8.0467	281.888889	8.888888888	0	0	1	0	42.288408	1	-0.4111751
6.7257	256535.403	0.8161894	177.101659	1.33786827	0	1.90593601	5.54727058	-0.2018844	5.17672956	0.29182469	-6.9077553	19	63	5.81150556	281.888889	8.888888888	0	0	1	0	42.288408	1	-0.4111751
6.8499	261811.741	0.76117402	156.336362	1.2281493	0	1.92423405	5.56762952	-0.2715804	5.05201625	0.20632231	-6.9077553	20	65	5.81150556	281.888889	8.888888888	0	0	1	0	42.288408	1	-0.4111751
6.9768	266894.802	0.78049323	159.398465	1.22929761	0	1.94259036	5.58685833	-0.2465488	5.07141341	0.2072561	-6.9077553	21	66	5.36446667	281.333333	8.333333333	0	0	1	0	42.288408	1	-0.4111751
7.1055	271897.716	0.70690747	131.982818	1.10272025	0	1.96086913	5.60542963	-0.3454419	4.88267932	0.09868652	-6.9077553	22	67	4.91742778	281.333333	8.333333333	0	0	1	0	42.288408	1	-0.4111751
7.6959	280550.36	0.55616379	93.6114413	2.28500217	0	2.04068772	5.63675681	-0.584896	4.53916329	0.82680452	-6.9077553	23	65	5.81150556	281.333333	8.333333333	0	0	1	0	42.288408	1	-0.4111751

0.0018	0	0.18587095	6.69716311	0.50842071	0	-6.3199686	-6.9077553	-1.677337	1.90183333	-0.6744811	-6.9077553	0	98	2.68223333	289.666667	16.66666666	0	0	0	0	18.9135952	1	-0.0428739
0.0036	0	0.11431472	3.30488801	0.37079287	0	-5.6268214	-6.9077553	-2.1600902	1.19570512	-0.9894184	-6.9077553	1	100	1.78815556	289.666667	16.66666666	0	0	0	0	18.9135952	1	-0.0428739
0.0324	0	0.43157792	49.1256638	1.58667588	0	-3.4295969	-6.9077553	-0.8379928	3.89440194	0.46227124	-6.9077553	2	93	3.57631111	292.444444	19.44444444	0	0	0	0	18.9135952	1	-0.0428739
0.1557	219.616577	0.58348663	90.1748974	2.68724517	0	-1.8598242	-1.511329	-0.5370214	4.50176218	0.98888863	-6.9077553	3	76	4.47038889	294.111111	21.11111111	0	0	1	0	18.9135952	1	-0.0428739
0.3798	3878.52657	0.71169345	173.741529	2.8142134	0	-0.9681105	1.35571313	-0.3387039	5.15757448	1.03503807	-6.9077553	4	63	5.36446667	294.111111	21.11111111	0	0	1	0	18.9135952	1	-0.0428739
0.5877	12012.9261	0.8844987	273.899266	2.32275681	0	-0.5315387	2.48606649	-0.1216043	5.61276405	0.84318519	-6.9077553	5	55	5.36446667	295.222222	22.2222222	0	0	1	0	18.9135952	1	-0.0428739
0.7794	16392.9324	0.71945205	160.461139	1.84015494	0	-0.2492309	2.79691129	-0.3278764	5.07805802	0.61039306	-6.9077553	6	42	5.81150556	296.333333	23.33333333	0	0	1	0	18.9135952	1	-0.0428739
1.0602	21648.2881	0.78156153	197.733423	2.50350933	0	0.05845757	3.07497257	-0.2451827	5.28692483	0.91809284	-6.9077553	7	30	7.59966111	295.777778	22.7777778	0	0	1	0	18.9135952	1	-0.0428739
1.3347	29969.7598	0.78007818	193.390369	1.95846688	0	0.28870655	3.40022223	-0.24708	5.26471595	0.67267243	-6.9077553	8	28	7.59966111	296.333333	23.33333333	0	0	1	0	18.9135952	1	-0.0428739
1.6317	36321.9133	0.70637484	151.51875	2.14336233	0	0.48962242	3.59244876	-0.3461946	5.02071598	0.76284223	-6.9077553	9	34	8.49373889	294.111111	21.11111111	0	0	1	0	18.9135952	1	-0.0428739
1.9791	43843.903	0.74688618	169.278862	2.45839331	0	0.6826422	3.78065848	-0.2905045	5.13155333	0.8999147	-6.9077553	10	40	8.0467	290.777778	17.7777778	0	0	1	0	18.9135952	1	-0.0428739
2.3085	56907.6569	0.8087024	206.338029	1.80822454	0	0.83659796	4.04144747	-0.2110885	5.32952059	0.59289832	-6.9077553	11	47	6.70558333	288	15	0	0	1	0	18.9135952	1	-0.0428739
2.6244	70302.3984	0.83783098	219.126202	1.73972471	0	0.9648523	4.25282014	-0.175746	5.38965239	0.55430152	-6.9077553	12	50	7.15262222	286.888889	13.88888888	0	0	1	0	18.9135952	1	-0.0428739
2.9961	88232.8997	0.93159599	268.374187	1.96294664	0	1.09731144	4.47999124	-0.0697832	5.59238595	0.67495604	-6.9077553	13	53	7.59966111	285.222222	12.22222222	0	0	1	0	18.9135952	1	-0.0428739
3.321	103234.513	0.88199791	221.463578	1.57842324	0	1.20026594	4.63701291	-0.1244324	5.40026266	0.45705974	-6.9077553	14	57	4.47038889	283.555556	10.55555555	0	0	1	0	18.9135952	1	-0.0428739
3.6243	118663.206	0.89336832	216.135015	1.52964316	0	1.28766117	4.7762977	-0.1116376	5.37590791	0.42568802	-6.9077553	15	64	5.81150556	282.444444	9.444444444	0	0	1	0	18.9135952	1	-0.0428739
3.9042	133178.717	0.88933482	216.759144	1.55058333	0	1.3620529	4.89169947	-0.1161577	5.37879141	0.43927591	-6.9077553	16	66	6.25854444	282.444444	9.444444444	0	0	1	0	18.9135952	1	-0.0428739
4.2426	150236.732	0.8878789	212.794008	1.57971029	0	1.44517629	5.01221892	-0.1177943	5.3603293	0.4578743	-6.9077553	17	64	5.81150556	281.888889	8.888888888	0	0	1	0	18.9135952	1	-0.0428739
4.6125	170935.005	1.00398696	284.024673	2.01438707	0	1.52877001	5.14128925	0.00497457	5.64906463	0.70081127	-6.9077553	18	62	8.0467	281.888889	8.888888888	0	0	1	0	18.9135952	1	-0.0428739
4.9527	188697.859	0.94632523	253.137277	1.86256594	0	1.59993288	5.24015241	-0.0541128	5.53393589	0.62249183	-6.9077553	19	63	5.81150556	281.888889	8.888888888	0	0	1	0	18.9135952	1	-0.0428739
5.3748	213487.111	1.03071018	304.066631	2.17712741	0	1.68172136	5.36358115	0.0312178	5.71725015	0.77846552	-6.9077553	20	65	5.81150556	281.888889	8.888888888	0	0	1	0	18.9135952	1	-0.0428739
5.7276	231843.204	0.95243197	252.638094	1.86923187	0	1.74529659	5.44606561	-0.0476872	5.53196196	0.62606242	-6.9077553	21	66	5.36446667	281.333333	8.333333333	0	0	1	0	18.9135952	1	-0.0428739
6.1254	250544.448	0.85708038	218.56326	1.72524182	0	1.81244406	5.52364033	-0.1530575	5.38708007	0.54594669	-6.9077553	22	67	4.91742778	281.333333	8.333333333	0	0	1	0	18.9135952	1	-0.0428739
7.0218	269678.737	0.62541615	118.476704	2.08982904	0	1.9490196	5.5972351	-0.4677403	4.77472479	0.73756066	-6.9077553	23	65	5.81150556	281.333333	8.333333333	0	0	1	0	18.9135952	1	-0.0428739
0.0081	321.067507	0.82051444	184.763412	1.7546047	0	-4.8158912	-1.1329941	-0.1966058	5.21908157	0.56281335	-6.9077553	0	89	4.47038889	295.222222	22.2222222	0	0	0	0	30.519848	1	0.21141735
0.0117	496.522696	0.41251349	41.8881989	0.65575427	0	-4.4481664	-0.6981141	-0.8830651	3.73502801	-0.4204454	-6.9077553	1	88	4.91742778	295.222222	22.2222222	0	0	0	0	30.519848	1	0.21141735
0.0207	891.296773	0.5514111	72.1191895	0.84916449	0	-3.8776216	-0.1139565	-0.5934628	4.27833403	-0.1623254	-6.9077553	2	86	5.36446667	295.222222	22.2222222	0	0	0	0	30.519848	1	0.21141735
0.0495	1329.95157	0.51196414	84.4235229	1.19789648	0	-3.0057826	0.28589415	-0.6675493	4.43585792	0.18140153	-6.9077553	3	98	2.68223333	289.666667	16.66666666	0	0	0	0	30.519848	1	0.21141735
0.0774	1812.45321	0.39170308	44.5877607	0.9674802	0	-2.5587685	0.59523288	-0.9347015	3.79748182	-0.0320272	-6.9077553	4	100	1.78815556	289.666667	16.66666666	0	0	0	0	30.519848	1	0.21141735
0.1395	2470.98547	0.44034593	58.3364753	1.18953008	0	-1.9696907	0.90502166	-0.8179263	4.06624469	0.17439866	-6.9077553	5	93	3.57631111	292.444444	19.44444444	0	0	0	0	30.519848	1	0.21141735
0.306	3517.80744	0.59373783	104.34223	2.6860533	0	-1.1841702	1.25812214	-0.5196346	4.64768575	0.98844517	-6.9077553	6	76	4.47038889	294.111111	21.11111111	0	0	0	0	30.519848	1	0.21141735
0.4815	4507.70004	0.65358456	115.258033	2.54851465	0	-0.730849	1.50600887	-0.4237545	4.74718205	0.93590301	-6.9077553	7	63	5.36446667	294.111111	21.11111111	0	0	0	1	30.519848	1	0.21141735
0.7632	7573.32163	0.66812816	122.401857	2.45786488	0	-0.2702352	2.02476379	-0.4017797	4.80731771	0.89969981	-6.9077553	8	55	5.36446667	295.222222	22.2222222	0	0	0	1	30.519848	1	0.21141735
1.1376	18182.4089	0.77134983	162.751878	2.66994887	0	0.12892078	2.90050958	-0.2583177	5.09223296	0.98243379	-6.9077553	9	42	5.81150556	296.333333	23.33333333	0	0	0	1	30.519848	1	0.21141735
1.8369	31932.4383	1.04229053	356.352317	4.87701502	0	0.60807937	3.46365368	0.04237969	5.8759227	1.58473838	-6.9077553	10	30	7.59966111	295.777778	22.7777778	0	0	0	1	30.519848	1	0.21141735
2.5722	52820.0103	0.82575935	204.215881	2.76664728	0	0.94476156	3.96690903	-0.1902416	5.31918257	1.0179976	-6.9077553	11	28	7.59966111	296.333333	23.33333333	0	0	0	1	30.519848	1	0.21141735
3.3453	75675.0867	0.77457564	182.677714	2.19288104	0	1.20755638	4.32646222	-0.2541498	5.20772895	0.78567214	-6.9077553	12	34	8.49373889	294.111111	21.11111111	0	0	0	1	30.519848	1	0.21141735
4.4352	106327.287	0.83168207	218.006503	2.38170248	0	1.48957271	4.66653136	-0.1831034	5.38452948	0.86823534	-6.9077553	13	40	8.0467	290.777778	17.7777778	0	0	0	1	30.519848	1	0.21141735
5.4729	131593.795	0.73856986	156.698179	1.45930496	0	1.69980864	4.87972746	-0.3016865	5.05432791	0.37864529	-6.9077553	14	47	6.70558333	288	15	0	0	0	1	30.519848	1	0.21141735
6.2955	157779.489	0.7190871	149.986734	1.49395178	0	1.83983509	5.06120476	-0.3283831	5.01055352	0.40209395	-6.9077553	15	50	7.15262222	286.888889	13.88888888	0	0	0	1	30.519848	1	0.21141735
6.9336	180610.782	0.70466138	143.434657	1.10276315	0	1.93637916	5.19634988	-0.3486198	4.96588655	0.09872538	-6.9077553	16	53	7.59966111	285.222222	12.22222222	0	0	0	1	30.519848	1	0.21141735
7.3215	199711.989	0.68976269	139.857445	0.84418914	0	1.99081523	5.29688128	-0.3699589	4.9406308	-0.1681948	-6.9077553	17	57	4.47038889	283.555556	10.55555555	0	0	0	1	30.519848	1	0.21141735
7.4952	214320.301	0.71892781	137.227949	0.69652783	0	2.01426282	5.36747629	-0.3286043	4.92165069	-0.3602129	-6.9077553	18	64	5.81150556	282.444444	9.444444444	0	0	0	1	30.519848	1	0.21141735
7.6509	230732.488	0.66907559	118.216311	0.61593205	0	2.03482329	5.44126331	-0.4003648	4.77252455	-0.4829964	-6.9077553	19	66	6.25854444	282.444444	9.444444444	0	0	0	1	30.519848	1	0.21141735
7.7796	244359.256	0.64419353	106.332407	0.53228466	0	2.05150492	5.4986436	-0.438205	4.66657951	-0.6286999	-6.9077553	20	64	5.81150556	281.888889	8.888888888	0	0	0	1	30.519848	1	0.21141735
7.9155	258107.618	0.66636097	118.375556	0.66868374	0	2.06882286	5.5533805	-0.4044242	4.7738707	-0.4009497	-6.9077553	21	62	8.0467	281.888889	8.888888888	0	0	0	1	30.519848	1	0.21141735
8.0442	271211.446	0.67292535	116.616887	0.56891301	0	2.08495133	5.60290245	-0.3946359	4.75890267	-0.5622715	-6.9077553	22	63	5.81150556	281.888889	8.888888888	0	0	0	1	30.519848	1	0.21141735
8.1873	287066.38	0.65947455	112.277196	0.56931982	0	2.10258417	5.65971696	-0.4147967	4.72097969	-0.561558	-6.9077553	23	65	5.81150556	281.888889	8.888888888	0	0	0	1	30.519848	1	0.21141735
0.0171	740.961624	0.964484	261.846164	2.00085089	0	-4.0686768	-0.2984578	-0.0351257	5.56776099	0.6940722	-6.9077553	0	89	4.47038889	295.222222	22.2222222	0	0	0	0	34.0724638	1	0.49277013
0.0306	1267.32695	0.5087807	61.9535604	0.89199244	0	-3.4867553	0.23769867	-0.6737746	4.12640122	-0.1131772	-6.9077553	1	88	4.91742778	295.222222	22.2222222	0	0	0	0	34.0724638	1	0.49277013
0.0477	1793.6924	0.4880087	59.2073268	0.79136618	0	-3.0428239	0.58483364	-0.715375	4.08106219	-0.2327317	-6.9077553	2	86	5.36446667	295.222222	22.2222222	0	0	0	0	34.0724638	1	0.49277013
0.0693	2627.10957	0.5033528	66.5875854	0.86479203	0	-2.6693104	0.96626479	-0.6844793	4.19853317	-0.1441106	-6.9077553	3	98	2.68223333	289.666667	16.66666666	0	0	0	0	34.0724638	1	0.49277013
0.1071	4298.287	0.7045066	130.125	1.23899106	0	-2.2339923	1.45844919	-0.3488392	4.86850321	0.21510417	-6.9077553	4	100	1.78815556	289.666667	16.66666666	0	0	0	0	34.0724638	1	0.49277013
0.1737	7175.43624	0.80672759	173.702122	1.46267834	0	-1.7504256	1.97080291	-0.2135304	5.15734765	0.38095268	-6.9077553	5	93	3.57631111	292.444444	19.44444444	0	0	0	0	34.0724638	1	0.49277013
0.288	11744.2013	0.89787665	214.633643	2.0209329	0	-1.2447948	2.46344475	-0.1066095	5.36893725	0.70405393	-6.9077553	6	76	4.47038889	294.111111	21.11111111	0	0	0	1	34.0724638	1	0.49277013
0.5544	23551.9275	1.14236419	376.673828	3.56710568	0	-0.5898688	3.15925012	0.13397496	5.93138229	1.27203483	-6.9077553	7	63	5.36446667	294.111111	21.11111111	0	0	0	1	34.0724638	1	0.49277013
1.0278	44673.8495	1.26126598	469.362375	3.76198656	0	0.0274206	3.79941069	0.2329085	6.15137726	1.32521294	-6.9077553	8	55	5.36446667	295.222222	22.2222222	0	0	0	1	34.0724638	1	0.49277013
1.5831	59433.656	1.19135026	422.83671	3.67528664	2	0.45938495	4.08487749	0.17592637	6.04698844	1.30190318	0.69364706	9	42	5.81150556	296.333333	23.33333333	0	0	0	1	34.0724638	1	0.49277013
2.2779	90344.4096	1.20870007	430.794851	3.64465743	2	0.82325397	4.50364021	0.19037245	6.06563431	1.29353671	0.69364706	10	30	7.59966111	295.777778	22.7777778	0	0	0	1	34.0724638	1	0.49277013
3.1365	126011.789	1.16634281	405.516247	3.77512396	2	1.14310753	4.83638341	0.15473006	6.00516341	1.32869808	0.69364706	11	28	7.59966111	296.333333	23.33333333	0	0	0	1	34.0724638	1	0.49277013
3.8322	148649.212	0.97593833	281.606687	2.73755925	2	1.34343905	5.00159597	-0.0233318	5.64051492	1.00743196	0.69364706	12	34	8.49373889	294.111111	21.11111111	0	0	0	1	34.0724638	1	0.49277013
4.4451	170382.532	0.95615381	257.462326	2.6647994	2	1.49180237	5.13805197	-0.0437912	5.55087729	0.98050398	0.69364706	13	40	8.0467	290.777778	17.7777778	0	0	0	1	34.0724638	1	0.49277013

4.8816	186606.43	0.87544184	214.246956	1.77000669	2	1.58547303	5.22900711	-0.1318849	5.36713402	0.57154814	0.69364706	14	47	6.70558333	288	15	0	0	0	1	34.0724638	1	0.49277013
5.3847	207364.987	0.87321426	222.646718	1.91247318	2	1.6835616	5.33448529	-0.1344298	5.40559078	0.64892001	0.69364706	15	50	7.15262222	286.888889	13.88888888	0	0	0	1	34.0724638	1	0.49277013
5.8086	222205.899	0.81281477	190.092207	1.56693574	2	1.75933958	5.40360892	-0.2060225	5.24751452	0.44975994	0.69364706	16	53	7.59966111	285.222222	12.22222222	0	0	0	1	34.0724638	1	0.49277013
6.1515	235659.852	0.75299953	158.941252	1.15378713	2	1.81669595	5.4623937	-0.2823635	5.06854094	0.14391602	0.69364706	17	57	4.47038889	283.555556	10.55555555	0	0	0	1	34.0724638	1	0.49277013
6.4809	251279.587	0.8633176	202.057164	1.43035322	2	1.86885939	5.52657019	-0.145815	5.30855559	0.3586203	0.69364706	18	64	5.81150556	282.444444	9.444444444	0	0	0	1	34.0724638	1	0.49277013
6.7806	265086.775	0.83337302	184.466615	1.39925616	2	1.91406559	5.580061	-0.1810747	5.21747392	0.33665519	0.69364706	19	66	6.25854444	282.444444	9.444444444	0	0	0	1	34.0724638	1	0.49277013
7.1037	281279.942	0.94468056	252.414715	1.91785705	2	1.96061578	5.63935397	-0.0558504	5.53107739	0.65172972	0.69364706	20	64	5.81150556	281.888889	8.888888888	0	0	0	1	34.0724638	1	0.49277013
7.5168	304964.823	1.08395173	339.221031	2.56505683	2	2.01714052	5.72019972	0.0815355	5.82665485	0.94237041	0.69364706	21	62	8.0467	281.888889	8.888888888	0	0	0	1	34.0724638	1	0.49277013
7.803	318016.047	0.93938269	245.2344	1.83262701	2	2.05450828	5.76210499	-0.0614684	5.50221856	0.60629598	0.69364706	22	63	5.81150556	281.888889	8.888888888	0	0	0	1	34.0724638	1	0.49277013
7.9677	324872.475	0.88522472	215.654436	1.62953061	2	2.07539587	5.7834358	-0.1207847	5.37368193	0.48890549	0.69364706	23	65	5.81150556	281.888889	8.888888888	0	0	0	1	34.0724638	1	0.49277013
0.0351	2282.43874	1.14455916	362.33162	2.53197198	0	-3.3495541	0.82568252	0.13589286	5.89256263	0.92939331	-6.9077553	0	89	4.47038889	295.222222	22.2222222	0	0	0	0	42.288408	1	-0.4111751
0.0846	4651.41876	0.67850418	118.586914	1.30721616	0	-2.469821	1.53738725	-0.3863919	4.77565458	0.2686645	-6.9077553	1	88	4.91742778	295.222222	22.2222222	0	0	0	0	42.288408	1	-0.4111751
0.1485	7861.9037	0.74367464	151.118453	1.51087866	0	-1.9071703	2.06215597	-0.2948079	5.0180706	0.41335302	-6.9077553	2	86	5.36446667	295.222222	22.2222222	0	0	0	0	42.288408	1	-0.4111751
0.2961	13555.3616	0.80387767	191.966606	1.9756801	0	-1.217058	2.60685593	-0.217065	5.25732664	0.68141872	-6.9077553	3	98	2.68223333	289.666667	16.66666666	0	0	0	0	42.288408	1	-0.4111751
0.4923	22958.605	0.92469795	239.270965	1.9702252	0	-0.708667	3.13373637	-0.0772073	5.47760083	0.67865528	-6.9077553	4	100	1.78815556	289.666667	16.66666666	0	0	0	0	42.288408	1	-0.4111751
0.7218	31641.1058	0.81094139	179.783333	1.82679946	0	-0.3260072	3.4544887	-0.2083271	5.19175798	0.60311276	-6.9077553	5	93	3.57631111	292.444444	19.44444444	0	0	1	0	42.288408	1	-0.4111751
1.0224	42046.3014	0.80525637	171.284852	1.77709979	0	0.0221528	3.73879521	-0.2153535	5.14333381	0.57554526	-6.9077553	6	76	4.47038889	294.111111	21.11111111	0	0	1	0	42.288408	1	-0.4111751
1.3779	56558.5804	0.8639018	198.017801	1.77595323	0	0.3205606	4.0352946	-0.1451393	5.28836198	0.57490023	-6.9077553	7	63	5.36446667	294.111111	21.11111111	0	0	1	0	42.288408	1	-0.4111751
1.872	76314.5408	0.89145642	221.946849	2.00797393	0	0.62700738	4.3348766	-0.1137776	5.40244244	0.69762411	-6.9077553	8	55	5.36446667	295.222222	22.2222222	0	0	1	0	42.288408	1	-0.4111751
2.6478	105864.224	1.01494965	294.822397	3.2673742	0	0.97372911	4.66216682	0.01582379	5.68637652	1.18429268	-6.9077553	9	42	5.81150556	296.333333	23.33333333	0	0	1	0	42.288408	1	-0.4111751
3.5982	142829.525	1.02716897	327.108724	3.72005994	0	1.28043372	4.96165878	0.02777952	5.79029566	1.31400856	-6.9077553	10	30	7.59966111	295.777778	22.7777778	0	0	1	0	42.288408	1	-0.4111751
4.5567	177573.162	1.01516514	333.314877	3.51740711	0	1.51659868	5.17938834	0.01603588	5.80909062	1.25800836	-6.9077553	11	28	7.59966111	296.333333	23.33333333	0	0	1	0	42.288408	1	-0.4111751
5.0562	191040.482	0.79423988	199.187359	2.14038306	0	1.62061521	5.25249059	-0.2291115	5.29425091	0.76145191	-6.9077553	12	34	8.49373889	294.111111	21.11111111	0	0	1	0	42.288408	1	-0.4111751
5.5557	205795.546	0.74897488	170.324901	1.84185659	0	1.71482443	5.32688804	-0.2877156	5.13771367	0.61131686	-6.9077553	13	40	8.0467	290.777778	17.7777778	0	0	1	0	42.288408	1	-0.4111751
6.0129	219505.021	0.7107225	153.105054	1.57088933	0	1.79390716	5.39137966	-0.3400672	5.03113084	0.45227829	-6.9077553	14	47	6.70558333	288	15	0	0	1	0	42.288408	1	-0.4111751
6.5367	234403.678	0.6858833	140.622866	1.69451831	0	1.87743245	5.45704902	-0.3755909	4.94608871	0.52798848	-6.9077553	15	50	7.15262222	286.888889	13.88888888	0	0	1	0	42.288408	1	-0.4111751
6.9588	251600.495	0.77528333	203.777002	1.69017527	0	1.94000705	5.52784647	-0.2532377	5.31703118	0.52542371	-6.9077553	16	53	7.59966111	285.222222	12.22222222	0	0	1	0	42.288408	1	-0.4111751
7.2783	266200.621	0.81457193	198.901408	1.40378023	0	1.98489732	5.58425399	-0.2038657	5.29281429	0.33988087	-6.9077553	17	57	4.47038889	283.555556	10.55555555	0	0	1	0	42.288408	1	-0.4111751
7.5357	278483.625	0.89477977	224.764519	1.57908854	0	2.01965173	5.62936285	-0.1100607	5.41505772	0.45748089	-6.9077553	18	64	5.81150556	282.444444	9.444444444	0	0	1	0	42.288408	1	-0.4111751
7.7814	291023.879	0.92468899	241.53291	1.7894115	0	2.05173627	5.67340876	-0.077217	5.48700988	0.58244548	-6.9077553	19	66	6.25854444	282.444444	9.444444444	0	0	1	0	42.288408	1	-0.4111751
8.0325	303274.339	0.86273084	202.74482	1.52723885	0	2.08349581	5.7146411	-0.1464941	5.31195307	0.424116	-6.9077553	20	64	5.81150556	281.888889	8.888888888	0	0	1	0	42.288408	1	-0.4111751
8.361	320672.166	0.95724696	257.619991	1.84466001	0	2.12357804	5.77042243	-0.0426497	5.55148948	0.61283694	-6.9077553	21	62	8.0467	281.888889	8.888888888	0	0	1	0	42.288408	1	-0.4111751
8.6931	338099.111	0.90002259	224.334328	1.64387756	0	2.16252961	5.82334204	-0.1042249	5.41314193	0.49766595	-6.9077553	22	63	5.81150556	281.888889	8.888888888	0	0	1	0	42.288408	1	-0.4111751
9.0549	355757.102	0.88673651	214.585316	1.60304997	0	2.20330605	5.87425101	-0.1190803	5.36871206	0.47253166	-6.9077553	23	65	5.81150556	281.888889	8.888888888	0	0	1	0	42.288408	1	-0.4111751
0.0018	0	0.58418107	80.7339172	2.82491279	0	-6.3199686	-6.9077553	-0.535834	4.39117116	1.03883142	-6.9077553	0	89	4.47038889	295.222222	22.2222222	0	0	0	0	18.9135952	1	-0.0428739
0.0018	0	0	0	0	0	-6.3199686	-6.9077553	-6.9077553	-6.9077553	-6.9077553	-6.9077553	1	88	4.91742778	295.222222	22.2222222	0	0	0	0	18.9135952	1	-0.0428739
0.0018	0	0	0	0	0	-6.3199686	-6.9077553	-6.9077553	-6.9077553	-6.9077553	-6.9077553	2	86	5.36446667	295.222222	22.2222222	0	0	0	0	18.9135952	1	-0.0428739
0.0018	0	0	0	0	0	-6.3199686	-6.9077553	-6.9077553	-6.9077553	-6.9077553	-6.9077553	3	98	2.68223333	289.666667	16.66666666	0	0	0	0	18.9135952	1	-0.0428739
0.0027	0	0.13822962	3.51805902	0.3471278	0	-5.9145035	-6.9077553	-1.9716307	1.25819363	-1.0551856	-6.9077553	4	100	1.78815556	289.666667	16.66666666	0	0	0	0	18.9135952	1	-0.0428739
0.0198	0	0.31914322	25.190971	1.10843377	0	-3.9220733	-6.9077553	-1.1389868	3.22652533	0.10384977	-6.9077553	5	93	3.57631111	292.444444	19.44444444	0	0	0	0	18.9135952	1	-0.0428739
0.126	87.7456094	0.58064917	92.8428562	2.83196246	0	-2.0714734	-2.4219813	-0.5418878	4.53091912	1.04132297	-6.9077553	6	76	4.47038889	294.111111	21.11111111	0	0	0	0	18.9135952	1	-0.0428739
0.333	2151.77103	0.68006174	153.668359	2.99274106	0	-1.0996128	0.76675586	-0.3841023	5.03480328	1.0965238	-6.9077553	7	63	5.36446667	294.111111	21.11111111	0	0	1	0	18.9135952	1	-0.0428739
0.5256	10363.2658	0.91608807	294.097948	2.38584743	0	-0.6432148	2.33836391	-0.0865518	5.68391627	0.86997343	-6.9077553	8	55	5.36446667	295.222222	22.2222222	0	0	1	0	18.9135952	1	-0.0428739
0.7011	15010.5629	0.74015409	176.299619	1.89586839	0	-0.3551047	2.70882076	-0.2995467	5.1721906	0.64020431	-6.9077553	9	42	5.81150556	296.333333	23.33333333	0	0	1	0	18.9135952	1	-0.0428739
0.9162	18987.3383	0.72010835	171.568237	1.9496451	0	-0.0875206	2.94382502	-0.3269659	5.1449869	0.66816014	-6.9077553	10	30	7.59966111	295.777778	22.7777778	0	0	1	0	18.9135952	1	-0.0428739
1.1934	26607.3101	0.81384386	198.055309	2.06143248	0	0.17680638	3.28122357	-0.2047588	5.28855138	0.7238861	-6.9077553	11	28	7.59966111	296.333333	23.33333333	0	0	1	0	18.9135952	1	-0.0428739
1.4661	33603.6459	0.72530096	162.142288	1.79165216	0	0.38260581	3.51466433	-0.3197908	5.08848044	0.58369618	-6.9077553	12	34	8.49373889	294.111111	21.11111111	0	0	1	0	18.9135952	1	-0.0428739
1.7631	40289.4049	0.68693922	141.100639	1.95408973	0	0.56707362	3.69611335	-0.3740548	4.94948048	0.67043609	-6.9077553	13	40	8.0467	290.777778	17.7777778	0	0	1	0	18.9135952	1	-0.0428739
2.043	46682.4018	0.66950896	135.450902	1.88062662	0	0.71441932	3.84338868	-0.3997182	4.90861661	0.63213662	-6.9077553	14	47	6.70558333	288	15	0	0	1	0	18.9135952	1	-0.0428739
2.358	59988.3146	0.82076154	222.211205	1.82445801	0	0.8578138	4.09416646	-0.196305	5.40363281	0.60183092	-6.9077553	15	50	7.15262222	286.888889	13.88888888	0	0	1	0	18.9135952	1	-0.0428739
2.637	72664.6085	0.86445224	228.616003	1.65203641	0	0.96964191	4.28586821	-0.1445031	5.43204813	0.50261384	-6.9077553	16	53	7.59966111	285.222222	12.22222222	0	0	1	0	18.9135952	1	-0.0428739
2.9403	86713.3433	0.88667619	224.437222	1.57299931	0	1.07851162	4.46261931	-0.1191483	5.41360049	0.45361971	-6.9077553	17	57	4.47038889	283.555556	10.55555555	0	0	1	0	18.9135952	1	-0.0428739
3.231	101529.032	0.91779009	230.89701	1.66179948	0	1.17279169	4.62035464	-0.0846976	5.4419761	0.50850261	-6.9077553	18	64	5.81150556	282.444444	9.444444444	0	0	1	0	18.9135952	1	-0.0428739
3.5604	117690.468	0.88173922	214.313332	1.59218677	0	1.2698729	4.76806652	-0.1247255	5.36744378	0.46573627	-6.9077553	19	66	6.25854444	282.444444	9.444444444	0	0	1	0	18.9135952	1	-0.0428739
3.8853	132996.59	0.85417332	199.068927	1.43948221	0	1.3572002	4.89033101	-0.1564511	5.29365616	0.36497792	-6.9077553	20	64	5.81150556	281.888889	8.888888888	0	0	1	0	18.9135952	1	-0.0428739
4.2543	150684.755	0.92112538	235.745522	1.67495519	0	1.44793024	5.01519657	-0.0810741	5.46275717	0.51638327	-6.9077553	21	62	8.0467	281.888889	8.888888888	0	0	1	0	18.9135952	1	-0.0428739
4.6539	170275.82	0.93258818	247.698057	1.74853722	0	1.53770558	5.13742546	-0.0687199	5.51221453	0.55935131	-6.9077553	22	63	5.81150556	281.888889	8.888888888	0	0	1	0	18.9135952	1	-0.0428739
5.0589	190172.649	0.91349338	236.164635	1.70681613	0	1.62114907	5.2479376	-0.089385	5.4645334	0.53521544	-6.9077553	23	65	5.81150556	281.888889	8.888888888	0	0	1	0	18.9135952	1	-0.0428739
0.0135	400.898312	0.8010604	178.124023	1.46593898	0	-4.3050656	-0.9115562	-0.2205714	5.18248568	0.3831779	-6.9077553	0	92	1.78815556	295.777778	22.7777778	0	0	0	0	30.519848	1	0.21141735
0.0297	1014.99131	0.58564154	92.6297947	0.97449228	0	-3.5166082	0.0158648	-0.5333413	4.52862164	-0.024813	-6.9077553	1	90	2.68223333	296.888889	23.88888888	0	0	0	0	30.519848	1	0.21141735
0.0729	1629.1076	0.57156102	101.590953	1.20769445	0	-2.6186666	0.48864603	-0.557636	4.62096432	0.18954081	-6.9077553	2	83	3.12927222	298.555556	25.55555555	0	0	0	0	30.519848	1	0.21141735
0.153	2418.65576	0.46348966	61.413075	1.56465423	0	-1.8773174	0.88362528	-0.766816	4.11763904	0.44830378	-6.9077553	3	77	4.47038889	299.111111	26.11111111	0	0	0	0	30.519848	1	0.21141735

0.2547	3084.47359	0.50979564	71.0674908	1.78092551	0	-1.3676689	1.12670516	-0.6717857	4.26364407	0.57769453	-6.9077553	4	72	4.47038889	300.222222	27.2222222	0	0	0	0	30.519848	1	0.21141735
0.4077	4702.52719	0.71785907	154.419818	2.58433048	0	-0.8972237	1.54831269	-0.33009	5.03968146	0.94985335	-6.9077553	5	63	6.25854444	301.333333	28.33333333	0	0	0	1	30.519848	1	0.21141735
0.6516	14120.0986	0.85156087	218.150946	2.80769061	0	-0.4283244	2.64767004	-0.1595107	5.38519182	1.0327184	-6.9077553	6	59	6.25854444	302.444444	29.44444444	0	0	0	1	30.519848	1	0.21141735
0.9171	24055.6336	0.74677434	153.075675	2.11755681	0	-0.0865388	3.18041078	-0.290654	5.03093894	0.75073511	-6.9077553	7	55	4.91742778	304.111111	31.11111111	0	0	0	1	30.519848	1	0.21141735
1.2141	32533.0203	0.72105343	147.145573	1.86954753	0	0.19400306	3.48228632	-0.3256561	4.99142918	0.62623118	-6.9077553	8	54	4.02335	305.222222	32.2222222	0	0	0	1	30.519848	1	0.21141735
1.4301	41954.6195	0.71203156	147.219645	1.41551196	0	0.35774437	3.73661238	-0.3382296	4.99193245	0.34819748	-6.9077553	9	53	4.02335	305.777778	32.7777778	0	0	0	1	30.519848	1	0.21141735
1.6029	51034.8787	0.71347404	148.952555	1.23536666	0	0.47181449	3.93252889	-0.3362086	5.00363455	0.21217697	-6.9077553	10	59	4.47038889	304.111111	31.11111111	0	0	0	1	30.519848	1	0.21141735
1.7091	57849.1813	0.6750434	128.367858	1.02108234	0	0.53596692	4.05785659	-0.391498	4.85490782	0.02184206	-6.9077553	11	59	4.47038889	304.111111	31.11111111	0	0	0	1	30.519848	1	0.21141735
1.7874	66401.6025	0.72224141	138.285414	0.93439589	0	0.58076205	4.19573625	-0.3240122	4.929327	-0.0667854	-6.9077553	12	63	3.57631111	302.444444	29.44444444	0	0	0	1	30.519848	1	0.21141735
1.854	73620.5724	0.69981271	129.701845	0.94632742	0	0.61734547	4.29893809	-0.3555146	4.86524603	-0.0541105	-6.9077553	13	69	1.78815556	301.333333	28.33333333	0	0	0	1	30.519848	1	0.21141735
1.9224	79701.3642	0.7179335	134.81967	0.99482848	0	0.65357441	4.37829925	-0.3299864	4.90394553	-0.0041802	-6.9077553	14	69	2.68223333	300.777778	27.7777778	0	0	0	1	30.519848	1	0.21141735
1.9773	87691.8394	0.62825625	100.067487	0.75290705	0	0.68173228	4.47384025	-0.4632167	4.60585482	-0.2824862	-6.9077553	15	73	3.57631111	300.222222	27.2222222	0	0	0	1	30.519848	1	0.21141735
2.0025	88832.3034	0.5316249	70.3566589	0.75073297	0	0.6943964	4.48676162	-0.6299379	4.25359165	-0.2853741	-6.9077553	16	75	3.57631111	299.666667	26.66666666	0	0	0	1	30.519848	1	0.21141735
2.0124	89183.2111	0.26791564	15.2020541	0.39263409	0	0.69932804	4.49070402	-1.3133576	2.72149634	-0.9323335	-6.9077553	17	77	4.02335	299.111111	26.11111111	0	0	0	1	30.519848	1	0.21141735
2.025	89665.7096	0.25486883	13.6864809	0.35692569	0	0.7055697	4.49609957	-1.3630903	2.61648161	-1.0274299	-6.9077553	18	79	1.78815556	298.555556	25.55555555	0	0	0	1	30.519848	1	0.21141735
2.0349	90060.4814	0.23521458	11.370147	0.36128118	0	0.71044668	4.50049256	-1.4430146	2.43107918	-1.0153346	-6.9077553	19	80	1.78815556	298.555556	25.55555555	0	0	0	1	30.519848	1	0.21141735
2.0466	90542.9845	0.21098647	9.35761202	0.34658197	0	0.71617988	4.50583575	-1.5512328	2.23629699	-1.0567547	-6.9077553	20	83	1.34111667	298	25	0	0	0	1	30.519848	1	0.21141735
2.0565	90937.7579	0.20644994	8.62736511	0.2997155	0	0.72100551	4.51018629	-1.5728652	2.15505504	-1.2015907	-6.9077553	21	86	0.89407778	297.444444	24.44444444	0	0	0	1	30.519848	1	0.21141735
2.061	91157.0774	0.22969465	10.6641754	0.34459248	0	0.7231913	4.51259511	-1.4666603	2.3669838	-1.062495	-6.9077553	22	87	1.34111667	297.444444	24.44444444	0	0	0	1	30.519848	1	0.21141735
2.0709	91595.7113	0.1868274	7.28071941	0.29497773	0	0.7279833	4.51739537	-1.6722318	1.98536702	-1.2174711	-6.9077553	23	90	2.23519444	296.888889	23.88888888	0	0	0	1	30.519848	1	0.21141735
0.0153	704.781701	0.95532203	253.908447	1.820732	0	-4.1799025	-0.3484493	-0.0446606	5.5369777	0.5997877	-6.9077553	0	92	1.78815556	295.777778	22.7777778	0	0	0	0	34.0724638	1	0.49277013
0.0252	1011.82826	0.50981474	61.3008756	0.74019415	0	-3.6809113	0.01274667	-0.6717483	4.11581044	-0.2994927	-6.9077553	1	90	2.68223333	296.888889	23.88888888	0	0	0	0	34.0724638	1	0.49277013
0.0531	1989.54551	0.58463429	96.822116	1.2783448	0	-2.9355784	0.68840873	-0.5350598	4.57288577	0.24634807	-6.9077553	2	83	3.12927222	298.555556	25.55555555	0	0	0	0	34.0724638	1	0.49277013
0.1179	5036.7998	0.95630307	250.818495	1.95290544	0	-2.1379185	1.61696944	-0.0436353	5.52473354	0.66983016	-6.9077553	3	77	4.47038889	299.111111	26.11111111	0	0	0	0	34.0724638	1	0.49277013
0.1818	7971.30234	0.89277558	210.09701	1.39084367	0	-1.7048481	2.07597333	-0.1123006	5.34757414	0.33062925	-6.9077553	4	72	4.47038889	300.222222	27.2222222	0	0	0	0	34.0724638	1	0.49277013
0.3591	15652.0646	1.09296475	356.57178	3.20731815	0	-1.0241544	2.75066672	0.08980849	5.87653837	1.16574686	-6.9077553	5	63	6.25854444	301.333333	28.33333333	0	0	0	1	34.0724638	1	0.49277013
1.0512	33087.1009	1.57069252	791.891905	5.98360797	1	0.04993237	3.49917373	0.45215308	6.67442616	1.78919084	0.0009995	6	59	6.25854444	302.444444	29.44444444	0	0	0	1	34.0724638	1	0.49277013
1.7361	54898.9535	1.22371751	433.903663	3.7405122	2	0.55164122	4.0055125	0.20271021	6.07282484	1.31948986	0.69364706	7	55	4.91742778	304.111111	31.11111111	0	0	0	1	34.0724638	1	0.49277013
2.3238	74553.5907	1.07149675	328.885241	2.68568371	2	0.84320378	4.31153162	0.06998934	5.79571192	0.98830761	0.69364706	8	54	4.02335	305.222222	32.2222222	0	0	0	1	34.0724638	1	0.49277013
2.8674	91180.9315	0.98099715	272.943398	2.07997748	2	1.0534057	4.51285676	-0.0181669	5.6092681	0.73283772	0.69364706	9	53	4.02335	305.777778	32.7777778	0	0	0	1	34.0724638	1	0.49277013
3.447	111338.87	0.94929249	249.469235	2.44042419	2	1.23750429	4.71258742	-0.0509855	5.51933961	0.89258155	0.69364706	10	59	4.47038889	304.111111	31.11111111	0	0	0	1	34.0724638	1	0.49277013
4.0041	129975.375	0.87382367	217.381311	2.20479947	2	1.38731884	4.8673527	-0.1337329	5.3816576	0.79109002	0.69364706	11	59	4.47038889	304.111111	31.11111111	0	0	0	1	34.0724638	1	0.49277013
4.2786	140296.476	0.83913184	195.689114	1.46606405	2	1.45362585	4.94376499	-0.1741964	5.27653236	0.38326316	0.69364706	12	63	3.57631111	302.444444	29.44444444	0	0	0	1	34.0724638	1	0.49277013
4.4793	149065.197	0.86112559	196.871321	1.49726656	2	1.49946678	5.00439048	-0.1483543	5.2825554	0.40430881	0.69364706	13	69	1.78815556	301.333333	28.33333333	0	0	0	1	34.0724638	1	0.49277013
4.6368	155561.398	0.82061279	183.081116	1.49685931	2	1.53402447	5.04704692	-0.196486	5.20993477	0.40403696	0.69364706	14	69	2.68223333	300.777778	27.7777778	0	0	0	1	34.0724638	1	0.49277013
4.7547	160145.857	0.74644156	151.445551	1.41326007	2	1.5591336	5.07609125	-0.2910992	5.02023276	0.34660648	0.69364706	15	73	3.57631111	300.222222	27.2222222	0	0	0	1	34.0724638	1	0.49277013
4.8591	164358.989	0.60611001	96.5001431	1.06738702	2	1.58085324	5.10205908	-0.4990453	4.56955485	0.06615005	0.69364706	16	75	3.57631111	299.666667	26.66666666	0	0	0	1	34.0724638	1	0.49277013
4.8978	165981.946	0.29751232	20.3733804	0.48245585	2	1.58878612	5.11188505	-1.2089441	3.01427825	-0.7267953	0.69364706	17	77	4.02335	299.111111	26.11111111	0	0	0	1	34.0724638	1	0.49277013
4.9311	167429.455	0.22067802	10.6104959	0.40769487	2	1.59556209	5.12056807	-1.5065293	2.36193793	-0.8947864	0.69364706	18	79	1.78815556	298.555556	25.55555555	0	0	0	1	34.0724638	1	0.49277013
4.959	168526.044	0.23264202	14.7022705	0.36923695	2	1.60120411	5.12709624	-1.4539652	2.68806995	-0.9936121	0.69364706	19	80	1.78815556	298.555556	25.55555555	0	0	0	1	34.0724638	1	0.49277013
4.995	170192.874	0.19896395	8.40823669	0.27957468	2	1.60843741	5.13693822	-1.6096182	2.12933071	-1.2709153	0.69364706	20	83	1.34111667	298	25	0	0	0	1	34.0724638	1	0.49277013
5.0238	171245.6	0.20140454	8.50612164	0.3090089	2	1.61418662	5.14310463	-1.5974869	2.14090365	-1.1711543	0.69364706	21	86	0.89407778	297.444444	24.44444444	0	0	0	1	34.0724638	1	0.49277013
5.0481	172298.335	0.17774564	6.52285654	0.26389085	2	1.61901193	5.14923329	-1.7217915	1.87546569	-1.3284374	0.69364706	22	87	1.34111667	297.444444	24.44444444	0	0	0	1	34.0724638	1	0.49277013
5.0778	173394.925	0.17506094	6.32199559	0.24002694	2	1.6248781	5.15557756	-1.7369251	1.84419308	-1.4228466	0.69364706	23	90	2.23519444	296.888889	23.88888888	0	0	0	1	34.0724638	1	0.49277013
0.0279	1614.89307	1.04493039	297.468359	2.03259061	0	-3.5791286	0.47988779	0.04490682	5.69531123	0.709803	-6.9077553	0	92	1.78815556	295.777778	22.7777778	0	0	0	0	42.288408	1	-0.4111751
0.0783	4117.64835	0.76577044	157.186105	1.5257003	0	-2.5472077	1.41552504	-0.2655678	5.05743685	0.42310874	-6.9077553	1	90	2.68223333	296.888889	23.88888888	0	0	0	0	42.288408	1	-0.4111751
0.216	9618.88774	0.87638436	215.302185	2.6219634	0	-1.5324769	2.2638326	-0.1308101	5.3720472	0.96430475	-6.9077553	2	83	3.12927222	298.555556	25.55555555	0	0	0	0	42.288408	1	-0.4111751
0.486	22097.1172	1.03230754	293.175677	2.78133118	0	-0.7215467	3.09549241	0.03276486	5.68077542	1.02328913	-6.9077553	3	77	4.47038889	299.111111	26.11111111	0	0	0	0	42.288408	1	-0.4111751
0.7965	35549.6275	0.94283368	243.581386	2.1721938	0	-0.2275281	3.57095781	-0.0578053	5.49545523	0.77619788	-6.9077553	4	72	4.47038889	300.222222	27.2222222	0	0	0	0	42.288408	1	-0.4111751
1.1781	49638.6165	0.90059763	223.810105	2.45446403	0	0.16390297	3.90478923	-0.1035869	5.41080241	0.89831576	-6.9077553	5	63	6.25854444	301.333333	28.33333333	0	0	1	0	42.288408	1	-0.4111751
1.7586	70511.8562	0.91554523	236.707752	2.80098451	0	0.56451804	4.25579505	-0.0871439	5.46683049	1.03032792	-6.9077553	6	59	6.25854444	302.444444	29.44444444	0	0	1	0	42.288408	1	-0.4111751
2.3085	92695.1724	0.92113049	234.803601	2.16494476	0	0.83659796	4.52932718	-0.0810685	5.45875368	0.77285665	-6.9077553	7	55	4.91742778	304.111111	31.11111111	0	0	1	0	42.288408	1	-0.4111751
2.8737	119910.715	0.96400724	256.308917	1.88721442	0	1.0556004	4.78675576	-0.0356197	5.54638733	0.63563163	-6.9077553	8	54	4.02335	305.222222	32.2222222	0	0	1	0	42.288408	1	-0.4111751
3.6162	159137.566	1.05654615	318.127576	2.39894872	0	1.28542375	5.0697753	0.05595127	5.76245563	0.87544737	-6.9077553	9	53	4.02335	305.777778	32.7777778	0	0	1	0	42.288408	1	-0.4111751
4.2633	187206.411	0.95536114	259.378999	2.37469236	0	1.45004351	5.23221715	-0.0446197	5.55829416	0.86528892	-6.9077553	10	59	4.47038889	304.111111	31.11111111	0	0	1	0	42.288408	1	-0.4111751
4.8015	209937.819	0.88786964	223.622753	1.87123317	0	1.56892837	5.34681615	-0.1178047	5.40996497	0.62713193	-6.9077553	11	59	4.47038889	304.111111	31.11111111	0	0	1	0	42.288408	1	-0.4111751
5.1669	227034.183	0.87566447	212.709475	1.50614726	0	1.6422729	5.425105	-0.1316309	5.35993197	0.41021863	-6.9077553	12	63	3.57631111	302.444444	29.44444444	0	0	1	0	42.288408	1	-0.4111751
5.4189	238267.594	0.8012788	170.633956	1.30901991	0	1.68989284	5.47339858	-0.2202991	5.13952652	0.27004233	-6.9077553	13	69	1.78815556	301.333333	28.33333333	0	0	1	0	42.288408	1	-0.4111751
5.6349	247461.138	0.74944509	149.825993	1.25137075	0	1.7289794	5.51125759	-0.2870888	5.00948125	0.22503835	-6.9077553	14	69	2.68223333	300.777778	27.7777778	0	0	1	0	42.288408	1	-0.4111751
5.8302	255434.741	0.7444811	148.495932	1.27017873	0	1.76305131	5.54297088	-0.2937255	5.0005643	0.2399446	-6.9077553	15	73	3.57631111	300.222222	27.2222222	0	0	1	0	42.288408	1	-0.4111751
5.9562	260490.632	0.61115254	98.8663295	1.05448892	0	1.78443269	5.56257074	-0.4907738	4.59377885	0.05400409	-6.9077553	16	75	3.57631111	299.666667	26.66666666	0	0	1	0	42.288408	1	-0.4111751
5.9967	262025.858	0.2439028	15.1079346	0.47471873	0	1.79120932	5.56844701	-1.4068939	2.71528626	-0.7429285	-6.9077553	17	77	4.02335	299.111111	26.11111111	0	0	1	0	42.288408	1	-0.4111751

6.0363	263429.485	0.24165606	13.8006481	0.33551949	0	1.79779124	5.57378952	-1.4161102	2.62478801	-1.0890992	-6.9077553	18	79	1.78815556	298.555556	25.55555555	0	0	1	0	42.288408	1	-0.4111751
6.0723	264833.136	0.23034554	15.8522171	0.40535154	0	1.80373745	5.57910373	-1.4638429	2.76337245	-0.9005366	-6.9077553	19	80	1.78815556	298.555556	25.55555555	0	0	1	0	42.288408	1	-0.4111751
6.093	265710.411	0.19740399	8.01425105	0.27567621	0	1.80714057	5.5824108	-1.61745	2.08134611	-1.2849074	-6.9077553	20	83	1.34111667	298	25	0	0	1	0	42.288408	1	-0.4111751
6.12	266894.732	0.17726638	6.42773183	0.31645441	0	1.8115621	5.58685807	-1.7244764	1.86077729	-1.1474211	-6.9077553	21	86	0.89407778	297.444444	24.44444444	0	0	1	0	42.288408	1	-0.4111751
6.1488	268166.776	0.17908689	6.64458275	0.25128037	0	1.81625694	5.59161282	-1.7143158	1.89395239	-1.3772142	-6.9077553	22	87	1.34111667	297.444444	24.44444444	0	0	1	0	42.288408	1	-0.4111751
6.1758	269307.254	0.18236	6.98103434	0.27832467	0	1.82063843	5.59585665	-1.6963039	1.94334033	-1.2753805	-6.9077553	23	90	2.23519444	296.888889	23.88888888	0	0	1	0	42.288408	1	-0.4111751
0.0018	0	0.1506348	4.24074936	0.4177871	0	-6.3199686	-6.9077553	-1.8862803	1.44497577	-0.8703926	-6.9077553	0	92	1.78815556	295.777778	22.7777778	0	0	0	0	18.9135952	1	-0.0428739
0.0018	0	0	0	0	0	-6.3199686	-6.9077553	-6.9077553	-6.9077553	-6.9077553	-6.9077553	1	90	2.68223333	296.888889	23.88888888	0	0	0	0	18.9135952	1	-0.0428739
0.0117	0	0.34732021	32.2107516	1.03138984	0	-4.4481664	-6.9077553	-1.0546331	3.47233134	0.03187635	-6.9077553	2	83	3.12927222	298.555556	25.55555555	0	0	0	0	18.9135952	1	-0.0428739
0.0711	234.250006	0.66733019	126.565933	2.42249899	0	-2.6436679	-1.4471065	-0.4029729	4.84077128	0.88521236	-6.9077553	3	77	4.47038889	299.111111	26.11111111	0	0	0	0	18.9135952	1	-0.0428739
0.2079	1854.86266	0.67559353	137.319259	2.28328524	0	-1.5706981	0.61834964	-0.3906846	4.92231585	0.82605317	-6.9077553	4	72	4.47038889	300.222222	27.2222222	0	0	1	0	18.9135952	1	-0.0428739
0.4725	11377.3234	0.9106929	279.578417	2.9346874	0	-0.7497175	2.43171009	-0.0924521	5.63328639	1.07694163	-6.9077553	5	63	6.25854444	301.333333	28.33333333	0	0	1	0	18.9135952	1	-0.0428739
0.5418	13793.9564	0.89686436	275.031529	2.55797607	0	-0.6128583	2.62430304	-0.1077363	5.61688938	0.9396072	-6.9077553	6	59	6.25854444	302.444444	29.44444444	0	0	1	0	18.9135952	1	-0.0428739
0.5418	13793.9564	0	0	0	0	-0.6128583	2.62430304	-6.9077553	-6.9077553	-6.9077553	-6.9077553	7	55	4.91742778	304.111111	31.11111111	0	0	1	0	18.9135952	1	-0.0428739
0.5418	13793.9564	0	0	0	0	-0.6128583	2.62430304	-6.9077553	-6.9077553	-6.9077553	-6.9077553	8	54	4.02335	305.222222	32.2222222	0	0	1	0	18.9135952	1	-0.0428739
0.5418	13793.9564	0	0	0	0	-0.6128583	2.62430304	-6.9077553	-6.9077553	-6.9077553	-6.9077553	9	53	4.02335	305.777778	32.7777778	0	0	1	0	18.9135952	1	-0.0428739
0.5418	13793.9564	0	0	0	0	-0.6128583	2.62430304	-6.9077553	-6.9077553	-6.9077553	-6.9077553	10	59	4.47038889	304.111111	31.11111111	0	0	1	0	18.9135952	1	-0.0428739
0.5418	13793.9564	0	0	0	0	-0.6128583	2.62430304	-6.9077553	-6.9077553	-6.9077553	-6.9077553	11	59	4.47038889	304.111111	31.11111111	0	0	1	0	18.9135952	1	-0.0428739
0.5418	13793.9564	0	0	0	0	-0.6128583	2.62430304	-6.9077553	-6.9077553	-6.9077553	-6.9077553	12	63	3.57631111	302.444444	29.44444444	0	0	1	0	18.9135952	1	-0.0428739
0.5418	13793.9564	0	0	0	0	-0.6128583	2.62430304	-6.9077553	-6.9077553	-6.9077553	-6.9077553	13	69	1.78815556	301.333333	28.33333333	0	0	1	0	18.9135952	1	-0.0428739
0.5418	13793.9564	0	0	0	0	-0.6128583	2.62430304	-6.9077553	-6.9077553	-6.9077553	-6.9077553	14	69	2.68223333	300.777778	27.7777778	0	0	1	0	18.9135952	1	-0.0428739
0.5418	13793.9564	0	0	0	0	-0.6128583	2.62430304	-6.9077553	-6.9077553	-6.9077553	-6.9077553	15	73	3.57631111	300.222222	27.2222222	0	0	1	0	18.9135952	1	-0.0428739
0.5418	13793.9564	0	0	0	0	-0.6128583	2.62430304	-6.9077553	-6.9077553	-6.9077553	-6.9077553	16	75	3.57631111	299.666667	26.66666666	0	0	1	0	18.9135952	1	-0.0428739
0.5418	13793.9564	0	0	0	0	-0.6128583	2.62430304	-6.9077553	-6.9077553	-6.9077553	-6.9077553	17	77	4.02335	299.111111	26.11111111	0	0	1	0	18.9135952	1	-0.0428739
0.5418	13793.9564	0	0	0	0	-0.6128583	2.62430304	-6.9077553	-6.9077553	-6.9077553	-6.9077553	18	79	1.78815556	298.555556	25.55555555	0	0	1	0	18.9135952	1	-0.0428739
0.5418	13793.9564	0	0	0	0	-0.6128583	2.62430304	-6.9077553	-6.9077553	-6.9077553	-6.9077553	19	80	1.78815556	298.555556	25.55555555	0	0	1	0	18.9135952	1	-0.0428739
0.5418	13793.9564	0	0	0	0	-0.6128583	2.62430304	-6.9077553	-6.9077553	-6.9077553	-6.9077553	20	83	1.34111667	298	25	0	0	1	0	18.9135952	1	-0.0428739
0.5418	13793.9564	0	0	0	0	-0.6128583	2.62430304	-6.9077553	-6.9077553	-6.9077553	-6.9077553	21	86	0.89407778	297.444444	24.44444444	0	0	1	0	18.9135952	1	-0.0428739
0.5418	13793.9564	0	0	0	0	-0.6128583	2.62430304	-6.9077553	-6.9077553	-6.9077553	-6.9077553	22	87	1.34111667	297.444444	24.44444444	0	0	1	0	18.9135952	1	-0.0428739
0.5418	13793.9564	0	0	0	0	-0.6128583	2.62430304	-6.9077553	-6.9077553	-6.9077553	-6.9077553	23	90	2.23519444	296.888889	23.88888888	0	0	1	0	18.9135952	1	-0.0428739
0.0081	321.125007	0.83622491	189.129333	1.73853922	0	-4.8158912	-1.1328156	-0.1776625	5.24243637	0.55362026	-6.9077553	0	85	4.47038889	295.222222	22.2222222	0	0	0	0	30.519848	1	0.21141735
0.0117	496.580155	0.5198015	66.240387	0.7214312	0	-4.4481664	-0.6979986	-0.6523863	4.19330545	-0.3251331	-6.9077553	1	89	5.36446667	294.666667	21.66666666	0	0	0	0	30.519848	1	0.21141735
0.0198	847.490513	0.52272277	64.3805203	0.75607056	0	-3.9220733	-0.1642964	-0.6467928	4.16482664	-0.2782988	-6.9077553	2	90	4.47038889	294.111111	21.11111111	0	0	0	0	30.519848	1	0.21141735
0.0288	1110.67318	0.5374022	67.4713257	0.83158464	0	-3.5473799	0.10586625	-0.6191494	4.21171752	-0.1832204	-6.9077553	3	94	3.57631111	293.555556	20.55555555	0	0	0	0	30.519848	1	0.21141735
0.036	1286.12829	0.43851388	44.8067017	0.78981817	0	-3.3242363	0.25241361	-0.822086	3.80238004	-0.2346872	-6.9077553	4	97	3.12927222	293	20	0	0	0	0	30.519848	1	0.21141735
0.0369	1286.12829	0.35458195	27.2699661	0.56755817	0	-3.2995437	0.25241361	-1.0339995	3.30582262	-0.5646517	-6.9077553	5	97	2.68223333	293	20	0	0	0	0	30.519848	1	0.21141735
0.0396	1329.99202	0.36320039	31.3062134	0.65224854	0	-3.2289262	0.28592455	-1.0100511	3.44384853	-0.4257976	-6.9077553	6	93	4.47038889	293.555556	20.55555555	0	0	0	0	30.519848	1	0.21141735
0.0396	1329.99202	0	0	0	0	-3.2289262	0.28592455	-6.9077553	-6.9077553	-6.9077553	-6.9077553	7	92	2.68223333	293.555556	20.55555555	0	0	0	0	30.519848	1	0.21141735
0.0396	1329.99202	0	0	0	0	-3.2289262	0.28592455	-6.9077553	-6.9077553	-6.9077553	-6.9077553	8	91	2.68223333	294.111111	21.11111111	0	0	0	0	30.519848	1	0.21141735
0.0675	1637.03853	0.2435866	14.4747757	1.15013406	0	-2.6956277	0.49349951	-1.4081858	2.67247661	0.14074759	-6.9077553	9	91	2.68223333	294.666667	21.66666666	0	0	0	0	30.519848	1	0.21141735
0.126	2001.0507	0.35631723	35.0303523	1.25243718	0	-2.0714734	0.694172	-1.0291313	3.55624344	0.22588952	-6.9077553	10	85	3.57631111	296.333333	23.33333333	0	0	0	0	30.519848	1	0.21141735
0.2664	3133.13435	0.57312423	97.7482159	2.36400546	0	-1.3227563	1.14235301	-0.5549095	4.58240518	0.86078033	-6.9077553	11	71	5.36446667	298.555556	25.55555555	0	0	0	0	30.519848	1	0.21141735
0.3654	3796.20721	0.56796355	84.8028498	1.76334728	0	-1.0067626	1.33426585	-0.5639389	4.44034094	0.56778081	-6.9077553	12	67	5.81150556	299.666667	26.66666666	0	0	0	1	30.519848	1	0.21141735
0.5508	8204.50943	0.76174838	170.57767	2.30902292	0	-0.5963835	2.10480581	-0.2708271	5.1391966	0.83725745	-6.9077553	13	55	6.25854444	301.888889	28.88888888	0	0	0	1	30.519848	1	0.21141735
0.8451	19969.2923	0.84125577	212.514956	2.82862709	0	-0.1683003	2.99424578	-0.1716715	5.35901707	1.04014493	-6.9077553	14	50	5.81150556	303.555556	30.55555555	0	0	0	1	30.519848	1	0.21141735
1.1574	29163.7352	0.71338216	141.492908	2.13696711	0	0.14617611	3.37296028	-0.3363372	4.95225666	0.75985543	-6.9077553	15	45	5.36446667	304.111111	31.11111111	0	0	0	1	30.519848	1	0.21141735
1.4184	38035.1537	0.69600988	137.796457	1.43343937	0	0.34952948	3.63853712	-0.3609557	4.92578491	0.36077409	-6.9077553	16	46	3.57631111	304.111111	31.11111111	0	0	0	1	30.519848	1	0.21141735
1.6479	49327.7389	0.73812166	156.508241	1.43265058	0	0.49950175	3.89850685	-0.3022927	5.05311506	0.36022404	-6.9077553	17	44	4.02335	304.666667	31.66666666	0	0	0	1	30.519848	1	0.21141735
1.8207	60485.1713	0.69757183	137.349691	1.12504546	0	0.59922104	4.10241477	-0.3587173	4.92253744	0.1187119	-6.9077553	18	49	3.12927222	303	30	0	0	0	1	30.519848	1	0.21141735
1.9422	69687.8529	0.63328451	115.708384	0.85746743	0	0.66382135	4.24404038	-0.4552577	4.75108174	-0.1526065	-6.9077553	19	53	3.12927222	301.333333	28.33333333	0	0	0	1	30.519848	1	0.21141735
2.0493	79616.7797	0.63229941	114.27306	0.83107238	0	0.71749827	4.37723743	-0.456812	4.7385996	-0.1838358	-6.9077553	20	58	1.78815556	299.111111	26.11111111	0	0	0	1	30.519848	1	0.21141735
2.1672	90224.2427	0.66534622	131.539003	0.94327481	0	0.77343601	4.50230924	-0.4059459	4.87931101	-0.057338	-6.9077553	21	51	2.68223333	298.555556	25.55555555	0	0	0	1	30.519848	1	0.21141735
2.313	101190.987	0.64895135	123.258548	0.93949495	0	0.83854538	4.61701958	-0.4308578	4.81429228	-0.061349	-6.9077553	22	53	2.23519444	297.444444	24.44444444	0	0	0	1	30.519848	1	0.21141735
2.4075	111473.385	0.74178009	148.564276	0.97606027	0	0.87858886	4.71379483	-0.2973553	5.00102443	-0.0232069	-6.9077553	23	62	2.68223333	296.333333	23.33333333	0	0	0	1	30.519848	1	0.21141735
0.0162	707.980943	1.01198309	283.225787	2.12331772	0	-4.122744	-0.3439266	0.01289953	5.64624794	0.75345068	-6.9077553	0	85	4.47038889	295.222222	22.2222222	0	0	0	0	34.0724638	1	0.49277013
0.0324	1365.9378	0.48658207	60.7774862	0.93693638	0	-3.4295969	0.31257306	-0.7182967	4.10723588	-0.0640732	-6.9077553	1	89	5.36446667	294.666667	21.66666666	0	0	0	0	34.0724638	1	0.49277013
0.0468	1804.57557	0.46948627	51.3817215	0.67112076	0	-3.0618721	0.59087942	-0.7539885	3.93930196	-0.3973172	-6.9077553	2	90	4.47038889	294.111111	21.11111111	0	0	0	0	34.0724638	1	0.49277013
0.0612	2374.80467	0.51877022	65.0931473	0.82534206	0	-2.7936081	0.86533619	-0.6543685	4.17583464	-0.1907465	-6.9077553	3	94	3.57631111	293.555556	20.55555555	0	0	0	0	34.0724638	1	0.49277013
0.0801	3208.2166	0.48702621	56.3240211	0.74656209	0	-2.5244794	1.16602686	-0.7173862	4.03113886	-0.2909379	-6.9077553	4	97	3.12927222	293	20	0	0	0	0	34.0724638	1	0.49277013
0.0963	3953.90077	0.49888579	58.1019084	0.74628178	0	-2.340287	1.37495551	-0.6933756	4.06221572	-0.2913129	-6.9077553	5	97	2.68223333	293	20	0	0	0	0	34.0724638	1	0.49277013
0.1143	4831.17652	0.46115141	50.4135956	0.64205117	0	-2.1689287	1.57529699	-0.7718627	3.92028073	-0.441531	-6.9077553	6	93	4.47038889	293.555556	20.55555555	0	0	0	0	34.0724638	1	0.49277013
0.1386	6015.49896	0.45267412	48.341815	0.59457249	0	-1.9761632	1.79450552	-0.7903761	3.87831761	-0.5182322	-6.9077553	7	92	2.68223333	293.555556	20.55555555	0	0	0	0	34.0724638	1	0.49277013

0.1656	7331.41228	0.48253946	54.2346069	0.65094846	0	-1.79818	1.99230456	-0.7266223	3.99333765	-0.4277898	-6.9077553	8	91	2.68223333	294.111111	21.11111111	0	0	0	0	34.0724638	1	0.49277013
0.2187	9883.78154	0.69274275	135.256041	1.21726615	0	-1.5200544	2.29099636	-0.3656541	4.90717698	0.19742866	-6.9077553	9	91	2.68223333	294.666667	21.66666666	0	0	0	0	34.0724638	1	0.49277013
0.3411	14580.7228	0.77594028	171.052117	1.77770592	0	-1.0755796	2.67976888	-0.2523918	5.14197413	0.57588609	-6.9077553	10	85	3.57631111	296.333333	23.33333333	0	0	0	1	34.0724638	1	0.49277013
0.693	28845.7766	1.22421335	447.889506	3.92543721	0	-0.3667253	3.36199826	0.20311499	6.1045488	1.36773245	-6.9077553	11	71	5.36446667	298.555556	25.55555555	0	0	0	1	34.0724638	1	0.49277013
1.3698	46669.6267	1.3423414	540.991481	4.72208421	1	0.31466474	3.84311499	0.29516009	6.29340538	1.55246202	0.0009995	12	67	5.81150556	299.666667	26.66666666	0	0	0	1	34.0724638	1	0.49277013
2.0259	64126.6955	1.22550246	463.472779	3.92413188	1	0.70601405	4.16087634	0.20416659	6.13874981	1.36739995	0.0009995	13	55	6.25854444	301.888889	28.88888888	0	0	0	1	34.0724638	1	0.49277013
2.6001	82979.9483	1.05832914	325.794352	2.57831538	1	0.95554991	4.41861104	0.05763582	5.78626943	0.94752401	0.0009995	14	50	5.81150556	303.555556	30.55555555	0	0	0	1	34.0724638	1	0.49277013
3.1581	103045.168	1.01846097	298.218901	2.5909664	1	1.14997058	4.63517712	0.01927403	5.69783114	0.95241682	0.0009995	15	45	5.36446667	304.111111	31.11111111	0	0	0	1	34.0724638	1	0.49277013
3.7908	124795.479	0.93848993	246.441056	2.5050799	1	1.33257708	4.82668424	-0.0624182	5.5071269	0.91871974	0.0009995	16	46	3.57631111	304.111111	31.11111111	0	0	0	1	34.0724638	1	0.49277013
4.4163	143101.746	0.90036604	231.690647	2.37029669	1	1.48530224	4.96356288	-0.1038438	5.44540738	0.86343693	0.0009995	17	44	4.02335	304.666667	31.66666666	0	0	0	1	34.0724638	1	0.49277013
4.9131	155127.378	0.79876952	183.14606	1.96113609	1	1.59190511	5.04425302	-0.2234317	5.21028944	0.67403372	0.0009995	18	49	3.12927222	303	30	0	0	0	1	34.0724638	1	0.49277013
5.3019	164885.678	0.70633245	150.20147	1.29654948	1	1.66806525	5.10525844	-0.3462545	5.01198419	0.26047747	0.0009995	19	53	3.12927222	301.333333	28.33333333	0	0	0	1	34.0724638	1	0.49277013
5.5899	175244.454	0.76684313	177.955212	1.33364239	1	1.7209614	5.16618759	-0.2641698	5.18153752	0.28866338	0.0009995	20	58	1.78815556	299.111111	26.11111111	0	0	0	1	34.0724638	1	0.49277013
5.8797	184820.415	0.66820521	139.414887	1.12889601	1	1.77150574	5.21939004	-0.4016645	4.93746146	0.12212561	0.0009995	21	51	2.68223333	298.555556	25.55555555	0	0	0	1	34.0724638	1	0.49277013
6.2631	193619.8	0.58552429	110.147227	1.24437975	1	1.83467527	5.26590161	-0.5335412	4.70182698	0.2194405	0.0009995	22	53	2.23519444	297.444444	24.44444444	0	0	0	1	34.0724638	1	0.49277013
6.5079	203574.246	0.77754565	172.31329	1.3017154	1	1.87301682	5.31603569	-0.2503277	5.14932008	0.26445086	0.0009995	23	62	2.68223333	296.333333	23.33333333	0	0	0	1	34.0724638	1	0.49277013
0.0369	2386.43597	1.12565928	352.842505	2.4637764	0	-3.2995437	0.87021998	0.11925686	5.86602463	0.90210109	-6.9077553	0	85	4.47038889	295.222222	22.2222222	0	0	0	0	42.288408	1	-0.4111751
0.0828	4626.09046	0.68168214	122.077723	1.36425632	0	-2.4913272	1.53192826	-0.3817259	4.80466611	0.31134219	-6.9077553	1	89	5.36446667	294.666667	21.66666666	0	0	0	0	42.288408	1	-0.4111751
0.1305	6907.00719	0.59159225	89.1482072	1.14710876	0	-2.0363821	1.9326812	-0.5232487	4.49031145	0.13811603	-6.9077553	2	90	4.47038889	294.111111	21.11111111	0	0	0	0	42.288408	1	-0.4111751
0.1872	9626.56146	0.57919772	83.2647802	1.08315725	0	-1.6755777	2.26462997	-0.5443863	4.42203766	0.08080296	-6.9077553	3	94	3.57631111	293.555556	20.55555555	0	0	0	0	42.288408	1	-0.4111751
0.2367	11995.2064	0.5660794	78.8818182	1.03048692	0	-1.4409618	2.48459046	-0.567256	4.36796344	0.03100137	-6.9077553	4	97	3.12927222	293	20	0	0	0	0	42.288408	1	-0.4111751
0.3024	15109.5338	0.55959827	76.2803336	0.99716521	0	-1.1960046	2.7153921	-0.5787507	4.33442826	-0.0018365	-6.9077553	5	97	2.68223333	293	20	0	0	0	0	42.288408	1	-0.4111751
0.369	18223.8649	0.54696599	72.0684518	0.93556904	0	-0.9969586	2.90278687	-0.6015421	4.27763026	-0.065532	-6.9077553	6	93	4.47038889	293.555556	20.55555555	0	0	0	0	42.288408	1	-0.4111751
0.4383	21206.6016	0.51030003	64.2108931	0.84542857	0	-0.8248517	3.05435968	-0.6707987	4.16218844	-0.1667295	-6.9077553	7	92	2.68223333	293.555556	20.55555555	0	0	0	0	42.288408	1	-0.4111751
0.5193	24896.2542	0.55896886	81.5598904	0.92701094	0	-0.6552735	3.21475752	-0.5798741	4.40134986	-0.0747118	-6.9077553	8	91	2.68223333	294.111111	21.11111111	0	0	1	0	42.288408	1	-0.4111751
0.7065	31310.4974	0.63028937	118.789138	1.47320806	0	-0.3474321	3.44398536	-0.4599909	4.77735839	0.38812094	-6.9077553	9	91	2.68223333	294.666667	21.66666666	0	0	1	0	42.288408	1	-0.4111751
0.9189	38371.921	0.71334535	143.821537	1.47528542	0	-0.084578	3.64735203	-0.3363888	4.96858016	0.38952908	-6.9077553	10	85	3.57631111	296.333333	23.33333333	0	0	1	0	42.288408	1	-0.4111751
1.323	51152.4867	0.83623588	190.771071	2.27121522	0	0.27990189	3.93483066	-0.1776494	5.25107937	0.82075522	-6.9077553	11	71	5.36446667	298.555556	25.55555555	0	0	1	0	42.288408	1	-0.4111751
1.7541	66995.667	0.85134372	194.429948	1.91064644	0	0.5619559	4.20464287	-0.1597654	5.27007708	0.64796488	-6.9077553	12	67	5.81150556	299.666667	26.66666666	0	0	1	0	42.288408	1	-0.4111751
2.3715	90041.2732	0.90782179	238.235673	2.70581375	0	0.86352267	4.50027926	-0.0956063	5.47326461	0.99577221	-6.9077553	13	55	6.25854444	301.888889	28.88888888	0	0	1	0	42.288408	1	-0.4111751
3.1365	122121.059	1.02261877	298.932445	2.97717142	0	1.14310753	4.80502102	0.02334416	5.70022096	1.09130949	-6.9077553	14	50	5.81150556	303.555556	30.55555555	0	0	1	0	42.288408	1	-0.4111751
3.9312	161903.84	1.00616738	290.094883	2.18413844	0	1.36894472	5.08700875	0.00714182	5.6702115	0.78167918	-6.9077553	15	45	5.36446667	304.111111	31.11111111	0	0	1	0	42.288408	1	-0.4111751
4.7871	203966.078	1.02251267	301.934674	2.28795296	0	1.5659248	5.3179586	0.0232405	5.71021399	0.82809449	-6.9077553	16	46	3.57631111	304.111111	31.11111111	0	0	1	0	42.288408	1	-0.4111751
5.4486	228491.642	0.87685514	222.520855	1.99690722	0	1.69535869	5.43150401	-0.1302737	5.40502532	0.69210024	-6.9077553	17	44	4.02335	304.666667	31.66666666	0	0	1	0	42.288408	1	-0.4111751
5.9265	244189.391	0.78642994	175.518273	1.43820208	0	1.77943382	5.49794821	-0.2389809	5.16774886	0.36408885	-6.9077553	18	49	3.12927222	303	30	0	0	1	0	42.288408	1	-0.4111751
6.2685	254152.493	0.65927534	135.133409	1.13855928	0	1.83553709	5.53793839	-0.4150984	4.9062699	0.13064159	-6.9077553	19	53	3.12927222	301.333333	28.33333333	0	0	1	0	42.288408	1	-0.4111751
6.5331	264124.805	0.71509178	156.012543	1.13103532	0	1.87688156	5.57642552	-0.3339469	5.04994281	0.12401718	-6.9077553	20	58	1.78815556	299.111111	26.11111111	0	0	1	0	42.288408	1	-0.4111751
6.7743	273079.336	0.70833172	157.519021	1.15847425	0	1.91313604	5.60976602	-0.343432	5.05955257	0.14796667	-6.9077553	21	51	2.68223333	298.555556	25.55555555	0	0	1	0	42.288408	1	-0.4111751
7.0461	279721.753	0.53431954	101.029543	0.85584365	0	1.95247427	5.63379895	-0.6248914	4.61542287	-0.1544998	-6.9077553	22	53	2.23519444	297.444444	24.44444444	0	0	1	0	42.288408	1	-0.4111751
7.2126	286185.541	0.73651816	156.125475	1.10841114	0	1.9758295	5.65664384	-0.3044646	5.05066642	0.10382937	-6.9077553	23	62	2.68223333	296.333333	23.33333333	0	0	1	0	42.288408	1	-0.4111751
0.0018	0	0.45023254	45.8314285	1.99160612	0	-6.3199686	-6.9077553	-0.7957725	3.82499189	0.68944339	-6.9077553	0	85	4.47038889	295.222222	22.2222222	0	0	0	0	18.9135952	1	-0.0428739
0.0018	0	0	0	0	0	-6.3199686	-6.9077553	-6.9077553	-6.9077553	-6.9077553	-6.9077553	1	89	5.36446667	294.666667	21.66666666	0	0	0	0	18.9135952	1	-0.0428739
0.0018	0	0	0	0	0	-6.3199686	-6.9077553	-6.9077553	-6.9077553	-6.9077553	-6.9077553	2	90	4.47038889	294.111111	21.11111111	0	0	0	0	18.9135952	1	-0.0428739
0.0018	0	0	0	0	0	-6.3199686	-6.9077553	-6.9077553	-6.9077553	-6.9077553	-6.9077553	3	94	3.57631111	293.555556	20.55555555	0	0	0	0	18.9135952	1	-0.0428739
0.0018	0	0	0	0	0	-6.3199686	-6.9077553	-6.9077553	-6.9077553	-6.9077553	-6.9077553	4	97	3.12927222	293	20	0	0	0	0	18.9135952	1	-0.0428739
0.0018	0	0	0	0	0	-6.3199686	-6.9077553	-6.9077553	-6.9077553	-6.9077553	-6.9077553	5	97	2.68223333	293	20	0	0	0	0	18.9135952	1	-0.0428739
0.0018	0	0	0	0	0	-6.3199686	-6.9077553	-6.9077553	-6.9077553	-6.9077553	-6.9077553	6	93	4.47038889	293.555556	20.55555555	0	0	0	0	18.9135952	1	-0.0428739
0.0018	0	0	0	0	0	-6.3199686	-6.9077553	-6.9077553	-6.9077553	-6.9077553	-6.9077553	7	92	2.68223333	293.555556	20.55555555	0	0	0	0	18.9135952	1	-0.0428739
0.0018	0	0	0	0	0	-6.3199686	-6.9077553	-6.9077553	-6.9077553	-6.9077553	-6.9077553	8	91	2.68223333	294.111111	21.11111111	0	0	0	0	18.9135952	1	-0.0428739
0.0018	0	0	0	0	0	-6.3199686	-6.9077553	-6.9077553	-6.9077553	-6.9077553	-6.9077553	9	91	2.68223333	294.666667	21.66666666	0	0	0	0	18.9135952	1	-0.0428739
0.0072	0	0.27243833	21.7677333	1.03961921	0	-4.9336743	-6.9077553	-1.2966792	3.08047469	0.03981593	-6.9077553	10	85	3.57631111	296.333333	23.33333333	0	0	0	0	18.9135952	1	-0.0428739
0.0567	230.493413	0.60147053	102.206623	2.36451915	0	-2.8699811	-1.4632039	-0.5067165	4.62700626	0.86099751	-6.9077553	11	71	5.36446667	298.555556	25.55555555	0	0	0	0	18.9135952	1	-0.0428739
0.2286	2939.44146	0.76740604	187.932448	2.86393314	0	-1.4757815	1.07855973	-0.263437	5.2360879	1.05254501	-6.9077553	12	67	5.81150556	299.666667	26.66666666	0	0	1	0	18.9135952	1	-0.0428739
0.4977	12743.384	0.91714447	282.15868	2.83660542	0	-0.6977578	2.54509071	-0.0854005	5.64247315	1.04296053	-6.9077553	13	55	6.25854444	301.888889	28.88888888	0	0	1	0	18.9135952	1	-0.0428739
0.6858	17512.8181	0.78039989	214.403858	2.09159946	0	-0.3771692	2.86299017	-0.2466682	5.36786609	0.73840705	-6.9077553	14	50	5.81150556	303.555556	30.55555555	0	0	1	0	18.9135952	1	-0.0428739
0.9459	26192.268	0.86948709	238.435013	2.24624454	0	-0.0556184	3.26550243	-0.1387024	5.47410098	0.80970482	-6.9077553	15	45	5.36446667	304.111111	31.11111111	0	0	1	0	18.9135952	1	-0.0428739
1.2762	36875.7694	0.84895246	213.852861	2.30791521	0	0.24388691	3.6075818	-0.1625749	5.36529289	0.83677781	-6.9077553	16	46	3.57631111	304.111111	31.11111111	0	0	1	0	18.9135952	1	-0.0428739
1.584	49527.7861	0.90243075	240.804231	1.90039883	0	0.45995329	3.90255404	-0.1015558	5.48398844	0.64258984	-6.9077553	17	44	4.02335	304.666667	31.66666666	0	0	1	0	18.9135952	1	-0.0428739
1.8621	60536.9974	0.83073638	201.575458	1.61464318	0	0.62170488	4.10327122	-0.1842397	5.30616875	0.47973313	-6.9077553	18	49	3.12927222	303	30	0	0	1	0	18.9135952	1	-0.0428739
2.2113	75615.5525	0.89081345	233.727106	1.84164334	0	0.79358058	4.32567521	-0.1144983	5.4541585	0.61120114	-6.9077553	19	53	3.12927222	301.333333	28.33333333	0	0	1	0	18.9135952	1	-0.0428739
2.511	89553.3436	0.89487107	229.440785	1.62528281	0	0.92068108	4.49484563	-0.1099588	5.43564934	0.48629693	-6.9077553	20	58	1.78815556	299.111111	26.11111111	0	0	1	0	18.9135952	1	-0.0428739
2.7783	100180.382	0.78026686	181.450297	1.46992693	0	1.02183923	4.60698237	-0.2468385	5.20098728	0.38589277	-6.9077553	21	51	2.68223333	298.555556	25.55555555	0	0	1	0	18.9135952	1	-0.0428739

3.1041	111930.398	0.75527735	180.123101	1.58697004	0	1.13272382	4.71788617	-0.2793471	5.19364606	0.4624565	-6.9077553	22	53	2.23519444	297.444444	24.44444444	0	0	1	0	18.9135952	1	-0.0428739
3.3651	123813.054	0.87514959	218.479149	1.62458117	0	1.21345768	4.81878087	-0.1322184	5.38669516	0.4858654	-6.9077553	23	62	2.68223333	296.333333	23.33333333	0	0	1	0	18.9135952	1	-0.0428739
0.0171	422.607646	0.75695324	165.258843	2.07898309	0	-4.0686768	-0.8589476	-0.2771336	5.10751904	0.73235977	-6.9077553	0	82	4.91742778	299.666667	26.66666666	0	0	0	0	30.519848	2	0.21141735
0.0675	1036.83972	0.48306499	64.6414751	1.76098497	0	-2.6956277	0.03714136	-0.7255361	4.1688717	0.56644099	-6.9077553	1	74	5.36446667	301.333333	28.33333333	0	0	0	0	30.519848	2	0.21141735
0.1332	2003.27991	0.56826484	92.4631716	1.502439	0	-2.0159035	0.69528485	-0.5634095	4.52682123	0.40775515	-6.9077553	2	68	5.36446667	302.444444	29.44444444	0	0	0	0	30.519848	2	0.21141735
0.2205	2833.46886	0.55848757	88.6438769	1.99216957	0	-1.5118576	1.04185457	-0.580734	4.48463824	0.68972612	-6.9077553	3	63	5.81150556	303.555556	30.55555555	0	0	0	0	30.519848	2	0.21141735
0.3492	3874.41432	0.65802648	124.723885	2.13789869	0	-1.0521105	1.35465258	-0.4169916	4.8261104	0.76029107	-6.9077553	4	60	4.91742778	304.666667	31.66666666	0	0	0	1	30.519848	2	0.21141735
0.54	7360.10533	0.76782299	170.785083	2.3214084	0	-0.6161861	1.9962101	-0.2628945	5.14041179	0.84260475	-6.9077553	5	55	4.91742778	306.888889	33.88888888	0	0	0	1	30.519848	2	0.21141735
0.8379	17073.0031	0.84868057	212.139752	3.03872395	0	-0.1768565	2.83755702	-0.1628948	5.35724998	1.1117667	-6.9077553	6	51	5.81150556	307.444444	34.4444444	0	0	0	1	30.519848	2	0.21141735
1.1466	26348.481	0.70853204	135.597315	1.94976807	0	0.13680104	3.27144858	-0.3431496	4.90969695	0.66822318	-6.9077553	7	49	5.36446667	308.555556	35.5555556	0	0	0	1	30.519848	2	0.21141735
1.3914	35087.3646	0.70450171	136.909323	1.59669943	0	0.33031043	3.55786958	-0.3488461	4.91932614	0.46856474	-6.9077553	8	52	4.47038889	306.888889	33.88888888	0	0	0	1	30.519848	2	0.21141735
1.6101	44437.0374	0.69690343	135.27662	1.39585393	0	0.47629629	3.7940958	-0.3596745	4.90732911	0.33422251	-6.9077553	9	52	4.47038889	307.444444	34.4444444	0	0	0	1	30.519848	2	0.21141735
1.7973	54183.2738	0.67007505	126.955764	1.10175191	0	0.58628554	3.99239071	-0.3988743	4.84384658	0.0978088	-6.9077553	10	54	3.57631111	306.888889	33.88888888	0	0	0	1	30.519848	2	0.21141735
1.9305	63393.6334	0.65551438	123.572662	0.90681117	0	0.65777904	4.14937921	-0.4208107	4.81683743	-0.0967189	-6.9077553	11	58	2.68223333	305.777778	32.7777778	0	0	0	1	30.519848	2	0.21141735
2.0214	71640.0812	0.72931346	142.351098	0.9795073	0	0.70379034	4.27166867	-0.3142814	4.95830355	-0.0196852	-6.9077553	12	65	1.78815556	304.111111	31.11111111	0	0	0	1	30.519848	2	0.21141735
2.1051	79947.1907	0.71610383	136.496745	0.9939535	0	0.74436297	4.38137881	-0.3325346	4.91630809	-0.0050593	-6.9077553	13	70	1.78815556	303	30	0	0	0	1	30.519848	2	0.21141735
2.1618	86666.5876	0.60506681	94.4119699	0.75330855	0	0.77094121	4.46207997	-0.500765	4.54767846	-0.2819538	-6.9077553	14	77	0.44703889	301.888889	28.88888888	0	0	0	1	30.519848	2	0.21141735
2.1879	87763.1843	0.46702046	51.2386517	0.71164638	0	0.78294218	4.47465349	-0.7592433	3.93651368	-0.3387699	-6.9077553	15	75	0.89407778	297.444444	24.44444444	0	0	0	1	30.519848	2	0.21141735
2.2077	88508.8622	0.45894397	51.9962047	0.77351544	0	0.79195125	4.48311398	-0.7766506	3.95118996	-0.2555177	-6.9077553	16	88	0.89407778	295.222222	22.2222222	0	0	0	1	30.519848	2	0.21141735
2.232	89140.3512	0.31070116	33.4987386	0.68684747	0	0.80289804	4.49022333	-1.1657104	3.51153764	-0.3741882	-6.9077553	17	46	2.23519444	296.888889	23.88888888	0	0	0	1	30.519848	2	0.21141735
2.5155	99060.3435	0.63665752	133.948227	1.62738832	0	0.92247159	4.59573929	-0.4499539	4.89746082	0.48759076	-6.9077553	18	42	2.23519444	296.333333	23.33333333	0	0	0	1	30.519848	2	0.21141735
2.8899	110006.572	0.60519417	106.31297	1.93296051	0	1.0612219	4.7005492	-0.5005549	4.66639669	0.65956998	-6.9077553	19	35	3.12927222	295.777778	22.7777778	0	0	0	1	30.519848	2	0.21141735
3.4155	120265.975	0.77483854	183.048052	2.80253413	0	1.2283239	4.78971406	-0.2538108	5.20975416	1.03088081	-6.9077553	20	36	4.91742778	294.666667	21.66666666	0	0	0	1	30.519848	2	0.21141735
3.8034	134982.963	0.60145579	103.320267	1.42326861	0	1.3358954	4.90515598	-0.506741	4.63784323	0.35365843	-6.9077553	21	43	2.68223333	293	20	0	0	0	1	30.519848	2	0.21141735
3.9834	151052.608	0.54307404	88.7788867	0.64556656	0	1.38213573	5.01763479	-0.6086699	4.48616012	-0.4360791	-6.9077553	22	54	0.44703889	290.222222	17.2222222	0	0	0	1	30.519848	2	0.21141735
4.0761	162445.747	0.48010743	67.1944829	0.48368047	0	1.40514065	5.09035024	-0.7316647	4.20760603	-0.7242654	-6.9077553	23	55	1.34111667	291.333333	18.33333333	0	0	0	1	30.519848	2	0.21141735
0.0306	1098.65962	0.96922418	265.152166	2.95076867	0	-3.4867553	0.09500069	-0.0302281	5.58030764	1.08240454	-6.9077553	0	82	4.91742778	299.666667	26.66666666	0	0	0	0	34.0724638	2	0.49277013
0.0873	3428.66246	0.7863884	162.783544	1.23135679	0	-2.4384048	1.23246185	-0.2390336	5.09242751	0.20892842	-6.9077553	1	74	5.36446667	301.333333	28.33333333	0	0	0	0	34.0724638	2	0.49277013
0.1629	6939.88684	0.83632024	180.92906	1.20561727	0	-1.8146188	1.93742955	-0.1775487	5.19811055	0.1878208	-6.9077553	2	68	5.36446667	302.444444	29.44444444	0	0	0	0	34.0724638	2	0.49277013
0.2619	10831.0384	0.92371403	231.142596	2.17463212	0	-1.3397925	2.38250826	-0.0782707	5.44303914	0.77731925	-6.9077553	3	63	5.81150556	303.555556	30.55555555	0	0	0	1	34.0724638	2	0.49277013
0.5184	22965.0672	1.14124274	380.530976	3.22951275	0	-0.6570081	3.13401779	0.13299365	5.94157021	1.17264087	-6.9077553	4	60	4.91742778	304.666667	31.66666666	0	0	0	1	34.0724638	2	0.49277013
1.1538	43726.5613	1.39107144	579.864111	4.75306974	0	0.14306084	3.7779786	0.33079288	6.36279551	1.55900104	-6.9077553	5	55	4.91742778	306.888889	33.88888888	0	0	0	1	34.0724638	2	0.49277013
1.98	61018.2119	1.42032372	647.628268	5.05452261	1	0.68309684	4.11118876	0.35158864	6.47331842	1.62048123	0.0009995	6	51	5.81150556	307.444444	34.4444444	0	0	0	1	34.0724638	2	0.49277013
2.5902	82481.2645	1.08254798	341.546368	2.72039507	1	0.95173509	4.41258329	0.08024082	5.83348638	1.00114464	0.0009995	7	49	5.36446667	308.555556	35.5555556	0	0	0	1	34.0724638	2	0.49277013
3.2517	107711.359	1.01476137	290.933482	2.45694289	1	1.17917794	4.67946433	0.01563845	5.67309809	0.89932478	0.0009995	8	52	4.47038889	306.888889	33.88888888	0	0	0	1	34.0724638	2	0.49277013
3.9951	133664.595	0.99239809	276.88024	2.97618831	1	1.38506861	4.89534112	-0.0066238	5.62358868	1.09097933	0.0009995	9	52	4.47038889	307.444444	34.4444444	0	0	0	1	34.0724638	2	0.49277013
4.5531	149709.327	0.81529177	187.082157	1.84063977	1	1.51580832	5.00870227	-0.2029834	5.23155321	0.61065635	0.0009995	10	54	3.57631111	306.888889	33.88888888	0	0	0	1	34.0724638	2	0.49277013
4.9167	162025.829	0.76354165	178.055074	1.51238968	1	1.59263757	5.08776193	-0.2684788	5.18209852	0.41435196	0.0009995	11	58	2.68223333	305.777778	32.7777778	0	0	0	1	34.0724638	2	0.49277013
5.1075	169538.65	0.79244995	175.070865	1.29321764	1	1.63071005	5.13308682	-0.2313648	5.16519655	0.25790637	0.0009995	12	65	1.78815556	304.111111	31.11111111	0	0	0	1	34.0724638	2	0.49277013
5.2434	174775.016	0.75390185	151.124638	1.2106784	1	1.65697014	5.16350525	-0.2811675	5.01811153	0.19200651	0.0009995	13	70	1.78815556	303	30	0	0	0	1	34.0724638	2	0.49277013
5.3802	180710.275	0.6736917	118.07481	1.12949401	1	1.68272555	5.19690059	-0.3934994	4.77132687	0.12265472	0.0009995	14	77	0.44703889	301.888889	28.88888888	0	0	0	1	34.0724638	2	0.49277013
5.4684	184263.244	0.45516209	50.6425034	0.73537414	1	1.69898607	5.21637084	-0.7849071	3.92481096	-0.3060169	0.0009995	15	75	0.89407778	297.444444	24.44444444	0	0	0	1	34.0724638	2	0.49277013
5.5305	186895.075	0.38499395	38.1392734	0.61773101	1	1.71027823	5.23055271	-0.9519336	3.64127077	-0.4800847	0.0009995	16	88	0.89407778	295.222222	22.2222222	0	0	0	1	34.0724638	2	0.49277013
5.5845	189396.596	0.36600917	43.2032837	0.5451903	1	1.7199949	5.24384849	-1.0023684	3.76593965	-0.6047878	0.0009995	17	46	2.23519444	296.888889	23.88888888	0	0	0	1	34.0724638	2	0.49277013
6.1983	205768.334	0.65299567	137.758122	1.48810521	1	1.82427506	5.3267558	-0.4246545	4.92550667	0.39817541	0.0009995	18	42	2.23519444	296.333333	23.33333333	0	0	0	1	34.0724638	2	0.49277013
6.9066	224560.715	0.72278127	157.034	1.61714097	1	1.93247748	5.41415057	-0.323266	5.05646871	0.48127794	0.0009995	19	35	3.12927222	295.777778	22.7777778	0	0	0	1	34.0724638	2	0.49277013
7.8795	253966.657	0.84628807	211.976902	2.2946888	1	2.06426445	5.53720693	-0.1657145	5.35648203	0.83103293	0.0009995	20	36	4.91742778	294.666667	21.66666666	0	0	0	1	34.0724638	2	0.49277013
8.6013	275001.004	0.74466764	172.714698	1.67812174	1	2.15191335	5.61677839	-0.2934753	5.15164688	0.51827088	0.0009995	21	43	2.68223333	293	20	0	0	0	1	34.0724638	2	0.49277013
9.1161	289934.462	0.6812519	143.249467	1.27882086	1	2.21004208	5.66965835	-0.3823563	4.96459462	0.24672012	0.0009995	22	54	0.44703889	290.222222	17.2222222	0	0	0	1	34.0724638	2	0.49277013
9.6183	304942.453	0.68965603	147.857639	1.35008497	1	2.26366753	5.72012636	-0.3701134	4.99625667	0.30090795	0.0009995	23	55	1.34111667	291.333333	18.33333333	0	0	0	1	34.0724638	2	0.49277013
0.0477	3371.39862	1.22657416	424.560735	2.80841064	0	-3.0428239	1.21562425	0.20504	6.05105742	1.03297473	-6.9077553	0	82	4.91742778	299.666667	26.66666666	0	0	0	0	42.288408	2	-0.4111751
0.198	9945.05128	1.02042183	293.503368	3.44236746	0	-1.6194882	2.29717562	0.02119561	5.68189252	1.23644991	-6.9077553	1	74	5.36446667	301.333333	28.33333333	0	0	0	0	42.288408	2	-0.4111751
0.4752	22100.6186	1.03939027	297.619749	3.14128727	0	-0.7440195	3.09565085	0.0395959	5.69582002	1.14495097	-6.9077553	2	68	5.36446667	302.444444	29.44444444	0	0	0	0	42.288408	2	-0.4111751
0.7785	35681.8121	0.95070044	243.41084	2.17982496	0	-0.2503863	3.57466912	-0.049505	5.49475482	0.77970323	-6.9077553	3	63	5.81150556	303.555556	30.55555555	0	0	0	0	42.288408	2	-0.4111751
1.1997	51042.2562	0.91596548	230.480469	2.47167082	0	0.18207153	3.93267343	-0.0866855	5.44017046	0.90529887	-6.9077553	4	60	4.91742778	304.666667	31.66666666	0	0	0	0	42.288408	2	-0.4111751
1.7505	69874.6477	0.89906042	225.819674	2.68721477	0	0.55990146	4.2467172	-0.1052934	5.41974121	0.98887732	-6.9077553	5	55	4.91742778	306.888889	33.88888888	0	0	1	0	42.288408	2	-0.4111751
2.2356	90799.814	0.93738501	241.364332	2.00767344	0	0.80450965	4.50866825	-0.063595	5.48631169	0.69747453	-6.9077553	6	51	5.81150556	307.444444	34.4444444	0	0	1	0	42.288408	2	-0.4111751
2.7963	119042.99	0.993495	276.182584	2.02590986	0	1.02829711	4.77949308	-0.0055202	5.6210658	0.7065124	-6.9077553	7	49	5.36446667	308.555556	35.5555556	0	0	1	0	42.288408	2	-0.4111751
3.4911	154732.047	1.04578649	310.875992	2.32312138	0	1.25021687	5.04170135	0.04572498	5.73939731	0.84334207	-6.9077553	8	52	4.47038889	306.888889	33.88888888	0	0	1	0	42.288408	2	-0.4111751
4.1328	184117.814	0.97873648	274.578717	2.27180659	0	1.41895514	5.21558128	-0.0204716	5.61524163	0.82101545	-6.9077553	9	52	4.47038889	307.444444	34.4444444	0	0	1	0	42.288408	2	-0.4111751
4.644	202635.315	0.85567265	221.210607	1.902575	0	1.53557606	5.31141282	-0.1546994	5.39911974	0.64373369	-6.9077553	10	54	3.57631111	306.888889	33.88888888	0	0	1	0	42.288408	2	-0.4111751
5.0328	218208.754	0.79376235	175.749512	1.33728691	0	1.61597649	5.38545677	-0.2297121	5.16906544	0.29139037	-6.9077553	11	58	2.68223333	305.777778	32.7777778	0	0	1	0	42.288408	2	-0.4111751

5.3199	230863.266	0.83112089	188.421336	1.3346549	0	1.67145451	5.44182995	-0.1837775	5.23868591	0.28942173	-6.9077553	12	65	1.78815556	304.111111	31.11111111	0	0	1	0	42.288408	2	-0.4111751
5.5413	240402.802	0.76754643	154.444137	1.22331312	0	1.71222913	5.48232002	-0.2632543	5.03983894	0.20237997	-6.9077553	13	70	1.78815556	303	30	0	0	1	0	42.288408	2	-0.4111751
5.7393	248595.932	0.69176275	125.852663	1.15653229	0	1.74733725	5.51583284	-0.3670677	4.83511983	0.1462904	-6.9077553	14	77	0.44703889	301.888889	28.88888888	0	0	1	0	42.288408	2	-0.4111751
5.8464	253157.742	0.4760403	55.3325318	0.78013431	0	1.76582609	5.53401673	-0.7401543	4.01337909	-0.2470082	-6.9077553	15	75	0.89407778	297.444444	24.44444444	0	0	1	0	42.288408	2	-0.4111751
5.9238	256272.056	0.43251472	46.2260487	0.65092575	0	1.77897813	5.5462435	-0.8358295	3.8335651	-0.4278246	-6.9077553	16	88	0.89407778	295.222222	22.2222222	0	0	1	0	42.288408	2	-0.4111751
5.9769	258333.845	0.38453393	45.6351174	0.55467825	0	1.78790204	5.55425659	-0.9531261	3.82069945	-0.5875658	-6.9077553	17	46	2.23519444	296.888889	23.88888888	0	0	1	0	42.288408	2	-0.4111751
6.5709	269631.645	0.54609384	95.214175	1.55681522	0	1.88265081	5.59706046	-0.6031349	4.55613933	0.44328434	-6.9077553	18	42	2.23519444	296.333333	23.33333333	0	0	1	0	42.288408	2	-0.4111751
7.2108	277948.853	0.586003	106.827642	1.92148561	0	1.9755799	5.62744071	-0.5327254	4.67122607	0.65361894	-6.9077553	19	35	3.12927222	295.777778	22.7777778	0	0	1	0	42.288408	2	-0.4111751
7.992	287608.47	0.59258393	102.074435	2.4952882	0	2.07844104	5.66160355	-0.5215767	4.6257121	0.91480491	-6.9077553	20	36	4.91742778	294.666667	21.66666666	0	0	1	0	42.288408	2	-0.4111751
8.4069	296632.245	0.55589523	94.8846512	1.17370341	0	2.1290528	5.69249651	-0.5853782	4.5526725	0.1610157	-6.9077553	21	43	2.68223333	293	20	0	0	1	0	42.288408	2	-0.4111751
8.7831	307017.919	0.6522227	138.698509	1.19188745	0	2.17282942	5.72690937	-0.4258372	4.93230978	0.1763768	-6.9077553	22	54	0.44703889	290.222222	17.2222222	0	0	1	0	42.288408	2	-0.4111751
9.2016	319237.175	0.73211683	166.779822	1.38658539	0	2.21937738	5.76593746	-0.3104502	5.11668051	0.32756511	-6.9077553	23	55	1.34111667	291.333333	18.33333333	0	0	1	0	42.288408	2	-0.4111751
0.0243	0	0.68734639	131.500977	3.13520842	0	-3.7172789	-6.9077553	-0.3734631	4.87902188	1.14301456	-6.9077553	0	82	4.91742778	299.666667	26.66666666	0	0	0	0	18.9135952	2	-0.0428739
0.0936	368.920281	0.60361476	106.521484	2.17736856	0	-2.3687249	-0.9944678	-0.5031638	4.66835608	0.77857623	-6.9077553	1	74	5.36446667	301.333333	28.33333333	0	0	0	0	18.9135952	2	-0.0428739
0.2979	6181.20272	0.8224004	221.998589	2.52577835	0	-1.2109974	1.82167464	-0.1943127	5.40267553	0.92694511	-6.9077553	2	68	5.36446667	302.444444	29.44444444	0	0	1	0	18.9135952	2	-0.0428739
0.5391	13265.0885	0.80491843	219.441086	2.52917594	0	-0.6178542	2.58521104	-0.2157727	5.39108835	0.92828884	-6.9077553	3	63	5.81150556	303.555556	30.55555555	0	0	1	0	18.9135952	2	-0.0428739
0.7371	17904.8343	0.80659991	221.73473	2.17378471	0	-0.3050317	2.8851266	-0.2136885	5.40148627	0.77692967	-6.9077553	4	60	4.91742778	304.666667	31.66666666	0	0	1	0	18.9135952	2	-0.0428739
0.9981	27693.3827	0.87411809	238.211207	2.10831804	0	-0.0019018	3.3212296	-0.1333964	5.4731619	0.74636469	-6.9077553	5	55	4.91742778	306.888889	33.88888888	0	0	1	0	18.9135952	2	-0.0428739
1.3671	39999.9629	0.90109938	244.348114	2.62488031	0	0.31269171	3.68890353	-0.1030306	5.498598	0.9654162	-6.9077553	6	51	5.81150556	307.444444	34.4444444	0	0	1	0	18.9135952	2	-0.0428739
1.7073	54868.8954	0.9406417	264.414393	2.02011302	0	0.53491318	4.00496484	-0.0601304	5.57752133	0.70364836	-6.9077553	7	49	5.36446667	308.555556	35.5555556	0	0	1	0	18.9135952	2	-0.0428739
2.0664	67680.3417	0.84989466	211.637003	1.92243732	0	0.72580796	4.21481054	-0.1614669	5.35487728	0.65411386	-6.9077553	8	52	4.47038889	306.888889	33.88888888	0	0	1	0	18.9135952	2	-0.0428739
2.475	84458.3341	0.88617595	235.356983	1.92121074	0	0.9062404	4.43627017	-0.1197119	5.46110769	0.65347595	-6.9077553	9	52	4.47038889	307.444444	34.4444444	0	0	1	0	18.9135952	2	-0.0428739
2.8404	96750.3575	0.78983499	182.285137	1.6857399	0	1.04394489	4.57214436	-0.2346659	5.20557763	0.52279761	-6.9077553	10	54	3.57631111	306.888889	33.88888888	0	0	1	0	18.9135952	2	-0.0428739
3.1509	107065.24	0.73048759	161.386639	1.36345551	0	1.14768813	4.6734477	-0.312675	5.08380917	0.31075546	-6.9077553	11	58	2.68223333	305.777778	32.7777778	0	0	1	0	18.9135952	2	-0.0428739
3.3462	116170.73	0.84092944	192.874676	1.39184627	0	1.20782537	4.75506953	-0.1720591	5.26204582	0.33134933	-6.9077553	12	65	1.78815556	304.111111	31.11111111	0	0	1	0	18.9135952	2	-0.0428739
3.5352	124961.454	0.80651623	172.920499	1.3664327	0	1.26276987	4.82801333	-0.2137921	5.15283773	0.31293504	-6.9077553	13	70	1.78815556	303	30	0	0	1	0	18.9135952	2	-0.0428739
3.7089	132792.507	0.78813962	167.238838	1.45399657	0	1.31073534	4.88879535	-0.236812	5.11942894	0.37500354	-6.9077553	14	77	0.44703889	301.888889	28.88888888	0	0	1	0	18.9135952	2	-0.0428739
3.8052	136871.834	0.52822348	71.3578326	0.93568078	0	1.33636855	4.91905227	-0.6363445	4.26772113	-0.0654127	-6.9077553	15	75	0.89407778	297.444444	24.44444444	0	0	1	0	18.9135952	2	-0.0428739
3.8781	139942.301	0.47596081	56.5599923	0.83209568	0	1.35534534	4.94123735	-0.7403209	4.03531957	-0.1826068	-6.9077553	16	88	0.89407778	295.222222	22.2222222	0	0	1	0	18.9135952	2	-0.0428739
3.9339	142315.58	0.40058194	53.339194	0.63356713	0	1.3696313	4.95805401	-0.9123437	3.97669016	-0.4548122	-6.9077553	17	46	2.23519444	296.888889	23.88888888	0	0	1	0	18.9135952	2	-0.0428739
4.4028	152825.596	0.56602903	110.431307	1.41004941	0	1.4822407	5.02930392	-0.5673448	4.70440273	0.34433369	-6.9077553	18	42	2.23519444	296.333333	23.33333333	0	0	1	0	18.9135952	2	-0.0428739
4.9221	162298.085	0.59541916	111.457695	1.69693813	0	1.59373527	5.08944084	-0.5168116	4.71365407	0.52941465	-6.9077553	19	35	3.12927222	295.777778	22.7777778	0	0	1	0	18.9135952	2	-0.0428739
5.6106	179986.897	0.71021346	162.838307	2.22631373	0	1.72465767	5.19288961	-0.3407827	5.09276387	0.80079625	-6.9077553	20	36	4.91742778	294.666667	21.66666666	0	0	1	0	18.9135952	2	-0.0428739
6.0831	195049.03	0.72439709	165.108571	1.44945289	0	1.80551443	5.27325609	-0.3210361	5.10660932	0.37187585	-6.9077553	21	43	2.68223333	293	20	0	0	1	0	18.9135952	2	-0.0428739
6.4998	210201.084	0.75675599	175.54674	1.38479278	0	1.87177141	5.34806937	-0.2773939	5.16791103	0.32627238	-6.9077553	22	54	0.44703889	290.222222	17.2222222	0	0	1	0	18.9135952	2	-0.0428739
7.0101	230546.76	0.82594679	206.091986	1.58353614	0	1.94735197	5.44045804	-0.1900149	5.32832746	0.46029171	-6.9077553	23	55	1.34111667	291.333333	18.33333333	0	0	1	0	18.9135952	2	-0.0428739
0.0252	524.321343	0.70536423	143.889251	2.40590385	0	-3.6809113	-0.6437451	-0.3476243	4.96905086	0.87834121	-6.9077553	0	68	5.36446667	302.444444	29.44444444	0	0	0	0	30.519848	2	0.21141735
0.0936	1367.70841	0.54871931	79.6470048	2.06810339	0	-2.3687249	0.31386753	-0.5983475	4.37761699	0.72711537	-6.9077553	1	63	5.81150556	303.555556	30.55555555	0	0	0	0	30.519848	2	0.21141735
0.1611	2510.27313	0.62597076	110.676003	1.5095106	0	-1.82573	0.92078985	-0.4668554	4.70661607	0.41244774	-6.9077553	2	60	4.91742778	304.666667	31.66666666	0	0	0	0	30.519848	2	0.21141735
0.2502	3125.11436	0.57299666	96.4956992	1.67162207	0	-1.3854947	1.13979082	-0.5551317	4.5695088	0.5143925	-6.9077553	3	55	4.91742778	306.888889	33.88888888	0	0	0	1	30.519848	2	0.21141735
0.4518	6807.91862	0.79455934	196.499948	2.73046112	0	-0.7945157	1.91823331	-0.2287098	5.28066725	1.00483668	-6.9077553	4	51	5.81150556	307.444444	34.4444444	0	0	0	1	30.519848	2	0.21141735
0.7092	11173.0323	0.82058065	194.569247	2.79766846	0	-0.3436177	2.41359254	-0.1965252	5.27079327	1.02914375	-6.9077553	5	49	5.36446667	308.555556	35.5555556	0	0	0	1	30.519848	2	0.21141735
0.945	14965.8839	0.69989567	131.2664	1.86685588	0	-0.0565704	2.70584002	-0.3553962	4.87723647	0.62479119	-6.9077553	6	52	4.47038889	306.888889	33.88888888	0	0	0	1	30.519848	2	0.21141735
1.1898	18597.4624	0.65183909	118.749749	1.66147277	0	0.17378523	2.92307891	-0.4264246	4.77702675	0.50830611	-6.9077553	7	52	4.47038889	307.444444	34.4444444	0	0	0	1	30.519848	2	0.21141735
1.3644	23605.4741	0.69512114	143.866241	1.31044581	0	0.31071477	3.161521	-0.3622316	4.96889094	0.2711302	-6.9077553	8	54	3.57631111	306.888889	33.88888888	0	0	0	1	30.519848	2	0.21141735
1.4994	27703.7977	0.66569834	137.026589	1.05957896	0	0.40506503	3.3216056	-0.4054176	4.92018228	0.05881495	-6.9077553	9	58	2.68223333	305.777778	32.7777778	0	0	0	1	30.519848	2	0.21141735
1.5777	30635.5572	0.77258231	163.500516	1.13886384	0	0.45596809	3.42219398	-0.2567232	5.09682226	0.13090881	-6.9077553	10	65	1.78815556	304.111111	31.11111111	0	0	0	1	30.519848	2	0.21141735
1.6344	32729.7449	0.75157595	150.980624	1.10967933	0	0.49127576	3.48831485	-0.2842534	5.01715813	0.10497184	-6.9077553	11	70	1.78815556	303	30	0	0	0	1	30.519848	2	0.21141735
1.674	34844.0417	0.70393541	127.170199	1.05622777	0	0.51521597	3.55091085	-0.3496491	4.8455342	0.05565017	-6.9077553	12	77	0.44703889	301.888889	28.88888888	0	0	0	1	30.519848	2	0.21141735
1.7082	36467.0011	0.53262349	70.3416041	0.88605529	0	0.53544018	3.59643519	-0.6280648	4.25337765	-0.119848	-6.9077553	13	75	0.89407778	297.444444	24.44444444	0	0	0	1	30.519848	2	0.21141735
1.737	37739.0507	0.48382539	59.502861	0.79208648	0	0.55215949	3.63072188	-0.7239665	4.0860412	-0.231823	-6.9077553	14	88	0.89407778	295.222222	22.2222222	0	0	0	1	30.519848	2	0.21141735
1.7631	38496.8344	0.28842071	28.6837221	0.61175846	0	0.56707362	3.65060199	-1.2398739	3.35636465	-0.4897844	-6.9077553	15	46	2.23519444	296.888889	23.88888888	0	0	0	1	30.519848	2	0.21141735
1.9782	43646.2045	0.60431917	117.357299	1.52709144	0	0.68218734	3.77613924	-0.5019994	4.76523164	0.42401953	-6.9077553	16	42	2.23519444	296.333333	23.33333333	0	0	0	1	30.519848	2	0.21141735
2.223	50210.6044	0.61967289	118.016982	1.54489944	0	0.79885763	3.91624616	-0.4769511	4.770837	0.4356059	-6.9077553	17	35	3.12927222	295.777778	22.7777778	0	0	0	1	30.519848	2	0.21141735
2.7603	56427.9002	0.81909267	198.233721	3.28137759	0	1.01533937	4.03298144	-0.1983379	5.28945179	1.18856804	-6.9077553	18	36	4.91742778	294.666667	21.66666666	0	0	0	1	30.519848	2	0.21141735
2.9439	64344.7315	0.68260193	130.876685	1.12160694	0	1.07973523	4.1642706	-0.3803795	4.87426319	0.1156536	-6.9077553	19	43	2.68223333	293	20	0	0	0	1	30.519848	2	0.21141735
3.0177	72452.7172	0.52164282	83.5003096	0.64286302	0	1.10449495	4.28294798	-0.648857	4.42486232	-0.4402693	-6.9077553	20	54	0.44703889	290.222222	17.2222222	0	0	0	1	30.519848	2	0.21141735
3.0978	80559.026	0.56446491	92.4691231	1.04456749	0	1.13069218	4.38900257	-0.570107	4.5268856	0.04455979	-6.9077553	21	55	1.34111667	291.333333	18.33333333	0	0	0	1	30.519848	2	0.21141735
3.2769	88383.0114	0.68540633	144.594987	1.76818556	0	1.18689785	4.48169109	-0.3762855	4.97394355	0.57051931	-6.9077553	22	46	3.12927222	294.111111	21.11111111	0	0	0	1	30.519848	2	0.21141735
3.8952	99676.3899	0.92821111	256.860285	4.58375623	0	1.35974503	4.60193887	-0.0734193	5.54853619	1.52273694	-6.9077553	23	40	5.36446667	295.777778	22.7777778	0	0	0	1	30.519848	2	0.21141735
0.0423	1730.91255	1.09840468	342.042629	3.34736003	0	-3.1629682	0.54922632	0.09476883	5.8349383	1.20847068	-6.9077553	0	68	5.36446667	302.444444	29.44444444	0	0	0	0	34.0724638	2	0.49277013
0.1305	5774.20187	0.88677371	212.273657	1.40226932	0	-2.0363821	1.75357321	-0.1190384	5.35788099	0.33880475	-6.9077553	1	63	5.81150556	303.555556	30.55555555	0	0	0	0	34.0724638	2	0.49277013

0.2151	9290.90087	0.89508089	210.338659	1.72652679	0	-1.5366522	2.22914315	-0.1097246	5.34872365	0.54669078	-6.9077553	2	60	4.91742778	304.666667	31.66666666	0	0	0	0	34.0724638	2	0.49277013
0.3249	13800.8654	0.93295976	250.823562	2.21052676	0	-1.1242378	2.62480376	-0.0683219	5.52475374	0.79368312	-6.9077553	3	55	4.91742778	306.888889	33.88888888	0	0	0	1	34.0724638	2	0.49277013
0.5904	30134.8335	1.33247877	519.682309	4.2041417	0	-0.526955	3.40571495	0.28779115	6.25321961	1.43630799	-6.9077553	4	51	5.81150556	307.444444	34.4444444	0	0	0	1	34.0724638	2	0.49277013
1.0818	50782.6319	1.32843789	517.520719	4.28068123	0	0.07862632	3.9275741	0.28475621	6.24905149	1.45434574	-6.9077553	5	49	5.36446667	308.555556	35.5555556	0	0	0	1	34.0724638	2	0.49277013
1.5795	64009.5026	1.09844441	347.07109	2.92707458	0	0.45710834	4.15904717	0.09480497	5.84953251	1.07434507	-6.9077553	6	52	4.47038889	306.888889	33.88888888	0	0	0	1	34.0724638	2	0.49277013
2.1447	81738.112	1.04064485	308.606514	2.72906008	0	0.76299968	4.40353261	0.04080105	5.73207029	1.00432362	-6.9077553	7	52	4.47038889	307.444444	34.4444444	0	0	0	1	34.0724638	2	0.49277013
2.6901	101951.66	0.97182432	264.339986	2.26174977	0	0.98957837	4.62450859	-0.0275518	5.57723988	0.81658079	-6.9077553	8	54	3.57631111	306.888889	33.88888888	0	0	0	1	34.0724638	2	0.49277013
3.0609	115963.561	0.85440856	208.001157	1.5605525	0	1.11870899	4.75328463	-0.1561761	5.33754845	0.44568052	-6.9077553	9	58	2.68223333	305.777778	32.7777778	0	0	0	1	34.0724638	2	0.49277013
3.2814	124656.619	0.8487248	197.72229	1.38364881	0	1.18827016	4.82557093	-0.1628427	5.28686853	0.32544654	-6.9077553	10	65	1.78815556	304.111111	31.11111111	0	0	0	1	34.0724638	2	0.49277013
3.4776	133084.514	0.83422306	188.452408	1.47673301	0	1.2463424	4.89099188	-0.1800565	5.23885081	0.39050916	-6.9077553	11	70	1.78815556	303	30	0	0	0	1	34.0724638	2	0.49277013
3.6081	137889.963	0.75928387	152.209321	1.34114527	0	1.28318132	4.92646325	-0.2740634	5.02526326	0.29426928	-6.9077553	12	77	0.44703889	301.888889	28.88888888	0	0	0	1	34.0724638	2	0.49277013
3.6648	139732.24	0.48501611	61.3721478	0.91366056	0	1.29877376	4.93973517	-0.7215135	4.11697241	-0.0892023	-6.9077553	13	75	0.89407778	297.444444	24.44444444	0	0	0	1	34.0724638	2	0.49277013
3.7044	141092.012	0.45034396	59.5177335	0.82888074	0	1.3095213	4.94941933	-0.7955256	4.08629111	-0.1864733	-6.9077553	14	88	0.89407778	295.222222	22.2222222	0	0	0	1	34.0724638	2	0.49277013
3.7341	142451.784	0.3533891	37.8539225	0.43446642	0	1.31750683	4.9590106	-1.0373598	3.63376102	-0.8313376	-6.9077553	15	46	2.23519444	296.888889	23.88888888	0	0	0	1	34.0724638	2	0.49277013
4.2516	154325.282	0.60356923	116.299552	1.54173	0	1.44729538	5.03906908	-0.5032391	4.7561778	0.43355358	-6.9077553	16	42	2.23519444	296.333333	23.33333333	0	0	0	1	34.0724638	2	0.49277013
4.8177	166942.454	0.84598812	230.499329	1.89045946	0	1.57229664	5.11765516	-0.1660686	5.44025229	0.63734873	-6.9077553	17	35	3.12927222	295.777778	22.7777778	0	0	0	1	34.0724638	2	0.49277013
5.4819	185187.774	0.88095233	234.71369	2.18814393	0	1.70145176	5.2213757	-0.1256173	5.45837069	0.78351057	-6.9077553	18	36	4.91742778	294.666667	21.66666666	0	0	0	1	34.0724638	2	0.49277013
5.9877	202530.261	0.7926544	182.302435	1.50359065	0	1.78970737	5.31089425	-0.2311072	5.20567253	0.40852087	-6.9077553	19	43	2.68223333	293	20	0	0	0	1	34.0724638	2	0.49277013
6.4638	217589.364	0.74476396	166.192226	1.36718219	0	1.86621738	5.38261423	-0.2933461	5.11315112	0.31348299	-6.9077553	20	54	0.44703889	290.222222	17.2222222	0	0	0	1	34.0724638	2	0.49277013
6.9975	234311.894	0.75917648	166.626686	1.48409051	0	1.94555294	5.45665738	-0.2742047	5.1157619	0.39547572	-6.9077553	21	55	1.34111667	291.333333	18.33333333	0	0	0	1	34.0724638	2	0.49277013
7.7409	255324.171	0.77307218	172.554839	1.70825432	0	2.04651796	5.54253791	-0.2560902	5.15072089	0.5360572	-6.9077553	22	46	3.12927222	294.111111	21.11111111	0	0	0	1	34.0724638	2	0.49277013
8.8596	288497.274	0.89954841	236.864843	2.57717279	0	2.18150162	5.6646891	-0.1047514	5.46749392	0.94708093	-6.9077553	23	40	5.36446667	295.777778	22.7777778	0	0	0	1	34.0724638	2	0.49277013
0.0603	4487.21562	1.35797084	526.420928	3.4791195	0	-2.8084232	1.50145521	0.30672768	6.26610304	1.24706663	-6.9077553	0	68	5.36446667	302.444444	29.44444444	0	0	0	0	42.288408	2	-0.4111751
0.2889	15124.5795	1.15965667	380.129368	4.18973956	0	-1.2416747	2.71638732	0.14898594	5.94051427	1.43287722	-6.9077553	1	63	5.81150556	303.555556	30.55555555	0	0	0	0	42.288408	2	-0.4111751
0.5886	28900.5548	1.03367051	292.93811	2.70548906	0	-0.5300084	3.36389539	0.03408303	5.67996477	0.99565224	-6.9077553	2	60	4.91742778	304.666667	31.66666666	0	0	0	0	42.288408	2	-0.4111751
0.9207	44345.6015	0.95865422	251.249259	2.02277653	0	-0.082621	3.79203608	-0.0411822	5.52644949	0.70496534	-6.9077553	3	55	4.91742778	306.888889	33.88888888	0	0	0	0	42.288408	2	-0.4111751
1.4571	63044.4707	0.97557447	270.889524	3.32767584	0	0.37644816	4.14385622	-0.0237043	5.60171477	1.20257458	-6.9077553	4	51	5.81150556	307.444444	34.4444444	0	0	1	0	42.288408	2	-0.4111751
2.0349	86041.7517	0.94609436	253.955754	2.57076199	0	0.71044668	4.45484429	-0.0543566	5.53716399	0.94459126	-6.9077553	5	49	5.36446667	308.555556	35.5555556	0	0	1	0	42.288408	2	-0.4111751
2.4948	105773.519	0.88787913	210.19331	1.57214547	0	0.91420857	4.66130965	-0.117794	5.34803239	0.4530771	-6.9077553	6	52	4.47038889	306.888889	33.88888888	0	0	1	0	42.288408	2	-0.4111751
3.1383	140112.023	1.03918713	305.444799	2.24196829	0	1.14368125	4.94244941	0.03940063	5.72177235	0.80780012	-6.9077553	7	52	4.47038889	307.444444	34.4444444	0	0	1	0	42.288408	2	-0.4111751
3.7692	169949.306	0.97649581	269.610177	2.05085543	0	1.32686278	5.13550608	-0.0227613	5.59698084	0.71874447	-6.9077553	8	54	3.57631111	306.888889	33.88888888	0	0	1	0	42.288408	2	-0.4111751
4.2435	190839.424	0.88350242	228.426085	1.79364859	0	1.4453884	5.2514376	-0.12273	5.43121706	0.58480923	-6.9077553	9	58	2.68223333	305.777778	32.7777778	0	0	1	0	42.288408	2	-0.4111751
4.5576	206211.483	0.91293379	232.109375	1.62858306	0	1.51679617	5.32890711	-0.0899972	5.44721301	0.48832419	-6.9077553	10	65	1.78815556	304.111111	31.11111111	0	0	1	0	42.288408	2	-0.4111751
4.8276	218381.963	0.80594727	171.699453	1.33632446	0	1.57434945	5.38625023	-0.214497	5.14575141	0.29067095	-6.9077553	11	70	1.78815556	303	30	0	0	1	0	42.288408	2	-0.4111751
5.0499	227811.345	0.7136359	133.446309	1.14831778	0	1.61936844	5.42852224	-0.3359821	4.89370671	0.13916853	-6.9077553	12	77	0.44703889	301.888889	28.88888888	0	0	1	0	42.288408	2	-0.4111751
5.1759	233338.164	0.52034095	66.9677316	0.82947518	0	1.64401324	5.45249303	-0.651351	4.20422582	-0.1857572	-6.9077553	13	75	0.89407778	297.444444	24.44444444	0	0	1	0	42.288408	2	-0.4111751
5.2794	237505.23	0.49666785	61.755991	0.82735835	0	1.66381245	5.47019385	-0.6978224	4.12320718	-0.1883094	-6.9077553	14	88	0.89407778	295.222222	22.2222222	0	0	1	0	42.288408	2	-0.4111751
5.3874	240646.879	0.39433276	55.8040487	0.95307884	0	1.68406289	5.48333478	-0.9280274	4.02186434	-0.047009	-6.9077553	15	46	2.23519444	296.888889	23.88888888	0	0	1	0	42.288408	2	-0.4111751
6.0687	256313.849	0.63900217	126.443868	1.71547813	0	1.80314441	5.54640657	-0.4462837	4.83980638	0.54027459	-6.9077553	16	42	2.23519444	296.333333	23.33333333	0	0	1	0	42.288408	2	-0.4111751
6.7005	269299.126	0.62143492	116.786291	1.70409148	0	1.90218215	5.59582647	-0.4741162	4.76035426	0.53361876	-6.9077553	17	35	3.12927222	295.777778	22.7777778	0	0	1	0	42.288408	2	-0.4111751
7.5591	280808.797	0.63533855	120.824587	2.55893104	0	2.02275214	5.63767756	-0.4520245	4.79434808	0.93998032	-6.9077553	18	36	4.91742778	294.666667	21.66666666	0	0	1	0	42.288408	2	-0.4111751
7.9776	288782.011	0.53445822	91.169296	1.14432517	0	2.07663761	5.66567558	-0.6246324	4.51272914	0.13568859	-6.9077553	19	43	2.68223333	293	20	0	0	1	0	42.288408	2	-0.4111751
8.2701	296288.13	0.57964534	112.884459	1.01585449	0	2.1126466	5.69133577	-0.5436151	4.72637367	0.01671403	-6.9077553	20	54	0.44703889	290.222222	17.2222222	0	0	1	0	42.288408	2	-0.4111751
8.559	303121.715	0.61187274	124.043723	1.09799546	0	2.14698336	5.71413772	-0.489598	4.82064217	0.09439655	-6.9077553	21	55	1.34111667	291.333333	18.33333333	0	0	1	0	42.288408	2	-0.4111751
9.0855	310742.215	0.60557527	106.215705	1.57673329	0	2.20667974	5.7389669	-0.4999264	4.6654814	0.45598919	-6.9077553	22	46	3.12927222	294.111111	21.11111111	0	0	1	0	42.288408	2	-0.4111751
10.2348	333009.084	0.84106656	224.813724	3.35925379	0	2.32579368	5.80817277	-0.1718962	5.41527661	1.2120165	-6.9077553	23	40	5.36446667	295.777778	22.7777778	0	0	1	0	42.288408	2	-0.4111751
0.0459	123.586807	0.77183388	175.50125	3.81571289	0	-3.0812902	-2.0827526	-0.2576912	5.16765186	1.33938955	-6.9077553	0	68	5.36446667	302.444444	29.44444444	0	0	0	0	18.9135952	2	-0.0428739
0.2178	3166.30547	0.79634821	208.593055	3.00717499	0	-1.5241781	1.15288122	-0.2264638	5.34039004	1.10133358	-6.9077553	1	63	5.81150556	303.555556	30.55555555	0	0	0	0	18.9135952	2	-0.0428739
0.4743	11889.3771	0.87863202	254.509677	2.51085098	0	-0.7459152	2.47572943	-0.1282516	5.53934279	0.92101992	-6.9077553	2	60	4.91742778	304.666667	31.66666666	0	0	1	0	18.9135952	2	-0.0428739
0.6561	16524.534	0.77365218	205.171179	1.83142105	0	-0.4214421	2.8049067	-0.2553411	5.32384952	0.60563807	-6.9077553	3	55	4.91742778	306.888889	33.88888888	0	0	1	0	18.9135952	2	-0.0428739
0.927	25668.4152	0.89463325	254.23126	2.3384586	0	-0.0758017	3.24530021	-0.1102243	5.53824826	0.84991953	-6.9077553	4	51	5.81150556	307.444444	34.4444444	0	0	1	0	18.9135952	2	-0.0428739
1.26	36578.046	0.88373364	235.44603	2.43882694	0	0.23111172	3.59947556	-0.1224687	5.46148596	0.89192711	-6.9077553	5	49	5.36446667	308.555556	35.5555556	0	0	1	0	18.9135952	2	-0.0428739
1.5894	49408.1668	0.89308408	237.960532	1.9762133	0	0.46335659	3.90013597	-0.1119555	5.47210903	0.68168843	-6.9077553	6	52	4.47038889	306.888889	33.88888888	0	0	1	0	18.9135952	2	-0.0428739
1.8729	60542.5968	0.85365959	212.428199	1.71134169	0	0.62748803	4.10336371	-0.157052	5.35860875	0.53786184	-6.9077553	7	52	4.47038889	307.444444	34.4444444	0	0	1	0	18.9135952	2	-0.0428739
2.2077	73688.0406	0.85412146	217.392053	1.80006359	0	0.79195125	4.29985408	-0.1565118	5.38170702	0.58837738	-6.9077553	8	54	3.57631111	306.888889	33.88888888	0	0	1	0	18.9135952	2	-0.0428739
2.5254	87612.4198	0.88261833	229.643635	1.69586303	0	0.92639947	4.47293418	-0.1237301	5.43653305	0.52878127	-6.9077553	9	58	2.68223333	305.777778	32.7777778	0	0	1	0	18.9135952	2	-0.0428739
2.718	96423.2259	0.83851253	192.506936	1.36851202	0	0.99989632	4.56875748	-0.1749339	5.26013738	0.31445449	-6.9077553	10	65	1.78815556	304.111111	31.11111111	0	0	1	0	18.9135952	2	-0.0428739
2.8926	104219.036	0.85666413	198.140706	1.53646756	0	1.06215575	4.6465044	-0.1535427	5.28898246	0.43013662	-6.9077553	11	70	1.78815556	303	30	0	0	1	0	18.9135952	2	-0.0428739
3.0159	109888.403	0.74495853	145.831062	1.30772812	0	1.10389829	4.69947444	-0.2930853	4.9824557	0.26905576	-6.9077553	12	77	0.44703889	301.888889	28.88888888	0	0	1	0	18.9135952	2	-0.0428739
3.1023	113660.684	0.53416506	71.6839396	0.94529359	0	1.13214377	4.73322635	-0.62518	4.27228068	-0.0552024	-6.9077553	13	75	0.89407778	297.444444	24.44444444	0	0	1	0	18.9135952	2	-0.0428739
3.1923	117608.42	0.50193703	63.8414014	0.90256912	0	1.16074166	4.76736913	-0.6872903	4.15641757	-0.1014027	-6.9077553	14	88	0.89407778	295.222222	22.2222222	0	0	1	0	18.9135952	2	-0.0428739
3.2715	121340.122	0.4873372	77.2266346	0.79388766	0	1.1852486	4.79860577	-0.7167491	4.34675735	-0.2295545	-6.9077553	15	46	2.23519444	296.888889	23.88888888	0	0	1	0	18.9135952	2	-0.0428739

3.8412	136066.622	0.66533595	149.177972	1.59862491	0	1.34578482	4.91315199	-0.4059613	5.00514674	0.46976917	-6.9077553	16	42	2.23519444	296.333333	23.33333333	0	0	1	0	18.9135952	2	-0.0428739
4.3461	146301.479	0.62825292	128.416193	1.63857309	0	1.46927889	4.98567625	-0.463222	4.85528429	0.4944359	-6.9077553	17	35	3.12927222	295.777778	22.7777778	0	0	1	0	18.9135952	2	-0.0428739
5.0121	160249.543	0.66965777	141.705384	2.33110995	0	1.61185499	5.07673848	-0.3994963	4.9537572	0.84677342	-6.9077553	18	36	4.91742778	294.666667	21.66666666	0	0	1	0	18.9135952	2	-0.0428739
5.4711	175751.297	0.72517072	169.949357	1.5089079	0	1.69947969	5.1690756	-0.3199701	5.13550637	0.41204866	-6.9077553	19	43	2.68223333	293	20	0	0	1	0	18.9135952	2	-0.0428739
5.8743	189915.496	0.72952727	171.1122	1.40126841	0	1.7705869	5.24658448	-0.3139887	5.14232533	0.33809122	-6.9077553	20	54	0.44703889	290.222222	17.2222222	0	0	1	0	18.9135952	2	-0.0428739
6.2496	202531.582	0.68818602	153.290833	1.30834258	0	1.83251746	5.31090077	-0.3722441	5.03234351	0.26952517	-6.9077553	21	55	1.34111667	291.333333	18.33333333	0	0	1	0	18.9135952	2	-0.0428739
6.9129	222447.158	0.75160762	175.382304	1.85449285	0	1.93338923	5.40469408	-0.2842113	5.16697389	0.61815035	-6.9077553	22	46	3.12927222	294.111111	21.11111111	0	0	1	0	18.9135952	2	-0.0428739
8.0397	257920.268	0.90317504	243.783971	3.24209214	0	2.08439177	5.55265438	-0.1007323	5.49628657	1.17652724	-6.9077553	23	40	5.36446667	295.777778	22.7777778	0	0	1	0	18.9135952	2	-0.0428739
0.0081	309.620283	0.8985076	221.017792	1.56625617	0	-4.8158912	-1.1691841	-0.1059078	5.39824773	0.44932643	-6.9077553	0	90	1.78815556	297.444444	24.44444444	0	0	0	0	30.519848	2	0.21141735
0.0153	660.530545	0.5665077	77.5697784	0.79501122	0	-4.1799025	-0.4131991	-0.566501	4.35119079	-0.228142	-6.9077553	1	90	2.23519444	296.888889	23.88888888	0	0	0	0	30.519848	2	0.21141735
0.0288	1011.44087	0.61315149	97.6442139	1.09287885	0	-3.5473799	0.01236412	-0.4875137	4.58134064	0.08972996	-6.9077553	2	84	1.78815556	296.888889	23.88888888	0	0	0	0	30.519848	2	0.21141735
0.0387	1274.62349	0.4636771	60.2219738	1.01628988	0	-3.2519157	0.24343507	-0.7664125	4.0980539	0.01714212	-6.9077553	3	85	2.23519444	296.888889	23.88888888	0	0	0	0	30.519848	2	0.21141735
0.0414	1318.4873	0.39978842	39.1095581	0.638105	0	-3.1844744	0.27724325	-0.9143216	3.66639246	-0.4476865	-6.9077553	4	81	2.23519444	296.888889	23.88888888	0	0	0	0	30.519848	2	0.21141735
0.0423	1362.35111	0.28468361	16.919714	0.93287736	0	-3.1629682	0.30994572	-1.2528703	2.82853855	-0.0684102	-6.9077553	5	78	2.23519444	296.333333	23.33333333	0	0	0	0	30.519848	2	0.21141735
0.0423	1362.35111	0	0	0	0	-3.1629682	0.30994572	-6.9077553	-6.9077553	-6.9077553	-6.9077553	6	76	2.68223333	295.777778	22.7777778	0	0	0	0	30.519848	2	0.21141735
0.0837	1669.39757	0.30682686	26.7488377	1.21884802	0	-2.4805163	0.51306167	-1.1782178	3.28652841	0.19872628	-6.9077553	7	69	3.12927222	296.888889	23.88888888	0	0	0	0	30.519848	2	0.21141735
0.1656	2266.50091	0.46743707	63.6516408	1.57640025	0	-1.79818	0.8186783	-0.7583535	4.15344081	0.45577808	-6.9077553	8	63	3.12927222	298	25	0	0	0	0	30.519848	2	0.21141735
0.3555	4631.33311	0.75403519	155.184038	2.98148986	0	-1.03423	1.53306065	-0.2809909	5.0446182	1.09275847	-6.9077553	9	59	4.47038889	299.111111	26.11111111	0	0	0	1	30.519848	2	0.21141735
0.6012	8086.2278	0.68467662	125.004207	2.05888708	0	-0.5088276	2.090286	-0.3773492	4.82835539	0.72265116	-6.9077553	10	54	3.57631111	300.777778	27.7777778	0	0	0	1	30.519848	2	0.21141735
1.0476	17026.6168	0.83632082	200.998913	2.71040049	0	0.04650183	2.83483654	-0.177548	5.30330448	0.99746529	-6.9077553	11	50	4.91742778	301.333333	28.33333333	0	0	0	1	30.519848	2	0.21141735
1.5471	27491.9623	0.84766791	218.764527	2.14812562	0	0.43638221	3.31393005	-0.1640873	5.3880005	0.76506107	-6.9077553	12	49	4.02335	302.444444	29.44444444	0	0	0	1	30.519848	2	0.21141735
2.2896	38648.3278	0.96767704	270.69411	3.46461441	0	0.82837713	3.65452938	-0.031824	5.60099313	1.24288993	-6.9077553	13	46	4.47038889	303	30	0	0	0	1	30.519848	2	0.21141735
3.0267	49926.9898	0.86435767	216.995765	2.49566747	0	1.10747292	3.91058176	-0.1446124	5.37988244	0.91495683	-6.9077553	14	44	4.47038889	303.555556	30.55555555	0	0	0	1	30.519848	2	0.21141735
3.7746	61057.4802	0.86117756	215.454084	2.1616869	0	1.32829442	4.1118321	-0.148294	5.37275246	0.77135139	-6.9077553	15	42	4.47038889	303.555556	30.55555555	0	0	0	1	30.519848	2	0.21141735
4.4226	77192.4495	0.91949132	246.479167	2.20128818	0	1.48672776	4.3463146	-0.0828477	5.50728153	0.7894969	-6.9077553	16	41	4.47038889	303.555556	30.55555555	0	0	0	1	30.519848	2	0.21141735
4.9869	92588.4528	0.86704713	213.771357	2.01943916	0	1.60681447	4.52817523	-0.1415093	5.36491169	0.70331489	-6.9077553	17	44	4.02335	302.444444	29.44444444	0	0	0	1	30.519848	2	0.21141735
5.5125	107767.359	0.84139628	204.473258	1.82090717	0	1.70701824	4.6799841	-0.1715047	5.32044209	0.59988385	-6.9077553	18	47	3.12927222	301.888889	28.88888888	0	0	0	1	30.519848	2	0.21141735
5.9544	121611.129	0.78962965	191.257367	1.52091753	0	1.78413044	4.80083671	-0.2349256	5.25362522	0.41997107	-6.9077553	19	52	2.23519444	300.222222	27.2222222	0	0	0	1	30.519848	2	0.21141735
6.2415	132311.056	0.83120574	200.0529	1.34354594	0	1.83122054	4.88516319	-0.1836756	5.29858683	0.29605637	-6.9077553	20	63	0.89407778	298	25	0	0	0	1	30.519848	2	0.21141735
6.4107	139006.783	0.8146579	179.355926	1.28677206	0	1.85796847	4.93452992	-0.2037603	5.18937782	0.25291364	-6.9077553	21	76	0.44703889	295.222222	22.2222222	0	0	0	1	30.519848	2	0.21141735
6.4422	140498.156	0.34392771	28.0543352	0.50211389	0	1.8628701	4.94520148	-1.0644204	3.33417882	-0.6869387	-6.9077553	22	77	0.89407778	294.666667	21.66666666	0	0	0	1	30.519848	2	0.21141735
6.4422	140498.156	0	0	0	0	1.8628701	4.94520148	-6.9077553	-6.9077553	-6.9077553	-6.9077553	23	77	0.89407778	294.666667	21.66666666	0	0	0	1	30.519848	2	0.21141735
0.0171	702.145636	0.94785945	249.251546		0	-4.0686768	-0.3521912	-0.0524946	5.51846662		-6.9077553	0	90	1.78815556	297.444444	24.44444444	0	0	0	0	34.0724638	2	0.49277013
0.027	1009.19211	0.45087337	50.876082		0	-3.6119184	0.01014052	-0.7943533	3.92941257		-6.9077553	1	90	2.23519444	296.888889	23.88888888	0	0	0	0	34.0724638	2	0.49277013
0.0405	1447.82998	0.46747421	51.3960612		0	-3.2064533	0.37075632	-0.7582742	3.939581		-6.9077553	2	84	1.78815556	296.888889	23.88888888	0	0	0	0	34.0724638	2	0.49277013
0.0504	1798.74027	0.45394542	47.5786521		0	-2.9877641	0.58764236	-0.7875778	3.86240519		-6.9077553	3	85	2.23519444	296.888889	23.88888888	0	0	0	0	34.0724638	2	0.49277013
0.0639	2281.24181	0.48542074	58.9617839		0	-2.7504359	0.82515821	-0.7206813	4.07690646		-6.9077553	4	81	2.23519444	296.888889	23.88888888	0	0	0	0	34.0724638	2	0.49277013
0.0837	3114.6537	0.52648059	68.2813776		0	-2.4805163	1.13643899	-0.6396432	4.22365172		-6.9077553	5	78	2.23519444	296.333333	23.33333333	0	0	0	0	34.0724638	2	0.49277013
0.1143	4537.3981	0.68678693	123.790025		0	-2.1689287	1.51257411	-0.3742762	4.81859486		-6.9077553	6	76	2.68223333	295.777778	22.7777778	0	0	0	0	34.0724638	2	0.49277013
0.1728	7369.15896	0.84421815	187.417773		0	-1.7556204	1.99743927	-0.1681605	5.23334554		-6.9077553	7	69	3.12927222	296.888889	23.88888888	0	0	0	0	34.0724638	2	0.49277013
0.2574	10547.6882	0.78385649	171.932617		0	-1.357124	2.35600151	-0.2422544	5.14710846		-6.9077553	8	63	3.12927222	298	25	0	0	0	0	34.0724638	2	0.49277013
0.387	14858.0711	0.88381614	218.694906		0	-0.9493306	2.69861053	-0.1223754	5.38768221		-6.9077553	9	59	4.47038889	299.111111	26.11111111	0	0	0	1	34.0724638	2	0.49277013
0.6039	21010.8808	0.99408244	276.49598		0	-0.5043467	3.04508803	-0.0049297	5.6221999		-6.9077553	10	54	3.57631111	300.777778	27.7777778	0	0	0	1	34.0724638	2	0.49277013
0.9144	28677.3874	1.1082715	377.543274		1	-0.0894872	3.35614379	0.1037035	5.93368785		0.0009995	11	50	4.91742778	301.333333	28.33333333	0	0	0	1	34.0724638	2	0.49277013
1.3095	36987.2584	1.05633121	324.464799		1	0.26964538	3.61060052	0.05574801	5.78218014		0.0009995	12	49	4.02335	302.444444	29.44444444	0	0	0	1	34.0724638	2	0.49277013
1.7622	48621.3428	0.9700355	273.993073		1	0.56656303	3.88408315	-0.0293923	5.61310647		0.0009995	13	46	4.47038889	303	30	0	0	0	1	34.0724638	2	0.49277013
2.349	63778.8626	0.92847466	248.354007		1	0.85398971	4.15543751	-0.0731357	5.5148592		0.0009995	14	44	4.47038889	303.555556	30.55555555	0	0	0	1	34.0724638	2	0.49277013
3.2319	85462.8612	0.93666604	257.953874		1	1.1730702	4.44809361	-0.0643614	5.55278466		0.0009995	15	42	4.47038889	303.555556	30.55555555	0	0	0	1	34.0724638	2	0.49277013
4.1319	114832.503	0.91362683	249.095219		1	1.41873735	4.74348328	-0.0892391	5.51783924		0.0009995	16	41	4.47038889	303.555556	30.55555555	0	0	0	1	34.0724638	2	0.49277013
5.1669	144790.331	0.84857395	211.202582		1	1.6422729	4.97529361	-0.1630203	5.35282251		0.0009995	17	44	4.02335	302.444444	29.44444444	0	0	0	1	34.0724638	2	0.49277013
6.1839	177807.932	0.8663416	222.8707		1	1.82194914	5.18070956	-0.1423224	5.40659627		0.0009995	18	47	3.12927222	301.888889	28.88888888	0	0	0	1	34.0724638	2	0.49277013
7.029	206667.205	0.83070369	203.621489		1	1.95004445	5.33111463	-0.184279	5.31626774		0.0009995	19	52	2.23519444	300.222222	27.2222222	0	0	0	1	34.0724638	2	0.49277013
7.5447	229236.378	0.84136462	201.710719		1	2.02084533	5.43475805	-0.1715423	5.30683955		0.0009995	20	63	0.89407778	298	25	0	0	0	1	34.0724638	2	0.49277013
7.8606	243859.246	0.79953238	169.953236		1	2.06186294	5.4965953	-0.2224783	5.1355292		0.0009995	21	76	0.44703889	295.222222	22.2222222	0	0	0	1	34.0724638	2	0.49277013
7.9605	248245.642	0.42137936	47.5920916		1	2.07449181	5.51442277	-0.8618514	3.86268762		0.0009995	22	77	0.89407778	294.666667	21.66666666	0	0	0	1	34.0724638	2	0.49277013
7.9605	248245.642	0	0		1	2.07449181	5.51442277	-6.9077553	-6.9077553		0.0009995	23	77	0.89407778	294.666667	21.66666666	0	0	0	1	34.0724638	2	0.49277013
0.0279	1522.54386	0.99183038	266.11569	1.82331263	0	-3.5791286	0.42103911	-0.0071954	5.5839349	0.60120328	-6.9077553	0	90	1.78815556	297.444444	24.44444444	0	0	0	0	42.288408	2	-0.4111751
0.0639	3277.09522	0.64236031	102.491846	1.0452776	0	-2.7504359	1.18726253	-0.4410504	4.629793	0.04523872	-6.9077553	1	90	2.23519444	296.888889	23.88888888	0	0	0	0	42.288408	2	-0.4111751
0.1089	5470.28498	0.61427406	92.9131055	0.99547943	0	-2.2173252	1.6995135	-0.4856875	4.53167547	-0.0035268	-6.9077553	2	84	1.78815556	296.888889	23.88888888	0	0	0	0	42.288408	2	-0.4111751
0.1692	8233.70186	0.59442235	87.2345062	0.98591955	0	-1.7766738	2.10835716	-0.5184843	4.46861143	-0.0131667	-6.9077553	3	85	2.23519444	296.888889	23.88888888	0	0	0	0	42.288408	2	-0.4111751
0.2367	11479.6235	0.6339178	101.189661	1.16837819	0	-1.4409618	2.4406607	-0.4542597	4.61700647	0.15647215	-6.9077553	4	81	2.23519444	296.888889	23.88888888	0	0	0	0	42.288408	2	-0.4111751
0.3213	15501.1876	0.7355983	142.228495	1.40559517	0	-1.13538	2.74098115	-0.3057126	4.95744191	0.34117201	-6.9077553	5	78	2.23519444	296.333333	23.33333333	0	0	0	0	42.288408	2	-0.4111751

0.4563	22458.8311	0.8917041	216.53457	1.72248983	0	-0.7846048	3.11172843	-0.1135001	5.37775483	0.5443512	-6.9077553	6	76	2.68223333	295.777778	22.7777778	0	0	1	0	42.288408	2	-0.4111751
0.7236	35291.7981	0.96616197	271.045455	2.15338196	0	-0.3235165	3.56367892	-0.0333893	5.60229023	0.76750389	-6.9077553	7	69	3.12927222	296.888889	23.88888888	0	0	1	0	42.288408	2	-0.4111751
1.0881	45097.6273	0.83586373	200.924826	2.66769417	0	0.08443306	3.80885181	-0.178094	5.30293582	0.98158928	-6.9077553	8	63	3.12927222	298	25	0	0	1	0	42.288408	2	-0.4111751
1.4904	58680.5517	0.81918349	179.47277	1.89481117	0	0.39904454	4.0721254	-0.1982272	5.19002907	0.6396468	-6.9077553	9	59	4.47038889	299.111111	26.11111111	0	0	1	0	42.288408	2	-0.4111751
1.9305	75361.56	0.8634349	197.778758	1.82593934	0	0.65777904	4.3223106	-0.1456793	5.28715408	0.60264208	-6.9077553	10	54	3.57631111	300.777778	27.7777778	0	0	1	0	42.288408	2	-0.4111751
2.5605	100494.254	0.93528791	240.434174	2.41118199	0	0.94020255	4.6101105	-0.0658323	5.48245051	0.88053173	-6.9077553	11	50	4.91742778	301.333333	28.33333333	0	0	1	0	42.288408	2	-0.4111751
3.303	126272.935	0.88570046	223.984242	2.25762251	0	1.19483115	4.83845363	-0.1202481	5.41158017	0.81475512	-6.9077553	12	49	4.02335	302.444444	29.44444444	0	0	1	0	42.288408	2	-0.4111751
4.4235	145982.012	0.81356	196.194001	3.53248227	0	1.48693124	4.98349026	-0.2051072	5.27910907	1.26228386	-6.9077553	13	46	4.47038889	303	30	0	0	1	0	42.288408	2	-0.4111751
5.4918	169053.005	0.78365989	179.503396	2.87703046	0	1.70325607	5.13021822	-0.2425049	5.1901997	1.05710619	-6.9077553	14	44	4.47038889	303.555556	30.55555555	0	0	1	0	42.288408	2	-0.4111751
6.462	191782.85	0.75810765	167.725939	2.28297031	0	1.86593887	5.25636896	-0.2756117	5.1223373	0.82591529	-6.9077553	15	42	4.47038889	303.555556	30.55555555	0	0	1	0	42.288408	2	-0.4111751
7.4799	224665.973	0.81373139	200.376547	2.12377282	0	2.01221942	5.41461919	-0.2048968	5.30020332	0.75366489	-6.9077553	16	41	4.47038889	303.555556	30.55555555	0	0	1	0	42.288408	2	-0.4111751
8.5095	261697.666	0.83981819	207.77759	2.03832496	0	2.14118319	5.56719371	-0.1733798	5.33647304	0.71261885	-6.9077553	17	44	4.02335	302.444444	29.44444444	0	0	1	0	42.288408	2	-0.4111751
9.3978	297970.859	0.84370783	211.726127	1.64153676	0	2.24047562	5.69699905	-0.1687645	5.35529831	0.49624185	-6.9077553	18	47	3.12927222	301.888889	28.88888888	0	0	1	0	42.288408	2	-0.4111751
10.1529	328737.505	0.82947345	203.31114	1.49442492	0	2.31775938	5.79526262	-0.1857593	5.31474244	0.4024104	-6.9077553	19	52	2.23519444	300.222222	27.2222222	0	0	1	0	42.288408	2	-0.4111751
10.7559	357522.276	0.92830018	245.248904	1.73158414	0	2.37545444	5.87920047	-0.0733235	5.50227771	0.54961402	-6.9077553	20	63	0.89407778	298	25	0	0	1	0	42.288408	2	-0.4111751
11.0916	373015.149	0.83160629	187.163748	1.46683664	0	2.40618807	5.92162171	-0.1831944	5.23198924	0.38378964	-6.9077553	21	76	0.44703889	295.222222	22.2222222	0	0	1	0	42.288408	2	-0.4111751
11.1681	376392.667	0.37755105	34.7259708	0.59780058	0	2.4130615	5.93063558	-0.9714044	3.54751664	-0.5128267	-6.9077553	22	77	0.89407778	294.666667	21.66666666	0	0	1	0	42.288408	2	-0.4111751
11.1681	376392.667	0	0	0	0	2.4130615	5.93063558	-6.9077553	-6.9077553	-6.9077553	-6.9077553	23	77	0.89407778	294.666667	21.66666666	0	0	1	0	42.288408	2	-0.4111751
0.0009	0	0	0	0	0	-7.0131158	-6.9077553	-6.9077553	-6.9077553	-6.9077553	-6.9077553	0	90	1.78815556	297.444444	24.44444444	0	0	0	0	18.9135952	2	-0.0428739
0.0009	0	0	0	0	0	-7.0131158	-6.9077553	-6.9077553	-6.9077553	-6.9077553	-6.9077553	1	90	2.23519444	296.888889	23.88888888	0	0	0	0	18.9135952	2	-0.0428739
0.0009	0	0	0	0	0	-7.0131158	-6.9077553	-6.9077553	-6.9077553	-6.9077553	-6.9077553	2	84	1.78815556	296.888889	23.88888888	0	0	0	0	18.9135952	2	-0.0428739
0.0009	0	0	0	0	0	-7.0131158	-6.9077553	-6.9077553	-6.9077553	-6.9077553	-6.9077553	3	85	2.23519444	296.888889	23.88888888	0	0	0	0	18.9135952	2	-0.0428739
0.0009	0	0	0	0	0	-7.0131158	-6.9077553	-6.9077553	-6.9077553	-6.9077553	-6.9077553	4	81	2.23519444	296.888889	23.88888888	0	0	0	0	18.9135952	2	-0.0428739
0.0009	0	0	0	0	0	-7.0131158	-6.9077553	-6.9077553	-6.9077553	-6.9077553	-6.9077553	5	78	2.23519444	296.333333	23.33333333	0	0	0	0	18.9135952	2	-0.0428739
0.0009	0	0	0	0	0	-7.0131158	-6.9077553	-6.9077553	-6.9077553	-6.9077553	-6.9077553	6	76	2.68223333	295.777778	22.7777778	0	0	0	0	18.9135952	2	-0.0428739
0.0009	0	0	0	0	0	-7.0131158	-6.9077553	-6.9077553	-6.9077553	-6.9077553	-6.9077553	7	69	3.12927222	296.888889	23.88888888	0	0	0	0	18.9135952	2	-0.0428739
0.0162	0	0.37601342	43.2027265	1.42546878	0	-4.122744	-6.9077553	-0.9754745	3.76592675	0.35520201	-6.9077553	8	63	3.12927222	298	25	0	0	0	0	18.9135952	2	-0.0428739
0.1566	264.084868	0.63035026	107.909418	2.96700502	0	-1.8540605	-1.3277052	-0.4598945	4.68130142	1.08789002	-6.9077553	9	59	4.47038889	299.111111	26.11111111	0	0	1	0	18.9135952	2	-0.0428739
0.3258	966.573795	0.52456426	75.8940118	1.92287753	0	-1.1214716	-0.0329636	-0.6432828	4.32935096	0.6543427	-6.9077553	10	54	3.57631111	300.777778	27.7777778	0	0	1	0	18.9135952	2	-0.0428739
0.6444	3895.27714	0.65462757	129.118865	3.25031173	0	-0.4394356	1.36002152	-0.4221624	4.86074116	1.17905852	-6.9077553	11	50	4.91742778	301.333333	28.33333333	0	0	1	0	18.9135952	2	-0.0428739
1.0089	9389.28844	0.66916534	135.301669	2.5415446	0	0.00886063	2.23967601	-0.4002308	4.90751426	0.93316539	-6.9077553	12	49	4.02335	302.444444	29.44444444	0	0	1	0	18.9135952	2	-0.0428739
1.503	15886.5055	0.6679812	135.439265	2.33424497	0	0.40746311	2.76553298	-0.4019993	4.90853069	0.8481168	-6.9077553	13	46	4.47038889	303	30	0	0	1	0	18.9135952	2	-0.0428739
2.2311	28580.0968	0.7609858	175.786291	2.86095422	0	0.80249474	3.35274555	-0.2718274	5.16927469	1.05150468	-6.9077553	14	44	4.47038889	303.555556	30.55555555	0	0	1	0	18.9135952	2	-0.0428739
3.2148	59956.5879	0.91198094	255.614965	3.20858365	0	1.16776515	4.09363744	-0.0910403	5.54367618	1.16614123	-6.9077553	15	42	4.47038889	303.555556	30.55555555	0	0	1	0	18.9135952	2	-0.0428739
4.0536	96502.6922	0.97150588	280.745088	2.60869837	0	1.39960538	4.56958127	-0.0278792	5.63745066	0.95923465	-6.9077553	16	41	4.47038889	303.555556	30.55555555	0	0	1	0	18.9135952	2	-0.0428739
4.9815	140725.437	0.99012237	284.490968	2.41906845	0	1.60573105	4.94681784	-0.0089173	5.65070502	0.88379582	-6.9077553	17	44	4.02335	302.444444	29.44444444	0	0	1	0	18.9135952	2	-0.0428739
5.8608	185113.337	0.98481761	274.838856	2.08135943	0	1.76828611	5.22097367	-0.0142839	5.61618859	0.73350159	-6.9077553	18	47	3.12927222	301.888889	28.88888888	0	0	1	0	18.9135952	2	-0.0428739
6.6627	223320.58	0.93162816	244.62258	1.76538744	0	1.89652481	5.4086128	-0.0697487	5.49972062	0.56893646	-6.9077553	19	52	2.23519444	300.222222	27.2222222	0	0	1	0	18.9135952	2	-0.0428739
7.2675	253423.054	0.94019599	242.462147	1.68922025	0	1.98341235	5.53506419	-0.0606039	5.49084973	0.52485884	-6.9077553	20	63	0.89407778	298	25	0	0	1	0	18.9135952	2	-0.0428739
7.7274	275271.16	0.83319605	185.7145	1.45890925	0	2.04477245	5.61776028	-0.1812868	5.22421593	0.37837427	-6.9077553	21	76	0.44703889	295.222222	22.2222222	0	0	1	0	18.9135952	2	-0.0428739
7.8579	281236.659	0.26350634	15.1198478	0.44950467	0	2.0615194	5.63920007	-1.32989	2.71607444	-0.7973868	-6.9077553	22	77	0.89407778	294.666667	21.66666666	0	0	1	0	18.9135952	2	-0.0428739
7.8579	281236.659	0	0	0	0	2.0615194	5.63920007	-6.9077553	-6.9077553	-6.9077553	-6.9077553	23	77	0.89407778	294.666667	21.66666666	0	0	1	0	18.9135952	2	-0.0428739
0.0135	405.930043	0.73705373	164.878627	1.40410655	0	-4.3050656	-0.899114	-0.3037386	5.10521568	0.34011314	-6.9077553	0	58	2.68223333	305.777778	32.7777778	0	0	0	0	30.519848	2	0.21141735
0.0333	1193.94576	0.90683157	222.78782	1.57735946	0	-3.4021979	0.1781008	-0.0966964	5.40622433	0.45638599	-6.9077553	1	65	1.78815556	304.111111	31.11111111	0	0	0	0	30.519848	2	0.21141735
0.0432	1457.24304	0.69938985	134.202615	1.52924763	0	-3.1419148	0.37723231	-0.3561182	4.89935816	0.42542957	-6.9077553	2	70	1.78815556	303	30	0	0	0	0	30.519848	2	0.21141735
0.0441	1501.10673	0.58925223	82.2652512	1.049052	0	-3.1212955	0.40686861	-0.5272053	4.40996095	0.04883969	-6.9077553	3	77	0.44703889	301.888889	28.88888888	0	0	0	0	30.519848	2	0.21141735
0.0441	1501.10673	0	0	0	0	-3.1212955	0.40686861	-6.9077553	-6.9077553	-6.9077553	-6.9077553	4	75	0.89407778	297.444444	24.44444444	0	0	0	0	30.519848	2	0.21141735
0.0441	1501.10673	0	0	0	0	-3.1212955	0.40686861	-6.9077553	-6.9077553	-6.9077553	-6.9077553	5	88	0.89407778	295.222222	22.2222222	0	0	0	0	30.519848	2	0.21141735
0.0441	1501.10673	0	0	0	0	-3.1212955	0.40686861	-6.9077553	-6.9077553	-6.9077553	-6.9077553	6	46	2.23519444	296.888889	23.88888888	0	0	0	0	30.519848	2	0.21141735
0.0909	1764.28944	0.33419862	34.6516043	1.18242785	0	-2.3979953	0.56831467	-1.093032	3.54537288	0.16841518	-6.9077553	7	42	2.23519444	296.333333	23.33333333	0	0	0	0	30.519848	2	0.21141735
0.1593	2523.64362	0.44475274	57.9908255	1.38038173	0	-1.8369661	0.92609991	-0.8079909	4.06030206	0.32308426	-6.9077553	8	35	3.12927222	295.777778	22.7777778	0	0	0	0	30.519848	2	0.21141735
0.3618	4129.8883	0.68371189	128.615217	3.14598036	0	-1.0166637	1.41849247	-0.3787571	4.85683291	1.14644338	-6.9077553	9	36	4.91742778	294.666667	21.66666666	0	0	0	0	30.519848	2	0.21141735
0.5058	5803.16486	0.532581	77.3536194	1.40139637	0	-0.6816139	1.75857574	-0.6281444	4.3484003	0.33818247	-6.9077553	10	43	2.68223333	293	20	0	0	0	1	30.519848	2	0.21141735
0.6309	7648.59405	0.48854241	70.5916774	0.8664065	0	-0.4606079	2.03465258	-0.7142842	4.25692642	-0.1422476	-6.9077553	11	54	0.44703889	290.222222	17.2222222	0	0	0	1	30.519848	2	0.21141735
0.7623	9463.10339	0.49930473	73.0682657	0.92732134	0	-0.2714151	2.24750605	-0.6925379	4.29140784	-0.0743773	-6.9077553	12	55	1.34111667	291.333333	18.33333333	0	0	0	1	30.519848	2	0.21141735
1.0377	12174.0768	0.63646164	114.719937	1.85033621	0	0.03700673	2.49939098	-0.4502612	4.74250255	0.61590766	-6.9077553	13	46	3.12927222	294.111111	21.11111111	0	0	0	1	30.519848	2	0.21141735
1.7811	19609.0427	0.9684296	284.967312	4.09576963	0	0.57723115	2.97604182	-0.0310474	5.65237799	1.41019877	-6.9077553	14	40	5.36446667	295.777778	22.7777778	0	0	0	1	30.519848	2	0.21141735
2.7945	29983.9245	0.97684285	285.520038	4.31059544	0	1.0276532	3.40069474	-0.0224063	5.65431571	1.46130801	-6.9077553	15	35	6.25854444	297.444444	24.44444444	0	0	0	1	30.519848	2	0.21141735
3.9078	49598.3517	1.01906073	310.993331	3.21182801	0	1.36297456	3.90397776	0.01986217	5.73977468	1.16715155	-6.9077553	16	31	6.70558333	299.111111	26.11111111	0	0	0	1	30.519848	2	0.21141735
5.0499	164970.128	1.2638329	444.88485	2.32628115	85	1.61936844	5.10577048	0.23494002	6.09781773	0.8447007	4.44266302	17	27	6.25854444	300.777778	27.7777778	0	0	0	1	30.519848	2	0.21141735
6.1785	292455.596	1.26254754	446.935407	2.94618335	192	1.82107552	5.67831627	0.23392328	6.10241632	1.08084992	5.25750058	18	24	7.59966111	301.888889	28.88888888	0	0	0	1	30.519848	2	0.21141735
7.2369	641579.066	2.1023736	975.933355	3.30631386	504	1.97919294	6.46393399	0.74354253	6.88339532	1.19613634	6.22257825	19	23	5.36446667	303.555556	30.55555555	0	0	0	1	30.519848	2	0.21141735

8.8335	1455480.68	3.08532261	1622.64431	4.12021848	1306	2.17855131	7.28309217	1.12698029	7.391813	1.41614887	7.17472508	20	23	5.81150556	303.555556	30.55555555	0	0	0	1	30.519848	2	0.21141735
11.1321	2130406.64	2.49901923	1308.39957	5.63723953	2000	2.40983283	7.66406862	0.91629842	7.17656073	1.72957188	7.60090296	21	22	6.25854444	304.111111	31.11111111	0	0	0	1	30.519848	2	0.21141735
12.1716	2234938.72	1.33699312	531.722727	2.8422619	2101	2.49910537	7.71196953	0.29117082	6.27612404	1.04495195	7.65016918	22	23	5.36446667	304.111111	31.11111111	0	0	0	1	30.519848	2	0.21141735
13.0806	2300661.21	1.01463671	307.966399	1.98246176	2146	2.57113022	7.74095228	0.01551572	5.72999393	0.68484368	7.67136139	23	24	4.91742778	303.555556	30.55555555	0	0	0	1	30.519848	2	0.21141735
0.0351	1302.49534	0.97870315	271.892347	2.6107515	0	-3.3495541	0.26504938	-0.0205057	5.60540988	0.96002107	-6.9077553	0	58	2.68223333	305.777778	32.7777778	0	0	0	0	34.0724638	2	0.49277013
0.0882	3617.08554	0.84820634	194.528469	1.33473736	0	-2.4281483	1.28594503	-0.1634531	5.27058366	0.28948347	-6.9077553	1	65	1.78815556	304.111111	31.11111111	0	0	0	0	34.0724638	2	0.49277013
0.1503	6473.91917	0.7578878	146.201144	1.0595482	0	-1.895122	1.86793612	-0.2759013	4.98499021	0.05878594	-6.9077553	2	70	1.78815556	303	30	0	0	0	0	34.0724638	2	0.49277013
0.1935	8316.19851	0.66029537	107.553569	0.86876758	0	-1.6424778	2.11832548	-0.4135547	4.67799833	-0.1395293	-6.9077553	3	77	0.44703889	301.888889	28.88888888	0	0	0	0	34.0724638	2	0.49277013
0.2259	9588.24899	0.51614226	62.6091173	0.67463472	0	-1.4876629	2.26064257	-0.6594373	4.13692688	-0.3921027	-6.9077553	4	75	0.89407778	297.444444	24.44444444	0	0	0	0	34.0724638	2	0.49277013
0.2439	10158.4779	0.44394288	47.655304	0.63161077	0	-1.410997	2.31840705	-0.8098094	3.86401492	-0.4578999	-6.9077553	5	88	0.89407778	295.222222	22.2222222	0	0	0	0	34.0724638	2	0.49277013
0.2664	10948.026	0.337691	36.1569263	0.57365265	0	-1.3227563	2.3932505	-1.0826671	3.58789619	-0.5539895	-6.9077553	6	46	2.23519444	296.888889	23.88888888	0	0	0	0	34.0724638	2	0.49277013
0.3708	14875.2368	0.8217119	199.127913	1.62749218	0	-0.9920924	2.69976509	-0.1951492	5.29395242	0.48765454	-6.9077553	7	42	2.23519444	296.333333	23.33333333	0	0	0	1	34.0724638	2	0.49277013
0.5229	21456.4669	0.90253209	228.417483	1.61971811	0	-0.648365	3.06607269	-0.1014436	5.4311794	0.48286933	-6.9077553	8	35	3.12927222	295.777778	22.7777778	0	0	0	1	34.0724638	2	0.49277013
0.7686	29910.8236	0.9617456	264.930804	2.24467429	0	-0.2631846	3.39825384	-0.0379661	5.57947245	0.80900583	-6.9077553	9	36	4.91742778	294.666667	21.66666666	0	0	0	1	34.0724638	2	0.49277013
1.0323	41803.8563	0.952348	255.562607	1.90910678	0	0.03178932	3.73301251	-0.0477753	5.54347133	0.64715915	-6.9077553	10	43	2.68223333	293	20	0	0	0	1	34.0724638	2	0.49277013
1.2753	50507.7613	0.85203908	213.806366	1.59038018	0	0.24318145	3.92214681	-0.1589499	5.36507545	0.46460168	-6.9077553	11	54	0.44703889	290.222222	17.2222222	0	0	0	1	34.0724638	2	0.49277013
1.4553	55913.0293	0.69114151	138.103682	1.11163765	0	0.37521206	4.02381532	-0.3679649	4.92801196	0.10673346	-6.9077553	12	55	1.34111667	291.333333	18.33333333	0	0	0	1	34.0724638	2	0.49277013
1.8279	68050.6639	0.87778042	217.930442	1.92932866	0	0.60316777	4.22026718	-0.1292202	5.38418053	0.65769028	-6.9077553	13	46	3.12927222	294.111111	21.11111111	0	0	0	1	34.0724638	2	0.49277013
2.412	90204.6283	1.00248993	283.000794	2.45970288	0	0.88045628	4.50209182	0.00348386	5.64545324	0.90044703	-6.9077553	14	40	5.36446667	295.777778	22.7777778	0	0	0	1	34.0724638	2	0.49277013
3.2436	119812.421	1.05272321	312.678369	2.89819494	0	1.17668382	4.78593571	0.05232981	5.74517828	1.06443309	-6.9077553	15	35	6.25854444	297.444444	24.44444444	0	0	0	1	34.0724638	2	0.49277013
4.4037	162493.218	1.08148653	336.692324	3.15661138	0	1.4824451	5.09064242	0.07926073	5.8191725	1.14981585	-6.9077553	16	31	6.70558333	299.111111	26.11111111	0	0	0	1	34.0724638	2	0.49277013
5.7393	207128.575	1.0478222	315.339159	3.08517547	0	1.74733725	5.33334456	0.04766782	5.75365193	1.12693261	-6.9077553	17	27	6.25854444	300.777778	27.7777778	0	0	0	1	34.0724638	2	0.49277013
7.2342	265700.302	1.15277147	399.431291	4.0086315	0	1.97881978	5.58237275	0.14303612	5.99004427	1.38869934	-6.9077553	18	24	7.59966111	301.888889	28.88888888	0	0	0	1	34.0724638	2	0.49277013
8.901	317667.395	1.00719885	306.55953	3.37978948	0	2.18616363	5.76100806	0.00816542	5.72541522	1.21810926	-6.9077553	19	23	5.36446667	303.555556	30.55555555	0	0	0	1	34.0724638	2	0.49277013
10.5228	362789.836	0.89080641	244.117664	3.19922038	0	2.35354433	5.89382646	-0.1145062	5.49765444	1.16321967	-6.9077553	20	23	5.81150556	303.555556	30.55555555	0	0	0	1	34.0724638	2	0.49277013
12.2013	420317.524	1.00779803	326.383512	3.54410711	0	2.5015425	6.04101282	0.00875956	5.78807617	1.26556837	-6.9077553	21	22	6.25854444	304.111111	31.11111111	0	0	0	1	34.0724638	2	0.49277013
13.6521	473173.929	0.9983001	315.579676	3.0957316	0	2.61389336	6.15946515	-0.0007001	5.75441436	1.13034723	-6.9077553	22	23	5.36446667	304.111111	31.11111111	0	0	0	1	34.0724638	2	0.49277013
15.1299	524701.925	0.96647705	288.190907	3.19999905	0	2.71667292	6.26283225	-0.0330636	5.6636266	1.16346296	-6.9077553	23	24	4.91742778	303.555556	30.55555555	0	0	0	1	34.0724638	2	0.49277013
0.0405	2859.20527	1.23190871	421.853693	2.66503525	0	-3.2064533	1.05089339	0.20937618	6.04466093	0.98059244	-6.9077553	0	58	2.68223333	305.777778	32.7777778	0	0	0	0	42.288408	2	-0.4111751
0.126	7718.54856	0.99124226	272.361431	1.93207799	0	-2.0714734	2.04375589	-0.007788	5.60713365	0.65911355	-6.9077553	1	65	1.78815556	304.111111	31.11111111	0	0	0	0	42.288408	2	-0.4111751
0.2241	12824.7476	0.93077766	234.382203	1.78403578	0	-1.4956629	2.55145468	-0.0706611	5.4569574	0.57943846	-6.9077553	2	70	1.78815556	303	30	0	0	0	0	42.288408	2	-0.4111751
0.3231	17596.3174	0.88585094	208.245756	1.74160475	0	-1.1297934	2.86774647	-0.1200784	5.33872371	0.55538098	-6.9077553	3	77	0.44703889	301.888889	28.88888888	0	0	0	0	42.288408	2	-0.4111751
0.3987	21149.2842	0.66624651	113.403088	1.25588571	0	-0.919546	3.05165334	-0.4045957	4.73095744	0.22863701	-6.9077553	4	75	0.89407778	297.444444	24.44444444	0	0	0	0	42.288408	2	-0.4111751
0.4698	24439.0674	0.56825329	82.1962273	1.01307475	0	-0.7554482	3.19622389	-0.5634298	4.40912157	0.01397662	-6.9077553	5	88	0.89407778	295.222222	22.2222222	0	0	1	0	42.288408	2	-0.4111751
0.5472	27947.1918	0.53667632	97.9210006	0.95093669	0	-0.6029409	3.3303525	-0.6204985	4.58417125	-0.0492567	-6.9077553	6	46	2.23519444	296.888889	23.88888888	0	0	1	0	42.288408	2	-0.4111751
0.8676	40719.1456	0.86480199	228.619821	1.88063787	0	-0.1420245	3.70672295	-0.1440991	5.43206483	0.6321426	-6.9077553	7	42	2.23519444	296.333333	23.33333333	0	0	1	0	42.288408	2	-0.4111751
1.2114	52288.3228	0.83321872	192.151628	1.85088664	0	0.19177672	3.9567922	-0.1812597	5.25828999	0.61620492	-6.9077553	8	35	3.12927222	295.777778	22.7777778	0	0	1	0	42.288408	2	-0.4111751
1.5993	65612.003	0.80639967	173.115465	1.90041286	0	0.46956603	4.18377389	-0.2139365	5.15396458	0.64259722	-6.9077553	9	36	4.91742778	294.666667	21.66666666	0	0	1	0	42.288408	2	-0.4111751
1.98	80510.5854	0.85366713	202.880596	1.67183243	0	0.68309684	4.38840109	-0.1570432	5.31262254	0.51451826	-6.9077553	10	43	2.68223333	293	20	0	0	1	0	42.288408	2	-0.4111751
2.2923	92037.9302	0.72960678	148.208889	1.23631845	0	0.82955568	4.52221164	-0.3138799	4.99862944	0.2129465	-6.9077553	11	54	0.44703889	290.222222	17.2222222	0	0	1	0	42.288408	2	-0.4111751
2.6604	107647.606	0.80415191	180.188703	1.33327285	0	0.97847649	4.67887228	-0.2167243	5.1940102	0.28838646	-6.9077553	12	55	1.34111667	291.333333	18.33333333	0	0	1	0	42.288408	2	-0.4111751
3.3237	132507.791	0.86231943	210.088916	2.01448496	0	1.20107862	4.88664899	-0.1469705	5.34753561	0.70085984	-6.9077553	13	46	3.12927222	294.111111	21.11111111	0	0	1	0	42.288408	2	-0.4111751
4.4991	157573.651	0.81606546	198.713366	3.29421936	0	1.50387738	5.05989932	-0.2020361	5.29186845	1.19247274	-6.9077553	14	40	5.36446667	295.777778	22.7777778	0	0	1	0	42.288408	2	-0.4111751
6.0417	186261.248	0.83056762	205.988514	3.94571104	0	1.79868543	5.22715561	-0.1844427	5.32782526	1.37288259	-6.9077553	15	35	6.25854444	297.444444	24.44444444	0	0	1	0	42.288408	2	-0.4111751
7.785	239928.54	1.03676541	341.465636	4.41723671	0	2.05219881	5.4803453	0.03706976	5.83324998	1.48574068	-6.9077553	16	31	6.70558333	299.111111	26.11111111	0	0	1	0	42.288408	2	-0.4111751
9.2295	308934.609	1.19222188	431.537422	4.21706551	0	2.22240488	5.73313287	0.17665711	6.06735655	1.43937661	-6.9077553	17	27	6.25854444	300.777778	27.7777778	0	0	1	0	42.288408	2	-0.4111751
10.5651	379825.507	1.19199653	431.472793	3.55791002	0	2.35755612	5.93971459	0.17646824	6.06720677	1.26945433	-6.9077553	18	24	7.59966111	301.888889	28.88888888	0	0	1	0	42.288408	2	-0.4111751
12.0294	453009.986	1.17788998	411.120813	3.35550656	0	2.48735365	6.11591638	0.1645733	6.01888955	1.21090072	-6.9077553	19	23	5.36446667	303.555556	30.55555555	0	0	1	0	42.288408	2	-0.4111751
13.5396	520931.235	1.15028558	387.706794	3.59029828	1	2.60561872	6.25561997	0.14087921	5.96025195	1.27851377	0.0009995	20	23	5.81150556	303.555556	30.55555555	0	0	1	0	42.288408	2	-0.4111751
15.1164	601436.41	1.20314423	441.701912	3.8499126	2	2.71578025	6.39932247	0.18576913	6.09063751	1.34831016	0.69364706	21	22	6.25854444	304.111111	31.11111111	0	0	1	0	42.288408	2	-0.4111751
16.5681	663598.474	1.07142619	344.565096	3.15671588	2	2.80747916	6.49767877	0.06992355	5.84228593	1.14984894	0.69364706	22	23	5.36446667	304.111111	31.11111111	0	0	1	0	42.288408	2	-0.4111751
18.0234	719765.849	1.02373494	309.370362	2.97557356	2	2.89167091	6.57892734	0.02443398	5.73454237	1.09077283	0.69364706	23	24	4.91742778	303.555556	30.55555555	0	0	1	0	42.288408	2	-0.4111751
0.0027	0	0.21041781	8.77035046	0.69165194	0	-5.9145035	-6.9077553	-1.553919	2.17149078	-0.3672277	-6.9077553	0	58	2.68223333	305.777778	32.7777778	0	0	0	0	18.9135952	2	-0.0428739
0.0036	0	0.06053963	0.5845502	0.09518638	0	-5.6268214	-6.9077553	-2.788074	-0.5352034	-2.3414676	-6.9077553	1	65	1.78815556	304.111111	31.11111111	0	0	0	0	18.9135952	2	-0.0428739
0.0036	0	0	0	0	0	-5.6268214	-6.9077553	-6.9077553	-6.9077553	-6.9077553	-6.9077553	2	70	1.78815556	303	30	0	0	0	0	18.9135952	2	-0.0428739
0.0036	0	0	0	0	0	-5.6268214	-6.9077553	-6.9077553	-6.9077553	-6.9077553	-6.9077553	3	77	0.44703889	301.888889	28.88888888	0	0	0	0	18.9135952	2	-0.0428739
0.0036	0	0	0	0	0	-5.6268214	-6.9077553	-6.9077553	-6.9077553	-6.9077553	-6.9077553	4	75	0.89407778	297.444444	24.44444444	0	0	0	0	18.9135952	2	-0.0428739
0.0036	0	0	0	0	0	-5.6268214	-6.9077553	-6.9077553	-6.9077553	-6.9077553	-6.9077553	5	88	0.89407778	295.222222	22.2222222	0	0	0	0	18.9135952	2	-0.0428739
0.0036	0	0	0	0	0	-5.6268214	-6.9077553	-6.9077553	-6.9077553	-6.9077553	-6.9077553	6	46	2.23519444	296.888889	23.88888888	0	0	0	0	18.9135952	2	-0.0428739
0.0396	0	0.41784134	45.2463715	1.57748966	0	-3.2289262	-6.9077553	-0.8702631	3.81214458	0.45646848	-6.9077553	7	42	2.23519444	296.333333	23.33333333	0	0	0	0	18.9135952	2	-0.0428739
0.1179	0	0.40626833	41.056433	1.66732876	0	-2.1379185	-6.9077553	-0.898283	3.71497189	0.51182238	-6.9077553	8	35	3.12927222	295.777778	22.7777778	0	0	0	0	18.9135952	2	-0.0428739
0.2484	175.455131	0.37435711	35.3054654	1.59950372	0	-1.3927149	-1.7346886	-0.9798774	3.5640661	0.47031841	-6.9077553	9	36	4.91742778	294.666667	21.66666666	0	0	1	0	18.9135952	2	-0.0428739

0.342	745.684337	0.3910232	52.6515832	1.1234929	0	-1.0729445	-0.2921128	-0.9364343	3.9637153	0.11733218	-6.9077553	10	43	2.68223333	293	20	0	0	1	0	18.9135952	2	-0.0428739
0.4077	1831.63681	0.45920908	79.2103756	0.82117039	0	-0.8972237	0.60575581	-0.7760744	4.37211992	-0.1958076	-6.9077553	11	54	0.44703889	290.222222	17.2222222	0	0	1	0	18.9135952	2	-0.0428739
0.495	4275.20206	0.61225899	126.611852	1.11461608	0	-0.7031975	1.45306525	-0.4889679	4.84113402	0.10940679	-6.9077553	12	55	1.34111667	291.333333	18.33333333	0	0	1	0	18.9135952	2	-0.0428739
0.702	7263.02743	0.57775746	114.96999	1.59854551	0	-0.3538219	1.98293442	-0.5468718	4.74467983	0.46971953	-6.9077553	13	46	3.12927222	294.111111	21.11111111	0	0	1	0	18.9135952	2	-0.0428739
1.1466	15537.2954	0.83698226	234.953916	3.33021008	0	0.13680104	2.74330765	-0.1767583	5.45939365	1.20333563	-6.9077553	14	40	5.36446667	295.777778	22.7777778	0	0	1	0	18.9135952	2	-0.0428739
1.773	31840.277	0.94584691	275.864404	4.32850945	0	0.57267303	3.46076347	-0.0546179	5.61991308	1.46545424	-6.9077553	15	35	6.25854444	297.444444	24.44444444	0	0	1	0	18.9135952	2	-0.0428739
2.5578	66258.2174	1.10190841	388.599341	3.94540657	0	0.93914751	4.19357459	0.0979507	5.96255141	1.37280544	-6.9077553	16	31	6.70558333	299.111111	26.11111111	0	0	1	0	18.9135952	2	-0.0428739
3.2625	94542.1956	1.01244353	314.050746	2.90345033	0	1.18249377	4.54905683	0.01335397	5.74955777	1.06624416	-6.9077553	17	27	6.25854444	300.777778	27.7777778	0	0	1	0	18.9135952	2	-0.0428739
4.2777	142541.204	1.18520436	442.121509	4.11744896	0	1.45341548	4.95963812	0.1707586	6.09158701	1.41547663	-6.9077553	18	24	7.59966111	301.888889	28.88888888	0	0	1	0	18.9135952	2	-0.0428739
5.2506	187978.015	1.09021798	355.068195	2.8737208	0	1.65834236	5.23633034	0.08729448	5.87231269	1.05595556	-6.9077553	19	23	5.36446667	303.555556	30.55555555	0	0	1	0	18.9135952	2	-0.0428739
6.2145	233574.871	1.05935638	336.724148	2.95141716	0	1.82688527	5.45350696	0.05860506	5.81926701	1.08262421	-6.9077553	20	23	5.81150556	303.555556	30.55555555	0	0	1	0	18.9135952	2	-0.0428739
7.2693	281715.571	1.0839373	357.332551	3.16515301	0	1.98366	5.6409015	0.08152219	5.87866966	1.15251729	-6.9077553	21	22	6.25854444	304.111111	31.11111111	0	0	1	0	18.9135952	2	-0.0428739
8.2134	326615.824	1.06352928	336.866569	2.75688016	0	2.10576697	5.78878769	0.06253271	5.81968988	1.01446233	-6.9077553	22	23	5.36446667	304.111111	31.11111111	0	0	1	0	18.9135952	2	-0.0428739
9.1305	370631.846	1.027942	306.720743	2.25116608	0	2.21162046	5.91521194	0.02853109	5.72594096	0.81189245	-6.9077553	23	24	4.91742778	303.555556	30.55555555	0	0	1	0	18.9135952	2	-0.0428739
0.0171	417.358526	0.77926932	174.068102	1.64681615	0	-4.0686768	-0.8714165	-0.2481161	5.15945236	0.49945086	-6.9077553	0	71	3.57631111	302.444444	29.44444444	0	0	0	0	30.519848	2	0.21141735
0.0621	1512.56208	0.67432617	136.381953	1.38517532	0	-2.7790093	0.41446586	-0.3925595	4.91546676	0.32654839	-6.9077553	1	70	2.68223333	303	30	0	0	0	0	30.519848	2	0.21141735
0.1233	2437.85799	0.54350068	86.8413948	1.29819657	0	-2.0931349	0.89152989	-0.6078861	4.46409492	0.26174605	-6.9077553	2	67	3.12927222	303.555556	30.55555555	0	0	0	0	30.519848	2	0.21141735
0.1971	2994.1329	0.45708824	61.6269769	1.12077304	0	-1.6240441	1.09698861	-0.7806935	4.12111594	0.1149105	-6.9077553	3	65	2.68223333	303.555556	30.55555555	0	0	0	0	30.519848	2	0.21141735
0.3456	4145.19888	0.58794741	100.818442	2.01763953	0	-1.0624732	1.42219198	-0.5294184	4.61333122	0.70242378	-6.9077553	4	67	4.02335	303	30	0	0	0	1	30.519848	2	0.21141735
0.5391	7734.95666	0.75366268	168.181523	2.19919277	0	-0.6178542	2.04587916	-0.2814844	5.12504983	0.78854498	-6.9077553	5	58	4.47038889	304.666667	31.66666666	0	0	0	1	30.519848	2	0.21141735
0.7812	12667.8725	0.75890257	163.675752	2.63441076	0	-0.2469241	2.539148	-0.2745651	5.09789346	0.96903906	-6.9077553	6	55	5.36446667	305.222222	32.2222222	0	0	0	1	30.519848	2	0.21141735
0.9891	18023.9914	0.71676966	143.147524	1.70628852	0	-0.0109598	2.89175921	-0.3316066	4.96388272	0.53490645	-6.9077553	7	54	4.47038889	305.222222	32.2222222	0	0	0	1	30.519848	2	0.21141735
1.1241	24797.0329	0.67918243	141.313737	1.07352722	0	0.11698272	3.21076433	-0.3853942	4.95098958	0.07188077	-6.9077553	8	60	2.68223333	304.111111	31.11111111	0	0	0	1	30.519848	2	0.21141735
1.2159	30531.048	0.8118524	176.286822	1.29432244	0	0.19548454	3.41877689	-0.2072057	5.17211801	0.25875966	-6.9077553	9	67	1.34111667	302.444444	29.44444444	0	0	0	1	30.519848	2	0.21141735
1.2933	36478.0701	0.69690381	127.67669	1.03813952	0	0.25719709	3.59673868	-0.359674	4.84950904	0.03839299	-6.9077553	10	73	2.23519444	300.777778	27.7777778	0	0	0	1	30.519848	2	0.21141735
1.3365	38566.407	0.55113633	74.4185282	0.81081581	0	0.29005426	3.65240754	-0.5939603	4.30971838	-0.2084818	-6.9077553	11	79	1.34111667	299.666667	26.66666666	0	0	0	1	30.519848	2	0.21141735
1.3518	39180.4975	0.38348268	34.261787	0.52319156	0	0.30143704	3.66820463	-0.9558565	3.53405984	-0.6458981	-6.9077553	12	83	1.34111667	299.111111	26.11111111	0	0	0	1	30.519848	2	0.21141735
1.3707	39882.3213	0.25758017	14.248073	0.41083368	0	0.31532156	3.68595822	-1.3525495	2.65669185	-0.8871357	-6.9077553	13	85	2.68223333	298.555556	25.55555555	0	0	0	1	30.519848	2	0.21141735
1.3824	40408.6845	0.2298252	11.5542755	0.34376016	0	0.32382112	3.69906947	-1.4660946	2.44714209	-1.0649063	-6.9077553	14	86	2.68223333	298	25	0	0	0	1	30.519848	2	0.21141735
1.3905	40759.5953	0.23775718	11.6126268	0.30985392	0	0.32966339	3.70771581	-1.4323082	2.45217913	-1.1684322	-6.9077553	15	90	2.23519444	298	25	0	0	0	1	30.519848	2	0.21141735
1.4013	41242.0969	0.21195191	9.599816	0.29491007	0	0.33740038	3.71948375	-1.5466889	2.2618481	-1.2176997	-6.9077553	16	91	3.57631111	298	25	0	0	0	1	30.519848	2	0.21141735
1.4094	41593.0051	0.21818201	9.59093391	0.29699259	0	0.34316408	3.72795605	-1.5178528	2.26092253	-1.2106866	-6.9077553	17	90	3.12927222	298	25	0	0	0	1	30.519848	2	0.21141735
1.4175	41987.7811	0.22000755	9.71992577	0.27173016	0	0.34889476	3.73740247	-1.5095584	2.27428086	-1.2992724	-6.9077553	18	91	2.68223333	298	25	0	0	0	1	30.519848	2	0.21141735
1.4292	42558.0075	0.22073814	9.99418875	0.31367603	0	0.35711485	3.75089152	-1.5062582	2.30210385	-1.1562117	-6.9077553	19	93	1.34111667	298	25	0	0	0	1	30.519848	2	0.21141735
1.4409	43128.2406	0.24032316	12.1159586	0.36273556	0	0.36526792	3.7642012	-1.4216183	2.49460601	-1.0113281	-6.9077553	20	93	2.23519444	298	25	0	0	0	1	30.519848	2	0.21141735
1.4625	44005.5146	0.28215075	22.2005793	0.61430903	0	0.3801473	3.78433768	-1.2617759	3.10016343	-0.4856306	-6.9077553	21	88	3.12927222	299.111111	26.11111111	0	0	0	1	30.519848	2	0.21141735
1.5903	47578.9648	0.44708725	60.7621451	1.03931201	0	0.46392268	3.86241177	-0.8027673	4.10698344	0.03952068	-6.9077553	22	83	2.68223333	300.222222	27.2222222	0	0	0	1	30.519848	2	0.21141735
1.7919	52538.8039	0.53280847	77.5057722	1.46940695	0	0.58327651	3.96157105	-0.6277182	4.35036532	0.3855392	-6.9077553	23	80	4.02335	300.777778	27.7777778	0	0	0	1	30.519848	2	0.21141735
0.0387	1463.19876	1.03542564	300.071173	3.04067248	0	-3.2519157	0.38130817	0.03577791	5.70402302	1.11240752	-6.9077553	0	71	3.57631111	302.444444	29.44444444	0	0	0	0	34.0724638	2	0.49277013
0.1125	4769.76385	0.88293904	211.18357	1.42172883	0	-2.1848021	1.56250643	-0.1233672	5.35273249	0.35257674	-6.9077553	1	70	2.68223333	303	30	0	0	0	0	34.0724638	2	0.49277013
0.1881	8184.79043	0.86910647	193.947417	1.28162675	0	-1.6707815	2.10239978	-0.1391397	5.26759223	0.24891012	-6.9077553	2	67	3.12927222	303.555556	30.55555555	0	0	0	0	34.0724638	2	0.49277013
0.2682	10990.9385	0.81305275	177.011302	1.46602691	0	-1.3160223	2.39716214	-0.2057301	5.17621923	0.38323784	-6.9077553	3	65	2.68223333	303.555556	30.55555555	0	0	0	1	34.0724638	2	0.49277013
0.4257	18606.2031	1.07238133	328.698571	3.00878627	0	-0.8540204	2.92354877	0.07081379	5.79514418	1.10186907	-6.9077553	4	67	4.02335	303	30	0	0	0	1	34.0724638	2	0.49277013
0.7056	34865.002	1.22808269	427.147759	3.39165887	0	-0.3487068	3.5515122	0.20626811	6.05713233	1.22161394	-6.9077553	5	58	4.47038889	304.666667	31.66666666	0	0	0	1	34.0724638	2	0.49277013
1.2141	49659.0545	1.20134083	428.099336	3.78458673	0	0.19400306	3.90520088	0.18427035	6.0593576	1.33120089	-6.9077553	6	55	5.36446667	305.222222	32.2222222	0	0	0	1	34.0724638	2	0.49277013
1.6992	59344.5868	1.0463883	330.614274	2.72278036	0	0.53015755	4.08337776	0.04629974	5.80095539	1.00202075	-6.9077553	7	54	4.47038889	305.222222	32.2222222	0	0	0	1	34.0724638	2	0.49277013
1.9944	71544.9451	0.88856562	231.504049	1.69230354	0	0.69034325	4.27033983	-0.117022	5.44460168	0.52668138	-6.9077553	8	60	2.68223333	304.111111	31.11111111	0	0	0	1	34.0724638	2	0.49277013
2.1672	78525.2987	0.81976207	179.863932	1.39491351	0	0.77343601	4.36343358	-0.197522	5.19220619	0.33354905	-6.9077553	9	67	1.34111667	302.444444	29.44444444	0	0	0	1	34.0724638	2	0.49277013
2.3607	86811.5586	0.80693771	174.051199	1.44784787	0	0.85895819	4.4637513	-0.2132703	5.15935525	0.37076867	-6.9077553	10	73	2.23519444	300.777778	27.7777778	0	0	0	1	34.0724638	2	0.49277013
2.5074	92922.3668	0.60512524	96.7420437	1.12216168	0	0.91924636	4.53177514	-0.5006686	4.57205843	0.11614764	-6.9077553	11	79	1.34111667	299.666667	26.66666666	0	0	0	1	34.0724638	2	0.49277013
2.583	96212.1498	0.34704653	29.7513195	0.60255759	0	0.94895151	4.56656604	-1.0554191	3.3929071	-0.5049138	-6.9077553	12	83	1.34111667	299.111111	26.11111111	0	0	0	1	34.0724638	2	0.49277013
2.6469	98975.5737	0.27954585	17.0388098	0.45348992	0	0.97338914	4.59488319	-1.2710181	2.83555236	-0.7885795	-6.9077553	13	85	2.68223333	298.555556	25.55555555	0	0	0	1	34.0724638	2	0.49277013
2.7144	101826.714	0.2516449	14.1910091	0.4409289	0	0.99857093	4.62328231	-1.3757703	2.65267907	-0.8166063	-6.9077553	14	86	2.68223333	298	25	0	0	0	1	34.0724638	2	0.49277013
2.7693	104283.081	0.24091303	12.5140921	0.37482071	0	1.01859458	4.64711872	-1.419177	2.52693529	-0.9786431	-6.9077553	15	90	2.23519444	298	25	0	0	0	1	34.0724638	2	0.49277013
2.8314	106783.318	0.27952037	21.0609202	0.42517319	0	1.04077129	4.67081108	-1.2711089	3.04746668	-0.8529095	-6.9077553	16	91	3.57631111	298	25	0	0	0	1	34.0724638	2	0.49277013
2.8989	109546.738	0.26303563	18.1864355	0.46291819	0	1.06433135	4.69636042	-1.3316712	2.900731	-0.7680471	-6.9077553	17	90	3.12927222	298	25	0	0	0	1	34.0724638	2	0.49277013
2.9628	112134.701	0.31492733	33.9748501	0.53308578	0	1.08613477	4.71970976	-1.1522431	3.52564998	-0.6271988	-6.9077553	18	91	2.68223333	298	25	0	0	0	1	34.0724638	2	0.49277013
3.0249	114284.025	0.27156548	20.0384327	0.42914111	0	1.10687803	4.73869555	-1.2998764	2.99770197	-0.843642	-6.9077553	19	93	1.34111667	298	25	0	0	0	1	34.0724638	2	0.49277013
3.0852	116608.804	0.25885559	16.8681258	0.43400838	0	1.12661649	4.75883335	-1.3476292	2.82548508	-0.83239	-6.9077553	20	93	2.23519444	298	25	0	0	0	1	34.0724638	2	0.49277013
3.2022	120600.415	0.44706368	62.6673227	0.79388029	0	1.16383807	4.79249101	-0.8028199	4.1378561	-0.2295638	-6.9077553	21	88	3.12927222	299.111111	26.11111111	0	0	0	1	34.0724638	2	0.49277013
3.6531	129685.553	0.60726801	124.42306	1.64024661	0	1.29557612	4.86512041	-0.4971397	4.82369557	0.49545608	-6.9077553	22	83	2.68223333	300.222222	27.2222222	0	0	0	1	34.0724638	2	0.49277013
4.4424	143496.704	0.71392892	163.176614	2.10243495	0	1.49119477	4.96631904	-0.3355722	5.09483927	0.7435717	-6.9077553	23	80	4.02335	300.777778	27.7777778	0	0	0	1	34.0724638	2	0.49277013

0.0459	3350.23615	1.27894386	460.238477	2.84708527	0	-3.0812902	1.20932928	0.24681622	6.13174696	1.04664693	-6.9077553	0	71	3.57631111	302.444444	29.44444444	0	0	0	0	42.288408	2	-0.4111751
0.198	9569.63799	1.01108348	288.47959	3.18841119	0	-1.6194882	2.25869987	0.01201106	5.66462781	1.15983632	-6.9077553	1	70	2.68223333	303	30	0	0	0	0	42.288408	2	-0.4111751
0.4563	22138.2823	1.0511901	305.036368	2.67086526	0	-0.7846048	3.09735351	0.0508738	5.72043429	0.98277683	-6.9077553	2	67	3.12927222	303.555556	30.55555555	0	0	0	0	42.288408	2	-0.4111751
0.7272	35889.7625	0.99440697	271.13982	2.07377143	0	-0.3185537	3.58047995	-0.0046036	5.60263832	0.72985099	-6.9077553	3	65	2.68223333	303.555556	30.55555555	0	0	0	0	42.288408	2	-0.4111751
1.035	47505.1255	0.85691941	196.664885	1.7801039	0	0.03440143	3.86085866	-0.1532451	5.28150627	0.57723334	-6.9077553	4	67	4.02335	303	30	0	0	1	0	42.288408	2	-0.4111751
1.3977	62311.0543	0.8625168	197.414431	1.74280633	0	0.33482803	4.13215489	-0.1467419	5.28531029	0.55607027	-6.9077553	5	58	4.47038889	304.666667	31.66666666	0	0	1	0	42.288408	2	-0.4111751
1.9395	81341.1844	0.89476677	225.976952	2.57983743	0	0.66243021	4.39866476	-0.1100752	5.42043744	0.94811393	-6.9077553	6	55	5.36446667	305.222222	32.2222222	0	0	1	0	42.288408	2	-0.4111751
2.4435	99184.8472	0.81856673	187.678292	1.90579638	0	0.89343144	4.59699533	-0.1989795	5.23473461	0.64542454	-6.9077553	7	54	4.47038889	305.222222	32.2222222	0	0	1	0	42.288408	2	-0.4111751
2.8278	116442.618	0.84957607	197.055804	1.38488484	0	1.03949902	4.75740719	-0.1618414	5.28349203	0.32633881	-6.9077553	8	60	2.68223333	304.111111	31.11111111	0	0	1	0	42.288408	2	-0.4111751
3.2139	135727.846	0.9216586	231.432146	1.64776981	0	1.16748515	4.91065911	-0.080496	5.44429105	0.50002944	-6.9077553	9	67	1.34111667	302.444444	29.44444444	0	0	1	0	42.288408	2	-0.4111751
3.6324	156068.791	0.90106792	216.554637	1.70083257	0	1.28989359	5.05030329	-0.1030655	5.3778475	0.53170565	-6.9077553	10	73	2.23519444	300.777778	27.7777778	0	0	1	0	42.288408	2	-0.4111751
3.9078	168534.534	0.68306797	121.0763	1.25339304	0	1.36297456	5.12714661	-0.379698	4.79642918	0.22665182	-6.9077553	11	79	1.34111667	299.666667	26.66666666	0	0	1	0	42.288408	2	-0.4111751
4.0275	173754.313	0.34745023	27.7721544	0.58627652	0	1.39314584	5.15764806	-1.0542599	3.32406988	-0.5322595	-6.9077553	12	83	1.34111667	299.111111	26.11111111	0	0	1	0	42.288408	2	-0.4111751
4.1391	178667.057	0.31734525	30.9081401	0.6059251	0	1.42047837	5.18552966	-1.1446188	3.43105194	-0.4993499	-6.9077553	13	85	2.68223333	298.555556	25.55555555	0	0	1	0	42.288408	2	-0.4111751
4.2444	183275.159	0.30497373	28.5968237	0.56926271	0	1.44560047	5.21099408	-1.184256	3.35333062	-0.5616581	-6.9077553	14	86	2.68223333	298	25	0	0	1	0	42.288408	2	-0.4111751
4.3308	186915.838	0.28721366	20.7590052	0.57018574	0	1.46575228	5.2306638	-1.2440532	3.03302831	-0.5600408	-6.9077553	15	90	2.23519444	298	25	0	0	1	0	42.288408	2	-0.4111751
4.4073	190030.169	0.26926178	21.4670539	0.50997763	0	1.48326226	5.24718811	-1.3083643	3.06656597	-0.6714295	-6.9077553	16	91	3.57631111	298	25	0	0	1	0	42.288408	2	-0.4111751
4.4721	192574.267	0.22990415	12.5082058	0.44122193	0	1.4978581	5.26048708	-1.4657526	2.52646484	-0.8159434	-6.9077553	17	90	3.12927222	298	25	0	0	1	0	42.288408	2	-0.4111751
4.5162	194548.139	0.22805305	11.1685704	0.34899536	0	1.50767093	5.27068477	-1.4738016	2.41319315	-1.0498354	-6.9077553	18	91	2.68223333	298	25	0	0	1	0	42.288408	2	-0.4111751
4.5621	196565.868	0.22440018	10.5656391	0.32228526	0	1.51778304	5.28100267	-1.4898779	2.35770179	-1.1292202	-6.9077553	19	93	1.34111667	298	25	0	0	1	0	42.288408	2	-0.4111751
4.6053	198188.835	0.21823881	10.1827113	0.3288909	0	1.52720781	5.28922533	-1.5175937	2.32078951	-1.1089933	-6.9077553	20	93	2.23519444	298	25	0	0	1	0	42.288408	2	-0.4111751
4.7205	201611.149	0.35330832	37.4367027	1.05354738	0	1.55191473	5.3063458	-1.0375878	3.62267829	0.05311165	-6.9077553	21	88	3.12927222	299.111111	26.11111111	0	0	1	0	42.288408	2	-0.4111751
5.1021	211064.89	0.54706196	93.156379	1.2517775	0	1.62965222	5.35217036	-0.6013669	4.53429031	0.22536309	-6.9077553	22	83	2.68223333	300.222222	27.2222222	0	0	1	0	42.288408	2	-0.4111751
5.6988	225864.324	0.65278973	129.334842	1.62560354	0	1.74025563	5.41993891	-0.4249695	4.86241244	0.48649412	-6.9077553	23	80	4.02335	300.777778	27.7777778	0	0	1	0	42.288408	2	-0.4111751
0.0099	0	0.56151919	86.0996826	2.12463913	0	-4.6152205	-6.9077553	-0.57533	4.45551734	0.75407252	-6.9077553	0	71	3.57631111	302.444444	29.44444444	0	0	0	0	18.9135952	2	-0.0428739
0.045	0	0.45108203	53.722049	1.58175268	0	-3.1010928	-6.9077553	-0.7938916	3.98384213	0.45916553	-6.9077553	1	70	2.68223333	303	30	0	0	0	0	18.9135952	2	-0.0428739
0.1422	355.983156	0.57556442	91.7600911	1.7714191	0	-1.9505208	-1.0300667	-0.5506682	4.51918836	0.57234534	-6.9077553	2	67	3.12927222	303.555556	30.55555555	0	0	0	0	18.9135952	2	-0.0428739
0.2745	3061.04969	0.70371013	158.920015	1.7052822	0	-1.292804	1.11908452	-0.3499687	5.06840732	0.53431685	-6.9077553	3	65	2.68223333	303.555556	30.55555555	0	0	1	0	18.9135952	2	-0.0428739
0.4878	10292.9019	0.8038614	220.148883	2.10839612	0	-0.7178498	2.33155168	-0.2170852	5.3943086	0.74640171	-6.9077553	4	67	4.02335	303	30	0	0	1	0	18.9135952	2	-0.0428739
0.6705	16807.9487	0.81293551	221.748403	1.88554179	0	-0.3997316	2.8219114	-0.2058741	5.40154793	0.63474541	-6.9077553	5	58	4.47038889	304.666667	31.66666666	0	0	1	0	18.9135952	2	-0.0428739
0.9045	24352.1041	0.85154836	228.488657	2.16641353	0	-0.100373	3.19265932	-0.1595253	5.43149094	0.77353453	-6.9077553	6	55	5.36446667	305.222222	32.2222222	0	0	1	0	18.9135952	2	-0.0428739
1.1862	33842.6517	0.83879006	214.422973	2.19652306	0	0.17075492	3.52175144	-0.1746033	5.36795524	0.78733085	-6.9077553	7	54	4.47038889	305.222222	32.2222222	0	0	1	0	18.9135952	2	-0.0428739
1.4022	42932.625	0.84309514	215.030664	1.61201274	0	0.33804243	3.75965532	-0.1694901	5.37078529	0.47810369	-6.9077553	8	60	2.68223333	304.111111	31.11111111	0	0	1	0	18.9135952	2	-0.0428739
1.5273	48622.1745	0.85510517	192.877614	1.42985118	0	0.42350147	3.88410026	-0.155362	5.26206105	0.35826949	-6.9077553	9	67	1.34111667	302.444444	29.44444444	0	0	1	0	18.9135952	2	-0.0428739
1.6362	53482.3037	0.75367409	148.984472	1.25003115	0	0.49237648	3.97936952	-0.2814693	5.0038488	0.22396813	-6.9077553	10	73	2.23519444	300.777778	27.7777778	0	0	1	0	18.9135952	2	-0.0428739
1.701	56333.4506	0.57383569	80.3395521	0.86771329	0	0.53121631	4.03130626	-0.553671	4.3862745	-0.1407421	-6.9077553	11	79	1.34111667	299.666667	26.66666666	0	0	1	0	18.9135952	2	-0.0428739
1.7361	57956.4063	0.35426763	29.599312	0.56063319	0	0.55164122	4.05970837	-1.0348839	3.3877849	-0.5769063	-6.9077553	12	83	1.34111667	299.111111	26.11111111	0	0	1	0	18.9135952	2	-0.0428739
1.7838	60105.7356	0.27300998	15.9692268	0.41360304	0	0.57874592	4.09612191	-1.2945907	2.77072616	-0.8804337	-6.9077553	13	85	2.68223333	298.555556	25.55555555	0	0	1	0	18.9135952	2	-0.0428739
1.8306	62298.924	0.27546138	16.3335301	0.45406521	0	0.60464378	4.13196021	-1.2856841	2.79328128	-0.7873145	-6.9077553	14	86	2.68223333	298	25	0	0	1	0	18.9135952	2	-0.0428739
1.8828	64623.7088	0.27887085	16.8473448	0.45344935	0	0.63276003	4.16859683	-1.273427	2.82425242	-0.7886688	-6.9077553	15	90	2.23519444	298	25	0	0	1	0	18.9135952	2	-0.0428739
1.9458	67518.7105	0.26804088	15.6178327	0.44262071	0	0.6656732	4.21241956	-1.312892	2.74847741	-0.8127853	-6.9077553	16	91	3.57631111	298	25	0	0	1	0	18.9135952	2	-0.0428739
2.0088	70413.7268	0.27553068	16.7688738	0.48816496	0	0.69753753	4.25440243	-1.2854335	2.81958405	-0.7150555	-6.9077553	17	90	3.12927222	298	25	0	0	1	0	18.9135952	2	-0.0428739
2.0772	73528.0522	0.26929517	15.9548886	0.46869434	0	0.73102083	4.2976806	-1.3082407	2.76982795	-0.7556731	-6.9077553	18	91	2.68223333	298	25	0	0	1	0	18.9135952	2	-0.0428739
2.1384	76335.3361	0.2634718	14.8843913	0.44578861	0	0.76005789	4.33514905	-1.3300207	2.70038028	-0.8056697	-6.9077553	19	93	1.34111667	298	25	0	0	1	0	18.9135952	2	-0.0428739
2.1951	78703.9808	0.25314183	13.9479351	0.41222863	0	0.7862276	4.36570644	-1.3698628	2.63540317	-0.8837543	-6.9077553	20	93	2.23519444	298	25	0	0	1	0	18.9135952	2	-0.0428739
2.2842	82137.3173	0.43720819	58.1101592	1.00390332	0	0.82601585	4.40840462	-0.8250612	4.06235771	0.00489134	-6.9077553	21	88	3.12927222	299.111111	26.11111111	0	0	1	0	18.9135952	2	-0.0428739
2.6712	94456.0385	0.7008733	150.256232	1.78886804	0	0.98252781	4.54814511	-0.3540024	5.0123487	0.5821419	-6.9077553	22	83	2.68223333	300.222222	27.2222222	0	0	1	0	18.9135952	2	-0.0428739
3.1842	110570.145	0.77664356	186.080606	2.12464321	0	1.15820108	4.70565916	-0.251487	5.22618532	0.75407444	-6.9077553	23	80	4.02335	300.777778	27.7777778	0	0	1	0	18.9135952	2	-0.0428739
0.0108	360.875881	0.81909657	187.239036	1.46881658	0	-4.5282091	-1.016454	-0.1983332	5.23239141	0.38513762	-6.9077553	0	68	2.68223333	303.555556	30.55555555	0	0	0	0	30.519848	2	0.21141735
0.0234	887.241231	0.68032088	118.636736	1.07729476	0	-3.7550193	-0.1185119	-0.3837219	4.77607462	0.07538087	-6.9077553	1	73	2.23519444	301.888889	28.88888888	0	0	0	0	30.519848	2	0.21141735
0.0405	1413.60669	0.62363745	101.162957	1.15032477	0	-3.2064533	0.34685154	-0.4705839	4.61674254	0.14091325	-6.9077553	2	77	1.78815556	300.777778	27.7777778	0	0	0	0	30.519848	2	0.21141735
0.0441	1501.3343	0.47755677	53.7680359	0.88203949	0	-3.1212955	0.4070201	-0.7369804	3.98469776	-0.1243854	-6.9077553	3	80	1.34111667	300.222222	27.2222222	0	0	0	0	30.519848	2	0.21141735
0.0441	1501.3343	0	0	0	0	-3.1212955	0.4070201	-6.9077553	-6.9077553	-6.9077553	-6.9077553	4	84	2.68223333	299.666667	26.66666666	0	0	0	0	30.519848	2	0.21141735
0.0441	1501.3343	0	0	0	0	-3.1212955	0.4070201	-6.9077553	-6.9077553	-6.9077553	-6.9077553	5	88	1.78815556	299.111111	26.11111111	0	0	0	0	30.519848	2	0.21141735
0.0441	1501.3343	0	0	0	0	-3.1212955	0.4070201	-6.9077553	-6.9077553	-6.9077553	-6.9077553	6	89	2.68223333	299.666667	26.66666666	0	0	0	0	30.519848	2	0.21141735
0.0441	1501.3343	0	0	0	0	-3.1212955	0.4070201	-6.9077553	-6.9077553	-6.9077553	-6.9077553	7	86	4.02335	300.222222	27.2222222	0	0	0	0	30.519848	2	0.21141735
0.0441	1501.3343	0	0	0	0	-3.1212955	0.4070201	-6.9077553	-6.9077553	-6.9077553	-6.9077553	8	87	3.57631111	300.222222	27.2222222	0	0	0	0	30.519848	2	0.21141735
0.0441	1501.3343	0	0	0	0	-3.1212955	0.4070201	-6.9077553	-6.9077553	-6.9077553	-6.9077553	9	87	3.57631111	300.222222	27.2222222	0	0	0	0	30.519848	2	0.21141735
0.0441	1501.3343	0	0	0	0	-3.1212955	0.4070201	-6.9077553	-6.9077553	-6.9077553	-6.9077553	10	87	4.02335	300.222222	27.2222222	0	0	0	0	30.519848	2	0.21141735
0.0441	1501.3343	0	0	0	0	-3.1212955	0.4070201	-6.9077553	-6.9077553	-6.9077553	-6.9077553	11	88	2.68223333	300.222222	27.2222222	0	0	0	0	30.519848	2	0.21141735
0.0459	1545.19801	0.01672199	0.03847351	0.3631584	0	-3.0812902	0.43579902	-4.0329488	-3.2321254	-1.0101663	-6.9077553	12	84	3.57631111	300.777778	27.7777778	0	0	0	0	30.519848	2	0.21141735
0.09	1747.4661	0.34414237	30.7283163	1.25872702	0	-2.4079456	0.55873889	-1.0637983	3.42521713	0.23089504	-6.9077553	13	78	3.57631111	301.888889	28.88888888	0	0	0	0	30.519848	2	0.21141735

0.1755	2181.93888	0.40730366	44.9118935	1.58211863	0	-1.7401162	0.78067208	-0.8957441	3.80472491	0.45939672	-6.9077553	14	76	4.02335	301.888889	28.88888888	0	0	0	0	30.519848	2	0.21141735
0.261	3164.21858	0.53663736	81.0177786	1.49340162	0	-1.3432349	1.15222212	-0.620571	4.39468096	0.40172587	-6.9077553	15	66	4.02335	304.111111	31.11111111	0	0	0	0	30.519848	2	0.21141735
0.3519	3769.8417	0.57559555	86.5790726	1.58496033	0	-1.0444082	1.32729824	-0.5506142	4.46106968	0.46119011	-6.9077553	16	63	3.57631111	304.666667	31.66666666	0	0	0	1	30.519848	2	0.21141735
0.5355	6750.77476	0.75970362	168.442727	2.34724815	0	-0.6245544	1.9098054	-0.2735115	5.12660173	0.85366958	-6.9077553	17	57	5.81150556	305.777778	32.7777778	0	0	0	1	30.519848	2	0.21141735
0.8028	13665.3321	0.8513275	213.808804	2.87367757	0	-0.2196497	2.61493529	-0.1597844	5.36508685	1.05594052	-6.9077553	18	56	5.36446667	305.777778	32.7777778	0	0	0	1	30.519848	2	0.21141735
1.1061	22042.9682	0.71619155	141.069826	2.14296661	0	0.10084031	3.09303902	-0.3324123	4.94926207	0.76265767	-6.9077553	19	55	5.81150556	306.333333	33.33333333	0	0	0	1	30.519848	2	0.21141735
1.323	30760.5611	0.6764099	128.132423	1.31057321	0	0.27990189	3.42626589	-0.3894787	4.85307209	0.27122734	-6.9077553	20	59	3.12927222	305.777778	32.7777778	0	0	0	1	30.519848	2	0.21141735
1.4814	38356.6905	0.70452638	145.435502	1.21418702	0	0.39298759	3.64695504	-0.3488111	4.97973958	0.19489799	-6.9077553	21	59	3.12927222	305.222222	32.2222222	0	0	0	1	30.519848	2	0.21141735
1.5966	44752.357	0.73206657	144.324112	0.99536073	0	0.46787637	3.80116646	-0.3105188	4.97206848	-0.0036459	-6.9077553	22	65	1.78815556	304.666667	31.66666666	0	0	0	1	30.519848	2	0.21141735
1.6758	51205.2671	0.69871053	131.863226	0.97935243	0	0.51629066	3.93586193	-0.3570886	4.8817728	-0.0198431	-6.9077553	23	70	1.34111667	304.111111	31.11111111	0	0	0	1	30.519848	2	0.21141735
0.0198	912.551532	1.02286884	290.75658	2.14173617	0	-3.9220733	-0.0904155	0.02358843	5.67248986	0.76208359	-6.9077553	0	68	2.68223333	303.555556	30.55555555	0	0	0	0	34.0724638	2	0.49277013
0.0423	1658.23885	0.72135597	129.608711	0.95782829	0	-3.1629682	0.50635897	-0.3252372	4.86452771	-0.0420433	-6.9077553	1	73	2.23519444	301.888889	28.88888888	0	0	0	0	34.0724638	2	0.49277013
0.0576	2316.19569	0.54666749	77.2273093	0.93884973	0	-2.8542327	0.8403577	-0.6020869	4.34676609	-0.0620353	-6.9077553	2	77	1.78815556	300.777778	27.7777778	0	0	0	0	34.0724638	2	0.49277013
0.0729	2974.15224	0.48714739	57.6494212	0.7548698	0	-2.6186666	1.09029521	-0.7171379	4.05439755	-0.2798861	-6.9077553	3	80	1.34111667	300.222222	27.2222222	0	0	0	0	34.0724638	2	0.49277013
0.09	3675.97263	0.34689813	28.1973074	0.48721399	0	-2.4079456	1.30208976	-1.0558456	3.33926195	-0.7170015	-6.9077553	4	84	2.68223333	299.666667	26.66666666	0	0	0	0	34.0724638	2	0.49277013
0.1035	4290.06587	0.29502274	18.7711629	0.41993395	0	-2.2681837	1.45653516	-1.217319	2.93237508	-0.8652793	-6.9077553	5	88	1.78815556	299.111111	26.11111111	0	0	0	0	34.0724638	2	0.49277013
0.1233	5211.20522	0.2668384	15.119923	0.37349866	0	-2.0931349	1.65100303	-1.3173715	2.71607941	-0.9821671	-6.9077553	6	89	2.68223333	299.666667	26.66666666	0	0	0	0	34.0724638	2	0.49277013
0.1422	6088.48104	0.26227077	14.6084638	0.36587981	0	-1.9505208	1.80656286	-1.3345722	2.68166953	-1.002721	-6.9077553	7	86	4.02335	300.222222	27.2222222	0	0	0	0	34.0724638	2	0.49277013
0.1593	6878.02876	0.24974823	13.8625793	0.34940587	0	-1.8369661	1.92847747	-1.3833059	2.62926521	-1.0486632	-6.9077553	8	87	3.57631111	300.222222	27.2222222	0	0	0	0	34.0724638	2	0.49277013
0.1728	7492.12173	0.25780293	14.2127146	0.36292604	0	-1.7556204	2.0139855	-1.3516884	2.65420732	-1.0108046	-6.9077553	9	87	3.57631111	300.222222	27.2222222	0	0	0	0	34.0724638	2	0.49277013
0.1872	8150.0786	0.23489091	12.1040649	0.35024369	0	-1.6755777	2.09815026	-1.4443858	2.49362396	-1.046275	-6.9077553	10	87	4.02335	300.222222	27.2222222	0	0	0	0	34.0724638	2	0.49277013
0.2043	8939.62739	0.2672663	16.4821279	0.35087661	0	-1.5881658	2.19060576	-1.3157751	2.80233731	-1.0444747	-6.9077553	11	88	2.68223333	300.222222	27.2222222	0	0	0	0	34.0724638	2	0.49277013
0.2745	11840.4288	0.76958642	191.142916	1.54065607	0	-1.292804	2.4716043	-0.2606035	5.25302663	0.43285721	-6.9077553	12	84	3.57631111	300.777778	27.7777778	0	0	0	0	34.0724638	2	0.49277013
0.4716	17709.3759	0.85361366	223.516445	2.09773665	0	-0.7516241	2.87415068	-0.1571058	5.40948947	0.74133557	-6.9077553	13	78	3.57631111	301.888889	28.88888888	0	0	0	0	34.0724638	2	0.49277013
0.819	26448.215	1.0749711	354.04647	3.1306048	0	-0.1996712	3.27522648	0.0732236	5.869431	1.14154559	-6.9077553	14	76	4.02335	301.888889	28.88888888	0	0	0	1	34.0724638	2	0.49277013
1.3815	35491.6832	1.27843574	498.4567	3.85485625	0	0.32316987	3.56932657	0.24641915	6.21151873	1.3495931	-6.9077553	15	66	4.02335	304.111111	31.11111111	0	0	0	1	34.0724638	2	0.49277013
1.9782	48872.6102	1.11396433	361.320631	2.99869976	0	0.68218734	3.88923758	0.10882241	5.88976851	1.0985122	-6.9077553	16	63	3.57631111	304.666667	31.66666666	0	0	0	1	34.0724638	2	0.49277013
2.5857	67900.5262	1.04796622	324.470903	2.75266602	0	0.94999626	4.21805851	0.04780513	5.78219895	1.01293312	-6.9077553	17	57	5.81150556	305.777778	32.7777778	0	0	0	1	34.0724638	2	0.49277013
3.123	85228.0551	0.9149175	230.126204	1.95234841	0	1.13879408	4.4453424	-0.087829	5.43863222	0.66954503	-6.9077553	18	56	5.36446667	305.777778	32.7777778	0	0	0	1	34.0724638	2	0.49277013
3.8718	114651.644	1.04113461	308.845928	3.31654974	0	1.35371952	4.74190707	0.04127112	5.73284578	1.19922648	-6.9077553	19	55	5.81150556	306.333333	33.33333333	0	0	0	1	34.0724638	2	0.49277013
4.6503	137607.858	0.87486001	215.121315	2.4209244	0	1.53693173	4.9244153	-0.132549	5.37120677	0.88456243	-6.9077553	20	59	3.12927222	305.777778	32.7777778	0	0	0	1	34.0724638	2	0.49277013
5.1912	150860.829	0.75984646	168.26777	1.64357855	0	1.64696488	5.01636438	-0.2733237	5.12556252	0.49748415	-6.9077553	21	59	3.12927222	305.222222	32.2222222	0	0	0	1	34.0724638	2	0.49277013
5.553	163350.672	0.77188528	173.971412	1.4230746	0	1.71433832	5.09590538	-0.2576247	5.15889674	0.3535222	-6.9077553	22	65	1.78815556	304.666667	31.66666666	0	0	0	1	34.0724638	2	0.49277013
5.7645	172248.773	0.79082973	169.078989	1.29760742	0	1.75171842	5.14894559	-0.2334089	5.13037191	0.26129248	-6.9077553	23	70	1.34111667	304.111111	31.11111111	0	0	0	1	34.0724638	2	0.49277013
0.0351	2312.51448	1.15533457	368.930715	2.40016034	0	-3.3495541	0.83876779	0.14525515	5.91061157	0.87595209	-6.9077553	0	68	2.68223333	303.555556	30.55555555	0	0	0	0	42.288408	2	-0.4111751
0.1062	6077.68515	0.90183181	221.462866	1.75007591	0	-2.2424312	1.80478841	-0.102219	5.40025944	0.5602304	-6.9077553	1	73	2.23519444	301.888889	28.88888888	0	0	0	0	42.288408	2	-0.4111751
0.1665	8974.1692	0.69079892	122.454553	1.23503136	0	-1.79276	2.19446179	-0.3684599	4.80774813	0.21190573	-6.9077553	2	77	1.78815556	300.777778	27.7777778	0	0	0	0	42.288408	2	-0.4111751
0.2205	11518.2688	0.58042704	82.890568	1.05737826	0	-1.5118576	2.44402118	-0.5422698	4.41753334	0.05673779	-6.9077553	3	80	1.34111667	300.222222	27.2222222	0	0	0	0	42.288408	2	-0.4111751
0.2574	13316.6827	0.43913157	45.2543469	0.76491598	0	-1.357124	2.58909267	-0.8206816	3.81232083	-0.2666828	-6.9077553	4	84	2.68223333	299.666667	26.66666666	0	0	0	0	42.288408	2	-0.4111751
0.2925	14939.6439	0.32761882	23.7909984	0.58144036	0	-1.2292906	2.70408528	-1.1128568	3.16934932	-0.5405285	-6.9077553	5	88	1.78815556	299.111111	26.11111111	0	0	0	0	42.288408	2	-0.4111751
0.3258	16518.7389	0.29783156	19.6490083	0.50292134	0	-1.1214716	2.80455596	-1.2078752	2.97807776	-0.6853351	-6.9077553	6	89	2.68223333	299.666667	26.66666666	0	0	0	0	42.288408	2	-0.4111751
0.3537	17746.9272	0.30254567	19.9670312	0.51309096	0	-1.0393062	2.87626873	-1.1922232	2.99413255	-0.6653551	-6.9077553	7	86	4.02335	300.222222	27.2222222	0	0	0	0	42.288408	2	-0.4111751
0.3879	19326.0222	0.27774023	16.9358264	0.46905799	0	-0.9470077	2.96150423	-1.277475	2.82949033	-0.7548992	-6.9077553	8	87	3.57631111	300.222222	27.2222222	0	0	0	0	42.288408	2	-0.4111751
0.4221	20861.256	0.25162004	13.993315	0.39144398	0	-0.862513	3.03794159	-1.3758687	2.63865118	-0.9353615	-6.9077553	9	87	3.57631111	300.222222	27.2222222	0	0	0	0	42.288408	2	-0.4111751
0.4518	22133.3056	0.25207635	14.1783105	0.41436872	0	-0.7945157	3.09712869	-1.3740641	2.6517839	-0.8785887	-6.9077553	10	87	4.02335	300.222222	27.2222222	0	0	0	0	42.288408	2	-0.4111751
0.4932	24107.1738	0.3242004	27.3433334	0.46629234	0	-0.7068405	3.18255095	-1.1233137	3.30850932	-0.7608002	-6.9077553	11	88	2.68223333	300.222222	27.2222222	0	0	1	0	42.288408	2	-0.4111751
0.5877	28497.4827	0.65193271	122.711533	1.09250568	0	-0.5315387	3.34985085	-0.4262812	4.80984449	0.08938876	-6.9077553	12	84	3.57631111	300.777778	27.7777778	0	0	1	0	42.288408	2	-0.4111751
0.8271	35928.8703	0.6994988	143.626057	1.65094774	0	-0.1898297	3.58156899	-0.3559626	4.96722006	0.50195504	-6.9077553	13	78	3.57631111	301.888889	28.88888888	0	0	1	0	42.288408	2	-0.4111751
1.1763	47014.4464	0.78047039	166.546956	1.86578432	0	0.16237392	3.85047619	-0.246578	5.11528329	0.62421734	-6.9077553	14	76	4.02335	301.888889	28.88888888	0	0	1	0	42.288408	2	-0.4111751
1.539	58933.3055	0.790997	162.639811	1.71088661	0	0.43113285	4.07642336	-0.2331977	5.09154415	0.53759604	-6.9077553	15	66	4.02335	304.111111	31.11111111	0	0	1	0	42.288408	2	-0.4111751
1.8981	72825.6715	0.79876403	166.598606	1.39568878	0	0.64085339	4.28808225	-0.2234386	5.11559336	0.33410428	-6.9077553	16	63	3.57631111	304.666667	31.66666666	0	0	1	0	42.288408	2	-0.4111751
2.4111	94246.074	0.91857878	233.277604	2.22880303	0	0.88008307	4.54591978	-0.0838396	5.45223347	0.80191325	-6.9077553	17	57	5.81150556	305.777778	32.7777778	0	0	1	0	42.288408	2	-0.4111751
3.2202	124098.089	0.94078395	253.163776	2.84676279	0	1.16944347	4.82108035	-0.0599794	5.53404056	1.0465337	-6.9077553	18	56	5.36446667	305.777778	32.7777778	0	0	1	0	42.288408	2	-0.4111751
4.0167	161825.58	0.98946775	277.84322	2.29523415	0	1.39046067	5.08652527	-0.009578	5.6270606	0.83127046	-6.9077553	19	55	5.81150556	306.333333	33.33333333	0	0	1	0	42.288408	2	-0.4111751
4.8141	197625.115	0.9579144	264.940198	2.13108781	0	1.57154911	5.28637694	-0.0419535	5.57950791	0.75710169	-6.9077553	20	59	3.12927222	305.777778	32.7777778	0	0	1	0	42.288408	2	-0.4111751
5.4648	223017.754	0.88005182	227.351876	1.88090699	0	1.69832752	5.40725587	-0.1266388	5.42650333	0.63228562	-6.9077553	21	59	3.12927222	305.222222	32.2222222	0	0	1	0	42.288408	2	-0.4111751
5.8608	236294.633	0.74219839	161.681765	1.34332886	0	1.76828611	5.4650837	-0.2967923	5.08563617	0.2958949	-6.9077553	22	65	1.78815556	304.666667	31.66666666	0	0	1	0	42.288408	2	-0.4111751
6.0291	243528.822	0.75561899	148.379011	1.1431041	0	1.79659775	5.49523941	-0.2788955	4.99977662	0.13462189	-6.9077553	23	70	1.34111667	304.111111	31.11111111	0	0	1	0	42.288408	2	-0.4111751
0.0009	0	0	0	0	0	-7.0131158	-6.9077553	-6.9077553	-6.9077553	-6.9077553	-6.9077553	0	68	2.68223333	303.555556	30.55555555	0	0	0	0	18.9135952	2	-0.0428739
0.0009	0	0	0	0	0	-7.0131158	-6.9077553	-6.9077553	-6.9077553	-6.9077553	-6.9077553	1	73	2.23519444	301.888889	28.88888888	0	0	0	0	18.9135952	2	-0.0428739
0.0009	0	0	0	0	0	-7.0131158	-6.9077553	-6.9077553	-6.9077553	-6.9077553	-6.9077553	2	77	1.78815556	300.777778	27.7777778	0	0	0	0	18.9135952	2	-0.0428739
0.0009	0	0	0	0	0	-7.0131158	-6.9077553	-6.9077553	-6.9077553	-6.9077553	-6.9077553	3	80	1.34111667	300.222222	27.2222222	0	0	0	0	18.9135952	2	-0.0428739

0.0009	0	0	0	0	0	-7.0131158	-6.9077553	-6.9077553	-6.9077553	-6.9077553	-6.9077553	4	84	2.68223333	299.666667	26.66666666	0	0	0	0	18.9135952	2	-0.0428739
0.0009	0	0	0	0	0	-7.0131158	-6.9077553	-6.9077553	-6.9077553	-6.9077553	-6.9077553	5	88	1.78815556	299.111111	26.11111111	0	0	0	0	18.9135952	2	-0.0428739
0.0009	0	0	0	0	0	-7.0131158	-6.9077553	-6.9077553	-6.9077553	-6.9077553	-6.9077553	6	89	2.68223333	299.666667	26.66666666	0	0	0	0	18.9135952	2	-0.0428739
0.0009	0	0	0	0	0	-7.0131158	-6.9077553	-6.9077553	-6.9077553	-6.9077553	-6.9077553	7	86	4.02335	300.222222	27.2222222	0	0	0	0	18.9135952	2	-0.0428739
0.0009	0	0	0	0	0	-7.0131158	-6.9077553	-6.9077553	-6.9077553	-6.9077553	-6.9077553	8	87	3.57631111	300.222222	27.2222222	0	0	0	0	18.9135952	2	-0.0428739
0.0009	0	0	0	0	0	-7.0131158	-6.9077553	-6.9077553	-6.9077553	-6.9077553	-6.9077553	9	87	3.57631111	300.222222	27.2222222	0	0	0	0	18.9135952	2	-0.0428739
0.0009	0	0	0	0	0	-7.0131158	-6.9077553	-6.9077553	-6.9077553	-6.9077553	-6.9077553	10	87	4.02335	300.222222	27.2222222	0	0	0	0	18.9135952	2	-0.0428739
0.0009	0	0	0	0	0	-7.0131158	-6.9077553	-6.9077553	-6.9077553	-6.9077553	-6.9077553	11	88	2.68223333	300.222222	27.2222222	0	0	0	0	18.9135952	2	-0.0428739
0.0009	0	0	0	0	0	-7.0131158	-6.9077553	-6.9077553	-6.9077553	-6.9077553	-6.9077553	12	84	3.57631111	300.777778	27.7777778	0	0	0	0	18.9135952	2	-0.0428739
0.0036	0	0.37611675	41.6883723	1.39623419	0	-5.6268214	-6.9077553	-0.9752005	3.73024624	0.33449471	-6.9077553	13	78	3.57631111	301.888889	28.88888888	0	0	0	0	18.9135952	2	-0.0428739
0.0423	58.4537886	0.60373701	107.894554	2.34942414	0	-3.1629682	-2.8225559	-0.5029616	4.68116367	0.8545958	-6.9077553	14	76	4.02335	301.888889	28.88888888	0	0	0	0	18.9135952	2	-0.0428739
0.1161	716.410488	0.56714118	85.5420696	1.64293838	0	-2.1533034	-0.3321071	-0.5653853	4.44901999	0.49709481	-6.9077553	15	66	4.02335	304.111111	31.11111111	0	0	0	0	18.9135952	2	-0.0428739
0.2547	3112.28576	0.66602831	132.540572	1.69522055	0	-1.3676689	1.13567868	-0.4049228	4.88689635	0.52840257	-6.9077553	16	63	3.57631111	304.666667	31.66666666	0	0	1	0	18.9135952	2	-0.0428739
0.4977	11350.0147	0.86435547	251.960648	2.69591539	0	-0.6977578	2.42930714	-0.1446149	5.52927689	0.99210867	-6.9077553	17	57	5.81150556	305.777778	32.7777778	0	0	1	0	18.9135952	2	-0.0428739
0.7452	20413.3183	0.87431358	258.309176	2.29113548	0	-0.2941026	3.01623653	-0.1331731	5.5541611	0.82948391	-6.9077553	18	56	5.36446667	305.777778	32.7777778	0	0	1	0	18.9135952	2	-0.0428739
1.0611	29405.6707	0.86525194	232.300993	2.54511211	0	0.05930611	3.38122154	-0.1435795	5.44803822	0.93456753	-6.9077553	19	55	5.81150556	306.333333	33.33333333	0	0	1	0	18.9135952	2	-0.0428739
1.359	40723.1327	0.86002693	219.227223	1.81879344	0	0.30674914	3.70682086	-0.1496295	5.3901133	0.598723	-6.9077553	20	59	3.12927222	305.777778	32.7777778	0	0	1	0	18.9135952	2	-0.0428739
1.5993	49636.1459	0.79848877	181.52553	1.52025087	0	0.46956603	3.90473946	-0.2237828	5.20140181	0.41953294	-6.9077553	21	59	3.12927222	305.222222	32.2222222	0	0	1	0	18.9135952	2	-0.0428739
1.8279	58292.5246	0.77445317	173.808917	1.38558563	0	0.60316777	4.06549102	-0.2543077	5.15796227	0.32684435	-6.9077553	22	65	1.78815556	304.666667	31.66666666	0	0	1	0	18.9135952	2	-0.0428739
2.0196	67208.3984	0.87468952	201.400106	1.5026099	0	0.70289947	4.20781309	-0.1327437	5.30529847	0.40786882	-6.9077553	23	70	1.34111667	304.111111	31.11111111	0	0	1	0	18.9135952	2	-0.0428739
0.0063	223.772595	0.91193223	224.11141	1.4419322	0	-5.0672056	-1.4926661	-0.0910936	5.41214775	0.36667729	-6.9077553	0	88	1.78815556	299.111111	26.11111111	0	0	0	0	30.519848	2	0.21141735
0.0135	530.819077	0.53994662	69.8064423	0.67856491	0	-4.3050656	-0.6314519	-0.6144347	4.24574063	-0.3863025	-6.9077553	1	89	2.68223333	299.666667	26.66666666	0	0	0	0	30.519848	2	0.21141735
0.0252	1013.32078	0.58752882	84.9947979	0.91884716	0	-3.6809113	0.0142192	-0.5301294	4.44260182	-0.0835478	-6.9077553	2	86	4.02335	300.222222	27.2222222	0	0	0	0	30.519848	2	0.21141735
0.0369	1276.50341	0.59839909	87.1207369	0.94646733	0	-3.2995437	0.24490771	-0.5118276	4.46730642	-0.0539628	-6.9077553	3	87	3.57631111	300.222222	27.2222222	0	0	0	0	30.519848	2	0.21141735
0.0396	1320.36712	0.52311079	64.2968547	0.72310797	0	-3.2289262	0.2786669	-0.6460522	4.16352627	-0.3228148	-6.9077553	4	87	3.57631111	300.222222	27.2222222	0	0	0	0	30.519848	2	0.21141735
0.0423	1364.23093	0.42986886	46.2739766	0.88163853	0	-3.1629682	0.3113236	-0.8419515	3.83460135	-0.1248395	-6.9077553	5	87	4.02335	300.222222	27.2222222	0	0	0	0	30.519848	2	0.21141735
0.0432	1364.23093	0.54477066	69.3606339	0.9611299	0	-3.1419148	0.3113236	-0.6055564	4.23933389	-0.0386058	-6.9077553	6	88	2.68223333	300.222222	27.2222222	0	0	0	0	30.519848	2	0.21141735
0.0756	1627.41357	0.24156035	15.1889665	1.21197446	0	-2.582299	0.48760627	-1.4165047	2.72063511	0.19307558	-6.9077553	7	84	3.57631111	300.777778	27.7777778	0	0	0	0	30.519848	2	0.21141735
0.1251	1770.20858	0.35760058	34.5943004	1.26777607	0	-2.0786419	0.57166213	-1.0255461	3.54371785	0.23805271	-6.9077553	8	78	3.57631111	301.888889	28.88888888	0	0	0	0	30.519848	2	0.21141735
0.2466	2683.17511	0.50552933	73.2789641	1.84937767	0	-1.3999877	0.98737346	-0.680173	4.29428723	0.61538976	-6.9077553	9	76	4.02335	301.888889	28.88888888	0	0	0	0	30.519848	2	0.21141735
0.3258	4828.63357	0.64543408	113.931019	1.70511454	0	-1.1214716	1.5747706	-0.4362841	4.73560195	0.53421859	-6.9077553	10	66	4.02335	304.111111	31.11111111	0	0	0	0	30.519848	2	0.21141735
0.4428	8747.7739	0.6267942	108.770192	1.6930758	0	-0.8146371	2.16891356	-0.4655429	4.68924652	0.52713734	-6.9077553	11	63	3.57631111	304.666667	31.66666666	0	0	0	1	30.519848	2	0.21141735
0.6804	16660.3138	0.78585989	184.707386	2.39325599	0	-0.3850744	2.8130895	-0.2397051	5.21877829	0.87307253	-6.9077553	12	57	5.81150556	305.777778	32.7777778	0	0	0	1	30.519848	2	0.21141735
0.99	26451.1046	0.80162652	190.821789	2.86635341	0	-0.0100503	3.27533572	-0.2198658	5.25134519	1.05338945	-6.9077553	13	56	5.36446667	305.777778	32.7777778	0	0	0	1	30.519848	2	0.21141735
1.3086	34484.4969	0.71776413	138.121756	2.13399355	0	0.26895786	3.54053886	-0.330222	4.92818496	0.75846362	-6.9077553	14	55	5.81150556	306.333333	33.33333333	0	0	0	1	30.519848	2	0.21141735
1.5192	45670.4232	0.72753463	145.981003	1.27114764	0	0.41818388	3.82147279	-0.3167201	4.98348335	0.24070653	-6.9077553	15	59	3.12927222	305.777778	32.7777778	0	0	0	1	30.519848	2	0.21141735
1.6857	55159.8543	0.73815877	152.554371	1.19624832	0	0.52218091	4.01025354	-0.3022425	5.02752762	0.18002585	-6.9077553	16	59	3.12927222	305.222222	32.2222222	0	0	0	1	30.519848	2	0.21141735
1.8135	63715.9269	0.70269689	135.009119	0.96431356	0	0.59525868	4.15445026	-0.3514076	4.90534973	-0.0353023	-6.9077553	17	65	1.78815556	304.666667	31.66666666	0	0	0	1	30.519848	2	0.21141735
1.8873	71188.7047	0.70231721	131.823064	0.94724199	0	0.63514724	4.26534821	-0.3519473	4.88146818	-0.0531455	-6.9077553	18	70	1.34111667	304.111111	31.11111111	0	0	0	1	30.519848	2	0.21141735
1.9476	76455.8463	0.65819515	113.021572	0.95112183	0	0.66659785	4.33672648	-0.4167357	4.72758755	-0.0490623	-6.9077553	19	73	1.78815556	303	30	0	0	0	1	30.519848	2	0.21141735
1.9755	77596.3018	0.47906854	56.4331842	0.77113896	0	0.68082153	4.35153266	-0.7338264	4.03307508	-0.2585907	-6.9077553	20	76	2.68223333	301.888889	28.88888888	0	0	0	1	30.519848	2	0.21141735
1.9881	78166.5343	0.29047196	17.8162548	0.38702379	0	0.68717941	4.3588544	-1.2328115	2.88016736	-0.9466886	-6.9077553	21	80	2.23519444	301.333333	28.33333333	0	0	0	1	30.519848	2	0.21141735
2.0034	78912.2183	0.24432676	12.5083008	0.30329833	0	0.69484574	4.36834875	-1.4051642	2.52647243	-1.1897467	-6.9077553	22	81	2.68223333	301.333333	28.33333333	0	0	0	1	30.519848	2	0.21141735
2.0034	78912.2183	0	0	0	0	0.69484574	4.36834875	-6.9077553	-6.9077553	-6.9077553	-6.9077553	23	83	2.68223333	300.777778	27.7777778	0	0	0	1	30.519848	2	0.21141735
0.0153	704.429735	0.9187234	246.907043	1.83706403	0	-4.1799025	-0.3489481	-0.0836823	5.50901597	0.60871286	-6.9077553	0	88	1.78815556	299.111111	26.11111111	0	0	0	0	34.0724638	2	0.49277013
0.0288	1143.0677	0.52486165	67.5415324	0.88276475	0	-3.5473799	0.13459007	-0.6427171	4.21275751	-0.1235644	-6.9077553	1	89	2.68223333	299.666667	26.66666666	0	0	0	0	34.0724638	2	0.49277013
0.0432	1713.29668	0.56799889	79.9577637	0.85887617	0	-3.1419148	0.5390029	-0.5638768	4.38151105	-0.1509669	-6.9077553	2	86	4.02335	300.222222	27.2222222	0	0	0	0	34.0724638	2	0.49277013
0.0657	2722.16369	0.5880117	88.1306836	1.03723289	0	-2.7226564	1.00179432	-0.5293092	4.4788321	0.03752013	-6.9077553	3	87	3.57631111	300.222222	27.2222222	0	0	0	0	34.0724638	2	0.49277013
0.1035	4345.12377	0.63117409	98.1000395	0.98347382	0	-2.2681837	1.46928436	-0.4585905	4.58599796	-0.015648	-6.9077553	4	87	3.57631111	300.222222	27.2222222	0	0	0	0	34.0724638	2	0.49277013
0.1449	6231.26647	0.59258274	86.4964175	0.84608551	0	-1.9317114	1.82974007	-0.5215787	4.46011456	-0.1659536	-6.9077553	5	87	4.02335	300.222222	27.2222222	0	0	0	0	34.0724638	2	0.49277013
0.1818	7898.08935	0.56967154	78.4871737	0.75473446	0	-1.7048481	2.06674748	-0.5609415	4.36294796	-0.2800652	-6.9077553	6	88	2.68223333	300.222222	27.2222222	0	0	0	0	34.0724638	2	0.49277013
0.2475	10775.3649	0.7984882	176.704971	1.43894781	0	-1.3963447	2.3773553	-0.2237835	5.17448717	0.36460687	-6.9077553	7	84	3.57631111	300.777778	27.7777778	0	0	0	0	34.0724638	2	0.49277013
0.4671	16933.8614	1.00671456	303.720159	2.6620706	0	-0.7612119	2.8293743	0.00768495	5.71611004	0.97947982	-6.9077553	8	78	3.57631111	301.888889	28.88888888	0	0	0	0	34.0724638	2	0.49277013
0.8586	26995.696	1.15744559	400.248707	3.35685811	0	-0.1524521	3.29571449	0.1470791	5.99208862	1.2113033	-6.9077553	9	76	4.02335	301.888889	28.88888888	0	0	0	1	34.0724638	2	0.49277013
1.4706	37703.4	1.32110533	536.256985	4.01175178	0	0.38567048	3.6297768	0.27922541	6.28461536	1.38947724	-6.9077553	10	66	4.02335	304.111111	31.11111111	0	0	0	1	34.0724638	2	0.49277013
2.0529	51980.9034	1.07173121	325.86307	2.73980986	0	0.71925343	3.95089565	0.07020793	5.78648033	1.00825345	-6.9077553	11	63	3.57631111	304.666667	31.66666666	0	0	0	1	34.0724638	2	0.49277013
2.6514	72200.8861	1.05512273	327.436678	2.79612018	1	0.9750878	4.27946617	0.0546044	5.79129774	1.02859038	0.0009995	12	57	5.81150556	305.777778	32.7777778	0	0	0	1	34.0724638	2	0.49277013
3.1878	91022.3489	0.93783375	244.64516	1.90043282	1	1.15933102	4.51111605	-0.0631169	5.49981292	0.64260772	0.0009995	13	56	5.36446667	305.777778	32.7777778	0	0	0	1	34.0724638	2	0.49277013
4.0833	123831.58	1.06442908	324.574372	3.70982893	1	1.40690549	4.81893049	0.06337761	5.78251778	1.31125528	0.0009995	14	55	5.81150556	306.333333	33.33333333	0	0	0	1	34.0724638	2	0.49277013
4.8348	144112.286	0.85363382	208.026198	2.24379254	1	1.57583976	4.9705997	-0.1570822	5.33766883	0.80861311	0.0009995	15	59	3.12927222	305.777778	32.7777778	0	0	0	1	34.0724638	2	0.49277013
5.3901	158062.593	0.77757137	171.344928	1.64204098	1	1.68456394	5.06299744	-0.2502946	5.14368448	0.49654878	0.0009995	16	59	3.12927222	305.222222	32.2222222	0	0	0	1	34.0724638	2	0.49277013
5.7798	170916.936	0.76959341	167.949083	1.4153011	1	1.75436908	5.14118353	-0.2605944	5.12366681	0.34804862	0.0009995	17	65	1.78815556	304.666667	31.66666666	0	0	0	1	34.0724638	2	0.49277013

6.021	180864.018	0.80625352	180.637506	1.3973646	1	1.79525336	5.19775099	-0.2141175	5.19649783	0.33530341	0.0009995	18	70	1.34111667	304.111111	31.11111111	0	0	0	1	34.0724638	2	0.49277013
6.2028	188537.422	0.72274387	137.597415	1.24085711	1	1.8250008	5.23930181	-0.3233177	4.9243394	0.21660793	0.0009995	19	73	1.78815556	303	30	0	0	0	1	34.0724638	2	0.49277013
6.2964	192616.748	0.42532737	45.1175537	0.76941035	1	1.83997804	5.26070765	-0.8525477	3.80929355	-0.260832	0.0009995	20	76	2.68223333	301.888889	28.88888888	0	0	0	1	34.0724638	2	0.49277013
6.3504	194766.087	0.29613307	19.9387126	0.47654696	1	1.8485178	5.27180442	-1.2135752	2.99271335	-0.7390928	0.0009995	21	80	2.23519444	301.333333	28.33333333	0	0	0	1	34.0724638	2	0.49277013
6.4008	196787.413	0.26809502	20.5324445	0.47774209	1	1.85642298	5.28212911	-1.3126907	3.022055	-0.7365933	0.0009995	22	81	2.68223333	301.333333	28.33333333	0	0	0	1	34.0724638	2	0.49277013
6.4008	196787.413	0	0	0	1	1.85642298	5.28212911	-6.9077553	-6.9077553	-6.9077553	0.0009995	23	83	2.68223333	300.777778	27.7777778	0	0	0	1	34.0724638	2	0.49277013
0.0288	1680.15517	1.05498628	303.111265	2.07180737	0	-3.5473799	0.51948116	0.05447519	5.71410325	0.72890391	-6.9077553	0	88	1.78815556	299.111111	26.11111111	0	0	0	0	42.288408	2	-0.4111751
0.0756	3978.51177	0.73052355	138.829759	1.39058289	0	-2.582299	1.38115914	-0.3126259	4.93325563	0.33044187	-6.9077553	1	89	2.68223333	299.666667	26.66666666	0	0	0	0	42.288408	2	-0.4111751
0.1368	6875.453	0.68647458	122.368236	1.29293958	0	-1.9892353	1.92810297	-0.3747304	4.807043	0.2576915	-6.9077553	2	86	4.02335	300.222222	27.2222222	0	0	0	0	42.288408	2	-0.4111751
0.2052	10166.9374	0.70228547	127.010948	1.28772214	0	-1.5837702	2.31923937	-0.3519924	4.84428116	0.25365114	-6.9077553	3	87	3.57631111	300.222222	27.2222222	0	0	0	0	42.288408	2	-0.4111751
0.2709	13281.6352	0.71424385	129.876271	1.29922548	0	-1.3060055	2.58645756	-0.3351317	4.86658993	0.2625377	-6.9077553	4	87	3.57631111	300.222222	27.2222222	0	0	0	0	42.288408	2	-0.4111751
0.3663	17669.4441	0.72383233	135.322045	1.402358	0	-1.0043026	2.87189342	-0.3218149	4.90766484	0.33886794	-6.9077553	5	87	4.02335	300.222222	27.2222222	0	0	0	0	42.288408	2	-0.4111751
0.4518	21575.3527	0.67211055	117.506373	1.16662758	0	-0.7945157	3.07159793	-0.3958457	4.76650108	0.15497398	-6.9077553	6	88	2.68223333	300.222222	27.2222222	0	0	0	0	42.288408	2	-0.4111751
0.5787	27208.9607	0.62750401	116.316102	1.24280846	0	-0.5469711	3.30358311	-0.4644129	4.7563201	0.21817801	-6.9077553	7	84	3.57631111	300.777778	27.7777778	0	0	1	0	42.288408	2	-0.4111751
0.8505	35565.2801	0.73677937	156.443256	1.84754631	0	-0.1619309	3.571398	-0.3041105	5.05269975	0.61439955	-6.9077553	8	78	3.57631111	301.888889	28.88888888	0	0	1	0	42.288408	2	-0.4111751
1.2204	48277.3436	0.82608292	183.691682	1.90587933	0	0.19917867	3.87698309	-0.1898503	5.21326415	0.64546805	-6.9077553	9	76	4.02335	301.888889	28.88888888	0	0	1	0	42.288408	2	-0.4111751
1.5885	60947.0073	0.82491819	178.268146	1.70662057	0	0.46279017	4.11002116	-0.1912596	5.18329447	0.53510092	-6.9077553	10	66	4.02335	304.111111	31.11111111	0	0	1	0	42.288408	2	-0.4111751
2.0007	77387.9866	0.82713238	180.842147	1.51348011	0	0.69349712	4.34884448	-0.1885823	5.19763006	0.41507222	-6.9077553	11	63	3.57631111	304.666667	31.66666666	0	0	1	0	42.288408	2	-0.4111751
2.6289	101819.682	0.95282804	254.186157	2.75366386	0	0.96656551	4.62321324	-0.0472719	5.53807083	1.01329542	-6.9077553	12	57	5.81150556	305.777778	32.7777778	0	0	1	0	42.288408	2	-0.4111751
3.5712	141556.975	1.01775383	299.749702	2.95032776	0	1.27290167	4.95270935	0.01858014	5.70295113	1.08225516	-6.9077553	13	56	5.36446667	305.777778	32.7777778	0	0	1	0	42.288408	2	-0.4111751
4.392	181663.974	1.00353823	294.795641	2.42052179	0	1.4797847	5.20216419	0.00452797	5.68628577	0.88439618	-6.9077553	14	55	5.81150556	306.333333	33.33333333	0	0	1	0	42.288408	2	-0.4111751
5.2335	218257.152	0.96869399	272.463402	2.20380415	0	1.65508027	5.38567854	-0.0307747	5.60750797	0.79063869	-6.9077553	15	59	3.12927222	305.777778	32.7777778	0	0	1	0	42.288408	2	-0.4111751
5.8059	238864.533	0.81421359	192.562746	1.61807193	0	1.75887464	5.47590077	-0.2043051	5.26042725	0.4818531	-6.9077553	16	59	3.12927222	305.222222	32.2222222	0	0	1	0	42.288408	2	-0.4111751
6.1965	250975.639	0.7340033	162.855343	1.33161919	0	1.82398462	5.52535986	-0.3078803	5.09286848	0.28714632	-6.9077553	17	65	1.78815556	304.666667	31.66666666	0	0	1	0	42.288408	2	-0.4111751
6.3675	258384.097	0.78718149	168.825206	1.28282406	0	1.85120693	5.5544511	-0.2380269	5.12886982	0.24984317	-6.9077553	18	70	1.34111667	304.111111	31.11111111	0	0	1	0	42.288408	2	-0.4111751
6.5034	263954.241	0.69698905	130.011615	1.17187379	0	1.87232512	5.57577955	-0.3595519	4.86763149	0.15945697	-6.9077553	19	73	1.78815556	303	30	0	0	1	0	42.288408	2	-0.4111751
6.5808	267292.757	0.39586688	38.3048777	0.61924593	0	1.88415632	5.58834827	-0.9241544	3.64560335	-0.4776392	-6.9077553	20	76	2.68223333	301.888889	28.88888888	0	0	1	0	42.288408	2	-0.4111751
6.6321	269354.349	0.2773949	17.1117618	0.42365998	0	1.8919215	5.59603151	-1.2787147	2.83982449	-0.8564665	-6.9077553	21	80	2.23519444	301.333333	28.33333333	0	0	1	0	42.288408	2	-0.4111751
6.6843	271284.362	0.38067453	56.3861673	0.66879766	0	1.89976149	5.60317126	-0.9631871	4.0322416	-0.4007796	-6.9077553	22	81	2.68223333	301.333333	28.33333333	0	0	1	0	42.288408	2	-0.4111751
6.6843	271284.362	0	0	0	0	1.89976149	5.60317126	-6.9077553	-6.9077553	-6.9077553	-6.9077553	23	83	2.68223333	300.777778	27.7777778	0	0	1	0	42.288408	2	-0.4111751
0.0009	0	0	0	0	0	-7.0131158	-6.9077553	-6.9077553	-6.9077553	-6.9077553	-6.9077553	0	88	1.78815556	299.111111	26.11111111	0	0	0	0	18.9135952	2	-0.0428739
0.0009	0	0	0	0	0	-7.0131158	-6.9077553	-6.9077553	-6.9077553	-6.9077553	-6.9077553	1	89	2.68223333	299.666667	26.66666666	0	0	0	0	18.9135952	2	-0.0428739
0.0009	0	0	0	0	0	-7.0131158	-6.9077553	-6.9077553	-6.9077553	-6.9077553	-6.9077553	2	86	4.02335	300.222222	27.2222222	0	0	0	0	18.9135952	2	-0.0428739
0.0009	0	0	0	0	0	-7.0131158	-6.9077553	-6.9077553	-6.9077553	-6.9077553	-6.9077553	3	87	3.57631111	300.222222	27.2222222	0	0	0	0	18.9135952	2	-0.0428739
0.0009	0	0	0	0	0	-7.0131158	-6.9077553	-6.9077553	-6.9077553	-6.9077553	-6.9077553	4	87	3.57631111	300.222222	27.2222222	0	0	0	0	18.9135952	2	-0.0428739
0.0009	0	0	0	0	0	-7.0131158	-6.9077553	-6.9077553	-6.9077553	-6.9077553	-6.9077553	5	87	4.02335	300.222222	27.2222222	0	0	0	0	18.9135952	2	-0.0428739
0.0009	0	0	0	0	0	-7.0131158	-6.9077553	-6.9077553	-6.9077553	-6.9077553	-6.9077553	6	88	2.68223333	300.222222	27.2222222	0	0	0	0	18.9135952	2	-0.0428739
0.0009	0	0	0	0	0	-7.0131158	-6.9077553	-6.9077553	-6.9077553	-6.9077553	-6.9077553	7	84	3.57631111	300.777778	27.7777778	0	0	0	0	18.9135952	2	-0.0428739
0.0117	0	0.4582967	57.3482463	1.63751539	0	-4.4481664	-6.9077553	-0.7780589	4.0491597	0.49379058	-6.9077553	8	78	3.57631111	301.888889	28.88888888	0	0	0	0	18.9135952	2	-0.0428739
0.0594	145.854835	0.64143904	118.008292	2.39095897	0	-2.8234611	-1.9183107	-0.4424833	4.77076336	0.87211268	-6.9077553	9	76	4.02335	301.888889	28.88888888	0	0	0	0	18.9135952	2	-0.0428739
0.1575	1198.81349	0.62095082	101.384291	1.83106981	0	-1.8483298	0.18216612	-0.4748943	4.61892802	0.60544637	-6.9077553	10	66	4.02335	304.111111	31.11111111	0	0	1	0	18.9135952	2	-0.0428739
0.3177	5501.23924	0.76307678	194.542003	1.98098515	0	-1.1466477	1.70515514	-0.269087	5.27065323	0.68409894	-6.9077553	11	63	3.57631111	304.666667	31.66666666	0	0	1	0	18.9135952	2	-0.0428739
0.5868	14814.4544	0.85682459	251.490306	2.61811323	0	-0.5330712	2.69567086	-0.1533556	5.52740842	0.9628358	-6.9077553	12	57	5.81150556	305.777778	32.7777778	0	0	1	0	18.9135952	2	-0.0428739
0.8406	23578.0217	0.90097327	276.533189	2.38774271	0	-0.1736394	3.1603574	-0.1031704	5.62233446	0.87076717	-6.9077553	13	56	5.36446667	305.777778	32.7777778	0	0	1	0	18.9135952	2	-0.0428739
1.2006	35077.2057	0.91001518	249.873008	2.74759369	0	0.18282143	3.55758002	-0.0931957	5.52095682	1.0110894	-6.9077553	14	55	5.81150556	306.333333	33.33333333	0	0	1	0	18.9135952	2	-0.0428739
1.4985	46824.5207	0.86853156	224.788826	1.81510238	0	0.40446461	3.84642837	-0.1398006	5.41516586	0.59669266	-6.9077553	15	59	3.12927222	305.777778	32.7777778	0	0	1	0	18.9135952	2	-0.0428739
1.7757	56413.1637	0.78689035	183.500913	1.6328556	0	0.57419471	4.03272026	-0.2383963	5.21222509	0.49094262	-6.9077553	16	59	3.12927222	305.222222	32.2222222	0	0	1	0	18.9135952	2	-0.0428739
2.0691	67912.7424	0.82022469	194.775451	1.54222201	0	0.72711373	4.21823841	-0.1969585	5.27185249	0.43387244	-6.9077553	17	65	1.78815556	304.666667	31.66666666	0	0	1	0	18.9135952	2	-0.0428739
2.3049	79687.4793	0.9113324	224.723432	1.68777722	0	0.83503729	4.37812502	-0.0917509	5.4148749	0.52400473	-6.9077553	18	70	1.34111667	304.111111	31.11111111	0	0	1	0	18.9135952	2	-0.0428739
2.4993	88533.6318	0.78276246	161.194806	1.39464046	0	0.91601069	4.4833938	-0.2436493	5.08261981	0.33335342	-6.9077553	19	73	1.78815556	303	30	0	0	1	0	18.9135952	2	-0.0428739
2.583	92393.6388	0.47906502	56.602266	0.85990963	0	0.94895151	4.52606896	-0.7338337	4.03606669	-0.1497657	-6.9077553	20	76	2.68223333	301.888889	28.88888888	0	0	1	0	18.9135952	2	-0.0428739
2.6415	94937.7391	0.3232134	24.0541635	0.5579486	0	0.97134694	4.55323183	-1.1263533	3.18034967	-0.5816978	-6.9077553	21	80	2.23519444	301.333333	28.33333333	0	0	1	0	18.9135952	2	-0.0428739
2.6838	96955.4762	0.26161531	15.1111515	0.42856188	0	0.9872337	4.57426218	-1.337065	2.71549916	-0.8449895	-6.9077553	22	81	2.68223333	301.333333	28.33333333	0	0	1	0	18.9135952	2	-0.0428739
2.6838	96955.4762	0	0	0	0	0.9872337	4.57426218	-6.9077553	-6.9077553	-6.9077553	-6.9077553	23	83	2.68223333	300.777778	27.7777778	0	0	1	0	18.9135952	2	-0.0428739
0.009	362.578026	0.85135333	194.98032	1.60810079	0	-4.7105307	-1.0117614	-0.1597541	5.27290376	0.4756755	-6.9077553	0	83	4.02335	299.666667	26.66666666	0	0	0	0	30.519848	2	0.21141735
0.0135	581.896976	0.46691203	55.0985779	0.82138748	0	-4.3050656	-0.5397448	-0.759475	4.00914206	-0.1955436	-6.9077553	1	87	4.02335	299.111111	26.11111111	0	0	0	0	30.519848	2	0.21141735
0.0261	1064.3986	0.63156973	97.0239868	1.02600724	0	-3.64582	0.063349	-0.4579648	4.57496854	0.02664898	-6.9077553	2	89	3.57631111	298.555556	25.55555555	0	0	0	0	30.519848	2	0.21141735
0.0351	1239.85363	0.55516253	76.5634216	0.9875639	0	-3.3495541	0.21579595	-0.5866947	4.3381325	-0.011502	-6.9077553	3	92	4.02335	298.555556	25.55555555	0	0	0	0	30.519848	2	0.21141735
0.0405	1371.44502	0.43091937	46.4571431	0.85861246	0	-3.2064533	0.31659384	-0.8395164	3.83855176	-0.1512736	-6.9077553	4	88	4.91742778	298.555556	25.55555555	0	0	0	0	30.519848	2	0.21141735
0.0423	1415.30882	0.52034044	62.7817879	0.62918174	0	-3.1629682	0.34805406	-0.651352	4.13968096	-0.461747	-6.9077553	5	87	4.02335	298.555556	25.55555555	0	0	0	0	30.519848	2	0.21141735
0.0981	1634.62769	0.31991463	25.8468057	1.66983389	0	-2.3217679	0.49202664	-1.1365801	3.25222571	0.51332284	-6.9077553	6	82	4.91742778	299.666667	26.66666666	0	0	0	0	30.519848	2	0.21141735
0.1962	1951.01722	0.48271165	67.8100541	2.28970393	0	-1.6286207	0.66886331	-0.7262663	4.21672522	0.82885916	-6.9077553	7	74	5.36446667	301.333333	28.33333333	0	0	0	0	30.519848	2	0.21141735

0.2844	2996.66815	0.6253752	115.3124	2.2725936	0	-1.2573736	1.0978347	-0.4678057	4.74765364	0.82136166	-6.9077553	8	68	5.36446667	302.444444	29.44444444	0	0	0	0	30.519848	2	0.21141735
0.3726	3694.64074	0.57856571	90.5737504	1.49712621	0	-0.9872498	1.30715395	-0.5454762	4.50617548	0.40421513	-6.9077553	9	63	5.81150556	303.555556	30.55555555	0	0	0	0	30.519848	2	0.21141735
0.5481	7300.63704	0.65421131	121.705158	2.03037297	0	-0.6012975	1.98809858	-0.4227975	4.8016096	0.70871191	-6.9077553	10	60	4.91742778	304.666667	31.66666666	0	0	0	1	30.519848	2	0.21141735
0.8307	16849.5986	0.76799196	177.327292	2.43647407	0	-0.1854866	2.82438618	-0.2626748	5.17800277	0.89096228	-6.9077553	11	55	4.91742778	306.888889	33.88888888	0	0	0	1	30.519848	2	0.21141735
1.1655	27364.5978	0.80623676	188.118994	2.93993616	0	0.15315018	3.30928667	-0.2141383	5.23708002	1.07872795	-6.9077553	12	51	5.81150556	307.444444	34.4444444	0	0	0	1	30.519848	2	0.21141735
1.485	39114.8068	0.7113491	135.82522	1.75355964	0	0.39541477	3.66652665	-0.3391872	4.91137628	0.56221791	-6.9077553	13	49	5.36446667	308.555556	35.5555556	0	0	0	1	30.519848	2	0.21141735
1.7784	51549.444	0.72790892	153.022515	1.55567829	0	0.57571408	3.94256082	-0.3162065	5.0305916	0.44255425	-6.9077553	14	52	4.47038889	306.888889	33.88888888	0	0	0	1	30.519848	2	0.21141735
2.1168	71494.2182	0.74077931	158.717046	1.68593711	0	0.74990551	4.26963057	-0.2987035	5.06712933	0.52291452	-6.9077553	15	52	4.47038889	307.444444	34.4444444	0	0	0	1	30.519848	2	0.21141735
2.3886	87103.3399	0.79371406	204.981995	1.82154331	0	0.87070742	4.46710671	-0.2297729	5.32292702	0.60023295	-6.9077553	16	54	3.57631111	306.888889	33.88888888	0	0	0	1	30.519848	2	0.21141735
2.5641	106879.106	0.62047397	105.498748	0.85801266	0	0.94160754	4.67170771	-0.4756613	4.65870856	-0.1519716	-6.9077553	17	58	2.68223333	305.777778	32.7777778	0	0	0	1	30.519848	2	0.21141735
2.6748	124661.49	0.74237352	147.58138	0.91262954	0	0.98387461	4.82561001	-0.2965566	4.99438653	-0.0903301	-6.9077553	18	65	1.78815556	304.111111	31.11111111	0	0	0	1	30.519848	2	0.21141735
2.772	142976.022	0.69439839	127.169244	0.85344103	0	1.01956908	4.96268393	-0.3632704	4.84552669	-0.1573078	-6.9077553	19	70	1.78815556	303	30	0	0	0	1	30.519848	2	0.21141735
2.8485	153920.759	0.67638572	120.734639	0.95931558	0	1.04679254	5.03644442	-0.3895144	4.79360336	-0.0404933	-6.9077553	20	77	0.44703889	301.888889	28.88888888	0	0	0	1	30.519848	2	0.21141735
2.8737	154973.493	0.50232349	61.0780727	0.92211533	0	1.0556004	5.04326054	-0.6865222	4.1121693	-0.0800011	-6.9077553	21	75	0.89407778	297.444444	24.44444444	0	0	0	1	30.519848	2	0.21141735
2.8953	155675.305	0.4306314	45.4515076	0.76340755	0	1.06308873	5.04777888	-0.8401833	3.81666799	-0.2686542	-6.9077553	22	88	0.89407778	295.222222	22.2222222	0	0	0	1	30.519848	2	0.21141735
2.9025	155982.355	0.2698037	15.6660662	0.46122104	0	1.06557243	5.0497493	-1.3063611	2.75156081	-0.7717121	-6.9077553	23	46	2.23519444	296.888889	23.88888888	0	0	0	1	30.519848	2	0.21141735
0.0171	749.783921	1.00080681	278.980035	2.1178201	0	-4.0686768	-0.2866374	0.00180518	5.6311438	0.75085938	-6.9077553	0	83	4.02335	299.666667	26.66666666	0	0	0	0	34.0724638	2	0.49277013
0.0324	1320.01314	0.5392267	71.3896772	0.88034826	0	-3.4295969	0.27839897	-0.6157664	4.26816729	-0.1263024	-6.9077553	1	87	4.02335	299.111111	26.11111111	0	0	0	0	34.0724638	2	0.49277013
0.0495	1890.24238	0.51186627	64.4282933	0.76599894	0	-3.0057826	0.63723396	-0.6677402	4.1655684	-0.2652699	-6.9077553	2	89	3.57631111	298.555556	25.55555555	0	0	0	0	34.0724638	2	0.49277013
0.0729	2899.10919	0.61537611	97.1813401	1.0941782	0	-2.6186666	1.06474839	-0.4838979	4.57658901	0.09091709	-6.9077553	3	92	4.02335	298.555556	25.55555555	0	0	0	0	34.0724638	2	0.49277013
0.1089	4434.34185	0.58011398	83.6555786	0.9010252	0	-2.2173252	1.48960469	-0.5428084	4.42672007	-0.1031128	-6.9077553	4	88	4.91742778	298.555556	25.55555555	0	0	0	0	34.0724638	2	0.49277013
0.1494	6320.48416	0.59843843	88.2296549	0.89540244	0	-1.901128	1.84395402	-0.511762	4.47995446	-0.1093658	-6.9077553	5	87	4.02335	298.555556	25.55555555	0	0	0	0	34.0724638	2	0.49277013
0.1854	8031.17213	0.61948328	93.5739746	0.81321125	0	-1.6852396	2.08345499	-0.4772566	4.53876298	-0.2055354	-6.9077553	6	82	4.91742778	299.666667	26.66666666	0	0	0	0	34.0724638	2	0.49277013
0.2826	12232.5305	0.91247813	239.74188	2.16279927	0	-1.2637228	2.50418059	-0.0904958	5.47956702	0.7718656	-6.9077553	7	74	5.36446667	301.333333	28.33333333	0	0	0	1	34.0724638	2	0.49277013
0.4905	22555.1093	1.12795505	371.02523	3.34336212	0	-0.71233	3.11600595	0.12129247	5.91627276	1.20727598	-6.9077553	8	68	5.36446667	302.444444	29.44444444	0	0	0	1	34.0724638	2	0.49277013
1.0161	43804.4944	1.35683002	539.973245	4.75286949	1	0.01597177	3.77975925	0.30588785	6.29152144	1.55895892	0.0009995	9	63	5.81150556	303.555556	30.55555555	0	0	0	1	34.0724638	2	0.49277013
1.6371	59109.3854	1.24283005	464.781341	3.67787591	1	0.49292638	4.07940664	0.21819537	6.14156921	1.30260725	0.0009995	10	60	4.91742778	304.666667	31.66666666	0	0	0	1	34.0724638	2	0.49277013
2.2185	74083.6262	1.10811339	359.661281	3.06863942	1	0.79683129	4.30520804	0.10356095	5.88516548	1.1215601	0.0009995	11	55	4.91742778	306.888889	33.88888888	0	0	0	1	34.0724638	2	0.49277013
2.8386	94838.5542	1.1260888	377.726597	2.99215277	1	1.04331097	4.55218656	0.11963803	5.93417329	1.09632727	0.0009995	12	51	5.81150556	307.444444	34.4444444	0	0	0	1	34.0724638	2	0.49277013
3.4722	118063.916	1.02166973	291.595836	2.74247222	1	1.2447884	4.77123461	0.02241659	5.67537215	1.00922435	0.0009995	13	49	5.36446667	308.555556	35.5555556	0	0	0	1	34.0724638	2	0.49277013
4.0653	136873.787	0.90205441	230.636096	2.36995344	1	1.40248754	4.91906654	-0.1019725	5.44084546	0.86329217	0.0009995	14	52	4.47038889	306.888889	33.88888888	0	0	0	1	34.0724638	2	0.49277013
4.6332	153054.71	0.87640942	222.596771	2.35468255	1	1.53324777	5.03080197	-0.1307816	5.40536643	0.85683051	0.0009995	15	52	4.47038889	307.444444	34.4444444	0	0	0	1	34.0724638	2	0.49277013
5.0634	163820.586	0.77164016	168.086313	1.72282301	1	1.62203819	5.09877794	-0.2579419	5.12448357	0.54454451	0.0009995	16	54	3.57631111	306.888889	33.88888888	0	0	0	1	34.0724638	2	0.49277013
5.4315	173513.426	0.73201418	155.126328	1.33491408	1	1.69221534	5.15626074	-0.3105902	5.04424625	0.28961576	0.0009995	17	58	2.68223333	305.777778	32.7777778	0	0	0	1	34.0724638	2	0.49277013
5.6322	181647.596	0.80458965	176.486932	1.29057976	1	1.72850013	5.20207403	-0.2161808	5.1732525	0.25586609	0.0009995	18	65	1.78815556	304.111111	31.11111111	0	0	0	1	34.0724638	2	0.49277013
5.7996	188737.706	0.77541187	159.265835	1.28077427	1	1.75778895	5.24036355	-0.2530721	5.070581	0.24824527	0.0009995	19	70	1.78815556	303	30	0	0	0	1	34.0724638	2	0.49277013
5.9535	195602.663	0.69832419	126.556012	1.15005725	1	1.78397928	5.27609049	-0.3576408	4.8406929	0.14068087	0.0009995	20	77	0.44703889	301.888889	28.88888888	0	0	0	1	34.0724638	2	0.49277013
6.0462	199725.853	0.45639531	52.3892407	0.75318849	1	1.79942998	5.2969507	-0.7822073	3.95872033	-0.2821129	0.0009995	21	75	0.89407778	297.444444	24.44444444	0	0	0	1	34.0724638	2	0.49277013
6.1227	202884.04	0.36898754	33.0543716	0.52089063	1	1.81200318	5.31263951	-0.9942859	3.49818408	-0.6502972	0.0009995	22	88	0.89407778	295.222222	22.2222222	0	0	0	1	34.0724638	2	0.49277013
6.2127	206684.92	0.56486237	96.3550195	0.82110077	1	1.82659558	5.33120035	-0.5694044	4.56804987	-0.1958923	0.0009995	23	46	2.23519444	296.888889	23.88888888	0	0	0	1	34.0724638	2	0.49277013
0.0351	2252.23574	1.13323001	353.945287	2.45225445	0	-3.3495541	0.81236729	0.12595401	5.86914517	0.89741549	-6.9077553	0	83	4.02335	299.666667	26.66666666	0	0	0	0	42.288408	2	-0.4111751
0.0846	4671.93486	0.73031762	138.992321	1.41493364	0	-2.469821	1.54178732	-0.3129074	4.93442588	0.34778913	-6.9077553	1	87	4.02335	299.111111	26.11111111	0	0	0	0	42.288408	2	-0.4111751
0.1431	7435.37764	0.6660219	113.901795	1.25618368	0	-1.9442116	2.00638385	-0.4049324	4.73534541	0.22887405	-6.9077553	2	89	3.57631111	298.555556	25.55555555	0	0	0	0	42.288408	2	-0.4111751
0.2016	10242.8049	0.66081754	109.717082	1.1864617	0	-1.6014697	2.32667312	-0.4127654	4.69791419	0.171818	-6.9077553	3	92	4.02335	298.555556	25.55555555	0	0	0	0	42.288408	2	-0.4111751
0.2709	13534.7385	0.68190141	118.633675	1.23687407	0	-1.3060055	2.60533349	-0.3814048	4.77604881	0.21339545	-6.9077553	4	88	4.91742778	298.555556	25.55555555	0	0	0	0	42.288408	2	-0.4111751
0.3645	17798.6152	0.72778606	140.324106	1.37242376	0	-1.0092287	2.87917684	-0.3163751	4.94396192	0.31730672	-6.9077553	5	87	4.02335	298.555556	25.55555555	0	0	0	0	42.288408	2	-0.4111751
0.5175	23390.7081	0.65428408	123.854676	1.54981689	0	-0.6587458	3.1523816	-0.4226864	4.81911698	0.43878182	-6.9077553	6	82	4.91742778	299.666667	26.66666666	0	0	0	0	42.288408	2	-0.4111751
0.8271	33930.6946	0.85591817	207.397325	2.48703358	0	-0.1898297	3.52434952	-0.1544128	5.33464122	0.91149267	-6.9077553	7	74	5.36446667	301.333333	28.33333333	0	0	1	0	42.288408	2	-0.4111751
1.1961	47757.1304	0.86516538	200.18388	1.94874238	0	0.17906626	3.86614932	-0.1436794	5.29924134	0.66769725	-6.9077553	8	68	5.36446667	302.444444	29.44444444	0	0	1	0	42.288408	2	-0.4111751
1.6722	65894.4189	0.90197789	222.752644	2.3308927	0	0.51414012	4.18806892	-0.1020572	5.40606642	0.84668025	-6.9077553	9	63	5.81150556	303.555556	30.55555555	0	0	1	0	42.288408	2	-0.4111751
2.2896	88340.662	0.87750484	214.625752	2.15004363	0	0.82837713	4.48121182	-0.1295339	5.36890048	0.76595313	-6.9077553	10	60	4.91742778	304.666667	31.66666666	0	0	1	0	42.288408	2	-0.4111751
3.0555	120118.455	0.97952626	271.709019	2.57684434	0	1.11694325	4.78848671	-0.0196659	5.60473539	0.94695352	-6.9077553	11	55	4.91742778	306.888889	33.88888888	0	0	1	0	42.288408	2	-0.4111751
3.8979	162679.881	1.05366203	322.730469	2.60474963	0	1.36043795	5.0917905	0.05322037	5.77682061	0.9577204	-6.9077553	12	51	5.81150556	307.444444	34.4444444	0	0	1	0	42.288408	2	-0.4111751
4.7898	206300.513	1.06898192	340.51419	2.86305041	0	1.56648866	5.32933876	0.06764175	5.83045974	1.05223685	-6.9077553	13	49	5.36446667	308.555556	35.5555556	0	0	1	0	42.288408	2	-0.4111751
5.3739	227887.951	0.85576273	206.77995	1.88119568	0	1.6815539	5.42885845	-0.1545943	5.33166002	0.63243901	-6.9077553	14	52	4.47038889	306.888889	33.88888888	0	0	1	0	42.288408	2	-0.4111751
5.9742	248791.91	0.83748397	201.514524	1.89903705	0	1.7874502	5.51662086	-0.1761598	5.30586642	0.64187339	-6.9077553	15	52	4.47038889	307.444444	34.4444444	0	0	1	0	42.288408	2	-0.4111751
6.4269	262278.735	0.73403973	157.804035	1.43940823	0	1.86049231	5.56941162	-0.3078307	5.06136032	0.36492657	-6.9077553	16	54	3.57631111	306.888889	33.88888888	0	0	1	0	42.288408	2	-0.4111751
6.7419	271533.513	0.67257494	140.722969	1.14984122	0	1.90834178	5.60408925	-0.395156	4.9468003	0.14049317	-6.9077553	17	58	2.68223333	305.777778	32.7777778	0	0	1	0	42.288408	2	-0.4111751
6.8814	277329.345	0.78120019	164.048425	1.15164421	0	1.92882212	5.62520938	-0.2456446	5.10016775	0.14205861	-6.9077553	18	65	1.78815556	304.111111	31.11111111	0	0	1	0	42.288408	2	-0.4111751
7.0092	282780.959	0.72709086	137.68871	1.06476131	0	1.94722357	5.64467614	-0.3173294	4.92500268	0.06368939	-6.9077553	19	70	1.78815556	303	30	0	0	1	0	42.288408	2	-0.4111751
7.1199	287091.793	0.65761914	114.691716	1.09297862	0	1.96289368	5.65980549	-0.4176099	4.74225652	0.08982117	-6.9077553	20	77	0.44703889	301.888889	28.88888888	0	0	1	0	42.288408	2	-0.4111751
7.1622	288714.75	0.39921692	39.3532351	0.57773018	0	1.9688172	5.66544264	-0.9157486	3.6726036	-0.5469189	-6.9077553	21	75	0.89407778	297.444444	24.44444444	0	0	1	0	42.288408	2	-0.4111751

7.2	290074.52	0.3554873	29.085115	0.5512461	0	1.97408103	5.6701413	-1.0314567	3.37026091	-0.5937615	-6.9077553	22	88	0.89407778	295.222222	22.2222222	0	0	1	0	42.288408	2	-0.4111751
7.2279	291218.154	0.34661859	40.4886632	0.4775246	0	1.97794854	5.67407609	-1.0566494	3.70104671	-0.7370477	-6.9077553	23	46	2.23519444	296.888889	23.88888888	0	0	1	0	42.288408	2	-0.4111751
0.0009	0	0	0	0	0	-7.0131158	-6.9077553	-6.9077553	-6.9077553	-6.9077553	-6.9077553	0	83	4.02335	299.666667	26.66666666	0	0	0	0	18.9135952	2	-0.0428739
0.0009	0	0	0	0	0	-7.0131158	-6.9077553	-6.9077553	-6.9077553	-6.9077553	-6.9077553	1	87	4.02335	299.111111	26.11111111	0	0	0	0	18.9135952	2	-0.0428739
0.0009	0	0	0	0	0	-7.0131158	-6.9077553	-6.9077553	-6.9077553	-6.9077553	-6.9077553	2	89	3.57631111	298.555556	25.55555555	0	0	0	0	18.9135952	2	-0.0428739
0.0009	0	0	0	0	0	-7.0131158	-6.9077553	-6.9077553	-6.9077553	-6.9077553	-6.9077553	3	92	4.02335	298.555556	25.55555555	0	0	0	0	18.9135952	2	-0.0428739
0.0009	0	0	0	0	0	-7.0131158	-6.9077553	-6.9077553	-6.9077553	-6.9077553	-6.9077553	4	88	4.91742778	298.555556	25.55555555	0	0	0	0	18.9135952	2	-0.0428739
0.0009	0	0	0	0	0	-7.0131158	-6.9077553	-6.9077553	-6.9077553	-6.9077553	-6.9077553	5	87	4.02335	298.555556	25.55555555	0	0	0	0	18.9135952	2	-0.0428739
0.0009	0	0	0	0	0	-7.0131158	-6.9077553	-6.9077553	-6.9077553	-6.9077553	-6.9077553	6	82	4.91742778	299.666667	26.66666666	0	0	0	0	18.9135952	2	-0.0428739
0.0468	64.4049603	0.67915554	128.239315	3.08551444	0	-3.0618721	-2.7271572	-0.3854338	4.85390597	1.12704244	-6.9077553	7	74	5.36446667	301.333333	28.33333333	0	0	0	0	18.9135952	2	-0.0428739
0.2025	3628.5529	0.81560644	217.493836	2.62847124	0	-1.5970154	1.28910947	-0.202598	5.38217511	0.96678278	-6.9077553	8	68	5.36446667	302.444444	29.44444444	0	0	0	0	18.9135952	2	-0.0428739
0.4689	11612.9036	0.85254324	238.565535	2.8505449	0	-0.7573658	2.45220297	-0.1583591	5.47464825	1.04786092	-6.9077553	9	63	5.81150556	303.555556	30.55555555	0	0	1	0	18.9135952	2	-0.0428739
0.6417	15846.5219	0.77375523	206.654114	1.88104216	0	-0.4436344	2.76301314	-0.2552081	5.33105129	0.63235744	-6.9077553	10	60	4.91742778	304.666667	31.66666666	0	0	1	0	18.9135952	2	-0.0428739
0.8559	22069.3051	0.81374712	207.31895	2.23661766	0	-0.1556017	3.09423304	-0.2048775	5.33426325	0.80541175	-6.9077553	11	55	4.91742778	306.888889	33.88888888	0	0	1	0	18.9135952	2	-0.0428739
1.1808	32891.3078	0.88543346	244.040123	2.66749602	0	0.16619217	3.49323883	-0.1205492	5.49733675	0.98151503	-6.9077553	12	51	5.81150556	307.444444	34.4444444	0	0	1	0	18.9135952	2	-0.0428739
1.5534	47729.5283	0.92910737	256.808575	2.24477132	0	0.44044608	3.8655712	-0.0724552	5.54833486	0.80904904	-6.9077553	13	49	5.36446667	308.555556	35.5555556	0	0	1	0	18.9135952	2	-0.0428739
1.8468	58136.7947	0.81918447	196.15607	1.65470577	0	0.61345441	4.06281596	-0.198226	5.27891572	0.50422737	-6.9077553	14	52	4.47038889	306.888889	33.88888888	0	0	1	0	18.9135952	2	-0.0428739
2.2185	72050.0135	0.85465076	219.725711	1.9508336	0	0.79683129	4.27737439	-0.155893	5.39238455	0.66876924	-6.9077553	15	52	4.47038889	307.444444	34.4444444	0	0	1	0	18.9135952	2	-0.0428739
2.5992	87736.776	0.85369281	214.042479	1.66750009	0	0.95520371	4.47435255	-0.1570132	5.36617917	0.51192507	-6.9077553	16	54	3.57631111	306.888889	33.88888888	0	0	1	0	18.9135952	2	-0.0428739
2.8701	96721.5962	0.71191072	153.497807	1.39756861	0	1.05434687	4.57184705	-0.3383991	5.03369279	0.33544929	-6.9077553	17	58	2.68223333	305.777778	32.7777778	0	0	1	0	18.9135952	2	-0.0428739
3.0402	104865.327	0.89585109	224.412264	1.58342198	0	1.1119233	4.65268646	-0.1088654	5.41348928	0.46021966	-6.9077553	18	65	1.78815556	304.111111	31.11111111	0	0	1	0	18.9135952	2	-0.0428739
3.1896	111864.869	0.77881714	161.778826	1.29460613	0	1.15989552	4.71730056	-0.2486958	5.08623631	0.25897864	-6.9077553	19	70	1.78815556	303	30	0	0	1	0	18.9135952	2	-0.0428739
3.3309	118341.51	0.75112575	147.417546	1.32238847	0	1.20324254	4.77358305	-0.2848517	4.99327579	0.28019547	-6.9077553	20	77	0.44703889	301.888889	28.88888888	0	0	1	0	18.9135952	2	-0.0428739
3.4218	122245.38	0.49180856	61.5770469	0.89936881	0	1.23016673	4.80603852	-0.7076345	4.12030543	-0.1049508	-6.9077553	21	75	0.89407778	297.444444	24.44444444	0	0	1	0	18.9135952	2	-0.0428739
3.5055	125929.935	0.42228526	45.3768166	0.76697368	0	1.25433316	4.83573362	-0.8597089	3.81502336	-0.2639998	-6.9077553	22	88	0.89407778	295.222222	22.2222222	0	0	1	0	18.9135952	2	-0.0428739
3.5505	127859.939	0.26769003	17.3302466	0.58629993	0	1.26708844	4.85094326	-1.3141969	2.85251103	-0.5322196	-6.9077553	23	46	2.23519444	296.888889	23.88888888	0	0	1	0	18.9135952	2	-0.0428739
0.0153	409.435188	0.87980282	211.778839	1.65643764	0	-4.1799025	-0.8905372	-0.1269215	5.35554724	0.50527282	-6.9077553	0	62	1.34111667	304.111111	31.11111111	0	0	0	0	30.519848	2	0.21141735
0.0306	1117.7858	0.77287248	157.361342	1.24644111	0	-3.4867553	0.11224399	-0.2563482	5.05855106	0.22109434	-6.9077553	1	71	1.78815556	301.333333	28.33333333	0	0	0	0	30.519848	2	0.21141735
0.0414	1380.96864	0.7645572	150.665426	1.500549	0	-3.1844744	0.32350904	-0.2671513	5.01506829	0.40649724	-6.9077553	2	75	1.78815556	300.222222	27.2222222	0	0	0	0	30.519848	2	0.21141735
0.0459	1468.69625	0.33137012	28.2738586	0.80602999	0	-3.0812902	0.38505575	-1.1015061	3.34197302	-0.2143945	-6.9077553	3	82	1.34111667	299.111111	26.11111111	0	0	0	0	30.519848	2	0.21141735
0.0459	1468.69625	0	0	0	0	-3.0812902	0.38505575	-6.9077553	-6.9077553	-6.9077553	-6.9077553	4	89	1.34111667	298	25	0	0	0	0	30.519848	2	0.21141735
0.0459	1468.69625	0	0	0	0	-3.0812902	0.38505575	-6.9077553	-6.9077553	-6.9077553	-6.9077553	5	90	1.34111667	298	25	0	0	0	0	30.519848	2	0.21141735
0.0459	1468.69625	0	0	0	0	-3.0812902	0.38505575	-6.9077553	-6.9077553	-6.9077553	-6.9077553	6	91	1.34111667	297.444444	24.44444444	0	0	0	0	30.519848	2	0.21141735
0.0459	1468.69625	0	0	0	0	-3.0812902	0.38505575	-6.9077553	-6.9077553	-6.9077553	-6.9077553	7	93	1.34111667	297.444444	24.44444444	0	0	0	0	30.519848	2	0.21141735
0.0459	1468.69625	0	0	0	0	-3.0812902	0.38505575	-6.9077553	-6.9077553	-6.9077553	-6.9077553	8	93	1.78815556	297.444444	24.44444444	0	0	0	0	30.519848	2	0.21141735
0.0459	1468.69625	0	0	0	0	-3.0812902	0.38505575	-6.9077553	-6.9077553	-6.9077553	-6.9077553	9	93	2.23519444	297.444444	24.44444444	0	0	0	0	30.519848	2	0.21141735
0.0459	1468.69625	0	0	0	0	-3.0812902	0.38505575	-6.9077553	-6.9077553	-6.9077553	-6.9077553	10	94	1.34111667	297.444444	24.44444444	0	0	0	0	30.519848	2	0.21141735
0.0459	1468.69625	0	0	0	0	-3.0812902	0.38505575	-6.9077553	-6.9077553	-6.9077553	-6.9077553	11	92	1.34111667	298	25	0	0	0	0	30.519848	2	0.21141735
0.0459	1468.69625	0	0	0	0	-3.0812902	0.38505575	-6.9077553	-6.9077553	-6.9077553	-6.9077553	12	88	2.68223333	299.111111	26.11111111	0	0	0	0	30.519848	2	0.21141735
0.0819	1658.09676	0.36374421	41.1674896	1.27863293	0	-2.5022563	0.50627333	-1.008559	3.71767315	0.24657326	-6.9077553	13	83	3.57631111	300.222222	27.2222222	0	0	0	0	30.519848	2	0.21141735
0.1836	2220.71276	0.53541102	85.8279608	1.96319877	0	-1.6949958	0.79827841	-0.6228546	4.45235649	0.67508441	-6.9077553	14	73	3.57631111	302.444444	29.44444444	0	0	0	0	30.519848	2	0.21141735
0.3303	3278.7868	0.51108879	73.0826663	1.50463624	0	-1.1077539	1.18777842	-0.6692573	4.2916049	0.40921556	-6.9077553	15	63	3.12927222	304.111111	31.11111111	0	0	0	0	30.519848	2	0.21141735
0.4824	4341.41336	0.51078074	72.0346824	1.4869847	0	-0.7289816	1.46843027	-0.669859	4.27716159	0.39742266	-6.9077553	16	55	2.68223333	306.333333	33.33333333	0	0	0	0	30.519848	2	0.21141735
0.6597	5912.79246	0.58254362	90.8694083	1.62121148	0	-0.4159701	1.77728733	-0.5386361	4.50943441	0.48379033	-6.9077553	17	50	3.12927222	307.444444	34.4444444	0	0	0	1	30.519848	2	0.21141735
0.9207	8631.40134	0.71425581	138.66518	1.99986404	0	-0.082621	2.15552272	-0.335115	4.93206947	0.69357911	-6.9077553	18	47	3.57631111	307.444444	34.4444444	0	0	0	1	30.519848	2	0.21141735
1.2078	13506.7767	0.82238865	192.977885	1.86979498	0	0.18880052	2.60326557	-0.194327	5.26258078	0.62636347	-6.9077553	19	44	2.23519444	308.555556	35.5555556	0	0	0	1	30.519848	2	0.21141735
1.5921	19857.0801	0.82731139	195.371011	2.04599635	0	0.4650539	2.98861098	-0.1883661	5.27490549	0.71637352	-6.9077553	20	43	2.23519444	309.111111	36.11111111	0	0	0	1	30.519848	2	0.21141735
1.9296	25935.2176	0.84745068	201.935417	2.82067611	0	0.65731273	3.25564035	-0.1643433	5.30795288	1.03733107	-6.9077553	21	88	5.81150556	296.333333	23.33333333	0	0	0	1	30.519848	2	0.21141735
1.9665	27438.6597	0.38008855	40.337581	0.56348773	0	0.67625531	3.3119894	-0.9647235	3.69730836	-0.5718366	-6.9077553	22	90	2.23519444	296.888889	23.88888888	0	0	0	1	30.519848	2	0.21141735
1.989	28359.8002	0.21262739	10.6303931	0.37819061	0	0.687632	3.34500792	-1.5435219	2.36381123	-0.9697163	-6.9077553	23	91	1.34111667	296.888889	23.88888888	0	0	0	1	30.519848	2	0.21141735
0.0261	932.956939	0.9804069	268.933472	2.07279151	0	-3.64582	-0.0683249	-0.0187681	5.59446775	0.72937858	-6.9077553	0	62	1.34111667	304.111111	31.11111111	0	0	0	0	34.0724638	2	0.49277013
0.0639	2315.00384	0.79689843	171.371791	1.35942059	0	-2.7504359	0.83984322	-0.225774	5.14384125	0.30779391	-6.9077553	1	71	1.78815556	301.333333	28.33333333	0	0	0	0	34.0724638	2	0.49277013
0.1035	4121.05606	0.71564726	130.403897	1.07367464	0	-2.2681837	1.41635208	-0.3331715	4.87064421	0.07201795	-6.9077553	2	75	1.78815556	300.222222	27.2222222	0	0	0	0	34.0724638	2	0.49277013
0.1341	5612.42435	0.48666298	55.9391085	0.63806455	0	-2.0091695	1.72516093	-0.7181307	4.02428163	-0.4477498	-6.9077553	3	82	1.34111667	299.111111	26.11111111	0	0	0	0	34.0724638	2	0.49277013
0.1548	6533.5639	0.32714373	23.4942255	0.3815918	0	-1.8656213	1.87710561	-1.1143036	3.15679723	-0.9607867	-6.9077553	4	89	1.34111667	298	25	0	0	0	0	34.0724638	2	0.49277013
0.1683	7191.52075	0.24310424	12.095696	0.28625523	0	-1.7820072	1.9730417	-1.41016	2.49293236	-1.2473841	-6.9077553	5	90	1.34111667	298	25	0	0	0	0	34.0724638	2	0.49277013
0.1818	7849.47759	0.22277358	10.3340597	0.28640985	0	-1.7048481	2.06057437	-1.4971205	2.33554196	-1.246846	-6.9077553	6	91	1.34111667	297.444444	24.44444444	0	0	0	0	34.0724638	2	0.49277013
0.1971	8375.84326	0.2243098	10.2429253	0.2618167	0	-1.6240441	2.12547114	-1.490279	2.32668488	-1.3362985	-6.9077553	7	93	1.34111667	297.444444	24.44444444	0	0	0	0	34.0724638	2	0.49277013
0.2088	8858.34492	0.21321583	9.07417532	0.24921597	0	-1.5663784	2.18147283	-1.5407712	2.2055427	-1.3854309	-6.9077553	8	93	1.78815556	297.444444	24.44444444	0	0	0	0	34.0724638	2	0.49277013
0.2232	9428.57391	0.19897884	8.18768597	0.23063931	0	-1.499687	2.24385091	-1.6095437	2.10275344	-1.4625738	-6.9077553	9	93	2.23519444	297.444444	24.44444444	0	0	0	0	34.0724638	2	0.49277013
0.2331	9779.48413	0.22644767	10.6056255	0.28685399	0	-1.4562877	2.28038898	-1.4808351	2.36147885	-1.2453019	-6.9077553	10	94	1.34111667	297.444444	24.44444444	0	0	0	0	34.0724638	2	0.49277013
0.2457	10218.1219	0.23039327	11.0105046	0.3031357	0	-1.403644	2.32426067	-1.4636365	2.3989406	-1.1902813	-6.9077553	11	92	1.34111667	298	25	0	0	0	0	34.0724638	2	0.49277013

0.2637	11051.5333	0.37696567	40.7443146	0.60089283	0	-1.3329432	2.40265966	-0.9729519	3.70734085	-0.5076759	-6.9077553	12	88	2.68223333	299.111111	26.11111111	0	0	0	0	34.0724638	2	0.49277013
0.3591	13785.9583	0.69294616	142.264335	1.69090775	0	-1.0241544	2.6237231	-0.3653609	4.95769387	0.52585674	-6.9077553	13	83	3.57631111	300.222222	27.2222222	0	0	0	1	34.0724638	2	0.49277013
0.54	21368.2194	0.90127434	230.622357	1.94900786	0	-0.6161861	3.06195154	-0.1028367	5.44078589	0.6678334	-6.9077553	14	73	3.57631111	302.444444	29.44444444	0	0	0	1	34.0724638	2	0.49277013
0.8154	34162.9863	1.05420341	306.65094	2.70759054	0	-0.2040765	3.53117206	0.05373355	5.72571336	0.9964284	-6.9077553	15	63	3.12927222	304.111111	31.11111111	0	0	0	1	34.0724638	2	0.49277013
1.1268	45645.2808	1.01666207	291.572029	2.32692441	0	0.11938176	3.82092213	0.01750791	5.6752905	0.84497706	-6.9077553	16	55	2.68223333	306.333333	33.33333333	0	0	0	1	34.0724638	2	0.49277013
1.4292	53137.0403	0.90950448	231.372117	1.85593923	0	0.35711485	3.97289306	-0.0937565	5.44403163	0.61892956	-6.9077553	17	50	3.12927222	307.444444	34.4444444	0	0	0	1	34.0724638	2	0.49277013
1.8927	67530.1295	0.98480308	268.07676	2.36590209	0	0.63800438	4.21258867	-0.0142987	5.59127709	0.86158196	-6.9077553	18	47	3.57631111	307.444444	34.4444444	0	0	0	1	34.0724638	2	0.49277013
2.3913	83233.5609	0.95160375	248.557875	1.90690662	0	0.87183715	4.42166266	-0.0485563	5.51567974	0.64600663	-6.9077553	19	44	2.23519444	308.555556	35.5555556	0	0	0	1	34.0724638	2	0.49277013
3.0393	105939.595	0.98619783	273.432096	2.25268046	0	1.11162723	4.66287851	-0.0128848	5.61105697	0.81256464	-6.9077553	20	43	2.23519444	309.111111	36.11111111	0	0	0	1	34.0724638	2	0.49277013
3.5811	124055.031	0.93735339	257.252025	2.24958593	1	1.27567002	4.82073332	-0.0636286	5.55006013	0.8111906	0.0009995	21	88	5.81150556	296.333333	23.33333333	0	0	0	1	34.0724638	2	0.49277013
3.6819	127213.227	0.53668863	117.008266	1.13255664	1	1.30342892	4.84587249	-0.6204756	4.76225313	0.12536016	0.0009995	22	90	2.23519444	296.888889	23.88888888	0	0	0	1	34.0724638	2	0.49277013
3.744	129625.733	0.30311662	31.3976909	0.60210701	1	1.32015456	4.86465904	-1.190344	3.4467662	-0.5056606	0.0009995	23	91	1.34111667	296.888889	23.88888888	0	0	0	1	34.0724638	2	0.49277013
0.0315	2009.2443	1.14197417	356.084128	2.25834386	0	-3.4577677	0.69825626	0.13363378	5.87516983	0.81507445	-6.9077553	0	62	1.34111667	304.111111	31.11111111	0	0	0	0	42.288408	2	-0.4111751
0.1071	6233.25089	0.97259549	257.475051	1.84525644	0	-2.2339923	1.83005843	-0.0267594	5.55092671	0.61316004	-6.9077553	1	71	1.78815556	301.333333	28.33333333	0	0	0	0	42.288408	2	-0.4111751
0.1845	10002.9866	0.8168256	176.109409	1.5394319	0	-1.6901058	2.30298368	-0.2011062	5.17111112	0.43206283	-6.9077553	2	75	1.78815556	300.222222	27.2222222	0	0	0	0	42.288408	2	-0.4111751
0.2466	12985.7244	0.5823069	84.1593071	1.1192471	0	-1.3999877	2.56392764	-0.5390418	4.4327234	0.11354929	-6.9077553	3	82	1.34111667	299.111111	26.11111111	0	0	0	0	42.288408	2	-0.4111751
0.2826	14652.5474	0.43351154	44.6060852	0.77768826	0	-1.2637228	2.68468245	-0.8335328	3.79789271	-0.2501445	-6.9077553	4	89	1.34111667	298	25	0	0	0	0	42.288408	2	-0.4111751
0.3204	16450.9623	0.33035103	24.1007313	0.5732569	0	-1.1381851	2.80044476	-1.1045769	3.18228367	-0.5546784	-6.9077553	5	90	1.34111667	298	25	0	0	0	0	42.288408	2	-0.4111751
0.3537	18030.0599	0.30706975	21.0043863	0.57711158	0	-1.0393062	2.89209582	-1.1774291	3.04477889	-0.5479884	-6.9077553	6	91	1.34111667	297.444444	24.44444444	0	0	0	0	42.288408	2	-0.4111751
0.3816	19258.2456	0.26429109	15.6041398	0.4567281	0	-0.9633823	2.95799123	-1.3269276	2.74760033	-0.7814799	-6.9077553	7	93	1.34111667	297.444444	24.44444444	0	0	0	0	42.288408	2	-0.4111751
0.4176	20925.0686	0.25991244	14.5824051	0.41023841	0	-0.8732312	3.04099568	-1.3435704	2.67988424	-0.8885822	-6.9077553	8	93	1.78815556	297.444444	24.44444444	0	0	0	0	42.288408	2	-0.4111751
0.4482	22416.438	0.2440512	12.8465971	0.38580937	0	-0.8025157	3.10983914	-1.4062881	2.5531568	-0.9498233	-6.9077553	9	93	2.23519444	297.444444	24.44444444	0	0	0	0	42.288408	2	-0.4111751
0.4842	24039.3969	0.24119186	12.4698578	0.36883197	0	-0.7252572	3.17973562	-1.418025	2.52339455	-0.9947065	-6.9077553	10	94	1.34111667	297.444444	24.44444444	0	0	1	0	42.288408	2	-0.4111751
0.5193	25662.3563	0.27121522	15.8070741	0.42850954	0	-0.6552735	3.24506415	-1.3011623	2.76052083	-0.8451113	-6.9077553	11	92	1.34111667	298	25	0	0	1	0	42.288408	2	-0.4111751
0.5733	28074.8636	0.34216655	33.3541545	0.54862576	0	-0.5563461	3.33491026	-1.0695394	3.50721232	-0.5985177	-6.9077553	12	88	2.68223333	299.111111	26.11111111	0	0	1	0	42.288408	2	-0.4111751
0.8208	35331.1223	0.65309104	126.736406	1.71119662	0	-0.1974758	3.56479253	-0.4245087	4.84211728	0.53777712	-6.9077553	13	83	3.57631111	300.222222	27.2222222	0	0	1	0	42.288408	2	-0.4111751
1.1484	46356.5722	0.78295295	168.116136	1.65252719	0	0.13836967	3.83638465	-0.2434063	5.12466097	0.5029107	-6.9077553	14	73	3.57631111	302.444444	29.44444444	0	0	1	0	42.288408	2	-0.4111751
1.4823	58463.4802	0.79366136	166.146521	1.45715168	0	0.39359494	4.06841939	-0.2298392	5.11287608	0.37716966	-6.9077553	15	63	3.12927222	304.111111	31.11111111	0	0	1	0	42.288408	2	-0.4111751
1.8693	72656.8771	0.84030549	192.361882	1.77959722	0	0.62556403	4.28576181	-0.1728004	5.2593836	0.57694883	-6.9077553	16	55	2.68223333	306.333333	33.33333333	0	0	1	0	42.288408	2	-0.4111751
2.3373	88409.7996	0.83991042	193.530409	1.91881843	0	0.84899642	4.48199413	-0.1732701	5.26543982	0.65223061	-6.9077553	17	50	3.12927222	307.444444	34.4444444	0	0	1	0	42.288408	2	-0.4111751
3.0069	112399.714	0.89189558	224.844422	2.4276	0	1.10090965	4.72207029	-0.1132856	5.41541315	0.88731496	-6.9077553	18	47	3.57631111	307.444444	34.4444444	0	0	1	0	42.288408	2	-0.4111751
3.7512	139748.305	0.8715052	225.703994	2.07048859	0	1.32207579	4.93985014	-0.1363867	5.41922881	0.72826748	-6.9077553	19	44	2.23519444	308.555556	35.5555556	0	0	1	0	42.288408	2	-0.4111751
4.6161	168456.387	0.85863115	222.925891	2.16760953	0	1.52955019	5.12668282	-0.1512519	5.40684387	0.77408619	-6.9077553	20	43	2.23519444	309.111111	36.11111111	0	0	1	0	42.288408	2	-0.4111751
5.1579	181647.88	0.72199277	152.445235	2.17220958	0	1.64052952	5.20207559	-0.3243561	5.02681197	0.77620514	-6.9077553	21	88	5.81150556	296.333333	23.33333333	0	0	1	0	42.288408	2	-0.4111751
5.2452	185244.703	0.36652009	37.6065057	0.60820365	0	1.65731337	5.22168307	-1.0009773	3.62720365	-0.4956027	-6.9077553	22	90	2.23519444	296.888889	23.88888888	0	0	1	0	42.288408	2	-0.4111751
5.3046	187920.397	0.25692683	14.5174505	0.44361762	0	1.66857437	5.23602377	-1.3550794	2.67542029	-0.8105407	-6.9077553	23	91	1.34111667	296.888889	23.88888888	0	0	1	0	42.288408	2	-0.4111751
0.0036	0	0.18133275	6.37634977	0.52180731	0	-5.6268214	-6.9077553	-1.701922	1.85275261	-0.6485423	-6.9077553	0	62	1.34111667	304.111111	31.11111111	0	0	0	0	18.9135952	2	-0.0428739
0.0036	0	0	0	0	0	-5.6268214	-6.9077553	-6.9077553	-6.9077553	-6.9077553	-6.9077553	1	71	1.78815556	301.333333	28.33333333	0	0	0	0	18.9135952	2	-0.0428739
0.0036	0	0	0	0	0	-5.6268214	-6.9077553	-6.9077553	-6.9077553	-6.9077553	-6.9077553	2	75	1.78815556	300.222222	27.2222222	0	0	0	0	18.9135952	2	-0.0428739
0.0036	0	0	0	0	0	-5.6268214	-6.9077553	-6.9077553	-6.9077553	-6.9077553	-6.9077553	3	82	1.34111667	299.111111	26.11111111	0	0	0	0	18.9135952	2	-0.0428739
0.0036	0	0	0	0	0	-5.6268214	-6.9077553	-6.9077553	-6.9077553	-6.9077553	-6.9077553	4	89	1.34111667	298	25	0	0	0	0	18.9135952	2	-0.0428739
0.0036	0	0	0	0	0	-5.6268214	-6.9077553	-6.9077553	-6.9077553	-6.9077553	-6.9077553	5	90	1.34111667	298	25	0	0	0	0	18.9135952	2	-0.0428739
0.0036	0	0	0	0	0	-5.6268214	-6.9077553	-6.9077553	-6.9077553	-6.9077553	-6.9077553	6	91	1.34111667	297.444444	24.44444444	0	0	0	0	18.9135952	2	-0.0428739
0.0036	0	0	0	0	0	-5.6268214	-6.9077553	-6.9077553	-6.9077553	-6.9077553	-6.9077553	7	93	1.34111667	297.444444	24.44444444	0	0	0	0	18.9135952	2	-0.0428739
0.0036	0	0	0	0	0	-5.6268214	-6.9077553	-6.9077553	-6.9077553	-6.9077553	-6.9077553	8	93	1.78815556	297.444444	24.44444444	0	0	0	0	18.9135952	2	-0.0428739
0.0036	0	0	0	0	0	-5.6268214	-6.9077553	-6.9077553	-6.9077553	-6.9077553	-6.9077553	9	93	2.23519444	297.444444	24.44444444	0	0	0	0	18.9135952	2	-0.0428739
0.0036	0	0	0	0	0	-5.6268214	-6.9077553	-6.9077553	-6.9077553	-6.9077553	-6.9077553	10	94	1.34111667	297.444444	24.44444444	0	0	0	0	18.9135952	2	-0.0428739
0.0036	0	0	0	0	0	-5.6268214	-6.9077553	-6.9077553	-6.9077553	-6.9077553	-6.9077553	11	92	1.34111667	298	25	0	0	0	0	18.9135952	2	-0.0428739
0.0036	0	0	0	0	0	-5.6268214	-6.9077553	-6.9077553	-6.9077553	-6.9077553	-6.9077553	12	88	2.68223333	299.111111	26.11111111	0	0	0	0	18.9135952	2	-0.0428739
0.0036	0	0	0	0	0	-5.6268214	-6.9077553	-6.9077553	-6.9077553	-6.9077553	-6.9077553	13	83	3.57631111	300.222222	27.2222222	0	0	0	0	18.9135952	2	-0.0428739
0.0504	0	0.61398902	105.15718	3.00008334	0	-2.9877641	-6.9077553	-0.4861509	4.65546569	1.09897334	-6.9077553	14	73	3.57631111	302.444444	29.44444444	0	0	0	0	18.9135952	2	-0.0428739
0.1647	0	0.51267375	72.0564638	2.35651596	0	-1.8036296	-6.9077553	-0.666167	4.27746391	0.85760851	-6.9077553	15	63	3.12927222	304.111111	31.11111111	0	0	0	0	18.9135952	2	-0.0428739
0.2826	503.763672	0.46645876	66.7009184	1.39773746	0	-1.2637228	-0.6836649	-0.7604441	4.20023371	0.33557002	-6.9077553	16	55	2.68223333	306.333333	33.33333333	0	0	1	0	18.9135952	2	-0.0428739
0.459	5792.72188	0.76319488	207.889609	1.89614884	0	-0.7787051	1.7567749	-0.2689324	5.33701202	0.64035215	-6.9077553	17	50	3.12927222	307.444444	34.4444444	0	0	1	0	18.9135952	2	-0.0428739
0.6444	11531.3823	0.75622825	196.601733	1.77913007	0	-0.4394356	2.44515893	-0.2780905	5.28118511	0.57668643	-6.9077553	18	47	3.57631111	307.444444	34.4444444	0	0	1	0	18.9135952	2	-0.0428739
0.8154	15351.5101	0.63556013	118.576377	1.48555683	0	-0.2040765	2.73127898	-0.4516764	4.77556572	0.3964626	-6.9077553	19	44	2.23519444	308.555556	35.5555556	0	0	1	0	18.9135952	2	-0.0428739
1.017	19793.2253	0.66331577	128.019017	1.45613629	0	0.01685712	2.98539024	-0.4089977	4.85218663	0.37647307	-6.9077553	20	43	2.23519444	309.111111	36.11111111	0	0	1	0	18.9135952	2	-0.0428739
1.2348	24804.4972	0.7980795	242.070296	2.66578964	0	0.21090901	3.21106529	-0.2242948	5.4892323	0.98087537	-6.9077553	21	88	5.81150556	296.333333	23.33333333	0	0	1	0	18.9135952	2	-0.0428739
1.2699	26295.8652	0.36936525	34.5224265	0.51588934	0	0.23893816	3.26944974	-0.9932656	3.54163812	-0.6599265	-6.9077553	22	90	2.23519444	296.888889	23.88888888	0	0	1	0	18.9135952	2	-0.0428739
1.2942	27436.3235	0.27927457	17.580381	0.69319337	0	0.25789274	3.31190426	-1.2719856	2.86684045	-0.3650047	-6.9077553	23	91	1.34111667	296.888889	23.88888888	0	0	1	0	18.9135952	2	-0.0428739
0.0063	223.048959	0.92032154	228.536906	1.5125885	0	-5.0672056	-1.4958907	-0.0819462	5.43170209	0.41448332	-6.9077553	0	85	1.34111667	298.555556	25.55555555	0	0	0	0	30.519848	2	0.21141735
0.0135	530.095457	0.46780521	58.8585358	0.84992516	0	-4.3050656	-0.6328135	-0.7575679	4.07515386	-0.1614311	-6.9077553	1	88	1.34111667	298.555556	25.55555555	0	0	0	0	30.519848	2	0.21141735

0.0315	1144.1884	0.65243378	106.699048	1.16512117	0	-3.4577677	0.13556916	-0.4255141	4.67002161	0.15368301	-6.9077553	2	92	1.34111667	298	25	0	0	0	0	30.519848	2	0.21141735
0.0405	1363.50732	0.57486038	80.5106445	1.06817188	0	-3.2064533	0.31079342	-0.55189	4.38840183	0.06688441	-6.9077553	3	95	1.34111667	297.444444	24.44444444	0	0	0	0	30.519848	2	0.21141735
0.0414	1363.50732	0.15859066	4.74279737	1.19501531	0	-3.1844744	0.31079342	-1.8351431	1.55683795	0.17899546	-6.9077553	4	96	2.23519444	297.444444	24.44444444	0	0	0	0	30.519848	2	0.21141735
0.0414	1363.50732	0	0	0	0	-3.1844744	0.31079342	-6.9077553	-6.9077553	-6.9077553	-6.9077553	5	98	1.78815556	297.444444	24.44444444	0	0	0	0	30.519848	2	0.21141735
0.0414	1363.50732	0	0	0	0	-3.1844744	0.31079342	-6.9077553	-6.9077553	-6.9077553	-6.9077553	6	93	3.12927222	298.555556	25.55555555	0	0	0	0	30.519848	2	0.21141735
0.0954	1653.68286	0.38738661	41.5803467	1.63785896	0	-2.3496767	0.50360936	-0.945754	3.72765167	0.49400025	-6.9077553	7	80	4.02335	300.777778	27.7777778	0	0	0	0	30.519848	2	0.21141735
0.2367	2330.04815	0.54967421	89.0547062	2.47250502	0	-1.4409618	0.84631802	-0.5966119	4.48926209	0.90563618	-6.9077553	8	72	4.47038889	302.444444	29.44444444	0	0	0	0	30.519848	2	0.21141735
0.3168	3405.78521	0.55817469	86.7709467	1.46512029	0	-1.1494846	1.22576909	-0.5812933	4.46328337	0.38261966	-6.9077553	9	62	3.57631111	304.111111	31.11111111	0	0	0	0	30.519848	2	0.21141735
0.4419	4267.64602	0.60350311	96.6503766	1.82246882	0	-0.8166717	1.45129669	-0.5033485	4.57111045	0.60074063	-6.9077553	10	55	3.12927222	305.777778	32.7777778	0	0	0	1	30.519848	2	0.21141735
0.5931	5479.46881	0.60273425	96.3445173	1.72335525	0	-0.5223923	1.70119065	-0.5046212	4.56794087	0.54485321	-6.9077553	11	50	3.57631111	306.888889	33.88888888	0	0	0	1	30.519848	2	0.21141735
0.7902	8329.19359	0.64716475	116.95333	1.64669702	0	-0.2354692	2.1198867	-0.4336104	4.76178352	0.49937857	-6.9077553	12	46	2.68223333	307.444444	34.4444444	0	0	0	1	30.519848	2	0.21141735
1.0287	13902.6865	0.77238447	168.798555	1.83900319	0	0.02829587	2.63215402	-0.256979	5.12871195	0.60976731	-6.9077553	13	41	2.68223333	309.111111	36.11111111	0	0	0	1	30.519848	2	0.21141735
1.2879	19286.8077	0.80703921	186.563883	2.06539366	0	0.25301298	2.95947317	-0.2131447	5.22877908	0.72580489	-6.9077553	14	38	3.12927222	309.666667	36.66666666	0	0	0	1	30.519848	2	0.21141735
1.5795	24347.9634	0.75512455	158.534698	1.81969348	0	0.45710834	3.19248928	-0.2795492	5.06597979	0.59921746	-6.9077553	15	36	3.12927222	309.111111	36.11111111	0	0	0	1	30.519848	2	0.21141735
1.8252	30030.3108	0.78856304	171.608431	1.60243063	0	0.60168957	3.40224053	-0.2362756	5.14522114	0.47214548	-6.9077553	16	38	3.12927222	308.555556	35.5555556	0	0	0	1	30.519848	2	0.21141735
2.0376	35681.3465	0.84003856	194.171842	1.53011529	0	0.71177264	3.57465607	-0.1731178	5.2687487	0.42599642	-6.9077553	17	42	1.34111667	308	35	0	0	0	1	30.519848	2	0.21141735
2.223	41186.5396	0.78017129	175.967005	1.41088482	0	0.79885763	3.71813577	-0.2469608	5.17030219	0.34492556	-6.9077553	18	54	1.34111667	304.666667	31.66666666	0	0	0	1	30.519848	2	0.21141735
2.3274	44573.5583	0.75845771	170.485806	1.23972663	0	0.84475176	3.79716325	-0.2751506	5.13865791	0.2156972	-6.9077553	19	61	0.89407778	303	30	0	0	0	1	30.519848	2	0.21141735
2.3859	46548.3747	0.85220742	195.138161	1.34188033	0	0.86957641	3.84051357	-0.1587526	5.27371295	0.29481681	-6.9077553	20	66	1.34111667	301.888889	28.88888888	0	0	0	1	30.519848	2	0.21141735
2.4129	47195.2341	0.75385017	155.366569	1.13838514	0	0.88082934	3.8543141	-0.281236	5.04579372	0.13048876	-6.9077553	21	66	1.34111667	301.333333	28.33333333	0	0	0	1	30.519848	2	0.21141735
2.4291	47432.4544	0.62760295	110.604872	0.77983475	0	0.88752082	3.85932777	-0.4642555	4.70597318	-0.2473917	-6.9077553	22	69	1.34111667	300.777778	27.7777778	0	0	0	1	30.519848	2	0.21141735
2.4354	47510.1921	0.47060755	52.8341152	0.39132251	0	0.89011101	3.86096531	-0.7516081	3.96717603	-0.935671	-6.9077553	23	72	1.78815556	299.666667	26.66666666	0	0	0	1	30.519848	2	0.21141735
0.0135	613.173276	0.99187047	271.258458	1.79078579	0	-4.3050656	-0.4874782	-0.0071551	5.60307577	0.58321277	-6.9077553	0	85	1.34111667	298.555556	25.55555555	0	0	0	0	34.0724638	2	0.49277013
0.0252	964.083547	0.54392782	69.6566772	0.75407622	0	-3.6809113	-0.0355406	-0.6071019	4.24359292	-0.2809366	-6.9077553	1	88	1.34111667	298.555556	25.55555555	0	0	0	0	34.0724638	2	0.49277013
0.0405	1534.31268	0.53713367	73.3376967	0.87190297	0	-3.2064533	0.42873406	-0.6196483	4.29508839	-0.1359309	-6.9077553	2	92	1.34111667	298	25	0	0	0	0	34.0724638	2	0.49277013
0.054	2060.67796	0.54586849	75.1151123	0.87982769	0	-2.9187712	0.7235202	-0.6035469	4.31903508	-0.1268933	-6.9077553	3	95	1.34111667	297.444444	24.44444444	0	0	0	0	34.0724638	2	0.49277013
0.0801	3157.27268	0.5757049	81.6875505	0.91736353	0	-2.5244794	1.15002526	-0.5504246	4.40291385	-0.085162	-6.9077553	4	96	2.23519444	297.444444	24.44444444	0	0	0	0	34.0724638	2	0.49277013
0.1134	4692.50518	0.57978635	82.0844265	0.89148011	0	-2.1768339	1.54617968	-0.5433723	4.40776049	-0.1137511	-6.9077553	5	98	1.78815556	297.444444	24.44444444	0	0	0	0	34.0724638	2	0.49277013
0.1575	6754.10273	0.59647852	88.123017	0.9217815	0	-1.8483298	1.91029818	-0.5150369	4.47874511	-0.0803628	-6.9077553	6	93	3.12927222	298.555556	25.55555555	0	0	0	0	34.0724638	2	0.49277013
0.2097	9037.23225	0.66530938	115.539509	1.03937208	0	-1.5620773	2.20146361	-0.4060012	4.74962119	0.03957842	-6.9077553	7	80	4.02335	300.777778	27.7777778	0	0	0	0	34.0724638	2	0.49277013
0.3564	14720.9759	0.94900297	259.95231	2.71466533	0	-1.0317016	2.68934134	-0.0512902	5.56050204	0.99903698	-6.9077553	8	72	4.47038889	302.444444	29.44444444	0	0	0	1	34.0724638	2	0.49277013
0.5544	24670.1164	1.08944647	332.022337	2.67527632	0	-0.5898688	3.20563318	0.08658721	5.80520526	0.9844264	-6.9077553	9	62	3.57631111	304.111111	31.11111111	0	0	0	1	34.0724638	2	0.49277013
0.8568	39468.1262	1.0837653	325.411784	2.52178592	0	-0.1545508	3.67551875	0.08136365	5.78509448	0.92536381	-6.9077553	10	55	3.12927222	305.777778	32.7777778	0	0	0	1	34.0724638	2	0.49277013
1.2105	50300.903	1.0644911	324.375119	2.45628656	0	0.1910335	3.91804291	0.06343582	5.7819037	0.89905772	-6.9077553	11	50	3.57631111	306.888889	33.88888888	0	0	0	1	34.0724638	2	0.49277013
1.5678	60637.5131	0.93968005	241.783139	1.91125765	0	0.44967336	4.10493022	-0.0611522	5.48804534	0.64828456	-6.9077553	12	46	2.68223333	307.444444	34.4444444	0	0	0	1	34.0724638	2	0.49277013
2.0115	73122.4316	0.99135393	271.915758	2.24466332	0	0.69888071	4.29214886	-0.0076755	5.60549598	0.80900094	-6.9077553	13	41	2.68223333	309.111111	36.11111111	0	0	0	1	34.0724638	2	0.49277013
2.6667	98409.4642	1.08358429	331.196085	2.47956395	0	0.98084175	4.58914714	0.08119677	5.80271362	0.90848594	-6.9077553	14	38	3.12927222	309.666667	36.66666666	0	0	0	1	34.0724638	2	0.49277013
3.357	118224.086	1.04151887	314.980728	2.49926997	0	1.21104772	4.77259031	0.04163977	5.75251463	0.91639871	-6.9077553	15	36	3.12927222	309.111111	36.11111111	0	0	0	1	34.0724638	2	0.49277013
3.9375	134662.111	0.96245799	262.151986	1.99847649	0	1.370546	4.90277618	-0.0372264	5.56892825	0.69288539	-6.9077553	16	38	3.12927222	308.555556	35.5555556	0	0	0	1	34.0724638	2	0.49277013
4.365	147262.794	0.85077136	201.010691	1.50252082	0	1.47361819	4.9922255	-0.1604371	5.30336307	0.40780957	-6.9077553	17	42	1.34111667	308	35	0	0	0	1	34.0724638	2	0.49277013
4.7853	159578.492	0.78665704	173.421373	1.53396822	0	1.56554872	5.07254218	-0.2386925	5.15573008	0.42850968	-6.9077553	18	54	1.34111667	304.666667	31.66666666	0	0	0	1	34.0724638	2	0.49277013
5.094	171336.195	0.7989114	183.833364	1.37848594	0	1.62806338	5.14363352	-0.2232543	5.21403515	0.32171093	-6.9077553	19	61	0.89407778	303	30	0	0	0	1	34.0724638	2	0.49277013
5.3235	181409.204	0.78229777	161.172273	1.23921724	0	1.67213098	5.20076079	-0.2442424	5.08248002	0.21528656	-6.9077553	20	66	1.34111667	301.888889	28.88888888	0	0	0	1	34.0724638	2	0.49277013
5.5512	192045.677	0.79723204	170.759048	1.33179193	0	1.71401412	5.25773845	-0.2253559	5.14025934	0.28727594	-6.9077553	21	66	1.34111667	301.333333	28.33333333	0	0	0	1	34.0724638	2	0.49277013
5.7492	200434.926	0.76458491	154.271751	1.21280101	0	1.74906071	5.30049462	-0.2671152	5.03872214	0.19375677	-6.9077553	22	69	1.34111667	300.777778	27.7777778	0	0	0	1	34.0724638	2	0.49277013
5.9085	207064.949	0.72598672	137.195986	1.10713463	0	1.77639199	5.33303734	-0.3188471	4.92141774	0.10267809	-6.9077553	23	72	1.78815556	299.666667	26.66666666	0	0	0	1	34.0724638	2	0.49277013
0.0261	1456.06887	1.03450993	288.446899	1.9727508	0	-3.64582	0.37642679	0.03489399	5.66451448	0.67993569	-6.9077553	0	85	1.34111667	298.555556	25.55555555	0	0	0	0	42.288408	2	-0.4111751
0.0711	3653.16298	0.71402351	130.787422	1.27576279	0	-2.6436679	1.29586706	-0.3354399	4.87358092	0.2443278	-6.9077553	1	88	1.34111667	298.555556	25.55555555	0	0	0	0	42.288408	2	-0.4111751
0.1224	6021.80788	0.61283426	95.4029691	1.05649352	0	-2.1004609	1.79555358	-0.4880303	4.55812018	0.05590151	-6.9077553	2	92	1.34111667	298	25	0	0	0	0	42.288408	2	-0.4111751
0.1845	9004.54461	0.64815389	106.054355	1.14659273	0	-1.6901058	2.19784046	-0.4320855	4.66396117	0.13766647	-6.9077553	3	95	1.34111667	297.444444	24.44444444	0	0	0	0	42.288408	2	-0.4111751
0.2538	12250.4649	0.65276896	106.926111	1.17731565	0	-1.3712087	2.50564551	-0.4250013	4.6721474	0.164086	-6.9077553	4	96	2.23519444	297.444444	24.44444444	0	0	0	0	42.288408	2	-0.4111751
0.3321	15935.0225	0.68436717	118.78455	1.29207076	0	-1.1023192	2.76858212	-0.3778006	4.77731977	0.25701982	-6.9077553	5	98	1.78815556	297.444444	24.44444444	0	0	0	0	42.288408	2	-0.4111751
0.4293	20368.5739	0.70472767	129.45921	1.29205096	0	-0.8455993	3.01404231	-0.3485259	4.86337358	0.25700451	-6.9077553	6	93	3.12927222	298.555556	25.55555555	0	0	0	0	42.288408	2	-0.4111751
0.6633	27851.1337	0.72428272	149.626412	2.20201416	0	-0.4105279	3.32690958	-0.3211937	5.00814829	0.7898265	-6.9077553	7	80	4.02335	300.777778	27.7777778	0	0	1	0	42.288408	2	-0.4111751
1.0098	40408.2637	0.86575975	206.590138	2.08032401	0	0.00975229	3.69905906	-0.1429934	5.33074166	0.73300423	-6.9077553	8	72	4.47038889	302.444444	29.44444444	0	0	1	0	42.288408	2	-0.4111751
1.3563	54696.0962	0.85844877	193.649026	1.57914516	0	0.3047604	4.00181062	-0.1514641	5.26605254	0.45751672	-6.9077553	9	62	3.57631111	304.111111	31.11111111	0	0	1	0	42.288408	2	-0.4111751
1.7712	71880.4142	0.88519201	213.890557	1.65423372	0	0.57165728	4.27501774	-0.1208216	5.36546914	0.50394222	-6.9077553	10	55	3.12927222	305.777778	32.7777778	0	0	1	0	42.288408	2	-0.4111751
2.3076	92827.1076	0.90729804	230.253487	1.99372649	0	0.83620802	4.53074948	-0.0961827	5.43918516	0.69050694	-6.9077553	11	50	3.57631111	306.888889	33.88888888	0	0	1	0	42.288408	2	-0.4111751
2.9745	118820.147	0.91742611	245.795673	2.16446281	0	1.09007596	4.77761939	-0.0850938	5.50450466	0.77263411	-6.9077553	12	46	2.68223333	307.444444	34.4444444	0	0	1	0	42.288408	2	-0.4111751
3.7926	152223.406	0.98252971	281.596999	2.52370769	0	1.3330518	5.02535579	-0.0166074	5.64048052	0.92612529	-6.9077553	13	41	2.68223333	309.111111	36.11111111	0	0	1	0	42.288408	2	-0.4111751
4.5036	182040.31	0.95997848	267.117484	2.18780209	0	1.50487708	5.20423364	-0.0398033	5.58769232	0.7833544	-6.9077553	14	38	3.12927222	309.666667	36.66666666	0	0	1	0	42.288408	2	-0.4111751
5.2092	211907.987	0.95941754	268.034279	2.08628814	0	1.65042629	5.35615688	-0.0403872	5.59111861	0.73586568	-6.9077553	15	36	3.12927222	309.111111	36.11111111	0	0	1	0	42.288408	2	-0.4111751

5.823	233745.357	0.86332278	233.213526	2.01717021	0	1.76181559	5.45423658	-0.145809	5.45195874	0.70219126	-6.9077553	16	38	3.12927222	308.555556	35.5555556	0	0	1	0	42.288408	2	-0.4111751
6.3198	248284.179	0.71941741	167.827516	1.52592048	0	1.84368756	5.514578	-0.3279245	5.12294272	0.42325295	-6.9077553	17	42	1.34111667	308	35	0	0	1	0	42.288408	2	-0.4111751
6.6987	258754.984	0.64016281	137.855237	1.26451158	0	1.90191348	5.55588547	-0.4444719	4.92621138	0.23547645	-6.9077553	18	54	1.34111667	304.666667	31.66666666	0	0	1	0	42.288408	2	-0.4111751
6.9102	266978.548	0.76969247	176.472822	1.28225955	0	1.93299858	5.58717206	-0.2604659	5.17317255	0.24940336	-6.9077553	19	61	0.89407778	303	30	0	0	1	0	42.288408	2	-0.4111751
7.047	272867.151	0.78298428	164.869822	1.23208949	0	1.95260199	5.60898872	-0.2433663	5.10516227	0.2095228	-6.9077553	20	66	1.34111667	301.888889	28.88888888	0	0	1	0	42.288408	2	-0.4111751
7.1793	278945.967	0.78656047	163.176871	1.24611129	0	1.97120189	5.63102168	-0.2388151	5.09484084	0.22082991	-6.9077553	21	66	1.34111667	301.333333	28.33333333	0	0	1	0	42.288408	2	-0.4111751
7.3044	284356.824	0.77835368	159.576495	1.26704242	0	1.98847691	5.65023339	-0.2492903	5.07252967	0.23747431	-6.9077553	22	69	1.34111667	300.777778	27.7777778	0	0	1	0	42.288408	2	-0.4111751
7.4061	288193.797	0.6916566	126.139804	1.14449884	0	2.00230398	5.66363663	-0.3672209	4.83739878	0.13584021	-6.9077553	23	72	1.78815556	299.666667	26.66666666	0	0	1	0	42.288408	2	-0.4111751
0.0009	0	0	0	0	0	-7.0131158	-6.9077553	-6.9077553	-6.9077553	-6.9077553	-6.9077553	0	85	1.34111667	298.555556	25.55555555	0	0	0	0	18.9135952	2	-0.0428739
0.0009	0	0	0	0	0	-7.0131158	-6.9077553	-6.9077553	-6.9077553	-6.9077553	-6.9077553	1	88	1.34111667	298.555556	25.55555555	0	0	0	0	18.9135952	2	-0.0428739
0.0009	0	0	0	0	0	-7.0131158	-6.9077553	-6.9077553	-6.9077553	-6.9077553	-6.9077553	2	92	1.34111667	298	25	0	0	0	0	18.9135952	2	-0.0428739
0.0009	0	0	0	0	0	-7.0131158	-6.9077553	-6.9077553	-6.9077553	-6.9077553	-6.9077553	3	95	1.34111667	297.444444	24.44444444	0	0	0	0	18.9135952	2	-0.0428739
0.0009	0	0	0	0	0	-7.0131158	-6.9077553	-6.9077553	-6.9077553	-6.9077553	-6.9077553	4	96	2.23519444	297.444444	24.44444444	0	0	0	0	18.9135952	2	-0.0428739
0.0009	0	0	0	0	0	-7.0131158	-6.9077553	-6.9077553	-6.9077553	-6.9077553	-6.9077553	5	98	1.78815556	297.444444	24.44444444	0	0	0	0	18.9135952	2	-0.0428739
0.0009	0	0	0	0	0	-7.0131158	-6.9077553	-6.9077553	-6.9077553	-6.9077553	-6.9077553	6	93	3.12927222	298.555556	25.55555555	0	0	0	0	18.9135952	2	-0.0428739
0.0009	0	0	0	0	0	-7.0131158	-6.9077553	-6.9077553	-6.9077553	-6.9077553	-6.9077553	7	80	4.02335	300.777778	27.7777778	0	0	0	0	18.9135952	2	-0.0428739
0.0333	0	0.55429681	86.8930393	2.53492567	0	-3.4021979	-6.9077553	-0.5882525	4.46468944	0.93055873	-6.9077553	8	72	4.47038889	302.444444	29.44444444	0	0	0	0	18.9135952	2	-0.0428739
0.1449	272.065583	0.57992197	90.5210827	2.27067615	0	-1.9317114	-1.2980433	-0.5431388	4.50559383	0.82051795	-6.9077553	9	62	3.57631111	304.111111	31.11111111	0	0	0	0	18.9135952	2	-0.0428739
0.3411	3272.93545	0.68527418	153.902971	2.30968706	0	-1.0755796	1.18599276	-0.3764781	5.03632884	0.83754491	-6.9077553	10	55	3.12927222	305.777778	32.7777778	0	0	1	0	18.9135952	2	-0.0428739
0.5319	10893.1977	0.89106412	274.339659	2.19078985	0	-0.6312998	2.38823033	-0.1142173	5.61437062	0.78471849	-6.9077553	11	50	3.57631111	306.888889	33.88888888	0	0	1	0	18.9135952	2	-0.0428739
0.675	15535.9577	0.73561039	177.617826	1.38374837	0	-0.3930426	2.74322156	-0.3056962	5.17963983	0.32551844	-6.9077553	12	46	2.68223333	307.444444	34.4444444	0	0	1	0	18.9135952	2	-0.0428739
0.8442	19766.3478	0.73236936	166.671272	1.41443764	0	-0.1693658	2.98403147	-0.3101058	5.11602944	0.34743877	-6.9077553	13	41	2.68223333	309.111111	36.11111111	0	0	1	0	18.9135952	2	-0.0428739
1.1034	28899.0685	0.90375635	247.552816	2.25933011	0	0.09839632	3.36384396	-0.1000896	5.511628	0.81551087	-6.9077553	14	38	3.12927222	309.666667	36.66666666	0	0	1	0	18.9135952	2	-0.0428739
1.4373	39945.4022	0.89686539	241.708179	2.28469659	0	0.36276635	3.68753861	-0.1077351	5.48773526	0.82667083	-6.9077553	15	36	3.12927222	309.111111	36.11111111	0	0	1	0	18.9135952	2	-0.0428739
1.7316	50985.158	0.85906973	221.55366	1.79589937	0	0.54904584	3.93155418	-0.1507418	5.40066933	0.58606261	-6.9077553	16	38	3.12927222	308.555556	35.5555556	0	0	1	0	18.9135952	2	-0.0428739
2.0304	61690.3592	0.7975951	192.598903	1.53971697	0	0.70823282	4.12214388	-0.2249012	5.260615	0.43224788	-6.9077553	17	42	1.34111667	308	35	0	0	1	0	18.9135952	2	-0.0428739
2.4021	76113.5074	0.84280226	209.1676	1.71734102	0	0.87634335	4.33223888	-0.1698371	5.34314062	0.5413593	-6.9077553	18	54	1.34111667	304.666667	31.66666666	0	0	1	0	18.9135952	2	-0.0428739
2.7018	90146.7126	0.91609939	235.535215	1.64455874	0	0.99391822	4.50144958	-0.0865394	5.46186468	0.49807999	-6.9077553	19	61	0.89407778	303	30	0	0	1	0	18.9135952	2	-0.0428739
2.9016	99430.6401	0.8727538	205.800306	1.5288387	0	1.06526231	4.59947037	-0.1349566	5.32691117	0.4251623	-6.9077553	20	66	1.34111667	301.888889	28.88888888	0	0	1	0	18.9135952	2	-0.0428739
3.0762	107614.729	0.8176043	179.292224	1.39902017	0	1.12369507	4.67856682	-0.2001545	5.18902259	0.33648664	-6.9077553	21	66	1.34111667	301.333333	28.33333333	0	0	1	0	18.9135952	2	-0.0428739
3.2805	117041.971	0.83756609	191.670085	1.60675802	0	1.18799585	4.76254114	-0.1760619	5.25578081	0.47484067	-6.9077553	22	69	1.34111667	300.777778	27.7777778	0	0	1	0	18.9135952	2	-0.0428739
3.4911	127047.224	0.86065139	201.619992	1.65340248	0	1.25021687	4.84456673	-0.1489045	5.30638966	0.5034399	-6.9077553	23	72	1.78815556	299.666667	26.66666666	0	0	1	0	18.9135952	2	-0.0428739
0.009	362.087166	0.83348672	188.78437	1.51333947	0	-4.7105307	-1.0131123	-0.1809385	5.24061076	0.41497935	-6.9077553	0	88	4.02335	299.111111	26.11111111	0	0	0	0	30.519848	2	0.21141735
0.0135	581.406165	0.45678282	52.4263855	0.78331709	0	-4.3050656	-0.5405872	-0.7813604	3.95942908	-0.2429419	-6.9077553	1	89	3.57631111	298.555556	25.55555555	0	0	0	0	30.519848	2	0.21141735
0.0252	1063.90778	0.6133501	91.0814303	0.97709216	0	-3.6809113	0.0628882	-0.4871903	4.51176492	-0.0221514	-6.9077553	2	90	4.02335	298.555556	25.55555555	0	0	0	0	30.519848	2	0.21141735
0.036	1327.09043	0.58530339	86.0154419	1.03570461	0	-3.3242363	0.28374214	-0.5339179	4.45453846	0.03604704	-6.9077553	3	89	3.57631111	298.555556	25.55555555	0	0	0	0	30.519848	2	0.21141735
0.063	1634.13679	0.29583213	27.1829427	1.05611623	0	-2.7646206	0.49172647	-1.2145885	3.30262646	0.05554466	-6.9077553	4	84	4.02335	299.666667	26.66666666	0	0	0	0	30.519848	2	0.21141735
0.1368	1749.88184	0.40628987	43.6858119	1.89363284	0	-1.9892353	0.56011957	-0.8982301	3.77704627	0.63902507	-6.9077553	5	76	4.91742778	301.333333	28.33333333	0	0	0	0	30.519848	2	0.21141735
0.2646	2742.63108	0.6745757	139.777536	3.30508251	0	-1.329536	1.00928225	-0.3921901	4.94005929	1.19576396	-6.9077553	6	73	5.36446667	301.888889	28.88888888	0	0	0	0	30.519848	2	0.21141735
0.3402	3542.14263	0.55339436	82.8271775	1.45693398	0	-1.0782216	1.26501408	-0.589879	4.41676831	0.37702035	-6.9077553	7	63	6.25854444	304.111111	31.11111111	0	0	0	0	30.519848	2	0.21141735
0.4905	4400.49009	0.60454112	93.5876567	1.83855415	0	-0.71233	1.48194314	-0.5016328	4.53890919	0.60952323	-6.9077553	8	58	5.81150556	305.222222	32.2222222	0	0	0	1	30.519848	2	0.21141735
0.6885	6572.50019	0.67121013	124.448207	2.07282909	0	-0.37324	1.88304644	-0.3971843	4.82389765	0.7293967	-6.9077553	9	55	4.47038889	306.333333	33.33333333	0	0	0	1	30.519848	2	0.21141735
0.9621	12144.9667	0.82430132	198.328767	2.68952219	0	-0.0386369	2.49699716	-0.1920067	5.28993114	0.9897353	-6.9077553	10	51	5.36446667	306.888889	33.88888888	0	0	0	1	30.519848	2	0.21141735
1.2177	17580.635	0.74916367	151.612442	1.77877614	0	0.19696383	2.86685489	-0.2874639	5.02133414	0.57648759	-6.9077553	11	52	4.47038889	307.444444	34.4444444	0	0	0	1	30.519848	2	0.21141735
1.5228	25019.1848	0.76344952	168.41518	1.80135199	0	0.42055075	3.21968289	-0.2685993	5.12643818	0.58909247	-6.9077553	12	50	4.02335	308	35	0	0	0	1	30.519848	2	0.21141735
1.9251	32357.7736	0.79009272	208.282404	2.20826533	0	0.65497791	3.47688519	-0.2343401	5.33889967	0.79266003	-6.9077553	13	51	3.57631111	308	35	0	0	0	1	30.519848	2	0.21141735
2.268	43214.6242	0.66934016	127.3117	1.630687	0	0.81889839	3.7662021	-0.39997	4.84664627	0.48961445	-6.9077553	14	51	4.02335	307.444444	34.4444444	0	0	0	1	30.519848	2	0.21141735
2.493	58878.2995	0.66196082	125.685891	1.01270911	0	0.9134868	4.07548958	-0.4110394	4.83379382	0.01361599	-6.9077553	15	54	2.68223333	306.888889	33.88888888	0	0	0	1	30.519848	2	0.21141735
2.6478	74266.0344	0.71221188	145.896462	0.96353149	0	0.97372911	4.30766717	-0.3379767	4.98290406	-0.0361128	-6.9077553	16	61	0.89407778	304.666667	31.66666666	0	0	0	1	30.519848	2	0.21141735
2.7432	88926.9247	0.72046575	137.375967	0.91719393	0	1.00912512	4.48782621	-0.3264704	4.92272873	-0.0853467	-6.9077553	17	72	2.68223333	303	30	0	0	0	1	30.519848	2	0.21141735
2.7864	91885.0163	0.54863608	74.8003184	0.80483047	0	1.02475044	4.52054886	-0.5984989	4.31483551	-0.2158819	-6.9077553	18	74	3.57631111	301.888889	28.88888888	0	0	0	1	30.519848	2	0.21141735
2.8053	92542.9749	0.41516218	39.5350371	0.60765775	0	1.03151049	4.52768394	-0.8766802	3.67721259	-0.4964992	-6.9077553	19	78	2.68223333	300.777778	27.7777778	0	0	0	1	30.519848	2	0.21141735
2.817	93025.4718	0.32090026	22.2100689	0.47629166	0	1.03567249	4.5328841	-1.1335135	3.10059076	-0.7396275	-6.9077553	20	80	2.68223333	300.222222	27.2222222	0	0	0	1	30.519848	2	0.21141735
2.8278	93288.6544	0.25324339	14.3143336	0.39586445	0	1.03949902	4.53570922	-1.3694633	2.66133124	-0.9241605	-6.9077553	21	82	2.68223333	299.666667	26.66666666	0	0	0	1	30.519848	2	0.21141735
2.835	93507.9687	0.27292672	15.4718103	0.4205305	0	1.04204194	4.53805735	-1.2948947	2.73908431	-0.8638631	-6.9077553	22	85	1.78815556	298.555556	25.55555555	0	0	0	1	30.519848	2	0.21141735
2.8404	93695.5965	0.26165827	14.1266225	0.39710399	0	1.04394489	4.54006187	-1.3369014	2.64813192	-0.921042	-6.9077553	23	89	2.23519444	298.555556	25.55555555	0	0	0	1	30.519848	2	0.21141735
0.0171	753.545192	0.99964142	280.43202	2.14953253	0	-4.0686768	-0.2816401	0.00064121	5.63633491	0.7657155	-6.9077553	0	88	4.02335	299.111111	26.11111111	0	0	0	0	34.0724638	2	0.49277013
0.0324	1323.77448	0.53708396	70.1437486	0.8672855	0	-3.4295969	0.28124224	-0.6197407	4.25056094	-0.1412347	-6.9077553	1	89	3.57631111	298.555556	25.55555555	0	0	0	0	34.0724638	2	0.49277013
0.0504	1894.00357	0.50210943	62.8124023	0.82798386	0	-2.9877641	0.63922072	-0.6869476	4.14016846	-0.1875546	-6.9077553	2	90	4.02335	298.555556	25.55555555	0	0	0	0	34.0724638	2	0.49277013
0.0765	2946.73433	0.62547539	97.323318	1.08561272	0	-2.5704645	1.08103685	-0.4676458	4.57804889	0.08306527	-6.9077553	3	89	3.57631111	298.555556	25.55555555	0	0	0	0	34.0724638	2	0.49277013
0.1188	4876.74072	0.66973577	111.660229	1.03471188	0	-2.1303139	1.58468214	-0.39938	4.71546955	0.035089	-6.9077553	4	84	4.02335	299.666667	26.66666666	0	0	0	0	34.0724638	2	0.49277013
0.1971	8241.1444	0.8062909	171.750449	1.50958041	0	-1.6240441	2.10926055	-0.2140712	5.14604837	0.41249396	-6.9077553	5	76	4.91742778	301.333333	28.33333333	0	0	0	0	34.0724638	2	0.49277013

0.3681	15902.1367	1.03873877	306.5081	2.7203684	0	-0.9994006	2.76651637	0.0389695	5.72524745	1.00113484	-6.9077553	6	73	5.36446667	301.888889	28.88888888	0	0	0	1	34.0724638	2	0.49277013
0.8775	34860.8737	1.39320875	586.821665	5.09484561	1	-0.1306783	3.55139379	0.33232705	6.37472267	1.62842562	0.0009995	7	63	6.25854444	304.111111	31.11111111	0	0	0	1	34.0724638	2	0.49277013
1.665	57077.7914	1.40240569	602.101857	4.92047991	2	0.50982512	4.04443262	0.33890192	6.40042829	1.59360928	0.69364706	8	58	5.81150556	305.222222	32.2222222	0	0	0	1	34.0724638	2	0.49277013
2.25	74797.4336	1.05558772	314.427692	2.67852614	2	0.81093022	4.31479694	0.05504459	5.75075732	0.98563997	0.69364706	9	55	4.47038889	306.333333	33.33333333	0	0	0	1	34.0724638	2	0.49277013
2.9142	98168.5777	1.10136484	355.528201	2.87571621	2	1.06959534	4.58669637	0.09745773	5.87360739	1.05664944	0.69364706	10	51	5.36446667	306.888889	33.88888888	0	0	0	1	34.0724638	2	0.49277013
3.5532	122700.708	1.03044563	297.31239	2.41657113	2	1.26784861	4.80975627	0.03096134	5.69478677	0.88276337	0.69364706	11	52	4.47038889	307.444444	34.4444444	0	0	0	1	34.0724638	2	0.49277013
4.1859	142786.376	0.93347906	243.205503	2.52913539	2	1.43172173	4.96135664	-0.0677661	5.49391089	0.92827281	0.69364706	12	50	4.02335	308	35	0	0	0	1	34.0724638	2	0.49277013
4.7799	160749.275	0.85852624	211.147822	2.06718824	2	1.56441963	5.07985207	-0.1513739	5.3525632	0.72667298	0.69364706	13	51	3.57631111	308	35	0	0	0	1	34.0724638	2	0.49277013
5.2443	172488.364	0.74933666	157.573704	1.72336975	2	1.65714177	5.15033558	-0.2872333	5.05989966	0.54486162	0.69364706	14	51	4.02335	307.444444	34.4444444	0	0	0	1	34.0724638	2	0.49277013
5.643	182507.787	0.69242759	138.822279	1.44177742	2	1.73041584	5.20679832	-0.3661085	4.93320175	0.36657002	0.69364706	15	54	2.68223333	306.888889	33.88888888	0	0	0	1	34.0724638	2	0.49277013
5.8995	191241.849	0.73891607	153.425329	1.1685549	2	1.7748676	5.25354408	-0.3012185	5.03322051	0.15662325	0.69364706	16	61	0.89407778	304.666667	31.66666666	0	0	0	1	34.0724638	2	0.49277013
6.0867	199487.733	0.76815216	155.842633	1.20858721	2	1.80610606	5.29575776	-0.2624665	5.04885315	0.19027915	0.69364706	17	72	2.68223333	303	30	0	0	0	1	34.0724638	2	0.49277013
6.2289	205673.958	0.55616181	77.6754846	0.88050804	2	1.82919975	5.32629705	-0.5848996	4.35255257	-0.1261212	0.69364706	18	74	3.57631111	301.888889	28.88888888	0	0	0	1	34.0724638	2	0.49277013
6.3207	209710.014	0.39478571	37.7494471	0.64081671	2	1.84382996	5.34573046	-0.9268823	3.63099732	-0.4434525	0.69364706	19	78	2.68223333	300.777778	27.7777778	0	0	0	1	34.0724638	2	0.49277013
6.3891	212430.323	0.30858123	27.8812963	0.55438027	2	1.85459341	5.35861875	-1.1725348	3.32799194	-0.5881022	0.69364706	20	80	2.68223333	300.222222	27.2222222	0	0	0	1	34.0724638	2	0.49277013
6.4449	214843.545	0.26871177	19.8260222	0.38753614	2	1.86328912	5.36991472	-1.3104014	2.98704577	-0.9453691	0.69364706	21	82	2.68223333	299.666667	26.66666666	0	0	0	1	34.0724638	2	0.49277013
6.4962	216773.558	0.22121192	10.2521437	0.30518642	2	1.87121739	5.37885791	-1.5041238	2.32758436	-1.1835611	0.69364706	22	85	1.78815556	298.555556	25.55555555	0	0	0	1	34.0724638	2	0.49277013
6.5412	218747.406	0.20513309	8.61181763	0.35294041	2	1.87812063	5.38792224	-1.5792332	2.15325152	-1.0386267	0.69364706	23	89	2.23519444	298.555556	25.55555555	0	0	0	1	34.0724638	2	0.49277013
0.0324	2095.63607	1.14051328	359.205022	2.45205165	0	-3.4295969	0.74033419	0.13235482	5.8838961	0.89733282	-6.9077553	0	88	4.02335	299.111111	26.11111111	0	0	0	0	42.288408	2	-0.4111751
0.0801	4433.46389	0.72608473	138.195469	1.40749086	0	-2.5244794	1.48940673	-0.3187123	4.92867636	0.34251882	-6.9077553	1	89	3.57631111	298.555556	25.55555555	0	0	0	0	42.288408	2	-0.4111751
0.1404	7284.69782	0.66798777	115.188047	1.236607	0	-1.9632598	1.98591322	-0.4019895	4.74657466	0.21317967	-6.9077553	2	90	4.02335	298.555556	25.55555555	0	0	0	0	42.288408	2	-0.4111751
0.2016	10179.7078	0.66027591	111.313125	1.22178257	0	-1.6014697	2.32049454	-0.4135841	4.71235616	0.20112906	-6.9077553	3	89	3.57631111	298.555556	25.55555555	0	0	0	0	42.288408	2	-0.4111751
0.2979	14822.1975	0.84091999	193.508415	1.609696	0	-1.2109974	2.69619335	-0.1720703	5.26532617	0.47666639	-6.9077553	4	84	4.02335	299.666667	26.66666666	0	0	0	0	42.288408	2	-0.4111751
0.5814	25237.6682	0.87495156	216.938988	2.6027429	0	-0.5423163	3.22837727	-0.1324445	5.37962076	0.95694999	-6.9077553	5	76	4.91742778	301.333333	28.33333333	0	0	0	0	42.288408	2	-0.4111751
0.9162	37113.8187	0.87858286	207.406082	2.31157594	0	-0.0875206	3.61401632	-0.1283075	5.33468344	0.83836203	-6.9077553	6	73	5.36446667	301.888889	28.88888888	0	0	1	0	42.288408	2	-0.4111751
1.3014	52500.3058	0.8922666	214.060383	2.13218546	0	0.26344061	3.96083804	-0.1128702	5.36626281	0.75761639	-6.9077553	7	63	6.25854444	304.111111	31.11111111	0	0	1	0	42.288408	2	-0.4111751
1.8666	73686.0197	0.92798318	245.501567	2.45056347	0	0.62411859	4.29982666	-0.0736646	5.50330741	0.89672597	-6.9077553	8	58	5.81150556	305.222222	32.2222222	0	0	1	0	42.288408	2	-0.4111751
2.5362	101552.193	0.96888372	261.758359	2.45741518	0	0.9306669	4.62058273	-0.0305791	5.5674256	0.89951691	-6.9077553	9	55	4.47038889	306.333333	33.33333333	0	0	1	0	42.288408	2	-0.4111751
3.2103	132492.193	0.99418031	279.872747	2.24000197	0	1.16636439	4.88653127	-0.0048313	5.6343386	0.80692307	-6.9077553	10	51	5.36446667	306.888889	33.88888888	0	0	1	0	42.288408	2	-0.4111751
4.0131	169419.033	1.04152525	323.178251	2.60189231	0	1.38956401	5.13238103	0.0416459	5.77820713	0.95662325	-6.9077553	11	52	4.47038889	307.444444	34.4444444	0	0	1	0	42.288408	2	-0.4111751
4.6854	195102.507	0.9170082	243.532986	2.33319966	0	1.54445129	5.27353022	-0.085549	5.49525651	0.84766907	-6.9077553	12	50	4.02335	308	35	0	0	1	0	42.288408	2	-0.4111751
5.2812	216968.975	0.84174709	203.724533	1.91889737	0	1.66415334	5.37975898	-0.1710884	5.31677366	0.65227173	-6.9077553	13	51	3.57631111	308	35	0	0	1	0	42.288408	2	-0.4111751
5.7555	232993.533	0.76418222	165.763787	1.51213094	0	1.75015592	5.45101499	-0.2676413	5.11056984	0.41418097	-6.9077553	14	51	4.02335	307.444444	34.4444444	0	0	1	0	42.288408	2	-0.4111751
6.1443	247070.032	0.77948634	181.234158	1.42725203	0	1.81552482	5.50967588	-0.247838	5.1997954	0.35645134	-6.9077553	15	54	2.68223333	306.888889	33.88888888	0	0	1	0	42.288408	2	-0.4111751
6.4287	257884.576	0.8114286	191.466253	1.38156476	0	1.86077234	5.55251598	-0.2077272	5.25471679	0.32394029	-6.9077553	16	61	0.89407778	304.666667	31.66666666	0	0	1	0	42.288408	2	-0.4111751
6.5601	263199.322	0.72804558	146.34664	1.20150119	0	1.88100585	5.57291542	-0.316019	4.98598488	0.18440372	-6.9077553	17	72	2.68223333	303	30	0	0	1	0	42.288408	2	-0.4111751
6.6564	267324.322	0.57266599	93.3525117	1.04017718	0	1.8955788	5.58846635	-0.555708	4.53639349	0.04035197	-6.9077553	18	74	3.57631111	301.888889	28.88888888	0	0	1	0	42.288408	2	-0.4111751
6.714	269780.68	0.36886001	36.0621643	0.62365329	0	1.9041949	5.59761304	-0.9946307	3.58527197	-0.4705585	-6.9077553	19	78	2.68223333	300.777778	27.7777778	0	0	1	0	42.288408	2	-0.4111751
6.7455	271228.201	0.26662628	14.8943726	0.35131138	0	1.90887562	5.60296422	-1.3181638	2.7010506	-1.0432399	-6.9077553	20	80	2.68223333	300.222222	27.2222222	0	0	1	0	42.288408	2	-0.4111751
6.7851	272807.285	0.24606523	12.8881392	0.32949825	0	1.91472903	5.60876929	-1.3981029	2.55638503	-1.1071539	-6.9077553	21	82	2.68223333	299.666667	26.66666666	0	0	1	0	42.288408	2	-0.4111751
6.8184	274210.953	0.23665173	13.9498868	0.36066261	0	1.91962484	5.61390136	-1.436949	2.63554308	-1.0170435	-6.9077553	22	85	1.78815556	298.555556	25.55555555	0	0	1	0	42.288408	2	-0.4111751
6.8499	275395.266	0.2083201	10.2886727	0.31951144	0	1.92423405	5.61821103	-1.5638906	2.33114075	-1.1378373	-6.9077553	23	89	2.23519444	298.555556	25.55555555	0	0	1	0	42.288408	2	-0.4111751
0.0009	0	0	0	0	0	-7.0131158	-6.9077553	-6.9077553	-6.9077553	-6.9077553	-6.9077553	0	88	4.02335	299.111111	26.11111111	0	0	0	0	18.9135952	2	-0.0428739
0.0009	0	0	0	0	0	-7.0131158	-6.9077553	-6.9077553	-6.9077553	-6.9077553	-6.9077553	1	89	3.57631111	298.555556	25.55555555	0	0	0	0	18.9135952	2	-0.0428739
0.0009	0	0	0	0	0	-7.0131158	-6.9077553	-6.9077553	-6.9077553	-6.9077553	-6.9077553	2	90	4.02335	298.555556	25.55555555	0	0	0	0	18.9135952	2	-0.0428739
0.0009	0	0	0	0	0	-7.0131158	-6.9077553	-6.9077553	-6.9077553	-6.9077553	-6.9077553	3	89	3.57631111	298.555556	25.55555555	0	0	0	0	18.9135952	2	-0.0428739
0.0009	0	0	0	0	0	-7.0131158	-6.9077553	-6.9077553	-6.9077553	-6.9077553	-6.9077553	4	84	4.02335	299.666667	26.66666666	0	0	0	0	18.9135952	2	-0.0428739
0.0288	0	0.49159487	66.2979067	2.15039087	0	-3.5473799	-6.9077553	-0.7080682	4.19417341	0.76611455	-6.9077553	5	76	4.91742778	301.333333	28.33333333	0	0	0	0	18.9135952	2	-0.0428739
0.1845	2566.55765	0.75990569	183.906431	2.68775331	0	-1.6901058	0.94295512	-0.2732459	5.21443254	0.98907763	-6.9077553	6	73	5.36446667	301.888889	28.88888888	0	0	0	0	18.9135952	2	-0.0428739
0.4491	10980.7272	0.89732164	266.583865	3.07666099	0	-0.8005097	2.39623273	-0.1072271	5.58569263	1.12416989	-6.9077553	7	63	6.25854444	304.111111	31.11111111	0	0	1	0	18.9135952	2	-0.0428739
0.612	14634.6864	0.73207193	188.729411	1.7132452	0	-0.491023	2.68346282	-0.3105114	5.2403196	0.53897287	-6.9077553	8	58	5.81150556	305.222222	32.2222222	0	0	1	0	18.9135952	2	-0.0428739
0.7938	19114.4115	0.70059113	148.183478	1.60689069	0	-0.2309237	2.95049489	-0.3544045	4.99845797	0.47492319	-6.9077553	9	55	4.47038889	306.333333	33.33333333	0	0	1	0	18.9135952	2	-0.0428739
1.0341	26969.6243	0.80602467	197.491119	1.80892419	0	0.03353148	3.29474829	-0.214401	5.28569868	0.59328496	-6.9077553	10	51	5.36446667	306.888889	33.88888888	0	0	1	0	18.9135952	2	-0.0428739
1.3464	36731.5945	0.84391112	210.715733	2.1960692	0	0.29743436	3.60366449	-0.1685238	5.35051473	0.78712429	-6.9077553	11	52	4.47038889	307.444444	34.4444444	0	0	1	0	18.9135952	2	-0.0428739
1.629	46373.4121	0.82746955	201.089471	1.69472314	0	0.48796633	3.83674784	-0.1881752	5.30375491	0.52810928	-6.9077553	12	50	4.02335	308	35	0	0	1	0	18.9135952	2	-0.0428739
1.9026	55874.3314	0.79589342	187.404207	1.5336383	0	0.64322137	4.02312298	-0.2270343	5.23327315	0.42829472	-6.9077553	13	51	3.57631111	308	35	0	0	1	0	18.9135952	2	-0.0428739
2.2194	66842.446	0.79165762	186.662287	1.61463165	0	0.79723689	4.20235326	-0.2323639	5.22930639	0.479726	-6.9077553	14	51	4.02335	307.444444	34.4444444	0	0	1	0	18.9135952	2	-0.0428739
2.5794	81117.0689	0.84372986	213.994023	1.73095154	0	0.94755681	4.39590573	-0.1687384	5.36595276	0.54924883	-6.9077553	15	54	2.68223333	306.888889	33.88888888	0	0	1	0	18.9135952	2	-0.0428739
2.8332	92349.5036	0.83079101	191.44595	1.37587581	0	1.04140681	4.52559116	-0.1841741	5.25461075	0.31981703	-6.9077553	16	61	0.89407778	304.666667	31.66666666	0	0	1	0	18.9135952	2	-0.0428739
2.9952	99854.4066	0.8316252	188.412174	1.49601203	0	1.09701101	4.60372321	-0.1831717	5.23863729	0.40347114	-6.9077553	17	72	2.68223333	303	30	0	0	1	0	18.9135952	2	-0.0428739
3.0879	103978.877	0.57368684	86.6915011	1.03402732	0	1.12749125	4.64419739	-0.55393	4.46236739	0.03442782	-6.9077553	18	74	3.57631111	301.888889	28.88888888	0	0	1	0	18.9135952	2	-0.0428739
3.1383	106259.793	0.39867875	38.7505668	0.68194784	0	1.14368125	4.66589638	-0.9170942	3.65717119	-0.3813368	-6.9077553	19	78	2.68223333	300.777778	27.7777778	0	0	1	0	18.9135952	2	-0.0428739

3.1743	107838.894	0.26416745	16.4426743	0.4549509	0	1.15508714	4.68064767	-1.3273938	2.79994086	-0.7853701	-6.9077553	20	80	2.68223333	300.222222	27.2222222	0	0	1	0	18.9135952	2	-0.0428739
3.2139	109461.851	0.24238166	13.0745503	0.40212991	0	1.16748515	4.69558523	-1.4131245	2.5707441	-0.9084964	-6.9077553	21	82	2.68223333	299.666667	26.66666666	0	0	1	0	18.9135952	2	-0.0428739
3.2535	111260.265	0.25329386	13.9845387	0.43126319	0	1.17973134	4.71188117	-1.3692647	2.63802384	-0.8387206	-6.9077553	22	85	1.78815556	298.555556	25.55555555	0	0	1	0	18.9135952	2	-0.0428739
3.294	113190.267	0.25577441	14.1593275	0.41312591	0	1.19210263	4.72907902	-1.3595573	2.65044422	-0.8815852	-6.9077553	23	89	2.23519444	298.555556	25.55555555	0	0	1	0	18.9135952	2	-0.0428739
0.0117	406.917123	0.91128778	225.359843	1.60320425	0	-4.4481664	-0.8966913	-0.0917998	5.41770286	0.47262784	-6.9077553	0	94	1.34111667	276.888889	3.888888888	0	0	0	0	30.519848	2	0.21141735
0.0324	1210.84876	0.69557808	140.277131	1.21364851	0	-3.4295969	0.19214709	-0.3615754	4.94362711	0.19445474	-6.9077553	1	58	1.78815556	284.666667	11.66666666	0	0	0	0	30.519848	2	0.21141735
0.09	1801.47855	0.58352029	108.717964	1.38947916	0	-2.4079456	0.58916269	-0.5369638	4.68876624	0.32964841	-6.9077553	2	36	3.12927222	290.222222	17.2222222	0	0	0	0	30.519848	2	0.21141735
0.18	2840.55891	0.51894291	82.077959	1.51663666	0	-1.7147984	1.04435281	-0.6540363	4.4076817	0.41715429	-6.9077553	3	31	3.57631111	293.555556	20.55555555	0	0	0	0	30.519848	2	0.21141735
0.3987	4169.56715	0.73887911	159.033195	3.58968953	0	-0.919546	1.42805203	-0.3012685	5.06911924	1.27834425	-6.9077553	4	22	5.36446667	296.333333	23.33333333	0	0	0	1	30.519848	2	0.21141735
0.7056	6632.49885	0.77410137	168.499232	3.2564078	0	-0.3487068	1.89213239	-0.2547615	5.12693713	1.18093172	-6.9077553	5	20	5.81150556	297.444444	24.44444444	0	0	0	1	30.519848	2	0.21141735
1.0557	17222.0584	0.8052114	187.226382	2.42946078	0	0.05420405	2.84624909	-0.2154093	5.23232382	0.88808086	-6.9077553	6	18	4.91742778	298.555556	25.55555555	0	0	0	1	30.519848	2	0.21141735
1.4841	36470.3855	0.87520805	223.236262	2.77741248	0	0.39480853	3.59652799	-0.1321517	5.40823516	1.02187971	-6.9077553	7	16	5.36446667	299.111111	26.11111111	0	0	0	1	30.519848	2	0.21141735
2.0259	57112.5714	0.92914406	278.703047	2.96027159	0	0.70601405	4.04504177	-0.0724158	5.63015046	1.08561877	-6.9077553	8	18	5.36446667	298.555556	25.55555555	0	0	0	1	30.519848	2	0.21141735
2.3796	83061.3378	0.77364048	166.005497	1.52464236	0	0.86693241	4.41959138	-0.2553562	5.11202692	0.42241554	-6.9077553	9	25	2.68223333	295.222222	22.2222222	0	0	0	1	30.519848	2	0.21141735
2.673	107673.645	0.790336	170.871633	1.38398097	0	0.98320144	4.67911413	-0.2340326	5.14091844	0.3256864	-6.9077553	10	29	1.34111667	293	20	0	0	0	1	30.519848	2	0.21141735
2.9286	136170.646	0.75548414	157.507152	1.12247209	0	1.07452449	4.91391619	-0.2790737	5.05947722	0.11642397	-6.9077553	11	31	1.34111667	290.777778	17.7777778	0	0	0	1	30.519848	2	0.21141735
3.1743	166516.72	0.77625356	165.130008	1.49384115	0	1.15508714	5.11510173	-0.2519887	5.10673915	0.40201995	-6.9077553	12	33	2.68223333	290.222222	17.2222222	0	0	0	1	30.519848	2	0.21141735
3.4083	197316.718	0.74576187	157.312184	1.26427659	0	1.22621363	5.28481521	-0.2920089	5.05823862	0.23529075	-6.9077553	13	35	3.12927222	290.222222	17.2222222	0	0	0	1	30.519848	2	0.21141735
3.5883	227260.716	0.68498032	129.809453	1.14492538	0	1.27767855	5.42610229	-0.3769063	4.86607533	0.13621251	-6.9077553	14	36	3.57631111	290.222222	17.2222222	0	0	0	1	30.519848	2	0.21141735
3.7791	261191.702	0.69625711	130.803868	1.28749128	0	1.32948589	5.56525846	-0.3606011	4.87370665	0.25347198	-6.9077553	15	40	3.57631111	289.111111	16.11111111	0	0	0	1	30.519848	2	0.21141735
3.9339	296809.349	0.65787409	114.679608	0.81019805	0	1.3696313	5.69309338	-0.4172228	4.74215094	-0.209243	-6.9077553	16	44	3.57631111	288	15	0	0	0	1	30.519848	2	0.21141735
4.0491	330536.697	0.62264234	104.23349	0.539083	0	1.39849463	5.80072071	-0.4721783	4.64664307	-0.6160324	-6.9077553	17	44	4.91742778	286.888889	13.88888888	0	0	0	1	30.519848	2	0.21141735
4.1553	367253.012	0.63962161	106.256712	0.52604969	0	1.42438463	5.90605374	-0.4453163	4.66586739	-0.6404604	-6.9077553	18	51	4.02335	285.777778	12.77777777	0	0	0	1	30.519848	2	0.21141735
4.2534	402634.17	0.64496361	104.00491	0.47028085	0	1.44771866	5.99803087	-0.4370121	4.64444772	-0.7523011	-6.9077553	19	63	2.23519444	284.666667	11.66666666	0	0	0	1	30.519848	2	0.21141735
4.3344	431673.442	0.6078437	91.7144531	0.44406132	0	1.46658319	6.0676717	-0.4961937	4.51869088	-0.8095432	-6.9077553	20	71	2.23519444	284.666667	11.66666666	0	0	0	1	30.519848	2	0.21141735
4.4046	457041.06	0.58260771	83.3938489	0.42967997	0	1.48264945	6.12477542	-0.5385263	4.42358654	-0.84239	-6.9077553	21	73	2.68223333	284.666667	11.66666666	0	0	0	1	30.519848	2	0.21141735
4.4667	480072.207	0.51754158	65.9728049	0.36703563	0	1.49664988	6.17393861	-0.6567351	4.18925777	-0.9995755	-6.9077553	22	79	3.57631111	284.666667	11.66666666	0	0	0	1	30.519848	2	0.21141735
4.4982	491420.906	0.42434921	41.7871198	0.28049464	0	1.50367732	6.19730304	-0.8548448	3.73261208	-1.2676419	-6.9077553	23	83	4.47038889	286.333333	13.33333333	0	0	0	1	30.519848	2	0.21141735
0.0144	654.066737	0.9609883	256.129736	1.75340538	0	-4.2405271	-0.4230182	-0.038753	5.545688	0.56212998	-6.9077553	0	94	1.34111667	276.888889	3.888888888	0	0	0	0	34.0724638	2	0.49277013
0.0252	1048.84083	0.54294872	76.8682098	0.96573655	0	-3.6809113	0.04863856	-0.6089003	4.3421054	-0.0338293	-6.9077553	1	58	1.78815556	284.666667	11.66666666	0	0	0	0	34.0724638	2	0.49277013
0.0927	3928.55596	0.92007579	240.097552	2.15582784	0	-2.3783868	1.36852643	-0.082213	5.48104947	0.76863855	-6.9077553	2	36	3.12927222	290.222222	17.2222222	0	0	0	0	34.0724638	2	0.49277013
0.1809	7922.66076	0.98100935	255.461874	1.82179665	0	-1.7098109	2.06985332	-0.0181545	5.54307709	0.60037194	-6.9077553	3	31	3.57631111	293.555556	20.55555555	0	0	0	0	34.0724638	2	0.49277013
0.2979	12377.3363	0.9818252	256.105138	2.26897348	0	-1.2109974	2.51594787	-0.017324	5.54559196	0.81976815	-6.9077553	4	22	5.36446667	296.333333	23.33333333	0	0	0	1	34.0724638	2	0.49277013
0.5985	31964.4812	1.47375287	635.925898	5.25893781	0	-0.5133288	3.46465661	0.38849043	6.45508362	1.6601192	-6.9077553	5	20	5.81150556	297.444444	24.44444444	0	0	0	1	34.0724638	2	0.49277013
0.9927	55171.9385	1.35612369	542.823951	3.89931819	0	-0.0073268	4.01047259	0.30536753	6.29678689	1.36105814	-6.9077553	6	18	4.91742778	298.555556	25.55555555	0	0	0	1	34.0724638	2	0.49277013
1.6245	72856.6091	1.32124889	531.25276	4.29415259	0	0.48520008	4.28850698	0.27933399	6.2752398	1.45748708	-6.9077553	7	16	5.36446667	299.111111	26.11111111	0	0	0	1	34.0724638	2	0.49277013
2.3976	101223.724	1.2231073	440.855573	3.39030895	0	0.87446824	4.61734304	0.20221184	6.08871959	1.22121597	-6.9077553	8	18	5.36446667	298.555556	25.55555555	0	0	0	1	34.0724638	2	0.49277013
2.988	123953.633	1.00838945	288.843559	2.21982333	0	1.09460427	4.81991564	0.00934564	5.66588869	0.79787799	-6.9077553	9	25	2.68223333	295.222222	22.2222222	0	0	0	1	34.0724638	2	0.49277013
3.5199	140835.278	0.94430867	253.382535	1.94346471	0	1.25843258	4.94759807	-0.0562438	5.53490429	0.66498673	-6.9077553	10	29	1.34111667	293	20	0	0	0	1	34.0724638	2	0.49277013
3.9582	155251.64	0.87166431	212.368423	1.6143661	0	1.37578938	5.04505372	-0.1362043	5.35832732	0.47956162	-6.9077553	11	31	1.34111667	290.777778	17.7777778	0	0	0	1	34.0724638	2	0.49277013
4.4757	167505.233	0.88945493	223.082663	2.13413383	0	1.49866276	5.12102056	-0.1160228	5.40754687	0.75852933	-6.9077553	12	33	2.68223333	290.222222	17.2222222	0	0	0	1	34.0724638	2	0.49277013
4.959	178953.626	0.82365	193.51916	1.81693818	0	1.60120411	5.18713229	-0.1927962	5.26538169	0.59770299	-6.9077553	13	35	3.12927222	290.222222	17.2222222	0	0	0	1	34.0724638	2	0.49277013
5.4468	192437.459	0.78768076	173.400513	1.57341809	0	1.69502828	5.25977641	-0.2373936	5.15560979	0.45388573	-6.9077553	14	36	3.57631111	290.222222	17.2222222	0	0	0	1	34.0724638	2	0.49277013
5.9679	208764.069	0.79300275	172.677232	1.67532765	0	1.78639511	5.34120955	-0.2306684	5.15142993	0.51660548	-6.9077553	15	40	3.57631111	289.111111	16.11111111	0	0	0	1	34.0724638	2	0.49277013
6.471	224049.397	0.76556233	160.697423	1.56502952	0	1.86733066	5.41187101	-0.2658393	5.07952946	0.44854345	-6.9077553	16	44	3.57631111	288	15	0	0	0	1	34.0724638	2	0.49277013
7.0533	241295.455	0.81390654	192.403485	1.92715968	0	1.95349559	5.48602628	-0.2046818	5.25959985	0.65656602	-6.9077553	17	44	4.91742778	286.888889	13.88888888	0	0	0	1	34.0724638	2	0.49277013
7.5726	258082.274	0.75497928	167.59746	1.66301327	0	2.02453647	5.5532823	-0.2797413	5.121571	0.50923232	-6.9077553	18	51	4.02335	285.777778	12.77777777	0	0	0	1	34.0724638	2	0.49277013
7.9578	274707.363	0.86509134	210.753778	1.57657374	0	2.07415258	5.61571004	-0.1437649	5.35069527	0.45588806	-6.9077553	19	63	2.23519444	284.666667	11.66666666	0	0	0	1	34.0724638	2	0.49277013
8.2296	285846.72	0.83299164	184.573533	1.40332638	0	2.10773741	5.65545922	-0.1815319	5.21805336	0.33955774	-6.9077553	20	71	2.23519444	284.666667	11.66666666	0	0	0	1	34.0724638	2	0.49277013
8.4339	294390.5	0.78415576	159.895873	1.25032964	0	2.1322593	5.68491051	-0.2418732	5.07452907	0.2242067	-6.9077553	21	73	2.68223333	284.666667	11.66666666	0	0	0	1	34.0724638	2	0.49277013
8.5959	301160.41	0.70407761	129.923242	1.15646278	0	2.15128535	5.70764637	-0.3494474	4.86695153	0.14623035	-6.9077553	22	79	3.57631111	284.666667	11.66666666	0	0	0	1	34.0724638	2	0.49277013
8.6895	305284.638	0.58809214	87.8427453	0.94042521	0	2.1621154	5.72124786	-0.5291727	4.47555961	-0.0603604	-6.9077553	23	83	4.47038889	286.333333	13.33333333	0	0	0	1	34.0724638	2	0.49277013
0.0306	1810.47822	1.06080408	307.53563	2.05283217	0	-3.4867553	0.59414321	0.05996942	5.7285942	0.7197074	-6.9077553	0	94	1.34111667	276.888889	3.888888888	0	0	0	0	42.288408	2	-0.4111751
0.1107	6079.24105	1.00088218	274.175233	2.12757308	0	-2.2009314	1.80504434	0.00188041	5.61377108	0.75545184	-6.9077553	1	58	1.78815556	284.666667	11.66666666	0	0	0	0	42.288408	2	-0.4111751
0.3537	16880.4344	1.06877249	320.071904	3.25073559	0	-1.0393062	2.82621446	0.067446	5.76854879	1.17918888	-6.9077553	2	36	3.12927222	290.222222	17.2222222	0	0	0	0	42.288408	2	-0.4111751
0.6543	32037.6092	1.06295895	307.383187	2.26116285	0	-0.4241893	3.46694171	0.06199681	5.72809839	0.81632137	-6.9077553	3	31	3.57631111	293.555556	20.55555555	0	0	0	0	42.288408	2	-0.4111751
1.0206	46781.1941	0.95963852	249.336725	2.2185492	0	0.02039069	3.84550266	-0.0401571	5.5188083	0.79730411	-6.9077553	4	22	5.36446667	296.333333	23.33333333	0	0	0	0	42.288408	2	-0.4111751
1.5543	68663.0049	1.0498438	315.369677	3.01265167	0	0.44102528	4.22922512	0.04959346	5.7537487	1.10315252	-6.9077553	5	20	5.81150556	297.444444	24.44444444	0	0	1	0	42.288408	2	-0.4111751
2.3058	103793.891	1.09736423	345.997904	2.95135637	0	0.83542769	4.64241675	0.09382201	5.84643561	1.08260362	-6.9077553	6	18	4.91742778	298.555556	25.55555555	0	0	1	0	42.288408	2	-0.4111751
3.0231	139064.776	1.10340803	356.474435	2.5873227	0	1.10628279	4.93494703	0.09930947	5.87626533	0.95101006	-6.9077553	7	16	5.36446667	299.111111	26.11111111	0	0	1	0	42.288408	2	-0.4111751
3.8403	176345.206	1.11719005	381.393654	3.09188802	1	1.34555049	5.17244914	0.11171136	5.94383468	1.12910529	0.0009995	8	18	5.36446667	298.555556	25.55555555	0	0	1	0	42.288408	2	-0.4111751
4.4505	204015.79	0.9684938	274.355135	2.01953449	1	1.49301645	5.3182023	-0.0309812	5.61442702	0.70336208	0.0009995	9	25	2.68223333	295.222222	22.2222222	0	0	1	0	42.288408	2	-0.4111751

5.0238	228393.708	0.91002239	245.280759	1.9270353	1	1.61418662	5.43107531	-0.0931878	5.50240759	0.6565015	0.0009995	10	29	1.34111667	293	20	0	0	1	0	42.288408	2	-0.4111751
5.616	249734.624	0.86493161	221.050294	1.7902205	1	1.72561967	5.52040285	-0.1439493	5.39839478	0.58289723	0.0009995	11	31	1.34111667	290.777778	17.7777778	0	0	1	0	42.288408	2	-0.4111751
6.0669	263480.43	0.73345594	160.732878	1.40777454	1	1.80284777	5.57398289	-0.3086253	5.07975007	0.34272021	0.0009995	12	33	2.68223333	290.222222	17.2222222	0	0	1	0	42.288408	2	-0.4111751
6.4494	274440.154	0.71006786	154.784338	1.29574563	1	1.8639871	5.61473686	-0.3409874	5.04203924	0.25985777	0.0009995	13	35	3.12927222	290.222222	17.2222222	0	0	1	0	42.288408	2	-0.4111751
6.8454	284980.542	0.66175759	141.705007	1.3370661	1	1.92357689	5.65242441	-0.411346	4.95375454	0.29122536	0.0009995	14	36	3.57631111	290.222222	17.2222222	0	0	1	0	42.288408	2	-0.4111751
7.2396	295636.61	0.67181264	144.541676	1.29377572	1	1.97956596	5.68913441	-0.3962884	4.9735748	0.25833749	0.0009995	15	40	3.57631111	289.111111	16.11111111	0	0	1	0	42.288408	2	-0.4111751
7.6005	303570.148	0.65474779	135.789998	1.25547406	1	2.02821403	5.71561601	-0.421979	4.91111692	0.22830943	0.0009995	16	44	3.57631111	288	15	0	0	1	0	42.288408	2	-0.4111751
8.0055	311504.087	0.665008	150.186215	1.57267361	1	2.08012881	5.74141567	-0.4064536	5.01188262	0.45341277	0.0009995	17	44	4.91742778	286.888889	13.88888888	0	0	1	0	42.288408	2	-0.4111751
8.4051	321164.666	0.71551445	174.415593	1.56003467	1	2.12883866	5.77195708	-0.3333569	5.16144665	0.44534885	0.0009995	18	51	4.02335	285.777778	12.77777777	0	0	1	0	42.288408	2	-0.4111751
8.5671	325815.57	0.70030772	151.031565	1.15034604	1	2.14792929	5.78633456	-0.3548085	5.01749547	0.14093172	0.0009995	19	63	2.23519444	284.666667	11.66666666	0	0	1	0	42.288408	2	-0.4111751
8.7048	331389.092	0.82696493	189.117085	1.43897601	1	2.1638746	5.80329621	-0.1887845	5.24237161	0.36462646	0.0009995	20	71	2.23519444	284.666667	11.66666666	0	0	1	0	42.288408	2	-0.4111751
8.829	336340.107	0.81745385	179.469939	1.47694242	1	2.17804176	5.81812584	-0.2003383	5.19001329	0.39065086	0.0009995	21	73	2.68223333	284.666667	11.66666666	0	0	1	0	42.288408	2	-0.4111751
8.9613	341098.355	0.85584638	209.940888	1.8861823	1	2.19291531	5.8321738	-0.1544966	5.34683077	0.63508487	0.0009995	22	79	3.57631111	284.666667	11.66666666	0	0	1	0	42.288408	2	-0.4111751
9.054	344419.433	0.74166907	162.751214	1.74918417	1	2.20320665	5.8418631	-0.2975047	5.09222888	0.55972102	0.0009995	23	83	4.47038889	286.333333	13.33333333	0	0	1	0	42.288408	2	-0.4111751
0.0018	0	0.16748893	5.34041262	0.42339128	0	-6.3199686	-6.9077553	-1.7808852	1.67549015	-0.8570994	-6.9077553	0	94	1.34111667	276.888889	3.888888888	0	0	0	0	18.9135952	2	-0.0428739
0.0018	0	0	0	0	0	-6.3199686	-6.9077553	-6.9077553	-6.9077553	-6.9077553	-6.9077553	1	58	1.78815556	284.666667	11.66666666	0	0	0	0	18.9135952	2	-0.0428739
0.0252	0	0.4354755	51.5123385	1.57020672	0	-3.6809113	-6.9077553	-0.829023	3.94184077	0.45184393	-6.9077553	2	36	3.12927222	290.222222	17.2222222	0	0	0	0	18.9135952	2	-0.0428739
0.1584	91.4139551	0.64501948	113.403663	2.89297362	0	-1.8426318	-2.3814773	-0.4369256	4.73096251	1.06263051	-6.9077553	3	31	3.57631111	293.555556	20.55555555	0	0	1	0	18.9135952	2	-0.0428739
0.3834	4106.7895	0.77840552	217.534672	3.06093945	0	-0.9586764	1.41288505	-0.2492238	5.38236285	1.11904852	-6.9077553	4	22	5.36446667	296.333333	23.33333333	0	0	1	0	18.9135952	2	-0.0428739
0.6264	14082.1719	0.98205962	344.277025	2.71125398	0	-0.4677661	2.6449806	-0.0170855	5.84144954	0.99778002	-6.9077553	5	20	5.81150556	297.444444	24.44444444	0	0	1	0	18.9135952	2	-0.0428739
0.8334	18840.6728	0.80051628	200.598947	1.82503131	0	-0.1822416	2.93607105	-0.22125	5.30131261	0.60214493	-6.9077553	6	18	4.91742778	298.555556	25.55555555	0	0	1	0	18.9135952	2	-0.0428739
1.1376	29739.02	0.96321494	293.709227	2.54165162	0	0.12892078	3.39249361	-0.036441	5.68259366	0.93320748	-6.9077553	7	16	5.36446667	299.111111	26.11111111	0	0	1	0	18.9135952	2	-0.0428739
1.5291	43739.1479	0.97126311	285.720097	2.53287424	0	0.42467933	3.7782664	-0.0281288	5.65501615	0.92974945	-6.9077553	8	18	5.36446667	298.555556	25.55555555	0	0	1	0	18.9135952	2	-0.0428739
1.8621	56435.8118	0.88884211	241.594827	1.79057023	0	0.62170488	4.03312164	-0.1167112	5.48726619	0.58309246	-6.9077553	9	25	2.68223333	295.222222	22.2222222	0	0	1	0	18.9135952	2	-0.0428739
2.1897	68380.4468	0.80646959	196.032066	1.57197588	0	0.78376455	4.22510154	-0.2138499	5.27828335	0.45296929	-6.9077553	10	29	1.34111667	293	20	0	0	1	0	18.9135952	2	-0.0428739
2.6316	85166.5833	0.87687574	233.027686	1.96918035	0	0.96759203	4.44462088	-0.1302502	5.45116156	0.67812509	-6.9077553	11	31	1.34111667	290.777778	17.7777778	0	0	1	0	18.9135952	2	-0.0428739
3.06	102593.942	0.85836645	224.018399	1.72463682	0	1.11841492	4.63078863	-0.1515599	5.41173265	0.54559615	-6.9077553	12	33	2.68223333	290.222222	17.2222222	0	0	1	0	18.9135952	2	-0.0428739
3.4974	116000.517	0.78470966	183.263873	1.85085369	0	1.25201984	4.75360327	-0.2411679	5.2109325	0.61618713	-6.9077553	13	35	3.12927222	290.222222	17.2222222	0	0	1	0	18.9135952	2	-0.0428739
3.96	130994.96	0.81696501	199.04417	1.79303359	0	1.37624403	4.87516648	-0.2009357	5.29353178	0.58446648	-6.9077553	14	36	3.57631111	290.222222	17.2222222	0	0	1	0	18.9135952	2	-0.0428739
4.4127	147652.319	0.798546	188.088252	1.68204275	0	1.48448675	4.99486709	-0.2237112	5.23691659	0.52060332	-6.9077553	15	40	3.57631111	289.111111	16.11111111	0	0	1	0	18.9135952	2	-0.0428739
4.8942	166362.211	0.82908126	207.736551	1.84387709	0	1.58805083	5.11417342	-0.1862317	5.33627551	0.61241266	-6.9077553	16	44	3.57631111	288	15	0	0	1	0	18.9135952	2	-0.0428739
5.3352	180304.624	0.73713254	160.838552	1.65020204	0	1.67432637	5.19465332	-0.3036319	5.0804073	0.50150353	-6.9077553	17	44	4.91742778	286.888889	13.88888888	0	0	1	0	18.9135952	2	-0.0428739
5.697	192307.944	0.70305653	141.727748	1.19867999	0	1.73993972	5.25910316	-0.3508966	4.95391501	0.18205484	-6.9077553	18	51	4.02335	285.777778	12.77777777	0	0	1	0	18.9135952	2	-0.0428739
5.9832	205277.636	0.83681541	189.779801	1.32309459	0	1.78895554	5.32436826	-0.1769575	5.24586972	0.2807289	-6.9077553	19	63	2.23519444	284.666667	11.66666666	0	0	1	0	18.9135952	2	-0.0428739
6.3108	221679.582	0.92922102	234.412775	1.7006737	0	1.84226245	5.40123753	-0.0723331	5.45708782	0.5316123	-6.9077553	20	71	2.23519444	284.666667	11.66666666	0	0	1	0	18.9135952	2	-0.0428739
6.6357	237596.248	0.89974342	218.586911	1.6731684	0	1.89246416	5.47057701	-0.1045348	5.38718827	0.51531657	-6.9077553	21	73	2.68223333	284.666667	11.66666666	0	0	1	0	18.9135952	2	-0.0428739
6.9228	251526.735	0.8064535	175.618865	1.45134178	0	1.93482031	5.52755326	-0.2138698	5.1683218	0.37317727	-6.9077553	22	79	3.57631111	284.666667	11.66666666	0	0	1	0	18.9135952	2	-0.0428739
7.1253	260588.201	0.68015713	123.211163	1.20392551	0	1.96365183	5.56294522	-0.3839623	4.81390777	0.18641775	-6.9077553	23	83	4.47038889	286.333333	13.33333333	0	0	1	0	18.9135952	2	-0.0428739
0.0081	320.235626	0.84124577	190.780884	1.67513001	0	-4.8158912	-1.1355804	-0.1716834	5.25113081	0.51648757	-6.9077553	0	93	4.47038889	293.555556	20.55555555	0	0	0	0	30.519848	2	0.21141735
0.0108	451.826951	0.45875732	54.781133	0.70019317	0	-4.5282091	-0.7922452	-0.7770565	4.0033641	-0.3549719	-6.9077553	1	92	2.68223333	293.555556	20.55555555	0	0	0	0	30.519848	2	0.21141735
0.0216	934.38532	0.5501289	81.7196554	1.04336405	0	-3.835062	-0.0667967	-0.5957866	4.40330679	0.04340813	-6.9077553	2	91	2.68223333	294.111111	21.11111111	0	0	0	0	30.519848	2	0.21141735
0.0486	1373.023	0.45474742	63.8225708	1.05194155	0	-3.0241317	0.31774293	-0.7858165	4.15612257	0.05158773	-6.9077553	3	91	2.68223333	294.666667	21.66666666	0	0	0	0	30.519848	2	0.21141735
0.0873	1855.52464	0.36734142	38.8772171	1.0737805	0	-2.4384048	0.61870627	-0.998745	3.66043412	0.07211646	-6.9077553	4	85	3.57631111	296.333333	23.33333333	0	0	0	0	30.519848	2	0.21141735
0.1917	2759.7015	0.49165545	66.9835794	1.91592157	0	-1.6518236	1.01548481	-0.7079452	4.20446244	0.65072055	-6.9077553	5	71	5.36446667	298.555556	25.55555555	0	0	0	0	30.519848	2	0.21141735
0.3015	3542.06454	0.54434254	79.9558226	1.8234343	0	-1.1989853	1.26499204	-0.6063412	4.38148677	0.60126997	-6.9077553	6	67	5.81150556	299.666667	26.66666666	0	0	0	1	30.519848	2	0.21141735
0.4887	6994.35368	0.74912049	159.233511	2.57842665	0	-0.7160065	1.94524617	-0.2875214	5.07037803	0.94756715	-6.9077553	7	55	6.25854444	301.888889	28.88888888	0	0	0	1	30.519848	2	0.21141735
0.7443	15782.14	0.86498481	218.396842	2.79045126	0	-0.2953111	2.75894228	-0.1438879	5.38631836	1.02656163	-6.9077553	8	50	5.81150556	303.555556	30.55555555	0	0	0	1	30.519848	2	0.21141735
1.0458	25105.1119	0.73471142	147.877659	2.21216586	0	0.04478214	3.22311132	-0.3069173	4.99639206	0.794424	-6.9077553	9	45	5.36446667	304.111111	31.11111111	0	0	0	1	30.519848	2	0.21141735
1.3302	36074.061	0.69505064	133.689206	1.57660897	0	0.28532931	3.58560179	-0.3623329	4.89552523	0.45591039	-6.9077553	10	46	3.57631111	304.111111	31.11111111	0	0	0	1	30.519848	2	0.21141735
1.539	46128.2199	0.71333773	150.603482	1.31254367	0	0.43113285	3.83144659	-0.3363994	5.01465708	0.27272858	-6.9077553	11	44	4.02335	304.666667	31.66666666	0	0	0	1	30.519848	2	0.21141735
1.7181	55180.8569	0.68207278	135.726199	1.20049504	0	0.54121903	4.01063422	-0.3811539	4.91064698	0.18356665	-6.9077553	12	49	3.12927222	303	30	0	0	0	1	30.519848	2	0.21141735
1.8531	62686.6427	0.60404012	108.59166	0.93424774	0	0.61685991	4.13816434	-0.5024605	4.68760382	-0.0669438	-6.9077553	13	53	3.12927222	301.333333	28.33333333	0	0	0	1	30.519848	2	0.21141735
1.9476	71730.3681	0.67815247	129.946456	0.88084077	0	0.66659785	4.27292814	-0.3869096	4.86713019	-0.1257438	-6.9077553	14	58	1.78815556	299.111111	26.11111111	0	0	0	1	30.519848	2	0.21141735
2.0448	80388.9947	0.68255071	137.258852	0.94159819	0	0.71529999	4.38688972	-0.3804544	4.92187586	-0.0591152	-6.9077553	15	51	2.68223333	298.555556	25.55555555	0	0	0	1	30.519848	2	0.21141735
2.1744	90773.5385	0.63860925	117.279595	0.91922866	0	0.77675276	4.50837883	-0.4468978	4.76456931	-0.0831331	-6.9077553	16	53	2.23519444	297.444444	24.44444444	0	0	0	1	30.519848	2	0.21141735
2.2743	101439.649	0.68317434	132.492566	0.88625776	0	0.82167231	4.61947389	-0.3795425	4.88653408	-0.1196197	-6.9077553	17	62	2.68223333	296.333333	23.33333333	0	0	0	1	30.519848	2	0.21141735
2.3328	109425.014	0.70507267	131.487545	0.9353758	0	0.84706926	4.69524865	-0.3480371	4.87891974	-0.0657384	-6.9077553	18	73	3.12927222	295.777778	22.7777778	0	0	0	1	30.519848	2	0.21141735
2.367	114144.539	0.57361437	85.4398258	0.81193005	0	0.86162333	4.73747429	-0.5540561	4.44782404	-0.2071102	-6.9077553	19	80	2.23519444	295.222222	22.2222222	0	0	0	1	30.519848	2	0.21141735
2.3733	114319.991	0.40443533	38.2689035	0.47710841	0	0.86428139	4.7390102	-0.9027939	3.64466378	-0.7379178	-6.9077553	20	84	3.12927222	295.222222	22.2222222	0	0	0	1	30.519848	2	0.21141735
2.3742	114363.854	0.19285333	7.25617123	0.19984631	0	0.86466054	4.73939381	-1.6406534	1.98199011	-1.6052153	-6.9077553	21	88	2.23519444	294.666667	21.66666666	0	0	0	1	30.519848	2	0.21141735
2.3769	114407.717	0.20579672	8.43368403	0.34841724	0	0.86579712	4.73977727	-1.576019	2.13235226	-1.0514885	-6.9077553	22	90	2.23519444	294.666667	21.66666666	0	0	0	1	30.519848	2	0.21141735
2.3805	114495.443	0.1803802	6.48140001	0.39878279	0	0.86731055	4.74054376	-1.7071599	1.86909081	-0.9168339	-6.9077553	23	89	3.12927222	295.222222	22.2222222	0	0	0	1	30.519848	2	0.21141735

0.0162	710.032604	1.0229049	290.487132	2.1455684	0	-4.122744	-0.341037	0.02362365	5.67156272	0.76387047	-6.9077553	0	93	4.47038889	293.555556	20.55555555	0	0	0	0	34.0724638	2	0.49277013
0.0315	1324.12559	0.44793564	50.2566923	0.85438807	0	-3.4577677	0.28150724	-0.8008757	3.91716362	-0.1562	-6.9077553	1	92	2.68223333	293.555556	20.55555555	0	0	0	0	34.0724638	2	0.49277013
0.0441	1675.03583	0.43735426	44.1620222	0.61574289	0	-3.1212955	0.51643138	-0.8247279	3.78788784	-0.4833031	-6.9077553	2	91	2.68223333	294.111111	21.11111111	0	0	0	0	34.0724638	2	0.49277013
0.0621	2332.99252	0.51134181	67.9884949	1.07494574	0	-2.7790093	0.84758033	-0.6687633	4.21935321	0.07320003	-6.9077553	3	91	2.68223333	294.666667	21.66666666	0	0	0	0	34.0724638	2	0.49277013
0.1197	4563.69177	0.69188428	131.945999	1.35764813	0	-2.1227667	1.51835099	-0.3668923	4.88240032	0.30649019	-6.9077553	4	85	3.57631111	296.333333	23.33333333	0	0	0	0	34.0724638	2	0.49277013
0.1791	7494.10607	0.88319131	207.332534	1.42033143	0	-1.719811	2.01425028	-0.1230818	5.33432877	0.35159406	-6.9077553	5	71	5.36446667	298.555556	25.55555555	0	0	0	0	34.0724638	2	0.49277013
0.2844	12088.1488	0.96973621	265.069878	2.33707552	0	-1.2573736	2.49230825	-0.0297005	5.57999726	0.84932816	-6.9077553	6	67	5.81150556	299.666667	26.66666666	0	0	0	1	34.0724638	2	0.49277013
0.5148	23326.5142	1.2764107	509.166199	4.24238014	0	-0.6639768	3.14963353	0.24483514	6.23277645	1.44536015	-6.9077553	7	55	6.25854444	301.888889	28.88888888	0	0	0	1	34.0724638	2	0.49277013
1.1421	44710.6596	1.35819983	546.318732	4.85853504	0	0.13286867	3.80023431	0.30689617	6.30320439	1.58094276	-6.9077553	8	50	5.81150556	303.555556	30.55555555	0	0	0	1	34.0724638	2	0.49277013
1.7649	64796.0012	1.20343018	423.89487	3.42987025	0	0.56809403	4.17125932	0.18600657	6.04948784	1.23281395	-6.9077553	9	45	5.36446667	304.111111	31.11111111	0	0	0	1	34.0724638	2	0.49277013
2.2284	78492.7676	0.95122663	252.04419	2.05455453	0	0.80128384	4.36301923	-0.0489522	5.5296084	0.72054565	-6.9077553	10	46	3.57631111	304.111111	31.11111111	0	0	0	1	34.0724638	2	0.49277013
2.7603	96231.5969	1.02892075	302.07601	2.47598767	0	1.01533937	4.56676815	0.02948186	5.71068198	0.90704317	-6.9077553	11	44	4.02335	304.666667	31.66666666	0	0	0	1	34.0724638	2	0.49277013
3.2913	114030.555	0.94029024	247.9433	2.12303115	0	1.19128262	4.73647521	-0.0605038	5.51320412	0.75331577	-6.9077553	12	49	3.12927222	303	30	0	0	0	1	34.0724638	2	0.49277013
3.7224	127941.956	0.83003754	198.331811	1.70639618	0	1.31436862	4.85158451	-0.1850803	5.28994648	0.53496951	-6.9077553	13	53	3.12927222	301.333333	28.33333333	0	0	0	1	34.0724638	2	0.49277013
4.0725	140192.138	0.7904183	183.157736	1.40723331	0	1.40425706	4.94302103	-0.2339286	5.21035319	0.34233595	-6.9077553	14	58	1.78815556	299.111111	26.11111111	0	0	0	1	34.0724638	2	0.49277013
4.3893	150177.506	0.7074223	159.014604	1.37069954	0	1.47916976	5.01182463	-0.3447149	5.06900234	0.31605051	-6.9077553	15	51	2.68223333	298.555556	25.55555555	0	0	0	1	34.0724638	2	0.49277013
4.7511	159437.088	0.67599335	144.278957	1.27889954	0	1.55837617	5.07165568	-0.3900938	4.97175556	0.24678159	-6.9077553	16	53	2.23519444	297.444444	24.44444444	0	0	0	1	34.0724638	2	0.49277013
4.9446	164295.707	0.70093476	141.356341	1.12892201	0	1.59829607	5.10167398	-0.3539148	4.95129102	0.12214861	-6.9077553	17	62	2.68223333	296.333333	23.33333333	0	0	0	1	34.0724638	2	0.49277013
5.0742	169253.872	0.72463444	144.263048	1.27480284	0	1.62416888	5.1314057	-0.3207089	4.97164529	0.24357566	-6.9077553	18	73	3.12927222	295.777778	22.7777778	0	0	0	1	34.0724638	2	0.49277013
5.1777	173377.596	0.60537196	96.2876953	1.03216208	0	1.64436094	5.15547762	-0.5002617	4.56735092	0.03262408	-6.9077553	19	80	2.23519444	295.222222	22.2222222	0	0	0	1	34.0724638	2	0.49277013
5.2119	174912.835	0.22356924	10.4770733	0.31858522	0	1.65094447	5.16429348	-1.4935712	2.34928481	-1.1407313	-6.9077553	20	84	3.12927222	295.222222	22.2222222	0	0	0	1	34.0724638	2	0.49277013
5.2524	176667.384	0.18881507	7.44637451	0.3508599	0	1.65868512	5.17427444	-1.661705	2.00786156	-1.0445222	-6.9077553	21	88	2.23519444	294.666667	21.66666666	0	0	0	1	34.0724638	2	0.49277013
5.2866	178027.176	0.19377984	8.0036862	0.31103478	0	1.66517532	5.18194183	-1.6358854	2.08002715	-1.1646406	-6.9077553	22	90	2.23519444	294.666667	21.66666666	0	0	0	1	34.0724638	2	0.49277013
5.328	179825.579	0.20407644	10.8028511	0.30526375	0	1.67297593	5.19199294	-1.5843725	2.37990266	-1.1833086	-6.9077553	23	89	3.12927222	295.222222	22.2222222	0	0	0	1	34.0724638	2	0.49277013
0.0369	2371.66749	1.12477331	351.333008	2.45807762	0	-3.2995437	0.86401485	0.11847019	5.86173736	0.89978633	-6.9077553	0	93	4.47038889	293.555556	20.55555555	0	0	0	0	42.288408	2	-0.4111751
0.0756	4257.87991	0.61189501	95.3807118	1.11591969	0	-2.582299	1.44900619	-0.4895616	4.55788686	0.11057462	-6.9077553	1	92	2.68223333	293.555556	20.55555555	0	0	0	0	42.288408	2	-0.4111751
0.1233	6538.79663	0.60449053	93.0241561	1.0685296	0	-2.0931349	1.87790607	-0.5017164	4.53286995	0.06721892	-6.9077553	2	91	2.68223333	294.111111	21.11111111	0	0	0	0	42.288408	2	-0.4111751
0.2601	11298.1008	0.76894765	166.467902	2.12766868	0	-1.3466891	2.42472315	-0.2614328	5.11480852	0.75549675	-6.9077553	3	91	2.68223333	294.666667	21.66666666	0	0	0	0	42.288408	2	-0.4111751
0.4662	20203.8182	0.91545938	232.791894	2.08988373	0	-0.7631406	3.0059211	-0.0872375	5.45014919	0.73758681	-6.9077553	4	85	3.57631111	296.333333	23.33333333	0	0	0	0	42.288408	2	-0.4111751
0.7569	32245.6759	0.90063363	223.158233	2.21554991	0	-0.2785241	3.47341496	-0.103547	5.40788557	0.79595189	-6.9077553	5	71	5.36446667	298.555556	25.55555555	0	0	0	0	42.288408	2	-0.4111751
1.1223	45671.6687	0.88092597	211.772437	2.26063936	0	0.11538015	3.82150006	-0.1256472	5.35551701	0.81608993	-6.9077553	6	67	5.81150556	299.666667	26.66666666	0	0	1	0	42.288408	2	-0.4111751
1.62	64173.4023	0.89278082	225.087958	2.4424742	0	0.48242615	4.16160441	-0.1122947	5.41649569	0.89342088	-6.9077553	7	55	6.25854444	301.888889	28.88888888	0	0	1	0	42.288408	2	-0.4111751
2.2158	85483.68	0.92019782	238.127171	2.62080775	0	0.79561351	4.44833718	-0.0820805	5.47280906	0.96386406	-6.9077553	8	50	5.81150556	303.555556	30.55555555	0	0	1	0	42.288408	2	-0.4111751
2.7855	111602.931	0.9441753	247.637391	1.90698821	0	1.02442739	4.71495627	-0.0563849	5.51196958	0.64604939	-6.9077553	9	45	5.36446667	304.111111	31.11111111	0	0	1	0	42.288408	2	-0.4111751
3.5145	147921.646	1.02015848	293.513002	2.20419289	0	1.25689727	4.99668947	0.02093775	5.68192534	0.79081498	-6.9077553	10	46	3.57631111	304.111111	31.11111111	0	0	1	0	42.288408	2	-0.4111751
4.2408	183759.635	1.02006945	296.035936	2.40762756	0	1.44475193	5.21363401	0.02085056	5.69048423	0.87905711	-6.9077553	11	44	4.02335	304.666667	31.66666666	0	0	1	0	42.288408	2	-0.4111751
4.8519	209067.96	0.92511292	245.286128	1.95056764	0	1.57937038	5.34266415	-0.0767591	5.50242948	0.66863297	-6.9077553	12	49	3.12927222	303	30	0	0	1	0	42.288408	2	-0.4111751
5.3037	227901.921	0.81418716	192.347361	1.46829746	0	1.66840469	5.42891975	-0.2043375	5.25930811	0.38478437	-6.9077553	13	53	3.12927222	301.333333	28.33333333	0	0	1	0	42.288408	2	-0.4111751
5.6394	242703.897	0.81977619	187.693071	1.32936968	0	1.72977768	5.49184629	-0.1975048	5.23481336	0.28545686	-6.9077553	14	58	1.78815556	299.111111	26.11111111	0	0	1	0	42.288408	2	-0.4111751
5.9841	255877.905	0.76824545	173.696904	1.29264262	0	1.78910595	5.5447043	-0.2623452	5.1573176	0.25746197	-6.9077553	15	51	2.68223333	298.555556	25.55555555	0	0	1	0	42.288408	2	-0.4111751
6.318	266437.77	0.69309632	148.062205	1.14413551	0	1.8434027	5.58514446	-0.3651445	4.99763924	0.13552298	-6.9077553	16	53	2.23519444	297.444444	24.44444444	0	0	1	0	42.288408	2	-0.4111751
6.5241	273906.468	0.76964932	166.512213	1.2256555	0	1.87550301	5.61279034	-0.2605218	5.11507467	0.20429136	-6.9077553	17	62	2.68223333	296.333333	23.33333333	0	0	1	0	42.288408	2	-0.4111751
6.6339	278181.36	0.76908612	155.344582	1.26133165	0	1.89219287	5.62827687	-0.2612529	5.0456522	0.23296053	-6.9077553	18	73	3.12927222	295.777778	22.7777778	0	0	1	0	42.288408	2	-0.4111751
6.7266	281558.975	0.60694948	97.1307077	1.07496947	0	1.90606982	5.64034548	-0.4976635	4.57606787	0.07322209	-6.9077553	19	80	2.23519444	295.222222	22.2222222	0	0	1	0	42.288408	2	-0.4111751
6.7608	282874.917	0.24058535	13.827966	0.48224414	0	1.91114123	5.64500835	-1.4205324	2.62676538	-0.7272333	-6.9077553	20	84	3.12927222	295.222222	22.2222222	0	0	1	0	42.288408	2	-0.4111751
6.7851	283708.322	0.25118791	21.9453464	0.39791065	0	1.91472903	5.6479502	-1.3775808	3.08860068	-0.9190178	-6.9077553	21	88	2.23519444	294.666667	21.66666666	0	0	1	0	42.288408	2	-0.4111751
6.8058	284326.123	0.45845417	91.7138035	1.08371046	0	1.91777519	5.65012542	-0.7777161	4.5186838	0.0813131	-6.9077553	22	90	2.23519444	294.666667	21.66666666	0	0	1	0	42.288408	2	-0.4111751
6.822	284984.067	0.1905101	7.35608758	0.26222708	0	1.92015268	5.65243678	-1.6528147	1.99566415	-1.3347382	-6.9077553	23	89	3.12927222	295.222222	22.2222222	0	0	1	0	42.288408	2	-0.4111751
0.0018	0	0.45057252	45.9067039	2.08759046	0	-6.3199686	-6.9077553	-0.7950193	3.82663295	0.73648942	-6.9077553	0	93	4.47038889	293.555556	20.55555555	0	0	0	0	18.9135952	2	-0.0428739
0.0018	0	0	0	0	0	-6.3199686	-6.9077553	-6.9077553	-6.9077553	-6.9077553	-6.9077553	1	92	2.68223333	293.555556	20.55555555	0	0	0	0	18.9135952	2	-0.0428739
0.0018	0	0	0	0	0	-6.3199686	-6.9077553	-6.9077553	-6.9077553	-6.9077553	-6.9077553	2	91	2.68223333	294.111111	21.11111111	0	0	0	0	18.9135952	2	-0.0428739
0.0018	0	0	0	0	0	-6.3199686	-6.9077553	-6.9077553	-6.9077553	-6.9077553	-6.9077553	3	91	2.68223333	294.666667	21.66666666	0	0	0	0	18.9135952	2	-0.0428739
0.0072	0	0.29284527	24.8875732	1.16351612	0	-4.9336743	-6.9077553	-1.2247019	3.21440879	0.15230566	-6.9077553	4	85	3.57631111	296.333333	23.33333333	0	0	0	0	18.9135952	2	-0.0428739
0.0738	103.654051	0.58961827	99.7806825	2.5088204	0	-2.6063965	-2.2570951	-0.5265854	4.60298462	0.9202112	-6.9077553	5	71	5.36446667	298.555556	25.55555555	0	0	0	0	18.9135952	2	-0.0428739
0.2826	5878.8042	0.8433392	234.174838	2.66996239	0	-1.2637228	1.77152346	-0.169201	5.45607228	0.98243885	-6.9077553	6	67	5.81150556	299.666667	26.66666666	0	0	1	0	18.9135952	2	-0.0428739
0.5274	13317.9845	0.86694224	265.530848	2.75696609	0	-0.639796	2.58919042	-0.1416301	5.58173479	1.01449348	-6.9077553	7	55	6.25854444	301.888889	28.88888888	0	0	1	0	18.9135952	2	-0.0428739
0.7245	17951.2772	0.77909747	197.793415	2.05488092	0	-0.3222735	2.88771697	-0.2483364	5.28722818	0.72070443	-6.9077553	8	50	5.81150556	303.555556	30.55555555	0	0	1	0	18.9135952	2	-0.0428739
0.9864	27548.3934	0.84661561	225.267773	1.93723824	0	-0.0136933	3.31598052	-0.165328	5.41729423	0.66177944	-6.9077553	9	45	5.36446667	304.111111	31.11111111	0	0	1	0	18.9135952	2	-0.0428739
1.2825	36523.7505	0.81293021	193.989623	1.93244354	0	0.2488113	3.59799013	-0.2058806	5.26780982	0.65930263	-6.9077553	10	46	3.57631111	304.111111	31.11111111	0	0	1	0	18.9135952	2	-0.0428739
1.5975	50470.2402	0.92887939	256.562991	1.96418283	0	0.46843991	3.92140367	-0.0727004	5.54737811	0.67558528	-6.9077553	11	44	4.02335	304.666667	31.66666666	0	0	1	0	18.9135952	2	-0.0428739
1.8585	60937.5212	0.83759155	207.154459	1.60541655	0	0.61976971	4.10986551	-0.1760315	5.33346952	0.47400596	-6.9077553	12	49	3.12927222	303	30	0	0	1	0	18.9135952	2	-0.0428739
2.1771	73407.2501	0.82686619	204.488965	1.66448423	0	0.77799372	4.29603633	-0.1889037	5.32051891	0.51011591	-6.9077553	13	53	3.12927222	301.333333	28.33333333	0	0	1	0	18.9135952	2	-0.0428739

2.457	86208.8891	0.89791978	227.64374	1.63514538	0	0.89894109	4.45678489	-0.1065615	5.42778626	0.4923431	-6.9077553	14	58	1.78815556	299.111111	26.11111111	0	0	1	0	18.9135952	2	-0.0428739
2.7243	96796.5883	0.77812251	179.911879	1.46862382	0	1.00221151	4.57262208	-0.249587	5.19247273	0.38500646	-6.9077553	15	51	2.68223333	298.555556	25.55555555	0	0	1	0	18.9135952	2	-0.0428739
3.0357	107922.532	0.75544104	179.995586	1.56651915	0	1.11044204	4.68142294	-0.2791307	5.19293788	0.44949421	-6.9077553	16	53	2.23519444	297.444444	24.44444444	0	0	1	0	18.9135952	2	-0.0428739
3.2229	115862.165	0.81320601	183.854661	1.33952977	0	1.17028158	4.75240988	-0.2055419	5.214151	0.29306489	-6.9077553	17	62	2.68223333	296.333333	23.33333333	0	0	1	0	18.9135952	2	-0.0428739
3.4308	126297.471	0.88950312	218.644531	1.71345949	0	1.23279347	4.83864793	-0.1159687	5.38745184	0.53909787	-6.9077553	18	73	3.12927222	295.777778	22.7777778	0	0	1	0	18.9135952	2	-0.0428739
3.5676	132032.494	0.75257321	149.848119	1.41513242	0	1.2718931	4.88305563	-0.2829291	5.00962891	0.34792951	-6.9077553	19	80	2.23519444	295.222222	22.2222222	0	0	1	0	18.9135952	2	-0.0428739
3.5982	133479.994	0.27406005	16.4143497	0.46151991	0	1.28043372	4.8939591	-1.2907658	2.79821686	-0.7710657	-6.9077553	20	84	3.12927222	295.222222	22.2222222	0	0	1	0	18.9135952	2	-0.0428739
3.6387	135102.948	0.23657691	12.5054525	0.43935051	0	1.29162648	4.90604447	-1.4372639	2.52624471	-0.8201843	-6.9077553	21	88	2.23519444	294.666667	21.66666666	0	0	1	0	18.9135952	2	-0.0428739
3.6675	136331.142	0.22431456	10.6422825	0.33928245	0	1.29951023	4.91509413	-1.4902578	2.36492894	-1.0779793	-6.9077553	22	90	2.23519444	294.666667	21.66666666	0	0	1	0	18.9135952	2	-0.0428739
3.6927	137471.605	0.22630422	10.9102434	0.39012228	0	1.3063579	4.92342466	-1.481466	2.38979377	-0.938735	-6.9077553	23	89	3.12927222	295.222222	22.2222222	0	0	1	0	18.9135952	2	-0.0428739
0.0081	273.827684	0.96418697	252.494583	1.77104735	0	-4.8158912	-1.291611	-0.0354334	5.53139376	0.57213558	-6.9077553	0	76	3.12927222	297.444444	24.44444444	0	0	0	0	30.519848	2	0.21141735
0.0126	493.146618	0.41034255	39.1284241	0.61937284	0	-4.3740585	-0.704923	-0.888329	3.66687472	-0.4774346	-6.9077553	1	79	3.12927222	296.888889	23.88888888	0	0	0	0	30.519848	2	0.21141735
0.0171	668.601752	0.27131629	17.0537262	0.66668615	0	-4.0686768	-0.4010721	-1.3007911	2.83642736	-0.403937	-6.9077553	2	82	2.68223333	296.333333	23.33333333	0	0	0	0	30.519848	2	0.21141735
0.0216	887.920785	0.25848429	13.9135742	0.35899508	0	-3.835062	-0.1177472	-1.3490591	2.6329368	-1.0216649	-6.9077553	3	86	2.23519444	295.777778	22.7777778	0	0	0	0	30.519848	2	0.21141735
0.0252	1019.51212	0.28557867	17.1086044	0.4298867	0	-3.6809113	0.02030458	-1.2497422	2.83963997	-0.8419101	-6.9077553	4	86	2.68223333	296.333333	23.33333333	0	0	0	0	30.519848	2	0.21141735
0.0324	1151.10336	0.25847006	14.042572	0.3760604	0	-3.4295969	0.14158928	-1.349114	2.64216478	-0.9753499	-6.9077553	5	86	1.34111667	296.333333	23.33333333	0	0	0	0	30.519848	2	0.21141735
0.0396	1370.42228	0.18853582	7.75299215	0.45097896	0	-3.2289262	0.31584836	-1.6631772	2.04820783	-0.7941196	-6.9077553	6	86	2.23519444	296.333333	23.33333333	0	0	0	0	30.519848	2	0.21141735
0.0405	1370.42228	0.24686883	12.4118357	0.34799933	0	-3.2064533	0.31584836	-1.3948556	2.51873107	-1.0526853	-6.9077553	7	87	1.34111667	296.333333	23.33333333	0	0	0	0	30.519848	2	0.21141735
0.0414	1370.42228	0.21844585	9.51376534	0.3021166	0	-3.1844744	0.31584836	-1.5166498	2.25284484	-1.1936377	-6.9077553	8	88	0.89407778	295.777778	22.7777778	0	0	0	0	30.519848	2	0.21141735
0.0432	1414.28607	0.14875633	4.39990377	0.19811578	0	-3.1419148	0.34733168	-1.8987458	1.48180992	-1.6138688	-6.9077553	9	88	2.23519444	295.777778	22.7777778	0	0	0	0	30.519848	2	0.21141735
0.0432	1414.28607	0	0	0	0	-3.1419148	0.34733168	-6.9077553	-6.9077553	-6.9077553	-6.9077553	10	88	2.68223333	295.777778	22.7777778	0	0	0	0	30.519848	2	0.21141735
0.0432	1414.28607	0	0	0	0	-3.1419148	0.34733168	-6.9077553	-6.9077553	-6.9077553	-6.9077553	11	86	2.68223333	296.333333	23.33333333	0	0	0	0	30.519848	2	0.21141735
0.0864	1646.89811	0.33164126	27.5685374	1.31588372	0	-2.4487676	0.4995006	-1.1006907	3.31671145	0.27526812	-6.9077553	12	82	3.57631111	296.888889	23.88888888	0	0	0	0	30.519848	2	0.21141735
0.1476	2186.49682	0.42008248	49.805901	1.27401307	0	-1.9132494	0.78275788	-0.8649265	3.90815355	0.24295643	-6.9077553	13	75	3.57631111	298.555556	25.55555555	0	0	0	0	30.519848	2	0.21141735
0.252	3172.57855	0.54069897	82.2750623	1.88623099	0	-1.3783262	1.15485983	-0.6130448	4.41008021	0.63511067	-6.9077553	14	69	4.47038889	299.111111	26.11111111	0	0	0	0	30.519848	2	0.21141735
0.3852	4187.8185	0.63575281	113.308013	2.08998232	0	-0.9539926	1.43241871	-0.4513738	4.73011872	0.73763396	-6.9077553	15	64	4.91742778	300.777778	27.7777778	0	0	0	1	30.519848	2	0.21141735
0.6147	10438.417	0.82169656	196.952451	2.71622985	0	-0.4866209	2.34558874	-0.1951678	5.28296741	0.99961292	-6.9077553	16	61	4.47038889	301.333333	28.33333333	0	0	0	1	30.519848	2	0.21141735
0.891	18718.474	0.80939262	184.942806	2.7505857	0	-0.1154109	2.92956437	-0.2102364	5.22005203	1.01217736	-6.9077553	17	59	4.47038889	301.333333	28.33333333	0	0	0	1	30.519848	2	0.21141735
1.152	29195.6836	0.76510589	167.041878	1.91602446	0	0.14149956	3.37405513	-0.2664349	5.11825053	0.65077423	-6.9077553	18	54	6.25854444	301.888889	28.88888888	0	0	0	1	30.519848	2	0.21141735
1.3653	38252.6256	0.69913731	148.010911	1.42226645	0	0.31137418	3.64423834	-0.3564788	4.99729275	0.35295455	-6.9077553	19	54	4.02335	301.888889	28.88888888	0	0	0	1	30.519848	2	0.21141735
1.5237	45125.0744	0.65426714	129.018754	1.17115558	0	0.42114159	3.80946023	-0.4227123	4.85996553	0.15884443	-6.9077553	20	54	3.12927222	301.888889	28.88888888	0	0	0	1	30.519848	2	0.21141735
1.6011	50582.611	0.64679039	117.038892	0.83555124	0	0.47069089	3.92362763	-0.4341881	4.76251484	-0.1784675	-6.9077553	21	55	3.12927222	300.777778	27.7777778	0	0	0	1	30.519848	2	0.21141735
1.6641	56141.2827	0.76795976	158.498089	1.06523154	0	0.50928444	4.02788923	-0.2627166	5.06574884	0.06413051	-6.9077553	22	62	2.68223333	299.666667	26.66666666	0	0	0	1	30.519848	2	0.21141735
1.7217	61752.1841	0.6930598	126.750618	0.92311263	0	0.54331217	4.12314554	-0.3651972	4.84222941	-0.0789213	-6.9077553	23	63	3.12927222	298.555556	25.55555555	0	0	0	1	30.519848	2	0.21141735
0.0135	582.304178	0.96909932	250.256731	1.72916658	0	-4.3050656	-0.5390465	-0.0303568	5.52249131	0.54821769	-6.9077553	0	76	3.12927222	297.444444	24.44444444	0	0	0	0	34.0724638	2	0.49277013
0.0225	933.214432	0.46760597	51.0523895	0.71721191	0	-3.79424	-0.0680493	-0.757993	3.93287194	-0.3309906	-6.9077553	1	79	3.12927222	296.888889	23.88888888	0	0	0	0	34.0724638	2	0.49277013
0.027	1064.80586	0.3825814	32.5752472	0.54532723	0	-3.6119184	0.06373119	-0.9582034	3.48358341	-0.6045372	-6.9077553	2	82	2.68223333	296.333333	23.33333333	0	0	0	0	34.0724638	2	0.49277013
0.0342	1371.85233	0.2165903	10.0780458	0.34859279	0	-3.3755296	0.31689057	-1.5251413	2.3104586	-1.0509863	-6.9077553	3	86	2.23519444	295.777778	22.7777778	0	0	0	0	34.0724638	2	0.49277013
0.0414	1635.03498	0.20374408	8.93237019	0.43108055	0	-3.1844744	0.49227562	-1.5859945	2.18979372	-0.8391432	-6.9077553	4	86	2.68223333	296.333333	23.33333333	0	0	0	0	34.0724638	2	0.49277013
0.0432	1678.89874	0.18384045	6.72217941	0.23112205	0	-3.1419148	0.51873352	-1.6882622	1.90556117	-1.460492	-6.9077553	5	86	1.34111667	296.333333	23.33333333	0	0	0	0	34.0724638	2	0.49277013
0.0495	1985.94536	0.22583788	10.4986354	0.32310012	0	-3.0057826	0.68659847	-1.4835197	2.35134054	-1.1267028	-6.9077553	6	86	2.23519444	296.333333	23.33333333	0	0	0	0	34.0724638	2	0.49277013
0.0558	2249.12783	0.21295382	9.73729924	0.27884083	0	-2.8859814	0.81098703	-1.5419951	2.27606649	-1.2735343	-6.9077553	7	87	1.34111667	296.333333	23.33333333	0	0	0	0	34.0724638	2	0.49277013
0.0621	2468.44687	0.24331495	12.5182321	0.40313779	0	-2.7790093	0.90399419	-1.4092971	2.52726603	-0.9059994	-6.9077553	8	88	0.89407778	295.777778	22.7777778	0	0	0	0	34.0724638	2	0.49277013
0.0693	2819.35696	0.21250667	9.26156235	0.28058457	0	-2.6693104	1.03686346	-1.5440872	2.22598072	-1.2673224	-6.9077553	9	88	2.23519444	295.777778	22.7777778	0	0	0	0	34.0724638	2	0.49277013
0.0747	3082.53983	0.21878902	9.57578913	0.29152687	0	-2.5942752	1.12607823	-1.5150872	2.25934237	-1.2291987	-6.9077553	10	88	2.68223333	295.777778	22.7777778	0	0	0	0	34.0724638	2	0.49277013
0.0792	3257.99481	0.18595518	6.75329666	0.20442982	0	-2.535779	1.18141881	-1.6768864	1.91017884	-1.5826508	-6.9077553	11	86	2.68223333	296.333333	23.33333333	0	0	0	0	34.0724638	2	0.49277013
0.1215	5012.51828	0.70205989	140.807129	1.225356	0	-2.107841	1.61213792	-0.3523132	4.94739818	0.20404717	-6.9077553	12	82	3.57631111	296.888889	23.88888888	0	0	0	0	34.0724638	2	0.49277013
0.2178	9137.20221	0.88482723	221.747463	1.88205555	0	-1.5241781	2.21246367	-0.1212333	5.40154369	0.63289575	-6.9077553	13	75	3.57631111	298.555556	25.55555555	0	0	0	0	34.0724638	2	0.49277013
0.5193	17347.0317	1.19371374	424.980644	3.54540943	0	-0.6552735	2.85347905	0.17790661	6.05204598	1.26593566	-6.9077553	14	69	4.47038889	299.111111	26.11111111	0	0	0	0	34.0724638	2	0.49277013
1.2114	27803.2319	1.44882174	668.35777	5.2314777	0	0.19177672	3.32518823	0.37144061	6.50482511	1.65488491	-6.9077553	15	64	4.91742778	300.777778	27.7777778	0	0	0	0	34.0724638	2	0.49277013
1.8072	42763.5259	1.12904031	377.516073	3.01376942	0	0.59177869	3.75570892	0.1222533	5.9336158	1.10352335	-6.9077553	16	61	4.47038889	301.333333	28.33333333	0	0	0	1	34.0724638	2	0.49277013
2.3751	59151.2472	0.99187952	277.066388	2.43788826	0	0.86503954	4.08011458	-0.007146	5.62426075	0.89154231	-6.9077553	17	59	4.47038889	301.333333	28.33333333	0	0	0	1	34.0724638	2	0.49277013
2.8836	78273.0632	0.96455715	264.317561	2.18891191	0	1.05903951	4.3602163	-0.03505	5.57715505	0.78386132	-6.9077553	18	54	6.25854444	301.888889	28.88888888	0	0	0	1	34.0724638	2	0.49277013
3.663	100953.525	0.93168221	241.289658	2.78891308	0	1.29828248	4.61467016	-0.0696907	5.48600226	1.02601044	-6.9077553	19	54	4.02335	301.888889	28.88888888	0	0	0	1	34.0724638	2	0.49277013
4.3218	118102.331	0.83063778	200.593366	2.24174891	0	1.46367198	4.77155993	-0.1843583	5.30128479	0.80770231	-6.9077553	20	54	3.12927222	301.888889	28.88888888	0	0	0	1	34.0724638	2	0.49277013
4.7565	131568.642	0.7713321	177.781962	1.53146697	0	1.5595121	4.87953631	-0.2583406	5.18056349	0.42687884	-6.9077553	21	55	3.12927222	300.777778	27.7777778	0	0	0	1	34.0724638	2	0.49277013
5.031	143590.515	0.82793609	189.708017	1.38737913	0	1.61561877	4.96697257	-0.1876122	5.24549141	0.32813697	-6.9077553	22	62	2.68223333	299.666667	26.66666666	0	0	0	1	34.0724638	2	0.49277013
5.2821	154990.31	0.88077187	208.678623	1.58629977	0	1.66432375	5.04336905	-0.1258219	5.34080017	0.46203432	-6.9077553	23	63	3.12927222	298.555556	25.55555555	0	0	0	1	34.0724638	2	0.49277013
0.0243	1340.3048	1.00364296	272.896184	1.88488212	0	-3.7172789	0.29364287	0.00463222	5.60909511	0.63439568	-6.9077553	0	76	3.12927222	297.444444	24.44444444	0	0	0	0	42.288408	2	-0.4111751
0.0594	3051.63301	0.60346422	93.2726988	1.10412519	0	-2.8234611	1.1160045	-0.5034128	4.53553817	0.09995863	-6.9077553	1	79	3.12927222	296.888889	23.88888888	0	0	0	0	42.288408	2	-0.4111751
0.0819	4148.22751	0.39569	36.329231	0.66023285	0	-2.5022563	1.42292217	-0.9246002	3.5926502	-0.4136492	-6.9077553	2	82	2.68223333	296.333333	23.33333333	0	0	0	0	42.288408	2	-0.4111751
0.1044	5200.95816	0.27047419	16.0479187	0.49855881	0	-2.2595256	1.64903512	-1.3038882	2.77564148	-0.69403	-6.9077553	3	86	2.23519444	295.777778	22.7777778	0	0	0	0	42.288408	2	-0.4111751

0.1314	6516.87148	0.29422293	18.8748474	0.49243164	0	-2.0295092	1.87454786	-1.2200245	2.93788319	-0.7063709	-6.9077553	4	86	2.68223333	296.333333	23.33333333	0	0	0	0	42.288408	2	-0.4111751
0.1548	7569.60228	0.27829388	16.9047476	0.49106088	0	-1.8656213	2.02427263	-1.2754907	2.82765366	-0.7091528	-6.9077553	5	86	1.34111667	296.333333	23.33333333	0	0	0	0	42.288408	2	-0.4111751
0.1827	8929.37983	0.26123311	14.6915273	0.44508543	0	-1.6999098	2.18945893	-1.3385214	2.68733902	-0.8072448	-6.9077553	6	86	2.23519444	296.333333	23.33333333	0	0	0	0	42.288408	2	-0.4111751
0.2079	10157.5656	0.25519071	14.1402686	0.43876859	0	-1.5706981	2.31831725	-1.3618331	2.64909737	-0.8215066	-6.9077553	7	87	1.34111667	296.333333	23.33333333	0	0	0	0	42.288408	2	-0.4111751
0.2349	11473.4794	0.24146957	12.6259104	0.43475911	0	-1.4485954	2.44012538	-1.416879	2.53583029	-0.8306657	-6.9077553	8	88	0.89407778	295.777778	22.7777778	0	0	0	0	42.288408	2	-0.4111751
0.2583	12570.0735	0.25278062	13.4879174	0.44567801	0	-1.3536336	2.53139842	-1.3712851	2.60186841	-0.8059173	-6.9077553	9	88	2.23519444	295.777778	22.7777778	0	0	0	0	42.288408	2	-0.4111751
0.2916	14149.1696	0.34009578	33.8342021	0.59536104	0	-1.2323723	2.64972661	-1.075592	3.52150174	-0.516909	-6.9077553	10	88	2.68223333	295.777778	22.7777778	0	0	0	0	42.288408	2	-0.4111751
0.3924	18704.6154	0.78483159	173.010585	1.56069156	0	-0.9354736	2.92882377	-0.2410128	5.15335856	0.44576957	-6.9077553	11	86	2.68223333	296.333333	23.33333333	0	0	0	0	42.288408	2	-0.4111751
0.603	26057.0079	0.72435787	156.533971	1.77654325	0	-0.5058381	3.26032513	-0.3210901	5.05327944	0.57523221	-6.9077553	12	82	3.57631111	296.888889	23.88888888	0	0	1	0	42.288408	2	-0.4111751
0.8748	36933.679	0.84369563	197.616114	1.72417485	0	-0.13376	3.60915092	-0.1687789	5.28633139	0.54532841	-6.9077553	13	75	3.57631111	298.555556	25.55555555	0	0	1	0	42.288408	2	-0.4111751
1.2366	48433.5464	0.81273693	174.13083	1.90219344	0	0.21236568	3.88021333	-0.2061181	5.15981266	0.64353323	-6.9077553	14	69	4.47038889	299.111111	26.11111111	0	0	1	0	42.288408	2	-0.4111751
1.6236	62348.2074	0.81300389	171.655487	1.6445392	0	0.48464591	4.13275096	-0.2057901	5.14549531	0.49806811	-6.9077553	15	64	4.91742778	300.777778	27.7777778	0	0	1	0	42.288408	2	-0.4111751
2.0574	79096.0491	0.82800559	180.232268	1.69231937	0	0.72144305	4.37067557	-0.1875284	5.19425194	0.52669073	-6.9077553	16	61	4.47038889	301.333333	28.33333333	0	0	1	0	42.288408	2	-0.4111751
2.6811	102606.127	0.90003525	229.195482	2.3695049	0	0.98622716	4.6309074	-0.1042109	5.43457963	0.86310297	-6.9077553	17	59	4.47038889	301.333333	28.33333333	0	0	1	0	42.288408	2	-0.4111751
3.4641	138100.826	0.9954862	288.843445	2.70304222	0	1.24245286	4.92799128	-0.00352	5.66588829	0.99474777	-6.9077553	18	54	6.25854444	301.888889	28.88888888	0	0	1	0	42.288408	2	-0.4111751
4.2507	174196.5	0.97232593	265.783109	2.21919443	0	1.44708368	5.16018972	-0.0270363	5.58268436	0.79759477	-6.9077553	19	54	4.02335	301.888889	28.88888888	0	0	1	0	42.288408	2	-0.4111751
4.9527	207116.973	0.93187929	249.902684	1.95201713	0	1.59993288	5.33328855	-0.0694795	5.52107558	0.66937542	-6.9077553	20	54	3.12927222	301.888889	28.88888888	0	0	1	0	42.288408	2	-0.4111751
5.4927	231108.032	0.88426656	226.104427	1.67540731	0	1.70341994	5.4428896	-0.1218665	5.42100138	0.516653	-6.9077553	21	55	3.12927222	300.777778	27.7777778	0	0	1	0	42.288408	2	-0.4111751
5.8167	244682.808	0.84915788	199.402561	1.42679749	0	1.76073309	5.4999668	-0.1623332	5.29533071	0.35613304	-6.9077553	22	62	2.68223333	299.666667	26.66666666	0	0	1	0	42.288408	2	-0.4111751
6.0444	254503.505	0.80257889	176.479064	1.40127262	0	1.79913222	5.53931854	-0.2186799	5.17320792	0.33809422	-6.9077553	23	63	3.12927222	298.555556	25.55555555	0	0	1	0	42.288408	2	-0.4111751
0.0009	0	0	0	0	0	-7.0131158	-6.9077553	-6.9077553	-6.9077553	-6.9077553	-6.9077553	0	76	3.12927222	297.444444	24.44444444	0	0	0	0	18.9135952	2	-0.0428739
0.0009	0	0	0	0	0	-7.0131158	-6.9077553	-6.9077553	-6.9077553	-6.9077553	-6.9077553	1	79	3.12927222	296.888889	23.88888888	0	0	0	0	18.9135952	2	-0.0428739
0.0009	0	0	0	0	0	-7.0131158	-6.9077553	-6.9077553	-6.9077553	-6.9077553	-6.9077553	2	82	2.68223333	296.333333	23.33333333	0	0	0	0	18.9135952	2	-0.0428739
0.0009	0	0	0	0	0	-7.0131158	-6.9077553	-6.9077553	-6.9077553	-6.9077553	-6.9077553	3	86	2.23519444	295.777778	22.7777778	0	0	0	0	18.9135952	2	-0.0428739
0.0009	0	0	0	0	0	-7.0131158	-6.9077553	-6.9077553	-6.9077553	-6.9077553	-6.9077553	4	86	2.68223333	296.333333	23.33333333	0	0	0	0	18.9135952	2	-0.0428739
0.0009	0	0	0	0	0	-7.0131158	-6.9077553	-6.9077553	-6.9077553	-6.9077553	-6.9077553	5	86	1.34111667	296.333333	23.33333333	0	0	0	0	18.9135952	2	-0.0428739
0.0009	0	0	0	0	0	-7.0131158	-6.9077553	-6.9077553	-6.9077553	-6.9077553	-6.9077553	6	86	2.23519444	296.333333	23.33333333	0	0	0	0	18.9135952	2	-0.0428739
0.0009	0	0	0	0	0	-7.0131158	-6.9077553	-6.9077553	-6.9077553	-6.9077553	-6.9077553	7	87	1.34111667	296.333333	23.33333333	0	0	0	0	18.9135952	2	-0.0428739
0.0009	0	0	0	0	0	-7.0131158	-6.9077553	-6.9077553	-6.9077553	-6.9077553	-6.9077553	8	88	0.89407778	295.777778	22.7777778	0	0	0	0	18.9135952	2	-0.0428739
0.0009	0	0	0	0	0	-7.0131158	-6.9077553	-6.9077553	-6.9077553	-6.9077553	-6.9077553	9	88	2.23519444	295.777778	22.7777778	0	0	0	0	18.9135952	2	-0.0428739
0.0009	0	0	0	0	0	-7.0131158	-6.9077553	-6.9077553	-6.9077553	-6.9077553	-6.9077553	10	88	2.68223333	295.777778	22.7777778	0	0	0	0	18.9135952	2	-0.0428739
0.0009	0	0	0	0	0	-7.0131158	-6.9077553	-6.9077553	-6.9077553	-6.9077553	-6.9077553	11	86	2.68223333	296.333333	23.33333333	0	0	0	0	18.9135952	2	-0.0428739
0.0009	0	0	0	0	0	-7.0131158	-6.9077553	-6.9077553	-6.9077553	-6.9077553	-6.9077553	12	82	3.57631111	296.888889	23.88888888	0	0	0	0	18.9135952	2	-0.0428739
0.0261	0	0.60977772	112.668117	2.88861166	0	-3.64582	-6.9077553	-0.4930222	4.72445535	1.06112212	-6.9077553	13	75	3.57631111	298.555556	25.55555555	0	0	0	0	18.9135952	2	-0.0428739
0.0927	504.984846	0.67238885	131.783625	2.42535503	0	-2.3783868	-0.6812486	-0.3954323	4.88116896	0.88639015	-6.9077553	14	69	4.47038889	299.111111	26.11111111	0	0	1	0	18.9135952	2	-0.0428739
0.2169	1984.25469	0.62309973	104.444138	1.96038022	0	-1.5283189	0.68574721	-0.4714451	4.64866194	0.67364842	-6.9077553	15	64	4.91742778	300.777778	27.7777778	0	0	1	0	18.9135952	2	-0.0428739
0.4149	8025.12353	0.77688619	198.314506	1.9517137	0	-0.8797178	2.08270166	-0.251175	5.28985923	0.66922005	-6.9077553	16	61	4.47038889	301.333333	28.33333333	0	0	1	0	18.9135952	2	-0.0428739
0.7101	19288.4556	0.89313898	256.446623	2.45211457	0	-0.3423495	2.9595586	-0.1118941	5.54692444	0.89735847	-6.9077553	17	59	4.47038889	301.333333	28.33333333	0	0	1	0	18.9135952	2	-0.0428739
1.0863	31231.9246	0.96308702	301.00015	3.17789791	0	0.08277743	3.44147281	-0.0365737	5.70711408	1.15653457	-6.9077553	18	54	6.25854444	301.888889	28.88888888	0	0	1	0	18.9135952	2	-0.0428739
1.4247	43529.0125	0.86920791	221.869515	2.07147736	0	0.35396127	3.77345064	-0.1390231	5.40209394	0.72874469	-6.9077553	19	54	4.02335	301.888889	28.88888888	0	0	1	0	18.9135952	2	-0.0428739
1.7874	58463.7289	0.88711457	224.916311	1.9379748	0	0.58076205	4.06842365	-0.1186545	5.41573283	0.66215938	-6.9077553	20	54	3.12927222	301.888889	28.88888888	0	0	1	0	18.9135952	2	-0.0428739
2.1294	73740.5502	0.87103255	217.130325	1.61335819	0	0.75584025	4.30056641	-0.1369285	5.38050235	0.47893747	-6.9077553	21	55	3.12927222	300.777778	27.7777778	0	0	1	0	18.9135952	2	-0.0428739
2.4003	87470.7436	0.92599437	234.394414	1.67157895	0	0.87559373	4.47131581	-0.0758078	5.45700949	0.51436672	-6.9077553	22	62	2.68223333	299.666667	26.66666666	0	0	1	0	18.9135952	2	-0.0428739
2.6676	101378.085	0.94089855	241.084649	1.78711883	0	0.98117919	4.61886681	-0.0598577	5.48515226	0.58116413	-6.9077553	23	63	3.12927222	298.555556	25.55555555	0	0	1	0	18.9135952	2	-0.0428739
0.0135	405.930043	0.73705373	164.878627	1.40410655	0	-4.3050656	-0.899114	-0.3037386	5.10521568	0.34011314	-6.9077553	0	58	2.68223333	305.777778	32.7777778	0	0	0	0	30.519848	2	0.21141735
0.0333	1193.94576	0.90683157	222.78782	1.57735946	0	-3.4021979	0.1781008	-0.0966964	5.40622433	0.45638599	-6.9077553	1	65	1.78815556	304.111111	31.11111111	0	0	0	0	30.519848	2	0.21141735
0.0432	1457.24304	0.69938985	134.202615	1.52924763	0	-3.1419148	0.37723231	-0.3561182	4.89935816	0.42542957	-6.9077553	2	70	1.78815556	303	30	0	0	0	0	30.519848	2	0.21141735
0.0441	1501.10673	0.58925223	82.2652512	1.049052	0	-3.1212955	0.40686861	-0.5272053	4.40996095	0.04883969	-6.9077553	3	77	0.44703889	301.888889	28.88888888	0	0	0	0	30.519848	2	0.21141735
0.0441	1501.10673	0	0	0	0	-3.1212955	0.40686861	-6.9077553	-6.9077553	-6.9077553	-6.9077553	4	75	0.89407778	297.444444	24.44444444	0	0	0	0	30.519848	2	0.21141735
0.0441	1501.10673	0	0	0	0	-3.1212955	0.40686861	-6.9077553	-6.9077553	-6.9077553	-6.9077553	5	88	0.89407778	295.222222	22.2222222	0	0	0	0	30.519848	2	0.21141735
0.0441	1501.10673	0	0	0	0	-3.1212955	0.40686861	-6.9077553	-6.9077553	-6.9077553	-6.9077553	6	46	2.23519444	296.888889	23.88888888	0	0	0	0	30.519848	2	0.21141735
0.0909	1764.28944	0.33419862	34.6516043	1.18242785	0	-2.3979953	0.56831467	-1.093032	3.54537288	0.16841518	-6.9077553	7	42	2.23519444	296.333333	23.33333333	0	0	0	0	30.519848	2	0.21141735
0.1593	2523.64362	0.44475274	57.9908255	1.38038173	0	-1.8369661	0.92609991	-0.8079909	4.06030206	0.32308426	-6.9077553	8	35	3.12927222	295.777778	22.7777778	0	0	0	0	30.519848	2	0.21141735
0.3582	4129.8883	0.68027741	127.61162	3.11355936	0	-1.0266638	1.41849247	-0.3837857	4.84899927	1.13608768	-6.9077553	9	36	4.91742778	294.666667	21.66666666	0	0	0	0	30.519848	2	0.21141735
0.5067	5790.40326	0.52159775	74.8757813	1.38981675	0	-0.6798362	1.75637462	-0.6489432	4.31584385	0.32989116	-6.9077553	10	43	2.68223333	293	20	0	0	0	1	30.519848	2	0.21141735
0.6318	7606.41707	0.49444459	72.0924994	0.89770908	0	-0.4591824	2.0291237	-0.7022998	4.27796388	-0.1067959	-6.9077553	11	54	0.44703889	290.222222	17.2222222	0	0	0	1	30.519848	2	0.21141735
0.7569	9371.29062	0.47674626	66.9126995	0.89995493	0	-0.2785241	2.23775753	-0.7386755	4.20340372	-0.1043	-6.9077553	12	55	1.34111667	291.333333	18.33333333	0	0	0	1	30.519848	2	0.21141735
1.0242	12083.9847	0.6095896	104.312743	1.78700292	0	0.02391182	2.49196375	-0.4933302	4.64740312	0.58109931	-6.9077553	13	46	3.12927222	294.111111	21.11111111	0	0	0	1	30.519848	2	0.21141735
1.7568	19164.7291	1.00413907	319.427096	4.36640649	0	0.56349397	2.95312374	0.00512591	5.7665322	1.47416935	-6.9077553	14	40	5.36446667	295.777778	22.7777778	0	0	0	1	30.519848	2	0.21141735
2.7387	29217.4946	0.98811733	291.489402	4.43716031	0	1.00748336	3.37480189	-0.0109423	5.67500708	1.49023995	-6.9077553	15	35	6.25854444	297.444444	24.44444444	0	0	0	1	30.519848	2	0.21141735
3.8547	49002.1019	0.99749303	296.392994	2.95347526	0	1.34929318	3.8918836	-0.0015081	5.69168963	1.08332106	-6.9077553	16	31	6.70558333	299.111111	26.11111111	0	0	0	1	30.519848	2	0.21141735
5.0076	163009.085	1.28252102	465.187695	2.31721281	95	1.61095676	5.09381207	0.2496071	6.14244312	0.84079655	4.55388742	17	27	6.25854444	300.777778	27.7777778	0	0	0	1	30.519848	2	0.21141735

6.1641	260280.577	1.17461308	391.724319	3.08956978	173	1.81874214	5.56176403	0.16178978	5.97056088	1.12835547	5.15329737	18	24	7.59966111	301.888889	28.88888888	0	0	0	1	30.519848	2	0.21141735
7.3098	584153.103	1.96349915	895.357227	3.437388	459	1.98921591	6.37016482	0.67523733	6.79722389	1.23500276	6.12905239	19	23	5.36446667	303.555556	30.55555555	0	0	0	1	30.519848	2	0.21141735
9.1305	1441437.16	2.91204445	1492.38853	4.57236845	1308	2.21162046	7.27339661	1.06919874	7.30813383	1.52025001	7.1762553	20	23	5.81150556	303.555556	30.55555555	0	0	0	1	30.519848	2	0.21141735
11.4237	2068703.68	2.3812687	1224.58301	5.53146463	1949	2.43569014	7.63467793	0.86805327	7.11035648	1.7106334	7.57507221	21	22	6.25854444	304.111111	31.11111111	0	0	0	1	30.519848	2	0.21141735
12.5748	2238570.09	1.64535339	734.372459	2.54026469	2124	2.53169481	7.71359303	0.49856277	6.5990177	0.93266187	7.66105685	22	23	5.36446667	304.111111	31.11111111	0	0	0	1	30.519848	2	0.21141735
13.5171	2352068.43	1.23277095	441.003074	2.39779718	2219	2.60395555	7.76305083	0.21007529	6.08905411	0.87496744	7.70481237	23	24	4.91742778	303.555556	30.55555555	0	0	0	1	30.519848	2	0.21141735
0.0351	1302.49534	0.97870315	271.892347	2.6107515	0	-3.3495541	0.26504938	-0.0205057	5.60540988	0.96002107	-6.9077553	0	58	2.68223333	305.777778	32.7777778	0	0	0	0	34.0724638	2	0.49277013
0.0882	3617.08554	0.84820634	194.528469	1.33473736	0	-2.4281483	1.28594503	-0.1634531	5.27058366	0.28948347	-6.9077553	1	65	1.78815556	304.111111	31.11111111	0	0	0	0	34.0724638	2	0.49277013
0.1503	6473.91917	0.7578878	146.201144	1.0595482	0	-1.895122	1.86793612	-0.2759013	4.98499021	0.05878594	-6.9077553	2	70	1.78815556	303	30	0	0	0	0	34.0724638	2	0.49277013
0.1935	8316.19851	0.66029537	107.553569	0.86876758	0	-1.6424778	2.11832548	-0.4135547	4.67799833	-0.1395293	-6.9077553	3	77	0.44703889	301.888889	28.88888888	0	0	0	0	34.0724638	2	0.49277013
0.2259	9588.24899	0.51614226	62.6091173	0.67463472	0	-1.4876629	2.26064257	-0.6594373	4.13692688	-0.3921027	-6.9077553	4	75	0.89407778	297.444444	24.44444444	0	0	0	0	34.0724638	2	0.49277013
0.2439	10158.4779	0.44394288	47.655304	0.63161077	0	-1.410997	2.31840705	-0.8098094	3.86401492	-0.4578999	-6.9077553	5	88	0.89407778	295.222222	22.2222222	0	0	0	0	34.0724638	2	0.49277013
0.2664	10948.026	0.337691	36.1569263	0.57365265	0	-1.3227563	2.3932505	-1.0826671	3.58789619	-0.5539895	-6.9077553	6	46	2.23519444	296.888889	23.88888888	0	0	0	0	34.0724638	2	0.49277013
0.3708	14875.2368	0.8217119	199.127913	1.62749218	0	-0.9920924	2.69976509	-0.1951492	5.29395242	0.48765454	-6.9077553	7	42	2.23519444	296.333333	23.33333333	0	0	0	1	34.0724638	2	0.49277013
0.5229	21456.4669	0.90253209	228.417483	1.61971811	0	-0.648365	3.06607269	-0.1014436	5.4311794	0.48286933	-6.9077553	8	35	3.12927222	295.777778	22.7777778	0	0	0	1	34.0724638	2	0.49277013
0.7686	29910.8236	0.9617456	264.930804	2.24467429	0	-0.2631846	3.39825384	-0.0379661	5.57947245	0.80900583	-6.9077553	9	36	4.91742778	294.666667	21.66666666	0	0	0	1	34.0724638	2	0.49277013
1.0323	41803.8563	0.952348	255.562607	1.90910678	0	0.03178932	3.73301251	-0.0477753	5.54347133	0.64715915	-6.9077553	10	43	2.68223333	293	20	0	0	0	1	34.0724638	2	0.49277013
1.2753	50507.7613	0.85203908	213.806366	1.59038018	0	0.24318145	3.92214681	-0.1589499	5.36507545	0.46460168	-6.9077553	11	54	0.44703889	290.222222	17.2222222	0	0	0	1	34.0724638	2	0.49277013
1.4553	55913.0293	0.69114151	138.103682	1.11163765	0	0.37521206	4.02381532	-0.3679649	4.92801196	0.10673346	-6.9077553	12	55	1.34111667	291.333333	18.33333333	0	0	0	1	34.0724638	2	0.49277013
1.8279	68050.6639	0.87778042	217.930442	1.92932866	0	0.60316777	4.22026718	-0.1292202	5.38418053	0.65769028	-6.9077553	13	46	3.12927222	294.111111	21.11111111	0	0	0	1	34.0724638	2	0.49277013
2.412	90204.6283	1.00248993	283.000794	2.45970288	0	0.88045628	4.50209182	0.00348386	5.64545324	0.90044703	-6.9077553	14	40	5.36446667	295.777778	22.7777778	0	0	0	1	34.0724638	2	0.49277013
3.2436	119812.421	1.05272321	312.678369	2.89819494	0	1.17668382	4.78593571	0.05232981	5.74517828	1.06443309	-6.9077553	15	35	6.25854444	297.444444	24.44444444	0	0	0	1	34.0724638	2	0.49277013
4.4037	162493.218	1.08148653	336.692324	3.15661138	0	1.4824451	5.09064242	0.07926073	5.8191725	1.14981585	-6.9077553	16	31	6.70558333	299.111111	26.11111111	0	0	0	1	34.0724638	2	0.49277013
5.7393	207128.575	1.0478222	315.339159	3.08517547	0	1.74733725	5.33334456	0.04766782	5.75365193	1.12693261	-6.9077553	17	27	6.25854444	300.777778	27.7777778	0	0	0	1	34.0724638	2	0.49277013
7.2342	265700.302	1.15277147	399.431291	4.0086315	0	1.97881978	5.58237275	0.14303612	5.99004427	1.38869934	-6.9077553	18	24	7.59966111	301.888889	28.88888888	0	0	0	1	34.0724638	2	0.49277013
8.901	317667.395	1.00719885	306.55953	3.37978948	0	2.18616363	5.76100806	0.00816542	5.72541522	1.21810926	-6.9077553	19	23	5.36446667	303.555556	30.55555555	0	0	0	1	34.0724638	2	0.49277013
10.5228	362789.836	0.89080641	244.117664	3.19922038	0	2.35354433	5.89382646	-0.1145062	5.49765444	1.16321967	-6.9077553	20	23	5.81150556	303.555556	30.55555555	0	0	0	1	34.0724638	2	0.49277013
12.2013	420317.524	1.00779803	326.383512	3.54410711	0	2.5015425	6.04101282	0.00875956	5.78807617	1.26556837	-6.9077553	21	22	6.25854444	304.111111	31.11111111	0	0	0	1	34.0724638	2	0.49277013
13.6521	473173.929	0.9983001	315.579676	3.0957316	0	2.61389336	6.15946515	-0.0007001	5.75441436	1.13034723	-6.9077553	22	23	5.36446667	304.111111	31.11111111	0	0	0	1	34.0724638	2	0.49277013
15.1299	524701.925	0.96647705	288.190907	3.19999905	0	2.71667292	6.26283225	-0.0330636	5.6636266	1.16346296	-6.9077553	23	24	4.91742778	303.555556	30.55555555	0	0	0	1	34.0724638	2	0.49277013
0.0405	2859.20527	1.23190871	421.853693	2.66503525	0	-3.2064533	1.05089339	0.20937618	6.04466093	0.98059244	-6.9077553	0	58	2.68223333	305.777778	32.7777778	0	0	0	0	42.288408	2	-0.4111751
0.126	7718.54856	0.99124226	272.361431	1.93207799	0	-2.0714734	2.04375589	-0.007788	5.60713365	0.65911355	-6.9077553	1	65	1.78815556	304.111111	31.11111111	0	0	0	0	42.288408	2	-0.4111751
0.2241	12824.7476	0.93077766	234.382203	1.78403578	0	-1.4956629	2.55145468	-0.0706611	5.4569574	0.57943846	-6.9077553	2	70	1.78815556	303	30	0	0	0	0	42.288408	2	-0.4111751
0.3231	17596.3174	0.88585094	208.245756	1.74160475	0	-1.1297934	2.86774647	-0.1200784	5.33872371	0.55538098	-6.9077553	3	77	0.44703889	301.888889	28.88888888	0	0	0	0	42.288408	2	-0.4111751
0.3987	21149.2842	0.66624651	113.403088	1.25588571	0	-0.919546	3.05165334	-0.4045957	4.73095744	0.22863701	-6.9077553	4	75	0.89407778	297.444444	24.44444444	0	0	0	0	42.288408	2	-0.4111751
0.4698	24439.0674	0.56825329	82.1962273	1.01307475	0	-0.7554482	3.19622389	-0.5634298	4.40912157	0.01397662	-6.9077553	5	88	0.89407778	295.222222	22.2222222	0	0	1	0	42.288408	2	-0.4111751
0.5472	27947.1918	0.53667632	97.9210006	0.95093669	0	-0.6029409	3.3303525	-0.6204985	4.58417125	-0.0492567	-6.9077553	6	46	2.23519444	296.888889	23.88888888	0	0	1	0	42.288408	2	-0.4111751
0.8676	40719.1456	0.86480199	228.619821	1.88063787	0	-0.1420245	3.70672295	-0.1440991	5.43206483	0.6321426	-6.9077553	7	42	2.23519444	296.333333	23.33333333	0	0	1	0	42.288408	2	-0.4111751
1.2114	52288.3228	0.83321872	192.151628	1.85088664	0	0.19177672	3.9567922	-0.1812597	5.25828999	0.61620492	-6.9077553	8	35	3.12927222	295.777778	22.7777778	0	0	1	0	42.288408	2	-0.4111751
1.5993	65612.003	0.80639967	173.115458	1.90041286	0	0.46956603	4.18377389	-0.2139365	5.15396458	0.64259722	-6.9077553	9	36	4.91742778	294.666667	21.66666666	0	0	1	0	42.288408	2	-0.4111751
1.98	80510.5854	0.85366713	202.880596	1.67183243	0	0.68309684	4.38840109	-0.1570432	5.31262254	0.51451826	-6.9077553	10	43	2.68223333	293	20	0	0	1	0	42.288408	2	-0.4111751
2.2923	92037.9302	0.72960678	148.208889	1.23631845	0	0.82955568	4.52221164	-0.3138799	4.99862944	0.2129465	-6.9077553	11	54	0.44703889	290.222222	17.2222222	0	0	1	0	42.288408	2	-0.4111751
2.6604	107647.606	0.80415191	180.188703	1.33327285	0	0.97847649	4.67887228	-0.2167243	5.1940102	0.28838646	-6.9077553	12	55	1.34111667	291.333333	18.33333333	0	0	1	0	42.288408	2	-0.4111751
3.3237	132507.791	0.86231943	210.088916	2.01448496	0	1.20107862	4.88664899	-0.1469705	5.34753561	0.70085984	-6.9077553	13	46	3.12927222	294.111111	21.11111111	0	0	1	0	42.288408	2	-0.4111751
4.4991	157573.651	0.81606546	198.713366	3.29421936	0	1.50387738	5.05989932	-0.2020361	5.29186845	1.19247274	-6.9077553	14	40	5.36446667	295.777778	22.7777778	0	0	1	0	42.288408	2	-0.4111751
6.0417	186261.248	0.83056762	205.988514	3.94571104	0	1.79868543	5.22715561	-0.1844427	5.32782526	1.37288259	-6.9077553	15	35	6.25854444	297.444444	24.44444444	0	0	1	0	42.288408	2	-0.4111751
7.785	239928.54	1.03676541	341.465636	4.41723671	0	2.05219881	5.4803453	0.03706976	5.83324998	1.48574068	-6.9077553	16	31	6.70558333	299.111111	26.11111111	0	0	1	0	42.288408	2	-0.4111751
9.2295	308934.609	1.19222188	431.537422	4.21706551	0	2.22240488	5.73313287	0.17665711	6.06735655	1.43937661	-6.9077553	17	27	6.25854444	300.777778	27.7777778	0	0	1	0	42.288408	2	-0.4111751
10.5651	379825.507	1.19199653	431.472793	3.55791002	0	2.35755612	5.93971459	0.17646824	6.06720677	1.26945433	-6.9077553	18	24	7.59966111	301.888889	28.88888888	0	0	1	0	42.288408	2	-0.4111751
12.0294	453009.986	1.17788998	411.120813	3.35550656	0	2.48735365	6.11591638	0.1645733	6.01888955	1.21090072	-6.9077553	19	23	5.36446667	303.555556	30.55555555	0	0	1	0	42.288408	2	-0.4111751
13.5396	520931.235	1.15028558	387.706794	3.59029828	1	2.60561872	6.25561997	0.14087921	5.96025195	1.27851377	0.0009995	20	23	5.81150556	303.555556	30.55555555	0	0	1	0	42.288408	2	-0.4111751
15.1164	601436.41	1.20314423	441.701912	3.8499126	2	2.71578025	6.39932247	0.18576913	6.09063751	1.34831016	0.69364706	21	22	6.25854444	304.111111	31.11111111	0	0	1	0	42.288408	2	-0.4111751
16.5681	663598.474	1.07142619	344.565096	3.15671588	2	2.80747916	6.49767877	0.06992355	5.84228593	1.14984894	0.69364706	22	23	5.36446667	304.111111	31.11111111	0	0	1	0	42.288408	2	-0.4111751
18.0234	719765.849	1.02373494	309.370362	2.97557356	2	2.89167091	6.57892734	0.02443398	5.73454237	1.09077283	0.69364706	23	24	4.91742778	303.555556	30.55555555	0	0	1	0	42.288408	2	-0.4111751
0.0027	0	0.21041781	8.77035046	0.69165194	0	-5.9145035	-6.9077553	-1.553919	2.17149078	-0.3672277	-6.9077553	0	58	2.68223333	305.777778	32.7777778	0	0	0	0	18.9135952	2	-0.0428739
0.0036	0	0.06053963	0.5845502	0.09518638	0	-5.6268214	-6.9077553	-2.788074	-0.5352034	-2.3414676	-6.9077553	1	65	1.78815556	304.111111	31.11111111	0	0	0	0	18.9135952	2	-0.0428739
0.0036	0	0	0	0	0	-5.6268214	-6.9077553	-6.9077553	-6.9077553	-6.9077553	-6.9077553	2	70	1.78815556	303	30	0	0	0	0	18.9135952	2	-0.0428739
0.0036	0	0	0	0	0	-5.6268214	-6.9077553	-6.9077553	-6.9077553	-6.9077553	-6.9077553	3	77	0.44703889	301.888889	28.88888888	0	0	0	0	18.9135952	2	-0.0428739
0.0036	0	0	0	0	0	-5.6268214	-6.9077553	-6.9077553	-6.9077553	-6.9077553	-6.9077553	4	75	0.89407778	297.444444	24.44444444	0	0	0	0	18.9135952	2	-0.0428739
0.0036	0	0	0	0	0	-5.6268214	-6.9077553	-6.9077553	-6.9077553	-6.9077553	-6.9077553	5	88	0.89407778	295.222222	22.2222222	0	0	0	0	18.9135952	2	-0.0428739
0.0036	0	0	0	0	0	-5.6268214	-6.9077553	-6.9077553	-6.9077553	-6.9077553	-6.9077553	6	46	2.23519444	296.888889	23.88888888	0	0	0	0	18.9135952	2	-0.0428739
0.0396	0	0.41784134	45.2463715	1.57748966	0	-3.2289262	-6.9077553	-0.8702631	3.81214458	0.45646848	-6.9077553	7	42	2.23519444	296.333333	23.33333333	0	0	0	0	18.9135952	2	-0.0428739

0.1179	0	0.40626833	41.056433	1.66732876	0	-2.1379185	-6.9077553	-0.898283	3.71497189	0.51182238	-6.9077553	8	35	3.12927222	295.777778	22.7777778	0	0	0	0	18.9135952	2	-0.0428739
0.2484	175.455131	0.37435711	35.3054654	1.59950372	0	-1.3927149	-1.7346886	-0.9798774	3.5640661	0.47031841	-6.9077553	9	36	4.91742778	294.666667	21.66666666	0	0	1	0	18.9135952	2	-0.0428739
0.342	745.684337	0.3910232	52.6515832	1.1234929	0	-1.0729445	-0.2921128	-0.9364343	3.9637153	0.11733218	-6.9077553	10	43	2.68223333	293	20	0	0	1	0	18.9135952	2	-0.0428739
0.4077	1831.63681	0.45920908	79.2103756	0.82117039	0	-0.8972237	0.60575581	-0.7760744	4.37211992	-0.1958076	-6.9077553	11	54	0.44703889	290.222222	17.2222222	0	0	1	0	18.9135952	2	-0.0428739
0.495	4275.20206	0.61225899	126.611852	1.11461608	0	-0.7031975	1.45306525	-0.4889679	4.84113402	0.10940679	-6.9077553	12	55	1.34111667	291.333333	18.33333333	0	0	1	0	18.9135952	2	-0.0428739
0.702	7263.02743	0.57775746	114.96999	1.59854551	0	-0.3538219	1.98293442	-0.5468718	4.74467983	0.46971953	-6.9077553	13	46	3.12927222	294.111111	21.11111111	0	0	1	0	18.9135952	2	-0.0428739
1.1466	15537.2954	0.83698226	234.953916	3.33021008	0	0.13680104	2.74330765	-0.1767583	5.45939365	1.20333563	-6.9077553	14	40	5.36446667	295.777778	22.7777778	0	0	1	0	18.9135952	2	-0.0428739
1.773	31840.277	0.94584691	275.864404	4.32850945	0	0.57267303	3.46076347	-0.0546179	5.61991308	1.46545424	-6.9077553	15	35	6.25854444	297.444444	24.44444444	0	0	1	0	18.9135952	2	-0.0428739
2.5578	66258.2174	1.10190841	388.599341	3.94540657	0	0.93914751	4.19357459	0.0979507	5.96255141	1.37280544	-6.9077553	16	31	6.70558333	299.111111	26.11111111	0	0	1	0	18.9135952	2	-0.0428739
3.2625	94542.1956	1.01244353	314.050746	2.90345033	0	1.18249377	4.54905683	0.01335397	5.74955777	1.06624416	-6.9077553	17	27	6.25854444	300.777778	27.7777778	0	0	1	0	18.9135952	2	-0.0428739
4.2777	142541.204	1.18520436	442.121509	4.11744896	0	1.45341548	4.95963812	0.1707586	6.09158701	1.41547663	-6.9077553	18	24	7.59966111	301.888889	28.88888888	0	0	1	0	18.9135952	2	-0.0428739
5.2506	187978.015	1.09021798	355.068195	2.8737208	0	1.65834236	5.23633034	0.08729448	5.87231269	1.05595556	-6.9077553	19	23	5.36446667	303.555556	30.55555555	0	0	1	0	18.9135952	2	-0.0428739
6.2145	233574.871	1.05935638	336.724148	2.95141716	0	1.82688527	5.45350696	0.05860506	5.81926701	1.08262421	-6.9077553	20	23	5.81150556	303.555556	30.55555555	0	0	1	0	18.9135952	2	-0.0428739
7.2693	281715.571	1.0839373	357.332551	3.16515301	0	1.98366	5.6409015	0.08152219	5.87866966	1.15251729	-6.9077553	21	22	6.25854444	304.111111	31.11111111	0	0	1	0	18.9135952	2	-0.0428739
8.2134	326615.824	1.06352928	336.866569	2.75688016	0	2.10576697	5.78878769	0.06253271	5.81968988	1.01446233	-6.9077553	22	23	5.36446667	304.111111	31.11111111	0	0	1	0	18.9135952	2	-0.0428739
9.1305	370631.846	1.027942	306.720743	2.25116608	0	2.21162046	5.91521194	0.02853109	5.72594096	0.81189245	-6.9077553	23	24	4.91742778	303.555556	30.55555555	0	0	1	0	18.9135952	2	-0.0428739
0.0081	318.354322	0.84122348	191.134491	1.71134472	0	-4.8158912	-1.1414541	-0.1717099	5.25298255	0.53786361	-6.9077553	0	87	4.02335	300.222222	27.2222222	0	0	0	0	30.519848	2	0.21141735
0.0126	537.673292	0.48541398	59.0094177	0.84862804	0	-4.3740585	-0.618646	-0.7206952	4.077714	-0.1629566	-6.9077553	1	88	2.68223333	300.222222	27.2222222	0	0	0	0	30.519848	2	0.21141735
0.0486	1371.50664	0.60338564	107.072571	1.28029022	0	-3.0241317	0.31663873	-0.5035428	4.67351618	0.24786756	-6.9077553	2	84	3.57631111	300.777778	27.7777778	0	0	0	0	30.519848	2	0.21141735
0.0909	1766.28069	0.43556453	56.0218584	1.19918953	0	-2.3979953	0.56944203	-0.8288191	4.02575979	0.18247949	-6.9077553	3	78	3.57631111	301.888889	28.88888888	0	0	0	0	30.519848	2	0.21141735
0.1674	2511.96483	0.4322091	52.7945887	1.42373092	0	-1.7873691	0.92146326	-0.8365347	3.96642764	0.35398297	-6.9077553	4	76	4.02335	301.888889	28.88888888	0	0	0	0	30.519848	2	0.21141735
0.2727	3293.48684	0.57284715	92.146451	1.83776751	0	-1.299383	1.19225042	-0.5553922	4.52339002	0.60909552	-6.9077553	5	66	4.02335	304.111111	31.11111111	0	0	0	0	30.519848	2	0.21141735
0.4113	4798.06769	0.65114683	117.245878	1.93187486	0	-0.8884324	1.56842167	-0.4274855	4.76428178	0.65900846	-6.9077553	6	63	3.57631111	304.666667	31.66666666	0	0	0	1	30.519848	2	0.21141735
0.6444	9627.96426	0.85214068	215.040752	2.87079234	0	-0.4394356	2.26477567	-0.1588308	5.3708322	1.05493635	-6.9077553	7	57	5.81150556	305.777778	32.7777778	0	0	0	1	30.519848	2	0.21141735
0.9441	17001.3967	0.80909816	185.638185	2.84901167	0	-0.0575232	2.83335432	-0.2105999	5.22380492	1.04732309	-6.9077553	8	56	5.36446667	305.777778	32.7777778	0	0	0	1	30.519848	2	0.21141735
1.2528	25201.7418	0.70894033	138.466541	1.93505361	0	0.22538105	3.22695279	-0.3425743	4.93063593	0.66065168	-6.9077553	9	55	5.81150556	306.333333	33.33333333	0	0	0	1	30.519848	2	0.21141735
1.4463	34804.9433	0.73315976	154.350327	1.3145786	0	0.36900857	3.54978816	-0.3090286	5.03923135	0.27427657	-6.9077553	10	59	3.12927222	305.777778	32.7777778	0	0	0	1	30.519848	2	0.21141735
1.6245	43851.206	0.69443288	138.745966	1.24075826	0	0.48520008	3.78082503	-0.3632208	4.93265188	0.21652833	-6.9077553	11	59	3.12927222	305.222222	32.2222222	0	0	0	1	30.519848	2	0.21141735
1.7343	50791.4524	0.70093055	135.536837	1.02929387	0	0.55060387	3.92774777	-0.3539208	4.90925084	0.02984408	-6.9077553	12	65	1.78815556	304.666667	31.66666666	0	0	0	1	30.519848	2	0.21141735
1.7991	56738.9662	0.70798175	131.765001	0.93963019	0	0.58728654	4.03847883	-0.3439255	4.88102763	-0.0612052	-6.9077553	13	70	1.34111667	304.111111	31.11111111	0	0	0	1	30.519848	2	0.21141735
1.8513	61709.9204	0.63537006	102.900054	0.89499691	0	0.6158881	4.12246091	-0.451975	4.63376788	-0.1098183	-6.9077553	14	73	1.78815556	303	30	0	0	0	1	30.519848	2	0.21141735
1.8756	62850.3802	0.4915064	59.4450548	0.77280419	0	0.62892861	4.14077289	-0.7082478	4.08506926	-0.2564364	-6.9077553	15	76	2.68223333	301.888889	28.88888888	0	0	0	1	30.519848	2	0.21141735
1.8918	63639.927	0.3436021	26.4722968	0.48265134	0	0.63752876	4.15325677	-1.0653649	3.27613656	-0.726391	-6.9077553	16	80	2.23519444	301.333333	28.33333333	0	0	0	1	30.519848	2	0.21141735
1.9035	64210.1576	0.25637397	13.8319503	0.33283421	0	0.6436943	4.16217699	-1.3572251	2.62705345	-1.0971108	-6.9077553	17	81	2.68223333	301.333333	28.33333333	0	0	0	1	30.519848	2	0.21141735
1.9161	64824.248	0.23546973	11.5480107	0.31757528	0	0.65029187	4.17169516	-1.4419351	2.44659978	-1.1438965	-6.9077553	18	83	2.68223333	300.777778	27.7777778	0	0	0	1	30.519848	2	0.21141735
1.9287	65394.4783	0.24919759	12.8532322	0.33498498	0	0.6568462	4.18045312	-1.3855043	2.55367311	-1.0906888	-6.9077553	19	84	2.68223333	300.777778	27.7777778	0	0	0	1	30.519848	2	0.21141735
1.9395	65789.2535	0.23558676	11.4414368	0.30290498	0	0.66243021	4.1864717	-1.4414403	2.43732897	-1.1910402	-6.9077553	20	85	2.23519444	300.222222	27.2222222	0	0	0	1	30.519848	2	0.21141735
1.9566	66403.3464	0.23563771	11.4619382	0.32671339	0	0.67120827	4.19576251	-1.4412249	2.43911906	-1.1156159	-6.9077553	21	84	3.57631111	300.222222	27.2222222	0	0	0	1	30.519848	2	0.21141735
1.9647	66666.5267	0.2409881	12.0403697	0.33420009	0	0.67533956	4.19971798	-1.4188667	2.48834819	-1.0930276	-6.9077553	22	84	2.68223333	300.222222	27.2222222	0	0	0	1	30.519848	2	0.21141735
1.9764	67149.0284	0.21938671	9.7607862	0.29899443	0	0.68127701	4.20692935	-1.5123715	2.2784754	-1.2039914	-6.9077553	23	83	3.57631111	300.222222	27.2222222	0	0	0	1	30.519848	2	0.21141735
0.0171	749.996847	1.00149801	279.370633	2.11955028	0	-4.0686768	-0.2863538	0.0024949	5.63254291	0.75167562	-6.9077553	0	87	4.02335	300.222222	27.2222222	0	0	0	0	34.0724638	2	0.49277013
0.0324	1320.22614	0.53988305	71.2428984	0.86928973	0	-3.4295969	0.2785602	-0.6145522	4.26610918	-0.1389291	-6.9077553	1	88	2.68223333	300.222222	27.2222222	0	0	0	0	34.0724638	2	0.49277013
0.0513	1934.31902	0.58739535	85.5701439	0.91631462	0	-2.9700645	0.66027218	-0.5303562	4.44934812	-0.0863048	-6.9077553	2	84	3.57631111	300.777778	27.7777778	0	0	0	0	34.0724638	2	0.49277013
0.1017	3947.53941	0.76525811	157.895682	1.62384047	0	-2.285728	1.37334574	-0.2662362	5.06194091	0.48540964	-6.9077553	3	78	3.57631111	301.888889	28.88888888	0	0	0	0	34.0724638	2	0.49277013
0.1647	6912.98142	0.88383805	208.536733	1.45400325	0	-1.8036296	1.93354565	-0.1223506	5.34012	0.37500814	-6.9077553	4	76	4.02335	301.888889	28.88888888	0	0	0	0	34.0724638	2	0.49277013
0.2295	9772.50647	0.86031744	194.109972	1.43344434	0	-1.4718522	2.2796753	-0.1492922	5.26843002	0.36077756	-6.9077553	5	66	4.02335	304.111111	31.11111111	0	0	0	0	34.0724638	2	0.49277013
0.351	15344.6289	1.01692641	292.400203	2.3517759	0	-1.0469691	2.73083067	0.01776762	5.67812684	0.85559586	-6.9077553	6	63	3.57631111	304.666667	31.66666666	0	0	0	1	34.0724638	2	0.49277013
0.6624	29193.8955	1.31818553	525.226066	4.28185753	0	-0.4118857	3.37399388	0.27701452	6.26383068	1.45462043	-6.9077553	7	57	5.81150556	305.777778	32.7777778	0	0	0	1	34.0724638	2	0.49277013
1.314	47459.9644	1.34245587	534.394769	4.44319954	1	0.27307592	3.85990757	0.2952453	6.2811367	1.49159977	0.0009995	8	56	5.36446667	305.777778	32.7777778	0	0	0	1	34.0724638	2	0.49277013
1.908	66406.6098	1.13406613	374.063613	3.25857747	2	0.64605557	4.19581166	0.12669091	5.92442854	1.18159758	0.69364706	9	55	5.81150556	306.333333	33.33333333	0	0	0	1	34.0724638	2	0.49277013
2.3634	80936.7861	0.94191528	248.42379	1.9498905	2	0.86010126	4.39368079	-0.0587788	5.51514014	0.66828593	0.69364706	10	59	3.12927222	305.777778	32.7777778	0	0	0	1	34.0724638	2	0.49277013
2.8611	97940.4518	0.94545245	250.553289	2.0135604	2	1.05120617	4.58436987	-0.0550345	5.52367562	0.70040101	0.69364706	11	59	3.12927222	305.222222	32.2222222	0	0	0	1	34.0724638	2	0.49277013
3.2427	111036.482	0.88565978	217.278523	1.65962622	2	1.17640632	4.70986782	-0.1202939	5.38118465	0.50719477	0.69364706	12	65	1.78815556	304.666667	31.66666666	0	0	0	1	34.0724638	2	0.49277013
3.4956	119836.026	0.80734753	177.719417	1.4119708	2	1.25150503	4.7861327	-0.2127632	5.18021163	0.34569444	0.69364706	13	70	1.34111667	304.111111	31.11111111	0	0	0	1	34.0724638	2	0.49277013
3.6828	127058.708	0.71152005	133.89024	1.24674724	2	1.30367333	4.84465711	-0.3389472	4.89702783	0.22133971	0.69364706	14	73	1.78815556	303	30	0	0	0	1	34.0724638	2	0.49277013
3.7836	131050.306	0.45534897	55.5261754	0.81912095	2	1.33067594	4.8755889	-0.7844975	4.01687255	-0.1983034	0.69364706	15	76	2.68223333	301.888889	28.88888888	0	0	0	1	34.0724638	2	0.49277013
3.852	133638.282	0.34737281	32.4980501	0.58029571	2	1.34859249	4.89514424	-1.0544821	3.48121086	-0.5424957	0.69364706	16	80	2.23519444	301.333333	28.33333333	0	0	0	1	34.0724638	2	0.49277013
3.906	135699.867	0.30964238	24.9451843	0.50678368	2	1.36251383	4.91045296	-1.1691129	3.21672088	-0.6776998	0.69364706	17	81	2.68223333	301.333333	28.33333333	0	0	0	1	34.0724638	2	0.49277013
3.9546	137586.007	0.25954377	14.5345018	0.39858719	2	1.37487946	4.92425649	-1.3449844	2.67659406	-0.9173233	0.69364706	18	83	2.68223333	300.777778	27.7777778	0	0	0	1	34.0724638	2	0.49277013
3.9933	138989.655	0.28027603	18.4253043	0.47851212	2	1.38461796	4.9344067	-1.2684188	2.91377923	-0.7349861	0.69364706	19	84	2.68223333	300.777778	27.7777778	0	0	0	1	34.0724638	2	0.49277013
4.0356	140568.744	0.31603762	30.2226381	0.54097561	2	1.39515499	4.94570376	-1.1487348	3.40862434	-0.6125343	0.69364706	20	85	2.23519444	300.222222	27.2222222	0	0	0	1	34.0724638	2	0.49277013
4.0734	141928.529	0.30872395	39.0910325	0.64311395	2	1.40447803	4.95533066	-1.1720739	3.66591867	-0.4398796	0.69364706	21	84	3.57631111	300.222222	27.2222222	0	0	0	1	34.0724638	2	0.49277013

4.1013	143244.446	0.22651402	10.836159	0.33771244	2	1.411304	4.96455956	-1.4805434	2.38298088	-1.0826038	0.69364706	22	84	2.68223333	300.222222	27.2222222	0	0	0	1	34.0724638	2	0.49277013
4.1454	144867.403	0.32381583	41.4901023	0.52355918	2	1.42199929	4.97582576	-1.1244969	3.725479	-0.645197	0.69364706	23	83	3.57631111	300.222222	27.2222222	0	0	0	1	34.0724638	2	0.49277013
0.0351	2258.84691	1.13575072	355.564273	2.459509	0	-3.3495541	0.81529707	0.12817395	5.87370884	0.90036824	-6.9077553	0	87	4.02335	300.222222	27.2222222	0	0	0	0	42.288408	2	-0.4111751
0.0819	4543.61724	0.72512253	136.358323	1.33665011	0	-2.5022563	1.51394351	-0.3200365	4.91529348	0.29091443	-6.9077553	1	88	2.68223333	300.222222	27.2222222	0	0	0	0	42.288408	2	-0.4111751
0.1629	8302.28949	0.76676212	161.27947	1.53061405	0	-1.8146188	2.11665176	-0.2642753	5.0831449	0.42632211	-6.9077553	2	84	3.57631111	300.777778	27.7777778	0	0	0	0	42.288408	2	-0.4111751
0.3708	16498.3408	0.91613532	237.595221	2.49849235	0	-0.9920924	2.80332043	-0.0865003	5.47057268	0.91608765	-6.9077553	3	78	3.57631111	301.888889	28.88888888	0	0	0	0	42.288408	2	-0.4111751
0.6381	28695.5289	0.95643713	249.526121	2.27134956	0	-0.4492603	3.35677617	-0.0434952	5.51956761	0.82081435	-6.9077553	4	76	4.02335	301.888889	28.88888888	0	0	0	0	42.288408	2	-0.4111751
0.936	40237.2054	0.84510448	190.492742	1.72784627	0	-0.0661398	3.69481693	-0.1671124	5.24961934	0.54745429	-6.9077553	5	66	4.02335	304.111111	31.11111111	0	0	1	0	42.288408	2	-0.4111751
1.2915	54672.5697	0.86957158	203.359375	1.6639633	0	0.25580433	4.00138041	-0.1386053	5.31497965	0.50980308	-6.9077553	6	63	3.57631111	304.666667	31.66666666	0	0	1	0	42.288408	2	-0.4111751
1.8063	74138.2548	0.91846898	234.053486	2.45467307	0	0.59128055	4.30594515	-0.083959	5.45555393	0.89840089	-6.9077553	7	57	5.81150556	305.777778	32.7777778	0	0	1	0	42.288408	2	-0.4111751
2.349	91133.8316	0.83683319	189.840913	2.19487483	0	0.85398971	4.51234008	-0.1769363	5.24619169	0.78658052	-6.9077553	8	56	5.36446667	305.777778	32.7777778	0	0	1	0	42.288408	2	-0.4111751
3.042	124124.725	1.01599787	297.448397	2.54123947	0	1.11251519	4.82129496	0.01685502	5.69524412	0.93304538	-6.9077553	9	55	5.81150556	306.333333	33.33333333	0	0	1	0	42.288408	2	-0.4111751
3.7494	158922.152	0.99461617	276.607944	2.11958759	0	1.32159583	5.06842077	-0.0043935	5.62260475	0.75169322	-6.9077553	10	59	3.12927222	305.777778	32.7777778	0	0	1	0	42.288408	2	-0.4111751
4.428	191883.01	0.97059109	265.515045	2.06221328	0	1.48794801	5.25689107	-0.0288202	5.58167527	0.72426461	-6.9077553	11	59	3.12927222	305.222222	32.2222222	0	0	1	0	42.288408	2	-0.4111751
4.9338	214627.233	0.8913496	222.940127	1.64891742	0	1.59610948	5.36890738	-0.1138973	5.40690773	0.50072524	-6.9077553	12	65	1.78815556	304.666667	31.66666666	0	0	1	0	42.288408	2	-0.4111751
5.2146	226858.277	0.8323601	186.341271	1.36943719	0	1.65146238	5.4243299	-0.1822894	5.22758515	0.31512981	-6.9077553	13	70	1.34111667	304.111111	31.11111111	0	0	1	0	42.288408	2	-0.4111751
5.4108	235331.926	0.70323027	131.351661	1.15197222	0	1.68839696	5.46100122	-0.3506499	4.87788578	0.14234315	-6.9077553	14	73	1.78815556	303	30	0	0	1	0	42.288408	2	-0.4111751
5.5341	240595.557	0.46791455	54.9522197	0.77081549	0	1.71092895	5.48312149	-0.7573347	4.00648227	-0.2590098	-6.9077553	15	76	2.68223333	301.888889	28.88888888	0	0	1	0	42.288408	2	-0.4111751
5.6088	243709.892	0.32883777	28.3423205	0.54382076	0	1.72433679	5.49598265	-1.1091543	3.34439139	-0.6072984	-6.9077553	16	80	2.23519444	301.333333	28.33333333	0	0	1	0	42.288408	2	-0.4111751
5.6727	246429.462	0.30741358	34.9893644	0.62151541	0	1.73566519	5.50707985	-1.1763136	3.55507272	-0.4739869	-6.9077553	17	81	2.68223333	301.333333	28.33333333	0	0	1	0	42.288408	2	-0.4111751
5.7267	248534.931	0.24807506	13.9777944	0.34740047	0	1.74513945	5.51558743	-1.390001	2.6375415	-1.0544027	-6.9077553	18	83	2.68223333	300.777778	27.7777778	0	0	1	0	42.288408	2	-0.4111751
5.7717	250377.197	0.2269721	10.9105176	0.32591766	0	1.75296666	5.52297256	-1.478532	2.38981889	-1.1180469	-6.9077553	19	84	2.68223333	300.777778	27.7777778	0	0	1	0	42.288408	2	-0.4111751
5.8149	251824.697	0.22741588	10.9183795	0.31943425	0	1.76042359	5.52873717	-1.4765873	2.39053914	-1.1380782	-6.9077553	20	85	2.23519444	300.222222	27.2222222	0	0	1	0	42.288408	2	-0.4111751
5.8599	253798.567	0.20557766	9.1394751	0.31155365	0	1.76813254	5.53654485	-1.5770789	2.21271236	-1.1629791	-6.9077553	21	84	3.57631111	300.222222	27.2222222	0	0	1	0	42.288408	2	-0.4111751
5.8995	255509.271	0.21712392	11.8678284	0.31688554	0	1.7748676	5.54326261	-1.5226919	2.4739155	-1.1460639	-6.9077553	22	84	2.68223333	300.222222	27.2222222	0	0	1	0	42.288408	2	-0.4111751
5.9553	257526.989	0.35961782	62.5686114	0.77015932	0	1.78428158	5.55112841	-1.0199366	4.13627972	-0.2598603	-6.9077553	23	83	3.57631111	300.222222	27.2222222	0	0	1	0	42.288408	2	-0.4111751
0.0009	0	0	0	0	0	-7.0131158	-6.9077553	-6.9077553	-6.9077553	-6.9077553	-6.9077553	0	87	4.02335	300.222222	27.2222222	0	0	0	0	18.9135952	2	-0.0428739
0.0009	0	0	0	0	0	-7.0131158	-6.9077553	-6.9077553	-6.9077553	-6.9077553	-6.9077553	1	88	2.68223333	300.222222	27.2222222	0	0	0	0	18.9135952	2	-0.0428739
0.0009	0	0	0	0	0	-7.0131158	-6.9077553	-6.9077553	-6.9077553	-6.9077553	-6.9077553	2	84	3.57631111	300.777778	27.7777778	0	0	0	0	18.9135952	2	-0.0428739
0.0144	0	0.43947652	50.2750081	1.55957031	0	-4.2405271	-6.9077553	-0.8198981	3.91752799	0.44505134	-6.9077553	3	78	3.57631111	301.888889	28.88888888	0	0	0	0	18.9135952	2	-0.0428739
0.0657	235.701316	0.64792861	121.583179	2.11409813	0	-2.7226564	-1.4409562	-0.4324326	4.80060686	0.74910121	-6.9077553	4	76	4.02335	301.888889	28.88888888	0	0	0	0	18.9135952	2	-0.0428739
0.1755	1367.44981	0.63371727	111.442103	1.86960827	0	-1.7401162	0.31367857	-0.4545756	4.71351417	0.62626366	-6.9077553	5	66	4.02335	304.111111	31.11111111	0	0	1	0	18.9135952	2	-0.0428739
0.3447	6773.1009	0.80731753	213.79702	1.97072358	0	-1.0650808	1.91310665	-0.2128003	5.36503174	0.67890807	-6.9077553	6	63	3.57631111	304.666667	31.66666666	0	0	1	0	18.9135952	2	-0.0428739
0.621	16441.501	0.85476337	245.658464	2.530547	0	-0.4764242	2.79986951	-0.1557614	5.50394628	0.92883058	-6.9077553	7	57	5.81150556	305.777778	32.7777778	0	0	1	0	18.9135952	2	-0.0428739
0.9117	25684.5548	0.93262853	283.936436	2.80771205	0	-0.0924443	3.24592876	-0.0686766	5.64875392	1.03272603	-6.9077553	8	56	5.36446667	305.777778	32.7777778	0	0	1	0	18.9135952	2	-0.0428739
1.2555	36631.4485	0.86576612	228.775769	2.34321506	0	0.2275339	3.60093442	-0.1429861	5.43274672	0.85195062	-6.9077553	9	55	5.81150556	306.333333	33.33333333	0	0	1	0	18.9135952	2	-0.0428739
1.548	48558.3902	0.86304659	224.550096	1.80825871	0	0.43696378	3.88278759	-0.1461286	5.41410328	0.59291721	-6.9077553	10	59	3.12927222	305.777778	32.7777778	0	0	1	0	18.9135952	2	-0.0428739
1.8486	59504.3395	0.81805329	196.281952	1.692006	0	0.6144286	4.08606605	-0.1996061	5.27955725	0.52650565	-6.9077553	11	59	3.12927222	305.222222	32.2222222	0	0	1	0	18.9135952	2	-0.0428739
2.1708	72779.6772	0.85756372	210.341895	1.6581039	0	0.77509576	4.2874505	-0.1524944	5.34873903	0.50627764	-6.9077553	12	65	1.78815556	304.666667	31.66666666	0	0	1	0	18.9135952	2	-0.0428739
2.4093	84176.0421	0.8917773	214.464328	1.60030253	0	0.87933625	4.43292223	-0.1134181	5.36814808	0.47081738	-6.9077553	13	70	1.34111667	304.111111	31.11111111	0	0	1	0	18.9135952	2	-0.0428739
2.5668	91341.9412	0.76939287	156.512121	1.38966282	0	0.94265999	4.51462101	-0.2608547	5.05313984	0.32978049	-6.9077553	14	73	1.78815556	303	30	0	0	1	0	18.9135952	2	-0.0428739
2.6586	95509.0013	0.45062525	50.1510704	0.76917252	0	0.97779967	4.55923097	-0.7949025	3.9150598	-0.2611407	-6.9077553	15	76	2.68223333	301.888889	28.88888888	0	0	1	0	18.9135952	2	-0.0428739
2.7126	97921.5144	0.30141404	20.6760539	0.5410518	0	0.99790758	4.5841765	-1.1959582	3.02902458	-0.6123937	-6.9077553	16	80	2.23519444	301.333333	28.33333333	0	0	1	0	18.9135952	2	-0.0428739
2.7648	100246.29	0.25070357	14.1297881	0.46594534	0	1.0169683	4.60764003	-1.3795032	2.64835597	-0.7615431	-6.9077553	17	81	2.68223333	301.333333	28.33333333	0	0	1	0	18.9135952	2	-0.0428739
2.8107	102264.021	0.25926564	14.9057438	0.43321669	0	1.03343356	4.62756768	-1.3460525	2.70181371	-0.8342116	-6.9077553	18	83	2.68223333	300.777778	27.7777778	0	0	1	0	18.9135952	2	-0.0428739
2.8539	104237.895	0.25105995	13.6190796	0.38145359	0	1.04868648	4.64668534	-1.3780883	2.61154514	-0.961148	-6.9077553	19	84	2.68223333	300.777778	27.7777778	0	0	1	0	18.9135952	2	-0.0428739
2.889	105685.394	0.21127102	9.4848625	0.33372026	0	1.06091042	4.66047616	-1.5498914	2.24980253	-1.0944602	-6.9077553	20	85	2.23519444	300.222222	27.2222222	0	0	1	0	18.9135952	2	-0.0428739
2.9313	107483.818	0.21731442	10.1055064	0.32574031	0	1.07544601	4.67734961	-1.521819	2.31317942	-1.1185896	-6.9077553	21	84	3.57631111	300.222222	27.2222222	0	0	1	0	18.9135952	2	-0.0428739
2.9691	109150.637	0.23895352	12.121178	0.38612216	0	1.08825888	4.69273808	-1.42731	2.49503667	-0.949015	-6.9077553	22	84	2.68223333	300.222222	27.2222222	0	0	1	0	18.9135952	2	-0.0428739
3.0141	111212.235	0.25589451	15.4339429	0.40689213	0	1.10330128	4.7114494	-1.3590898	2.73663396	-0.8967525	-6.9077553	23	83	3.57631111	300.222222	27.2222222	0	0	1	0	18.9135952	2	-0.0428739
0.0108	407.833563	0.87600613	207.444403	1.51828818	0	-4.5282091	-0.8944471	-0.1312413	5.33486818	0.41824192	-6.9077553	0	70	1.78815556	303	30	0	0	0	0	30.519848	2	0.21141735
0.0216	892.584775	0.77328189	152.917745	1.30717095	0	-3.835062	-0.1125141	-0.2558193	5.0299067	0.26862994	-6.9077553	1	77	0.44703889	301.888889	28.88888888	0	0	0	0	30.519848	2	0.21141735
0.036	1243.49495	0.64217508	107.60556	1.31439948	0	-3.3242363	0.21872979	-0.4413383	4.67848162	0.27414041	-6.9077553	2	75	0.89407778	297.444444	24.44444444	0	0	0	0	30.519848	2	0.21141735
0.0396	1331.22246	0.36630318	35.1586533	0.95249152	0	-3.2289262	0.28684857	-1.0015677	3.55989921	-0.0476247	-6.9077553	3	88	0.89407778	295.222222	22.2222222	0	0	0	0	30.519848	2	0.21141735
0.0405	1375.08619	0.36421806	28.9067669	0.4240863	0	-3.2064533	0.31924337	-1.0072607	3.36411031	-0.8554631	-6.9077553	4	46	2.23519444	296.888889	23.88888888	0	0	0	0	30.519848	2	0.21141735
0.0864	1769.86033	0.35811772	41.5803653	1.17918845	0	-2.4487676	0.57146549	-1.024105	3.72765212	0.16567413	-6.9077553	5	42	2.23519444	296.333333	23.33333333	0	0	0	0	30.519848	2	0.21141735
0.153	2530.07615	0.43785647	56.8355878	1.280352	0	-1.8773174	0.92864457	-0.8235829	4.04018027	0.24791577	-6.9077553	6	35	3.12927222	295.777778	22.7777778	0	0	0	0	30.519848	2	0.21141735
0.3366	4012.60771	0.68269408	128.188189	3.0064434	0	-1.08886	1.38969051	-0.3802447	4.85350721	1.10109035	-6.9077553	7	36	4.91742778	294.666667	21.66666666	0	0	0	0	30.519848	2	0.21141735
0.4761	5692.02403	0.54373297	80.844966	1.38321878	0	-0.7421274	1.73924157	-0.6074596	4.39254569	0.32513592	-6.9077553	8	43	2.68223333	293	20	0	0	0	1	30.519848	2	0.21141735
0.5796	7281.85429	0.46496492	66.0068572	0.78080365	0	-0.5454171	1.98552286	-0.7636449	4.18977378	-0.2461517	-6.9077553	9	54	0.44703889	290.222222	17.2222222	0	0	0	1	30.519848	2	0.21141735
0.7029	9044.35618	0.50596808	74.5976277	0.90829963	0	-0.3525406	2.2022515	-0.6793072	4.31212211	-0.0950806	-6.9077553	10	55	1.34111667	291.333333	18.33333333	0	0	0	1	30.519848	2	0.21141735
1.0008	11973.9104	0.62944607	108.847302	1.9117522	0	0.00079968	2.48281366	-0.4613277	4.68995519	0.64854315	-6.9077553	11	46	3.12927222	294.111111	21.11111111	0	0	0	1	30.519848	2	0.21141735

1.7541	19608.7416	0.97320433	295.760715	4.05643061	0	0.5619559	2.97602646	-0.0261342	5.68955411	1.40054992	-6.9077553	12	40	5.36446667	295.777778	22.7777778	0	0	0	1	30.519848	2	0.21141735
2.7954	30055.29	0.99645859	299.281061	4.4114105	0	1.02797521	3.40307196	-0.0025446	5.70138648	1.48442114	-6.9077553	13	35	6.25854444	297.444444	24.44444444	0	0	0	1	30.519848	2	0.21141735
3.6801	48918.1558	0.95848454	264.968527	2.39293974	0	1.30293993	3.89016905	-0.0413591	5.57961483	0.87294044	-6.9077553	14	31	6.70558333	299.111111	26.11111111	0	0	0	1	30.519848	2	0.21141735
4.6314	63929.6461	1.00972798	294.49929	2.45263556	0	1.5328592	4.15779884	0.01067084	5.68527999	0.89757083	-6.9077553	15	27	6.25854444	300.777778	27.7777778	0	0	0	1	30.519848	2	0.21141735
5.796	140898.653	1.21562777	426.405719	3.23506856	57	1.75716802	4.94804795	0.19608291	6.05539363	1.17435918	4.04306881	16	24	7.59966111	301.888889	28.88888888	0	0	0	1	30.519848	2	0.21141735
6.6195	247675.547	1.32941574	487.472268	2.59676213	151	1.89001984	5.51212365	0.28549148	6.18923545	0.95465036	5.01728646	17	23	5.36446667	303.555556	30.55555555	0	0	0	1	30.519848	2	0.21141735
7.8075	643491.468	2.16530725	1031.58116	3.34628832	505	2.05508481	6.46691032	0.77302399	6.93884898	1.20815056	6.22456041	18	23	5.81150556	303.555556	30.55555555	0	0	0	1	30.519848	2	0.21141735
9.4806	1244820.24	2.28947435	1101.0667	4.12967715	1067	2.24924761	7.12674722	0.82875893	7.00403563	1.41844135	6.97260719	19	22	6.25854444	304.111111	31.11111111	0	0	0	1	30.519848	2	0.21141735
11.5461	1982377.46	2.7192719	1438.89281	4.79605758	1829	2.44634772	7.59205264	1.00073184	7.27162991	1.56800273	7.5115252	20	23	5.36446667	304.111111	31.11111111	0	0	0	1	30.519848	2	0.21141735
13.0095	2285055.62	1.80993217	834.195341	3.5575244	2120	2.56567986	7.73414608	0.59384172	6.7264688	1.26934596	7.65917184	21	24	4.91742778	303.555556	30.55555555	0	0	0	1	30.519848	2	0.21141735
13.9347	2409415.43	1.30324059	485.343203	2.21738538	2217	2.63438213	7.78713985	0.26562095	6.18485834	0.79677962	7.70391066	22	27	3.57631111	302.444444	29.44444444	0	0	0	1	30.519848	2	0.21141735
14.6709	2607682.91	1.81134794	784.550275	2.61841895	2394	2.68586594	7.86621772	0.59462321	6.66511193	0.96295252	7.7807213	23	46	0.44703889	299.111111	26.11111111	0	0	0	1	30.519848	2	0.21141735
0.0198	933.956126	1.00227383	281.894322	1.98537609	0	-3.9220733	-0.0672557	0.00326848	5.6415358	0.68631192	-6.9077553	0	70	1.78815556	303	30	0	0	0	0	34.0724638	2	0.49277013
0.0342	1372.59408	0.671597	112.448723	0.93781799	0	-3.3755296	0.31743072	-0.3966089	4.72250621	-0.0631337	-6.9077553	1	77	0.44703889	301.888889	28.88888888	0	0	0	0	34.0724638	2	0.49277013
0.0477	1898.95937	0.45267967	49.750765	0.65515156	0	-3.0428239	0.6418325	-0.7903639	3.90704594	-0.4213635	-6.9077553	2	75	0.89407778	297.444444	24.44444444	0	0	0	0	34.0724638	2	0.49277013
0.0621	2425.3248	0.52781785	70.9785614	0.84093446	0	-2.7790093	0.88637769	-0.6371112	4.26239197	-0.1720531	-6.9077553	3	88	0.89407778	295.222222	22.2222222	0	0	0	0	34.0724638	2	0.49277013
0.0738	2907.82637	0.35991005	31.5534198	0.45926813	0	-2.6063965	1.06774969	-1.0191265	3.45171367	-0.7759461	-6.9077553	4	46	2.23519444	296.888889	23.88888888	0	0	0	0	34.0724638	2	0.49277013
0.1476	6302.75211	0.88482387	221.5126	1.66953166	0	-1.9132494	1.84114503	-0.1212371	5.40048399	0.51314193	-6.9077553	5	42	2.23519444	296.333333	23.33333333	0	0	0	0	34.0724638	2	0.49277013
0.2151	9570.1378	0.92725525	227.907161	1.50069173	0	-1.5366522	2.25875209	-0.0744485	5.42894275	0.40659229	-6.9077553	6	35	3.12927222	295.777778	22.7777778	0	0	0	0	34.0724638	2	0.49277013
0.3096	12508.4634	0.88265773	207.53417	2.22488273	0	-1.1724741	2.52648543	-0.1236855	5.33530082	0.80015357	-6.9077553	7	36	4.91742778	294.666667	21.66666666	0	0	0	1	34.0724638	2	0.49277013
0.4221	17657.565	0.98621405	272.542094	2.01791565	0	-0.862513	2.87122094	-0.0128684	5.60779674	0.70256056	-6.9077553	8	43	2.68223333	293	20	0	0	0	1	34.0724638	2	0.49277013
0.5625	24717.8629	0.96772424	253.834385	1.76451698	0	-0.5753641	3.20756663	-0.0317753	5.53668597	0.56844355	-6.9077553	9	54	0.44703889	290.222222	17.2222222	0	0	0	1	34.0724638	2	0.49277013
0.7704	34390.1665	0.95901714	257.415314	1.86395964	0	-0.2608454	3.53779974	-0.0408041	5.55069467	0.62323941	-6.9077553	10	55	1.34111667	291.333333	18.33333333	0	0	0	1	34.0724638	2	0.49277013
1.0476	44334.9818	0.91690698	235.138647	1.98673833	0	0.04650183	3.79179658	-0.0856592	5.46017958	0.68699747	-6.9077553	11	46	3.12927222	294.111111	21.11111111	0	0	0	1	34.0724638	2	0.49277013
1.395	55095.755	0.97842344	271.373988	2.71116867	0	0.33289442	4.00909082	-0.0207912	5.60350158	0.99774856	-6.9077553	12	40	5.36446667	295.777778	22.7777778	0	0	0	1	34.0724638	2	0.49277013
1.9728	77606.1874	1.10129298	357.152599	3.05085103	0	0.67945385	4.35166004	0.09739253	5.87816594	1.1157483	-6.9077553	13	35	6.25854444	297.444444	24.44444444	0	0	0	1	34.0724638	2	0.49277013
2.7135	108377.947	1.06368236	320.738799	2.63577096	0	0.99823931	4.68563385	0.0626765	5.7706302	0.96955505	-6.9077553	14	31	6.70558333	299.111111	26.11111111	0	0	0	1	34.0724638	2	0.49277013
3.6639	137987.664	1.07574995	334.944188	3.38776398	0	1.29852815	4.92717154	0.0739472	5.8139669	1.22046525	-6.9077553	15	27	6.25854444	300.777778	27.7777778	0	0	0	1	34.0724638	2	0.49277013
4.7574	185355.383	1.15514584	378.530556	3.01963453	0	1.5597013	5.22228037	0.14509192	5.93629944	1.10546692	-6.9077553	16	24	7.59966111	301.888889	28.88888888	0	0	0	1	34.0724638	2	0.49277013
5.9778	236065.763	1.14158284	372.668326	3.06575053	0	1.78805261	5.46411466	0.13329135	5.9206915	1.12061854	-6.9077553	17	23	5.36446667	303.555556	30.55555555	0	0	0	1	34.0724638	2	0.49277013
7.4178	292552.439	1.13498474	379.738789	3.68728699	0	2.00388252	5.67864735	0.12749989	5.93948625	1.30516212	-6.9077553	18	23	5.81150556	303.555556	30.55555555	0	0	0	1	34.0724638	2	0.49277013
9.1224	343133.299	0.98308056	298.685157	3.52817594	0	2.21073293	5.83812191	-0.0160475	5.69939338	1.2610644	-6.9077553	19	22	6.25854444	304.111111	31.11111111	0	0	0	1	34.0724638	2	0.49277013
10.5012	373526.124	0.84137369	217.915858	3.1061489	0	2.35148954	5.92299062	-0.1715315	5.3841136	1.13370555	-6.9077553	20	23	5.36446667	304.111111	31.11111111	0	0	0	1	34.0724638	2	0.49277013
11.9772	411847.909	0.88250948	245.825915	3.01921625	0	2.48300484	6.02065656	-0.1238533	5.50462769	1.10532843	-6.9077553	21	24	4.91742778	303.555556	30.55555555	0	0	0	1	34.0724638	2	0.49277013
13.2525	450249.311	0.82376527	216.74592	2.37122159	0	2.58418621	6.10980368	-0.1926565	5.37873041	0.8638269	-6.9077553	22	27	3.57631111	302.444444	29.44444444	0	0	0	1	34.0724638	2	0.49277013
14.3469	484385.422	0.80166676	201.133147	1.91018436	0	2.66353389	6.18288298	-0.2198156	5.30397208	0.64772313	-6.9077553	23	46	0.44703889	299.111111	26.11111111	0	0	0	1	34.0724638	2	0.49277013
0.0324	2061.20383	1.13287495	351.612109	2.306553	0	-3.4295969	0.72377523	0.12564093	5.86253145	0.83618765	-6.9077553	0	70	1.78815556	303	30	0	0	0	0	42.288408	2	-0.4111751
0.0954	5147.98651	0.85833201	196.290946	1.54285529	0	-2.3496767	1.6387999	-0.1515999	5.27960307	0.43428272	-6.9077553	1	77	0.44703889	301.888889	28.88888888	0	0	0	0	42.288408	2	-0.4111751
0.153	7867.54104	0.62193888	96.7172089	1.08109283	0	-1.8773174	2.06287266	-0.4733069	4.57180169	0.07889698	-6.9077553	2	75	0.89407778	297.444444	24.44444444	0	0	0	0	42.288408	2	-0.4111751
0.2088	10543.2312	0.62398086	98.6443344	1.06490178	0	-1.5663784	2.3555789	-0.4700343	4.59153094	0.06382119	-6.9077553	3	88	0.89407778	295.222222	22.2222222	0	0	0	0	42.288408	2	-0.4111751
0.2448	12305.5578	0.46117182	64.9843079	0.81490822	0	-1.4073137	2.51013228	-0.7718186	4.17416121	-0.2034534	-6.9077553	4	46	2.23519444	296.888889	23.88888888	0	0	0	0	42.288408	2	-0.4111751
0.4572	22507.4014	0.93551261	258.561838	1.89925165	0	-0.7826343	3.11388864	-0.0655923	5.55513875	0.64198632	-6.9077553	5	42	2.23519444	296.333333	23.33333333	0	0	0	0	42.288408	2	-0.4111751
0.7776	37693.9948	0.99266164	283.040072	2.13366528	0	-0.251543	3.62952732	-0.0063585	5.64559202	0.75830986	-6.9077553	6	35	3.12927222	295.777778	22.7777778	0	0	1	0	42.288408	2	-0.4111751
1.1862	52695.2228	0.91567449	233.433697	2.37615617	0	0.17075492	3.96454378	-0.0870028	5.45290237	0.86590489	-6.9077553	7	36	4.91742778	294.666667	21.66666666	0	0	1	0	42.288408	2	-0.4111751
1.5003	64915.9454	0.80645577	174.664107	1.37844053	0	0.40566509	4.17310869	-0.213867	5.16287047	0.321678	-6.9077553	8	43	2.68223333	293	20	0	0	1	0	42.288408	2	-0.4111751
1.8342	78303.2902	0.81332776	181.647869	1.39476075	0	0.60660842	4.36060239	-0.2053923	5.20207553	0.33343961	-6.9077553	9	54	0.44703889	290.222222	17.2222222	0	0	1	0	42.288408	2	-0.4111751
2.1402	90104.6089	0.74695206	154.079733	1.21749995	0	0.76089928	4.50098242	-0.2904164	5.03747671	0.19762055	-6.9077553	10	55	1.34111667	291.333333	18.33333333	0	0	1	0	42.288408	2	-0.4111751
2.6568	106696.022	0.78134485	170.787838	1.89146928	0	0.97712239	4.66999325	-0.2454597	5.14042793	0.63788248	-6.9077553	11	46	3.12927222	294.111111	21.11111111	0	0	1	0	42.288408	2	-0.4111751
3.6261	134865.325	0.90389417	241.576718	3.26862133	0	1.28815769	4.9042841	-0.0999373	5.48719123	1.18467418	-6.9077553	12	40	5.36446667	295.777778	22.7777778	0	0	1	0	42.288408	2	-0.4111751
5.0247	165431.464	0.8454626	216.640384	3.77097033	0	1.61436575	5.10856304	-0.1666893	5.37824338	1.3275975	-6.9077553	13	35	6.25854444	297.444444	24.44444444	0	0	1	0	42.288408	2	-0.4111751
6.8292	216035.92	0.99979251	316.553086	4.51081379	0	1.92120754	5.37544932	0.0007922	5.75749411	1.50669924	-6.9077553	14	31	6.70558333	299.111111	26.11111111	0	0	1	0	42.288408	2	-0.4111751
8.2098	272077.866	1.14727314	400.754441	4.36341971	0	2.10532856	5.60609197	0.13825919	5.99335137	1.47348524	-6.9077553	15	27	6.25854444	300.777778	27.7777778	0	0	1	0	42.288408	2	-0.4111751
8.2098	272077.866	0	0	0	0	2.10532856	5.60609197	-6.9077553	-6.9077553	-6.9077553	-6.9077553	16	24	7.59966111	301.888889	28.88888888	0	0	1	0	42.288408	2	-0.4111751
8.2098	272077.866	0	0	0	0	2.10532856	5.60609197	-6.9077553	-6.9077553	-6.9077553	-6.9077553	17	23	5.36446667	303.555556	30.55555555	0	0	1	0	42.288408	2	-0.4111751
8.2098	272077.866	0	0	0	0	2.10532856	5.60609197	-6.9077553	-6.9077553	-6.9077553	-6.9077553	18	23	5.81150556	303.555556	30.55555555	0	0	1	0	42.288408	2	-0.4111751
8.2098	272077.866	0	0	0	0	2.10532856	5.60609197	-6.9077553	-6.9077553	-6.9077553	-6.9077553	19	22	6.25854444	304.111111	31.11111111	0	0	1	0	42.288408	2	-0.4111751
8.2098	272077.866	0	0	0	0	2.10532856	5.60609197	-6.9077553	-6.9077553	-6.9077553	-6.9077553	20	23	5.36446667	304.111111	31.11111111	0	0	1	0	42.288408	2	-0.4111751
8.2098	272077.866	0	0	0	0	2.10532856	5.60609197	-6.9077553	-6.9077553	-6.9077553	-6.9077553	21	24	4.91742778	303.555556	30.55555555	0	0	1	0	42.288408	2	-0.4111751
8.2098	272077.866	0	0	0	0	2.10532856	5.60609197	-6.9077553	-6.9077553	-6.9077553	-6.9077553	22	27	3.57631111	302.444444	29.44444444	0	0	1	0	42.288408	2	-0.4111751
8.2098	272077.866	0	0	0	0	2.10532856	5.60609197	-6.9077553	-6.9077553	-6.9077553	-6.9077553	23	46	0.44703889	299.111111	26.11111111	0	0	1	0	42.288408	2	-0.4111751
0.0018	0	0.1061632	1.98204601	0.29212055	0	-6.3199686	-6.9077553	-2.2334023	0.68463405	-1.2271713	-6.9077553	0	70	1.78815556	303	30	0	0	0	0	18.9135952	2	-0.0428739
0.0018	0	0	0	0	0	-6.3199686	-6.9077553	-6.9077553	-6.9077553	-6.9077553	-6.9077553	1	77	0.44703889	301.888889	28.88888888	0	0	0	0	18.9135952	2	-0.0428739

0.0018	0	0	0	0	0	-6.3199686	-6.9077553	-6.9077553	-6.9077553	-6.9077553	-6.9077553	2	75	0.89407778	297.444444	24.44444444	0	0	0	0	18.9135952	2	-0.0428739
0.0018	0	0	0	0	0	-6.3199686	-6.9077553	-6.9077553	-6.9077553	-6.9077553	-6.9077553	3	88	0.89407778	295.222222	22.2222222	0	0	0	0	18.9135952	2	-0.0428739
0.0018	0	0	0	0	0	-6.3199686	-6.9077553	-6.9077553	-6.9077553	-6.9077553	-6.9077553	4	46	2.23519444	296.888889	23.88888888	0	0	0	0	18.9135952	2	-0.0428739
0.0171	0	0.36484831	37.8692663	1.41049351	0	-4.0686768	-6.9077553	-1.0055365	3.63416627	0.34464837	-6.9077553	5	42	2.23519444	296.333333	23.33333333	0	0	0	0	18.9135952	2	-0.0428739
0.0972	0	0.47608151	57.4275259	2.00378418	0	-2.3309846	-6.9077553	-0.7400679	4.05054115	0.69553641	-6.9077553	6	35	3.12927222	295.777778	22.7777778	0	0	0	0	18.9135952	2	-0.0428739
0.2178	131.59135	0.39153358	39.3018744	1.71053758	0	-1.5241781	-2.0204834	-0.9351332	3.67129766	0.53739214	-6.9077553	7	36	4.91742778	294.666667	21.66666666	0	0	1	0	18.9135952	2	-0.0428739
0.3141	614.113875	0.37969044	46.9239285	1.18274461	0	-1.1580439	-0.4859479	-0.9657687	3.84854906	0.16868281	-6.9077553	8	43	2.68223333	293	20	0	0	1	0	18.9135952	2	-0.0428739
0.3735	1328.67717	0.42007764	67.8017726	0.77960633	0	-0.9848373	0.28493618	-0.864938	4.21660309	-0.2476843	-6.9077553	9	54	0.44703889	290.222222	17.2222222	0	0	1	0	18.9135952	2	-0.0428739
0.4527	3379.40351	0.544938	110.009588	0.98961189	0	-0.7925256	1.21799508	-0.6052499	4.70057662	-0.0094325	-6.9077553	10	55	1.34111667	291.333333	18.33333333	0	0	1	0	18.9135952	2	-0.0428739
0.6723	6313.12065	0.61430421	128.881236	1.88308991	0	-0.3970506	1.8427885	-0.4856385	4.85889909	0.6334449	-6.9077553	11	46	3.12927222	294.111111	21.11111111	0	0	1	0	18.9135952	2	-0.0428739
1.1124	14015.081	0.83093062	232.334899	3.39625971	0	0.10651984	2.64020532	-0.1840062	5.44818417	1.22296914	-6.9077553	12	40	5.36446667	295.777778	22.7777778	0	0	1	0	18.9135952	2	-0.0428739
1.7424	31360.0591	0.96824847	293.151406	4.36846749	0	0.55526347	3.44556697	-0.0312343	5.68069263	1.47464115	-6.9077553	13	35	6.25854444	297.444444	24.44444444	0	0	1	0	18.9135952	2	-0.0428739
2.5407	65576.5072	1.09766231	387.880778	4.06761815	0	0.93243963	4.18323276	0.09409335	5.9607006	1.40330342	-6.9077553	14	31	6.70558333	299.111111	26.11111111	0	0	1	0	18.9135952	2	-0.0428739
3.2319	95020.4668	1.03670899	327.924642	2.95615323	0	1.1730702	4.55410283	0.03701539	5.79278688	1.08422706	-6.9077553	15	27	6.25854444	300.777778	27.7777778	0	0	1	0	18.9135952	2	-0.0428739
4.2363	143616.102	1.21429947	463.583473	4.29032521	0	1.44369025	4.96715074	0.19499052	6.13898862	1.45659559	-6.9077553	16	24	7.59966111	301.888889	28.88888888	0	0	1	0	18.9135952	2	-0.0428739
5.211	189698.363	1.09606223	356.340432	2.7932724	1	1.65077178	5.24544052	0.09263591	5.87588935	1.02757175	0.0009995	17	23	5.36446667	303.555556	30.55555555	0	0	1	0	18.9135952	2	-0.0428739
6.1506	234393.176	1.06651429	341.913494	2.92248067	1	1.81654964	5.45700421	0.06533285	5.83456069	1.07277492	0.0009995	18	23	5.81150556	303.555556	30.55555555	0	0	1	0	18.9135952	2	-0.0428739
7.1865	281021.926	1.06488891	345.353551	3.01479437	1	1.97220427	5.63843625	0.06380911	5.84457157	1.10386327	0.0009995	19	22	6.25854444	304.111111	31.11111111	0	0	1	0	18.9135952	2	-0.0428739
8.1576	324439.106	1.04592808	326.218026	2.6887293	1	2.09895001	5.78210095	0.04586024	5.78756901	0.98944056	0.0009995	20	23	5.36446667	304.111111	31.11111111	0	0	1	0	18.9135952	2	-0.0428739
9.1746	385073.724	1.06806986	329.965155	2.42147055	16	2.2164388	5.9534374	0.06678898	5.79899009	0.88478791	2.77265122	21	24	4.91742778	303.555556	30.55555555	0	0	1	0	18.9135952	2	-0.0428739
10.1709	479812.028	1.09920369	341.16932	2.04195889	65	2.3195307	6.1733965	0.09549533	5.83238183	0.71439919	4.17440265	22	27	3.57631111	302.444444	29.44444444	0	0	1	0	18.9135952	2	-0.0428739
11.3058	667906.551	1.23787099	412.804718	2.09572713	185	2.42531587	6.50414977	0.21420048	6.02297707	0.74037762	5.22036123	23	46	0.44703889	299.111111	26.11111111	0	0	1	0	18.9135952	2	-0.0428739
0.0135	363.345551	0.82172203	183.737671	1.70242105	0	-4.3050656	-1.0096525	-0.1951369	5.21351448	0.53263861	-6.9077553	0	89	3.57631111	291.888889	18.88888888	0	0	0	0	30.519848	2	0.21141735
0.0549	1073.24541	0.50249639	71.0396941	1.73632978	0	-2.9022419	0.07161847	-0.6861787	4.26325287	0.55234933	-6.9077553	1	79	5.36446667	294.111111	21.11111111	0	0	0	0	30.519848	2	0.21141735
0.1629	1888.00992	0.56221682	92.6103516	2.40031738	0	-1.8146188	0.63605284	-0.5740906	4.52841172	0.8760175	-6.9077553	2	73	6.70558333	295.222222	22.2222222	0	0	0	0	30.519848	2	0.21141735
0.2628	2816.47283	0.59561975	100.500132	2.06177239	0	-1.336362	1.03584032	-0.5164753	4.61016899	0.7240509	-6.9077553	3	66	4.91742778	297.444444	24.44444444	0	0	0	0	30.519848	2	0.21141735
0.3726	3847.81876	0.61874709	102.812612	1.93900949	0	-0.9872498	1.34776629	-0.4784438	4.63291776	0.66269287	-6.9077553	4	64	5.36446667	298	25	0	0	0	0	30.519848	2	0.21141735
0.5175	4960.6502	0.61326433	98.2317547	1.77602259	0	-0.6587458	1.60173839	-0.4873299	4.58733971	0.57493926	-6.9077553	5	59	4.91742778	299.111111	26.11111111	0	0	0	1	30.519848	2	0.21141735
0.7497	8003.49356	0.69937282	137.370882	2.33963604	0	-0.2880822	2.08000308	-0.3561425	4.92269171	0.8504227	-6.9077553	6	53	6.25854444	300.222222	27.2222222	0	0	0	1	30.519848	2	0.21141735
1.0089	12957.2757	0.75708146	168.521457	2.47398504	0	0.00886063	2.56173464	-0.2769644	5.12706902	0.90623435	-6.9077553	7	50	6.25854444	300.777778	27.7777778	0	0	0	1	30.519848	2	0.21141735
1.2105	17146.0549	0.67741333	124.307826	1.49886853	0	0.1910335	2.84182643	-0.3879985	4.82276901	0.40537746	-6.9077553	8	53	4.91742778	300.222222	27.2222222	0	0	0	1	30.519848	2	0.21141735
1.413	20747.395	0.65775133	132.287865	1.30623915	0	0.3457151	3.03246889	-0.4174092	4.8849879	0.26791739	-6.9077553	9	53	4.91742778	299.666667	26.66666666	0	0	0	1	30.519848	2	0.21141735
1.5399	23621.9397	0.59771923	109.386857	0.9098251	0	0.43171748	3.16221826	-0.5129625	4.69489989	-0.0934044	-6.9077553	10	57	3.57631111	298	25	0	0	0	1	30.519848	2	0.21141735
1.5993	25870.7667	0.77396289	163.686849	1.16840201	0	0.46956603	3.25315229	-0.2549401	5.09796125	0.15649251	-6.9077553	11	67	1.78815556	295.777778	22.7777778	0	0	0	1	30.519848	2	0.21141735
1.6668	28708.2798	0.78433751	162.377734	1.24106873	0	0.51090562	3.35722041	-0.2416417	5.08993147	0.21677832	-6.9077553	12	70	1.78815556	295.222222	22.2222222	0	0	0	1	30.519848	2	0.21141735
1.737	31905.368	0.7683551	153.93283	1.33086728	0	0.55215949	3.46280561	-0.2622026	5.03652283	0.28658193	-6.9077553	13	75	1.78815556	294.111111	21.11111111	0	0	0	1	30.519848	2	0.21141735
1.7937	34493.3287	0.60435528	92.4859018	0.99924493	0	0.58428053	3.54079492	-0.5019397	4.52706703	0.0002449	-6.9077553	14	79	2.23519444	293.555556	20.55555555	0	0	0	1	30.519848	2	0.21141735
1.8153	35326.7412	0.27437548	16.3546982	0.48375678	0	0.59625074	3.56466852	-1.2896197	2.79457635	-0.724108	-6.9077553	15	83	3.12927222	293	20	0	0	0	1	30.519848	2	0.21141735
1.8315	36072.4235	0.19852671	7.94125705	0.25433114	0	0.6051353	3.5855564	-1.6118071	2.0721975	-1.365194	-6.9077553	16	87	3.57631111	293	20	0	0	0	1	30.519848	2	0.21141735
1.8459	36730.3831	0.18230669	6.64858627	0.22904648	0	0.61296696	3.60363152	-1.6965946	1.89455464	-1.4694739	-6.9077553	17	91	3.57631111	292.444444	19.44444444	0	0	0	1	30.519848	2	0.21141735
1.8576	37212.8817	0.15380907	5.01778999	0.24902657	0	0.61928533	3.61668186	-1.8655627	1.61318887	-1.3861881	-6.9077553	18	94	3.12927222	291.888889	18.88888888	0	0	0	1	30.519848	2	0.21141735
1.8684	37563.7931	0.15700018	4.89967314	0.21562725	0	0.62508245	3.62606726	-1.8451591	1.58937257	-1.5295771	-6.9077553	19	93	4.02335	291.888889	18.88888888	0	0	0	1	30.519848	2	0.21141735
1.8783	38002.4327	0.15275585	4.50664902	0.18211291	0	0.63036711	3.63767649	-1.8723894	1.50577574	-1.6976523	-6.9077553	20	90	4.91742778	291.888889	18.88888888	0	0	0	1	30.519848	2	0.21141735
1.8873	38397.2035	0.13898716	3.79891815	0.17784632	0	0.63514724	3.64801068	-1.9662046	1.33497953	-1.7212284	-6.9077553	21	90	3.57631111	291.888889	18.88888888	0	0	0	1	30.519848	2	0.21141735
1.8936	38616.523	0.12386157	3.06545067	0.14988072	0	0.63847978	3.65370614	-2.0805496	1.12052076	-1.8912657	-6.9077553	22	89	4.47038889	291.888889	18.88888888	0	0	0	1	30.519848	2	0.21141735
1.9053	39099.0273	0.14276925	3.98232475	0.18602179	0	0.64463948	3.66612316	-1.9395457	1.38211683	-1.6765302	-6.9077553	23	90	4.47038889	291.888889	18.88888888	0	0	0	1	30.519848	2	0.21141735
0.0234	904.932528	0.97344139	267.442949	2.41171555	0	-3.7550193	-0.0987904	-0.0258909	5.58891001	0.8807529	-6.9077553	0	89	3.57631111	291.888889	18.88888888	0	0	0	0	34.0724638	2	0.49277013
0.0612	2260.04364	0.75440579	164.630034	1.38648233	0	-2.7936081	0.8158265	-0.2805002	5.10370681	0.32749083	-6.9077553	1	79	5.36446667	294.111111	21.11111111	0	0	0	0	34.0724638	2	0.49277013
0.1494	6405.54535	0.91808996	224.041494	1.7128503	0	-1.901128	1.85732018	-0.0843713	5.41183574	0.53874248	-6.9077553	2	73	6.70558333	295.222222	22.2222222	0	0	0	0	34.0724638	2	0.49277013
0.2538	10169.3189	0.85895387	191.954438	1.86618029	0	-1.3712087	2.31947356	-0.1508765	5.25726325	0.62442943	-6.9077553	3	66	4.91742778	297.444444	24.44444444	0	0	0	1	34.0724638	2	0.49277013
0.4068	18827.5745	1.13055384	364.449173	2.99388859	0	-0.8994336	2.93537564	0.12359177	5.89838984	1.09690703	-6.9077553	4	64	5.36446667	298	25	0	0	0	1	34.0724638	2	0.49277013
0.7236	37409.0029	1.24274583	433.594549	4.08558135	0	-0.3235165	3.62193813	0.21812766	6.07211218	1.40770876	-6.9077553	5	59	4.91742778	299.111111	26.11111111	0	0	0	1	34.0724638	2	0.49277013
1.1043	53413.6829	1.19681587	425.50591	3.54877907	1	0.09921165	3.97808567	0.18049979	6.05328119	1.26688537	0.0009995	6	53	6.25854444	300.222222	27.2222222	0	0	0	1	34.0724638	2	0.49277013
1.7253	70303.7344	1.16380571	401.160462	3.88988267	1	0.54540095	4.25283914	0.1525543	5.99436399	1.35863604	0.0009995	7	50	6.25854444	300.777778	27.7777778	0	0	0	1	34.0724638	2	0.49277013
2.286	91287.0402	1.03288355	308.533909	2.39778228	1	0.82680357	4.51401978	0.03332215	5.73183499	0.87496122	0.0009995	8	53	4.91742778	300.222222	27.2222222	0	0	0	1	34.0724638	2	0.49277013
2.7621	108541.216	0.89538009	240.129091	2.28120431	1	1.01599126	4.68713918	-0.1093907	5.48118082	0.82514178	0.0009995	9	53	4.91742778	299.666667	26.66666666	0	0	0	1	34.0724638	2	0.49277013
3.0582	119779.89	0.84800706	225.317724	1.64021612	1	1.11782651	4.78566415	-0.1636878	5.41751595	0.4954375	0.0009995	10	57	3.57631111	298	25	0	0	0	1	34.0724638	2	0.49277013
3.2346	127672.171	0.82287473	182.242068	1.32775568	1	1.17390527	4.84947364	-0.1937368	5.20534133	0.28424292	0.0009995	11	67	1.78815556	295.777778	22.7777778	0	0	0	1	34.0724638	2	0.49277013
3.4119	135322.184	0.77616774	162.070511	1.3279783	1	1.22726932	4.90766587	-0.2520991	5.08803766	0.28441045	0.0009995	12	70	1.78815556	295.222222	22.2222222	0	0	0	1	34.0724638	2	0.49277013
3.5253	140165.19	0.75872343	152.381696	1.30536131	1	1.25996554	4.94282879	-0.2748008	5.0263951	0.26724565	0.0009995	13	75	1.78815556	294.111111	21.11111111	0	0	0	1	34.0724638	2	0.49277013
3.5829	142536.046	0.52316612	71.7017365	0.93187088	1	1.27617253	4.95960194	-0.6459466	4.27252891	-0.0694885	0.0009995	14	79	2.23519444	293.555556	20.55555555	0	0	0	1	34.0724638	2	0.49277013
3.6189	143501.034	0.39395437	55.0528564	0.67483654	1	1.28617011	4.96634921	-0.928985	4.00831191	-0.391804	0.0009995	15	83	3.12927222	293	20	0	0	0	1	34.0724638	2	0.49277013

3.6495	144378.312	0.32694065	47.1880385	0.73688389	1	1.29459017	4.97244395	-1.1149226	3.85416163	-0.3039688	0.0009995	16	87	3.57631111	293	20	0	0	0	1	34.0724638	2	0.49277013
3.6801	145211.738	0.32036361	42.3701639	0.81117944	1	1.30293993	4.97819982	-1.1351821	3.74646803	-0.208034	0.0009995	17	91	3.57631111	292.444444	19.44444444	0	0	0	1	34.0724638	2	0.49277013
3.7044	146264.464	0.18761621	10.0291635	0.28705138	1	1.3095213	4.98542322	-1.668041	2.3055969	-1.2446164	0.0009995	18	94	3.12927222	291.888889	18.88888888	0	0	0	1	34.0724638	2	0.49277013
3.7179	146834.692	0.15144374	4.39911804	0.19865003	1	1.31315899	4.98931422	-1.8809597	1.48163137	-1.6111893	0.0009995	19	93	4.02335	291.888889	18.88888888	0	0	0	1	34.0724638	2	0.49277013
3.7323	147361.052	0.14199831	3.95060539	0.1892924	1	1.31702467	4.9928925	-1.9449225	1.37412192	-1.6591934	0.0009995	20	90	4.91742778	291.888889	18.88888888	0	0	0	1	34.0724638	2	0.49277013
3.753	148194.467	0.13971573	3.69947914	0.1679499	1	1.32255552	4.99853212	-1.9610135	1.30846231	-1.778153	0.0009995	21	90	3.57631111	291.888889	18.88888888	0	0	0	1	34.0724638	2	0.49277013
3.7683	148676.969	0.12920178	3.32042313	0.4032802	1	1.32662397	5.00178269	-2.0386699	1.20039334	-0.9056471	0.0009995	22	89	4.47038889	291.888889	18.88888888	0	0	0	1	34.0724638	2	0.49277013
3.7845	149334.921	0.13817383	3.63821708	0.18336258	1	1.33091378	5.00619827	-1.9720315	1.29176857	-1.6908509	0.0009995	23	90	4.47038889	291.888889	18.88888888	0	0	0	1	34.0724638	2	0.49277013
0.0405	2732.46806	1.17396745	384.144132	2.55017801	0	-3.2064533	1.00557116	0.16124044	5.95102043	0.93655522	-6.9077553	0	89	3.57631111	291.888889	18.88888888	0	0	0	0	42.288408	2	-0.4111751
0.1638	7970.18882	1.01343809	292.231125	3.39103665	0	-1.8091091	2.07583364	0.01433486	5.67754843	1.22143052	-6.9077553	1	79	5.36446667	294.111111	21.11111111	0	0	0	0	42.288408	2	-0.4111751
0.4041	18294.5492	1.02074269	288.649813	2.80065392	0	-0.9060929	2.90665782	0.02150969	5.6652177	1.03020993	-6.9077553	2	73	6.70558333	295.222222	22.2222222	0	0	0	0	42.288408	2	-0.4111751
0.6849	29549.2076	0.92316378	225.819411	2.10100125	0	-0.3784824	3.38609077	-0.078866	5.41974004	0.74288987	-6.9077553	3	66	4.91742778	297.444444	24.44444444	0	0	0	0	42.288408	2	-0.4111751
1.0116	43204.0127	0.88659777	208.155884	1.81414206	0	0.01153324	3.76595652	-0.1192366	5.33829205	0.59616374	-6.9077553	4	64	5.36446667	298	25	0	0	0	0	42.288408	2	-0.4111751
1.5552	62479.0339	0.93826728	249.762676	2.86131297	0	0.44160415	4.13484705	-0.0626552	5.52051517	1.05163003	-6.9077553	5	59	4.91742778	299.111111	26.11111111	0	0	0	0	42.288408	2	-0.4111751
2.1213	87249.2405	0.94577565	252.355798	2.27366301	0	0.75202911	4.46878032	-0.0546931	5.53084395	0.82183191	-6.9077553	6	53	6.25854444	300.222222	27.2222222	0	0	1	0	42.288408	2	-0.4111751
2.6982	115302.845	0.97511394	267.16115	2.04626693	0	0.99258488	4.74757077	-0.024176	5.58785578	0.7165057	-6.9077553	7	50	6.25854444	300.777778	27.7777778	0	0	1	0	42.288408	2	-0.4111751
3.3075	140961.739	0.9268321	243.09645	2.14032568	0	1.19619262	4.94849559	-0.0749045	5.49346239	0.76142512	-6.9077553	8	53	4.91742778	300.222222	27.2222222	0	0	1	0	42.288408	2	-0.4111751
3.8115	163220.801	0.92537101	257.886384	2.00107487	0	1.33802281	5.09511002	-0.0764805	5.55252299	0.69418408	-6.9077553	9	53	4.91742778	299.666667	26.66666666	0	0	1	0	42.288408	2	-0.4111751
4.1976	181572.737	0.9066648	244.058202	1.76912207	0	1.43451293	5.20166183	-0.0968801	5.49741083	0.57104851	-6.9077553	10	57	3.57631111	298	25	0	0	1	0	42.288408	2	-0.4111751
4.4091	192087.363	0.86749397	209.417254	1.48558609	0	1.48367059	5.25795549	-0.1409946	5.34433347	0.39648228	-6.9077553	11	67	1.78815556	295.777778	22.7777778	0	0	1	0	42.288408	2	-0.4111751
4.5711	199409.154	0.81227247	176.653071	1.34190589	0	1.51975388	5.29536378	-0.2066891	5.17419342	0.29483584	-6.9077553	12	70	1.78815556	295.222222	22.2222222	0	0	1	0	42.288408	2	-0.4111751
4.7448	207182.537	0.76771632	156.086666	1.31734202	0	1.55704928	5.33360505	-0.2630333	5.05041781	0.2763749	-6.9077553	13	75	1.78815556	294.111111	21.11111111	0	0	1	0	42.288408	2	-0.4111751
4.8735	212665.524	0.59514004	91.1859703	1.0019894	0	1.58381236	5.35972532	-0.5172797	4.51291202	0.00298495	-6.9077553	14	79	2.23519444	293.555556	20.55555555	0	0	1	0	42.288408	2	-0.4111751
4.9302	214770.993	0.26801348	16.7458961	0.49289252	0	1.59537956	5.36957697	-1.3129938	2.81821293	-0.7054374	-6.9077553	15	83	3.12927222	293	20	0	0	1	0	42.288408	2	-0.4111751
4.9806	216964.172	0.20995779	9.14264951	0.31532131	0	1.60555037	5.37973684	-1.5560972	2.2130596	-1.1509968	-6.9077553	16	87	3.57631111	293	20	0	0	1	0	42.288408	2	-0.4111751
5.0247	218718.712	0.19605623	8.21779399	0.33908073	0	1.61436575	5.38779106	-1.6242662	2.10642348	-1.0785722	-6.9077553	17	91	3.57631111	292.444444	19.44444444	0	0	1	0	42.288408	2	-0.4111751
5.0733	220692.599	0.17370946	6.38122954	0.27570504	0	1.62399149	5.39677531	-1.7446309	1.85351749	-1.2848032	-6.9077553	18	94	3.12927222	291.888889	18.88888888	0	0	1	0	42.288408	2	-0.4111751
5.1084	222096.246	0.17678322	6.60052412	0.30099035	0	1.63088624	5.40311533	-1.7271903	1.88730055	-1.1973602	-6.9077553	19	93	4.02335	291.888889	18.88888888	0	0	1	0	42.288408	2	-0.4111751
5.1381	223368.29	0.1609681	5.52109504	0.29589956	0	1.63668336	5.40882641	-1.8203559	1.70875733	-1.2143614	-6.9077553	20	90	4.91742778	291.888889	18.88888888	0	0	1	0	42.288408	2	-0.4111751
5.1804	225210.556	0.16223884	5.367892	0.24662606	0	1.64488227	5.41704021	-1.8125409	1.68062156	-1.3958355	-6.9077553	21	90	3.57631111	291.888889	18.88888888	0	0	1	0	42.288408	2	-0.4111751
5.2038	226219.438	0.16403893	5.48188606	0.24588176	0	1.64938913	5.42150991	-1.8015739	1.70163162	-1.3988458	-6.9077553	22	89	4.47038889	291.888889	18.88888888	0	0	1	0	42.288408	2	-0.4111751
5.2497	228281.034	0.2140557	15.5738573	0.32718109	0	1.65817093	5.43058185	-1.5368582	2.7456579	-1.1141897	-6.9077553	23	90	4.47038889	291.888889	18.88888888	0	0	1	0	42.288408	2	-0.4111751
0.0081	0	0.42014515	46.4625969	1.70399749	0	-4.8158912	-6.9077553	-0.8647777	3.83866914	0.53356364	-6.9077553	0	89	3.57631111	291.888889	18.88888888	0	0	0	0	18.9135952	2	-0.0428739
0.0081	0	0	0	0	0	-4.8158912	-6.9077553	-6.9077553	-6.9077553	-6.9077553	-6.9077553	1	79	5.36446667	294.111111	21.11111111	0	0	0	0	18.9135952	2	-0.0428739
0.0756	0	0.52301219	74.0915039	2.77609558	0	-2.582299	-6.9077553	-0.6462403	4.30531437	1.02140563	-6.9077553	2	73	6.70558333	295.222222	22.2222222	0	0	0	0	18.9135952	2	-0.0428739
0.2907	3712.15514	0.76934163	188.678315	2.8549889	0	-1.2354635	1.31188196	-0.2609212	5.24004883	1.04941816	-6.9077553	3	66	4.91742778	297.444444	24.44444444	0	0	1	0	18.9135952	2	-0.0428739
0.4878	10582.5129	0.84555534	243.322471	2.3801002	0	-0.7178498	2.3592974	-0.1665797	5.49439171	0.86756265	-6.9077553	4	64	5.36446667	298	25	0	0	1	0	18.9135952	2	-0.0428739
0.6354	13904.684	0.68802238	156.373428	1.49624317	0	-0.4535006	2.63229768	-0.3724815	5.05225331	0.40362553	-6.9077553	5	59	4.91742778	299.111111	26.11111111	0	0	1	0	18.9135952	2	-0.0428739
0.8181	18107.7241	0.74931824	172.02032	1.83979189	0	-0.2007707	2.89639382	-0.2872578	5.14761842	0.61019585	-6.9077553	6	53	6.25854444	300.222222	27.2222222	0	0	1	0	18.9135952	2	-0.0428739
1.0359	24484.8654	0.75337787	167.094444	1.58236896	0	0.03527061	3.19809603	-0.2818619	5.11856517	0.45955483	-6.9077553	7	50	6.25854444	300.777778	27.7777778	0	0	1	0	18.9135952	2	-0.0428739
1.2951	34989.7149	0.8575218	226.317383	1.93222406	0	0.25858791	3.55508274	-0.1525432	5.42194278	0.65918911	-6.9077553	8	53	4.91742778	300.222222	27.2222222	0	0	1	0	18.9135952	2	-0.0428739
1.5066	42005.1008	0.7852278	185.256882	1.57867951	0	0.40985546	3.73781486	-0.2405087	5.22174881	0.45722199	-6.9077553	9	53	4.91742778	299.666667	26.66666666	0	0	1	0	18.9135952	2	-0.0428739
1.7127	50026.7455	0.82898256	199.956502	1.48576282	0	0.53807107	3.91257776	-0.1863506	5.29810486	0.39660115	-6.9077553	10	57	3.57631111	298	25	0	0	1	0	18.9135952	2	-0.0428739
1.8765	57535.3853	0.90378311	219.953211	1.58486402	0	0.62940834	4.05241753	-0.10006	5.39341939	0.46112938	-6.9077553	11	67	1.78815556	295.777778	22.7777778	0	0	1	0	18.9135952	2	-0.0428739
1.9998	63228.4479	0.84285836	193.278969	1.57092397	0	0.69304718	4.14677014	-0.1697706	5.26413975	0.45230032	-6.9077553	12	70	1.78815556	295.222222	22.2222222	0	0	1	0	18.9135952	2	-0.0428739
2.1087	68086.0859	0.74920888	151.052702	1.30313817	0	0.74607164	4.22078756	-0.2874036	5.01763542	0.26554241	-6.9077553	13	75	1.78815556	294.111111	21.11111111	0	0	1	0	18.9135952	2	-0.0428739
2.1969	71946.1056	0.57992526	86.989487	0.96020041	0	0.78704728	4.27593121	-0.5431332	4.46579877	-0.0395724	-6.9077553	14	79	2.23519444	293.555556	20.55555555	0	0	1	0	18.9135952	2	-0.0428739
2.2401	73832.2502	0.26702676	16.8746376	0.47911	0	0.80652051	4.30180918	-1.3166684	2.82587102	-0.73374	-6.9077553	15	83	3.12927222	293	20	0	0	1	0	18.9135952	2	-0.0428739
2.2743	75367.4788	0.22348198	10.4683733	0.3567536	0	0.82167231	4.32238913	-1.4939598	2.34845417	-1.0279108	-6.9077553	16	87	3.57631111	293	20	0	0	1	0	18.9135952	2	-0.0428739
2.304	76595.6648	0.19656907	8.16126598	0.31080971	0	0.83464674	4.33855354	-1.621667	2.09952182	-1.1653622	-6.9077553	17	91	3.57631111	292.444444	19.44444444	0	0	1	0	18.9135952	2	-0.0428739
2.331	77823.8507	0.18032416	6.80748647	0.27578392	0	0.84629736	4.3544608	-1.7074689	1.91816984	-1.2845181	-6.9077553	18	94	3.12927222	291.888889	18.88888888	0	0	1	0	18.9135952	2	-0.0428739
2.3625	79227.4933	0.18561589	7.03117283	0.28812591	0	0.85972038	4.372336	-1.6787028	1.95049574	-1.240893	-6.9077553	19	93	4.02335	291.888889	18.88888888	0	0	1	0	18.9135952	2	-0.0428739
2.3823	80060.9021	0.16635939	5.65304635	0.25143673	0	0.86806641	4.38280011	-1.7876117	1.73237146	-1.3765946	-6.9077553	20	90	4.91742778	291.888889	18.88888888	0	0	1	0	18.9135952	2	-0.0428739
2.4102	81245.2239	0.16354524	5.32134862	0.24642264	0	0.87970973	4.39748434	-1.8045697	1.67191468	-1.3966573	-6.9077553	21	90	3.57631111	291.888889	18.88888888	0	0	1	0	18.9135952	2	-0.0428739
2.4318	82297.9534	0.15434255	4.82267602	0.23405131	0	0.88863172	4.41035839	-1.8621226	1.5735363	-1.4479515	-6.9077553	22	89	4.47038889	291.888889	18.88888888	0	0	1	0	18.9135952	2	-0.0428739
2.4444	82912.045	0.16574855	5.3732436	0.23133731	0	0.89379969	4.41779241	-1.7912683	1.68161784	-1.459565	-6.9077553	23	90	4.47038889	291.888889	18.88888888	0	0	1	0	18.9135952	2	-0.0428739
0.0171	502.209263	0.83959839	193.735175	1.72100491	0	-4.0686768	-0.6867492	-0.1736413	5.26649731	0.54348926	-6.9077553	0	36	3.12927222	290.222222	17.2222222	0	0	0	0	30.519848	2	0.21141735
0.0711	1884.44795	0.76524289	181.709684	1.7239109	0	-2.6436679	0.63416543	-0.2662561	5.20241578	0.5451754	-6.9077553	1	31	3.57631111	293.555556	20.55555555	0	0	0	0	30.519848	2	0.21141735
0.234	2974.56744	0.6619417	129.837373	3.26987723	0	-1.4524342	1.09043476	-0.4110682	4.86629039	1.18505821	-6.9077553	2	22	5.36446667	296.333333	23.33333333	0	0	0	0	30.519848	2	0.21141735
0.4068	4056.60103	0.73191977	154.120158	2.94712989	0	-0.8994336	1.40059192	-0.310719	5.03773903	1.08117103	-6.9077553	3	20	5.81150556	297.444444	24.44444444	0	0	0	0	30.519848	2	0.21141735
0.7092	7095.09618	0.74802435	158.057966	2.6813478	0	-0.3436177	1.9595448	-0.2889838	5.06296817	0.98669246	-6.9077553	4	18	4.91742778	298.555556	25.55555555	0	0	0	1	30.519848	2	0.21141735
1.0647	22830.911	0.87251502	233.242623	2.88251984	0	0.06269307	3.12815916	-0.13523	5.4520835	1.05901171	-6.9077553	5	16	5.36446667	299.111111	26.11111111	0	0	0	1	30.519848	2	0.21141735

1.4373	40792.0573	0.79253878	172.491093	2.20260797	0	0.36276635	3.7085119	-0.2312529	5.1503514	0.790096	-6.9077553	6	18	5.36446667	298.555556	25.5555555	0	0	0	1	30.519848	2	0.21141735
1.7469	58335.9084	0.72897339	151.271246	1.34533319	0	0.55784279	4.06623497	-0.3147472	5.01908117	0.29738474	-6.9077553	7	25	2.68223333	295.222222	22.2222222	0	0	0	1	30.519848	2	0.21141735
2.0493	77710.2579	0.79575457	178.127941	1.49030486	0	0.71749827	4.35300014	-0.2272086	5.18250768	0.39965148	-6.9077553	8	29	1.34111667	293	20	0	0	0	1	30.519848	2	0.21141735
2.358	97452.6905	0.73212951	152.071622	1.3773003	0	0.8578138	4.5793773	-0.3104329	5.02435818	0.32085107	-6.9077553	9	31	1.34111667	290.777778	17.7777778	0	0	0	1	30.519848	2	0.21141735
2.6118	115955.227	0.754028	176.092614	1.47775193	0	0.96003964	4.75321277	-0.2810005	5.17101575	0.39119844	-6.9077553	10	33	2.68223333	290.222222	17.2222222	0	0	0	1	30.519848	2	0.21141735
2.8521	136637.062	0.69625283	133.94859	1.23193428	0	1.04805557	4.91733555	-0.3606072	4.89746353	0.20939692	-6.9077553	11	35	3.12927222	290.222222	17.2222222	0	0	0	1	30.519848	2	0.21141735
3.0888	161043.24	0.72835834	141.994772	1.22011691	0	1.12778267	5.08167911	-0.3155901	4.95579728	0.19976594	-6.9077553	12	36	3.57631111	290.222222	17.2222222	0	0	0	1	30.519848	2	0.21141735
3.339	192895.531	0.71039581	138.238647	1.28132827	0	1.20567136	5.26215394	-0.3405263	4.92898875	0.24867739	-6.9077553	13	40	3.57631111	289.111111	16.11111111	0	0	0	1	30.519848	2	0.21141735
3.5523	227371.311	0.68877182	128.748525	1.49722689	0	1.26759528	5.42658881	-0.3713944	4.85786885	0.40428234	-6.9077553	14	44	3.57631111	288	15	0	0	0	1	30.519848	2	0.21141735
3.7305	258566.056	0.61225059	102.011285	0.99765254	0	1.31654227	5.55515507	-0.4889816	4.62509324	-0.0013484	-6.9077553	15	44	4.91742778	286.888889	13.88888888	0	0	0	1	30.519848	2	0.21141735
3.8997	292263.459	0.63428538	107.903694	1.05695367	0	1.36089963	5.67765908	-0.453681	4.68124838	0.05633655	-6.9077553	16	51	4.02335	285.777778	12.77777777	0	0	0	1	30.519848	2	0.21141735
4.0131	323511.901	0.65605963	112.803502	0.5374679	0	1.38956401	5.77923899	-0.4199805	4.72565625	-0.6190274	-6.9077553	17	63	2.23519444	284.666667	11.66666666	0	0	0	1	30.519848	2	0.21141735
4.1013	352594.985	0.62540132	99.0831075	0.47861006	0	1.411304	5.86532288	-0.467764	4.59596906	-0.7347819	-6.9077553	18	71	2.23519444	284.666667	11.66666666	0	0	0	1	30.519848	2	0.21141735
4.1733	377642.033	0.58708782	86.7098389	0.45277081	0	1.42870709	5.93394939	-0.530879	4.46257889	-0.790163	-6.9077553	19	73	2.68223333	284.666667	11.66666666	0	0	0	1	30.519848	2	0.21141735
4.2309	397669.128	0.52351624	69.3602142	0.39355871	0	1.44241474	5.98562284	-0.6452789	4.23932784	-0.9299873	-6.9077553	20	79	3.57631111	284.666667	11.66666666	0	0	0	1	30.519848	2	0.21141735
4.266	410686.721	0.39469068	36.1800287	0.28652543	0	1.45067662	6.01783312	-0.9271225	3.58853491	-1.246444	-6.9077553	21	83	4.47038889	286.333333	13.33333333	0	0	0	1	30.519848	2	0.21141735
4.266	410686.721	0	0	0	0	1.45067662	6.01783312	-6.9077553	-6.9077553	-6.9077553	-6.9077553	22	84	3.12927222	287.444444	14.44444444	0	0	0	1	30.519848	2	0.21141735
4.2894	418363.784	0.38239112	42.1500761	0.46792727	0	1.45614686	6.03635374	-0.9586996	3.74126021	-0.7573076	-6.9077553	23	83	4.91742778	289.111111	16.11111111	0	0	0	1	30.519848	2	0.21141735
0.0423	1817.18925	1.08871908	344.077849	3.06728728	0	-3.1629682	0.59784109	0.08591994	5.84087084	1.12111952	-6.9077553	0	36	3.12927222	290.222222	17.2222222	0	0	0	0	34.0724638	2	0.49277013
0.1233	5782.5528	0.96979836	259.047982	1.60592973	0	-2.0931349	1.75501816	-0.0296365	5.55701716	0.47432536	-6.9077553	1	31	3.57631111	293.555556	20.55555555	0	0	0	0	34.0724638	2	0.49277013
0.2556	10853.7493	0.98915214	259.68564	2.38229432	0	-1.3641416	2.38460271	-0.0098967	5.55947567	0.8684837	-6.9077553	2	22	5.36446667	296.333333	23.33333333	0	0	0	1	34.0724638	2	0.49277013
0.495	24780.0055	1.3638456	549.748766	4.59403498	0	-0.7031975	3.21007745	0.31104131	6.3094632	1.52497637	-6.9077553	3	20	5.81150556	297.444444	24.44444444	0	0	0	1	34.0724638	2	0.49277013
0.954	54465.1778	1.38991017	566.31924	3.97517186	0	-0.0470916	3.99757992	0.32995833	6.33915971	1.38031951	-6.9077553	4	18	4.91742778	298.555556	25.55555555	0	0	0	1	34.0724638	2	0.49277013
1.6344	71034.7258	1.42625791	635.450893	4.83299393	2	0.49127576	4.26318293	0.35575505	6.45433639	1.57567303	0.69364706	5	16	5.36446667	299.111111	26.11111111	0	0	0	1	34.0724638	2	0.49277013
2.5038	98077.1296	1.27495264	492.205907	3.9148346	2	0.91780958	4.5857644	0.24369307	6.19889917	1.36502849	0.69364706	6	18	5.36446667	298.555556	25.55555555	0	0	0	1	34.0724638	2	0.49277013
3.1788	123334.925	1.03492367	304.453583	2.33536621	2	1.15650377	4.81491173	0.03529346	5.71852192	0.84859682	0.69364706	7	25	2.68223333	295.222222	22.2222222	0	0	0	1	34.0724638	2	0.49277013
3.7908	145337.462	0.93852584	244.264545	1.78726789	2	1.33257708	4.97906524	-0.06238	5.49825593	0.58124749	0.69364706	8	29	1.34111667	293	20	0	0	0	1	34.0724638	2	0.49277013
4.3794	164011.143	0.91531325	232.82306	1.79597105	2	1.47691173	5.09994047	-0.087397	5.45028306	0.58610249	0.69364706	9	31	1.34111667	290.777778	17.7777778	0	0	0	1	34.0724638	2	0.49277013
4.9293	177506.895	0.82913478	193.188727	2.10183022	2	1.59519699	5.17901509	-0.1861672	5.26367275	0.74328416	0.69364706	10	33	2.68223333	290.222222	17.2222222	0	0	0	1	34.0724638	2	0.49277013
5.4405	192650.614	0.79178689	178.545967	1.658112	2	1.69387097	5.26088345	-0.2322008	5.18485169	0.50628252	0.69364706	11	35	3.12927222	290.222222	17.2222222	0	0	0	1	34.0724638	2	0.49277013
5.9463	209025.393	0.78473074	170.537394	1.55041895	2	1.78276918	5.34246053	-0.2411411	5.13896046	0.43916996	0.69364706	12	36	3.57631111	290.222222	17.2222222	0	0	0	1	34.0724638	2	0.49277013
6.507	223836.598	0.80263601	177.059465	1.87962143	2	1.87287852	5.41092078	-0.2186088	5.17649129	0.63160227	0.69364706	13	40	3.57631111	289.111111	16.11111111	0	0	0	1	34.0724638	2	0.49277013
7.0731	241786.5	0.81373031	186.958019	1.73812677	2	1.95629886	5.48805924	-0.2048981	5.23088944	0.55338313	0.69364706	14	44	3.57631111	288	15	0	0	0	1	34.0724638	2	0.49277013
7.7544	260804.267	0.77750162	172.061974	2.01014587	2	2.04826042	5.56377403	-0.2503842	5.14786054	0.69870464	0.69364706	15	44	4.91742778	286.888889	13.88888888	0	0	0	1	34.0724638	2	0.49277013
8.3412	278473.788	0.74561361	162.263516	1.74865236	2	2.12120709	5.62932753	-0.2922075	5.08922782	0.55941712	0.69364706	16	51	4.02335	285.777778	12.77777777	0	0	0	1	34.0724638	2	0.49277013
8.7174	295582.283	0.85111379	205.80708	1.45856195	2	2.16532103	5.68895063	-0.1600352	5.32694408	0.37813635	0.69364706	17	63	2.23519444	284.666667	11.66666666	0	0	0	1	34.0724638	2	0.49277013
9.0117	308739.779	0.86526685	202.761229	1.54264043	2	2.19852373	5.73250202	-0.1435623	5.31203401	0.43414354	0.69364706	18	71	2.23519444	284.666667	11.66666666	0	0	0	1	34.0724638	2	0.49277013
9.2295	317703.552	0.78981573	168.400665	1.43860771	2	2.22240488	5.76112187	-0.2346903	5.12635199	0.36437065	0.69364706	19	73	2.68223333	284.666667	11.66666666	0	0	0	1	34.0724638	2	0.49277013
9.3771	323856.509	0.73161753	141.847144	1.30673385	2	2.23827055	5.78030363	-0.3111315	4.95475708	0.26829576	0.69364706	20	79	3.57631111	284.666667	11.66666666	0	0	0	1	34.0724638	2	0.49277013
9.4626	327277.9	0.6012197	92.1901933	0.91935666	2	2.24734719	5.79081271	-0.507133	4.52386461	-0.082994	0.69364706	21	83	4.47038889	286.333333	13.33333333	0	0	0	1	34.0724638	2	0.49277013
9.4842	328199.035	0.28294009	19.1593005	0.52880243	2	2.24962726	5.79362328	-1.258992	2.95284046	-0.6352511	0.69364706	22	84	3.12927222	287.444444	14.44444444	0	0	0	1	34.0724638	2	0.49277013
9.5796	331654.539	0.38475152	45.0907881	0.98969319	2	2.25963584	5.8040969	-0.9525619	3.80870015	-0.0093504	0.69364706	23	83	4.91742778	289.111111	16.11111111	0	0	0	1	34.0724638	2	0.49277013
0.0522	3940.0944	1.32714188	498.057326	2.94163754	0	-2.9526728	1.37145845	0.28378088	6.21071719	1.0793063	-6.9077553	0	36	3.12927222	290.222222	17.2222222	0	0	0	0	42.288408	2	-0.4111751
0.252	13648.2828	1.17549504	388.420608	3.72492665	0	-1.3783262	2.61368697	0.16253972	5.96209137	1.31531559	-6.9077553	1	31	3.57631111	293.555556	20.55555555	0	0	0	0	42.288408	2	-0.4111751
0.5373	27504.6931	1.12083796	348.487628	2.76343524	0	-0.6211987	3.314393	0.11496838	5.8536056	1.01683636	-6.9077553	2	22	5.36446667	296.333333	23.33333333	0	0	0	0	42.288408	2	-0.4111751
0.9216	45235.643	1.06910466	311.071977	2.58414104	0	-0.081644	3.81190745	0.06775646	5.74002754	0.94978007	-6.9077553	3	20	5.81150556	297.444444	24.44444444	0	0	0	0	42.288408	2	-0.4111751
1.4175	67074.9278	1.05397845	309.500681	2.83456969	0	0.34889476	4.20582523	0.05352034	5.73496352	1.04224287	-6.9077553	4	18	4.91742778	298.555556	25.55555555	0	0	1	0	42.288408	2	-0.4111751
2.1393	101500.616	1.1182719	366.975842	3.10533724	0	0.76047867	4.62007472	0.11267839	5.90529874	1.1334443	-6.9077553	5	16	5.36446667	299.111111	26.11111111	0	0	1	0	42.288408	2	-0.4111751
2.8791	140239.031	1.12802325	369.380893	2.64141267	0	1.05747775	4.94335546	0.12135287	5.91183105	0.97169239	-6.9077553	6	18	5.36446667	298.555556	25.55555555	0	0	1	0	42.288408	2	-0.4111751
3.528	167180.154	1.00641301	296.933296	2.33449263	0	1.26073114	5.11907798	0.00738567	5.69351089	0.84822285	-6.9077553	7	25	2.68223333	295.222222	22.2222222	0	0	1	0	42.288408	2	-0.4111751
4.0095	183915.476	0.83558395	196.138843	1.68222405	0	1.38866655	5.21448172	-0.1784284	5.27882789	0.52071103	-6.9077553	8	29	1.34111667	293	20	0	0	1	0	42.288408	2	-0.4111751
4.5783	209954.632	0.92614939	250.590215	1.87863005	0	1.52132775	5.34689623	-0.0756406	5.52382298	0.63107497	-6.9077553	9	31	1.34111667	290.777778	17.7777778	0	0	1	0	42.288408	2	-0.4111751
5.1156	234284.117	0.91978498	249.886987	1.86517549	0	1.63229469	5.45653882	-0.0825287	5.52101277	0.62389114	-6.9077553	10	33	2.68223333	290.222222	17.2222222	0	0	1	0	42.288408	2	-0.4111751
5.6565	253863.798	0.85419158	212.715448	1.78447176	0	1.73280533	5.53680183	-0.1564298	5.35996005	0.57968267	-6.9077553	11	35	3.12927222	290.222222	17.2222222	0	0	1	0	42.288408	2	-0.4111751
6.1164	266549.552	0.70395345	147.710265	1.41612468	0	1.81097369	5.58556391	-0.3496235	4.99525945	0.34862994	-6.9077553	12	36	3.57631111	290.222222	17.2222222	0	0	1	0	42.288408	2	-0.4111751
6.534	278247.953	0.68941143	153.023892	1.41479847	0	1.87701931	5.62851623	-0.3704676	5.0306006	0.34769366	-6.9077553	13	40	3.57631111	289.111111	16.11111111	0	0	1	0	42.288408	2	-0.4111751
6.912	288673.517	0.63862174	130.615337	1.23629063	0	1.93325903	5.66529981	-0.4468783	4.8722643	0.21292401	-6.9077553	14	44	3.57631111	288	15	0	0	1	0	42.288408	2	-0.4111751
7.3242	297973.651	0.70792592	173.578586	1.63901117	0	1.99118393	5.69700842	-0.3440042	5.1566362	0.49470305	-6.9077553	15	44	4.91742778	286.888889	13.88888888	0	0	1	0	42.288408	2	-0.4111751
7.6689	307209.536	0.64689461	135.378549	1.19912385	0	2.03717319	5.7275333	-0.4340272	4.90808231	0.18242476	-6.9077553	16	51	4.02335	285.777778	12.77777777	0	0	1	0	42.288408	2	-0.4111751
7.8534	314757.383	0.75910161	165.916082	1.19625706	0	2.06094656	5.75180531	-0.2743032	5.11148816	0.18003315	-6.9077553	17	63	2.23519444	284.666667	11.66666666	0	0	1	0	42.288408	2	-0.4111751
7.9524	318883.568	0.80669715	174.374023	1.38793238	0	2.07347377	5.76482918	-0.2135681	5.16120829	0.32853538	-6.9077553	18	71	2.23519444	284.666667	11.66666666	0	0	1	0	42.288408	2	-0.4111751
8.0271	321901.152	0.7481166	149.724198	1.26738987	0	2.08282332	5.77424762	-0.2888606	5.0088016	0.23774828	-6.9077553	19	73	2.68223333	284.666667	11.66666666	0	0	1	0	42.288408	2	-0.4111751

8.1108	325633.653	0.7726479	163.267463	1.327856	0	2.09319651	5.78577606	-0.2566384	5.09539586	0.28431842	-6.9077553	20	79	3.57631111	284.666667	11.66666666	0	0	1	0	42.288408	2	-0.4111751
8.1549	327388.234	0.61337561	107.341717	1.06347905	0	2.09861897	5.79114978	-0.4871488	4.67602668	0.06248553	-6.9077553	21	83	4.47038889	286.333333	13.33333333	0	0	1	0	42.288408	2	-0.4111751
8.1684	327958.435	0.20160791	8.60435079	0.24836737	0	2.10027305	5.79288993	-1.5964826	2.15238419	-1.3888281	-6.9077553	22	84	3.12927222	287.444444	14.44444444	0	0	1	0	42.288408	2	-0.4111751
8.2638	331192.626	0.55785787	111.225162	1.3144609	0	2.11188453	5.80270318	-0.5818601	4.71156563	0.2741871	-6.9077553	23	83	4.91742778	289.111111	16.11111111	0	0	1	0	42.288408	2	-0.4111751
0.0117	0	0.51139458	72.3967692	2.07208411	0	-4.4481664	-6.9077553	-0.6686603	4.28217549	0.72903741	-6.9077553	0	36	3.12927222	290.222222	17.2222222	0	0	0	0	18.9135952	2	-0.0428739
0.1116	0	0.61006151	99.453301	2.91147263	0	-2.1928342	-6.9077553	-0.4925577	4.59969825	1.06900242	-6.9077553	1	31	3.57631111	293.555556	20.55555555	0	0	0	0	18.9135952	2	-0.0428739
0.3483	3200.39588	0.78228951	209.769427	3.46575905	0	-1.0546911	1.16358693	-0.2442529	5.34601373	1.24322016	-6.9077553	2	22	5.36446667	296.333333	23.33333333	0	0	1	0	18.9135952	2	-0.0428739
0.5787	12547.9823	1.00022054	357.211029	2.82740307	0	-0.5469711	2.52963957	0.00121979	5.87832952	1.03971227	-6.9077553	3	20	5.81150556	297.444444	24.44444444	0	0	1	0	18.9135952	2	-0.0428739
0.7713	17311.8747	0.82373703	217.340318	1.8599999	0	-0.2596779	2.85145043	-0.1926907	5.38146901	0.62111392	-6.9077553	4	18	4.91742778	298.555556	25.55555555	0	0	1	0	18.9135952	2	-0.0428739
1.0368	25263.6864	0.87929625	240.195895	2.25302486	0	0.03613905	3.22940763	-0.1274968	5.48145898	0.81271745	-6.9077553	5	16	5.36446667	299.111111	26.11111111	0	0	1	0	18.9135952	2	-0.0428739
1.3824	39248.0155	0.99107679	303.544271	2.51152929	0	0.32382112	3.66992636	-0.0079548	5.71553076	0.92128993	-6.9077553	6	18	5.36446667	298.555556	25.55555555	0	0	1	0	18.9135952	2	-0.0428739
1.7199	50723.8	0.89185164	241.784688	1.91960482	0	0.54226615	3.92641494	-0.1133348	5.48805174	0.65264015	-6.9077553	7	25	2.68223333	295.222222	22.2222222	0	0	1	0	18.9135952	2	-0.0428739
2.0223	61931.9625	0.81271344	202.422503	1.6270247	0	0.70423548	4.12605255	-0.206147	5.31036205	0.48736744	-6.9077553	8	29	1.34111667	293	20	0	0	1	0	18.9135952	2	-0.0428739
2.3805	74717.5413	0.83838236	214.644629	1.73549878	0	0.86731055	4.31372827	-0.1750889	5.36898843	0.55187089	-6.9077553	9	31	1.34111667	290.777778	17.7777778	0	0	1	0	18.9135952	2	-0.0428739
2.8368	92722.8179	0.87547137	232.967348	1.98904991	0	1.04267666	4.52962537	-0.1318512	5.4509026	0.68815972	-6.9077553	10	33	2.68223333	290.222222	17.2222222	0	0	1	0	18.9135952	2	-0.0428739
3.2616	107501.641	0.82779655	203.866989	1.84795974	0	1.18221787	4.67751541	-0.1877806	5.31747267	0.61462318	-6.9077553	11	35	3.12927222	290.222222	17.2222222	0	0	1	0	18.9135952	2	-0.0428739
3.7224	122160.858	0.78074044	179.86676	1.75188351	0	1.31436862	4.80534687	-0.2462325	5.19222192	0.56126215	-6.9077553	12	36	3.57631111	290.222222	17.2222222	0	0	1	0	18.9135952	2	-0.0428739
4.1634	138204.13	0.80077901	187.304401	1.59257352	0	1.42633205	4.92873903	-0.2209223	5.23274044	0.46597899	-6.9077553	13	40	3.57631111	289.111111	16.11111111	0	0	1	0	18.9135952	2	-0.0428739
4.6458	157265.579	0.83692699	210.223327	1.72414136	0	1.53596359	5.05794232	-0.1768243	5.34817518	0.545309	-6.9077553	14	44	3.57631111	288	15	0	0	1	0	18.9135952	2	-0.0428739
5.0904	172291.907	0.76941425	178.610609	1.70692277	0	1.62735641	5.14919598	-0.2608269	5.18521366	0.53527788	-6.9077553	15	44	4.91742778	286.888889	13.88888888	0	0	1	0	18.9135952	2	-0.0428739
5.4999	186548.998	0.75482392	165.139526	1.50665873	0	1.70472991	5.22869929	-0.2799468	5.10679678	0.41055794	-6.9077553	16	51	4.02335	285.777778	12.77777777	0	0	1	0	18.9135952	2	-0.0428739
5.7978	198941.12	0.80189242	178.303838	1.26287897	0	1.75747854	5.29301393	-0.2195345	5.18349466	0.23418554	-6.9077553	17	63	2.23519444	284.666667	11.66666666	0	0	1	0	18.9135952	2	-0.0428739
6.0732	211967.236	0.86964307	202.06993	1.48483915	0	1.80388565	5.35643643	-0.1385232	5.30861877	0.39597969	-6.9077553	18	71	2.23519444	284.666667	11.66666666	0	0	1	0	18.9135952	2	-0.0428739
6.3531	225077.294	0.85404924	194.885387	1.50359381	0	1.84894288	5.41644831	-0.1565962	5.27241676	0.40852297	-6.9077553	19	73	2.68223333	284.666667	11.66666666	0	0	1	0	18.9135952	2	-0.0428739
6.6483	239345.018	0.80271186	171.865735	1.38739386	0	1.89436118	5.47791028	-0.2185145	5.14671938	0.32814758	-6.9077553	20	79	3.57631111	284.666667	11.66666666	0	0	1	0	18.9135952	2	-0.0428739
6.858	249034.853	0.69424471	128.172512	1.2145649	0	1.92541585	5.51759688	-0.3634914	4.85338491	0.19520891	-6.9077553	21	83	4.47038889	286.333333	13.33333333	0	0	1	0	18.9135952	2	-0.0428739
6.9399	252722.489	0.26254664	21.2177533	0.4850839	0	1.93728737	5.53229596	-1.3335249	3.05488538	-0.721374	-6.9077553	22	84	3.12927222	287.444444	14.44444444	0	0	1	0	18.9135952	2	-0.0428739
7.2144	265563.903	0.71921992	161.709631	1.49170522	0	1.97607903	5.58185926	-0.3281987	5.08580851	0.40059006	-6.9077553	23	83	4.91742778	289.111111	16.11111111	0	0	1	0	18.9135952	2	-0.0428739
0.0063	223.025448	0.9196624	228.244771	1.51212994	0	-5.0672056	-1.4959956	-0.0826619	5.43042299	0.41418032	-6.9077553	0	92	1.34111667	298	25	0	0	0	0	30.519848	2	0.21141735
0.0126	486.208184	0.52570401	65.2169538	0.87177127	0	-4.3740585	-0.7190638	-0.6411165	4.1777348	-0.1360818	-6.9077553	1	95	1.34111667	297.444444	24.44444444	0	0	0	0	30.519848	2	0.21141735
0.0288	1056.43742	0.58650128	88.0337728	0.98693212	0	-3.5473799	0.05584846	-0.5318769	4.47773188	-0.0121413	-6.9077553	2	96	2.23519444	297.444444	24.44444444	0	0	0	0	30.519848	2	0.21141735
0.0369	1231.89252	0.63000075	98.9739855	1.15715271	0	-3.2995437	0.20936305	-0.4604482	4.59486715	0.14682624	-6.9077553	3	98	1.78815556	297.444444	24.44444444	0	0	0	0	30.519848	2	0.21141735
0.0468	1582.80277	0.34985174	31.5989213	0.87814019	0	-3.0618721	0.45982877	-1.0473915	3.45315463	-0.1288109	-6.9077553	4	93	3.12927222	298.555556	25.55555555	0	0	0	0	30.519848	2	0.21141735
0.0945	1670.5302	0.32869026	29.5175067	1.34360245	0	-2.3591554	0.51373949	-1.1096017	3.38501741	0.29609839	-6.9077553	5	80	4.02335	300.777778	27.7777778	0	0	0	0	30.519848	2	0.21141735
0.2223	2237.2409	0.53638039	85.9632345	2.42541977	0	-1.5037275	0.80569025	-0.6210491	4.45393133	0.88641682	-6.9077553	6	72	4.47038889	302.444444	29.44444444	0	0	0	0	30.519848	2	0.21141735
0.3042	3359.07269	0.62456324	111.49179	1.76750921	0	-1.1900699	1.21196261	-0.4691029	4.71395993	0.57013694	-6.9077553	7	62	3.57631111	304.111111	31.11111111	0	0	0	0	30.519848	2	0.21141735
0.4122	4161.63033	0.58318138	90.4062663	1.69317691	0	-0.8862466	1.42614716	-0.5375438	4.50432464	0.52719702	-6.9077553	8	55	3.12927222	305.777778	32.7777778	0	0	0	1	30.519848	2	0.21141735
0.5688	5407.10661	0.62453364	103.571704	1.78879872	0	-0.5642264	1.68789905	-0.4691502	4.64027382	0.58210316	-6.9077553	9	50	3.57631111	306.888889	33.88888888	0	0	0	1	30.519848	2	0.21141735
0.7695	8386.90593	0.64074166	117.84544	1.67994834	0	-0.2620143	2.1267909	-0.4435694	4.76938242	0.51935812	-6.9077553	10	46	2.68223333	307.444444	34.4444444	0	0	0	1	30.519848	2	0.21141735
1.0215	14182.6623	0.77408131	171.738086	1.93101937	0	0.02127214	2.65209076	-0.2547873	5.14597638	0.65856576	-6.9077553	11	41	2.68223333	309.111111	36.11111111	0	0	0	1	30.519848	2	0.21141735
1.2672	19192.976	0.78433686	174.920015	1.83563322	0	0.23680974	2.95459648	-0.2416425	5.16433453	0.60793412	-6.9077553	12	38	3.12927222	309.666667	36.66666666	0	0	0	1	30.519848	2	0.21141735
1.548	24264.0089	0.75917958	159.599947	1.81954584	0	0.43696378	3.18903535	-0.2742006	5.07267662	0.59913637	-6.9077553	13	36	3.12927222	309.111111	36.11111111	0	0	0	1	30.519848	2	0.21141735
1.7964	30127.9939	0.78739354	170.427565	1.56513214	0	0.58578466	3.40548796	-0.2377579	5.13831623	0.44860898	-6.9077553	14	38	3.12927222	308.555556	35.5555556	0	0	0	1	30.519848	2	0.21141735
2.0106	35693.1015	0.82914567	187.570444	1.47716311	0	0.69843318	3.57498545	-0.1861541	5.23415981	0.39080017	-6.9077553	15	42	1.34111667	308	35	0	0	0	1	30.519848	2	0.21141735
2.1978	41032.9934	0.77982462	177.237868	1.43035859	0	0.78745686	3.71440083	-0.2474047	5.17749836	0.35862406	-6.9077553	16	54	1.34111667	304.666667	31.66666666	0	0	0	1	30.519848	2	0.21141735
2.304	44409.3979	0.75347584	168.56058	1.24300372	0	0.83464674	3.79347363	-0.281732	5.12730114	0.21833498	-6.9077553	17	61	0.89407778	303	30	0	0	0	1	30.519848	2	0.21141735
2.3652	46584.8944	0.82404793	190.444523	1.32816023	0	0.86086258	3.8412978	-0.1923138	5.24936618	0.28454734	-6.9077553	18	66	1.34111667	301.888889	28.88888888	0	0	0	1	30.519848	2	0.21141735
2.3877	47138.0784	0.76898575	160.22751	1.22072151	0	0.87033056	3.85310235	-0.2613833	5.07660098	0.20026094	-6.9077553	19	66	1.34111667	301.333333	28.33333333	0	0	0	1	30.519848	2	0.21141735
2.4003	47440.9184	0.69193792	124.532244	0.97161422	0	0.87559373	3.85950619	-0.3668149	4.8245727	-0.0277678	-6.9077553	20	69	1.34111667	300.777778	27.7777778	0	0	0	1	30.519848	2	0.21141735
2.4093	47506.4458	0.62712274	111.560767	0.78288631	0	0.87933625	3.86088645	-0.4650197	4.7145784	-0.2434913	-6.9077553	21	72	1.78815556	299.666667	26.66666666	0	0	0	1	30.519848	2	0.21141735
2.4138	47557.196	0.38838227	33.7181976	0.2907104	0	0.88120227	3.86195414	-0.9431937	3.51806734	-1.2319937	-6.9077553	22	78	2.23519444	299.666667	26.66666666	0	0	0	1	30.519848	2	0.21141735
2.4138	47557.196	0	0	0	0	0.88120227	3.86195414	-6.9077553	-6.9077553	-6.9077553	-6.9077553	23	83	2.23519444	299.111111	26.11111111	0	0	0	1	30.519848	2	0.21141735
0.0135	613.113024	0.99223934	271.570469	1.78931958	0	-4.3050656	-0.4875763	-0.0067836	5.60422534	0.58239414	-6.9077553	0	92	1.34111667	298	25	0	0	0	0	34.0724638	2	0.49277013
0.0252	964.023312	0.53692	67.7252948	0.74450955	0	-3.6809113	-0.035603	-0.6200454	4.21547451	-0.2936873	-6.9077553	1	95	1.34111667	297.444444	24.44444444	0	0	0	0	34.0724638	2	0.49277013
0.0369	1446.52488	0.51878016	67.6610906	0.83403177	0	-3.2995437	0.36985512	-0.6543493	4.21452606	-0.1802855	-6.9077553	2	96	2.23519444	297.444444	24.44444444	0	0	0	0	34.0724638	2	0.49277013
0.0504	2016.75406	0.53076172	70.4427734	0.80847206	0	-2.9877641	0.70198504	-0.6315598	4.25481485	-0.211373	-6.9077553	3	98	1.78815556	297.444444	24.44444444	0	0	0	0	34.0724638	2	0.49277013
0.0792	3201.07624	0.60747588	92.4267426	1.03721189	0	-2.535779	1.16379943	-0.496798	4.52642718	0.0374999	-6.9077553	4	93	3.12927222	298.555556	25.55555555	0	0	0	0	34.0724638	2	0.49277013
0.1386	5869.54145	0.78898343	168.544715	1.4870913	0	-1.9761632	1.76994687	-0.2357433	5.12720702	0.39749429	-6.9077553	5	80	4.02335	300.777778	27.7777778	0	0	0	0	34.0724638	2	0.49277013
0.2106	8973.7198	0.83831711	189.017017	1.56152515	0	-1.5577947	2.19441171	-0.1751667	5.24184234	0.4463032	-6.9077553	6	72	4.47038889	302.444444	29.44444444	0	0	0	0	34.0724638	2	0.49277013
0.3438	14574.0179	1.01300245	288.161001	2.6971716	0	-1.0676952	2.67930896	0.01390532	5.66352282	0.99257436	-6.9077553	7	62	3.57631111	304.111111	31.11111111	0	0	0	1	34.0724638	2	0.49277013
0.5454	25562.6963	1.12730081	357.617955	2.81117875	0	-0.6062358	3.24117323	0.12071279	5.87946805	1.03395954	-6.9077553	8	55	3.12927222	305.777778	32.7777778	0	0	0	1	34.0724638	2	0.49277013
0.8928	43192.3298	1.19461819	399.938755	3.11119973	0	-0.1133927	3.76568608	0.17866336	5.99131392	1.13532978	-6.9077553	9	50	3.57631111	306.888889	33.88888888	0	0	0	1	34.0724638	2	0.49277013

1.2006	52645.0816	0.99916478	283.058891	2.11764205	0	0.18282143	3.96359181	0.00016477	5.6456585	0.75077534	-6.9077553	10	46	2.68223333	307.444444	34.4444444	0	0	0	1	34.0724638	2	0.49277013
1.548	62046.3627	0.95350694	249.875243	1.95635338	0	0.43696378	4.12789801	-0.0465604	5.52096577	0.67159324	-6.9077553	11	41	2.68223333	309.111111	36.11111111	0	0	0	1	34.0724638	2	0.49277013
2.052	75505.1209	1.06753943	325.517578	2.50314178	0	0.71881493	4.32421372	0.0662927	5.78541954	0.91794607	-6.9077553	12	38	3.12927222	309.666667	36.66666666	0	0	0	1	34.0724638	2	0.49277013
2.7495	101362.183	1.07240738	327.002419	2.61620353	0	1.01141908	4.61870994	0.07083806	5.78997063	0.96210639	-6.9077553	13	36	3.12927222	309.111111	36.11111111	0	0	0	1	34.0724638	2	0.49277013
3.3867	121186.098	0.99021213	277.744439	2.20857161	0	1.219856	4.79733562	-0.0088267	5.62670501	0.79279866	-6.9077553	14	38	3.12927222	308.555556	35.5555556	0	0	0	1	34.0724638	2	0.49277013
3.9303	139010.465	0.90241226	226.119361	1.68078987	0	1.36871576	4.93455641	-0.1015763	5.42106743	0.51985863	-6.9077553	15	42	1.34111667	308	35	0	0	0	1	34.0724638	2	0.49277013
4.3551	151379.027	0.85853409	208.222855	1.57037509	0	1.47134757	5.01979341	-0.1513648	5.33861373	0.45195109	-6.9077553	16	54	1.34111667	304.666667	31.66666666	0	0	0	1	34.0724638	2	0.49277013
4.608	160117.667	0.74419536	158.279999	1.21105501	0	1.52779392	5.07591521	-0.2941089	5.06437193	0.19231727	-6.9077553	17	61	0.89407778	303	30	0	0	0	1	34.0724638	2	0.49277013
4.8141	169600.944	0.85903518	196.493962	1.43483098	0	1.57154911	5.13345419	-0.150782	5.28063679	0.36174376	-6.9077553	18	66	1.34111667	301.888889	28.88888888	0	0	0	1	34.0724638	2	0.49277013
5.0022	177770.547	0.78478916	161.449518	1.26228632	0	1.60987782	5.18049928	-0.2410668	5.0841987	0.23371651	-6.9077553	19	66	1.34111667	301.333333	28.33333333	0	0	0	1	34.0724638	2	0.49277013
5.193	185675.808	0.76523964	155.005159	1.25947456	0	1.64731156	5.22400757	-0.2662603	5.04346485	0.23148828	-6.9077553	20	69	1.34111667	300.777778	27.7777778	0	0	0	1	34.0724638	2	0.49277013
5.3847	194008.902	0.74090476	145.331554	1.19719141	0	1.6835616	5.2679092	-0.2985344	4.9790246	0.18081326	-6.9077553	21	72	1.78815556	299.666667	26.66666666	0	0	0	1	34.0724638	2	0.49277013
5.5134	199520.34	0.66378667	113.343668	1.04384138	0	1.70718149	5.2959212	-0.4082891	4.73043334	0.04386509	-6.9077553	22	78	2.23519444	299.666667	26.66666666	0	0	0	1	34.0724638	2	0.49277013
5.5602	201581.948	0.24644092	14.3453991	0.45889561	0	1.71563408	5.30620095	-1.3965834	2.66349898	-0.7767558	-6.9077553	23	83	2.23519444	299.111111	26.11111111	0	0	0	1	34.0724638	2	0.49277013
0.0252	1409.85575	1.04154693	292.252459	1.99335098	0	-3.6809113	0.34419643	0.04166669	5.67762144	0.69031868	-6.9077553	0	92	1.34111667	298	25	0	0	0	0	42.288408	2	-0.4111751
0.0693	3562.18975	0.70923879	128.723473	1.26968454	0	-2.6693104	1.27065614	-0.342154	4.85767425	0.23955576	-6.9077553	1	95	1.34111667	297.444444	24.44444444	0	0	0	0	42.288408	2	-0.4111751
0.1206	5930.8347	0.60493121	93.3957648	1.06386653	0	-2.115276	1.78033356	-0.5009888	4.53685671	0.06284947	-6.9077553	2	96	2.23519444	297.444444	24.44444444	0	0	0	0	42.288408	2	-0.4111751
0.1836	8957.43596	0.65616657	109.185826	1.18464246	0	-1.6949958	2.19259565	-0.4198178	4.69306041	0.17028479	-6.9077553	3	98	1.78815556	297.444444	24.44444444	0	0	0	0	42.288408	2	-0.4111751
0.2547	12338.1766	0.70887249	129.496292	1.24726732	0	-1.3676689	2.51277929	-0.3426699	4.86365997	0.22175645	-6.9077553	4	93	3.12927222	298.555556	25.55555555	0	0	0	0	42.288408	2	-0.4111751
0.4752	20319.857	0.79870711	188.300096	2.28817637	0	-0.7440195	3.0116478	-0.2235097	5.23804225	0.82819209	-6.9077553	5	80	4.02335	300.777778	27.7777778	0	0	0	0	42.288408	2	-0.4111751
0.7848	32821.3592	0.92454759	232.774573	2.33870342	0	-0.2423264	3.49110996	-0.0773697	5.45007478	0.85002418	-6.9077553	6	72	4.47038889	302.444444	29.44444444	0	0	1	0	42.288408	2	-0.4111751
1.1142	45486.3501	0.85780476	197.029244	1.71593004	0	0.10813666	3.81743427	-0.1522137	5.28335724	0.54053784	-6.9077553	7	62	3.57631111	304.111111	31.11111111	0	0	1	0	42.288408	2	-0.4111751
1.4868	61132.3131	0.88266846	207.436122	1.54498586	0	0.39662616	4.11305694	-0.1236733	5.33482827	0.4356618	-6.9077553	8	55	3.12927222	305.777778	32.7777778	0	0	1	0	42.288408	2	-0.4111751
1.9287	78794.3644	0.89971924	219.637793	1.76184878	0	0.6568462	4.36685417	-0.1045617	5.39198435	0.56693112	-6.9077553	9	50	3.57631111	306.888889	33.88888888	0	0	1	0	42.288408	2	-0.4111751
2.5434	101612.913	0.91624082	239.834233	2.33404872	0	0.93350177	4.62118047	-0.0863852	5.47995216	0.84803275	-6.9077553	10	46	2.68223333	307.444444	34.4444444	0	0	1	0	42.288408	2	-0.4111751
3.2382	131112.869	0.97182635	271.157525	2.30925786	0	1.17501762	4.87606618	-0.0275497	5.60270361	0.83735915	-6.9077553	11	41	2.68223333	309.111111	36.11111111	0	0	1	0	42.288408	2	-0.4111751
3.9888	165810.962	1.02247814	305.23252	2.3567035	0	1.38349043	5.11085439	0.02320676	5.72107712	0.85768805	-6.9077553	12	38	3.12927222	309.666667	36.66666666	0	0	1	0	42.288408	2	-0.4111751
4.7052	198566.604	0.99860708	287.438756	2.23485316	0	1.54866828	5.29112962	-0.000393	5.66101329	0.80462288	-6.9077553	13	36	3.12927222	309.111111	36.11111111	0	0	1	0	42.288408	2	-0.4111751
5.391	227997.628	0.95942588	272.390358	2.1841695	0	1.6847309	5.42933961	-0.0403785	5.60723985	0.7816934	-6.9077553	14	38	3.12927222	308.555556	35.5555556	0	0	1	0	42.288408	2	-0.4111751
5.9562	245454.536	0.80042189	199.390799	1.74339537	0	1.78443269	5.50311582	-0.2213678	5.29527173	0.55640801	-6.9077553	15	42	1.34111667	308	35	0	0	1	0	42.288408	2	-0.4111751
6.363	255560.563	0.63821094	128.6383	1.29615743	0	1.85049996	5.54346333	-0.4475208	4.85701237	0.26017528	-6.9077553	16	54	1.34111667	304.666667	31.66666666	0	0	1	0	42.288408	2	-0.4111751
6.5745	263779.182	0.742448	167.643052	1.22844238	0	1.88319853	5.57511611	-0.2964565	5.12184299	0.20656072	-6.9077553	17	61	0.89407778	303	30	0	0	1	0	42.288408	2	-0.4111751
6.7212	270374.557	0.8186214	182.700968	1.36331345	0	1.90526671	5.59981194	-0.1989128	5.20785624	0.31065134	-6.9077553	18	66	1.34111667	301.888889	28.88888888	0	0	1	0	42.288408	2	-0.4111751
6.8463	275801.427	0.76601437	154.900039	1.22637961	0	1.92370836	5.61968477	-0.2652497	5.04278646	0.2048815	-6.9077553	19	66	1.34111667	301.333333	28.33333333	0	0	1	0	42.288408	2	-0.4111751
6.9588	281058.733	0.78276343	158.316609	1.20037109	0	1.94000705	5.63856722	-0.2436481	5.0646032	0.18346348	-6.9077553	20	69	1.34111667	300.777778	27.7777778	0	0	1	0	42.288408	2	-0.4111751
7.0515	285010.842	0.70995635	128.686248	1.07839795	0	1.95324036	5.65253073	-0.3411443	4.85738503	0.07640344	-6.9077553	21	72	1.78815556	299.666667	26.66666666	0	0	1	0	42.288408	2	-0.4111751
7.1424	288216.682	0.62944031	104.065468	1.03564038	0	1.96604885	5.66371604	-0.4613368	4.64502981	0.03598508	-6.9077553	22	78	2.23519444	299.666667	26.66666666	0	0	1	0	42.288408	2	-0.4111751
7.1712	289269.396	0.36926943	48.0556259	0.72252798	0	1.970073	5.66736188	-0.9935243	3.87238002	-0.3236161	-6.9077553	23	83	2.23519444	299.111111	26.11111111	0	0	1	0	42.288408	2	-0.4111751
0.0009	0	0	0	0	0	-7.0131158	-6.9077553	-6.9077553	-6.9077553	-6.9077553	-6.9077553	0	92	1.34111667	298	25	0	0	0	0	18.9135952	2	-0.0428739
0.0009	0	0	0	0	0	-7.0131158	-6.9077553	-6.9077553	-6.9077553	-6.9077553	-6.9077553	1	95	1.34111667	297.444444	24.44444444	0	0	0	0	18.9135952	2	-0.0428739
0.0009	0	0	0	0	0	-7.0131158	-6.9077553	-6.9077553	-6.9077553	-6.9077553	-6.9077553	2	96	2.23519444	297.444444	24.44444444	0	0	0	0	18.9135952	2	-0.0428739
0.0009	0	0	0	0	0	-7.0131158	-6.9077553	-6.9077553	-6.9077553	-6.9077553	-6.9077553	3	98	1.78815556	297.444444	24.44444444	0	0	0	0	18.9135952	2	-0.0428739
0.0009	0	0	0	0	0	-7.0131158	-6.9077553	-6.9077553	-6.9077553	-6.9077553	-6.9077553	4	93	3.12927222	298.555556	25.55555555	0	0	0	0	18.9135952	2	-0.0428739
0.0009	0	0	0	0	0	-7.0131158	-6.9077553	-6.9077553	-6.9077553	-6.9077553	-6.9077553	5	80	4.02335	300.777778	27.7777778	0	0	0	0	18.9135952	2	-0.0428739
0.0333	0	0.55434338	86.9034153	2.53521983	0	-3.4021979	-6.9077553	-0.5881686	4.46480884	0.93067472	-6.9077553	6	72	4.47038889	302.444444	29.44444444	0	0	0	0	18.9135952	2	-0.0428739
0.144	272.058849	0.57839166	90.0416905	2.25898507	0	-1.937942	-1.2980679	-0.5457766	4.5002839	0.81535821	-6.9077553	7	62	3.57631111	304.111111	31.11111111	0	0	0	0	18.9135952	2	-0.0428739
0.3393	3212.90859	0.68342499	152.913918	2.27629856	0	-1.0808706	1.16748783	-0.3791762	5.02988168	0.8229899	-6.9077553	8	55	3.12927222	305.777778	32.7777778	0	0	1	0	18.9135952	2	-0.0428739
0.5337	10887.7682	0.88796001	271.153917	2.19343482	0	-0.6279214	2.38773181	-0.117703	5.60269031	0.78592453	-6.9077553	9	50	3.57631111	306.888889	33.88888888	0	0	1	0	18.9135952	2	-0.0428739
0.6705	15301.6797	0.728508	173.342928	1.35899835	0	-0.3997316	2.72802796	-0.315385	5.15527764	0.30748349	-6.9077553	10	46	2.68223333	307.444444	34.4444444	0	0	1	0	18.9135952	2	-0.0428739
0.8415	19568.028	0.71788153	161.554811	1.40754378	0	-0.1725693	2.97394811	-0.3300587	5.08485066	0.34255639	-6.9077553	11	41	2.68223333	309.111111	36.11111111	0	0	1	0	18.9135952	2	-0.0428739
1.1169	29508.705	0.91035412	251.645578	2.30695298	0	0.11055699	3.38471919	-0.0928237	5.52802563	0.83636098	-6.9077553	12	38	3.12927222	309.666667	36.66666666	0	0	1	0	18.9135952	2	-0.0428739
1.4346	39710.5678	0.87658017	229.991147	2.20243822	0	0.36088606	3.68164252	-0.130587	5.43804517	0.79001897	-6.9077553	13	36	3.12927222	309.111111	36.11111111	0	0	1	0	18.9135952	2	-0.0428739
1.728	50904.1136	0.87870555	231.340012	1.82547652	0	0.54696467	3.92996338	-0.128168	5.44389286	0.60238871	-6.9077553	14	38	3.12927222	308.555556	35.5555556	0	0	1	0	18.9135952	2	-0.0428739
2.0412	61864.0059	0.7791121	185.406789	1.5488832	0	0.71353787	4.12495469	-0.2483176	5.22255766	0.43817957	-6.9077553	15	42	1.34111667	308	35	0	0	1	0	18.9135952	2	-0.0428739
2.4201	75921.3033	0.82241263	202.531102	1.70136759	0	0.88380886	4.32971049	-0.1942978	5.3108984	0.53201998	-6.9077553	16	54	1.34111667	304.666667	31.66666666	0	0	1	0	18.9135952	2	-0.0428739
2.7306	90431.6003	0.89844325	229.472192	1.62047844	0	1.00452137	4.50460483	-0.1059793	5.43578621	0.48333835	-6.9077553	17	61	0.89407778	303	30	0	0	1	0	18.9135952	2	-0.0428739
2.934	99698.6319	0.85024383	194.131603	1.43211108	0	1.07636668	4.60216199	-0.1610567	5.26854144	0.35984766	-6.9077553	18	66	1.34111667	301.888889	28.88888888	0	0	1	0	18.9135952	2	-0.0428739
3.0933	107187.461	0.79207624	171.59108	1.40579517	0	1.12923848	4.6745886	-0.2318359	5.14512003	0.34131419	-6.9077553	19	66	1.34111667	301.333333	28.33333333	0	0	1	0	18.9135952	2	-0.0428739
3.2733	115196.627	0.8263176	186.711523	1.5627803	0	1.18579865	4.74664915	-0.1895666	5.22957013	0.44710616	-6.9077553	20	69	1.34111667	300.777778	27.7777778	0	0	1	0	18.9135952	2	-0.0428739
3.4758	124394.905	0.86464654	200.865156	1.66318197	0	1.24582467	4.82346926	-0.1442786	5.3026388	0.50933369	-6.9077553	21	72	1.78815556	299.666667	26.66666666	0	0	1	0	18.9135952	2	-0.0428739
3.6225	131031.273	0.73729252	146.294622	1.3785596	0	1.2871644	4.87544365	-0.3034152	4.98562938	0.32176432	-6.9077553	22	78	2.23519444	299.666667	26.66666666	0	0	1	0	18.9135952	2	-0.0428739
3.663	132610.377	0.41460398	43.9083903	0.68219452	0	1.29828248	4.88742287	-0.8780225	3.7821282	-0.3809757	-6.9077553	23	83	2.23519444	299.111111	26.11111111	0	0	1	0	18.9135952	2	-0.0428739

0.0189	445.111089	0.83338175	193.137488	2.14522552	0	-3.9685934	-0.8071873	-0.1810642	5.26340748	0.76371073	-6.9077553	0	87	2.23519444	299.111111	26.11111111	0	0	0	0	30.519848	2	0.21141735
0.0459	1282.96089	0.63182411	117.173332	1.18142878	0	-3.0812902	0.24994974	-0.4575628	4.76366284	0.16757061	-6.9077553	1	79	2.68223333	300.777778	27.7777778	0	0	0	0	30.519848	2	0.21141735
0.099	2393.52224	0.65274358	124.245531	1.35707829	0	-2.3126354	0.87318373	-0.4250401	4.82226774	0.30607068	-6.9077553	2	71	2.68223333	302.444444	29.44444444	0	0	0	0	30.519848	2	0.21141735
0.2106	3335.72889	0.64497769	128.088812	2.44901546	0	-1.5577947	1.20499095	-0.4369903	4.85273167	0.89609434	-6.9077553	3	65	4.02335	303.555556	30.55555555	0	0	0	1	30.519848	2	0.21141735
0.4041	5668.08421	0.75408162	168.453016	3.00780796	0	-0.9060929	1.73502759	-0.2809294	5.12666281	1.10154397	-6.9077553	4	61	3.12927222	304.666667	31.66666666	0	0	0	1	30.519848	2	0.21141735
0.6813	11483.1269	0.88331545	246.210075	2.79417043	0	-0.3837525	2.44096581	-0.1229414	5.5061892	1.02789308	-6.9077553	5	54	3.57631111	305.777778	32.7777778	0	0	0	1	30.519848	2	0.21141735
1.0089	19412.6025	0.83211828	201.146162	2.58402361	0	0.00886063	2.96597398	-0.1825797	5.30403679	0.94973464	-6.9077553	6	49	4.02335	307.444444	34.4444444	0	0	0	1	30.519848	2	0.21141735
1.2519	27484.2094	0.81765764	201.138194	1.84720436	0	0.2246624	3.31364802	-0.2000893	5.30399718	0.61421455	-6.9077553	7	51	4.02335	306.333333	33.33333333	0	0	0	1	30.519848	2	0.21141735
1.3851	30487.6132	0.64145753	114.853938	1.18799447	0	0.32577234	3.41735328	-0.4424546	4.74366992	0.17310796	-6.9077553	8	50	2.68223333	306.333333	33.33333333	0	0	0	1	30.519848	2	0.21141735
1.5003	33065.2976	0.64392823	116.054459	1.22140479	0	0.40566509	3.49851456	-0.4386162	4.75406817	0.20082006	-6.9077553	9	52	3.12927222	306.333333	33.33333333	0	0	0	1	30.519848	2	0.21141735
1.5633	35502.9352	0.68360568	123.216741	0.97758626	0	0.44679897	3.56964354	-0.3789122	4.81395304	-0.0216463	-6.9077553	10	81	2.23519444	300.222222	27.2222222	0	0	0	1	30.519848	2	0.21141735
1.5849	36467.9375	0.46019876	52.6135203	0.66236679	0	0.46052131	3.59646087	-0.7739262	3.96299213	-0.4104272	-6.9077553	11	89	1.34111667	298	25	0	0	0	1	30.519848	2	0.21141735
1.5993	37169.7578	0.29006884	18.3131866	0.38817173	0	0.46956603	3.61552237	-1.2341955	2.90767599	-0.9437346	-6.9077553	12	94	2.23519444	296.888889	23.88888888	0	0	0	1	30.519848	2	0.21141735
1.611	37696.123	0.2390063	11.7582022	0.30022335	0	0.4768551	3.62958378	-1.4270901	2.4646361	-1.1999033	-6.9077553	13	94	1.34111667	297.444444	24.44444444	0	0	0	1	30.519848	2	0.21141735
1.6227	38222.4908	0.22631695	10.7718682	0.27134369	0	0.48409143	3.64345027	-1.48141	2.37703077	-1.3006904	-6.9077553	14	95	2.23519444	296.888889	23.88888888	0	0	0	1	30.519848	2	0.21141735
1.6398	38880.4449	0.2374433	11.832322	0.31187163	0	0.49457428	3.66051714	-1.4336237	2.47091945	-1.1619623	-6.9077553	15	93	2.23519444	296.888889	23.88888888	0	0	0	1	30.519848	2	0.21141735
1.6479	39231.3562	0.2326821	11.3252123	0.33815723	0	0.49950175	3.66950182	-1.4537937	2.42711971	-1.0812915	-6.9077553	16	95	1.78815556	296.333333	23.33333333	0	0	0	1	30.519848	2	0.21141735
1.6614	39801.5825	0.2281703	11.1139699	0.29751984	0	0.50766062	3.6839318	-1.4732899	2.40829284	-1.2089189	-6.9077553	17	94	2.68223333	296.888889	23.88888888	0	0	0	1	30.519848	2	0.21141735
1.6722	40284.0868	0.24858562	12.943854	0.35610839	0	0.51414012	3.69598135	-1.3879533	2.56069834	-1.0297159	-6.9077553	18	94	2.23519444	296.888889	23.88888888	0	0	0	1	30.519848	2	0.21141735
1.6866	40898.1792	0.26368129	15.3892126	0.44022298	0	0.52271467	3.71111	-1.3292288	2.73373176	-0.8182049	-6.9077553	19	89	3.57631111	298	25	0	0	0	1	30.519848	2	0.21141735
1.7037	41687.7282	0.33005953	23.9752358	0.6059233	0	0.53280236	3.73023078	-1.1054571	3.17706316	-0.4993529	-6.9077553	20	91	4.47038889	298	25	0	0	0	1	30.519848	2	0.21141735
1.7226	42521.1387	0.33827502	25.3883057	0.64689691	0	0.54383478	3.75002485	-1.0809442	3.23432805	-0.4340237	-6.9077553	21	93	4.47038889	296.888889	23.88888888	0	0	0	1	30.519848	2	0.21141735
1.7415	43310.6865	0.30732977	21.1921808	0.60036986	0	0.55474681	3.7684225	-1.1765854	3.05367947	-0.5085451	-6.9077553	22	94	4.91742778	296.333333	23.33333333	0	0	0	1	30.519848	2	0.21141735
1.7766	44144.9708	0.3910287	48.8483417	1.01882318	0	0.57470143	3.78750166	-0.9364202	3.8887409	0.01962926	-6.9077553	23	94	4.47038889	295.777778	22.7777778	0	0	0	1	30.519848	2	0.21141735
0.0171	776.067651	0.93149545	238.502903	1.62510353	0	-4.0686768	-0.2522279	-0.069891	5.47438567	0.48618668	-6.9077553	0	87	2.23519444	299.111111	26.11111111	0	0	0	0	34.0724638	2	0.49277013
0.0315	1214.70545	0.5179624	74.3280869	0.70244932	0	-3.4577677	0.19532452	-0.6559239	4.30850235	-0.3517594	-6.9077553	1	79	2.68223333	300.777778	27.7777778	0	0	0	0	34.0724638	2	0.49277013
0.0684	2412.20192	0.66140119	119.688572	1.07487246	0	-2.6823825	0.88095446	-0.4118839	4.78490149	0.07313192	-6.9077553	2	71	2.68223333	302.444444	29.44444444	0	0	0	0	34.0724638	2	0.49277013
0.117	4473.17008	0.86847009	195.206272	1.29387184	0	-2.1455813	1.49832088	-0.1398713	5.27406193	0.25841173	-6.9077553	3	65	4.02335	303.555556	30.55555555	0	0	0	0	34.0724638	2	0.49277013
0.171	6985.30575	0.86298269	192.848649	1.24978027	0	-1.7660917	1.94395191	-0.1462025	5.26191087	0.22376758	-6.9077553	4	61	3.12927222	304.666667	31.66666666	0	0	0	0	34.0724638	2	0.49277013
0.2277	9281.52303	0.79489681	161.639137	1.1299011	0	-1.4797263	2.22813339	-0.2282857	5.08537249	0.12301475	-6.9077553	5	54	3.57631111	305.777778	32.7777778	0	0	0	0	34.0724638	2	0.49277013
0.3042	12336.4053	0.89010755	225.33704	1.72067045	0	-1.1900699	2.51263573	-0.1152902	5.41760168	0.54329501	-6.9077553	6	49	4.02335	307.444444	34.4444444	0	0	0	1	34.0724638	2	0.49277013
0.4194	17330.4666	0.96669149	265.58902	2.07685447	0	-0.8689302	2.85252373	-0.0328419	5.58195384	0.73133586	-6.9077553	7	51	4.02335	306.333333	33.33333333	0	0	0	1	34.0724638	2	0.49277013
0.6687	27293.2183	1.13438245	368.834781	2.85022369	0	-0.4024197	3.30667489	0.12696955	5.91035151	1.04774827	-6.9077553	8	50	2.68223333	306.333333	33.33333333	0	0	0	1	34.0724638	2	0.49277013
0.9108	33808.6258	0.91461272	232.960299	1.98632949	0	-0.0934319	3.52074555	-0.0881618	5.45087234	0.68679177	-6.9077553	9	52	3.12927222	306.333333	33.33333333	0	0	0	1	34.0724638	2	0.49277013
1.0431	38868.5593	0.81983424	188.88159	1.4550801	0	0.04219705	3.66021141	-0.1974341	5.24112561	0.37574796	-6.9077553	10	81	2.23519444	300.222222	27.2222222	0	0	0	1	34.0724638	2	0.49277013
1.0962	40798.5654	0.44449586	51.6615383	0.84448223	0	0.09184965	3.70867143	-0.8085673	3.94473292	-0.1678481	-6.9077553	11	89	1.34111667	298	25	0	0	0	1	34.0724638	2	0.49277013
1.1403	42640.8444	0.30787532	23.4786252	0.61083058	0	0.13129139	3.75283603	-1.1748176	3.15613303	-0.4912999	-6.9077553	12	94	2.23519444	296.888889	23.88888888	0	0	0	1	34.0724638	2	0.49277013
1.1826	43912.8945	0.44416911	67.3361661	0.83585739	0	0.1677154	3.78223077	-0.8093011	4.20971233	-0.1781016	-6.9077553	13	94	1.34111667	297.444444	24.44444444	0	0	0	1	34.0724638	2	0.49277013
1.2276	45009.4885	0.3905751	53.6086963	0.69137917	0	0.20506104	3.80689554	-0.9375779	3.98172995	-0.3676215	-6.9077553	14	95	2.23519444	296.888889	23.88888888	0	0	0	1	34.0724638	2	0.49277013
1.2447	45623.5806	0.23659754	13.2872772	0.40100268	0	0.21889454	3.82044662	-1.4371771	2.58688223	-0.9112965	-6.9077553	15	93	2.23519444	296.888889	23.88888888	0	0	0	1	34.0724638	2	0.49277013
1.2699	46588.586	0.23746189	13.5404271	0.36651431	0	0.23893816	3.84137704	-1.4335458	2.60575366	-1.000993	-6.9077553	16	95	1.78815556	296.333333	23.33333333	0	0	0	1	34.0724638	2	0.49277013
1.2933	47466.5239	0.39533153	55.8462853	0.64069902	0	0.25719709	3.86004577	-0.9255042	4.02262092	-0.4436359	-6.9077553	17	94	2.68223333	296.888889	23.88888888	0	0	0	1	34.0724638	2	0.49277013
1.3203	48431.5262	0.32676245	33.7091899	0.57142506	0	0.27785898	3.88017162	-1.1154662	3.51780016	-0.5578735	-6.9077553	18	94	2.23519444	296.888889	23.88888888	0	0	0	1	34.0724638	2	0.49277013
1.3518	49615.848	0.29635702	20.14503	0.51667361	0	0.30143704	3.90433045	-1.2128218	3.00300725	-0.6584103	-6.9077553	19	89	3.57631111	298	25	0	0	0	1	34.0724638	2	0.49277013
1.3833	50887.8982	0.26473092	16.2222255	0.45152626	0	0.32447195	3.92964479	-1.3252711	2.78644389	-0.7929095	-6.9077553	20	91	4.47038889	298	25	0	0	0	1	34.0724638	2	0.49277013
1.413	52203.8087	0.23676417	12.6909892	0.44357962	0	0.3457151	3.95517461	-1.436476	2.54097102	-0.8106261	-6.9077553	21	93	4.47038889	296.888889	23.88888888	0	0	0	1	34.0724638	2	0.49277013
1.4526	53739.0425	0.24473401	13.1624562	0.38275658	0	0.37335505	3.98415839	-1.4035056	2.57744452	-0.9577468	-6.9077553	22	94	4.91742778	296.333333	23.33333333	0	0	0	1	34.0724638	2	0.49277013
1.4886	55186.548	0.27624559	18.6730286	0.43115177	0	0.39783608	4.01073735	-1.2828515	2.92713371	-0.8389784	-6.9077553	23	94	4.47038889	295.777778	22.7777778	0	0	0	1	34.0724638	2	0.49277013
0.0252	1451.41211	1.05241783	300.202474	1.9846019	0	-3.6809113	0.3732257	0.05203995	5.70446049	0.68592209	-6.9077553	0	87	2.23519444	299.111111	26.11111111	0	0	0	0	42.288408	2	-0.4111751
0.1035	5618.88225	0.96940236	253.443472	1.85647478	0	-2.2681837	1.72631071	-0.0300445	5.53514475	0.61921792	-6.9077553	1	79	2.68223333	300.777778	27.7777778	0	0	0	0	42.288408	2	-0.4111751
0.2781	14667.2246	1.03817545	302.70083	2.44187597	0	-1.2797745	2.68568357	0.03842756	5.71274826	0.89317602	-6.9077553	2	71	2.68223333	302.444444	29.44444444	0	0	0	0	42.288408	2	-0.4111751
0.5769	30608.9736	1.1029926	350.935665	2.86511194	0	-0.5500863	3.42132589	0.09893324	5.86060576	1.05295639	-6.9077553	3	65	4.02335	303.555556	30.55555555	0	0	1	0	42.288408	2	-0.4111751
0.8658	45638.6905	0.99462577	280.661118	2.00194058	0	-0.1441013	3.82077774	-0.0043838	5.63715152	0.69461639	-6.9077553	4	61	3.12927222	304.666667	31.66666666	0	0	1	0	42.288408	2	-0.4111751
1.26	59896.7365	0.92290674	232.468358	2.17584396	0	0.23111172	4.09263872	-0.0791441	5.44875842	0.7778761	-6.9077553	5	54	3.57631111	305.777778	32.7777778	0	0	1	0	42.288408	2	-0.4111751
1.6893	74318.0863	0.85230561	191.449686	2.04414032	0	0.52431424	4.3083678	-0.1586375	5.25463026	0.7154664	-6.9077553	6	49	4.02335	307.444444	34.4444444	0	0	1	0	42.288408	2	-0.4111751
2.0943	90910.5946	0.84592814	190.831823	1.60972453	0	0.73921937	4.50988755	-0.1661394	5.25139777	0.4766841	-6.9077553	7	51	4.02335	306.333333	33.33333333	0	0	1	0	42.288408	2	-0.4111751
2.5128	107437.088	0.79939844	170.350974	1.32385306	0	0.92139767	4.67691476	-0.2226456	5.13786673	0.28130156	-6.9077553	8	50	2.68223333	306.333333	33.33333333	0	0	1	0	42.288408	2	-0.4111751
2.9826	126277.46	0.82486482	190.757423	1.53579841	0	1.0927954	4.83848946	-0.1913242	5.25100783	0.42970129	-6.9077553	9	52	3.12927222	306.333333	33.33333333	0	0	1	0	42.288408	2	-0.4111751
3.2985	139842.312	0.80934739	184.385884	1.38341329	0	1.19346782	4.9405226	-0.2102923	5.21703618	0.32527643	-6.9077553	10	81	2.23519444	300.222222	27.2222222	0	0	1	0	42.288408	2	-0.4111751
3.4191	145237.548	0.46317465	54.9763949	0.74307183	0	1.22937736	4.97837755	-0.7674944	4.0069221	-0.2956177	-6.9077553	11	89	1.34111667	298	25	0	0	1	0	42.288408	2	-0.4111751
3.5172	149711.653	0.29851745	20.1194206	0.48524489	0	1.25766522	5.00871781	-1.2055826	3.00173525	-0.7210429	-6.9077553	12	94	2.23519444	296.888889	23.88888888	0	0	1	0	42.288408	2	-0.4111751
3.6081	153615.533	0.29519746	19.3032082	0.50120643	0	1.28318132	5.03445945	-1.216729	2.96032311	-0.688744	-6.9077553	13	94	1.34111667	297.444444	24.44444444	0	0	1	0	42.288408	2	-0.4111751

3.6927	157343.961	0.28186169	17.5964602	0.45598067	0	1.3063579	5.0584406	-1.2627972	2.86775459	-0.7831142	-6.9077553	14	95	2.23519444	296.888889	23.88888888	0	0	1	0	42.288408	2	-0.4111751
3.7782	160940.786	0.26875289	16.1423314	0.44658621	0	1.32924771	5.08104272	-1.310249	2.78150705	-0.8038861	-6.9077553	15	93	2.23519444	296.888889	23.88888888	0	0	1	0	42.288408	2	-0.4111751
3.8538	164011.241	0.26473016	15.2948376	0.4414365	0	1.34905967	5.09994107	-1.3252739	2.72758074	-0.8154583	-6.9077553	16	95	1.78815556	296.333333	23.33333333	0	0	1	0	42.288408	2	-0.4111751
3.9303	167388.756	0.2563414	14.1545582	0.39574082	0	1.36871576	5.12032496	-1.3573517	2.65010736	-0.924472	-6.9077553	17	94	2.68223333	296.888889	23.88888888	0	0	1	0	42.288408	2	-0.4111751
3.9942	170152.176	0.26050632	15.0361294	0.40774321	0	1.38484331	5.13669907	-1.3412968	2.71052243	-0.8946682	-6.9077553	18	94	2.23519444	296.888889	23.88888888	0	0	1	0	42.288408	2	-0.4111751
4.0599	172915.596	0.26408488	14.8257287	0.38525067	0	1.40115834	5.15280937	-1.3277052	2.69643155	-0.9512687	-6.9077553	19	89	3.57631111	298	25	0	0	1	0	42.288408	2	-0.4111751
4.1283	175986.061	0.24961981	13.1792048	0.34843688	0	1.4178657	5.17041048	-1.3838182	2.57871607	-1.0514323	-6.9077553	20	91	4.47038889	298	25	0	0	1	0	42.288408	2	-0.4111751
4.1823	178442.434	0.2519633	13.6541616	0.36839975	0	1.43086133	5.18427165	-1.3745109	2.61411759	-0.9958759	-6.9077553	21	93	4.47038889	296.888889	23.88888888	0	0	1	0	42.288408	2	-0.4111751
4.2408	180898.816	0.24486181	12.8700712	0.34779986	0	1.44475193	5.19794337	-1.4029857	2.55498225	-1.053257	-6.9077553	22	94	4.91742778	296.333333	23.33333333	0	0	1	0	42.288408	2	-0.4111751
4.3227	183530.633	0.26971752	19.3205473	0.63763268	0	1.46388021	5.21238704	-1.3066794	2.96122091	-0.4484258	-6.9077553	23	94	4.47038889	295.777778	22.7777778	0	0	1	0	42.288408	2	-0.4111751
0.0072	0	0.50907769	95.335859	2.53406143	0	-4.9336743	-6.9077553	-0.6731922	4.5574165	0.93021787	-6.9077553	0	87	2.23519444	299.111111	26.11111111	0	0	0	0	18.9135952	2	-0.0428739
0.0477	43.8637829	0.47246051	57.8488444	1.62756432	0	-3.0428239	-3.1041244	-0.7476868	4.05785076	0.48769884	-6.9077553	1	79	2.68223333	300.777778	27.7777778	0	0	1	0	18.9135952	2	-0.0428739
0.0963	438.66772	0.52349204	75.548376	1.16008554	0	-2.340287	-0.821736	-0.645325	4.32478643	0.14935538	-6.9077553	2	71	2.68223333	302.444444	29.44444444	0	0	1	0	18.9135952	2	-0.0428739
0.2358	1930.03643	0.64871482	114.331842	2.10722518	0	-1.4447713	0.65805687	-0.4312218	4.73911386	0.74584645	-6.9077553	3	65	4.02335	303.555556	30.55555555	0	0	1	0	18.9135952	2	-0.0428739
0.405	5756.63252	0.68284729	140.187084	1.77215349	0	-0.9038682	1.75052637	-0.3800207	4.94298498	0.57275959	-6.9077553	4	61	3.12927222	304.666667	31.66666666	0	0	1	0	18.9135952	2	-0.0428739
0.6408	11947.0761	0.75785828	175.190482	1.84739021	0	-0.4450379	2.48057027	-0.2759402	5.16587956	0.6143151	-6.9077553	5	54	3.57631111	305.777778	32.7777778	0	0	1	0	18.9135952	2	-0.0428739
0.9963	23063.1215	0.84849707	220.208386	2.41331663	0	-0.0037069	3.13827823	-0.1631108	5.39457885	0.88141628	-6.9077553	6	49	4.02335	307.444444	34.4444444	0	0	1	0	18.9135952	2	-0.0428739
1.4616	38811.1308	0.8584505	226.134097	2.23528356	0	0.37953173	3.65873285	-0.151462	5.42113259	0.80481536	-6.9077553	7	51	4.02335	306.333333	33.33333333	0	0	1	0	18.9135952	2	-0.0428739
1.9233	56993.475	0.85244799	222.669073	1.9004651	0	0.65404246	4.04295433	-0.1584707	5.40569118	0.6426247	-6.9077553	8	50	2.68223333	306.333333	33.33333333	0	0	1	0	18.9135952	2	-0.0428739
2.4327	74273.172	0.81199635	199.073984	1.81018368	0	0.88900175	4.30776327	-0.2070287	5.29368156	0.5939806	-6.9077553	9	52	3.12927222	306.333333	33.33333333	0	0	1	0	18.9135952	2	-0.0428739
2.6748	84672.9521	0.80741775	175.923051	1.42440376	0	0.98387461	4.43880802	-0.2126763	5.17005237	0.35445511	-6.9077553	10	81	2.23519444	300.222222	27.2222222	0	0	1	0	18.9135952	2	-0.0428739
2.7531	88313.6425	0.47338389	56.4098925	0.76056943	0	1.01272755	4.48090592	-0.7457384	4.03266227	-0.2723739	-6.9077553	11	89	1.34111667	298	25	0	0	1	0	18.9135952	2	-0.0428739
2.8089	90813.8797	0.28202657	17.9918429	0.47927324	0	1.03279295	4.50882315	-1.2622145	2.88997406	-0.7334001	-6.9077553	12	94	2.23519444	296.888889	23.88888888	0	0	1	0	18.9135952	2	-0.0428739
2.8647	93445.7029	0.26870681	15.8245082	0.43650501	0	1.05246363	4.53739125	-1.3104198	2.76162308	-0.8266671	-6.9077553	13	94	1.34111667	297.444444	24.44444444	0	0	1	0	18.9135952	2	-0.0428739
2.9169	95858.216	0.2803201	16.8954531	0.43139563	0	1.07052141	4.56288062	-1.2682621	2.82710372	-0.8384143	-6.9077553	14	95	2.23519444	296.888889	23.88888888	0	0	1	0	18.9135952	2	-0.0428739
2.9736	98358.4511	0.26235798	14.7673592	0.41107014	0	1.08977334	4.58862864	-1.334241	2.692487	-0.8865617	-6.9077553	15	93	2.23519444	296.888889	23.88888888	0	0	1	0	18.9135952	2	-0.0428739
3.0384	101253.463	0.26275057	14.8657752	0.39951446	0	1.11133106	4.61763679	-1.3327514	2.69912887	-0.9150054	-6.9077553	16	95	1.78815556	296.333333	23.33333333	0	0	1	0	18.9135952	2	-0.0428739
3.1041	104148.474	0.26213764	15.1139769	0.41768335	0	1.13272382	4.64582712	-1.3350781	2.7156861	-0.8706404	-6.9077553	17	94	2.68223333	296.888889	23.88888888	0	0	1	0	18.9135952	2	-0.0428739
3.1626	106824.164	0.26968486	15.6876737	0.43229655	0	1.15139447	4.67119351	-1.3068	2.75293903	-0.8363329	-6.9077553	18	94	2.23519444	296.888889	23.88888888	0	0	1	0	18.9135952	2	-0.0428739
3.2319	109894.633	0.26214136	15.285375	0.42611818	0	1.1730702	4.69953113	-1.3350639	2.72696191	-0.8506945	-6.9077553	19	89	3.57631111	298	25	0	0	1	0	18.9135952	2	-0.0428739
3.3057	113140.545	0.29073969	19.1338486	0.5038815	0	1.19564825	4.72863964	-1.2318934	2.95151121	-0.6834315	-6.9077553	20	91	4.47038889	298	25	0	0	1	0	18.9135952	2	-0.0428739
3.3876	116605.786	0.2832646	18.0667161	0.48186363	0	1.22012171	4.75880747	-1.2578498	2.89412671	-0.728021	-6.9077553	21	93	4.47038889	296.888889	23.88888888	0	0	1	0	18.9135952	2	-0.0428739
3.4497	119456.934	0.27426894	16.7176885	0.45820988	0	1.23828727	4.7829643	-1.2900067	2.81652717	-0.7782479	-6.9077553	22	94	4.91742778	296.333333	23.33333333	0	0	1	0	18.9135952	2	-0.0428739
3.555	123111.453	0.3529948	43.4741378	0.77414403	0	1.26835506	4.81309819	-1.038473	3.77218923	-0.2547064	-6.9077553	23	94	4.47038889	295.777778	22.7777778	0	0	1	0	18.9135952	2	-0.0428739
0.0009	0	0	0	0	0	-7.0131158	-6.9077553	-6.9077553	-6.9077553	-6.9077553	-6.9077553	0	76	2.68223333	301.888889	28.88888888	0	0	0	0	30.519848	2	0.21141735
0.0027	43.8637829	0.66892934	109.393234	1.14255559	0	-5.9145035	-3.1041244	-0.400583	4.69495819	0.13414235	-6.9077553	1	78	2.68223333	301.333333	28.33333333	0	0	0	0	30.519848	2	0.21141735
0.0063	219.318913	0.40824518	38.7656021	0.56921679	0	-5.0672056	-1.5126792	-0.8934409	3.65755911	-0.5617387	-6.9077553	2	81	3.12927222	300.777778	27.7777778	0	0	0	0	30.519848	2	0.21141735
0.0108	438.637827	0.33562202	24.4237885	0.46714268	0	-4.5282091	-0.821804	-1.0887946	3.19559854	-0.7589822	-6.9077553	3	84	3.12927222	300.777778	27.7777778	0	0	0	0	30.519848	2	0.21141735
0.0144	570.229192	0.28469753	18.7616806	0.60563862	0	-4.2405271	-0.5599648	-1.2528216	2.93186982	-0.499822	-6.9077553	4	86	4.02335	300.222222	27.2222222	0	0	0	0	30.519848	2	0.21141735
0.0189	789.548128	0.28444204	17.1590973	0.42537374	0	-3.9685934	-0.2350287	-1.2537163	2.84258676	-0.852439	-6.9077553	5	88	3.12927222	300.222222	27.2222222	0	0	0	0	30.519848	2	0.21141735
0.0252	1008.86708	0.27579032	17.0496456	0.44908101	0	-3.6809113	0.00981872	-1.284495	2.83618807	-0.7983277	-6.9077553	6	90	2.23519444	299.666667	26.66666666	0	0	0	0	30.519848	2	0.21141735
0.0297	1096.59459	0.30096478	19.1996857	0.4911006	0	-3.5166082	0.09312105	-1.1974449	2.95494599	-0.7090721	-6.9077553	7	90	2.68223333	299.666667	26.66666666	0	0	0	0	30.519848	2	0.21141735
0.0324	1184.32213	0.26948979	15.5207354	0.54212236	0	-3.4295969	0.17001458	-1.3075209	2.74224133	-0.6104206	-6.9077553	8	89	2.68223333	299.666667	26.66666666	0	0	0	0	30.519848	2	0.21141735
0.0333	1228.18592	0.22125983	9.78220367	0.24277847	0	-3.4021979	0.2063521	-1.5039082	2.280667	-1.4114954	-6.9077553	9	88	3.12927222	300.222222	27.2222222	0	0	0	0	30.519848	2	0.21141735
0.0639	1579.0961	0.34101074	33.3975327	1.37196507	0	-2.7504359	0.45748567	-1.0729131	3.50851197	0.31697269	-6.9077553	10	81	4.02335	301.888889	28.88888888	0	0	0	0	30.519848	2	0.21141735
0.1584	1954.87635	0.40212729	43.8410668	1.86075498	0	-1.8426318	0.67083835	-0.9085029	3.78059379	0.62151958	-6.9077553	11	75	5.81150556	301.888889	28.88888888	0	0	0	0	30.519848	2	0.21141735
0.2691	3028.21857	0.61958052	111.221394	2.3691503	0	-1.3126722	1.10830469	-0.4770999	4.71153175	0.86295337	-6.9077553	12	65	4.91742778	304.111111	31.11111111	0	0	0	0	30.519848	2	0.21141735
0.369	4276.11047	0.60769145	98.267367	1.78604401	0	-0.9969586	1.45327766	-0.4964438	4.58770217	0.58056286	-6.9077553	13	57	6.25854444	305.222222	32.2222222	0	0	0	0	30.519848	2	0.21141735
0.5418	10360.8141	0.71667361	146.034698	2.08886083	0	-0.6128583	2.33812733	-0.3317404	4.9838511	0.73709748	-6.9077553	14	52	5.81150556	306.888889	33.88888888	0	0	0	1	30.519848	2	0.21141735
0.7794	19946.4982	0.75492408	165.263642	2.01775614	0	-0.2492309	2.99310373	-0.2798143	5.10754808	0.70248155	-6.9077553	15	50	4.47038889	308	35	0	0	0	1	30.519848	2	0.21141735
1.0782	26922.8849	0.7271933	149.024461	2.37579364	0	0.07529298	3.29301381	-0.3171887	5.00411717	0.86575237	-6.9077553	16	46	5.36446667	308.555556	35.5555556	0	0	0	1	30.519848	2	0.21141735
1.3302	34992.7369	0.69607261	137.435073	1.52954603	0	0.28532931	3.5551691	-0.3608657	4.92315888	0.42562455	-6.9077553	17	48	2.68223333	308.555556	35.5555556	0	0	0	1	30.519848	2	0.21141735
1.548	44447.5575	0.67953756	130.979516	1.3001545	0	0.43696378	3.79433251	-0.3848723	4.87504858	0.26325195	-6.9077553	18	49	3.12927222	307.444444	34.4444444	0	0	0	1	30.519848	2	0.21141735
1.7352	55445.3963	0.71822181	150.236694	1.16077174	0	0.55112268	4.01541672	-0.3295855	5.01221867	0.1499462	-6.9077553	19	52	1.34111667	307.444444	34.4444444	0	0	0	1	30.519848	2	0.21141735
1.8513	62343.048	0.7729048	167.57169	1.2363118	0	0.6158881	4.13266821	-0.2563064	5.12141723	0.21294112	-6.9077553	20	70	3.57631111	301.333333	28.33333333	0	0	0	1	30.519848	2	0.21141735
1.9224	67503.143	0.61824871	99.1560275	0.94315628	0	0.65357441	4.21218897	-0.4792483	4.59670473	-0.0574636	-6.9077553	21	84	2.68223333	299.111111	26.11111111	0	0	0	1	30.519848	2	0.21141735
1.9413	68336.5555	0.39925652	37.9500296	0.64171764	0	0.66335785	4.22445948	-0.9156496	3.63629664	-0.4420498	-6.9077553	22	87	0.44703889	299.111111	26.11111111	0	0	0	1	30.519848	2	0.21141735
1.9566	69082.2418	0.27395557	15.9343082	0.43429565	0	0.67120827	4.23531218	-1.2911458	2.76853729	-0.8317298	-6.9077553	23	83	0.89407778	300.222222	27.2222222	0	0	0	1	30.519848	2	0.21141735
0.0009	0	0	0	0	0	-7.0131158	-6.9077553	-6.9077553	-6.9077553	-6.9077553	-6.9077553	0	76	2.68223333	301.888889	28.88888888	0	0	0	0	34.0724638	2	0.49277013
0.0072	263.182695	0.67303562	111.871477	1.19302586	0	-4.9336743	-1.3311144	-0.3944723	4.71735963	0.17733067	-6.9077553	1	78	2.68223333	301.333333	28.33333333	0	0	0	0	34.0724638	2	0.49277013
0.0171	701.820524	0.51865833	64.4301536	0.82061291	0	-4.0686768	-0.3526537	-0.6545837	4.16559727	-0.1964859	-6.9077553	2	81	3.12927222	300.777778	27.7777778	0	0	0	0	34.0724638	2	0.49277013
0.0261	1052.73081	0.3565186	29.5345154	0.55445395	0	-3.64582	0.05233702	-1.0285679	3.38559345	-0.5879696	-6.9077553	3	84	3.12927222	300.777778	27.7777778	0	0	0	0	34.0724638	2	0.49277013

0.0342	1272.04984	0.31708956	22.0749851	0.49697648	0	-3.3755296	0.24141547	-1.1454223	3.09449037	-0.6972024	-6.9077553	4	86	4.02335	300.222222	27.2222222	0	0	0	0	34.0724638	2	0.49277013
0.0405	1491.36866	0.2085104	8.88909367	0.21736164	0	-3.2064533	0.40036456	-1.5629819	2.18493759	-1.5216027	-6.9077553	5	88	3.12927222	300.222222	27.2222222	0	0	0	0	34.0724638	2	0.49277013
0.0441	1666.82368	0.2500186	12.8475485	0.32756007	0	-3.1212955	0.51151959	-1.3822283	2.55323085	-1.1130356	-6.9077553	6	90	2.23519444	299.666667	26.66666666	0	0	0	0	34.0724638	2	0.49277013
0.0513	1973.87015	0.25426152	13.9391289	0.41817969	0	-2.9700645	0.68050265	-1.3654667	2.63477165	-0.8694556	-6.9077553	7	90	2.68223333	299.666667	26.66666666	0	0	0	0	34.0724638	2	0.49277013
0.0621	2412.50815	0.26421418	15.2823741	0.45078154	0	-2.7790093	0.88108135	-1.3272176	2.72676558	-0.7945565	-6.9077553	8	89	2.68223333	299.666667	26.66666666	0	0	0	0	34.0724638	2	0.49277013
0.0693	2719.55446	0.25338691	13.3657322	0.34637344	0	-2.6693104	1.0008357	-1.3688989	2.59276895	-1.0573549	-6.9077553	9	88	3.12927222	300.222222	27.2222222	0	0	0	0	34.0724638	2	0.49277013
0.1017	4106.66659	0.62144703	112.713752	1.35917483	0	-2.285728	1.41285513	-0.4740967	4.72486031	0.30761325	-6.9077553	10	81	4.02335	301.888889	28.88888888	0	0	0	0	34.0724638	2	0.49277013
0.1719	7298.117	0.85085825	196.58108	1.64266811	0	-1.7608424	1.98775338	-0.1603351	5.28108005	0.4969304	-6.9077553	11	75	5.81150556	301.888889	28.88888888	0	0	0	0	34.0724638	2	0.49277013
0.2655	11615.3971	0.94722432	252.280123	2.06633333	0	-1.3261404	2.45241764	-0.0531642	5.53054403	0.72625953	-6.9077553	12	65	4.91742778	304.111111	31.11111111	0	0	0	0	34.0724638	2	0.49277013
0.4725	21212.3595	1.19244969	431.638111	3.70572669	0	-0.7497175	3.05463115	0.17684801	6.06758985	1.31014919	-6.9077553	13	57	6.25854444	305.222222	32.2222222	0	0	0	1	34.0724638	2	0.49277013
1.0404	41166.5746	1.31652658	508.240689	4.69704818	0	0.03960525	3.71765092	0.27575618	6.2309571	1.54714714	-6.9077553	14	52	5.81150556	306.888889	33.88888888	0	0	0	1	34.0724638	2	0.49277013
1.5876	58529.717	1.14631693	377.936626	2.92033426	0	0.46222344	4.06955169	0.13742611	5.93472917	1.07204045	-6.9077553	15	50	4.47038889	308	35	0	0	0	1	34.0724638	2	0.49277013
2.1789	75608.8423	1.08289215	344.268027	3.02562213	0	0.77882016	4.32558646	0.08055841	5.84142341	1.10744719	-6.9077553	16	46	5.36446667	308.555556	35.5555556	0	0	0	1	34.0724638	2	0.49277013
2.6523	92742.0858	0.97655333	268.09779	2.13325367	0	0.97542719	4.52983315	-0.0227024	5.59135553	0.75811702	-6.9077553	17	48	2.68223333	308.555556	35.5555556	0	0	0	1	34.0724638	2	0.49277013
3.0816	106945.906	0.91472723	228.902794	1.82967685	0	1.12544894	4.6723325	-0.0880367	5.4333018	0.60468576	-6.9077553	18	49	3.12927222	307.444444	34.4444444	0	0	0	1	34.0724638	2	0.49277013
3.5343	121714.664	0.87488885	211.18107	1.67499544	0	1.26251526	4.8016877	-0.1325161	5.35272065	0.51640728	-6.9077553	19	52	1.34111667	307.444444	34.4444444	0	0	0	1	34.0724638	2	0.49277013
3.8187	131650.98	0.88353835	225.061017	1.70037166	0	1.33991005	4.88016192	-0.1226894	5.41637599	0.53143478	-6.9077553	20	70	3.57631111	301.333333	28.33333333	0	0	0	1	34.0724638	2	0.49277013
3.9789	137645.417	0.69062265	129.468201	1.26487235	0	1.3810054	4.9246882	-0.3687148	4.86344303	0.23576149	-6.9077553	21	84	2.68223333	299.111111	26.11111111	0	0	0	1	34.0724638	2	0.49277013
4.0374	139663.15	0.33019224	33.5493089	0.69557425	0	1.39560092	4.93924061	-1.1050563	3.51304607	-0.3615809	-6.9077553	22	87	0.44703889	299.111111	26.11111111	0	0	0	1	34.0724638	2	0.49277013
4.0923	141417.709	0.38070748	53.5713771	0.85736109	0	1.40910716	4.95172505	-0.9631007	3.98103358	-0.1527304	-6.9077553	23	83	0.89407778	300.222222	27.2222222	0	0	0	1	34.0724638	2	0.49277013
0.0009	0	0	0	0	0	-7.0131158	-6.9077553	-6.9077553	-6.9077553	-6.9077553	-6.9077553	0	76	2.68223333	301.888889	28.88888888	0	0	0	0	42.288408	2	-0.4111751
0.009	394.774045	0.69078912	119.01317	1.2680059	0	-4.7105307	-0.9269118	-0.3684741	4.77924256	0.23823384	-6.9077553	1	78	2.68223333	301.333333	28.33333333	0	0	0	0	42.288408	2	-0.4111751
0.036	1710.68768	0.63684572	101.194002	1.19670601	0	-3.3242363	0.53747983	-0.4496588	4.61704937	0.18040807	-6.9077553	2	81	3.12927222	300.777778	27.7777778	0	0	0	0	42.288408	2	-0.4111751
0.0684	3289.78352	0.51607482	64.8061049	0.94172859	0	-2.6823825	1.19112569	-0.6595677	4.17141524	-0.0589769	-6.9077553	3	84	3.12927222	300.777778	27.7777778	0	0	0	0	42.288408	2	-0.4111751
0.0945	4561.83355	0.36096991	29.9751692	0.62939203	0	-2.3591554	1.51794382	-1.0161942	3.40040271	-0.4614134	-6.9077553	4	86	4.02335	300.222222	27.2222222	0	0	0	0	42.288408	2	-0.4111751
0.1179	5658.42808	0.28376112	18.6577055	0.52285136	0	-2.1379185	1.73332284	-1.2561046	2.92631282	-0.6465473	-6.9077553	5	88	3.12927222	300.222222	27.2222222	0	0	0	0	42.288408	2	-0.4111751
0.1404	6711.15823	0.29100822	19.0009729	0.49644501	0	-1.9632598	1.90392054	-1.2309733	2.94454281	-0.6982703	-6.9077553	6	90	2.23519444	299.666667	26.66666666	0	0	0	0	42.288408	2	-0.4111751
0.171	8202.52657	0.28962567	18.3589513	0.48494541	0	-1.7660917	2.10456413	-1.2357192	2.91017174	-0.721659	-6.9077553	7	90	2.68223333	299.666667	26.66666666	0	0	0	0	42.288408	2	-0.4111751
0.1962	9386.84973	0.28672082	17.9731936	0.49848056	0	-1.6286207	2.23941627	-1.2457646	2.88893704	-0.6941866	-6.9077553	8	89	2.68223333	299.666667	26.66666666	0	0	0	0	42.288408	2	-0.4111751
0.2304	11055.2774	0.41820145	53.6921194	0.72450623	0	-1.4679384	2.40299836	-0.8694037	3.98328486	-0.3208856	-6.9077553	9	88	3.12927222	300.222222	27.2222222	0	0	0	0	42.288408	2	-0.4111751
0.4167	17524.8389	0.79226213	183.089598	2.35344574	0	-0.8753887	2.8636763	-0.2316016	5.2099811	0.85630535	-6.9077553	10	81	4.02335	301.888889	28.88888888	0	0	0	0	42.288408	2	-0.4111751
0.6696	28210.2562	0.87836356	220.25057	2.09052882	0	-0.4010748	3.33972106	-0.1285569	5.39477039	0.73789529	-6.9077553	11	75	5.81150556	301.888889	28.88888888	0	0	0	0	42.288408	2	-0.4111751
0.9963	39614.8803	0.8569415	195.009749	2.08709296	0	-0.0037069	3.67923006	-0.1532194	5.27305468	0.73625119	-6.9077553	12	65	4.91742778	304.111111	31.11111111	0	0	1	0	42.288408	2	-0.4111751
1.3716	55090.3532	0.8723389	201.48771	1.87823999	0	0.31597794	4.00899278	-0.1354316	5.30573335	0.63086743	-6.9077553	13	57	6.25854444	305.222222	32.2222222	0	0	1	0	42.288408	2	-0.4111751
1.9278	75169.3864	0.94031295	248.233212	2.84457131	0	0.65637946	4.31975736	-0.0604796	5.5143727	1.04576386	-6.9077553	14	52	5.81150556	306.888889	33.88888888	0	0	1	0	42.288408	2	-0.4111751
2.5695	101166.959	0.92480426	241.46406	2.31369964	0	0.94371133	4.6167821	-0.0770924	5.48672478	0.83927994	-6.9077553	15	50	4.47038889	308	35	0	0	1	0	42.288408	2	-0.4111751
3.2328	130955.765	0.98454245	274.087157	2.30730812	0	1.17334863	4.87486723	-0.0145631	5.61344979	0.83651484	-6.9077553	16	46	5.36446667	308.555556	35.5555556	0	0	1	0	42.288408	2	-0.4111751
3.8943	164141.917	0.99581357	279.819473	2.05920311	0	1.35951395	5.1007375	-0.0031915	5.63414823	0.72280457	-6.9077553	17	48	2.68223333	308.555556	35.5555556	0	0	1	0	42.288408	2	-0.4111751
4.6224	196576.596	0.98974474	282.756644	2.27495093	0	1.53091405	5.28105724	-0.0092984	5.64459015	0.82239796	-6.9077553	18	49	3.12927222	307.444444	34.4444444	0	0	1	0	42.288408	2	-0.4111751
5.1111	217757.057	0.86505925	209.761136	1.55297492	0	1.63141465	5.38338462	-0.143802	5.3459742	0.44081611	-6.9077553	19	52	1.34111667	307.444444	34.4444444	0	0	1	0	42.288408	2	-0.4111751
5.4342	231792.554	0.83963752	195.548311	1.45435709	0	1.69271232	5.44584712	-0.1735947	5.27581258	0.3752513	-6.9077553	20	70	3.57631111	301.333333	28.33333333	0	0	1	0	42.288408	2	-0.4111751
5.6277	239845.826	0.70119175	130.514099	1.20409347	0	1.72770083	5.48000049	-0.3535488	4.87148892	0.18655713	-6.9077553	21	84	2.68223333	299.111111	26.11111111	0	0	1	0	42.288408	2	-0.4111751
5.679	242126.735	0.24718238	13.032944	0.45114875	0	1.73677516	5.48946542	-1.3935914	2.56755703	-0.7937441	-6.9077553	22	87	0.44703889	299.111111	26.11111111	0	0	1	0	42.288408	2	-0.4111751
5.7303	244363.783	0.24878231	13.5431433	0.38839839	0	1.74576789	5.49866212	-1.3871655	2.60595422	-0.9431523	-6.9077553	23	83	0.89407778	300.222222	27.2222222	0	0	1	0	42.288408	2	-0.4111751
0.0009	0	0	0	0	0	-7.0131158	-6.9077553	-6.9077553	-6.9077553	-6.9077553	-6.9077553	0	76	2.68223333	301.888889	28.88888888	0	0	0	0	18.9135952	2	-0.0428739
0.0009	0	0	0	0	0	-7.0131158	-6.9077553	-6.9077553	-6.9077553	-6.9077553	-6.9077553	1	78	2.68223333	301.333333	28.33333333	0	0	0	0	18.9135952	2	-0.0428739
0.0009	0	0	0	0	0	-7.0131158	-6.9077553	-6.9077553	-6.9077553	-6.9077553	-6.9077553	2	81	3.12927222	300.777778	27.7777778	0	0	0	0	18.9135952	2	-0.0428739
0.0009	0	0	0	0	0	-7.0131158	-6.9077553	-6.9077553	-6.9077553	-6.9077553	-6.9077553	3	84	3.12927222	300.777778	27.7777778	0	0	0	0	18.9135952	2	-0.0428739
0.0009	0	0	0	0	0	-7.0131158	-6.9077553	-6.9077553	-6.9077553	-6.9077553	-6.9077553	4	86	4.02335	300.222222	27.2222222	0	0	0	0	18.9135952	2	-0.0428739
0.0009	0	0	0	0	0	-7.0131158	-6.9077553	-6.9077553	-6.9077553	-6.9077553	-6.9077553	5	88	3.12927222	300.222222	27.2222222	0	0	0	0	18.9135952	2	-0.0428739
0.0009	0	0	0	0	0	-7.0131158	-6.9077553	-6.9077553	-6.9077553	-6.9077553	-6.9077553	6	90	2.23519444	299.666667	26.66666666	0	0	0	0	18.9135952	2	-0.0428739
0.0009	0	0	0	0	0	-7.0131158	-6.9077553	-6.9077553	-6.9077553	-6.9077553	-6.9077553	7	90	2.68223333	299.666667	26.66666666	0	0	0	0	18.9135952	2	-0.0428739
0.0009	0	0	0	0	0	-7.0131158	-6.9077553	-6.9077553	-6.9077553	-6.9077553	-6.9077553	8	89	2.68223333	299.666667	26.66666666	0	0	0	0	18.9135952	2	-0.0428739
0.0009	0	0	0	0	0	-7.0131158	-6.9077553	-6.9077553	-6.9077553	-6.9077553	-6.9077553	9	88	3.12927222	300.222222	27.2222222	0	0	0	0	18.9135952	2	-0.0428739
0.0009	0	0	0	0	0	-7.0131158	-6.9077553	-6.9077553	-6.9077553	-6.9077553	-6.9077553	10	81	4.02335	301.888889	28.88888888	0	0	0	0	18.9135952	2	-0.0428739
0.0171	0	0.65739176	114.137939	3.56400977	0	-4.0686768	-6.9077553	-0.4179551	4.73741647	1.2711668	-6.9077553	11	75	5.81150556	301.888889	28.88888888	0	0	0	0	18.9135952	2	-0.0428739
0.0846	191.6869	0.58564077	100.055189	2.29931315	0	-2.469821	-1.6466887	-0.5333426	4.60573192	0.83304527	-6.9077553	12	65	4.91742778	304.111111	31.11111111	0	0	0	0	18.9135952	2	-0.0428739
0.3015	6878.86622	0.8787769	259.474455	2.74701358	0	-1.1989853	1.92859921	-0.1280869	5.55866211	1.01087832	-6.9077553	13	57	6.25854444	305.222222	32.2222222	0	0	1	0	18.9135952	2	-0.0428739
0.5373	13724.8574	0.850979	245.688126	2.7162415	0	-0.6211987	2.61928145	-0.1601934	5.50406702	0.99961721	-6.9077553	14	52	5.81150556	306.888889	33.88888888	0	0	1	0	18.9135952	2	-0.0428739
0.7101	17851.2234	0.72081264	160.68985	1.70588525	0	-0.3423495	2.88212806	-0.3259897	5.07948233	0.53467022	-6.9077553	15	50	4.47038889	308	35	0	0	1	0	18.9135952	2	-0.0428739
0.9522	26184.6055	0.82510319	213.483547	1.93438789	0	-0.0489802	3.26520985	-0.1910356	5.36356445	0.66030777	-6.9077553	16	46	5.36446667	308.555556	35.5555556	0	0	1	0	18.9135952	2	-0.0428739
1.2105	33702.1234	0.75946747	169.461455	1.84561785	0	0.1910335	3.51759052	-0.2738219	5.1326314	0.61335577	-6.9077553	17	48	2.68223333	308.555556	35.5555556	0	0	1	0	18.9135952	2	-0.0428739

1.5129	45594.5019	0.87634205	228.664132	1.94371015	0	0.41402834	3.81980907	-0.1308583	5.43225863	0.66511294	-6.9077553	18	49	3.12927222	307.444444	34.4444444	0	0	1	0	18.9135952	2	-0.0428739
1.7451	54339.3322	0.78153235	178.791	1.38358206	0	0.55681186	3.99526672	-0.24522	5.18622312	0.32539833	-6.9077553	19	52	1.34111667	307.444444	34.4444444	0	0	1	0	18.9135952	2	-0.0428739
1.9251	61842.2636	0.78492249	173.733438	1.39589813	0	0.65497791	4.12460318	-0.2408971	5.15752791	0.33425416	-6.9077553	20	70	3.57631111	301.333333	28.33333333	0	0	1	0	18.9135952	2	-0.0428739
2.0727	68084.3121	0.7256626	139.972216	1.28816223	0	0.72885211	4.22076151	-0.319293	4.94145109	0.25399257	-6.9077553	21	84	2.68223333	299.111111	26.11111111	0	0	1	0	18.9135952	2	-0.0428739
2.1177	70145.908	0.38945808	39.0508423	0.68309761	0	0.75033059	4.25059173	-0.9404347	3.66489005	-0.3796547	-6.9077553	22	87	0.44703889	299.111111	26.11111111	0	0	1	0	18.9135952	2	-0.0428739
2.1654	72251.3721	0.25393952	13.7016026	0.3795211	0	0.7726051	4.28016516	-1.3667289	2.61758578	-0.9662136	-6.9077553	23	83	0.89407778	300.222222	27.2222222	0	0	1	0	18.9135952	2	-0.0428739
0.0171	494.048103	0.8488911	193.815945	1.83573829	0	-4.0686768	-0.7031003	-0.1626471	5.26691413	0.60799133	-6.9077553	0	60	3.57631111	304.666667	31.66666666	0	0	0	0	30.519848	2	0.21141735
0.1458	2628.11126	0.89896409	233.001694	4.25442355	0	-1.9255195	0.96664587	-0.1054004	5.45105001	1.4481943	-6.9077553	1	57	4.91742778	304.111111	31.11111111	0	0	0	0	30.519848	2	0.21141735
0.2529	4973.0682	0.65264117	119.515124	1.34947358	0	-1.3747611	1.60423806	-0.4251968	4.78345129	0.30045533	-6.9077553	2	70	1.78815556	303	30	0	0	0	0	30.519848	2	0.21141735
0.5994	7177.33063	0.79282076	175.905154	3.75514471	0	-0.5118261	1.97106685	-0.2308976	5.16995064	1.32339309	-6.9077553	3	51	4.47038889	306.888889	33.88888888	0	0	0	1	30.519848	2	0.21141735
1.0287	11334.1254	0.81157628	202.301657	2.66657479	0	0.02829587	2.42790635	-0.2075455	5.30976488	0.98116974	-6.9077553	4	50	3.57631111	306.888889	33.88888888	0	0	0	1	30.519848	2	0.21141735
1.6704	18972.6873	0.81991016	193.517422	2.62129954	0	0.51306312	2.94305314	-0.1973416	5.26537271	0.96405162	-6.9077553	5	52	3.57631111	306.888889	33.88888888	0	0	0	1	30.519848	2	0.21141735
2.2608	26047.2637	0.8055045	189.142114	2.49350534	0	0.81571873	3.25995111	-0.2150458	5.24250394	0.91409045	-6.9077553	6	43	4.02335	308	35	0	0	0	1	30.519848	2	0.21141735
2.6145	32256.039	0.75123095	158.742853	1.57972946	0	0.96107287	3.47373628	-0.2847119	5.06729192	0.45788643	-6.9077553	7	48	3.12927222	307.444444	34.4444444	0	0	0	1	30.519848	2	0.21141735
2.9619	40532.4413	0.75534405	168.409185	1.50498781	0	1.08583095	3.70212734	-0.2792589	5.12640258	0.40944903	-6.9077553	8	50	2.23519444	306.888889	33.88888888	0	0	0	1	30.519848	2	0.21141735
3.2022	46487.412	0.76319285	182.195313	1.40325539	0	1.16383807	3.83920308	-0.2689351	5.20508475	0.33950719	-6.9077553	9	57	0.44703889	304.666667	31.66666666	0	0	0	1	30.519848	2	0.21141735
3.276	48155.9539	0.64427683	118.969977	0.76814717	0	1.18662317	3.87446555	-0.4380759	4.77887957	-0.262473	-6.9077553	10	73	0.89407778	301.333333	28.33333333	0	0	0	1	30.519848	2	0.21141735
3.2859	48550.7268	0.43963775	45.7430753	0.55407255	0	1.18964059	3.88262976	-0.8195322	3.82306228	-0.5886564	-6.9077553	11	76	0.44703889	300.222222	27.2222222	0	0	0	1	30.519848	2	0.21141735
3.2958	48945.5049	0.41050716	40.2086737	0.67006475	0	1.19264893	3.89072797	-0.8879289	3.6941076	-0.3988896	-6.9077553	12	75	1.78815556	300.777778	27.7777778	0	0	0	1	30.519848	2	0.21141735
3.303	49164.8203	0.36425245	30.3482094	0.55010962	0	1.19483115	3.89519867	-1.0071665	3.41277047	-0.5958215	-6.9077553	13	76	2.23519444	300.222222	27.2222222	0	0	0	1	30.519848	2	0.21141735
3.3066	49296.4117	0.23247848	12.107275	0.39003572	0	1.19592047	3.89787158	-1.4546653	2.4938891	-0.9389564	-6.9077553	14	79	3.12927222	300.222222	27.2222222	0	0	0	1	30.519848	2	0.21141735
3.3084	49384.1399	0.19983141	8.1242466	0.26533109	0	1.19646469	3.89964957	-1.6052895	2.09497608	-1.323015	-6.9077553	15	83	4.02335	299.666667	26.66666666	0	0	0	1	30.519848	2	0.21141735
3.3084	49384.1399	0	0	0	0	1.19646469	3.89964957	-6.9077553	-6.9077553	-6.9077553	-6.9077553	16	87	4.02335	299.111111	26.11111111	0	0	0	1	30.519848	2	0.21141735
3.3084	49384.1399	0	0	0	0	1.19646469	3.89964957	-6.9077553	-6.9077553	-6.9077553	-6.9077553	17	89	3.57631111	298.555556	25.55555555	0	0	0	1	30.519848	2	0.21141735
3.3084	49384.1399	0	0	0	0	1.19646469	3.89964957	-6.9077553	-6.9077553	-6.9077553	-6.9077553	18	92	4.02335	298.555556	25.55555555	0	0	0	1	30.519848	2	0.21141735
3.3084	49384.1399	0	0	0	0	1.19646469	3.89964957	-6.9077553	-6.9077553	-6.9077553	-6.9077553	19	88	4.91742778	298.555556	25.55555555	0	0	0	1	30.519848	2	0.21141735
3.3084	49384.1399	0	0	0	0	1.19646469	3.89964957	-6.9077553	-6.9077553	-6.9077553	-6.9077553	20	87	4.02335	298.555556	25.55555555	0	0	0	1	30.519848	2	0.21141735
3.3255	49515.7286	0.18946901	10.3172423	0.71239863	0	1.20162004	3.90231056	-1.6582658	2.33391342	-0.3377149	-6.9077553	21	82	4.91742778	299.666667	26.66666666	0	0	0	1	30.519848	2	0.21141735
3.4479	50699.5055	0.41092396	55.6939554	1.12960681	0	1.23776535	3.92593588	-0.8869165	4.01988957	0.12275449	-6.9077553	22	74	5.36446667	301.333333	28.33333333	0	0	0	1	30.519848	2	0.21141735
3.7611	55107.7934	0.54298673	78.394694	1.32265385	0	1.32471147	4.00930929	-0.6088304	4.361769	0.28039598	-6.9077553	23	68	5.36446667	302.444444	29.44444444	0	0	0	1	30.519848	2	0.21141735
0.0432	2044.17409	1.12645405	370.320188	3.29262689	0	-3.1419148	0.71548292	0.11996204	5.9143707	1.19198936	-6.9077553	0	60	3.57631111	304.666667	31.66666666	0	0	0	0	34.0724638	2	0.49277013
0.099	4684.74945	0.88946699	214.468845	1.37112931	0	-2.3126354	1.54452587	-0.1160092	5.36816914	0.31636378	-6.9077553	1	57	4.91742778	304.111111	31.11111111	0	0	0	0	34.0724638	2	0.49277013
0.1728	7402.63707	0.83607557	184.209913	1.29444476	0	-1.7556204	2.00197138	-0.1778409	5.21608137	0.25885408	-6.9077553	2	70	1.78815556	303	30	0	0	0	0	34.0724638	2	0.49277013
0.2547	10018.2621	0.85033425	187.478215	1.91979662	0	-1.3676689	2.30450945	-0.1609505	5.23366799	0.65274001	-6.9077553	3	51	4.47038889	306.888889	33.88888888	0	0	0	1	34.0724638	2	0.49277013
0.3375	13836.3881	0.89679361	213.37846	1.47293738	0	-1.0861898	2.62737421	-0.1078151	5.36307209	0.38793731	-6.9077553	4	50	3.57631111	306.888889	33.88888888	0	0	0	1	34.0724638	2	0.49277013
0.513	22387.3795	1.03432648	295.730689	2.3917446	0	-0.6674794	3.10854205	0.03471682	5.68945259	0.87244108	-6.9077553	5	52	3.57631111	306.888889	33.88888888	0	0	0	1	34.0724638	2	0.49277013
0.8064	35535.7106	1.06596899	312.455258	2.68744421	0	-0.2151754	3.57056626	0.06482191	5.74446448	0.98896266	-6.9077553	6	43	4.02335	308	35	0	0	0	1	34.0724638	2	0.49277013
1.1808	53531.5686	1.04459278	303.17565	2.41152705	0	0.16619217	3.98029023	0.04458398	5.71431564	0.88067477	-6.9077553	7	48	3.12927222	307.444444	34.4444444	0	0	0	1	34.0724638	2	0.49277013
1.5813	68205.4551	0.8992706	223.670506	1.8810396	0	0.45824729	4.22253921	-0.1050599	5.41017848	0.63235608	-6.9077553	8	50	2.23519444	306.888889	33.88888888	0	0	0	1	34.0724638	2	0.49277013
1.9341	81648.9558	0.83340298	192.634646	1.41540699	0	0.6596421	4.40244128	-0.1810388	5.26080056	0.34812337	-6.9077553	9	57	0.44703889	304.666667	31.66666666	0	0	0	1	34.0724638	2	0.49277013
2.1663	91405.5441	0.82723999	185.880148	1.37789716	0	0.77302064	4.51531707	-0.1884523	5.22510748	0.32128402	-6.9077553	10	73	0.89407778	301.333333	28.33333333	0	0	0	1	34.0724638	2	0.49277013
2.2671	94871.01	0.53517015	76.5095476	0.83228329	0	0.81850148	4.55252872	-0.6233037	4.33742861	-0.1823816	-6.9077553	11	76	0.44703889	300.222222	27.2222222	0	0	0	1	34.0724638	2	0.49277013
2.3445	97678.2926	0.42081864	44.6631967	0.58529823	0	0.85207216	4.58168959	-0.8631798	3.79917221	-0.5339267	-6.9077553	12	75	1.78815556	300.777778	27.7777778	0	0	0	1	34.0724638	2	0.49277013
2.3895	99608.2963	0.32357857	27.482959	0.48417213	0	0.87108414	4.6012555	-1.1252277	3.31360252	-0.7232515	-6.9077553	13	76	2.23519444	300.222222	27.2222222	0	0	0	1	34.0724638	2	0.49277013
2.43	101451.698	0.273671	21.7472656	0.49178374	0	0.88789126	4.61959266	-1.2921813	3.07953401	-0.7076849	-6.9077553	14	79	3.12927222	300.222222	27.2222222	0	0	0	1	34.0724638	2	0.49277013
2.4777	103381.702	0.26913123	17.0940886	0.45805884	0	0.90733071	4.63843766	-1.3088474	2.8387912	-0.7785769	-6.9077553	15	83	4.02335	299.666667	26.66666666	0	0	0	1	34.0724638	2	0.49277013
2.5344	105794.211	0.27951452	21.1808268	0.47203012	0	0.92995692	4.66150525	-1.2711298	3.05314359	-0.7485962	-6.9077553	16	87	4.02335	299.111111	26.11111111	0	0	0	1	34.0724638	2	0.49277013
2.5929	108250.591	0.27373613	19.0417574	0.44035064	0	0.95277694	4.68445807	-1.2919442	2.94668684	-0.8179156	-6.9077553	17	89	3.57631111	298.555556	25.55555555	0	0	0	1	34.0724638	2	0.49277013
2.6379	110312.189	0.25143072	15.1216394	0.42107391	0	0.96998315	4.7033235	-1.3766184	2.71619292	-0.8625748	-6.9077553	18	92	4.02335	298.555556	25.55555555	0	0	0	1	34.0724638	2	0.49277013
2.6982	113163.337	0.24285963	13.1411607	0.39725708	0	0.99258488	4.72884107	-1.4111625	2.57582543	-0.9206576	-6.9077553	19	88	4.91742778	298.555556	25.55555555	0	0	0	1	34.0724638	2	0.49277013
2.7675	116102.209	0.32365197	29.8266665	0.52150627	0	1.01794439	4.75447953	-1.1250015	3.39543637	-0.6491183	-6.9077553	20	87	4.02335	298.555556	25.55555555	0	0	0	1	34.0724638	2	0.49277013
2.9583	123979.216	0.56492366	97.3350531	1.13926783	0	1.08461478	4.820122	-0.5692961	4.57816946	0.13126317	-6.9077553	21	82	4.91742778	299.666667	26.66666666	0	0	0	1	34.0724638	2	0.49277013
3.5226	137532.487	0.74774705	189.475877	2.19550213	0	1.25919935	4.92386743	-0.2893541	5.244267	0.78686615	-6.9077553	22	74	5.36446667	301.333333	28.33333333	0	0	0	1	34.0724638	2	0.49277013
4.689	157161.982	1.14562753	447.25082	3.83461733	0	1.54521934	5.05728337	0.13682505	6.10312179	1.3443304	-6.9077553	23	68	5.36446667	302.444444	29.44444444	0	0	0	1	34.0724638	2	0.49277013
0.0432	2974.50781	1.21490219	408.355136	2.46365665	0	-3.1419148	1.09041472	0.19548634	6.01213968	0.90205251	-6.9077553	0	60	3.57631111	304.666667	31.66666666	0	0	0	0	42.288408	2	-0.4111751
0.1422	7569.03671	0.96227854	252.027006	2.06587455	0	-1.9505208	2.02419792	-0.0374127	5.52954022	0.72603759	-6.9077553	1	57	4.91742778	304.111111	31.11111111	0	0	0	0	42.288408	2	-0.4111751
0.324	16576.0764	1.01238908	285.663405	2.07747416	0	-1.1270118	2.8080208	0.01330024	5.65481771	0.73163405	-6.9077553	2	70	1.78815556	303	30	0	0	0	0	42.288408	2	-0.4111751
0.6426	30325.2326	1.03556022	294.875486	2.67960026	0	-0.4422328	3.4120131	0.03590775	5.68655658	0.98604075	-6.9077553	3	51	4.47038889	306.888889	33.88888888	0	0	1	0	42.288408	2	-0.4111751
1.008	47693.5964	0.99799847	273.663832	2.10406224	0	0.00796817	3.86481811	-0.001002	5.61190412	0.74434503	-6.9077553	4	50	3.57631111	306.888889	33.88888888	0	0	1	0	42.288408	2	-0.4111751
1.386	64279.3922	0.92868725	234.386217	1.81065194	0	0.3264219	4.16325464	-0.072907	5.45697452	0.5942391	-6.9077553	5	52	3.57631111	306.888889	33.88888888	0	0	1	0	42.288408	2	-0.4111751
1.926	83569.028	0.93941691	252.917917	2.77738241	0	0.65544531	4.42568494	-0.061432	5.53306895	1.02186889	-6.9077553	6	43	4.02335	308	35	0	0	1	0	42.288408	2	-0.4111751
2.4723	104164.159	0.87091125	214.397537	2.08851908	0	0.90514889	4.6459777	-0.1370676	5.3678366	0.73693393	-6.9077553	7	48	3.12927222	307.444444	34.4444444	0	0	1	0	42.288408	2	-0.4111751

3.042	125972.809	0.84482347	212.951126	1.85293786	0	1.11251519	4.83607402	-0.1674446	5.36106738	0.61731195	-6.9077553	8	50	2.23519444	306.888889	33.88888888	0	0	1	0	42.288408	2	-0.4111751
3.5253	142910.728	0.75631668	171.92944	1.47269512	0	1.25996554	4.96222715	-0.2779738	5.14708998	0.38777293	-6.9077553	9	57	0.44703889	304.666667	31.66666666	0	0	1	0	42.288408	2	-0.4111751
3.8232	155996.553	0.85415622	198.754603	1.50716555	0	1.34108777	5.04984032	-0.1564711	5.29207594	0.41089404	-6.9077553	10	73	0.89407778	301.333333	28.33333333	0	0	1	0	42.288408	2	-0.4111751
3.9744	162751.581	0.55420907	78.5093122	0.94349652	0	1.37987379	5.09223114	-0.5884105	4.36322998	-0.0571033	-6.9077553	11	76	0.44703889	300.222222	27.2222222	0	0	1	0	42.288408	2	-0.4111751
4.0806	167313.4	0.42830199	43.7129155	0.67487723	0	1.40624404	5.11987467	-0.8455947	3.77766648	-0.3917438	-6.9077553	12	75	1.78815556	300.777778	27.7777778	0	0	1	0	42.288408	2	-0.4111751
4.1517	169945.251	0.34433855	27.5364233	0.52391226	0	1.42351789	5.13548222	-1.06323	3.31554593	-0.6445242	-6.9077553	13	76	2.23519444	300.222222	27.2222222	0	0	1	0	42.288408	2	-0.4111751
4.2129	172664.808	0.27159632	19.4153568	0.47712977	0	1.43815125	5.15135798	-1.2997633	2.96611585	-0.7378731	-6.9077553	14	79	3.12927222	300.222222	27.2222222	0	0	1	0	42.288408	2	-0.4111751
4.2651	174814.119	0.26902304	15.9031298	0.43687715	0	1.45046563	5.16372895	-1.309248	2.76657881	-0.8258169	-6.9077553	15	83	4.02335	299.666667	26.66666666	0	0	1	0	42.288408	2	-0.4111751
4.3335	177796.861	0.25630974	14.7122755	0.45190525	0	1.46637553	5.18064729	-1.3574747	2.68875018	-0.7920723	-6.9077553	16	87	4.02335	299.111111	26.11111111	0	0	1	0	42.288408	2	-0.4111751
4.3875	180165.505	0.25188096	14.2259898	0.42275206	0	1.47875959	5.19388145	-1.3748364	2.65514085	-0.8586068	-6.9077553	17	89	3.57631111	298.555556	25.55555555	0	0	1	0	42.288408	2	-0.4111751
4.4514	182753.466	0.2437983	13.406293	0.4226259	0	1.49321865	5.20814353	-1.4073207	2.59579881	-0.8589045	-6.9077553	18	92	4.02335	298.555556	25.55555555	0	0	1	0	42.288408	2	-0.4111751
4.5054	185122.12	0.25510748	14.9138987	0.46813202	0	1.50527668	5.22102112	-1.3621581	2.70236063	-0.7568711	-6.9077553	19	88	4.91742778	298.555556	25.55555555	0	0	1	0	42.288408	2	-0.4111751
4.5594	187534.618	0.32831678	29.6384806	0.56329956	0	1.51719104	5.23396879	-1.1107351	3.38910728	-0.57217	-6.9077553	20	87	4.02335	298.555556	25.55555555	0	0	1	0	42.288408	2	-0.4111751
4.8033	194573.138	0.52806857	97.6041138	1.22965423	0	1.56930318	5.27081326	-0.6366372	4.58092989	0.20754593	-6.9077553	21	82	4.91742778	299.666667	26.66666666	0	0	1	0	42.288408	2	-0.4111751
5.4477	207923.364	0.62412982	130.903184	2.07764286	0	1.6951935	5.33717438	-0.4697959	4.87446564	0.73171521	-6.9077553	22	74	5.36446667	301.333333	28.33333333	0	0	1	0	42.288408	2	-0.4111751
6.1677	222599.579	0.65856201	144.655762	2.00470703	0	1.819326	5.40537904	-0.4161793	4.97436378	0.69599663	-6.9077553	23	68	5.36446667	302.444444	29.44444444	0	0	1	0	42.288408	2	-0.4111751
0.0432	0	0.79738114	182.947183	4.43872265	0	-3.1419148	-6.9077553	-0.2251692	5.20920296	1.49059191	-6.9077553	0	60	3.57631111	304.666667	31.66666666	0	0	0	0	18.9135952	2	-0.0428739
0.2106	47.0606856	0.52512356	74.1340306	2.84991455	0	-1.5577947	-3.0352908	-0.6422192	4.30588817	1.04763984	-6.9077553	1	57	4.91742778	304.111111	31.11111111	0	0	1	0	18.9135952	2	-0.0428739
0.3312	310.243378	0.41444101	48.9152541	1.32387053	0	-1.1050329	-1.1671801	-0.8784147	3.89010973	0.28131474	-6.9077553	2	70	1.78815556	303	30	0	0	1	0	18.9135952	2	-0.0428739
0.5778	1672.60237	0.61509749	136.078881	2.60866931	0	-0.5485275	0.51497841	-0.4843501	4.91324207	0.95922351	-6.9077553	3	51	4.47038889	306.888889	33.88888888	0	0	1	0	18.9135952	2	-0.0428739
0.8541	7707.48425	0.77675298	194.263933	2.13526444	0	-0.157707	2.04232157	-0.2513463	5.26922286	0.75905872	-6.9077553	4	50	3.57631111	306.888889	33.88888888	0	0	1	0	18.9135952	2	-0.0428739
1.1439	14683.366	0.7802632	178.905813	2.01881304	0	0.13444348	2.68678339	-0.2468432	5.18686507	0.70300495	-6.9077553	5	52	3.57631111	306.888889	33.88888888	0	0	1	0	18.9135952	2	-0.0428739
1.4985	23093.8735	0.75068641	167.206337	2.49730051	0	0.40446461	3.13961067	-0.285436	5.11923458	0.91561071	-6.9077553	6	43	4.02335	308	35	0	0	1	0	18.9135952	2	-0.0428739
1.8144	36304.2636	0.88749508	234.553486	1.85602659	0	0.59575483	3.59196273	-0.1182262	5.45768791	0.6189766	-6.9077553	7	48	3.12927222	307.444444	34.4444444	0	0	1	0	18.9135952	2	-0.0428739
2.1213	47231.5631	0.8029838	193.35713	1.55887584	0	0.75202911	3.85508355	-0.2181762	5.26454406	0.44460623	-6.9077553	8	50	2.23519444	306.888889	33.88888888	0	0	1	0	18.9135952	2	-0.0428739
2.4381	59093.2917	0.79624176	195.600253	1.56281731	0	0.89121905	4.07913433	-0.2265973	5.27607816	0.44712983	-6.9077553	9	57	0.44703889	304.666667	31.66666666	0	0	1	0	18.9135952	2	-0.0428739
2.6613	69787.2534	0.88041958	213.856193	1.56928265	0	0.97881473	4.24546571	-0.1262215	5.36530847	0.45125564	-6.9077553	10	73	0.89407778	301.333333	28.33333333	0	0	1	0	18.9135952	2	-0.0428739
2.7999	75928.4923	0.55819504	80.101664	0.930066	0	1.0295837	4.32980518	-0.581257	4.38330911	-0.0714251	-6.9077553	11	76	0.44703889	300.222222	27.2222222	0	0	1	0	18.9135952	2	-0.0428739
2.9142	80928.9675	0.42602951	42.9904381	0.67801126	0	1.06959534	4.39358418	-0.8509022	3.76100098	-0.3871176	-6.9077553	12	75	1.78815556	300.777778	27.7777778	0	0	1	0	18.9135952	2	-0.0428739
3.0015	84920.568	0.36350545	30.480801	0.57185002	0	1.09911216	4.4417281	-1.0092138	3.41712982	-0.5571313	-6.9077553	13	76	2.23519444	300.222222	27.2222222	0	0	1	0	18.9135952	2	-0.0428739
3.078	88473.5387	0.28769087	18.2117819	0.46506101	0	1.12428004	4.48271481	-1.2423988	2.90212365	-0.7634387	-6.9077553	14	79	3.12927222	300.222222	27.2222222	0	0	1	0	18.9135952	2	-0.0428739
3.1428	91412.4089	0.27572004	16.6072574	0.4319192	0	1.14511412	4.51539217	-1.284749	2.80990001	-0.8372042	-6.9077553	15	83	4.02335	299.666667	26.66666666	0	0	1	0	18.9135952	2	-0.0428739
3.2085	94351.2799	0.24461733	13.0253104	0.3697352	0	1.16580354	4.54703544	-1.4039805	2.56697119	-0.9922672	-6.9077553	16	87	4.02335	299.111111	26.11111111	0	0	1	0	18.9135952	2	-0.0428739
3.2643	96807.661	0.2330557	11.5957041	0.34628564	0	1.18304534	4.57273646	-1.4521962	2.45072093	-1.0576077	-6.9077553	17	89	3.57631111	298.555556	25.55555555	0	0	1	0	18.9135952	2	-0.0428739
3.3318	99834.2531	0.22234172	10.5731047	0.33478617	0	1.2035127	4.60352136	-1.4990523	2.35840806	-1.0912807	-6.9077553	18	92	4.02335	298.555556	25.55555555	0	0	1	0	18.9135952	2	-0.0428739
3.4092	103317.591	0.26093252	23.9688153	0.47106951	0	1.22647766	4.63781734	-1.3396684	3.17679534	-0.750629	-6.9077553	19	88	4.91742778	298.555556	25.55555555	0	0	1	0	18.9135952	2	-0.0428739
3.4956	107221.464	0.30554148	26.012914	0.45299677	0	1.25150503	4.67490578	-1.1824022	3.25863155	-0.7896652	-6.9077553	20	87	4.02335	298.555556	25.55555555	0	0	1	0	18.9135952	2	-0.0428739
3.7755	118954.631	0.66032473	132.214228	1.39833761	0	1.32853282	4.77875057	-0.4135103	4.88443111	0.33599899	-6.9077553	21	82	4.91742778	299.666667	26.66666666	0	0	1	0	18.9135952	2	-0.0428739
4.5063	141253.254	0.7394564	164.040333	2.32689212	0	1.50547642	4.95056149	-0.3004885	5.10011842	0.84496319	-6.9077553	22	74	5.36446667	301.333333	28.33333333	0	0	1	0	18.9135952	2	-0.0428739
5.3676	172697.648	0.85695617	216.088787	2.26406449	0	1.68038088	5.15154816	-0.1532023	5.375694	0.81760323	-6.9077553	23	68	5.36446667	302.444444	29.44444444	0	0	1	0	18.9135952	2	-0.0428739
0.009	316.101193	0.82464446	187.854329	1.29412376	0	-4.7105307	-1.1485343	-0.191591	5.23567214	0.25860625	-6.9077553	0	97	3.12927222	293	20	0	0	0	0	30.519848	2	0.21141735
0.0126	491.5563	0.42630666	42.4689941	0.70841759	0	-4.3740585	-0.7081465	-0.8502533	3.74879781	-0.3433109	-6.9077553	1	97	2.68223333	293	20	0	0	0	0	30.519848	2	0.21141735
0.0216	886.33054	0.46739097	51.1271271	0.77492256	0	-3.835062	-0.1195377	-0.7584519	3.93433478	-0.2537026	-6.9077553	2	93	4.47038889	293.555556	20.55555555	0	0	0	0	30.519848	2	0.21141735
0.0351	1237.24072	0.52070821	65.4147135	0.86104457	0	-3.3495541	0.21369159	-0.6506468	4.1807625	-0.1484483	-6.9077553	3	92	2.68223333	293.555556	20.55555555	0	0	0	0	30.519848	2	0.21141735
0.0405	1368.83195	0.3660417	34.799942	0.87511047	0	-3.2064533	0.31468807	-1.0022798	3.54964446	-0.1322631	-6.9077553	4	91	2.68223333	294.111111	21.11111111	0	0	0	0	30.519848	2	0.21141735
0.0603	1632.01462	0.3006452	26.5839455	1.01410796	0	-2.8084232	0.49042777	-1.1985038	3.2803451	0.01499498	-6.9077553	5	91	2.68223333	294.666667	21.66666666	0	0	0	0	30.519848	2	0.21141735
0.1107	1895.19745	0.35567791	36.5589621	1.18183817	0	-2.2009314	0.63985054	-1.0309221	3.59895371	0.16791678	-6.9077553	6	85	3.57631111	296.333333	23.33333333	0	0	0	0	30.519848	2	0.21141735
0.2403	2970.24917	0.543214	85.7911241	2.20484606	0	-1.4258671	1.08898246	-0.6084127	4.45192721	0.79111114	-6.9077553	7	71	5.36446667	298.555556	25.55555555	0	0	0	0	30.519848	2	0.21141735
0.3627	4247.52077	0.65063869	115.21358	2.25417395	0	-1.0141792	1.44657087	-0.428265	4.7467963	0.81322711	-6.9077553	8	67	5.81150556	299.666667	26.66666666	0	0	0	0	30.519848	2	0.21141735
0.549	11562.1946	0.75561347	166.091787	2.21951854	0	-0.5996568	2.44782718	-0.2789028	5.11254659	0.79774075	-6.9077553	9	55	6.25854444	301.888889	28.88888888	0	0	0	1	30.519848	2	0.21141735
0.8505	23641.5972	0.84092844	211.490438	2.74271623	0	-0.1619309	3.16305005	-0.1720603	5.35418452	1.00931329	-6.9077553	10	50	5.81150556	303.555556	30.55555555	0	0	0	1	30.519848	2	0.21141735
1.1484	32948.7308	0.71857326	144.612703	2.16623524	0	0.13836967	3.49498309	-0.3290969	4.97406607	0.77345227	-6.9077553	11	45	5.36446667	304.111111	31.11111111	0	0	0	1	30.519848	2	0.21141735
1.4058	41792.2366	0.68564931	130.957045	1.43603526	0	0.34060654	3.73273452	-0.3759316	4.874877	0.36258215	-6.9077553	12	46	3.57631111	304.111111	31.11111111	0	0	0	1	30.519848	2	0.21141735
1.6335	52148.6449	0.72565644	152.574126	1.42391835	0	0.49072495	3.95411737	-0.3193015	5.0276571	0.35411451	-6.9077553	13	44	4.02335	304.666667	31.66666666	0	0	0	1	30.519848	2	0.21141735
1.8162	61420.1327	0.65894472	125.567455	1.11999241	0	0.59674641	4.11775396	-0.4155992	4.83285107	0.11421437	-6.9077553	14	49	3.12927222	303	30	0	0	0	1	30.519848	2	0.21141735
1.9656	71377.9696	0.65997462	124.000871	0.93060983	0	0.67579754	4.26800328	-0.4140398	4.82029665	-0.0708412	-6.9077553	15	53	3.12927222	301.333333	28.33333333	0	0	0	1	30.519848	2	0.21141735
2.0664	80715.0002	0.69119998	135.03043	0.91311019	0	0.72580796	4.39093682	-0.3678804	4.90550757	-0.0898042	-6.9077553	16	58	1.78815556	299.111111	26.11111111	0	0	0	1	30.519848	2	0.21141735
2.1726	90476.1873	0.66089701	131.053703	0.92887245	0	0.77592461	4.50509774	-0.4126453	4.87561481	-0.0727079	-6.9077553	17	51	2.68223333	298.555556	25.55555555	0	0	0	1	30.519848	2	0.21141735
2.313	101908.798	0.66935779	132.220791	1.01698059	0	0.83854538	4.62408809	-0.3999437	4.88448075	0.01782085	-6.9077553	18	53	2.23519444	297.444444	24.44444444	0	0	0	1	30.519848	2	0.21141735
2.4084	112174.513	0.72676734	154.622218	1.04384455	0	0.87896263	4.72006472	-0.3177739	5.0409913	0.04386812	-6.9077553	19	62	2.68223333	296.333333	23.33333333	0	0	0	1	30.519848	2	0.21141735
2.4696	119257.023	0.74457096	147.56721	1.02657565	0	0.90405619	4.7812894	-0.293605	4.99429051	0.02720229	-6.9077553	20	73	3.12927222	295.777778	22.7777778	0	0	0	1	30.519848	2	0.21141735
2.4903	123204.349	0.49176345	60.7569952	0.47531472	0	0.91240319	4.81385247	-0.707726	4.10689868	-0.7416765	-6.9077553	21	80	2.23519444	295.222222	22.2222222	0	0	0	1	30.519848	2	0.21141735

2.4939	123292.078	0.34367293	28.1030807	0.8849991	0	0.91384775	4.81456427	-1.0651593	3.33591479	-0.1210393	-6.9077553	22	84	3.12927222	295.222222	22.2222222	0	0	0	1	30.519848	2	0.21141735
2.4957	123335.941	0.21643746	9.39053726	0.25949097	0	0.91456925	4.81491997	-1.525844	2.23980899	-1.3451871	-6.9077553	23	88	2.23519444	294.666667	21.66666666	0	0	0	1	30.519848	2	0.21141735
0.0135	686.066585	1.01346118	282.916295	1.90945748	0	-4.3050656	-0.3753241	0.01435762	5.64515461	0.64734273	-6.9077553	0	97	3.12927222	293	20	0	0	0	0	34.0724638	2	0.49277013
0.0234	1080.84062	0.45847524	49.5639815	0.62322088	0	-3.7550193	0.07866387	-0.7776702	3.90328457	-0.471251	-6.9077553	1	97	2.68223333	293	20	0	0	0	0	34.0724638	2	0.49277013
0.0387	1563.34223	0.39701728	39.1668163	0.71982451	0	-3.2519157	0.44746543	-0.9212599	3.6678554	-0.3273596	-6.9077553	2	93	4.47038889	293.555556	20.55555555	0	0	0	0	34.0724638	2	0.49277013
0.0459	1870.38857	0.4055303	38.7410736	0.64084458	0	-3.0812902	0.62668071	-0.9000968	3.65692618	-0.4434091	-6.9077553	3	92	2.68223333	293.555556	20.55555555	0	0	0	0	34.0724638	2	0.49277013
0.0657	2616.07307	0.49603003	60.4586126	0.8228765	0	-2.7226564	0.96205655	-0.6991048	4.10197558	-0.1937346	-6.9077553	4	91	2.68223333	294.111111	21.11111111	0	0	0	0	34.0724638	2	0.49277013
0.099	3895.15168	0.65380586	111.676111	1.29974231	0	-2.3126354	1.35998932	-0.4234165	4.71561177	0.26293511	-6.9077553	5	91	2.68223333	294.666667	21.66666666	0	0	0	0	34.0724638	2	0.49277013
0.1629	6645.88502	0.7701617	163.860654	1.41081066	0	-1.8146188	1.89414833	-0.2598572	5.0990225	0.34487304	-6.9077553	6	85	3.57631111	296.333333	23.33333333	0	0	0	0	34.0724638	2	0.49277013
0.2727	11195.7021	0.93872277	249.490763	2.09713833	0	-1.299383	2.41561928	-0.0621704	5.5194259	0.74105044	-6.9077553	7	71	5.36446667	298.555556	25.55555555	0	0	0	0	34.0724638	2	0.49277013
0.6867	21568.481	1.29881061	528.165999	4.60445397	0	-0.3758578	3.0712794	0.26221857	6.26941252	1.52724125	-6.9077553	8	67	5.81150556	299.666667	26.66666666	0	0	0	1	34.0724638	2	0.49277013
1.5219	41261.3594	1.49674685	708.47535	5.69350144	1	0.41995955	3.71995069	0.40396188	6.56311668	1.73950105	0.0009995	9	55	6.25854444	301.888889	28.88888888	0	0	0	1	34.0724638	2	0.49277013
2.2572	69409.0256	1.20170692	422.286643	3.49139501	1	0.81412511	4.24003132	0.18457479	6.0456867	1.25058775	0.0009995	10	50	5.81150556	303.555556	30.55555555	0	0	0	1	34.0724638	2	0.49277013
2.8098	85590.6333	0.9483074	253.737963	2.01796402	1	1.03311331	4.44958754	-0.0520226	5.53630603	0.70258452	0.0009995	11	45	5.36446667	304.111111	31.11111111	0	0	0	1	34.0724638	2	0.49277013
3.3867	108325.749	0.98967144	271.966142	2.46593457	1	1.219856	4.68515211	-0.0093723	5.60568126	0.90297632	0.0009995	12	46	3.57631111	304.111111	31.11111111	0	0	0	1	34.0724638	2	0.49277013
4.1355	132046.163	0.96050365	260.456994	2.86973454	1	1.41960824	4.88315916	-0.0392569	5.5624416	1.05456794	0.0009995	13	44	4.02335	304.666667	31.66666666	0	0	0	1	34.0724638	2	0.49277013
4.725	147354.662	0.81089957	185.965291	2.04236902	1	1.55286756	4.99284914	-0.2083786	5.22556543	0.71459993	0.0009995	14	49	3.12927222	303	30	0	0	0	1	34.0724638	2	0.49277013
5.1741	160658.447	0.75478147	169.158912	1.43731298	1	1.64366541	5.07928689	-0.280003	5.13084449	0.36347089	0.0009995	15	53	3.12927222	301.333333	28.33333333	0	0	0	1	34.0724638	2	0.49277013
5.4594	171206.369	0.74448652	167.182817	1.28156057	1	1.69733889	5.1428755	-0.2937182	5.11909391	0.24885852	0.0009995	16	58	1.78815556	299.111111	26.11111111	0	0	0	1	34.0724638	2	0.49277013
5.7906	182843.952	0.72602994	168.380414	1.34177797	1	1.75623591	5.20863853	-0.3187876	5.12623172	0.29474058	0.0009995	17	51	2.68223333	298.555556	25.55555555	0	0	0	1	34.0724638	2	0.49277013
6.174	193066.203	0.59475393	109.839688	1.21114353	1	1.82034693	5.26303833	-0.5179276	4.69903102	0.1923903	0.0009995	18	53	2.23519444	297.444444	24.44444444	0	0	0	1	34.0724638	2	0.49277013
6.4116	201450.036	0.74796133	160.986151	1.25768904	1	1.85810885	5.30554635	-0.2890679	5.08132455	0.23007074	0.0009995	19	62	2.68223333	296.333333	23.33333333	0	0	0	1	34.0724638	2	0.49277013
6.5556	207783.555	0.73296146	140.887329	1.16257019	1	1.88031965	5.33650175	-0.3092988	4.94796758	0.15149303	0.0009995	20	73	3.12927222	295.777778	22.7777778	0	0	0	1	34.0724638	2	0.49277013
6.6843	213266.718	0.62556586	100.263167	0.96742334	1	1.89976149	5.36254827	-0.4675014	4.60780837	-0.032086	0.0009995	21	80	2.23519444	295.222222	22.2222222	0	0	0	1	34.0724638	2	0.49277013
6.7338	215372.187	0.23188138	11.9243786	0.41720224	1	1.90713962	5.37237228	-1.457226	2.47866878	-0.8717901	0.0009995	22	84	3.12927222	295.222222	22.2222222	0	0	0	1	34.0724638	2	0.49277013
6.7743	217038.996	0.20446595	8.69087253	0.34456957	1	1.91313604	5.38008165	-1.5824749	2.1623884	-1.0625613	0.0009995	23	88	2.23519444	294.666667	21.66666666	0	0	0	1	34.0724638	2	0.49277013
0.0306	1868.71495	1.09646479	327.955167	2.26884299	0	-3.4867553	0.62578599	0.09300279	5.79287996	0.81971066	-6.9077553	0	97	3.12927222	293	20	0	0	0	0	42.288408	2	-0.4111751
0.0702	3798.72076	0.60890172	93.7304577	1.11459957	0	-2.656407	1.33492758	-0.4944575	4.54043386	0.109392	-6.9077553	1	97	2.68223333	293	20	0	0	0	0	42.288408	2	-0.4111751
0.1143	5816.45505	0.54976868	75.5398298	1.06388427	0	-2.1689287	1.76086289	-0.5964404	4.3246733	0.06286612	-6.9077553	2	93	4.47038889	293.555556	20.55555555	0	0	0	0	42.288408	2	-0.4111751
0.1656	8316.69009	0.55632293	76.3312089	0.98356133	0	-1.79818	2.11838458	-0.5846104	4.33509498	-0.0155591	-6.9077553	3	92	2.68223333	293.555556	20.55555555	0	0	0	0	42.288408	2	-0.4111751
0.2268	11222.5098	0.6590887	116.047464	1.23775841	0	-1.4836867	2.41801067	-0.4153811	4.7540079	0.2141096	-6.9077553	4	91	2.68223333	294.111111	21.11111111	0	0	0	0	42.288408	2	-0.4111751
0.4059	17817.536	0.78194378	181.7129	1.99483839	0	-0.9016485	2.88023926	-0.2446944	5.20243347	0.69106421	-6.9077553	5	91	2.68223333	294.666667	21.66666666	0	0	0	0	42.288408	2	-0.4111751
0.5994	25868.0184	0.82743417	191.617206	1.83192621	0	-0.5118261	3.25304605	-0.1882179	5.25550488	0.60591371	-6.9077553	6	85	3.57631111	296.333333	23.33333333	0	0	0	0	42.288408	2	-0.4111751
0.927	37465.5474	0.86667289	205.997811	2.3059129	0	-0.0758017	3.62344847	-0.1419405	5.3278704	0.83591022	-6.9077553	7	71	5.36446667	298.555556	25.55555555	0	0	1	0	42.288408	2	-0.4111751
1.2942	51943.1832	0.86830887	200.604665	1.93321108	0	0.25789274	3.95016974	-0.1400568	5.30134111	0.65969953	-6.9077553	8	67	5.81150556	299.666667	26.66666666	0	0	1	0	42.288408	2	-0.4111751
1.8486	71438.6858	0.89945013	230.589869	2.57090006	0	0.6144286	4.26885354	-0.1048605	5.44064501	0.94464495	-6.9077553	9	55	6.25854444	301.888889	28.88888888	0	0	1	0	42.288408	2	-0.4111751
2.4408	94189.3313	0.90627504	226.611797	2.24600081	0	0.89232585	4.54531754	-0.0973096	5.42324282	0.80959635	-6.9077553	10	50	5.81150556	303.555556	30.55555555	0	0	1	0	42.288408	2	-0.4111751
3.1545	127563.853	0.99905452	285.793171	2.35900725	0	1.14883	4.84862488	5.422299396	5.65527187	0.85866469	-6.9077553	11	45	5.36446667	304.111111	31.11111111	0	0	1	0	42.288408	2	-0.4111751
3.9429	168168.148	1.04115539	305.765019	2.37310484	0	1.37191649	5.1249703	0.04129106	5.72282016	0.86462046	-6.9077553	12	46	3.57631111	304.111111	31.11111111	0	0	1	0	42.288408	2	-0.4111751
4.7178	202907.94	1.00995263	295.036404	2.4109848	0	1.55134259	5.31275731	0.01089308	5.68710214	0.88044998	-6.9077553	13	44	4.02335	304.666667	31.66666666	0	0	1	0	42.288408	2	-0.4111751
5.2002	222326.332	0.84645342	198.505539	1.53429242	0	1.64869709	5.40415076	-0.1655194	5.29082204	0.42872086	-6.9077553	14	49	3.12927222	303	30	0	0	1	0	42.288408	2	-0.4111751
5.616	239604.475	0.83345527	200.123732	1.47312709	0	1.72561967	5.47899371	-0.1809761	5.29894083	0.38806601	-6.9077553	15	53	3.12927222	301.333333	28.33333333	0	0	1	0	42.288408	2	-0.4111751
5.9553	253521.955	0.8367754	202.867934	1.43315145	0	1.78428158	5.53545437	-0.1770052	5.31256012	0.36057335	-6.9077553	16	58	1.78815556	299.111111	26.11111111	0	0	1	0	42.288408	2	-0.4111751
6.2136	262818.952	0.71970503	157.469893	1.15815251	0	1.82674044	5.5714692	-0.3275253	5.05924064	0.14768914	-6.9077553	17	51	2.68223333	298.555556	25.55555555	0	0	1	0	42.288408	2	-0.4111751
6.5178	271978.404	0.65522825	136.566741	1.08594617	0	1.8745369	5.60572634	-0.4212466	4.91682077	0.08337208	-6.9077553	18	53	2.23519444	297.444444	24.44444444	0	0	1	0	42.288408	2	-0.4111751
6.7122	278602.523	0.71633035	150.546115	1.11273038	0	1.90392677	5.62978971	-0.3322188	5.01427609	0.10771508	-6.9077553	19	62	2.68223333	296.333333	23.33333333	0	0	1	0	42.288408	2	-0.4111751
6.813	282337.181	0.7149851	132.706212	1.10135814	0	1.91883255	5.64310558	-0.3340959	4.88814529	0.09745165	-6.9077553	20	73	3.12927222	295.777778	22.7777778	0	0	1	0	42.288408	2	-0.4111751
6.8796	284837.412	0.5732226	85.2645165	0.95702774	0	1.92856051	5.65192204	-0.5547382	4.44577011	-0.0428785	-6.9077553	21	80	2.23519444	295.222222	22.2222222	0	0	1	0	42.288408	2	-0.4111751
6.8976	285495.365	0.21720614	9.78596878	0.41532135	0	1.93117353	5.6542293	-1.522315	2.28105178	-0.8762978	-6.9077553	22	84	3.12927222	295.222222	22.2222222	0	0	1	0	42.288408	2	-0.4111751
6.9084	285934.004	0.23023701	14.9120076	0.49549286	0	1.93273806	5.65576453	-1.4643121	2.70223383	-0.7001862	-6.9077553	23	88	2.23519444	294.666667	21.66666666	0	0	1	0	42.288408	2	-0.4111751
0.0009	0	0	0	0	0	-7.0131158	-6.9077553	-6.9077553	-6.9077553	-6.9077553	-6.9077553	0	97	3.12927222	293	20	0	0	0	0	18.9135952	2	-0.0428739
0.0009	0	0	0	0	0	-7.0131158	-6.9077553	-6.9077553	-6.9077553	-6.9077553	-6.9077553	1	97	2.68223333	293	20	0	0	0	0	18.9135952	2	-0.0428739
0.0009	0	0	0	0	0	-7.0131158	-6.9077553	-6.9077553	-6.9077553	-6.9077553	-6.9077553	2	93	4.47038889	293.555556	20.55555555	0	0	0	0	18.9135952	2	-0.0428739
0.0009	0	0	0	0	0	-7.0131158	-6.9077553	-6.9077553	-6.9077553	-6.9077553	-6.9077553	3	92	2.68223333	293.555556	20.55555555	0	0	0	0	18.9135952	2	-0.0428739
0.0009	0	0	0	0	0	-7.0131158	-6.9077553	-6.9077553	-6.9077553	-6.9077553	-6.9077553	4	91	2.68223333	294.111111	21.11111111	0	0	0	0	18.9135952	2	-0.0428739
0.0009	0	0	0	0	0	-7.0131158	-6.9077553	-6.9077553	-6.9077553	-6.9077553	-6.9077553	5	91	2.68223333	294.666667	21.66666666	0	0	0	0	18.9135952	2	-0.0428739
0.0009	0	0	0	0	0	-7.0131158	-6.9077553	-6.9077553	-6.9077553	-6.9077553	-6.9077553	6	85	3.57631111	296.333333	23.33333333	0	0	0	0	18.9135952	2	-0.0428739
0.036	0	0.65575839	122.082156	3.22412951	0	-3.3242363	-6.9077553	-0.4204391	4.80470242	1.17097311	-6.9077553	7	71	5.36446667	298.555556	25.55555555	0	0	0	0	18.9135952	2	-0.0428739
0.2016	3577.07742	0.83333289	229.676227	2.88955224	0	-1.6014697	1.27482562	-0.1811228	5.43667496	1.06144757	-6.9077553	8	67	5.81150556	299.666667	26.66666666	0	0	0	0	18.9135952	2	-0.0428739
0.4608	12218.1021	0.89035416	264.546549	2.77964889	0	-0.7747912	2.50300048	-0.1150134	5.57802101	1.02268431	-6.9077553	9	55	6.25854444	301.888889	28.88888888	0	0	1	0	18.9135952	2	-0.0428739
0.6588	16873.2474	0.77642337	205.729368	2.0579662	0	-0.4173353	2.82578864	-0.2517702	5.32656642	0.72220401	-6.9077553	10	50	5.81150556	303.555556	30.55555555	0	0	1	0	18.9135952	2	-0.0428739
0.9027	25595.4627	0.85854596	231.950502	1.96906261	0	-0.102365	3.24245417	-0.151351	5.44652831	0.67806532	-6.9077553	11	45	5.36446667	304.111111	31.11111111	0	0	1	0	18.9135952	2	-0.0428739

1.197	34245.5666	0.80362872	194.055954	2.03811562	0	0.17981843	3.53358631	-0.2173743	5.26815169	0.71251619	-6.9077553	12	46	3.57631111	304.111111	31.11111111	0	0	1	0	18.9135952	2	-0.0428739
1.5174	48114.8624	0.92925322	256.400193	2.05478625	0	0.41699834	3.8736119	-0.0722984	5.54674338	0.72065838	-6.9077553	13	44	4.02335	304.666667	31.66666666	0	0	1	0	18.9135952	2	-0.0428739
1.7757	58572.7643	0.83465406	205.146369	1.57658572	0	0.57419471	4.07028689	-0.1795406	5.32372859	0.45589566	-6.9077553	14	49	3.12927222	303	30	0	0	1	0	18.9135952	2	-0.0428739
2.088	71271.3121	0.84533155	213.253197	1.74286012	0	0.73620667	4.26650792	-0.1668441	5.36248487	0.55610111	-6.9077553	15	53	3.12927222	301.333333	28.33333333	0	0	1	0	18.9135952	2	-0.0428739
2.3841	85020.1356	0.90926558	235.724188	1.69906551	0	0.86882169	4.44289988	-0.0940189	5.46266667	0.53066679	-6.9077553	16	58	1.78815556	299.111111	26.11111111	0	0	1	0	18.9135952	2	-0.0428739
2.646	95724.1681	0.77899988	177.559158	1.42013728	0	0.97304907	4.56148125	-0.2484615	5.17930947	0.35145745	-6.9077553	17	51	2.68223333	298.555556	25.55555555	0	0	1	0	18.9135952	2	-0.0428739
2.9817	107461.164	0.73816867	171.182536	1.54657228	0	1.09249361	4.67713882	-0.3022291	5.14273629	0.43668743	-6.9077553	18	53	2.23519444	297.444444	24.44444444	0	0	1	0	18.9135952	2	-0.0428739
3.2085	117608.42	0.85660626	211.315104	1.55461387	0	1.16580354	4.76736913	-0.1536102	5.35335514	0.44187024	-6.9077553	19	62	2.68223333	296.333333	23.33333333	0	0	1	0	18.9135952	2	-0.0428739
3.4047	127356.756	0.89309594	219.383547	1.72481236	0	1.22515683	4.8470001	-0.1119422	5.39082611	0.54569787	-6.9077553	20	73	3.12927222	295.777778	22.7777778	0	0	1	0	18.9135952	2	-0.0428739
3.5235	132414.959	0.68805209	124.88012	1.24260226	0	1.25945481	4.88594817	-0.3724384	4.82736224	0.21801222	-6.9077553	21	80	2.23519444	295.222222	22.2222222	0	0	1	0	18.9135952	2	-0.0428739
3.564	134169.505	0.24097148	12.8635539	0.45022562	0	1.27088351	4.89911142	-1.4189354	2.55447577	-0.7957878	-6.9077553	22	84	3.12927222	295.222222	22.2222222	0	0	1	0	18.9135952	2	-0.0428739
3.5964	135397.698	0.22854122	11.4390013	0.43695222	0	1.27993335	4.90822375	-1.4716727	2.4371161	-0.8256455	-6.9077553	23	88	2.23519444	294.666667	21.66666666	0	0	1	0	18.9135952	2	-0.0428739
0.0009	0	0	0	0	0	-7.0131158	-6.9077553	-6.9077553	-6.9077553	-6.9077553	-6.9077553	0	82	2.68223333	296.333333	23.33333333	0	0	0	0	30.519848	2	0.21141735
0.0027	43.8637829	0.48303217	54.2776108	0.81800449	0	-5.9145035	-3.1041244	-0.7256039	3.99413024	-0.1996657	-6.9077553	1	86	2.23519444	295.777778	22.7777778	0	0	0	0	30.519848	2	0.21141735
0.0063	219.318913	0.3033177	19.8841209	0.37442389	0	-5.0672056	-1.5126792	-1.1896831	2.98997176	-0.9796995	-6.9077553	2	86	2.68223333	296.333333	23.33333333	0	0	0	0	30.519848	2	0.21141735
0.0072	263.182695	0.23684573	11.3423758	0.26232302	0	-4.9336743	-1.3311144	-1.436133	2.42863394	-1.3343738	-6.9077553	3	86	1.34111667	296.333333	23.33333333	0	0	0	0	30.519848	2	0.21141735
0.0081	307.046481	0.17881404	6.15669537	0.37074494	0	-4.8158912	-1.1775046	-1.7158321	1.81770258	-0.9895473	-6.9077553	4	86	2.23519444	296.333333	23.33333333	0	0	0	0	30.519848	2	0.21141735
0.0117	482.501588	0.23120227	10.9760666	0.27815279	0	-4.4481664	-0.7267007	-1.4601464	2.39580824	-1.275996	-6.9077553	5	87	1.34111667	296.333333	23.33333333	0	0	0	0	30.519848	2	0.21141735
0.0153	614.092962	0.23287977	11.9755306	0.41707224	0	-4.1799025	-0.4859819	-1.4529481	2.48294895	-0.872101	-6.9077553	6	88	0.89407778	295.777778	22.7777778	0	0	0	0	30.519848	2	0.21141735
0.0216	921.139558	0.28325362	16.9322499	0.43733123	0	-3.835062	-0.0810587	-1.2578884	2.82927914	-0.8247804	-6.9077553	7	88	2.23519444	295.777778	22.7777778	0	0	0	0	30.519848	2	0.21141735
0.027	1008.867	0.2684136	15.1229439	0.42297232	0	-3.6119184	0.00981864	-1.3115075	2.71627918	-0.8580871	-6.9077553	8	88	2.68223333	295.777778	22.7777778	0	0	0	0	30.519848	2	0.21141735
0.0387	1359.77726	0.34813631	35.1903311	0.77697314	0	-3.2519157	0.30805605	-1.0522928	3.56079978	-0.2510633	-6.9077553	9	86	2.68223333	296.333333	23.33333333	0	0	0	0	30.519848	2	0.21141735
0.0765	1710.68751	0.33476067	32.9478527	1.0107708	0	-2.5704645	0.53747973	-1.0913567	3.49495644	0.01170206	-6.9077553	10	82	3.57631111	296.888889	23.88888888	0	0	0	0	30.519848	2	0.21141735
0.1404	2413.92475	0.42523631	56.2090944	1.20161535	0	-1.9632598	0.88166813	-0.8527614	4.02909636	0.18449864	-6.9077553	11	75	3.57631111	298.555556	25.55555555	0	0	0	0	30.519848	2	0.21141735
0.2412	3183.43583	0.52268938	76.5917707	1.71097074	0	-1.4221288	1.15827514	-0.6468566	4.3385027	0.53764519	-6.9077553	12	69	4.47038889	299.111111	26.11111111	0	0	0	0	30.519848	2	0.21141735
0.4068	4905.48452	0.71276101	145.026834	2.42313783	0	-0.8994336	1.5905577	-0.3372071	4.97692568	0.88547593	-6.9077553	13	64	4.91742778	300.777778	27.7777778	0	0	0	1	30.519848	2	0.21141735
0.6714	10212.8129	0.8449188	211.819568	2.99091573	0	-0.3983902	2.32374101	-0.1673319	5.35573954	1.09591389	-6.9077553	14	61	4.47038889	301.333333	28.33333333	0	0	0	1	30.519848	2	0.21141735
0.9351	14355.8332	0.79773033	176.947632	2.65001587	0	-0.0671018	2.66422601	-0.2247319	5.17585948	0.97494291	-6.9077553	15	59	4.47038889	301.333333	28.33333333	0	0	0	1	30.519848	2	0.21141735
1.1646	20623.6961	0.76222917	170.179075	1.71232252	0	0.15237768	3.0264892	-0.2701969	5.13685714	0.53843448	-6.9077553	16	54	6.25854444	301.888889	28.88888888	0	0	0	1	30.519848	2	0.21141735
1.3536	25482.2083	0.68037356	142.852548	1.45618388	0	0.30276771	3.23801974	-0.3836446	4.96181997	0.37650572	-6.9077553	17	54	4.02335	301.888889	28.88888888	0	0	0	1	30.519848	2	0.21141735
1.4805	28497.056	0.62024153	115.483184	1.14875631	0	0.39237987	3.34983587	-0.4760353	4.74913358	0.13955002	-6.9077553	18	54	3.12927222	301.888889	28.88888888	0	0	0	1	30.519848	2	0.21141735
1.5597	30928.9644	0.69235962	138.101207	1.05763175	0	0.4444935	3.43172543	-0.3662065	4.92799404	0.05697727	-6.9077553	19	55	3.12927222	300.777778	27.7777778	0	0	0	1	30.519848	2	0.21141735
1.6119	32874.6679	0.70918623	141.169307	0.99919595	0	0.47741361	3.49273281	-0.3422281	4.94996701	0.00019593	-6.9077553	20	62	2.68223333	299.666667	26.66666666	0	0	0	1	30.519848	2	0.21141735
1.6605	34950.8155	0.77069615	157.960775	1.195974	0	0.50711876	3.55397041	-0.2591644	5.06235307	0.1797967	-6.9077553	21	63	3.12927222	298.555556	25.55555555	0	0	0	1	30.519848	2	0.21141735
1.7172	37553.5848	0.80239808	170.827396	1.2429158	0	0.54069506	3.62579547	-0.218905	5.14065952	0.21826431	-6.9077553	22	64	2.23519444	298.555556	25.55555555	0	0	0	1	30.519848	2	0.21141735
1.7667	39300.6178	0.74336208	143.612464	1.11450188	0	0.5691134	3.67126568	-0.2952277	4.96712542	0.10930442	-6.9077553	23	67	2.68223333	298	25	0	0	0	1	30.519848	2	0.21141735
0.0009	0	0	0	0	0	-7.0131158	-6.9077553	-6.9077553	-6.9077553	-6.9077553	-6.9077553	0	82	2.68223333	296.333333	23.33333333	0	0	0	0	34.0724638	2	0.49277013
0.0036	131.59135	0.46261307	49.1747233	0.75920471	0	-5.6268214	-2.0204834	-0.768705	3.89540007	-0.2741675	-6.9077553	1	86	2.23519444	295.777778	22.7777778	0	0	0	0	34.0724638	2	0.49277013
0.0108	438.637827	0.32799208	24.5015678	0.50896353	0	-4.5282091	-0.821804	-1.1117216	3.19877792	-0.6734161	-6.9077553	2	86	2.68223333	296.333333	23.33333333	0	0	0	0	34.0724638	2	0.49277013
0.0135	526.365391	0.25771083	14.8499667	0.47943481	0	-4.3050656	-0.6398616	-1.3520443	2.69806496	-0.7330637	-6.9077553	3	86	1.34111667	296.333333	23.33333333	0	0	0	0	34.0724638	2	0.49277013
0.0171	657.956723	0.23267861	10.935709	0.26286936	0	-4.0686768	-0.4170974	-1.4538086	2.39212493	-1.3323012	-6.9077553	4	86	2.23519444	296.333333	23.33333333	0	0	0	0	34.0724638	2	0.49277013
0.0234	877.275669	0.21953126	9.64947728	0.28079946	0	-3.7550193	-0.1297948	-1.5117158	2.26700737	-1.2665596	-6.9077553	5	87	1.34111667	296.333333	23.33333333	0	0	0	0	34.0724638	2	0.49277013
0.0288	1052.73091	0.21716875	10.7212664	0.36932619	0	-3.5473799	0.05233711	-1.5224864	2.37232255	-0.9933711	-6.9077553	6	88	0.89407778	295.777778	22.7777778	0	0	0	0	34.0724638	2	0.49277013
0.0351	1272.04984	0.20629905	9.11493247	0.25154081	0	-3.3495541	0.24141547	-1.5735928	2.2100237	-1.3761824	-6.9077553	7	88	2.23519444	295.777778	22.7777778	0	0	0	0	34.0724638	2	0.49277013
0.0387	1403.64105	0.20305105	9.19756317	0.24694298	0	-3.2519157	0.33978179	-1.5893851	2.21904729	-1.3945565	-6.9077553	8	88	2.68223333	295.777778	22.7777778	0	0	0	0	34.0724638	2	0.49277013
0.045	1666.82372	0.26922493	16.2753089	0.35556167	0	-3.1010928	0.51151962	-1.3085006	2.78971061	-1.0312481	-6.9077553	9	86	2.68223333	296.333333	23.33333333	0	0	0	0	34.0724638	2	0.49277013
0.0783	2995.26952	0.72013046	138.214606	1.32620126	0	-2.5472077	1.09736802	-0.3269352	4.92881483	0.28307241	-6.9077553	10	82	3.57631111	296.888889	23.88888888	0	0	0	0	34.0724638	2	0.49277013
0.1395	5455.77273	0.77629432	167.91794	1.35759264	0	-1.9696907	1.69685754	-0.2519362	5.12348136	0.30644934	-6.9077553	11	75	3.57631111	298.555556	25.55555555	0	0	0	0	34.0724638	2	0.49277013
0.2196	9343.27608	0.91997408	233.802778	1.6558891	0	-1.5159476	2.23476397	-0.0823234	5.45448221	0.50494181	-6.9077553	12	69	4.47038889	299.111111	26.11111111	0	0	0	0	34.0724638	2	0.49277013
0.5031	17975.902	1.23749109	467.294196	3.78433896	0	-0.6869663	2.88908771	0.21389377	6.14696117	1.33113544	-6.9077553	13	64	4.91742778	300.777778	27.7777778	0	0	0	0	34.0724638	2	0.49277013
1.0944	27324.1942	1.41682571	638.392504	4.87028478	0	0.09020627	3.30780914	0.34912451	6.45895487	1.58335772	-6.9077553	14	61	4.47038889	301.333333	28.33333333	0	0	0	1	34.0724638	2	0.49277013
1.7226	42835.2818	1.18929136	411.123433	3.39457952	0	0.54383478	3.75738545	0.17419811	6.01889593	1.22247444	-6.9077553	15	59	4.47038889	301.333333	28.33333333	0	0	0	1	34.0724638	2	0.49277013
2.4075	66032.6793	1.13732886	395.095434	3.36625489	0	0.87858886	4.1901649	0.12956128	5.97912987	1.21409784	-6.9077553	16	54	6.25854444	301.888889	28.88888888	0	0	0	1	34.0724638	2	0.49277013
2.9304	83966.4982	0.90769919	225.204039	1.77241429	0	1.07513893	4.4304298	-0.0957412	5.41701127	0.57290667	-6.9077553	17	54	4.02335	301.888889	28.88888888	0	0	0	1	34.0724638	2	0.49277013
3.546	103579.824	0.88878449	216.407643	2.19073593	0	1.26582021	4.64035222	-0.116776	5.37716849	0.78469389	-6.9077553	18	54	3.12927222	301.888889	28.88888888	0	0	0	1	34.0724638	2	0.49277013
4.1175	120617.819	0.80054653	187.892495	1.87440753	0	1.41524618	4.79263532	-0.2212123	5.23587529	0.62882598	-6.9077553	19	55	3.12927222	300.777778	27.7777778	0	0	0	1	34.0724638	2	0.49277013
4.3848	131817.117	0.85396604	207.747317	1.52068301	0	1.47814401	4.88142307	-0.1566935	5.33632733	0.41981696	-6.9077553	20	62	2.68223333	299.666667	26.66666666	0	0	0	1	34.0724638	2	0.49277013
4.5972	141256.122	0.85564448	196.321438	1.53595966	0	1.52544742	4.95058179	-0.1547323	5.2797584	0.42980622	-6.9077553	21	63	3.12927222	298.555556	25.55555555	0	0	0	1	34.0724638	2	0.49277013
4.8492	152493.451	0.88205397	209.268485	1.58944157	0	1.57881374	5.02712821	-0.124369	5.34362282	0.4640117	-6.9077553	22	64	2.23519444	298.555556	25.55555555	0	0	0	1	34.0724638	2	0.49277013
5.1201	164454.962	0.85165213	195.406951	1.53293303	0	1.63317397	5.10264282	-0.1594036	5.27508943	0.42783504	-6.9077553	23	67	2.68223333	298	25	0	0	0	1	34.0724638	2	0.49277013
0.0009	0	0	0	0	0	-7.0131158	-6.9077553	-6.9077553	-6.9077553	-6.9077553	-6.9077553	0	82	2.68223333	296.333333	23.33333333	0	0	0	0	42.288408	2	-0.4111751
0.0045	175.455131	0.47037134	51.435276	0.81123424	0	-5.4036779	-1.7346886	-0.7521091	3.94034368	-0.2079665	-6.9077553	1	86	2.23519444	295.777778	22.7777778	0	0	0	0	42.288408	2	-0.4111751

0.0126	570.229192	0.41031782	37.9603509	0.73046112	0	-4.3740585	-0.5599648	-0.8883891	3.63656856	-0.3127112	-6.9077553	2	86	2.68223333	296.333333	23.33333333	0	0	0	0	42.288408	2	-0.4111751
0.0297	1403.64105	0.3135184	21.6763354	0.51099757	0	-3.5166082	0.33978179	-1.1567127	3.07626726	-0.6694354	-6.9077553	3	86	1.34111667	296.333333	23.33333333	0	0	0	0	42.288408	2	-0.4111751
0.0423	2017.73407	0.33709151	24.9313442	0.60123294	0	-3.1629682	0.70247062	-1.0844387	3.21616592	-0.507111	-6.9077553	4	86	2.23519444	296.333333	23.33333333	0	0	0	0	42.288408	2	-0.4111751
0.0639	3070.46484	0.30302564	20.1610819	0.49606498	0	-2.7504359	1.1221546	-1.1906432	3.00380371	-0.6990345	-6.9077553	5	87	1.34111667	296.333333	23.33333333	0	0	0	0	42.288408	2	-0.4111751
0.081	3903.87638	0.26722358	15.7091338	0.42066253	0	-2.5133061	1.36222613	-1.3159344	2.75430597	-0.86355	-6.9077553	6	88	0.89407778	295.777778	22.7777778	0	0	0	0	42.288408	2	-0.4111751
0.1008	4825.01518	0.25736434	14.3463967	0.38842669	0	-2.2946169	1.57402111	-1.3533845	2.66356851	-0.9430796	-6.9077553	7	88	2.23519444	295.777778	22.7777778	0	0	0	0	42.288408	2	-0.4111751
0.1269	6097.0653	0.2958205	20.6546715	0.46274787	0	-2.0643559	1.80797156	-1.2146277	3.02798993	-0.7684143	-6.9077553	8	88	2.68223333	295.777778	22.7777778	0	0	0	0	42.288408	2	-0.4111751
0.2043	10081.2408	0.8057856	187.564294	1.61212282	0	-1.5881658	2.31077554	-0.2146973	5.23412702	0.47817194	-6.9077553	9	86	2.68223333	296.333333	23.33333333	0	0	0	0	42.288408	2	-0.4111751
0.4302	19472.4044	0.89459058	232.437344	2.36774873	0	-0.8435051	2.96904966	-0.1102719	5.448625	0.86236185	-6.9077553	10	82	3.57631111	296.888889	23.88888888	0	0	0	0	42.288408	2	-0.4111751
0.6831	30204.8291	0.87996215	218.079209	1.86696703	0	-0.381114	3.40803492	-0.1267406	5.38486293	0.62485069	-6.9077553	11	75	3.57631111	298.555556	25.55555555	0	0	1	0	42.288408	2	-0.4111751
0.9945	42276.2719	0.85170539	192.239681	1.79238362	0	-0.0055152	3.74424964	-0.1593412	5.25874813	0.58410413	-6.9077553	12	69	4.47038889	299.111111	26.11111111	0	0	1	0	42.288408	2	-0.4111751
1.3446	55204.2662	0.82644606	179.176735	1.6338876	0	0.29609657	4.01105835	-0.1894114	5.18837825	0.49157405	-6.9077553	13	64	4.91742778	300.777778	27.7777778	0	0	1	0	42.288408	2	-0.4111751
1.7919	72383.7804	0.87970609	207.588736	2.00903394	0	0.58327651	4.28199606	-0.1270313	5.33556371	0.69815161	-6.9077553	14	61	4.47038889	301.333333	28.33333333	0	0	1	0	42.288408	2	-0.4111751
2.3553	90866.6426	0.84899122	200.837972	2.1989243	0	0.85666811	4.50940397	-0.1625293	5.30250345	0.78842295	-6.9077553	15	59	4.47038889	301.333333	28.33333333	0	0	1	0	42.288408	2	-0.4111751
3.051	120533.651	0.96345186	270.715172	2.63296126	0	1.11546941	4.79193727	-0.0361954	5.60107094	0.96848889	-6.9077553	16	54	6.25854444	301.888889	28.88888888	0	0	1	0	42.288408	2	-0.4111751
3.8178	158616.66	1.01630255	288.46268	2.33978644	0	1.33967434	5.06649665	0.01715456	5.66456918	0.85048696	-6.9077553	17	54	4.02335	301.888889	28.88888888	0	0	1	0	42.288408	2	-0.4111751
4.527	193867.347	0.97444463	267.490205	2.01214538	0	1.51005947	5.2671793	-0.0248619	5.58908669	0.69969836	-6.9077553	18	54	3.12927222	301.888889	28.88888888	0	0	1	0	42.288408	2	-0.4111751
5.0769	218654.071	0.87615307	221.618377	1.66320741	0	1.62470084	5.38749547	-0.1310738	5.40096139	0.50934898	-6.9077553	19	55	3.12927222	300.777778	27.7777778	0	0	1	0	42.288408	2	-0.4111751
5.4405	235680.447	0.89234305	224.872525	1.62506542	0	1.69387097	5.46248109	-0.1127846	5.41553813	0.48616324	-6.9077553	20	62	2.68223333	299.666667	26.66666666	0	0	1	0	42.288408	2	-0.4111751
5.7024	246751.098	0.80632313	177.227247	1.36280912	0	1.74088714	5.50838418	-0.2140313	5.17743843	0.31028161	-6.9077553	21	63	3.12927222	298.555556	25.55555555	0	0	1	0	42.288408	2	-0.4111751
5.9256	256142.991	0.80416587	169.276556	1.29311076	0	1.77928195	5.54573975	-0.216707	5.13153971	0.25782378	-6.9077553	22	64	2.23519444	298.555556	25.55555555	0	0	1	0	42.288408	2	-0.4111751
6.1389	264777.487	0.77906149	162.691703	1.28537232	0	1.81464557	5.57889358	-0.2483825	5.09186316	0.2518261	-6.9077553	23	67	2.68223333	298	25	0	0	1	0	42.288408	2	-0.4111751
0.0009	0	0	0	0	0	-7.0131158	-6.9077553	-6.9077553	-6.9077553	-6.9077553	-6.9077553	0	82	2.68223333	296.333333	23.33333333	0	0	0	0	18.9135952	2	-0.0428739
0.0009	0	0	0	0	0	-7.0131158	-6.9077553	-6.9077553	-6.9077553	-6.9077553	-6.9077553	1	86	2.23519444	295.777778	22.7777778	0	0	0	0	18.9135952	2	-0.0428739
0.0009	0	0	0	0	0	-7.0131158	-6.9077553	-6.9077553	-6.9077553	-6.9077553	-6.9077553	2	86	2.68223333	296.333333	23.33333333	0	0	0	0	18.9135952	2	-0.0428739
0.0009	0	0	0	0	0	-7.0131158	-6.9077553	-6.9077553	-6.9077553	-6.9077553	-6.9077553	3	86	1.34111667	296.333333	23.33333333	0	0	0	0	18.9135952	2	-0.0428739
0.0009	0	0	0	0	0	-7.0131158	-6.9077553	-6.9077553	-6.9077553	-6.9077553	-6.9077553	4	86	2.23519444	296.333333	23.33333333	0	0	0	0	18.9135952	2	-0.0428739
0.0009	0	0	0	0	0	-7.0131158	-6.9077553	-6.9077553	-6.9077553	-6.9077553	-6.9077553	5	87	1.34111667	296.333333	23.33333333	0	0	0	0	18.9135952	2	-0.0428739
0.0009	0	0	0	0	0	-7.0131158	-6.9077553	-6.9077553	-6.9077553	-6.9077553	-6.9077553	6	88	0.89407778	295.777778	22.7777778	0	0	0	0	18.9135952	2	-0.0428739
0.0009	0	0	0	0	0	-7.0131158	-6.9077553	-6.9077553	-6.9077553	-6.9077553	-6.9077553	7	88	2.23519444	295.777778	22.7777778	0	0	0	0	18.9135952	2	-0.0428739
0.0009	0	0	0	0	0	-7.0131158	-6.9077553	-6.9077553	-6.9077553	-6.9077553	-6.9077553	8	88	2.68223333	295.777778	22.7777778	0	0	0	0	18.9135952	2	-0.0428739
0.0009	0	0	0	0	0	-7.0131158	-6.9077553	-6.9077553	-6.9077553	-6.9077553	-6.9077553	9	86	2.68223333	296.333333	23.33333333	0	0	0	0	18.9135952	2	-0.0428739
0.0009	0	0	0	0	0	-7.0131158	-6.9077553	-6.9077553	-6.9077553	-6.9077553	-6.9077553	10	82	3.57631111	296.888889	23.88888888	0	0	0	0	18.9135952	2	-0.0428739
0.0171	0	0.50952042	70.1668498	1.96600829	0	-4.0686768	-6.9077553	-0.6723246	4.25089023	0.67651376	-6.9077553	11	75	3.57631111	298.555556	25.55555555	0	0	0	0	18.9135952	2	-0.0428739
0.0972	413.094142	0.68432377	132.441582	2.60097306	0	-2.3309846	-0.8816619	-0.3778639	4.88614921	0.95627003	-6.9077553	12	69	4.47038889	299.111111	26.11111111	0	0	0	0	18.9135952	2	-0.0428739
0.27	4561.88883	0.791008	196.22994	2.1853749	0	-1.3093333	1.51795594	-0.2331838	5.27929223	0.78224487	-6.9077553	13	64	4.91742778	300.777778	27.7777778	0	0	1	0	18.9135952	2	-0.0428739
0.4842	12378.2828	0.82898796	225.579832	2.0276779	0	-0.7252572	2.51602433	-0.1863441	5.41867855	0.7073843	-6.9077553	14	61	4.47038889	301.333333	28.33333333	0	0	1	0	18.9135952	2	-0.0428739
0.738	20166.7241	0.8345819	235.088846	2.16000604	0	-0.3038115	3.00408351	-0.1796269	5.45996777	0.77057387	-6.9077553	15	59	4.47038889	301.333333	28.33333333	0	0	1	0	18.9135952	2	-0.0428739
1.125	31850.8999	0.95312564	281.069368	3.40558599	0	0.11778304	3.46109703	-0.0469599	5.63860506	1.22571062	-6.9077553	16	54	6.25854444	301.888889	28.88888888	0	0	1	0	18.9135952	2	-0.0428739
1.4499	44378.9135	0.83903677	207.934297	1.82887991	0	0.37149459	3.79278697	-0.1743096	5.33722696	0.60425034	-6.9077553	17	54	4.02335	301.888889	28.88888888	0	0	1	0	18.9135952	2	-0.0428739
1.8099	59491.1324	0.89422363	230.044219	1.93394165	0	0.5932716	4.08584408	-0.1106817	5.43827589	0.66007717	-6.9077553	18	54	3.12927222	301.888889	28.88888888	0	0	1	0	18.9135952	2	-0.0428739
2.1825	76648.8369	0.91243784	238.085164	1.73512423	0	0.78047101	4.33924748	-0.0905399	5.47263264	0.55165517	-6.9077553	19	55	3.12927222	300.777778	27.7777778	0	0	1	0	18.9135952	2	-0.0428739
2.4336	89526.9746	0.93378736	244.104783	1.71214311	0	0.88937164	4.49455114	-0.0674362	5.49760167	0.53832976	-6.9077553	20	62	2.68223333	299.666667	26.66666666	0	0	1	0	18.9135952	2	-0.0428739
2.637	99768.7113	0.89946929	222.977634	1.6687722	0	0.96964191	4.60286464	-0.1048392	5.40707596	0.51268721	-6.9077553	21	63	3.12927222	298.555556	25.55555555	0	0	1	0	18.9135952	2	-0.0428739
2.8305	109435.083	0.88571955	213.739135	1.60688434	0	1.04045337	4.69534066	-0.1202265	5.36476096	0.47491924	-6.9077553	22	64	2.23519444	298.555556	25.55555555	0	0	1	0	18.9135952	2	-0.0428739
3.0258	118981.204	0.86465989	206.597134	1.66503259	0	1.10717552	4.77897394	-0.1442632	5.33077553	0.51044511	-6.9077553	23	67	2.68223333	298	25	0	0	1	0	18.9135952	2	-0.0428739
0.0153	366.900778	0.76229423	170.034363	1.91099548	0	-4.1799025	-0.999942	-0.2701117	5.13600643	0.64814745	-6.9077553	0	92	4.02335	298	25	0	0	0	0	30.519848	2	0.21141735
0.0459	1026.08643	0.50160632	71.4310877	1.24732545	0	-3.0812902	0.02672609	-0.6879481	4.26874718	0.22180301	-6.9077553	1	81	4.02335	299.666667	26.66666666	0	0	0	0	30.519848	2	0.21141735
0.1098	1821.44825	0.54990038	90.9647819	1.40579492	0	-2.2090947	0.60018079	-0.5962013	4.51048341	0.34131401	-6.9077553	2	75	4.02335	300.777778	27.7777778	0	0	0	0	30.519848	2	0.21141735
0.1836	2699.49666	0.50795806	72.5874678	1.4824911	0	-1.6949958	0.9934357	-0.6753897	4.28480606	0.39439816	-6.9077553	3	63	4.02335	303.555556	30.55555555	0	0	0	0	30.519848	2	0.21141735
0.2754	3235.36808	0.55925964	87.5949851	1.62702987	0	-1.2895307	1.17445174	-0.579355	4.47273516	0.48737061	-6.9077553	4	58	3.12927222	304.666667	31.66666666	0	0	0	0	30.519848	2	0.21141735
0.4203	4539.274	0.66709066	125.421911	2.03113589	0	-0.8667865	1.51298736	-0.4033314	4.83169132	0.7090874	-6.9077553	5	51	3.12927222	305.777778	32.7777778	0	0	0	1	30.519848	2	0.21141735
0.5805	7320.79492	0.6935967	133.223468	1.6807635	0	-0.5438655	1.99085551	-0.3644239	4.89203544	0.51984295	-6.9077553	6	52	2.68223333	305.777778	32.7777778	0	0	0	1	30.519848	2	0.21141735
0.792	12301.4782	0.77628836	172.012384	1.95387664	0	-0.2331939	2.50980072	-0.2519439	5.14757229	0.67032709	-6.9077553	7	58	1.78815556	305.222222	32.2222222	0	0	0	1	30.519848	2	0.21141735
1.0251	15950.4922	0.65411984	123.244706	1.49691949	0	0.02479017	2.76955238	-0.4229371	4.81417997	0.40407714	-6.9077553	8	51	1.78815556	306.333333	33.33333333	0	0	0	1	30.519848	2	0.21141735
1.3437	21282.569	0.74812162	159.087471	2.3538227	0	0.295427	3.05793537	-0.2888539	5.06946047	0.85646544	-6.9077553	9	48	4.91742778	306.333333	33.33333333	0	0	0	1	30.519848	2	0.21141735
1.5399	26946.4083	0.73308822	155.747563	1.42207238	0	0.43171748	3.29388713	-0.3091261	5.04824293	0.35281819	-6.9077553	10	50	4.02335	305.777778	32.7777778	0	0	0	1	30.519848	2	0.21141735
1.6758	30771.1083	0.68013334	130.750776	1.25202912	0	0.51629066	3.4266087	-0.3839972	4.87330069	0.22556391	-6.9077553	11	59	5.81150556	301.333333	28.33333333	0	0	0	1	30.519848	2	0.21141735
1.7505	33564.8174	0.75517742	150.068336	1.0169276	0	0.55990146	3.51350821	-0.2794793	5.01109743	0.0177688	-6.9077553	12	70	2.23519444	299.666667	26.66666666	0	0	0	1	30.519848	2	0.21141735
1.8099	36072.4113	0.7517995	149.461544	1.20982927	0	0.5932716	3.58555607	-0.2839564	5.00704582	0.19130547	-6.9077553	13	71	1.34111667	298.555556	25.55555555	0	0	0	1	30.519848	2	0.21141735
1.8495	37853.3834	0.68154573	119.536932	0.96301885	0	0.61491533	3.63374678	-0.3819257	4.78363374	-0.0366444	-6.9077553	14	77	1.34111667	297.444444	24.44444444	0	0	0	1	30.519848	2	0.21141735
1.863	38379.7482	0.29307175	20.0208211	0.47669913	0	0.62218809	3.64755598	-1.2239315	2.99682274	-0.7387742	-6.9077553	15	82	0.44703889	296.333333	23.33333333	0	0	0	1	30.519848	2	0.21141735

1.8756	38906.1134	0.22558584	10.9098325	0.31557659	0	0.62892861	3.6611771	-1.4846314	2.38975611	-1.1501901	-6.9077553	16	88	0.44703889	295.777778	22.7777778	0	0	0	1	30.519848	2	0.21141735
1.8828	39257.0228	0.22223963	10.7534666	0.39815468	0	0.63276003	3.67015583	-1.4995095	2.37532117	-0.9184063	-6.9077553	17	93	0.44703889	295.777778	22.7777778	0	0	0	1	30.519848	2	0.21141735
1.8927	39651.7983	0.22012812	10.1393939	0.29788529	0	0.63800438	3.68016152	-1.509013	2.31652684	-1.2076954	-6.9077553	18	95	0.89407778	295.777778	22.7777778	0	0	0	1	30.519848	2	0.21141735
1.9062	40178.1636	0.22771063	10.7180593	0.33981883	0	0.64511173	3.69334854	-1.4752977	2.3720234	-1.0764042	-6.9077553	19	95	1.78815556	296.333333	23.33333333	0	0	0	1	30.519848	2	0.21141735
1.917	40529.075	0.22244805	10.4857388	0.32116707	0	0.65076146	3.70204429	-1.4985763	2.35011148	-1.132685	-6.9077553	20	93	1.34111667	297.444444	24.44444444	0	0	0	1	30.519848	2	0.21141735
1.9305	41143.1679	0.22046645	10.5224355	0.3438942	0	0.65777904	3.71708219	-1.5074841	2.35360472	-1.0645176	-6.9077553	21	91	1.34111667	297.444444	24.44444444	0	0	0	1	30.519848	2	0.21141735
1.9368	41362.4859	0.20372805	9.22799138	0.31952892	0	0.66103713	3.72239851	-1.5860728	2.22234977	-1.1377828	-6.9077553	22	93	0.89407778	297.444444	24.44444444	0	0	0	1	30.519848	2	0.21141735
1.9485	41888.8527	0.20961345	9.3401771	0.34297103	0	0.66705985	3.73504362	-1.5577308	2.23443227	-1.0671978	-6.9077553	23	94	2.23519444	297.444444	24.44444444	0	0	0	1	30.519848	2	0.21141735
0.0198	867.999531	1.00078483	285.55878	2.34536434	0	-3.9220733	-0.1404127	0.00178324	5.65445139	0.85286704	-6.9077553	0	92	4.02335	298	25	0	0	0	0	34.0724638	2	0.49277013
0.0387	1525.95623	0.64126369	103.359119	0.96978278	0	-3.2519157	0.42327636	-0.4427563	4.63821919	-0.0296525	-6.9077553	1	81	4.02335	299.666667	26.66666666	0	0	0	0	34.0724638	2	0.49277013
0.1008	4111.18763	0.85668365	203.431542	1.71735006	0	-2.2946169	1.41395516	-0.15352	5.31533446	0.54136456	-6.9077553	2	75	4.02335	300.777778	27.7777778	0	0	0	0	34.0724638	2	0.49277013
0.171	7370.25196	0.90132894	214.277694	1.41003164	0	-1.7660917	1.99758756	-0.1027761	5.36727748	0.34432109	-6.9077553	3	63	4.02335	303.555556	30.55555555	0	0	0	0	34.0724638	2	0.49277013
0.2475	10404.4366	0.89669531	209.771737	1.57743656	0	-1.3963447	2.34232842	-0.1079246	5.34602474	0.45643484	-6.9077553	4	58	3.12927222	304.666667	31.66666666	0	0	0	0	34.0724638	2	0.49277013
0.3969	17145	1.06986816	324.9976	2.78451538	0	-0.9240709	2.84176491	0.06846968	5.78382087	1.02443291	-6.9077553	5	51	3.12927222	305.777778	32.7777778	0	0	0	1	34.0724638	2	0.49277013
0.6165	29237.191	1.17354259	385.282307	2.79313234	0	-0.483697	3.37547576	0.16087878	5.95397892	1.02752163	-6.9077553	6	52	2.68223333	305.777778	32.7777778	0	0	0	1	34.0724638	2	0.49277013
0.9153	42159.3229	1.0521698	306.1835	2.45245233	0	-0.0885034	3.74147956	0.05180447	5.72418786	0.89749615	-6.9077553	7	58	1.78815556	305.222222	32.2222222	0	0	0	1	34.0724638	2	0.49277013
1.1367	48712.4183	0.87169914	210.041254	1.6037646	0	0.12812933	3.88595452	-0.1361644	5.34730872	0.47297708	-6.9077553	8	51	1.78815556	306.333333	33.33333333	0	0	0	1	34.0724638	2	0.49277013
1.7712	56441.4601	1.38831657	613.033245	4.86634599	1	0.57165728	4.03322171	0.32881195	6.4184208	1.58254882	0.0009995	9	48	4.91742778	306.333333	33.33333333	0	0	0	1	34.0724638	2	0.49277013
2.3013	69482.387	0.9992083	281.56311	2.46212069	1	0.83347418	4.24108769	0.00020828	5.64036017	0.90142912	0.0009995	10	50	4.02335	305.777778	32.7777778	0	0	0	1	34.0724638	2	0.49277013
2.8413	88636.9879	0.95910787	266.316745	2.4349821	1	1.04426169	4.48456052	-0.0407096	5.58469013	0.89035	0.0009995	11	59	5.81150556	301.333333	28.33333333	0	0	0	1	34.0724638	2	0.49277013
3.1851	103637.68	0.86022494	199.79708	1.47781772	1	1.15848369	4.64091062	-0.1493996	5.29730725	0.39124293	0.0009995	12	70	2.23519444	299.666667	26.66666666	0	0	0	1	34.0724638	2	0.49277013
3.4686	115561.419	0.7575888	150.056101	1.24626465	1	1.24375105	4.74981081	-0.2762954	5.0110159	0.22095287	0.0009995	13	71	1.34111667	298.555556	25.55555555	0	0	0	1	34.0724638	2	0.49277013
3.6945	124432.689	0.71190938	134.15569	1.24615077	1	1.30684523	4.82377296	-0.338401	4.89900844	0.22086157	0.0009995	14	77	1.34111667	297.444444	24.44444444	0	0	0	1	34.0724638	2	0.49277013
3.7611	126845.206	0.33287141	29.2340022	0.58058615	1	1.32471147	4.84297538	-1.0969994	3.3753667	-0.5419962	0.0009995	15	82	0.44703889	296.333333	23.33333333	0	0	0	1	34.0724638	2	0.49277013
3.8295	129696.344	0.26139731	16.7293251	0.45819841	1	1.34273425	4.86520361	-1.3378955	2.81722295	-0.7782925	0.0009995	16	88	0.44703889	295.777778	22.7777778	0	0	0	1	34.0724638	2	0.49277013
3.8754	131582.494	0.26842848	22.040714	0.48506755	1	1.35464888	4.87964158	-1.3114523	3.09293675	-0.7214077	0.0009995	17	93	0.44703889	295.777778	22.7777778	0	0	0	1	34.0724638	2	0.49277013
3.9258	133205.461	0.31280518	36.7722691	0.57887977	1	1.36757015	4.89190026	-1.1589829	3.6047712	-0.5449345	0.0009995	18	95	0.89407778	295.777778	22.7777778	0	0	0	1	34.0724638	2	0.49277013
3.978	134652.951	0.37399026	49.7617356	0.59673316	1	1.38077918	4.90270816	-0.9808552	3.90726642	-0.5146108	0.0009995	19	95	1.78815556	296.333333	23.33333333	0	0	0	1	34.0724638	2	0.49277013
4.0248	136012.734	0.33083578	39.76116	0.52864361	1	1.39247522	4.91275586	-1.1031151	3.68291571	-0.6355509	0.0009995	20	93	1.34111667	297.444444	24.44444444	0	0	0	1	34.0724638	2	0.49277013
4.0572	137328.655	0.25195013	19.0449948	0.43893173	1	1.40049308	4.92238428	-1.3745629	2.94685683	-0.8211357	0.0009995	21	91	1.34111667	297.444444	24.44444444	0	0	0	1	34.0724638	2	0.49277013
4.0806	138293.662	0.19832536	8.65190711	0.30867738	1	1.40624404	4.92938664	-1.6128168	2.15789535	-1.1722242	0.0009995	22	93	0.89407778	297.444444	24.44444444	0	0	0	1	34.0724638	2	0.49277013
4.1274	140048.199	0.28473801	31.4957604	0.49715856	1	1.41764767	4.94199379	-1.2526799	3.4498847	-0.6968369	0.0009995	23	94	2.23519444	297.444444	24.44444444	0	0	0	1	34.0724638	2	0.49277013
0.0333	2155.65925	1.13666121	358.957682	2.44485177	0	-3.4021979	0.76856038	0.12897459	5.88320729	0.89439343	-6.9077553	0	92	4.02335	298	25	0	0	0	0	42.288408	2	-0.4111751
0.1287	6086.16204	0.90277042	221.780734	2.70828218	0	-2.0502712	1.80618197	-0.1011799	5.40169372	0.99668372	-6.9077553	1	81	4.02335	299.666667	26.66666666	0	0	0	0	42.288408	2	-0.4111751
0.3555	15720.4578	1.02083697	289.332868	3.16410416	0	-1.03423	2.75502652	0.02160195	5.66758127	1.15218596	-6.9077553	2	75	4.02335	300.777778	27.7777778	0	0	0	0	42.288408	2	-0.4111751
0.6336	28675.8581	0.98230593	259.387717	2.12506597	0	-0.4563374	3.35609046	-0.016835	5.55832778	0.75427331	-6.9077553	3	63	4.02335	303.555556	30.55555555	0	0	0	0	42.288408	2	-0.4111751
0.9279	41066.011	0.90942924	223.180715	1.81230196	0	-0.0748313	3.71520515	-0.0938391	5.4079863	0.59514947	-6.9077553	4	58	3.12927222	304.666667	31.66666666	0	0	0	0	42.288408	2	-0.4111751
1.2969	56669.2483	0.89231017	215.921646	1.75899122	0	0.2599768	4.03724935	-0.1128214	5.37492022	0.56530882	-6.9077553	5	51	3.12927222	305.777778	32.7777778	0	0	1	0	42.288408	2	-0.4111751
1.7757	74687.1372	0.90360392	226.117099	2.04926501	0	0.57419471	4.31332127	-0.1002581	5.42105743	0.71796906	-6.9077553	6	52	2.68223333	305.777778	32.7777778	0	0	1	0	42.288408	2	-0.4111751
2.2644	91959.207	0.83987579	194.845563	1.82902884	0	0.81730982	4.52135595	-0.1733113	5.27221239	0.60433172	-6.9077553	7	58	1.78815556	305.222222	32.2222222	0	0	1	0	42.288408	2	-0.4111751
2.7558	111003.656	0.86927432	209.198847	1.76722801	0	1.01370778	4.70957214	-0.1389468	5.34329	0.56997792	-6.9077553	8	51	1.78815556	306.333333	33.33333333	0	0	1	0	42.288408	2	-0.4111751
3.3597	137220.087	0.94587801	249.603367	2.08174509	0	1.21185168	4.9215934	-0.054585	5.51987713	0.73368678	-6.9077553	9	48	4.91742778	306.333333	33.33333333	0	0	1	0	42.288408	2	-0.4111751
4.077	173964.539	1.00592891	290.089868	2.16201533	0	1.40536142	5.15885723	0.00690502	5.67019421	0.77150324	-6.9077553	10	50	4.02335	305.777778	32.7777778	0	0	1	0	42.288408	2	-0.4111751
4.6989	206579.601	1.00918239	307.210361	2.3750507	0	1.54732844	5.33069066	0.0101309	5.72753598	0.86543974	-6.9077553	11	59	5.81150556	301.333333	28.33333333	0	0	1	0	42.288408	2	-0.4111751
5.0184	220990.216	0.860785	199.557262	1.46941365	0	1.61311116	5.39812296	-0.1487495	5.29610624	0.38554376	-6.9077553	12	70	2.23519444	299.666667	26.66666666	0	0	1	0	42.288408	2	-0.4111751
5.2614	231413.388	0.78363128	161.873756	1.33155065	0	1.66039715	5.44420999	-0.2425414	5.08682292	0.28709489	-6.9077553	13	71	1.34111667	298.555556	25.55555555	0	0	1	0	42.288408	2	-0.4111751
5.4495	238682.054	0.72309693	136.9054	1.25207038	0	1.69552386	5.47513654	-0.32283	4.91929748	0.22559684	-6.9077553	14	77	1.34111667	297.444444	24.44444444	0	0	1	0	42.288408	2	-0.4111751
5.4999	240699.777	0.3740701	36.1776341	0.6383543	0	1.70472991	5.48355457	-0.9806423	3.58846873	-0.4472965	-6.9077553	15	82	0.44703889	296.333333	23.33333333	0	0	1	0	42.288408	2	-0.4111751
5.5386	242191.151	0.20689	9.16317323	0.36219424	0	1.71174176	5.48973142	-1.5707462	2.21530167	-1.0128175	-6.9077553	16	88	0.44703889	295.777778	22.7777778	0	0	1	0	42.288408	2	-0.4111751
5.5728	243682.524	0.19362073	7.77025805	0.34661634	0	1.71789762	5.49587035	-1.6367026	2.05043206	-1.0566559	-6.9077553	17	93	0.44703889	295.777778	22.7777778	0	0	1	0	42.288408	2	-0.4111751
5.6079	245305.456	0.19939996	8.32895523	0.26666228	0	1.72417632	5.50250827	-1.6074401	2.11985808	-1.3180292	-6.9077553	18	95	0.89407778	295.777778	22.7777778	0	0	1	0	42.288408	2	-0.4111751
5.6394	246577.525	0.18965717	7.70365949	0.29189878	0	1.72977768	5.5076805	-1.6572784	2.04182527	-1.2279282	-6.9077553	19	95	1.78815556	296.333333	23.33333333	0	0	1	0	42.288408	2	-0.4111751
5.6772	248200.482	0.2079022	9.21630932	0.30535028	0	1.73645815	5.51424084	-1.5658891	2.22108316	-1.1830261	-6.9077553	20	93	1.34111667	297.444444	24.44444444	0	0	1	0	42.288408	2	-0.4111751
5.7132	249735.729	0.20493863	9.34830093	0.31011052	0	1.74277929	5.52040728	-1.5801771	2.23530157	-1.1676071	-6.9077553	21	91	1.34111667	297.444444	24.44444444	0	0	1	0	42.288408	2	-0.4111751
5.7519	251446.395	0.21483659	9.76083232	0.30266757	0	1.74953024	5.5272338	-1.5332337	2.27848012	-1.1918217	-6.9077553	22	93	0.89407778	297.444444	24.44444444	0	0	1	0	42.288408	2	-0.4111751
5.7825	252762.334	0.21427498	9.5974534	0.29645025	0	1.75483612	5.53245361	-1.5358391	2.26160198	-1.2125083	-6.9077553	23	94	2.23519444	297.444444	24.44444444	0	0	1	0	42.288408	2	-0.4111751
0.0099	0	0.59813786	97.2446716	2.6484211	0	-4.6152205	-6.9077553	-0.5122636	4.57724047	0.97434116	-6.9077553	0	92	4.02335	298	25	0	0	0	0	18.9135952	2	-0.0428739
0.0396	88.5800473	0.45371946	64.7475216	1.66899132	0	-3.2289262	-2.4126227	-0.7880746	4.17051087	0.51281843	-6.9077553	1	81	4.02335	299.666667	26.66666666	0	0	0	0	18.9135952	2	-0.0428739
0.1323	703.2243	0.57332733	91.1175952	1.85942433	0	-2.0226832	-0.3506584	-0.5545558	4.5121619	0.6208046	-6.9077553	2	75	4.02335	300.777778	27.7777778	0	0	0	0	18.9135952	2	-0.0428739
0.3384	6496.72157	0.83667292	224.635781	2.51750989	0	-1.0835267	1.87145159	-0.1771276	5.41448479	0.92366741	-6.9077553	3	63	4.02335	303.555556	30.55555555	0	0	1	0	18.9135952	2	-0.0428739
0.5445	13682.2137	0.80125187	219.978627	1.99318459	0	-0.6078873	2.61616981	-0.2203327	5.39353494	0.69023524	-6.9077553	4	58	3.12927222	304.666667	31.66666666	0	0	1	0	18.9135952	2	-0.0428739
0.7047	17742.3608	0.70559877	162.972119	1.39274854	0	-0.3499831	2.8760114	-0.3472923	5.09358527	0.33199691	-6.9077553	5	51	3.12927222	305.777778	32.7777778	0	0	1	0	18.9135952	2	-0.0428739

0.9171	24215.0337	0.80031657	198.684819	1.86198787	0	-0.0865388	3.18701496	-0.2214992	5.29172478	0.62218158	-6.9077553	6	52	2.68223333	305.777778	32.7777778	0	0	1	0	18.9135952	2	-0.0428739
1.1691	32543.1208	0.82446278	203.661607	1.89126718	0	0.15623422	3.48259673	-0.1918111	5.31646474	0.63777567	-6.9077553	7	58	1.78815556	305.222222	32.2222222	0	0	1	0	18.9135952	2	-0.0428739
1.4265	40069.9924	0.78377261	179.705624	1.66951682	0	0.35522389	3.69065269	-0.2423613	5.19132566	0.51313305	-6.9077553	8	51	1.78815556	306.333333	33.33333333	0	0	1	0	18.9135952	2	-0.0428739
1.7748	54183.021	0.90925121	254.587209	2.19780248	0	0.57368774	3.99238605	-0.0940347	5.53964737	0.78791289	-6.9077553	9	48	4.91742778	306.333333	33.33333333	0	0	1	0	18.9135952	2	-0.0428739
2.124	68212.154	0.86866918	224.455884	1.86611309	0	0.7533011	4.22263742	-0.1396424	5.41368363	0.62439344	-6.9077553	10	50	4.02335	305.777778	32.7777778	0	0	1	0	18.9135952	2	-0.0428739
2.4894	85929.4264	0.96482887	270.578414	2.07878526	0	0.91204172	4.45353797	-0.0347686	5.60056564	0.73226465	-6.9077553	11	59	5.81150556	301.333333	28.33333333	0	0	1	0	18.9135952	2	-0.0428739
2.6937	95760.9937	0.88954949	213.87839	1.54777708	0	0.99091571	4.56186588	-0.1159166	5.36541226	0.43746564	-6.9077553	12	70	2.23519444	299.666667	26.66666666	0	0	1	0	18.9135952	2	-0.0428739
2.8494	103056.831	0.76633617	157.324557	1.33465656	0	1.04710845	4.6352903	-0.2648303	5.05831727	0.28942297	-6.9077553	13	71	1.34111667	298.555556	25.55555555	0	0	1	0	18.9135952	2	-0.0428739
2.9826	109059.005	0.70380164	131.604334	1.22999428	0	1.0927954	4.69189823	-0.3498389	4.87980755	0.2078222	-6.9077553	14	77	1.34111667	297.444444	24.44444444	0	0	1	0	18.9135952	2	-0.0428739
3.033	111339.92	0.26418914	15.4536133	0.49744695	0	1.10955223	4.71259685	-1.327312	2.73790755	-0.6962581	-6.9077553	15	82	0.44703889	296.333333	23.33333333	0	0	1	0	18.9135952	2	-0.0428739
3.078	113401.527	0.26598261	15.3993311	0.47466934	0	1.12428004	4.73094367	-1.3205718	2.73438901	-0.7430323	-6.9077553	16	88	0.44703889	295.777778	22.7777778	0	0	1	0	18.9135952	2	-0.0428739
3.1248	115550.84	0.24710551	13.1573029	0.46966366	0	1.13937028	4.74971926	-1.3939012	2.57705296	-0.7536115	-6.9077553	17	93	0.44703889	295.777778	22.7777778	0	0	1	0	18.9135952	2	-0.0428739
3.1644	117305.399	0.24570372	12.9924067	0.42740813	0	1.15196346	4.76478931	-1.3995672	2.56444205	-0.847679	-6.9077553	18	95	0.89407778	295.777778	22.7777778	0	0	1	0	18.9135952	2	-0.0428739
3.213	119323.132	0.23528096	12.2122475	0.39928871	0	1.16720508	4.78184359	-1.4427337	2.50252122	-0.9155692	-6.9077553	19	95	1.78815556	296.333333	23.33333333	0	0	1	0	18.9135952	2	-0.0428739
3.267	121647.91	0.23986748	12.373644	0.39355561	0	1.18387213	4.80113911	-1.4235084	2.51564954	-0.9299952	-6.9077553	20	93	1.34111667	297.444444	24.44444444	0	0	1	0	18.9135952	2	-0.0428739
3.3075	123446.321	0.2429849	12.8541083	0.42297863	0	1.19619262	4.81581452	-1.4106489	2.55374127	-0.8580722	-6.9077553	21	91	1.34111667	297.444444	24.44444444	0	0	1	0	18.9135952	2	-0.0428739
3.357	125507.92	0.21761209	10.2580389	0.37845036	0	1.21104772	4.83237683	-1.5204564	2.32815916	-0.9690315	-6.9077553	22	93	0.89407778	297.444444	24.44444444	0	0	1	0	18.9135952	2	-0.0428739
3.3966	127130.885	0.24739461	16.7690291	0.37080275	0	1.22277493	4.84522501	-1.3927366	2.81959331	-0.9893918	-6.9077553	23	94	2.23519444	297.444444	24.44444444	0	0	1	0	18.9135952	2	-0.0428739
0.0153	404.198675	0.8277362	183.774689	1.65313351	0	-4.1799025	-0.9033778	-0.1878534	5.21371593	0.50327731	-6.9077553	0	98	2.68223333	289.666667	16.66666666	0	0	0	0	30.519848	2	0.21141735
0.036	1150.01313	0.59250176	104.456479	1.03034559	0	-3.3242363	0.14064254	-0.5217151	4.64878009	0.03086434	-6.9077553	1	100	1.78815556	289.666667	16.66666666	0	0	0	0	30.519848	2	0.21141735
0.0864	1764.10679	0.50991423	76.5366385	1.17702457	0	-2.4487676	0.56821119	-0.6715536	4.33778263	0.16383894	-6.9077553	2	93	3.57631111	292.444444	19.44444444	0	0	0	0	30.519848	2	0.21141735
0.1809	2567.85803	0.45649083	58.0400763	1.68940241	0	-1.7098109	0.94346146	-0.7819984	4.06115097	0.52496661	-6.9077553	3	76	4.47038889	294.111111	21.11111111	0	0	0	0	30.519848	2	0.21141735
0.3618	3692.14718	0.6905258	140.744461	3.23472512	0	-1.0166637	1.30647899	-0.3688548	4.94695302	1.17425305	-6.9077553	4	63	5.36446667	294.111111	21.11111111	0	0	0	0	30.519848	2	0.21141735
0.5724	4757.01965	0.640974	111.432475	2.50411153	0	-0.5579172	1.55983154	-0.4432075	4.71342778	0.91833326	-6.9077553	5	55	5.36446667	295.222222	22.2222222	0	0	0	1	30.519848	2	0.21141735
0.8946	8469.58841	0.72063655	144.844383	2.82362613	0	-0.1113786	2.13659998	-0.3262337	4.97566685	1.03837601	-6.9077553	6	42	5.81150556	296.333333	23.33333333	0	0	0	1	30.519848	2	0.21141735
1.602	22133.1812	1.06094617	364.874046	5.29961832	0	0.47125285	3.09712307	0.06010323	5.89955495	1.66782348	-6.9077553	7	30	7.59966111	295.777778	22.7777778	0	0	0	1	30.519848	2	0.21141735
2.2815	40255.2462	0.82465853	201.567053	2.71013911	0	0.82483312	3.69526518	-0.191574	5.30612706	0.99736888	-6.9077553	8	28	7.59966111	296.333333	23.33333333	0	0	0	1	30.519848	2	0.21141735
3.0033	60666.2771	0.78087565	183.317604	2.05639846	0	1.09971168	4.10540446	-0.2460596	5.21122565	0.7214423	-6.9077553	9	34	8.49373889	294.111111	21.11111111	0	0	0	1	30.519848	2	0.21141735
3.9123	84026.0506	0.80745088	198.003264	2.16287878	0	1.36412544	4.43113878	-0.2126354	5.28828857	0.77190234	-6.9077553	10	40	8.0467	290.777778	17.7777778	0	0	0	1	30.519848	2	0.21141735
4.8852	105750.932	0.71553659	148.799289	1.62177976	0	1.58621023	4.66109609	-0.333326	5.00260506	0.48414058	-6.9077553	11	47	6.70558333	288	15	0	0	0	1	30.519848	2	0.21141735
5.8752	131654.017	0.7266454	158.042443	1.68978516	0	1.7707401	4.880185	-0.3179414	5.06286995	0.52519301	-6.9077553	12	50	7.15262222	286.888889	13.88888888	0	0	0	1	30.519848	2	0.21141735
6.4962	151527.688	0.67798665	143.458673	1.02766909	0	1.87121739	5.02077496	-0.3871538	4.96605397	0.02826582	-6.9077553	13	53	7.59966111	285.222222	12.22222222	0	0	0	1	30.519848	2	0.21141735
6.93	173337.695	0.64276022	122.09127	0.7448619	0	1.93585981	5.15524746	-0.440429	4.80477707	-0.2932148	-6.9077553	14	57	4.47038889	283.555556	10.55555555	0	0	0	1	30.519848	2	0.21141735
7.1685	189113.056	0.75813806	163.827992	0.97382099	0	1.96969643	5.2423503	-0.2755716	5.09882315	-0.0255014	-6.9077553	15	64	5.81150556	282.444444	9.444444444	0	0	0	1	30.519848	2	0.21141735
7.3809	205417.846	0.71348261	142.370018	0.83319234	0	1.99889558	5.32505104	-0.3361966	4.95843645	-0.1812913	-6.9077553	16	66	6.25854444	282.444444	9.444444444	0	0	0	1	30.519848	2	0.21141735
7.542	221572.804	0.65939254	112.660986	0.58998227	0	2.0204874	5.40075574	-0.4149209	4.72439206	-0.5259693	-6.9077553	17	64	5.81150556	281.888889	8.888888888	0	0	0	1	30.519848	2	0.21141735
7.6977	238138.436	0.69458484	130.326059	0.64475784	0	2.04092158	5.47285637	-0.3630023	4.87004713	-0.4373307	-6.9077553	18	62	8.0467	281.888889	8.888888888	0	0	0	1	30.519848	2	0.21141735
7.8642	254622.13	0.68641927	123.758552	0.59823402	0	2.06232081	5.53978453	-0.3748109	4.81834058	-0.5121031	-6.9077553	19	63	5.81150556	281.888889	8.888888888	0	0	0	1	30.519848	2	0.21141735
8.0262	269747.372	0.64602767	110.763563	0.58125335	0	2.08271119	5.59748957	-0.4353662	4.7074069	-0.5408496	-6.9077553	20	65	5.81150556	281.888889	8.888888888	0	0	0	1	30.519848	2	0.21141735
8.1603	285172.973	0.62290596	101.274899	0.56017677	0	2.09928093	5.65309943	-0.4717556	4.61784847	-0.5777193	-6.9077553	21	66	5.36446667	281.333333	8.333333333	0	0	0	1	30.519848	2	0.21141735
8.2971	299150.179	0.59278589	89.8347296	0.57857353	0	2.11590606	5.70094906	-0.5212365	4.49798278	-0.5454627	-6.9077553	22	67	4.91742778	281.333333	8.333333333	0	0	0	1	30.519848	2	0.21141735
9.2205	318474.122	0.69901643	151.615147	3.18553188	0	2.22142927	5.76354436	-0.3566515	5.02135198	1.15893314	-6.9077553	23	65	5.81150556	281.333333	8.333333333	0	0	0	1	30.519848	2	0.21141735
0.0135	657.921657	0.97999123	261.407628	1.76561914	0	-4.3050656	-0.4171506	-0.0191918	5.56608481	0.56906763	-6.9077553	0	98	2.68223333	289.666667	16.66666666	0	0	0	0	34.0724638	2	0.49277013
0.0261	1052.69567	0.53019673	74.6509835	0.63381127	0	-3.64582	0.05230367	-0.6326228	4.31283709	-0.4544275	-6.9077553	1	100	1.78815556	289.666667	16.66666666	0	0	0	0	34.0724638	2	0.49277013
0.0792	2956.48872	0.76034197	161.048613	1.89347322	0	-2.535779	1.0843405	-0.2726727	5.08171247	0.63894082	-6.9077553	2	93	3.57631111	292.444444	19.44444444	0	0	0	0	34.0724638	2	0.49277013
0.1521	6285.34333	0.88602721	206.898992	1.44858692	0	-1.8832171	1.83837956	-0.1198796	5.33223555	0.37127864	-6.9077553	3	76	4.47038889	294.111111	21.11111111	0	0	0	0	34.0724638	2	0.49277013
0.2556	9896.95328	0.92069961	222.189046	2.36287444	0	-1.3641416	2.292328	-0.0815359	5.40353308	0.86030198	-6.9077553	4	63	5.36446667	294.111111	21.11111111	0	0	0	1	34.0724638	2	0.49277013
0.3888	16986.2941	1.10320932	349.655564	2.8615766	0	-0.9446902	2.83246566	0.09912953	5.85695143	1.05172213	-6.9077553	5	55	5.36446667	295.222222	22.2222222	0	0	0	1	34.0724638	2	0.49277013
0.6894	35740.4679	1.31000414	487.519742	4.32158583	0	-0.3719336	3.57631158	0.27079336	6.18933284	1.4638538	-6.9077553	6	42	5.81150556	296.333333	23.33333333	0	0	0	1	34.0724638	2	0.49277013
1.0575	55981.1374	1.1954286	414.402277	3.66512591	0	0.05590763	4.02503266	0.17934095	6.0268396	1.2991355	-6.9077553	7	30	7.59966111	295.777778	22.7777778	0	0	0	1	34.0724638	2	0.49277013
1.6479	80829.1962	1.17544732	410.876096	4.22199454	0	0.49950175	4.39235061	0.16249916	6.01829413	1.44054448	-6.9077553	8	28	7.59966111	296.333333	23.33333333	0	0	0	1	34.0724638	2	0.49277013
2.3202	110333.235	1.07929276	351.959003	3.21567193	0	0.84165339	4.70351426	0.07723208	5.86351754	1.16834726	-6.9077553	9	34	8.49373889	294.111111	21.11111111	0	0	0	1	34.0724638	2	0.49277013
2.9889	136174.569	1.00235696	289.142665	3.02751396	0	1.09490543	4.913945	0.00335134	5.66692367	1.10807206	-6.9077553	10	40	8.0467	290.777778	17.7777778	0	0	0	1	34.0724638	2	0.49277013
3.4758	154178.338	0.88594741	221.493819	1.87762158	0	1.24582467	5.03811646	-0.1199696	5.4003992	0.6305383	-6.9077553	11	47	6.70558333	288	15	0	0	0	1	34.0724638	2	0.49277013
3.9051	172038.676	0.92453739	257.61894	2.17605431	0	1.36228339	5.14772513	-0.0773808	5.5514854	0.77797273	-6.9077553	12	50	7.15262222	286.888889	13.88888888	0	0	0	1	34.0724638	2	0.49277013
4.2876	186794.391	0.86078807	214.985478	1.78207706	0	1.45572714	5.23001385	-0.1487459	5.37057513	0.57834056	-6.9077553	13	53	7.59966111	285.222222	12.22222222	0	0	0	1	34.0724638	2	0.49277013
4.5621	196955.364	0.76502816	162.320953	1.15747831	0	1.51778304	5.2829822	-0.2665364	5.08958173	0.14710734	-6.9077553	14	57	4.47038889	283.555556	10.55555555	0	0	0	1	34.0724638	2	0.49277013
4.7763	206485.004	0.81297975	175.828404	1.24402279	0	1.56366619	5.33023263	-0.2058198	5.16951423	0.21915383	-6.9077553	15	64	5.81150556	282.444444	9.444444444	0	0	0	1	34.0724638	2	0.49277013
4.9941	216269.467	0.84399755	190.927299	1.44517454	0	1.60825722	5.37652978	-0.1684216	5.25189796	0.36892182	-6.9077553	16	66	6.25854444	282.444444	9.444444444	0	0	0	1	34.0724638	2	0.49277013
5.2299	228220.102	0.92732914	233.627848	1.74895122	0	1.65439216	5.43031491	-0.0743689	5.45373374	0.55958791	-6.9077553	17	64	5.81150556	281.888889	8.888888888	0	0	0	1	34.0724638	2	0.49277013
5.5539	244163.313	1.0541253	332.539497	2.48022224	0	1.71450038	5.49784141	0.05365953	5.80676165	0.90875128	-6.9077553	18	62	8.0467	281.888889	8.888888888	0	0	0	1	34.0724638	2	0.49277013
5.8248	258093.305	0.98751091	270.008825	1.97174295	0	1.76212466	5.55332504	-0.0115556	5.59845835	0.67942494	-6.9077553	19	63	5.81150556	281.888889	8.888888888	0	0	0	1	34.0724638	2	0.49277013

6.0453	268101.145	0.92330995	236.39874	1.75634242	0	1.79928111	5.59136805	-0.0787078	5.46552419	0.56380268	-6.9077553	20	65	5.81150556	281.888889	8.888888888	0	0	0	1	34.0724638	2	0.49277013
6.1875	274012.186	0.8111297	176.273227	1.38985163	0	1.82253113	5.61317623	-0.2080952	5.17204089	0.32991624	-6.9077553	21	66	5.36446667	281.333333	8.333333333	0	0	0	1	34.0724638	2	0.49277013
6.354	280656.971	0.75016125	168.161983	1.45610352	0	1.84908454	5.63713674	-0.2861349	5.12493364	0.37645057	-6.9077553	22	67	4.91742778	281.333333	8.333333333	0	0	0	1	34.0724638	2	0.49277013
6.9489	291403.961	0.64704634	127.118452	2.59689853	0	1.93858337	5.67471392	-0.4337931	4.84512721	0.95470286	-6.9077553	23	65	5.81150556	281.333333	8.333333333	0	0	0	1	34.0724638	2	0.49277013
0.0315	1983.5127	1.10296519	337.709817	2.2648026	0	-3.4577677	0.68537339	0.09890841	5.82218996	0.81792904	-6.9077553	0	98	2.68223333	289.666667	16.66666666	0	0	0	0	42.288408	2	-0.4111751
0.1062	5673.80327	0.93948658	240.259601	1.96634242	0	-2.2424312	1.7360359	-0.0613579	5.48172417	0.67668361	-6.9077553	1	100	1.78815556	289.666667	16.66666666	0	0	0	0	42.288408	2	-0.4111751
0.3177	13840.7173	0.98995777	271.277344	3.23796594	0	-1.1466477	2.62768703	-0.0090834	5.60314539	1.17525413	-6.9077553	2	93	3.57631111	292.444444	19.44444444	0	0	0	0	42.288408	2	-0.4111751
0.5499	23725.2453	0.90717842	217.233436	1.87001925	0	-0.5980188	3.16658183	-0.0963144	5.38097712	0.62648333	-6.9077553	3	76	4.47038889	294.111111	21.11111111	0	0	0	0	42.288408	2	-0.4111751
0.8577	35032.6159	0.8851868	206.314876	2.04949791	0	-0.1535009	3.55630805	-0.1208275	5.32940837	0.71808264	-6.9077553	4	63	5.36446667	294.111111	21.11111111	0	0	0	0	42.288408	2	-0.4111751
1.1907	48556.3482	0.84553981	191.224747	1.68259657	0	0.17454137	3.88274554	-0.166598	5.25345465	0.52093232	-6.9077553	5	55	5.36446667	295.222222	22.2222222	0	0	0	0	42.288408	2	-0.4111751
1.7343	67685.6185	0.9274002	245.633045	2.62417097	0	0.55060387	4.2148885	-0.0742924	5.50384281	0.96514603	-6.9077553	6	42	5.81150556	296.333333	23.33333333	0	0	1	0	42.288408	2	-0.4111751
2.4813	101446.386	1.09607757	358.270294	3.723101	0	0.90878262	4.6195403	0.09264989	5.8812905	1.31482548	-6.9077553	7	30	7.59966111	295.777778	22.7777778	0	0	1	0	42.288408	2	-0.4111751
3.3984	143351.703	1.08340529	369.83274	3.60394593	0	1.22330473	4.96530805	0.08103172	5.91305355	1.28230677	-6.9077553	8	28	7.59966111	296.333333	23.33333333	0	0	1	0	42.288408	2	-0.4111751
4.131	166784.252	0.97421939	303.499482	3.27169552	1	1.41851951	5.11670707	-0.0250928	5.7153832	1.18561396	0.0009995	9	34	8.49373889	294.111111	21.11111111	0	0	1	0	42.288408	2	-0.4111751
4.6044	177673.598	0.71971647	153.661033	1.95588481	1	1.52701237	5.17995377	-0.3275095	5.0347556	0.67135383	0.0009995	10	40	8.0467	290.777778	17.7777778	0	0	1	0	42.288408	2	-0.4111751
4.9932	189772.692	0.69142391	142.48574	1.33930884	1	1.60807699	5.24583227	-0.3675569	4.95924895	0.29290007	0.0009995	11	47	6.70558333	288	15	0	0	1	0	42.288408	2	-0.4111751
5.3847	203319.549	0.72979757	157.79199	1.29993826	1	1.6835616	5.31478379	-0.3136188	5.06128398	0.26308575	0.0009995	12	50	7.15262222	286.888889	13.88888888	0	0	1	0	42.288408	2	-0.4111751
5.6952	213749.111	0.68594307	138.676347	1.15501939	1	1.73962371	5.36480763	-0.3755039	4.93214999	0.14498254	0.0009995	13	53	7.59966111	285.222222	12.22222222	0	0	1	0	42.288408	2	-0.4111751
6.0093	225736.426	0.76798412	170.706841	1.23613085	1	1.79330827	5.41937249	-0.262685	5.13995356	0.21279487	0.0009995	14	57	4.47038889	283.555556	10.55555555	0	0	1	0	42.288408	2	-0.4111751
6.1956	233684.299	0.79623045	169.093731	1.236209	1	1.82383936	5.45397534	-0.2266115	5.1304591	0.21285804	0.0009995	15	64	5.81150556	282.444444	9.444444444	0	0	1	0	42.288408	2	-0.4111751
6.345	240062.666	0.75849175	150.651544	1.19132793	1	1.8476671	5.48090416	-0.2751058	5.01497615	0.17590764	0.0009995	16	66	6.25854444	282.444444	9.444444444	0	0	1	0	42.288408	2	-0.4111751
6.4764	245512.904	0.71955041	133.910865	1.13683539	1	1.8681648	5.50335358	-0.3277399	4.89718186	0.12912767	0.0009995	17	64	5.81150556	281.888889	8.888888888	0	0	1	0	42.288408	2	-0.4111751
6.6087	251288.734	0.76854291	158.642219	1.25746892	1	1.88838696	5.52660659	-0.2619586	5.06665778	0.22989584	0.0009995	18	62	8.0467	281.888889	8.888888888	0	0	1	0	42.288408	2	-0.4111751
6.7383	257250.974	0.81892978	177.334039	1.29497634	1	1.90780767	5.55005605	-0.1985366	5.17804082	0.25926434	0.0009995	19	63	5.81150556	281.888889	8.888888888	0	0	1	0	42.288408	2	-0.4111751
6.8697	262558.999	0.771807	153.845436	1.19539109	1	1.92712044	5.57047963	-0.2577259	5.03595493	0.1793096	0.0009995	20	65	5.81150556	281.888889	8.888888888	0	0	1	0	42.288408	2	-0.4111751
6.9921	267664.184	0.77828733	159.072079	1.20745042	1	1.94478094	5.58973689	-0.2493755	5.06936371	0.18933889	0.0009995	21	66	5.36446667	281.333333	8.333333333	0	0	1	0	42.288408	2	-0.4111751
7.137	272974.932	0.69064644	130.713024	1.12772687	1	1.96529252	5.60938363	-0.3686804	4.87301191	0.12109033	0.0009995	22	67	4.91742778	281.333333	8.333333333	0	0	1	0	42.288408	2	-0.4111751
7.7148	281800.403	0.56317681	95.4454707	2.27367156	1	2.04314056	5.64120258	-0.5723876	4.55856557	0.82183567	0.0009995	23	65	5.81150556	281.333333	8.333333333	0	0	1	0	42.288408	2	-0.4111751
0.0018	0	0.18587095	6.69716311	0.50842071	0	-6.3199686	-6.9077553	-1.677337	1.90183333	-0.6744811	-6.9077553	0	98	2.68223333	289.666667	16.66666666	0	0	0	0	18.9135952	2	-0.0428739
0.0036	0	0.11431472	3.30488801	0.37079287	0	-5.6268214	-6.9077553	-2.1600902	1.19570512	-0.9894184	-6.9077553	1	100	1.78815556	289.666667	16.66666666	0	0	0	0	18.9135952	2	-0.0428739
0.0324	0	0.43157792	49.1256638	1.58667588	0	-3.4295969	-6.9077553	-0.8379928	3.89440194	0.46227124	-6.9077553	2	93	3.57631111	292.444444	19.44444444	0	0	0	0	18.9135952	2	-0.0428739
0.1548	175.734282	0.58160518	89.5446921	2.69775189	0	-1.8656213	-1.7331079	-0.5402455	4.49474902	0.9927894	-6.9077553	3	76	4.47038889	294.111111	21.11111111	0	0	1	0	18.9135952	2	-0.0428739
0.3789	3816.12556	0.71226888	173.051424	2.84073967	0	-0.970483	1.33949767	-0.3378968	5.15359458	1.04441642	-6.9077553	4	63	5.36446667	294.111111	21.11111111	0	0	1	0	18.9135952	2	-0.0428739
0.5868	11892.0806	0.87440834	267.368236	2.3155618	0	-0.5330712	2.47595677	-0.1330648	5.58863061	0.8400841	-6.9077553	5	55	5.36446667	295.222222	22.2222222	0	0	1	0	18.9135952	2	-0.0428739
0.7722	16025.3843	0.71402822	156.10509	1.81769169	0	-0.2585117	2.77423638	-0.3354333	5.05053584	0.59811739	-6.9077553	6	42	5.81150556	296.333333	23.33333333	0	0	1	0	18.9135952	2	-0.0428739
1.0647	21432.5525	0.7749323	194.643317	2.50980675	0	0.06269307	3.06495757	-0.25369	5.27117388	0.92060412	-6.9077553	7	30	7.59966111	295.777778	22.7777778	0	0	1	0	18.9135952	2	-0.0428739
1.3428	29833.9004	0.77653474	192.69956	1.9027226	0	0.29475699	3.39567886	-0.251627	5.26113748	0.64381123	-6.9077553	8	28	7.59966111	296.333333	23.33333333	0	0	1	0	18.9135952	2	-0.0428739
1.6515	36575.9394	0.71046581	154.291135	2.16418528	0	0.50168397	3.59941797	-0.3404279	5.03884779	0.77250594	-6.9077553	9	34	8.49373889	294.111111	21.11111111	0	0	1	0	18.9135952	2	-0.0428739
1.9827	43357.0308	0.71754696	157.995913	2.43271969	0	0.68445955	3.76949194	-0.3305242	5.0625755	0.88942082	-6.9077553	10	40	8.0467	290.777778	17.7777778	0	0	1	0	18.9135952	2	-0.0428739
2.2977	55428.9865	0.80562945	205.184866	1.85948225	0	0.83190862	4.01512072	-0.2148909	5.32391623	0.62083573	-6.9077553	11	47	6.70558333	288	15	0	0	1	0	18.9135952	2	-0.0428739
2.6037	68592.7523	0.82741502	213.108134	1.75343825	0	0.95693351	4.22820146	-0.188241	5.3618044	0.56214872	-6.9077553	12	50	7.15262222	286.888889	13.88888888	0	0	1	0	18.9135952	2	-0.0428739
2.9556	85010.8667	0.92970678	267.446971	1.96874266	0	1.08370168	4.44279086	-0.071811	5.58892505	0.67790291	-6.9077553	13	53	7.59966111	285.222222	12.22222222	0	0	1	0	18.9135952	2	-0.0428739
3.2895	100714.464	0.88677098	227.630665	1.60673354	0	1.19073558	4.61229935	-0.1190415	5.42772882	0.47482545	-6.9077553	14	57	4.47038889	283.555556	10.55555555	0	0	1	0	18.9135952	2	-0.0428739
3.5622	114233.797	0.88504884	211.998298	1.46474384	0	1.27037833	4.73825595	-0.1209832	5.35658296	0.38236285	-6.9077553	15	64	5.81150556	282.444444	9.444444444	0	0	1	0	18.9135952	2	-0.0428739
3.861	129817.984	0.88864356	215.727739	1.57756153	0	1.35092622	4.86614105	-0.1169344	5.37402178	0.45651401	-6.9077553	16	66	6.25854444	282.444444	9.444444444	0	0	1	0	18.9135952	2	-0.0428739
4.1904	146371.373	0.88047315	210.595714	1.55559469	0	1.43279619	4.98615388	-0.1261607	5.349945	0.44250054	-6.9077553	17	64	5.81150556	281.888889	8.888888888	0	0	1	0	18.9135952	2	-0.0428739
4.5531	165608.855	0.96869775	263.207836	1.89827726	0	1.51580832	5.10963475	-0.0307709	5.57294777	0.64147343	-6.9077553	18	62	8.0467	281.888889	8.888888888	0	0	1	0	18.9135952	2	-0.0428739
4.9113	184882.988	0.94888796	256.076142	1.86117416	0	1.59153867	5.21972854	-0.0514112	5.54547874	0.62174471	-6.9077553	19	63	5.81150556	281.888889	8.888888888	0	0	1	0	18.9135952	2	-0.0428739
5.3082	208145.349	1.02683249	299.525652	2.13660831	0	1.66925279	5.33824143	0.02745221	5.7022034	0.75968759	-6.9077553	20	65	5.81150556	281.888889	8.888888888	0	0	1	0	18.9135952	2	-0.0428739
5.6853	228525.903	0.96940114	261.973989	1.9059642	0	1.7378839	5.43165394	-0.0300457	5.56824904	0.64551255	-6.9077553	21	66	5.36446667	281.333333	8.333333333	0	0	1	0	18.9135952	2	-0.0428739
6.0741	247382.278	0.86935453	222.867694	1.75445232	0	1.80403383	5.51093887	-0.1388546	5.40658278	0.56272655	-6.9077553	22	67	4.91742778	281.333333	8.333333333	0	0	1	0	18.9135952	2	-0.0428739
6.9363	265712.14	0.63237965	121.163761	2.11488753	0	1.93676849	5.58241731	-0.4566853	4.79715128	0.74947436	-6.9077553	23	65	5.81150556	281.333333	8.333333333	0	0	1	0	18.9135952	2	-0.0428739
0.0081	320.450567	0.82658458	186.785934	1.75263679	0	-4.8158912	-1.1349115	-0.189244	5.22996858	0.5616918	-6.9077553	0	89	4.47038889	295.222222	22.2222222	0	0	0	0	30.519848	2	0.21141735
0.0117	495.905695	0.40612438	40.5744629	0.65328294	0	-4.4481664	-0.699355	-0.8986365	3.70316352	-0.4242154	-6.9077553	1	88	4.91742778	295.222222	22.2222222	0	0	0	0	30.519848	2	0.21141735
0.0207	890.679853	0.54503345	70.4794739	0.84377928	0	-3.8776216	-0.1146481	-0.605075	4.25533571	-0.1686799	-6.9077553	2	86	5.36446667	295.222222	22.2222222	0	0	0	0	30.519848	2	0.21141735
0.0477	1373.18135	0.47089895	72.292985	1.16975365	0	-3.0428239	0.31785817	-0.7509904	4.28074093	0.15764768	-6.9077553	3	98	2.68223333	289.666667	16.66666666	0	0	0	0	30.519848	2	0.21141735
0.0801	1899.54665	0.36762004	40.0552843	0.86146784	0	-2.5244794	0.64214156	-0.9979889	3.69028557	-0.1479574	-6.9077553	4	100	1.78815556	289.666667	16.66666666	0	0	0	0	30.519848	2	0.21141735
0.1467	2586.08498	0.4488711	57.5316987	1.28897311	0	-1.9193656	0.95053175	-0.7987942	4.05235346	0.25462138	-6.9077553	5	93	3.57631111	292.444444	19.44444444	0	0	0	0	30.519848	2	0.21141735
0.3141	3545.50095	0.54208608	81.4112588	2.3349442	0	-1.1580439	1.26596147	-0.6104874	4.39952586	0.84841618	-6.9077553	6	76	4.47038889	294.111111	21.11111111	0	0	0	0	30.519848	2	0.21141735
0.4923	4635.46938	0.65820104	114.866675	2.39323055	0	-0.708667	1.53395317	-0.4167267	4.74378081	0.87306191	-6.9077553	7	63	5.36446667	294.111111	21.11111111	0	0	0	1	30.519848	2	0.21141735
0.7983	7223.14287	0.7012777	136.602206	2.84457792	0	-0.2252708	1.97742859	-0.3534264	4.91708042	1.04576618	-6.9077553	8	55	5.36446667	295.222222	22.2222222	0	0	0	1	30.519848	2	0.21141735
1.1556	14595.735	0.80188811	180.889917	2.58175851	0	0.14461969	2.68079787	-0.2195399	5.19789418	0.94885802	-6.9077553	9	42	5.81150556	296.333333	23.33333333	0	0	0	1	30.519848	2	0.21141735

1.8693	26668.4784	1.01106407	339.619995	4.57012655	0	0.62556403	3.28351978	0.01199187	5.82783028	1.51975968	-6.9077553	10	30	7.59966111	295.777778	22.7777778	0	0	0	1	30.519848	2	0.21141735
2.6109	43409.8947	0.77722309	184.587739	2.21229524	0	0.95969499	3.77071044	-0.2507421	5.21813032	0.79448246	-6.9077553	11	28	7.59966111	296.333333	23.33333333	0	0	0	1	30.519848	2	0.21141735
3.3345	61648.9374	0.79558051	193.001458	2.43703768	0	1.20432274	4.12147221	-0.2274271	5.26270292	0.89119349	-6.9077553	12	34	8.49373889	294.111111	21.11111111	0	0	0	1	30.519848	2	0.21141735
4.3038	82867.5328	0.79940017	195.789926	2.25916151	0	1.45949835	4.41725541	-0.2226435	5.27704738	0.81543627	-6.9077553	13	40	8.0467	290.777778	17.7777778	0	0	0	1	30.519848	2	0.21141735
5.2308	110941.054	0.72946072	151.021208	1.60567627	0	1.65456423	4.70900803	-0.3140798	5.0174269	0.47416762	-6.9077553	14	47	6.70558333	288	15	0	0	0	1	30.519848	2	0.21141735
6.0588	135119.417	0.70952162	145.158882	1.51943864	0	1.80151176	4.90616636	-0.3417559	4.97783577	0.41899887	-6.9077553	15	50	7.15262222	286.888889	13.88888888	0	0	0	1	30.519848	2	0.21141735
6.651	155075.877	0.7222053	158.692297	1.0611127	0	1.89476722	5.04392098	-0.3240621	5.06697339	0.06026004	-6.9077553	16	53	7.59966111	285.222222	12.22222222	0	0	0	1	30.519848	2	0.21141735
7.0173	175417.683	0.66167463	128.437961	0.76833822	0	1.94837853	5.16717559	-0.4114712	4.85545378	-0.2622246	-6.9077553	17	57	4.47038889	283.555556	10.55555555	0	0	0	1	30.519848	2	0.21141735
7.2036	189896.716	0.70308372	133.33991	0.71717149	0	1.9745809	5.24648559	-0.350858	4.89290908	-0.3310469	-6.9077553	18	64	5.81150556	282.444444	9.444444444	0	0	0	1	30.519848	2	0.21141735
7.335	202626.33	0.66942299	118.075878	0.62822567	0	1.99265741	5.31136848	-0.3998464	4.77133592	-0.4632653	-6.9077553	19	66	6.25854444	282.444444	9.444444444	0	0	0	1	30.519848	2	0.21141735
7.4772	216399.791	0.65960915	117.770817	0.66887887	0	2.01185839	5.3771322	-0.4145929	4.768749	-0.4006584	-6.9077553	20	64	5.81150556	281.888889	8.888888888	0	0	0	1	30.519848	2	0.21141735
7.6032	229354.697	0.69738105	129.317411	0.63638175	0	2.02856921	5.43527406	-0.3589904	4.86227766	-0.4503865	-6.9077553	21	62	8.0467	281.888889	8.888888888	0	0	0	1	30.519848	2	0.21141735
7.7481	244923.179	0.65377277	111.310656	0.5737698	0	2.04744765	5.50094869	-0.423467	4.71233398	-0.5537857	-6.9077553	22	63	5.81150556	281.888889	8.888888888	0	0	0	1	30.519848	2	0.21141735
7.8633	258149.812	0.63263869	101.965218	0.51786709	0	2.06220637	5.55354396	-0.4562764	4.62464156	-0.6561075	-6.9077553	23	65	5.81150556	281.888889	8.888888888	0	0	0	1	30.519848	2	0.21141735
0.0171	739.82339	0.97688516	263.905192	1.99993282	0	-4.0686768	-0.299993	-0.022363	5.57559371	0.69361348	-6.9077553	0	89	4.47038889	295.222222	22.2222222	0	0	0	0	34.0724638	2	0.49277013
0.0306	1266.18871	0.50577574	61.077535	0.87914969	0	-3.4867553	0.23680083	-0.6796867	4.1121605	-0.1276633	-6.9077553	1	88	4.91742778	295.222222	22.2222222	0	0	0	0	34.0724638	2	0.49277013
0.0459	1748.69038	0.45183229	47.9880694	0.67511811	0	-3.0812902	0.55943884	-0.7922334	3.87097326	-0.3913875	-6.9077553	2	86	5.36446667	295.222222	22.2222222	0	0	0	0	34.0724638	2	0.49277013
0.0684	2538.23848	0.50189816	67.8696875	0.89696419	0	-2.6823825	0.93186422	-0.6873676	4.21760424	-0.1076251	-6.9077553	3	98	2.68223333	289.666667	16.66666666	0	0	0	0	34.0724638	2	0.49277013
0.1026	4030.39581	0.6659712	117.728169	1.12222069	0	-2.2769173	1.39411267	-0.4050084	4.76838681	0.11620018	-6.9077553	4	100	1.78815556	289.666667	16.66666666	0	0	0	0	34.0724638	2	0.49277013
0.171	7046.20789	0.82140396	177.91536	1.50791028	0	-1.7660917	1.95263149	-0.1955236	5.18131355	0.41138772	-6.9077553	5	93	3.57631111	292.444444	19.44444444	0	0	0	0	34.0724638	2	0.49277013
0.2664	11102.9514	0.88752811	209.741635	1.85016099	0	-1.3227563	2.40730103	-0.118189	5.34588123	0.61581301	-6.9077553	6	76	4.47038889	294.111111	21.11111111	0	0	0	0	34.0724638	2	0.49277013
0.513	21878.2002	1.1279778	363.345746	3.37468903	0	-0.6674794	3.08553642	0.12131262	5.8953576	1.21659946	-6.9077553	7	63	5.36446667	294.111111	21.11111111	0	0	0	1	34.0724638	2	0.49277013
0.981	42466.3778	1.27291448	478.932422	3.94547964	0	-0.0191828	3.7487362	0.24209442	6.17156159	1.37282395	-6.9077553	8	55	5.36446667	295.222222	22.2222222	0	0	0	1	34.0724638	2	0.49277013
1.521	58871.452	1.17616211	405.277969	3.45894246	0	0.41936801	4.07537327	0.16310655	6.00457564	1.24125196	-6.9077553	9	42	5.81150556	296.333333	23.33333333	0	0	0	1	34.0724638	2	0.49277013
2.1744	87769.1815	1.19830205	422.407369	3.69892349	0	0.77675276	4.47472182	0.18173976	6.04597255	1.30831214	-6.9077553	10	30	7.59966111	295.777778	22.7777778	0	0	0	1	34.0724638	2	0.49277013
3.0006	121258.773	1.16863548	401.18944	3.69690021	0	1.09881227	4.79793513	0.15669215	5.99443623	1.30776515	-6.9077553	11	28	7.59966111	296.333333	23.33333333	0	0	0	1	34.0724638	2	0.49277013
3.2769	129670.438	0.96604437	277.928237	2.56919896	0	1.18689785	4.86500385	-0.0335109	5.62736654	0.94398331	-6.9077553	12	34	8.49373889	294.111111	21.11111111	0	0	0	1	34.0724638	2	0.49277013
3.3696	130109.597	0.77223754	162.046477	1.31811583	0	1.21479404	4.86838484	-0.257169	5.08788936	0.27696168	-6.9077553	13	40	8.0467	290.777778	17.7777778	0	0	0	1	34.0724638	2	0.49277013
3.4803	131335.788	0.77378498	169.34005	1.32275155	0	1.2471185	4.87776492	-0.2551697	5.13191473	0.28046979	-6.9077553	14	47	6.70558333	288	15	0	0	0	1	34.0724638	2	0.49277013
3.5892	133143.258	0.72346137	162.885928	1.31495175	0	1.27792934	4.89143319	-0.3223268	5.09305627	0.27456017	-6.9077553	15	50	7.15262222	286.888889	13.88888888	0	0	0	1	34.0724638	2	0.49277013
3.7062	136382.731	0.81583405	214.804808	1.57509953	0	1.31000709	4.91547247	-0.2023193	5.3697344	0.45495315	-6.9077553	16	53	7.59966111	285.222222	12.22222222	0	0	0	1	34.0724638	2	0.49277013
3.8205	139923.784	0.77027316	172.316483	1.24212887	0	1.3403813	4.94110502	-0.2597127	5.14933861	0.21763148	-6.9077553	17	57	4.47038889	283.555556	10.55555555	0	0	0	1	34.0724638	2	0.49277013
3.9555	146040.993	0.87228923	212.535339	1.60051748	0	1.37510702	4.9838942	-0.1354885	5.35911298	0.4709516	-6.9077553	18	64	5.81150556	282.444444	9.444444444	0	0	0	1	34.0724638	2	0.49277013
4.1139	153207.225	0.87778802	209.29905	1.56040157	0	1.41437148	5.03179794	-0.1292116	5.34376887	0.44558386	-6.9077553	19	66	6.25854444	282.444444	9.444444444	0	0	0	1	34.0724638	2	0.49277013
4.2696	159989.089	0.85411398	199.906702	1.54283468	0	1.45152015	5.07511187	-0.1565205	5.29785577	0.43426938	-6.9077553	20	64	5.81150556	281.888889	8.888888888	0	0	0	1	34.0724638	2	0.49277013
4.3893	165447.637	0.95966265	288.251057	2.24368286	0	1.47916976	5.1086608	-0.040132	5.6638353	0.80856425	-6.9077553	21	62	8.0467	281.888889	8.888888888	0	0	0	1	34.0724638	2	0.49277013
4.5189	170475.92	0.85661761	206.434489	1.70283996	0	1.5082686	5.13859992	-0.153597	5.32998796	0.5328845	-6.9077553	22	63	5.81150556	281.888889	8.888888888	0	0	0	1	34.0724638	2	0.49277013
4.6548	176440.634	0.90416834	229.361496	1.79928932	0	1.53789895	5.17299014	-0.0996343	5.43530371	0.58794739	-6.9077553	23	65	5.81150556	281.888889	8.888888888	0	0	0	1	34.0724638	2	0.49277013
0.0342	2226.93536	1.14598929	362.805981	2.52180543	0	-3.3755296	0.80107531	0.1371405	5.89387096	0.92537155	-6.9077553	0	89	4.47038889	295.222222	22.2222222	0	0	0	0	42.288408	2	-0.4111751
0.0837	4596.16656	0.67524865	117.478036	1.30098516	0	-2.4805163	1.52544015	-0.3911944	4.7662599	0.26389015	-6.9077553	1	88	4.91742778	295.222222	22.2222222	0	0	0	0	42.288408	2	-0.4111751
0.1494	7842.82378	0.73004819	145.587917	1.49119265	0	-1.901128	2.05972644	-0.3132759	4.98078702	0.40024661	-6.9077553	2	86	5.36446667	295.222222	22.2222222	0	0	0	0	42.288408	2	-0.4111751
0.2943	13147.9779	0.79920173	186.946392	1.97559087	0	-1.2231556	2.57634403	-0.2228914	5.23082725	0.68137358	-6.9077553	3	98	2.68223333	289.666667	16.66666666	0	0	0	0	42.288408	2	-0.4111751
0.4815	22343.917	0.93165266	240.895959	1.99921168	0	-0.730849	3.10659887	-0.0697224	5.48436928	0.69325301	-6.9077553	4	100	1.78815556	289.666667	16.66666666	0	0	0	0	42.288408	2	-0.4111751
0.7065	30841.6347	0.78705188	169.39	1.73108252	0	-0.3474321	3.42889798	-0.2381914	5.13220965	0.54932445	-6.9077553	5	93	3.57631111	292.444444	19.44444444	0	0	1	0	42.288408	2	-0.4111751
1.0035	41116.537	0.79872843	168.69652	1.81306189	0	0.00349389	3.71643472	-0.2234831	5.12810729	0.59556847	-6.9077553	6	76	4.47038889	294.111111	21.11111111	0	0	1	0	42.288408	2	-0.4111751
1.3734	55926.9344	0.85462822	193.374088	1.76953333	0	0.31728942	4.02406398	-0.1559193	5.26463176	0.57128082	-6.9077553	7	63	5.36446667	294.111111	21.11111111	0	0	1	0	42.288408	2	-0.4111751
1.9377	76970.4572	0.91076543	236.263557	2.39121218	0	0.6615017	4.34343467	-0.0923725	5.46495218	0.87221854	-6.9077553	8	55	5.36446667	295.222222	22.2222222	0	0	1	0	42.288408	2	-0.4111751
2.6802	106726.871	1.01329464	292.273826	3.05018407	0	0.98589142	4.67028233	0.01419344	5.67769454	1.11552973	-6.9077553	9	42	5.81150556	296.333333	23.33333333	0	0	1	0	42.288408	2	-0.4111751
3.6549	142622.837	1.03348536	333.111265	3.86379027	0	1.29606873	4.96021065	0.03390407	5.80847957	1.35190742	-6.9077553	10	30	7.59966111	295.777778	22.7777778	0	0	1	0	42.288408	2	-0.4111751
4.608	181166.821	1.00496235	316.173808	3.22096709	0	1.52779392	5.19942379	0.00594464	5.75629525	1.16999207	-6.9077553	11	28	7.59966111	296.333333	23.33333333	0	0	1	0	42.288408	2	-0.4111751
5.1444	196332.778	0.79986654	195.01729	2.07305662	0	1.63790874	5.27981616	-0.222061	5.27309335	0.72950641	-6.9077553	12	34	8.49373889	294.111111	21.11111111	0	0	1	0	42.288408	2	-0.4111751
5.6637	212806.868	0.75460715	172.325607	1.60962887	0	1.73407739	5.36038973	-0.2802337	5.14939155	0.47662471	-6.9077553	13	40	8.0467	290.777778	17.7777778	0	0	1	0	42.288408	2	-0.4111751
6.1758	227948.98	0.69364433	142.310729	1.48337484	0	1.82063843	5.42912622	-0.3643553	4.95801992	0.3949937	-6.9077553	14	47	6.70558333	288	15	0	0	1	0	42.288408	2	-0.4111751
6.6276	240416.15	0.64150806	122.619553	1.42726275	0	1.89124275	5.48237554	-0.4423759	4.80909465	0.35645885	-6.9077553	15	50	7.15262222	286.888889	13.88888888	0	0	1	0	42.288408	2	-0.4111751
7.0146	253250.959	0.67457943	148.044104	1.51912671	0	1.94799369	5.53438488	-0.3921845	4.99751699	0.4187937	-6.9077553	16	53	7.59966111	285.222222	12.22222222	0	0	1	0	42.288408	2	-0.4111751
7.3287	266810.286	0.78776826	192.420621	1.35666995	0	1.99179815	5.58654161	-0.2372827	5.25968891	0.30576996	-6.9077553	17	57	4.47038889	283.555556	10.55555555	0	0	1	0	42.288408	2	-0.4111751
7.5672	278543.193	0.90588598	227.473968	1.59069813	0	2.02382312	5.62957673	-0.0977386	5.4270402	0.46480145	-6.9077553	18	64	5.81150556	282.444444	9.444444444	0	0	1	0	42.288408	2	-0.4111751
7.8246	291760.038	0.94454945	251.739237	1.81990446	0	2.05727262	5.6759351	-0.0559891	5.52839775	0.59933333	-6.9077553	19	66	6.25854444	282.444444	9.444444444	0	0	1	0	42.288408	2	-0.4111751
8.0829	304408.917	0.86442348	205.990309	1.54298236	0	2.08975072	5.7183752	-0.1445363	5.32783398	0.43436503	-6.9077553	20	64	5.81150556	281.888889	8.888888888	0	0	1	0	42.288408	2	-0.4111751
8.4159	320964.118	0.92004749	236.194278	1.74570378	0	2.13012277	5.77133245	-0.0822437	5.46465891	0.55773046	-6.9077553	21	62	8.0467	281.888889	8.888888888	0	0	1	0	42.288408	2	-0.4111751
8.7309	336854.578	0.90826643	227.331853	1.63568569	0	2.16686846	5.81965429	-0.0951171	5.42641525	0.49267327	-6.9077553	22	63	5.81150556	281.888889	8.888888888	0	0	1	0	42.288408	2	-0.4111751
9.0387	353019.644	0.93267108	242.469687	1.80141712	0	2.20151536	5.86652654	-0.0686311	5.49088082	0.58912861	-6.9077553	23	65	5.81150556	281.888889	8.888888888	0	0	1	0	42.288408	2	-0.4111751

0.0018	0	0.58418107	80.7339172	2.82491279	0	-6.3199686	-6.9077553	-0.535834	4.39117116	1.03883142	-6.9077553	0	89	4.47038889	295.222222	22.2222222	0	0	0	0	18.9135952	2	-0.0428739
0.0018	0	0	0	0	0	-6.3199686	-6.9077553	-6.9077553	-6.9077553	-6.9077553	-6.9077553	1	88	4.91742778	295.222222	22.2222222	0	0	0	0	18.9135952	2	-0.0428739
0.0018	0	0	0	0	0	-6.3199686	-6.9077553	-6.9077553	-6.9077553	-6.9077553	-6.9077553	2	86	5.36446667	295.222222	22.2222222	0	0	0	0	18.9135952	2	-0.0428739
0.0018	0	0	0	0	0	-6.3199686	-6.9077553	-6.9077553	-6.9077553	-6.9077553	-6.9077553	3	98	2.68223333	289.666667	16.66666666	0	0	0	0	18.9135952	2	-0.0428739
0.0027	0	0.13822962	3.51805902	0.3471278	0	-5.9145035	-6.9077553	-1.9716307	1.25819363	-1.0551856	-6.9077553	4	100	1.78815556	289.666667	16.66666666	0	0	0	0	18.9135952	2	-0.0428739
0.0198	0	0.31914322	25.190971	1.10843377	0	-3.9220733	-6.9077553	-1.1389868	3.22652533	0.10384977	-6.9077553	5	93	3.57631111	292.444444	19.44444444	0	0	0	0	18.9135952	2	-0.0428739
0.126	87.732864	0.58042132	92.7448275	2.83158719	0	-2.0714734	-2.422125	-0.5422796	4.52986271	1.0411905	-6.9077553	6	76	4.47038889	294.111111	21.11111111	0	0	0	0	18.9135952	2	-0.0428739
0.3285	2085.03106	0.66921238	149.769549	2.94380697	0	-1.1132184	0.73526325	-0.4001606	5.00910445	1.08004327	-6.9077553	7	63	5.36446667	294.111111	21.11111111	0	0	1	0	18.9135952	2	-0.0428739
0.5229	10511.0455	0.92477353	297.646231	2.42059326	0	-0.648365	2.35252179	-0.0771256	5.695909	0.8844257	-6.9077553	8	55	5.36446667	295.222222	22.2222222	0	0	1	0	18.9135952	2	-0.0428739
0.693	14817.5425	0.72934864	167.516927	1.86060006	0	-0.3667253	2.69587927	-0.3142333	5.12109037	0.62143637	-6.9077553	9	42	5.81150556	296.333333	23.33333333	0	0	1	0	18.9135952	2	-0.0428739
0.918	19193.2235	0.75422876	195.626156	1.95024902	0	-0.0855579	2.95460937	-0.2807346	5.27621058	0.66846969	-6.9077553	10	30	7.59966111	295.777778	22.7777778	0	0	1	0	18.9135952	2	-0.0428739
1.1664	26264.9942	0.7800147	183.453748	1.81730597	0	0.15392208	3.26827511	-0.2471613	5.21196803	0.59790528	-6.9077553	11	28	7.59966111	296.333333	23.33333333	0	0	1	0	18.9135952	2	-0.0428739
1.4517	33731.759	0.73617852	166.579419	1.89230895	0	0.37273528	3.51846944	-0.3049252	5.11547819	0.63832607	-6.9077553	12	34	8.49373889	294.111111	21.11111111	0	0	1	0	18.9135952	2	-0.0428739
1.7505	41013.1158	0.70646121	152.381401	2.04236024	0	0.55990146	3.7139163	-0.3460725	5.02639315	0.71459563	-6.9077553	13	40	8.0467	290.777778	17.7777778	0	0	1	0	18.9135952	2	-0.0428739
2.0376	48635.0007	0.69815762	149.625882	1.72736954	0	0.71177264	3.88436401	-0.3578791	5.00814474	0.5471785	-6.9077553	14	47	6.70558333	288	15	0	0	1	0	18.9135952	2	-0.0428739
2.3652	62051.7399	0.81937752	219.525434	1.97142062	0	0.86086258	4.12798467	-0.1979907	5.39147265	0.67926153	-6.9077553	15	50	7.15262222	286.888889	13.88888888	0	0	1	0	18.9135952	2	-0.0428739
2.6622	75788.3036	0.88175918	237.841241	1.75244843	0	0.97915285	4.32795717	-0.1247028	5.4716076	0.56158438	-6.9077553	16	53	7.59966111	285.222222	12.22222222	0	0	1	0	18.9135952	2	-0.0428739
2.9853	91027.4096	0.90714402	233.532556	1.63547288	0	1.09370024	4.51117165	-0.0963523	5.45332577	0.49254325	-6.9077553	17	57	4.47038889	283.555556	10.55555555	0	0	1	0	18.9135952	2	-0.0428739
3.2805	106194.038	0.93514531	239.260123	1.69771334	0	1.18799585	4.66527738	-0.0659846	5.47755552	0.52987111	-6.9077553	18	64	5.81150556	282.444444	9.444444444	0	0	1	0	18.9135952	2	-0.0428739
3.5685	120333.142	0.86733484	203.170654	1.49891386	0	1.27214534	4.79027239	-0.1411779	5.31405121	0.40540768	-6.9077553	19	66	6.25854444	282.444444	9.444444444	0	0	1	0	18.9135952	2	-0.0428739
3.8691	135880.252	0.89494607	219.367983	1.64266219	0	1.35302192	4.91178136	-0.1098751	5.39075516	0.49692679	-6.9077553	20	64	5.81150556	281.888889	8.888888888	0	0	1	0	18.9135952	2	-0.0428739
4.1877	153118.487	0.95893162	259.709989	1.87794727	0	1.43215166	5.03121858	-0.0408932	5.55956943	0.63071166	-6.9077553	21	62	8.0467	281.888889	8.888888888	0	0	1	0	18.9135952	2	-0.0428739
4.5387	173024.671	0.99899957	281.686959	2.02262871	0	1.51264063	5.15343997	-4.29E-07	5.64079993	0.70489229	-6.9077553	22	63	5.81150556	281.888889	8.888888888	0	0	1	0	18.9135952	2	-0.0428739
4.9167	194174.593	0.98541282	274.823958	1.98417911	0	1.59263757	5.26876287	-0.0136803	5.61613438	0.68570914	-6.9077553	23	65	5.81150556	281.888889	8.888888888	0	0	1	0	18.9135952	2	-0.0428739
0.0135	400.772316	0.79993534	177.754011	1.4631849	0	-4.3050656	-0.9118697	-0.2219751	5.18040626	0.38129871	-6.9077553	0	92	1.78815556	295.777778	22.7777778	0	0	0	0	30.519848	2	0.21141735
0.027	927.137818	0.5678381	84.3918376	0.91810385	0	-3.6119184	-0.074575	-0.5641594	4.43548254	-0.0843562	-6.9077553	1	90	2.68223333	296.888889	23.88888888	0	0	0	0	30.519848	2	0.21141735
0.0693	1585.09441	0.55558164	95.903019	1.18183753	0	-2.6693104	0.46127465	-0.5859414	4.56334789	0.16791624	-6.9077553	2	83	3.12927222	298.555556	25.55555555	0	0	0	0	30.519848	2	0.21141735
0.1521	2418.50641	0.45940822	59.4936895	1.53791162	0	-1.8832171	0.88356356	-0.7756417	4.08588706	0.43107543	-6.9077553	3	77	4.47038889	299.111111	26.11111111	0	0	0	0	30.519848	2	0.21141735
0.2331	3043.0221	0.50698446	71.0231879	1.64232432	0	-1.4562877	1.1131797	-0.6773044	4.26302049	0.49672121	-6.9077553	4	72	4.47038889	300.222222	27.2222222	0	0	0	0	30.519848	2	0.21141735
0.3969	4627.77033	0.70717269	149.705507	2.53681661	0	-0.9240709	1.53229125	-0.3450673	5.00867676	0.93130411	-6.9077553	5	63	6.25854444	301.333333	28.33333333	0	0	0	1	30.519848	2	0.21141735
0.6363	10182.5605	0.83998068	209.736372	2.92228527	0	-0.4520851	2.3207747	-0.1731866	5.34585614	1.07270808	-6.9077553	6	59	6.25854444	302.444444	29.44444444	0	0	0	1	30.519848	2	0.21141735
0.9018	20547.5843	0.7421544	152.992704	2.05913541	0	-0.1033625	3.02279205	-0.2968515	5.03039677	0.72277171	-6.9077553	7	55	4.91742778	304.111111	31.11111111	0	0	0	1	30.519848	2	0.21141735
1.1943	28949.3073	0.72351074	147.463666	1.88656212	0	0.17756024	3.36558082	-0.3222587	4.99358859	0.63528612	-6.9077553	8	54	4.02335	305.222222	32.2222222	0	0	0	1	30.519848	2	0.21141735
1.4346	38260.9663	0.67721152	135.629184	1.38085229	0	0.36088606	3.64445636	-0.3882961	4.90993195	0.32342484	-6.9077553	9	53	4.02335	305.777778	32.7777778	0	0	0	1	30.519848	2	0.21141735
1.6083	49124.9034	0.71076483	147.211889	1.21682138	0	0.47517772	3.89438646	-0.3400077	4.99187976	0.19706351	-6.9077553	10	59	4.47038889	304.111111	31.11111111	0	0	0	1	30.519848	2	0.21141735
1.7055	56282.5066	0.65411335	123.57052	0.9067048	0	0.53385832	4.03040154	-0.422947	4.8168201	-0.0968361	-6.9077553	11	59	4.47038889	304.111111	31.11111111	0	0	0	1	30.519848	2	0.21141735
1.7775	64066.6331	0.73372526	146.262598	0.96720209	0	0.57520788	4.15993929	-0.3082586	4.98541046	-0.0323144	-6.9077553	12	63	3.57631111	302.444444	29.44444444	0	0	0	1	30.519848	2	0.21141735
1.8396	70824.3002	0.7088722	129.961192	0.94937554	0	0.60954816	4.26021628	-0.3426703	4.86724358	-0.0508981	-6.9077553	13	69	1.78815556	301.333333	28.33333333	0	0	0	1	30.519848	2	0.21141735
1.9071	78551.229	0.71425318	138.725247	1.06712769	0	0.64558376	4.36376374	-0.3351187	4.93250255	0.06590729	-6.9077553	14	69	2.68223333	300.777778	27.7777778	0	0	0	1	30.519848	2	0.21141735
1.9683	85832.0111	0.66829322	114.594619	0.87728786	0	0.67717023	4.45240368	-0.401533	4.74140957	-0.1297809	-6.9077553	15	73	3.57631111	300.222222	27.2222222	0	0	0	1	30.519848	2	0.21141735
1.998	87508.4442	0.57262964	81.0818315	0.75787301	0	0.69214668	4.47174672	-0.5557713	4.39547124	-0.2759208	-6.9077553	16	75	3.57631111	299.666667	26.66666666	0	0	0	1	30.519848	2	0.21141735
2.0061	87903.2176	0.22649402	11.2528542	0.3408259	0	0.69619254	4.47624779	-1.4806313	2.42071067	-1.0734537	-6.9077553	17	77	4.02335	299.111111	26.11111111	0	0	0	1	30.519848	2	0.21141735
2.0187	88341.8547	0.24967578	12.8360432	0.35927531	0	0.70245374	4.48122532	-1.3835949	2.55233499	-1.0208868	-6.9077553	18	79	1.78815556	298.555556	25.55555555	0	0	0	1	30.519848	2	0.21141735
2.0286	88736.6307	0.23853436	11.8986497	0.39843334	0	0.7073459	4.48568405	-1.4290584	2.47650897	-0.9177084	-6.9077553	19	80	1.78815556	298.555556	25.55555555	0	0	0	1	30.519848	2	0.21141735
2.0358	89043.6763	0.24019986	11.7401352	0.34164438	0	0.71088886	4.48913822	-1.4221294	2.4630985	-1.0710622	-6.9077553	20	83	1.34111667	298	25	0	0	0	1	30.519848	2	0.21141735
2.043	89350.7236	0.22913642	10.6349792	0.37416235	0	0.71441932	4.49258053	-1.469083	2.36424252	-0.9803964	-6.9077553	21	86	0.89407778	297.444444	24.44444444	0	0	0	1	30.519848	2	0.21141735
2.052	89745.4954	0.20933609	9.07328415	0.34650571	0	0.71881493	4.49698898	-1.5590486	2.2054445	-1.0569742	-6.9077553	22	87	1.34111667	297.444444	24.44444444	0	0	0	1	30.519848	2	0.21141735
2.0565	89964.8149	0.2150332	9.19674225	0.3078202	0	0.72100551	4.49942976	-1.5323232	2.21895805	-1.174996	-6.9077553	23	90	2.23519444	296.888889	23.88888888	0	0	0	1	30.519848	2	0.21141735
0.0153	704.682344	0.96151453	256.787323	1.82170546	0	-4.1799025	-0.3485901	-0.0382061	5.5482521	0.60032191	-6.9077553	0	92	1.78815556	295.777778	22.7777778	0	0	0	0	34.0724638	2	0.49277013
0.0252	1011.7289	0.50729145	60.603954	0.73947941	0	-3.6809113	0.01264857	-0.6767003	4.10437664	-0.3004574	-6.9077553	1	90	2.68223333	296.888889	23.88888888	0	0	0	0	34.0724638	2	0.49277013
[truncated: 1,214,169 more chars]
